# Linking Thermal Conductivity to Equations of State Using the Residual Entropy Scaling Theory

**DOI:** 10.1021/acs.iecr.4c02946

**Published:** 2024-10-15

**Authors:** Zhuo Li, Yuanyuan Duan, Xiaoxian Yang

**Affiliations:** 1 Key Laboratory for Thermal Science and Power Engineering of Ministry of Education, Beijing Key Laboratory for CO_2_ Utilization and Reduction Technology, Tsinghua University, Beijing 100084,People’s Republic of China; 2 Southwest United Graduate School, Kunming 650092, People’s Republic of China; 3 Applied Thermodynamics, Chemnitz University of Technology, Chemnitz 09107, Germany

## Abstract

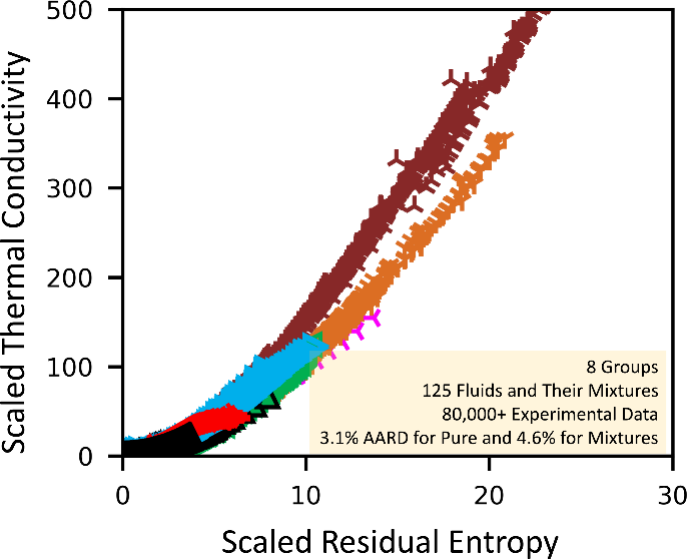

In recent years, the application of the residual entropy scaling (RES) method for modeling transport properties has become increasingly prominent. Based on Yang et al. (*Ind. Eng. Chem. Res*. **2021**, *60*, 13052) in modeling the thermal conductivity of refrigerants, we present here an RES model that extends Yang et al.’s approach to a wider range of pure fluids and their mixtures. All fluids available in the REFPROP 10.0 software, i.e., those with reference equations of state (EoS), were studied. A total of 71,554 experimental data of 125 pure fluids and 16,702 experimental data of 164 mixtures were collected from approximately 647 references, mainly based on the NIST ThermoData Engine (TDE) database 10.1. As a result, over 68.2% (corresponding to the standard deviation of a normal distribution) of the well-screened experimental data agree with the developed RES model within 3.1% and 4.6% for pure fluids and mixtures, respectively. Comparative analysis against the various models implemented in the REFPROP 10.0 (one of the state-of-the-art software packages for thermophysical property calculations) reveals that our RES model demonstrates analogous statistical agreement with experimental data yet with much fewer parameters. Regarding the average absolute value of the relative deviation (AARD) from experimental values to model predictions, the developed RES model shows a smaller or equal AARD for 74 pure fluids out of 125 and 76 mixtures out of 164. Besides, a detailed examination of the impact of the critical enhancement term on mixture calculations was conducted. To use our model easily, a software package written in Python is provided in the Supporting Information.

## 1 Introduction

Thermal conductivity, an essential transport property, represents the spontaneous energy transfer process resulting from temperature differences. Experimental and modeling studies on the thermal conductivity of fluids are of great scientific importance. In contrast to extensively studied thermophysical properties such as density, experimental data on the thermal conductivity of fluids are relatively sparse and limited to certain temperature and pressure ranges.^1−3^ This limitation poses a challenge to constructing empirical multiparameter models, which rely heavily on extensive experimental data, especially as the scope of investigation expands to include a growing number of pure fluids and fluid mixtures. Consequently, the pursuit of semiempirical models, which require fewer parameters and less experimental data for parameter-fitting, is becoming an important focus in thermal conductivity modeling.

Current semiempirical thermal conductivity models include those depending on the free volume theory,^4^ the hard-sphere theory,^5^ the friction theory,^6,7^ the extended corresponding states (ECS),^8,9^ and the residual entropy scaling (RES).^10^ Among these models, the RES one is attracting more and more attention. According to the isomorph theory,^11,12^ the dimensionless transport properties of fluids, especially viscosity and thermal conductivity, can be expressed as a univariate function of residual entropy within a group of similar fluids. As a result, the RES model requires a significantly smaller number of experimental data points for parameter fitting than most other models. Furthermore, owing to the analogous nature of the relation between dimensionless transport properties and dimensionless residual entropy of different fluids, ideally, no additional fitting parameters are required to predict mixtures. Researchers such as Bell et al.,^13−16^ Yang et al.,^17−22^ Liu et al.,^23−26^ Kang et al.,^27−29^ Hopp et al.,^30−32^ and Lötgering-Lin et al.^33−35^ have effectively utilized this approach to model both the viscosity and thermal conductivity of pure fluids, yielding promising results.

In this paper, we focus on developing RES methods for thermal conductivity. In recent years, numerous researchers have made significant progress. Hopp and Gross^30^ developed a RES model for the thermal conductivity of water and 147 organic substances by using the equation of state (EOS) of the perturbed-chain polar statistical associating fluid theory (PCP-SAFT)^36,37^ to determine the residual entropy. The RES model showed an average relative deviation of 4.2% from the experimental data. They also investigated a universal modeling approach integrating RES with the group contribution method.^31^ Dehlouz et al.^38^ introduced a new dimensionless mathematical form for the relation of thermal conductivity and residual entropy similar to that initially proposed by Rosenfeld.^39^ They used *tc*-PR EOS^40^ and *I*-PC-SAFT EOS^41^ to calculate the residual entropy and developed a RES model for 119 pure fluids with an average deviation of about 3.4%. Liu et al.^26^ created a RES model with critical enhancement correction for CO_2_ using the crossover multiparameter EOS.^42^ Regarding mixtures, the research focus was primarily on refrigerants and their mixtures. Fouad^43^ and Kang et al.^28^ used the PC-SAFT EOS,^44^ while Liu et al.^24^ used the cubic-plus-association (CPA) EOS^45^ to calculate the residual entropy. In addition, Rokni et al.^46,47^ developed RES models based on Hopp and Gross’s research^30^ for typical fluids in combustion processes, including hydrocarbon mixtures, to predict the thermal conductivity of fluids under extreme conditions. The studies above on mixtures are all tailored to a single class of fluids; therefore, one of the main objectives of this study is to extend the model to cover a broader range of fluids.

The RES model developed by Yang et al.^48,49^ is an essential foundation of this work. Yang et al.^48^ utilized REFPROP 10.0^50^ for the residual entropy calculation, formulating an RES model for the thermal conductivity of refrigerants and their mixtures and integrating the critical enhancement crossover model developed by Olchowy and Sengers.^51^ Satisfactory agreements with experimental data were achieved for the mixtures without any additional adjustable binary interaction parameters (BIP). Later, for viscosity, Yang et al.^49^ classified different fluids into different groups and developed a RES model for 124 fluids ranging from light gases (e.g., helium) to dense fluids (e.g., hexadecane). This work aims to use the same fluid classification method of viscosity^49^ to extend the thermal conductivity model developed by Yang et al.^48^ from 39 refrigerants to 125 pure fluids. This study will also employ reference EOS to calculate residual entropy, fully capitalizing on the benefits of REFPROP 10.0 in thermodynamic property computations. All fluids implemented in the REFPROP 10.0, namely those with reference EOS, were studied. A total of 71,554 experimental data points for 125 pure fluids and 16,702 experimental data points for 164 mixtures were compiled from approximately 647 references, mainly based on the NIST ThermoData Engine (TDE) database 10.1.^52^ Given the significantly expanded range of fluid types considered, the analysis focused mainly on the results of mixtures from a fluid-group perspective. Furthermore, a detailed examination of the impact of the critical enhancement term on the mixtures was conducted.

## 2 Theory

Fluid thermal conductivity λ is calculated with the sum of the dilute gas term λ_ρ→0_(*T*), the residual term λ_res_(*s*^r^), and the critical enhancement term Δλ_C_(*T*,ρ), expressed as follows:
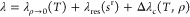
1

The dilute gas thermal conductivity λ_ρ→0_(*T*) is a function of temperature *T*. For pure fluids, a polynomial expression is employed:
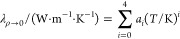
2

The parameters *a*_*i*_ were determined by fitting them to calculations from REFPROP 10.0^50^ for each pure fluid. These values are listed in Table S1 in the Supporting Information (SI) and have been implemented in the OilMixProp 1.0 software package.^53^ We could potentially use REFPROP 10.0 to calculate λ_ρ→0_. However, REFPROP 10.0 uses a variety of different methods to calculate λ_ρ→0_, which are hard to replicate in our coding. Besides, REFPROP 10.0 fails to calculate dilute gas thermal conductivity of many mixtures especially those involving alcohols. Therefore, the proposed eq 2 could be used in combination with a proper mixing rule for calculating the thermal conductivity of the dilute gas thermal conductivity (see the following).

As described by Bell^13^ and Yang et al.,^48^ the residual term of thermal conductivity λ_res_(*s*^r^) can be determined using the formula:
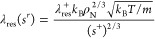
3where
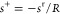
4

Here, ρ_N_ in units of m^–3^ represents the number density, *m* in units of kg is the mass of one molecule, *s*^r^ in units of J·mol^–1^·K^–1^ denotes the molar residual entropy, which is the difference between the actual fluid entropy and the ideal gas entropy at the same temperature and density, and *R* = 8.31446261815324 J·mol^–1^·K^–1^ is the molar gas constant. In this study, the number density ρ_N_ and molar residual entropy *s*^r^ were computed using the reference EOS implemented in REFPROP 10.0, with the interface of Python CoolProp package version 6.4.1.^54^ The reference EOS and thermal conductivity models used in REFPROP 10.0 are listed in Table S2 of the SI. The plus-scaled dimensionless residual thermal conductivity is related to the plus-scaled dimensionless residual entropy *s*^+^ using the following polynomial equations
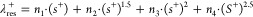
5or
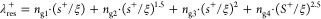
6

Equation 5 is tailored for a pure fluid with fluid-specific fitted parameter *n*_k_ (*k* = 1, 2, 3, 4), while eq 6 is designed for a group of pure fluids with group-specific fitted parameters *n*_g*k*_ (*k* = 1, 2, 3, 4) and a fluid-specific scaling factor ξ for each individual fluid.

The critical enhancement of thermal conductivity is determined using a crossover model introduced by Olchowy and Sengers^51^ as
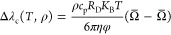
7
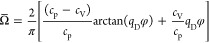
8
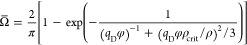
9
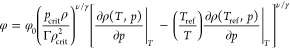
10

Here, *η* represents the viscosity, *c*_p_ and *c*_V_ denote the isobaric and isochoric specific heat capacities, respectively, and ρ_crit_ and *p*_crit_ stand for the molar density and pressure at the critical point, respectively. These parameters are calculated using the default models in REFPROP 10.0. The values of *R*_D_ = 1.02, ν = 0.63, and γ = 1.239 are universal constants, while Γ, φ_0_, *T*_ref_, and *q*_D_ are fluid-specific parameters acquired from REFPROP 10.0 and detailed in Table S3 in the SI.

A predictive mixing rule is adopted to extend the RES model to mixtures. Similar to the previous work,^48^ the dilute gas thermal conductivity for mixtures λ_ρ→0,mix_ is calculated with REFPROP 10.0 via the Python package CoolProp 6.4.1.^54,55^ However, REFPROP 10.0 cannot calculate the dilute gas thermal conductivity λ_ρ→0,mix_ of some mixture especially those involving alcohols and water. For these mixtures, a mixing rule similar to the Wilke approximation^56^ was adopted:
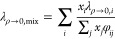
11with
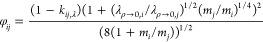
12where *x*_*i*_ is the mole fraction of component *i* and *m*_*i*_ is the mass of one molecule of component *i.* The binary interaction coefficients for dilute gases *k*_*ij,*λ_ are all set to 0, which could be fitted if sufficient accurate dilute gas data for a mixture are available. Different from our previous work,^48^ a new mass fraction weighted mixing rule was used to as the effective mass of one particle of the mixture (to replace *m* in eq 3 for mixture calculation):
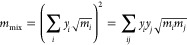
13where *y*_*i*_ is the mass fraction of component *i*. This will be discussed in more detail in Section 3.2. Subsequently, in contrast to our previous work,^48^ the mole fraction weighted average coefficient *n*_*k*,mix_ is employed to substitute the parameters *n*_*k*_ in eq 6, i.e.,
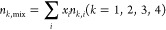
14where *n*_*k,i*_ (*k* = 1,2,3,4) are fluid-specific fitted parameters *n*_*k*_ of component *i*. It is essential to mention that *n*_*k*_ (*k* = 1, 2, 3, 4) parameters of a pure fluid are replaced by *n*_g1_/ξ, *n*_g2_/ξ^1.5^, *n*_g3_/ξ^2^, and *n*_g4_/ξ^2.5^, respectively, only if the pure fluid does not have fluid-specific fitted parameters.

For the critical enhancement term of the thermal conductivity of fluid mixtures, we implemented the identical mixing rule as the ECS model devised by Chichester and Huber,^57^ wherein, for mixtures, the parameter Γ, φ_0_, *T*_ref_, and *q*_D_ in eqs 7–10 are replaced with the mole-fraction-weighted average of each individual component
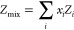
15where *x*_*i*_ is the mole fraction of component *i* and *Z* is one of the parameters Γ, φ_0_, *T*_ref_, and *q*_D_. The critical parameters (*T*_crit_, ρ_crit_, and *p*_crit_) of the mixtures were also acquired from REFPROP 10.0 as well.

## 3 Results

### 3.1 Data Collection and Selection

**Figure 1:**
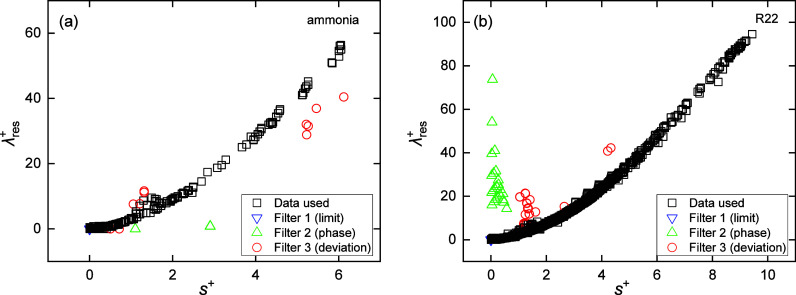
Characteristic λ_res_^+^ vs *s*^+^ curves of (a) ammonia^58−63^ and (b) R22.^64−80^ The definitions of Filter 1, 2, and 3 can be found in the footnote of Table 1.

A total of 71,554 experimental thermal conductivity values (λ, *T*, *p*) for 125 pure fluids and 16,702 values (λ, *T*, *p*, *x*) for 164 mixtures (including binary and multicomponent) from approximately 647 references were collected. Detailed citation information is provided in the Supporting Information—Detailed Plots and References (SI-DPR). The literature is primarily sourced from the NIST TDE 10.1,^52^ supplemented by recently published experimental data we gathered. All data were carefully examined to address issues such as units, magnitudes, or transcription errors. Subsequently, the same methods (see footnote in Table 1) as used in our previous work^48,49^ were adopted to filter out approximately 3.9% pure fluid data and 5.7% of mixture data. Table 1 presents the number of experimental data collected, the number of sources, and the number and proportion of data filtered out by each filter. Figure 1 shows experimental data of two example fluids (ammonia^58−64^ and R22^65−81^) processed with these three filters. Data filtered by filter 1 are all concentrated at zero because their *s*^+^ and λ_res_^+^ cannot be correctly calculated. To a certain extent, these filters also serve for data evaluation, e.g., data filtered out by filter 3 are usually of low quality as they are obviously not consistent with other data. For detailed information on Table 1 for each fluid, please refer to Tables S4 and S5 in the Supporting Information (SI). The experimental data’s temperature, pressure, and composition ranges are also summarized in these two tables.

**Table 1:** Statistical Experimental Data Summary of Pure Fluids and Their Mixtures^a^

		*S*_D_	*N*_tot_	*N*_use_	*N*_lim_	*N*_phase_	*N*_dev_	*N*_REFPROP,fail_
pure fluids	total	567	71554	68765	1544	468	777	72
	*N*/*N*_tot_			96.10%	2.16%	0.65%	1.09%	0.10%
mixtures	total	124	16702	15758	685	156	103	1976
	*N*/*N*_tot_			94.35%	4.10%	0.93%	0.62%	11.83%

a *S*_D_: number of data sources; *N*_tot_: total number of experimental data; *N*_use_: number of adopted data for parameter-fitting and comparison analysis; *N*_lim_: number of data filtered by Filter 1, which removed those exceeding limits of the reference EoS in REFPROP 10.0; *N*_phase_: number of data filtered by Filter 2, which removed those reported in conflicting phases; *N*_dev_: number of data filtered by Filter 3, which removed those deviating from the RES model by more than 30%. *N*_REFPROP,fail_: number of data whose thermal conductivity cannot be calculated with the recommended models in REFPROP 10.0 using the given temperature and pressure while these data will still be used for the evaluation of the RES model. Please note: some literature have both data of pure fluid and mixtures; therefore, the total number of literature source was 647.

All experimental data, except those removed by Filter 1, are displayed in characteristic λ_res_^+^ vs *s*^+^ curves as provided in the SI-DPR. For each pure fluid, the experimental (*s*^+^, λ_res_^+^) points collapse into a characteristic single line with some noise due to the poor quality of some data. A few fluids (such as He and CO) only have experimental data with *s*^+^ values close to zero, so the range of residual entropy corresponding to characteristic lines is limited.

### 3.2 Correlation for Pure Fluids

In this section, the RES model for 125 pure fluids, whose reference EoS and experimental thermal conductivity data are both available, was established. These pure fluids were divided into 8 groups, similar to our previous work.^49^ The number and detailed description of each group are presented in Table 2. The classification of groups meets the following objectives: with as few groups as possible, the group-specific parameter *n*_gk_ fitted to each group and the fluid-specific scaling factor ξ can produce the best statistical consistency. For pure fluids with sufficient quantity and good quality experimental data, the fluid-specific parameter *n*_k_ could also be fitted. The results are listed in Tables 3 and 4. In Table 3, for the additional 26 (= 151–125) pure fluids implemented in REFPROP 10.0 (151 pure fluids in total) without available data, the group number was determined by the following steps. (1) Carrying out calculations using REFPROP 10.0 in both liquid and gas phases and using these data as reference data; (2) fitting ξ using each group’s group-specific fitted parameters with these reference data; (3) assigning the group number of this fluid where its ξ is most close to 1.

**Table 2:** Fluid Groups and Their Brief Descriptions

group number	group abbreviation	detailed description
1	LG	light gases with quantum effects at low temperatures, mainly hydrogen and its spin isomers and helium
2	G	gaseous fluids, e.g., the noble gases
3	LHC	a majority of light hydrocarbons and halogenated hydrocarbons (refrigerants)
4	B	fluids with benzene rings and similar fluids
5	MHC	medium hydrocarbons and similar fluids
6	HHC	heavy hydrocarbons and dense fluids
7	LA	fluids with light intermolecular association among molecules like methanol
8	SA	fluids with strong intermolecular association among molecules, such as water

**Table 3:** Fluid Information and the Fluid-Specific Fitted Parameters

REFPROP fluid name	group number	ξ	*Z*^a^	*n*_1_	*n*_2_	*n*_3_	*n*_4_
13BUTADIENE	2	0.971	1	0.000000	5.379592	–3.515631	1.143689
1BUTENE	5	0.8916	1	0.000000	4.218309	–2.607745	0.973758
1BUTYNE	4	1	0				
1PENTENE	4	0.9643	0				
22DIMETHYLBUTANE	3	0.9836	1	0.000000	2.787455	–0.347996	0.254597
23DIMETHYLBUTANE	3	0.9782	1	0.000000	3.418932	–0.754166	0.319323
3METHYLPENTANE	5	0.9109	1	0.000000	5.003443	–2.239168	0.664843
ACETONE	4	1.059	0				
ACETYLENE	3	1.1945	1	0.000000	1.750292	–0.098956	0.143796
AMMONIA	7	0.9804	1	0.357901	2.508824	0.437440	–0.007305
ARGON	2	0.9929	1	2.212697	–4.335464	4.685634	–0.731662
BENZENE	4	0.9864	1	6.114857	–11.805067	9.165699	–1.624275
BUTANE	3	0.9873	1	9.914508	–15.844874	9.840589	–1.467766
C11	5	0.7956	1	5.167730	–5.907157	4.958787	–0.634295
C12	5	0.8045	0				
C16	5	0.7121	1	12.868089	–9.340009	5.387528	–0.571729
C1CC6	3	1.0211	1	12.813721	–19.138957	10.658325	–1.477798
C22	6	0.741	1	0.000000	10.736853	–2.041242	0.296740
C2BUTENE	5	0.9302	1	0.888244	3.084085	–2.425022	0.976326
C3CC6	4	0.9774	1	13.331441	–19.894384	11.138446	–1.554099
C4F10	3	1	0				
C5F12	5	0.8689	0				
C6F14	5	0.8686	0				
CF3I	3	1	0				
CHLORINE	3	0.9156	1	0.000000	–1.813594	4.704503	–1.005004
CHLOROBENZENE	4	1.0177	1	0.000000	0.813486	1.321055	–0.125706
CO	2	1	0				
CO2	3	1.002	1	1.706852	–2.225083	2.920715	–0.376879
COS	3	1	0				
CYCLOBUTENE	3	1	0				
CYCLOHEX	3	1.008	1	0.000000	2.971082	–0.481603	0.256643
CYCLOPEN	3	0.9864	0				
CYCLOPRO	3	1.235	1	4.574498	–12.114472	10.732103	–2.418843
D2	1	0.9476	0				
D2O	8	1.1823	1	5.261652	–6.384364	5.908181	–1.256585
D4	6	0.8105	1	0.000000	–4.121521	6.562392	–0.964812
D5	6	0.7271	1	0.000000	–5.170102	7.532869	–1.060288
D6	5	1	0				
DEA	7	0.9878	0				
DECANE	5	0.8113	1	4.264299	–3.187923	3.467454	–0.428095
DEE	3	0.9879	1	5.071657	–14.357684	11.583587	–2.101609
DMC	3	0.9874	1	0.000000	–1.097291	2.780174	–0.374842
DME	3	1.0457	1	0.759606	–0.891232	2.426799	–0.393419
EBENZENE	4	0.996	1	5.914624	–8.923543	6.023321	–0.807386
EGLYCOL	7	1.2492	1	0.000000	–0.071257	2.106998	–0.414900
ETHANE	3	1.0094	1	2.264652	–2.973246	2.834533	–0.168146
ETHANOL	7	1.6238	1	0.256349	7.523893	–4.554179	0.861844
ETHYLENE	3	0.982	1	0.889433	–0.284962	2.094853	–0.445207
ETHYLENEOXIDE	3	1.2018	1	0.000000	0.098128	1.778352	–0.332378
FLUORINE	2	0.9429	1	0.000000	–0.114084	2.560271	–0.433969
H2S	7	1.445	1	0.432501	2.740587	–1.819681	0.938402
HCL	7	0.8174	0				
HELIUM	1	1.2966	0				
HEPTANE	5	0.8636	1	1.639500	–4.109500	4.754431	–0.712181
HEXANE	5	0.8867	1	12.117771	–17.462543	10.233398	–1.465975
HYDROGEN	1	1.0925	1	2.263272	–7.356751	10.699531	–3.575533
IBUTENE	3	1.0042	1	4.864538	–6.452786	4.039782	–0.328573
IHEXANE	5	0.8797	1	0.000000	0.813682	1.580508	–0.152544
IOCTANE	5	0.8769	1	8.197005	–15.049807	9.373205	–1.270155
IPENTANE	3	0.9565	0				
ISOBUTAN	3	1.0344	1	7.423475	–12.623751	8.360390	–1.271135
KRYPTON	2	0.9803	1	4.126446	–6.776568	5.542582	–0.773972
MD2M	6	1	0				
MD3M	6	1	0				
MD4M	6	1	0				
MDM	6	0.8909	1	8.916267	2.640074	–2.421293	0.851311
MEA	7	1.2444	0				
METHANE	2	0.9897	1	2.149868	–3.171791	3.076996	–0.191412
METHANOL	7	1.6895	1	3.286199	3.920777	–3.533019	0.823359
MILPRF23699	6	1	0				
MLINOLEA	6	0.8121	1	0.000000	5.038723	1.660785	–0.324476
MLINOLEN	6	1	0				
MM	6	0.9762	1	0.000000	–2.998729	4.897334	–0.700838
MOLEATE	6	0.7932	1	0.000000	7.481170	0.592821	–0.199701
MPALMITA	6	1	0				
MSTEARAT	6	0.7428	0				
MXYLENE	4	0.9819	0				
N2O	2	1.0618	1	2.513468	–5.101075	5.298646	–1.002263
NEON	2	1	0				
NEOPENTN	3	1	0				
NF3	3	1	0				
NITROGEN	2	0.9628	1	2.343833	–4.109131	4.450635	–0.669527
NONANE	5	0.8295	1	1.426501	–10.319122	8.964770	–1.374319
NOVEC649	4	1	0				
OCTANE	5	0.853	1	0.000000	3.192025	0.047318	0.121680
ORTHOHYD	1	1	0				
OXYGEN	2	0.9973	1	3.578576	–7.686498	7.651484	–1.602941
OXYLENE	4	1.0148	1	13.315169	–19.183399	10.385784	–1.404669
PARAHYD	1	1	0				
PENTANE	3	0.9701	1	17.306659	–19.435781	9.257508	–1.127877
POE5	6	1	0				
POE7	6	1	0				
POE9	6	1	0				
PROPADIENE	3	1	0				
PROPANE	3	0.9964	1	8.726474	–13.787569	8.781108	–1.303329
PROPYLEN	4	0.9227	1	2.978741	–3.719802	3.952515	–0.647533
PROPYLENEOXIDE	3	1.0695	0				
PROPYNE	3	1.0758	0				
PXYLENE	4	0.9924	1	10.975030	–14.386000	7.788535	–0.960621
R11	3	0.9455	1	2.448812	–3.077300	3.779976	–0.574054
R1123	3	1	0				
R113	3	0.976	1	0.408042	–1.181694	3.113587	–0.513467
R114	3	0.9883	0				
R115	3	0.9707	1	0.832945	–0.549647	1.989828	–0.178955
R116	3	0.8263	0				
R12	3	0.9634	1	3.952520	–5.736267	5.068745	–0.778955
R1216	3	1	0				
R1224YDZ	3	1.0468	1	0.000000	5.697113	–3.681056	1.095443
R123	3	1.0185	1	11.296696	–16.704536	9.929254	–1.487969
R1233ZDE	3	1.015	1	1.381582	–2.889227	3.573098	–0.518453
R1234YF	3	1.0446	1	0.000000	0.561791	1.133353	–0.022522
R1234ZEE	3	1.0445	1	0.000000	–0.195660	1.970080	–0.248027
R1234ZEZ	3	1	0				
R124	3	1.0364	1	1.936501	–1.918713	2.181608	–0.162050
R1243ZF	3	1	0				
R125	3	1.0138	1	2.118214	–2.548352	2.867573	–0.336718
R13	3	0.9743	1	4.401745	–6.523089	5.339968	–0.796013
R1336MZZZ	3	1.0718	1	0.000000	2.110048	–0.096833	0.183517
R134A	3	1.0434	1	1.388264	–0.760952	1.664978	–0.125953
R14	3	0.9856	1	0.000000	1.856857	–0.091191	0.328629
R141B	3	1.0224	1	5.244290	–12.966918	10.001388	–1.724927
R142B	3	1.0185	1	1.585603	–1.288917	1.985725	–0.151916
R143A	3	1.0277	1	0.000000	0.808867	1.155897	–0.078609
R150	3	1.0501	1	0.000000	3.214526	–0.336963	0.125032
R152A	3	1.0498	1	0.986161	–0.163102	1.481922	–0.139125
R161	3	1.0454	1	0.000000	–1.364383	3.073718	–0.504590
R21	3	1.0163	1	0.000000	0.905175	1.252424	–0.118433
R218	3	0.943	1	4.546313	–5.628770	4.395302	–0.546137
R22	3	1.0388	1	4.091164	–5.280935	4.457871	–0.683280
R227EA	3	1.0511	1	4.622243	–14.334938	12.669269	–2.640429
R23	3	1.046	1	4.763672	–5.377349	4.149939	–0.596530
R236EA	3	1	0				
R236FA	3	1.022	1	2.665792	–7.207912	6.766258	–1.198062
R245CA	3	0.883	0				
R245FA	3	1.0533	1	0.259408	–2.453743	3.588802	–0.553704
*R*32	3	0.9671	1	0.833010	0.496007	1.424641	–0.174255
R365MFC	3	1.0094	0				
R40	3	1	0				
R41	3	0.8575	1	0.000000	5.494781	–1.103109	0.051828
RC318	3	0.9223	1	0.000000	6.665295	–3.424906	0.930574
RE143A	3	1	0				
RE245CB2	3	1.0231	1	1.713390	–1.188678	1.611470	–0.019372
RE245FA2	3	1.7541	0				
RE347MCC	3	0.9543	0				
SF6	2	0.854	1	0.000000	5.719873	–2.019152	0.506107
SO2	2	0.8837	1	0.000000	1.707698	1.647666	–0.360041
T2BUTENE	3	1	0				
TOLUENE	4	1.0079	1	9.949329	–15.028331	9.102394	–1.327497
VINYLCHLORIDE	3	1	0				
WATER	8	1.182	1	0.000000	–0.482377	4.465204	–1.296961
XENON	2	0.9925	1	1.370170	–1.432450	1.997402	0.005468

a The quantity and quality of the experimental data are good (*Z* = 1) or not good (*Z* = 0) enough to fit fluid-specific *n*_1_, *n*_2_, *n*_3_, and *n*_4_ parameters.

**Table 4:** Group-Specific Fitted Parameters of Each Group

group number	*n*_g1_	*n*_g2_	*n*_g3_	*n*_g4_
1	2.391631	–8.1473	12.52226	–4.38311
2	2.173335	–4.8767	5.754321	–1.18193
3	3.629822	–5.32944	4.534105	–0.64328
4	10.62084	–16.0687	9.495404	–1.35573
5	0	–0.15825	1.789146	–0.20526
6	0	0.895835	2.305079	–0.32201
7	9.110479	–7.56132	4.512561	–0.63366
8	0	2.124187	3.034116	–1.08806

Note that, for the 39 pure refrigerants studied in our previous work,^48^ apart from *n*-hexane in group 5 and ammonia in group 7, the remaining 37 fluids are all in group 3. Group 3 is the largest group containing 77 pure fluids mainly light hydrocarbons and halogenated hydrocarbons. With the new classification, a few additional experimental data and more comprehensive parameter fitting strategy, this study refitted the parameters of the 39 fluids, achieving a good consistency. Experimental data of each group collapse into the λ_res_^+^ vs s^+^/ξ curves are shown in Figure 3.

As shown in Tables 3 and 4, the first group-specific fitted parameter *n*_g1_ is zero for groups 5, 6, and 8, and the first fluid-specific fitted parameter *n*_1_ is also zero for many pure fluids. The first fitted parameters (*n*_1_ and *n*_g1_) being zero indicates the potential of constructing the RES model with simpler equations, i.e., one term less than eqs 5 and 6. One strategy is to optimize the functional form (e.g., exponents of *s*^+^) of eqs 5 and 6, which are, however, outside the scope of this study and will be explored in the future.

**Figure 2:**
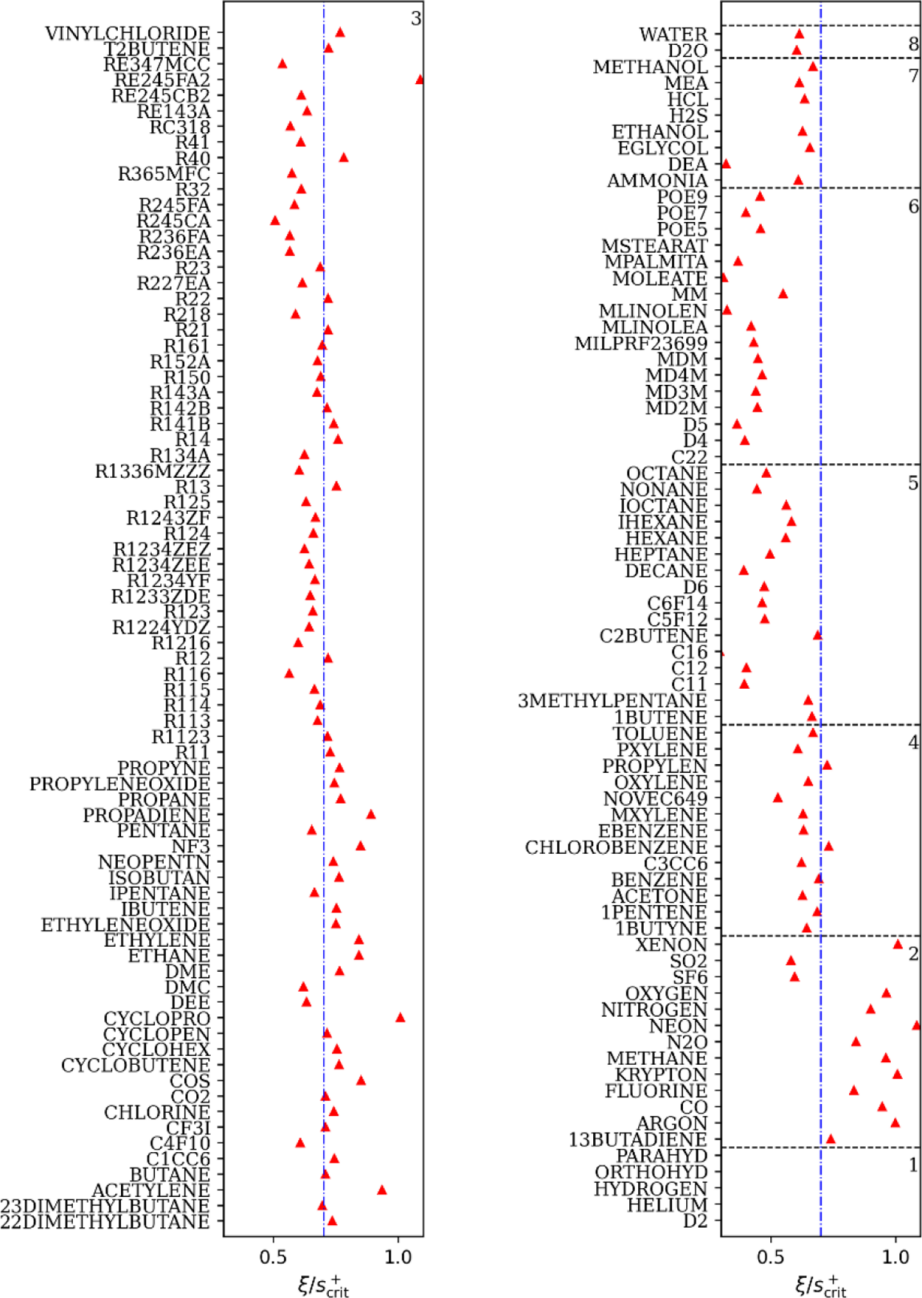
Scaling factor ξ. The denominator *s*_crit_^+^ is the plus-scaled dimensionless residual entropy at the critical point calculated with REFPROP 10.0^50^ of each pure fluid. The number at the top right of each box indicates the group number. The vertical dashed dotted line denotes ξ/*s*_crit_^+^ = 0.7. Values not shown in the figure: C16:0.29, C22:0.26, methyl stearate: 0.26, parahydrogen: 1.63, orthohydrogen: 1.61, hydrogen: 1.76, helium: 4.21, and deuterium (D2): 1.31. The names used in REFPROP 10.0 were adopted.

**Figure 3:**
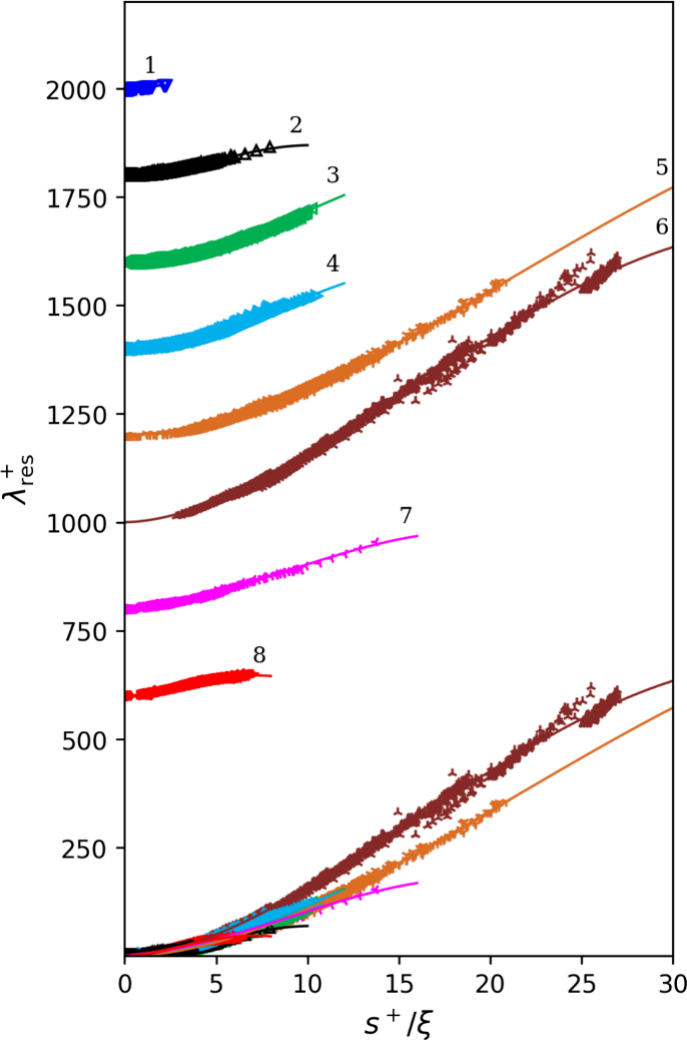
Values of λ_res_^+^ as a function of *s*^+^/ξ of each group of pure fluids, where λ_res_^+^ is the dimensionless residual thermal conductivity, *s*^+^ is the dimensionless residual entropy, and ξ is the scaling factor. The curves are calculated with the global *n*_gk_ parameters. All groups are shown at the bottom; at the top, each group is individually illustrated but stacked by powers of 20 and with group number labeled.

In our previous work, Yang et al.^49^ found a relation between the fluid-specific scaling factor ξ_μ_ of the viscosity’s RES model and the *s*_crit_^+^ (plus-scaled dimensionless residual entropy at the critical point). Subsequently, Jager et al.^81^ studied ξ_μ_ of long-chain alkane and applied the results to a new RES model.^82^ Inspired by this work on viscosity’s ξ_μ_, the scaling factor of the thermal conductivity was also analyzed in this work. Figure 2 shows how the scaling factor ξ of the thermal conductivity of each pure fluid varies with *s*_crit_^+^. The ξ/*s*_crit_^+^ of each pure fluid is similar in each group, and the value of the fluid ξ/*s*_crit_^+^ of Group 3, which contains the largest number of fluids, is roughly 0.7; this is consistent with the viscosity’s RES model of Yang et al.^49^ However, the scaling factor of the thermal conductivity model is more dispersed (for fluids in Group 3: the standard deviation of the viscosity scaling factor is 0.054, while the thermal conductivity scaling factor is 0.100). Experimental data of each group collapse into the individual global λ_res_^+^ vs *s*^+^/ξ curves are shown in Figure 3.

**Figure 4:**
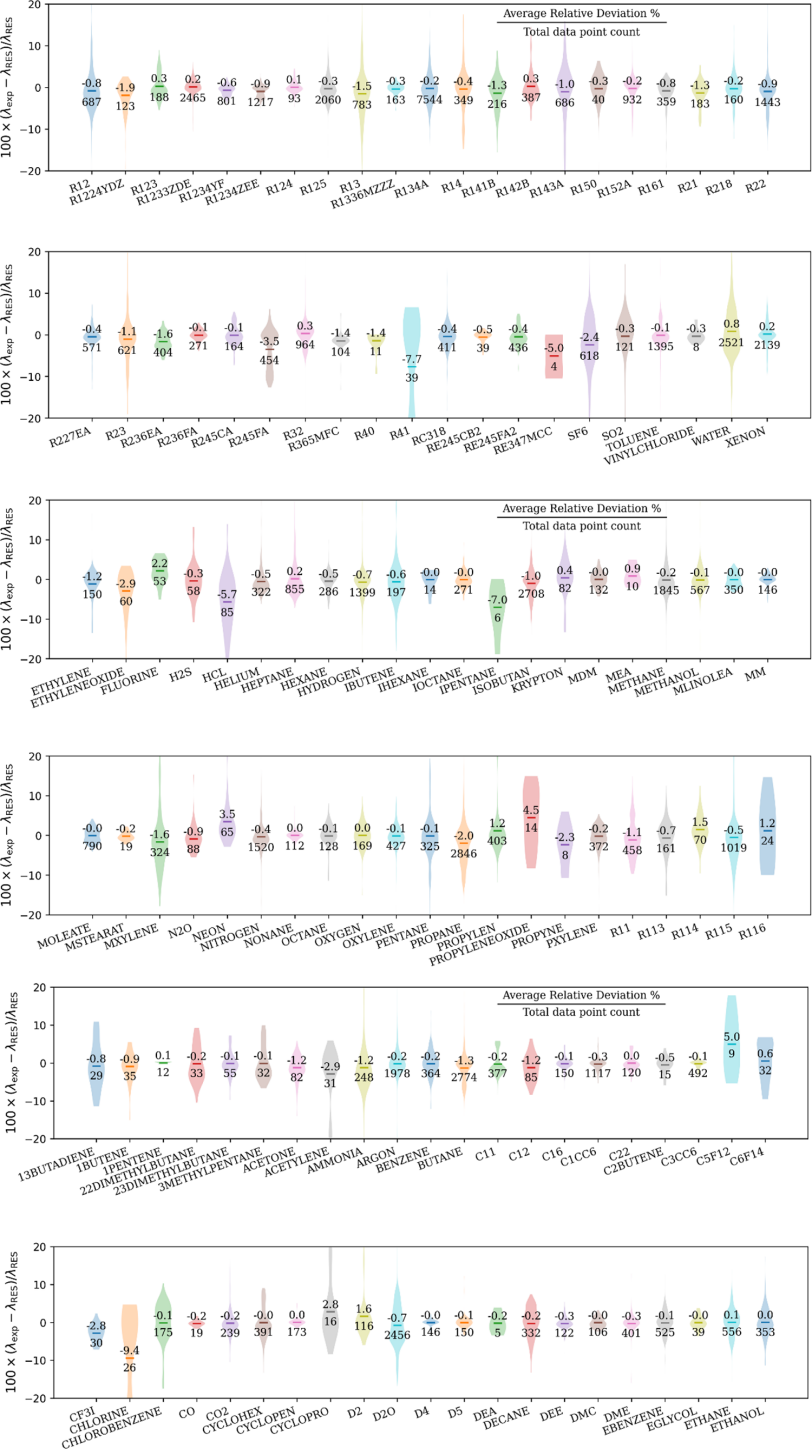
Relative deviations of the experimental thermal conductivity λ_exp_ from values λ_RES_ calculated with the RES model. The short line indicates the average relative deviation; the shape shows the distribution of the relative deviation; and the colors are for a clear illustration only. Fluid-specific *n*_k_ parameters are preferred, and only if they are not available in Table 3, global parameters *n*_gk_ are used.

Figure 4 shows the relative deviation of the experimental value λ_exp_ from the λ_RES_ calculated with the RES model; see SI-DPR for more detailed plots of each pure fluid. Please note that in calculating λ_RES_, the fluid-specific fitted parameter *n*_k_ is always preferred, while the global parameter *n*_gk_ is used only if *n*_k_ is not available in Table 3. The results showed that over 68.2% (corresponding to the standard deviation of a normal distribution) of the experimental data deviated from the RES model by less than 3.1%. To better reproduce some results of this work, a Python package implementing the developed RES method were provided in the SI, and the example calculations of each pure fluid and mixture are available in Table S6 and S7 in the SI. Here, we define the average relative deviation (ARD) of the experimental values λ_exp_ from model calculations and the average of the absolute value of the relative deviation (AARD) as
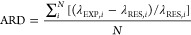
16
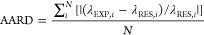
17where *N* is the total number of experimental data points of a given fluid, and the ARD and AARD represent the system offset and scatter from the experimental data to the model, respectively. ARD for each pure fluid is shown in Figure 4, and AARD is listed in Table S4 of the SI. Considering the presence of low-quality data and the uncertainties introduced by dilute-gas calculation, only 86 and 105 of the 125 pure fluids have ARD absolute values less than 1.0% and 2.0%, respectively. For fluids with larger ARD, the possible reasons are as follows: very few experimental data are available (RE347mcc: ARD = −5.0%, 4 points; isopentane: ARD = 4.9%, 6 points); the experimental data cover a limited range of temperature and pressure (neon: ARD = 3.5%, experimental data are available only in the gas phase, i.e., near *s*^+^ = 0); the dilute gas thermal conductivity is inaccurate (acetylene: ARD = −2.9%; R245fa: ARD = −3.5%); the consistency of experimental data was poor (R41: ARD = −7.7%). For the 18 pure fluids of which more than 1000 experimental data points are available, absolute values of ARD are mainly less than 1.5% except propane (ARD = −2.2%) and water (ARD = 1.7%). For the 61 fluids with more than 200 experimental data points, the absolute ARD of RES model compared to experimental data is less than 2.5% except R245FA (ARD = −3.5%) and R123 (ARD = 4.6%).

At last, the performance of the RES model was compared to the various recommended models implemented in REFPROP 10.0. The REFPROP models cannot calculate very few experimental data (0.10%, see Table 1). According to Table S4, the RES model shows smaller or equal AARD for 74 pure fluids out of 125 compared to REFPROP 10.0.

### 3.3 Prediction for Mixtures

**Figure 5:**
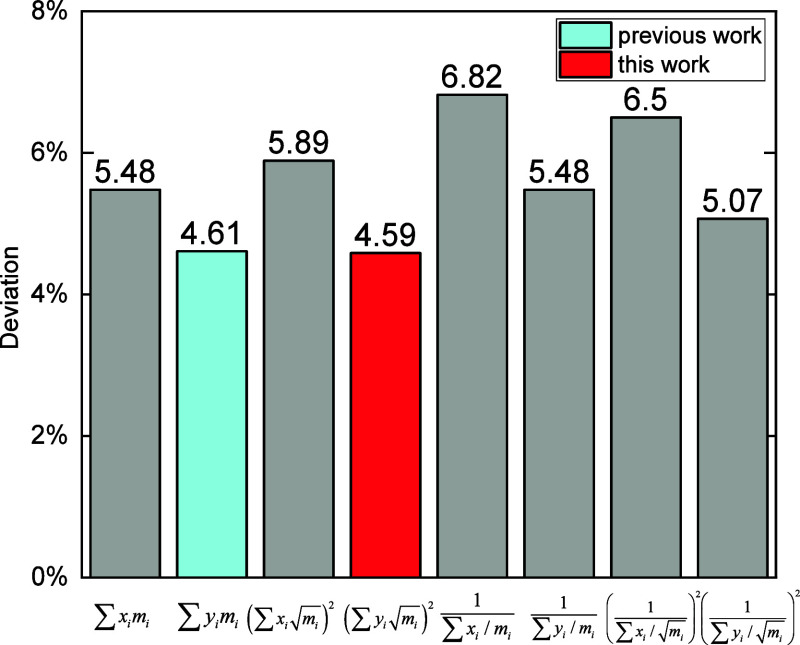
Influence of different mixing rules of for *m*_mix_ on the prediction of thermal conductivity of mixtures, where *x*_*i*_ represents the mole fraction, *y*_*i*_ represents the mass fraction, and *m*_*i*_ is the molecular mass for component *i*. Deviation represents the maximum deviation corresponding to the standard deviation of a normal distribution (≈68.2%) of the selected experiment data.

In this section, the mixing rules described in Section 2 were utilized to calculate thermal conductivity of mixtures, and the results were compared to experimental data. At first, various mixing rules for *m*_mix_ of mixtures in eq 3 were investigated. In the previous work,^49^*m*_mix_ was determined as the mass fraction weighted average . In this work, a few other mixing rules for *m*_mix_ were studied, see Figure 5, and as a result,  yielded the best performance. The slightly better performance of the mass fraction weighted average of  over *m* is consistent with the factor that  is in eq 3 rather than *m*.

**Figure 6:**
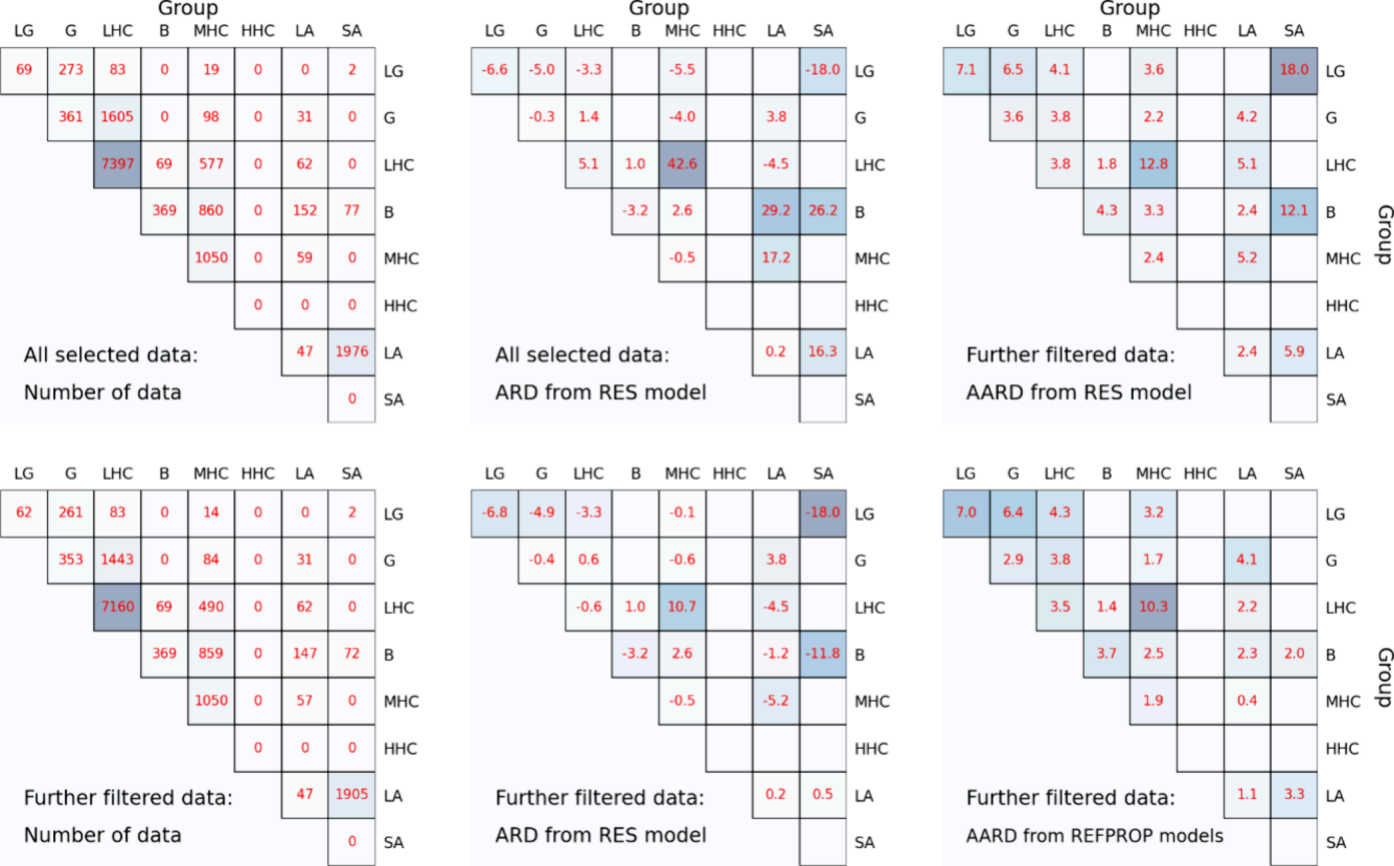
Statistical summary of the relative deviation of the experimental data from model calculations for binary mixtures. All selected data: all experimental data collected and passed the three filters; Further filtered data: experimental data further filtered so that they can be calculated with the REFPROP models. ARD and AARD: the average relative deviation and the average of the absolute value of relative deviation of the experimental values from the model calculations, respectively. Please note that the blank areas in the figure indicate either the absence of available data or the inability of REFPROP to perform calculations.

With the new mixing rule for *m*_mix_, over 68.2% of the well-evaluated experimental data (see Table 1) exhibit agreement with the RES model within 4.6%. The performance of the RES model varies for mixtures from different groups, see Figure 6. For group LHC + LHC (“group *a* + *b*″ denotes binary mixtures with one component from group *a* and another from *b*), 7210 filtered experimental data points are available, and the ARD is a mere −0.4%. For group G + LHC, the ARD is only 0.6% and there are 1,575 experimental data points. Of course, there are also bad cases, e.g., for group LHC + MHC, there are 490 data points while the ARD is as high as 11.1%. It should be noted that the relatively large ARD and AARD for group LG + LG (ARD = 6.6%, AARD = 6.8%) may be attributed, in part, to the limited availability of experimental data (only 69 points) and the failure of the RES methods for quantum fluids, as reported by Yang et al.^49^ Bell et al.^83^ proposed a simple empirical model that introduces a thermal length scale relative to the packing length scale  to modify the classical residual entropy, quantifying the impact of quantum effects on the residual entropy scaling. This approach will improve the accuracy of residual entropy scaling models for quantum fluids such as hydrogen. However, in this study, a consistent mathematical expression was pursued for all fluids, and thus this improvement was not adopted.

The performance of the RES model for the mixture prediction was also compared to the REFPROP models. Each binary mixture has up to four additional binary interaction parameters in the ECS model (the most used model in REFPROP), while the RES model developed in this work does not require any additional adjustable parameters. The REFPROP models failed to calculate approximately 12% of the well-evaluated experimental data (see Table 1), mainly for mixtures of water and alcohol, as detailed in Table S5. After filtering out these data, the statistical results of both the RES and REFPROP models compared to the experimental data are shown in Figure 6. Detailed information is given in the 164 figures of each mixture in the SI-DPR. According to Table S5 in the SI, the RES model yields equal or lower AARD for 76 of all 164 mixtures compared to REFPROP models. Overall, without introducing any adjustable parameters, the developed RES model achieved a similar level of agreement with mixture experimental data as the REFPROP models.

The deviation from experimental data to both the RES and the REFPROP models exhibit certain similarities. For group LHC + MHC, both models have an AARD exceeding 10%, and the deviations for the mixtures of group LG + LG and LG + G also surpass 6%, which all exceed the typical uncertainty (5%) of experimental thermal conductivity data. This reflects the limitations of existing models in mixture calculation and might also imply that higher-quality experimental data is needed for some mixture groups. For cyclohexane (group LG) + decane (group MHC),^84,85^ the ARD for the RES and REFPROP models are 14.8% and 12%, respectively, and for D_2_ (group LG) + H_2_ (group LG),^86−88^ both models exhibit significant negative deviations (RES: −7.7%, REFPROP: −7.6%). These indicate the need for adding adjustable parameters to the mixing rules to improve model performance, which will be our future work.

**Figure 7:**
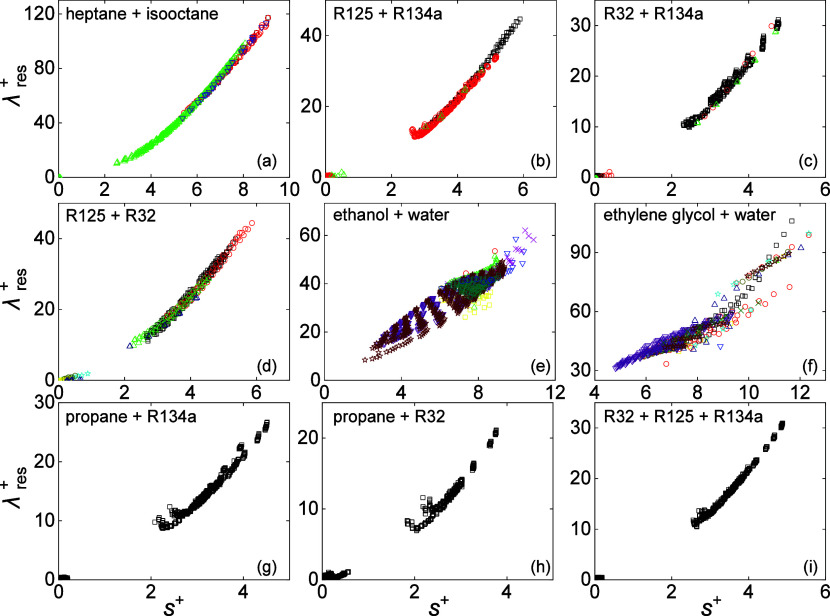
Relation between λ_res_^+^ and *s*^+^ for mixtures that have more than 500 experimental data points: (a) heptane + isooctane,^116−119^ (b) R125 + R134a,^102−104^ (c) R32 + R134a,^103,104^ (d) R125 + R32,^103,120−125^ (e) ethanol + water,^89−101^ (f) ethylene glycol + water,^97,105−115^ (g) propane + R134a,^104^ (h) propane + R32,^104^ and (i) R32 + R125 + R134a.^103^ Different colors and symbols represent experimental data from different sources. For specific literature information, please refer to SI-DPR.

To better illustrate the calculation results of mixtures, the *s*^+^ vs λ_res_^+^ plots of nine mixtures of which more than 500 experimental data were available, are illustrated in Figure 7. Figures of other mixtures are provided in the SI-DPR. Five mixtures have more than 1000 data (ethanol + water,^89−101^ R125 + R134a,^102−104^ R32 + R134a,^103,104^ propane + R32,^104^ and R32 + R125 + R134a^103^) and four mixtures have data between 500 and 1000 (ethylene glycol + water,^97,105−115^ heptane + isooctane,^116−119^ R125 + R32,^103,120−125^ and propane + R134a^104^). Among these 9 mixtures, for binary mixtures of heptane + isooctane, R125 + R134a, R32 + R134a, and R125 + R32, the *s*^+^ vs λ_res_^+^ characteristic lines are distinct, and the AARD of the experimental data from model prediction is less than 3%. The model prediction is worse for group LA + SA: the AARD is 5.4% for water + ethanol, and 6.3% for ethylene glycol + water. Especially, the maximum absolute deviation for water + ethanol and ethylene glycol + water exceeds 20% (21.2% and 23.7%, respectively). Cautions should be taken when using this model to calculate the thermal conductivity of fluids containing water and alcohols. For propane + R32 and propane + R134a, there are “no data” between *s*^+^ = 1 and 2, and the deviation from the *s*^+^ vs λ_res_^+^ characteristic line increases at *s*^+^ ≈ 2. This is because the experimental data in this range are either considered to be in the two-phase region by REFPROP 10.0 and thus filtered out by Filter 2, or have high deviation but not reaching the criteria of Filter 3. Data of three multicomponent mixtures: R32 + R125 + R134a,^103^ R32 + R1234yf + CO_2_,^103^ and R32 + R125 + R134a + R1234yf + CO_2_^103^ were also collected in this work. Without introducing additional adjustable parameters, the AARD of the RES model is 2.3%, 4.0%, and 1.5% for these mixtures, respectively.

The critical enhancement model proposed by Olchowy and Sengers^51^ was incorporated in many multiparameter thermal conductivity models for pure fluids, including propane^126^ and CO_2_^127^ and was successfully applied to refrigerants in our previous work.^48^ When the critical enhancement model was applied to mixtures in this work, a “strange behavior” was observed: For certain mixtures, the calculated critical term Δλ_C,mix_ at conditions far away from the critical point is less than zero, which is abnormal. Further investigation reveals that REFPROP 10.0 considered these experimental points in a two-phase region and yielded negative infinity value of specific heat capacities needed in eqs 7 and 8. To solve this problem, Δλ_C,mix_ was forced to be zero if it was calculated to be negative. As a result, the critical enhancement model proposed by Olchowy and Sengers^51^ obviously improved the accuracy of the model. Although there are a few cases where the deviation increases, the introduction of the critical enhancement term reduces the AARD of 29 mixtures, and the AARD of 7 mixtures is reduced by more than 2%. All mixtures with a change in AARD for more than 1% and with sufficient experimental data are shown in Table 5. It is worth mentioning that for CO_2_+ethane, the ARD decreases significantly from 10.2% to 1.7%.

**Table 5:** Mixtures for Which the Critical Enhancement Term Changes AARD by More than 1%

	with critical enhancement		without critical enhancement		
mixture name	ARD/%	AARD/%	ARD/%	AARD/%	AARD change/%
CO_2_ + ethane	1.7	5.8	10.2	10.9	5.1
helium + R14	–3.4	3.6	8	8	4.4
R14 + R22	1.7	5.9	8.1	9.4	3.5
CO_2_ + ethylene	–1.9	2.8	4.1	5.5	2.7
argon + R14	–0.7	3	4.8	5.1	2.1
R143a + R1234yf	0.6	1.7	2.9	3.5	1.8
R134a + R1234ze(E)	1.6	5.7	3.2	7.3	1.6
R143a + R1234ze(E)	–0.8	2.7	0.8	4.1	1.4
R125 + R1234ze(E)	0	2.4	1.3	3.4	1
R152a + R218	–8.2	10.2	–7	9.2	–1
R125 + R143a	–8.4	8.4	–4.6	5.5	–2.9

**Figure 8:**
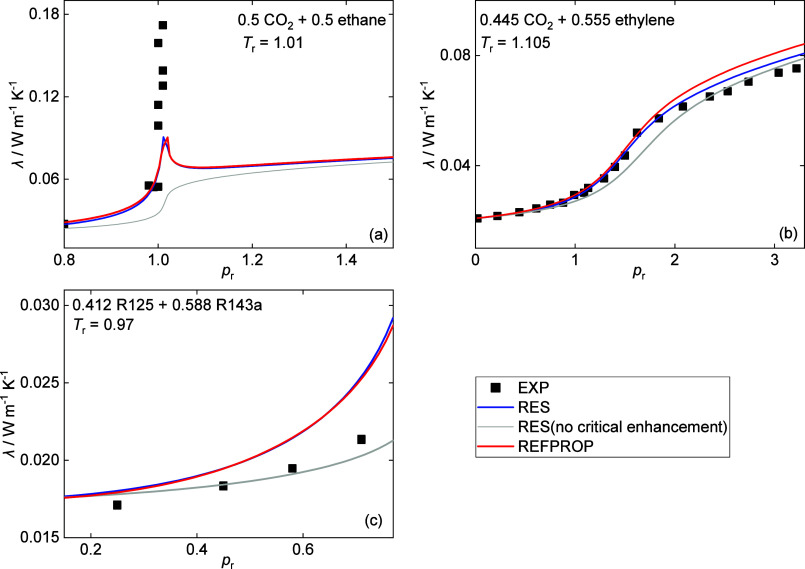
Thermal conductivity experimental data and model prediction of some fluid mixtures with temperature close to the critical point, where *p*_r_ = *p*/*p*_c_ is the reduced pressure relative to critical pressure: (a) CO_2_ + ethane,^128^ (b) CO_2_ + ethylene,^129^ and (c) R125 + R143a.^121^ The molar fraction of each component is indicated. The critical temperature and pressure were calculated with REFPROP 10.0.

Three fluid mixtures were selected to show the impact of the critical enhancement term with their λ vs *p*_r_ plots shown in Figure 7. For CO_2_ + ethane^128^ and CO_2_ + ethylene,^129^ the thermal conductivity values predicted by the RES model without the critical enhancement term are generally lower than the experimental data, while those with the critical enhancement term are generally closer to the experimental data. Of course, the critical enhancement model of Olchowy and Sengers^51^ is not perfect: (1) As shown by the data of CO_2_ + ethane^129^ in Figure 8a, the peak values of experiment data cannot be predicted by the model; (2) the AARD increases for some mixtures (e.g., R125 + R143a,^121^ see Figure 8c) after introducing the critical term, likely due to the overcorrection of critical effects by the critical term.

## 4 Conclusion, Discussion, and Future Work

In this work, we collected experimental thermal conductivity data for 125 pure fluids and 164 mixtures and developed a residual entropy scaling (RES) model for thermal conductivity. Using a simple polynomial equation, this method uses reference equations of state (EOS) in REFPROP 10.0 to calculate the residual entropy and link residual thermal conductivity to residual entropy. The 125 fluids were classified into 8 different groups, and data of each group collapsed into a characterized residual thermal conductivity vs residual entropy curve. More than 68.2% (corresponding to a standard deviation of a normal distribution) of the evaluated experimental data agree with the RES model within 3.1% for pure fluids. Compared to the various models implemented in REFPROP 10.0, the RES model provides a smaller or equal AARD for 74 out of 125 pure fluids, indicating a similar accuracy level with fewer adjustable parameters. The accuracy of the RES model is affected by the accuracy of the residual entropy, and this implies that the accuracy of our model can be further improved with updates to the reference EOS in REFPROP.

Using simple mixing rules without adjustable parameters, more than 68.2% of the well-evaluated experimental data agree with the RES model within 4.6% for mixtures. The mixing rules proposed in this work lead to a promising result for mixtures of the same group. The situation is different for mixtures from different groups, which may be related to the degree of asymmetry within the system. Nevertheless, without introducing new parameters, the RES model yielded a lower AARD than the REFPROP model for 76 of the 164 mixtures. Further refinement of the mixing rules for different pairs of fluid properties will be carried out to achieve better prediction results.

## Supporting Information Available

Tables of dilute gas calculation parameters of pure fluids; of reference equation of state and the recommended thermal conductivity model in REFPROP 10.0; of names, group, and constants of pure fluids; for statistics of the experimental data of pure fluids; for statistics of the experimental data of fluid mixtures; for sample thermal conductivity calculations of pure substances with the recommended models in REFPROP 10.0 and the RES model; for sample thermal conductivity calculations of binaries with the recommended models in REFPROP 10.0 and the RES model; and for thermal conductivity as a function of residual entropy for each group and figures for relative deviation from experimental data of pure fluids to model predictions and relative deviation from experimental data of fluid mixtures to model predictions (PDF)
Python package for residual entropy scaling model calculation (ZIP)
Detailed plots and references of all collected experimental data (PDF)
