# Supplementary material for: Linking Thermal Conductivity to Equations of State Using the Residual Entropy Scaling Theory
Source: Ind Eng Chem Res. 2024 Oct 15;63(42):18160–75. doi: 10.1021/acs.iecr.4c02946 (PMC11503615; doi:10.1021/acs.iecr.4c02946)
Supplement: Supplementary file 1 — ie4c02946_si_001.pdf [file ie4c02946_si_001.pdf]

Supporting Information

Linking Thermal Conductivity to Equations of State Using Residual Entropy Scaling Theory

Zhuo Li[1], Yuanyuan Duan[*1,2], Xiaoxian Yang[†3]

[1] *Key Laboratory for Thermal Science and Power Engineering of Ministry of Education, Beijing Key Laboratory for CO_2 Utilization and Reduction Technology, Tsinghua University, Beijing 100084,People's Republic of China*

[2] *Southwest United Graduate School，Kunming 650092, People's Republic of China*

[3] *Chemnitz University of Technology, Applied Thermodynamics, 09107 Chemnitz, Germany*

[*] Corresponding author. Yuanyuan Duan. Email address: yyduan@tsinghua.edu.cn. ORCID ID: https://orcid.org/0000-0002-4117-7545.

[†] Corresponding author. Xiaoxian Yang. Email address: xiaoxian.yang@mb.tu-chmenitz.de. ORCID ID: https://orcid.org/0000-0003-4655-3156.

1. Python package for residual entropy scaling model calculation

1. code_SI.py: the python code for thermal conductivity calculation of pure fluids and mixtures based on residual entropy scaling and REFPROP 10.0 [1]

2. Fluid_Constants.txt: fluid constant mainly obtained from REFPROP 10.0

3. RES_Parameter.txt: the residual entropy scaling parameters obtained in this work.

4. Samples_pure_fluids.txt: input data file with pure fluid samples

5. Samples_binaries.txt: input data file with mixture samples

6. Table_S5_SI.txt: output file for Table S

7. Table_S6_SI.txt: output file for Table S6

2. List of Tables

Table S1 Dilute gas calculation parameters and their effective temperature range (from REFPROP 10.0) of pure fluids.

$\lambda_0(Wm^{-1}K^{-1}) = n_0(T(K))^4 + n_1(T(K))^3 + n_2(T(K))^2 + n_3(T(K)) + n_4$

REFPROP fluid name	T_{min}/K	T_{max}/K	n_0	n_1	n_2	n_3	n_4
13BUTADIENE	164.25	426	-1.446579E-14	-2.270800E-10	3.449043E-07	-4.672394E-05	5.105997E-03
1BUTENE	87.8	525	-3.170603E-13	4.112662E-10	3.394034E-09	2.182551E-05	9.674438E-05
1BUTYNE	147.44	432	6.684582E-13	-1.043248E-09	6.371508E-07	-7.655634E-05	5.737486E-03
1PENTENE	107.797	466	-3.602263E-13	3.536089E-10	4.152410E-08	7.725935E-06	1.173560E-03
22DIMETHYLBUTANE	174.2	1000	1.414883E-13	-3.943701E-10	4.242909E-07	-7.412797E-05	7.767641E-03
23DIMETHYLBUTANE	145.05	1000	8.512754E-14	-2.862246E-10	3.524699E-07	-5.679339E-05	6.473059E-03
3METHYLPENTANE	110.263	1000	7.018218E-14	-2.551391E-10	3.302141E-07	-4.398613E-05	4.569371E-03
ACETONE	178.5	1000	6.888268E-15	-8.068206E-11	2.008444E-07	-3.073848E-05	5.233615E-03
ACETYLENE	191.75	310	-4.314537E-13	2.964310E-10	7.440059E-08	3.330753E-05	7.300000E-04
AMMONIA	195.49	725	8.186185E-14	-2.102747E-10	2.941563E-07	-2.081390E-05	9.912748E-03
ARGON	83.806	2000	-3.007171E-15	1.745872E-11	-4.080949E-08	7.000342E-05	-7.837994E-05
BENZENE	278.674	1000	8.887965E-14	-2.787443E-10	3.138950E-07	-4.188019E-05	1.770862E-03
BUTANE	134.895	575	2.937669E-27	-4.461514E-24	1.603703E-07	2.294908E-06	1.626760E-03
C11	247.606	1000	1.630766E-13	-4.119738E-10	4.552407E-07	-1.198141E-04	1.299558E-02
C12	263.6	1000	-1.223197E-27	-1.023633E-10	2.129770E-07	-4.012369E-05	4.363430E-03
C16	291.329	800	1.947177E-14	-1.074637E-10	2.270348E-07	-6.635676E-05	8.323227E-03
C1CC6	146.7	600	-2.866772E-28	-6.927197E-11	2.222198E-07	-3.157393E-05	2.899680E-03
C22	317.04	1000	4.405121E-14	-1.627030E-10	2.116061E-07	-4.369200E-05	5.640033E-03
C2BUTENE	134.3	525	-2.715026E-13	2.825037E-10	5.070087E-08	1.072954E-05	1.219088E-03
C3CC6	178.2	650	1.479505E-27	-1.518772E-10	3.473260E-07	-9.667549E-05	1.074020E-02
C4F10	144	450	4.352570E-13	-6.257327E-10	3.037339E-07	-4.254549E-06	2.956272E-04
C5F12	148.21	500	3.129941E-13	-5.347595E-10	3.282499E-07	-2.869350E-05	2.412697E-03
C6F14	187.07	450	3.063405E-13	-4.822182E-10	2.734682E-07	-1.244328E-05	9.573030E-04
CF3I	195.15	420	2.223369E-13	-3.452409E-10	1.976765E-07	-1.028319E-05	5.988733E-04
CHLORINE	172.17	440	3.631463E-13	-5.387268E-10	2.764271E-07	-5.913013E-06	1.858511E-03
CHLOROBENZENE	227.9	700	1.559002E-13	-4.269825E-10	4.390178E-07	-8.284202E-05	7.771745E-03
CO	68.16	500	3.894816E-14	-4.140323E-12	-6.241220E-08	1.057469E-04	-1.304719E-03
CO2	216.592	2000	4.004284E-15	-1.789454E-11	1.587771E-08	7.555339E-05	-6.818247E-03
COS	134.3	650	1.362067E-13	-2.697845E-10	1.887821E-07	-1.355896E-06	1.439718E-03
CYCLOBUTENE	150	448	-1.616573E-14	-1.009688E-10	2.493571E-07	-3.181525E-05	3.713137E-03
CYCLOHEX	279.86	700	4.352992E-14	-2.401352E-10	3.848030E-07	-8.199033E-05	7.136052E-03

REFPROP fluid name	T_{min}/K	T_{max}/K	n_0	n_1	n_2	n_3	n_4
CYCLOPEN	179.7	550	-1.921056E-13	7.899387E-11	2.286610E-07	-4.902080E-05	4.811556E-03
CYCLOPRO	273	473	2.843419E-13	-7.635576E-10	7.360410E-07	-1.610931E-04	1.608570E-02
D2	18.724	1000	8.439859E-15	2.141693E-10	-4.450482E-07	5.550860E-04	-1.387883E-03
D2O	254.415	825	-4.692355E-14	8.469276E-11	1.661214E-08	4.843594E-05	3.045985E-04
D4	290.25	590	5.282446E-14	-2.323507E-10	2.951541E-07	-6.521638E-05	9.623381E-03
D5	226	630	-9.692191E-14	8.576987E-11	5.450005E-08	1.453923E-07	4.085299E-03
D6	270.2	673	1.614875E-13	-3.715117E-10	3.325556E-07	-7.071660E-05	8.767948E-03
DEA	301.1	740	1.490352E-13	-3.863015E-10	3.569242E-07	-4.088182E-05	7.556335E-04
DECANE	243.5	1000	2.477876E-27	-1.580250E-10	3.118280E-07	-8.329772E-05	1.055430E-02
DEE	270	500	4.149997E-14	-1.614424E-10	2.191932E-07	-6.365181E-06	2.015129E-03
DMC	277.06	600	1.666159E-13	-4.865324E-10	5.006185E-07	-1.132936E-04	1.463099E-02
DME	131.66	525	-1.931083E-12	1.635713E-09	-2.879544E-07	5.677840E-05	-2.051678E-03
EBENZENE	178.2	700	4.184186E-14	-2.024695E-10	3.047963E-07	-5.894244E-05	5.099277E-03
EGLYCOL	260.6	750	2.870071E-13	-7.347210E-10	6.799705E-07	-1.287968E-04	9.399708E-03
ETHANE	90.368	675	-3.477381E-13	3.822604E-10	4.894296E-08	3.254114E-05	-6.809238E-04
ETHANOL	159	650	3.422529E-14	-2.039507E-10	2.964835E-07	-3.422853E-05	4.224988E-03
ETHYLENE	103.986	450	-1.103485E-12	1.215752E-09	-2.487656E-07	7.117533E-05	-1.847254E-03
ETHYLENEOXIDE	160.654	500	-4.615713E-13	6.641448E-10	-1.288406E-07	3.025611E-05	7.066490E-04
FLUORINE	53.481	300	7.981334E-13	-5.474502E-10	9.842400E-08	8.302545E-05	-3.044798E-05
H2S	187.7	760	1.141410E-14	-3.041718E-11	5.136678E-08	3.338742E-05	3.263586E-04
HCL	159.07	670	5.854284E-14	-9.313697E-11	3.631510E-08	4.594933E-05	-1.219832E-04
HELIUM	2.177	2000	-3.378269E-14	1.709281E-10	-3.347972E-07	5.381719E-04	1.925738E-02
HEPTANE	182.55	600	-7.592322E-14	-2.402868E-11	2.048821E-07	-2.832319E-05	3.420013E-03
HEXANE	177.83	600	1.934599E-26	-1.402855E-10	2.792585E-07	-4.679197E-05	6.674200E-03
HYDROGEN	13.957	1000	-1.078782E-13	5.038431E-10	-7.362058E-07	8.042707E-04	-2.724009E-03
IBUTENE	132.4	550	-2.501422E-13	2.675137E-10	7.369216E-08	1.108642E-05	1.089518E-03
IHEXANE	119.6	1000	4.988978E-14	-2.065669E-10	2.818064E-07	-3.185402E-05	3.202536E-03
IOCTANE	165.77	1000	4.579541E-14	-1.842189E-10	2.710897E-07	-4.427065E-05	5.249852E-03
IPENTANE	112.65	500	-2.593054E-13	2.562816E-10	9.999762E-08	3.646188E-06	-2.839119E-04
ISOBUTAN	113.73	575	-4.919930E-27	6.704626E-24	1.297396E-07	2.613731E-05	-2.379010E-03
KRYPTON	115.775	750	3.581024E-15	3.894394E-12	-2.234788E-08	4.015235E-05	-7.121606E-04
MD2M	205.2	600	2.388411E-13	-4.754767E-10	3.667881E-07	-5.928731E-05	5.647597E-03
MD3M	192	630	-1.142598E-13	1.756977E-10	-6.053663E-08	5.538927E-05	-5.478040E-03
MD4M	214.15	655	-9.586053E-14	1.480493E-10	-4.860153E-08	4.975794E-05	-4.974525E-03
MDM	187.2	570	9.783670E-15	-1.544373E-10	2.340248E-07	-4.032621E-05	6.283575E-03

REFPROP fluid name	T_{min}/K	T_{max}/K	n_0	n_1	n_2	n_3	n_4
MEA	283.7	675	-4.502464E-17	-2.270825E-11	7.393247E-08	4.488977E-05	-6.446587E-03
METHANE	90.694	625	-5.465157E-13	8.143846E-10	-2.961510E-07	1.565373E-04	-3.662997E-03
METHANOL	175.61	620	-2.118939E-13	2.463249E-10	4.154415E-08	1.761727E-05	1.296266E-03
MILPRF23699	250	1000	2.128050E-14	-8.189394E-11	1.112245E-07	-1.569167E-05	1.690991E-03
MLINOLEA	238.1	1000	8.125098E-28	-2.076684E-11	6.381005E-08	3.010551E-06	-1.090420E-04
MLINOLEN	218.65	1000	1.896867E-28	-1.961038E-11	5.876685E-08	3.359650E-06	-2.712500E-04
MM	204.93	580	2.069178E-13	-4.626304E-10	3.990619E-07	-6.735081E-05	7.961889E-03
MOLEATE	253.47	1000	1.418925E-28	-1.886765E-11	5.727347E-08	3.316688E-06	-2.712500E-04
MPALMITA	302.71	1000	7.196024E-29	-2.096511E-11	6.144309E-08	3.435298E-06	-2.712500E-04
MSTEARAT	311.84	1000	1.926140E-27	-1.938353E-11	5.831276E-08	3.346645E-06	-2.712500E-04
MXYLENE	225.3	700	1.035446E-13	-2.845340E-10	3.411402E-07	-6.585389E-05	5.350607E-03
N2O	182.33	525	1.299067E-13	-3.126380E-10	2.509262E-07	2.003244E-06	1.723866E-03
NEON	24.556	725	-3.046444E-13	6.061412E-10	-4.717322E-07	2.600919E-04	-1.659178E-04
NEOPENTN	256.6	550	9.994349E-14	-2.452138E-10	2.602602E-07	-2.530937E-06	-1.163970E-03
NF3	0	0	-4.867347E-15	2.699197E-11	-5.774697E-08	1.010248E-04	-6.962657E-05
NITROGEN	63.151	2000	-4.867347E-15	2.699197E-11	-5.774697E-08	1.010248E-04	-6.962657E-05
NONANE	219.7	1000	3.128323E-28	-1.522749E-10	2.964471E-07	-6.955008E-05	8.787650E-03
NOVEC649	165	500	-2.725419E-13	3.360550E-10	-9.252747E-08	5.992749E-05	-4.638961E-03
OCTANE	216.37	1000	-1.395763E-27	-1.564545E-10	3.016056E-07	-6.518970E-05	7.729300E-03
ORTHOHYD	0	0	-1.078782E-13	5.038431E-10	-7.362058E-07	8.042707E-04	-2.724009E-03
OXYGEN	54.361	2000	-3.749964E-15	2.127655E-11	-4.803376E-08	1.027844E-04	-7.577039E-04
OXYLENE	247.985	700	2.430512E-13	-5.815481E-10	5.049805E-07	-9.355817E-05	1.002834E-02
PARAHYD	13.803	1000	-1.002522E-12	2.462028E-09	-2.128616E-06	1.133804E-03	-9.813236E-03
PENTANE	143.47	650	-1.441618E-13	9.381576E-11	1.613734E-07	-9.992367E-06	1.770742E-03
POE5	250	1000	2.347560E-14	-9.075954E-11	1.234715E-07	-1.664810E-05	1.480545E-03
POE7	250	1000	1.068632E-14	-6.354532E-11	1.273343E-07	-3.420648E-05	5.029386E-03
POE9	250	1000	-2.320769E-14	1.115591E-11	7.711321E-08	-2.258616E-05	4.258831E-03
PROPADIENE	136.65	400	-7.415030E-13	6.244777E-10	-4.393187E-08	3.304515E-05	3.875437E-04
PROPANE	85.525	650	2.784026E-27	-4.412509E-24	1.457529E-07	2.207303E-05	-1.247780E-03
PROPYLEN	87.953	1000	4.258880E-14	-1.923241E-10	2.770612E-07	-1.445427E-05	1.905776E-03
PROPYLENEOXIDE	161.244	489	-3.243956E-13	2.547070E-10	9.110114E-08	-1.473019E-07	2.262462E-03
PROPYNE	273	474	1.633401E-13	-3.378457E-10	2.748060E-07	-8.798028E-07	8.747891E-04
PXYLENE	286.4	700	1.968717E-13	-5.082104E-10	5.078682E-07	-1.127406E-04	1.079917E-02
R11	162.68	625	8.424996E-14	-1.799675E-10	1.317443E-07	1.434339E-06	3.747591E-04
R1123	200	480	-1.713968E-13	9.035651E-11	8.336344E-08	2.535510E-05	-3.135782E-04

REFPROP fluid name	T_{min}/K	T_{max}/K	n_0	n_1	n_2	n_3	n_4
R113	236.93	525	1.807431E-26	-2.871458E-23	2.263013E-08	3.808559E-05	-5.069020E-03
R114	273.15	507	1.333200E-14	-4.995926E-11	3.931774E-08	3.795320E-05	-2.832476E-03
R115	173.75	550	2.028240E-13	-4.093053E-10	2.973809E-07	-2.654390E-05	2.976812E-03
R116	173.1	425	-4.194774E-26	5.602712E-23	-2.698017E-20	8.410311E-05	-1.105104E-02
R12	116.099	525	1.327774E-13	-2.658386E-10	1.846254E-07	-3.929380E-06	6.740249E-04
R1216	117.654	400	-8.177206E-14	-9.342469E-11	1.770389E-07	7.705730E-06	-4.595398E-05
R1224YDZ	263	473.15	2.144612E-13	-3.798121E-10	2.316692E-07	-6.391251E-07	-1.450766E-03
R123	166	600	-3.518673E-27	5.894425E-24	-3.623695E-21	5.695000E-05	-7.780000E-03
R1233ZDE	195.15	550	-1.376448E-26	2.207584E-23	-1.272105E-08	8.602366E-05	-1.400330E-02
R1234YF	122.77	410	3.268969E-26	-3.696355E-23	6.360357E-09	7.913497E-05	-1.027780E-02
R1234ZEE	169	420	2.520074E-26	-2.825278E-23	1.574259E-09	8.076153E-05	-1.035890E-02
R1234ZEZ	238	440	2.595524E-13	-6.199043E-10	5.131946E-07	-9.569927E-05	9.743027E-03
R124	120	470	7.770208E-14	-1.434891E-10	1.640257E-07	-8.417876E-07	7.082782E-04
R1243ZF	200	430	2.651625E-13	-4.686866E-10	3.352146E-07	-2.026076E-05	6.297902E-04
R125	172.52	500	-3.624304E-27	3.880024E-24	4.245064E-08	4.973509E-05	-4.608200E-03
R13	92	403	8.134189E-14	-2.156784E-10	1.887187E-07	1.632252E-07	5.284880E-04
R1336MZZZ	182.65	500	2.218639E-13	-3.955305E-10	2.917586E-07	-1.980649E-05	1.077141E-04
R134A	169.85	455	-2.822913E-26	3.607363E-23	-1.701545E-20	8.009820E-05	-1.052480E-02
R14	120	623	5.960945E-13	-9.510198E-10	5.502591E-07	-5.707339E-05	4.628527E-03
R141B	169.68	500	-2.441943E-13	3.050011E-10	1.068073E-09	1.288142E-05	-1.881570E-04
R142B	142.72	470	-1.919692E-14	-6.681814E-11	1.545094E-07	-5.046204E-06	9.689613E-04
R143A	161.34	650	8.805137E-27	-1.502978E-23	2.624990E-08	6.563070E-05	-7.008520E-03
R150	237.52	600	-1.206252E-13	1.514638E-10	9.403677E-09	1.001321E-05	2.330330E-03
R152A	154.56	500	-1.191481E-26	1.645919E-23	-8.325823E-21	9.732830E-05	-1.494200E-02
R161	130	450	5.375960E-12	-6.947524E-09	3.338240E-06	-5.886753E-04	3.591928E-02
R21	200	473	3.798563E-14	-1.428188E-10	1.453221E-07	-2.710985E-06	1.270925E-03
R218	125.45	440	-2.747276E-13	2.015124E-10	7.707831E-08	8.172358E-06	-2.662632E-04
R22	115.73	550	-4.581509E-14	3.488730E-12	1.001585E-07	3.573282E-06	8.509250E-04
R227EA	146.35	475	-1.102605E-13	-2.127425E-11	1.340348E-07	9.630985E-06	-3.226459E-05
R23	118.02	475	3.252495E-15	-3.871619E-12	1.610835E-09	5.338707E-05	-2.521091E-03
R236EA	240	420	1.228426E-12	-2.350487E-10	-2.207421E-07	1.187023E-04	-4.939200E-03
R236FA	179.6	400	1.683830E-13	-1.787694E-10	2.021441E-07	-1.376212E-05	2.128249E-03
R245CA	196	450	1.125505E-13	-2.918924E-10	3.638423E-07	-6.104317E-05	5.799944E-03
R245FA	170	440	-7.349843E-27	7.544373E-24	-3.261923E-21	9.069160E-05	-1.436440E-02
R32	136.34	435	4.675106E-27	-4.668571E-24	2.061070E-07	-5.528007E-05	1.065480E-02

REFPROP fluid name	T_{min}/K	T_{max}/K	n_0	n_1	n_2	n_3	n_4
R365MFC	239	500	7.000961E-13	4.410871E-10	-5.823973E-07	2.120361E-04	-1.505491E-02
R40	230	630	-3.201843E-14	3.721611E-11	1.061092E-07	-1.104760E-05	3.617173E-03
R41	129.82	425	-7.743300E-13	9.038667E-10	-2.766208E-07	8.527925E-05	-1.553647E-03
RC318	233.35	623	1.038546E-13	-2.607043E-10	2.279920E-07	-1.213263E-05	1.394366E-03
RE143A	240	420	9.707976E-14	-2.475253E-10	2.278598E-07	-7.781653E-06	1.452999E-03
RE245CB2	250	500	2.136963E-14	-1.233442E-10	1.519755E-07	4.675177E-06	6.902954E-04
RE245FA2	250	1000	1.740371E-13	-4.212328E-10	2.909188E-07	-2.026740E-05	3.304479E-03
RE347MCC	150.65	500	3.350917E-13	-7.215420E-10	5.012676E-07	-5.462600E-05	5.181439E-16
SF6	223.555	625	2.305983E-13	-4.475096E-10	2.943511E-07	-6.770954E-06	-1.152813E-03
SO2	197.7	525	-1.791568E-14	-1.190900E-11	5.740966E-08	1.359594E-05	9.674486E-04
T2BUTENE	167.6	525	-1.867496E-13	1.513589E-10	1.139464E-07	2.559678E-06	1.961285E-03
TOLUENE	178	700	8.191459E-14	-2.647787E-10	3.256451E-07	-5.866100E-05	5.661607E-03
VINYLCHLORIDE	190	450	-2.071018E-13	2.356304E-10	2.176537E-08	1.177141E-05	8.066188E-04
WATER	273.16	2000	6.286152E-15	-4.166540E-11	1.073151E-07	1.995105E-05	3.879285E-03
XENON	161.405	750	1.367932E-14	-2.397535E-11	8.219222E-09	1.810778E-05	-1.401728E-04

Table S2 Reference equation of state (EoS) and the recommended thermal conductivity model in REFPROP 10.0[1]

REFPROP fluid name and reference EOS [a]	Recommended viscosity model in REFPROP 10.0[1]
13BUTADIENE[1]	ECS model[2]
1BUTENE[3]	ECS model[2]
1PENTENE[1]	ECS model[2]
22DIMETHYLBUTANE[4]	ECS model[2]
23DIMETHYLBUTANE[4]	ECS model[2]
3METHYLPENTANE[4]	ECS model[2]
ACETONE[5]	ECS model[2]
ACETYLENE[1]	ECS model[2]
AMMONIA[1]	Reference correlation of Monogenidou et al. [6]
ARGON[7]	Reference correlation of Lemmon and Jacobsen [8]
BENZENE[9]	Reference correlation of Assael et al.[10]
BUTANE[11]	Reference correlation of Perkins et al.[12]
C11[13]	Reference correlation of Assael et al.[14]
C12[15]	Reference correlation of Huber et al.[16]
C16[17]	Reference correlation of Monogenidou et al. [18]
C1CC6[1]	Reference correlation of Perkins et al.[19]
C2217	ECS model[2]
C2BUTENE3	ECS model[2]
C3CC6[1]	Reference correlation of Perkins et al.[20]
C5F12[21]	ECS model[2]
C6F14[21]	ECS model[2]
CF3I[22]	ECS model[2]
CHLORINE[23]	ECS model[2]
CHLOROBENZENE[1]	ECS model[2]
CO[5]	ECS model[2]
CO2[24]	Reference correlation of Huber et al.[27]
CYCLOHEX[28]	Reference correlation of Koutian et al.[29]
CYCLOPEN[30]	Reference correlation of Vassiliou et al.[31]
CYCLOPRO[32]	ECS model[2]
D2[33]	Reference correlation of Assael et al.[34]
D2O[35]	Reference correlation.[36]
D4[37]	ECS model[2]
D5[38]	ECS model[2]
DEA[39]	ECS model[2]
DECANE[5]	Reference correlation of Huber and Perkins [40]
DEE[41]	ECS model[2]
DMC[42]	ECS model[2]
DME[43]	ECS model[2]
EBENZENE[44]	Reference correlation of Mylona et al.[45]
EGLYCOL[1]	ECS model[2]
ETHANE[45]	Reference correlation of Bücker and Wagner [46]
ETHANOL[47]	Reference correlation of Assael et al.[48]
ETHYLENE[49]	Reference correlation of Assael et al.[50]
ETHYLENEOXIDE[51]	ECS model[2]
FLUORINE[52]	ECS model[2]
H2S[5]	ECS model[2]
HCL[53]	

HELIUM[54]	Reference correlation of Hands and Arp[55]
HEPTANE[1]	Reference correlation of Assael et al.[56]
HEXANE[1]	Reference correlation of Assael et al.[57]
HYDROGEN[58]	Reference correlation of Assael et al.[34]
IBUTENE[3]	ECS model[2]
IHEXANE[5]	ECS model[2]
IOCTANE[1]	ECS model[2]
IPENTANE[5]	Reference correlation of Vassiliou et al.[31]
ISOBUTAN[11]	Reference correlation of Perkins [59]
KRYPTON[5]	ECS model[2]
MDM[60]	ECS model[2]
MEA[1]	ECS model[2]
METHANE[61]	Reference correlation of Friend et al.[62]
METHANOL[63]	Reference correlation of Sykioti et [al64]
MLINOLEA[65]	Reference correlation of Perkins and Huber[66]
MM[67]	ECS model[2]
MOLEATE[65]	Reference correlation of Perkins and Huber[66]
MSTEARAT[65]	ECS model[2]
MXYLENE[44]	Reference correlation of Mylona et al.[45]
N2O[5]	ECS model[2]
NEON[1]	ECS model[2]
NITROGEN[68]	Reference correlation of Lemmon and Jacobsen [8]
NONANE[5]	Reference correlation of Huber and Perkins [40]
OCTANE[1]	Reference correlation of Huber and Perkins [40]
OXYGEN[69]	Reference correlation of Lemmon and Jacobsen [8]
OXYLENE[44]	Reference correlation of Mylona et al.[45]
PENTANE[1]	Reference correlation of Vassiliou et al.[31]
PROPANE[70]	Reference correlation of Vogel and Herrmann [71]
PROPYLEN[1]	Reference correlation of Assael et al.[50]
PROPYLENEOXIDE[1]	ECS model[2]
PROPYNE[1]	ECS model[2]
PXYLENE[44]	Reference correlation of Mylona et al.[45]
R11[72]	ECS model[73]
R113[74]	Reference correlation[1]
R114[75]	ECS model[2]
R115[76]	ECS model[1]
R116[5]	Reference correlation[1]
R12[74]	ECS model[73]
R1224YDZ[77]	ECS model[2]
R123[78]	Reference correlation of Laesecke et al.[79]
R1233ZDE[80]	Reference correlation of Perkins and Huber[81]
R1234YF[82]	Reference correlation of Perkins and Huber[83]
R1234ZEE[84]	Reference correlation of Perkins and Huber[83]
R124[85]	ECS model[1]
R125[86]	Reference correlation of Perkins and Huber[87]
R13[88]	ECS model[1]
R1336MZZZ[89]	ECS model[2]
R134A[90]	Reference correlation of Perkins et al.[91]
R14[75]	ECS model[1]
R141B[5]	ECS model[1]
R142B[5]	ECS model[1]

R143A92	ECS model[2]
R150[93]	ECS model[2]
R152A[94]	Reference correlation of Krauss et al.[95]
R161[96]	Reference correlation of Tsolakidou et al.[97]
R21[75]	ECS model[1]
R218[5]	ECS model[2]
R22[98]	ECS model[73]
R227EA[76]	ECS model[1]
R23[99]	Reference correlation of Shan et al.[100]
R236EA[101]	ECS model[2]
R236FA[102]	ECS model[2]
R245CA[103]	ECS model[2]
R245FA[104]	Reference correlation of Perkins et al.[105]
R32[232]	Reference correlation[1]
R365MFC[76]	ECS model[2]
R40[41]	ECS model[2]
R41[5]	ECS model[73]
RC318[75]	ECS model[2]
RE245CB2[1]	ECS model[2]
RE245FA2[1]	ECS model[2]
RE347MCC[1]	ECS model[2]
SF6[107]	Reference correlation of Assael et al.[108]
SO2[109]	ECS model[2]
TOLUENE[5]	Reference correlation of Assael et al.[110]
VINYLCHLORIDE[1]	ECS model[2]
WATER[111]	Reference correlation[112]
XENON[5]	ECS model[2]

[a] For those without a published EoS, REFPROP 10.0[1] is cited.

Table S3 Names, group and constants of pure fluids.

REFPROP fluid name	IUPAC chemical name	Group	M / g·mol^{-1}	T_{crit} / K	p_{crit} / MPa	R_D	γ	φ_0 / nm	Γ	q_D^{-1} / nm	T_{ref} / K
13BUTADIENE	Buta-1,3-diene	2	54.090	425.135	4.305	1.02	1.239	0.207	0.057	0.593	637.7
1BUTENE	1-Butene	5	56.106	419.290	4.005	1.02	1.239	0.211	0.057	0.607	628.94
1BUTYNE	But-1-yne	4	54.090	432.000	4.142	1.02	1.239	0.199	0.054	0.588	648
1PENTENE	Pent-1-ene	4	70.133	465.740	3.598	1.02	1.239	0.223	0.058	0.652	698.61
22DIMETHYLBUTANE	2,2-Dimethylbutane	3	86.175	490.000	3.138	1.02	1.239	0.24	0.059	0.703	735
23DIMETHYLBUTANE	2,3-Dimethylbutane	3	86.175	500.600	3.161	1.02	1.239	0.238	0.058	0.701	750.9
3METHYLPENTANE	3-Methylpentane	5	86.175	506.000	3.185	1.02	1.239	0.237	0.059	0.703	759
ACETONE	Propanone	4	58.079	508.100	4.692	1.02	1.239	0.196	0.052	0.586	762.15
ACETYLENE	Ethyne	3	26.037	308.300	5.988	1.02	1.239	0.166	0.056	0.47	462.45
AMMONIA	Ammonia	7	17.031	405.560	11.363	1.02	1.239	0.14	0.053	0.4	608.34
ARGON	Argon	2	39.948	150.687	4.863	1.01	1.242	0.13	0.055	0.32	301.374
BENZENE	Benzene	4	78.112	562.020	4.907	1.02	1.239	0.216	0.0569	0.62	843
BUTANE	n-Butane	3	58.122	425.125	3.796	1.03	1.239	0.194	0.0496	0.87535	637.68
C11	Undecane	5	156.308	638.800	1.990	1.02	1.239	0.267	0.059	0.866	958.2
C12	Dodecane	5	170.335	658.100	1.817	1.03	1.239	0.194	0.0496	1.52	987.15
C16	Hexadecane	5	226.441	722.100	1.480	1.02	1.239	0.291	0.063	0.998	1083.15
C1CC6	Methylcyclohexane	3	98.186	572.200	3.470	1.01	1.242	0.15	0.052	0.624	858.3
C22	Docosane	6	310.601	792.200	1.174	1.02	1.239	0.31	0.067	1.114	1188.3
C2BUTENE	cis-2-Butene	5	56.106	435.750	4.226	1.02	1.239	0.21	0.058	0.607	653.63
C3CC6	n-Propylcyclohexane	4	126.239	630.800	2.860	1.01	1.242	0.15	0.052	0.624	958.725
C4F10	Decafluorobutane	3	238.027	386.326	2.322	1.02	1.239	0.233	0.061	0.715	579.49
C5F12	Dodecafluoropentane	5	288.034	421.000	2.063	1.02	1.239	0.244	0.062	0.765	630.83
C6F14	Tetradecafluorohexane	5	338.042	448.000	1.742	1.02	1.239	0.254	0.06	0.812	672
CF3I	Trifluoroiodomethane	3	195.910	396.440	3.953	1.02	1.239	0.21	0.057	0.598	594.66
CHLORINE	Chlorine	3	70.906	416.865	7.642	1.02	1.239	0.179	0.056	0.486	625.3
CHLOROBENZENE	Chlorobenzene	4	112.557	632.350	4.521	1.02	1.239	0.16	0.098	0.666	948.53
CO	Carbonmonoxide	2	28.010	132.860	3.494	1.02	1.239	0.164	0.059	0.437	199.29
CO2	Carbondioxide	3	44.010	304.128	7.377	1.02	1.239	0.15	0.052	0.4	456.19
COS	Carbonoxidesulfide	3	60.075	378.770	6.370	1.02	1.239	0.182	0.056	0.5	568.16
CYCLOBUTENE	1-Cyclobutene	3	54.090	448.000	5.150	1.02	1.239	0.2	0.056	0.567	672
CYCLOHEX	Cyclohexane	3	84.160	553.600	4.081	1.02	1.239	0.23	0.058	0.668	830.4
CYCLOPEN	Cyclopentane	3	70.133	511.720	4.583	1.02	1.239	0.216	0.058	0.624	767.58
CYCLOPRO	Cyclopropane	3	42.081	398.300	5.580	1.02	1.239	0.191	0.057	0.534	597.45

REFPROP fluid name	IUPAC chemical name	Group	M / g·mol^{-1}	T_{crit} / K	p_{crit} / MPa	R_D	γ	φ_0 / nm	Γ	q_D^{-1} / nm	T_{ref} / K
D2	Deuterium	1	4.028	38.340	1.680	1.01	1.242	0.15	0.052	0.4	57.51
D2O	Deuteriumoxide	8	20.028	643.847	21.662	0	0	0	0	0	0
D4	Octamethylcyclotetrasiloxane	6	296.616	586.500	1.347	1.02	1.239	0.298	0.064	0.983	879.75
D5	Decamethylcyclopentasiloxane	6	370.770	618.300	1.093	1.02	1.239	0.319	0.064	1.068	927.45
D6	Dodecamethylcyclohexasiloxane	5	444.924	645.780	0.961	1.02	1.239	0.341	0.072	1.17	968.67
DEA	2,2'-Iminodiethanol	7	105.136	736.500	4.951	1.02	1.239	0.185	0.068	0.662	1104.75
DECANE	Decane	5	142.282	617.700	2.103	1.03	1.239	0.194	0.0496	0.70864	926.55
DEE	Diethylether	3	74.122	466.700	3.720	1.02	1.239	0.196	0.066	0.645	700.05
DMC	Dimethylestercarbonicacid	3	90.078	557.000	4.909	1.02	1.239	0.204	0.059	0.62	835.5
DME	Methoxymethane	3	46.068	400.378	5.337	1.02	1.239	0.189	0.057	0.54	600.57
EBENZENE	Phenylethane	4	106.165	617.120	3.622	1.02	1.239	0.235	0.056	0.706	925.7
EGLYCOL	1,2-Ethandiol	7	62.068	719.000	10.509	1.02	1.239	0.166	0.073	0.542	1078.5
ETHANE	Ethane	3	30.069	305.322	4.872	1.01	1.242	0.19	0.0563	0.545	610.66
ETHANOL	Ethylalcohol	7	46.068	514.710	6.268	1.02	1.239	0.1643	0.05885	0.53	772.06
ETHYLENE	Ethene	3	28.054	282.350	5.042	1.02	1.239	0.181	0.058	0.49	423.53
ETHYLENEOXIDE	Ethyleneoxide	3	44.053	468.920	3.705	1.02	1.239	0.176	0.028	0.506	703.38
FLUORINE	Fluorine	2	37.997	144.414	5.172	1.02	1.239	0.145	0.056	0.385	216.621
H2S	Hydrogensulfide	7	34.081	373.100	9.000	1.02	1.239	0.164	0.058	0.447	559.65
HCL	Hydrogenchloride	7	36.461	324.680	8.314	1.02	1.239	0.154	0.054	0.424	487
HELIUM	Helium-4	1	4.003	5.195	0.228	0	0	0	0	0	0
HEPTANE	Heptane	5	100.202	540.200	2.736	1.02	1.239	0.245	0.0586	0.8	810.2
HEXANE	Hexane	5	86.175	507.820	3.044	1.02	1.239	0.2364	0.05803	0.737	761.73
HYDROGEN	Hydrogen(normal)	1	2.016	33.145	1.296	1.01	1.242	0.15	0.052	0.4	49.718
IBUTENE	2-Methyl-1-propene	3	56.106	418.090	4.010	1.02	1.239	0.212	0.058	0.611	627.14
IHEXANE	2-Methylpentane	5	86.175	497.700	3.040	1.02	1.239	0.238	0.059	0.708	746.6
IOCTANE	2,2,4-Trimethylpentane	5	114.229	544.000	2.572	1.02	1.239	0.256	0.059	0.771	816
IPENTANE	2-Methylbutane	3	72.149	460.350	3.378	1.02	1.239	0.227	0.058	0.664	690.53
ISOBUTAN	2-Methylpropane	3	58.122	407.810	3.629	1.03	1.239	0.194	0.0496	0.65766	611.73
KRYPTON	Krypton	2	83.798	209.480	5.525	1.02	1.239	0.168	0.058	0.437	314.22
MD2M	Decamethyltetrasiloxane	6	310.685	599.400	1.144	1.02	1.239	0.311	0.066	1.049	899.1
MD3M	Dodecamethylpentasiloxane	6	384.839	628.960	0.961	1.02	1.239	0.33	0.066	1.127	943.44
MD4M	Tetradecamethylhexasiloxane	6	458.993	653.200	0.840	1.02	1.239	0.347	0.07	1.208	979.8
MDM	Octamethyltrisiloxane	6	236.532	565.361	1.438	1.02	1.239	0.295	0.064	0.956	848.04

REFPROP fluid name	IUPAC chemical name	Group	M / g·mol^{-1}	T_{crit} / K	p_{crit} / MPa	R_D	γ	φ_0 / nm	Γ	q_D^{-1} / nm	T_{ref} / K
MEA	Ethanolamine	7	61.083	671.400	8.125	1.02	1.239	0.173	0.065	0.559	1007.1
METHANE	Methane	2	16.043	190.564	4.599	1.03	1.239	0.194	0.0496	0.4	285.846
METHANOL	Methanol	7	32.042	512.600	8.104	1.03	1.239	0.1487	0.05283	0.7	768.9
MILPRF23699	MIL-PRF-23699	6	557.600	930.000	1.080	1.02	1.239	0.365	0.087	1.32	1395
MLINOLEA	Methyl(Z,Z)-9,12-octadecadienoate	6	294.472	799.000	1.341	1.03	1.239	0.194	0.0496	0.875	1198.5
MLINOLEN	Methyl(Z,Z,Z)-9,12,15-octadecatrienoate	6	292.456	772.000	1.369	1.02	1.239	0.284	0.073	1.056	1158
MM	Hexamethyldisiloxane	6	162.377	518.700	1.931	1.02	1.239	0.268	0.062	0.84	778.05
MOLEATE	Methylcis-9-octadecenoate	6	296.488	782.000	1.246	1.03	1.239	0.194	0.0496	0.875	1173
MPALMITA	Methylhexadecanoate	6	270.451	755.000	1.350	1.03	1.239	0.194	0.0496	0.875	1132.5
MSTEARAT	Methyloctadecanoate	6	298.504	775.000	1.239	1.03	1.239	0.194	0.0496	0.875	1162.5
MXYLENE	1,3-Dimethylbenzene	4	106.165	616.890	3.535	1.02	1.239	0.235	0.057	0.713	925.3
N2O	Dinitrogenmonoxide	2	44.013	309.520	7.245	1.02	1.239	0.159	0.057	0.446	464.28
NEON	Neon	2	20.179	44.400	2.662	1.02	1.239	0.131	0.06	0.331	66.74
NEOPENTN	2,2-Dimethylpropane	3	72.149	433.740	3.196	1.02	1.239	0.23	0.057	0.664	650.61
NF3	Nitrogentrifluoride	3	71.019	234.000	4.461	0	0	0	0	0	0
NITROGEN	Nitrogen	2	28.014	126.192	3.396	1.01	1.242	0.17	0.055	0.4	252.384
NONANE	Nonane	5	128.255	594.550	2.281	1.03	1.239	0.194	0.0496	1.0431	891.825
NOVEC649	1,1,1,2,2,4,5,5,5-Nonafluoro-4-(trifluoromethyl)-3-pentanone	4	316.044	441.810	1.869	1.02	1.239	0.251	0.061	0.334	662.72
OCTANE	Octane	5	114.229	568.740	2.484	1.03	1.239	0.194	0.0496	0.68628	853.98
ORTHOHYD	Orthohydrogen	1	2.016	33.220	1.311	0	0	0	0	0	0
OXYGEN	Oxygen	2	31.999	154.581	5.043	1.01	1.242	0.24	0.055	0.51	309.162
OXYLENE	1,2-Dimethylbenzene	4	106.165	630.259	3.738	1.02	1.239	0.236	0.058	0.711	945.4
PARAHYD	Parahydrogen	1	2.016	32.938	1.286	1.01	1.242	0.15	0.052	0.5	49.407
PENTANE	Pentane	3	72.149	469.700	3.368	1.02	1.239	0.227	0.058	0.668	704.55
POE5	pentaerythritoltetrapentanoate	6	472.612	890.000	1.270	1.02	1.239	0.343	0.082	1.218	1335
POE7	pentaerythritoltetraheptanoate	6	584.835	940.000	1.030	1.02	1.239	0.367	0.09	1.349	1410
POE9	pentaerythritoltetranonanoate	6	697.051	970.000	0.885	1.02	1.239	0.405	0.096	1.476	1455
PROPADIENE	1,2-Propadiene	3	40.064	398.000	5.216	1.02	1.239	0.195	0.055	0.541	597
PROPANE	Propane	3	44.096	369.890	4.251	1.03	1.239	0.194	0.0496	0.71664	554.73
PROPYLEN	Propene	4	42.080	364.211	4.555	1.02	1.239	0.198	0.057	0.43	546.32
PROPYLENEOXIDE	1,2-Epoxypropane	3	58.079	488.110	5.437	1.02	1.239	0.194	0.056	0.567	732.17
PROPYNE	Propyne	3	40.064	402.380	5.626	1.02	1.239	0.186	0.058	0.535	603.57

REFPROP fluid name	IUPAC chemical name	Group	M / g·mol^{-1}	T_{crit} / K	p_{crit} / MPa	R_D	γ	φ_0 / nm	Γ	q_{D}^{-1} / nm	T_{ref} / K
PXYLENE	1,4-Dimethylbenzene	4	106.165	616.168	3.532	1.02	1.239	0.235	0.056	0.71	924.3
R11	Trichlorofluoromethane	3	137.368	471.110	4.408	1.03	1.239	0.194	0.0496	0.52854	706.665
R1123	Trifluoroethylene	3	82.025	331.730	4.543	1.02	1.239	0.153	0.075	0.538	497.6
R113	1,1,2-Trichloro-1,2,2-trifluoroethane	3	187.375	487.210	3.392	1.03	1.239	0.194	0.0496	0.5	730.8
R114		3	170.921	418.830	3.257	1.02	1.239	0.223	0.059	0.656	628.25
R115	Chloropentafluoroethane	3	154.466	353.100	3.129	1.03	1.239	0.194	0.0496	0.37293	529.65
R116	Hexafluoroethane	3	138.012	293.030	3.048	1.03	1.239	0.194	0.0496	0.5	439.545
R12	Dichlorodifluoromethane	3	120.913	385.120	4.136	1.03	1.239	0.194	0.0496	0.52854	577.68
R1216	Hexafluoropropene	3	150.023	358.900	3.150	1.03	1.239	0.194	0.0496	0.5835	538.3
R1224YDZ	(Z)-1-Chloro-2,3,3,3-tetrafluoropropene	3	148.487	428.690	3.337	1.02	1.239	0.214	0.058	0.646	643.04
R123	2,2-Dichloro-1,1,1-trifluoroethane	3	152.931	456.831	3.662	1.02	1.239	0.216	0.058	0.643	685.25
R1233ZDE	trans-1-Chloro-3,3,3-trifluoro-1-propene	3	130.496	439.600	3.624	1.02	1.239	0.213	0.059	0.598	659.4
R1234YF	2,3,3,3-Tetrafluoroprop-1-ene	3	114.042	367.850	3.382	1.03	1.239	0.194	0.0496	0.5835	551.775
R1234ZEE	trans-1,3,3,3-Tetrafluoropropene	3	114.042	382.513	3.635	1.03	1.239	0.194	0.0496	0.5835	573.78
R1234ZEZ	cis-1,3,3,3-Tetrafluoropropene	3	114.042	423.270	3.531	1.02	1.239	0.206	0.055	0.62	634.91
R124	1-Chloro-1,2,2,2-tetrafluoroethane	3	136.476	395.425	3.624	1.03	1.239	0.194	0.0496	0.5	593.138
R1243ZF	3,3,3-Trifluoropropene	3	96.051	376.930	3.518	1.02	1.239	0.205	0.056	0.604	565.4
R125	Pentafluoroethane	3	120.021	339.173	3.618	1.03	1.239	0.194	0.0496	0.58346	508.748
R13	Chlorotrifluoromethane	3	104.459	302.000	3.879	1.03	1.239	0.194	0.0496	0.34964	453
R1336MZZZ	(Z)-1,1,1,4,4,4-Hexafluoro-2-butene	3	164.056	444.500	2.903	1.02	1.239	0.221	0.058	0.681	666.75
R134A	1,1,1,2-Tetrafluoroethane	3	102.032	374.210	4.059	1.03	1.239	0.194	0.0496	0.52854	561.411
R14	Tetrafluoromethane	3	88.005	227.510	3.750	1.03	1.239	0.194	0.0496	0.22657	341.265
R141B	1,1-Dichloro-1-fluoroethane	3	116.950	477.500	4.212	1.03	1.239	0.194	0.0496	0.5	719.94
R142B	1-Chloro-1,1-difluoroethane	3	100.495	410.260	4.055	1.03	1.239	0.194	0.0496	0.61565	615.39
R143A	1,1,1-Trifluoroethane	3	84.041	345.857	3.761	1.02	1.239	0.193	0.055	0.23	518.79
R150	1,2-Dichloroethane	3	98.959	561.600	5.226	1.02	1.239	0.204	0.056	0.603	842.4
R152A	1,1-Difluoroethane	3	66.051	386.411	4.517	1.03	1.239	0.1894	0.0487	0.437	579.617
R161	Fluoroethane	3	48.060	375.250	5.046	1.02	1.239	0.183	0.055	0.3104	562.88
R21	Dichlorofluoromethane	3	102.923	451.480	5.181	1.03	1.239	0.194	0.0496	0.5	677.22
R218	Octafluoropropane	3	188.019	345.020	2.640	1.02	1.239	0.219	0.061	0.659	517.53
R22	Chlorodifluoromethane	3	86.468	369.295	4.990	1.03	1.239	0.194	0.0496	0.52854	553.943
R227EA	1,1,1,2,3,3,3-Heptafluoropropane	3	170.029	374.900	2.925	1.03	1.239	0.194	0.0496	0.5	562.328

REFPROP fluid name	IUPAC chemical name	Group	M / g·mol^{-1}	T_{crit} / K	p_{crit} / MPa	R_D	γ	φ_0 / nm	Γ	q_D^{-1} / nm	T_{ref} / K
R23	Trifluoromethane	3	70.014	299.293	4.832	1.03	1.239	0.194	0.0496	0.52854	618.66
R236EA	1,1,1,2,3,3-Hexafluoropropane	3	152.038	412.440	3.420	1.02	1.239	0.208	0.06	0.636	618.66
R236FA	1,1,1,3,3,3-Hexafluoropropane	3	152.038	398.070	3.200	1.02	1.239	0.209	0.06	0.641	597.105
R245CA	1,1,2,2,3-Pentafluoropropane	3	134.048	447.570	3.941	1.02	1.239	0.205	0.06	0.624	671.36
R245FA	1,1,1,3,3-Pentafluoropropane	3	134.048	427.010	3.651	1.02	1.239	0.204	0.06	0.626	640.52
R32	Difluoromethane	3	52.024	351.255	5.782	1.03	1.239	0.194	0.0496	0.55829	526.883
R365MFC	1,1,1,3,3-Pentafluorobutane	3	148.075	460.000	3.266	1.02	1.239	0.218	0.06	0.669	690
R40	Methylchloride	3	50.488	416.300	6.690	1.02	1.239	0.18	0.056	0.505	624.45
R41	Fluoromethane	3	34.033	317.280	5.897	1.03	1.239	0.194	0.0496	0.5	475.92
RC318	Octafluorocyclobutane	3	200.040	388.380	2.778	1.02	1.239	0.222	0.062	0.677	582.57
RE143A	Methyltrifluoromethylether	3	100.040	377.921	3.635	1.02	1.239	0.198	0.054	0.588	566.88
RE245CB2	Methyl-pentafluoroethyl-ether	3	150.047	406.813	2.886	1.02	1.239	0.217	0.057	0.66	610.2
RE245FA2	2,2,2-Trifluoroethyl-difluoromethyl-ether	3	150.047	444.880	3.433	1.02	1.239	0.212	0.061	0.653	667.32
RE347MCC	1,1,1,2,2,3,3-Heptafluoro-3-methoxypropane	3	200.055	437.700	2.478	1.02	1.239	0.231	0.058	0.5553	656.55
SF6	Sulfurhexafluoride	2	146.055	318.723	3.755	1.01	1.242	0.19	0.052	0.35	478.08
SO2	Sulfurdioxide	2	64.064	430.640	7.887	1.02	1.239	0.167	0.059	0.485	645.96
T2BUTENE	trans-2-Butene	3	56.106	428.610	4.027	1.02	1.239	0.21	0.057	0.609	642.92
TOLUENE	Methylbenzene	4	92.138	591.750	4.126	1.02	1.239	0.22	0.05	0.62	887.625
VINYLCHLORIDE	Chloroethylene	3	62.498	424.964	5.590	1.02	1.239	0.195	0.059	0.551	637.45
WATER	Water	8	18.015	647.096	22.064	1.01	1.239	0.13	0.06	0.4	970.644
XENON	Xenon	2	131.293	289.733	5.842	1.02	1.239	0.182	0.058	0.479	434.6

Table S4 Statistics of the experimental values of pure fluids. [a]

REFPROP fluid name	S_D	N_{tot}	N_{use}	N_{lim}	N_{phase}	N_{dev}	$N_{REFPROP,fail}$	Selected experimental data T/K	p/MPa	<|δRES%|>	<|δREFPROP%|>
13BUTADIENE	9	33	31	2	0	0	0	223.2 - 473.2	0.01 - 4.17	5.9	9.6
1BUTENE	8	35	35	0	0	0	0	173.2 - 673.2	0 - 1.79	2.6	3.6
1PENTENE	1	12	12	0	0	0	0	257.4 - 301.7	0.1 - 0.1	0.3	0.1
22DIMETHYLBUTANE	6	33	33	0	0	0	2	183.2 - 483.1	0 - 2.84	4.1	5.0
23DIMETHYLBUTANE	6	78	55	23	0	0	0	173.2 - 473.1	0 - 311.4	2.1	6.4
3METHYLPENTANE	7	32	32	0	0	0	0	213.2 - 463.1	0 - 1.67	3.8	4.8
ACETONE	15	92	87	1	0	4	0	253.2 - 571.7	0.01 - 0.49	2.5	1.8
ACETYLENE	7	42	31	11	0	0	0	193.2 - 423.1	0.05 - 4.26	4.0	3.6
AMMONIA	7	305	283	8	3	11	0	195.5 - 674.2	0.01 - 39.73	5.1	5.9
ARGON	32	2109	2055	12	14	28	0	90 - 2998.7	0.03 - 98.07	2.8	2.0
BENZENE	37	427	371	49	5	2	0	279.1 - 680.6	0.01 - 151.4	2.3	2.5
BUTANE	3	2891	2886	0	5	0	0	135.8 - 673.2	0 - 70.1	2.7	1.2
C11	7	378	378	0	0	0	7	284.6 - 677.6	0 - 49.03	2.6	2.3
C12	9	91	90	0	0	1	2	294.1 - 645.7	0 - 50	3.0	3.9
C16	7	157	153	4	0	0	0	307.2 - 693.1	0.1 - 49.03	1.3	2.2
C1CC6	5	1358	1332	0	8	18	0	293.1 - 595.7	0.01 - 60.1	1.2	0.8
C22	7	132	120	12	0	0	13	317.4 - 613.1	0 - 49.1	1.4	1.9
C2BUTENE	3	15	15	0	0	0	1	183.2 - 383.1	0 - 1.72	1.4	3.2
C3CC6	1	668	665	0	3	0	0	300.9 - 605.9	0.05 - 60.17	0.8	0.7
C5F12	1	11	9	0	0	2	0	173.2 - 333.1	0 - 0.28	7.1	6.9
C6F14	4	32	29	0	0	3	4	193.2 - 343.1	0 - 0.15	5.9	5.1
CF3I	1	30	30	0	0	0	0	266.6 - 336.7	0.1 - 0.91	3.0	2.6
CHLORINE	8	63	26	4	1	32	0	193.2 - 523.2	0.01 - 5.37	10.2	11.0
CHLOROBENZENE	5	221	178	41	2	0	0	289 - 620.1	0 - 184.8	4.0	5.0
CO	1	19	19	0	0	0	0	87.4 - 376.9	0.1 - 0.1	0.7	0.4
CO2	14	254	252	1	0	1	2	200.9 - 842.2	0.1 - 196	2.1	2.2
CYCLOHEX	14	399	394	5	0	0	0	280.8 - 633.1	0.01 - 145.6	3.2	2.8
CYCLOPEN	6	173	173	0	0	0	0	234.7 - 454.4	0.1 - 149.9	1.8	0.5
CYCLOPRO	6	17	16	1	0	0	0	273.1 - 422.1	0.1 - 3.65	4.4	14.5
D2	24	130	116	5	0	9	0	19.9 - 873.1	0.02 - 60	3.1	3.9

REFPROP fluid name	S_D	N_{tot}	N_{use}	N_{lim}	N_{phase}	N_{dev}	$N_{REFPROP,fail}$	Selected experimental data			
								T/K	p/MPa	<|δRES%|>	<|δREFPROP%|>
D2O	16	2634	2598	0	0	36	0	262.5 - 1042.9	0 - 400	4.8	2.6
D4	3	149	146	3	0	0	0	295.6 - 512.6	0.1 - 10	0.5	0.8
D5	3	150	150	0	0	0	0	294 - 512.8	0.1 - 10	0.9	1.5
DEA	1	6	5	1	0	0	1	325.4 - 442.1	0 - 0	2.1	0.3
DECANE	14	338	337	1	0	0	1	253.1 - 677.6	0 - 49	3.2	5.3
DEE	7	163	127	30	5	1	0	273.1 - 507.2	0.01 - 30.2	1.8	1.9
DMC	1	109	106	3	0	0	0	290.5 - 391	0.25 - 30.12	1.1	1.1
DME	7	420	407	3	5	5	0	234 - 387.7	0 - 30.6	1.2	1.3
EBENZENE	13	537	527	9	0	1	0	258 - 683.3	0 - 118.7	2.1	1.6
EGLYCOL	9	40	39	0	1	0	0	273.1 - 453.1	0.1 - 0.1	1.3	1.7
ETHANE	12	606	603	0	0	3	0	226.9 - 673.2	0.01 - 70.11	2.6	2.6
ETHANOL	26	398	395	0	0	3	1	213.2 - 573.1	0 - 39.9	2.8	2.6
ETHYLENE	7	155	155	0	0	0	0	273.1 - 673.2	0.1 - 16.78	2.1	2.3
ETHYLENEOXIDE	10	61	60	0	1	0	0	173.2 - 673.2	0 - 3.56	4.0	5.0
FLUORINE	7	88	53	35	0	0	0	63.1 - 450	0 - 3.19	3.0	7.5
H2S	7	66	66	0	0	0	0	246.9 - 594	0.1 - 34.81	4.1	4.2
HCL	11	95	85	0	0	10	0	163.2 - 632.1	0.02 - 8.96	6.9	3.1
HELIUM	11	324	322	0	0	2	6	82.8 - 2798.9	0.1 - 98.07	2.1	1.8
HEPTANE	42	909	866	27	6	10	0	183.2 - 677.7	0 - 187.2	3.1	3.0
HEXANE	21	293	288	2	3	0	0	183.2 - 682.9	0 - 50	1.7	2.0
HYDROGEN	34	1428	1422	5	1	0	0	14.6 - 1499.7	0 - 68.69	2.6	1.9
IBUTENE	9	210	199	0	4	7	0	173.2 - 673.2	0 - 50.62	3.8	3.4
IHEXANE	4	14	14	0	0	0	1	213.2 - 463.1	0 - 1.8	1.9	9.8
IOCTANE	7	273	271	2	0	0	0	190 - 620	0 - 100	1.5	2.8
IPENTANE	2	9	6	3	0	0	0	273.1 - 423.1	0.03 - 1.87	7.0	4.9
ISOBUTAN	3	2886	2878	0	4	4	0	115.6 - 673.2	0.01 - 106.5	2.5	1.3
KRYPTON	10	213	82	114	0	17	0	125.5 - 1120	0.1 - 50.74	3.3	3.9
MDM	1	132	132	0	0	0	0	304.7 - 500.5	0.48 - 10	1.6	1.5
MEA	1	10	10	0	0	0	1	297.8 - 447.1	0 - 0.11	2.1	0.5
METHANE	28	2058	1991	3	4	60	0	98.9 - 900.1	0 - 110	3.1	2.5
METHANOL	26	653	633	0	5	15	0	259.2 - 601.1	0.01 - 60	2.8	3.0

REFPROP fluid name	S_D	N_{tot}	N_{use}	N_{lim}	N_{phase}	N_{dev}	$N_{REFPROP,fail}$	Selected experimental data			
								T/K	p/MPa	<|δRES%|>	<|δREFPROP%|>
MLINOLEA	1	350	350	0	0	0	0	301.5 - 506.7	0.09 - 42.75	1.5	0.5
MM	3	146	146	0	0	0	0	292.2 - 507.6	0.01 - 10	0.9	1.0
MOLEATE	2	790	790	0	0	0	0	301.6 - 508.9	0.08 - 41.9	1.5	0.8
MSTEARAT	2	19	19	0	0	0	7	313.1 - 513.1	0 - 0	1.0	7.2
MXYLENE	12	384	330	0	4	50	0	258.2 - 698	0 - 98.07	5.6	5.2
N2O	3	92	92	0	0	0	0	190.3 - 702.5	0.1 - 35.12	2.4	2.3
NEON	10	201	65	135	0	1	0	273.1 - 1000	0 - 0.1	3.9	2.2
NITROGEN	36	1580	1579	0	1	0	0	76.4 - 1135.1	0.03 - 251.59	2.3	2.5
NONANE	7	115	115	0	0	0	1	223.1 - 677.6	0 - 0.1	0.7	2.0
OCTANE	16	134	132	0	0	2	0	223.1 - 677.7	0 - 0.1	2.2	2.5
OXYGEN	9	182	179	1	1	1	0	83.2 - 950.1	0 - 49.03	2.4	2.5
OXYLENE	13	448	428	17	3	0	0	257.8 - 697.9	0 - 120.1	1.9	1.5
PENTANE	14	338	327	0	2	9	1	153.2 - 623.6	0.06 - 550.2	4.0	4.4
PROPANE	18	3079	3050	0	8	21	0	91.2 - 810.1	0 - 81.2	4.4	2.1
PROPYLEN	6	428	423	0	5	0	0	260 - 633.1	0.1 - 50	3.3	4.1
PROPYLENEOXIDE	4	15	14	1	0	0	1	203.2 - 453.1	0 - 3.19	7.5	6.4
PROPYNE	2	8	8	0	0	0	0	273.1 - 373.1	0.26 - 3.34	4.2	8.3
PXYLENE	14	411	376	34	0	1	0	287.3 - 703.3	0 - 151.7	2.1	1.7
R11	16	467	458	0	8	1	0	163.2 - 485.9	0 - 58.94	3.6	4.1
R113	8	177	161	16	0	0	0	243.1 - 449.3	0 - 100.01	3.2	3.7
R114	4	151	72	79	0	0	0	282.4 - 433.1	0.13 - 40	3.7	4.2
R115	10	1083	1027	37	2	17	0	175.3 - 439.9	0.1 - 60	4.6	4.9
R116	1	24	22	0	0	2	0	222.4 - 261.9	0.36 - 1.35	7.3	24.6
R12	26	719	700	1	5	13	7	116.1 - 468.3	0 - 60	3.7	4.0
R1224YDZ	1	123	123	0	0	0	0	306.8 - 453.1	0.19 - 4.07	2.5	4.6
R123	13	378	343	23	9	3	0	198 - 463	0.01 - 75.05	5.7	8.2
R1233ZDE	2	2499	2465	0	34	0	0	203.6 - 474.2	0.1 - 66.62	1.3	1.1
R1234YF	1	801	801	0	0	0	0	241.9 - 344.5	0.07 - 21.73	1.1	0.6
R1234ZEE	5	1395	1333	0	1	61	0	203.2 - 343.5	0.01 - 23.32	1.2	0.7
R124	6	95	93	0	2	0	0	234.2 - 366.2	0.03 - 30.6	1.0	1.1
R125	27	2183	2138	22	18	5	0	172.7 - 513.2	0 - 69.86	2.1	1.4

REFPROP fluid name	S_D	N_{tot}	N_{use}	N_{lim}	N_{phase}	N_{dev}	$N_{REFPROP,fail}$	Selected experimental data			
								T/K	p/MPa	<|δRES%|>	<|δREFPROP%|>
R13	13	826	820	0	2	4	0	92.5 - 451.9	0.1 - 58.94	4.6	5.1
R1336MZZZ	1	166	166	0	0	0	0	313.4 - 496.2	0.11 - 4.11	0.9	1.1
R134A	32	8208	7991	17	111	89	0	169.9 - 533	0 - 70.01	3.3	4.1
R14	5	432	378	48	6	0	0	121.5 - 698.2	0.1 - 58.94	4.6	6.1
R141B	9	223	217	0	6	0	0	193.6 - 394	0.01 - 30.4	3.1	3.1
R142B	11	397	397	0	0	0	0	193.1 - 504.6	0 - 69.58	2.6	2.6
R143A	6	706	705	0	1	0	0	233.2 - 499	0.1 - 50	5.2	3.1
R150	9	40	40	0	0	0	0	253.2 - 687.4	0.01 - 3.1	2.3	2.2
R152A	18	991	939	24	26	2	0	189.6 - 510	0.02 - 60	2.0	4.5
R161	1	370	359	0	11	0	0	234 - 377.7	0.08 - 5.25	1.8	1.8
R21	3	203	189	12	1	1	0	209.6 - 464.5	0.15 - 58.94	2.2	2.5
R218	2	200	181	14	0	5	1	130 - 434.7	0 - 39.32	2.5	8.1
R22	26	1622	1523	51	23	25	2	116.7 - 514.3	0.01 - 60	3.0	3.4
R227EA	3	600	600	0	0	0	0	259.3 - 344.2	0.04 - 2.94	2.3	2.8
R23	9	792	760	26	0	6	5	118.3 - 487.4	0.1 - 58.94	3.9	6.5
R236EA	2	404	404	0	0	0	0	281.2 - 333.8	0.05 - 0.52	2.1	1.4
R236FA	2	271	271	0	0	0	0	252.6 - 374.6	0.03 - 30	1.0	1.2
R245CA	2	164	164	0	0	0	0	281.6 - 393.1	0.02 - 0.27	1.7	1.8
R245FA	6	454	454	0	0	0	0	244.4 - 436.7	0.01 - 14.7	4.2	4.2
R32	14	1035	999	13	17	6	0	205.4 - 465.6	0.01 - 50	2.3	3.2
R365MFC	2	104	104	0	0	0	0	263.1 - 377.4	0.01 - 0.44	1.7	0.5
R40	2	11	11	0	0	0	0	273.1 - 673.2	0.1 - 0.1	1.4	1.3
R41	14	81	39	6	0	36	0	183.2 - 622.1	0.03 - 4.3	10.2	22.7
RC318	5	445	435	8	0	2	0	234.6 - 433.7	0.06 - 60	2.2	3.7
RE245CB2	3	49	39	0	10	0	0	275.2 - 323.1	0.05 - 10.06	0.9	0.9
RE245FA2	7	438	438	0	0	0	0	282.6 - 323.1	0.03 - 0.21	1.9	1.7
RE347MCC	3	4	4	0	0	0	0	296.1 - 323.1	0.07 - 0.17	5.1	11.7
SF6	10	681	650	15	0	16	1	227 - 929.9	0.1 - 112.8	5.6	5.3
SO2	19	144	121	22	1	0	0	203.2 - 783	0 - 4.25	3.9	6.1
TOLUENE	62	1526	1405	89	29	3	3	183.1 - 702	0 - 369.3	1.9	1.8
VINYLCHLORIDE	1	8	8	0	0	0	0	273.1 - 673.2	0.1 - 0.1	1.2	0.5

REFPROP fluid name	S_D	N_{tot}	N_{use}	N_{lim}	N_{phase}	N_{dev}	$N_{REFPROP,fail}$	Selected experimental data			
								T/K	p/MPa	<|δRES%|>	<|δREFPROP%|>
WATER	62	2830	2673	54	24	79	0	275 - 997.7	0 - 250	5.6	3.0
XENON	55	2650	2243	359	17	31	1	154.8 - 1120	0.01 - 400	2.0	3.0
Total		71554	68765	1544	468	777	72				
N / N_{tot}			96.10%	2.16%	0.65%	1.09%	0.10%				

[a] S_D: number of data sources; N_{tot}: total number of experimental data; N_{use}: number of adopted data; N_{lim}: number of data exceeding limits of the reference EoS in REFPROP 10.0; N_{phase}: number of data reported in conflicting phases; N_{dev}: number of data deviating from the correlation equation by more than 30%. $N_{REFPROP,fail}$: number of data that cannot be calculated with the recommended model in REFPROP 10.0 using the given temperature and pressure. Relative deviations $\delta_{RES} = (\lambda_{exp} - \lambda_{RES})/\ \lambda_{RES}$ and $\delta_{REFPROP} = (\lambda_{exp} - \lambda_{REFPROP})/\ \lambda_{REFPROP}$, where λ_{exp}, λ_{RES} and $\lambda_{REFPROP}$ are experimental data, data calculated with the RES model and the REFPROP models, respectively. The symbol <| δ |> denotes the average of the absolute value of δ.

Table S5 Statistical experimental data summary of fluid mixtures. [a]

Mixtures	S_D	Groups	N_{tot}	N_{use}	N_{lim}	N_{phase}	N_{dev}	$N_{REFPROP,fail}$	Selected experimental data				
									x_1	T/K	p/MPa	<|δRES%|>	<|δREFPROP%|>
ACETONE+ETHANOL	3	4+7	36	36	0	0	0	24	0 - 1	253.2 - 323.1	0 - 0.1	2.9	2.3
ACETONE+HEPTANE	1	4+5	21	21	0	0	0	0	0 - 1	253.2 - 293.1	0 - 0	2.1	1.5
ACETONE+METHANOL	2	4+7	10	10	0	0	0	6	0 - 1	273.1 - 323.1	0.1 - 0.1	2.4	4.4
ACETONE+R150	1	4+3	11	11	0	0	0	0	0 - 1	273.1 - 273.1	0.1 - 0.1	1.9	2.2
ACETONE+TOLUENE	1	4+4	5	5	0	0	0	0	0 - 1	273.1 - 273.1	0.1 - 0.1	1.7	2.6
ACETONE+WATER	3	4+8	79	72	4	2	1	61	0 - 1	233.2 - 333.1	0.1 - 0.1	12.3	2.1
AMMONIA+HYDROGEN	2	7+1	35	0	35	0	0	0	0	/	/	/	/
AMMONIA+NITROGEN	2	7+2	31	31	0	0	0	0	0.092 - 0.902	298.1 - 422.4	0.1 - 0.1	4.3	4.2
AMMONIA+WATER	1	7+8	5	5	0	0	0	5	0.04 - 0.261	298.1 - 323.1	0.1 - 0.1	9.7	/
ARGON+CO2	1	2+3	1	1	0	0	0	0	0.493 - 0.493	593.2 - 593.2	0.1 - 0.1	89.9	89.9
ARGON+DEE	1	2+3	22	22	0	0	0	0	0 - 1	323.1 - 373.1	0.1 - 0.1	1.6	1.3
ARGON+DME	2	2+3	23	23	0	0	0	0	0.317 - 0.99	330 - 370	0.1 - 0.1	2.2	2.2
ARGON+HELIUM	3	2+1	72	72	0	0	0	0	0 - 1	305.1 - 768.9	0.1 - 0.1	2.2	2.2
ARGON+HEPTANE	1	2+5	15	15	0	0	0	0	0.95 - 0.99	330 - 370	0.1 - 0.1	1.3	1.2
ARGON+HEXANE	2	2+5	37	36	0	1	0	0	0 - 1	323.1 - 373.1	0.1 - 0.1	2.4	1.5
ARGON+HYDROGEN	1	2+1	7	7	0	0	0	0	0 - 1	298.1 - 298.1	0.1 - 0.1	8.5	8.5
ARGON+KRYPTON	1	2+2	52	52	0	0	0	0	0.25 - 0.985	103.7 - 273.8	0.1 - 0.1	5.7	6.4
ARGON+METHANE	1	2+2	3	3	0	0	0	0	0.856 - 0.95	273.1 - 273.1	0.1 - 0.1	10.4	10.7
ARGON+NEON	1	2+2	23	23	0	0	0	0	0 - 0.8	373 - 780.9	0.1 - 0.1	1.3	1.3
ARGON+NITROGEN	2	2+2	25	25	0	0	0	0	0 - 1	323.7 - 593.2	0.1 - 9.78	1.9	1.8
ARGON+PENTANE	1	2+3	22	22	0	0	0	0	0 - 1	323.1 - 373.1	0.1 - 0.1	2.7	0.8
ARGON+PROPANE	1	2+3	1	1	0	0	0	0	0.471 - 0.471	591.2 - 591.2	0.1 - 0.1	1.4	1.5
ARGON+R14	1	2+3	91	91	0	0	0	0	0 - 0.845	301 - 301	0.79 - 15.24	3	2.7
ARGON+XENON	1	2+2	6	6	0	0	0	0	0.242 - 0.759	313.1 - 366.1	0.1 - 0.1	2.4	2.5
BENZENE+C16	1	4+5	7	7	0	0	0	0	0 - 1	294.8 - 294.8	0.1 - 0.1	8.7	8.9
BENZENE+CHLOROBENZENE	1	4+4	151	151	0	0	0	0	0.324 - 0.812	304.3 - 580.9	0.1 - 30	8.3	6.9
BENZENE+CYCLOHEX	1	4+3	6	6	0	0	0	0	0 - 1	294.7 - 294.7	0.1 - 0.1	1.6	1.3
BENZENE+DECANE	1	4+5	5	5	0	0	0	0	0 - 1	294.7 - 294.7	0.1 - 0.1	2.4	3.1
BENZENE+DEE	1	4+3	5	5	0	0	0	0	0 - 1	298.1 - 298.1	0.1 - 0.1	1.6	3.1
BENZENE+ETHANOL	2	4+7	46	41	5	0	0	34	0 - 1	279.1 - 348.1	0.1 - 0.1	1.7	2
BENZENE+HEPTANE	1	4+5	12	12	0	0	0	0	0 - 1	298.2 - 318.2	0.1 - 0.1	5.1	4.8
BENZENE+HEXANE	3	4+5	245	242	2	1	0	0	0 - 1	294.7 - 483.1	0.1 - 50	6.7	5
BENZENE+IOCTANE	2	4+5	179	109	70	0	0	0	0 - 1	313.1 - 344.1	0.1 - 250.7	1.9	1.8

Mixtures	S_D	Groups	N_{tot}	N_{use}	N_{lim}	N_{phase}	N_{dev}	$N_{REFPROP,fail}$	Selected experimental data x_1	T/K	p/MPa	<|δRES%|>	<|δREFPROP%|>
BENZENE+METHANOL	3	4+7	26	26	0	0	0	21	0 - 1	273.1 - 298.1	0.1 - 0.1	2	1.7
BENZENE+PXYLENE	1	4+4	12	12	0	0	0	0	0 - 1	298.2 - 318.2	0.1 - 0.1	1.8	2.2
BENZENE+TOLUENE	5	4+4	201	201	0	0	0	0	0 - 1	273.1 - 578.2	0 - 30	1.5	1.4
BUTANE+METHANE	1	3+2	53	53	0	0	0	0	0.606 - 0.606	277.6 - 444.2	0.1 - 34.47	4.4	6.3
C11+C16	1	5+5	30	30	0	0	0	0	0 - 1	292.4 - 363.8	0.1 - 0.1	1.4	0.9
C11+HEPTANE	1	5+5	26	26	0	0	0	0	0.176 - 1	287.9 - 363.8	0.1 - 0.1	1.5	0.8
C12+HEXANE	1	5+5	6	6	0	0	0	0	0 - 1	333.1 - 333.1	0 - 0	5.3	1.1
C16+CYCLOHEX	1	5+3	6	6	0	0	0	0	0 - 1	295.9 - 295.9	0.1 - 0.1	5.4	7.8
C16+HEPTANE	1	5+5	22	22	0	0	0	0	0.129 - 1	289.5 - 362.2	0.1 - 0.1	8.4	2.5
C16+PENTANE	1	5+3	5	5	0	0	0	0	0 - 1	298.8 - 298.8	0.1 - 0.1	7.4	7
CO+HYDROGEN	1	2+1	7	7	0	0	0	0	0 - 1	298.1 - 298.1	0.1 - 0.1	9.2	9.1
CO+NITROGEN	1	2+2	15	15	0	0	0	0	0.245 - 0.755	373.1 - 773.1	0.1 - 0.1	3.3	3.2
CO2+ETHYLENE	2	3+3	43	43	0	0	0	0	0.202 - 0.512	315.2 - 591.2	0.1 - 20.25	2.8	4.5
CO2+HELIUM	2	3+1	4	4	0	0	0	0	0.26 - 0.75	273.1 - 590.2	0.1 - 0.1	6.7	6.7
CO2+HYDROGEN	1	3+1	11	11	0	0	0	0	0 - 1	298.1 - 298.1	0.1 - 0.1	9.6	9.5
CO2+METHANE	3	3+2	425	425	0	0	0	0	0 - 1	212.1 - 435.5	0.22 - 70.64	3.4	4
CO2+N2O	1	3+2	27	27	0	0	0	0	0.219 - 0.751	323.1 - 723.1	0.1 - 0.1	5.2	5.1
CO2+NEON	1	3+2	4	4	0	0	0	0	0.31 - 0.74	273.1 - 273.1	0.1 - 0.1	7.4	7.5
CO2+NITROGEN	5	3+2	233	214	3	0	16	0	0 - 1	273.1 - 623.2	0.1 - 303.97	4.9	5.2
CO2+OXYGEN	1	3+2	4	4	0	0	0	0	0.222 - 0.73	369.1 - 370.1	0.1 - 0.1	0.5	0.5
CO2+PROPANE	1	3+3	3	3	0	0	0	0	0.291 - 0.635	368.1 - 369.1	0.1 - 0.1	0.8	0.2
CYCLOHEX+DECANE	2	3+5	459	381	0	69	9	0	0 - 1	293.1 - 523.1	0.01 - 5.07	14.9	12.1
CYCLOHEX+ETHANOL	2	3+7	21	21	0	0	0	15	0 - 1	298.1 - 323.1	0.1 - 0.1	10.1	2.1
CYCLOHEX+HEXANE	1	3+5	5	5	0	0	0	0	0 - 1	294.8 - 294.8	0.1 - 0.1	2	2.4
CYCLOHEX+NITROGEN	1	3+2	348	335	13	0	0	0	0.245 - 0.741	315.1 - 395.9	0.1 - 0.51	5.3	3.9
CYCLOHEX+TOLUENE	1	3+4	5	5	0	0	0	0	0 - 1	294.5 - 294.5	0.1 - 0.1	3.3	2.8
CYCLOPEN+PENTANE	1	3+3	59	59	0	0	0	0	0.75 - 0.75	301.5 - 344	0.01 - 0.12	17.6	17.8
CYCLOPEN+R365MFC	1	3+3	235	235	0	0	0	0	0.556 - 0.556	354.8 - 415.7	0.1 - 0.61	9.4	7.5
CYCLOPEN+TOLUENE	1	3+4	37	37	0	0	0	0	0.467 - 0.467	234.8 - 309.6	0.1 - 15.22	1.3	0.5
D2+HELIUM	1	1+1	20	19	1	0	0	0	0 - 1	303.1 - 363.1	0.1 - 0.1	4	4
D2+HYDROGEN	3	1+1	50	50	0	0	0	0	0 - 1	273.1 - 448.1	0.09 - 0.1	8.1	8
D2+NEON	1	1+2	20	20	0	0	0	0	0 - 0.905	303.1 - 363.1	0.1 - 0.1	7.3	7.2
D2+NITROGEN	2	1+2	12	12	0	0	0	0	0.399 - 0.778	313.1 - 448.1	0.05 - 0.1	5.1	5
D2+WATER	1	1+8	2	1	1	0	0	2	0.5 - 0.5	525.6 - 525.6	0.1 - 0.1	22.6	/

									Selected experimental data				
Mixtures	S_D	Groups	N_{tot}	N_{use}	N_{lim}	N_{phase}	N_{dev}	$N_{REFPROP,fail}$	x_1	T/K	p/MPa	<|δRES%|>	<|δREFPROP%|>
D2+XENON	1	1+2	9	9	0	0	0	0	0.241 - 0.745	313.1 - 366.1	0.1 - 0.1	1.7	1.7
DEA+MEA	1	7+7	35	34	1	0	0	0	0 - 1	298 - 363	0.1 - 0.1	2	1
DECANE+HEPTANE	1	5+5	10	10	0	0	0	0	0 - 1	298.6 - 323.5	0.1 - 0.1	5.7	6.8
DEE+H2S	1	3+7	28	28	0	0	0	0	0 - 1	353.1 - 473.2	0.1 - 0.1	2.4	2.3
DEE+HELIUM	1	3+1	22	4	18	0	0	0	0 - 1	323.1 - 373.1	0.1 - 0.1	3.2	2.8
DEE+HEXANE	1	3+5	14	11	0	3	0	0	0 - 1	323.1 - 373.1	0.1 - 0.1	8.2	7.6
DEE+METHANOL	1	3+7	9	9	0	0	0	7	0 - 1	273.1 - 273.1	0.1 - 0.1	4	4.4
DEE+NEON	1	3+2	20	4	16	0	0	0	0 - 1	323.1 - 373.1	0.1 - 0.1	1.5	1
DEE+NITROGEN	1	3+2	22	4	18	0	0	0	0 - 1	323.1 - 373.1	0.1 - 0.1	1.7	1.2
DEE+R150	1	3+3	5	5	0	0	0	0	0 - 1	273.1 - 273.1	0.1 - 0.1	3.3	2.6
DEE+TOLUENE	1	3+4	5	5	0	0	0	0	0 - 1	273.1 - 273.1	0.1 - 0.1	4	4.5
DME+PROPANE	2	3+3	6	6	0	0	0	0	0.315 - 0.503	368.1 - 590.2	0.1 - 0.1	8.3	7.8
EGLYCOL+WATER	12	7+8	524	496	24	4	0	440	0 - 1	253.2 - 454.4	0.1 - 100	4.4	2.3
ETHANE+METHANE	1	3+2	24	24	0	0	0	0	0.3 - 0.65	194.2 - 330.3	0.1 - 0.1	1.6	1.5
ETHANE+NITROGEN	1	3+2	62	62	0	0	0	0	0 - 1	348.1 - 348.1	0.1 - 303.97	3.1	2.4
ETHANOL+HEPTANE	1	7+5	21	19	2	0	0	15	0 - 1	253.2 - 293.1	0 - 0	3.7	0.5
ETHANOL+HEXANE	1	7+5	29	29	0	0	0	29	0.384 - 0.849	297.9 - 329.2	0.1 - 0.1	5.7	/
ETHANOL+METHANOL	2	7+7	13	13	0	0	0	9	0 - 1	291.1 - 323.1	0.1 - 0.1	3.7	2
ETHANOL+R150	1	7+3	7	2	5	0	0	0	0 - 1	303.1 - 303.1	0.1 - 0.1	1.7	0.2
ETHANOL+TOLUENE	1	7+4	12	12	0	0	0	8	0 - 1	298.1 - 313.1	0.1 - 0.1	1.5	2.4
ETHANOL+WATER	15	7+8	1102	1038	54	6	4	960	0 - 1	213.2 - 574.4	0 - 75.2	5.4	4.4
ETHYLENE+METHANE	2	3+2	71	71	0	0	0	0	0 - 1	303.7 - 590.2	0.1 - 7.84	3.2	3.3
ETHYLENE+NITROGEN	2	3+2	64	64	0	0	0	0	0.2 - 0.792	315.2 - 591.2	0.1 - 20.92	1.9	1.5
HCL+WATER	1	7+8	5	0	4	1	0	0	/	/	/	/	/
HELIUM+HEXANE	1	1+5	22	18	3	1	0	0	0 - 1	323.1 - 373.1	0.1 - 0.1	4.3	3.2
HELIUM+METHANE	2	1+2	6	6	0	0	0	0	0.299 - 0.94	273.1 - 590.2	0.1 - 0.1	19.5	19.5
HELIUM+NEON	2	1+2	21	21	0	0	0	0	0.2 - 0.8	273.1 - 735.9	0.1 - 0.1	9.3	9.3
HELIUM+NITROGEN	2	1+2	9	9	0	0	0	0	0.219 - 0.837	273.1 - 591.2	0.1 - 0.1	25	25
HELIUM+PENTANE	1	1+3	22	21	1	0	0	0	0 - 1	323.1 - 373.1	0.1 - 0.1	2.2	1.4
HELIUM+R14	1	1+3	98	43	55	0	0	0	0 - 0	301 - 301	0.91 - 15.24	3.6	4.4
HEPTANE+HEXANE	1	5+5	60	60	0	0	0	0	0 - 1	423.1 - 623.1	0.1 - 2	3.5	3.3
HEPTANE+IOCTANE	5	5+5	694	562	132	0	0	0	0.251 - 0.774	280 - 540	0.1 - 266.75	1.8	1.6
HEPTANE+NITROGEN	1	5+2	2	2	0	0	0	0	0.146 - 0.5	310.9 - 344.2	0.01 - 0.02	12.9	12.7
HEPTANE+OCTANE	1	5+5	291	291	0	0	0	0	0.275 - 0.774	280 - 540	0.1 - 50	2.6	1.8

Mixtures	S_D	Groups	N_{tot}	N_{use}	N_{lim}	N_{phase}	N_{dev}	$N_{REFPROP,fail}$	Selected experimental data				
									x_1	T/K	p/MPa	<|δRES%|>	<|δREFPROP%|>
HEPTANE+TOLUENE	2	5+4	22	22	0	0	0	0	0 - 1	253.2 - 313.1	0 - 0.1	0.8	0.8
HEXANE+METHANOL	1	5+7	9	9	0	0	0	9	0.11 - 0.11	299.3 - 324.2	0.1 - 0.1	7.3	/
HEXANE+NEON	1	5+2	22	21	0	1	0	0	0 - 1	323.1 - 373.1	0.1 - 0.1	4	2.5
HEXANE+NITROGEN	1	5+2	22	21	0	1	0	0	0 - 1	323.1 - 373.1	0.1 - 0.1	3.1	1.6
HEXANE+OCTANE	2	5+5	30	30	0	0	0	0	0 - 1	293.6 - 333.1	0.1 - 0.1	5.9	6.3
HEXANE+OXYLENE	1	5+4	207	207	0	0	0	0	0.291 - 0.787	273.1 - 473.1	0.1 - 50	1.5	0.8
HEXANE+PENTANE	2	5+3	88	84	0	4	0	0	0 - 1	323.1 - 623.1	0.1 - 2	5.6	3.2
HEXANE+PXYLENE	1	5+4	234	233	1	0	0	0	0 - 0.787	313.1 - 493.3	0.1 - 50	2	1.7
HEXANE+TOLUENE	1	5+4	1	1	0	0	0	0	0.516 - 0.516	313.1 - 313.1	0.1 - 0.1	0.8	1.7
HYDROGEN+KRYPTON	1	1+2	9	9	0	0	0	0	0.347 - 0.747	313.1 - 366.1	0.1 - 0.1	11.3	11.3
HYDROGEN+N2O	1	1+2	7	7	0	0	0	0	0 - 1	298.1 - 298.1	0.1 - 0.1	12.4	12.3
HYDROGEN+NEON	1	1+2	15	15	0	0	0	0	0.335 - 0.85	313.1 - 448.1	0.1 - 0.1	11.1	11.1
HYDROGEN+NITROGEN	5	1+2	61	61	0	0	0	0	0 - 1	298.1 - 448.1	0.05 - 0.1	4	4
HYDROGEN+OXYGEN	1	1+2	9	9	0	0	0	0	0.204 - 0.791	313.1 - 366.1	0.05 - 0.05	9.4	9.3
HYDROGEN+XENON	1	1+2	9	9	0	0	0	0	0.392 - 0.84	313.1 - 366.1	0.1 - 0.1	6.1	6.1
IOCTANE+OCTANE	2	5+5	13	13	0	0	0	0	0 - 1	295.2 - 313.1	0.1 - 0.1	1.2	1.7
METHANE+NITROGEN	3	2+2	185	185	0	0	0	0	0.07 - 0.749	221.3 - 425	0.1 - 15.45	2.5	1.1
METHANE+PROPANE	2	2+3	31	31	0	0	0	0	0 - 1	323.1 - 423.1	0.1 - 0.1	2.1	2
METHANOL+R150	1	7+3	7	2	5	0	0	0	0 - 1	303.1 - 303.1	0.1 - 0.1	1.9	2.1
METHANOL+TOLUENE	3	7+4	22	7	15	0	0	16	0 - 1	273.1 - 323.1	0.1 - 0.1	2.1	2.1
METHANOL+WATER	7	7+8	356	246	109	0	1	315	0 - 1	233.2 - 343.1	0.1 - 0.1	5.6	1.5
NEON+NITROGEN	1	2+2	15	15	0	0	0	0	0.195 - 0.797	313.1 - 448.1	0.1 - 0.1	14.4	14.5
NEON+OXYGEN	1	2+2	18	18	0	0	0	0	0.256 - 0.771	313.1 - 448.1	0.1 - 0.1	7.3	7.3
NEON+PENTANE	1	2+3	20	20	0	0	0	0	0 - 0.914	323.1 - 373.1	0.1 - 0.1	3.6	3
NITROGEN+OXYGEN	3	2+2	19	19	0	0	0	0	0.218 - 0.773	313.1 - 592.2	0.05 - 0.1	1.7	1.8
NITROGEN+PENTANE	1	2+3	22	22	0	0	0	0	0 - 1	323.1 - 373.1	0.1 - 0.1	2.4	1.2
NITROGEN+PROPANE	1	2+3	1	1	0	0	0	0	0.525 - 0.525	591.2 - 591.2	0.1 - 0.1	0.4	0.5
NITROGEN+R11	1	2+3	5	4	0	1	0	0	0.25 - 1	292 - 292	0.1 - 0.1	3	2.8
NITROGEN+R12	1	2+3	5	5	0	0	0	0	0 - 1	292 - 292	0.1 - 0.1	1.2	0.6
R12+R134A	1	3+3	24	0	0	21	3	0	/	/	/	/	/
R125+R134A	3	3+3	1161	1160	1	0	0	0	0.191 - 0.785	232.8 - 386.6	0.07 - 20	2.2	2.6
R125+R143A	1	3+3	34	34	0	0	0	0	0.412 - 0.412	254.7 - 372.2	0.1 - 2.65	8.4	8
R125+R32	9	3+3	523	522	0	0	1	0	0 - 1	213 - 409.8	0 - 30	3.6	3.2
R134A+R32	3	3+3	324	324	0	0	0	0	0 - 1	193.2 - 323.1	2 - 30	2.1	2.2

Mixtures	S_D	Groups	N_{tot}	N_{use}	N_{lim}	N_{phase}	N_{dev}	$N_{REFPROP,fail}$	Selected experimental data x_1	T/K	p/MPa	<|δRES%|>	<|δREFPROP%|>
R32+R134A	3	3+3	1340	1340	0	0	0	0	0.299 - 0.7	253.4 - 385.1	0.08 - 11.69	1.4	1.4
R134A+R365MFC	1	3+3	363	363	0	0	0	0	0.098 - 0.797	311.2 - 388.6	0.1 - 0.67	9.4	10.8
R14+R22	1	3+3	204	188	0	0	16	0	0.17 - 0.785	232 - 394.1	0.1 - 19.26	5.9	6.2
R142B+R22	2	3+3	91	91	0	0	0	0	0.209 - 0.72	164.8 - 323.1	2.1 - 20.1	1.4	0.8
R152A+R22	2	3+3	88	88	0	0	0	0	0.249 - 0.781	176.6 - 323.1	2.1 - 20.1	2.1	1.8
R40+SO2	1	3+2	41	41	0	0	0	0	0 - 1	273.1 - 473.1	0.1 - 0.1	4.2	4.5
CO2+ETHANE	1	3+3	221	117	83	0	21	0	0.25 - 0.74	291.1 - 318.3	0.99 - 10.69	5.8	7.1
PENTANE+IPENTANE	1	3+3	36	31	0	5	0	0	0.377 - 0.831	345.3 - 395.5	0.1 - 0.5	5.3	0.8
ETHANE+R116	1	3+3	6	6	0	0	0	0	0.078 - 0.857	303 - 303	0.1 - 0.1	2	2.3
PROPANE+R116	1	3+3	6	6	0	0	0	0	0.159 - 0.846	303 - 303	0.1 - 0.1	1	0.4
BUTANE+R116	1	3+3	6	6	0	0	0	0	0.142 - 0.834	303 - 303	0.1 - 0.1	1.3	0.6
PROPANE+R218	1	3+3	5	5	0	0	0	0	0.139 - 0.87	303 - 303	0.1 - 0.1	2.6	3.2
BUTANE+R218	1	3+3	6	6	0	0	0	0	0.14 - 0.889	303 - 303	0.1 - 0.1	2.1	2.5
R125+R152A	1	3+3	12	12	0	0	0	0	0.5 - 0.5	264.9 - 393.9	1 - 3.11	3.8	4.3
R125+R1234ZEE	1	3+3	12	12	0	0	0	0	0.5 - 0.5	262.7 - 395.4	1 - 3.08	2.4	2.2
R143A+R1234ZEE	1	3+3	12	12	0	0	0	0	0.5 - 0.5	264.9 - 404.1	0.91 - 3.04	2.7	3.3
R143A+R1234YF	1	3+3	12	12	0	0	0	0	0.5 - 0.5	265.4 - 393.8	1.06 - 3.09	1.7	1.9
R1234YF+R1234ZEE	1	3+3	12	12	0	0	0	0	0.5 - 0.5	274.8 - 414.2	0.94 - 2.96	3.4	2.1
R125+R1234YF	1	3+3	12	12	0	0	0	0	0.5 - 0.5	259.8 - 394.2	1.11 - 3.1	2.9	2.5
R134A+R1234ZEE	1	3+3	12	12	0	0	0	0	0.5 - 0.5	274.7 - 403.9	0.87 - 3.14	5.7	5.5
PROPANE+R32	1	3+3	1259	1230	0	9	20	0	0.3 - 0.7	227.9 - 346.9	0.02 - 10.76	5	3.4
PROPANE+R134A	1	3+3	981	967	1	4	9	0	0.299 - 0.7	242.6 - 348.5	0.08 - 19.69	2.9	1.9
R134A+R1234YF	1	3+3	12	12	0	0	0	0	0.504 - 0.504	254.9 - 385.1	0.96 - 3.04	4.5	4
R152A+R218	1	3+3	126	114	3	9	0	0	0.376 - 0.376	291.2 - 404.5	0.29 - 10	10.2	10.4
R152A+R134A	1	3+3	130	130	0	0	0	0	0.204 - 0.204	293 - 401	0 - 10	6.6	5.1
R32+R1234YF	1	3+3	36	36	0	0	0	0	0.252 - 0.75	264.2 - 394.8	0.88 - 4	3.4	6.1
R32+R125+R134A	1	3+3+3	1038	1023	0	13	2	0	0.3 - 0.333	248.6 - 347	0.08 - 12.26	2.5	2.4
R32+R1234YF+CO2	1	3+3+3	12	12	0	0	0	0	0.434 - 0.434	254.6 - 373.9	1.05 - 4.53	3.8	4.3
R32+R125+R134A+R1234YF+CO2	1	3+3+3+3+3	12	12	0	0	0	0	0.2 - 0.2	253.9 - 375	1.04 - 5.03	1.8	3.5
Total			16702	15758	685	156	103	1976					
N / N_{tot}				94.35%	4.10%	0.93%	0.62%	11.83%					

[a] S_D: number of data sources; N_{tot}: total number of experimental data; N_{use}: number of adopted data; N_{lim}: number of data exceeding limits of the reference EoSs and mixture models in REFPROP 10.0; N_{phase}: number of data reported in conflicting phases; N_{dev}: number of data deviating from the correlation equation by more than 30%. $N_{REFPROP,fail}$: number of data that cannot be calculated with the default models in REFPROP 10.0 using the given temperature and pressure. x_1 and x_2 are mole fractions of the first and second components, respectively,

in the ‘Mixture’ column. Relative deviations $\delta_{\mathrm{RES}} = (\lambda_{\mathrm{exp}} - \lambda_{\mathrm{RES}})/ \lambda_{\mathrm{RES}}$ and $\delta_{\mathrm{REFPROP}} = (\lambda_{\mathrm{exp}} - \lambda_{\mathrm{REFPROP}})/ \lambda_{\mathrm{REFPROP}}$, where λ_{exp}, λ_{RES} and $\lambda_{\mathrm{REFPROP}}$ are experimental data, data calculated with the RES model and the REFPROP models, respectively. The symbol $<|\delta|>$ denotes the average of the absolute of the relative deviations.

Table S6 Sample thermal conductivity calculations of pure substances with the REFPROP 10.0 and the RES model. [a]

REFPROP fluid name	T/ K	p/ kPa	ρ_{EoS}/ kg·m^{-3}	s_{res}/ J·mol^{-1}·K^{-1}	λ_{exp}/ W·m^{-1}·K^{-1}	λ_{RES}/ W·m^{-1}·K^{-1}	$\lambda_{REFPROP}$/ W·m^{-1}·K^{-1}
13BUTADIENE	297.15	101.325	2.276	-0.192	0.0157	0.01566	0.01559
1BUTENE	423.15	101.1	1.626	-0.088	0.03094	0.03096	0.031
1PENTENE	276.474	101.325	657.65	-43.795	0.12098	0.12091	0.12088
22DIMETHYLBUTANE	339.124	101.325	3.21	-0.255	0.01791	0.01795	0.01786
23DIMETHYLBUTANE	361.15	204100	741.732	-55.526	0.1553	0.15523	0.17001
3METHYLPENTANE	213.15	0.1244	735.52	-66.905	0.1412	0.142	0.13285
ACETONE	292.738	101.325	790.727	-47.845	0.15899	0.15903	0.15571
ACETYLENE	323.15	101.1	0.984	-0.048	0.0246	0.02457	0.02465
AMMONIA	206.195	23050	730.047	-50.382	0.6495	0.64951	0.64017
ARGON	322.2	693	10.363	-0.094	0.01921	0.01921	0.01911
BENZENE	535.511	3512.577	524.149	-18.139	0.0749	0.0749	0.07109
BUTANE	248.776	50736	665.462	-47.578	0.15103	0.15103	0.15104
C11	616.587	101.325	3.162	-0.318	0.03938	0.03938	0.03927
C12	323	30000	749.36	-93.732	0.137	0.13703	0.14006
C16	629.885	101.325	4.597	-0.794	0.03335	0.03336	0.0328
C1CC6	595.417	932	20.172	-1.08	0.05114	0.05114	0.04913
C22	393.15	39200	759.979	-136.056	0.1505	0.1505	0.15089
C2BUTENE	263.15	58.1427	655.329	-41.161	0.1224	0.12227	0.12901
C3CC6	511.188	312	10.17	-1.196	0.03313	0.03313	0.03312
C5F12	213.15	0.643	1858.75	-71.89	0.0747	0.07536	0.06738
C6F14	328.15	94.2457	1585.191	-48.034	0.0534	0.05364	0.06279
CF3I	306.85	558.5	49.005	-1.467	0.00862	0.00864	0.00827
CHLORINE	293.138	101.325	2.988	-0.143	0.01297	0.01298	0.01297
CHLOROBENZENE	457.961	4900	925.784	-35.442	0.102	0.10199	0.09827
CO	296.886	101.325	1.15	-0.019	0.02489	0.02487	0.02481
CO2	325.102	5575	119.842	-3.003	0.02434	0.02434	0.02439
CYCLOHEX	453.111	101	2.293	-0.166	0.0285	0.02851	0.02866
CYCLOPEN	355.978	146100	792.254	-46.319	0.1621	0.16208	0.16161
CYCLOPRO	399.129	101.325	1.293	-0.056	0.02791	0.0279	0.02778
D2	338.15	101.1	0.145	-0.006	0.14403	0.144	0.14457
D2O	624.686	98.1	0.379	-0.027	0.0508	0.0508	0.0506
D4	453.35	5990	786.686	-52.863	0.0838	0.0838	0.08383
D5	303.62	2000	950.906	-106.672	0.1155	0.1155	0.11668
DEA	383.3	0.1351	1034.418	-98.821	0.2126	0.21413	0.21356
DECANE	553.111	14000	550.292	-37.256	0.0978	0.0978	0.09001
DEE	422.134	101.325	2.169	-0.169	0.02778	0.02774	0.02761
DMC	290.675	10136	1081.881	-60.284	0.1751	0.17506	0.17346
DME	335.66	152.2	2.559	-0.204	0.02198	0.02198	0.02195
EBENZENE	464.611	365.1868	11.233	-1.466	0.026	0.02598	0.02525
EGLYCOL	413.116	101.325	1024.381	-71.974	0.259	0.25885	0.25487
ETHANE	307.632	3230	51.961	-2.727	0.02725	0.02725	0.02737
ETHANOL	474.11	2550	38.667	-5.634	0.0441	0.04407	0.04267
ETHYLENE	371.425	4840	51.01	-1.779	0.03514	0.03513	0.03527
ETHYLENEOXIDE	273.15	65.7302	1.307	-0.39	0.01034	0.01034	0.01028
FLUORINE	423.15	101.1	1.092	-0.01	0.0368	0.03684	0.03479
H2S	297.042	10556.53	799.975	-24.366	0.154	0.15397	0.16006
HCL	478.05	101.325	0.931	-0.015	0.02335	0.0234	0.02323
HELIUM	298.944	6880	10.73	-0.238	0.1596	0.15962	0.16008
HEPTANE	338.133	101.325	644.712	-48.969	0.1131	0.11311	0.11035
HEXANE	333.134	76.3802	621.938	-42.97	0.109	0.10902	0.10997

REFPROP fluid name	T/ K	p/ kPa	ρ_{EoS}/ kg·m^{-3}	s_{res}/ J·mol^{-1}·K^{-1}	λ_{exp}/ W·m^{-1}·K^{-1}	λ_{RES}/ W·m^{-1}·K^{-1}	$\lambda_{REFPROP}$/ W·m^{-1}·K^{-1}
HYDROGEN	274.049	13.999	0.012	-0.001	0.1722	0.17219	0.17379
IBUTENE	330.8	29230	598.165	-32.297	0.1079	0.10788	0.10878
IHEXANE	438.15	1186.426	486.862	-25.228	0.0779	0.07807	0.06992
IOCTANE	400	40000	657.344	-44.619	0.0944	0.09439	0.09271
IPENTANE	293.138	101.325	620.076	-40.065	0.111	0.11094	0.10323
ISOBUTAN	504.861	6885	141.929	-5.504	0.05428	0.05428	0.05491
KRYPTON	463	101.325	2.206	-0.012	0.01372	0.01372	0.01365
MDM	372.58	6000	751.692	-62.595	0.09	0.09002	0.09021
MEA	356.3	2.8286	965.054	-78.516	0.2329	0.23307	0.23386
METHANE	198.651	14450	296.573	-14.03	0.0968	0.0968	0.09688
METHANOL	308.4	10000	786.49	-58.539	0.2052	0.20521	0.20299
MLINOLEA	351.901	19964	857.815	-141.283	0.1431	0.14311	0.1423
MM	401.61	4016	654.379	-41.383	0.08115	0.08115	0.08088
MOLEATE	301.84	9878	873.397	-169.696	0.14523	0.14524	0.14486
MSTEARAT	493.15	2.2497	720.828	-86.581	0.1277	0.12783	0.1165
MXYLENE	306.242	58839.9	889.914	-66.065	0.1447	0.1447	0.14503
N2O	364.083	101.325	1.477	-0.032	0.02303	0.023	0.02294
NEON	321.977	101.325	0.763	-0.005	0.05172	0.0517	0.05158
NITROGEN	326.666	1396	14.393	-0.219	0.02871	0.0287	0.0283
NONANE	297.624	0.5617	714.42	-72.361	0.1316	0.13161	0.1268
OCTANE	417.865	101.325	3.495	-0.67	0.02183	0.02181	0.02207
OXYGEN	167.31	101.325	2.343	-0.045	0.01538	0.01538	0.01535
OXYLENE	306.242	58839.9	905.055	-66.247	0.1454	0.14539	0.14605
PENTANE	305.792	35420	651.036	-45.257	0.1283	0.12837	0.12858
PROPANE	375.9	4650	187.506	-9.123	0.06572	0.06572	0.06554
PROPYLEN	359.973	100	1.417	-0.073	0.0245	0.0245	0.02432
PROPYLENEOXIDE	303.15	87.0244	817.436	-42.405	0.1479	0.14801	0.16438
PROPYNE	353.15	2244.418	510.949	-23.378	0.1013	0.10165	0.0923
PXYLENE	480.31	473.7448	678.311	-33.619	0.088	0.08799	0.0878
R11	316.239	58940	1557.83	-41.829	0.1044	0.10441	0.10087
R113	429.963	2000	1186.127	-24.764	0.049	0.04899	0.04734
R114	332.884	40000	1504.072	-37.961	0.0729	0.07284	0.07247
R115	213.56	39320	1705.097	-50.422	0.092	0.092	0.09208
R116	248.575	908.7636	1326.235	-27.139	0.0673	0.06751	0.05471
R12	347.231	100	4.237	-0.114	0.0125	0.0125	0.0124
R1224YDZ	356.95	2000	1186.125	-30.785	0.05826	0.05826	0.06145
R123	283.758	50020	1594.316	-51.504	0.0972	0.0972	0.09691
R1233ZDE	242.54	7100	1400.37	-54.602	0.10332	0.10332	0.1027
R1234YF	301.619	199	9.45	-0.516	0.0142	0.0142	0.0141
R1234ZEE	261.996	10412	1300.242	-43.098	0.0925	0.0925	0.09245
R124	315.51	30200	1422.52	-37.129	0.0784	0.07841	0.07875
R125	251.905	42	2.435	-0.151	0.01066	0.01066	0.01062
R13	338.133	100	3.738	-0.06	0.01502	0.01502	0.01496
R1336MZZZ	455.38	503	23.15	-0.839	0.02393	0.02392	0.02393
R134A	263.15	101.325	4.887	-0.493	0.01065	0.01065	0.01059
R14	153.564	39320	1679.493	-40.108	0.1076	0.10761	0.10882
R141B	326.797	140	6.287	-0.59	0.01222	0.01223	0.01198
R142B	303.589	1642.5	1102.034	-32.96	0.08103	0.08103	0.08014
R143A	473	14000	417.768	-8.505	0.04889	0.04889	0.04995
R150	382.526	93.331	2.968	-0.21	0.01346	0.01346	0.01341
R152A	321.36	20000	909.236	-30.837	0.10744	0.10744	0.10228

REFPROP fluid name	T/ K	p/ kPa	ρ_{EoS}/ kg·m^{-3}	s_{res}/ J·mol^{-1}·K^{-1}	λ_{exp}/ W·m^{-1}·K^{-1}	λ_{RES}/ W·m^{-1}·K^{-1}	$\lambda_{REFPROP}$/ W·m^{-1}·K^{-1}
R161	333.74	396.8	7.183	-0.553	0.01972	0.01972	0.01966
R21	375.824	39320	1298.1	-31.152	0.0946	0.0946	0.094
R218	386.172	101.325	5.984	-0.114	0.02001	0.02	0.0199
R22	232.758	2060	1412.409	-40.182	0.1155	0.1155	0.11398
R227EA	292.861	142	10.347	-0.572	0.01308	0.01308	0.01296
R23	130.764	19710	1681.94	-62.09	0.1819	0.1819	0.23737
R236EA	319.48	85	4.975	-0.302	0.01571	0.01571	0.01561
R236FA	332.168	315	18.658	-0.967	0.01553	0.01553	0.01547
R245CA	293.6	60	3.375	-0.287	0.01281	0.01281	0.01271
R245FA	297.06	121.3	6.894	-0.704	0.01257	0.01257	0.01252
R32	454.01	10000	186.249	-4.577	0.03854	0.03854	0.03534
R365MFC	349.13	101.3	5.326	-0.42	0.01724	0.01731	0.01717
R40	341.424	101.325	1.822	-0.126	0.01345	0.01345	0.01329
R41	259.15	1369.595	722.802	-24.455	0.1894	0.18953	0.16593
RC318	323.1	238	18.762	-0.613	0.01378	0.01378	0.0137
RE245CB2	298.15	6010	1292.126	-40.78	0.0737	0.07369	0.07383
RE245FA2	313.02	67	3.958	-0.292	0.01428	0.01428	0.01423
RE347MCC	296.15	66.2244	1410.17	-48.286	0.0753	0.0753	0.06561
SF6	260.003	101.325	6.969	-0.179	0.0102	0.01021	0.01017
SO2	650	100	1.186	-0.021	0.0276	0.02759	0.02788
TOLUENE	326.186	176500	933.903	-63.615	0.1677	0.1677	0.16841
VINYLCHLORIDE	573.184	101.325	1.332	-0.031	0.0368	0.03683	0.03711
WATER	849.543	100	0.255	-0.009	0.076	0.07601	0.07615
XENON	1069	101.1	1.493	-0.005	0.0172	0.0172	0.01599
WATER	284.143	0.1	0.001	-0.003	0	0.0173	0.01748
ETHANOL	284.143	0.2	0.004	-0.004	0	0.01398	0.01395

[a] Temperature T, pressure p, thermal conductivity λ_{exp} are experimental values from literature; density ρ_{EoS} and residual entropy s_{res} are calculated with reference equations of state implemented in REFPROP 10.0; λ_{RES} and $\lambda_{REFPROP}$ are calculated thermal conductivities with the residual entropy scaling (RES) and the recommended model in REFPROP 10.0, respectively.

Table S7 Sample thermal conductivity calculations of binaries with the recommended models in REFPROP 10.0 and the RES model.[a]

Mixture	Group	x_1	T/ K	p/ kPa	ρ_{EoS}/ kg·m^{-3}	s_{res}/ J·mol^{-1}·K^{-1}	λ_{EXP}/ W·m^{-1}·K^{-1}	λ_{RES}/ W·m^{-1}·K^{-1}	$\lambda_{REFPROP}$/ W·m^{-1}·K^{-1}
ARGON +NEON	2+2	0.4	393.97	101	0.866	-0.006	0.04	0.03984	0.0398
BENZENE +C16	4+5	0.970108	294.795	101	872.25	-52.287	0.14	0.13763	0.14823
BUTANE +METHANE	3+2	0.606	377.567	24131.7	418.548	-18.135	0.081	0.08121	0.08616
C11 +C16	5+5	0.812946	294.545	101.325	747.806	-95.556	0.137	0.13609	0.13946
CO2 +ETHANE	3+3	0.5	318.11	1091.673	16.112	-0.554	0.022	0.02172	0.02165
CO2 +NITROGEN	3+2	0.335	423.134	101	0.958	-0.014	0.032	0.03136	0.03132
D2 +HELIUM	1+1	0.2965	303.134	101	0.161	-0.005	0.141	0.14785	0.14771
ETHANOL +WATER	7+8	0.281115	284.143	101	917.723	-70.897	0.33	0.28867	/

[a] Temperature T, pressure p, thermal conductivity λ_{exp} are experimental values from literature; x_1 is model fraction of

component one in the 'Mixture' column; density ρ_{EoS} and residual entropy s_{res} are calculated with reference equations of state implemented in REFPROP 10.0; λ_{RES} and $\lambda_{\mathrm{REFPROP}}$ are calculated thermal conductivities with the residual entropy scaling (RES) and the recommended models in REFPROP 10.0, respectively.

3. Thermal conductivity as a function of residual entropy for each group

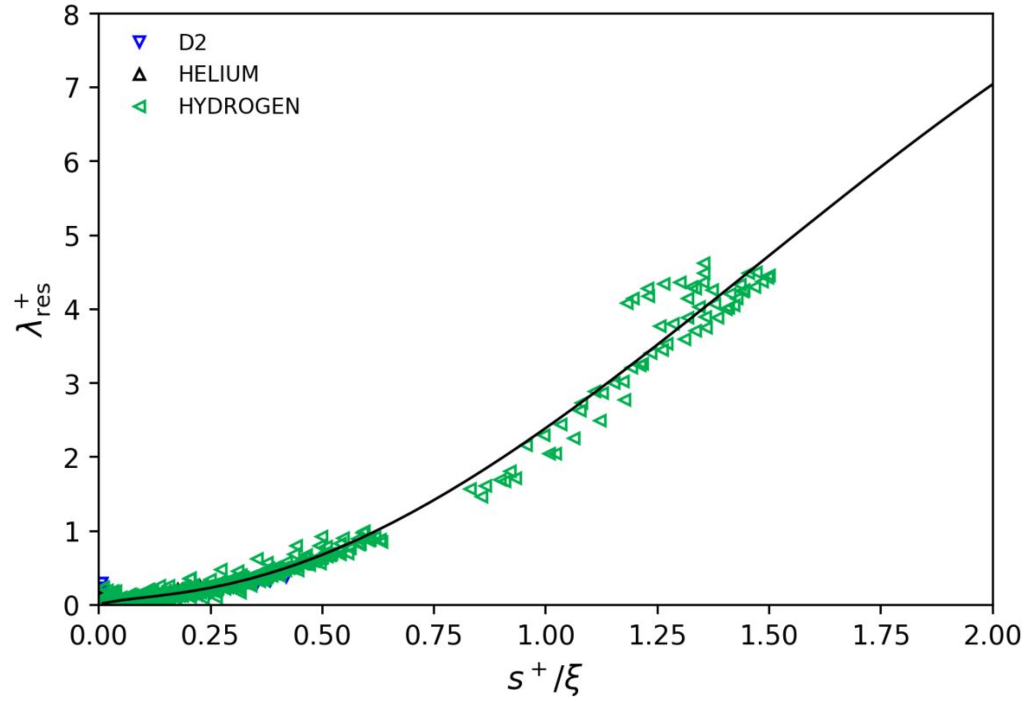

Figure S3-1 Values of λ+ res as a function of s^+/ξ for pure fluids of group 1, where is λ+ res the plus-scaled residual thermal conductivity, s^+ is the plus-scaled residual entropy, and ξ is the scaling factor.

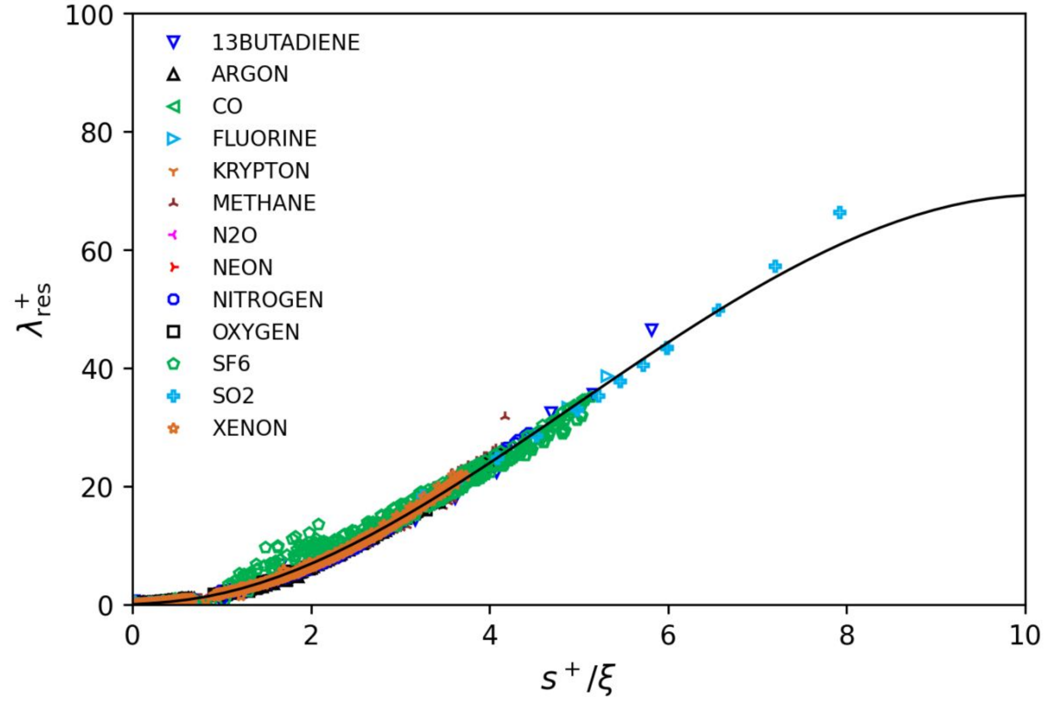

Figure S3-2 Values of λ+ res as a function of s^+/ξ for pure fluids of group 2, where is λ+ res the plus-scaled residual thermal conductivity, s^+ is the plus-scaled residual entropy, and ξ is the scaling factor.

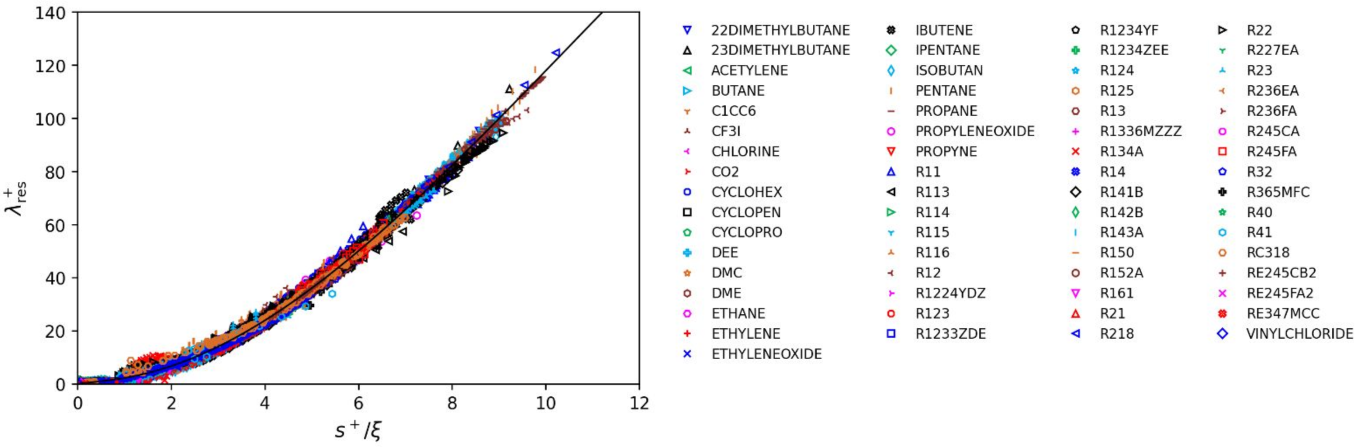

Figure S3-3 Values of λ+ res as a function of s^+/ξ for pure fluids of group 3, where is λ+ res the plus-scaled residual thermal conductivity, s^+ is the plus-scaled residual entropy, and ξ is the scaling factor.

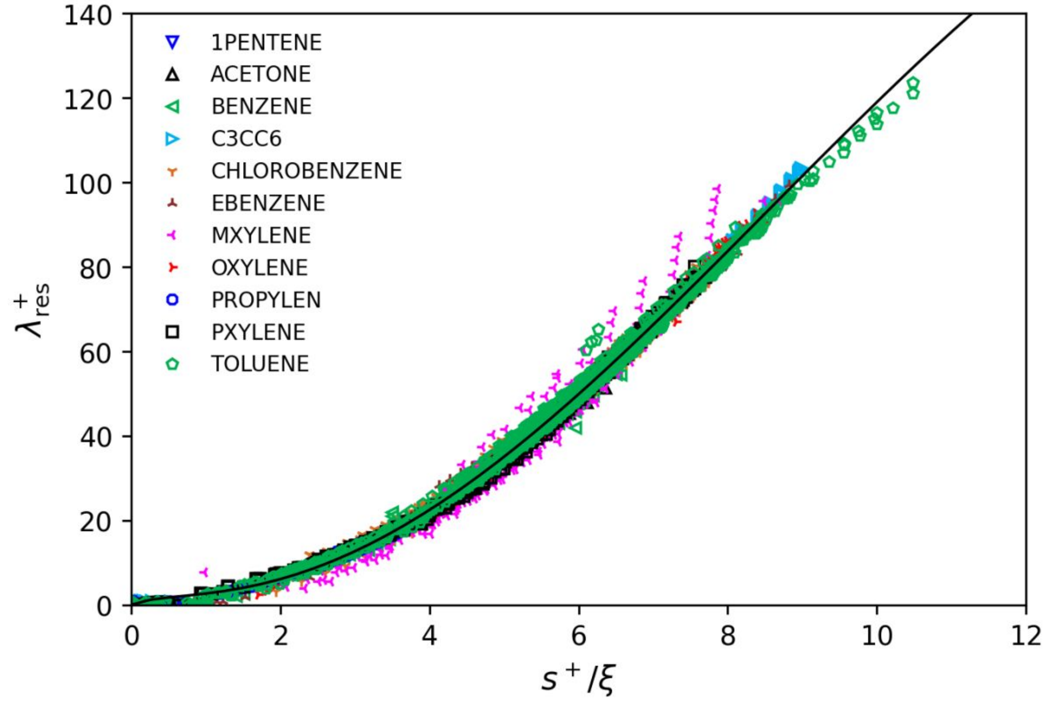

Figure S3-4 Values of λ+ res as a function of s^+/ξ for pure fluids of group 4, where is λ+ res the plus-scaled residual thermal conductivity, s^+ is the plus-scaled residual entropy, and ξ is the scaling factor.

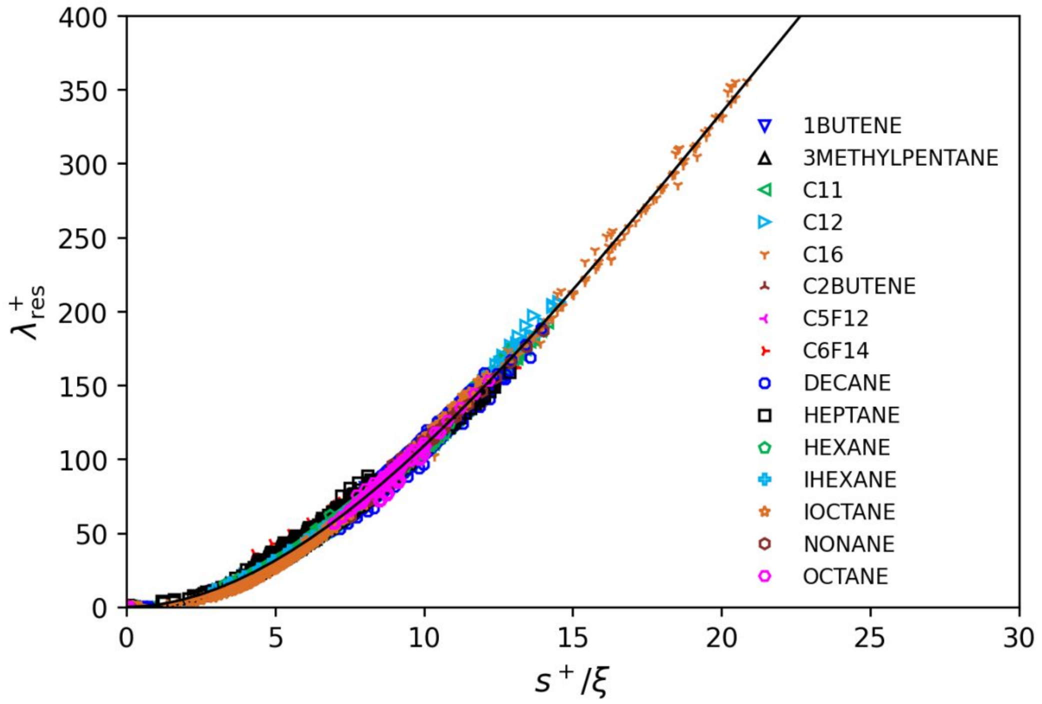

Figure S3-5 Values of λ+ res as a function of s^+/ξ for pure fluids of group 5, where is λ+ res the plus-scaled residual thermal conductivity, s^+ is the plus-scaled residual entropy, and ξ is the scaling factor.

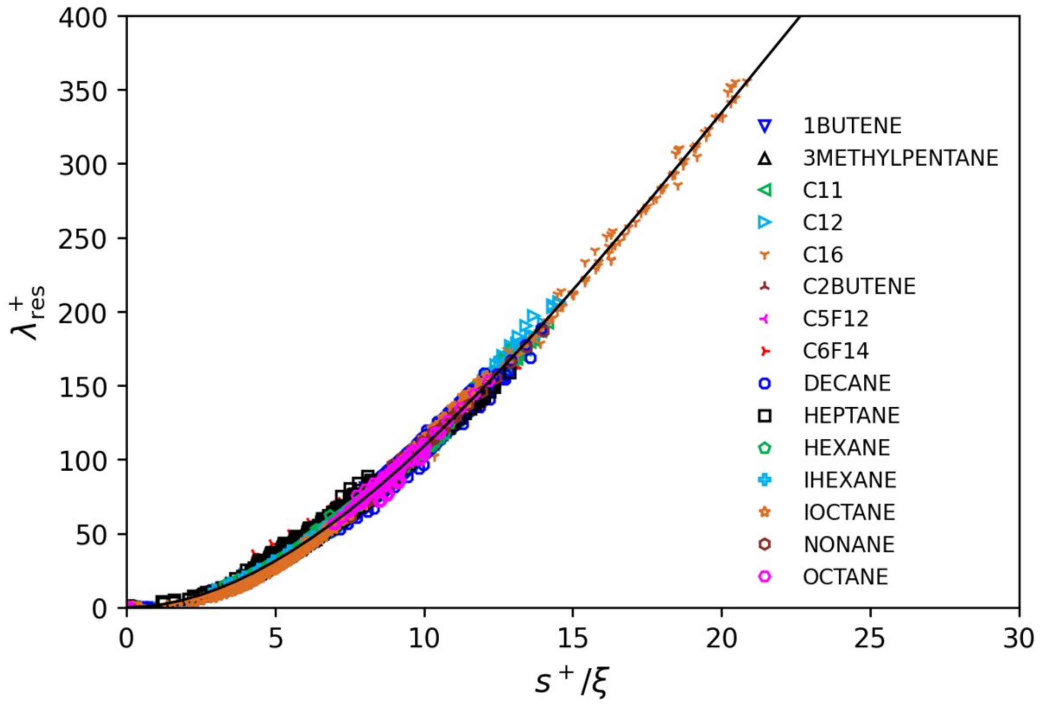

Figure S3-6 Values of λ+ res as a function of s^+/ξ for pure fluids of group 6, where is λ+ res the plus-scaled residual thermal conductivity, s^+ is the plus-scaled residual entropy, and ξ is the scaling factor.

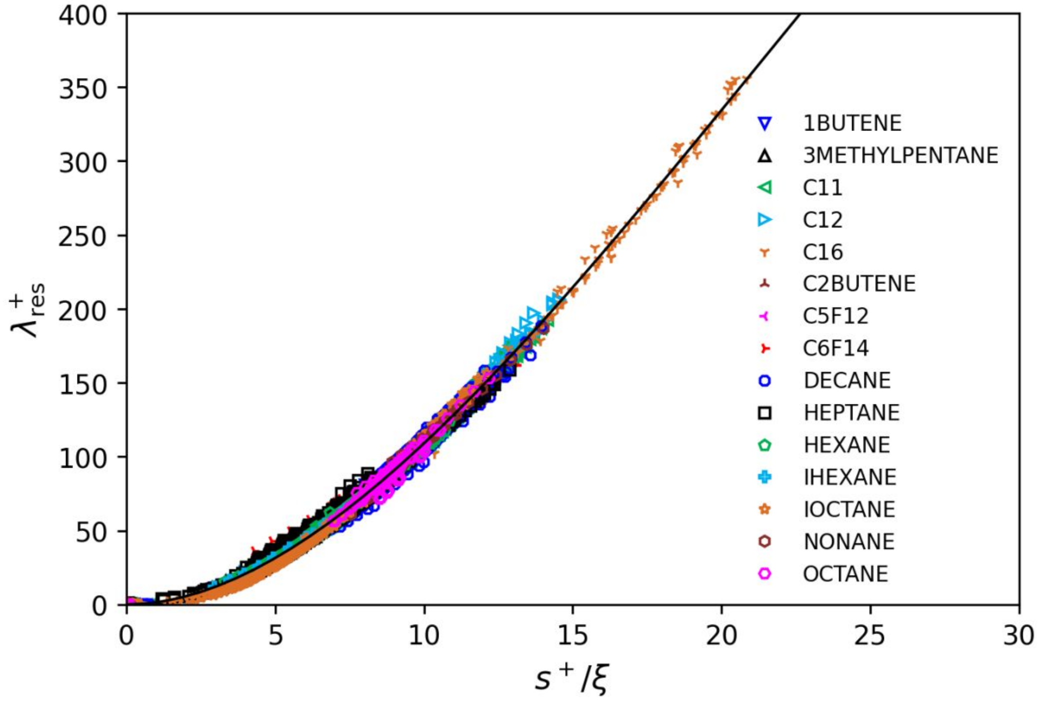

Figure S3-7 Values of λ+ res as a function of s^+/ξ for pure fluids of group 7, where is λ+ res the plus-scaled residual thermal conductivity, s^+ is the plus-scaled residual entropy, and ξ is the scaling factor.

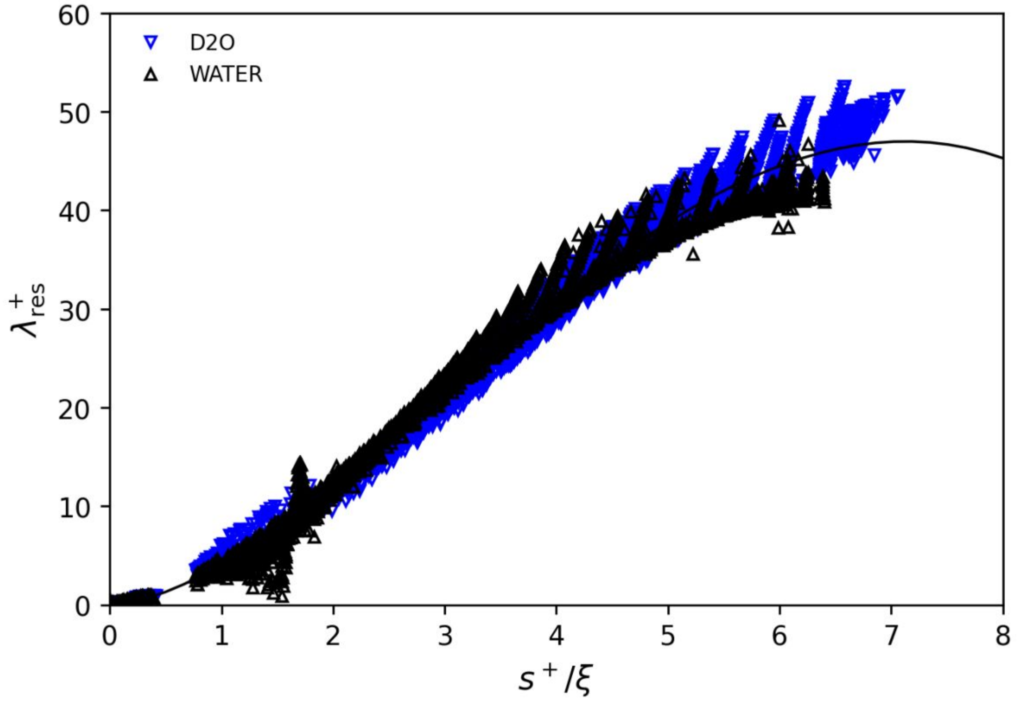

Figure S3-8 Values of λ+ res as a function of s^+/ξ for pure fluids of group 8, where is λ+ res the plus-scaled residual thermal conductivity, s^+ is the plus-scaled residual entropy, and ξ is the scaling factor.

4. Relative deviation from experimental data of pure fluids to model predictions

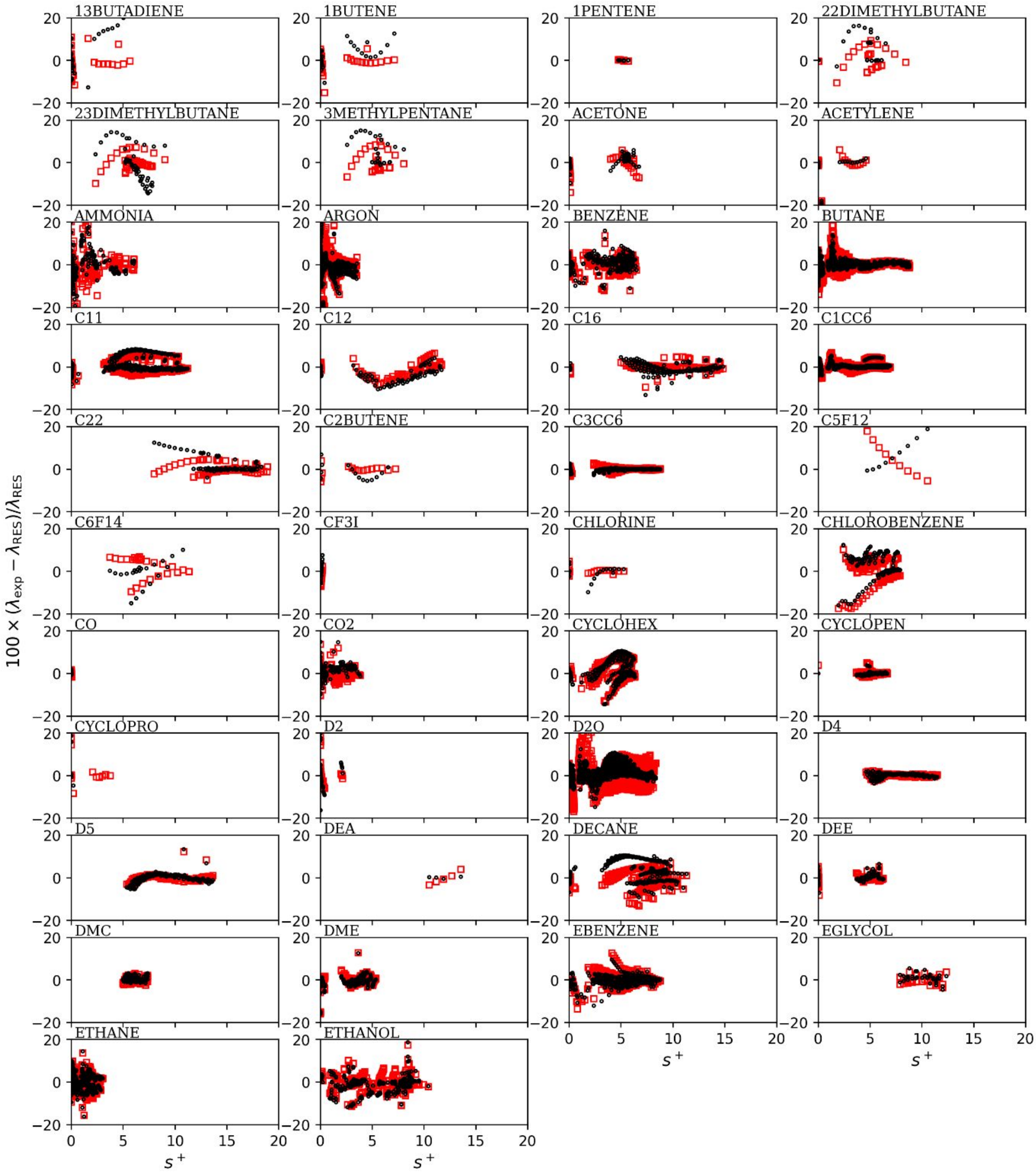

Figure S4-1 Relative deviation of the experimental thermal conductivity λ_{exp} from values λ_{RES} calculated with the RES model (□) and the recommended models in REFPROP 10.0 (∘).

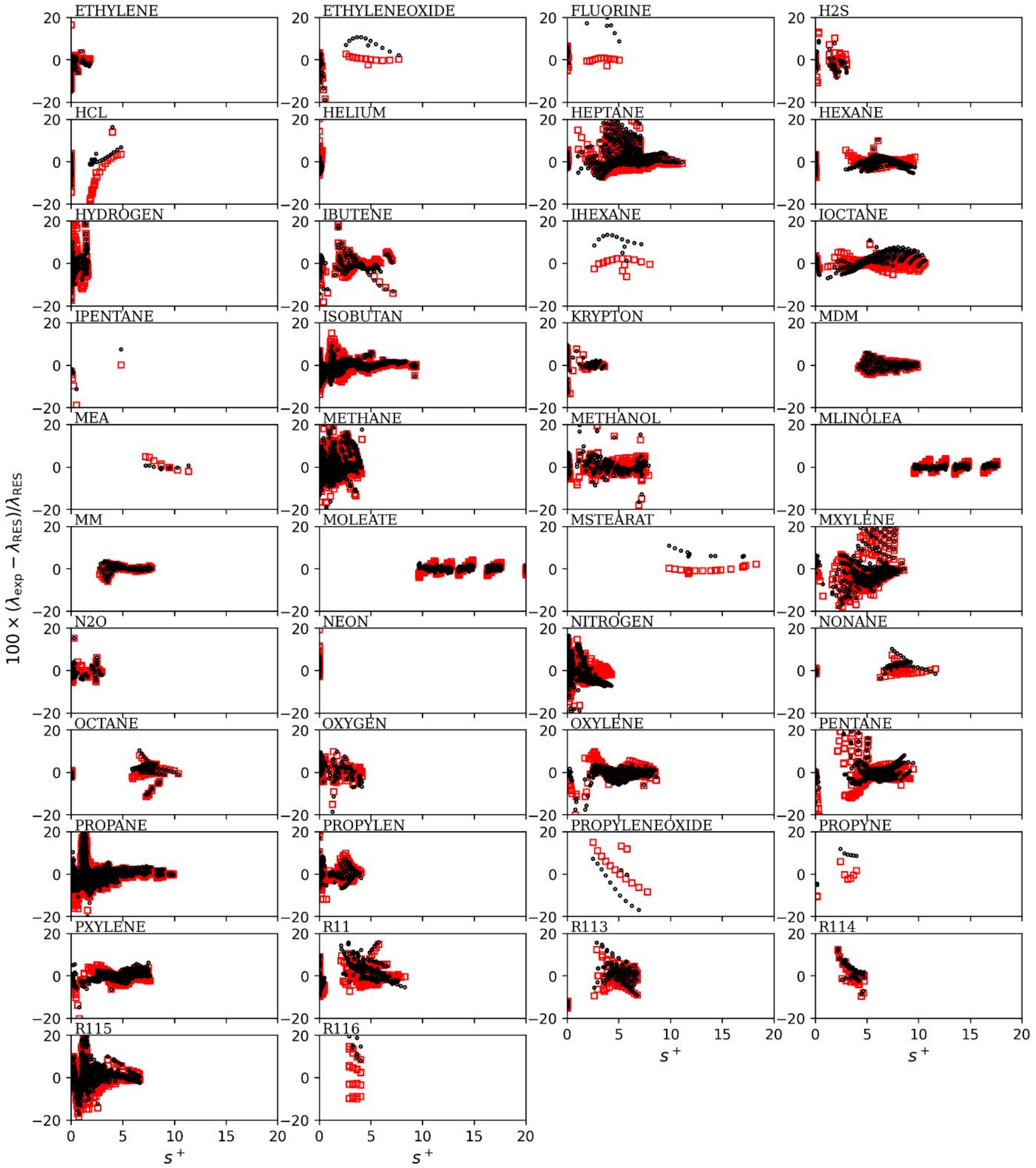

Figure S4-2 Relative deviation of the experimental thermal conductivity λ_{exp} from values λ_{RES} calculated with the RES model (□) and the recommended models in REFPROP 10.0 (∘).

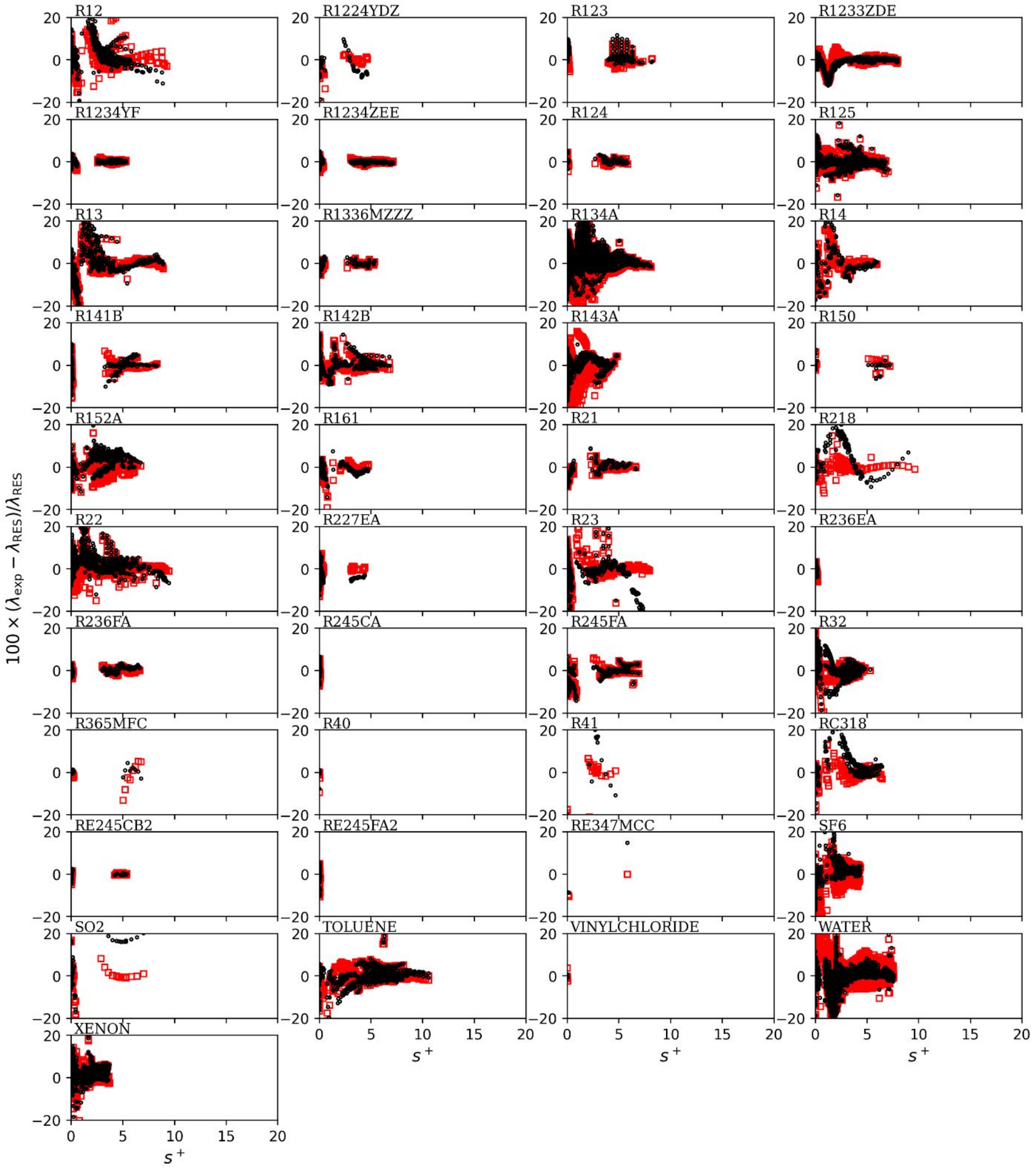

Figure S4-3 Relative deviation of the experimental thermal conductivity λ_{exp} from values λ_{RES} calculated with the RES model (□) and the recommended models in REFPROP 10.0 (∘).

5. Relative deviation from experimental data of fluid mixtures to model predictions

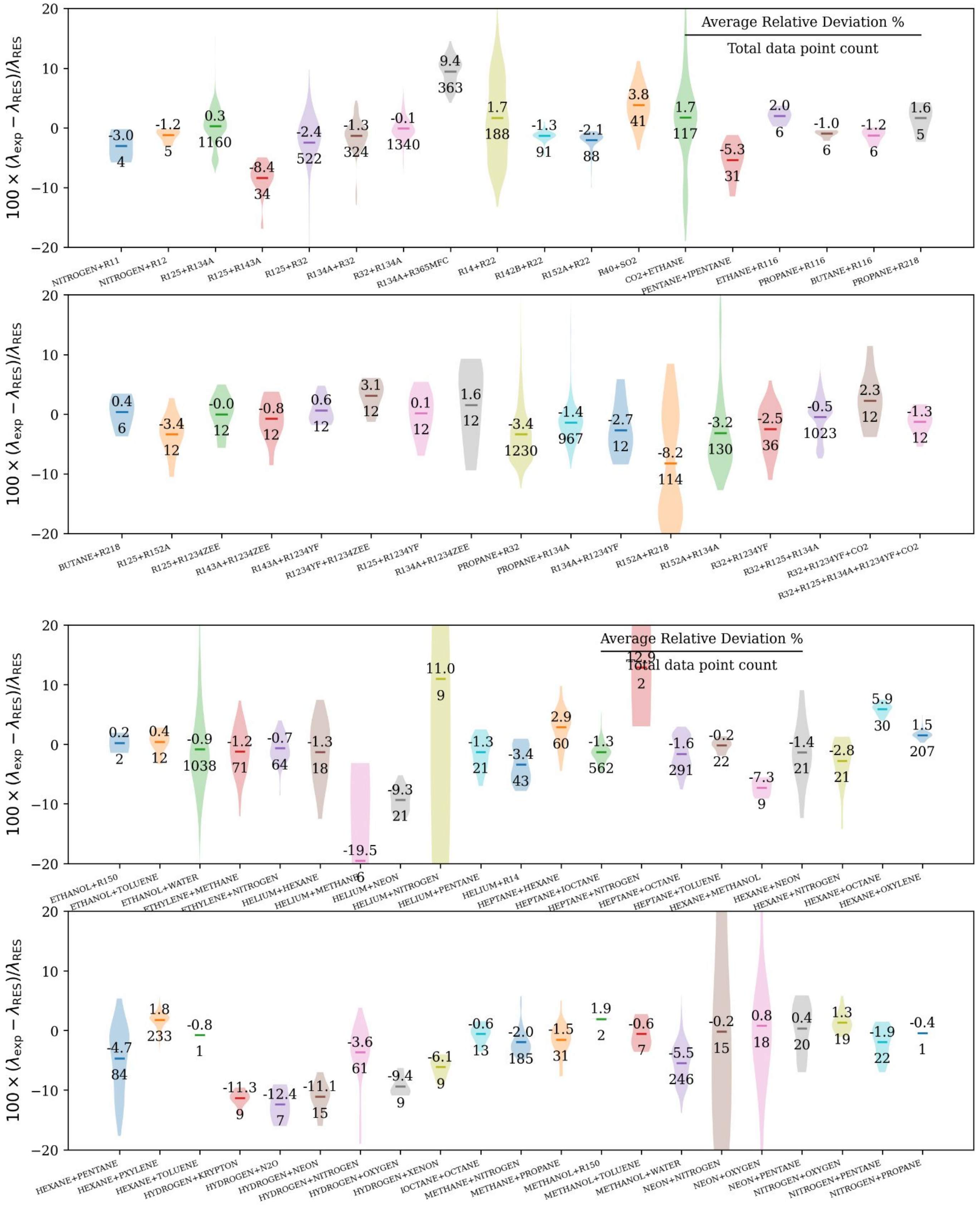

Figure S5-1. Relative deviations of the experimental thermal conductivity λ_{exp} of selected mixtures from values λ_{RES} calculated with the RES model. The short line indicates the average relative deviation.

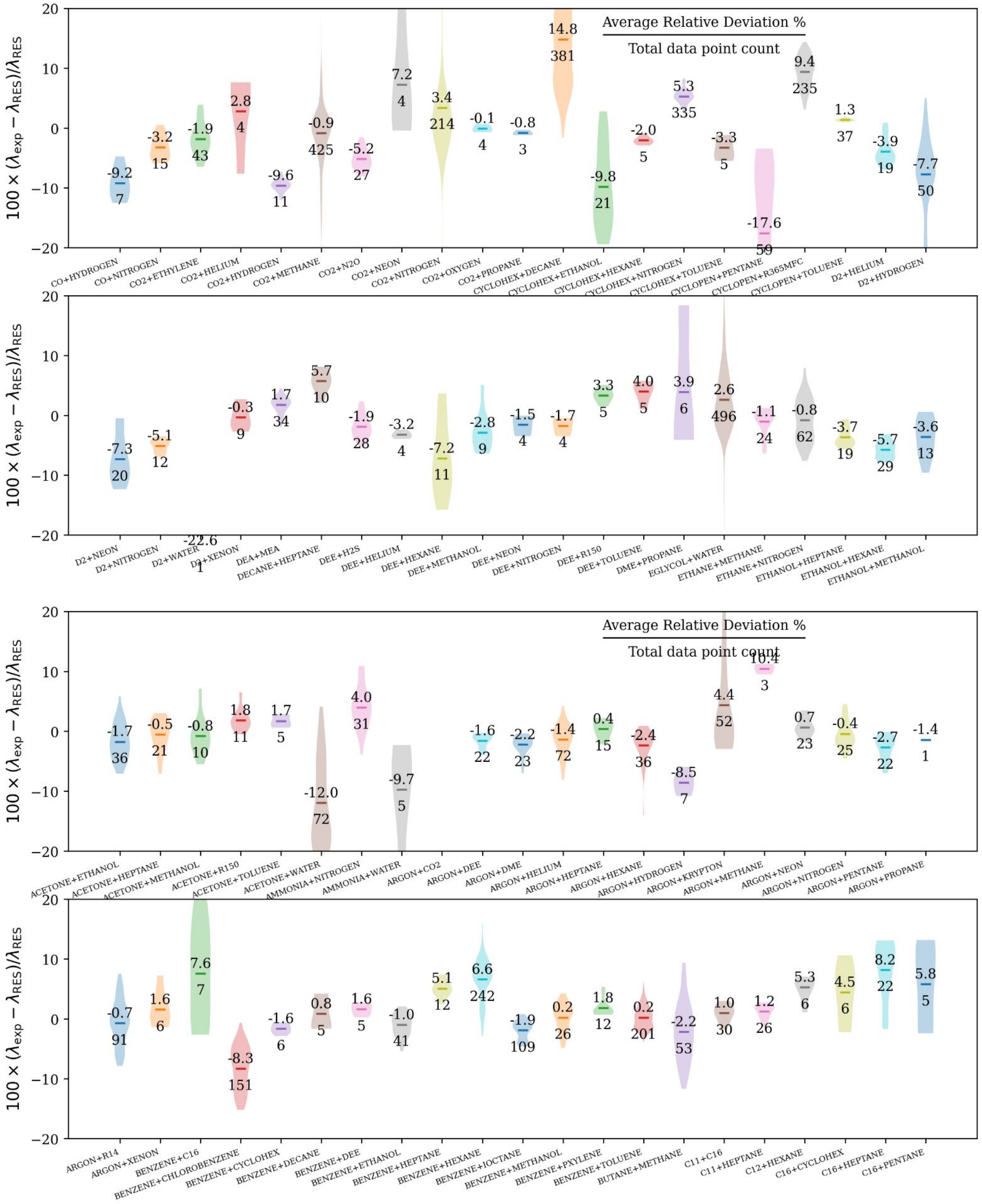

Figure S5-2. Relative deviations of the experimental thermal conductivity λ_{exp} of selected mixtures from values λ_{RES} calculated with the RES model. The short line indicates the average relative deviation.

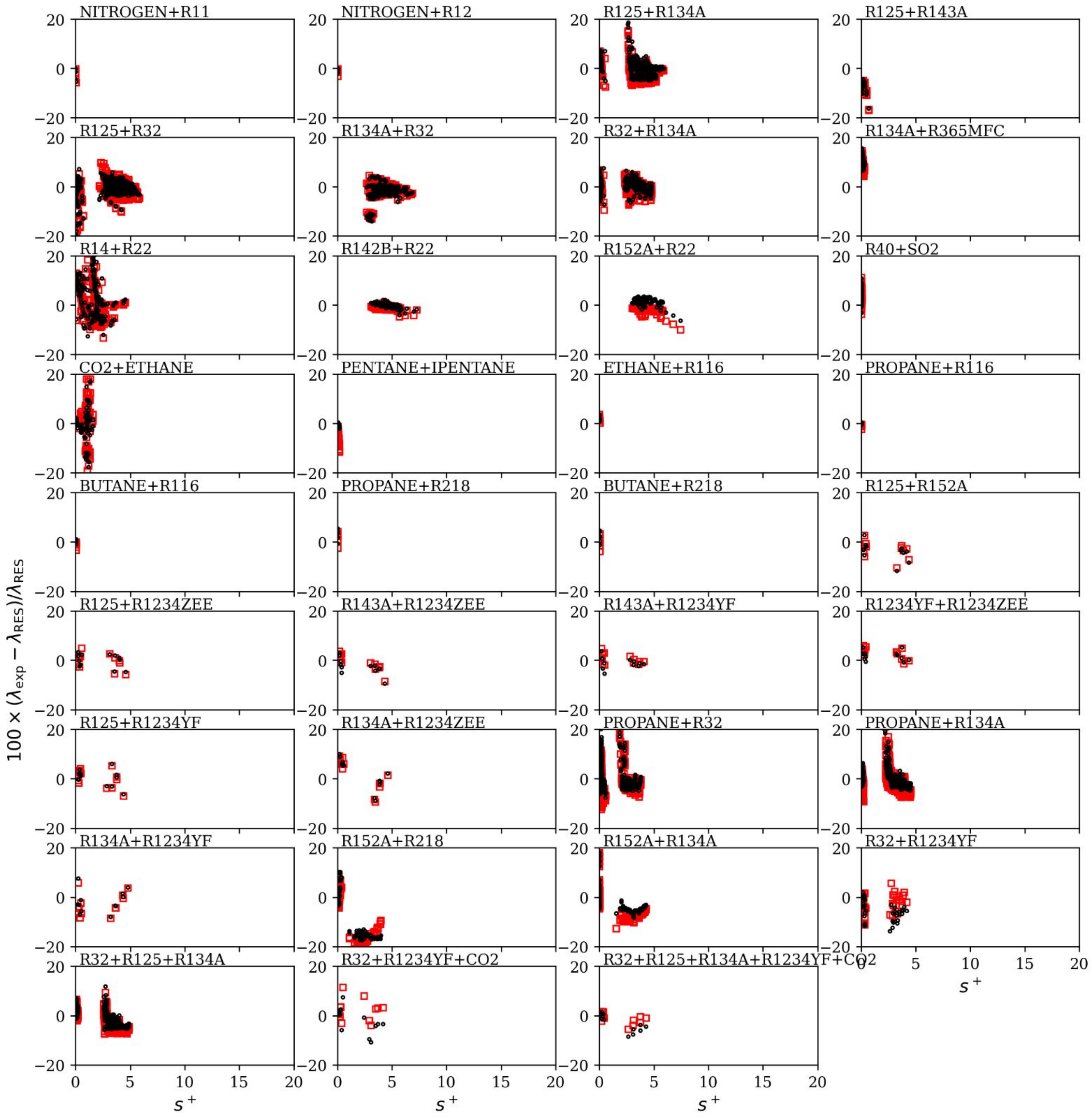

Figure S5-3 Relative deviation of the experimental thermal conductivity λ_{exp} from values λ_{RES} calculated with the RES model (□), the recommended models in REFPROP 10.0 (∘).

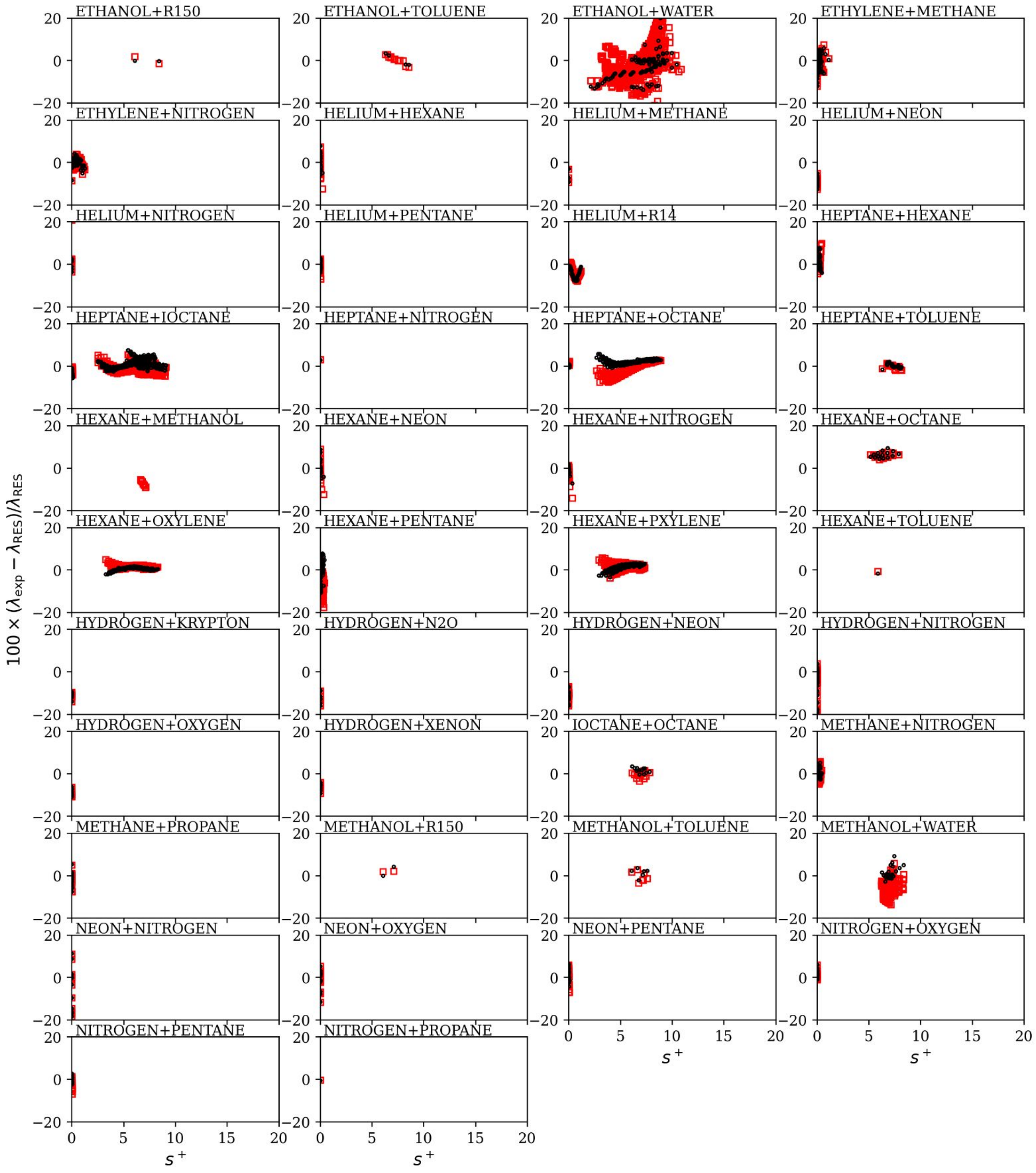

Figure S5-4 Relative deviation of the experimental thermal conductivity λ_{exp} from values λ_{RES} calculated with the RES model (□), the recommended models in REFPROP 10.0 (∘).

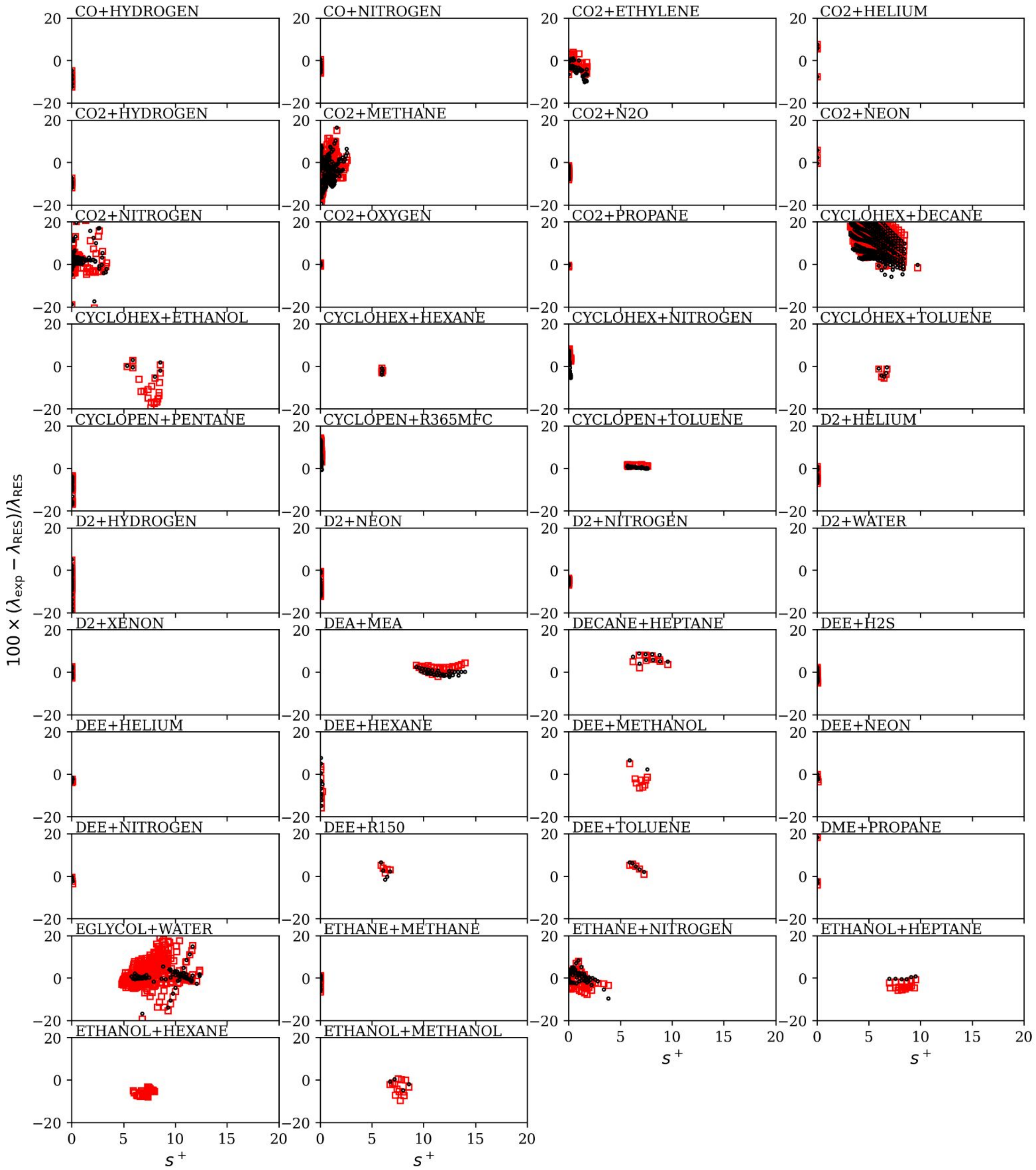

Figure S5-5 Relative deviation of the experimental thermal conductivity λ_{exp} from values λ_{RES} calculated with the RES model (□), the recommended models in REFPROP 10.0 (∘).

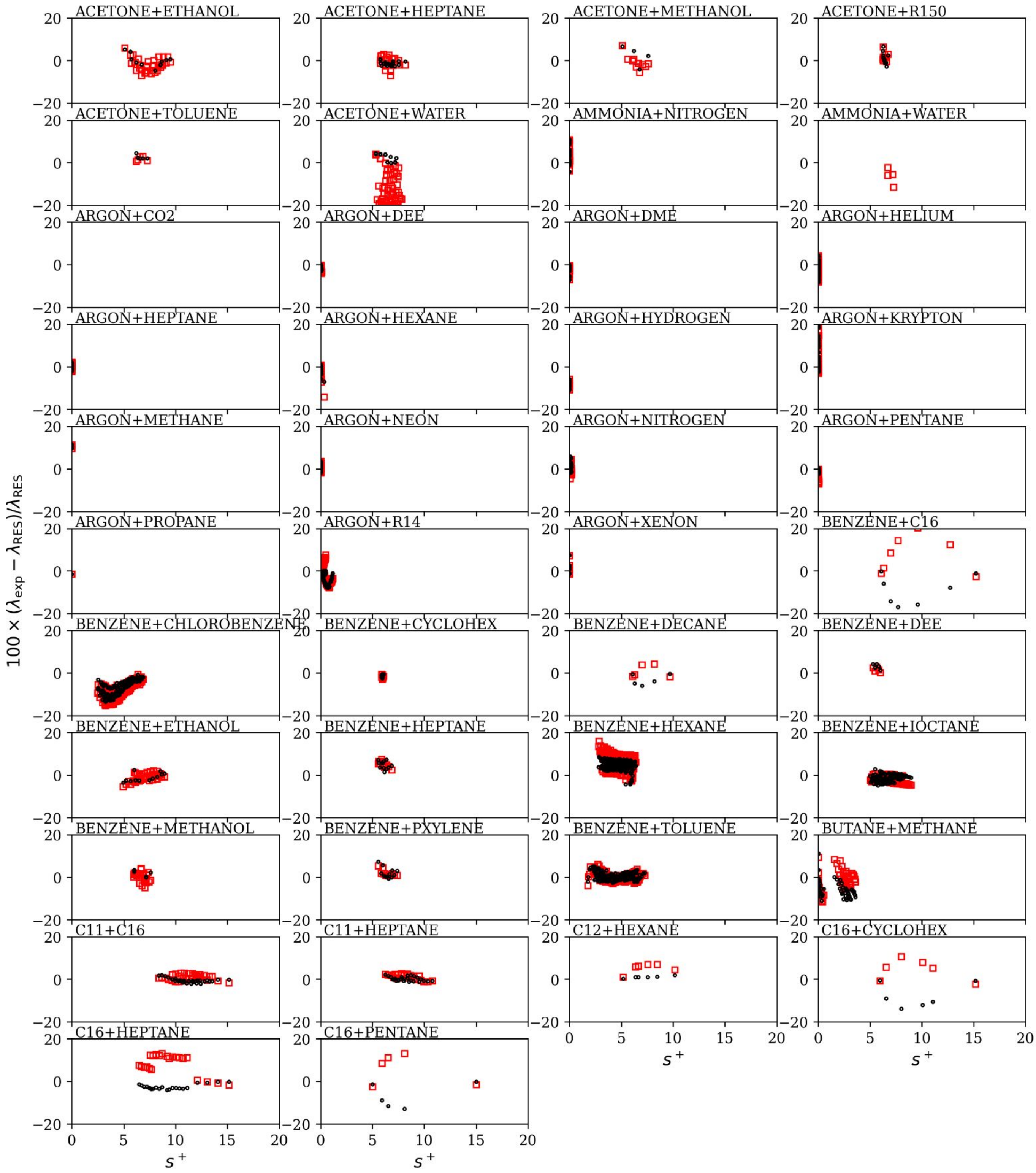

Figure S5-6 Relative deviation of the experimental thermal conductivity λ_{exp} from values λ_{RES} calculated with the RES model (□), the recommended models in REFPROP 10.0 (∘).

6. Reference

(1) Lemmon, E. W.; Bell, I.H.; Huber, M. L.; McLinden, M. O. NIST Standard Reference Database 23, NIST Reference FluidThermodynamic andTransport Properties, version 10.0; Standard Reference Data Program, National Institute of Standards and Technology: Gaithersburg, MD, 2018.

(2) Huber, M. L. Models for the Viscosity, Thermal Conductivity, and Surface Tension of Selected Pure

Fluids as Implemented in REFPROP v10.0. NIST Interagency/Internal Report (NISTIR) 2018, No. 8209.
(3) Lemmon, E. W.; Ihmels, E. C. Thermodynamic Properties of the Butenes. Part II. Short Fundamental Equations of State. FluidPhase Equilib. 2005, 228−229, 173−187.
(4) Gao, K. H.; Wu, J.T.; Lemmon, E. W. Equations of State for the Thermodynamic Properties of Three Hexane Isomers: 3-Methylpentane, 2,2-Dimethylbutane, and 2,3-Dimethylbutane. J.Phys.Chem. Ref. Data 2021, 50, 033103.
(5) Lemmon, E. W.; Span, R. Short fundamental equations of state for 20 industrial fluids. J.Chem. Eng.Data 2006, 51, 785−850
(6) Monogenidou, S. A.; Assael, M. J.; Huber, M. L. Reference Correlation for the Thermal Conductivity of Ammonia from the TriplePoint Temperature to 680 K and Pressures up to 80 MPa. J.Phys.Chem. Ref.Data 2018, 47, 023102.
(7) Tegeler, C.; Span, R.; Wagner, W. A New Equation of State for Argon Covering the Fluid Region for Temperatures from the Melting Line to 700 K at Pressures up to 1000 MPa. J.Phys.Chem. Ref.Data 1999, 28, 779−850.
(8) Lemmon, E. W.; Jacobsen, R. T. Viscosity and Thermal Conductivity Equations for Nitrogen, Oxygen, Argon, and Air. Int.J. Thermophys. 2004, 25, 21−69.
(9) Thol, M.; Lemmon, E. W.; Span, R. Equation of State for Benzene for Temperatures from the Melting Line up to 725 K with Pressures up to 500 MPa. HighTemp. -HighPressures 2012, 41, 81−97.
(10) Assael, M. J.; Mihailidou, E. K.; Huber, M. L.; Perkins, R. A. Reference Correlation of the Thermal Conductivity of Benzene from the Triple Point to 72
(11) Bücker, D.; Wagner, W. Reference equations of state for the thermodynamic properties of fluid phase n-butane and isobutane. J. Phys.Chem. Ref.Data 2006, 35, 929−1019.
(12) Perkins, R. A.; Ramires, M. L. V.; Nieto de Castro, C. A.; Cusco, L. Measurement and correlation of the thermal conductivity of butane from 135 to 600 K at pressures to 70 MPa. J.Chem. Eng.Data 2002, 47, 1263−1271.
(13) Aleksandrov, I. S.; Gerasimov, A. A.; Grigor'ev, B. A. Using Fundamental Equations of State for Calculating the Thermodynamic Properties of Normal Undecane. Therm. Eng. 2011, 58, 691−698.
(14) Assael, M. J.; Papalas, T. B.; Huber, M. L. Reference Correlations for the Viscosity and Thermal Conductivity of nUndecane. J.Phys.Chem. Ref.Data 2017, 46, 033103
(15) Lemmon, E. W.; Huber, M. L. Thermodynamic properties of ndodecane. Energy Fuels 2004, 18, 960−967.
(16) Huber, M. L.; Laesecke, A.; Perkins, R. Transport properties of ndodecane. Energy Fuels 2004, 18, 968−975
(17) Romeo, R.; Lemmon, E. W. Thermodynamic Properties of nhexadecane and n-docosane. Int.J. Thermophys. 2022.
(18) Monogenidou, S. A.; Assael, M. J.; Huber, M. L. Reference Correlations for Thermal Conductivity of n-Hexadecane from the Triple Point to 700 K and up to 50 MPa. J.Phys.Chem. Ref.Data 2018, 47, 013103.
(19) Perkins, R. A.; Hammerschmidt, U.; Huber, M. L. Measurement and correlation of the thermal conductivity of methylcyclohexane and propylcyclohexane from (300 to 600) K at pressures to 60 MPa. J. Chem. Eng.Data 2008, 53, 2120−2127.
(20) Perkins, R. A.; Hammerschmidt, U.; Huber, M. L. Measurement and correlation of the thermal conductivity of methylcyclohexane and propylcyclohexane from (300 to 600) K at pressures to 60 MPa. J. Chem. Eng.Data 2008, 53, 2120−2127.
(21) Gao, K.; Köster, A.; Thol, M.; Wu, J. T.; Lemmon, E. W. Equations of State for the Thermodynamic Properties of nPerfluorobutane, n-Perfluoropentane, and n-Perfluorohexane. Ind. Eng.Chem. Res. 2021, 60, 17207−17227.
(22) Lemmon, E. W.; Span, R. Thermodynamic Properties of R227ea, R-365mfc, R-115, and R-13I1. J.Chem. Eng.Data 2015, 60, 3745−3758.
(23) Thol, M.; Herrig, S.; Span, R.; Lemmon, E. W. A fundamental equation of state for the calculation of thermodynamic properties of chlorine. AIChE J. 2021, 67 (9), No. e17326. (74) Thol, M.; Alexandrov, I. S.; Lemmon, E. W. Unpublished Helmholtz Equation ofStateforChlorobenzene, 2018.
(24) Span, R.; Wagner, W. A new equation of state for carbon dioxide covering the fluid region from the triple-point temperature to 1100 K at pressures up to 800 MPa. J.Phys.Chem. Ref.Data 1996, 25, 1509−1596.
(25) Huber, M. L.; Sykioti, E. A.; Assael, M. J.; Perkins, R. A. Reference Correlation of the Thermal Conductivity of Carbon Dioxide from the Triple Point to 1100 K and up to 200 MPa. J.Phys.Chem. Ref.Data

2016, 45, 013102.
(26) Mulero, A.; Cachadiña, I. Recommended Correlations for the Surface Tension of Several Fluids Included in the REFPROP Program. J.Phys.Chem. Ref.Data 2014, 43, 023104.
(27) Huber, M. L.; Sykioti, E. A.; Assael, M. J.; Perkins, R. A. Reference Correlation of the Thermal Conductivity of Carbon Dioxide from the Triple Point to 1100 K and up to 200 MPa. J.Phys.Chem. Ref.Data 2016, 45, 013102.
(28) Zhou, Y.; Liu, J.; Penoncello, S. G.; Lemmon, E. W. An Equation of State for the Thermodynamic Properties of Cyclohexane. J.Phys. Chem. Ref.Data 2014, 43, 043105.
(29) Koutian, A.; Assael, M. J.; Huber, M. L.; Perkins, R. A. Reference Correlation of the Thermal Conductivity of Cyclohexane from the Triple Point to 640 K and up to 175 MPa. J.Phys.Chem. Ref.Data 2017, 46, 013102.
(30) Gedanitz, H.; Davila, M. J.; Lemmon, E. W. Speed of Sound Measurements and aFundamental Equation of State for Cyclopentane. J.Chem. Eng.Data 2015, 60, 1331−1337.
(31) Vassiliou, C. M.; Assael, M. J.; Huber, M. L.; Perkins, R. A. Reference Correlations of the Thermal Conductivity of Cyclopentane, iso-Pentane, and n-Pentane. J.Phys.Chem. Ref.Data 2015, 44, 033102.
(32) Polt, A.; Platzer, B.; Maurer, G. Parameter der Thermischen Zustandsgleichung von Bender fuer 14 Mehratomige Reine Stoffe. Chem. Technol. (Leipzig) 1992, 44, 216−224.
(33) Richardson, I.A.; Leachman, J.W.; Lemmon, E. W. Fundamental Equation of State for Deuterium. J.Phys.Chem. Ref.Data 2014, 43, 013103.
(34) Assael, M. J.; Assael, J.-A. M.; Huber, M. L.; Perkins, R. A.; Takata, Y. Correlation of the Thermal Conductivity of Normal and Parahydrogen from the Triple Point to 1000 K and up to 100 MPa. J. Phys.Chem. Ref.Data 2011, 40, 033101.
(35) Herrig, S.; Thol, M.; Harvey, A. H.; Lemmon, E. W. AReference Equation of State for Heavy Water. J.Phys.Chem. Ref.Data 2018, 47, 043102.
(36) International Association for the Properties of Water and Steam. Viscosity and Thermal Conductivity of Heavy Water Substance, Physical Chemistry of Aqueous Systems: Proceedings of the 12th International Conference on the Properties of Water and Steam, Orlando, Florida, September 11−16, 1994, pp A107−A138.
(37) Thol, M.; Rutkai, G.; Koster, A.; Dubberke, F. H.; Windmann, T.; Span, R.; Vrabec, J.Thermodynamic Properties of Octamethylcyclotetrasiloxane. J.Chem. Eng.Data 2016, 61, 2580−2595.
(38) Thol, M.; Javed, M. A.; Baumhögger, E.; Span, R.; Vrabec, J. Thermodynamic Properties of Dodecamethylpentasiloxane, Tetradecamethylhexasiloxane, and Decamethylcyclopentasiloxane. Ind.Eng. Chem. Res. 2019, 58, 9617−9635.
(39) Colonna, P.; Nannan, N. R.; Guardone, A. Multiparameter Equations of State for Siloxanes: [(CH3)3-Si-O1/2]2-[O-Si-(CH3)2]i =1, ...,3, and [O-Si-(CH3)2]6. FluidPhaseEquilib. 2008, 263, 115− 130.
(40) Huber, M. L.; Perkins, R. A. Thermal conductivity correlations for minor constituent fluids in natural gas: n-octane, n-nonane and ndecane. FluidPhaseEquilib. 2005, 227, 47−55.
(41) Thol, M.; Piazza, L.; Span, R. A New Functional Form for Equations of State for Some Weakly Associating Fluids. Int.J. Thermophys. 2014, 35, 783−811.
(42) Zhou, Y.; Wu, J.T.; Lemmon, E. W. Thermodynamic Properties of Dimethyl Carbonate. J.Phys.Chem. Ref.Data 2011, 40, 043106.
(43) Wu, J.T.; Zhou, Y.; Lemmon, E. W. An Equation of State for the Thermodynamic Properties of Dimethyl Ether. J.Phys.Chem. Ref.Data 2011, 40, 023104.
(44) Zhou, Y.; Lemmon, E. W.; Wu, J.T. Thermodynamic Properties of o-Xylene, m-Xylene, p-Xylene, and Ethylbenzene. J.Phys.Chem. Ref. Data 2012, 41, 023103.
(45) Mylona, S. K.; Antoniadis, K. D.; Assael, M. J.; Huber, M. L.; Perkins, R. A. Reference Correlations of the Thermal Conductivity of oXylene, m-Xylene, p-Xylene, and Ethylbenzene from the Triple Point to 700 K and Moderate Pressures. J.Phys.Chem. Ref.Data 2014, 43, 043104.
(46) Bücker, D.; Wagner, W. A reference equation of state for the thermodynamic properties of ethane for temperatures from the melting line to 675 K and pressures up to 900 MPa. J.Phys.Chem. Ref.Data 2006, 35, 205−266. (101) Friend, D. G.; Ingham, H.; Ely, J.F. Thermophysical Properties of Ethane. J.Phys.Chem. Ref.Data 1991, 20, 275−347.
(47) Schroeder, J. A.; Penoncello, S. G.; Schroeder, J. S. A Fundamental Equation of State for Ethanol. J.Phys.Chem. Ref.Data 2014, 43, 043102.
(48) Assael, M. J.; Sykioti, E. A.; Huber, M. L.; Perkins, R. A. Reference Correlation of the Thermal

Conductivity of Ethanol from the Triple Point to 600 K and up to 245 MPa. J.Phys.Chem. Ref.Data 2013, 42, 023102.
(49) Smukula, J.; Span, R.; Wagner, W. A New Equation of State for Ethylene Covering the Fluid Region for Temperatures from the Melting Line to 450 Kat Pressures up to 300 MPa. J.Phys.Chem. Ref.Data 2000, 29, 1052−1122.
(50) Assael, M. J.; Koutian, A.; Huber, M. L.; Perkins, R. A. Reference Correlations of the Thermal Conductivity of Ethene and Propene. J. Phys.Chem. Ref.Data 2016, 45, 033104.
(51) Thol, M.; Rutkai, G.; Koster, A.; Kortmann, M.; Span, R.; Vrabec, J.Fundamental equation of state for ethylene oxide based on a hybrid dataset. Chem. Eng.Sci. 2015, 121, 87−99
(52) de Reuck, K. M. International Thermodynamic Tables oftheFluid State-11 Fluorine; International Union of Pure and Applied Chemistry, Pergamon Press: Oxford, UK, 1990.
(53) Thol, M.; Dubberke, F. H.; Baumhögger, E.; Span, R.; Vrabec, J. Speed of Sound Measurements and aFundamental Equation of State for Hydrogen Chloride. J.Chem. Eng.Data 2018, 63, 2533−2547.
(54) Ortiz-Vega, D. O.; Hall, K. R.; Holste, J.C.; Arp, V. D.; Harvey, A. H.; Lemmon, E. W. Unpublished Helmholtz Equation ofStatefor Helium-4, 2015.
(55) Hands, B. A.; Arp, V. D. ACorrelation of Thermal Conductivity Data for Helium. Cryogenics 1981, 21, 697−703.
(56) Assael, M. J.; Bogdanou, I.; Mylona, S. K.; Huber, M. L.; Perkins, R. A.; Vesovic, V. Reference Correlation of the Thermal Conductivity of n-Heptane from the Triple Point to 600 K and up to 250 MPa. J.Phys.Chem. Ref.Data 2013, 42, 023101.
(57) Assael, M. J.; Mylona, S. K.; Tsiglifisi, C. A.; Huber, M. L.; Perkins, R. A. Reference Correlation of the Thermal Conductivity of nHexane from the Triple Point to 600 K and up to 500 MPa. J.Phys. Chem. Ref.Data 2013, 42, 013106.
(58) Leachman, J.W.; Jacobsen, R. T.; Penoncello, S. G.; Lemmon, E. W. Fundamental Equations of State for Parahydrogen, Normal Hydrogen, and Orthohydrogen. J.Phys.Chem. Ref.Data 2009, 38, 721− 748.
(59) Perkins, R. A. Measurement and correlation of the thermal conductivity of isobutane from 114 to 600 K at pressures to 70 MPa. J. Chem. Eng.Data 2002, 47, 1272−1279.
(60) Thol, M.; Dubberke, F. H.; Baumhogger, E.; Vrabec, J.; Span, R. Speed of Sound Measurements and Fundamental Equations of State for Octamethyltrisiloxane and Decamethyltetrasiloxane. J.Chem. Eng.Data 2017, 62, 2633−2648.
(61) Setzmann, U.; Wagner, W. A New Equation of State and Tables of Thermodynamic Properties for Methane Covering the Range from the Melting Line to 625 K at Pressures up to 100 MPa. J.Phys.Chem. Ref.Data 1991, 20, 1061−1155.
(62) Friend, D. G.; Ely, J. F.; Ingham, H. Tables forthe Thermophysical Properties ofMethane, NIST Technical Note 1325, NIST: Gaithersburg, MD, 1989
(63) de Reuck, K. M.; Craven, R. J. B. Methanol, International Thermodynamic Tables oftheFluid State-12; IUPAC, Blackwell Scientific Publications: London, UK, 1993
(64) Sykioti, E. A.; Assael, M. J.; Huber, M. L.; Perkins, R. A. Reference Correlation of the Thermal Conductivity of Methanol from the Triple Point to 660 K and up to 245 MPa. J.Phys.Chem. Ref.Data 2013, 42, 043101.
(65) Huber, M. L.; Lemmon, E. W.; Kazakov, A.; Ott, L. S.; Bruno, T. J.Model for the Thermodynamic Properties of aBiodiesel Fuel. Energy Fuels 2009, 23, 3790−3797.
(66) Perkins, R. A.; Huber, M. L. Measurement and Correlation of the Thermal Conductivities of Biodiesel Constituent Fluids: Methyl Oleate and Methyl Linoleate. Energy Fuels 2011, 25, 2383−2388.
(67) Thol, M.; Dubberke, F. H.; Rutkai, G.; Windmann, T.; Köster, A.; Span, R.; Vrabec, J. Fundamental Equation of State Correlation for Hexamethyldisiloxane Based on Experimental and Molecular Simulation Data. FluidPhaseEquilib. 2016, 418, 133−151
(68) Span, R.; Lemmon, E. W.; Jacobsen, R. T.; Wagner, W.; Yokozeki, A. A Reference Equation of State for the Thermodynamic Properties of Nitrogen for Temperatures from 63.151 to 1000 K and Pressures to 2200 MPa. J.Phys.Chem. Ref.Data 2000, 29, 1361−1433.
(69) Schmidt, R.; Wagner, W. A new form of the equation of state for pure substances and its application to oxygen. FluidPhaseEquilib. 1985, 19, 175−200.
(70) Marsh, K. N.; Perkins, R. A.; Ramires, M. L. V. Measurement and Correlation of the Thermal Conductivity of Propane. J.Chem. Eng. Data 2002, 47, 932−940.
(71) Vogel, E.; Herrmann, S. New Formulation for the Viscosity of Propane. J.Phys.Chem. Ref.Data 2016,

45, 043103.
(72) Jacobsen, R. T.; Penoncello, S. G.; Lemmon, E. W. A Fundamental Equation for Trichlorofluoromethane (R-11). Fluid PhaseEquilib. 1992, 80, 45−56.
(73) McLinden, M. O.; Klein, S. A.; Perkins, R. A. An extended corresponding states model for the thermal conductivity of refrigerants and refrigerant mixtures. Int.J.Refrig. 2000, 23, 43−63.
(74) Marx, V.; Pruß, A.; Wagner, W. NeueZustandsgleichungen fuerR 12,R22,R11undR113.Beschreibung desthermodynamishchen Zustandsverhaltens beiTemperaturen bis525KundDruecken bis200 MPa; Series 19 (Waermetechnik/Kaeltetechnik); VDI Verlag: Duesseldorf, 1992; No. 57
(75) Platzer, B.; Polt, A.; Maurer, G. Thermophysical Properties of Refrigerants; Springer-Verlag: Berlin, 1990.
(76) Lemmon, E. W.; Span, R. Thermodynamic Properties of R227ea, R-365mfc, R-115, and R-13I1. J.Chem. Eng.Data 2015, 60, 3745−3758.
(77) Akasaka, R.; Fukushima, M.; Lemmon, E. W., A Helmholtz Energy Equation of State for cis-1-chloro-2,3,3,3-Tetrafluoropropene (R-1224yd(Z)). European Conference on Thermophysical Properties, Graz, Austria, September 3−8, 2017.
(78) Younglove, B. A.; McLinden, M. O. An International Standard Equation of State for the Thermodynamic Properties of Refrigerant 123 (2,2-Dichloro-1,1,1-Trifluoroethane). J.Phys.Chem. Ref.Data 1994, 23, 731−779.
(79) Laesecke, A.; Perkins, R. A.; Howley, J. B. An improved correlation for the thermal conductivity of HCFC123 (2,2-dichloro1,1,1-trifluoroethane). Int.J.Refrig. 1996, 19, 231−238.
(80) Mondejar, M. E.; McLinden, M. O.; Lemmon, E. W. Thermodynamic Properties of trans-1-Chloro-3,3,3-trifluoropropene (R1233zd(E)): Vapor Pressure, (p, rho, T) Behavior, and Speed of Sound Measurements, and Equation of State. J.Chem. Eng.Data 2015, 60, 2477−2489
(81) Perkins, R. A.; Huber, M. L.; Assael, M. J. Measurement and Correlation of the Thermal Conductivity of trans-1-Chloro-3,3,3trifluoropropene (R1233zd(E)). J.Chem. Eng.Data 2017, 62, 2659− 2665.
(82) Richter, M.; McLinden, M. O.; Lemmon, E. W. Thermodynamic Properties of 2,3,3,3-Tetrafluoroprop-1-ene (R1234yf): Vapor Pressure and p-rho-T Measurements and an Equation of State. J.Chem. Eng.Data 2011, 56, 3254−3264.
(83) Perkins, R. A.; Huber, M. L. Measurement and Correlation of the Thermal Conductivity of 2,3,3,3-Tetrafluoroprop-1-ene (R1234yf) and trans-1,3,3,3-Tetrafluoropropene (R1234ze(E)). J.Chem. Eng. Data 2011, 56, 4868−4874.
(84) Thol, M.; Lemmon, E. W. Equation of State for the Thermodynamic Properties of trans-1,3,3,3-Tetrafluoropropene R1234ze(E). Int.J.Thermophys. 2016, 37, 28.
(85) de Vries, B.; Tillner-Roth, R.; Baehr, H. D. Thermodynamic Properties of HCFC 124, 19th International Congress of Refrigeration. International Institute of Refrigeration: The Hague, The Netherlands, IVa:582−589, 1995.
(86) Lemmon, E. W.; Jacobsen, R. T. A New Functional Form and New Fitting Techniques for Equations of State with Application to Pentafluoroethane (HFC-125). J.Phys.Chem. Ref.Data 2005, 34, 69− 108.
(87) Perkins, R. A.; Huber, M. L. Measurement and correlation of the thermal conductivity of pentafluoroethane (R125) from 190 to 512 Kat pressures to 70 MPa. J.Chem. Eng.Data 2006, 51, 898−904.
(88) Magee, J.W.; Outcalt, S. L.; Ely, J.F. Molar heat capacity C-v, vapor pressure, and (p, rho, T) measurements from 92 to 350 K at pressures to 35 MPa and anew equation of state for chlorotrifluoromethane (R13). Int.J.Thermophys. 2000, 21, 1097−1121.
(89) McLinden, M. O.; Akasaka, R. Thermodynamic Properties of cis-1,1,1,4,4,4-Hexafluorobutene [R-1336mzz(Z)]: Vapor Pressure, (p, ρ, T) Behavior, and Speed of Sound Measurements and Equation of State. J.Chem. Eng.Data 2020, 65, 4201−4214.
(90) Tillner-Roth, R.; Baehr, H. D. An International Standard Formulation of the Thermodynamic Properties of 1,1,1,2-Tetrafluoroethane (HFC-134a) for Temperatures from 170 to 455 K at Pressures up to 70 MPa. J.Phys.Chem. Ref.Data 1994, 23, 657−729.
(91) Perkins, R. A.; Laesecke, A.; Howley, J.B.; Ramires, M. L. V.; Gurova, A. N.; Cusco, L. Experimental Thermal Conductivity Values for the IUPAC Round-Robin Sample of 1,1,1,2-Tetrafluoroethane (R134a). NISTInteragency/Internal Report (NISTIR) 2000, No. 6605.
(92) Lemmon, E. W.; Jacobsen, R. T. An international standard formulation for the thermodynamic properties of 1,1,1-trifluoroethane (HFC-143a) for temperatures from 161 to 450 K and pressures to 50 MPa. J.Phys.Chem. Ref.Data 2000, 29, 521−552.

(93) Thol, M.; Rutkai, G.; Koster, A.; Miroshnichenko, S.; Wagner, W.; Vrabec, J.; Span, R. Equation of State for Dichloroethane Based on a Hybrid Data Set. Mol.Phys. 2017, 115, 1166−1185.
(94) Outcalt, S. L.; McLinden, M. O. A modified Benedict-WebbRubin equation of state for the thermodynamic properties of R152a (1,1-difluoroethane). J.Phys.Chem. Ref.Data 1996, 25, 605−636.
(95) Krauss, R.; Weiss, V. C.; Edison, T. A.; Sengers, J.V.; Stephan, K. Transport properties of 1,1-difluoroethane (R152a). Int.J. Thermophys. 1996, 17, 731−757.
(96) Qi, H. Y.; Fang, D.; Gao, K. H.; Meng, X. Y.; Wu, J. T. Compressed Liquid Densities and Helmholtz Energy Equation of State for Fluoroethane (R161). Int.J.Thermophys. 2016, 37, 55.
(97) Tsolakidou, C. M.; Assael, M. J.; Huber, M. L.; Perkins, R. A. Correlations for the Viscosity and Thermal Conductivity of Ethyl Fluoride (R161). J.Phys.Chem. Ref.Data 2017, 46, 023103.
(98) Kamei, A.; Beyerlein, S. W.; Jacobsen, R. T. Application of Nonlinear Regression in the Development of a Wide Range Formulation for HCFC-22. Int.J.Thermophys. 1995, 16, 1155−1164.
(99) Penoncello, S. G.; Lemmon, E. W.; Jacobsen, R. T.; Shan, Z. J.A fundamental equation for trifluoromethane (R-23). J.Phys.Chem. Ref. Data 2003, 32, 1473−1499.
(100) Shan, Z.; Penoncello, S. G.; Jacobsen, R. T. A Generalized Model for Viscosity and Thermal Conductivity of Trifluoromethane (R-23). ASHRAE Trans. 2000, 106, 757−767.
(101) Rui, X. F.; Pan, J.; Wang, Y. G. An equation of state for the thermodynamic properties of 1,1,1,2,3,3-hexafluoropropane (R236ea). FluidPhaseEquilib. 2013, 341, 75−85.
(102) Pan, J.; Rui, X. F.; Zhao, X. D.; Qiu, L. M. An equation of state for the thermodynamic properties of 1,1,1,3,3,3-hexafluoropropane (HFC-236fa). FluidPhaseEquilib. 2012, 321, 10−16.
(103) Zhou, Y.; Lemmon, E. W. Equation of State for the Thermodynamic Properties of 1,1,2,2,3-Pentafluoropropane (R245ca). Int.J.Thermophys. 2016, 37, 27.
(104) Akasaka, R.; Zhou, Y.; Lemmon, E. W. AFundamental Equation of State for 1,1,1,3,3-Pentafluoropropane (R-245fa). J.Phys.Chem. Ref. Data 2015, 44, 013104.
(105) Perkins, R. A.; Huber, M. L.; Assael, M. J.Measurements of the Thermal Conductivity of 1,1,1,3,3-Pentafluoropropane (R245fa) and Correlations for the Viscosity and Thermal Conductivity Surfaces. J. Chem. Eng.Data 2016, 61, 3286−3294.
(106) Tillner-Roth, R.; Yokozeki, A. An international standard equation of state for difluoromethane (R-32) for temperatures from the triple point at 136.34 to 435 K and pressures up to 70 MPa. J.Phys. Chem. Ref.Data 1997, 26, 1273−1328.
(107) Guder, C.; Wagner, W. A Reference Equation of State for the Thermodynamic Properties of Sulfur Hexafluoride (SF6)for Temperatures from the Melting Line to 625 K and Pressures up to 150 MPa. J. Phys.Chem. Ref.Data 2009, 38, 33−94.
(108) Assael, M. J.; Koini, I. A.; Antoniadis, K. D.; Huber, M. L.; Abdulagatov, I.M.; Perkins, R. A. Reference Correlation of the Thermal Conductivity of Sulfur Hexafluoride from the Triple Point to 1000 K and up to 150 MPa. J.Phys.Chem. Ref.Data 2012, 41, 023104.
(109) Gao, K. H.; Wu, J. T.; Zhang, P. G.; Lemmon, E. W. A Helmholtz Energy Equation of State for Sulfur Dioxide. J.Chem. Eng. Data 2016, 61, 2859−2872.
(110) Assael, M. J.; Mylona, S. K.; Huber, M. L.; Perkins, R. A. Reference Correlation of the Thermal Conductivity of Toluene from the Triple Point to 1000 K and up to 1000 MPa. J.Phys.Chem. Ref.Data 2012, 41, 023101.
(111) Wagner, W.; Pruß, A. The IAPWS formulation 1995 for the thermodynamic properties of ordinary water substance for general and scientific use. J.Phys.Chem. Ref.Data 2002, 31, 387−535.
(112) International Association for the Properties of Water and Steam, IAPWS R15−11 (2011), Release ontheIAPWS Formulation 2011fortheThermal Conductvity ofOrdinary Water Substance.
