# Supplementary material for: Linking Thermal Conductivity to Equations of State Using the Residual Entropy Scaling Theory
Source: Ind Eng Chem Res. 2024 Oct 15;63(42):18160–75. doi: 10.1021/acs.iecr.4c02946 (PMC11503615; doi:10.1021/acs.iecr.4c02946)
Supplement: Supplementary file 3 — ie4c02946_si_003.pdf [file ie4c02946_si_003.pdf]

Supporting Information - Detailed plots and References

Linking Thermal Conductivity to Equations of State Using Residual Entropy Scaling Theory

Zhuo Li[1], Yuanyuan Duan[*1,2], Xiaoxian Yang[†3]

[1] *Key Laboratory for Thermal Science and Power Engineering of Ministry of Education, Beijing Key Laboratory for CO_2 Utilization and Reduction Technology, Tsinghua University, Beijing 100084,People's Republic of China*

[2] *Southwest United Graduate School，Kunming 650092, People's Republic of China*

[3] *Chemnitz University of Technology, Applied Thermodynamics, 09107 Chemnitz, Germany*

[*] Corresponding author. Yuanyuan Duan. Email address: yyduan@tsinghua.edu.cn. ORCID ID: https://orcid.org/0000-0002-4117-7545.

[†] Corresponding author. Xiaoxian Yang. Email address: xiaoxian.yang@mb.tu-chmenitz.de. ORCID ID: https://orcid.org/0000-0003-4655-3156.

1.Thermal conductivity as a function of residual entropy for each pure fluids

In this section, the dimensionless residual thermal conductivity λ^{+}_{res} as a function of dimensionless residual entropy s^{+} for experimental data of each pure fluids are plotted. The legends denote the sources of the data, showing the published year, the first three letters of the first author's family name, and the first three letters of the second author's family name ('xxx' if there is no second author). The full citations are given at the Reference section.

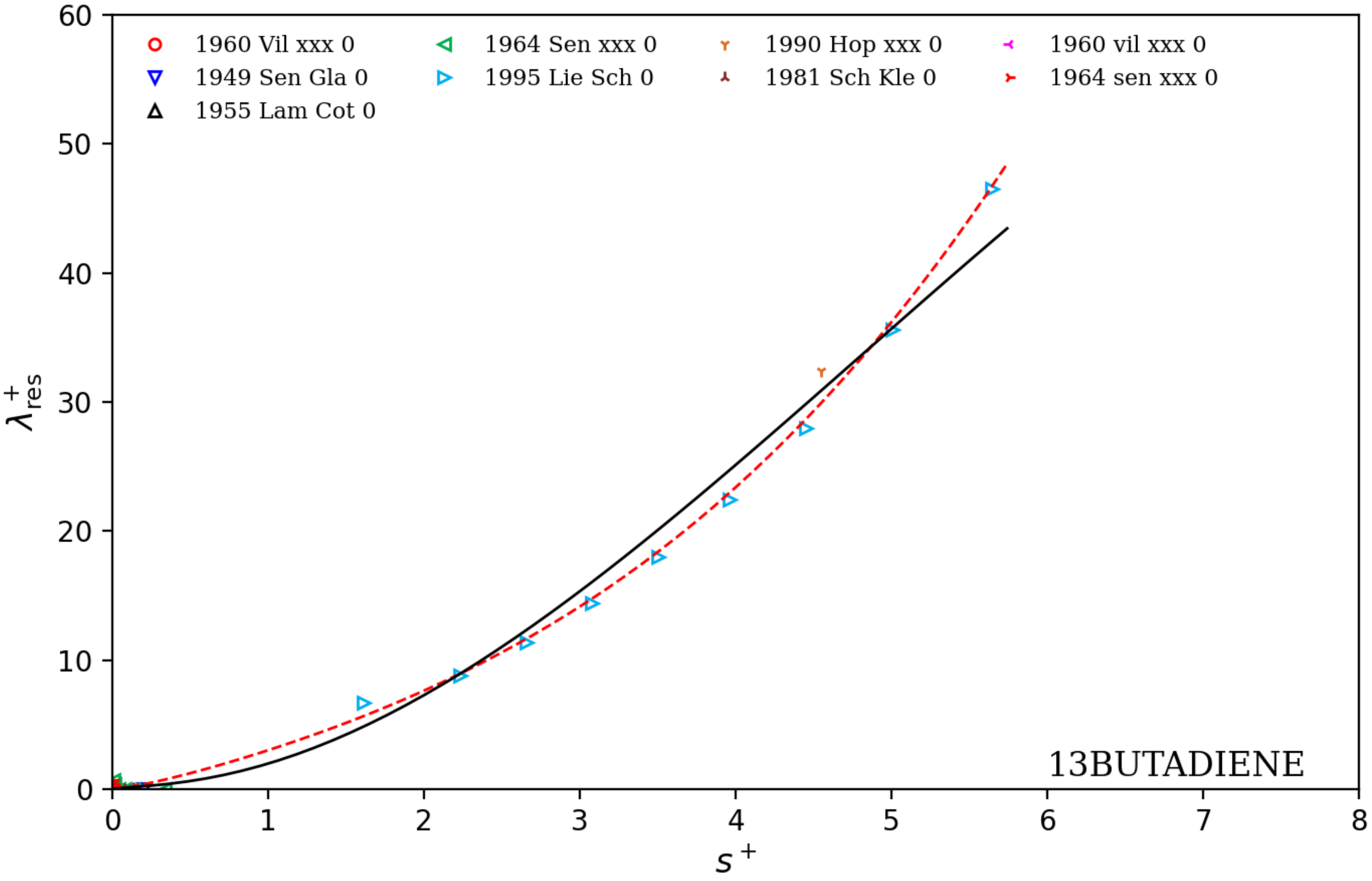

Figure DPR1. 13BUTADIENE

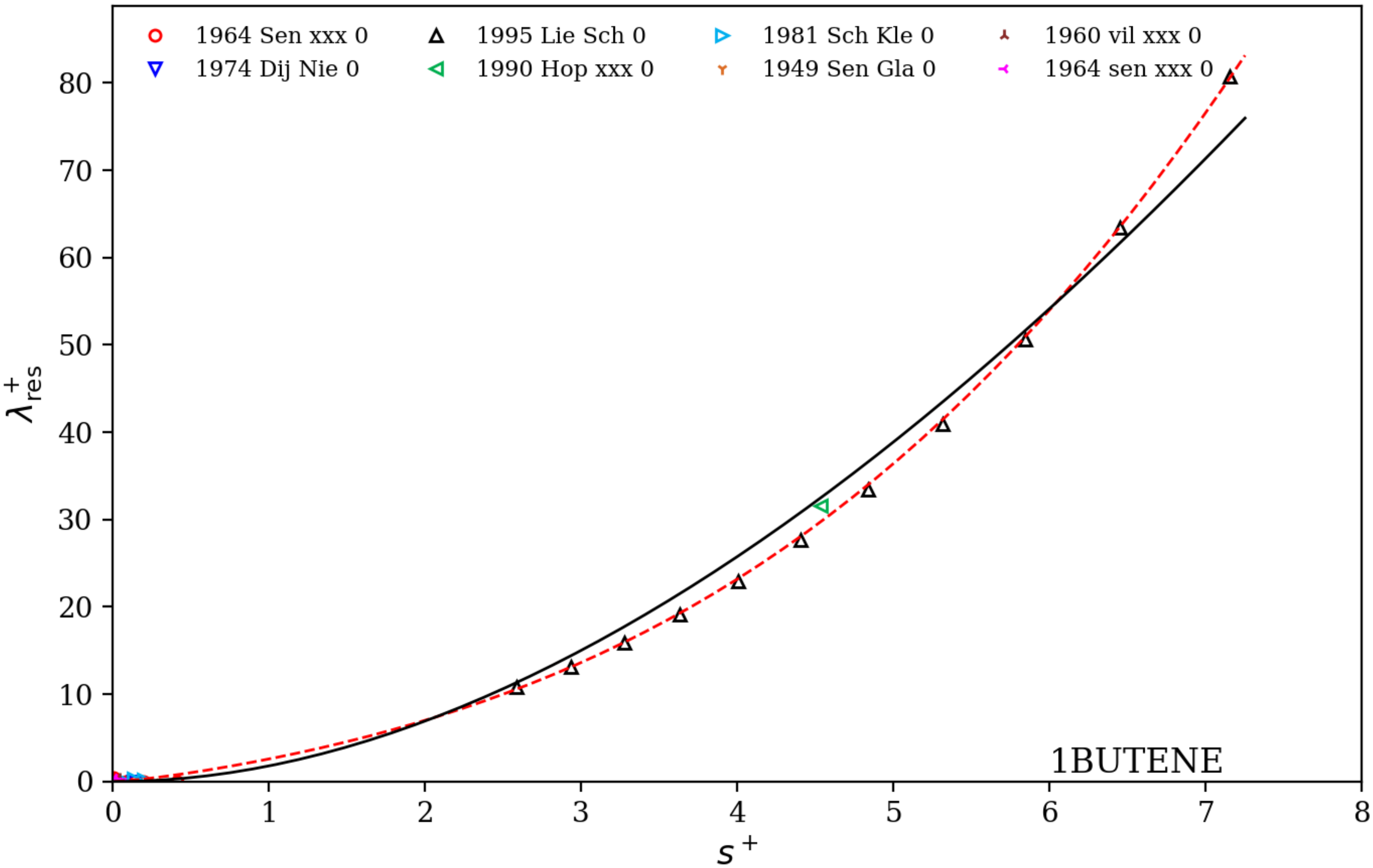

Figure DPR1. 1BUTENE

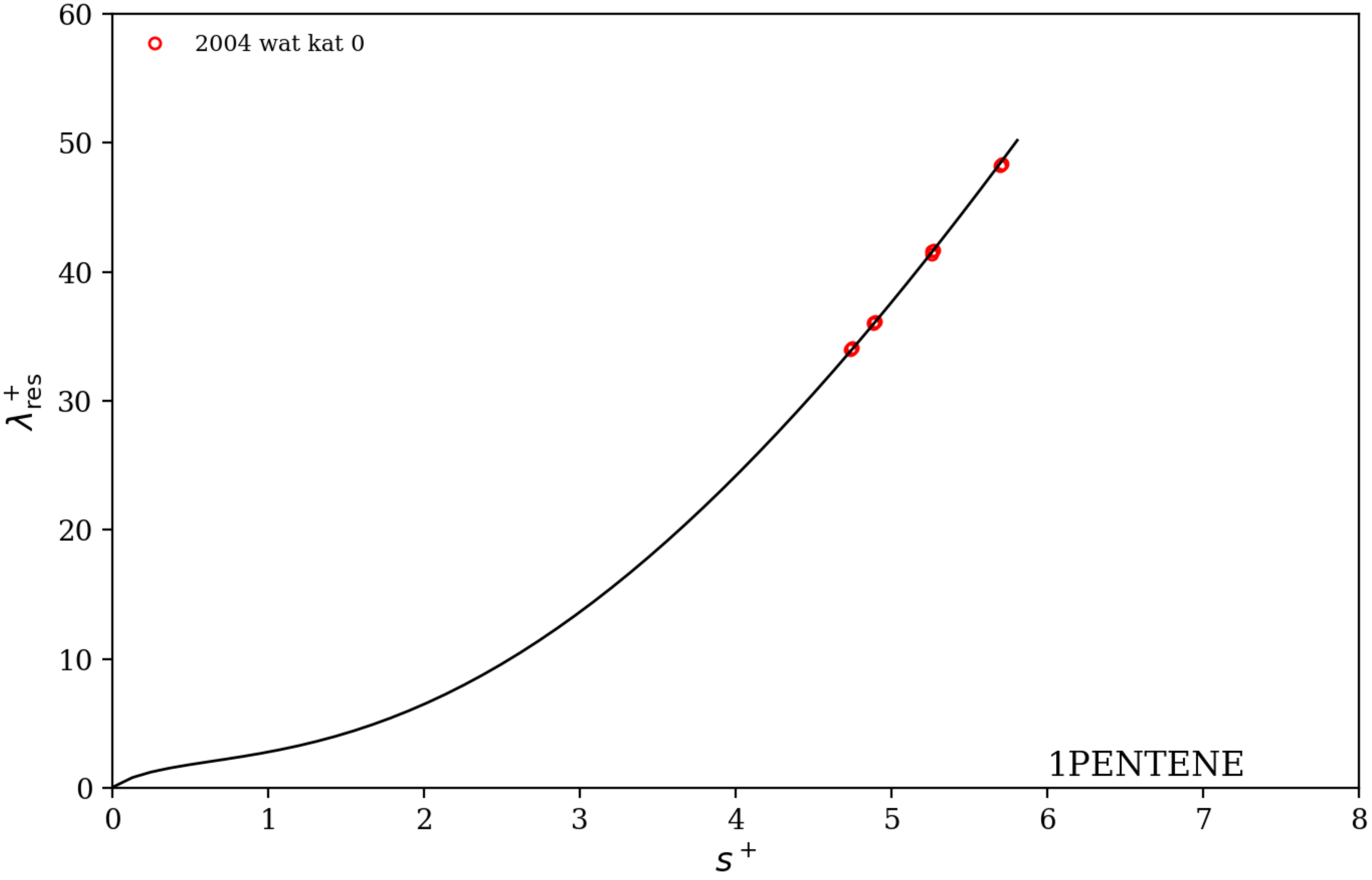

Figure DPR1. 1PENTENE

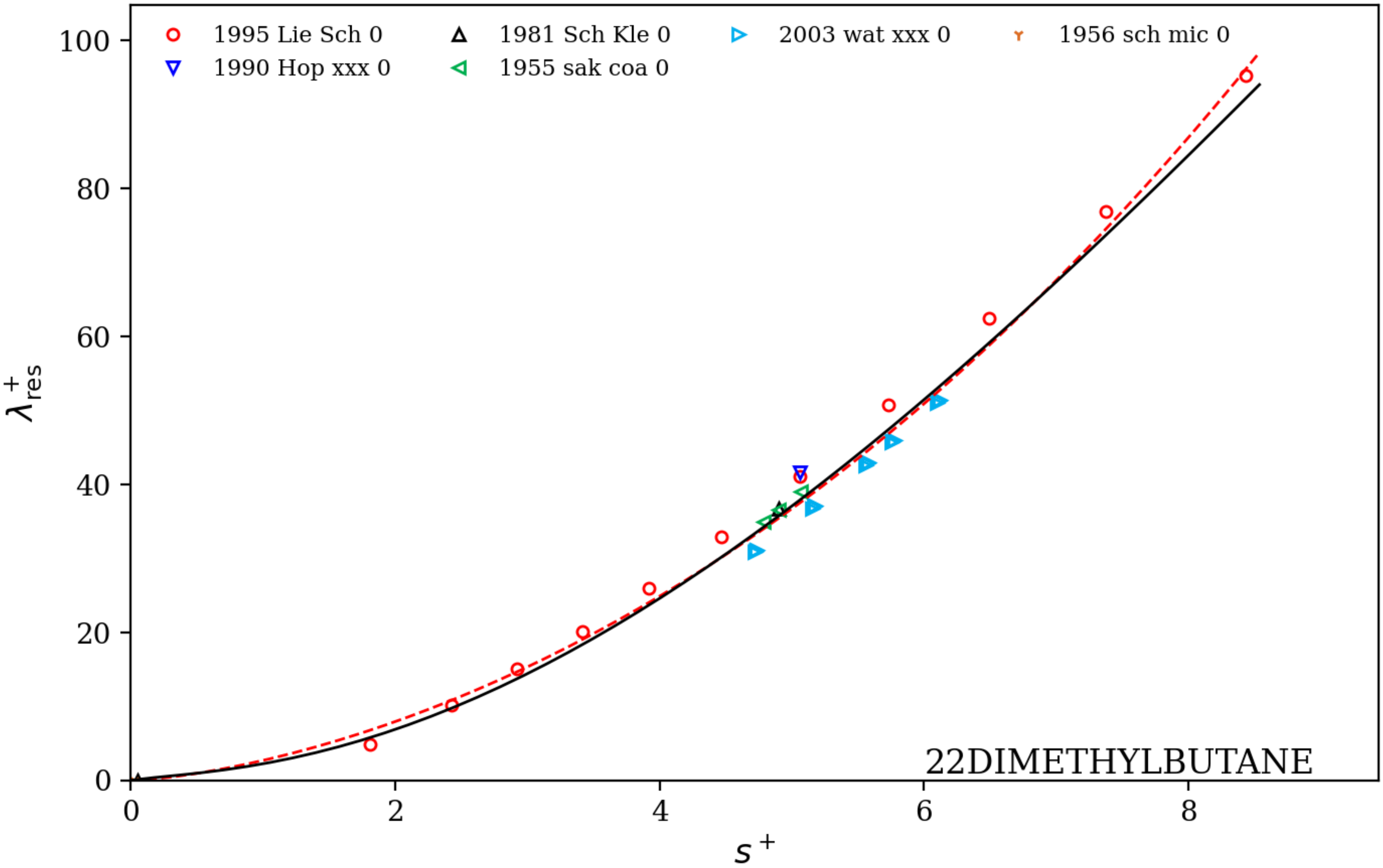

Figure DPR1. 22DIMETHYLBUTANE

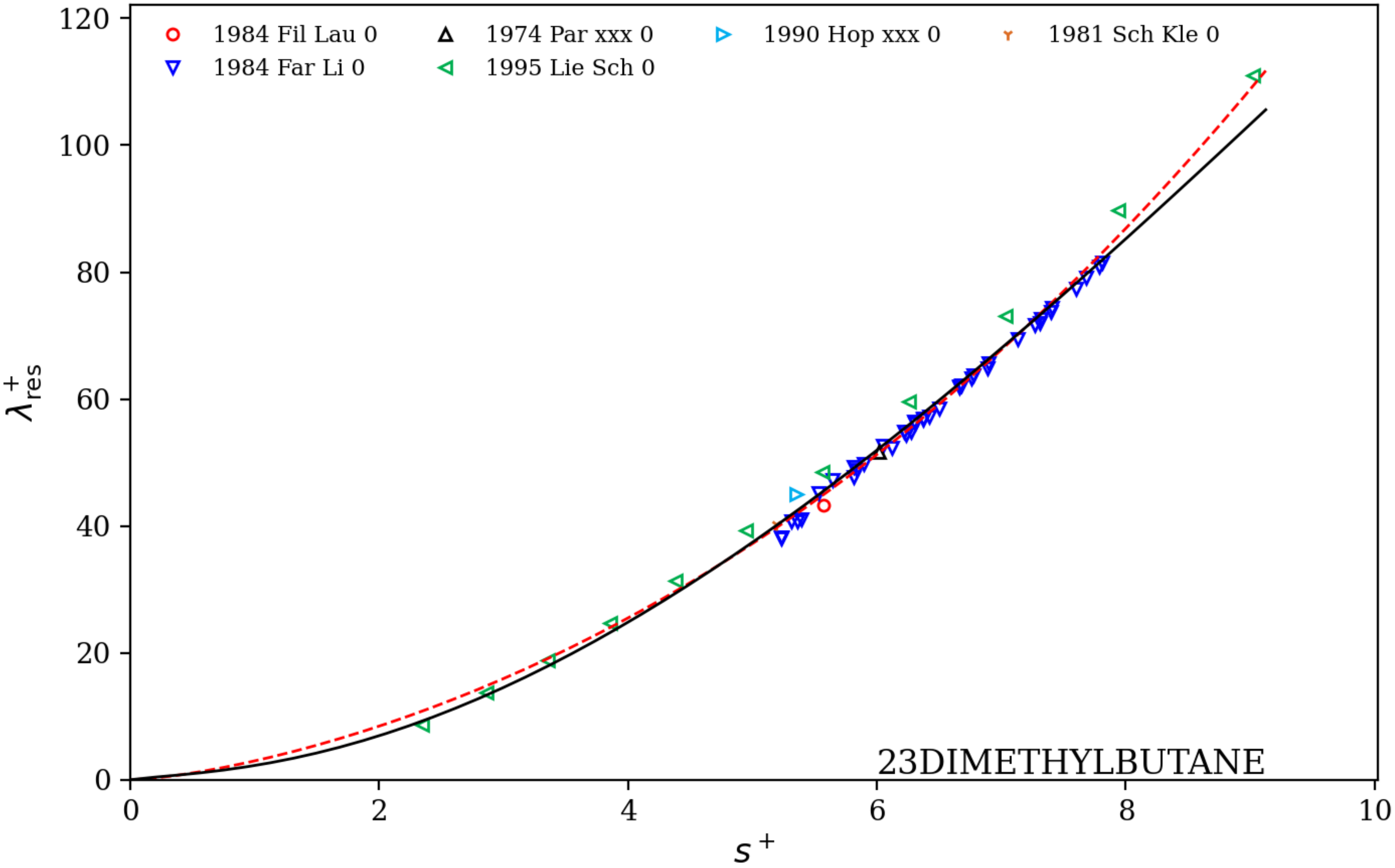

Figure DPR1. 23DIMETHYLBUTANE

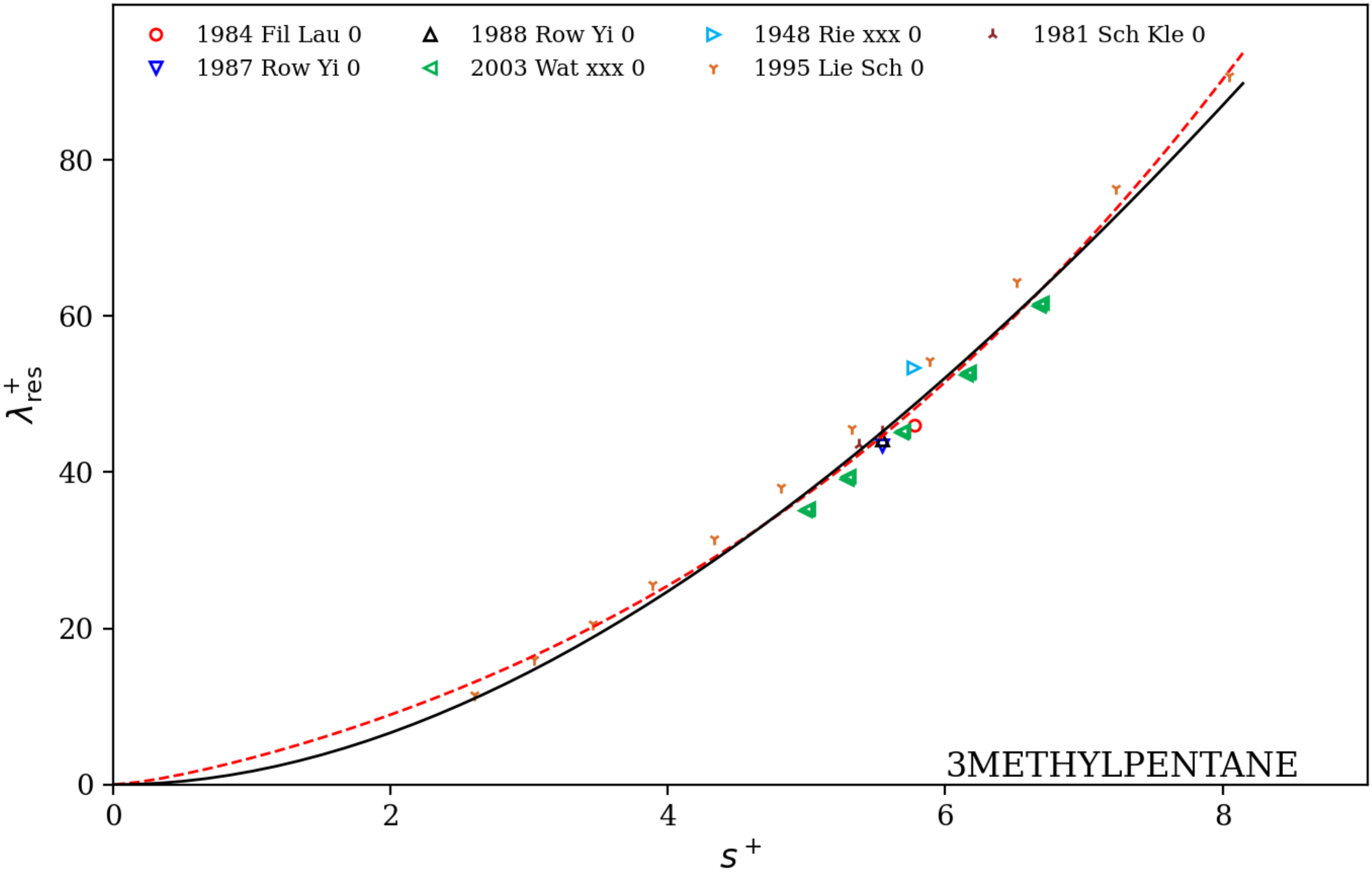

Figure DPR1. 3METHYLPENTANE

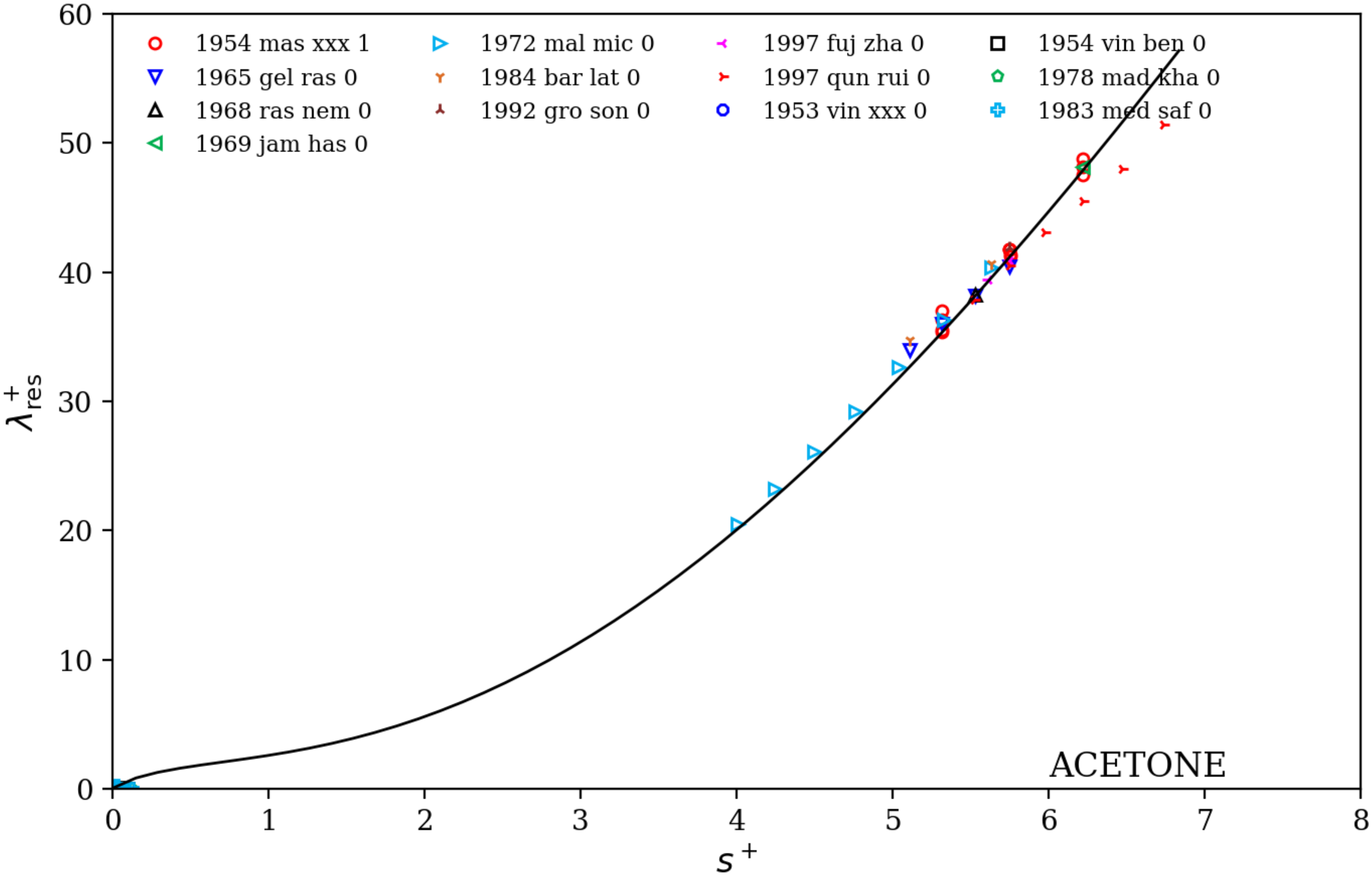

Figure DPR1. ACETONE

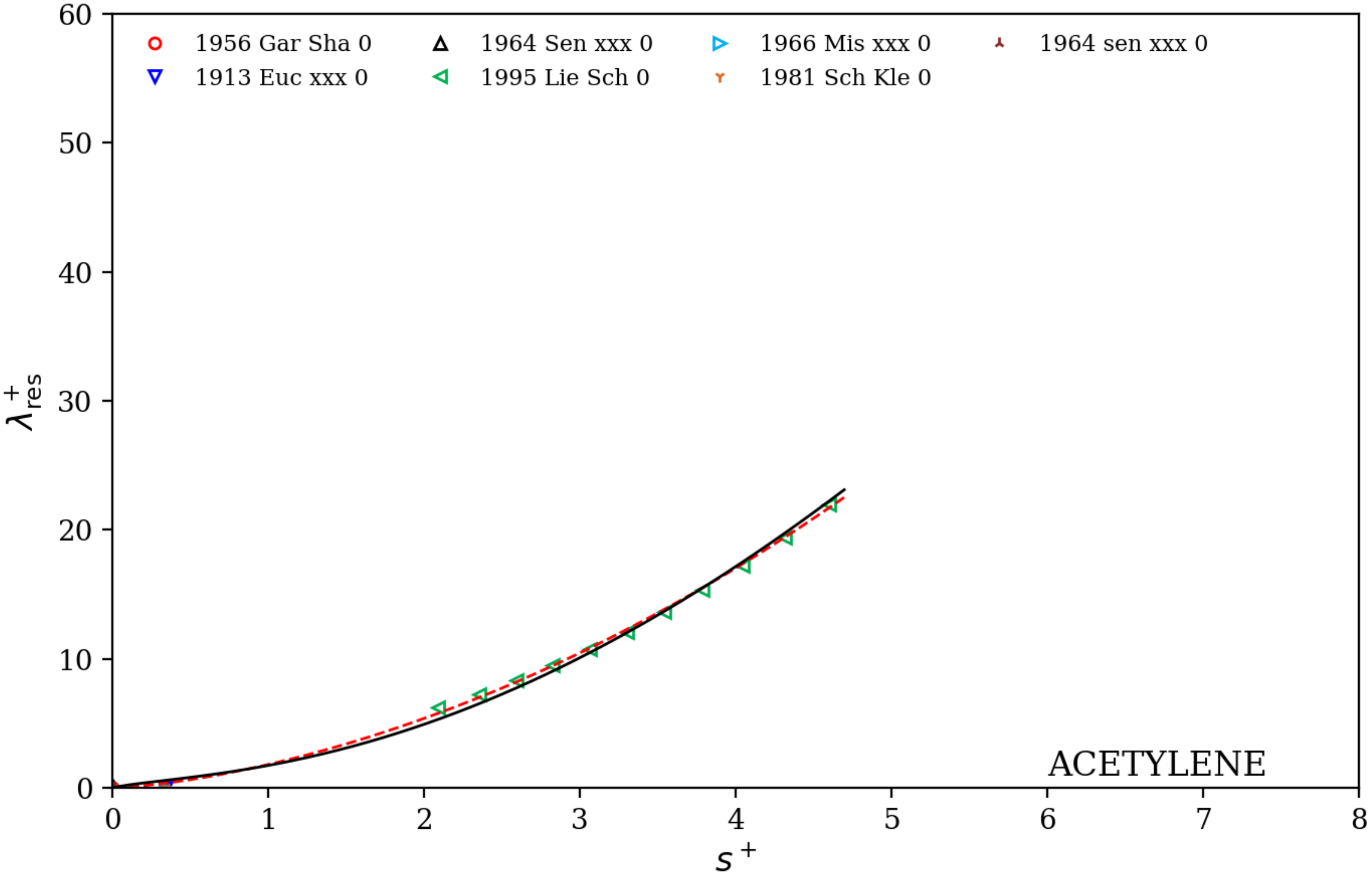

Figure DPR1. ACETYLENE

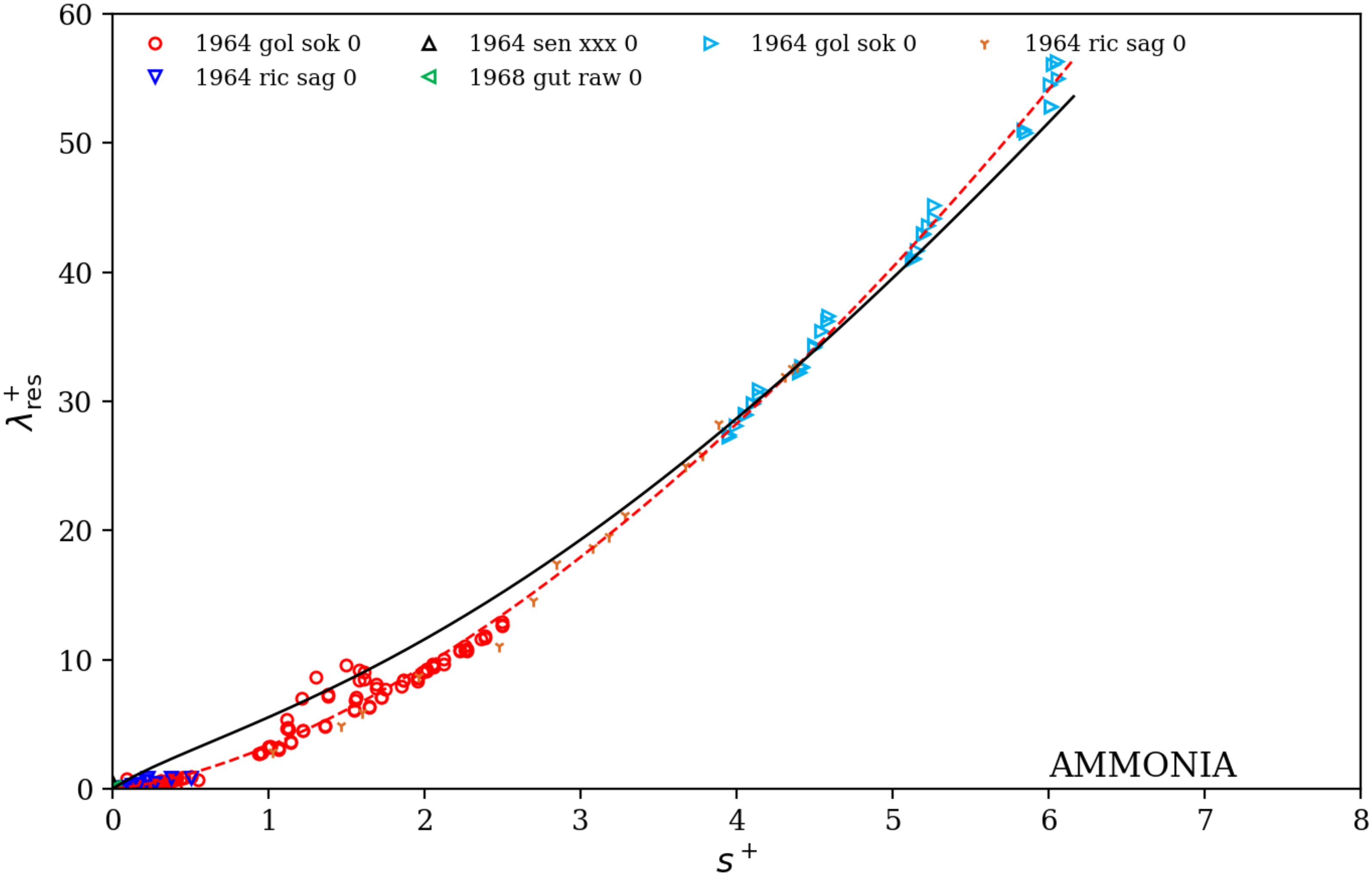

Figure DPR1. AMMONIA

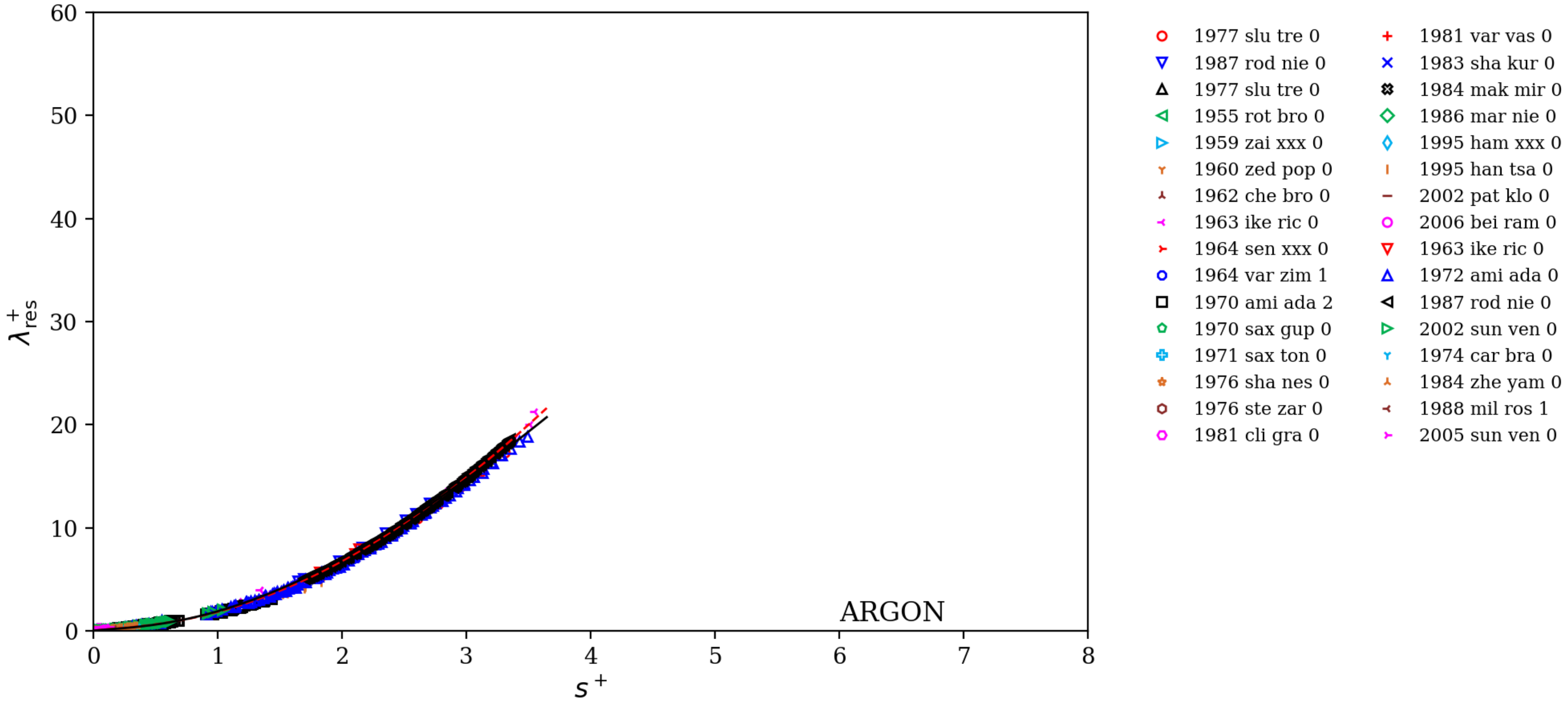

Figure DPR1. ARGON

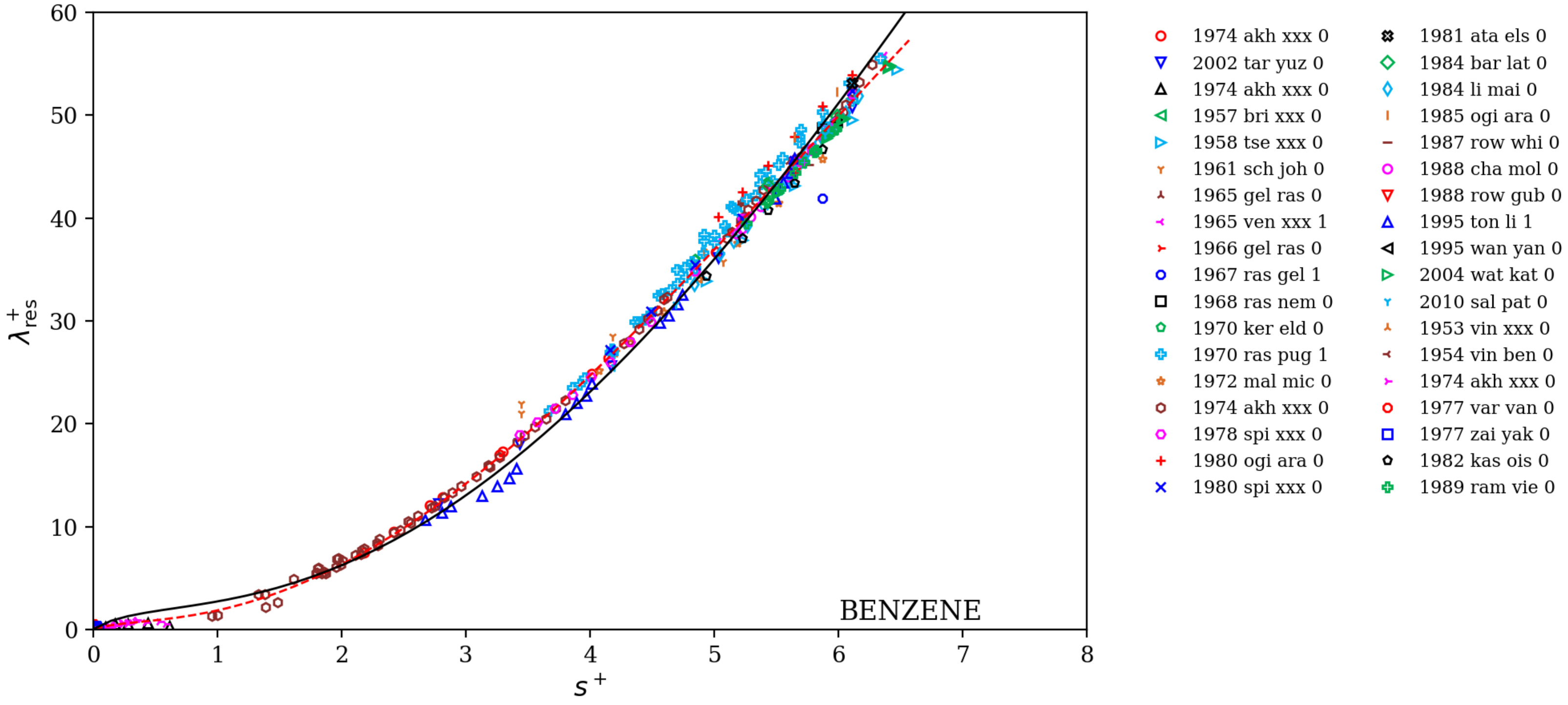

Figure DPR1. BENZENE

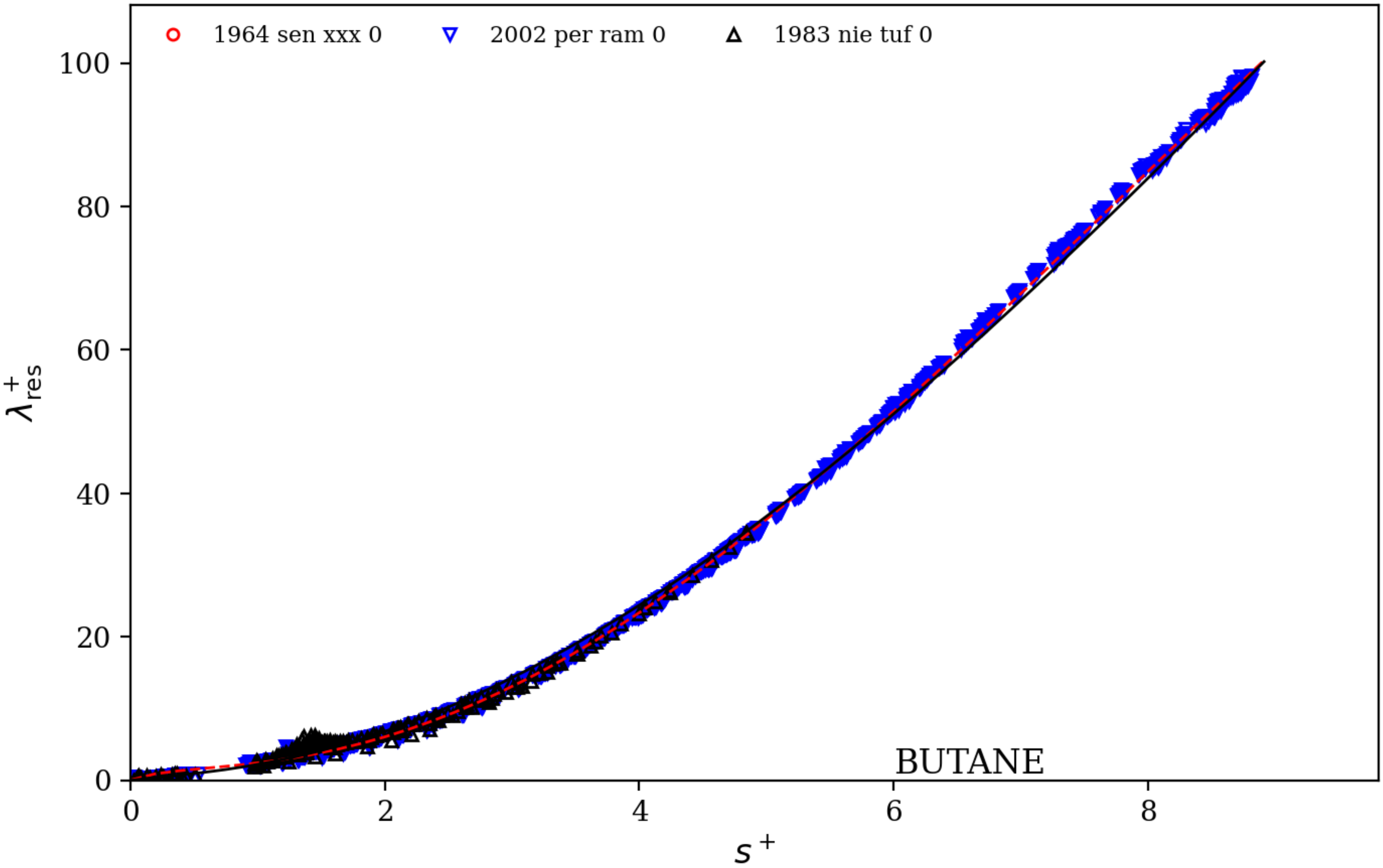

Figure DPR1. BUTANE

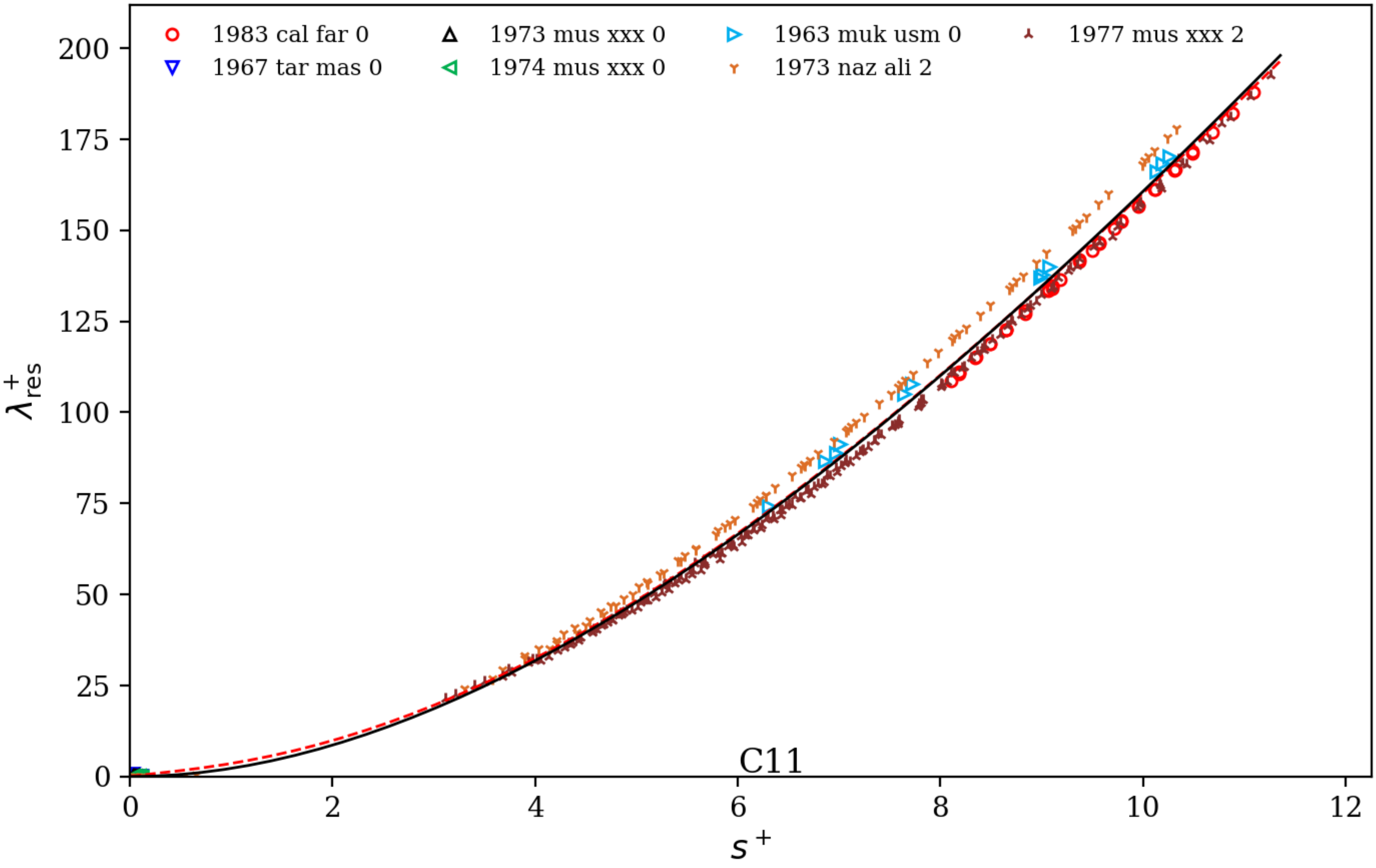

Figure DPR1. C11

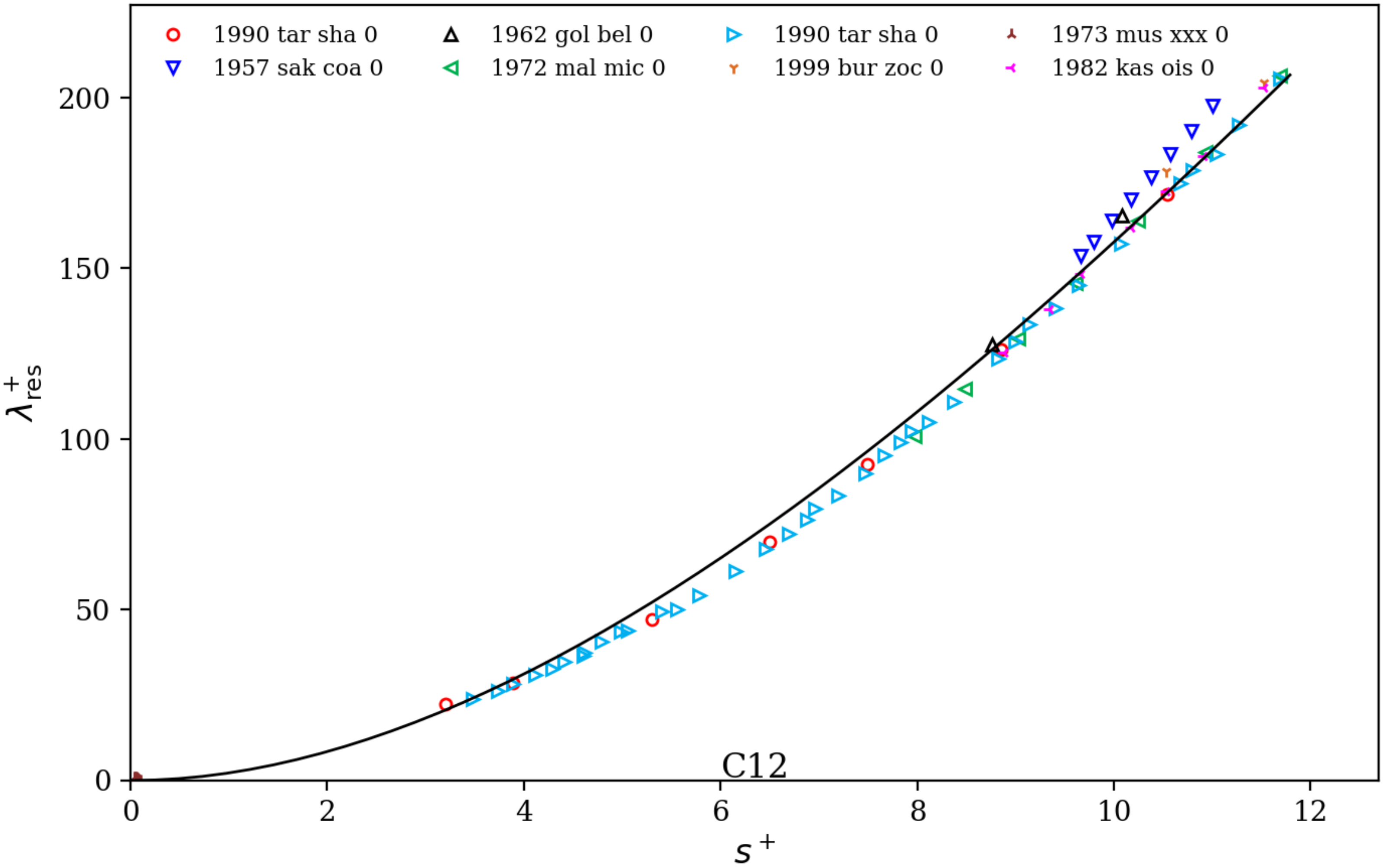

Figure DPR1. C12

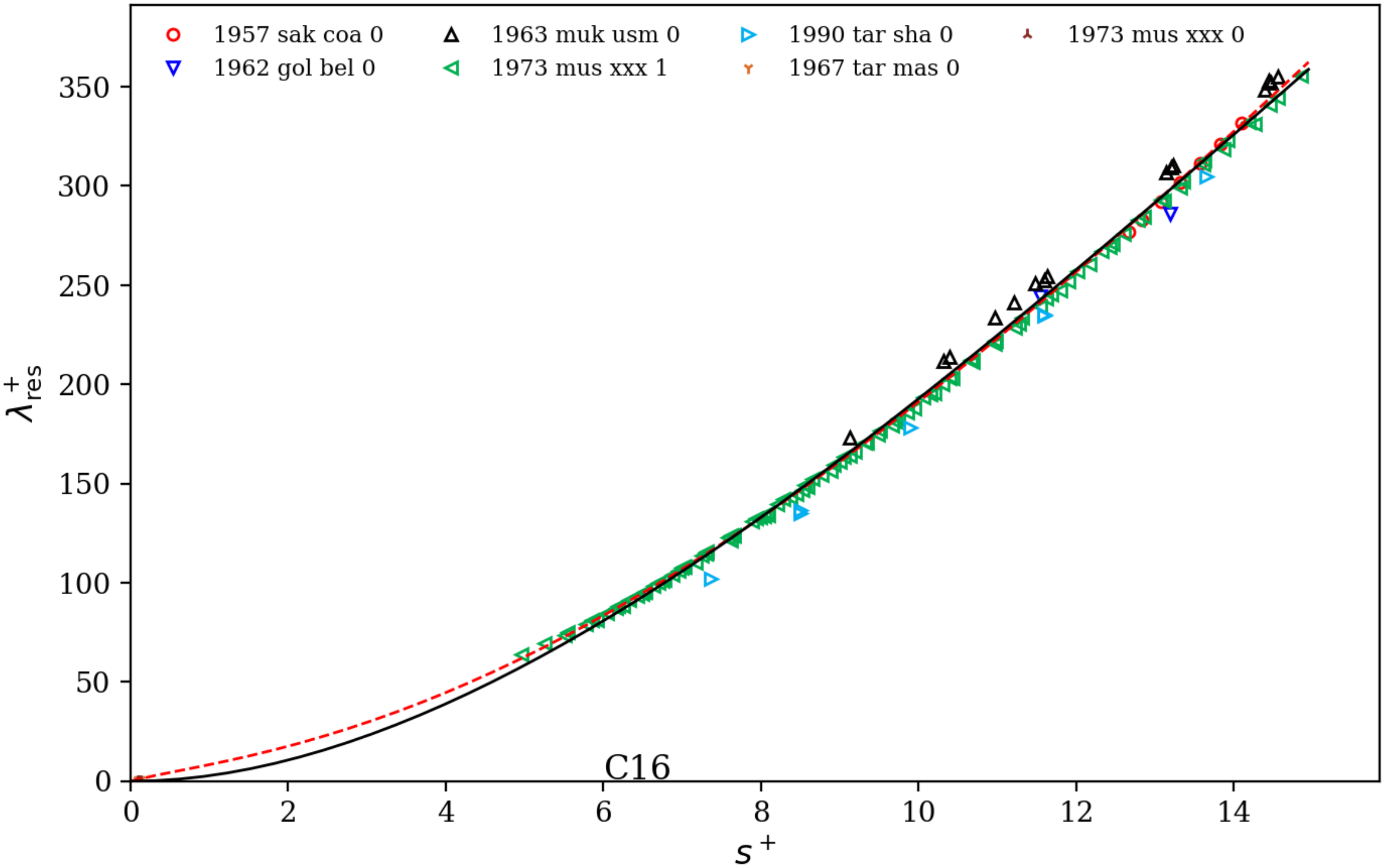

Figure DPR1. C16

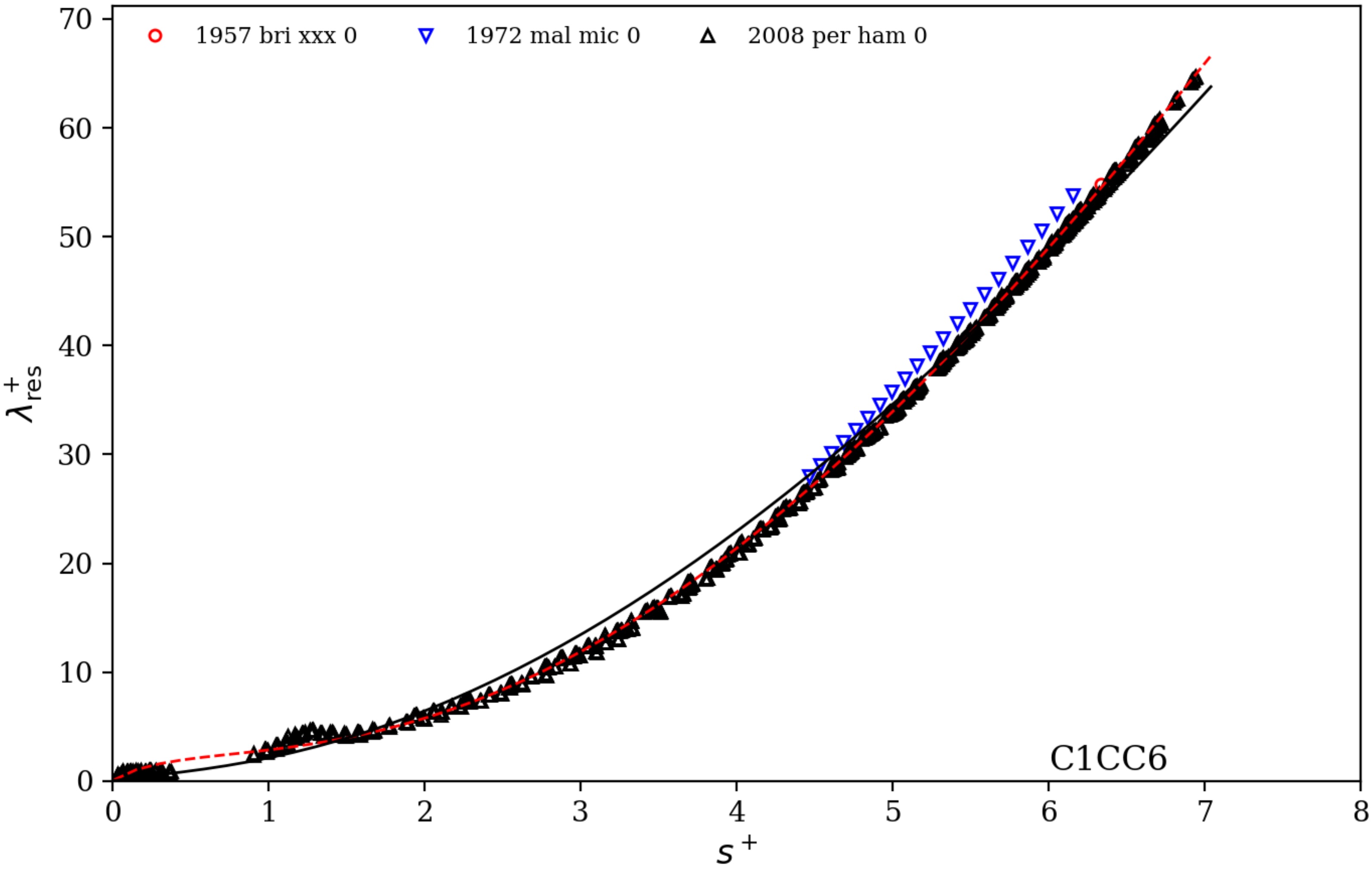

Figure DPR1. C1CC6

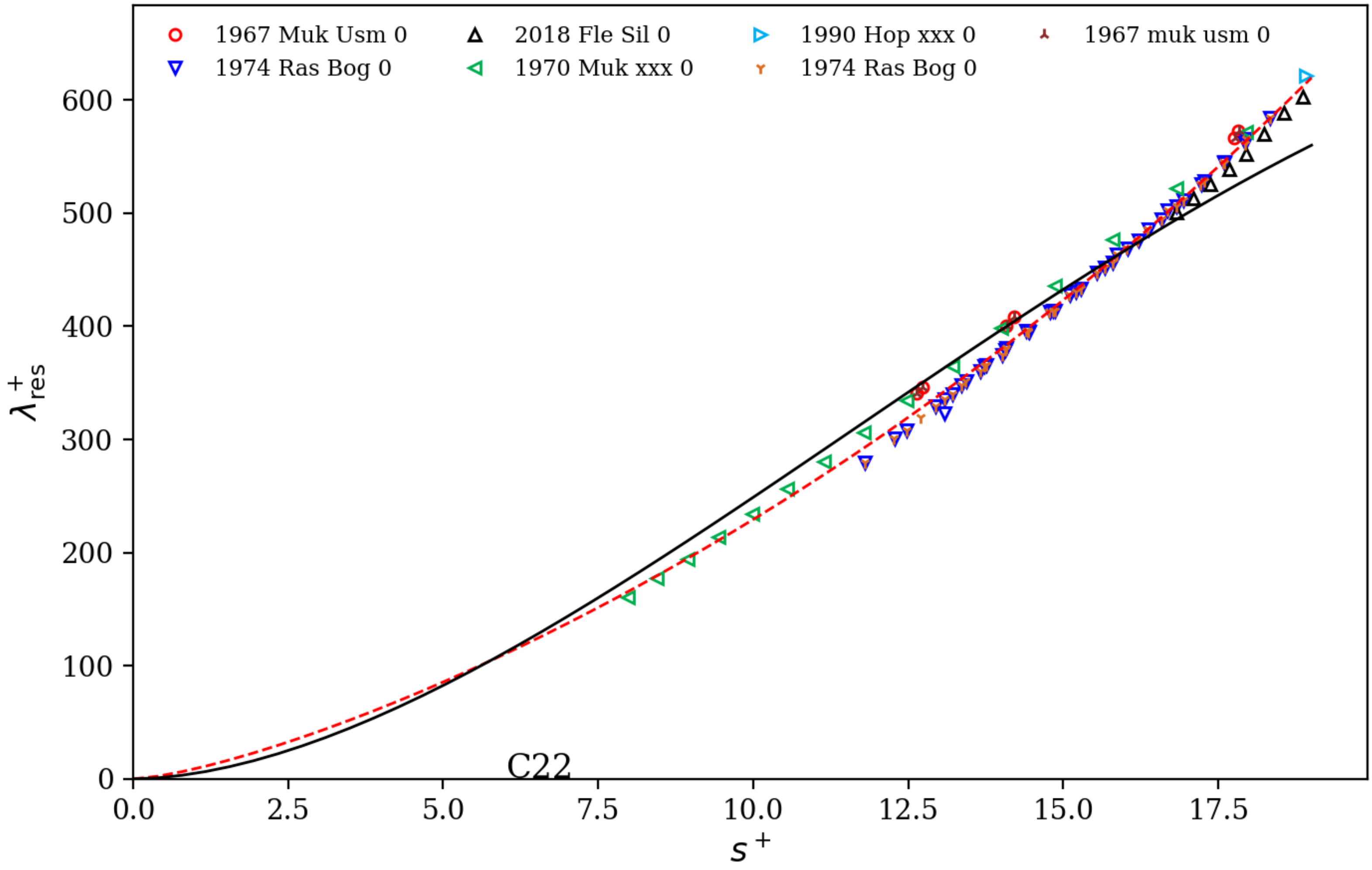

Figure DPR1. C22

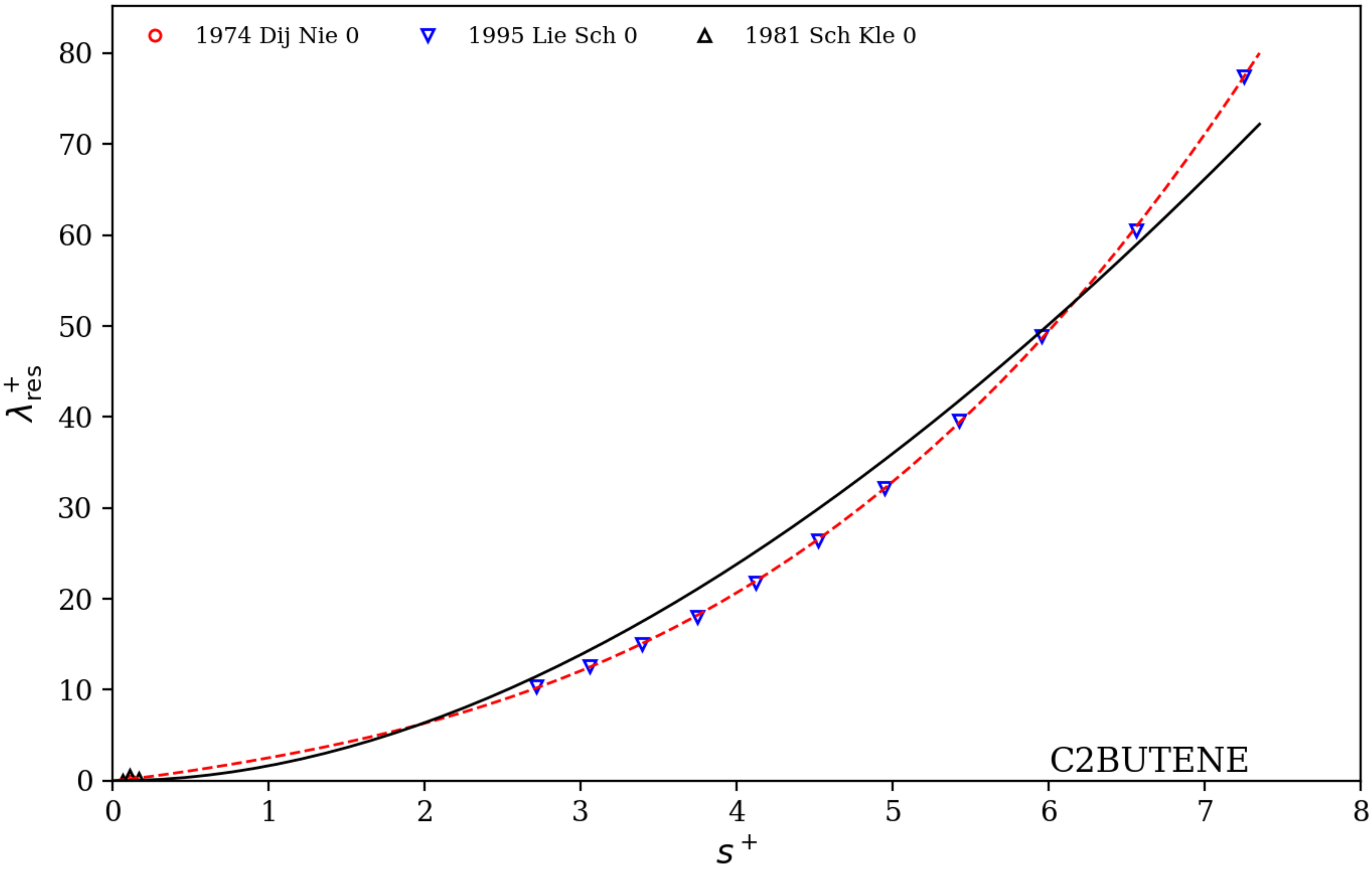

Figure DPR1. C2BUTENE

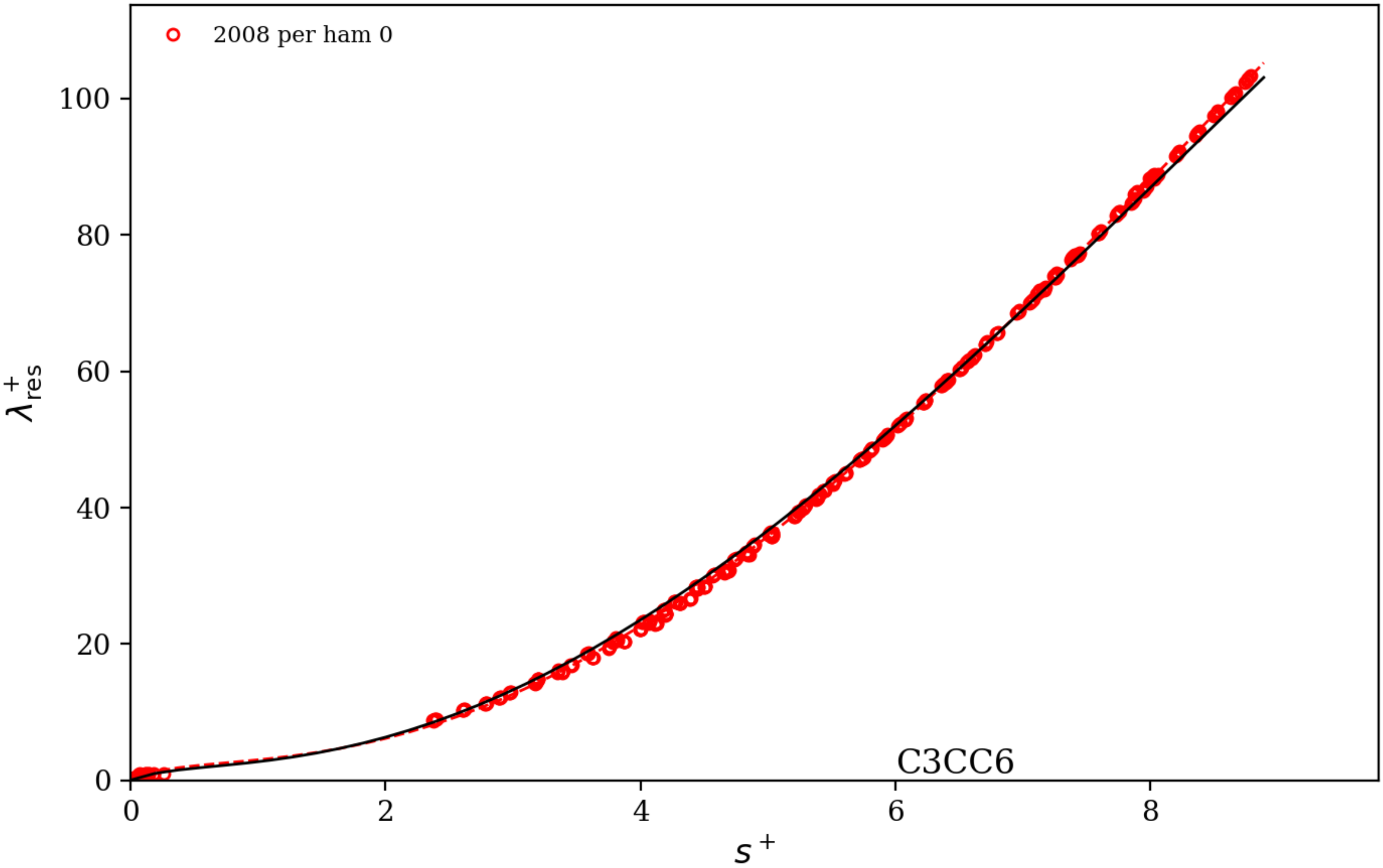

Figure DPR1. C3CC6

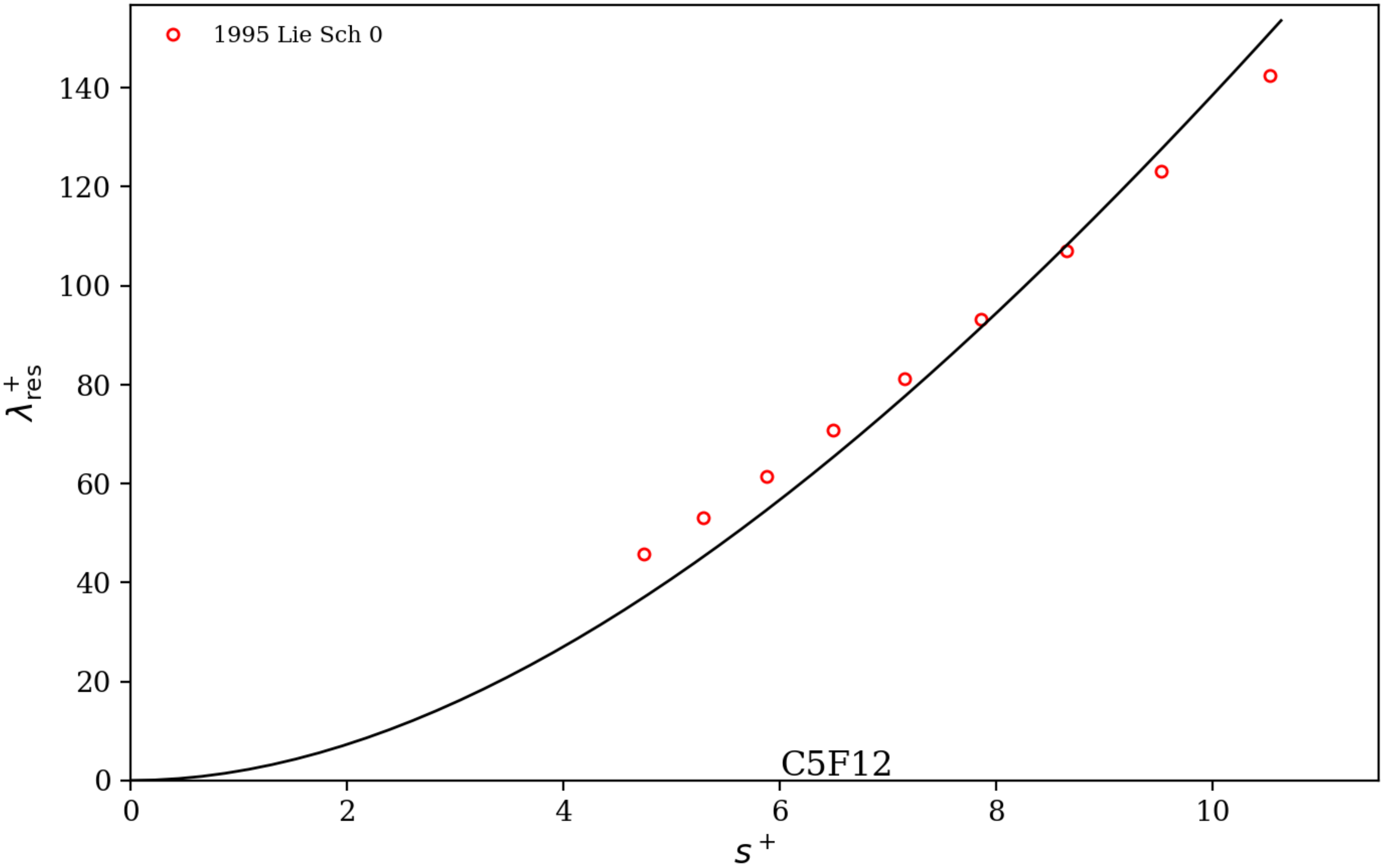

Figure DPR1. C5F12

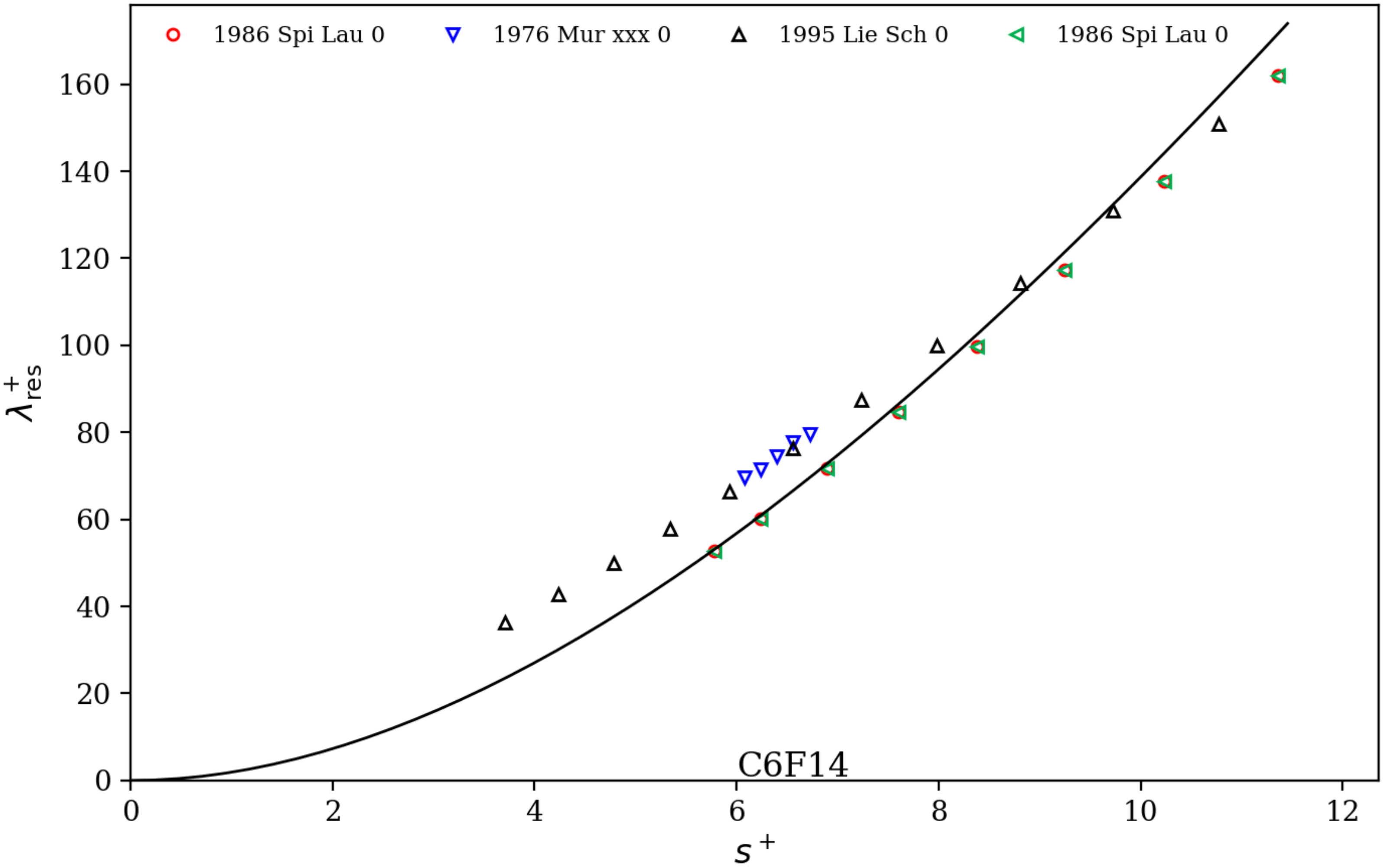

Figure DPR1. C6F14

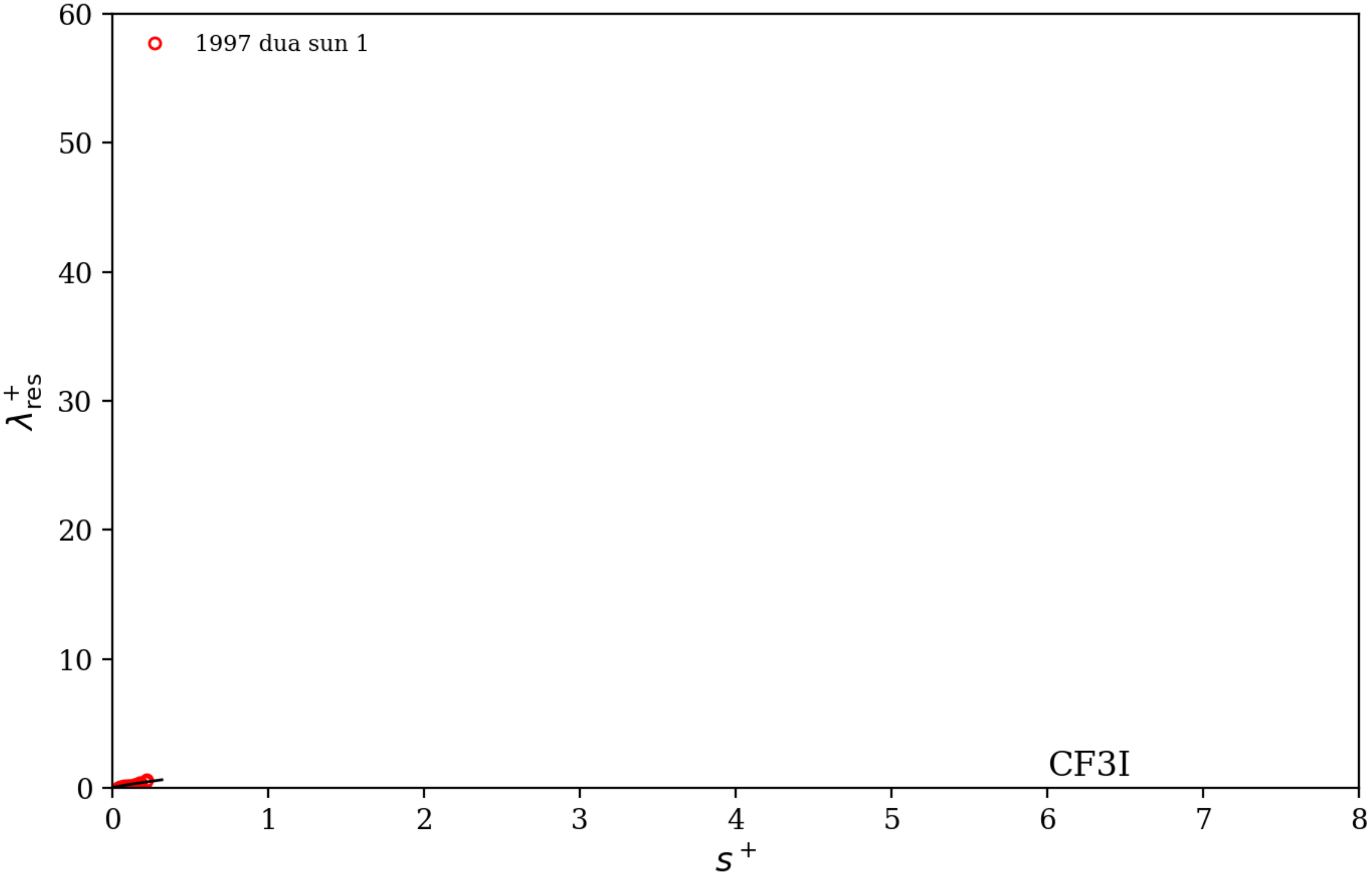

Figure DPR1. CF3I

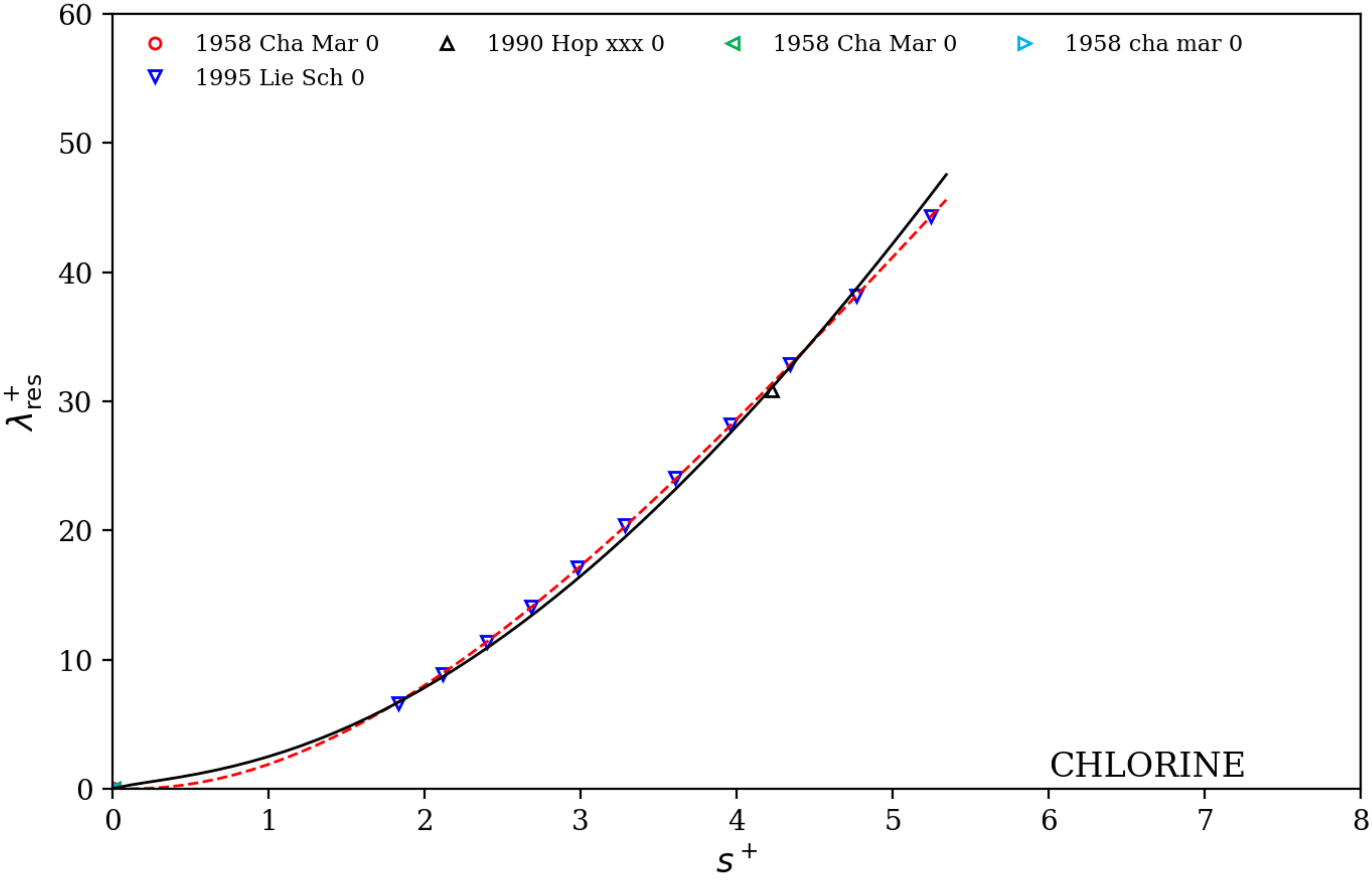

Figure DPR1. CHLORINE

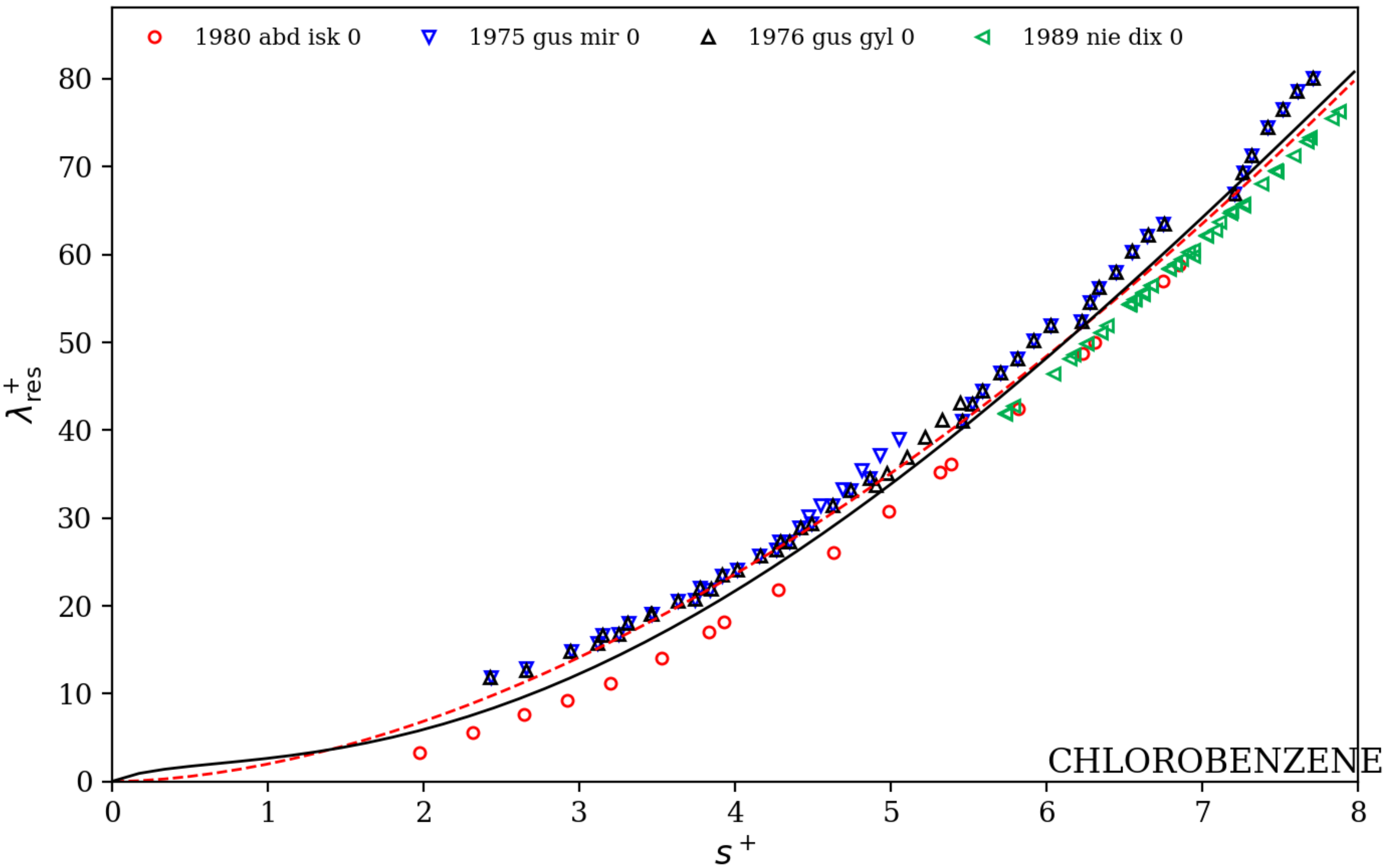

Figure DPR1. CHLOROBENZENE

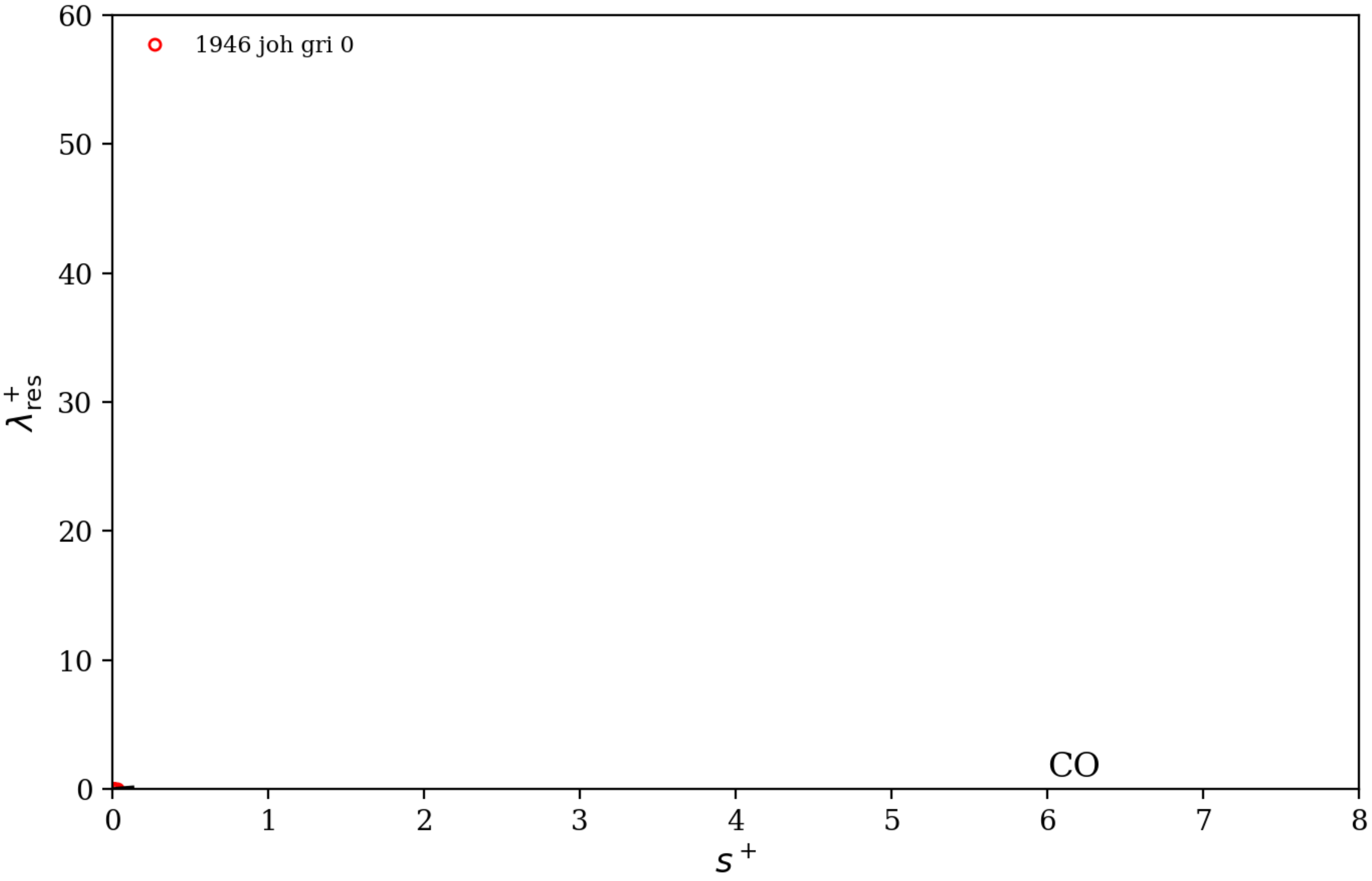

Figure DPR1. CO

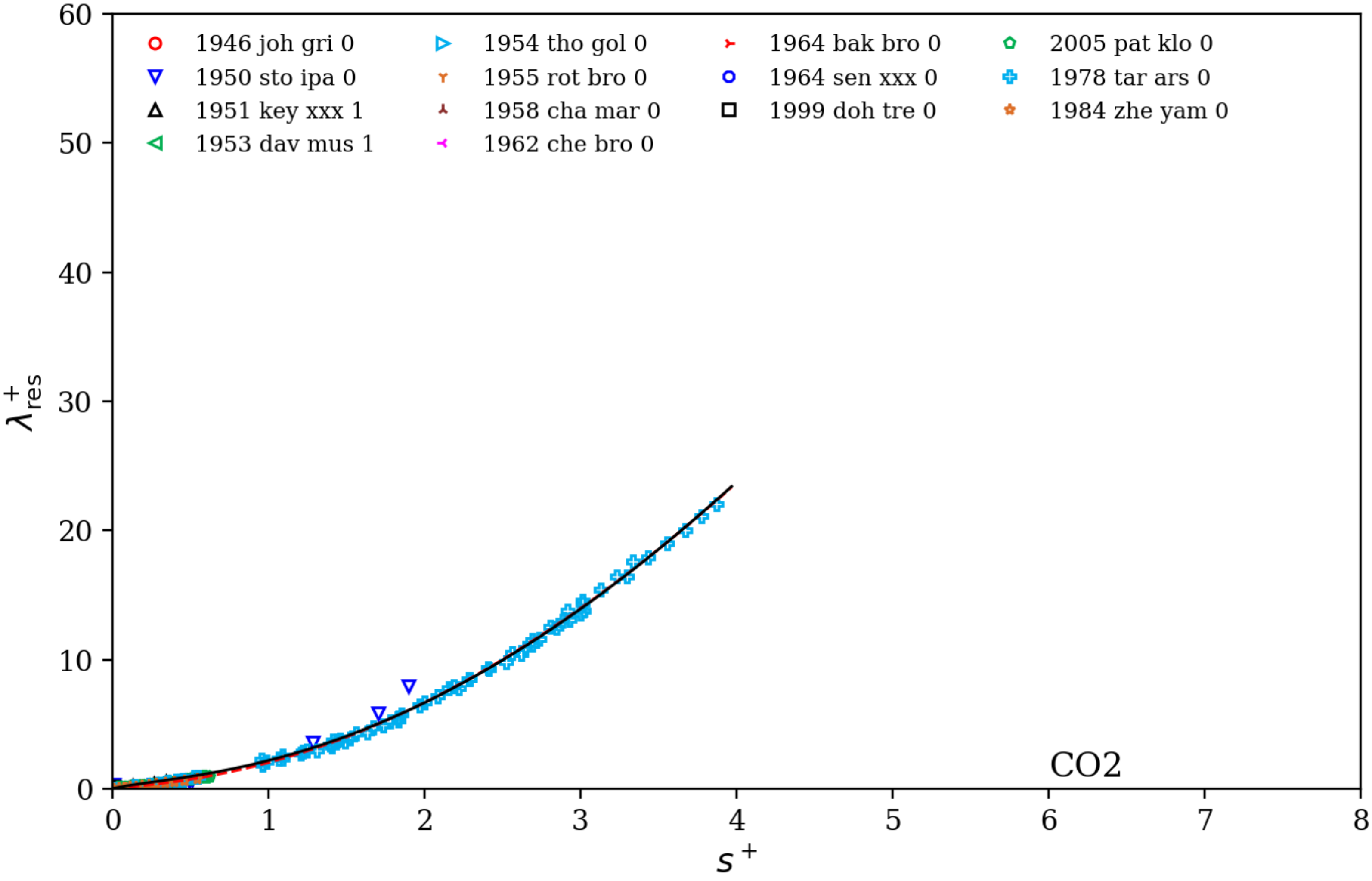

Figure DPR1. CO2

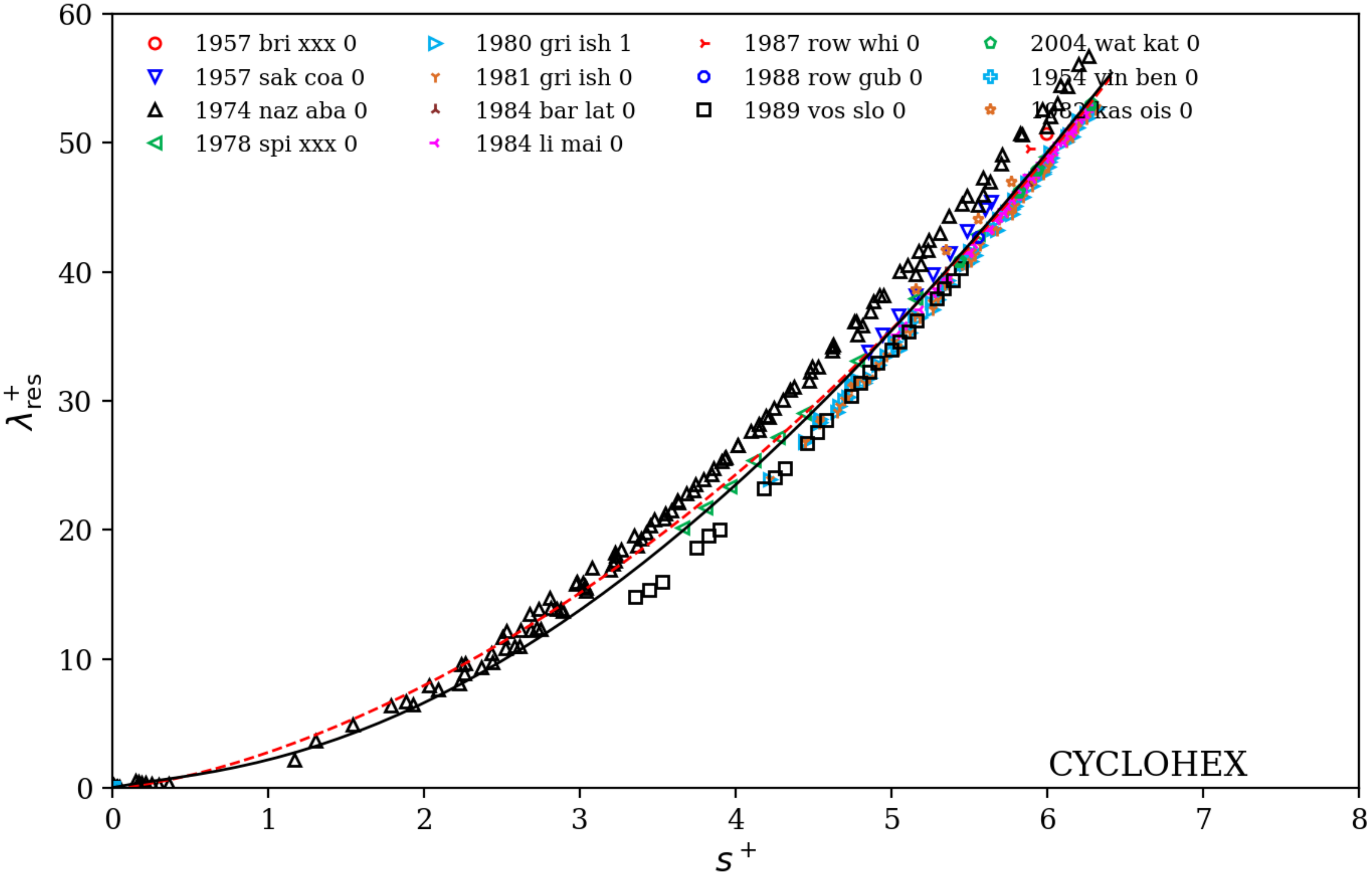

Figure DPR1. CYCLOHEX

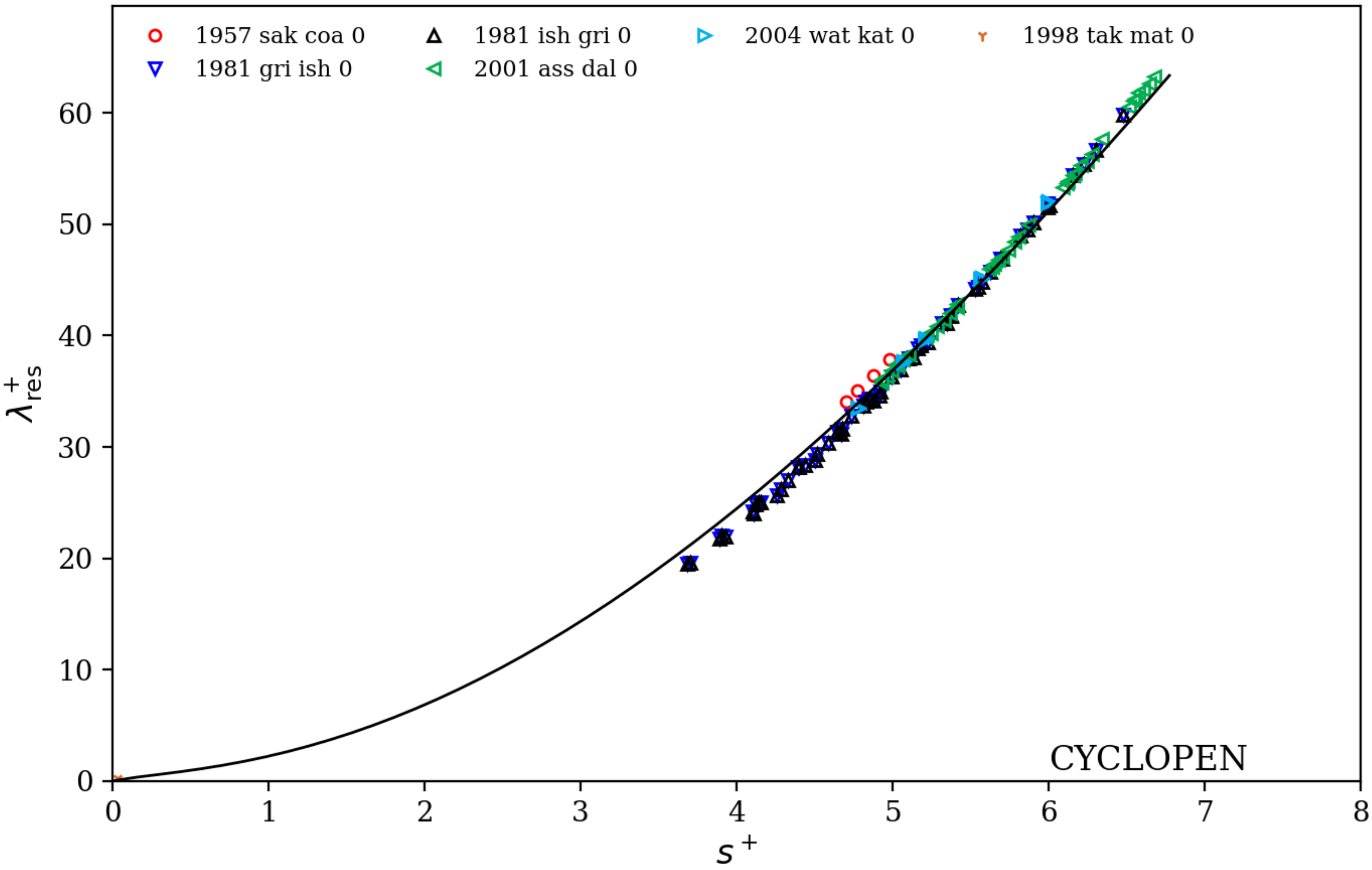

Figure DPR1. CYCLOPEN

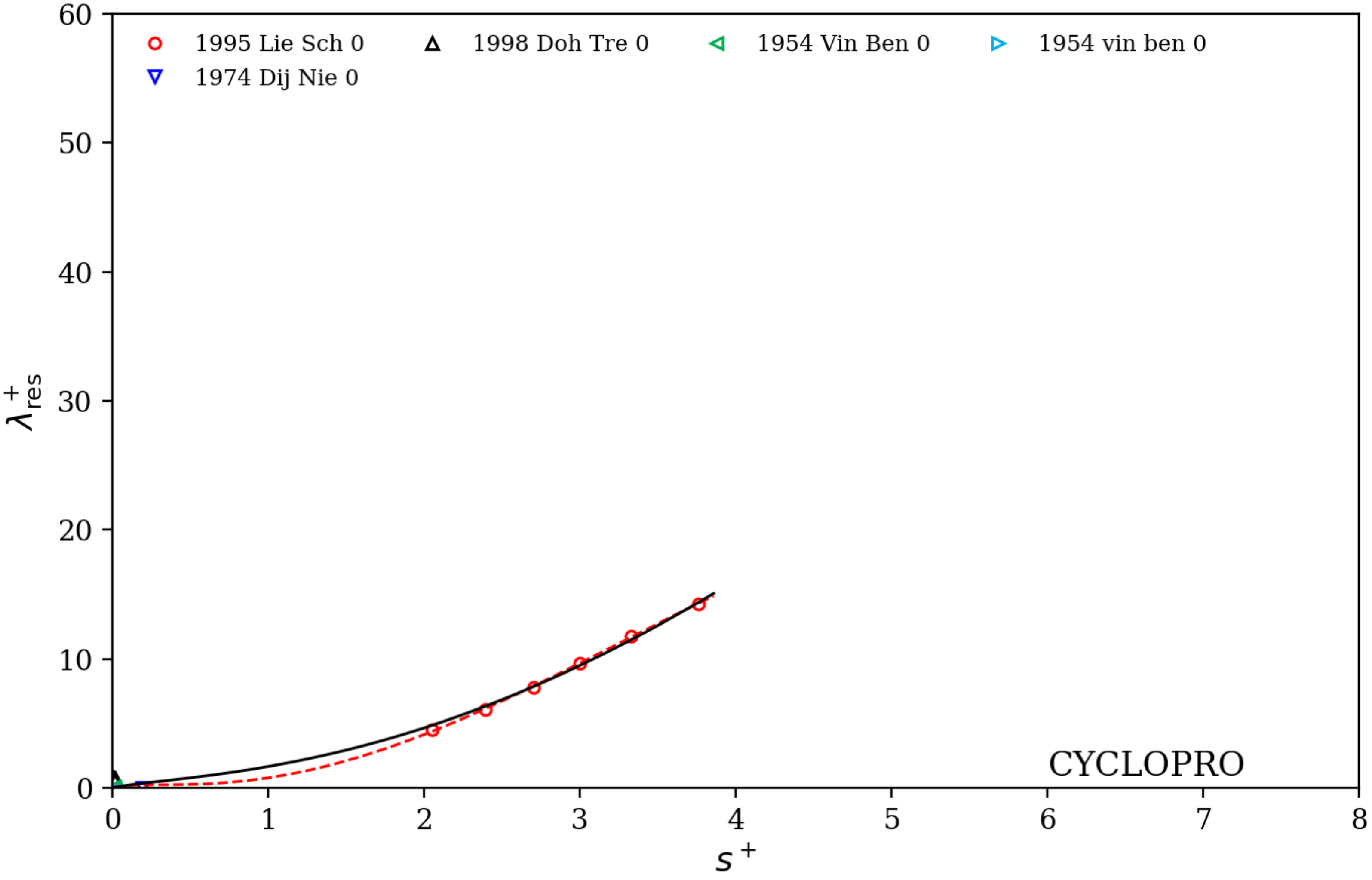

Figure DPR1. CYCLOPRO

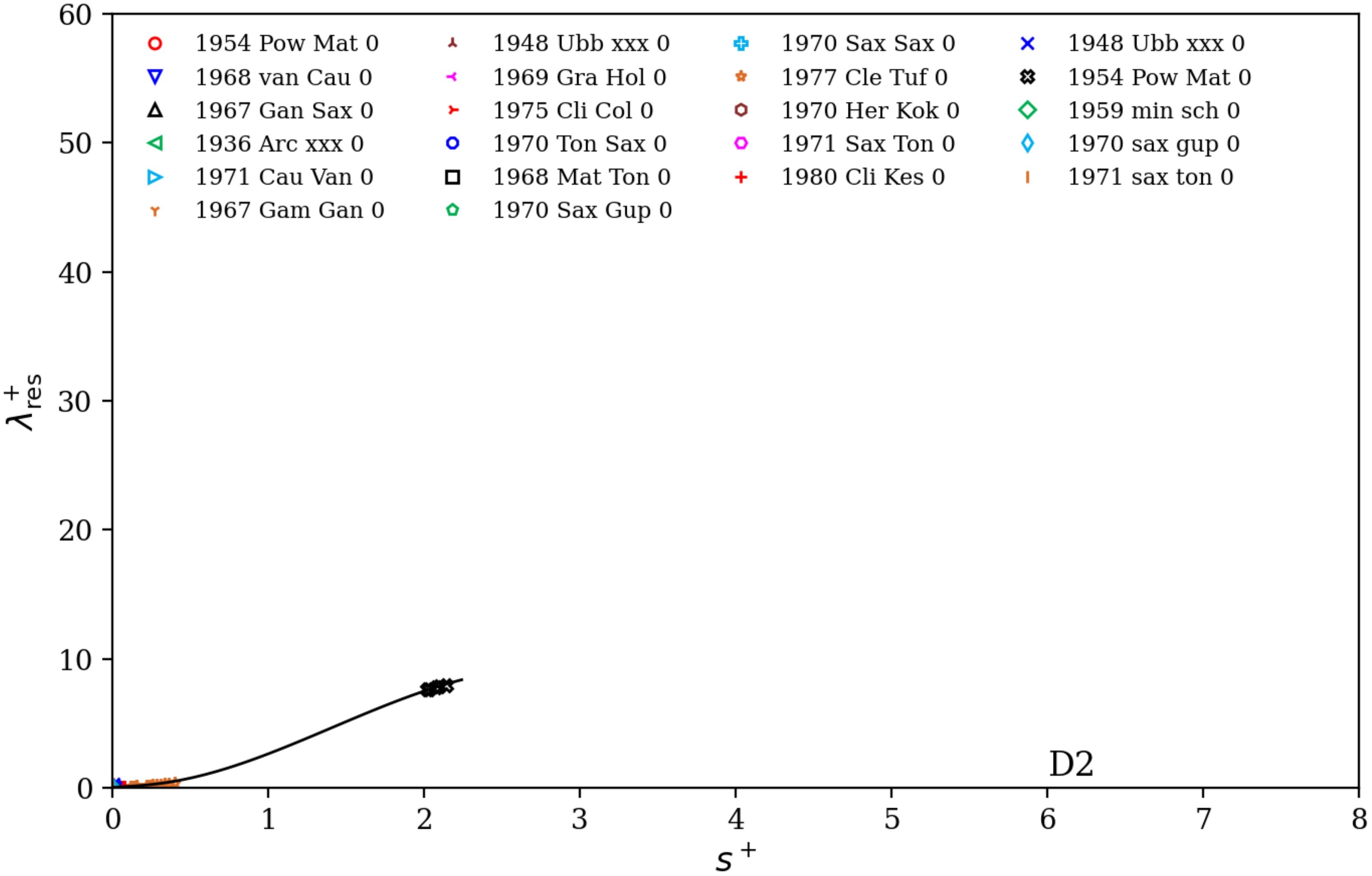

Figure DPR1. D2

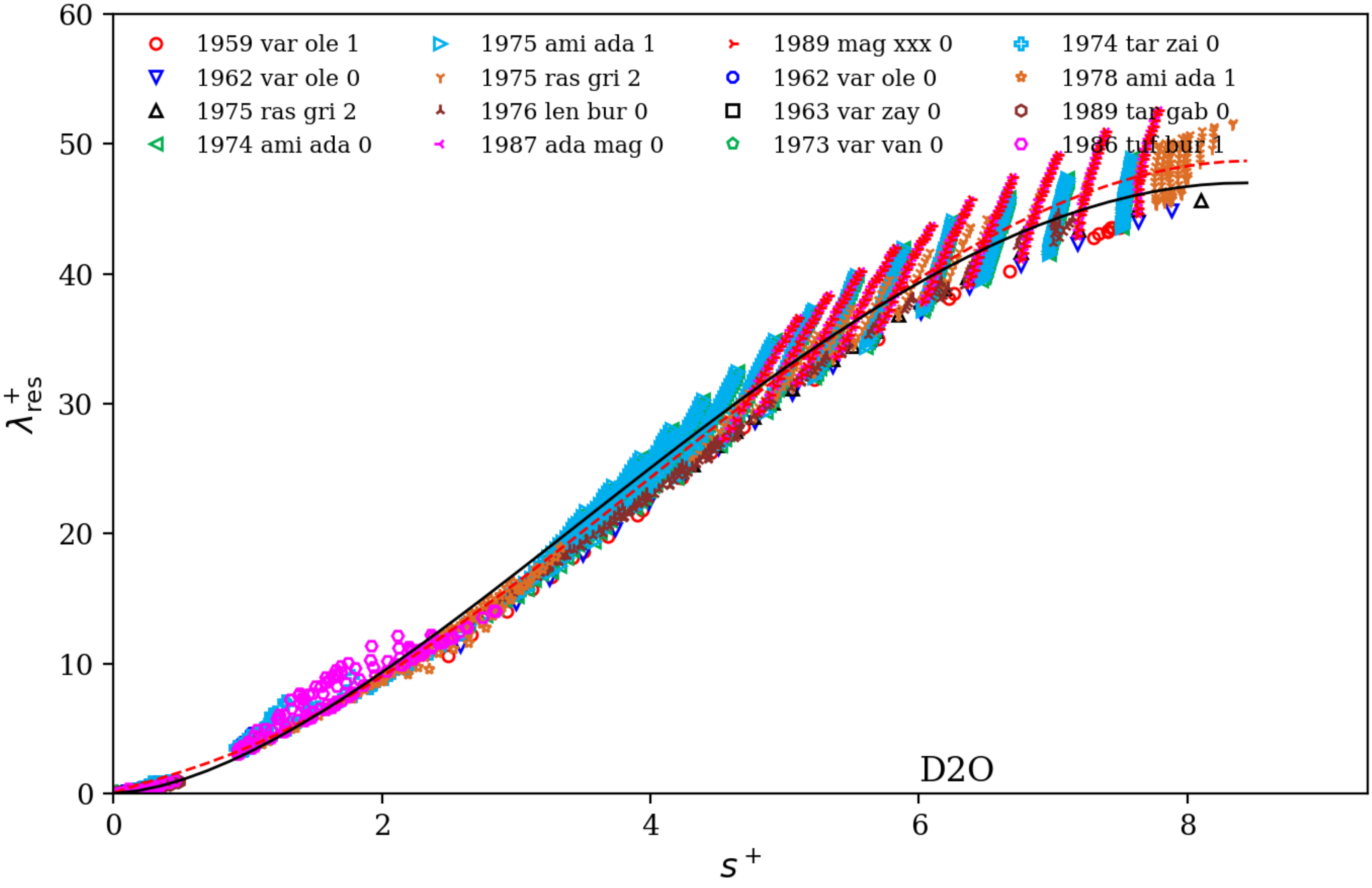

Figure DPR1. D2O

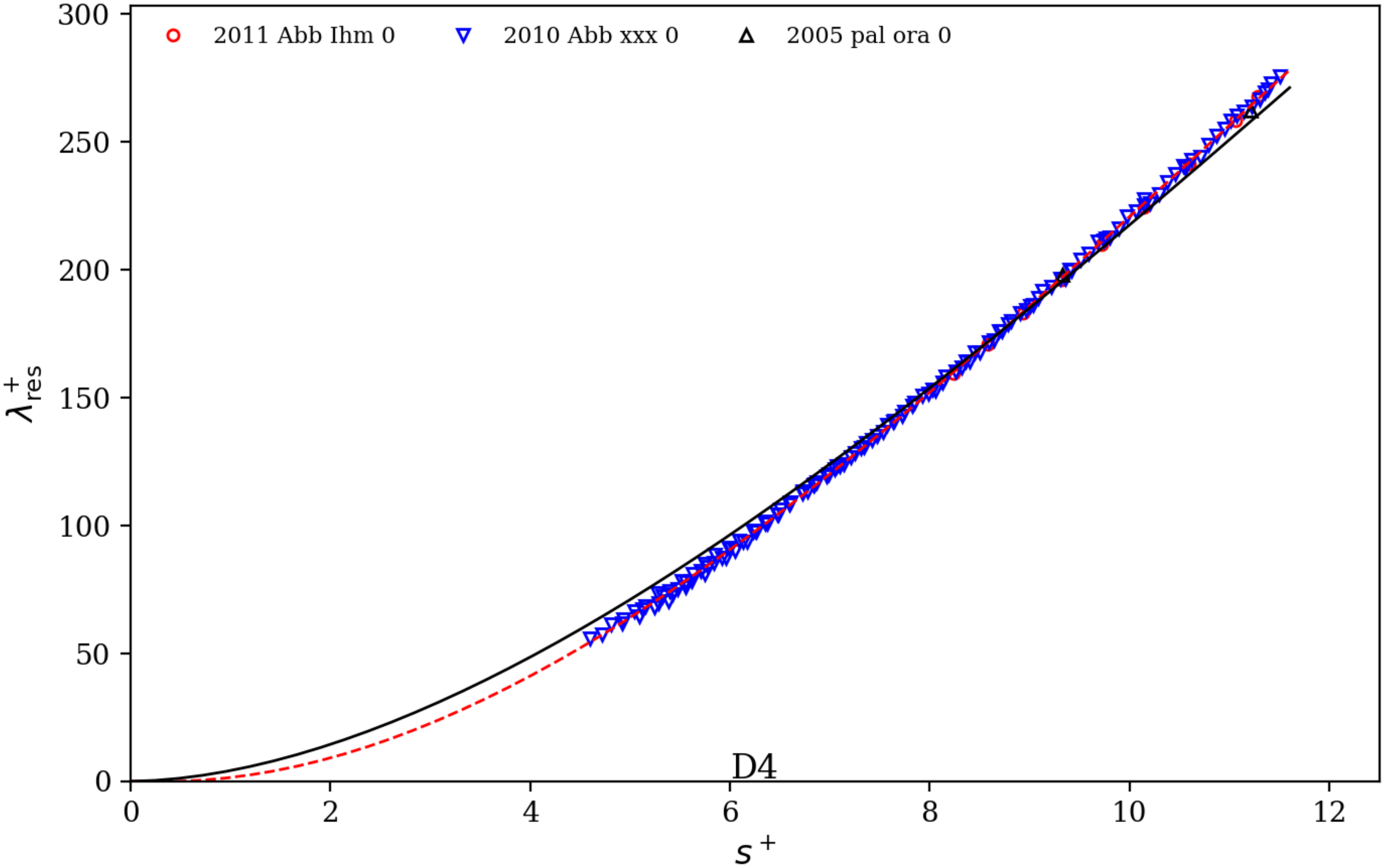

Figure DPR1. D4

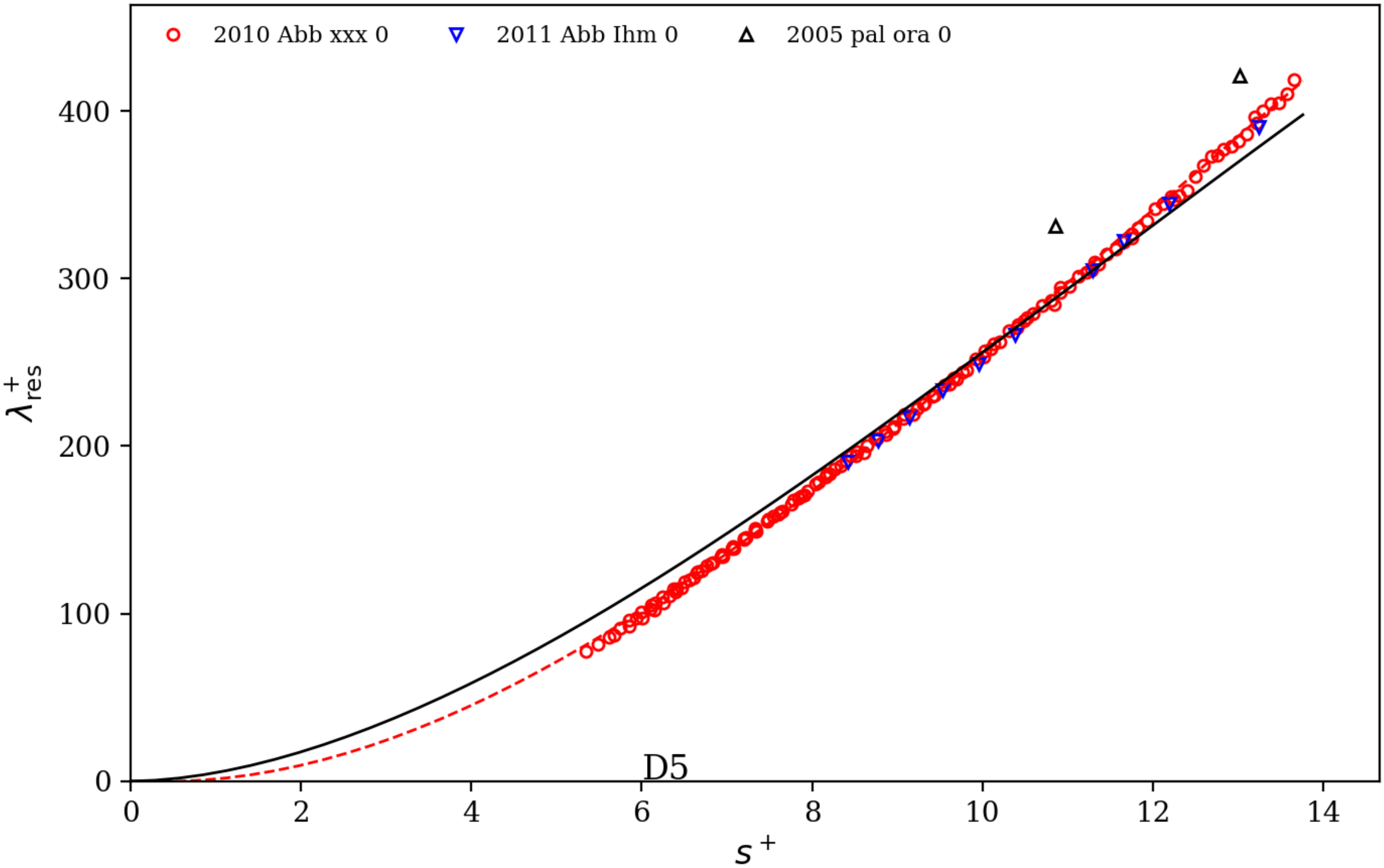

Figure DPR1. D5

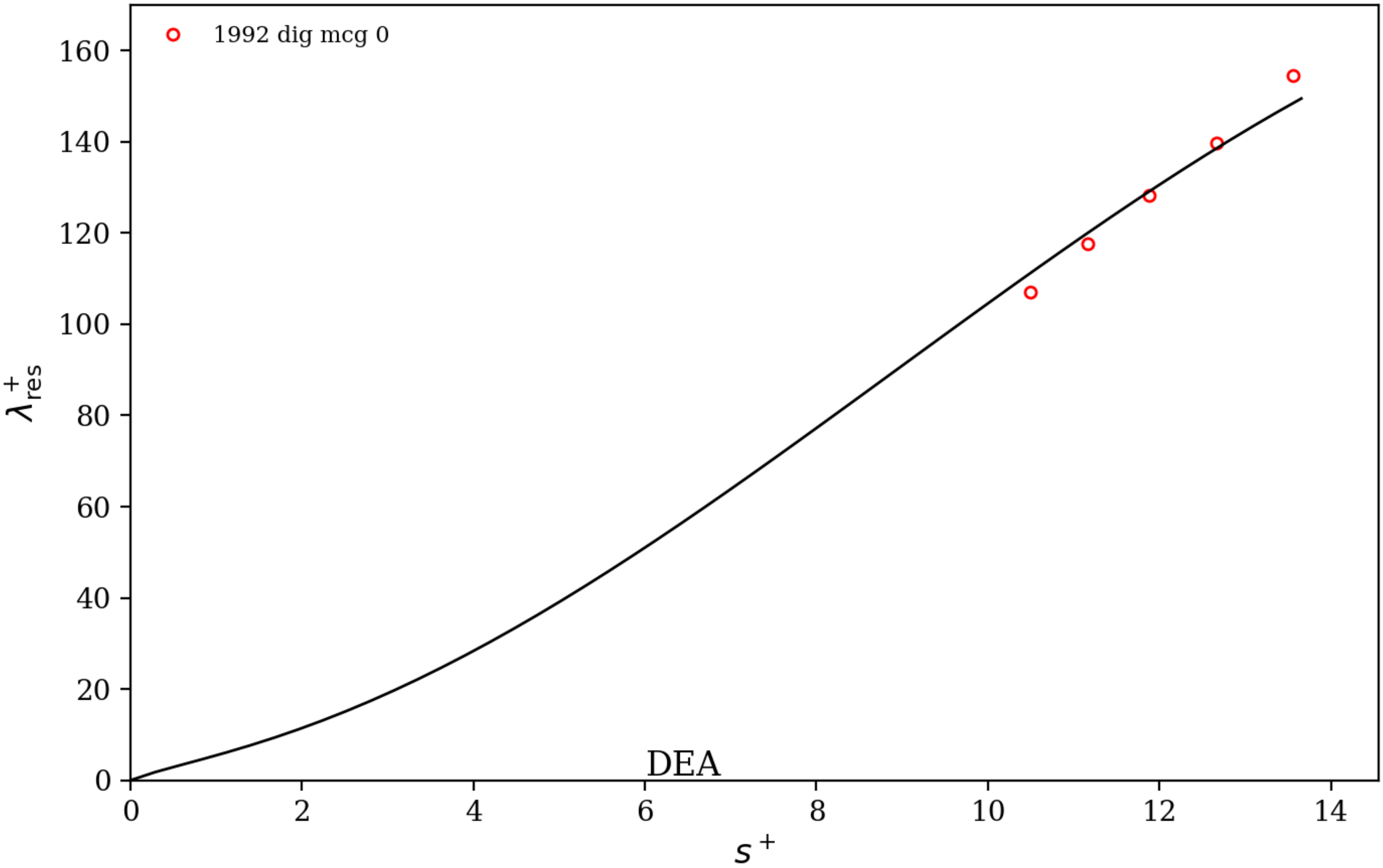

Figure DPR1. DEA

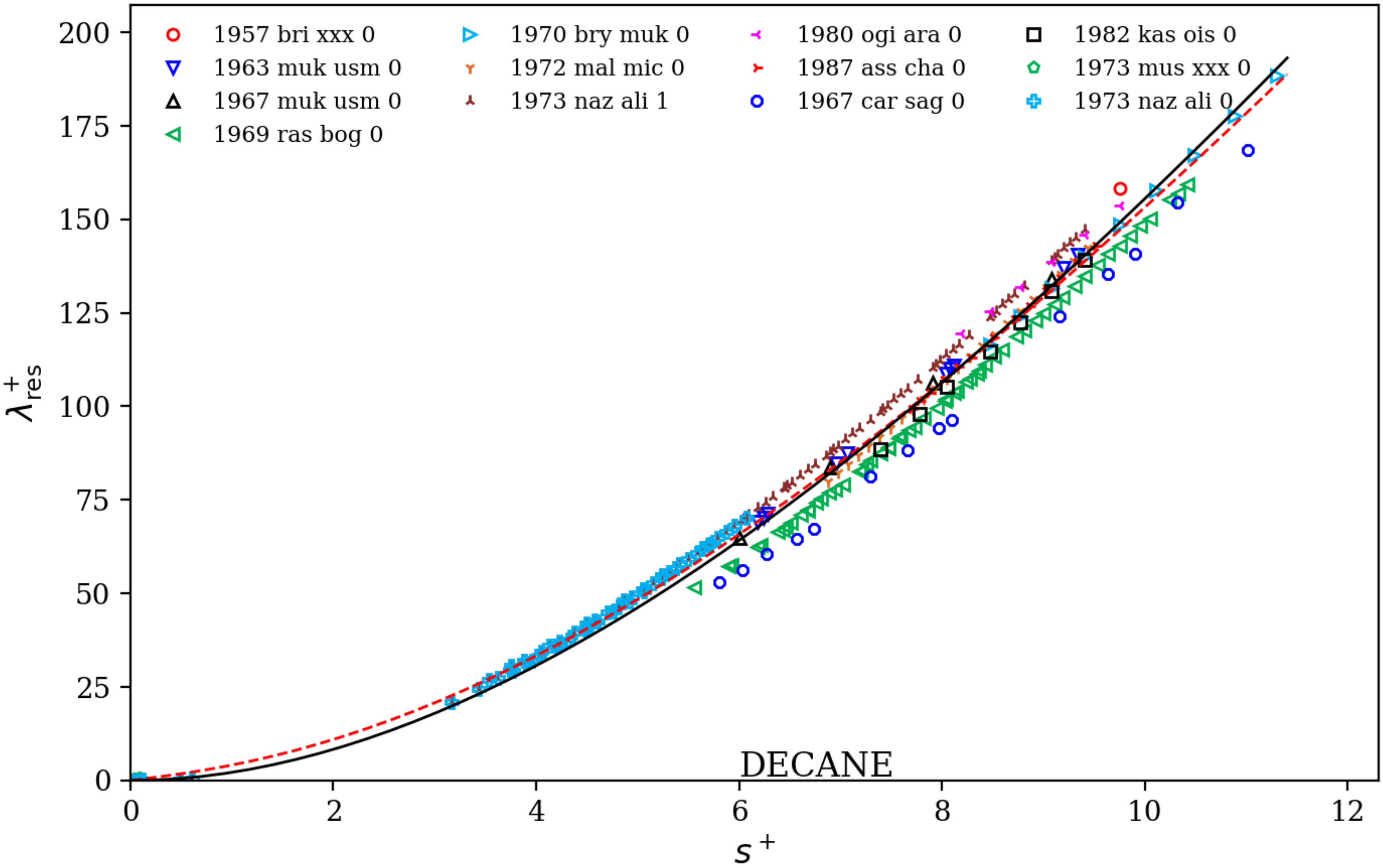

Figure DPR1. DECANE

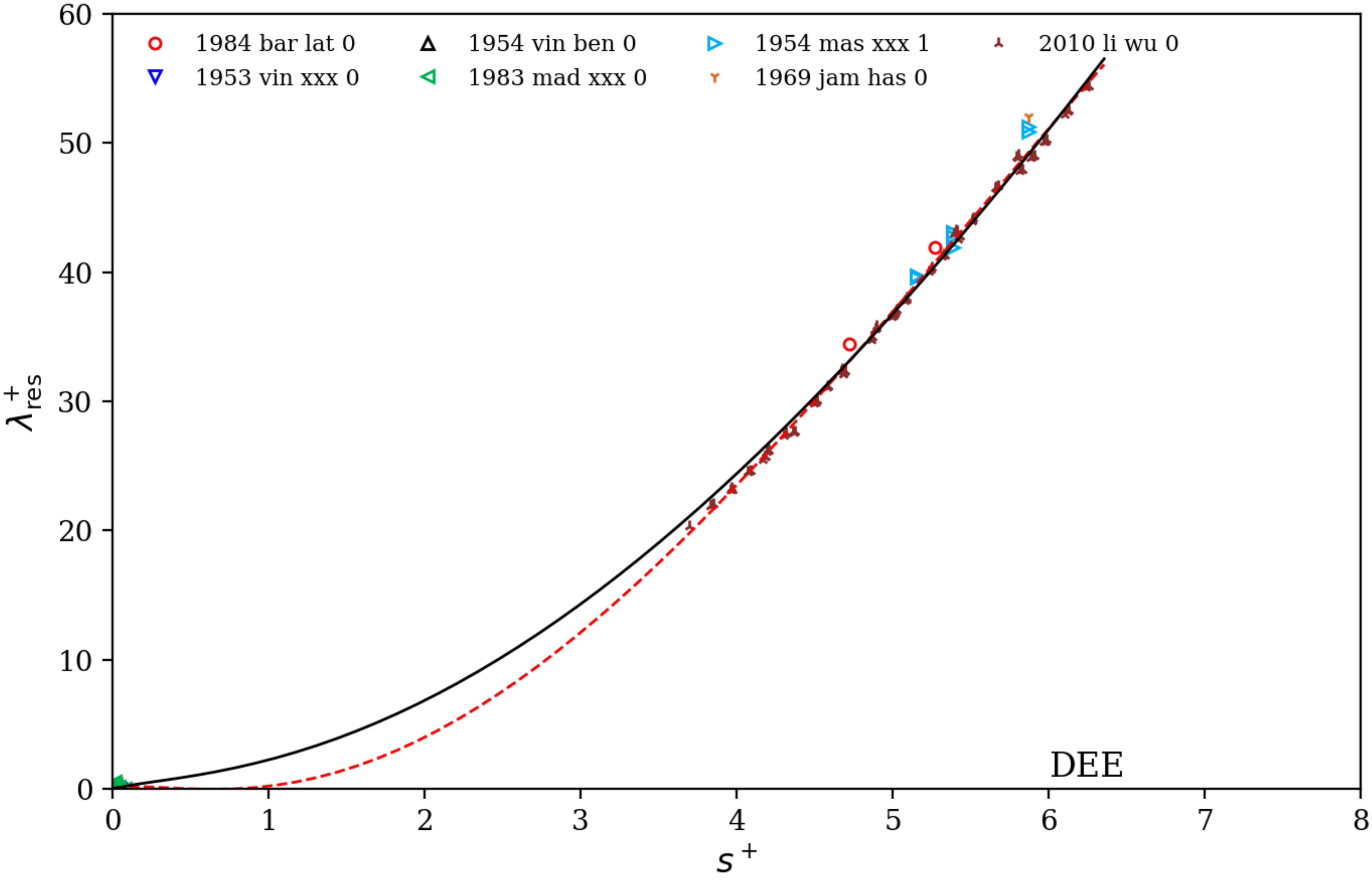

Figure DPR1. DEE

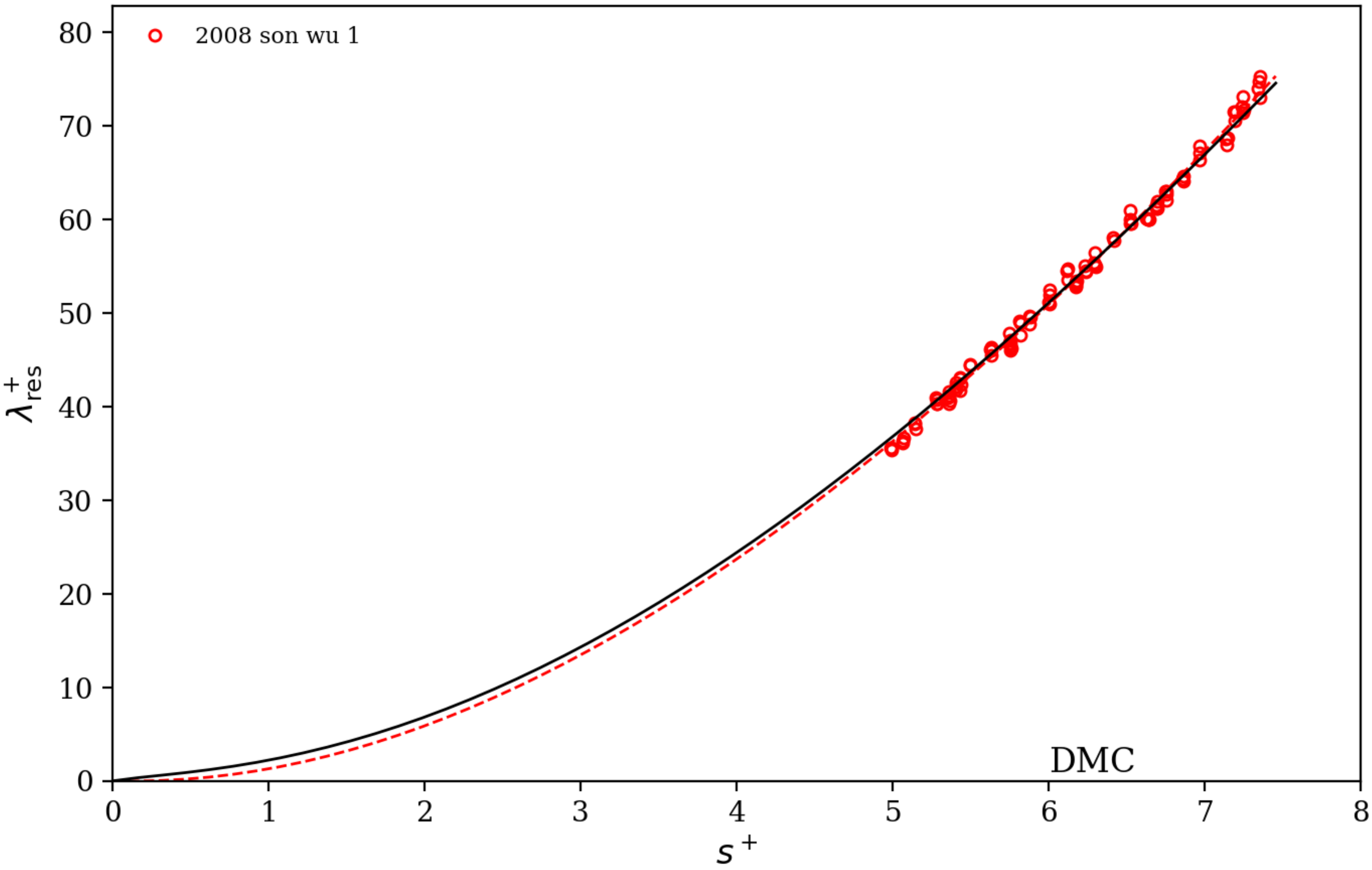

Figure DPR1. DMC

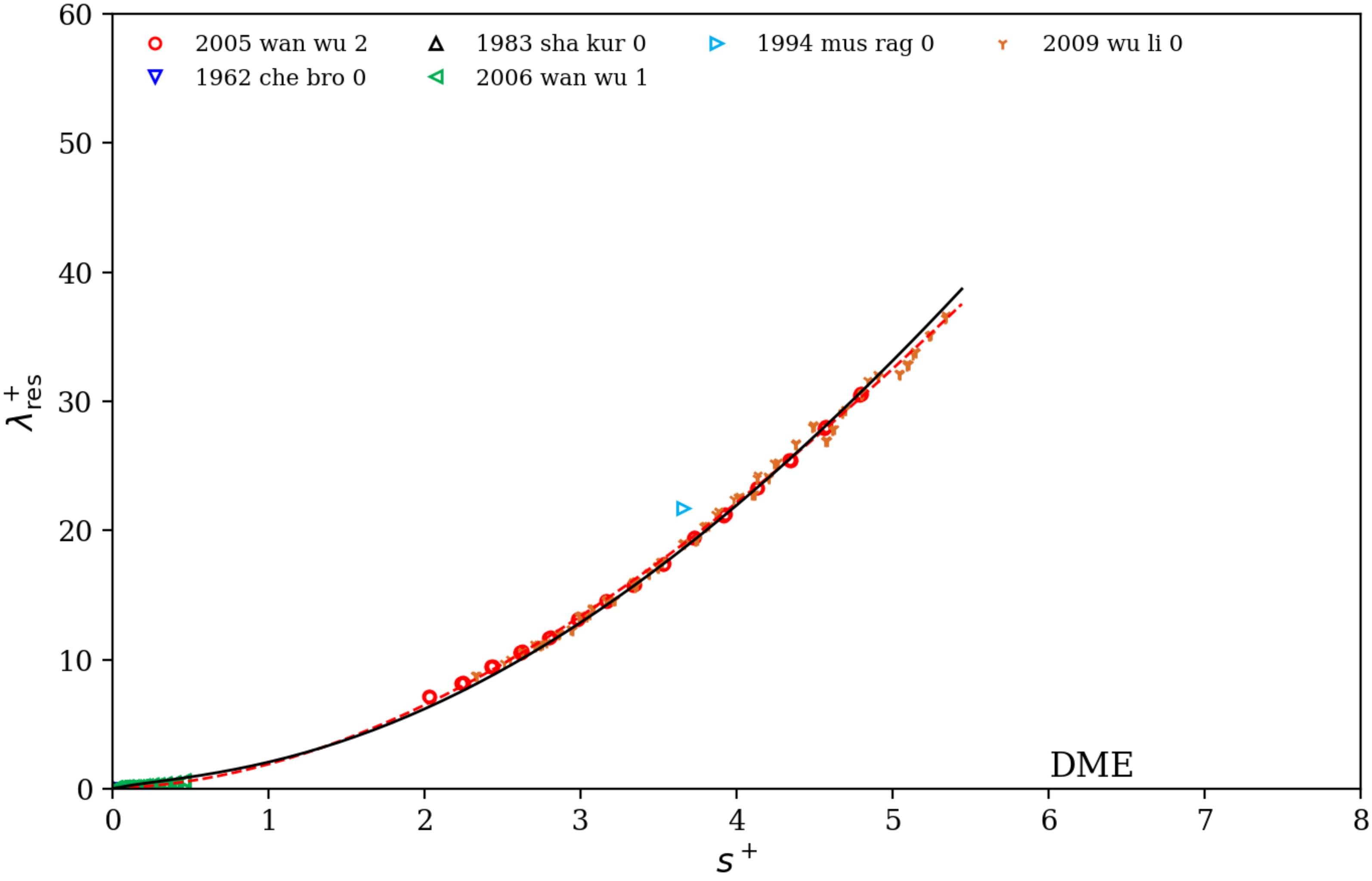

Figure DPR1. DME

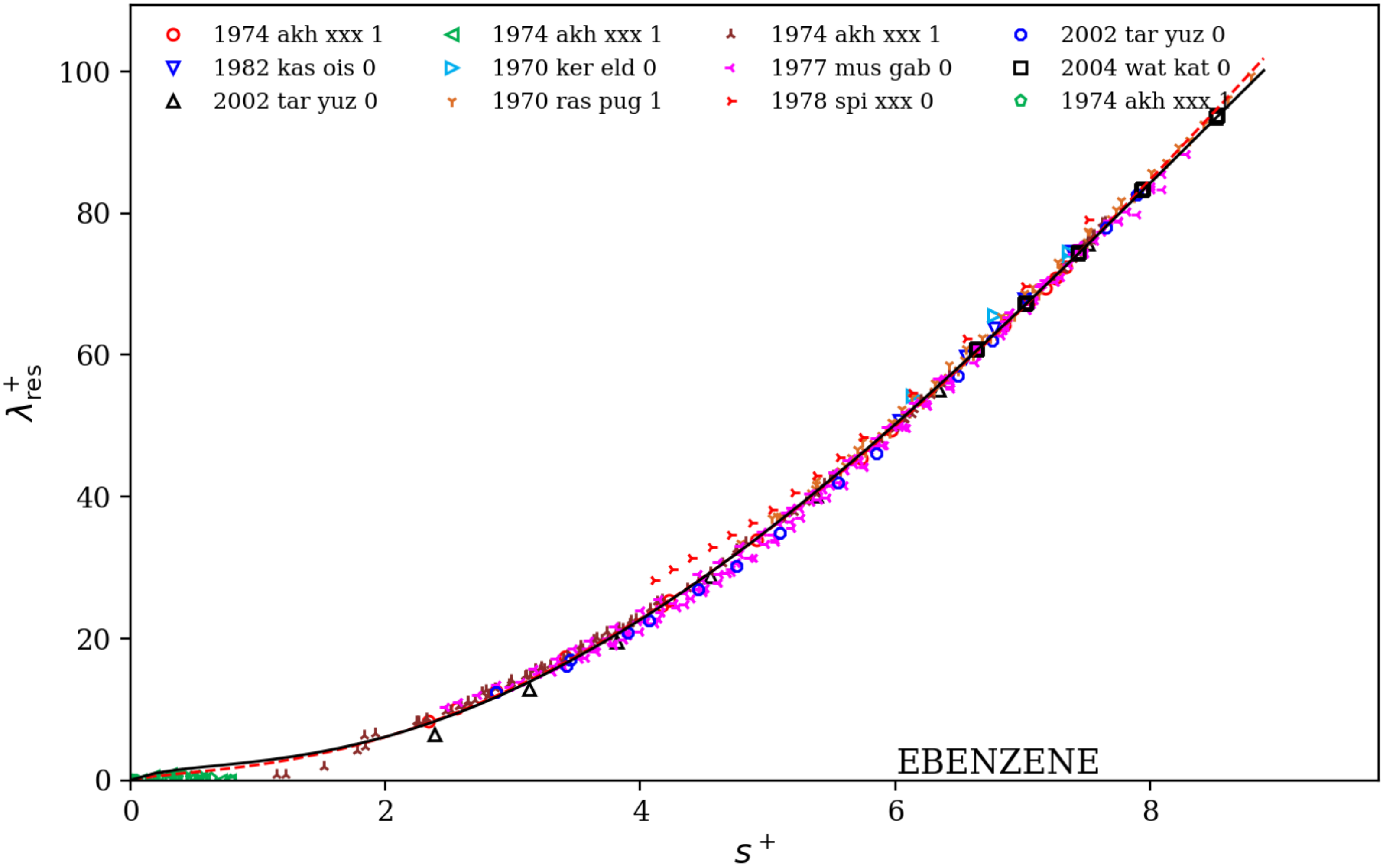

Figure DPR1. EBENZENE

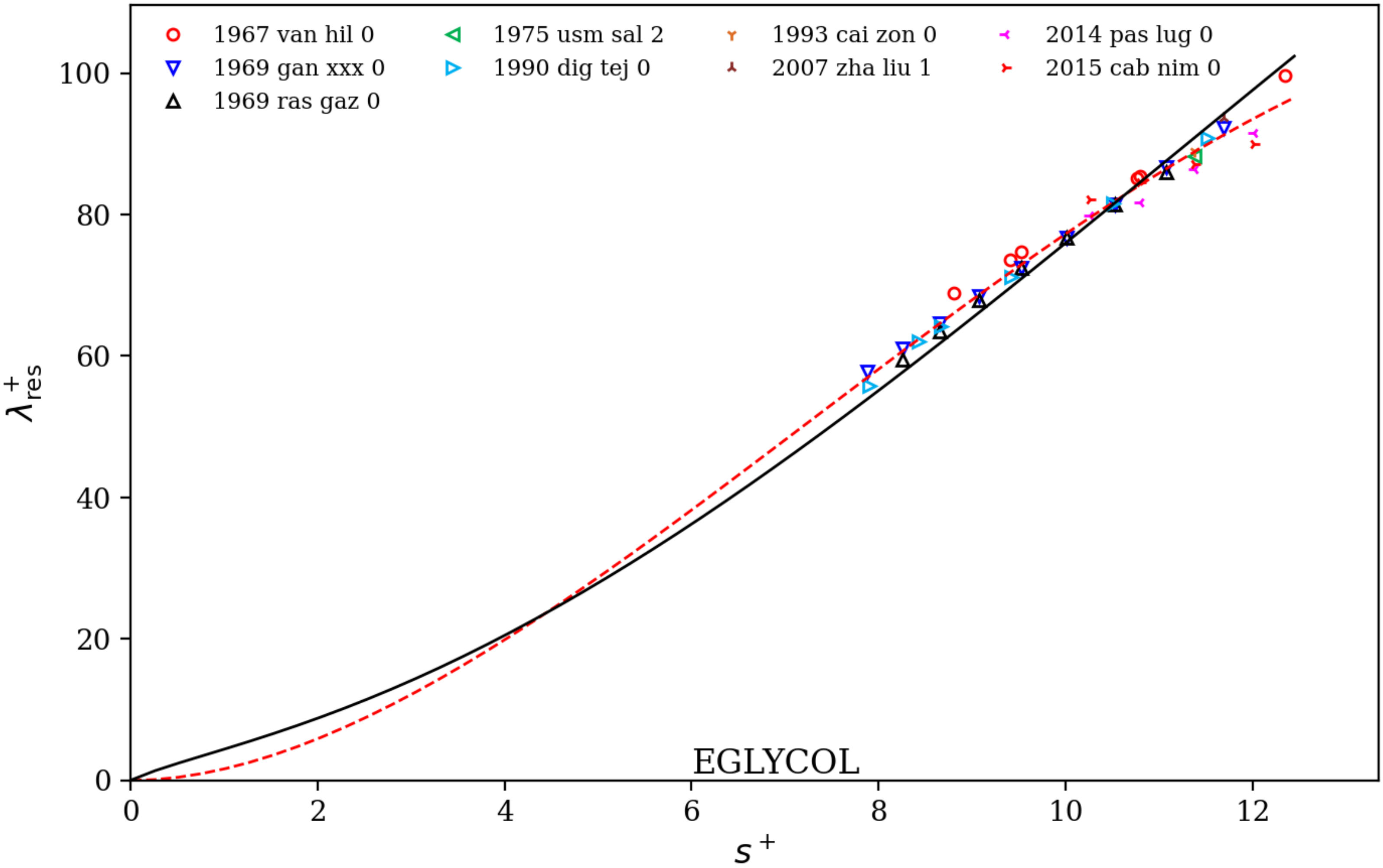

Figure DPR1. EGLYCOL

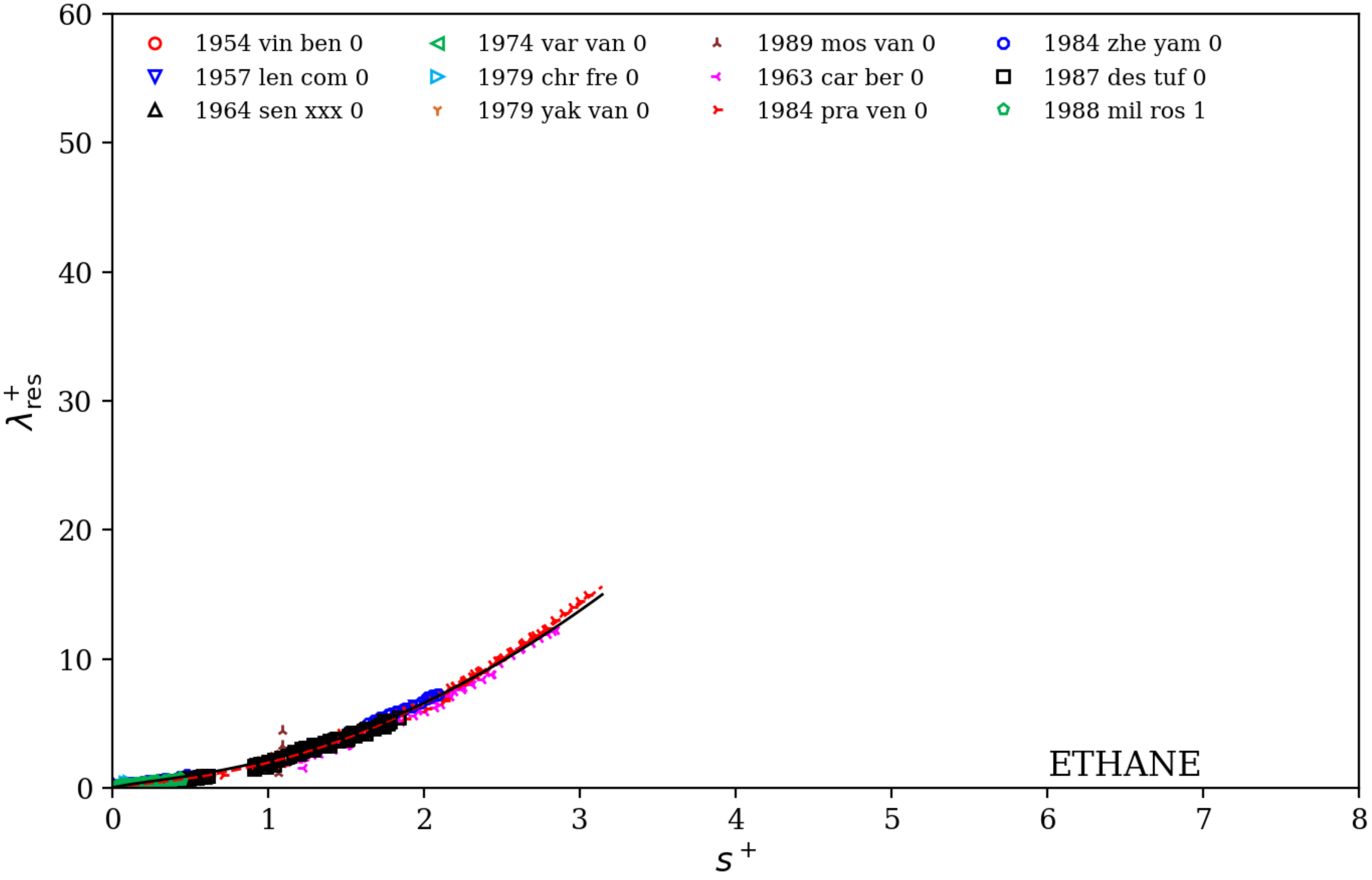

Figure DPR1. ETHANE

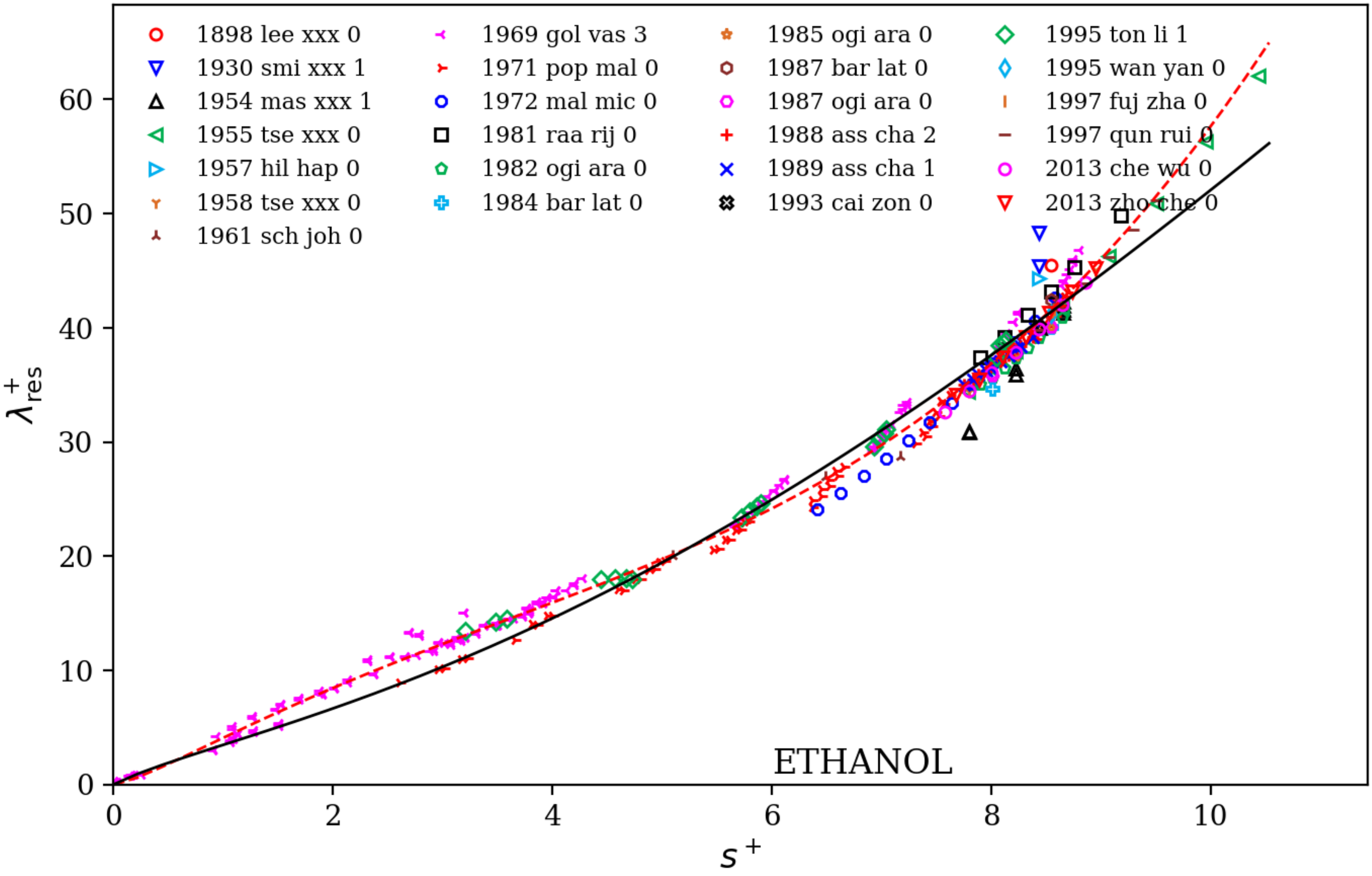

Figure DPR1. ETHANOL

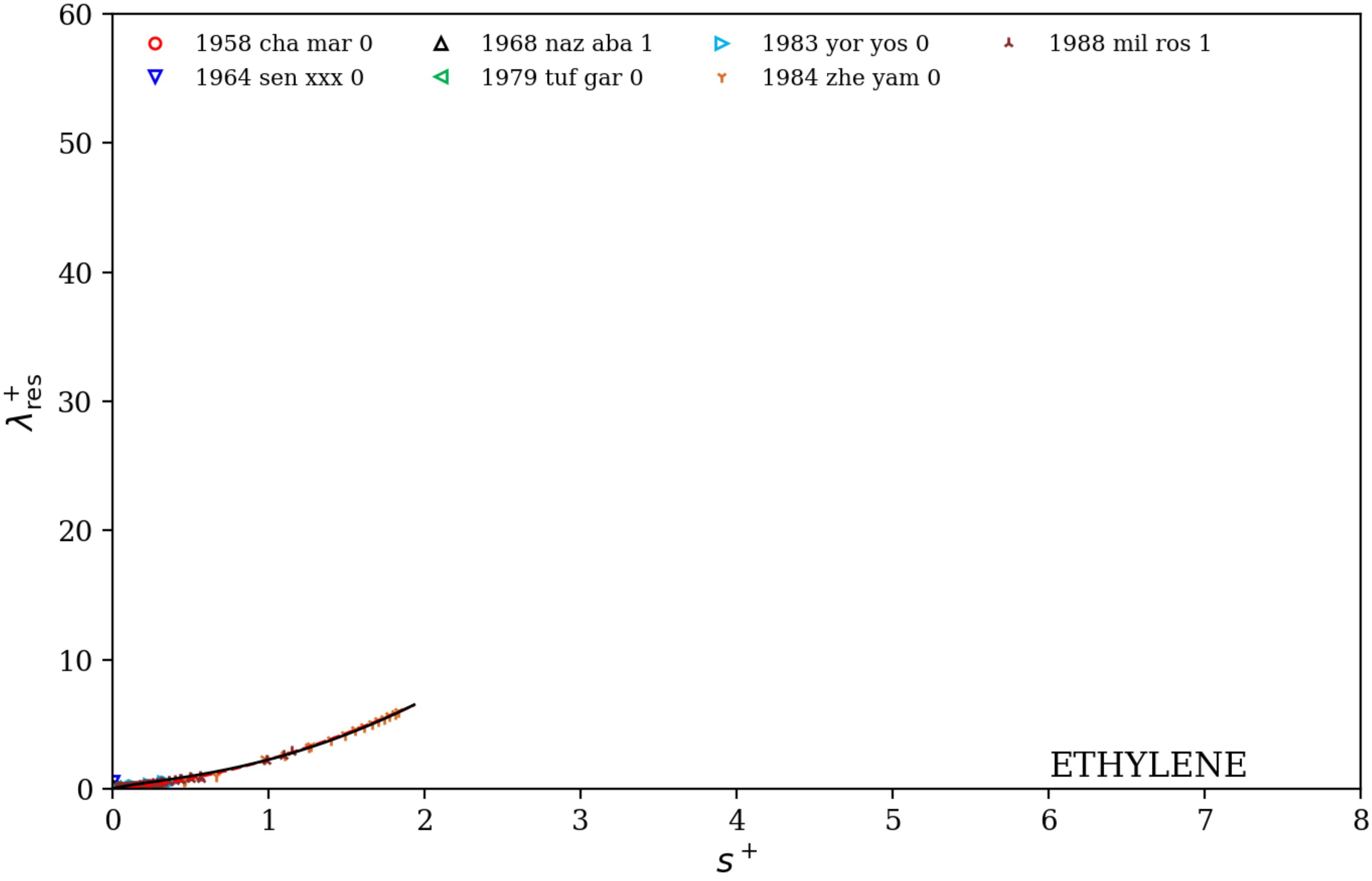

Figure DPR1. ETHYLENE

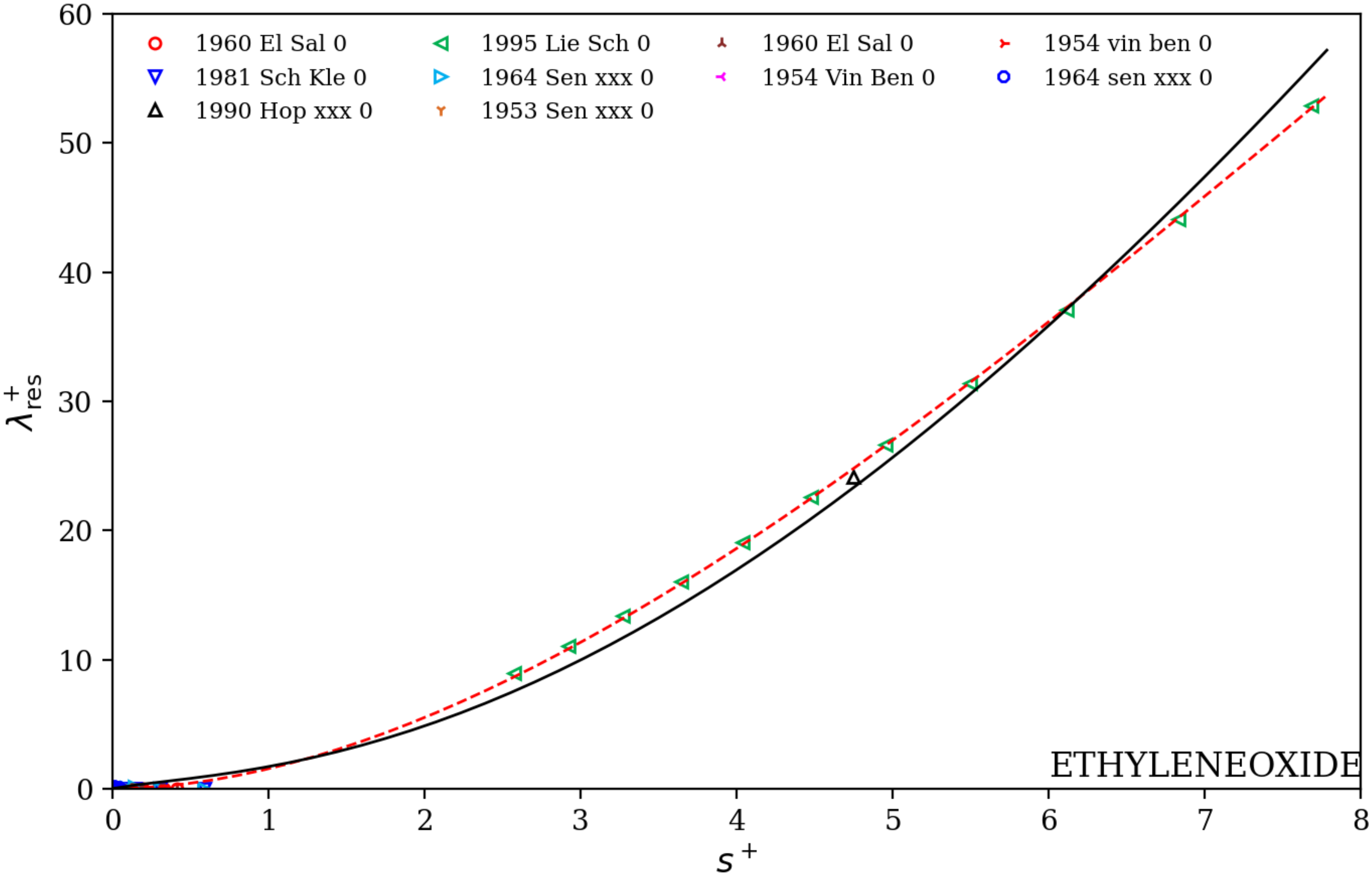

Figure DPR1. ETHYLENEOXIDE

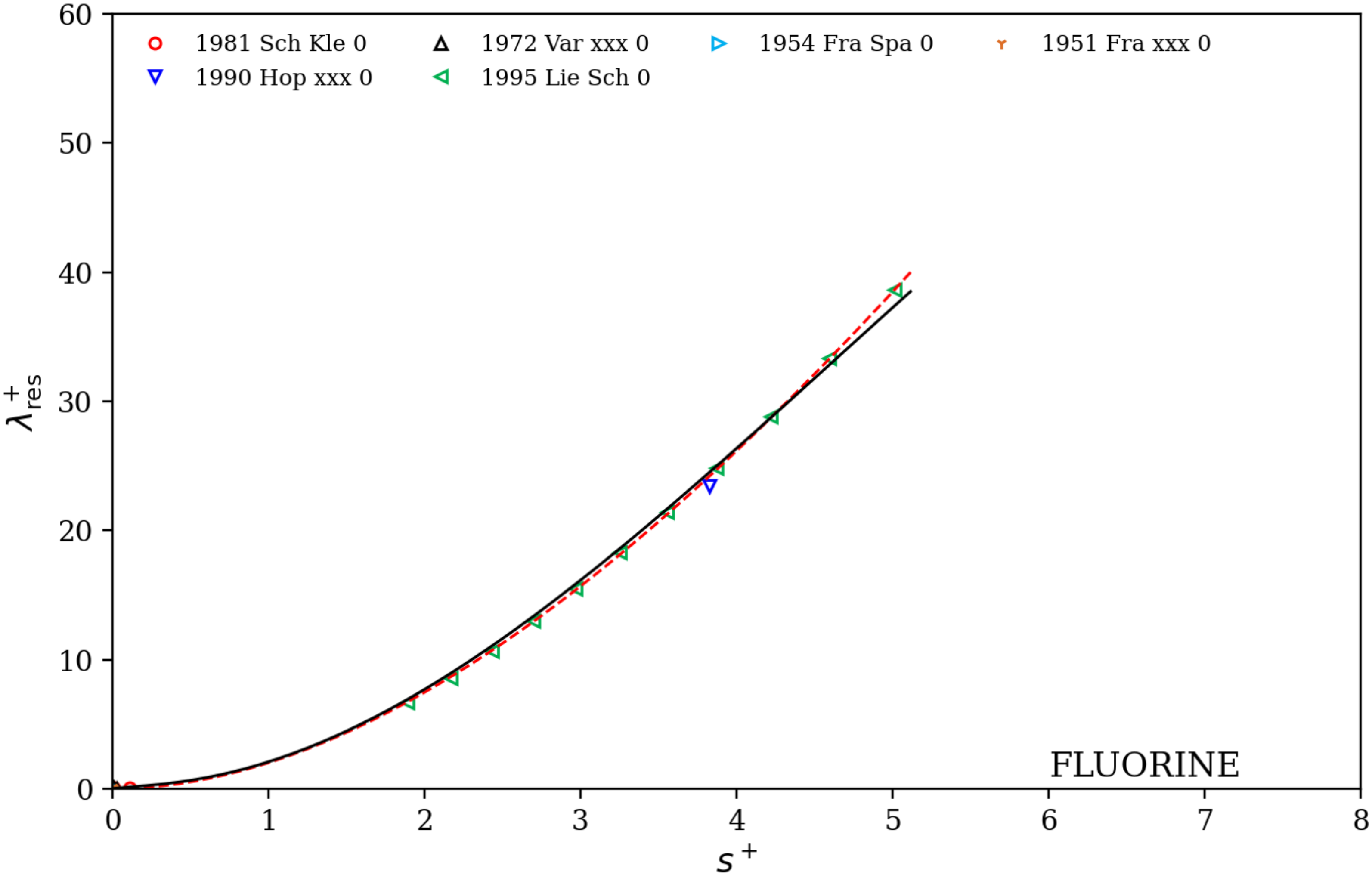

Figure DPR1. FLUORINE

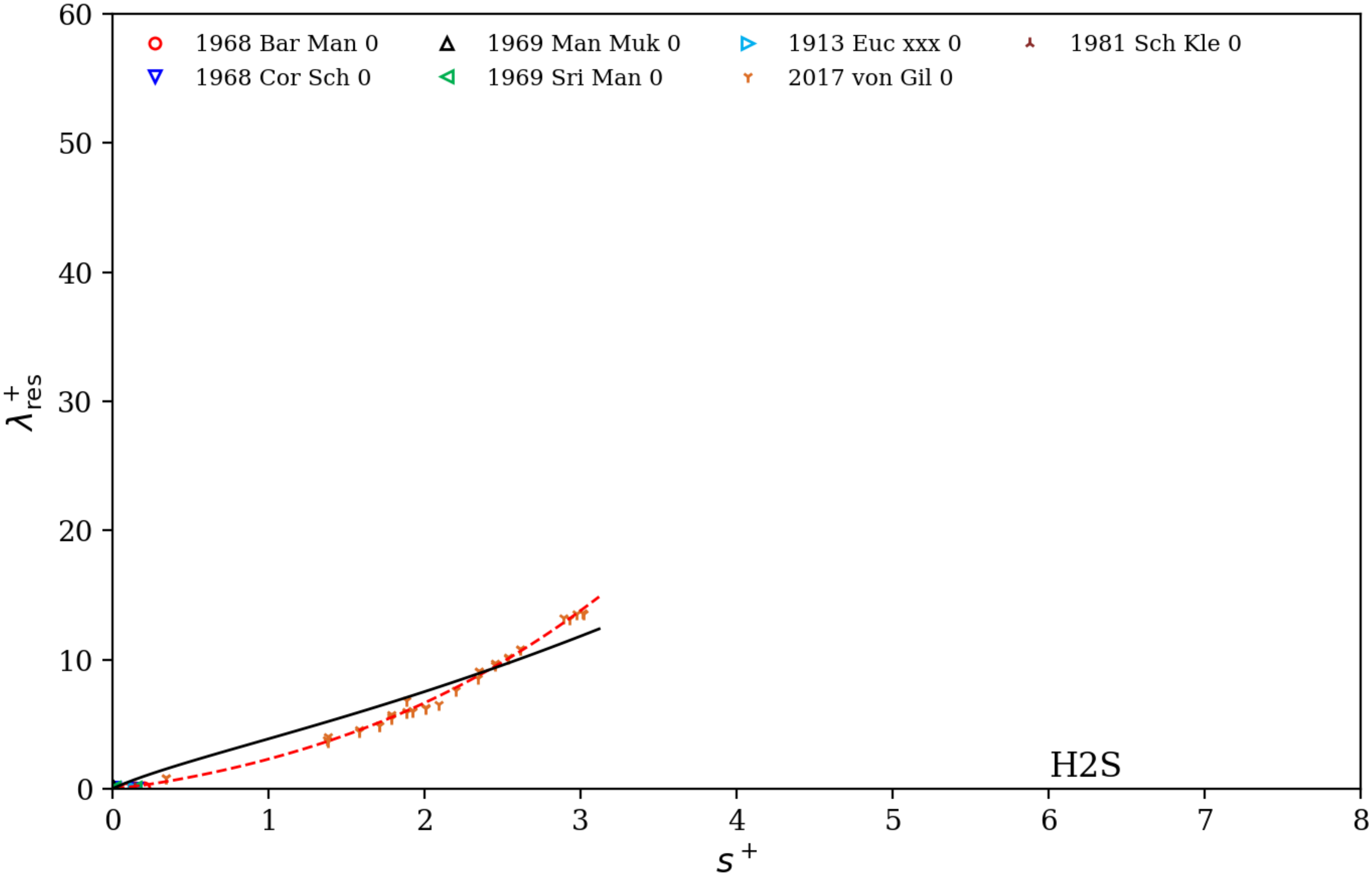

Figure DPR1. H2S

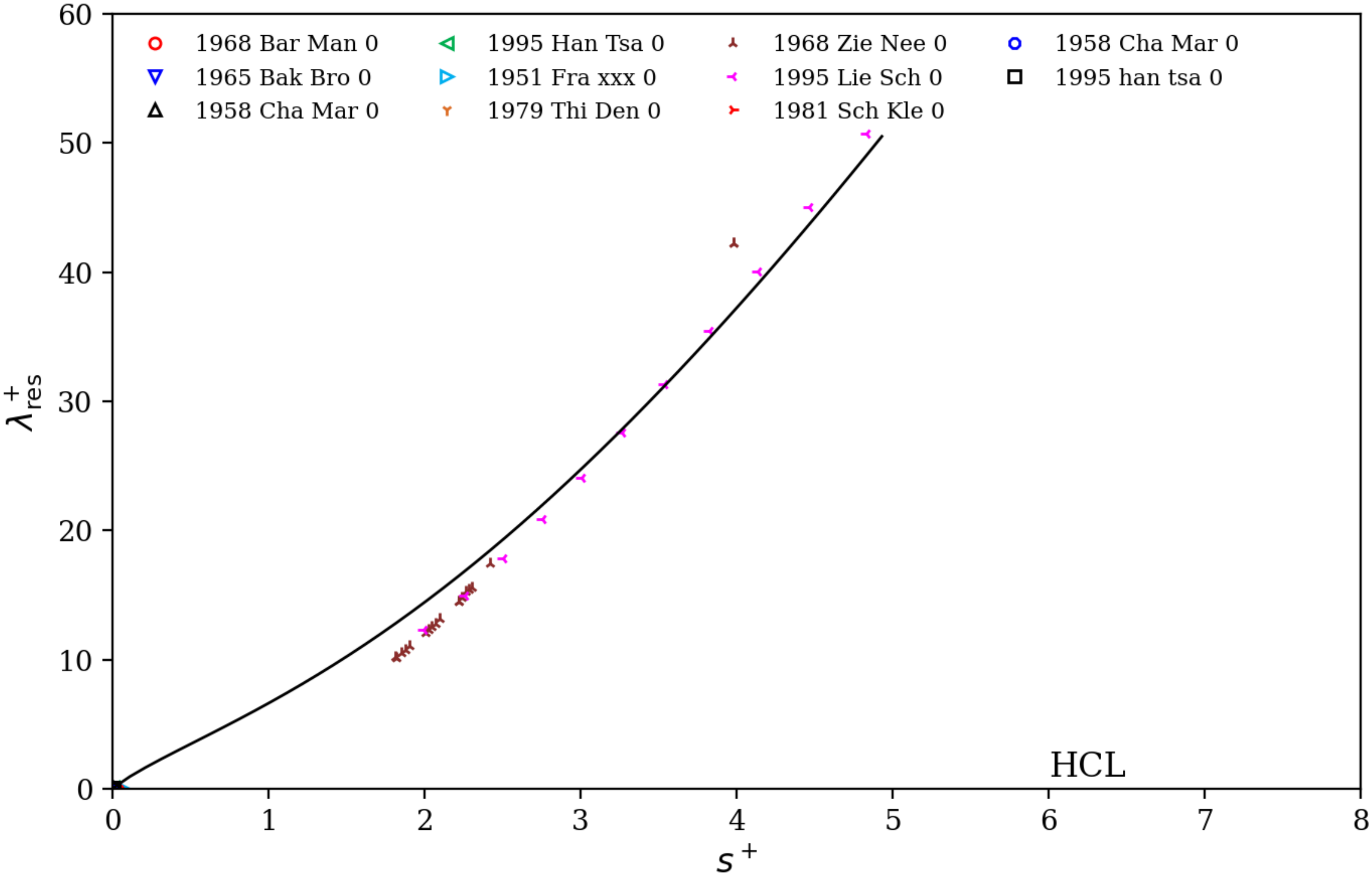

Figure DPR1. HCL

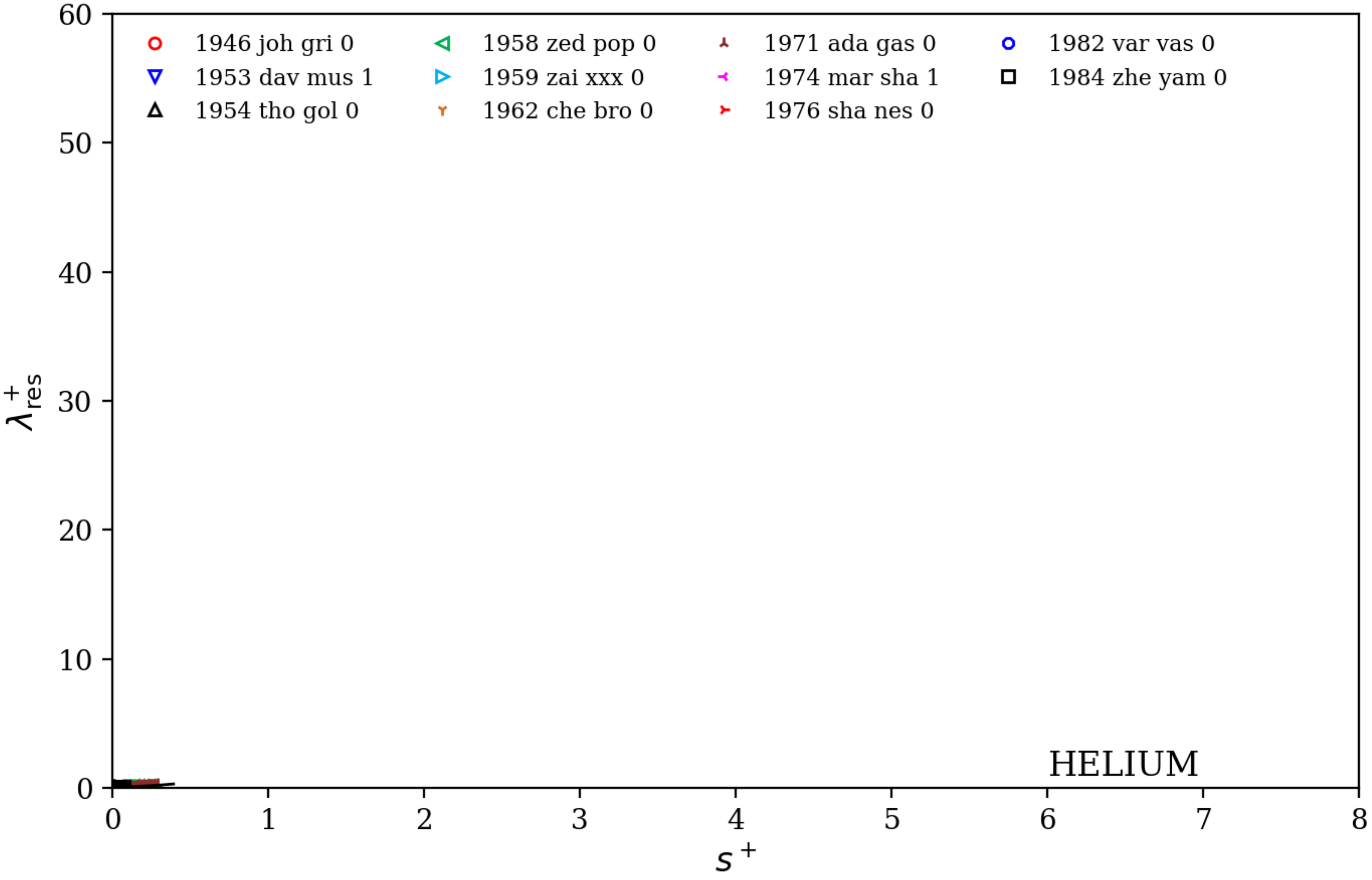

Figure DPR1. HELIUM

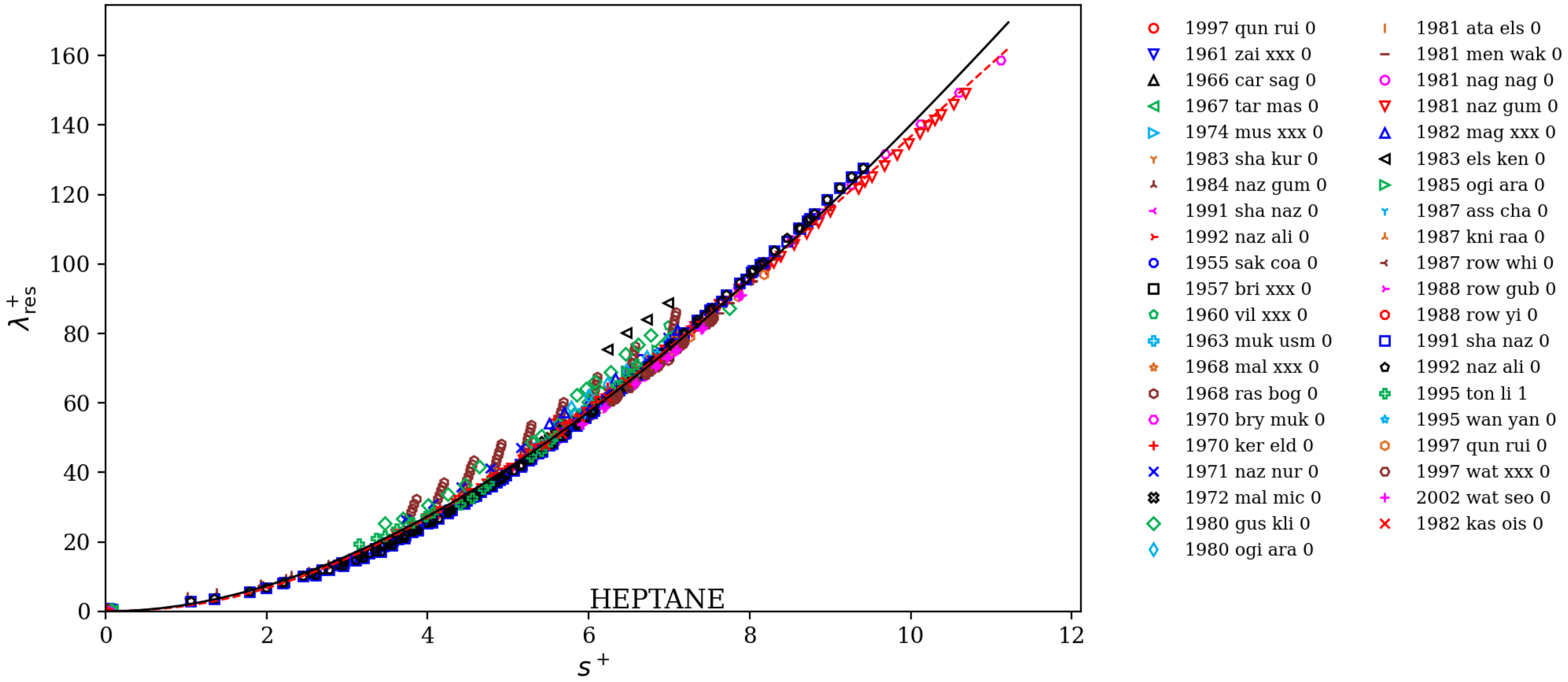

Figure DPR1. HEPTANE

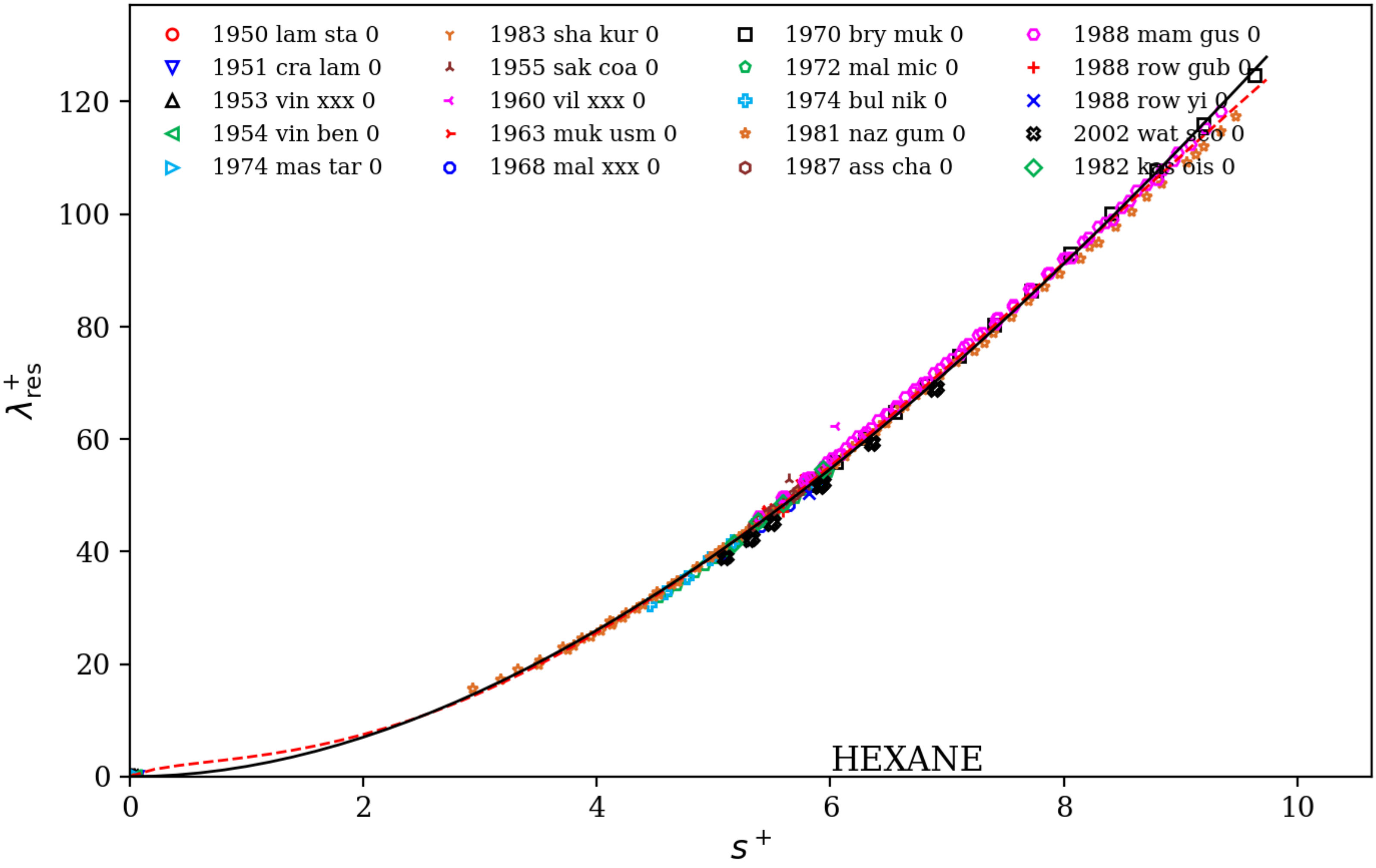

Figure DPR1. HEXANE

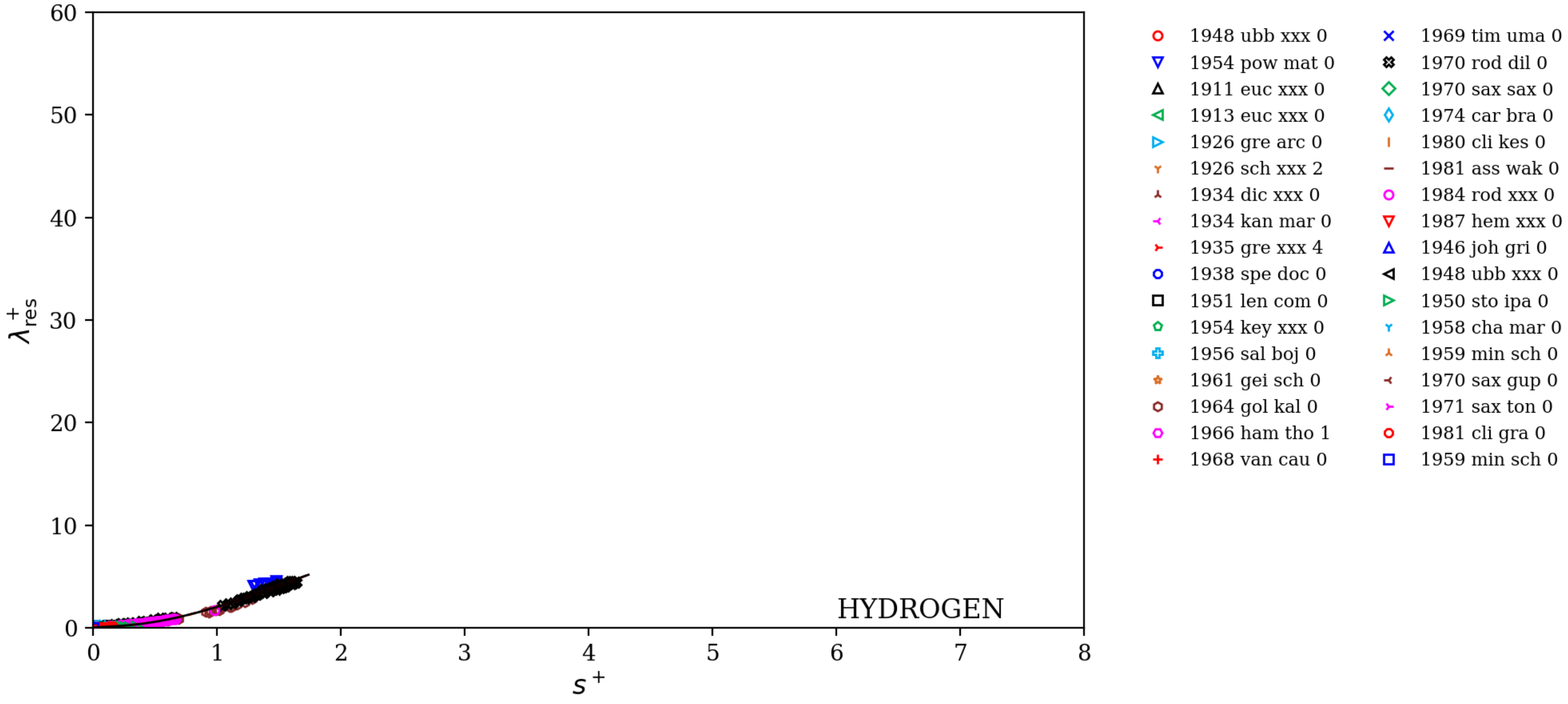

Figure DPR1. HYDROGEN

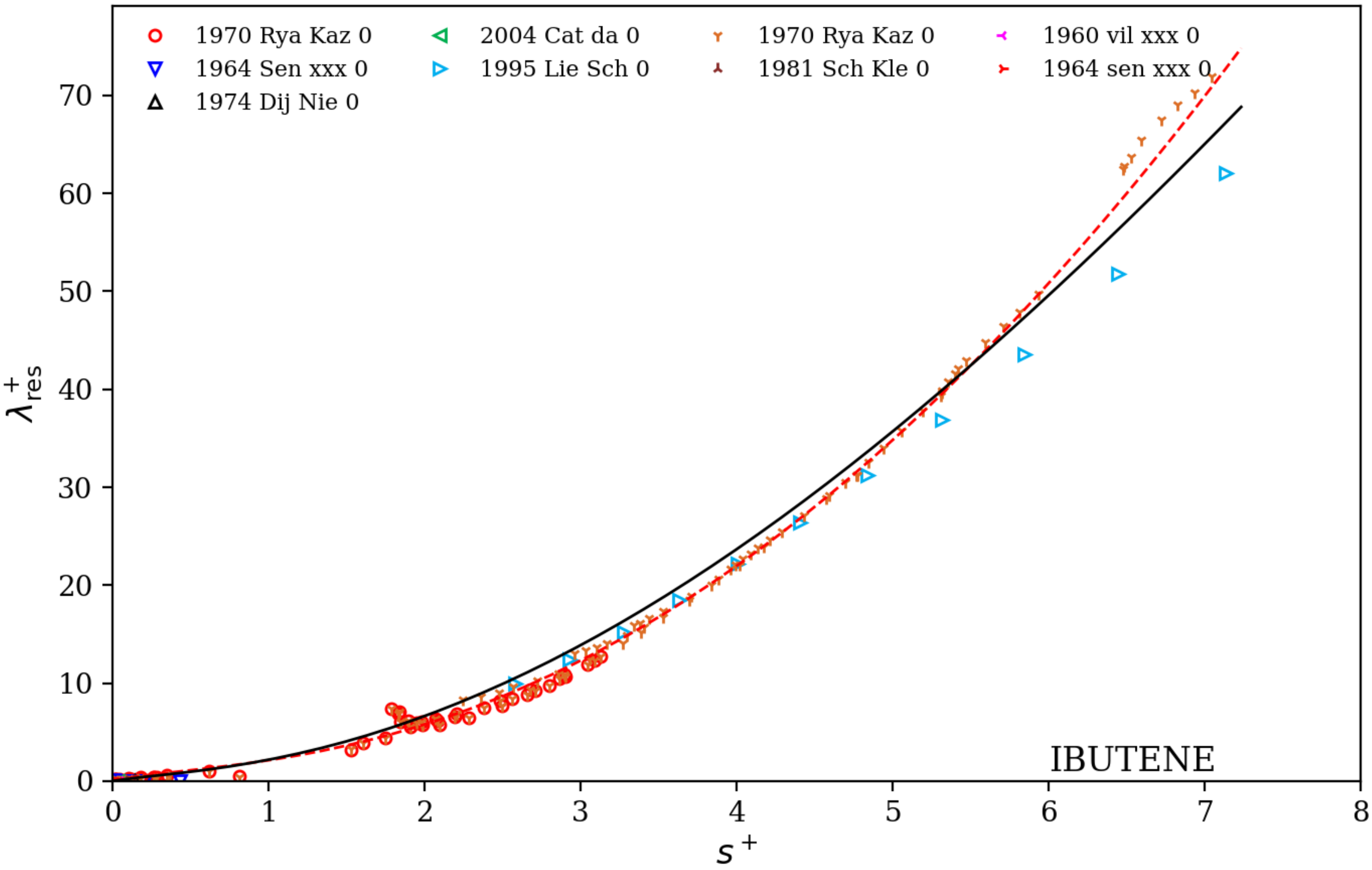

Figure DPR1. IBUTENE

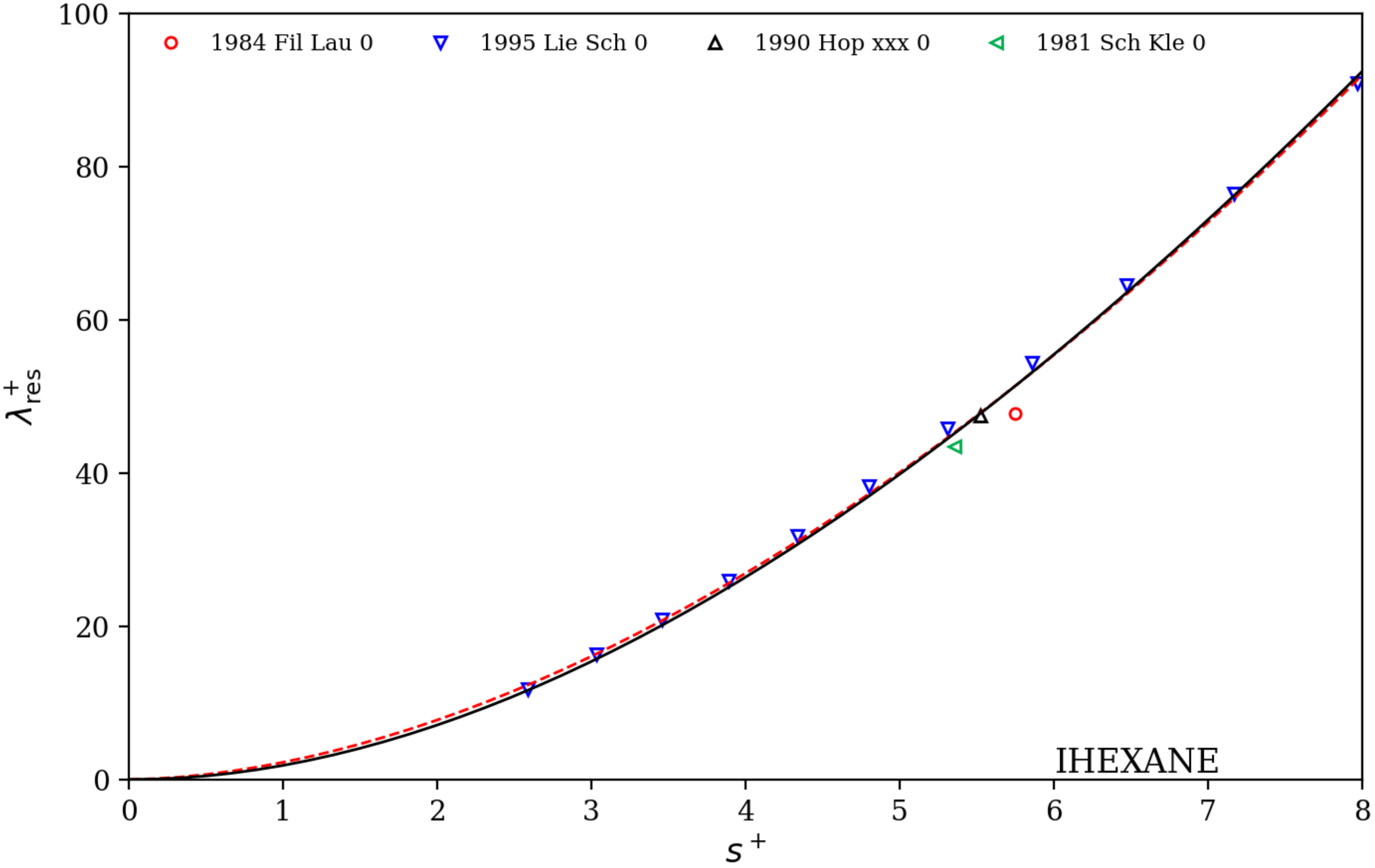

Figure DPR1. IHEXANE

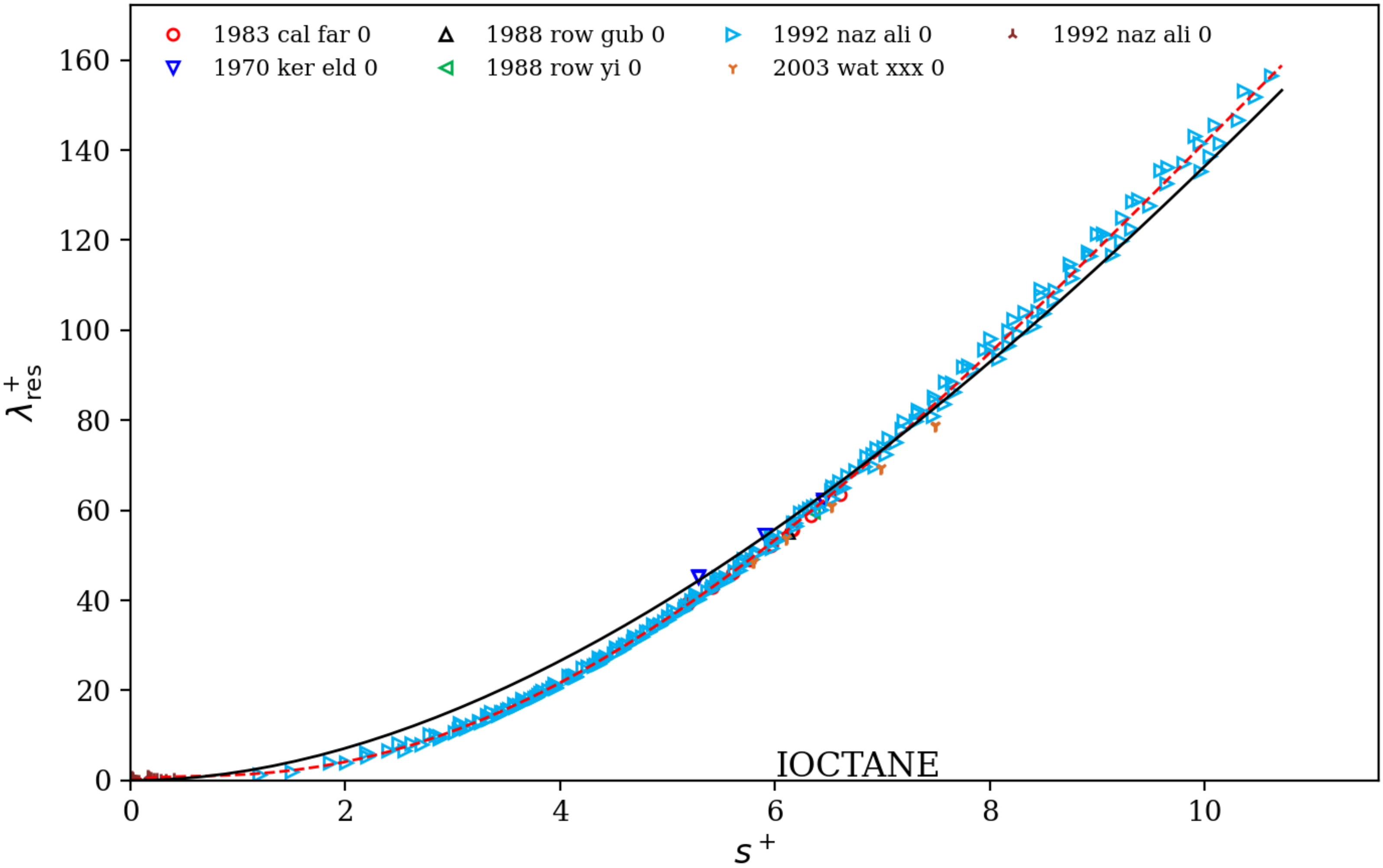

Figure DPR1. IOCTANE

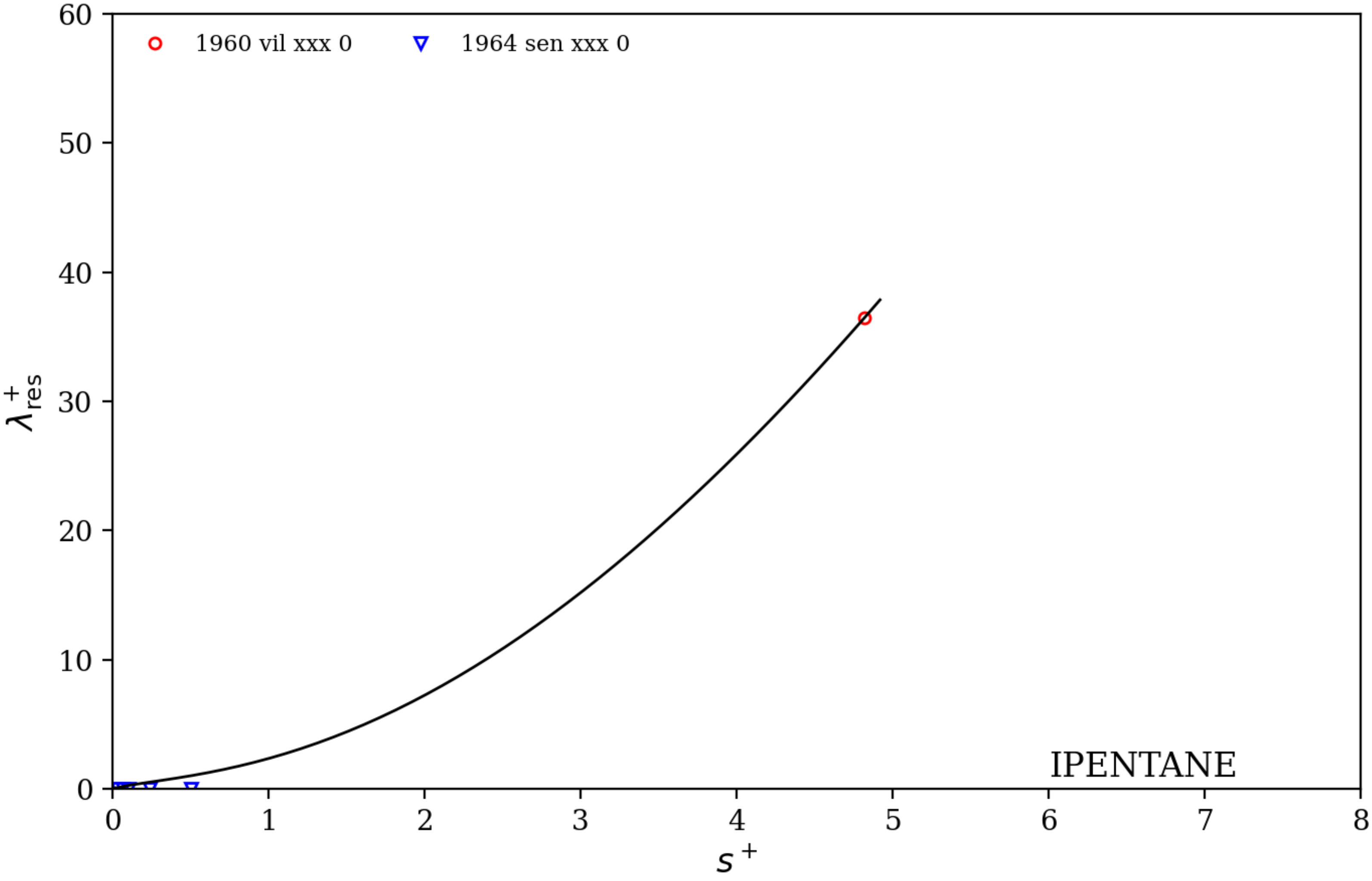

Figure DPR1. IPENTANE

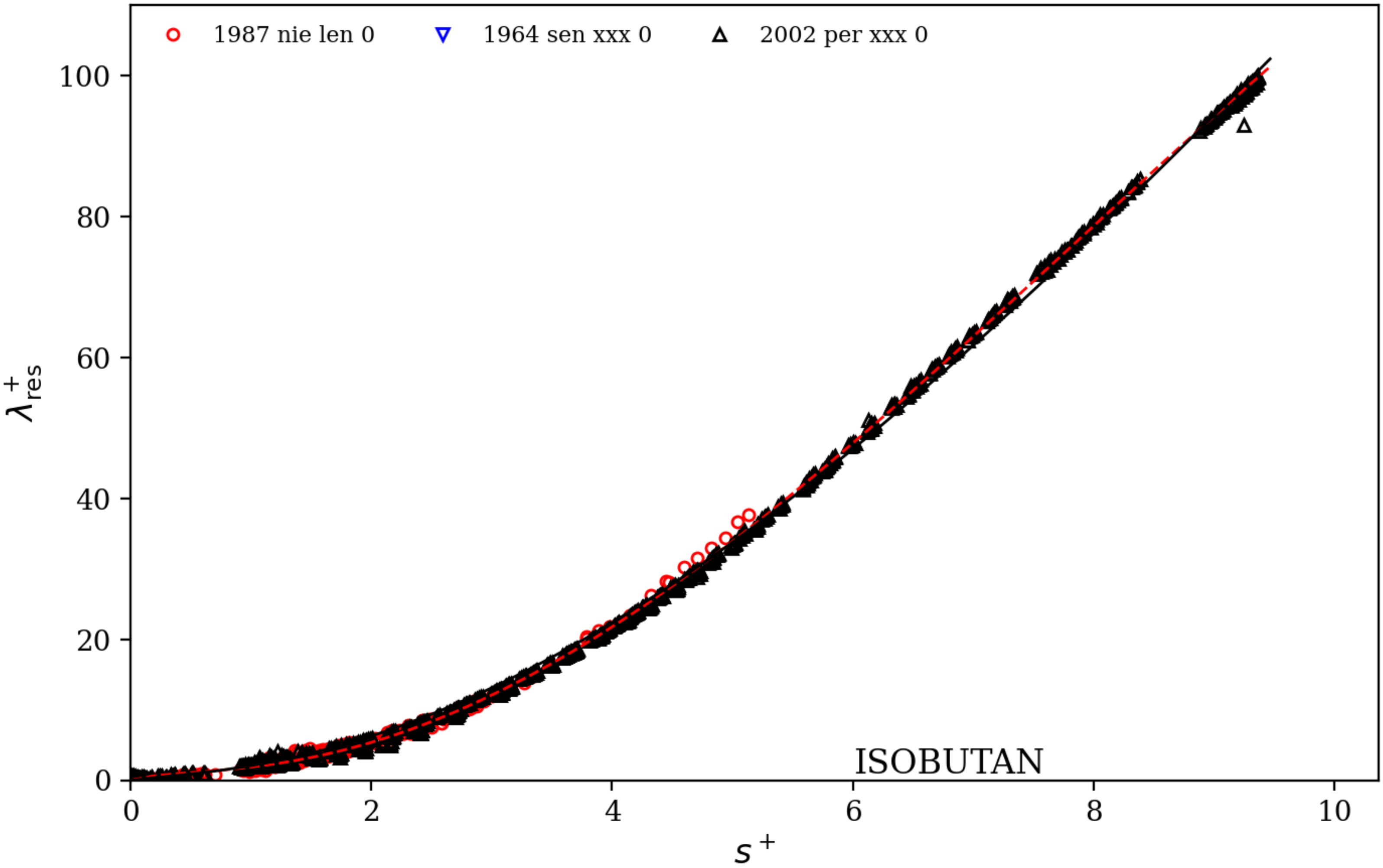

Figure DPR1. ISOBUTAN

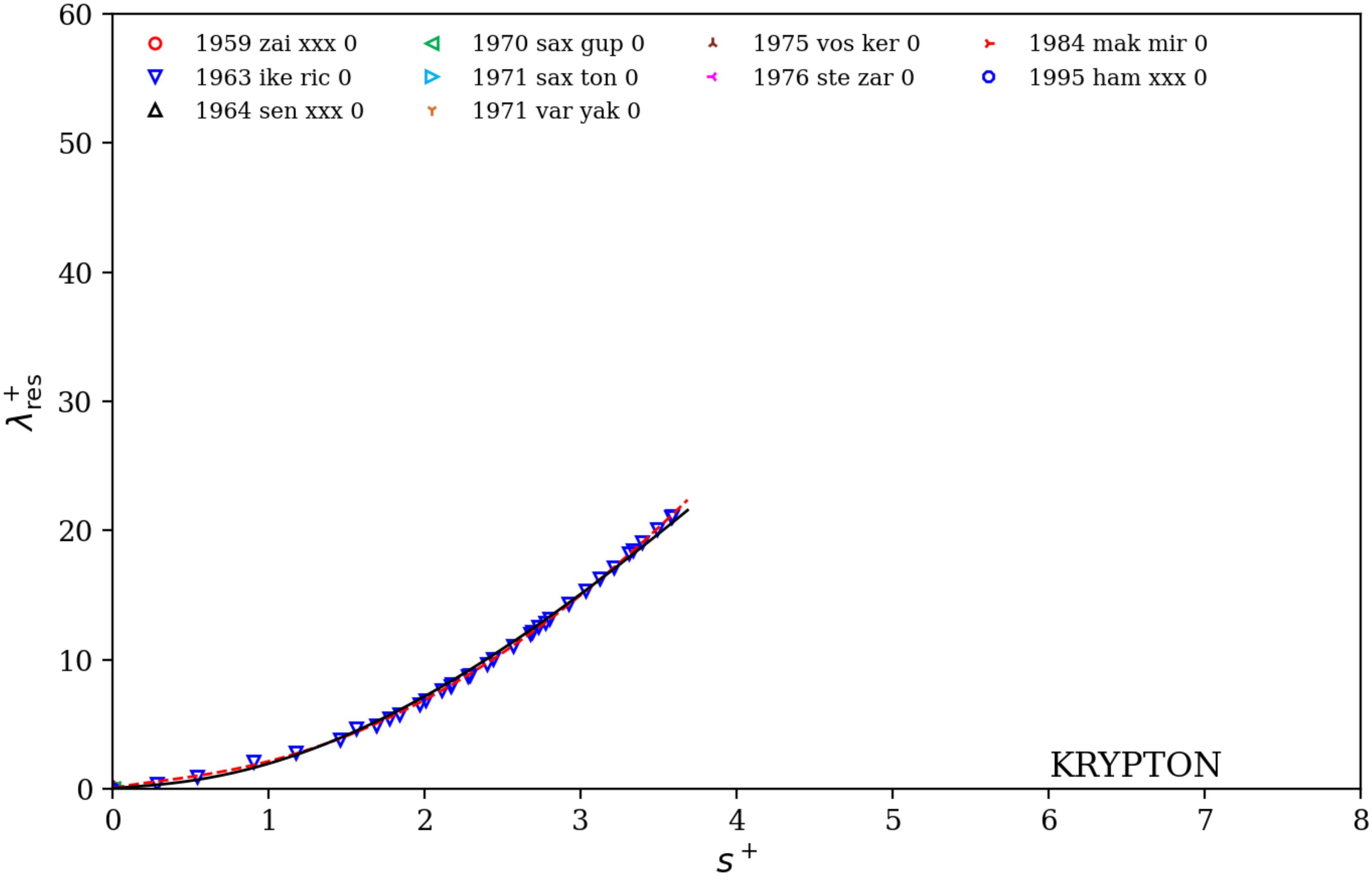

Figure DPR1. KRYPTON

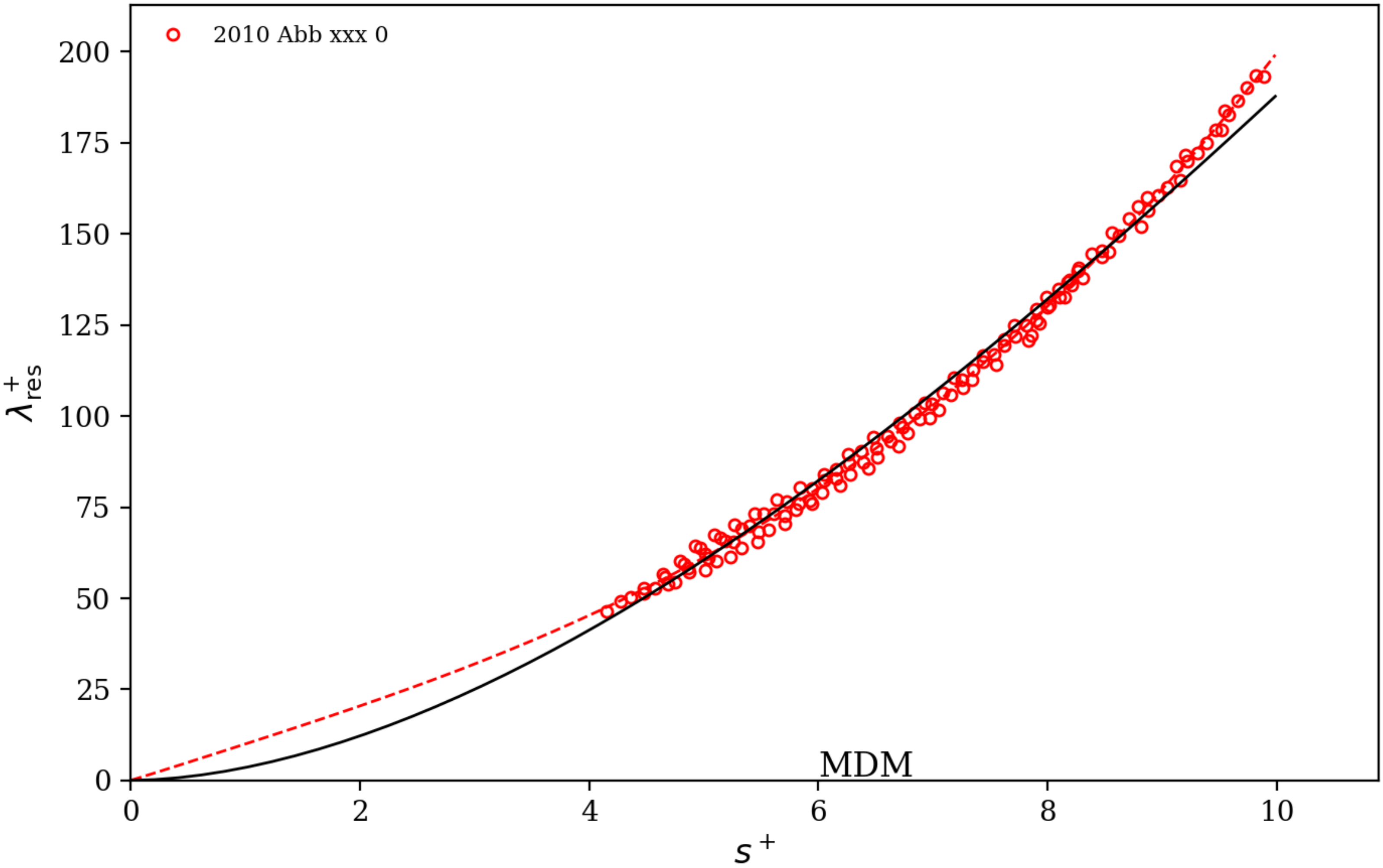

Figure DPR1. MDM

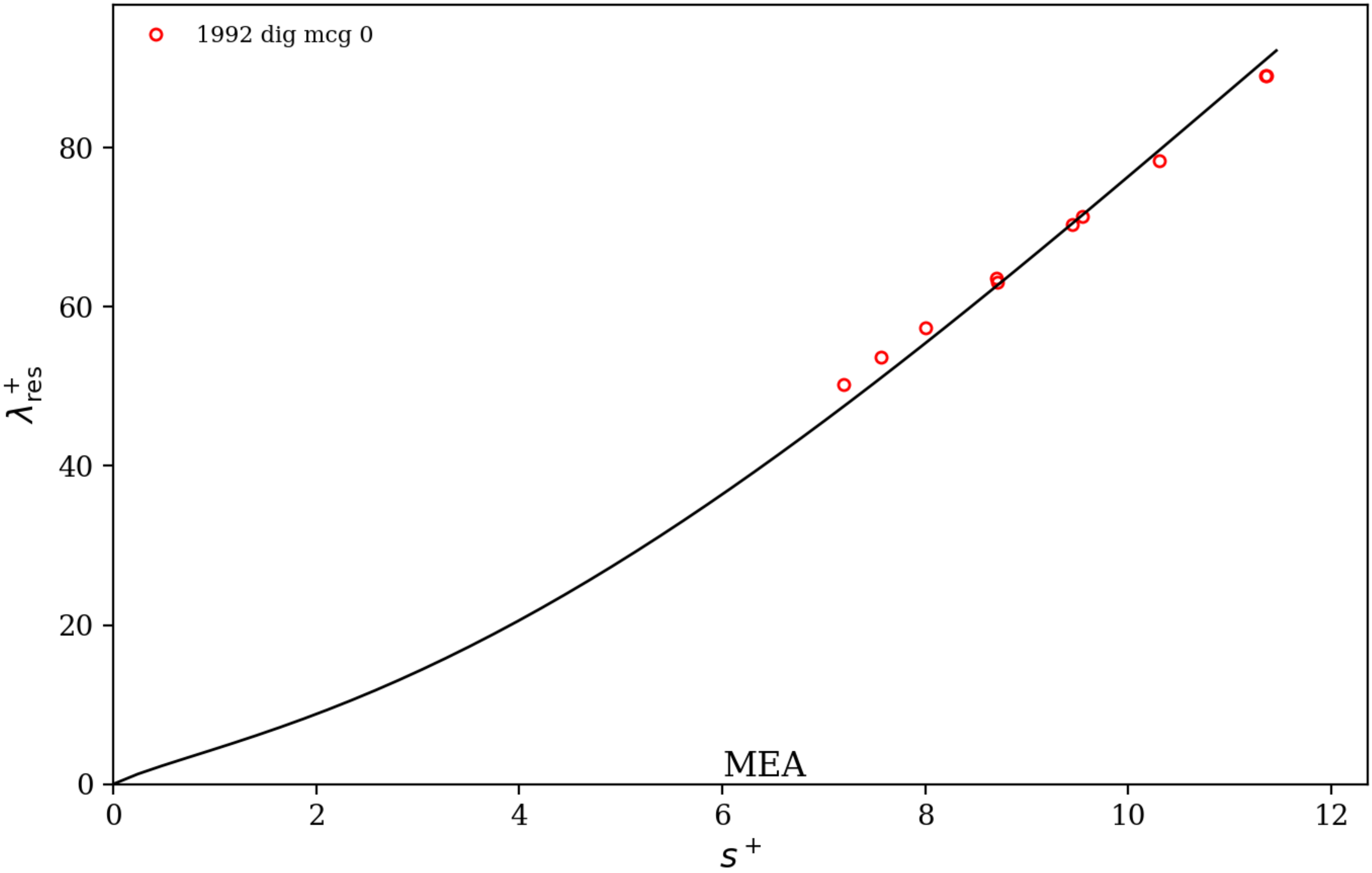

Figure DPR1. MEA

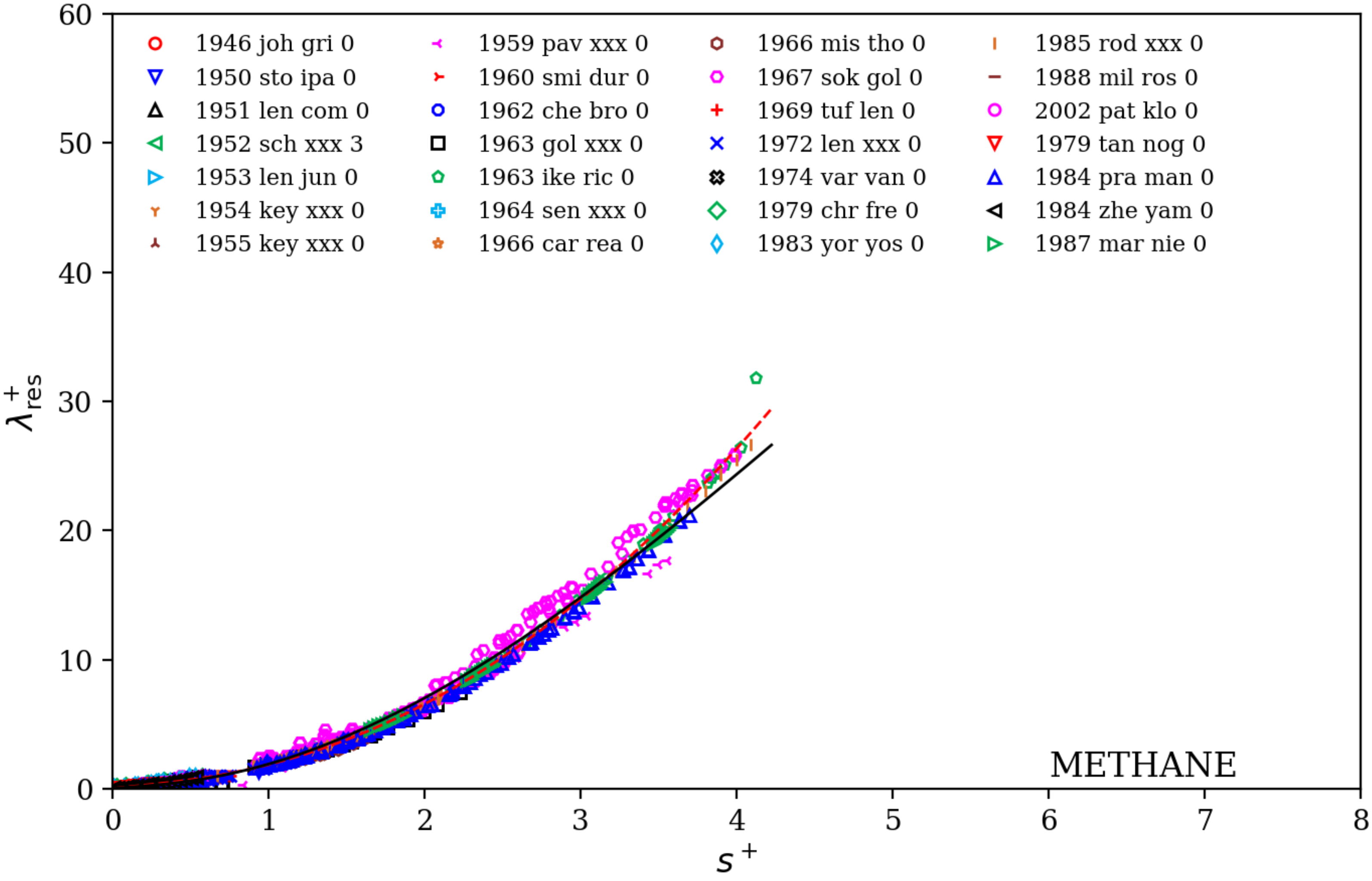

Figure DPR1. METHANE

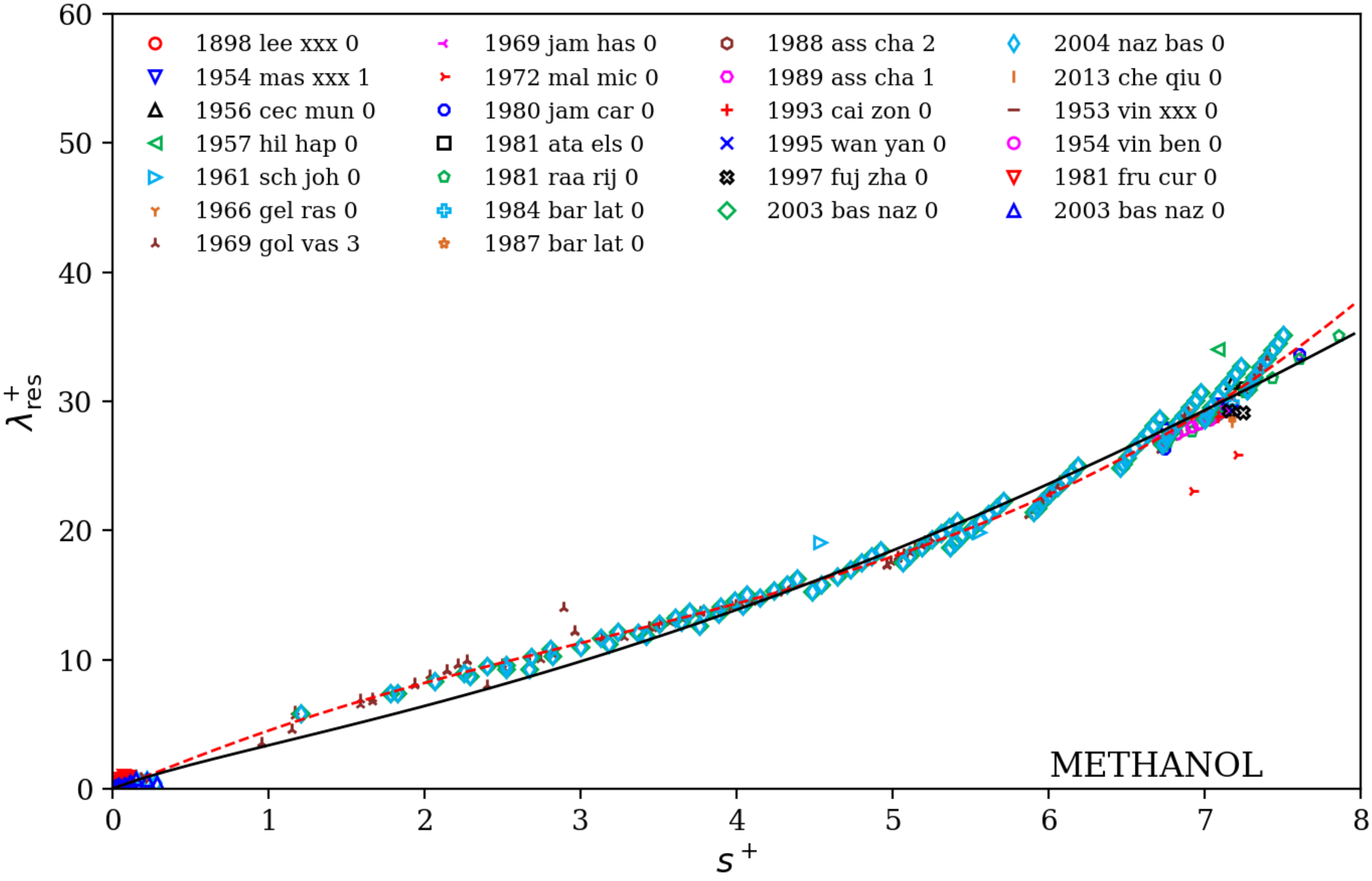

Figure DPR1. METHANOL

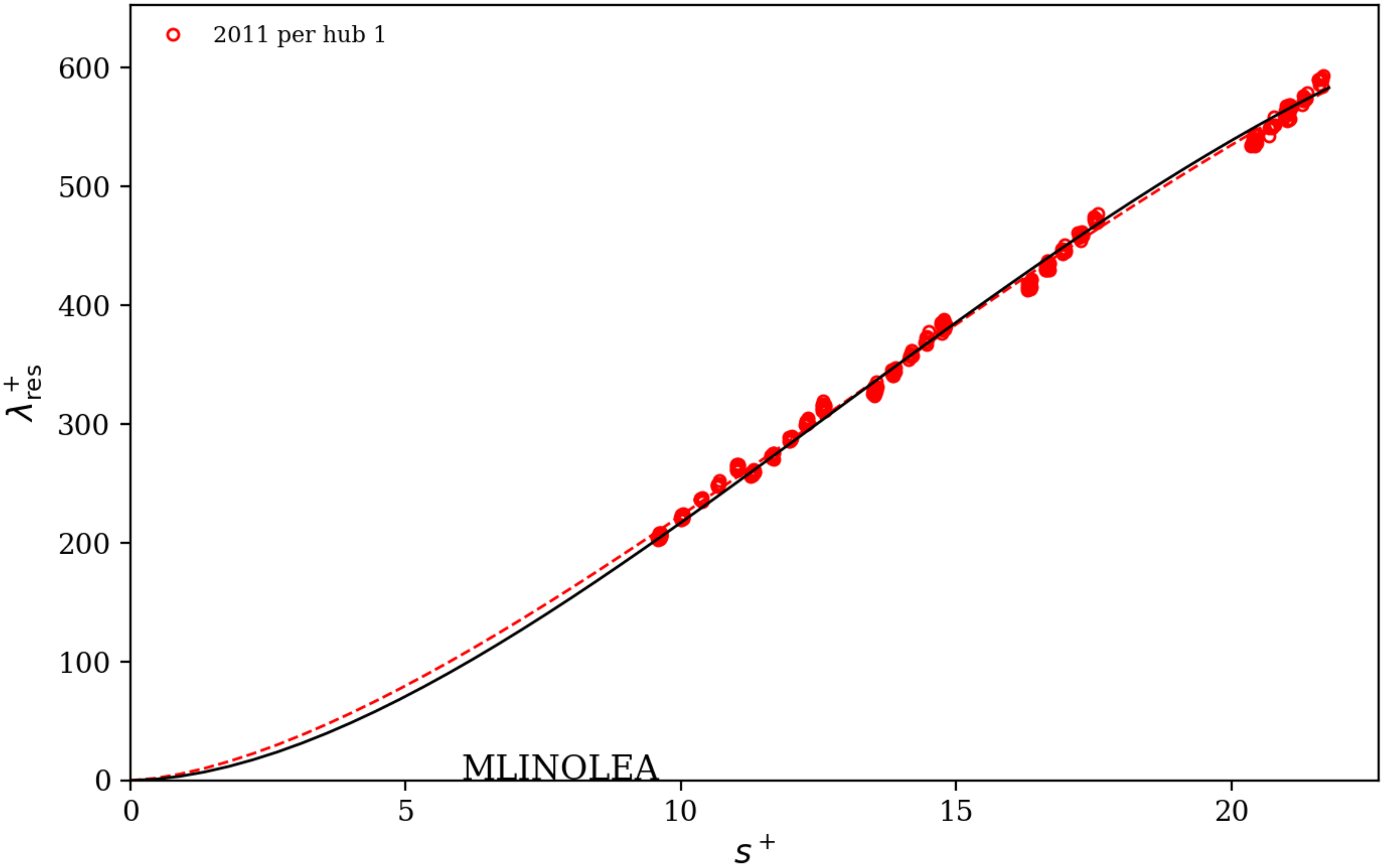

Figure DPR1. MLINOLEA

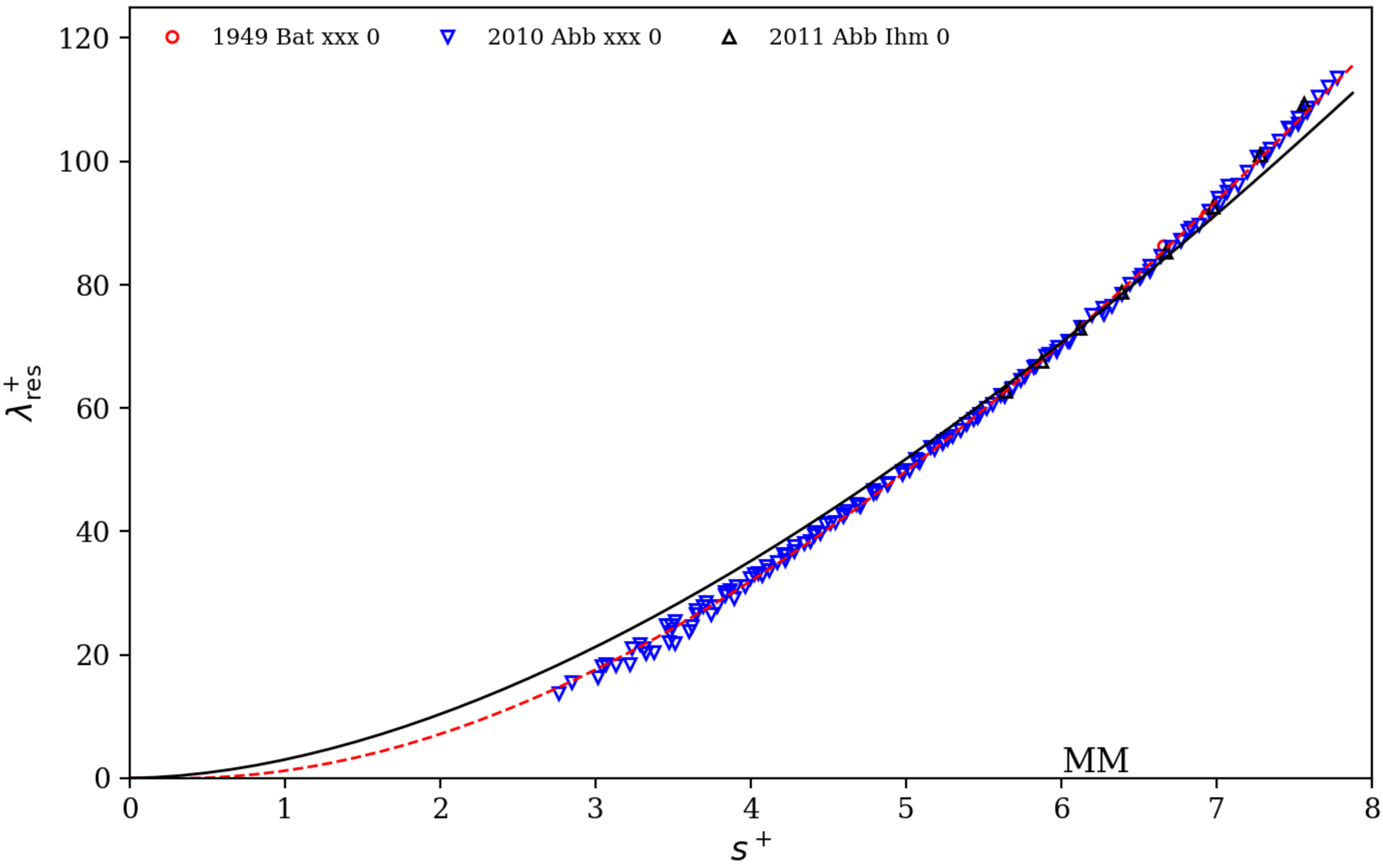

Figure DPR1. MM

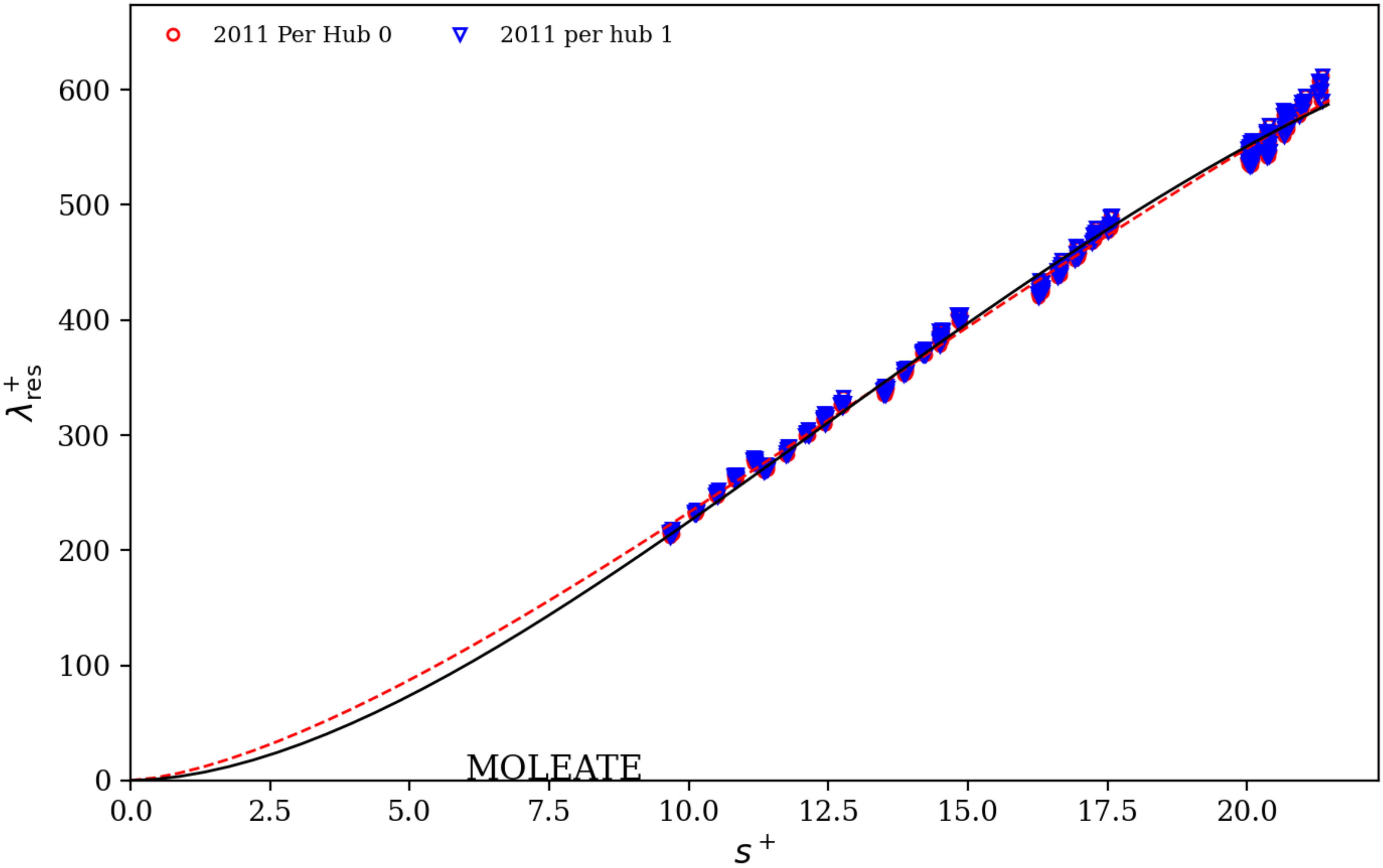

Figure DPR1. MOLEATE

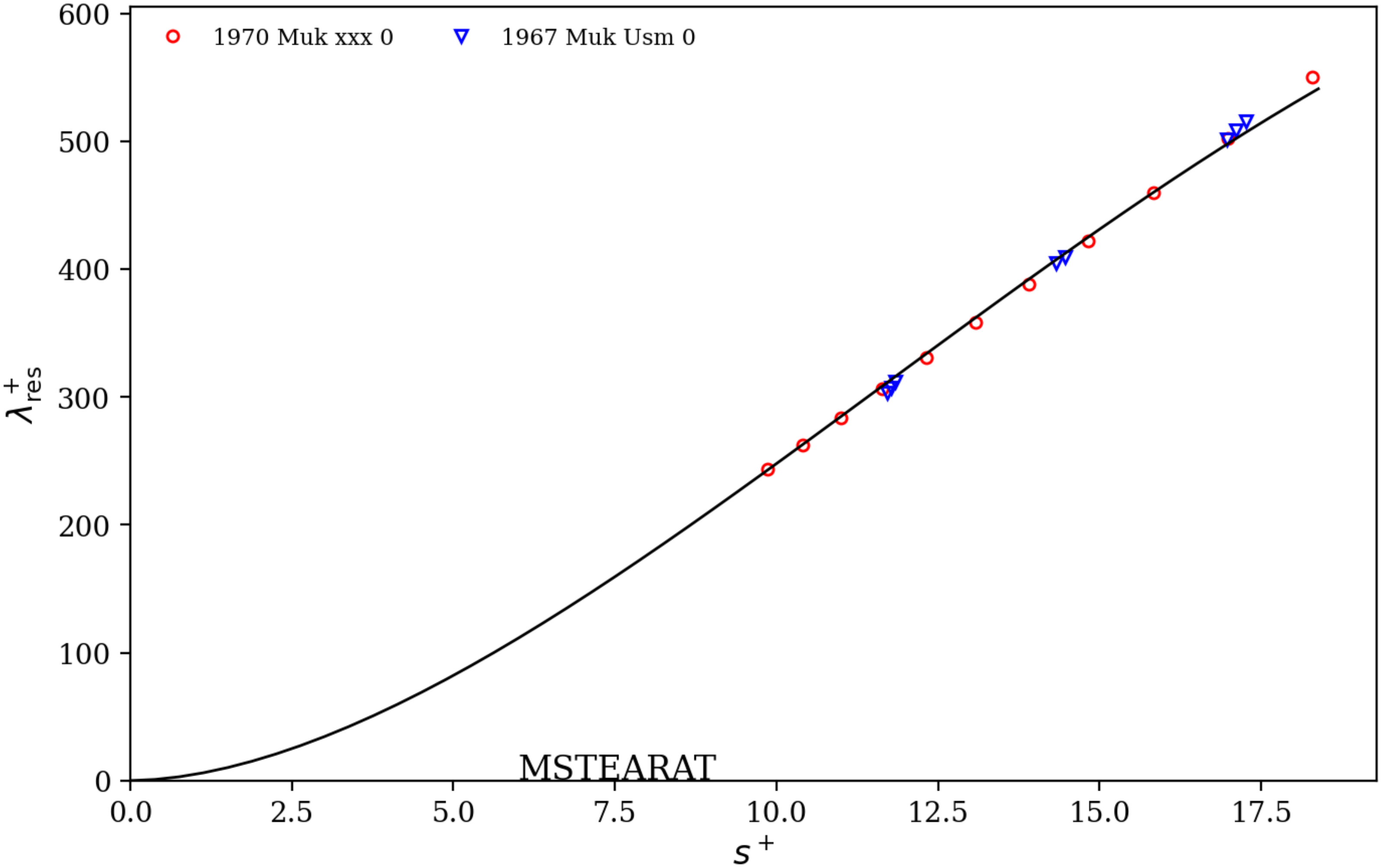

Figure DPR1. MSTEARAT

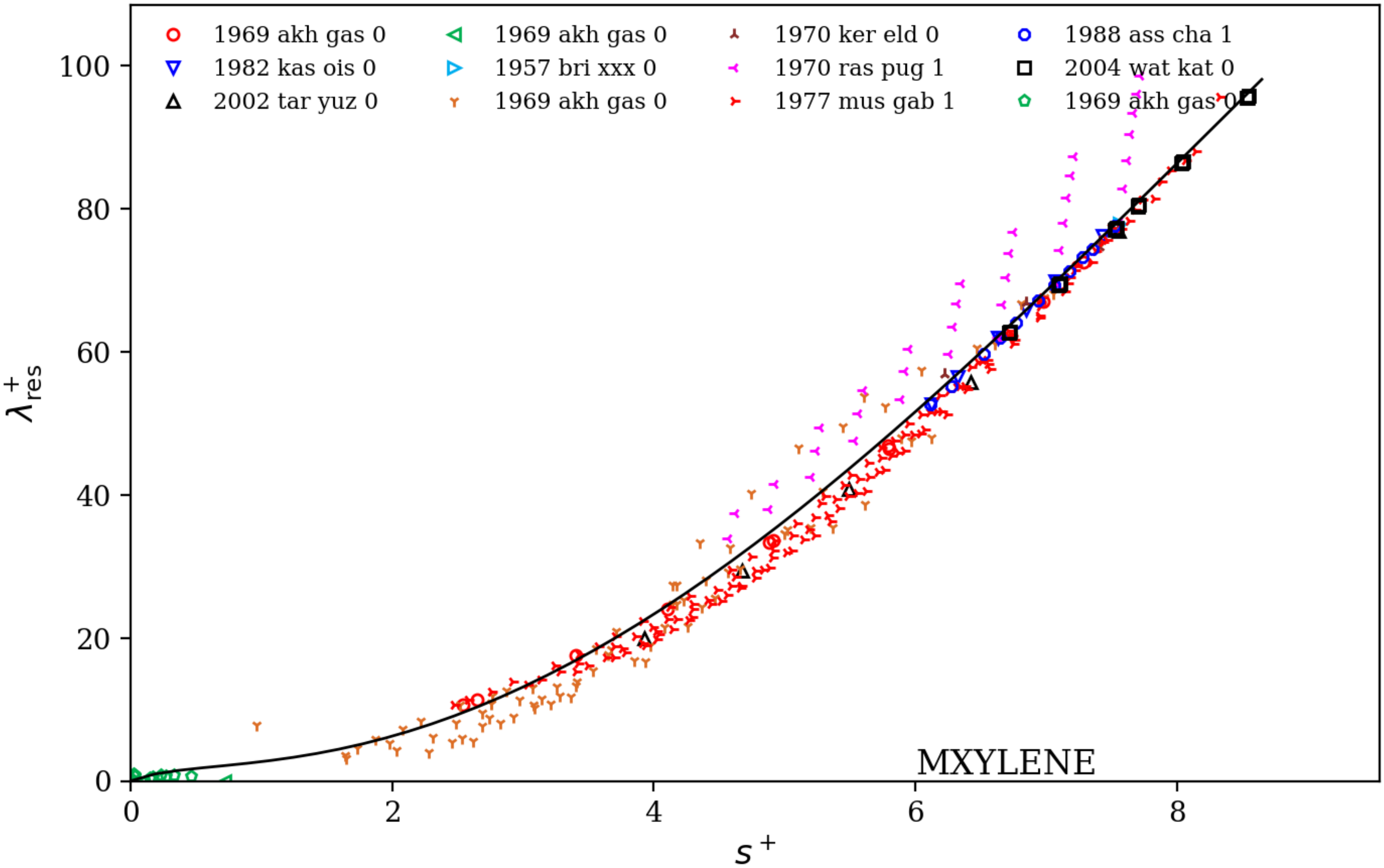

Figure DPR1. MXYLENE

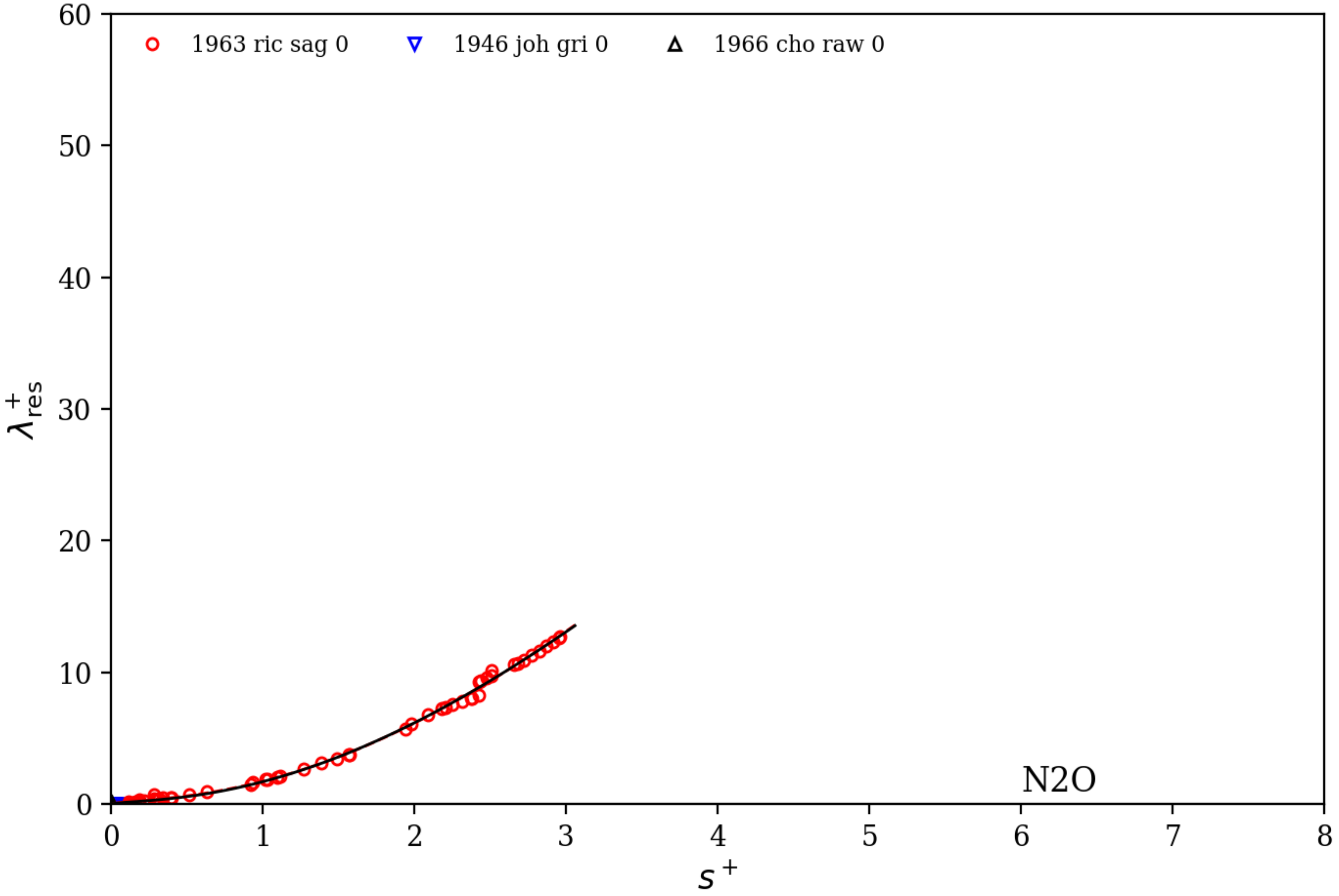

Figure DPR1. N2O

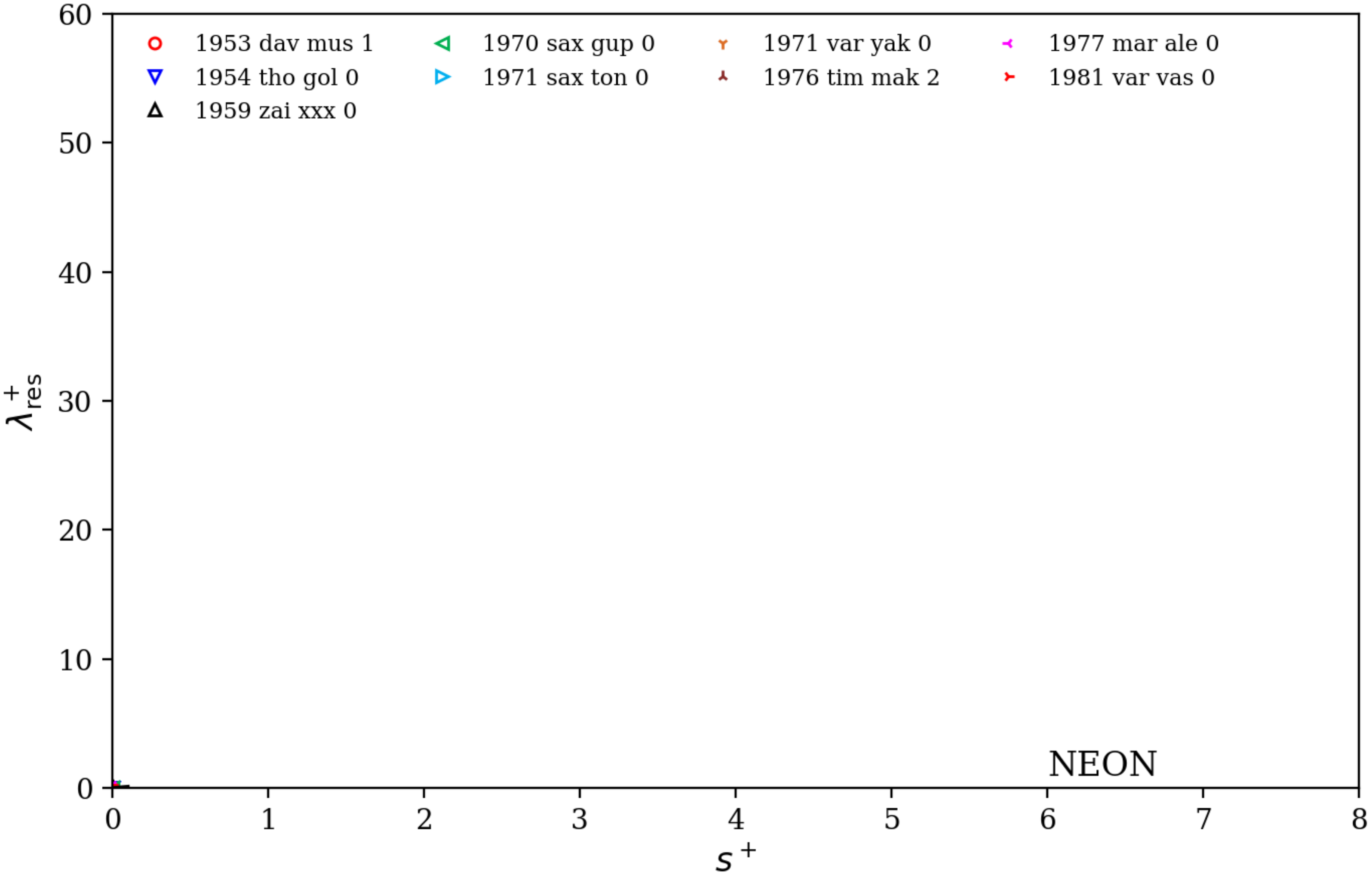

Figure DPR1. NEON

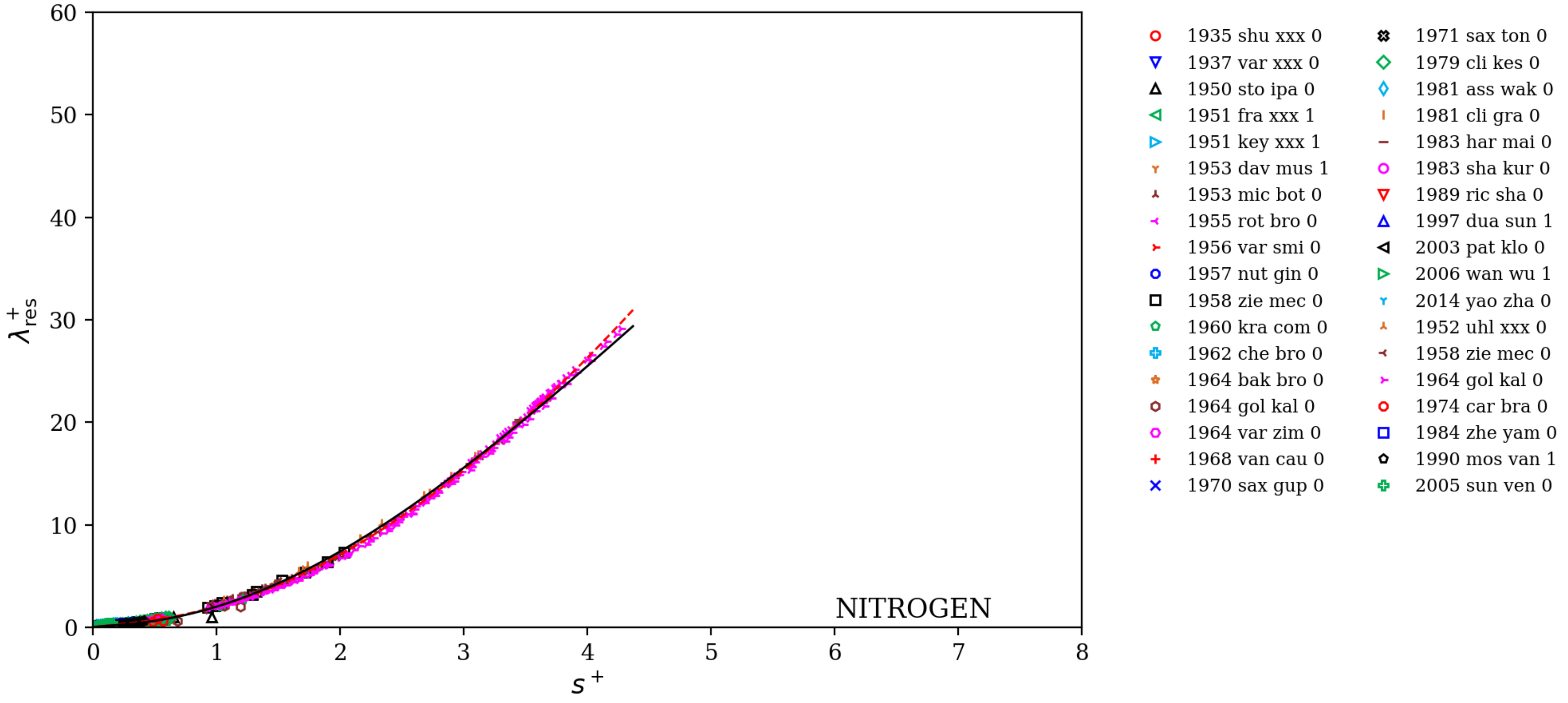

Figure DPR1. NITROGEN

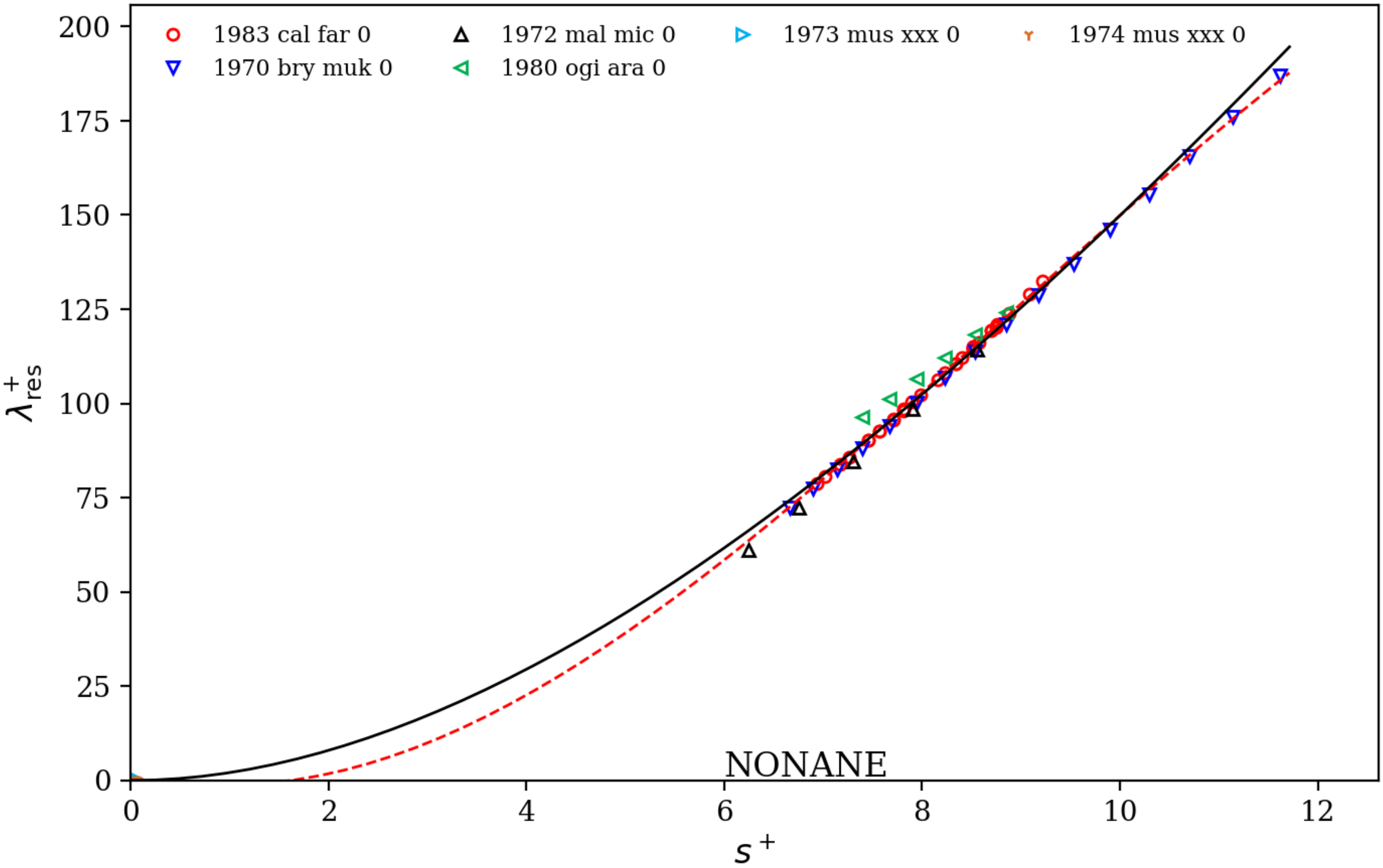

Figure DPR1. NONANE

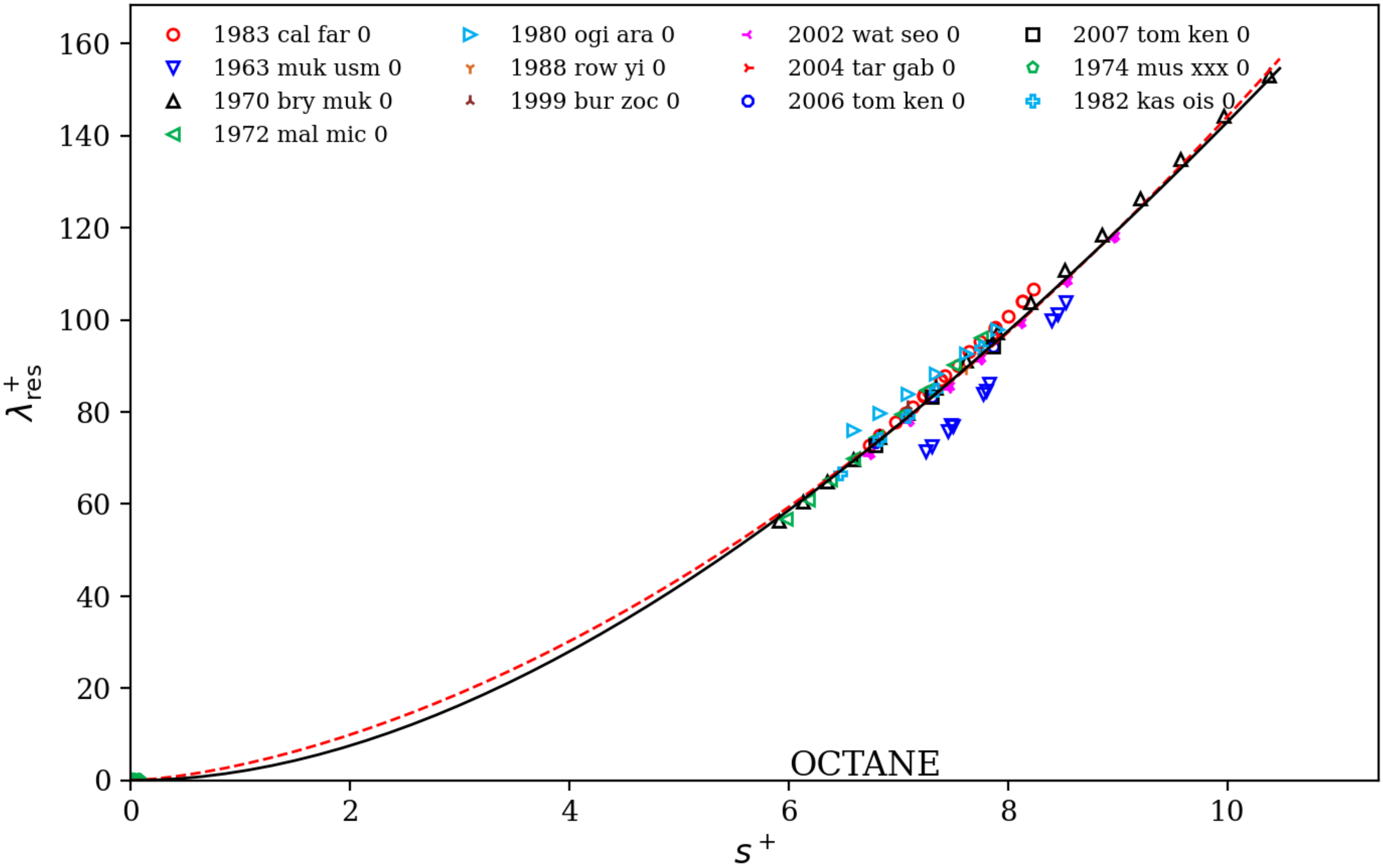

Figure DPR1. OCTANE

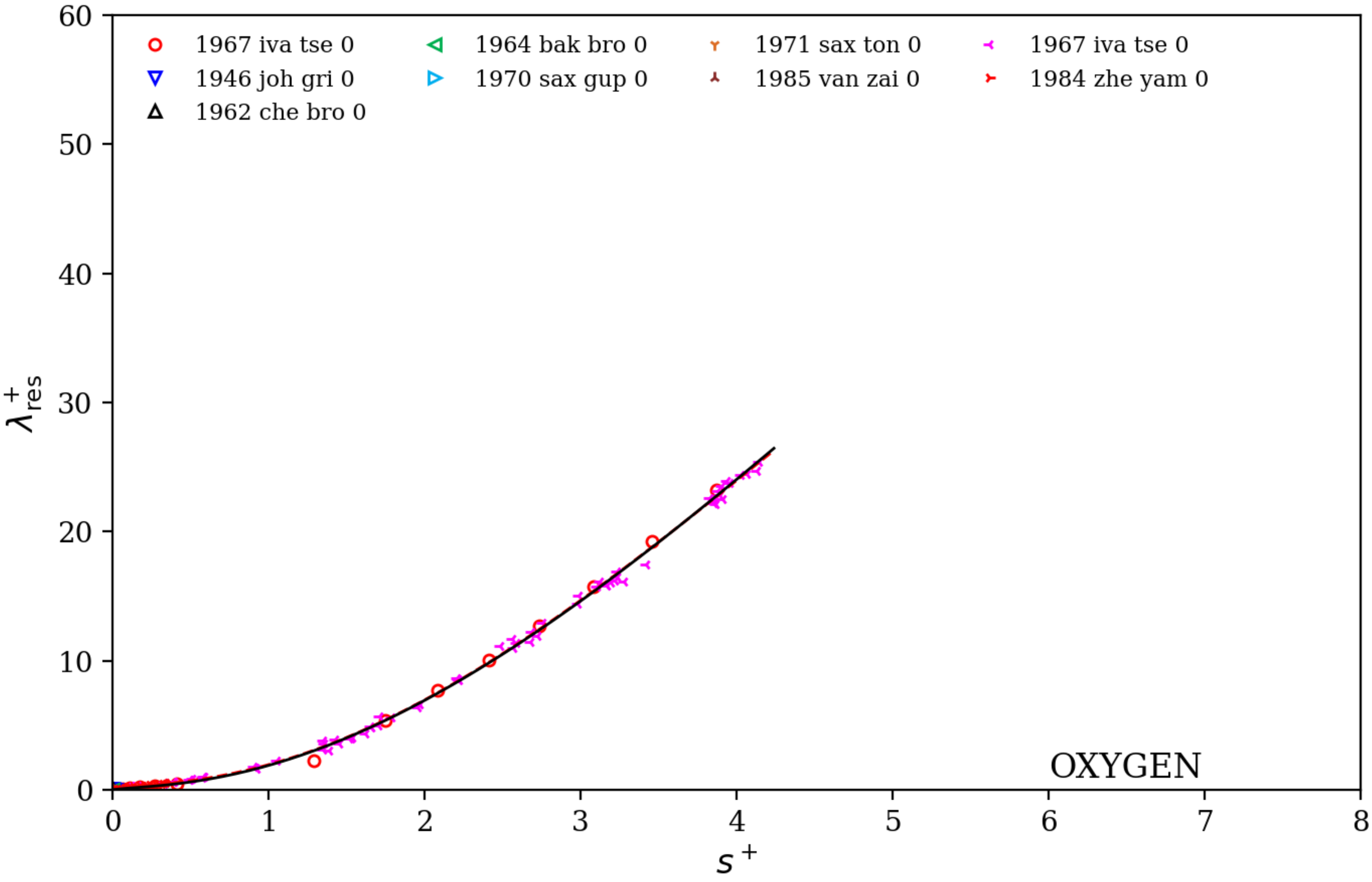

Figure DPR1. OXYGEN

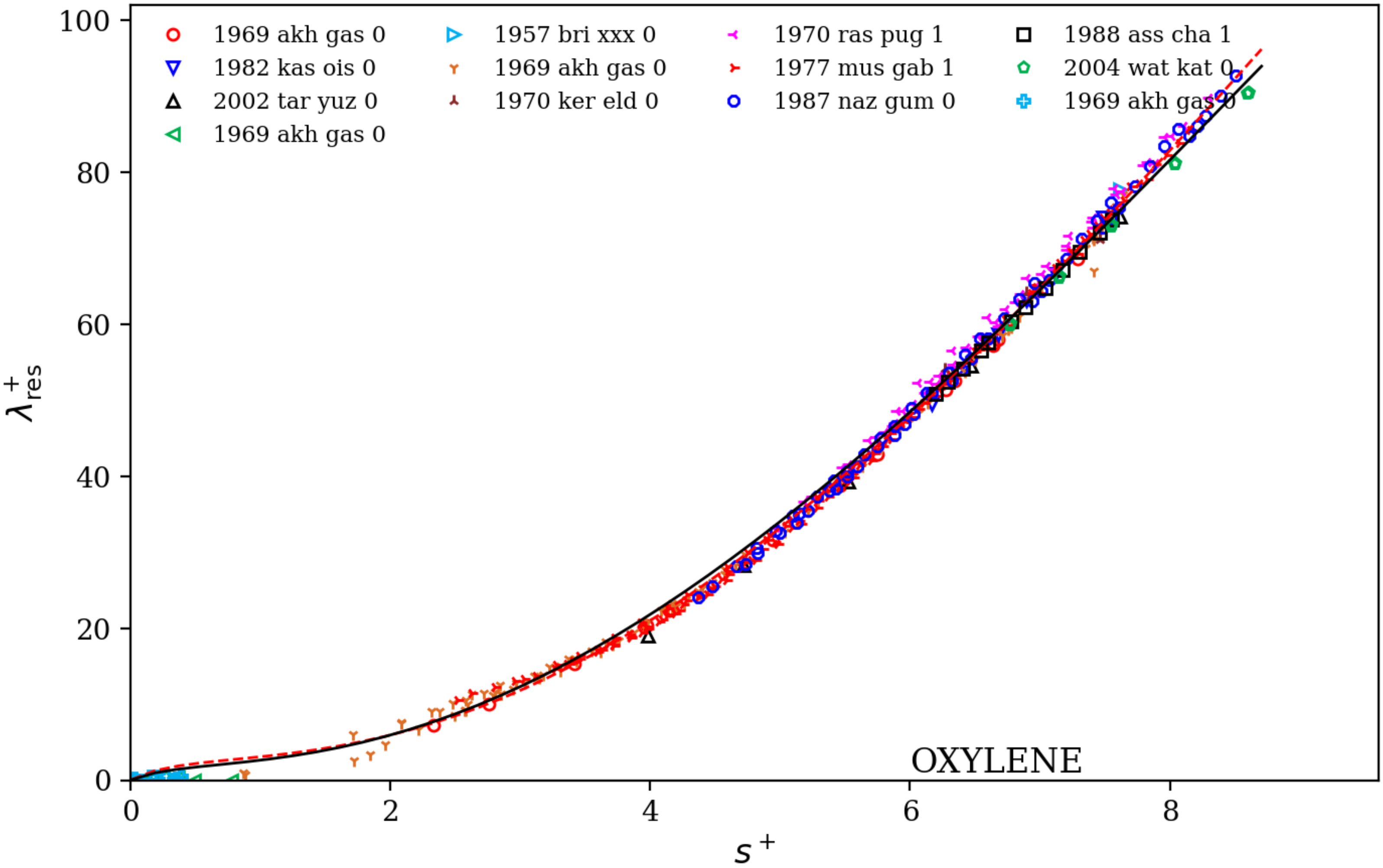

Figure DPR1. OXYLENE

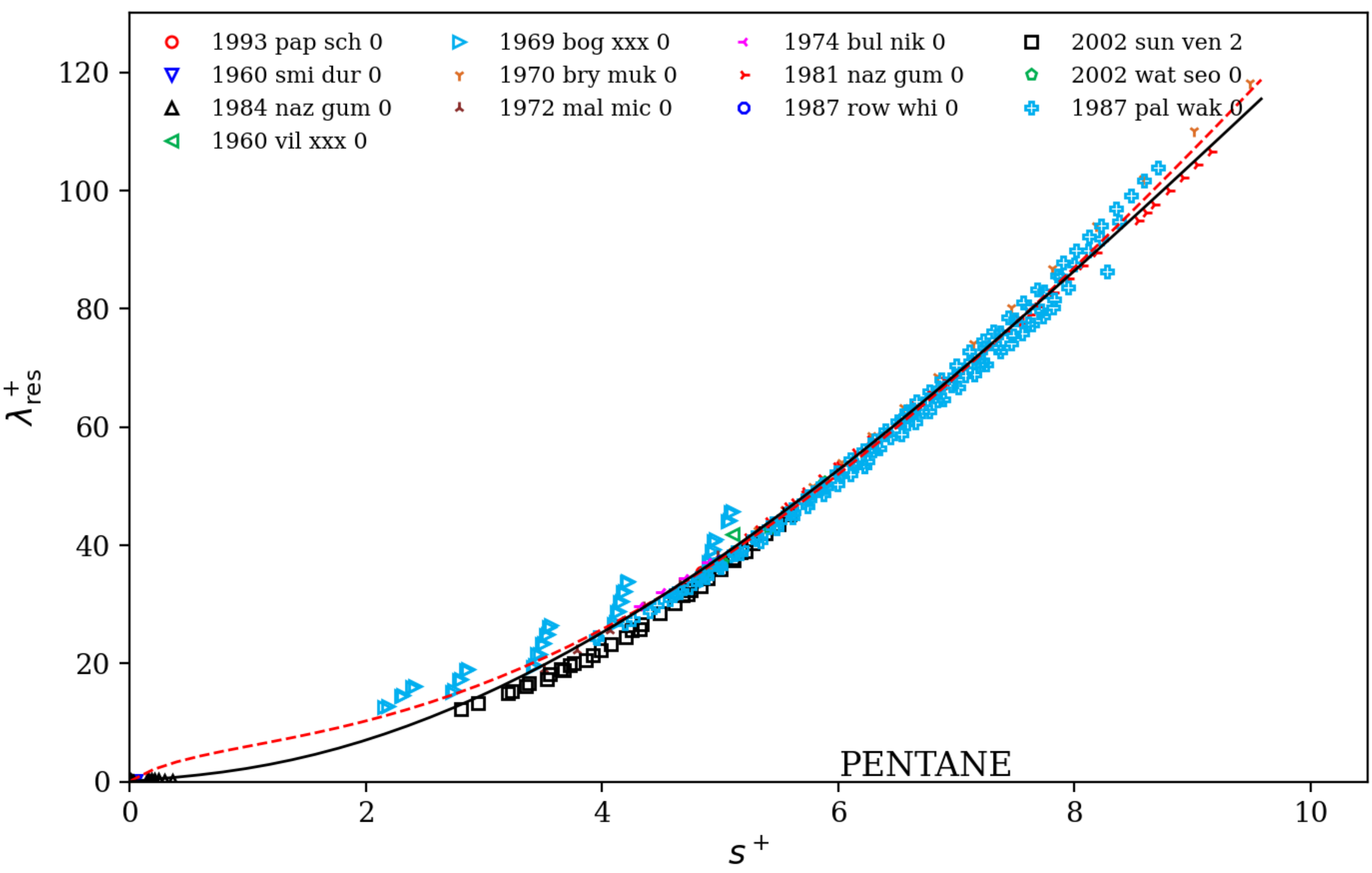

Figure DPR1. PENTANE

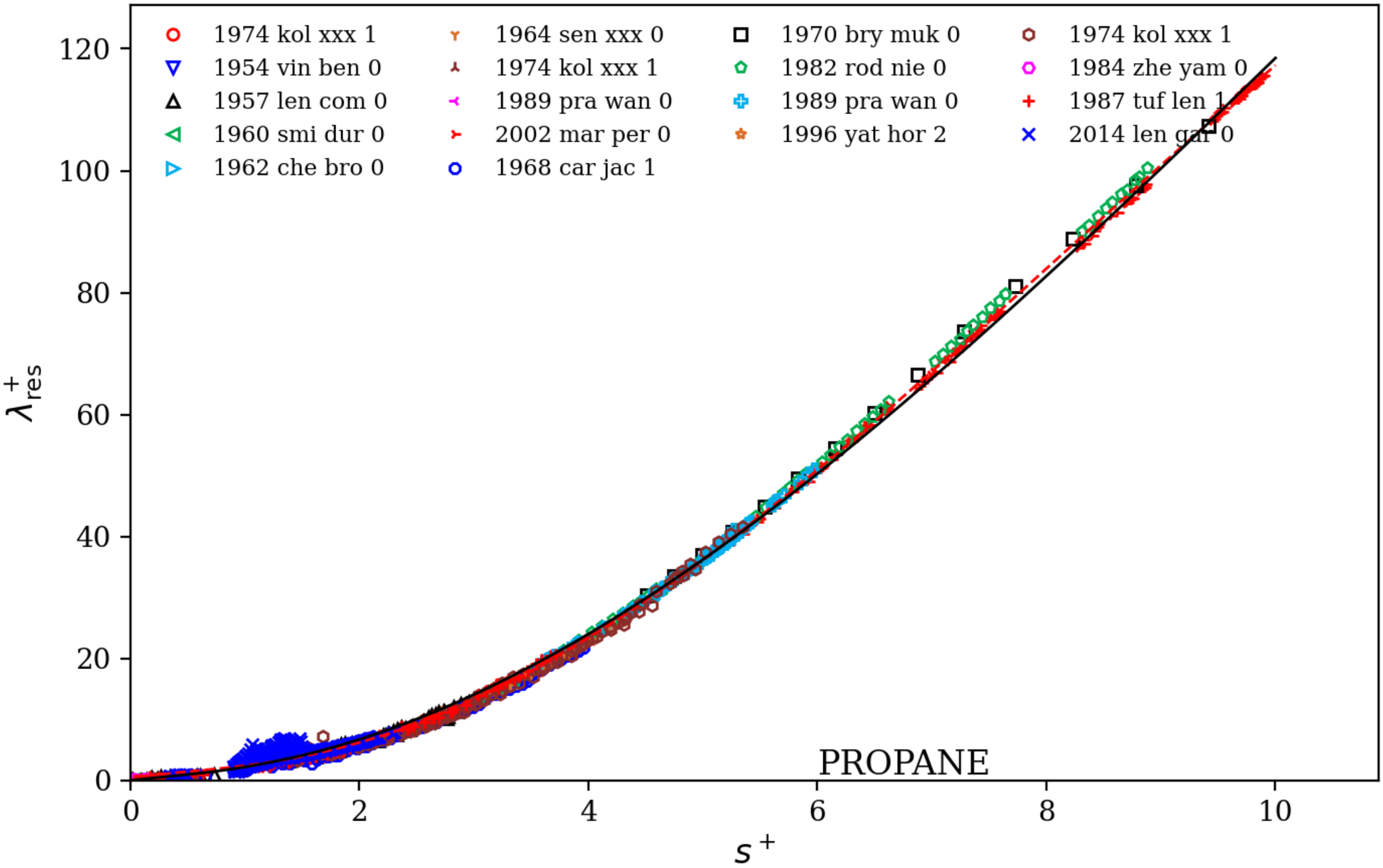

Figure DPR1. PROPANE

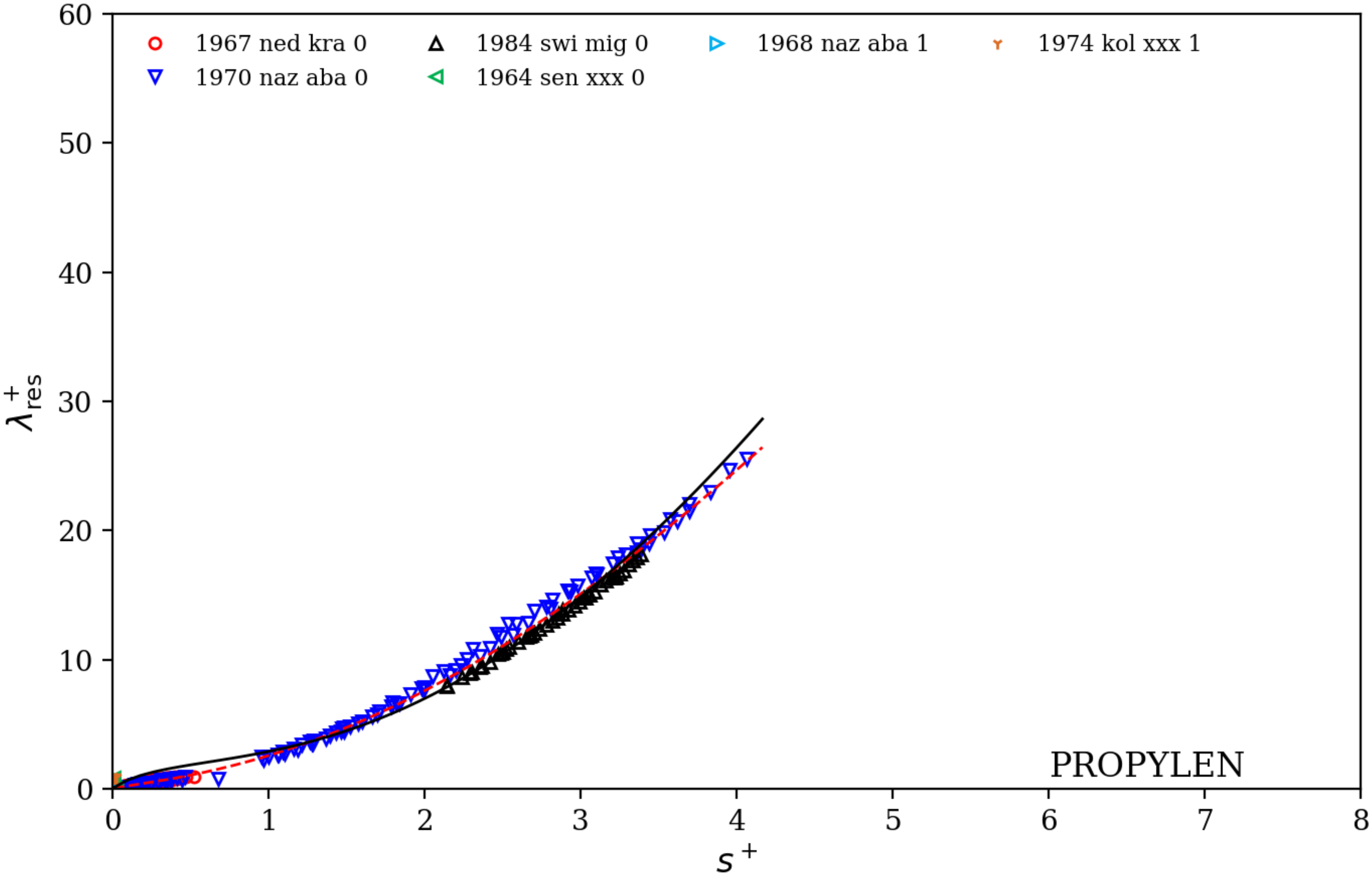

Figure DPR1. PROPYLEN

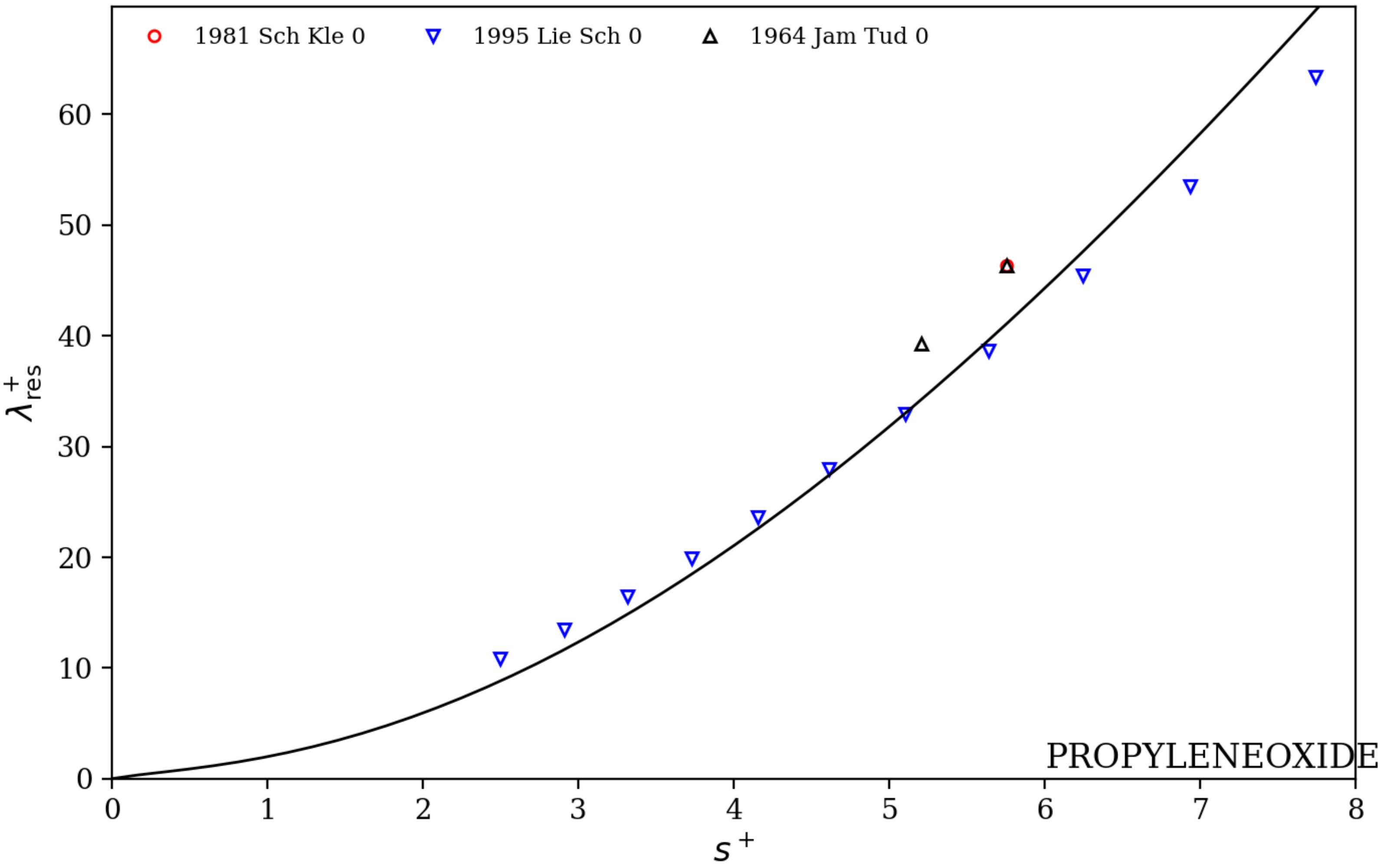

Figure DPR1. PROPYLENEOXIDE

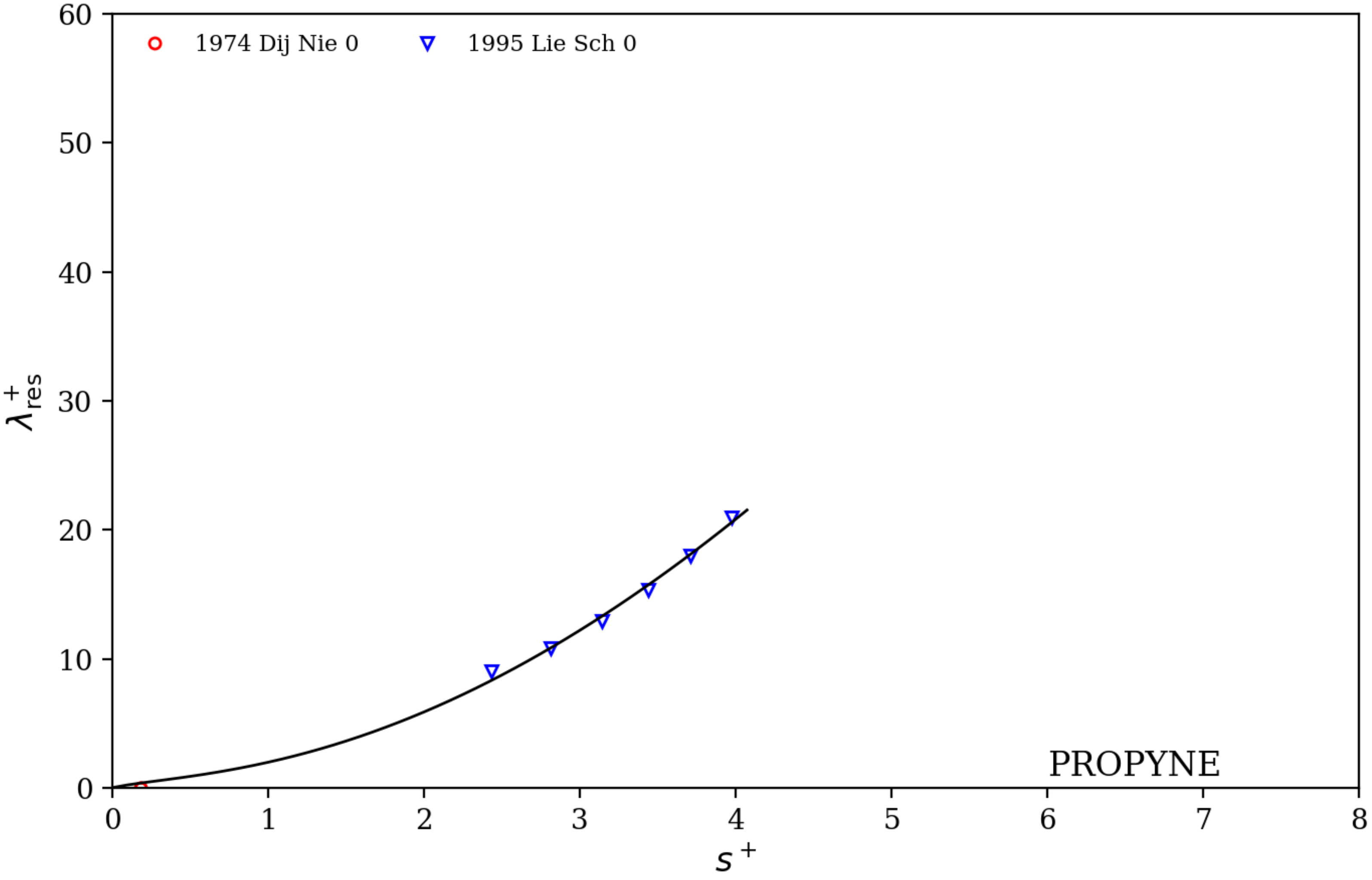

Figure DPR1. PROPYNE

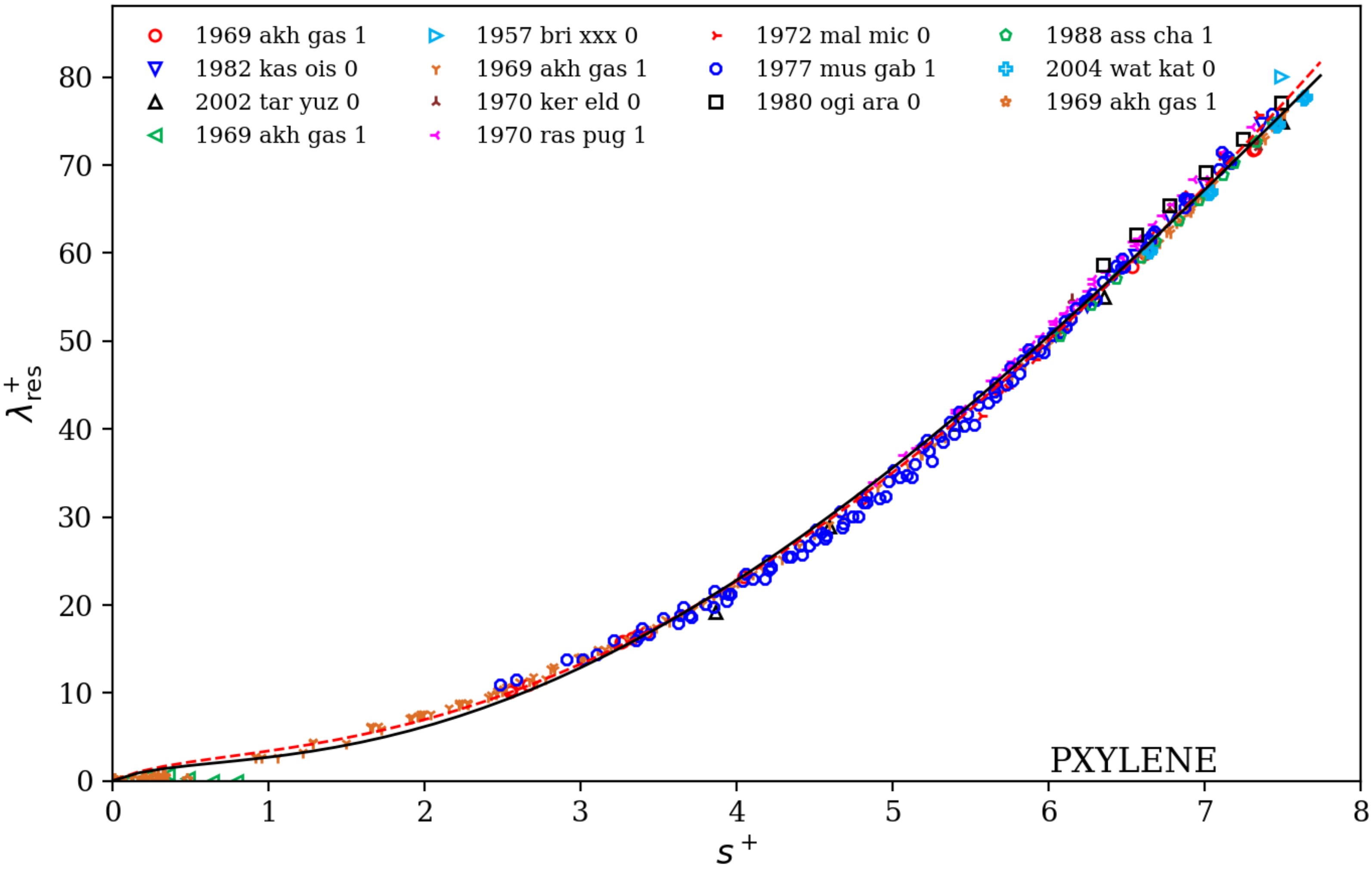

Figure DPR1. PXYLENE

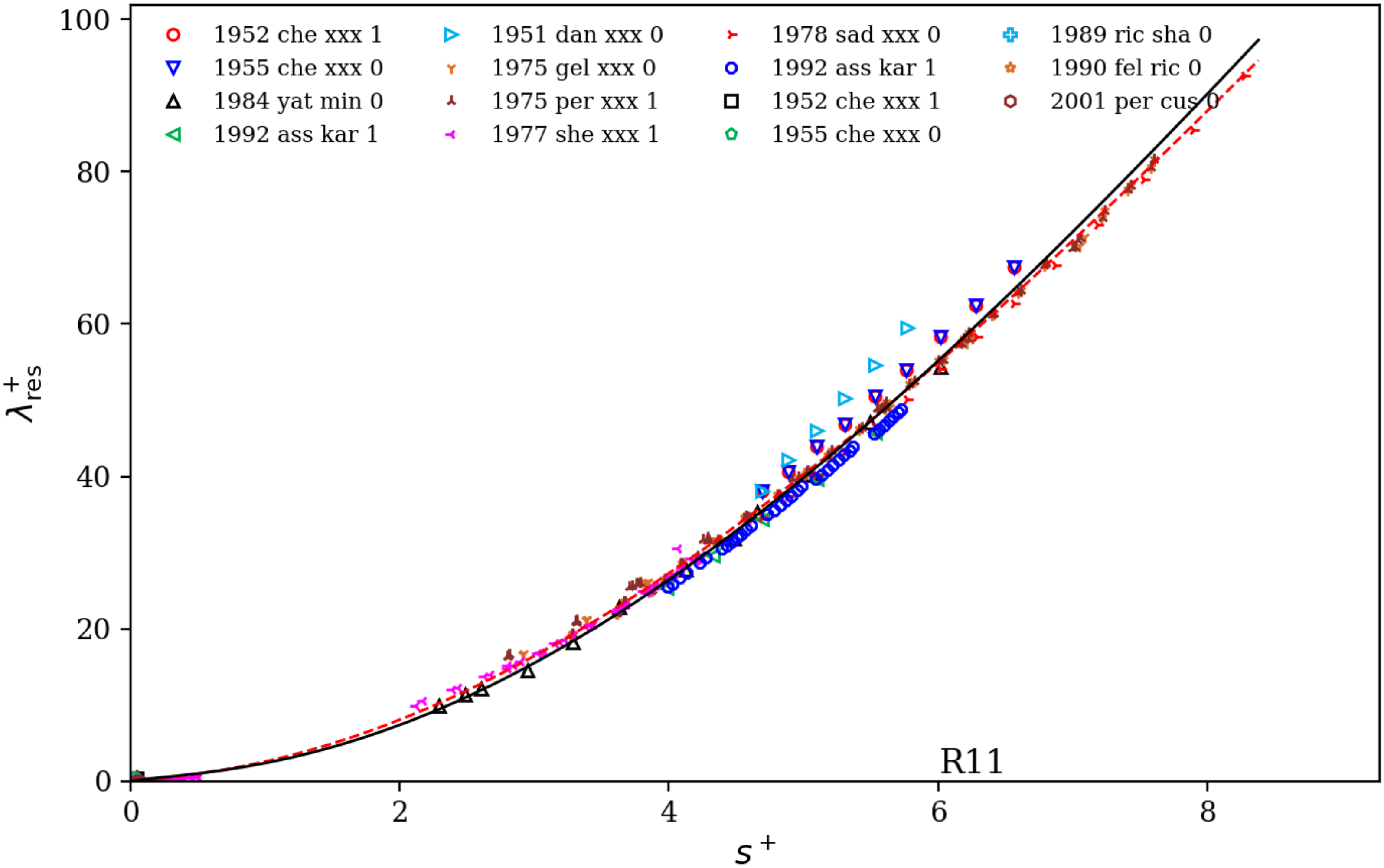

Figure DPR1. R11

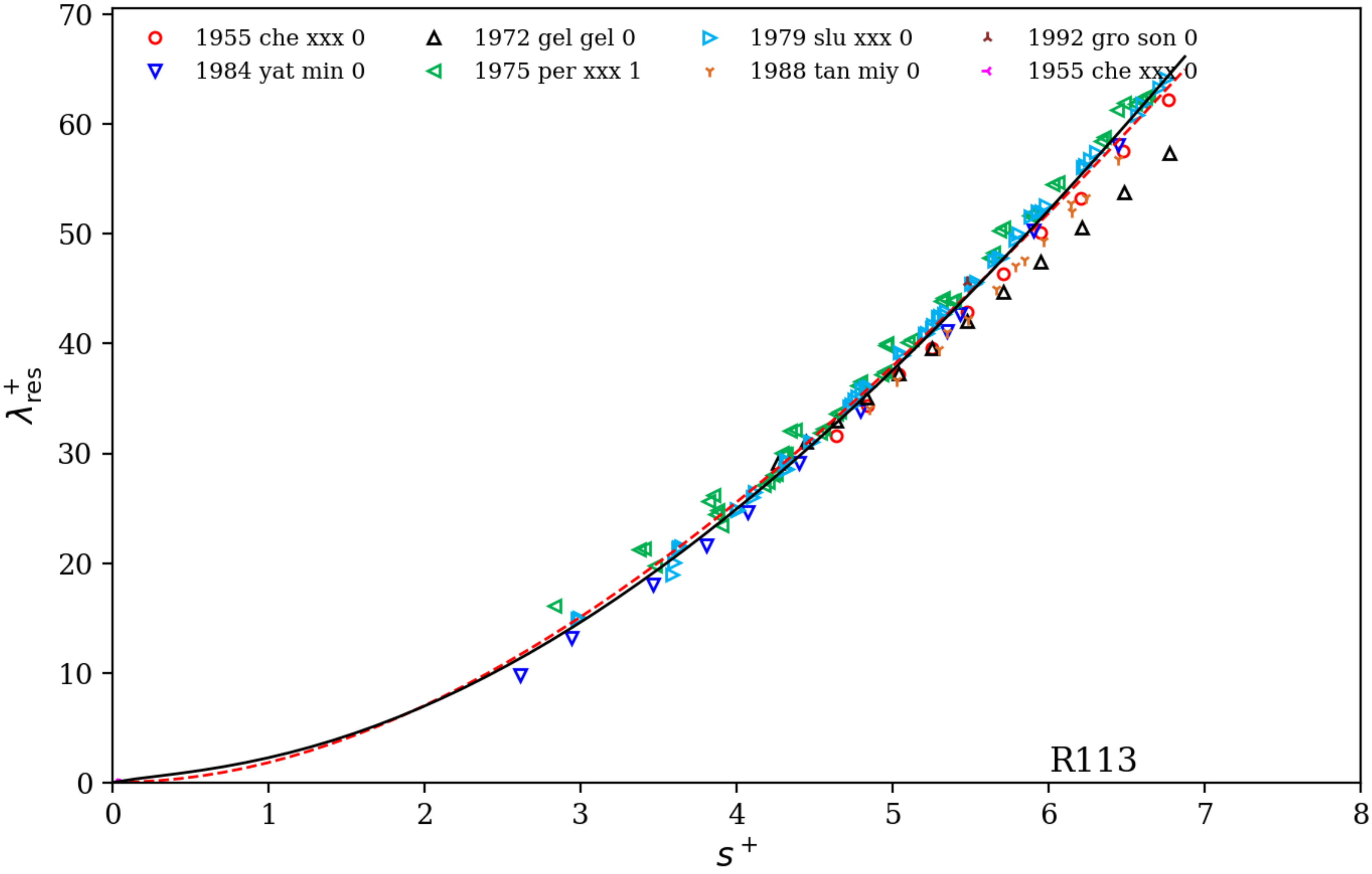

Figure DPR1. R113

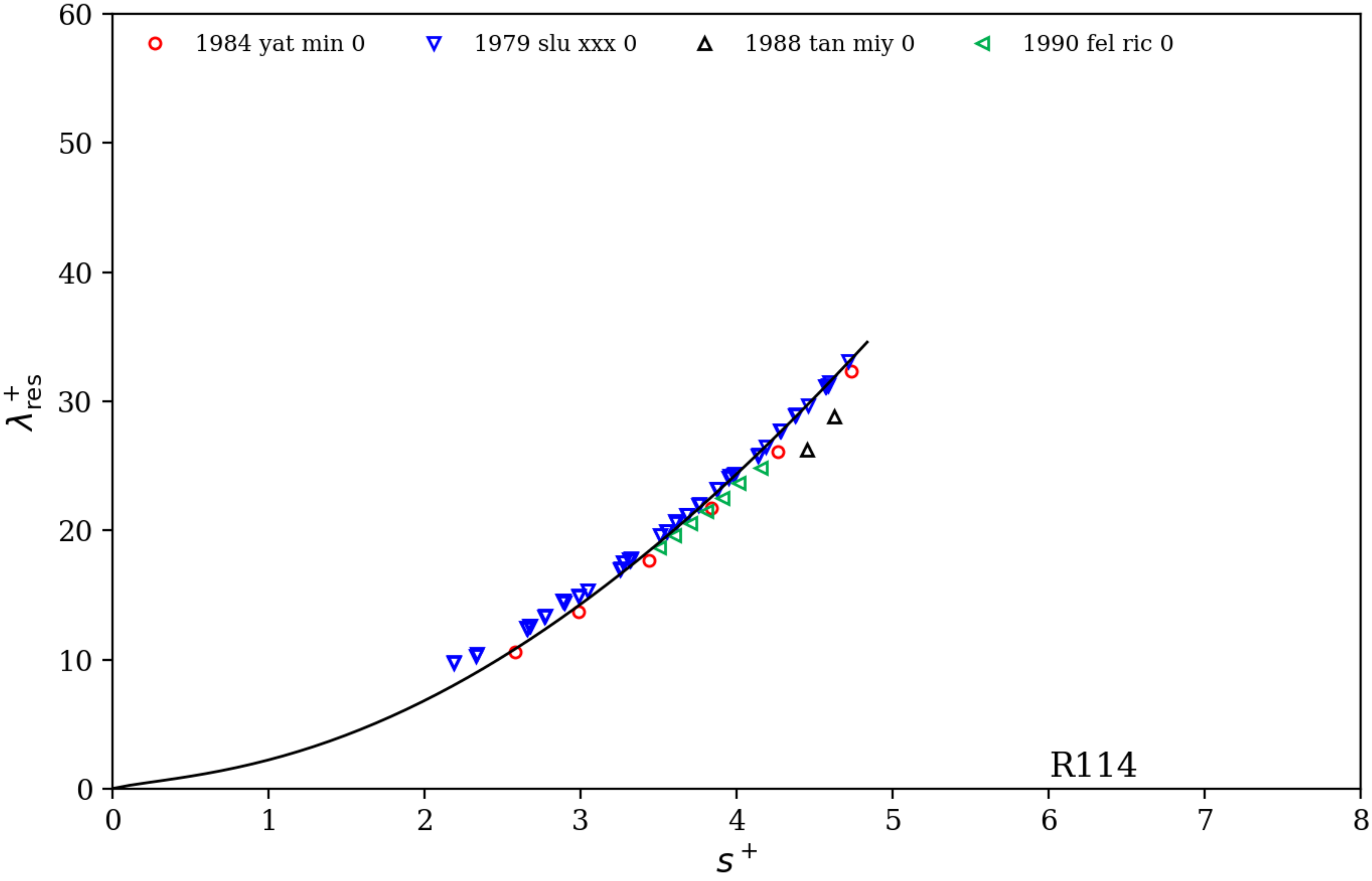

Figure DPR1. R114

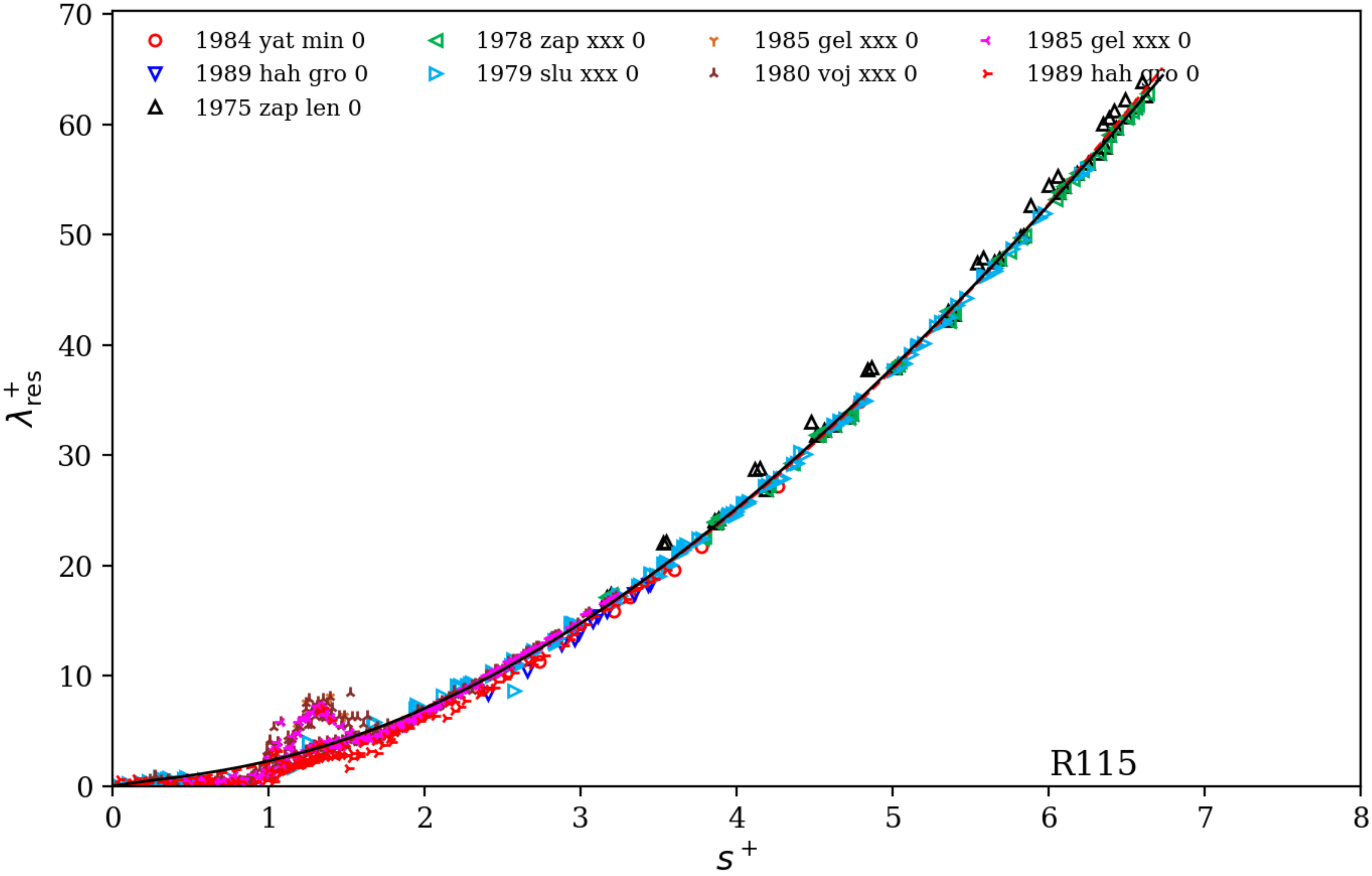

Figure DPR1. R115

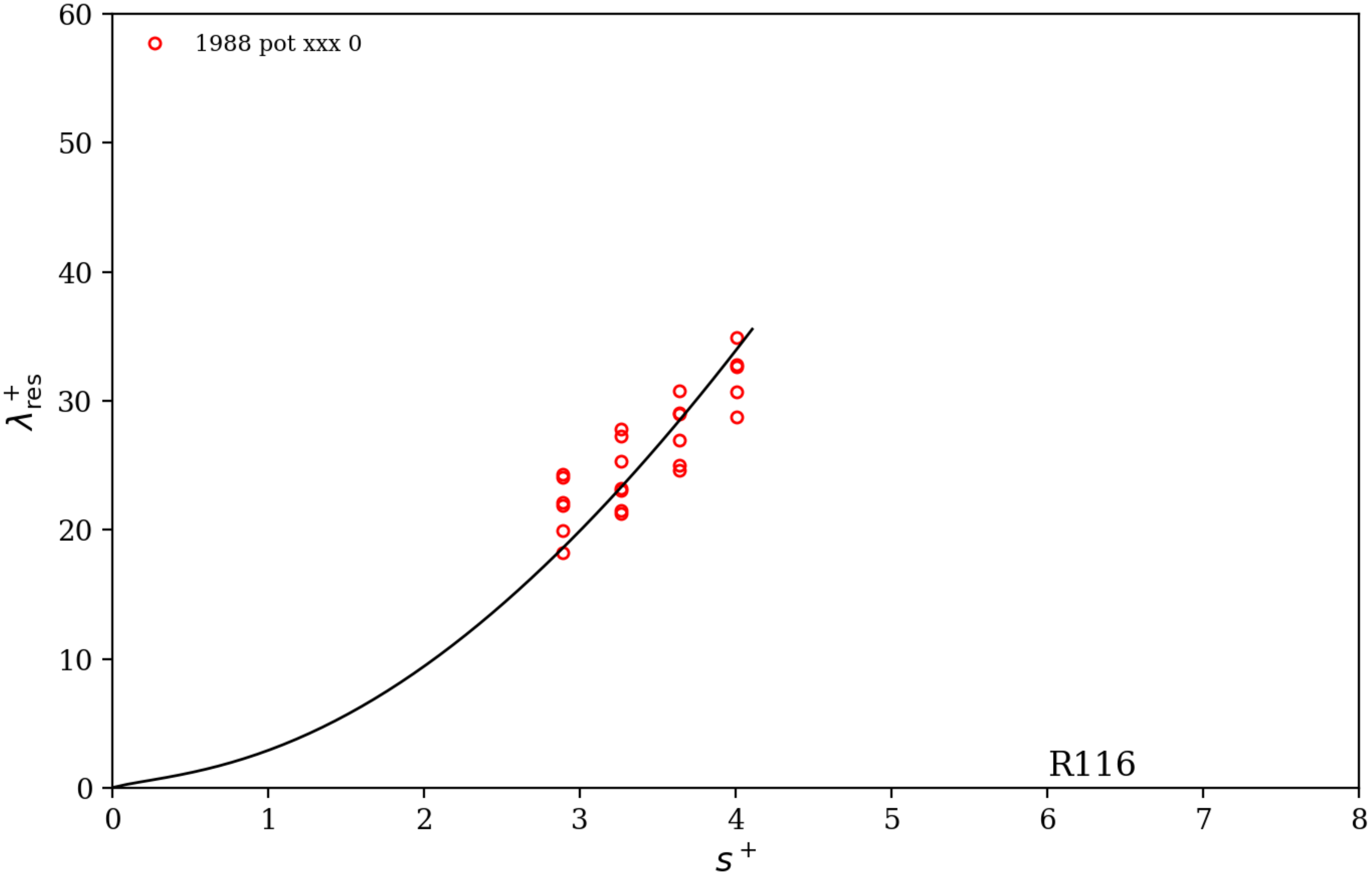

Figure DPR1. R116

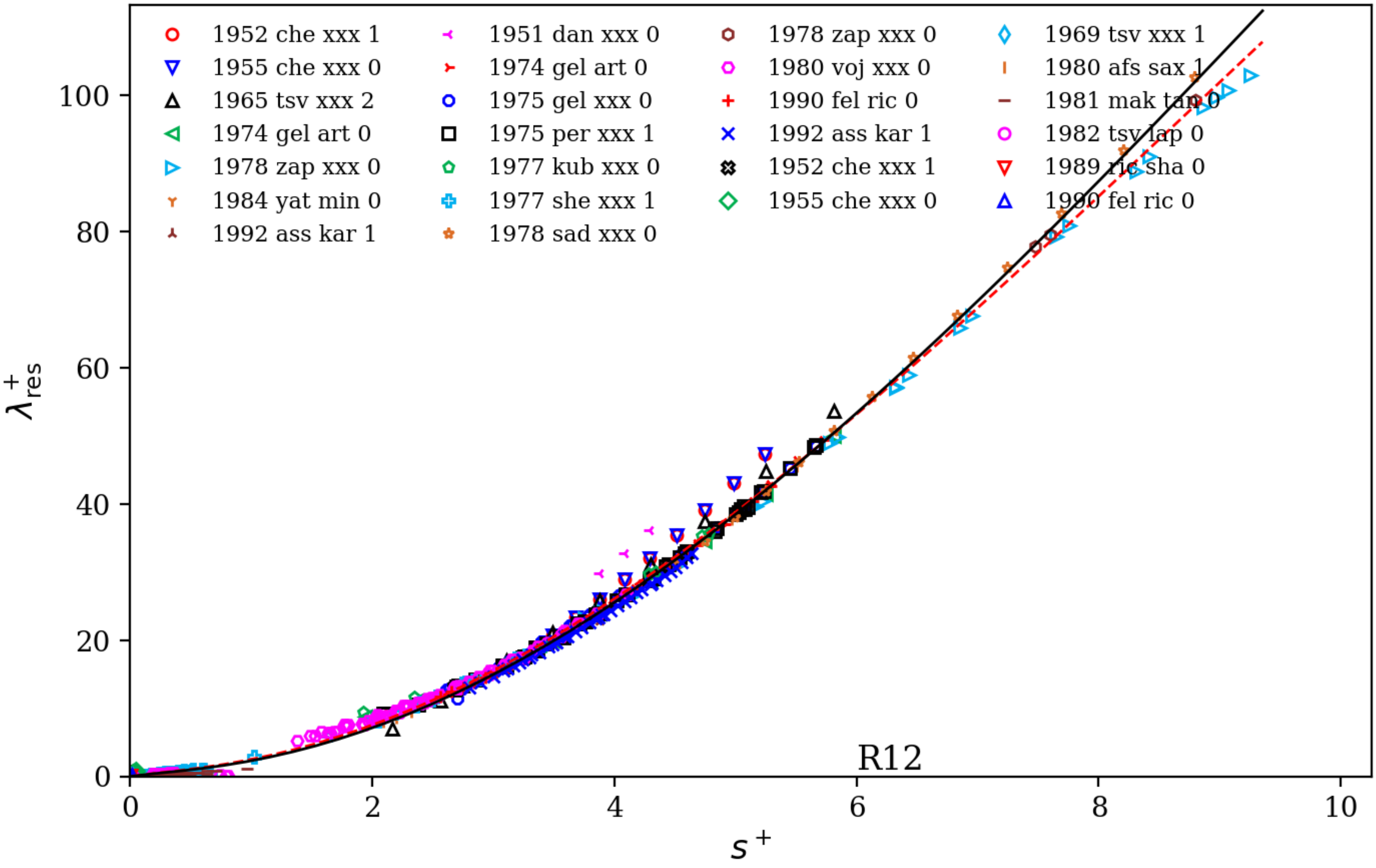

Figure DPR1. R12

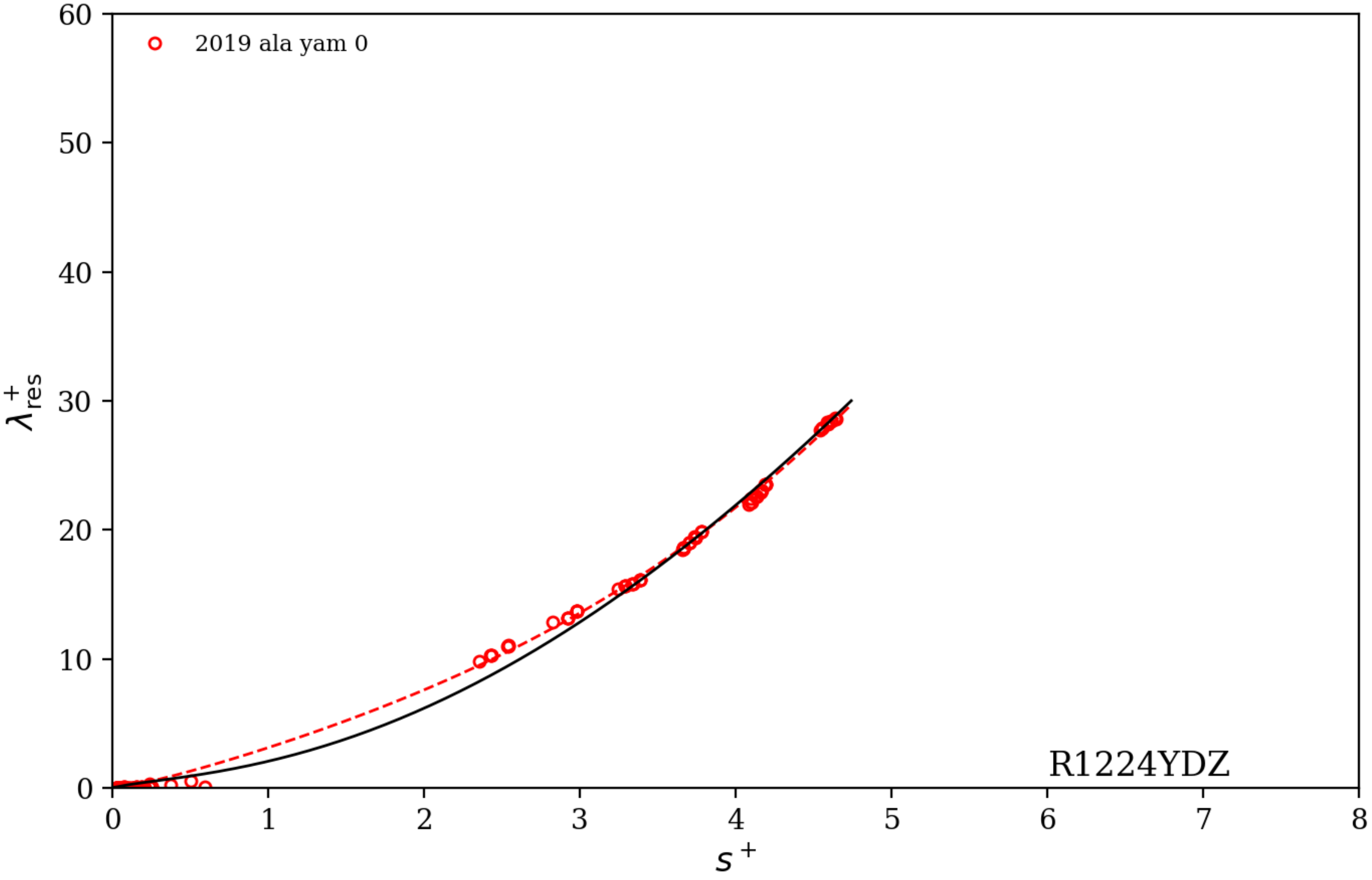

Figure DPR1. R1224YDZ

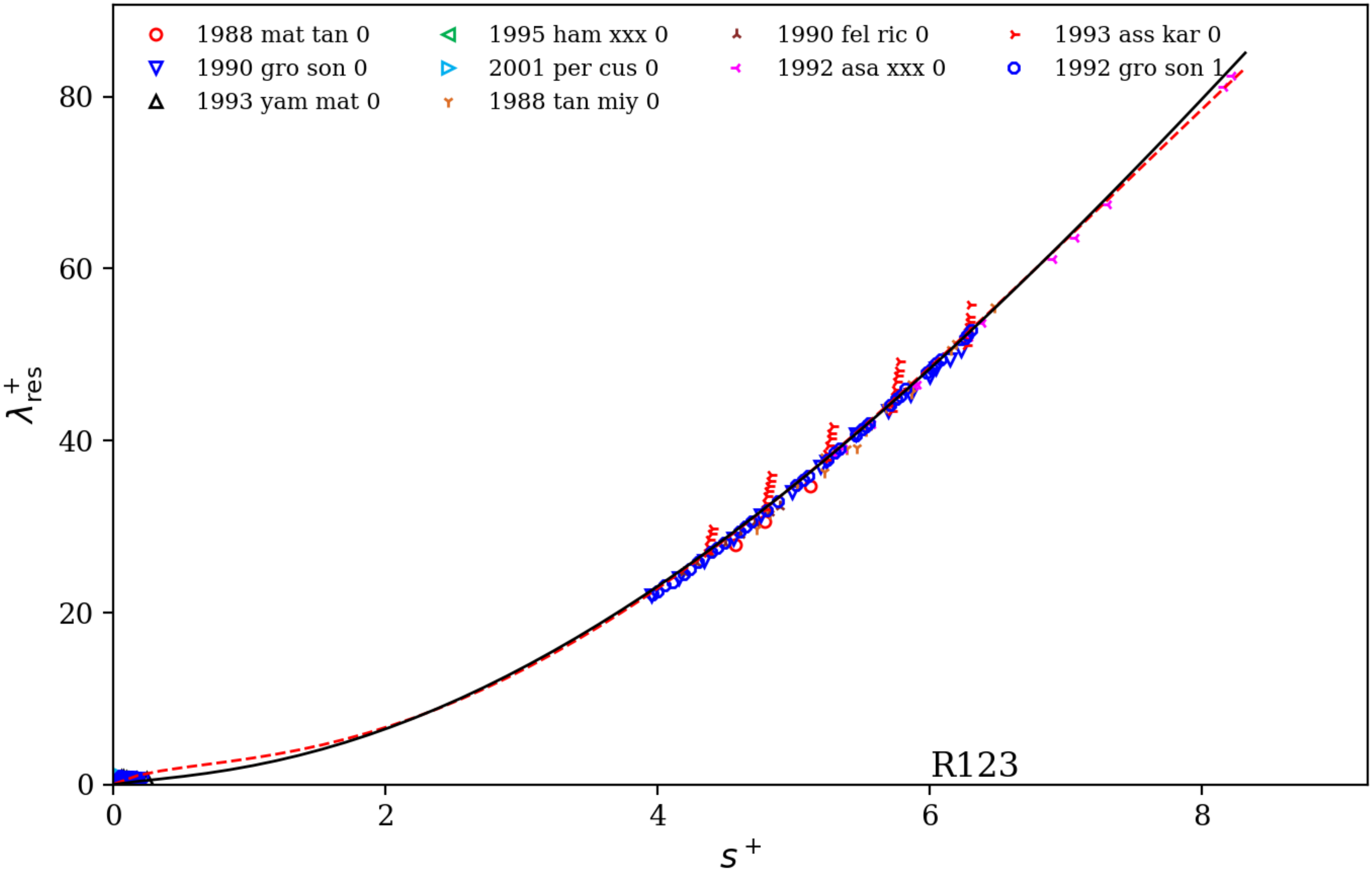

Figure DPR1. R123

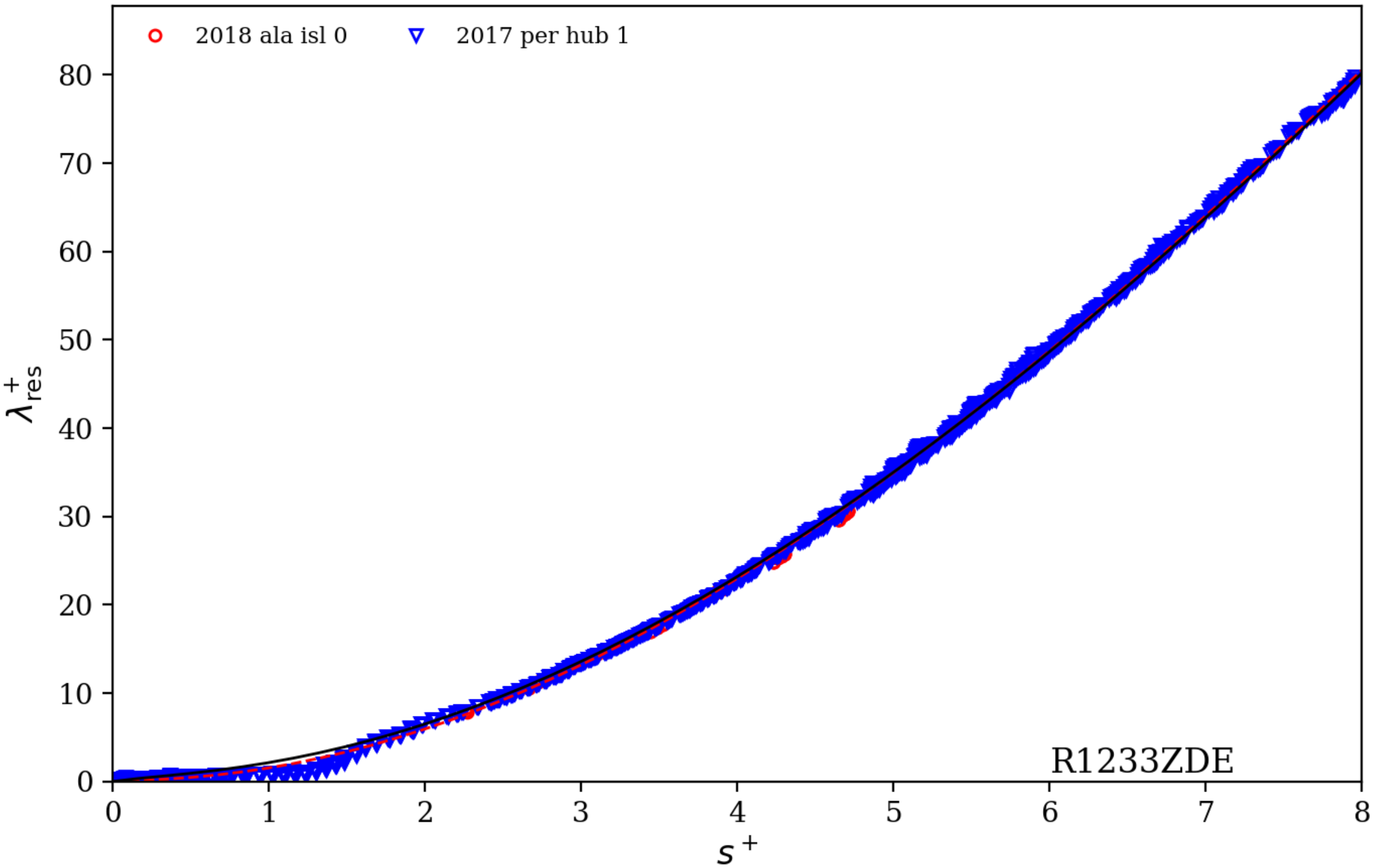

Figure DPR1. R1233ZDE

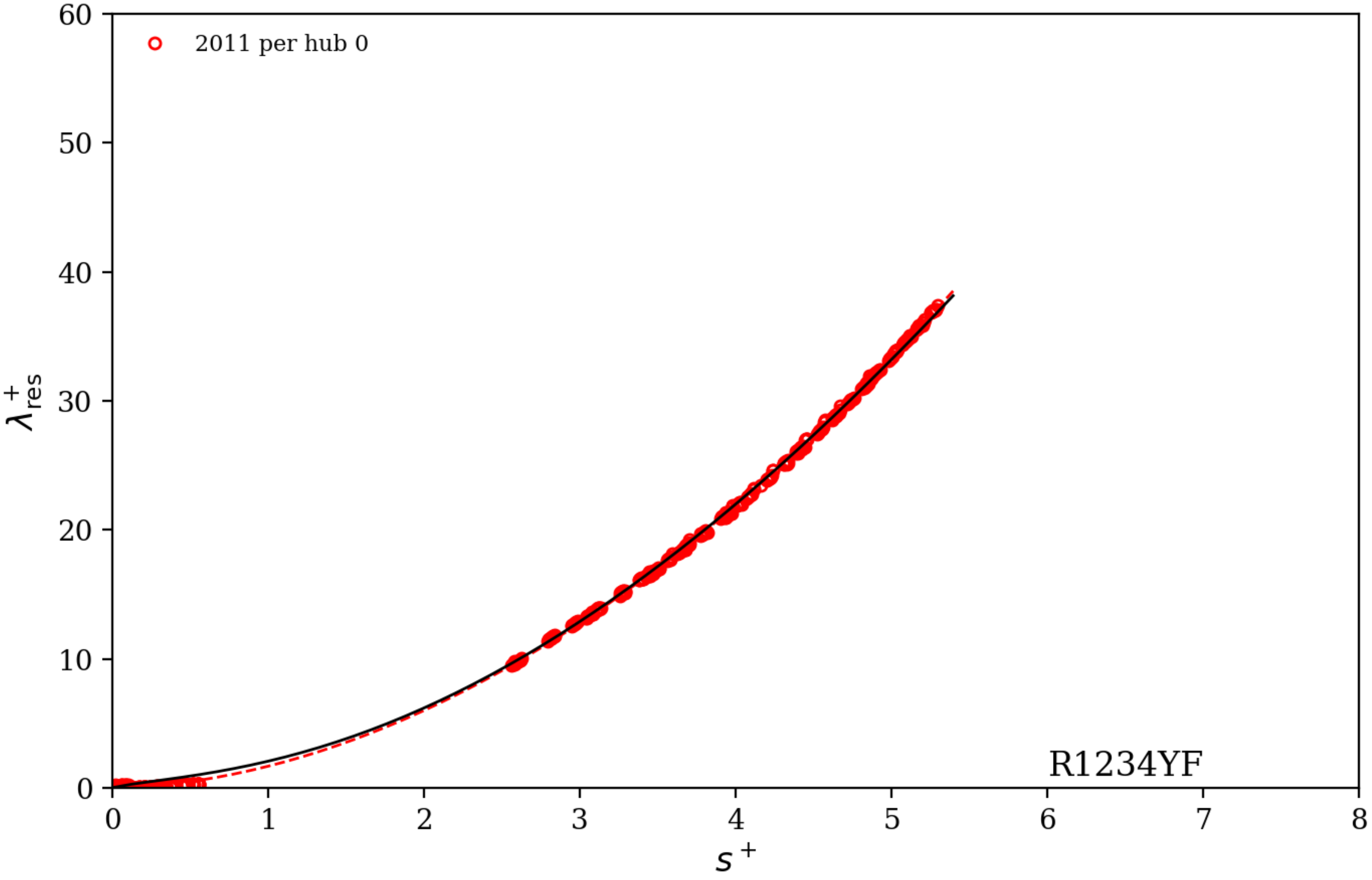

Figure DPR1. R1234YF

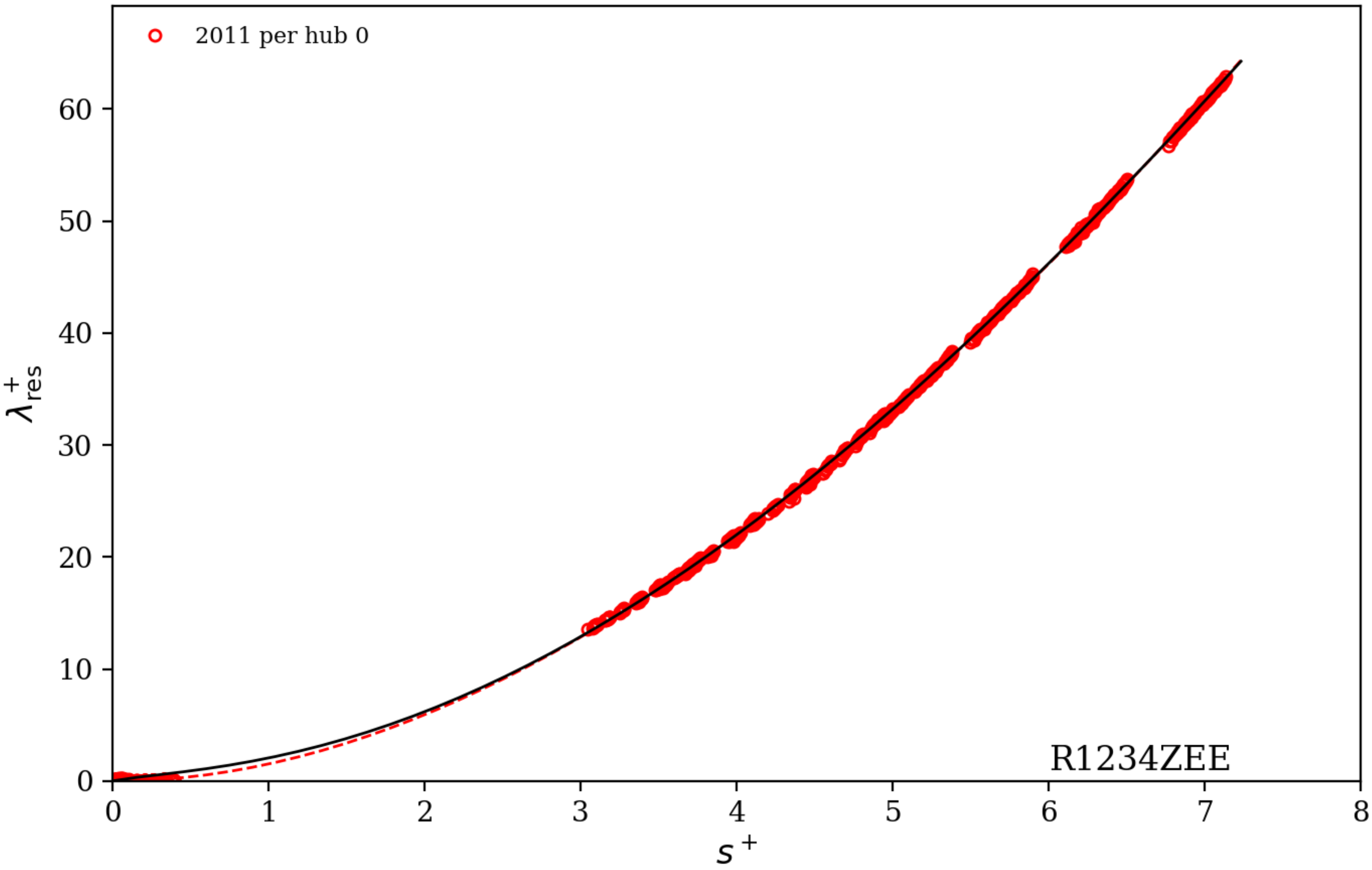

Figure DPR1. R1234ZEE

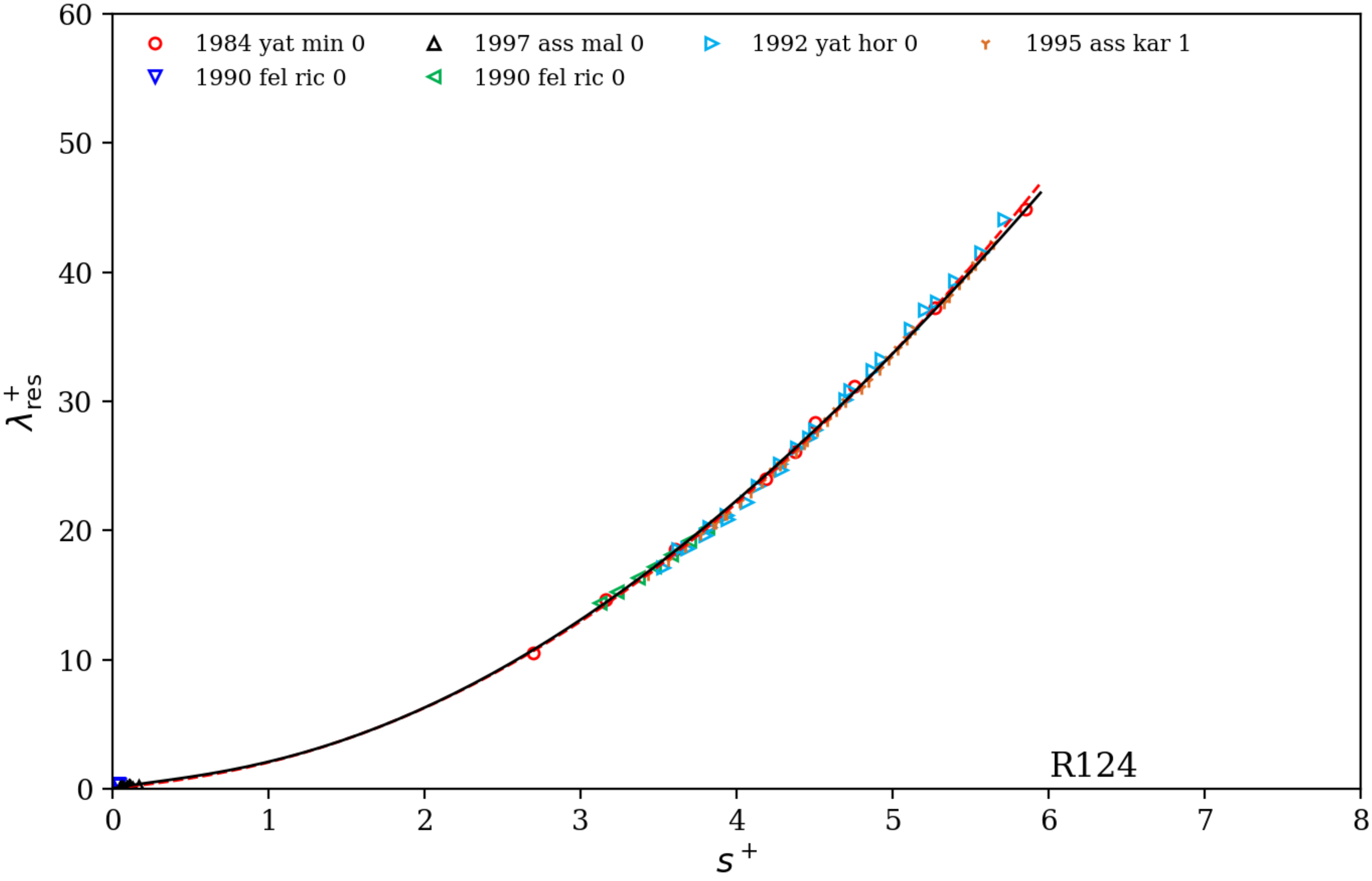

Figure DPR1. R124

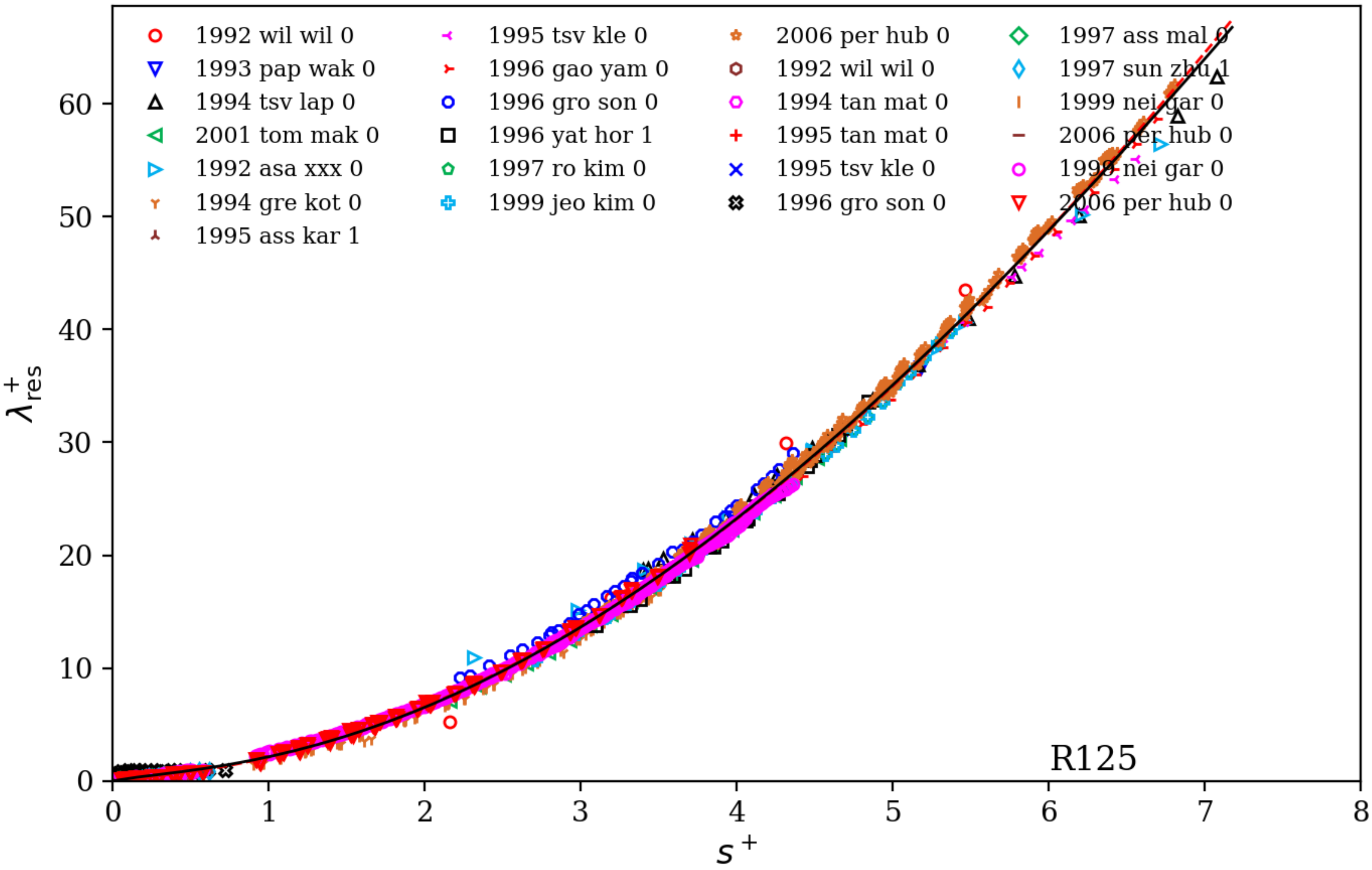

Figure DPR1. R125

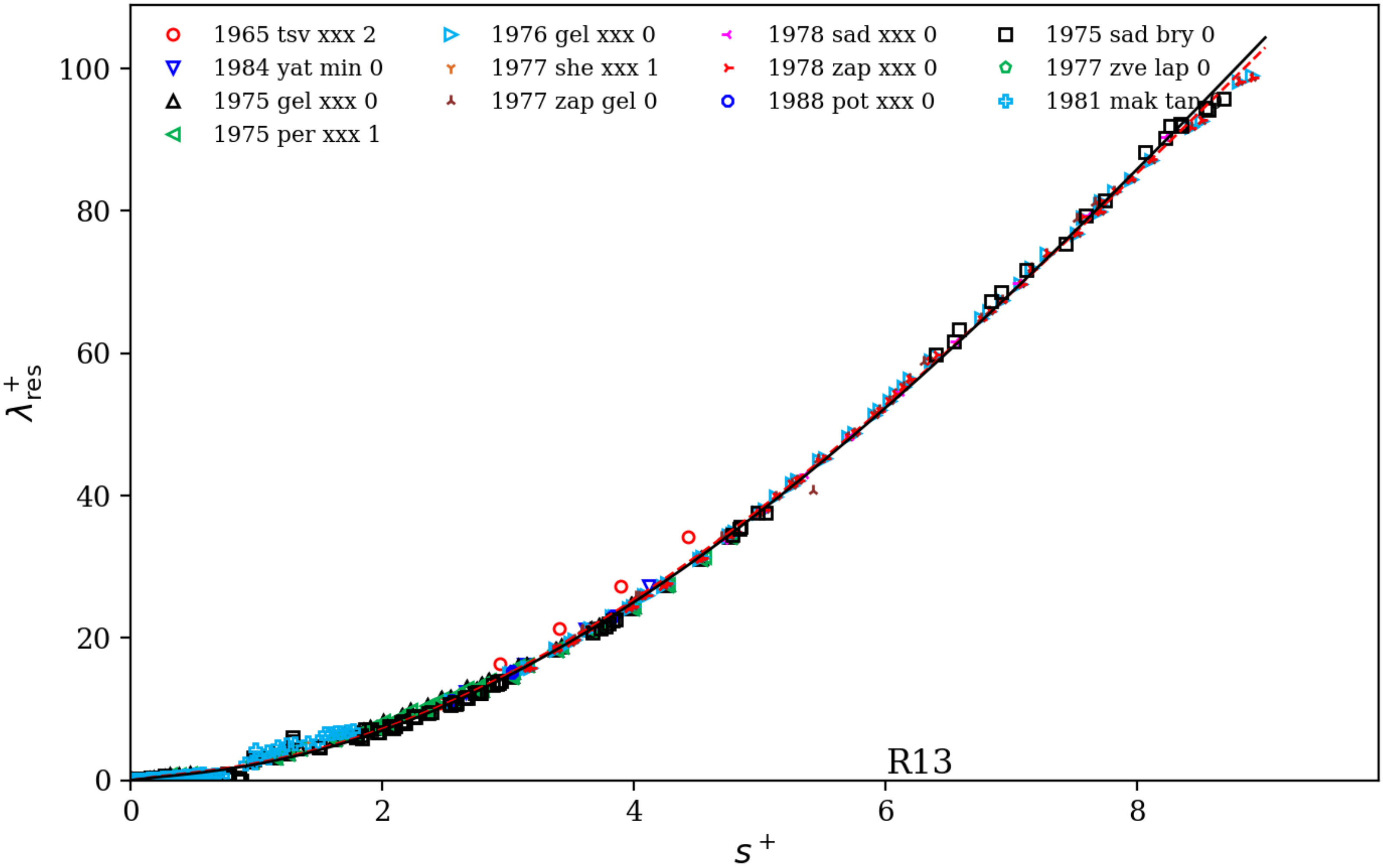

Figure DPR1. R13

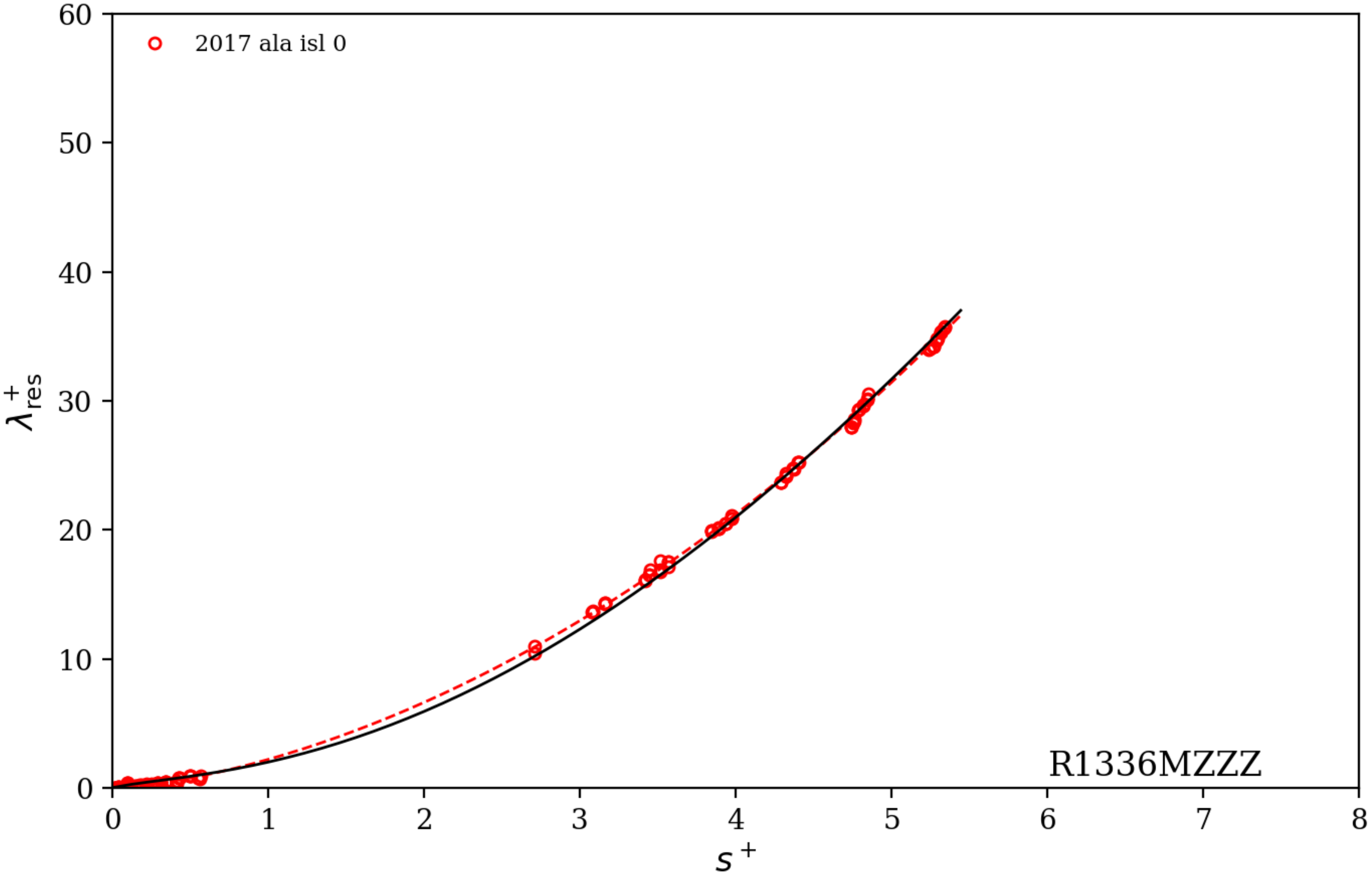

Figure DPR1. R1336MZZZ

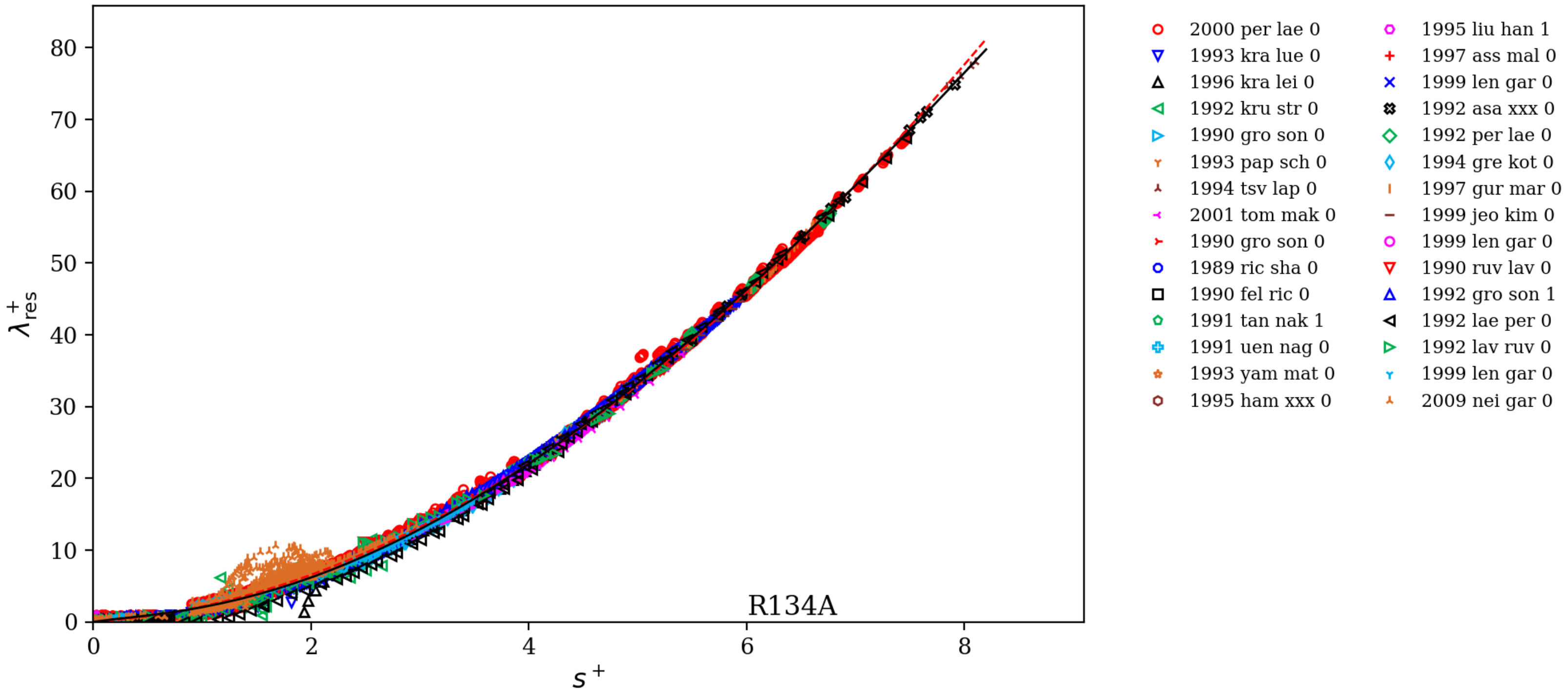

Figure DPR1. R134A

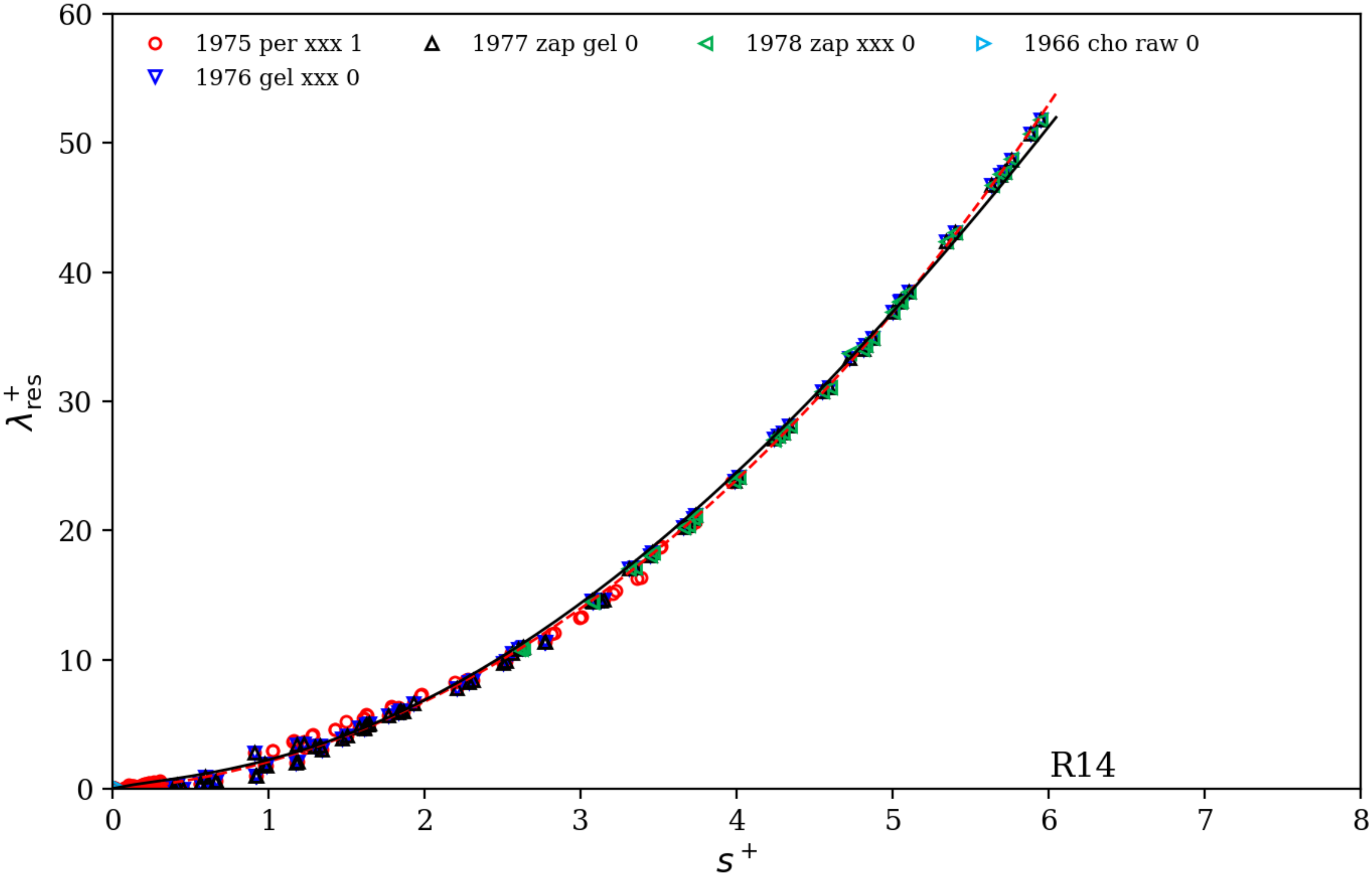

Figure DPR1. R14

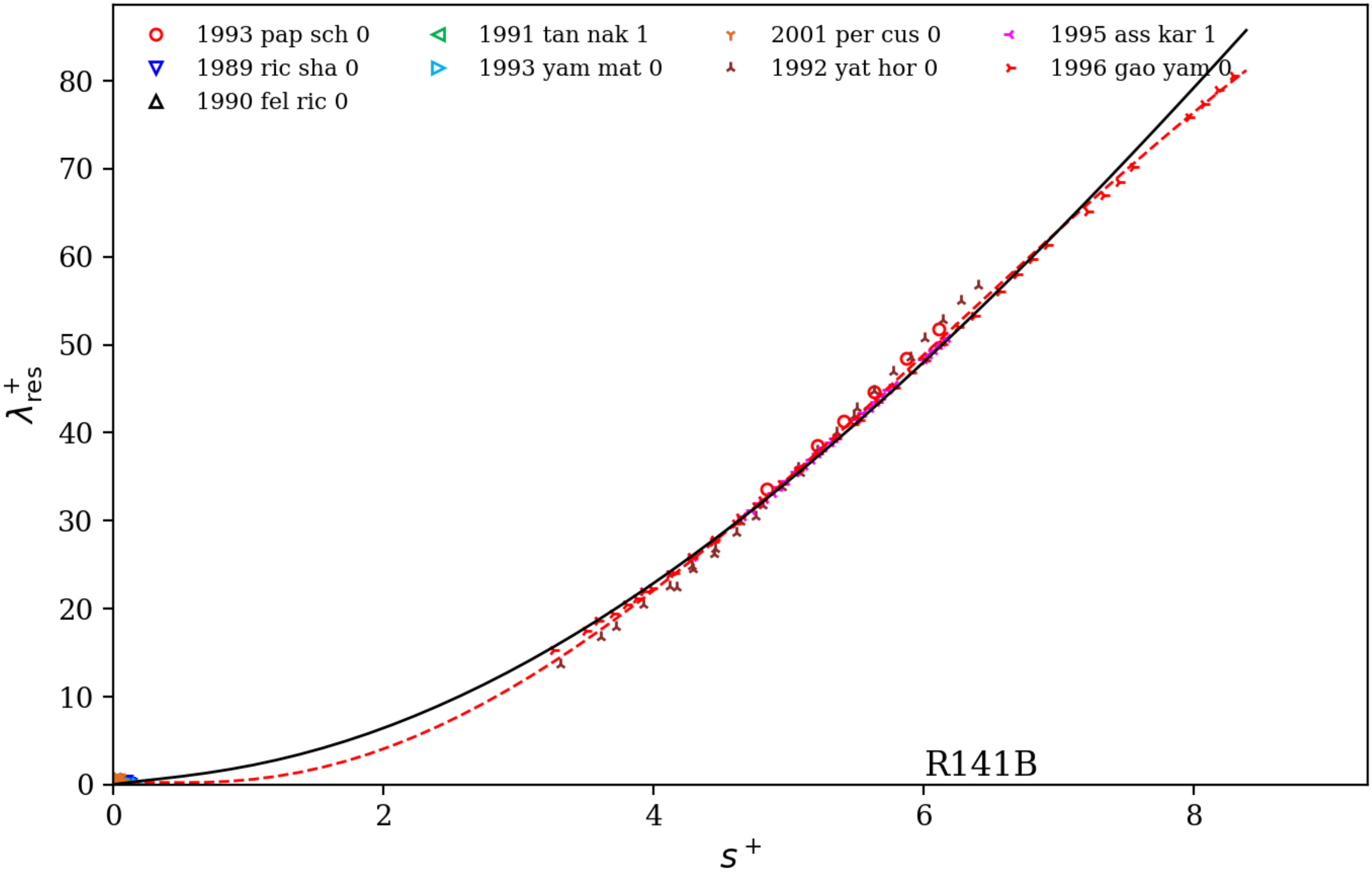

Figure DPR1. R141B

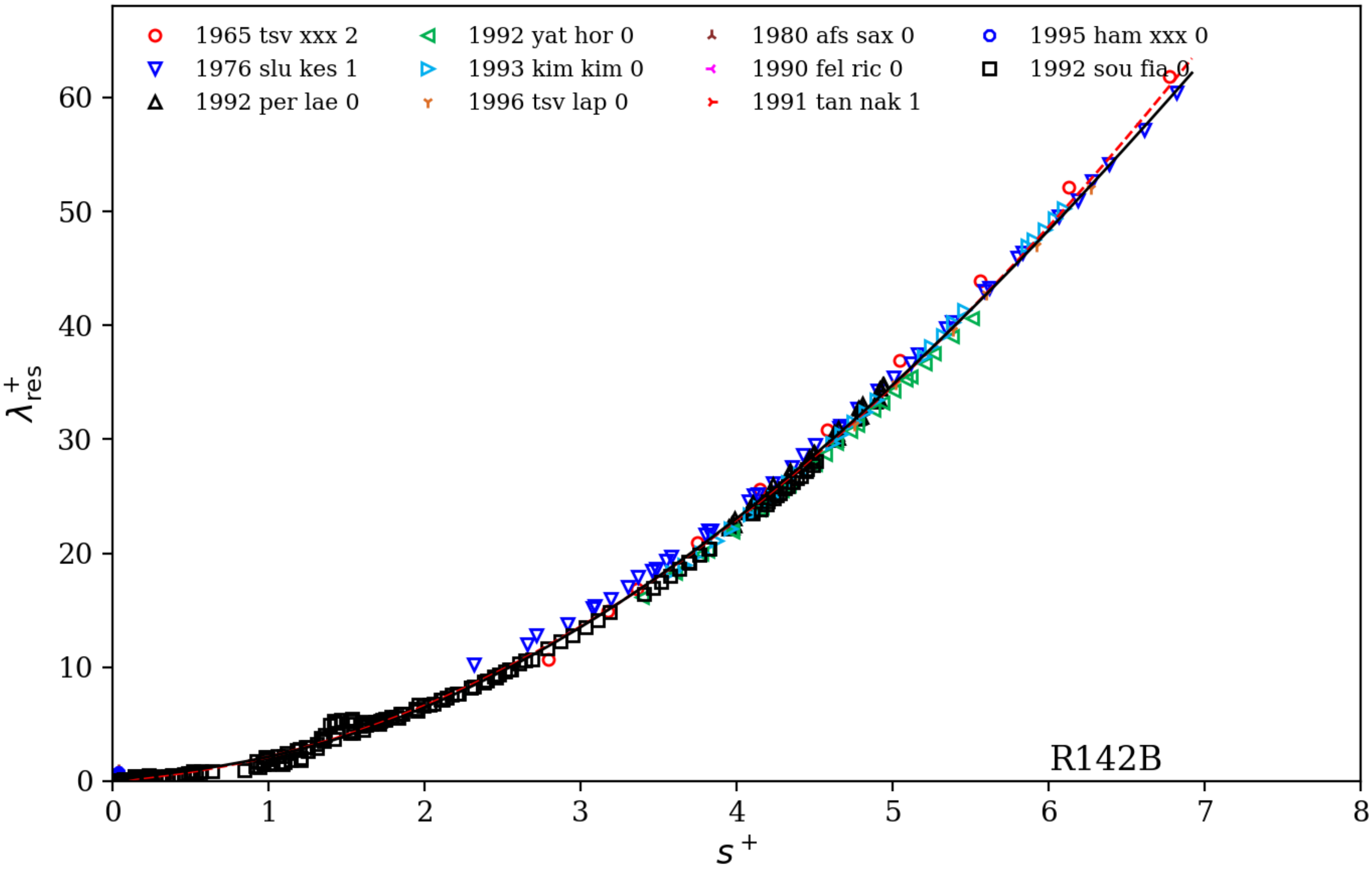

Figure DPR1. R142B

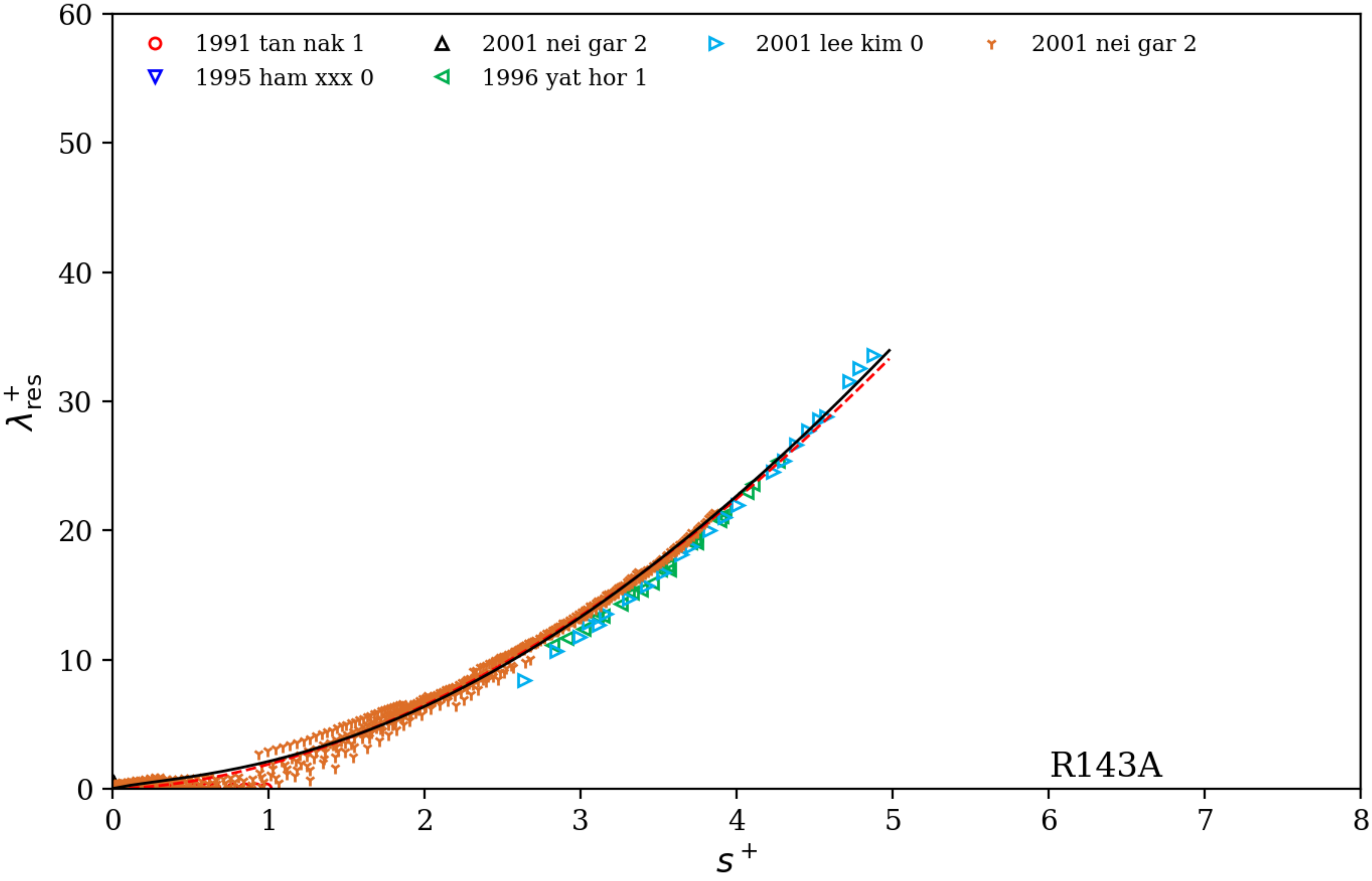

Figure DPR1. R143A

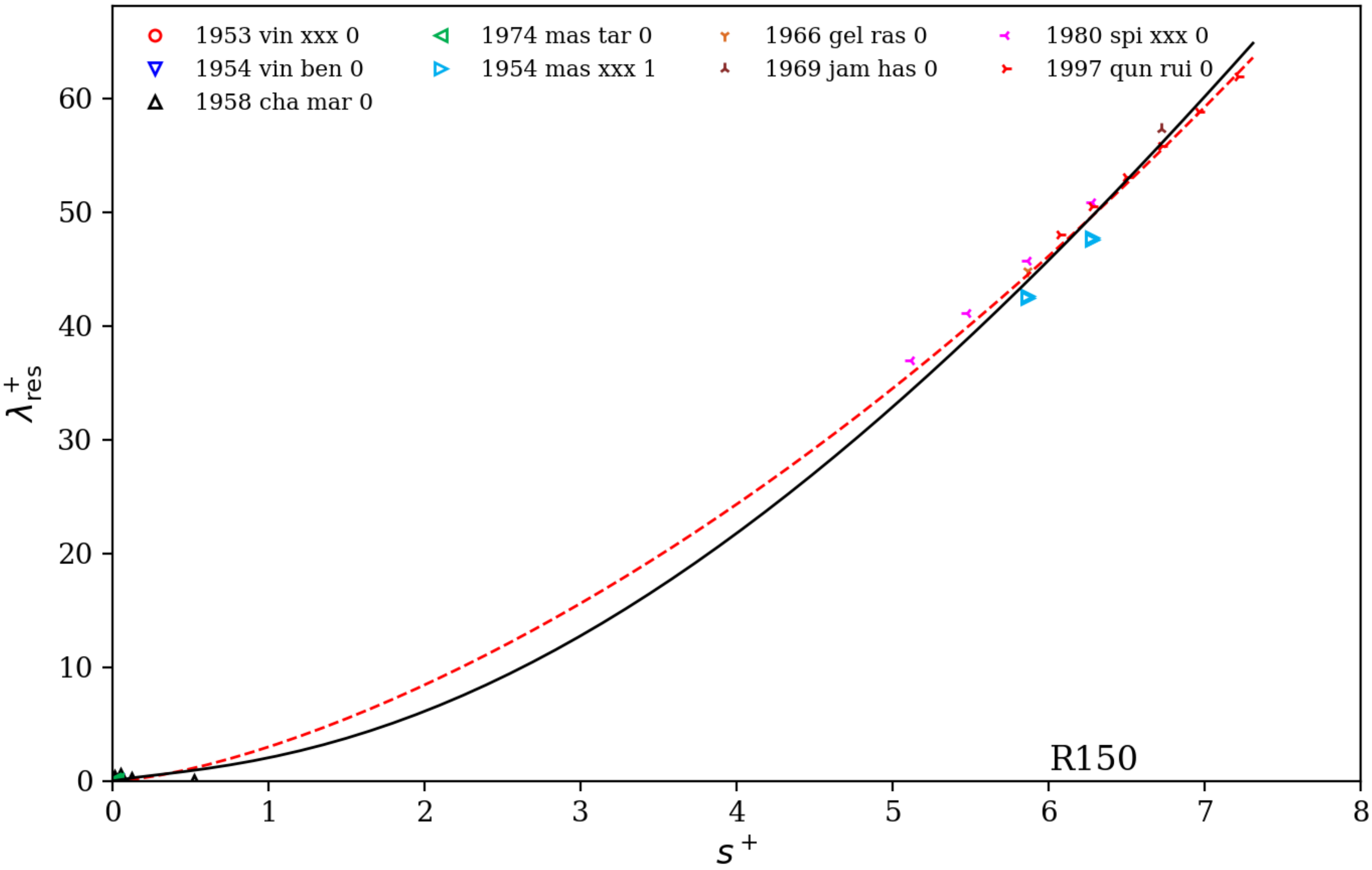

Figure DPR1. R150

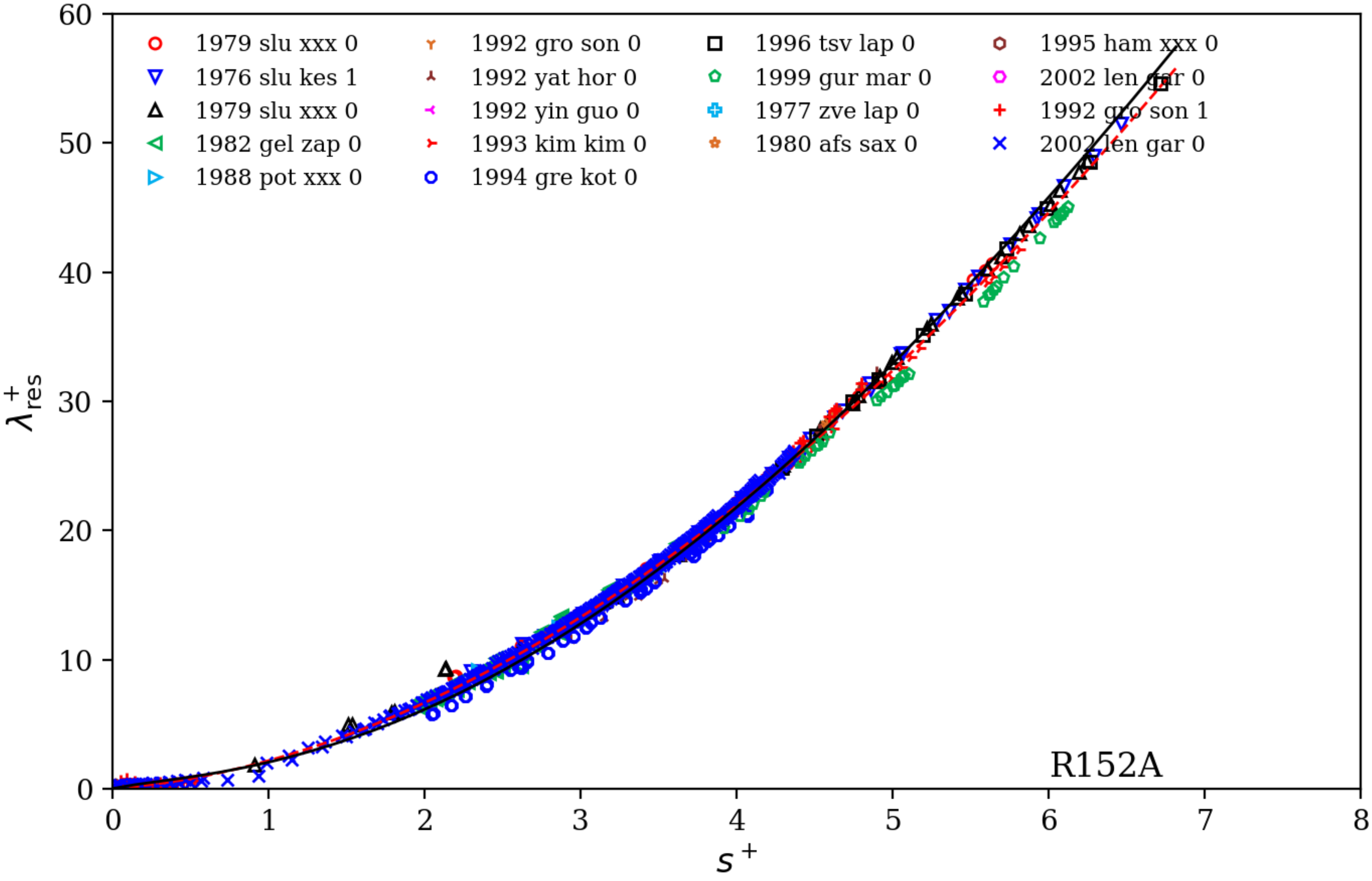

Figure DPR1. R152A

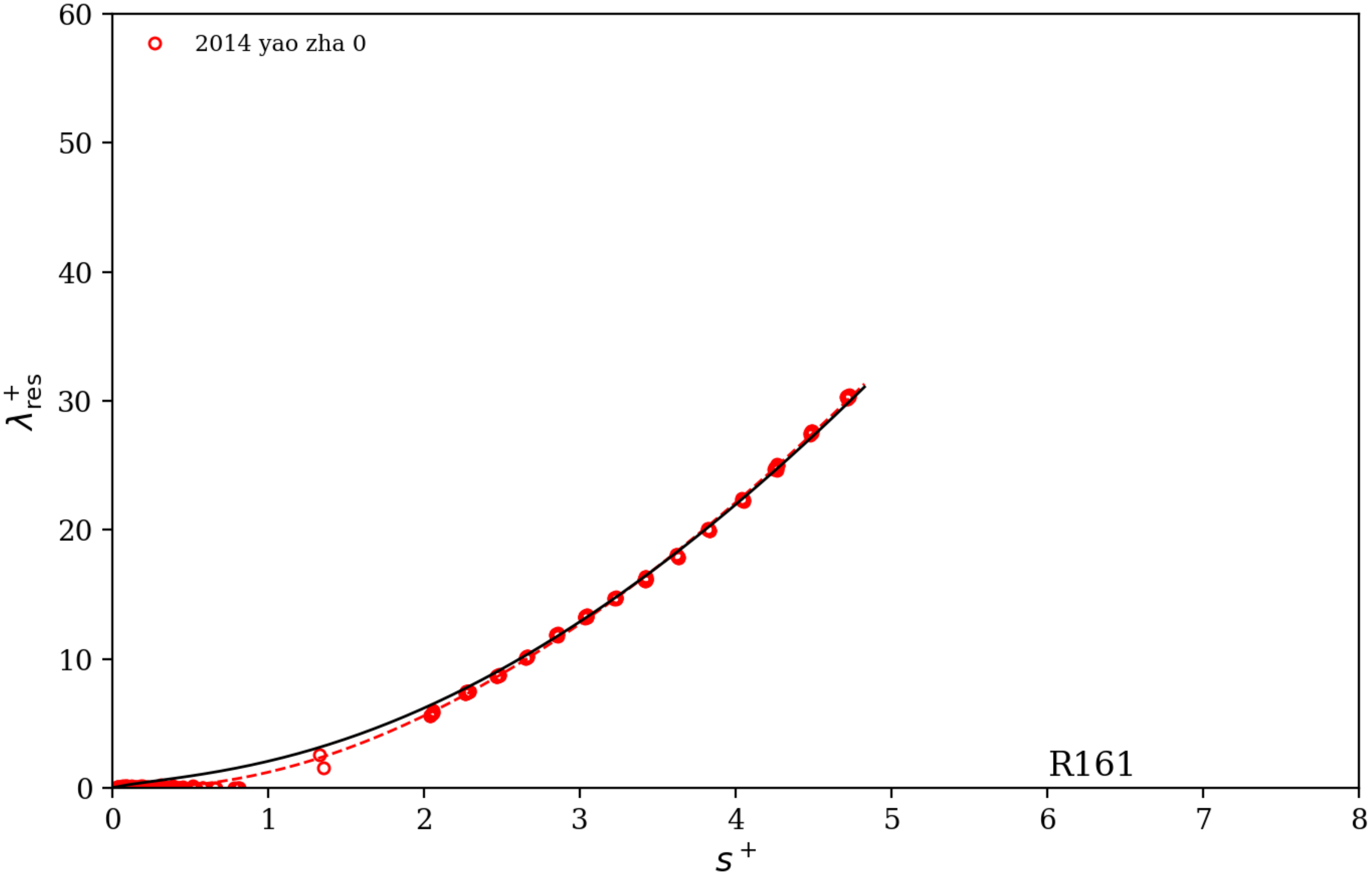

Figure DPR1. R161

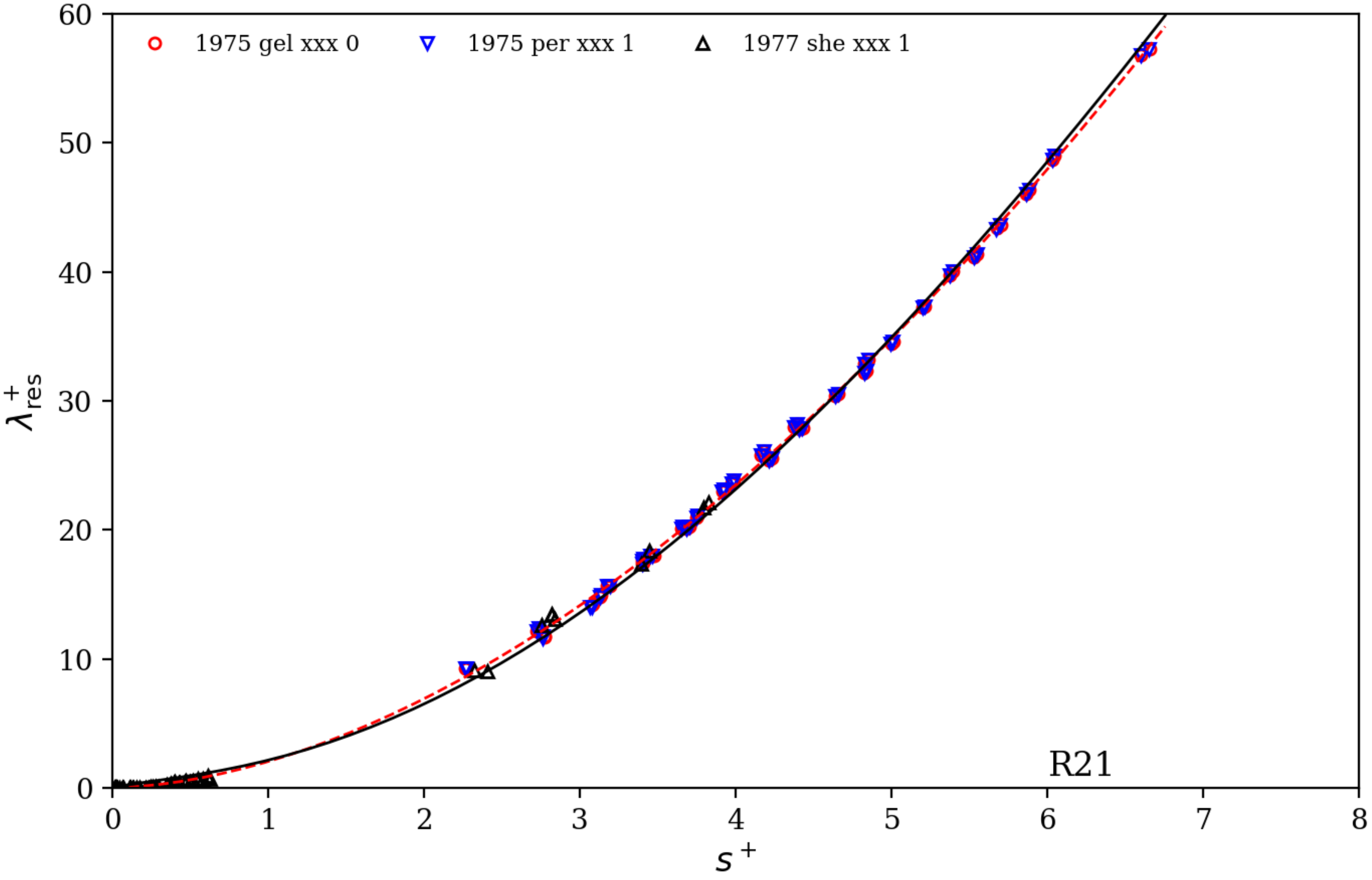

Figure DPR1. R21

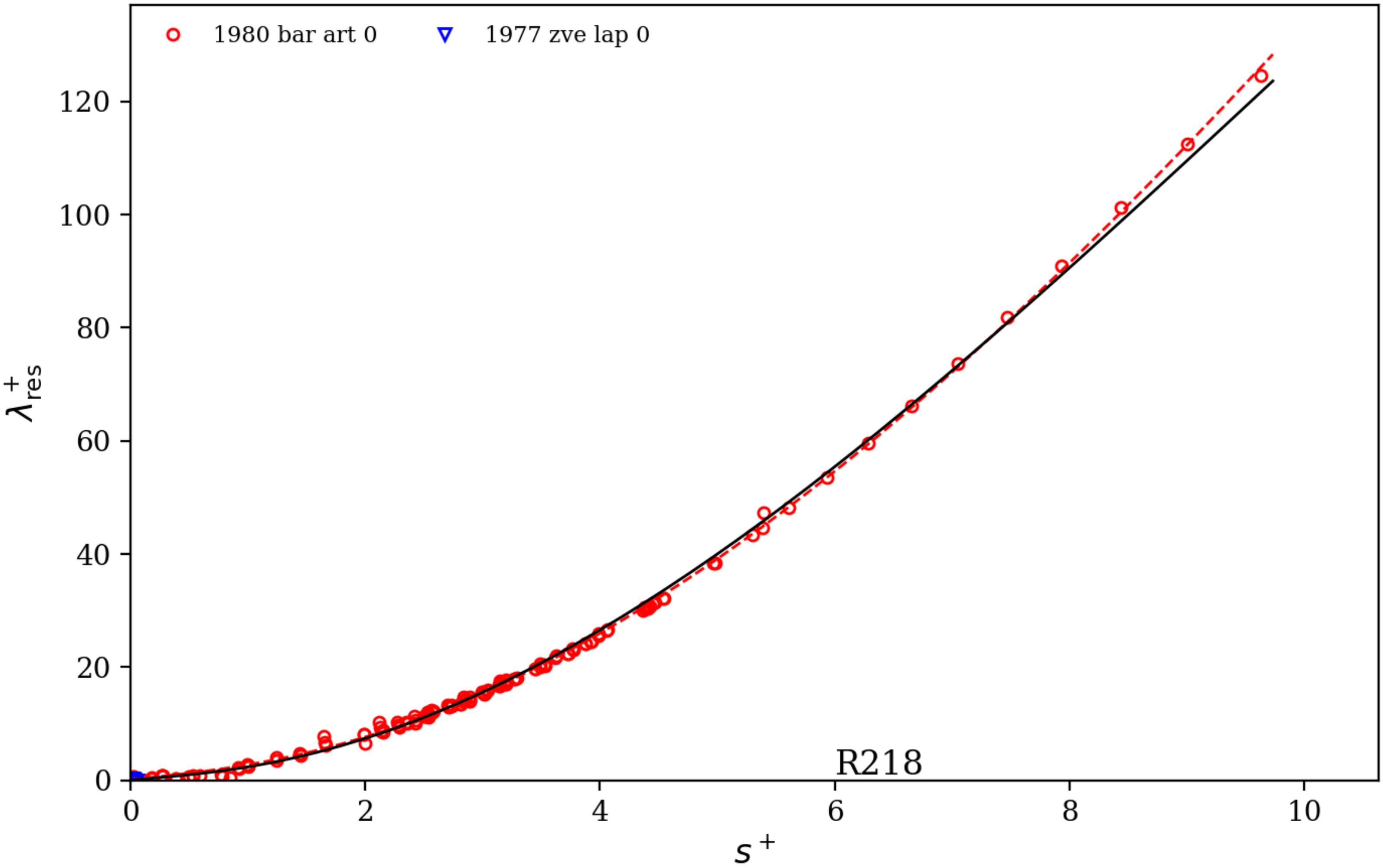

Figure DPR1. R218

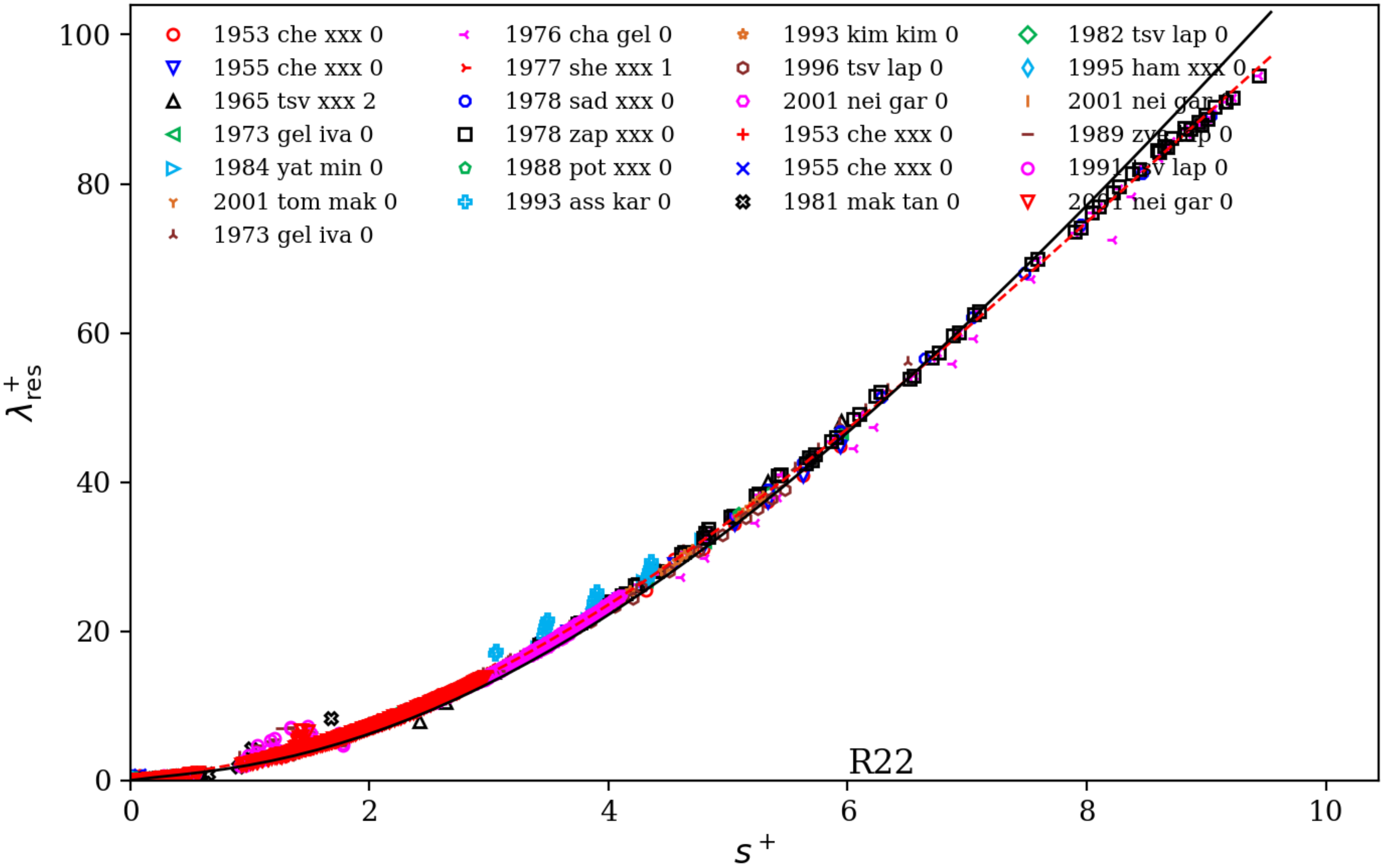

Figure DPR1. R22

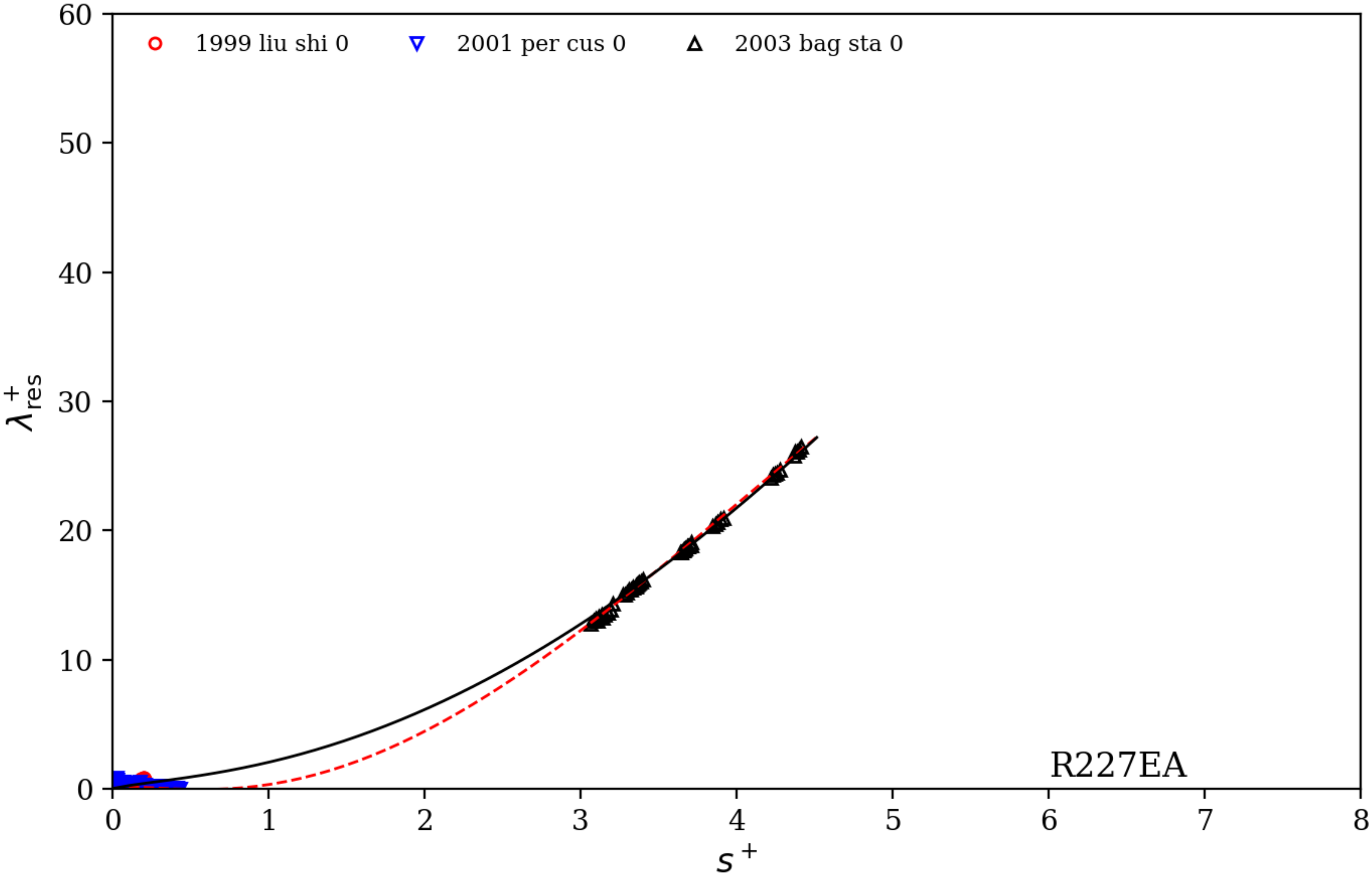

Figure DPR1. R227EA

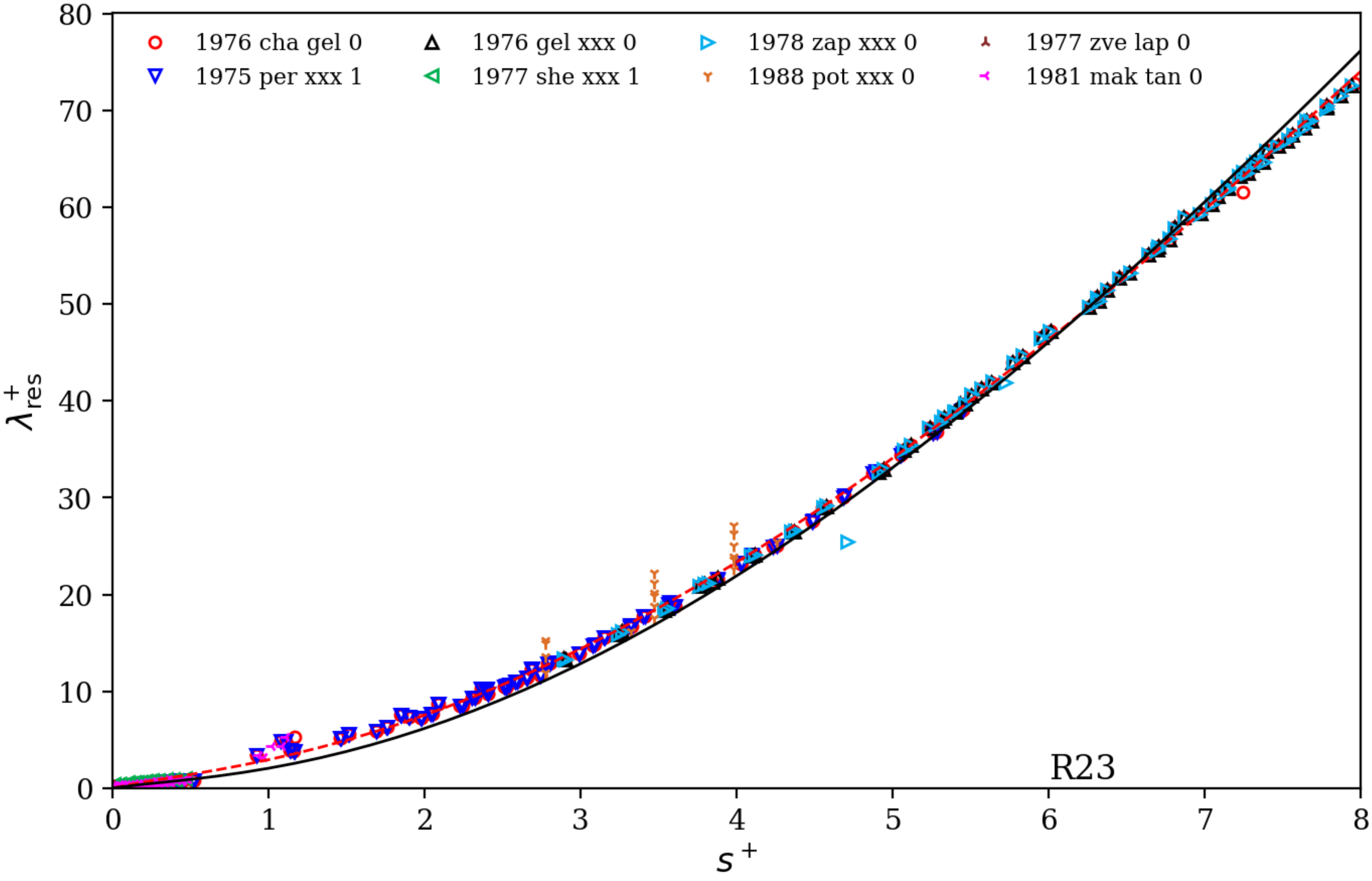

Figure DPR1. R23

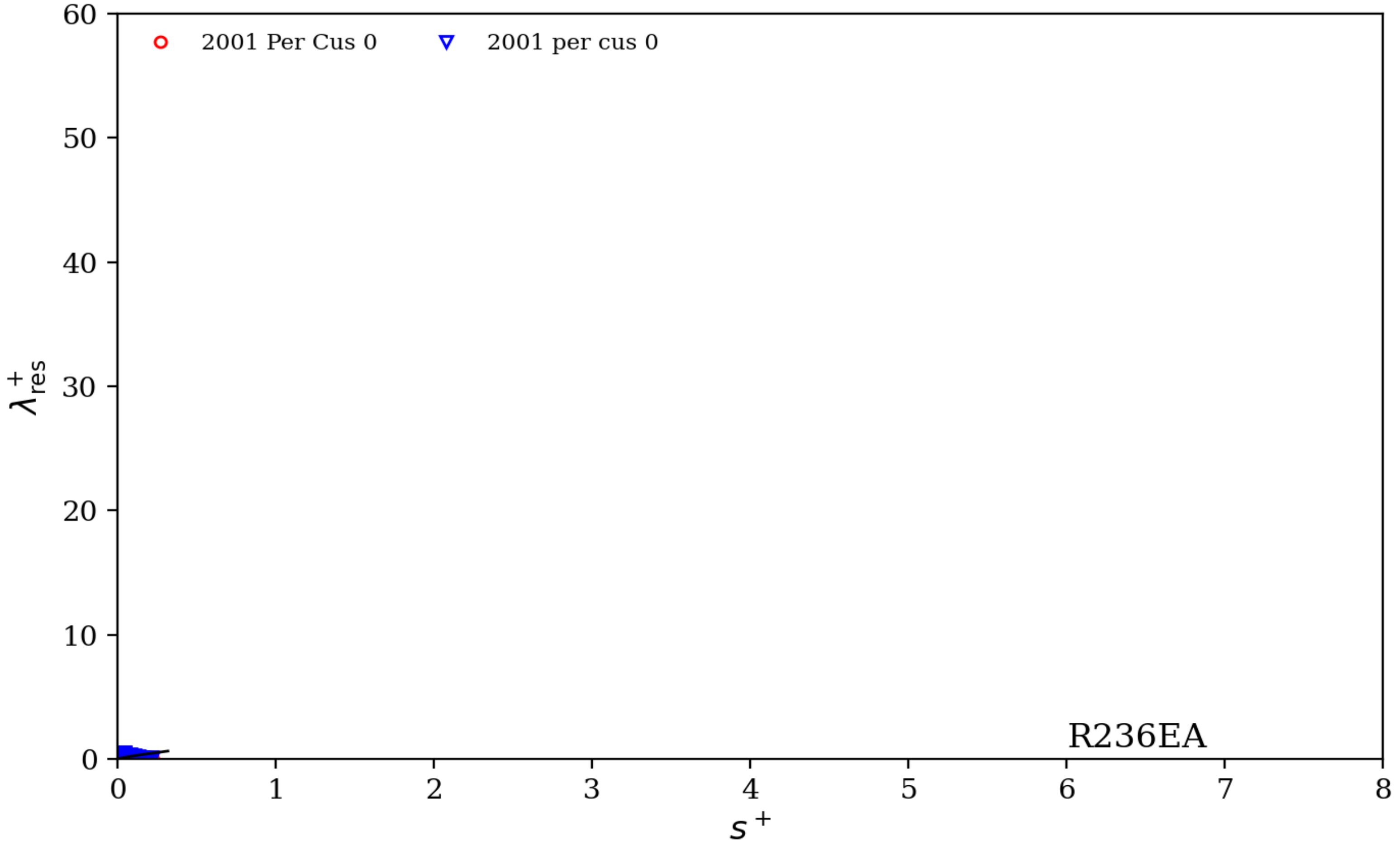

Figure DPR1. R236EA

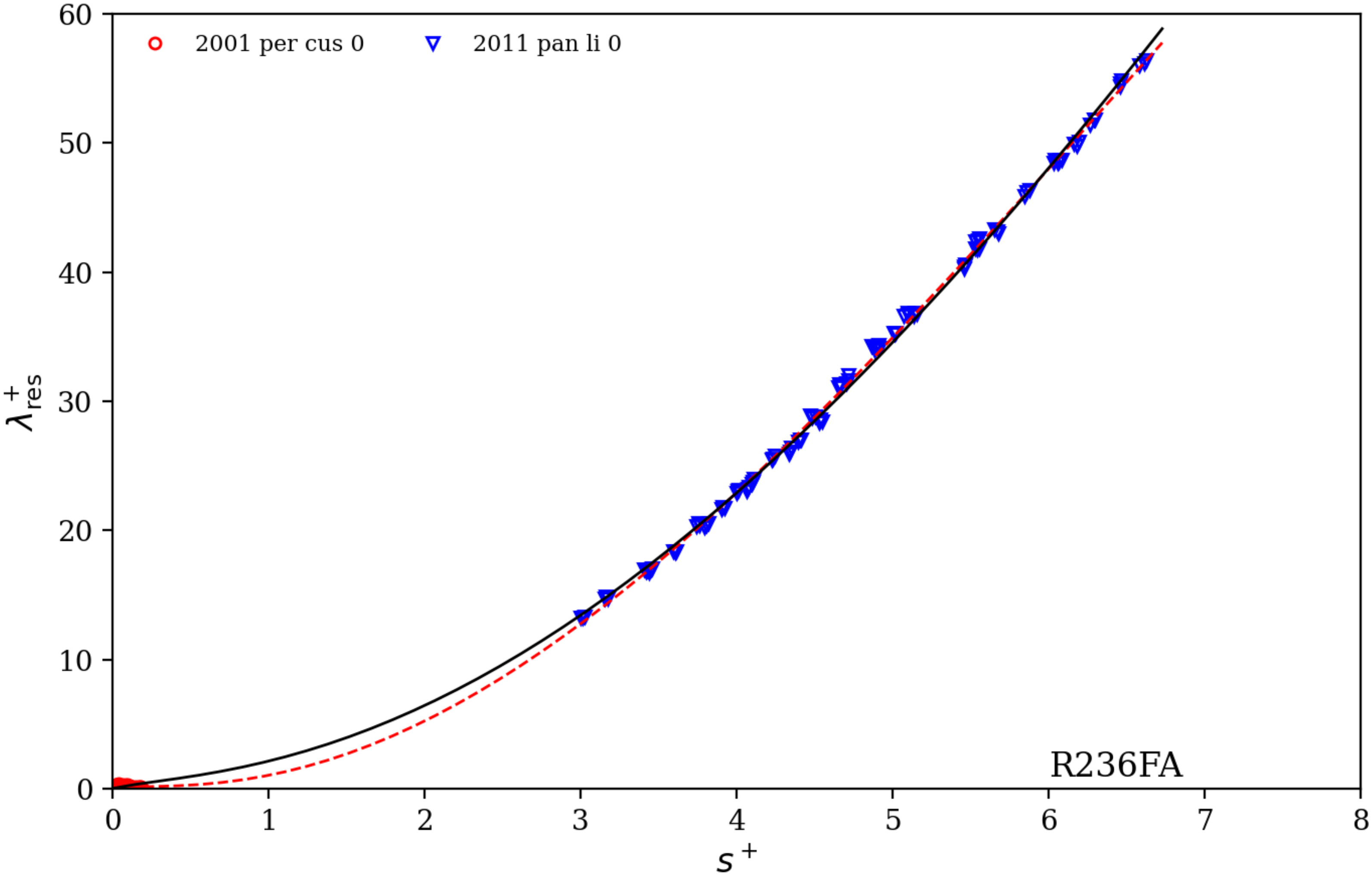

Figure DPR1. R236FA

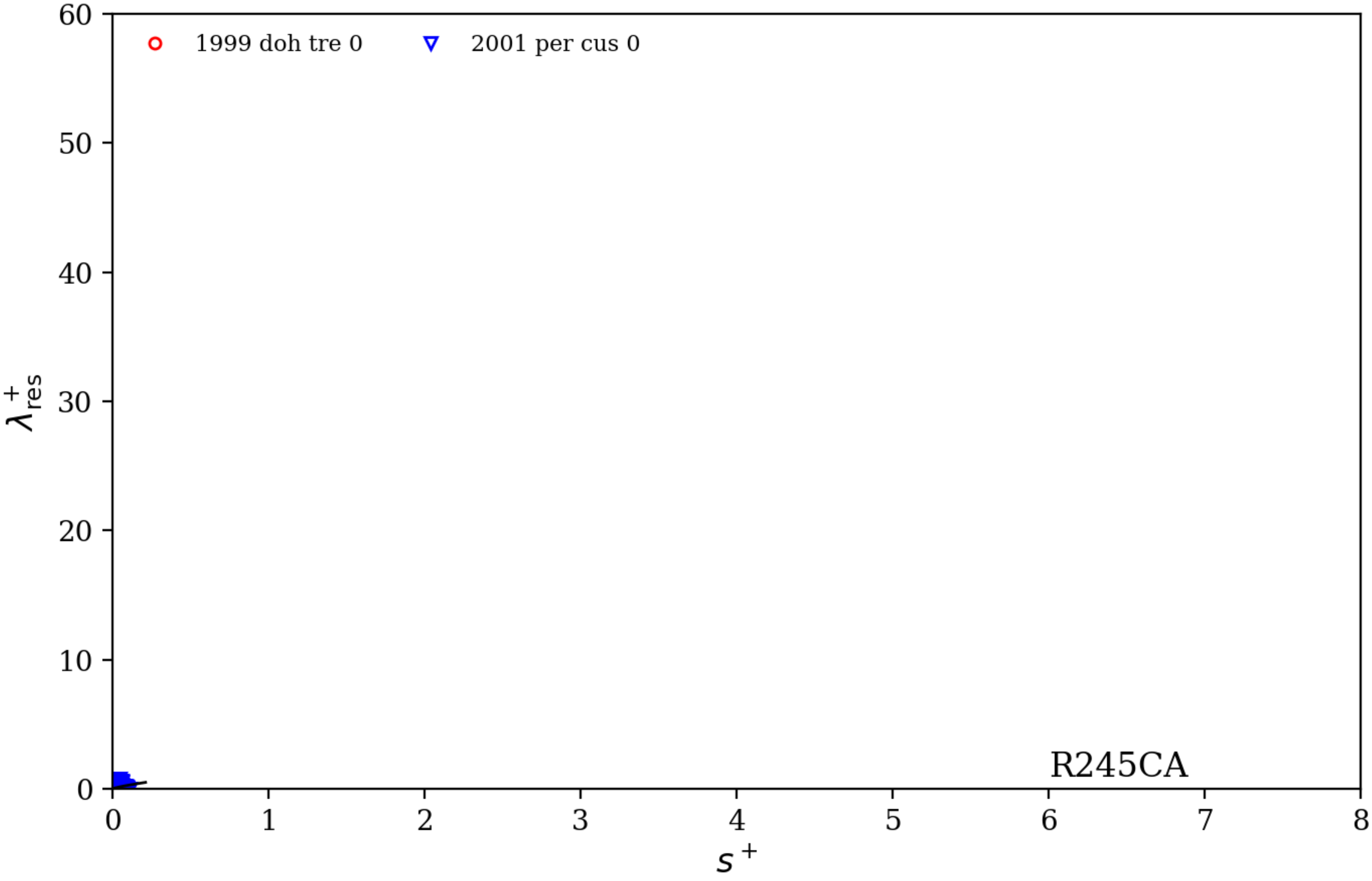

Figure DPR1. R245CA

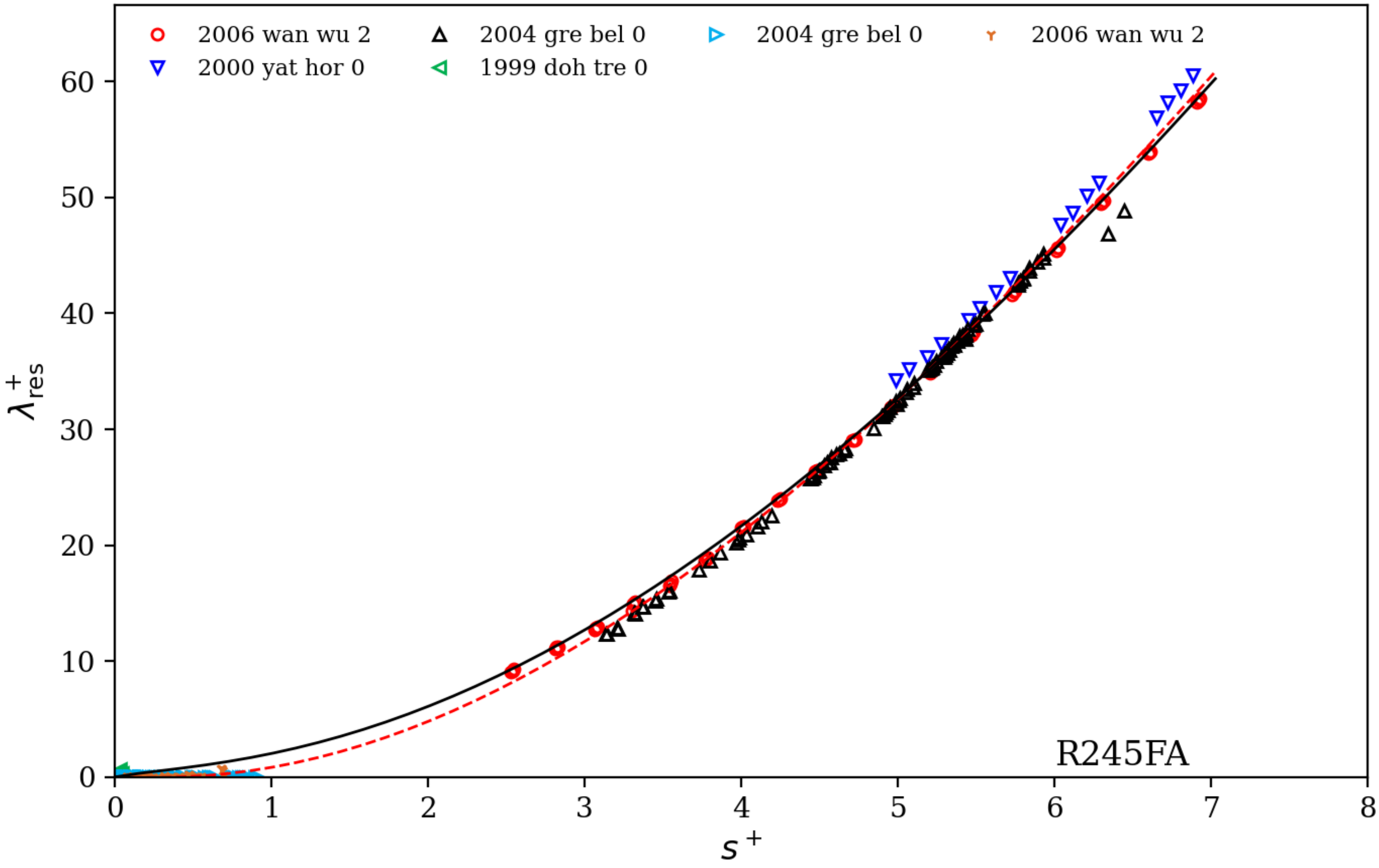

Figure DPR1. R245FA

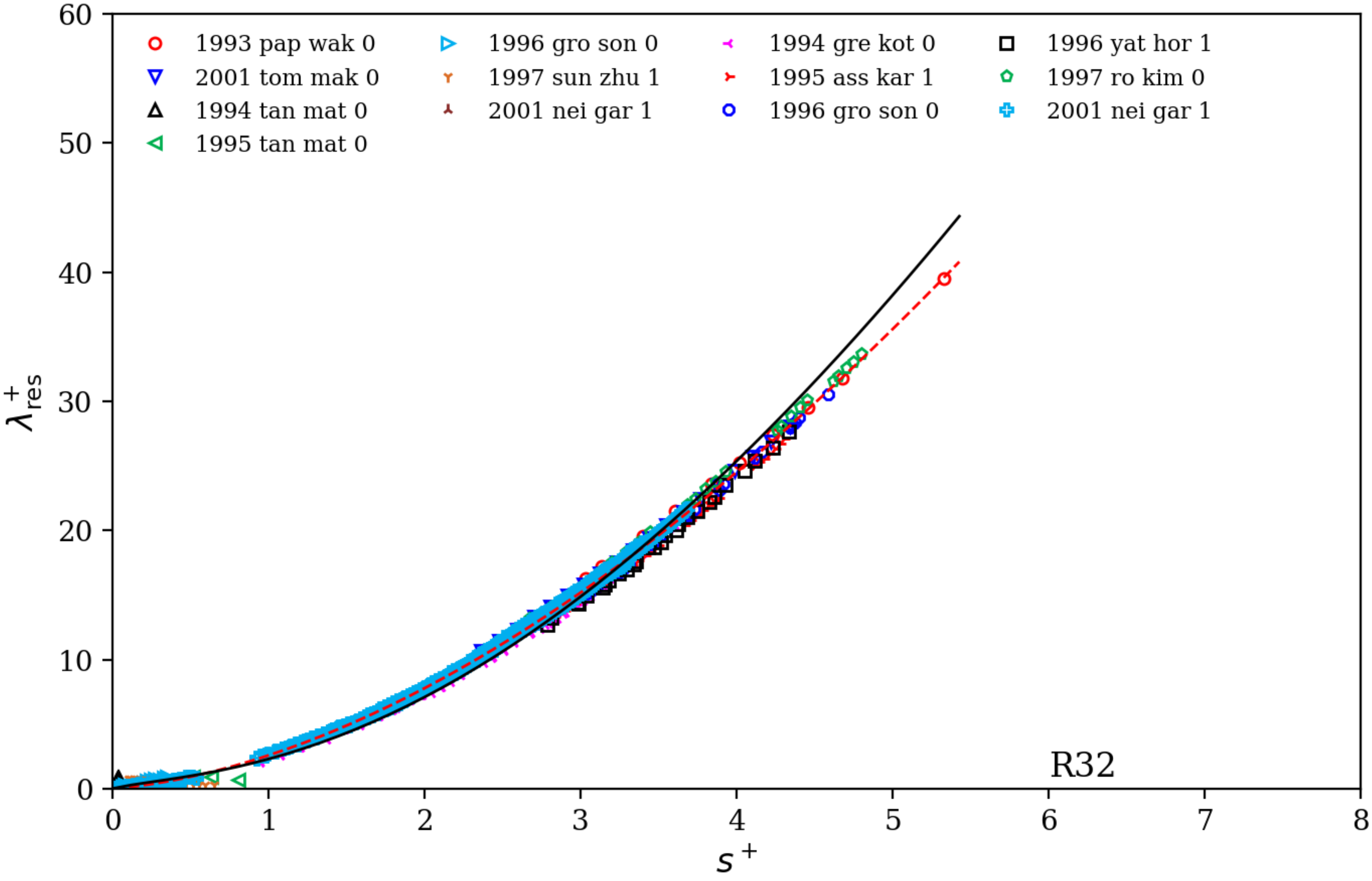

Figure DPR1. R32

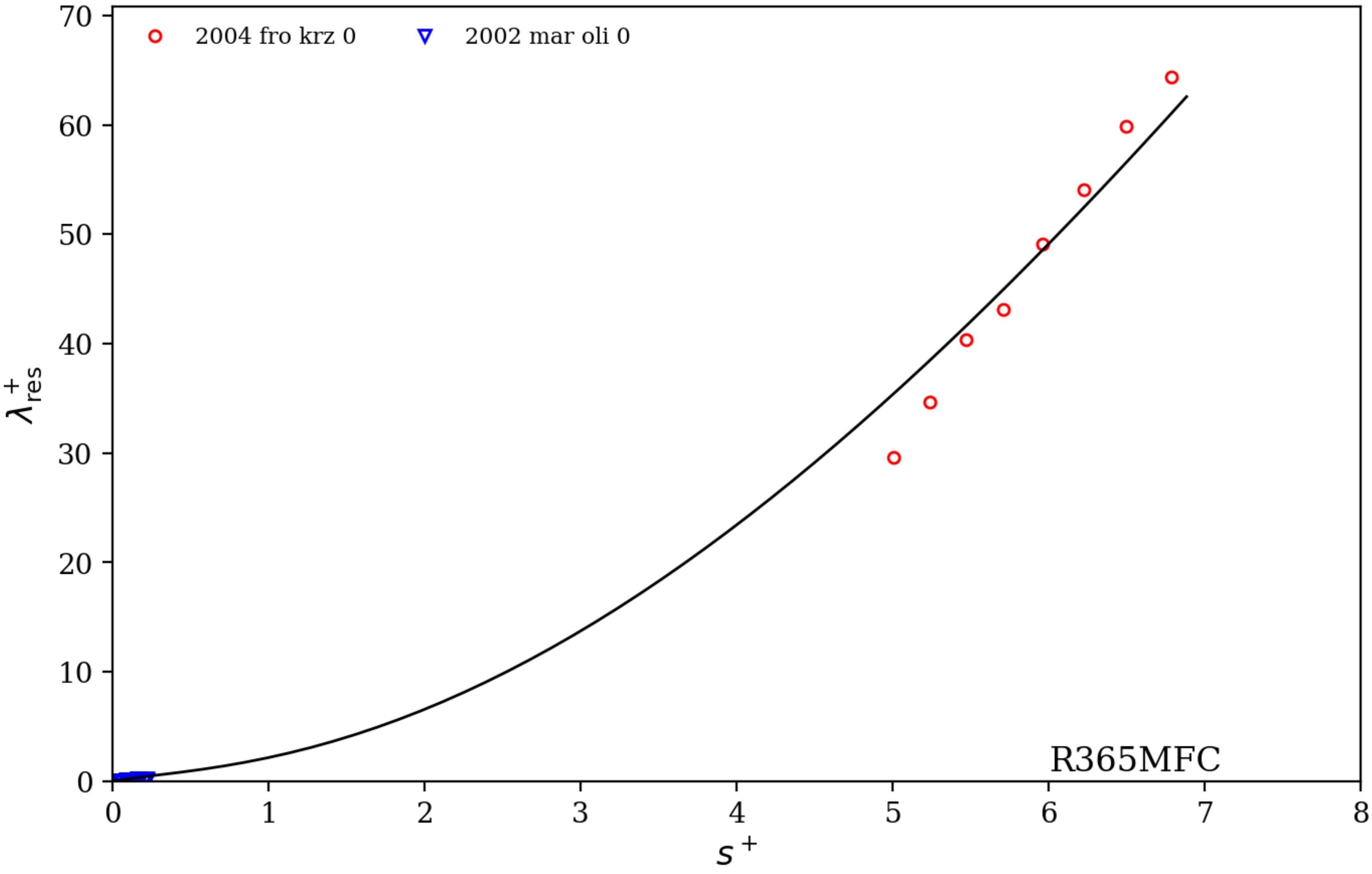

Figure DPR1. R365MFC

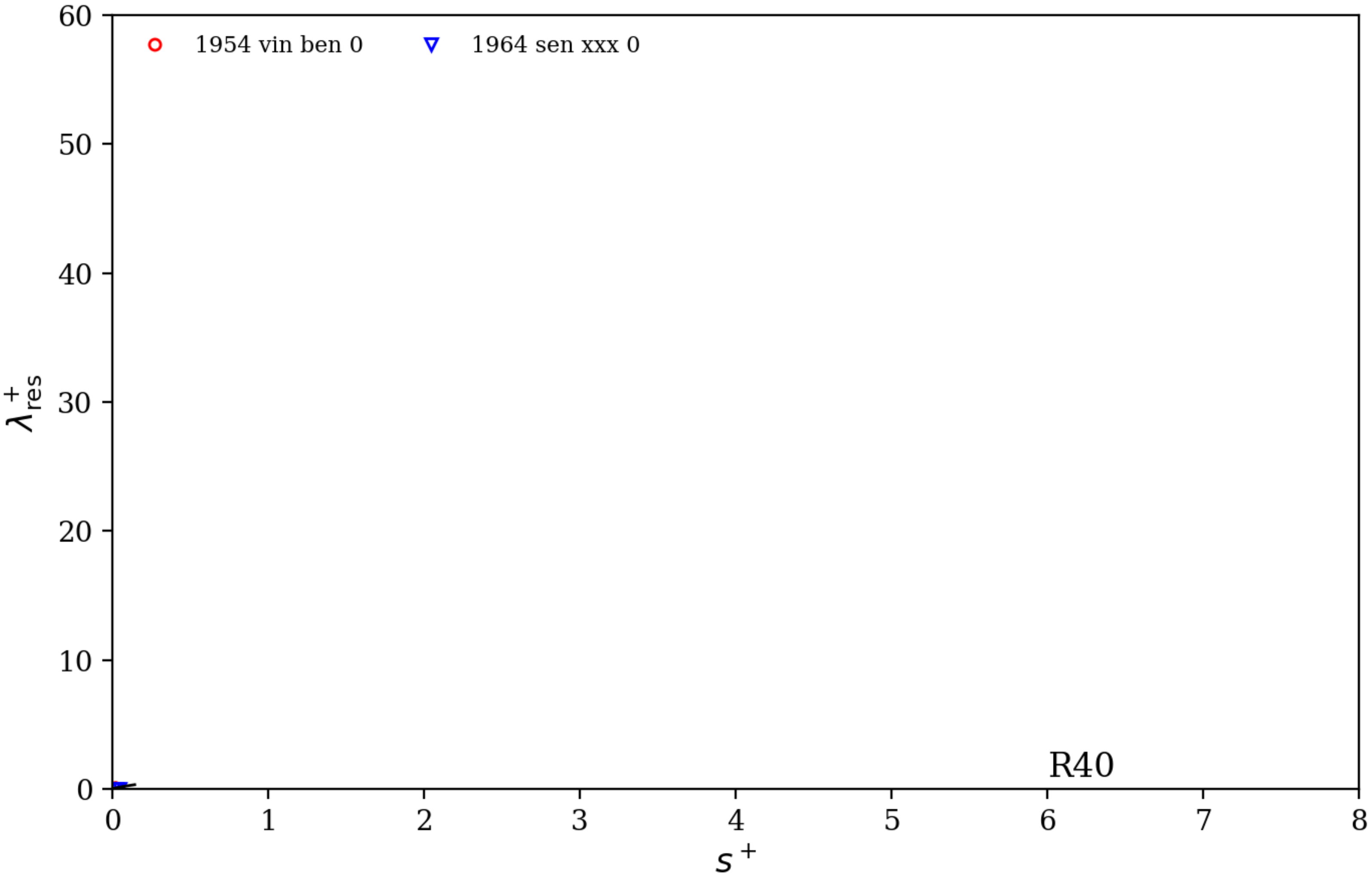

Figure DPR1. R40

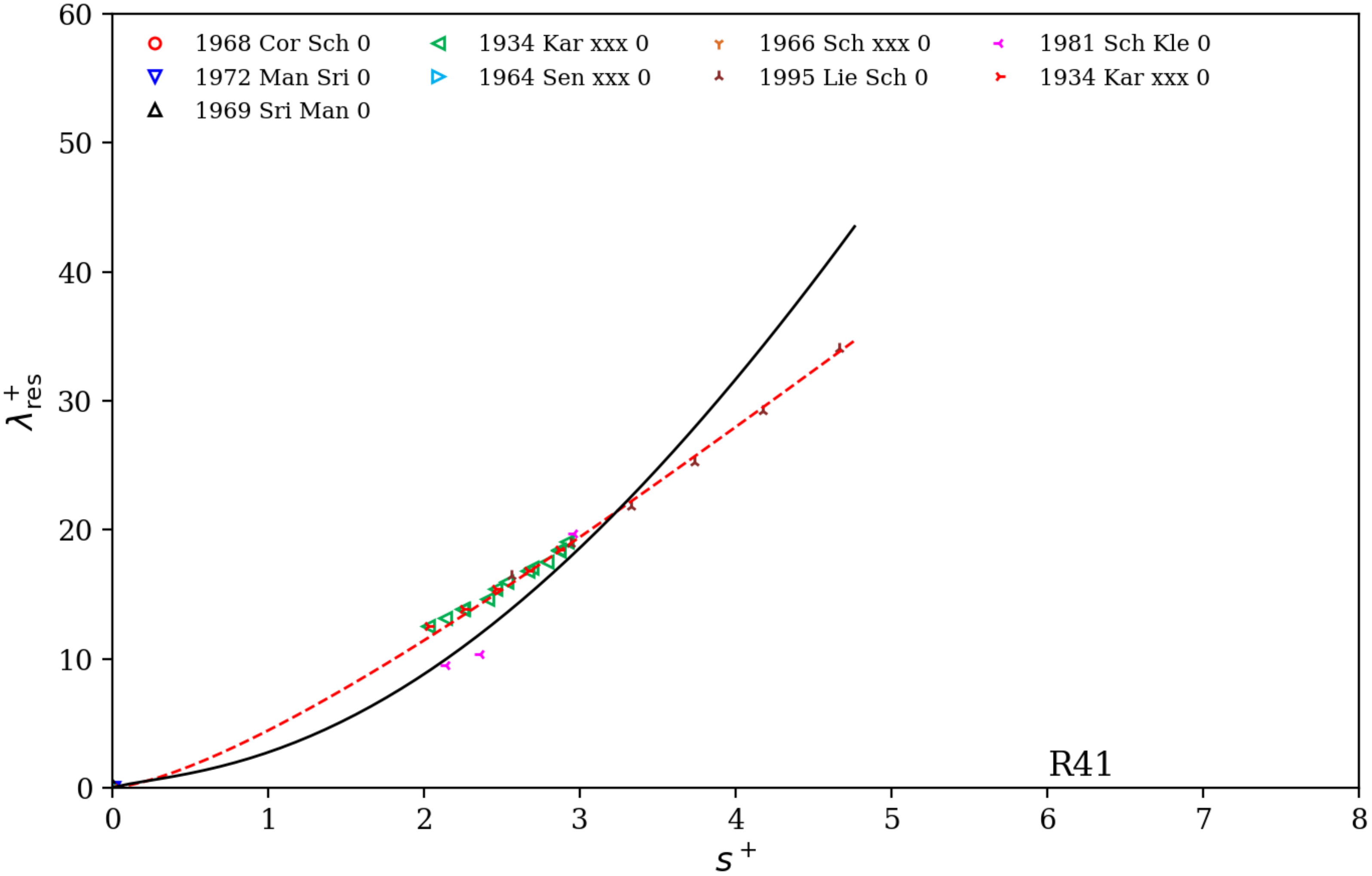

Figure DPR1. R41

Figure DPR1. CO_2

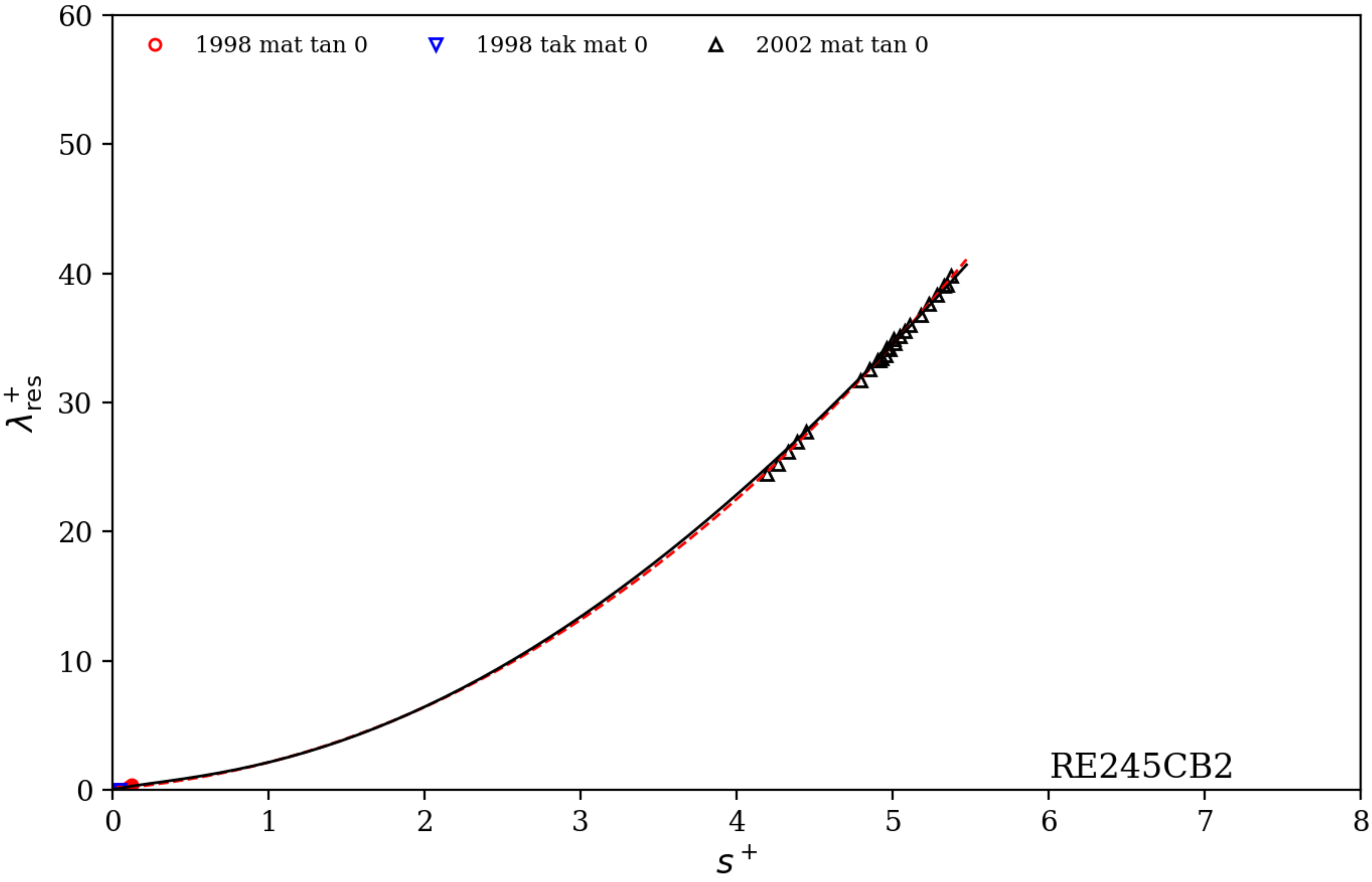

Figure DPR1. RE245CB2

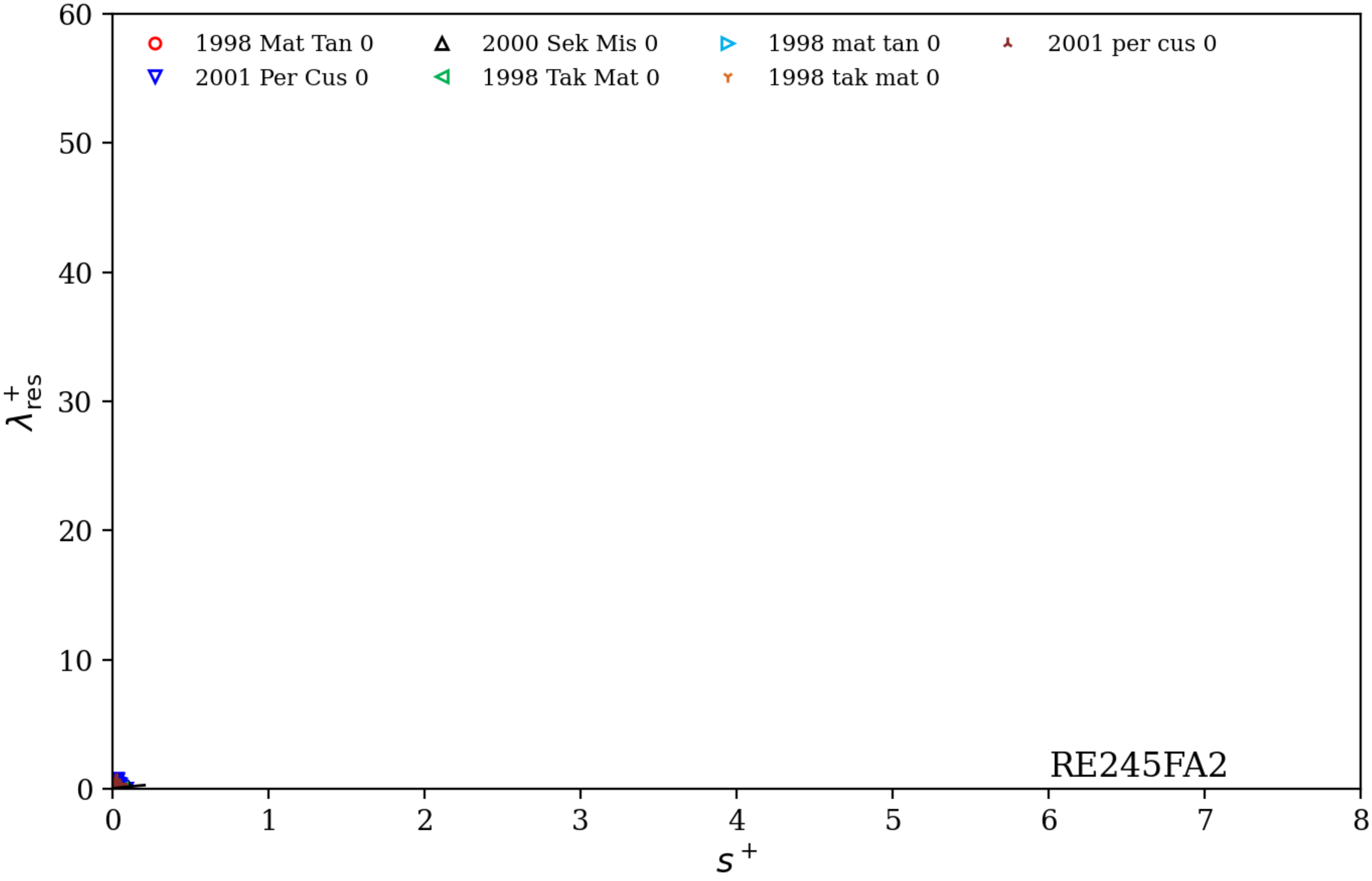

Figure DPR1. RE245FA2

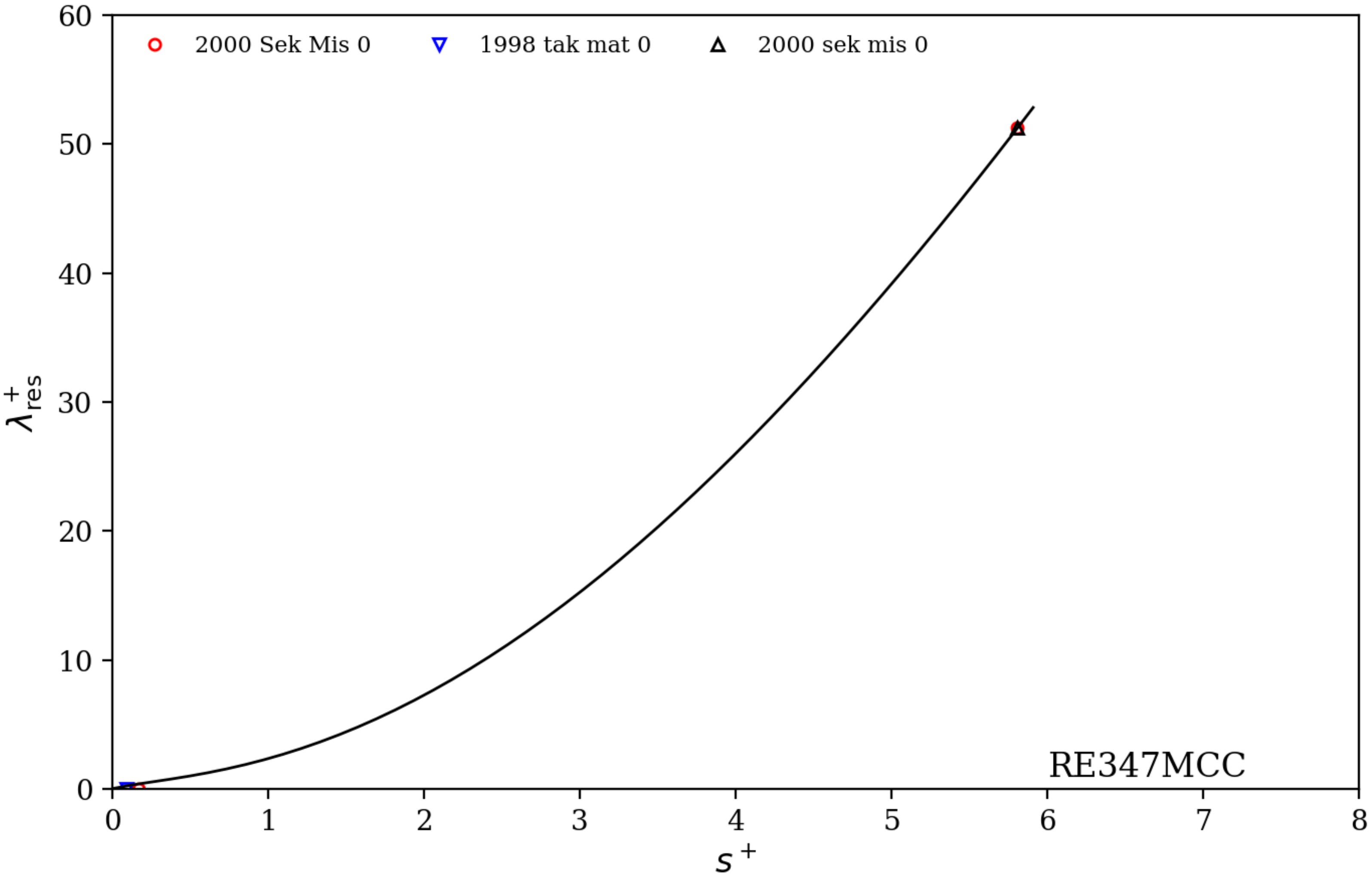

Figure DPR1. RE347MCC

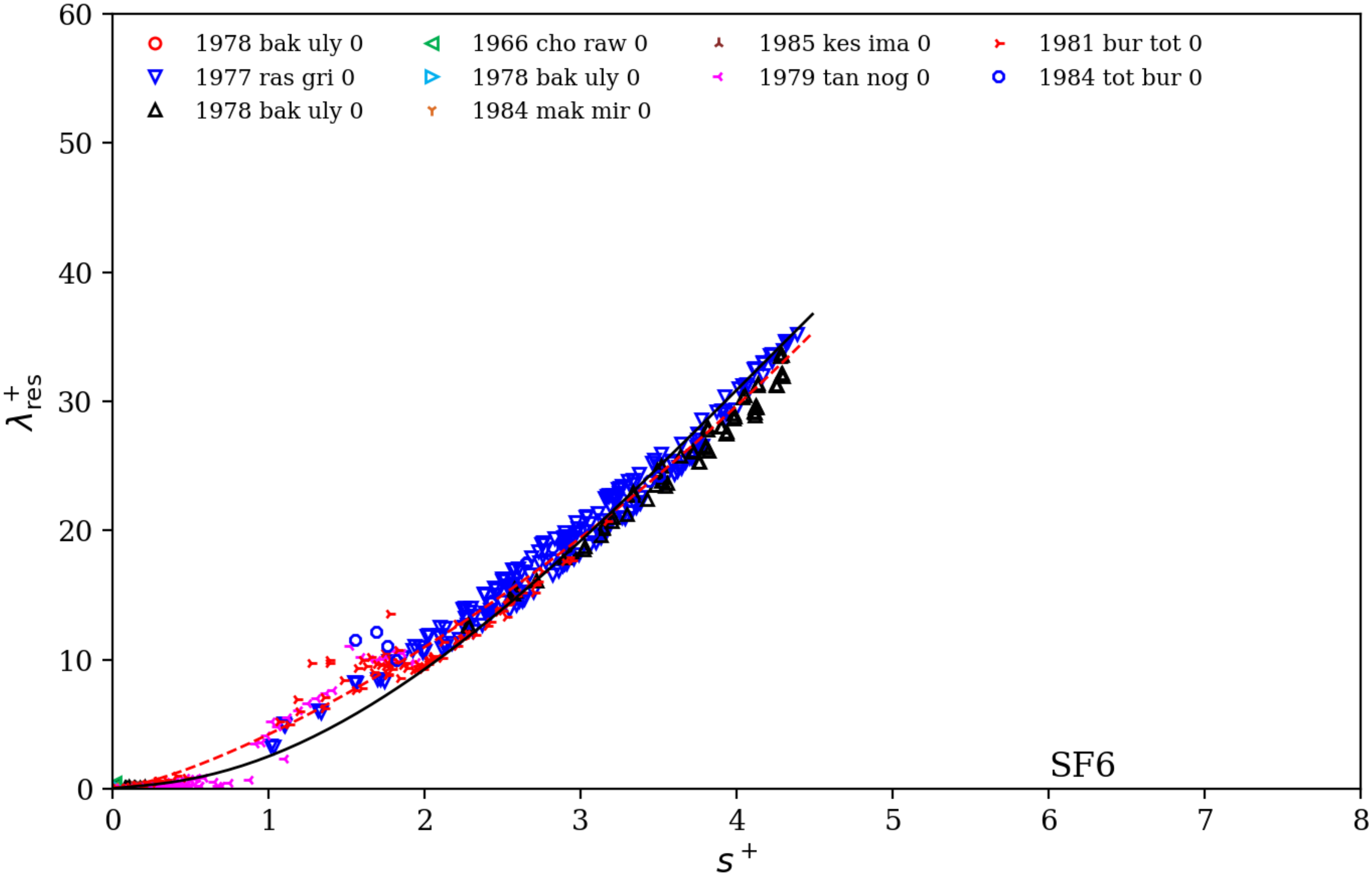

Figure DPR1. SF6

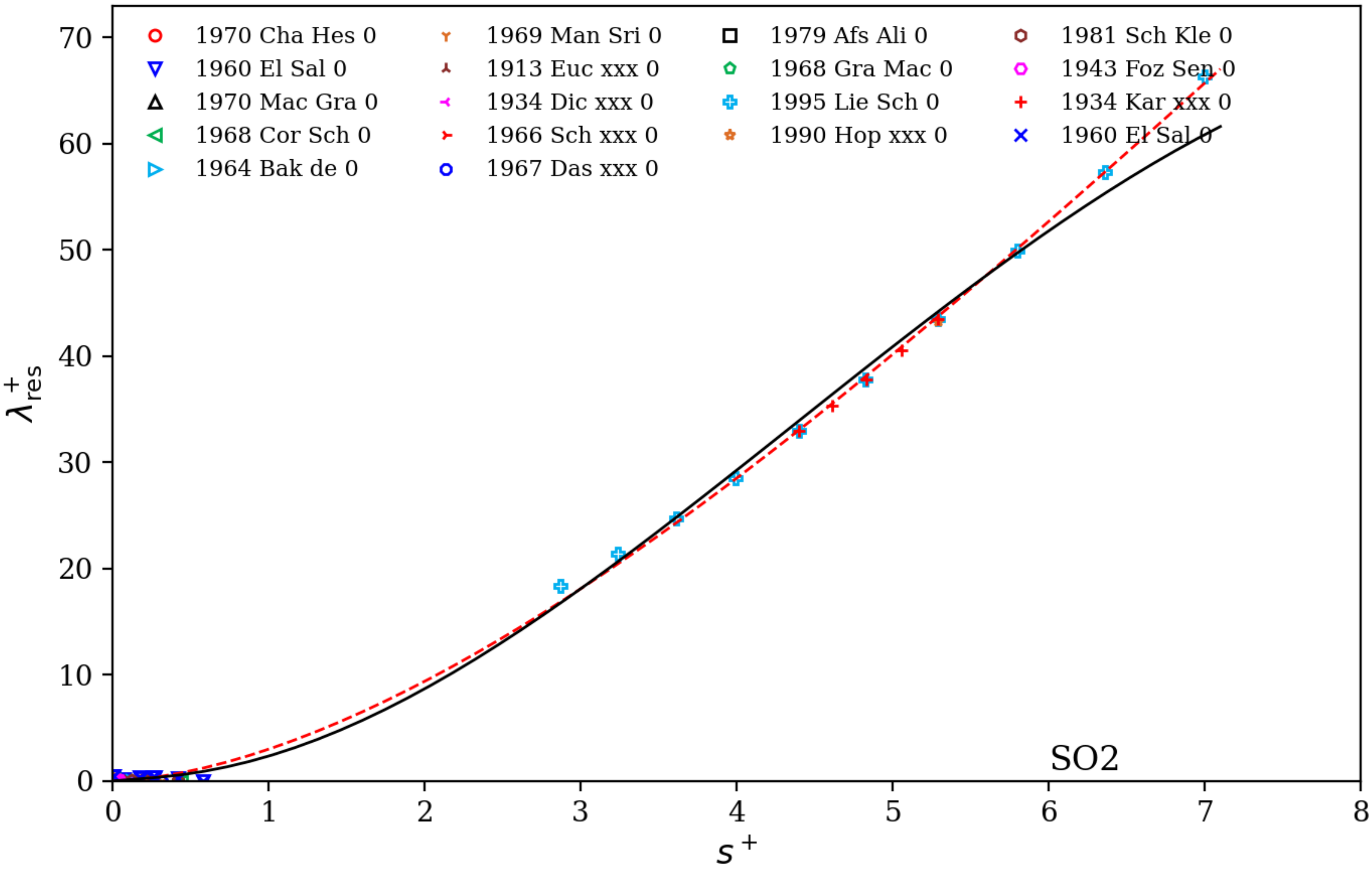

Figure DPR1. SO2

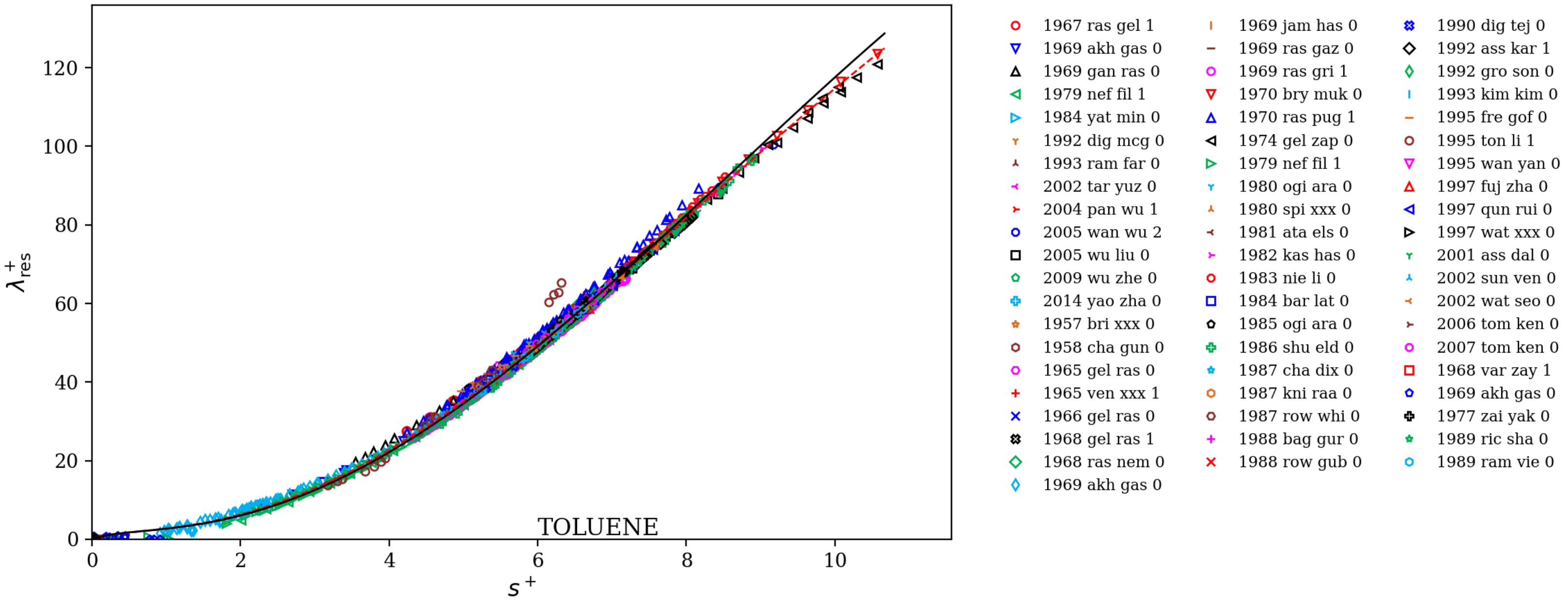

Figure DPR1. TOLUENE

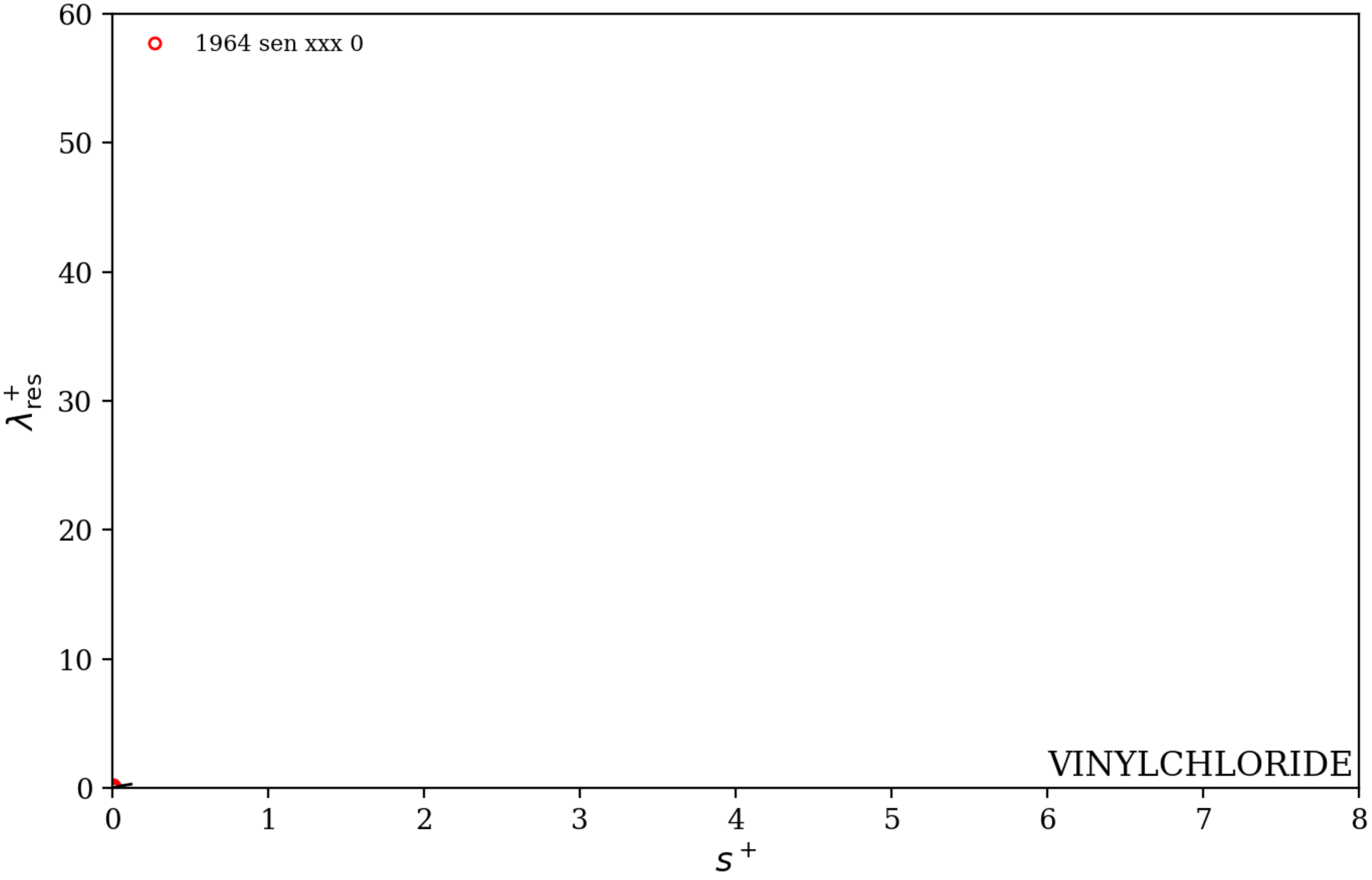

Figure DPR1. VINYLCHLORIDE

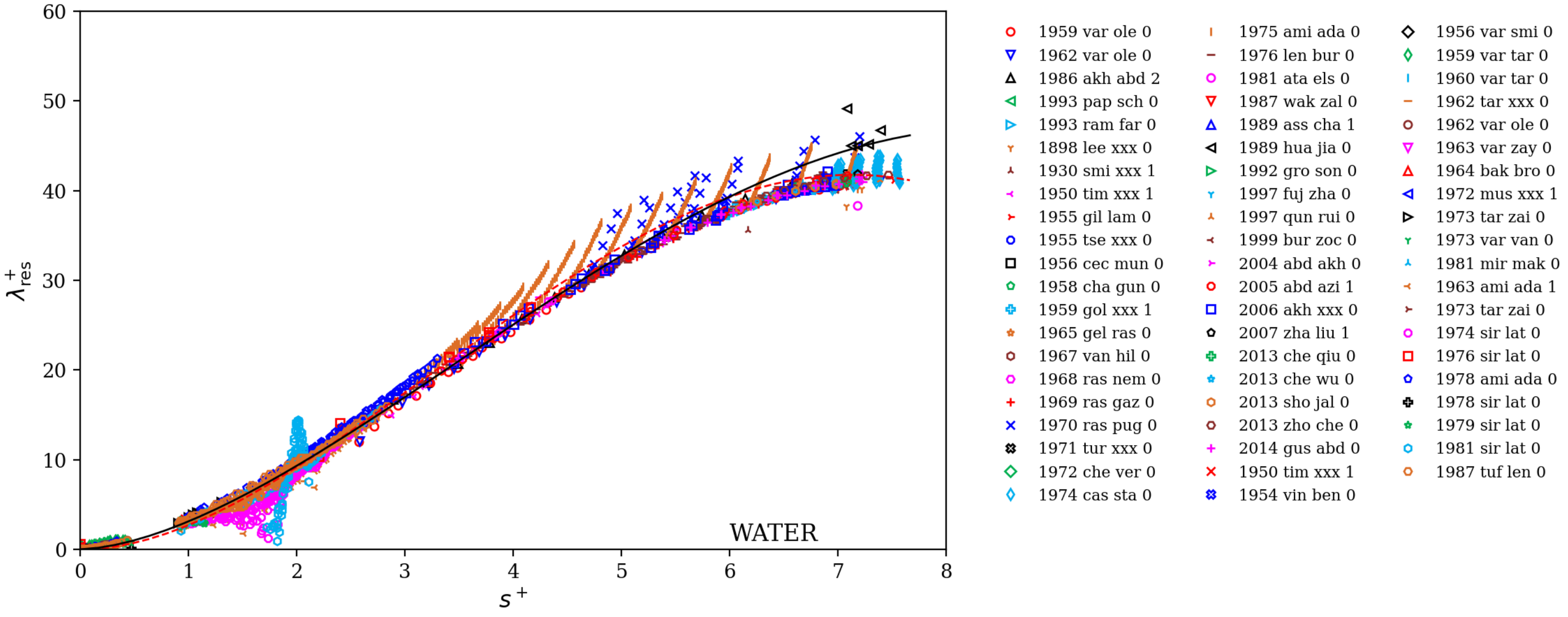

Figure DPR1. WATER

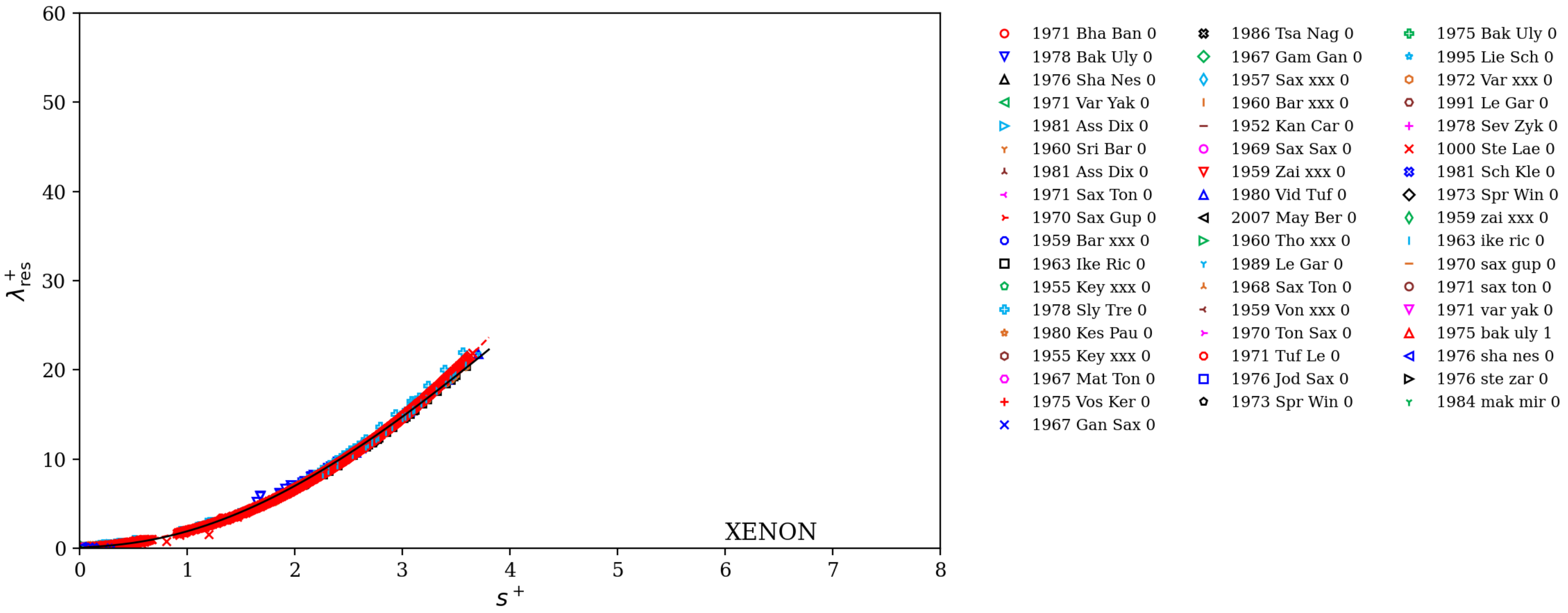

Figure DPR1. XENON

2. Relative deviation from experimental values of each pure fluid to models

In this section, relative deviation of the experimental thermal conductivity λ_{exp} from values λ_{RES} calculated with the RES model and the best-selected models in REFPORP 10.0 are presented for each pure fluid. The legends denote the sources of the data, showing the published year, the first three letters of the first author's family name, and the first three letters of the second author's family name (' xxx' if there is no second author). The full citations are given at the Reference section.

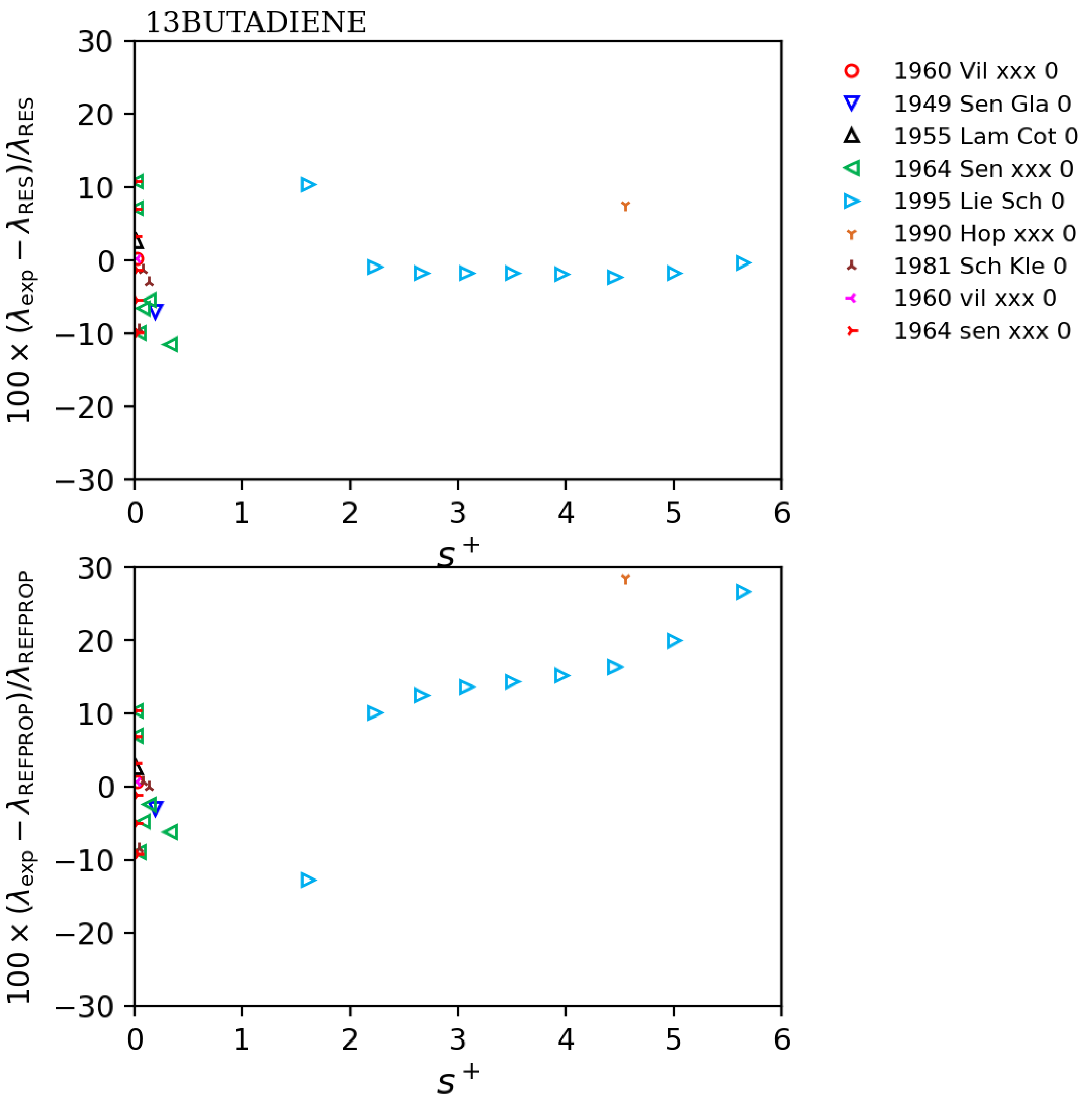

Figure DPR2. 13BUTADIENE

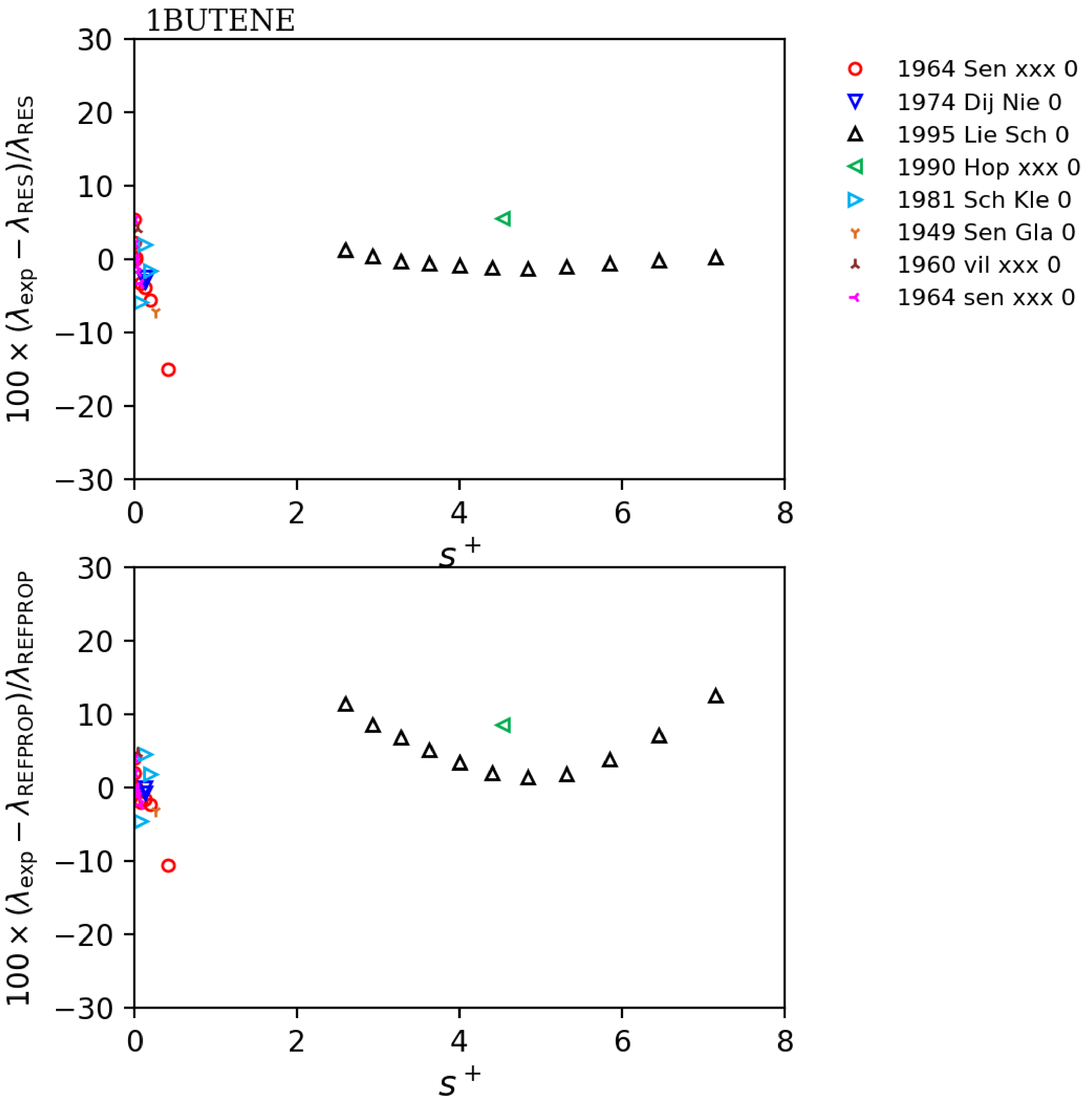

Figure DPR2. 1BUTENE

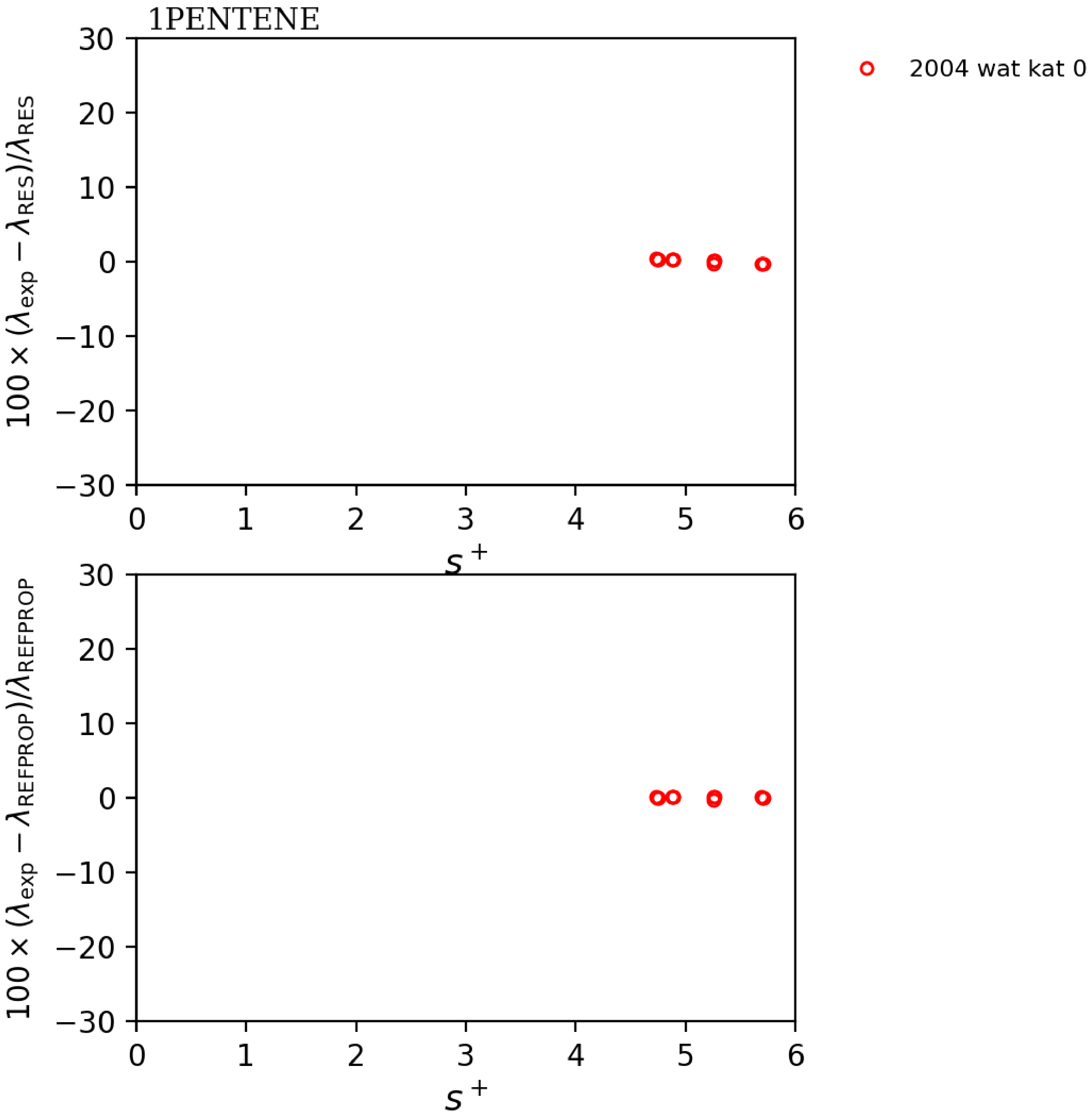

Figure DPR2. 1PENTENE

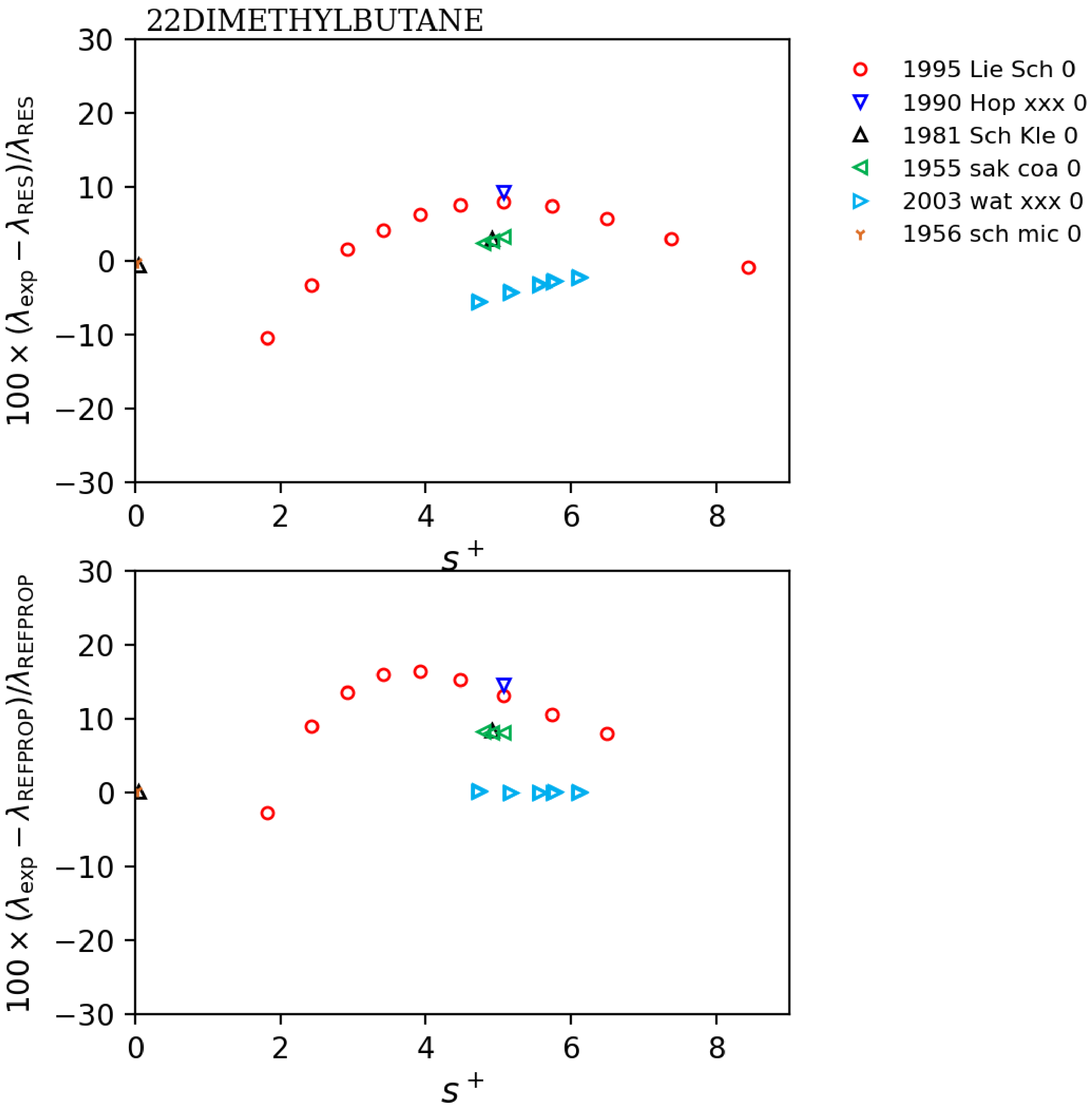

Figure DPR2. 22DIMETHYLBUTANE

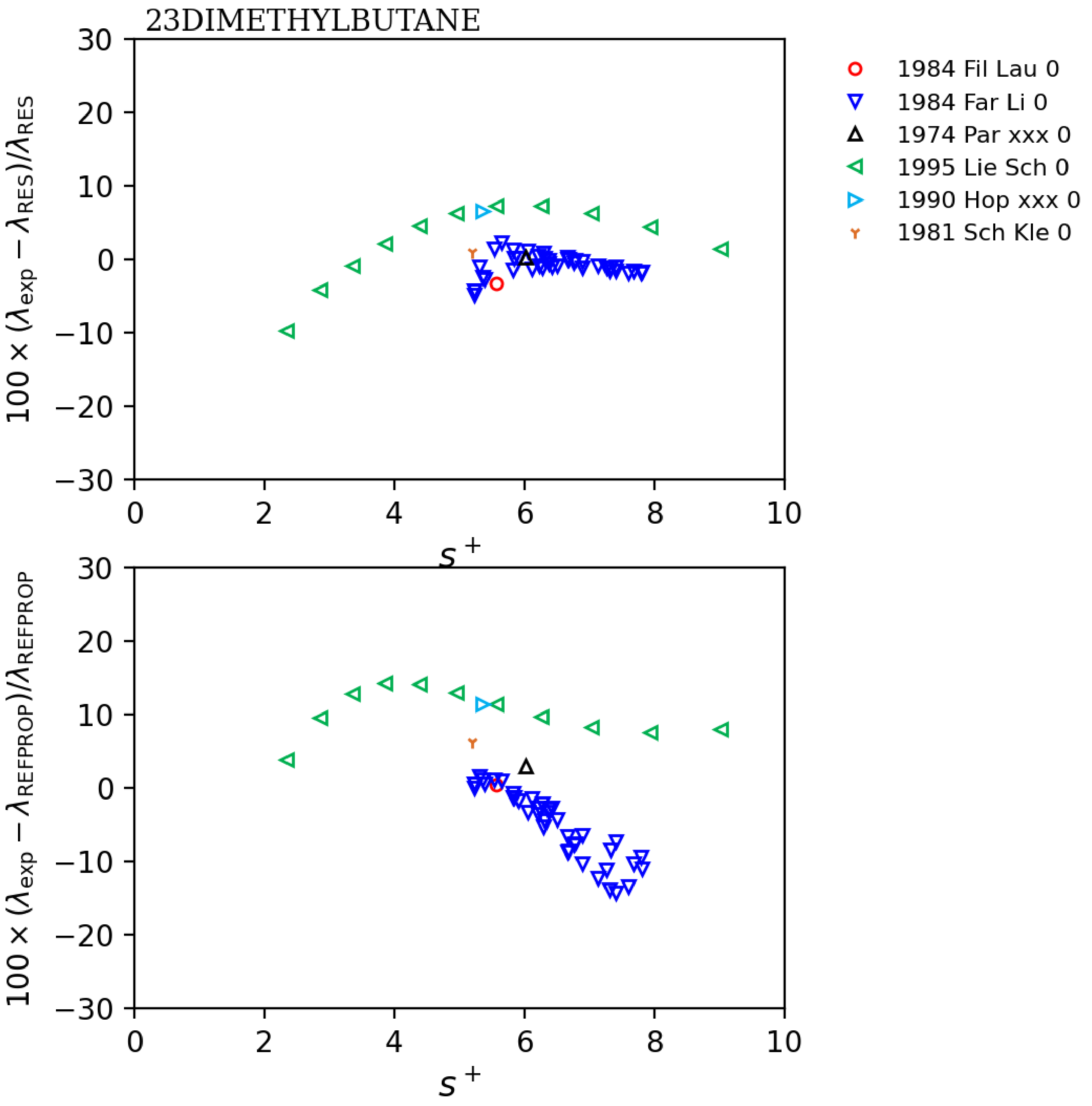

Figure DPR2. 23DIMETHYLBUTANE

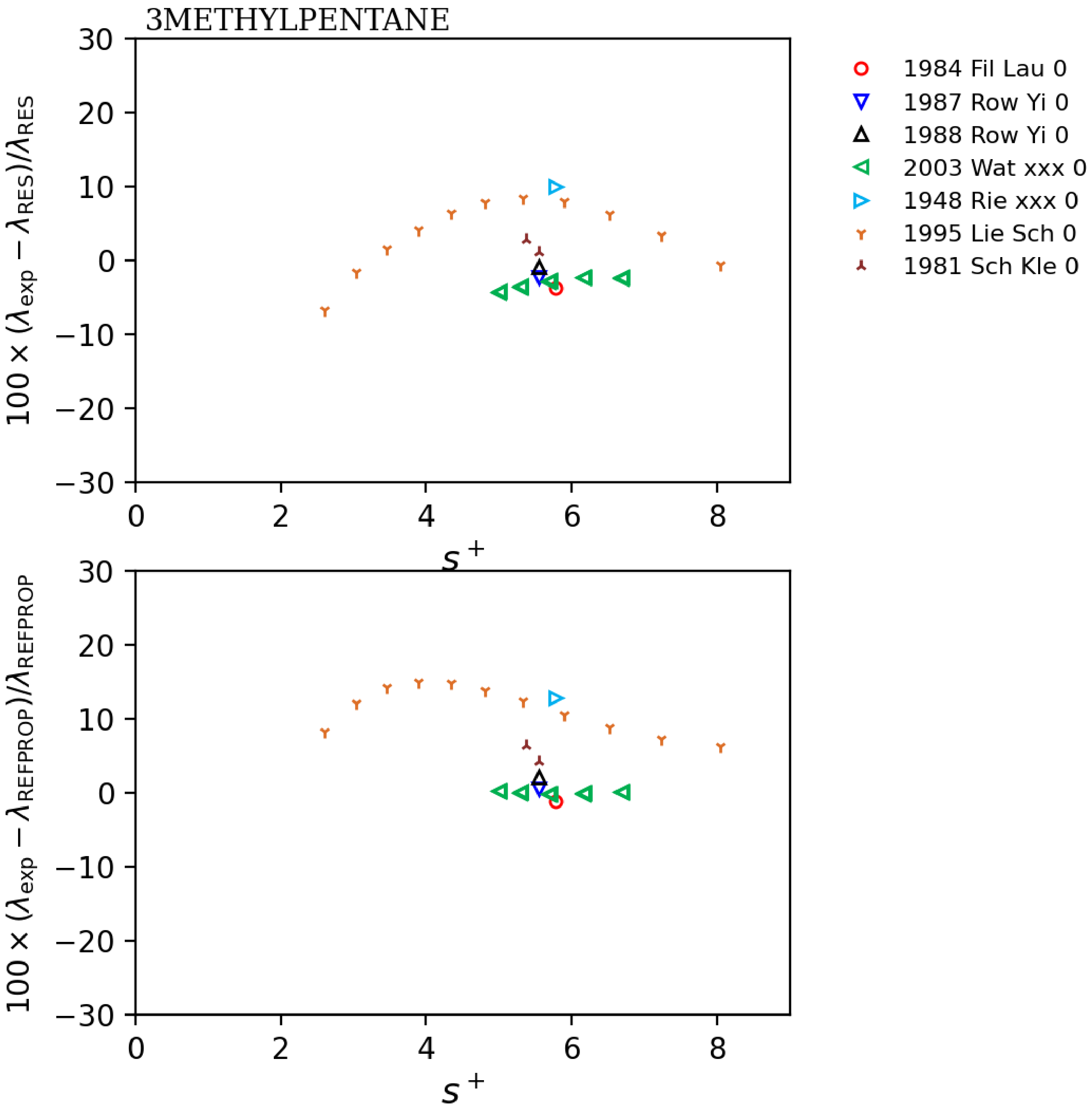

Figure DPR2. 3METHYLPENTANE

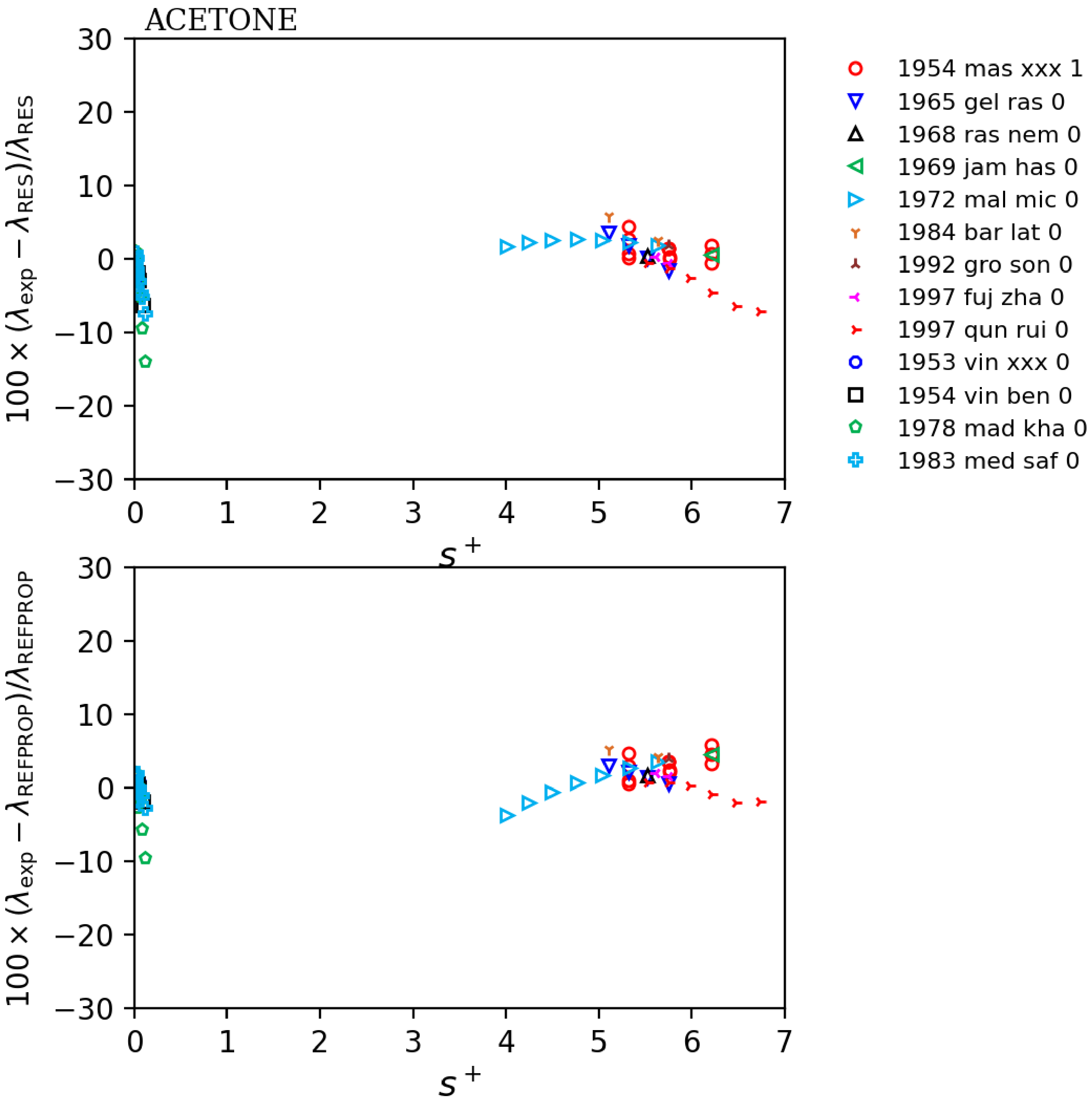

Figure DPR2. ACETONE

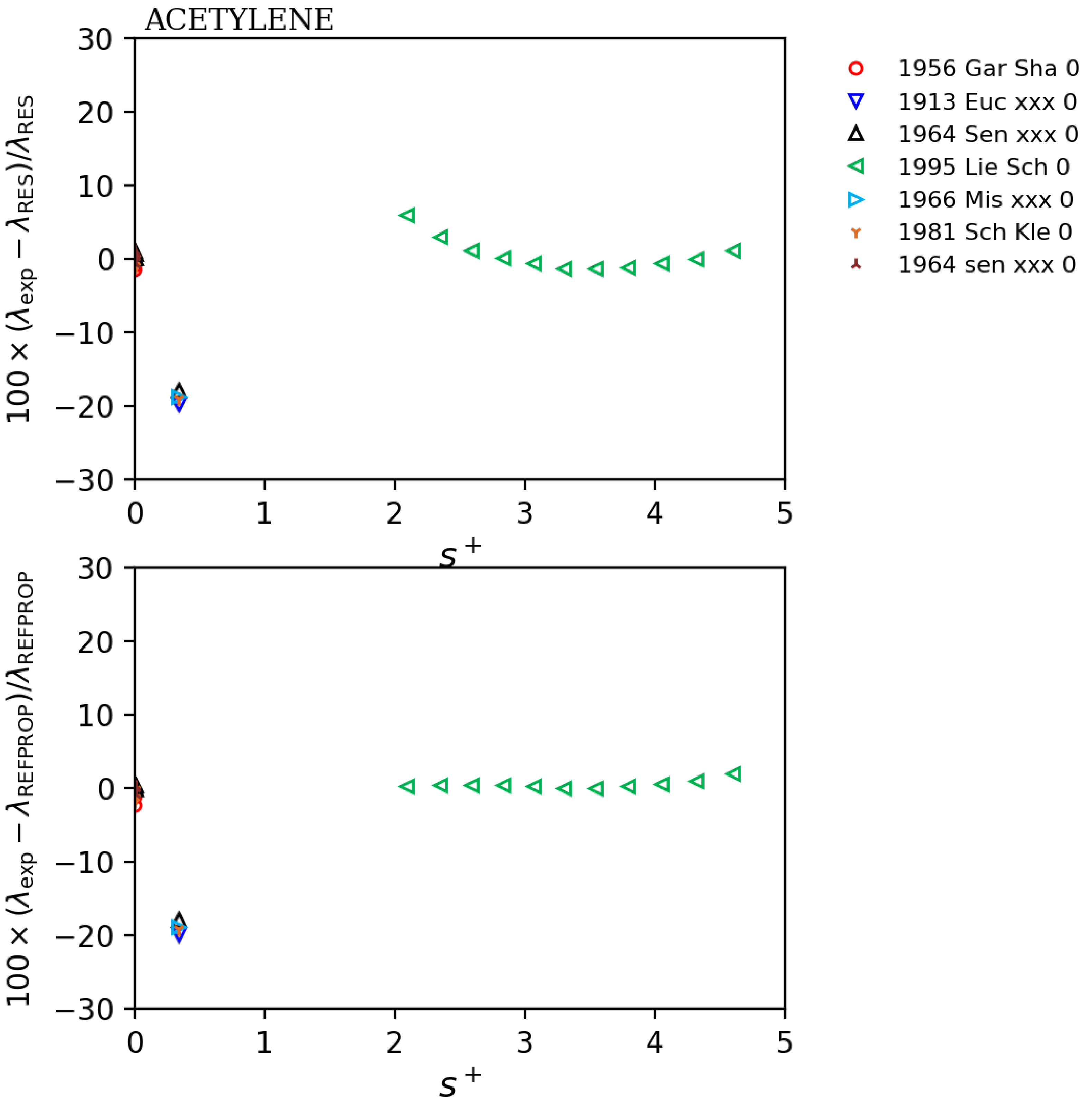

Figure DPR2. ACETYLENE

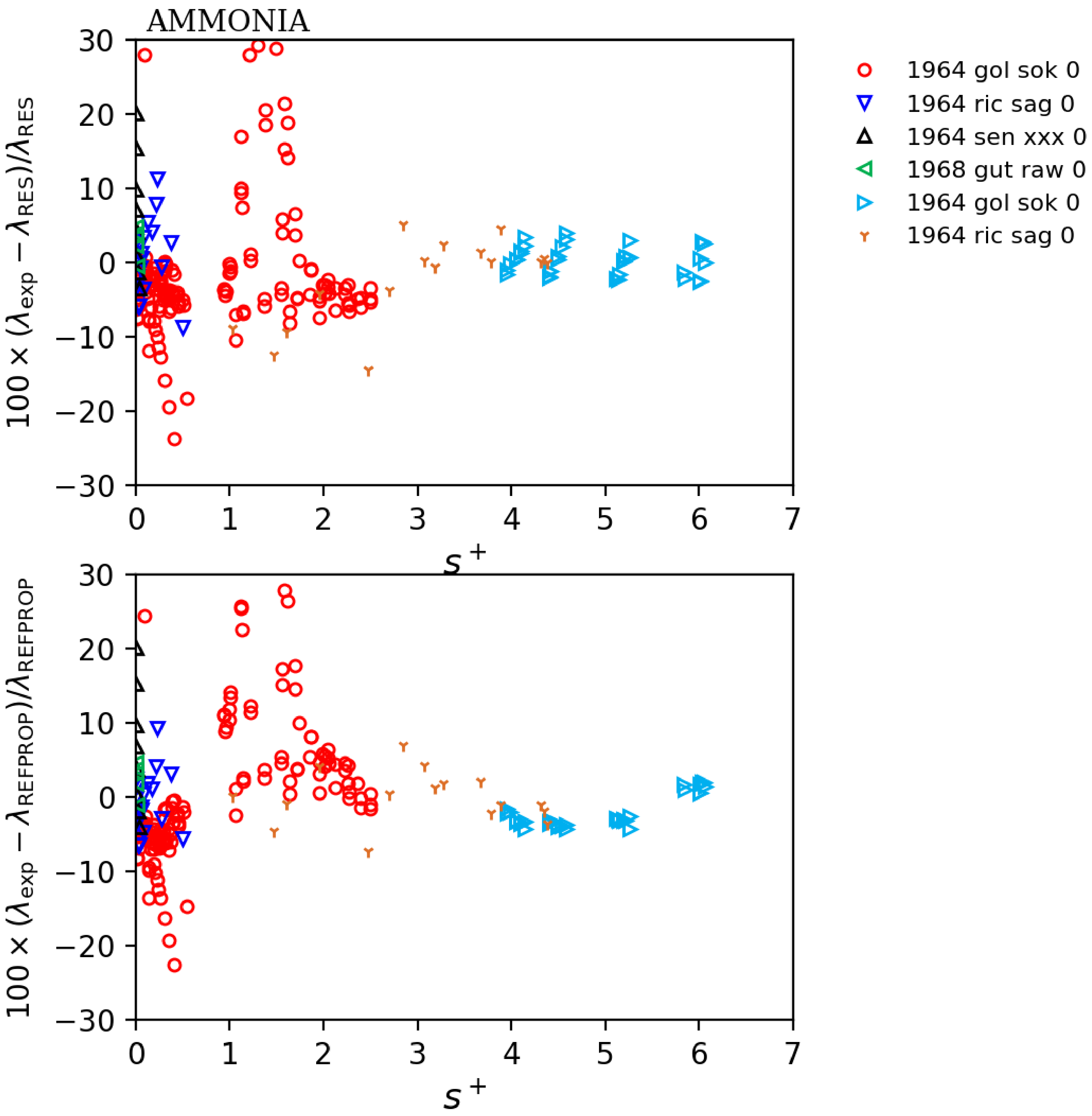

Figure DPR2. AMMONIA

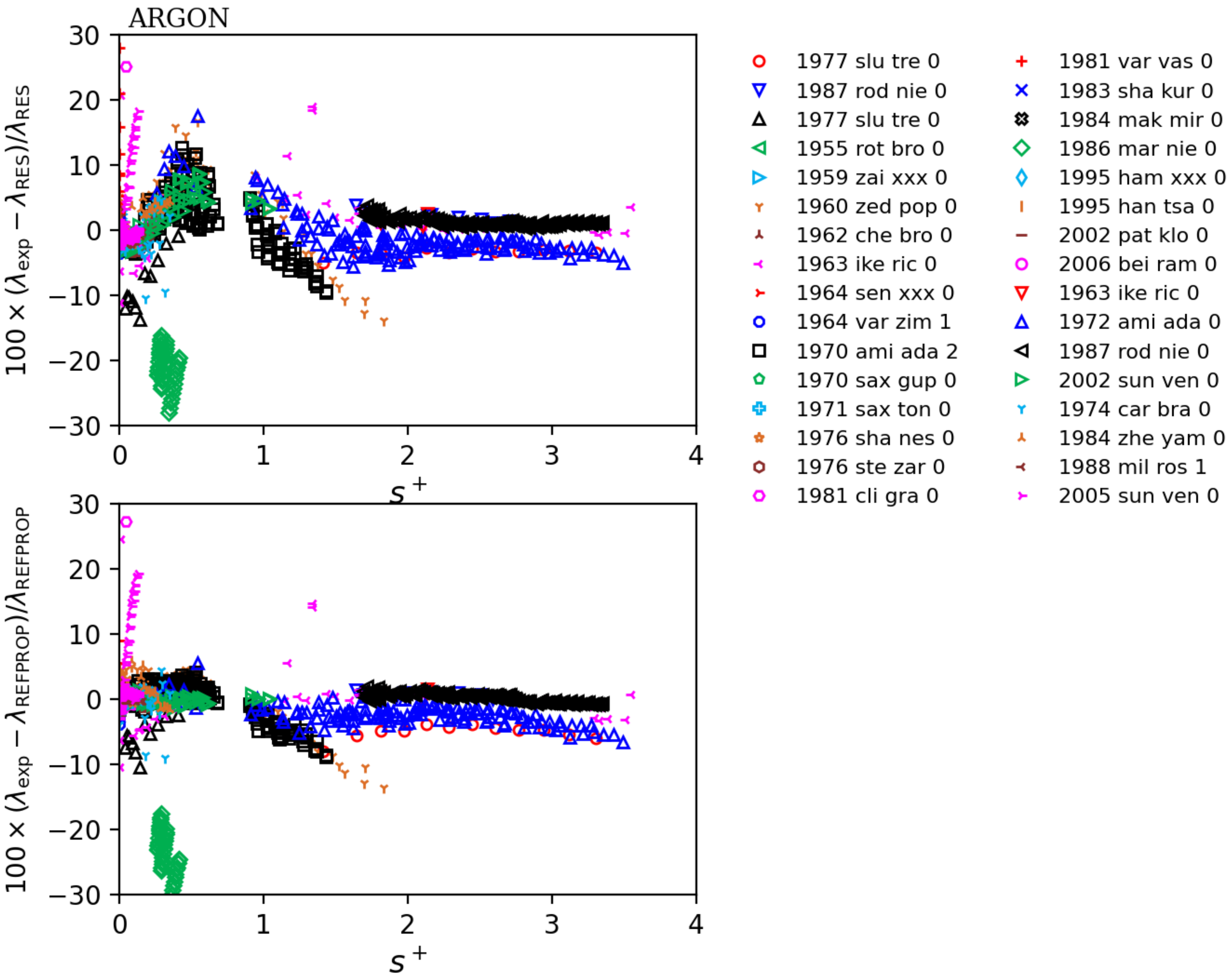

Figure DPR2. ARGON

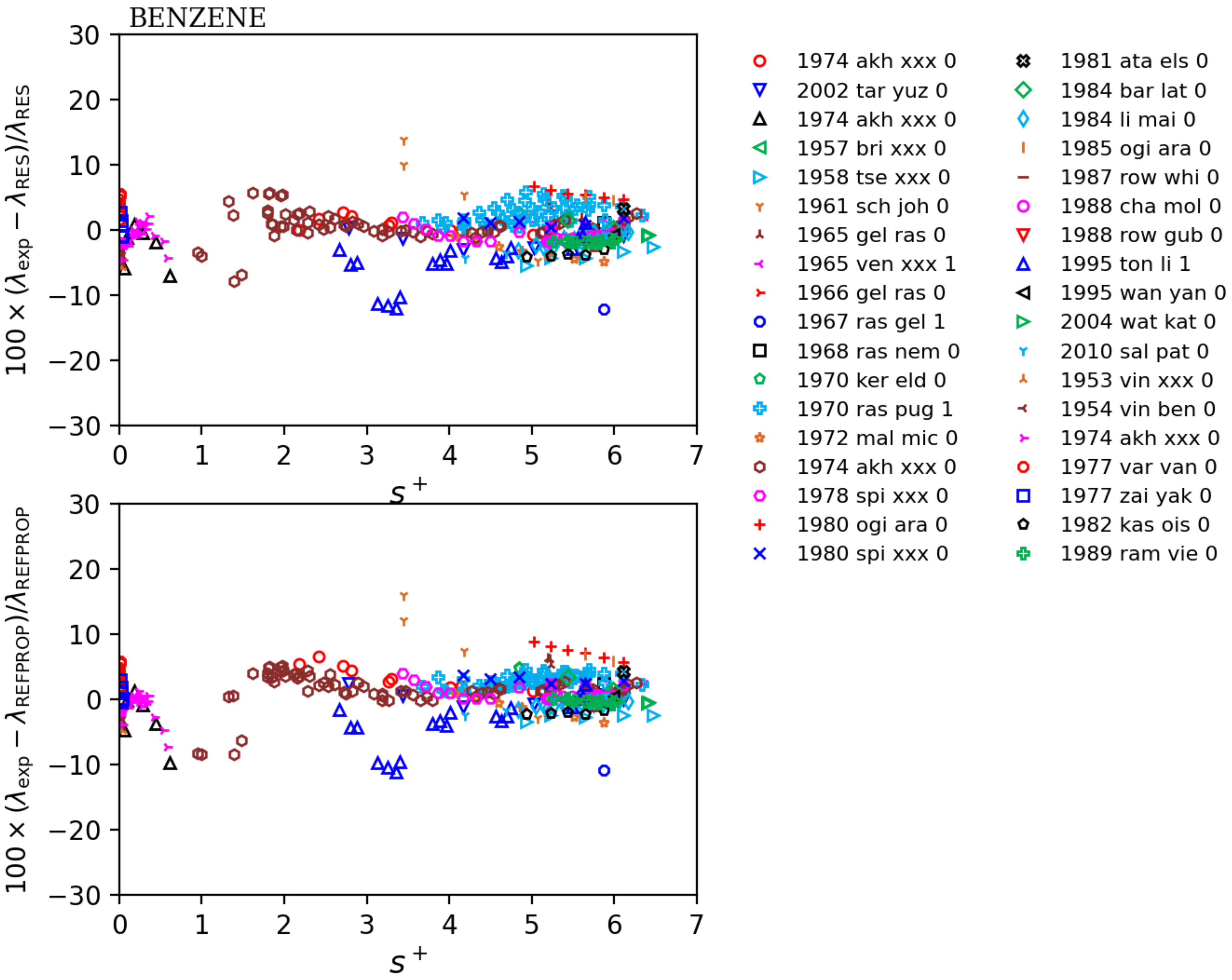

Figure DPR2. BENZENE

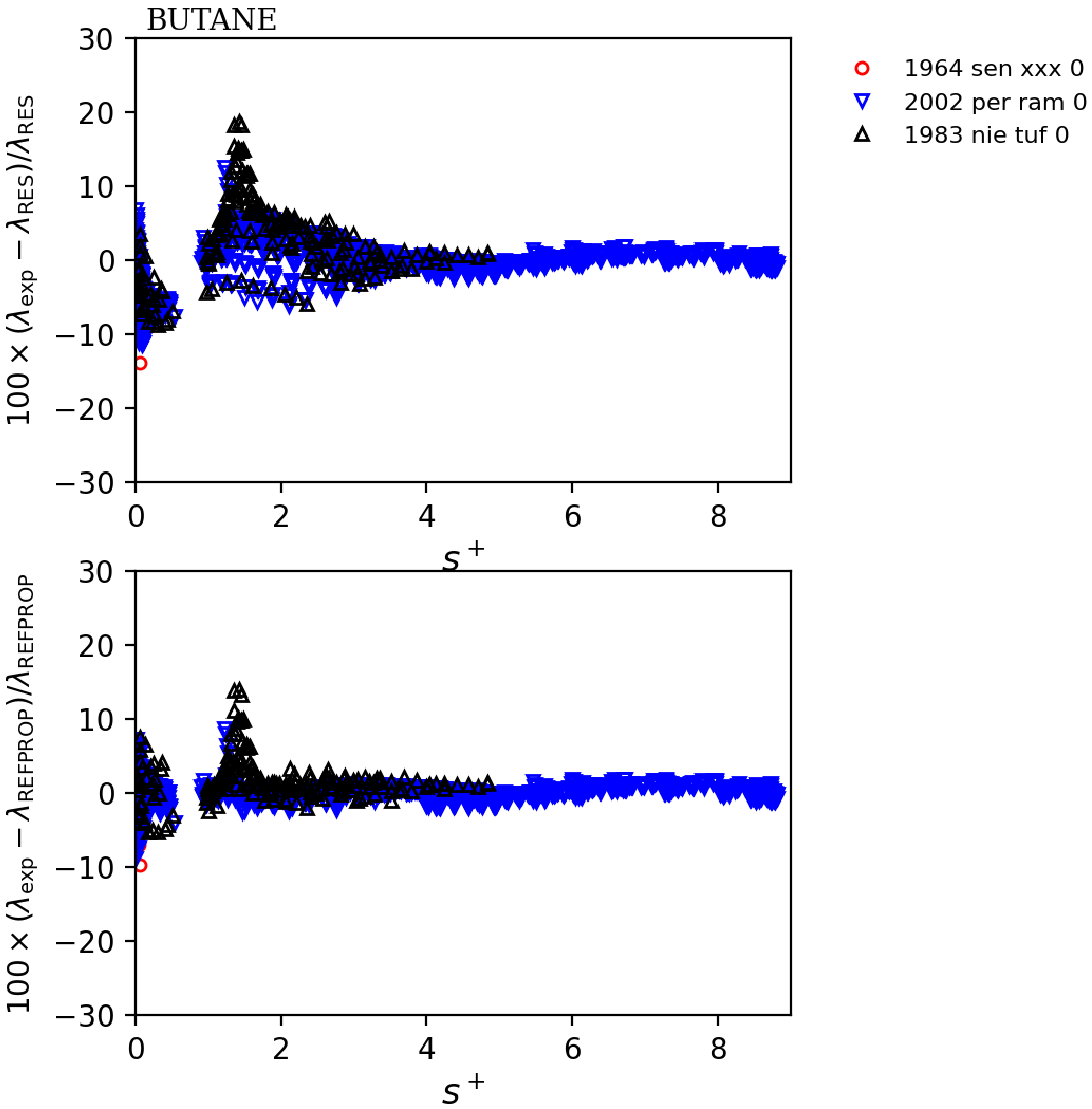

Figure DPR2. BUTANE

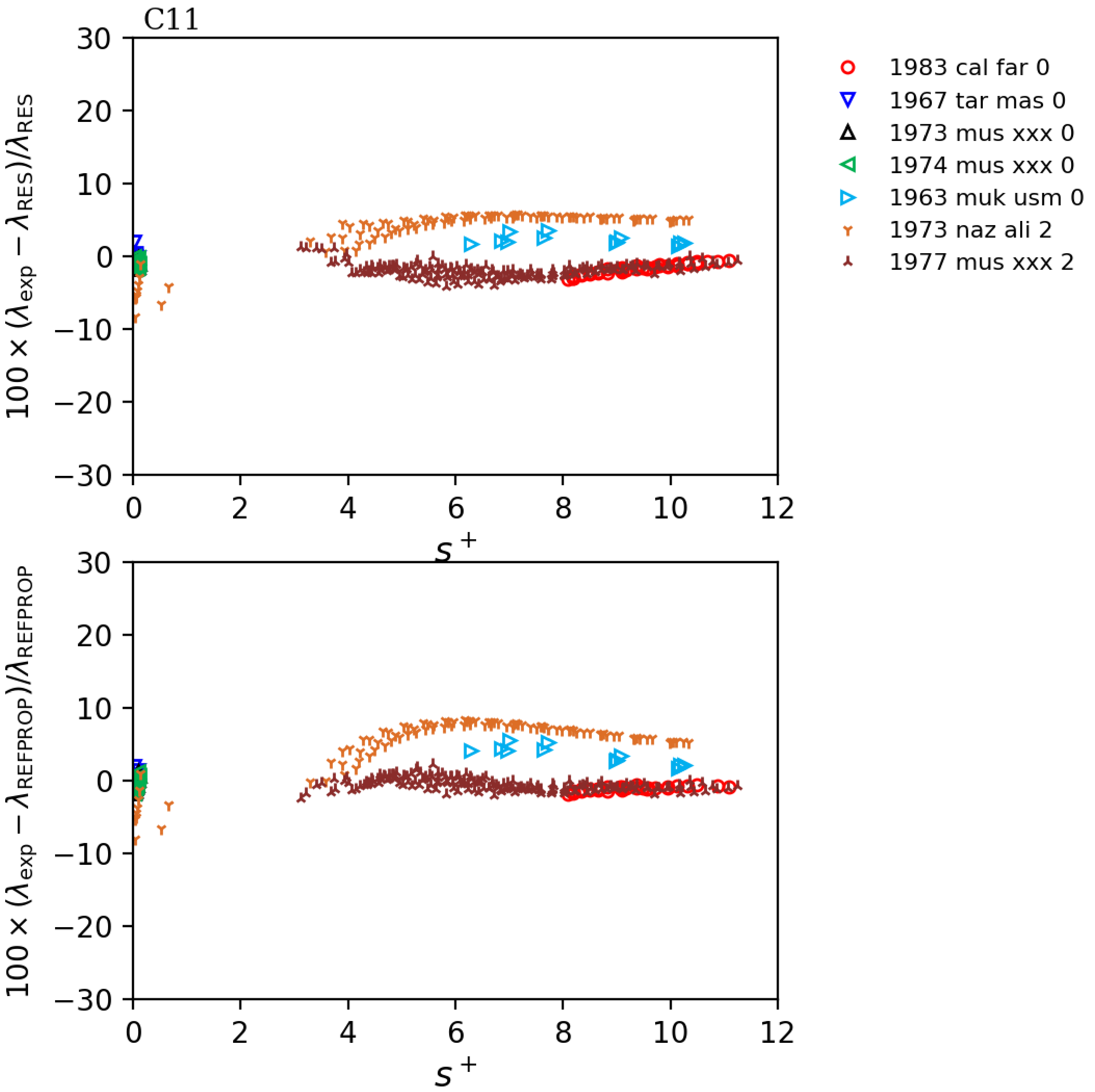

Figure DPR2. C11

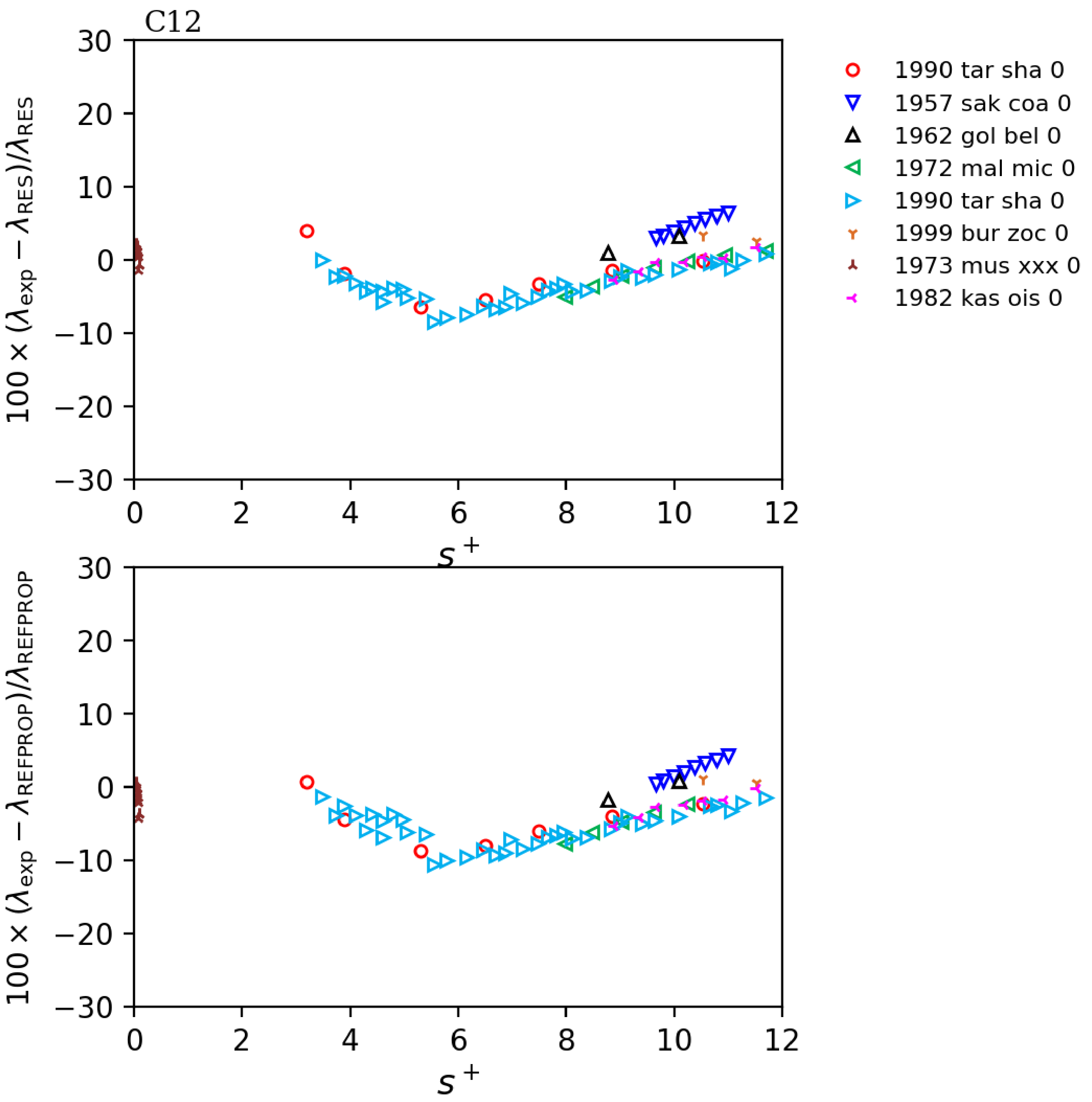

Figure DPR2. C12

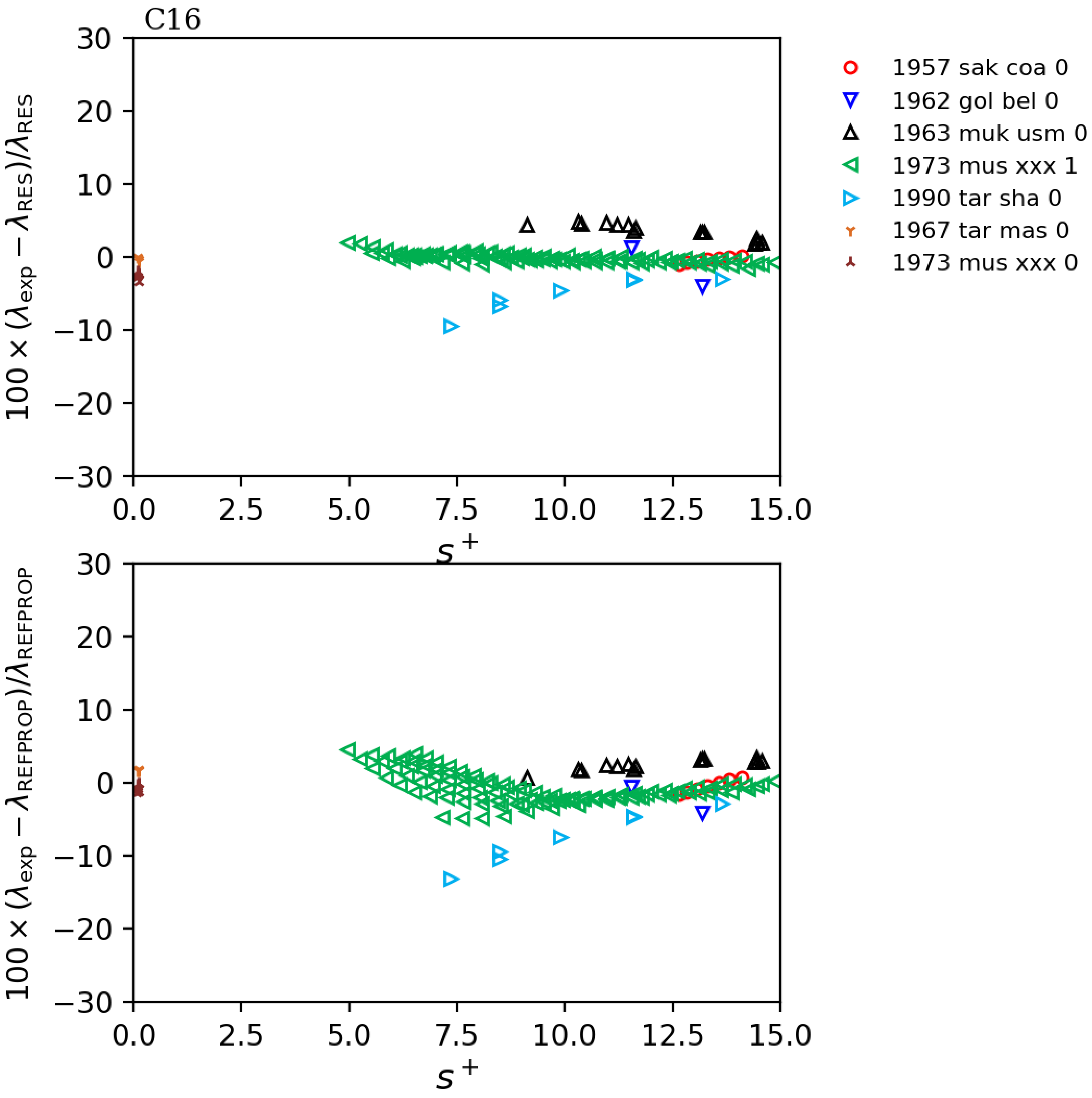

Figure DPR2. C16

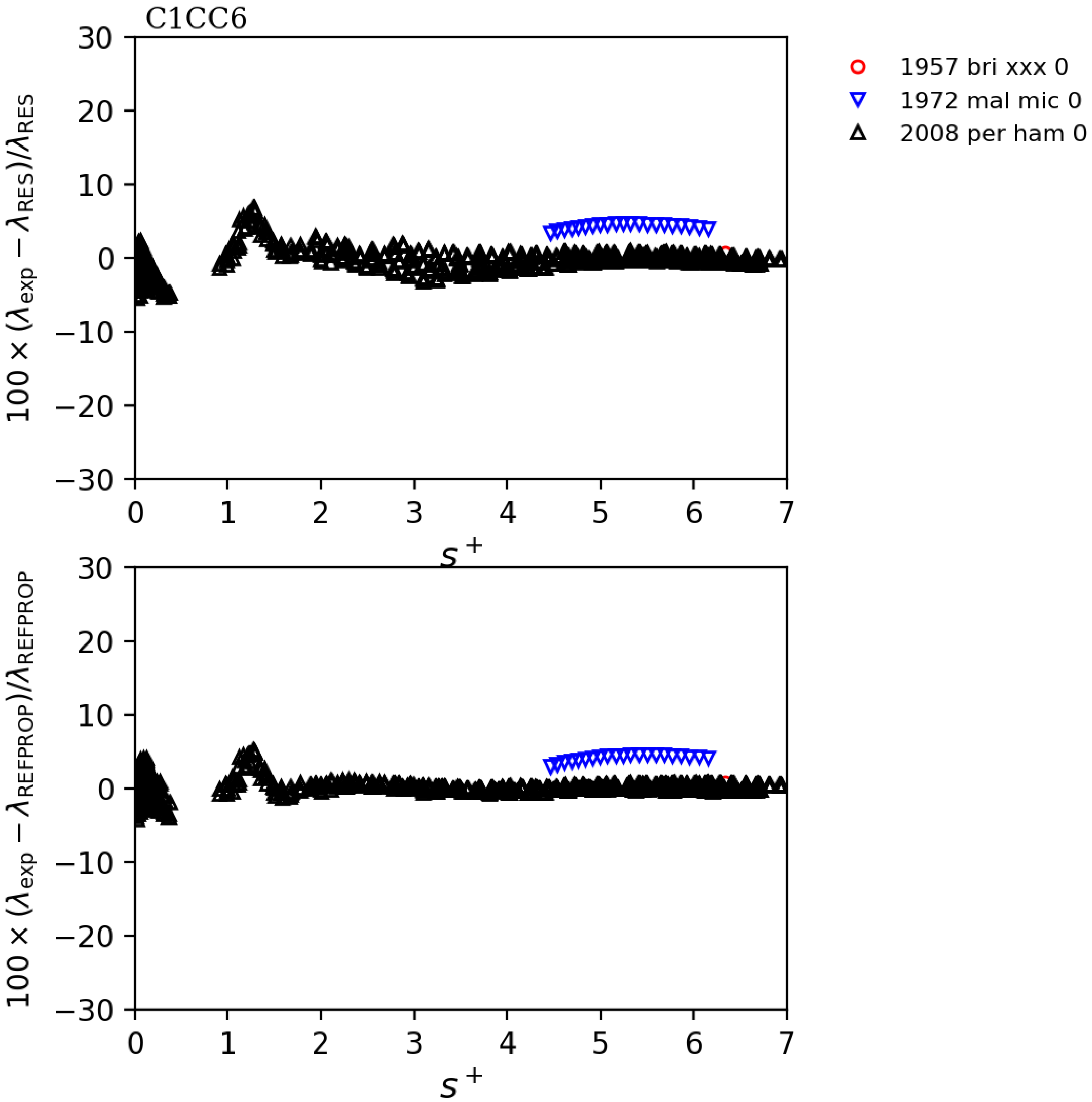

Figure DPR2. C1CC6

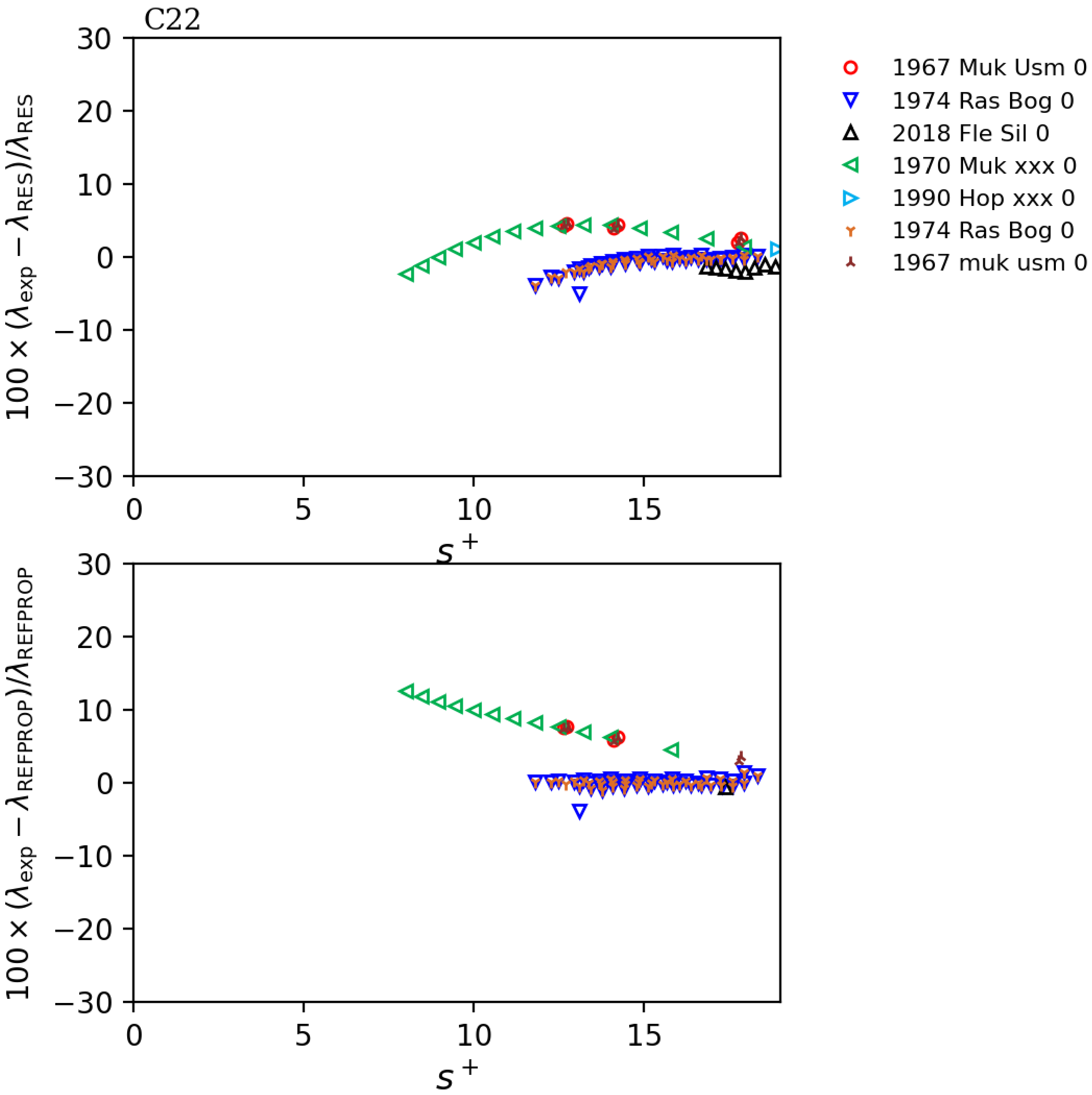

Figure DPR2. C22

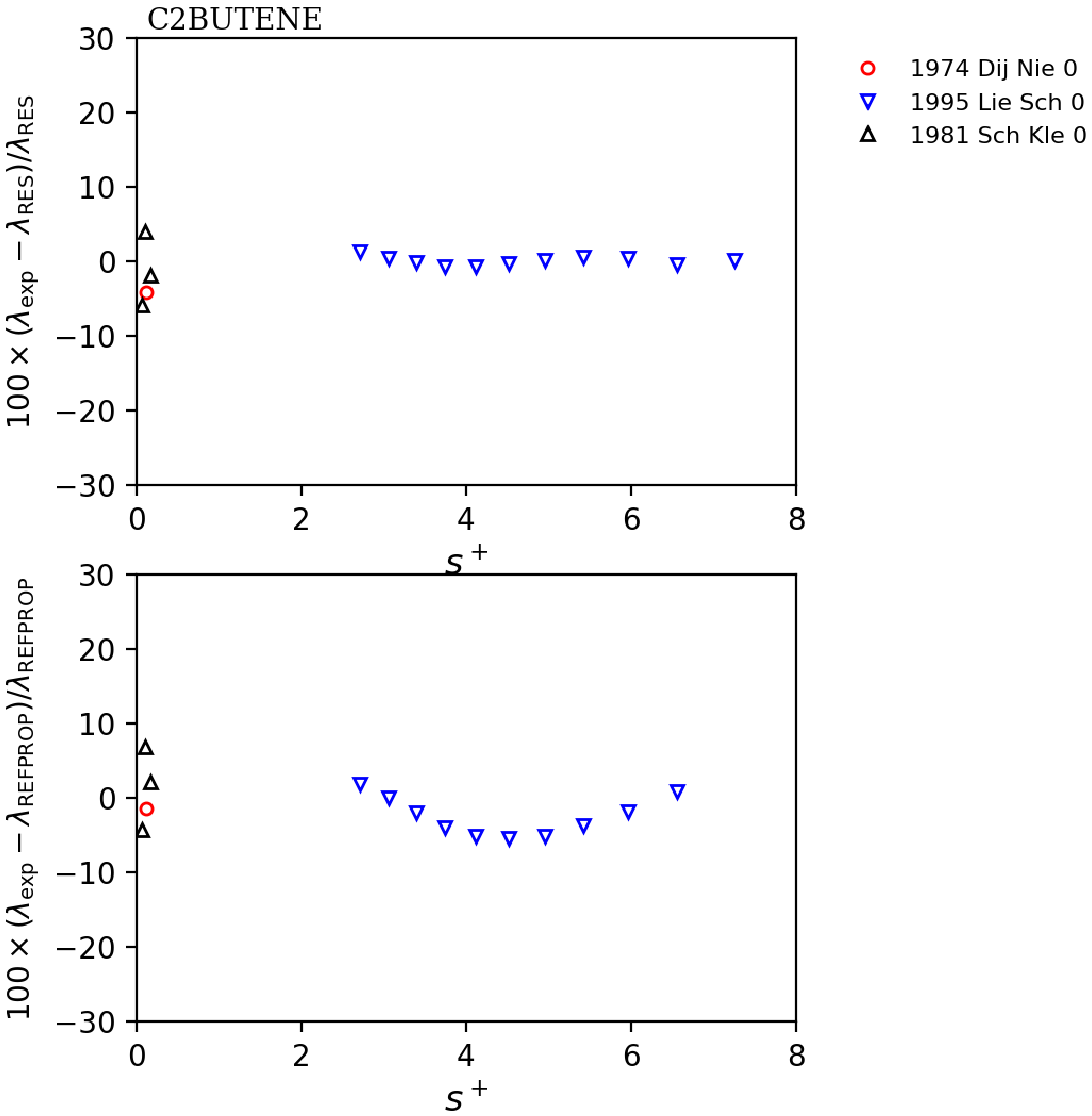

Figure DPR2. C2BUTENE

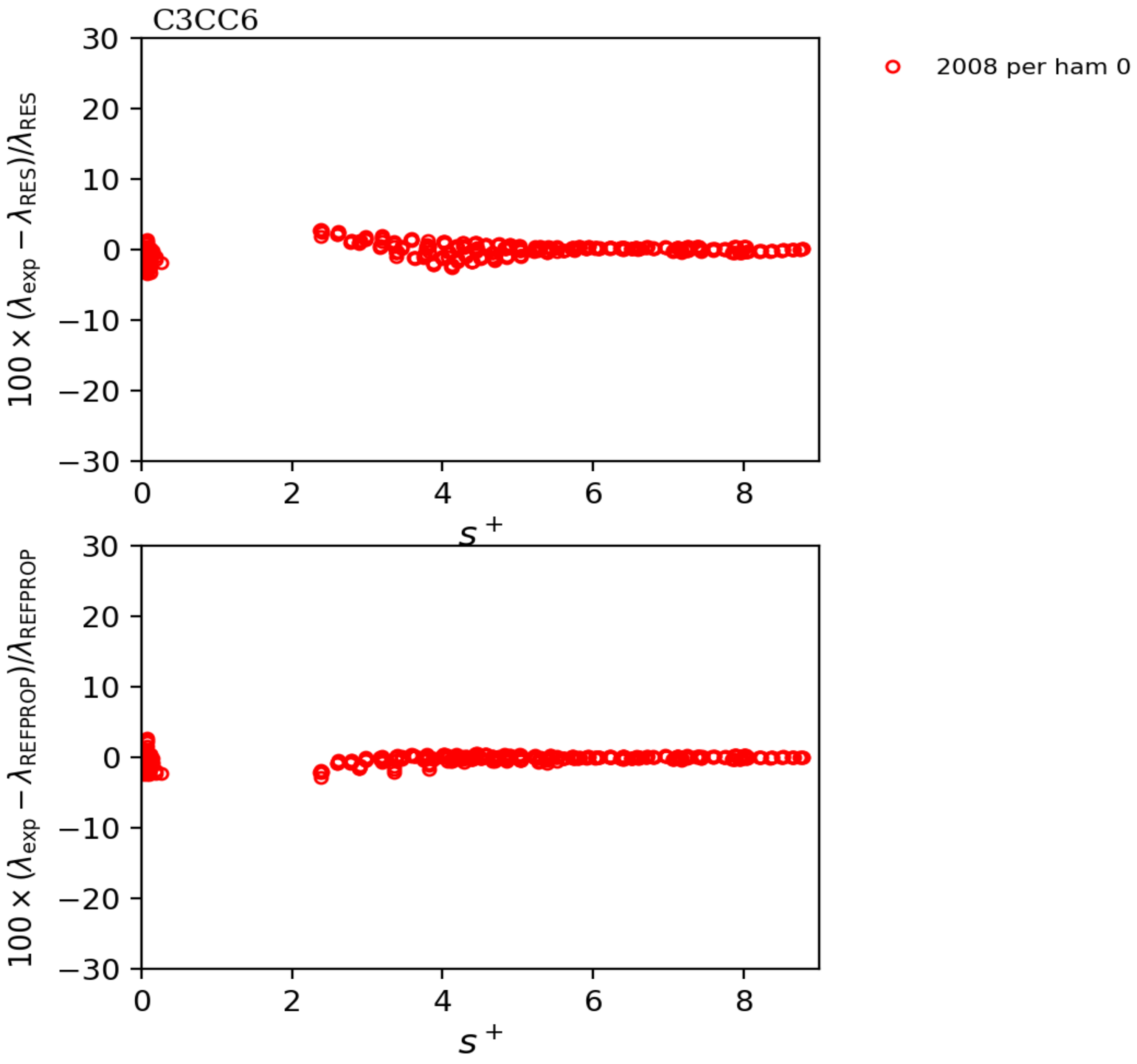

Figure DPR2. C3CC6

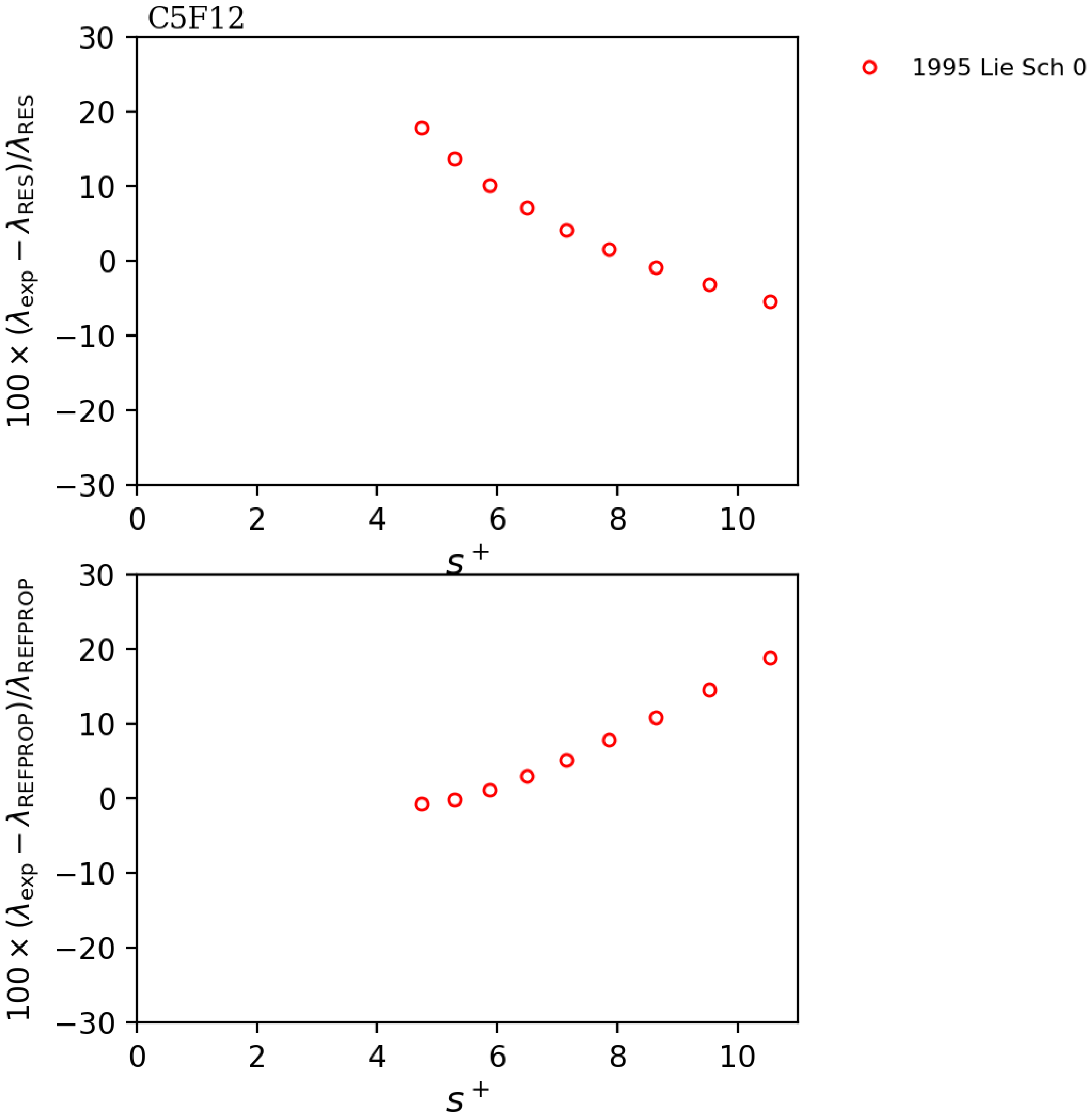

Figure DPR2. C5F12

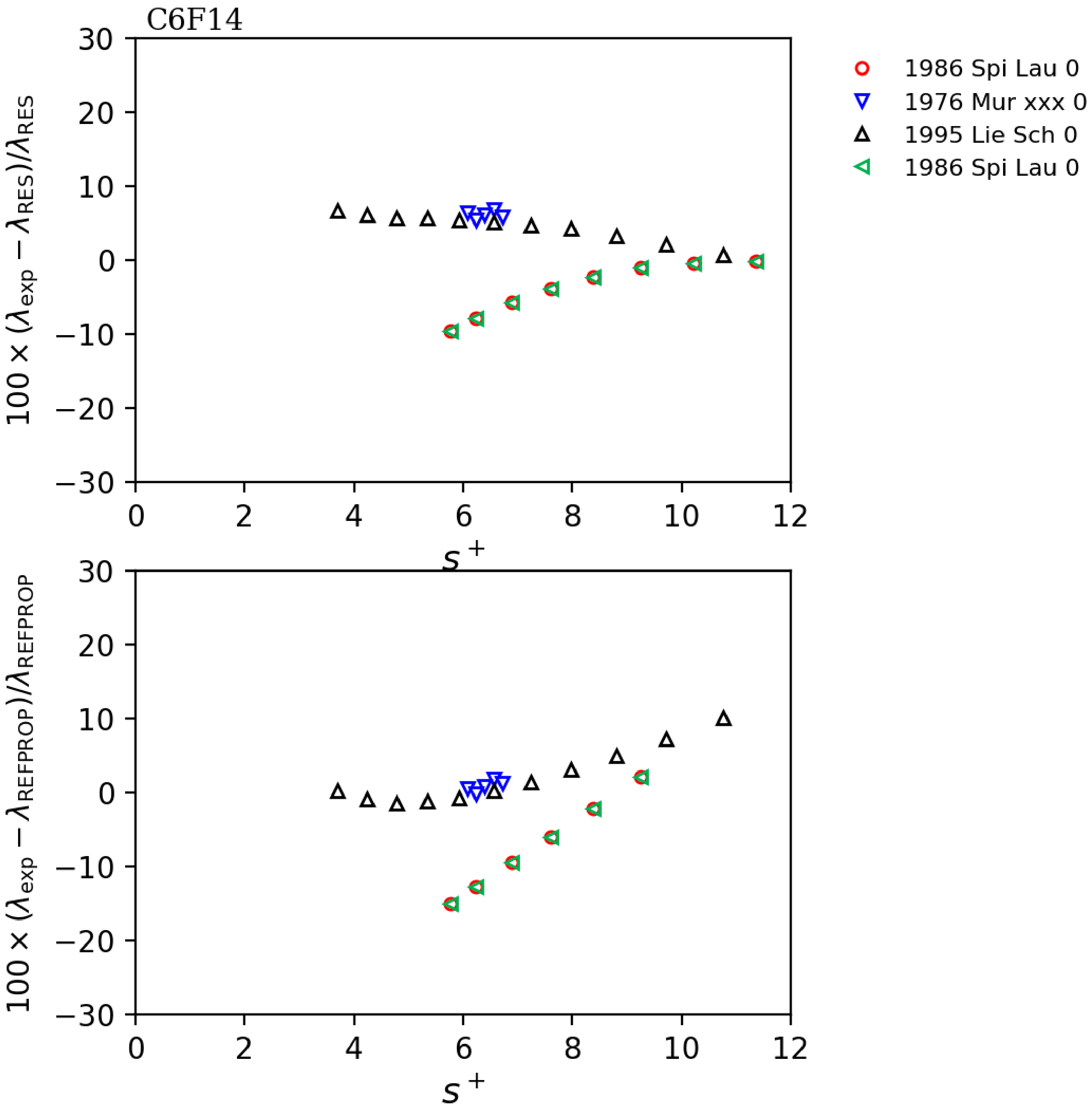

Figure DPR2. C6F14

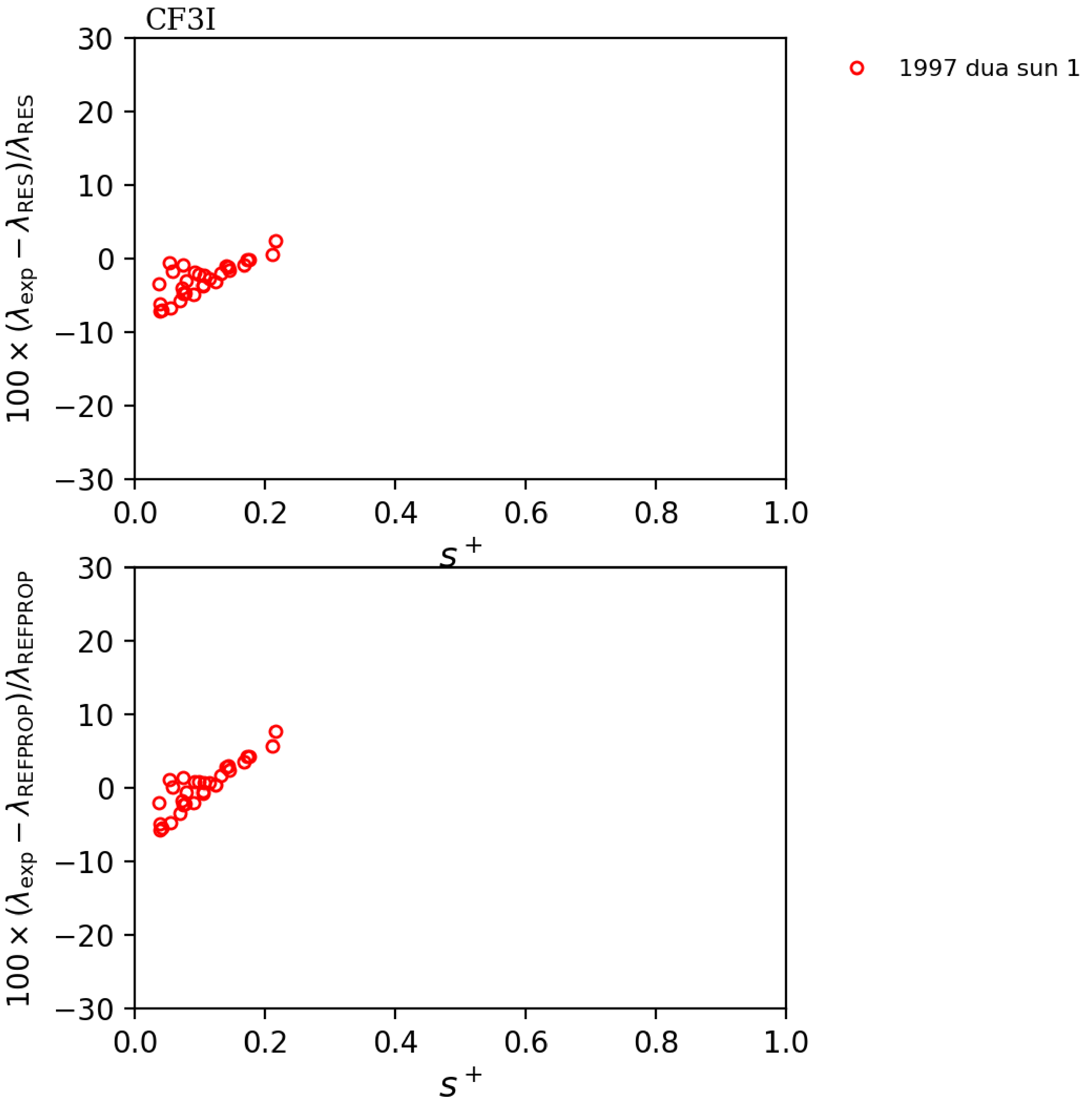

Figure DPR2. CF3I

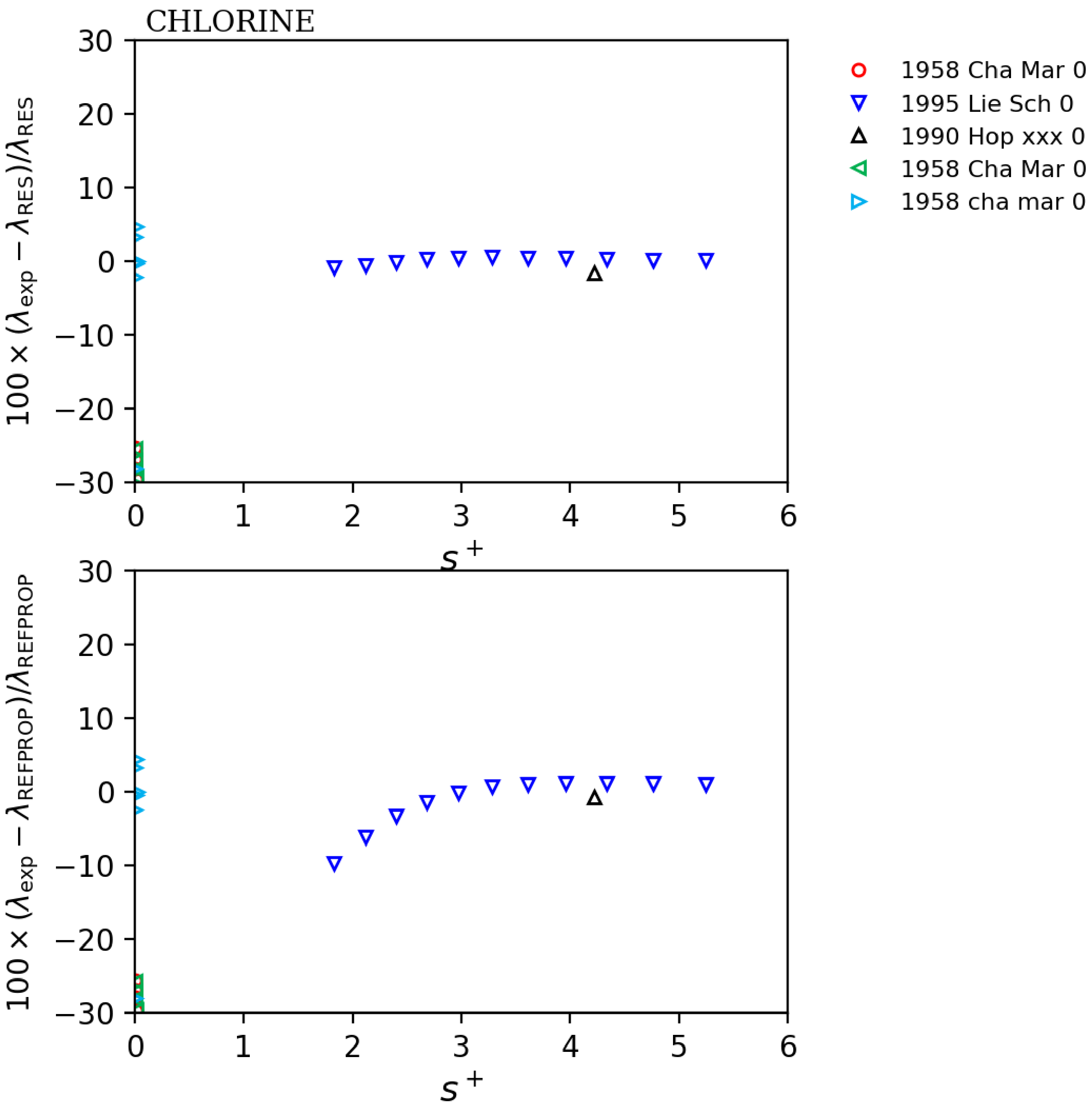

Figure DPR2. CHLORINE

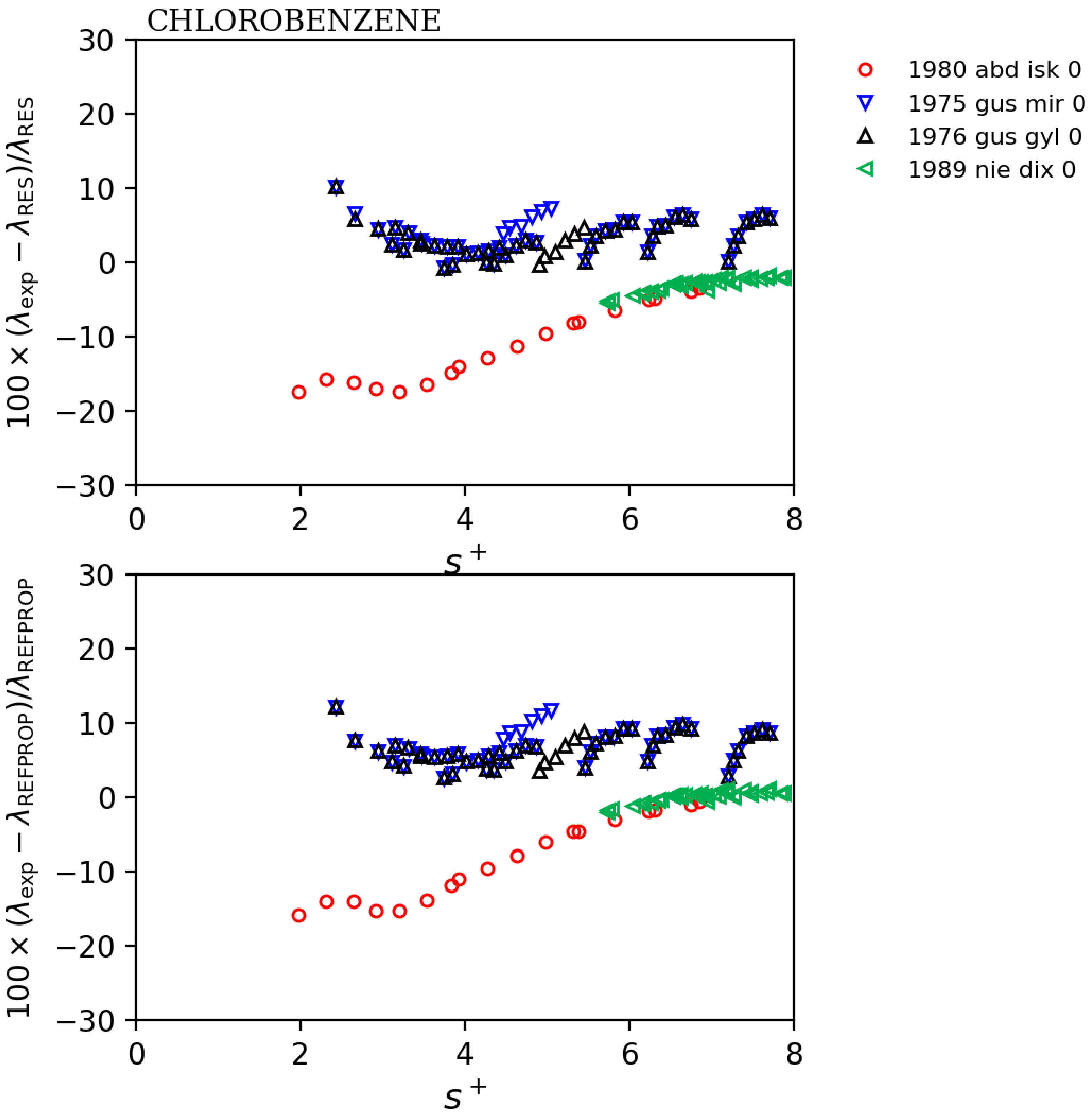

Figure DPR2. CHLOROBENZENE

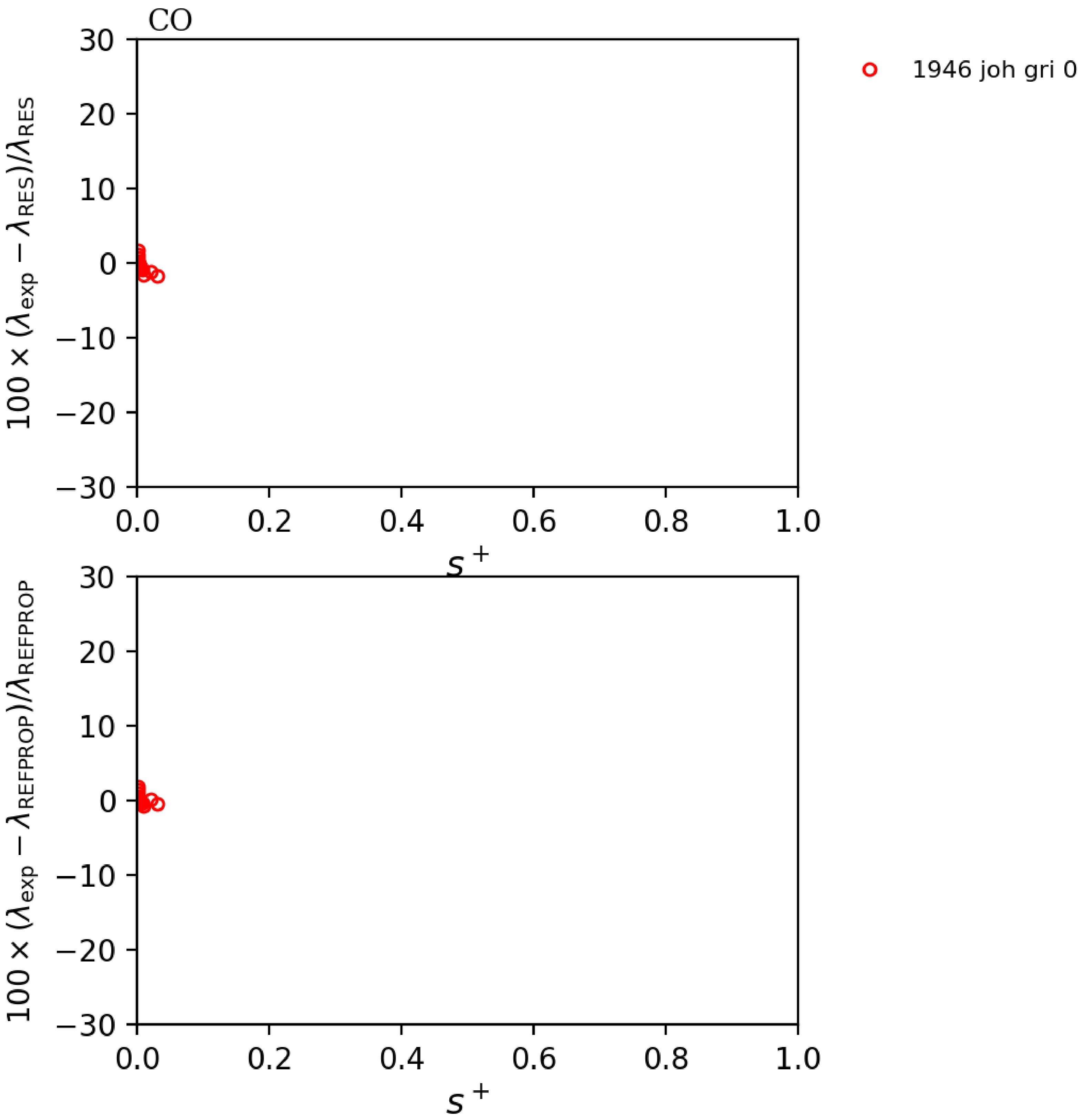

Figure DPR2. CO

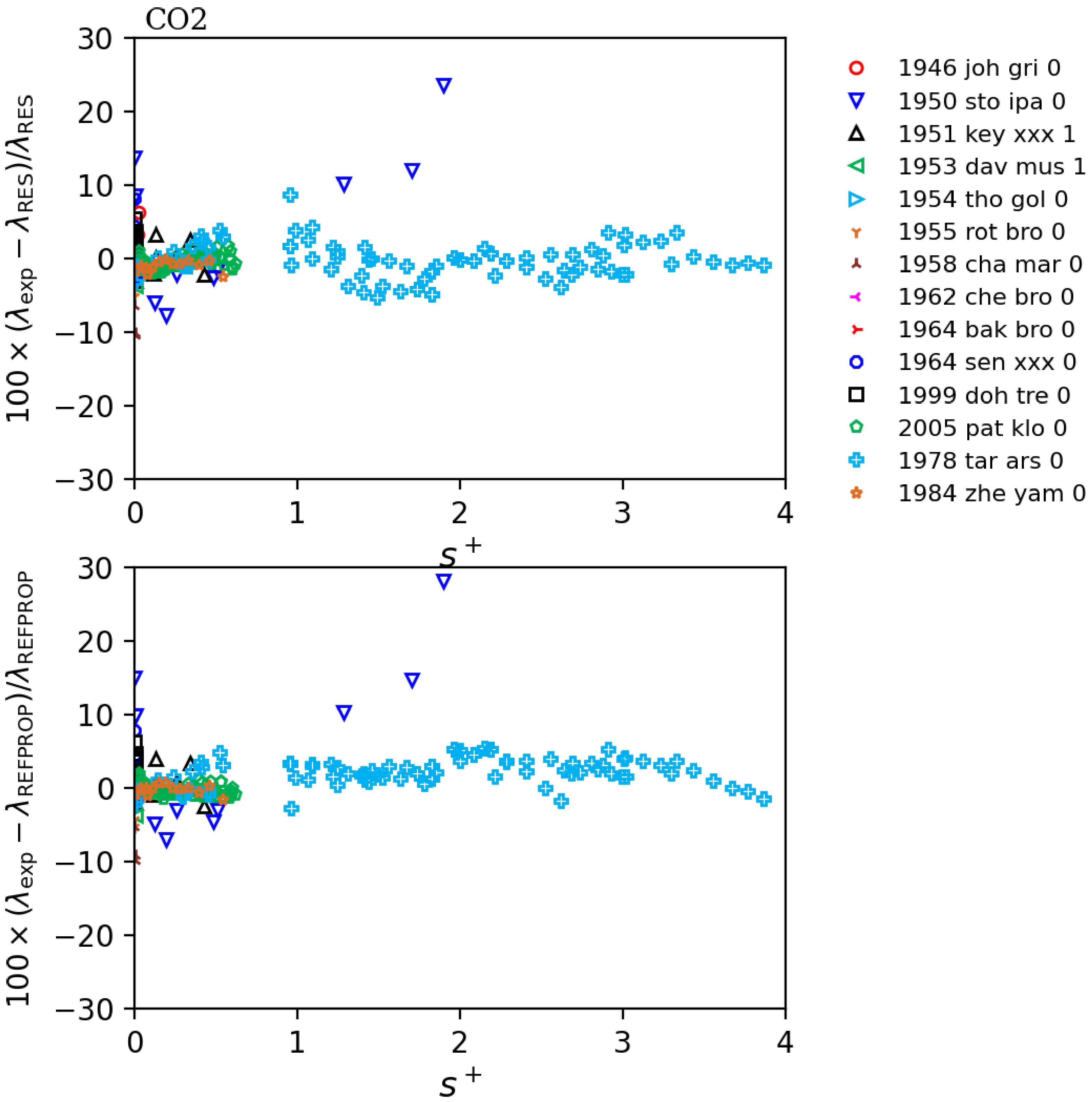

Figure DPR2. CO2

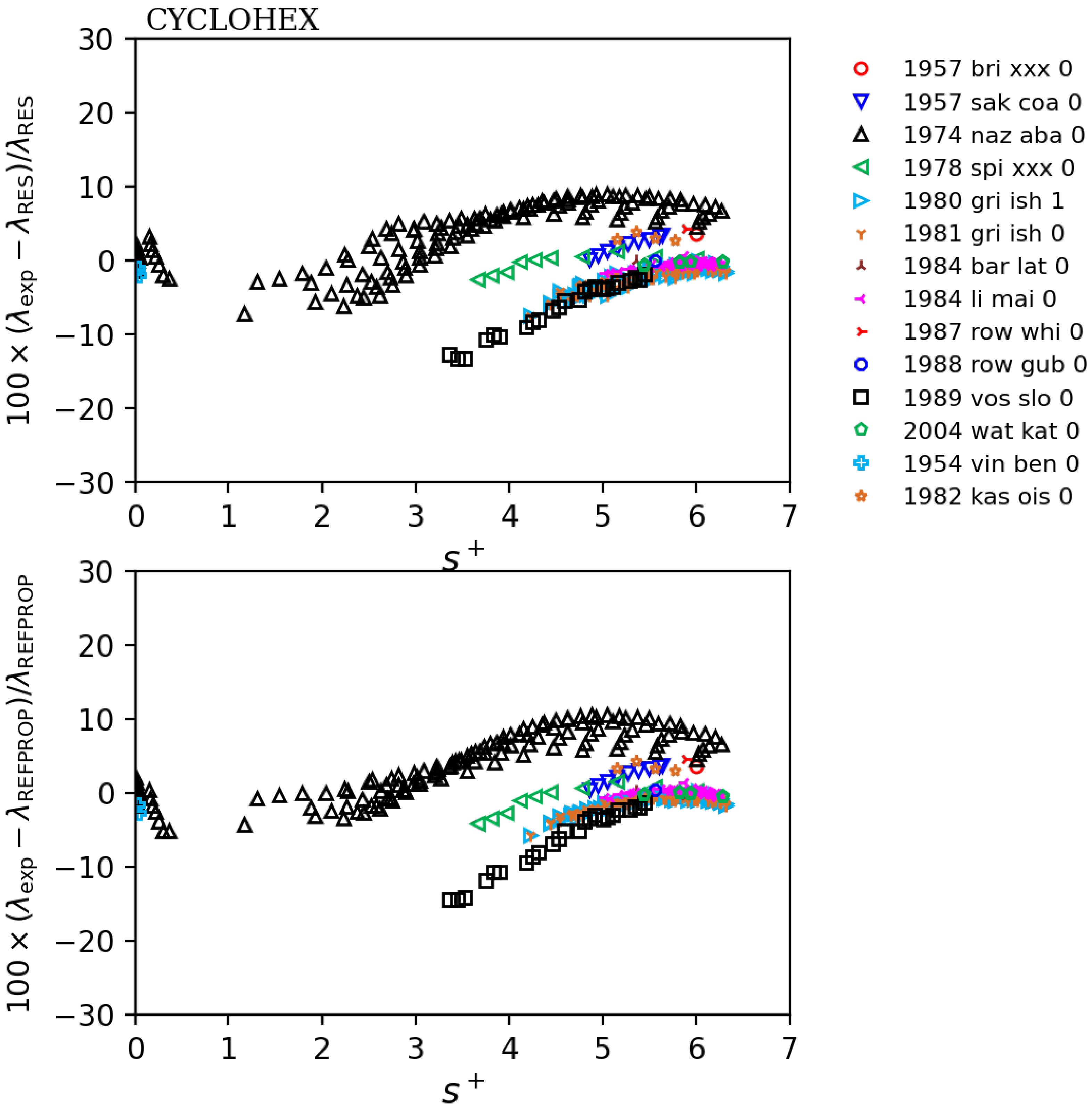

Figure DPR2. CYCLOHEX

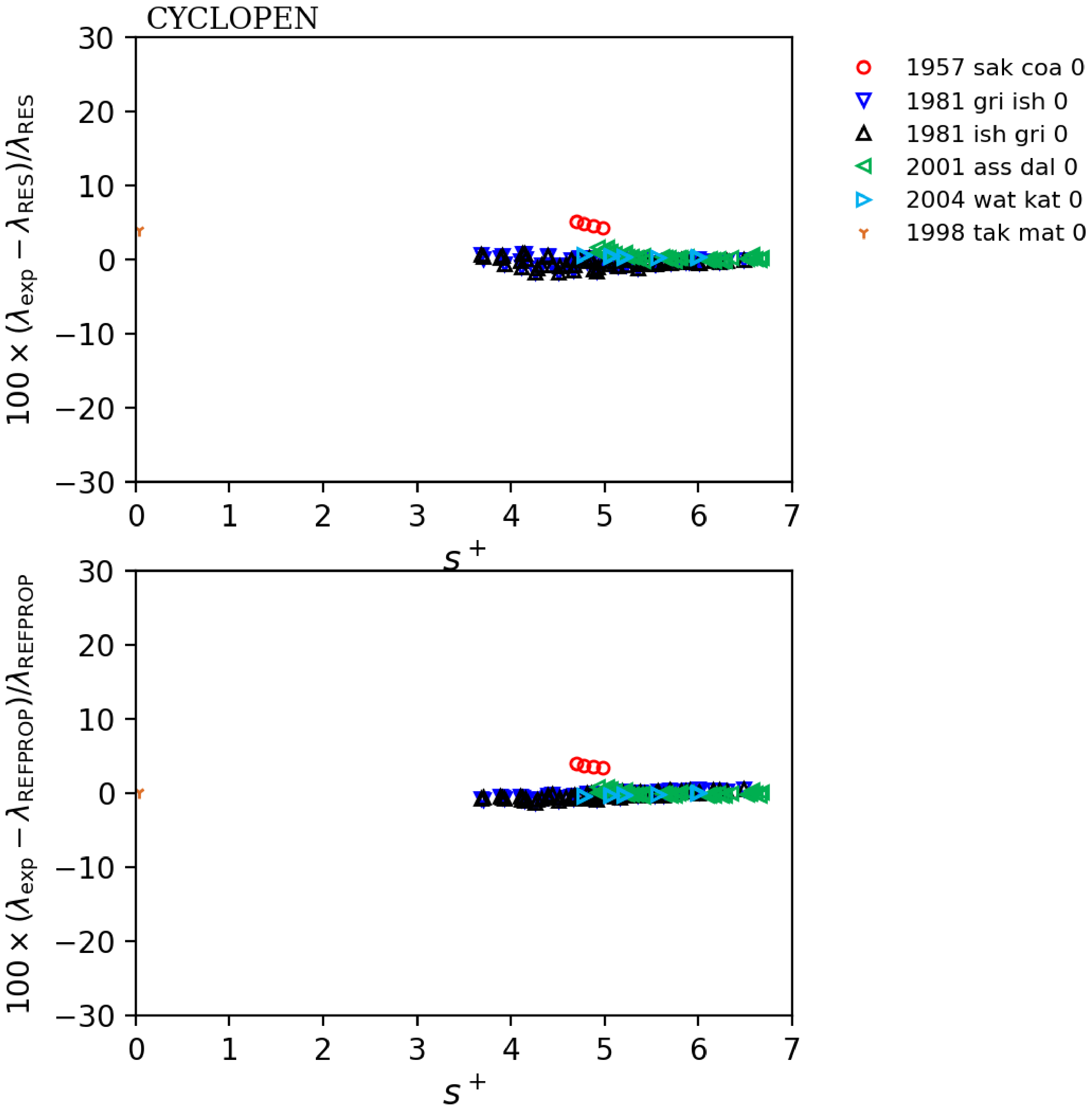

Figure DPR2. CYCLOPEN

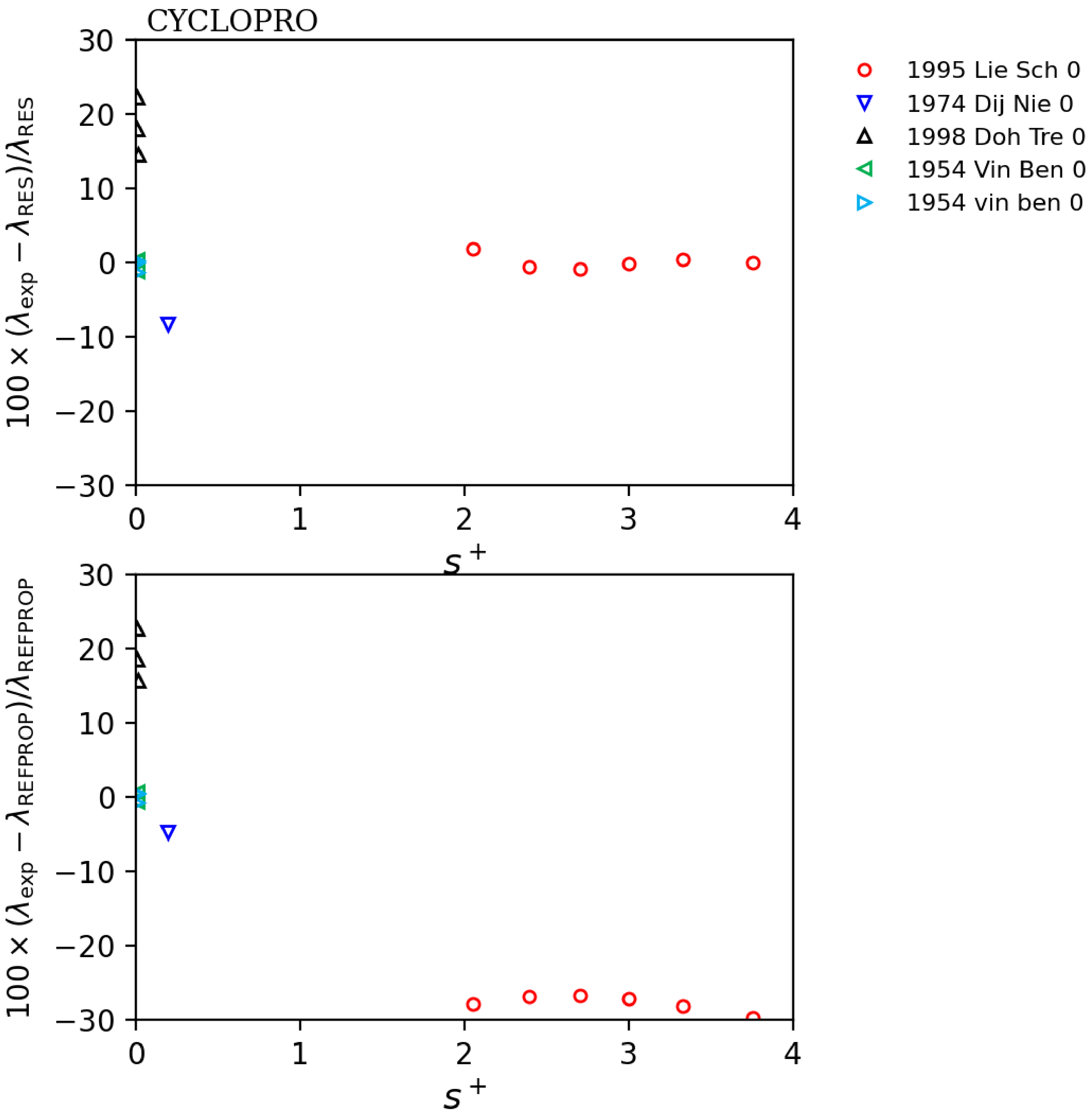

Figure DPR2. CYCLOPRO

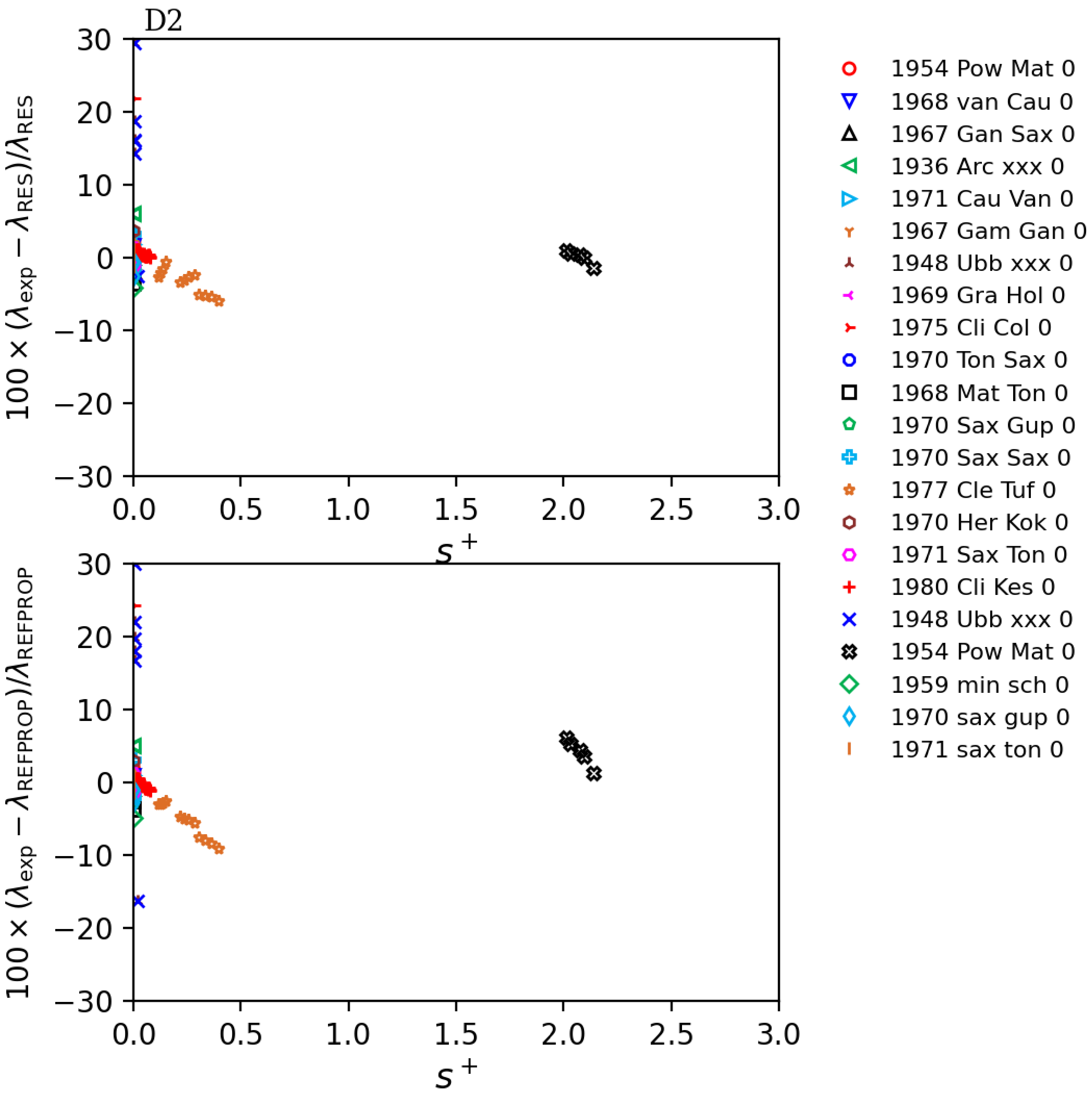

Figure DPR2. D2

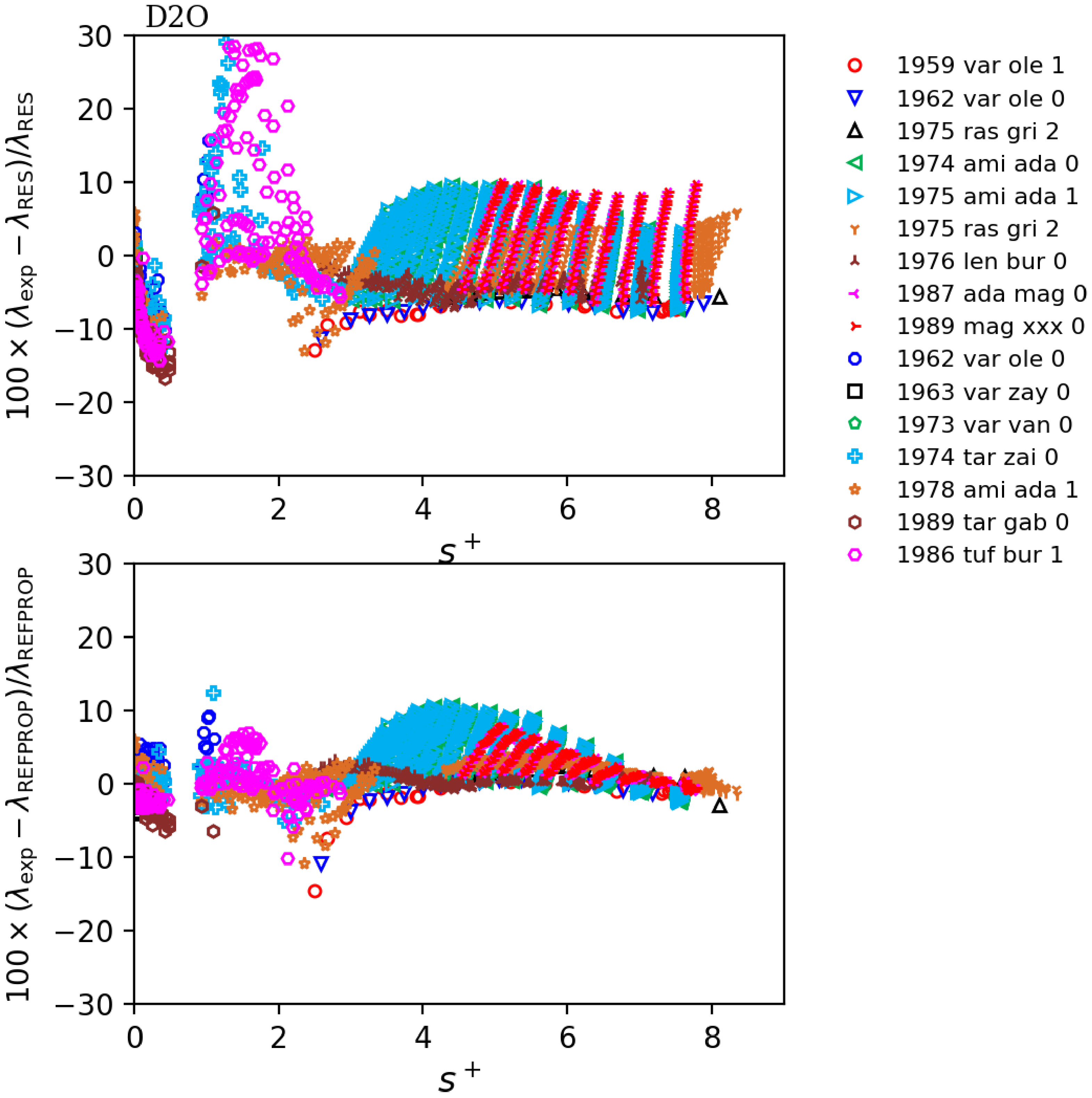

Figure DPR2. D2O

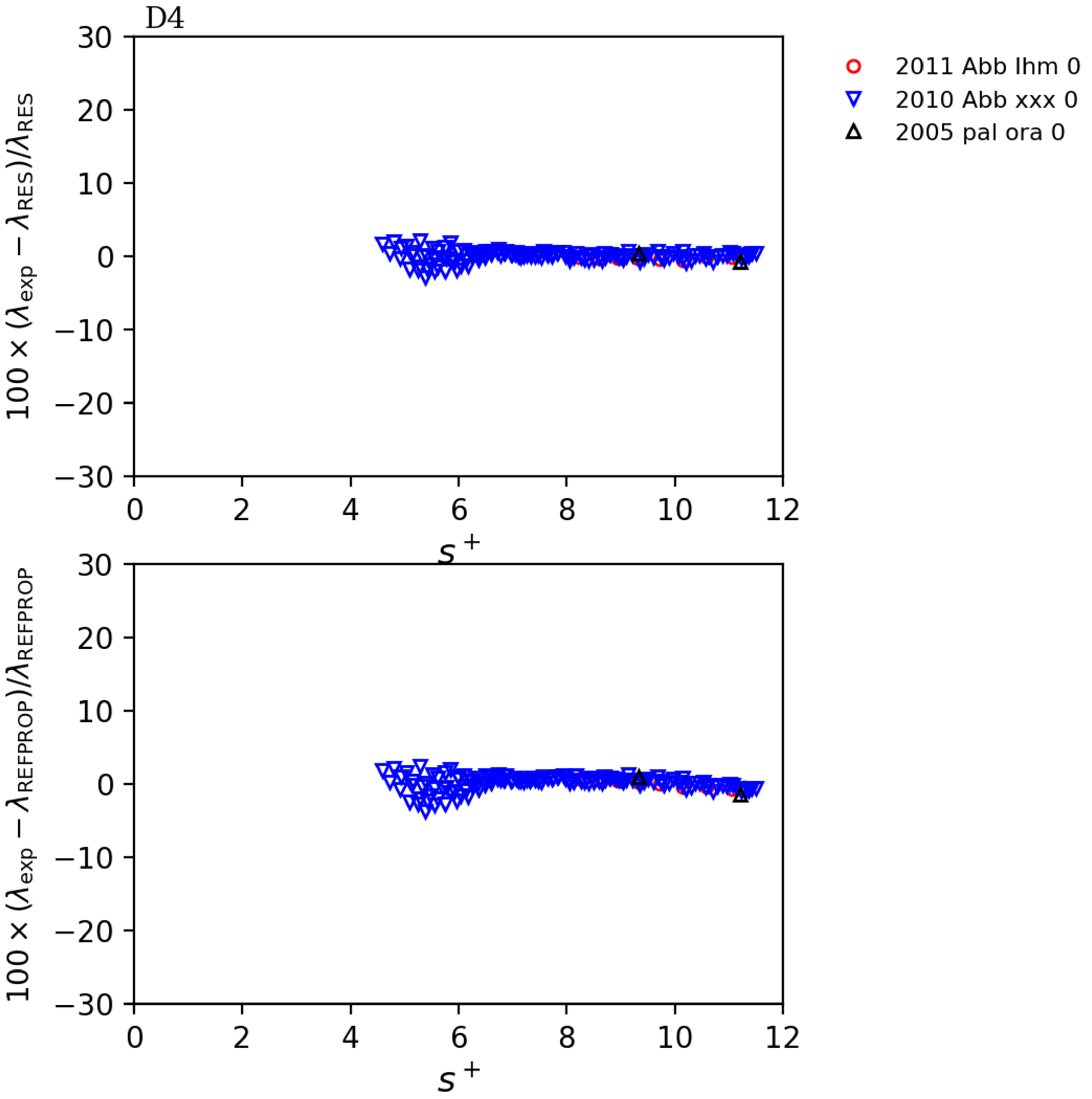

Figure DPR2. D4

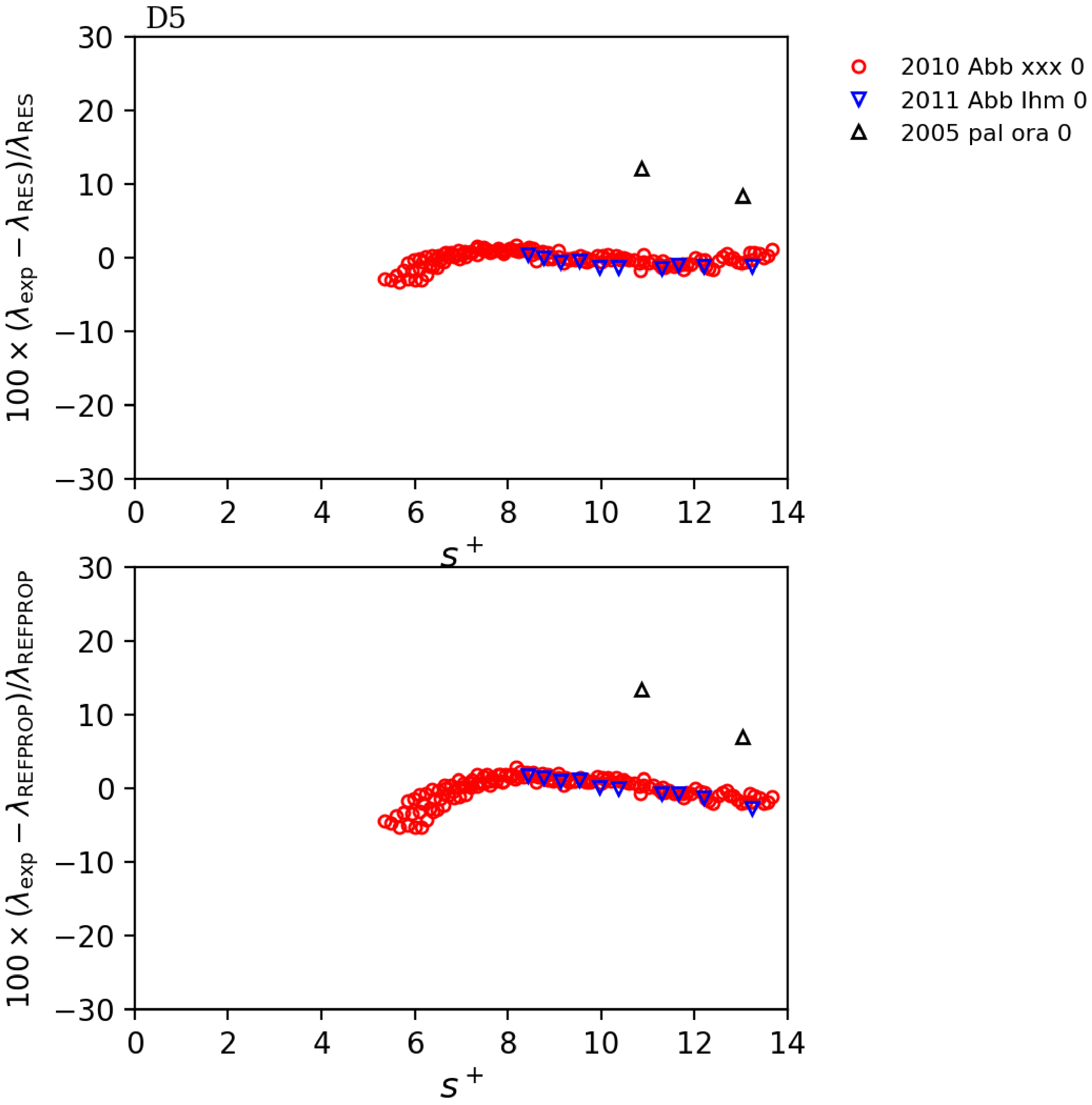

Figure DPR2. D5

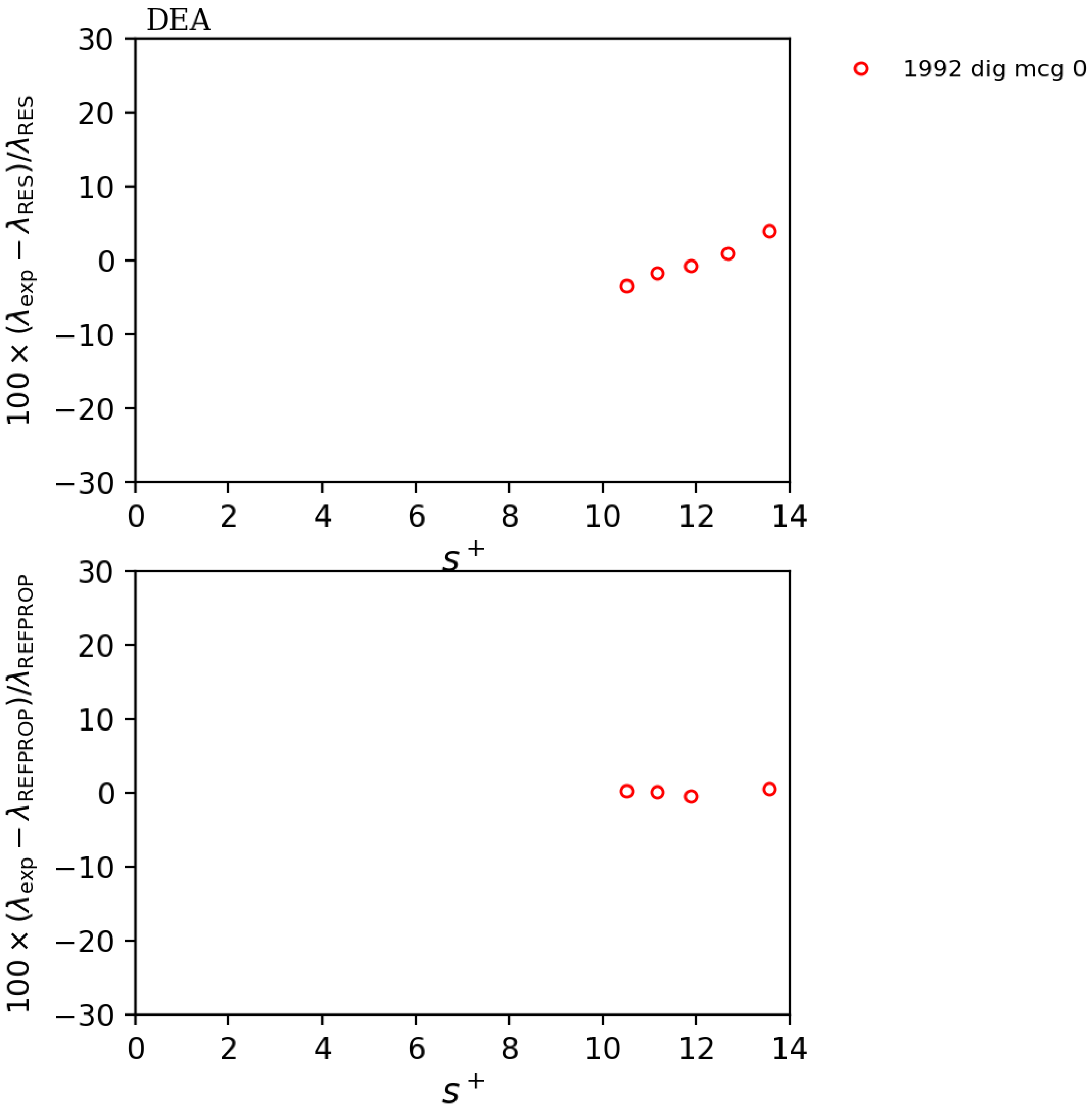

Figure DPR2. DEA

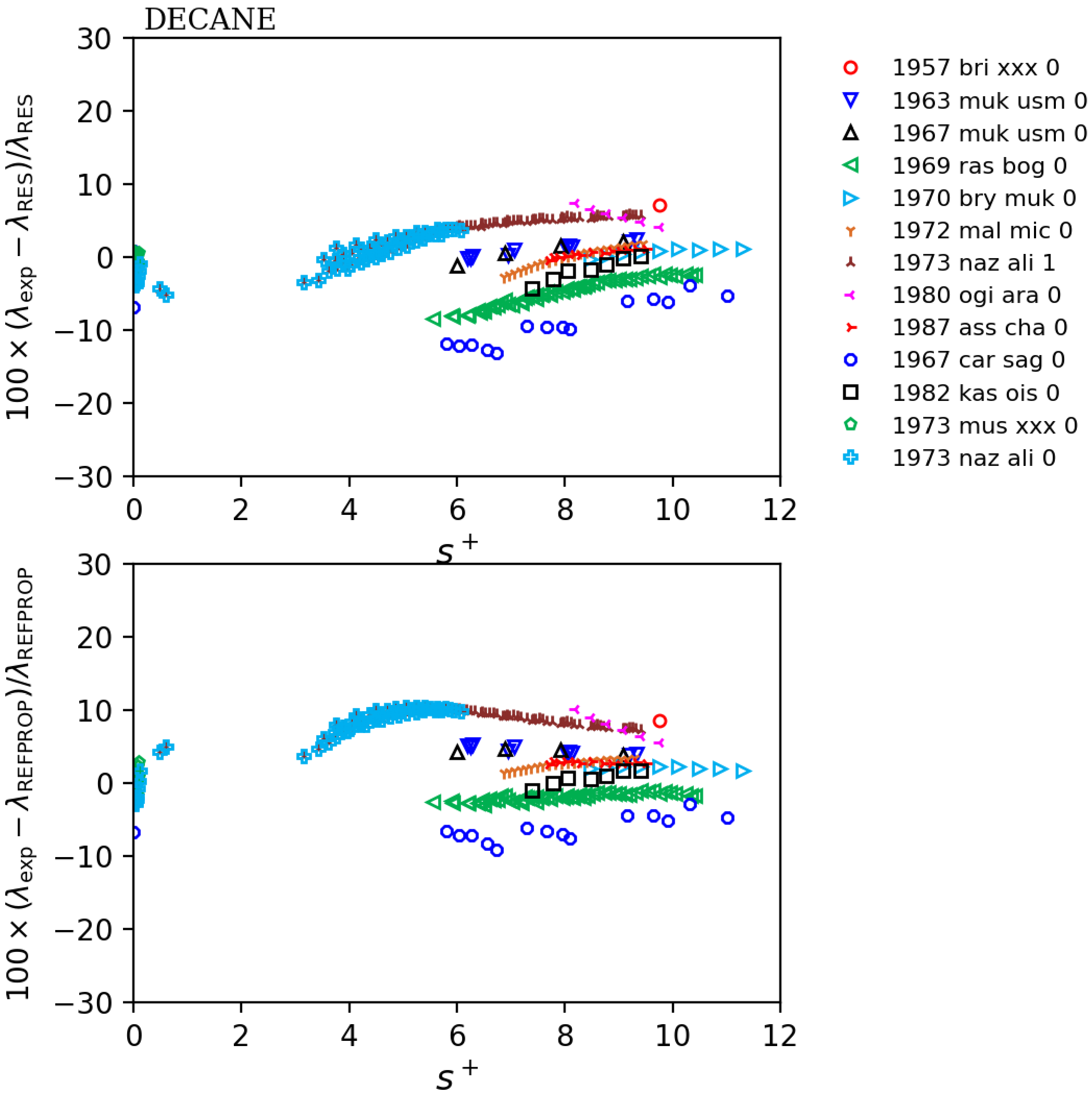

Figure DPR2. DECANE

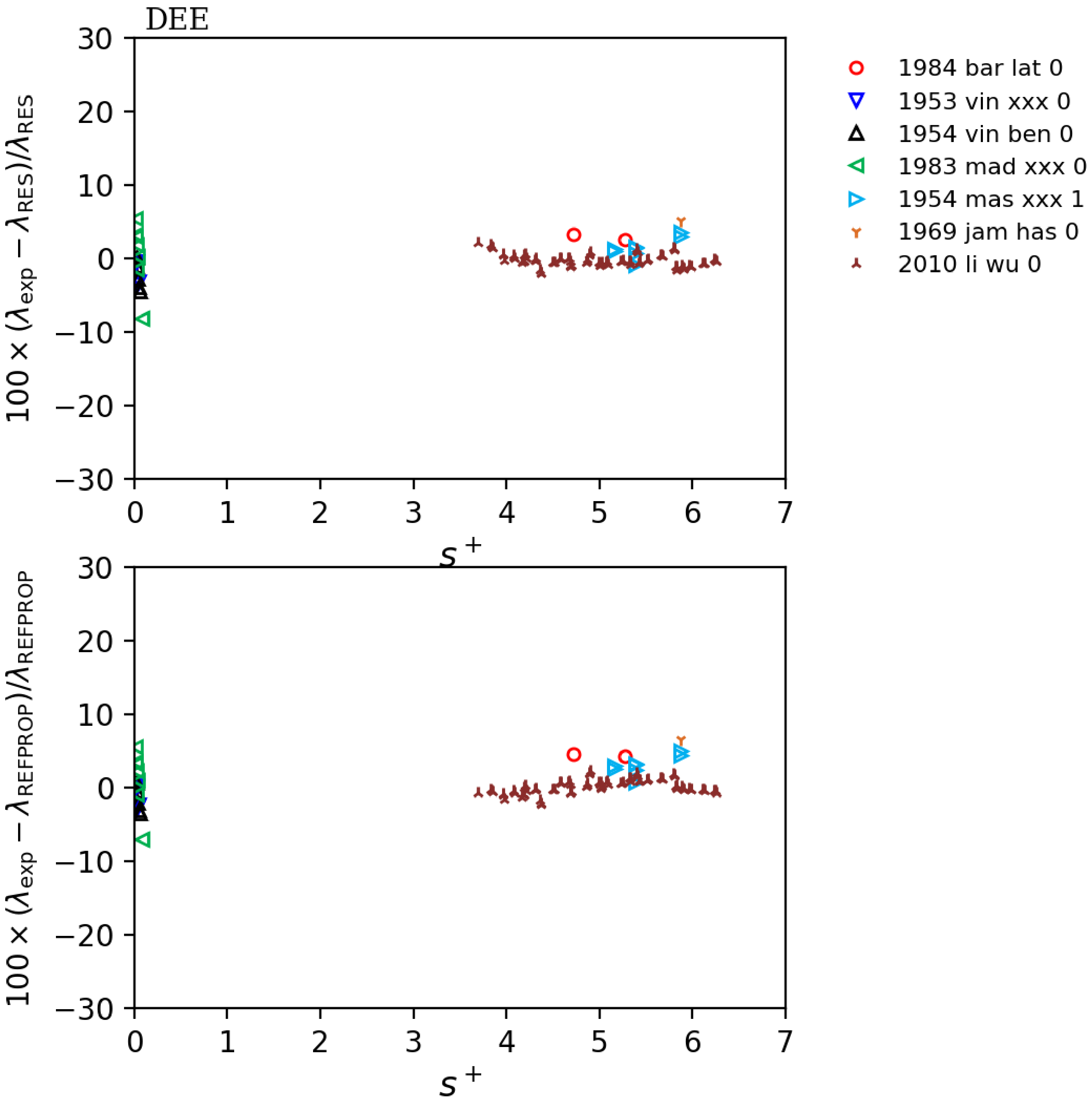

Figure DPR2. DEE

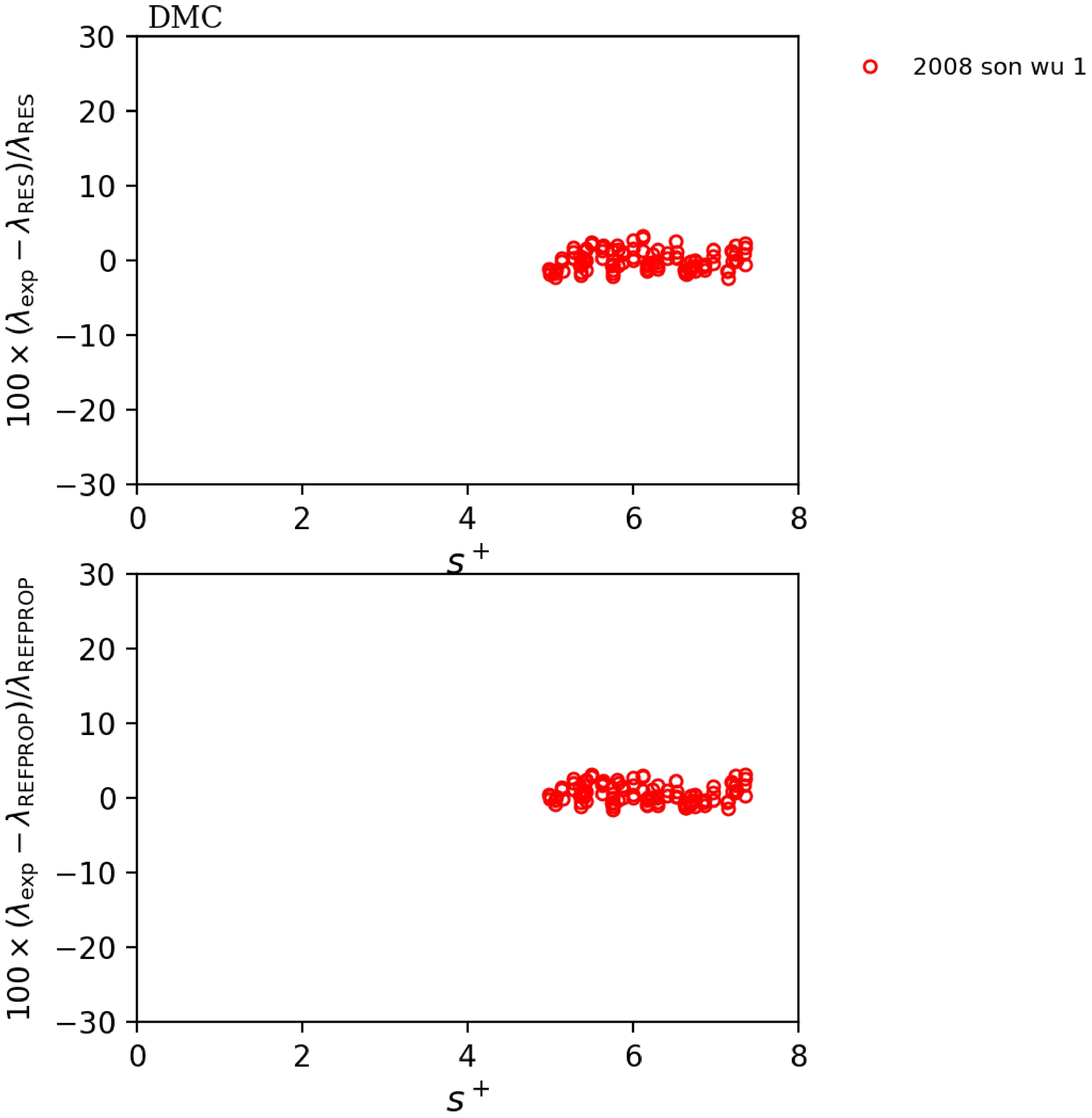

Figure DPR2. DMC

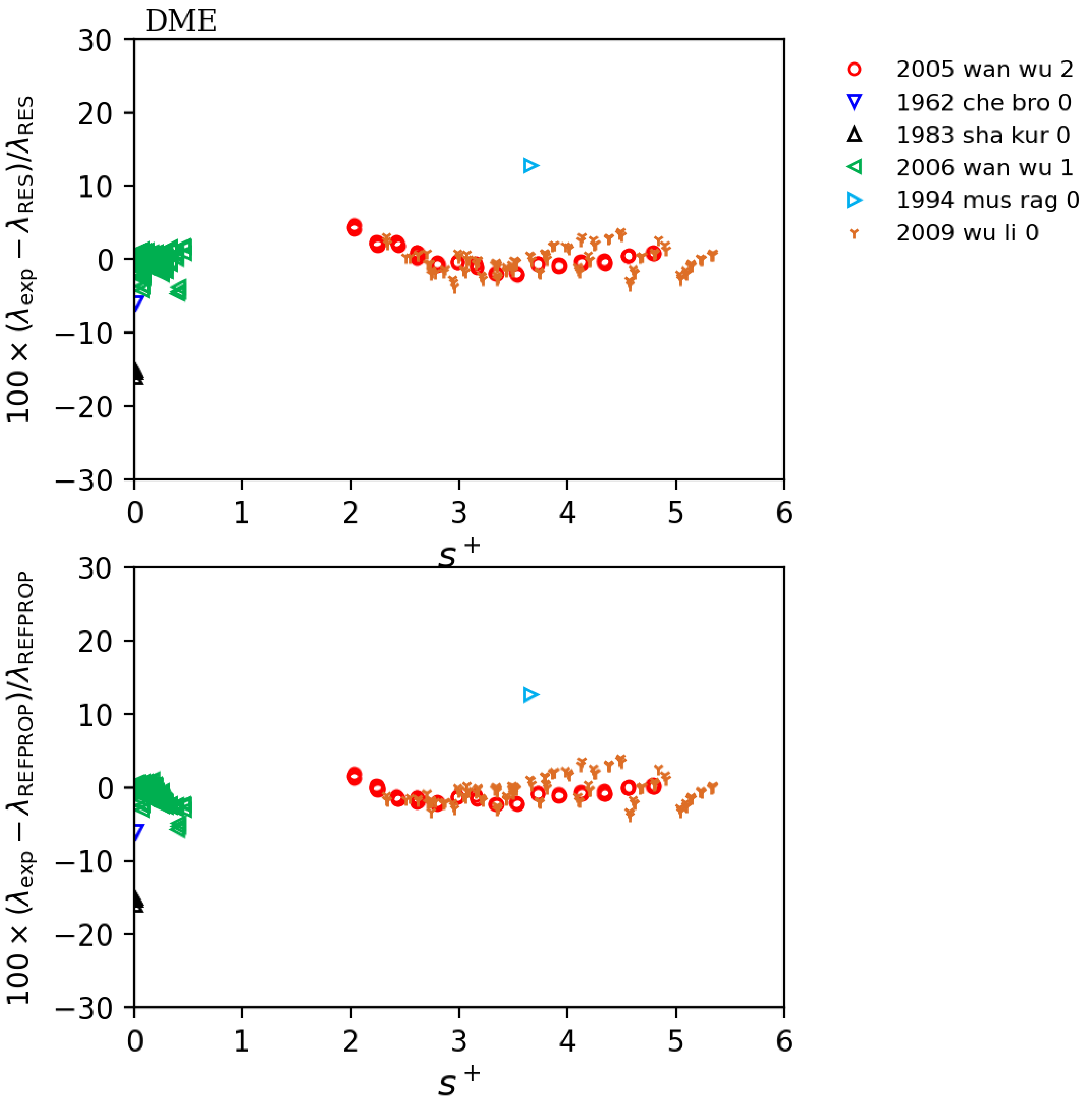

Figure DPR2. DME

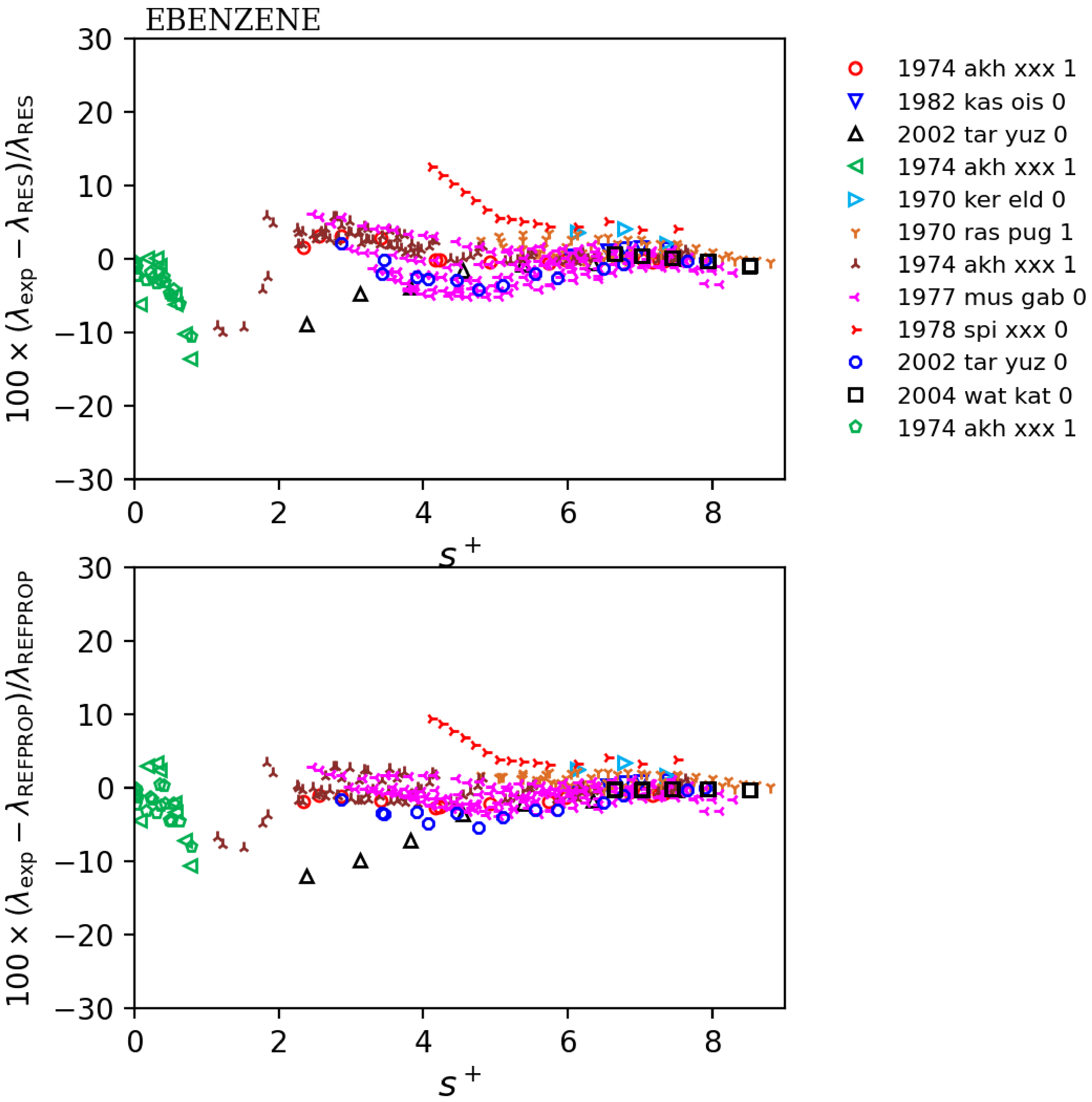

Figure DPR2. EBENZENE

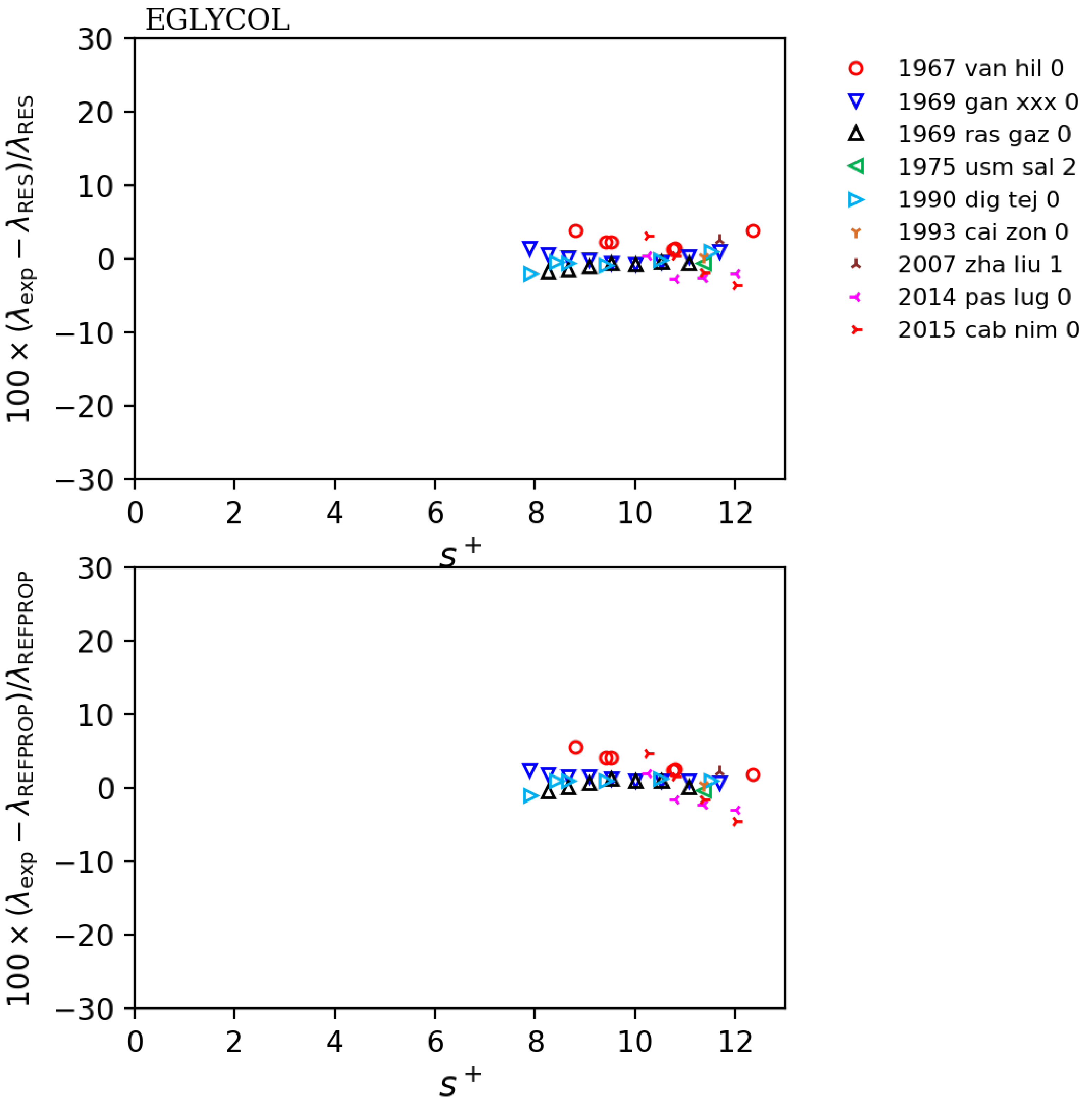

Figure DPR2. EGLYCOL

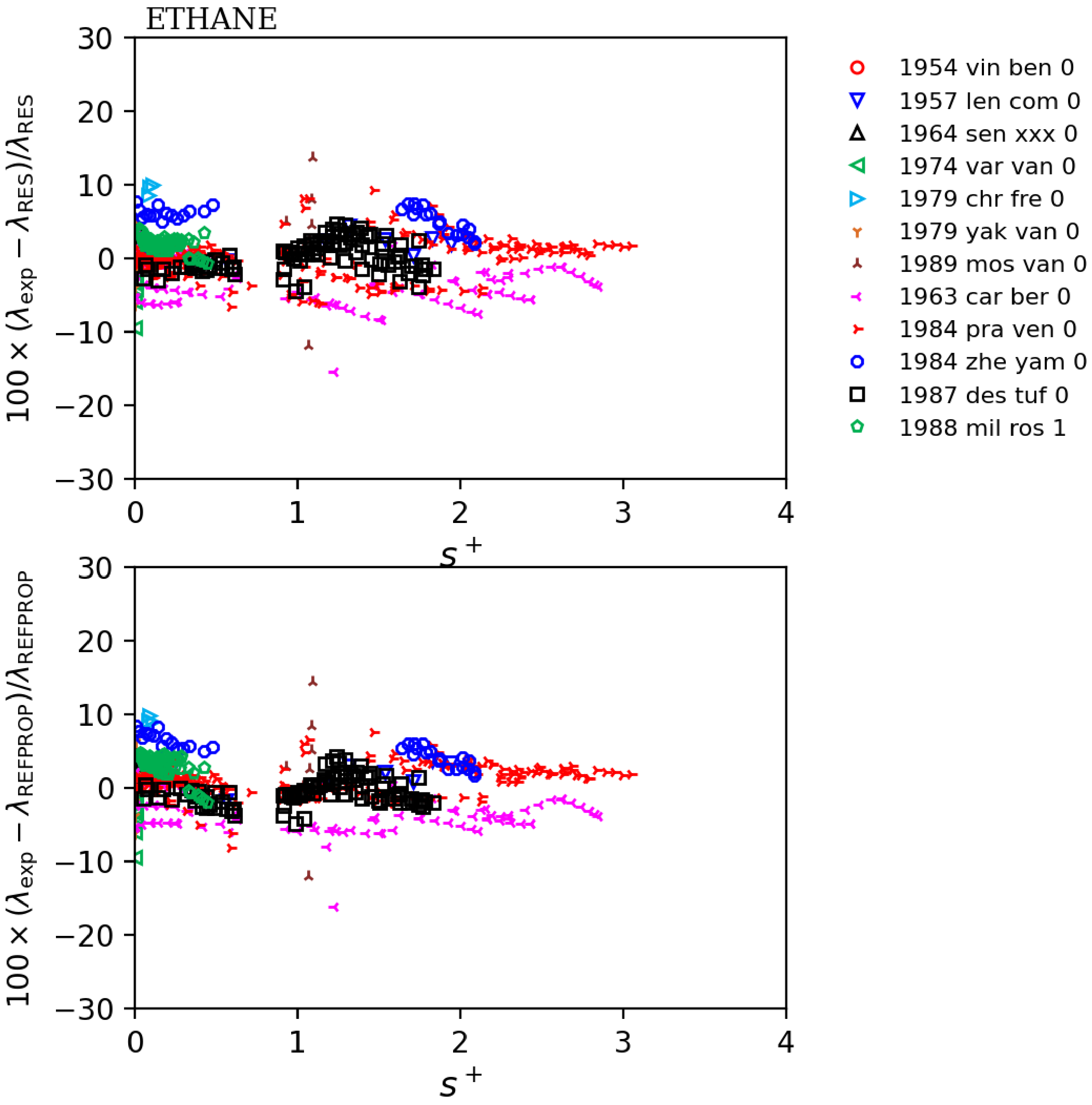

Figure DPR2. ETHANE

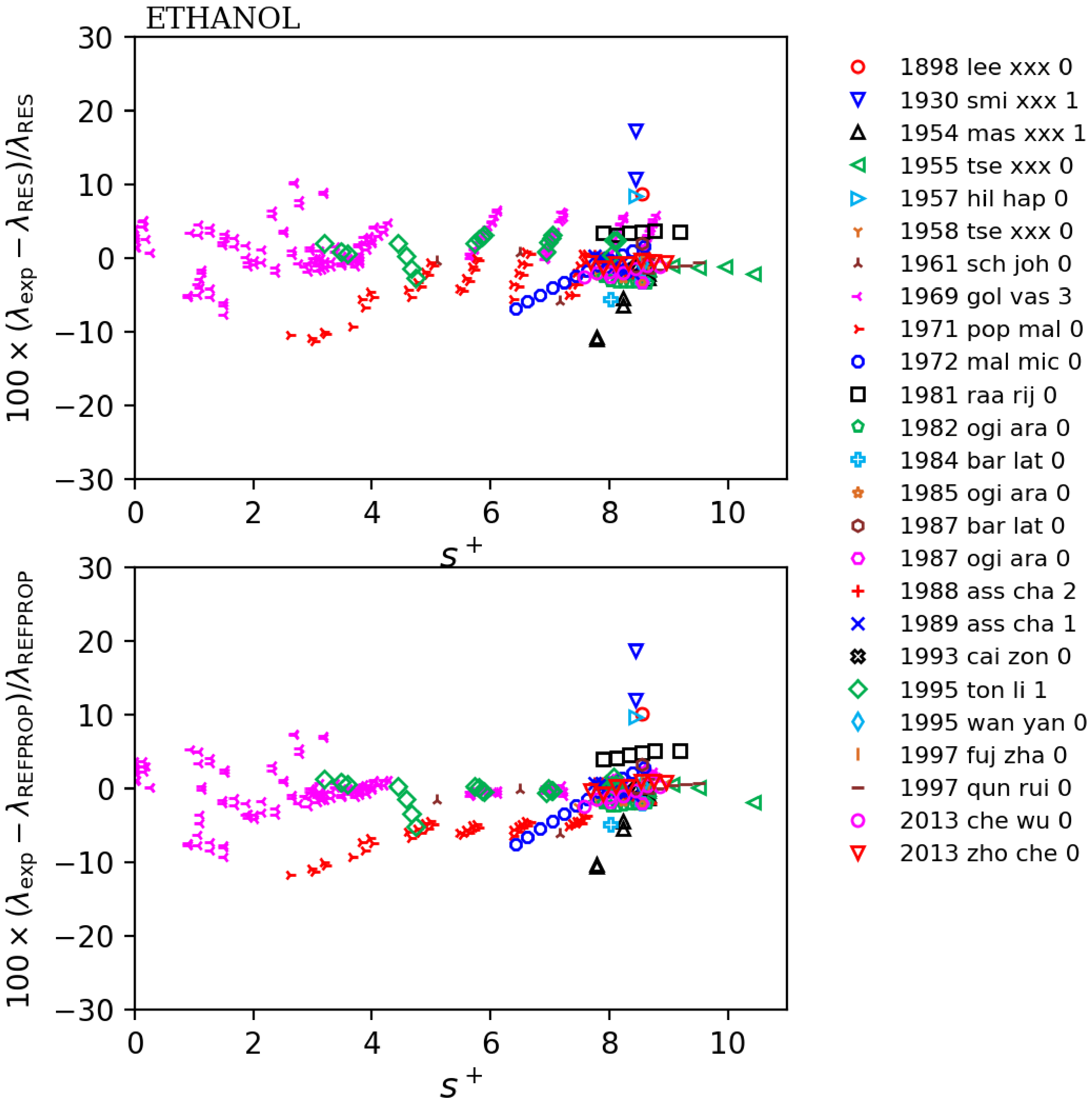

Figure DPR2. ETHANOL

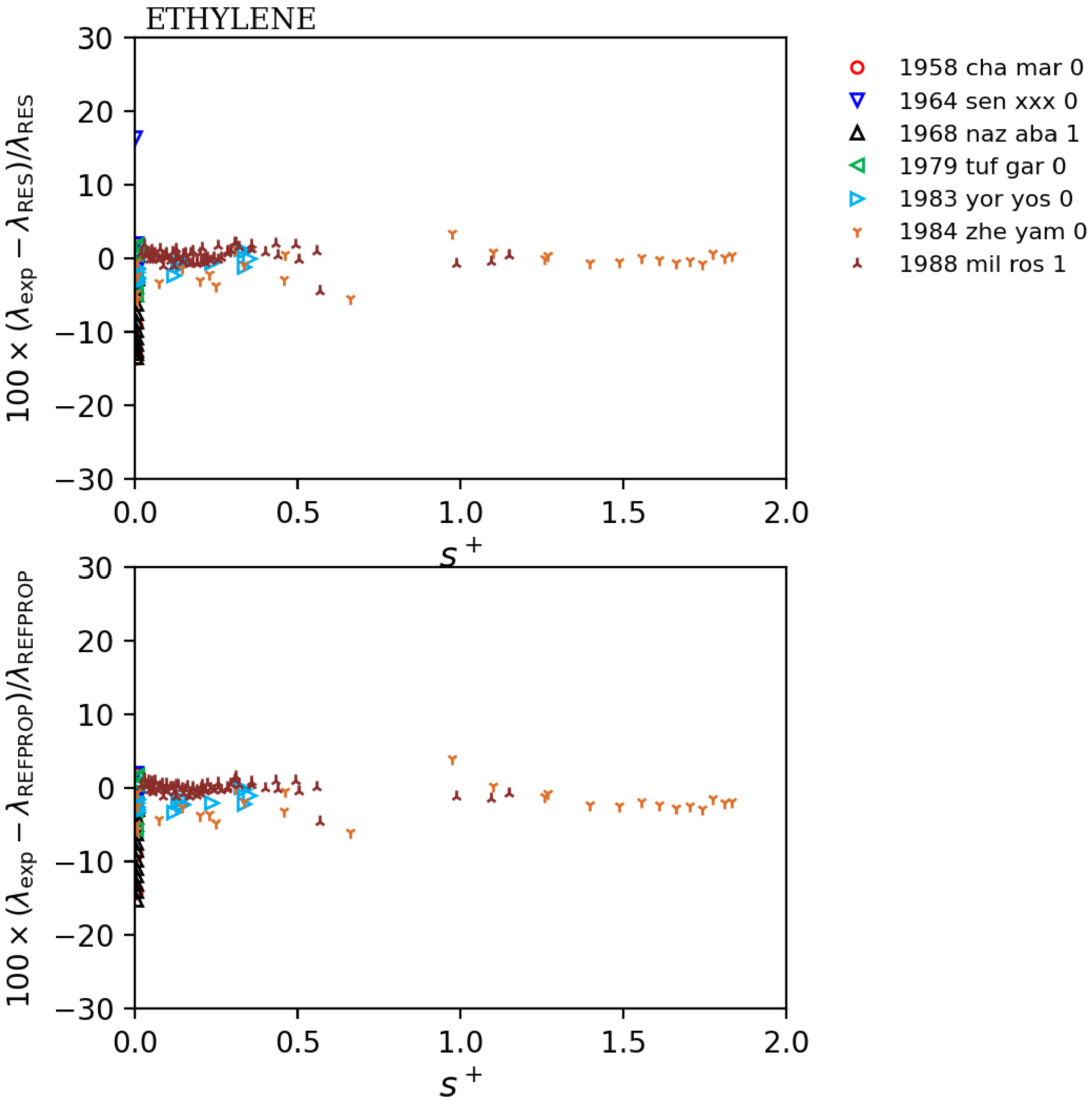

Figure DPR2. ETHYLENE

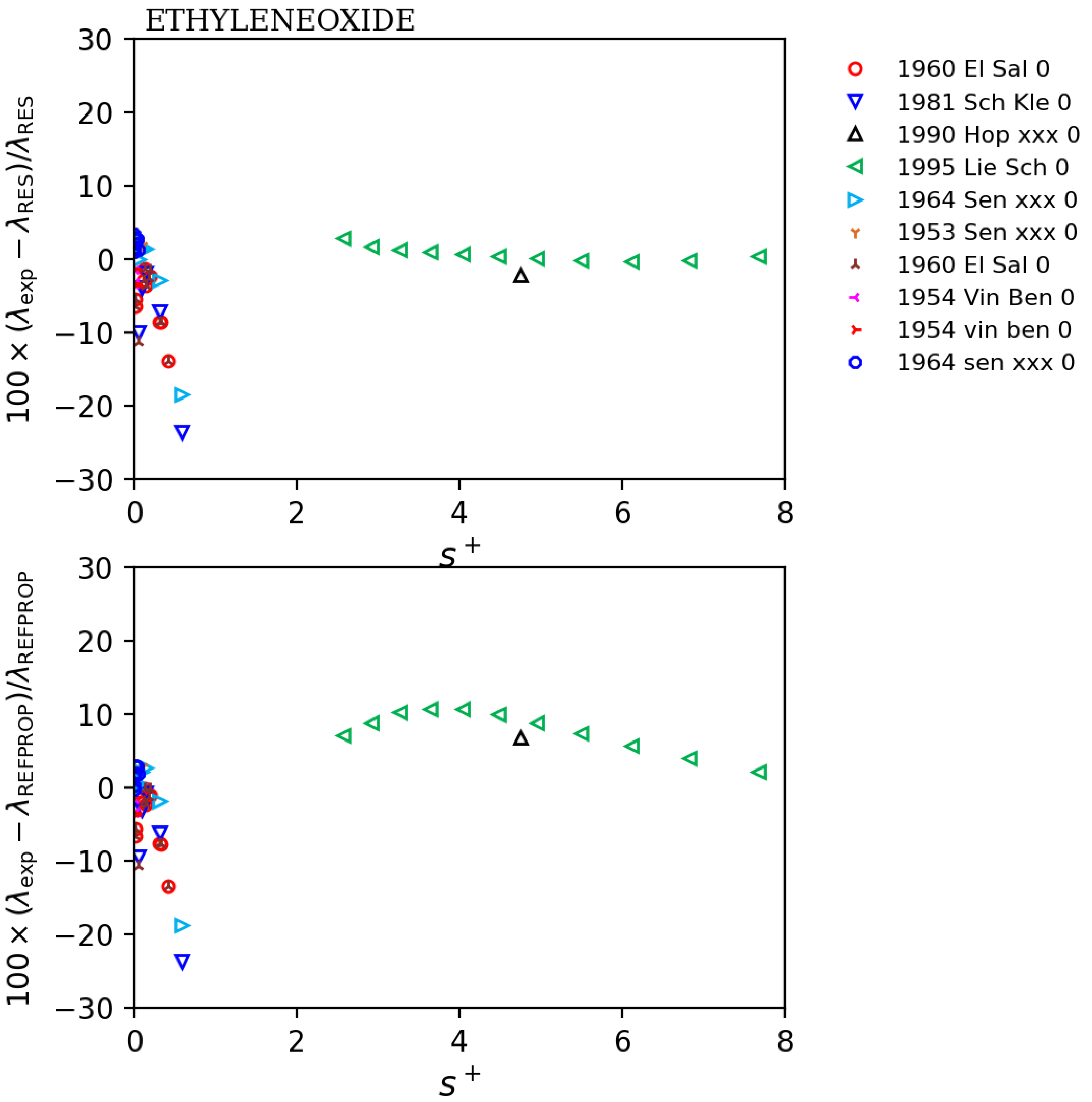

Figure DPR2. ETHYLENEOXIDE

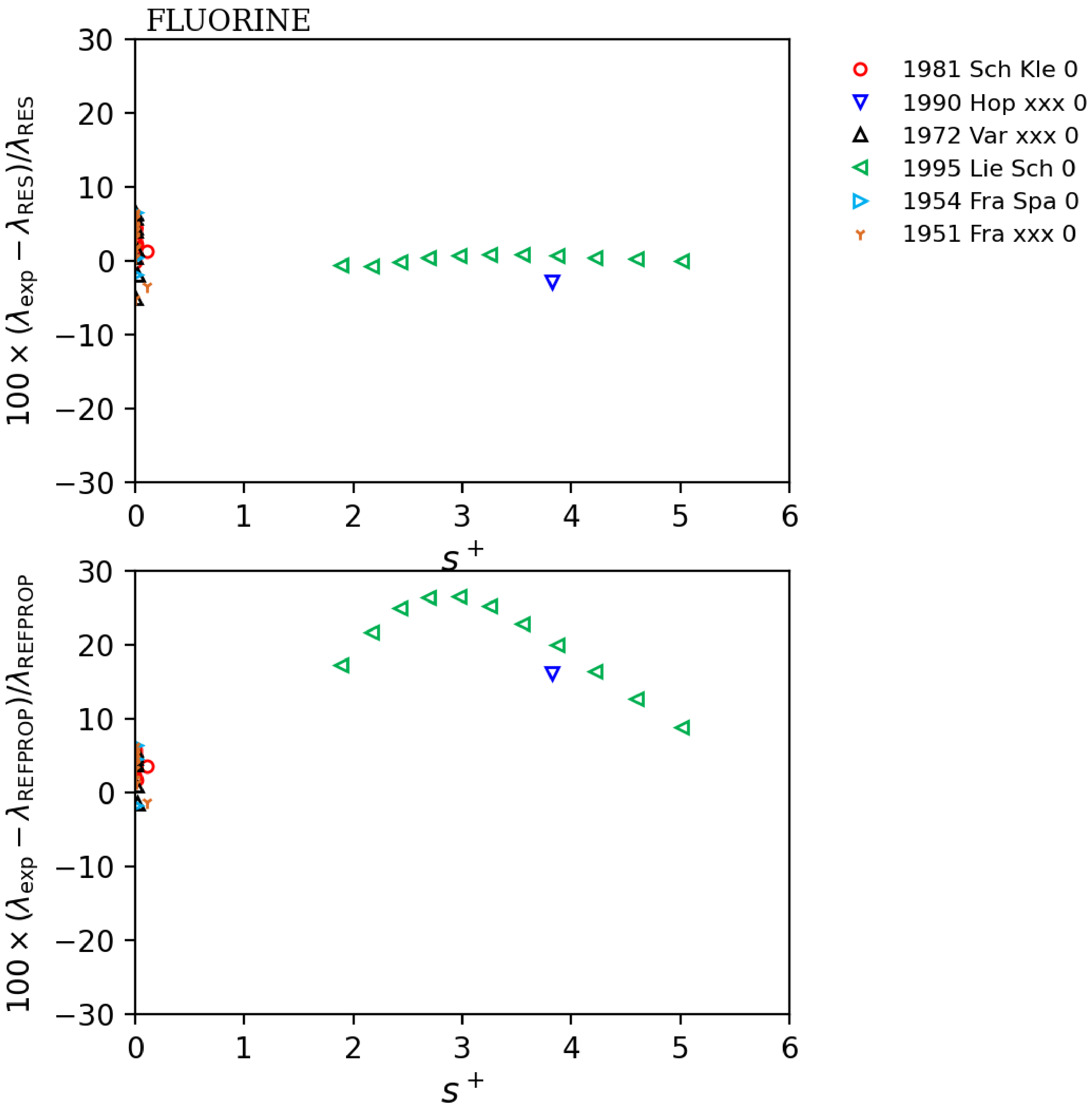

Figure DPR2. FLUORINE

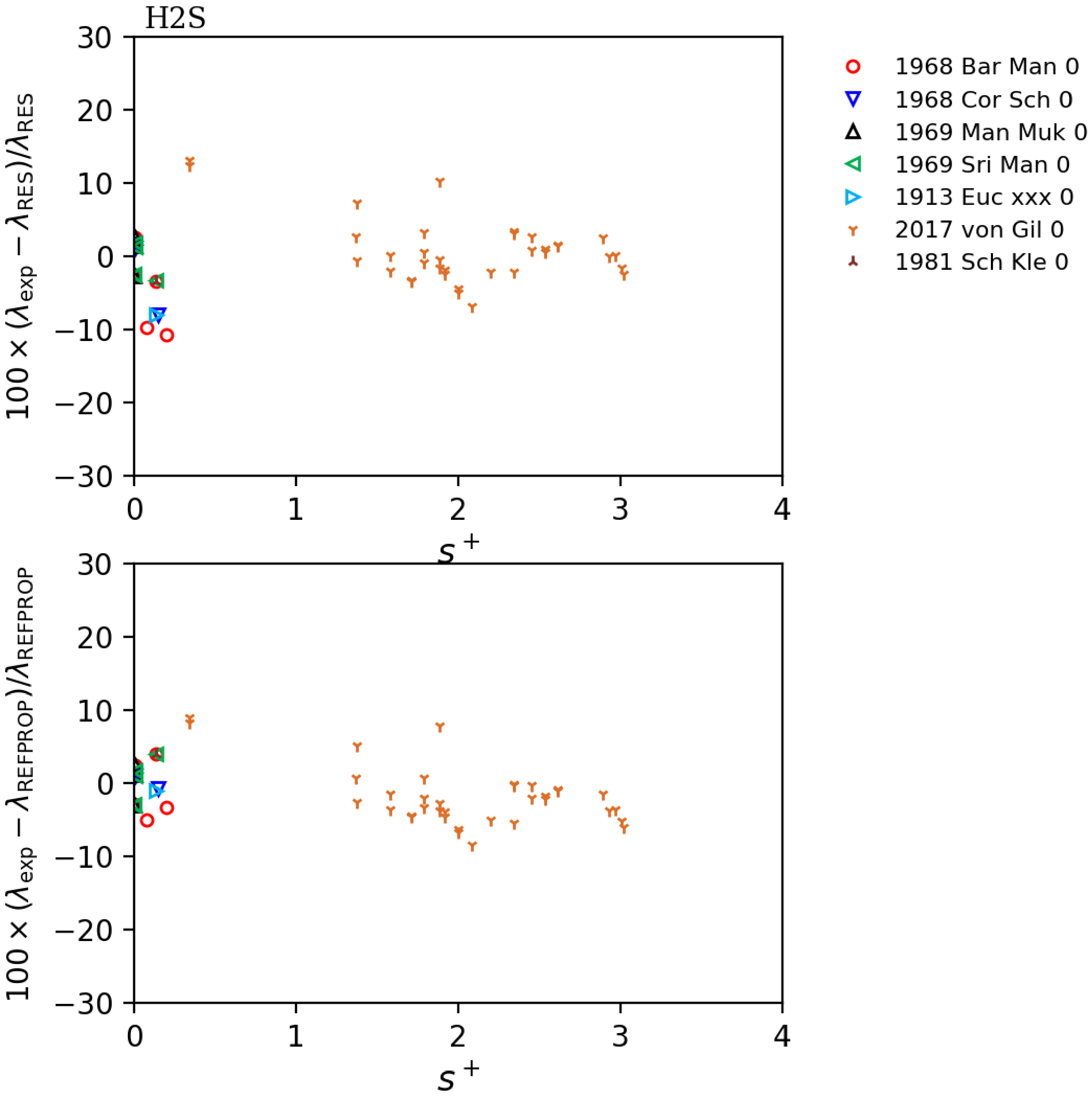

Figure DPR2. H2S

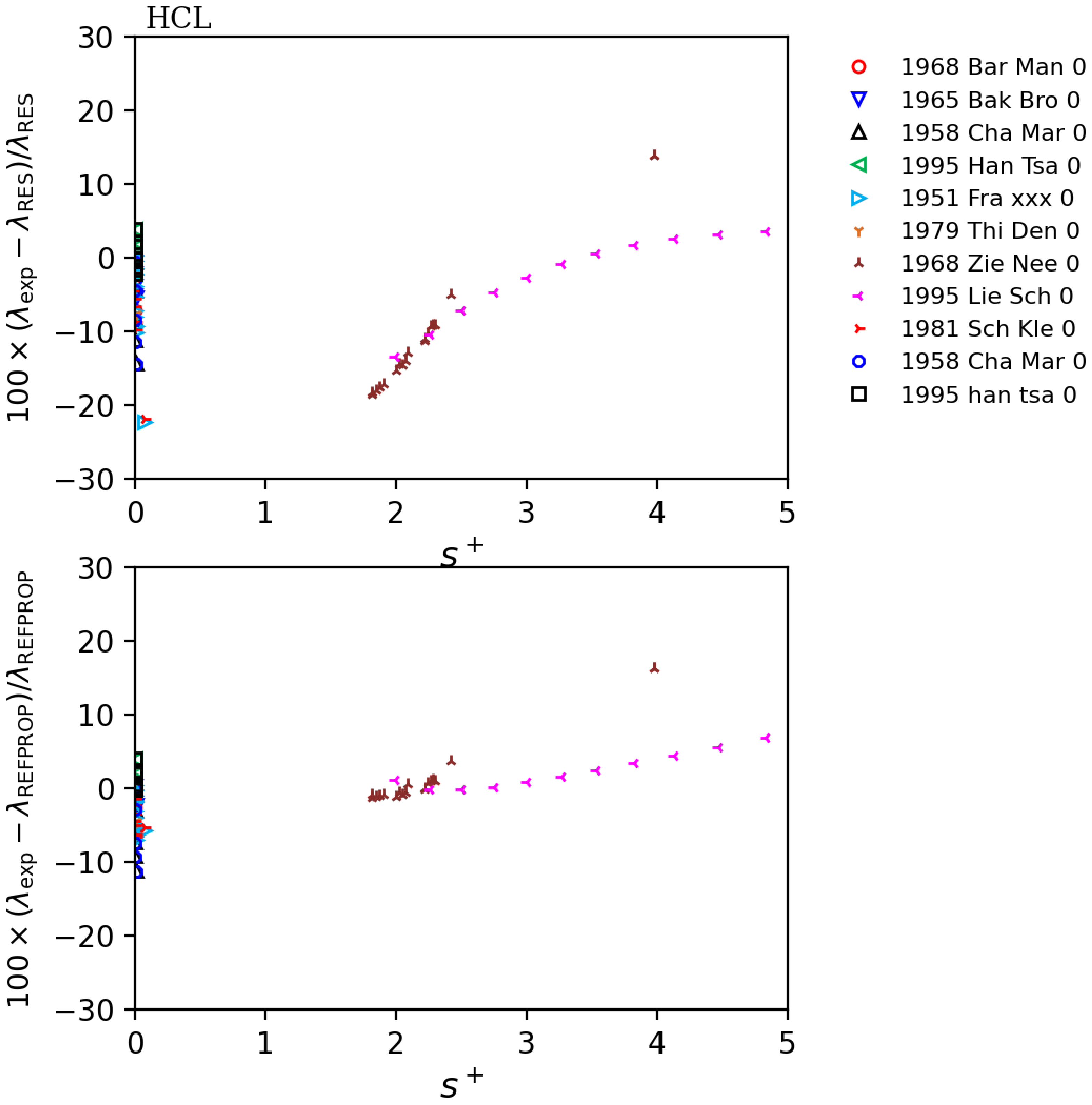

Figure DPR2. HCL

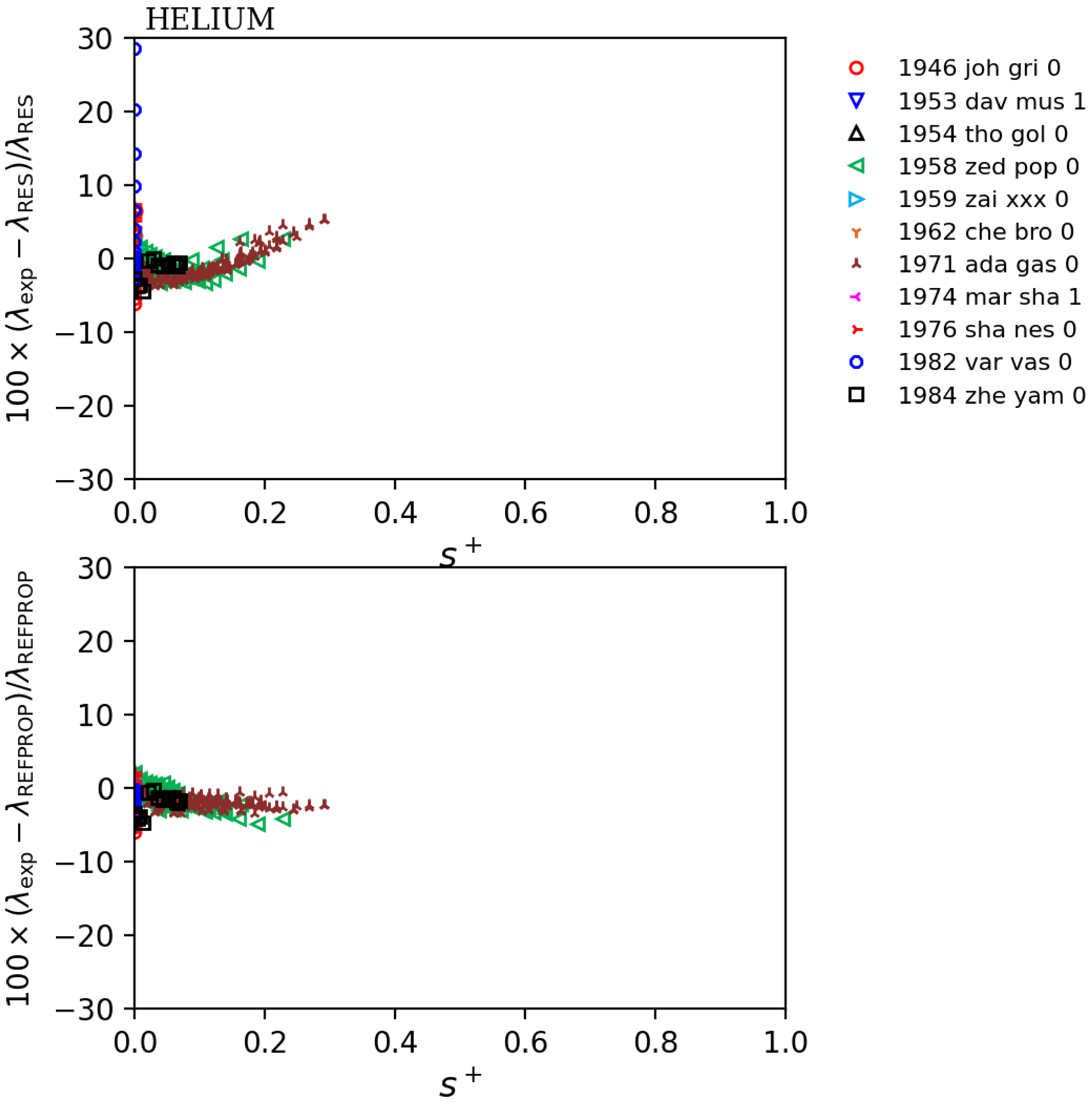

Figure DPR2. HELIUM

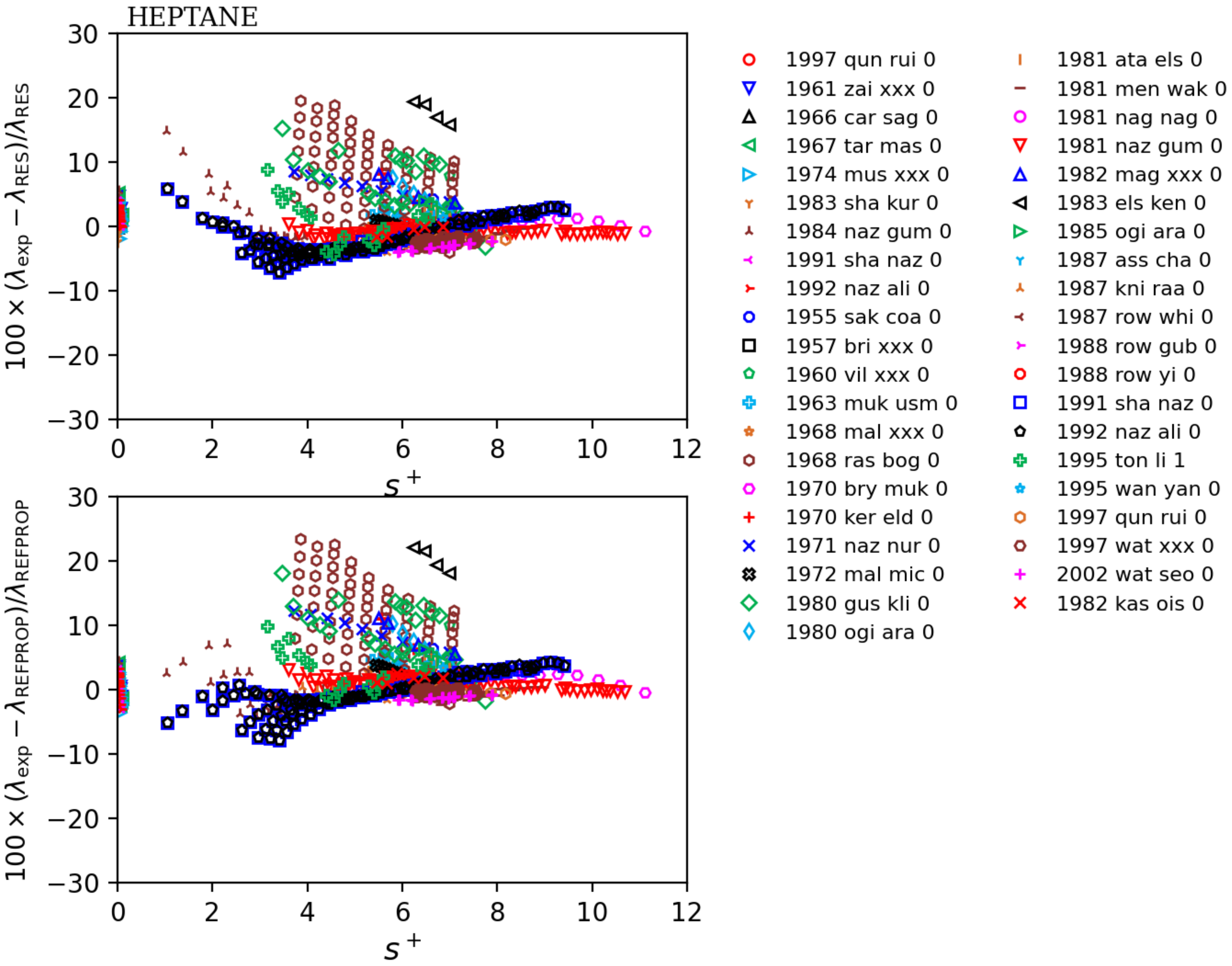

Figure DPR2. HEPTANE

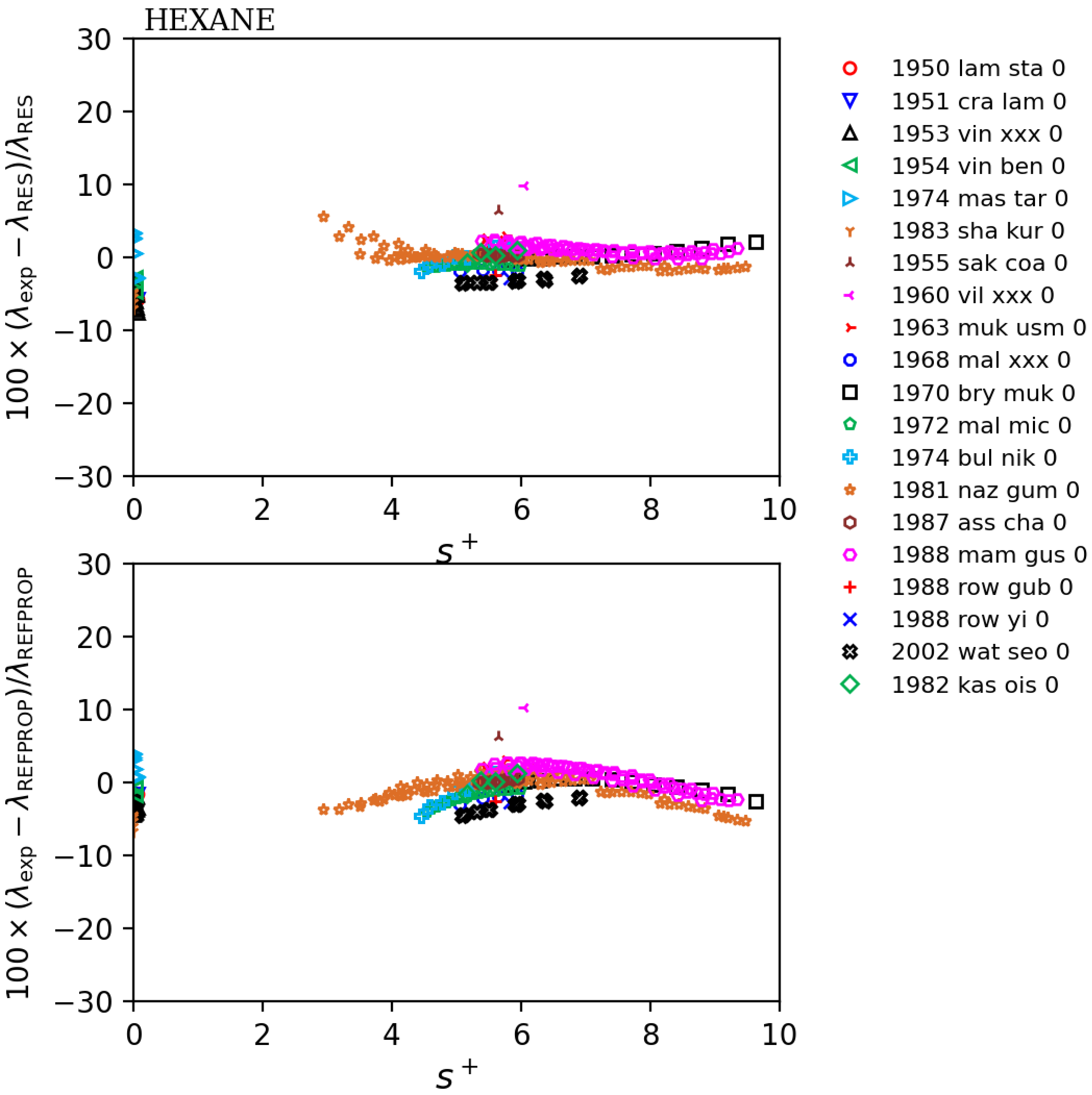

Figure DPR2. HEXANE

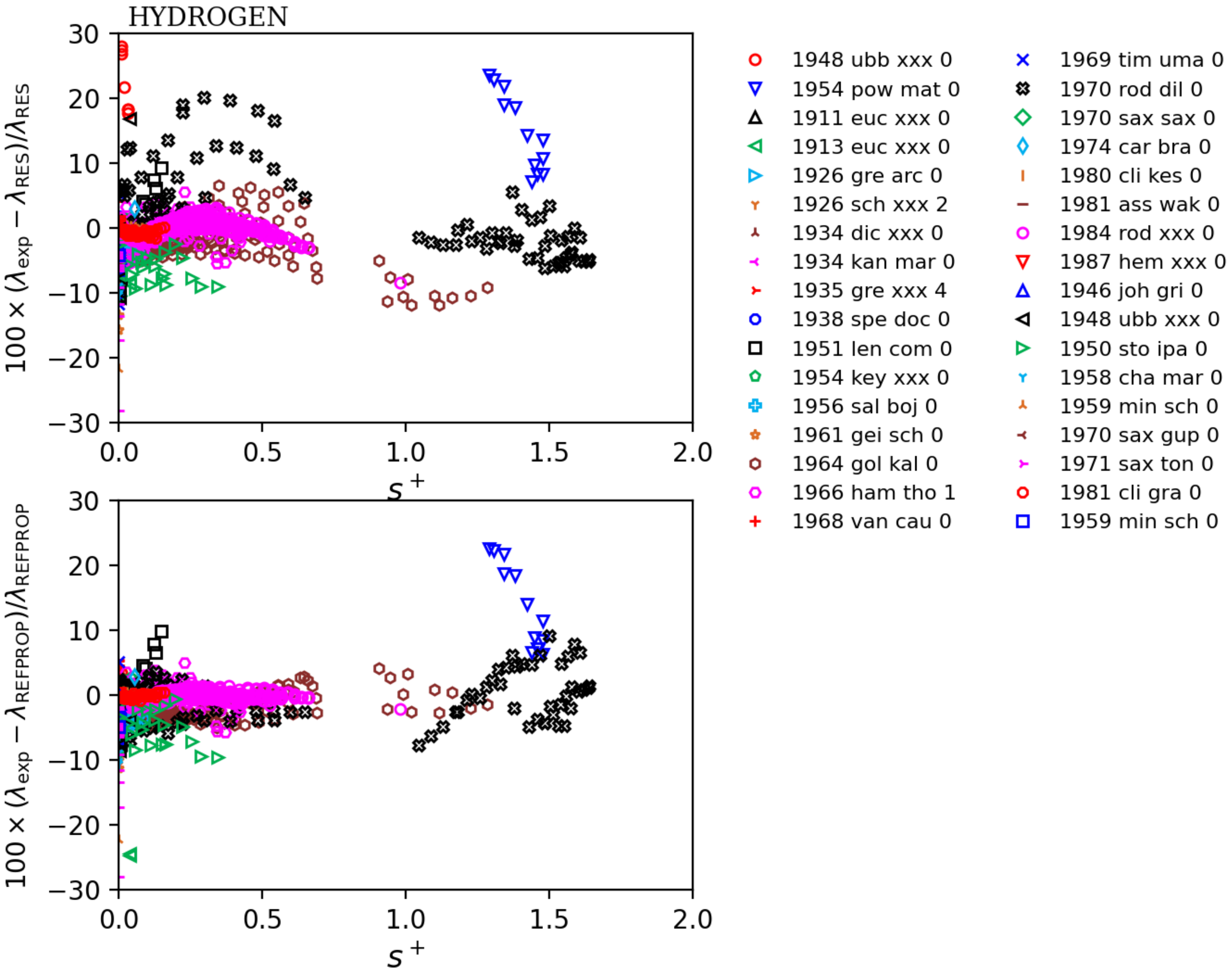

Figure DPR2. HYDROGEN

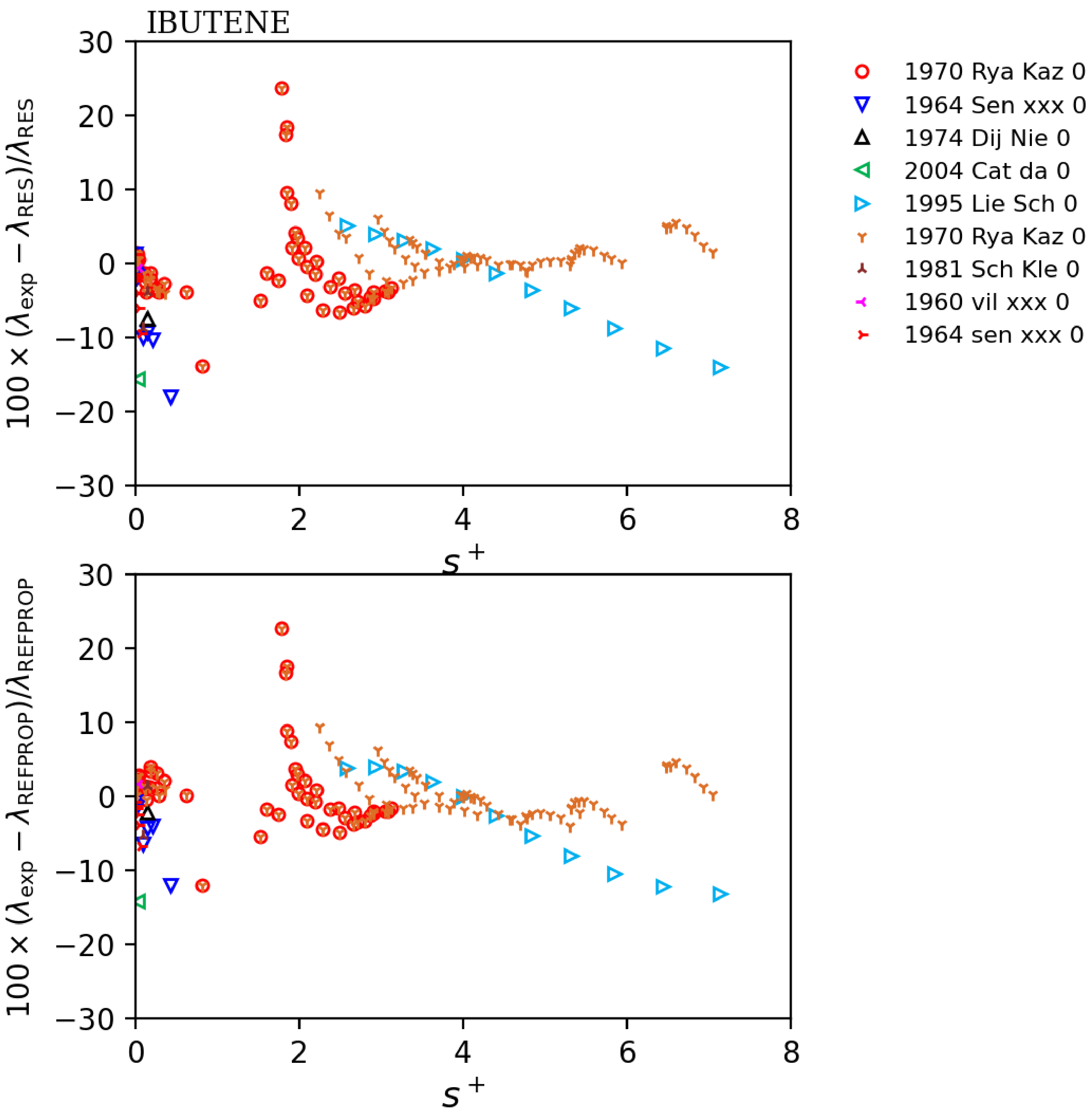

Figure DPR2. IBUTENE

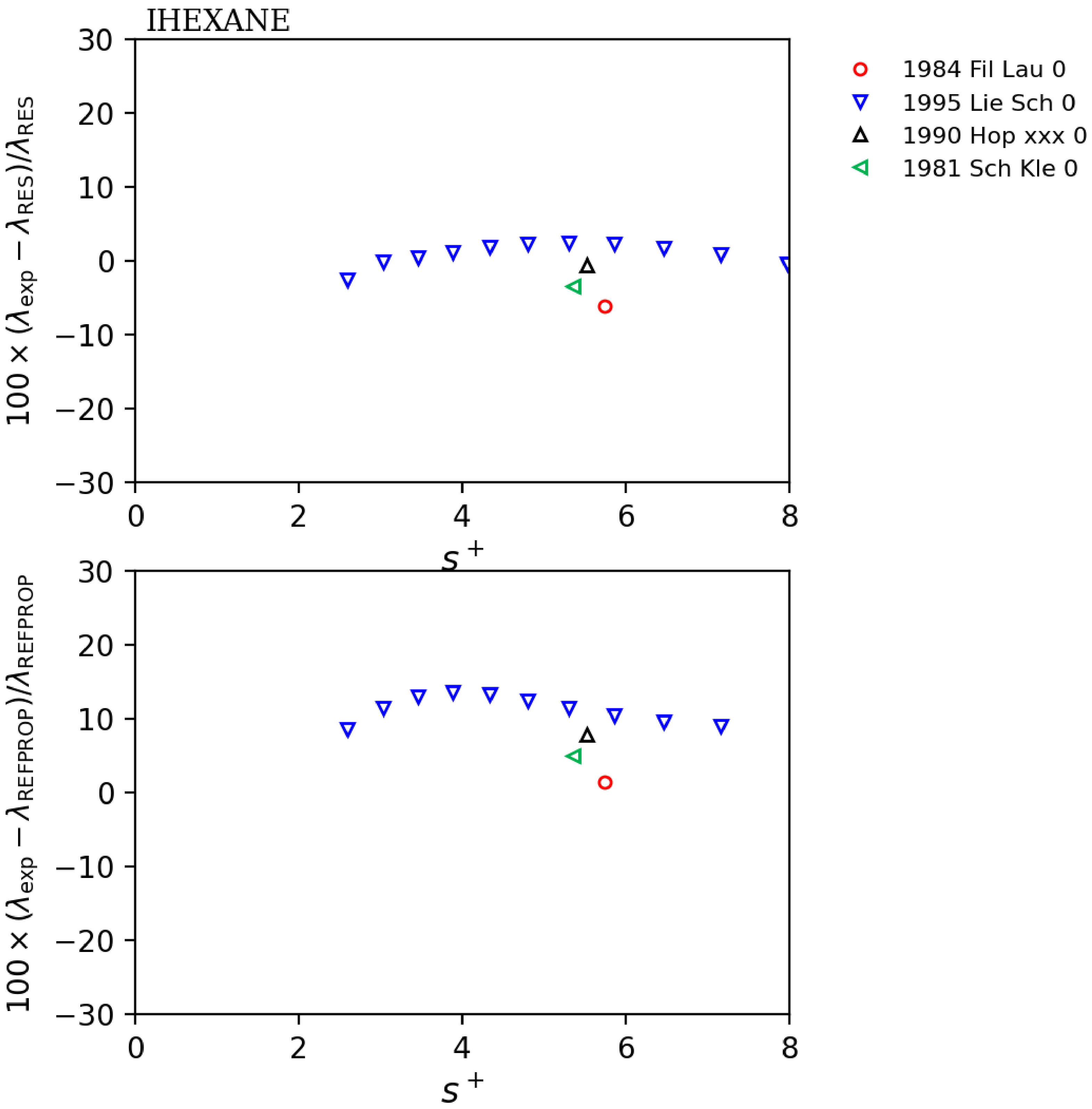

Figure DPR2. IHEXANE

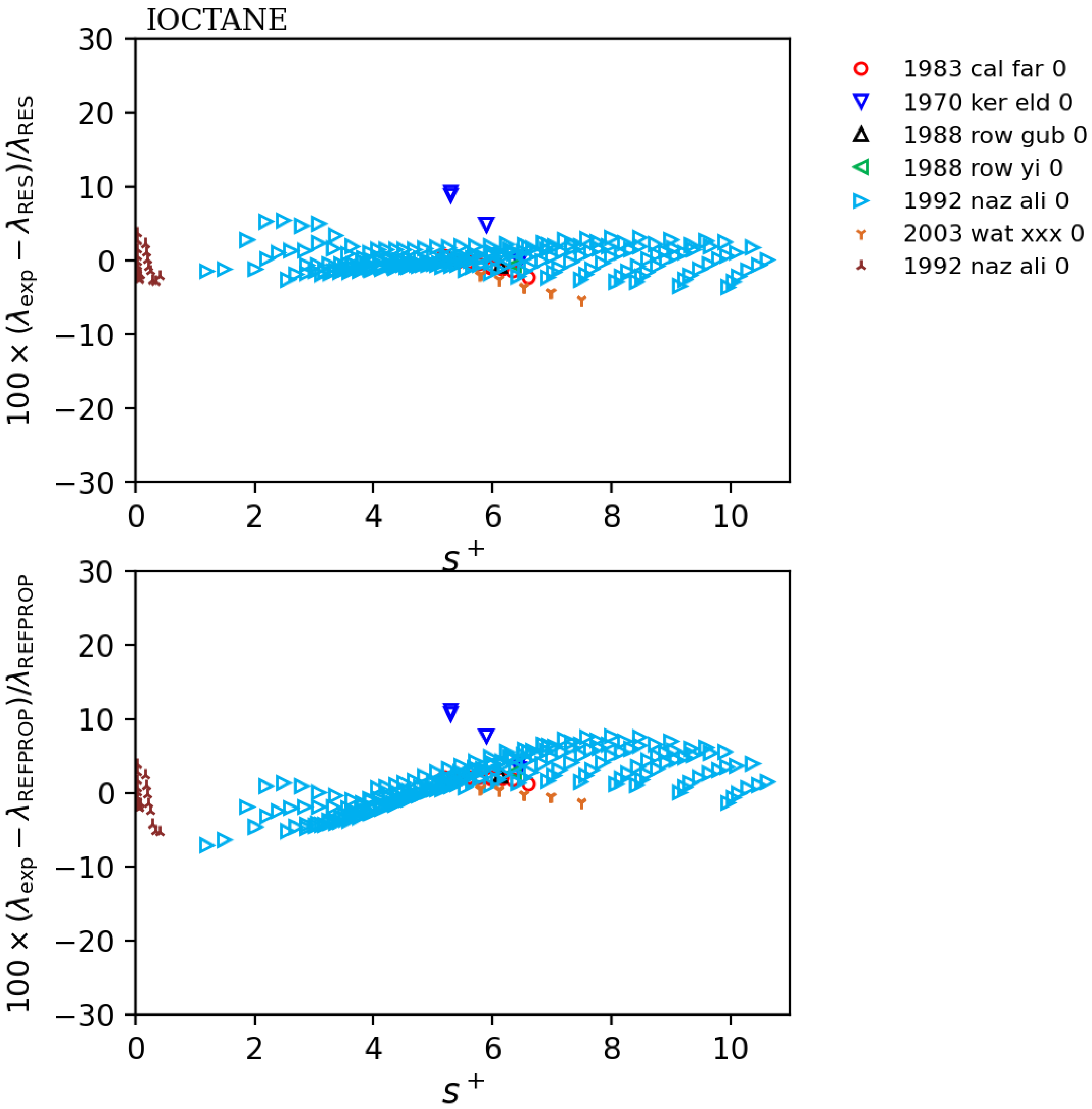

Figure DPR2. IOCTANE

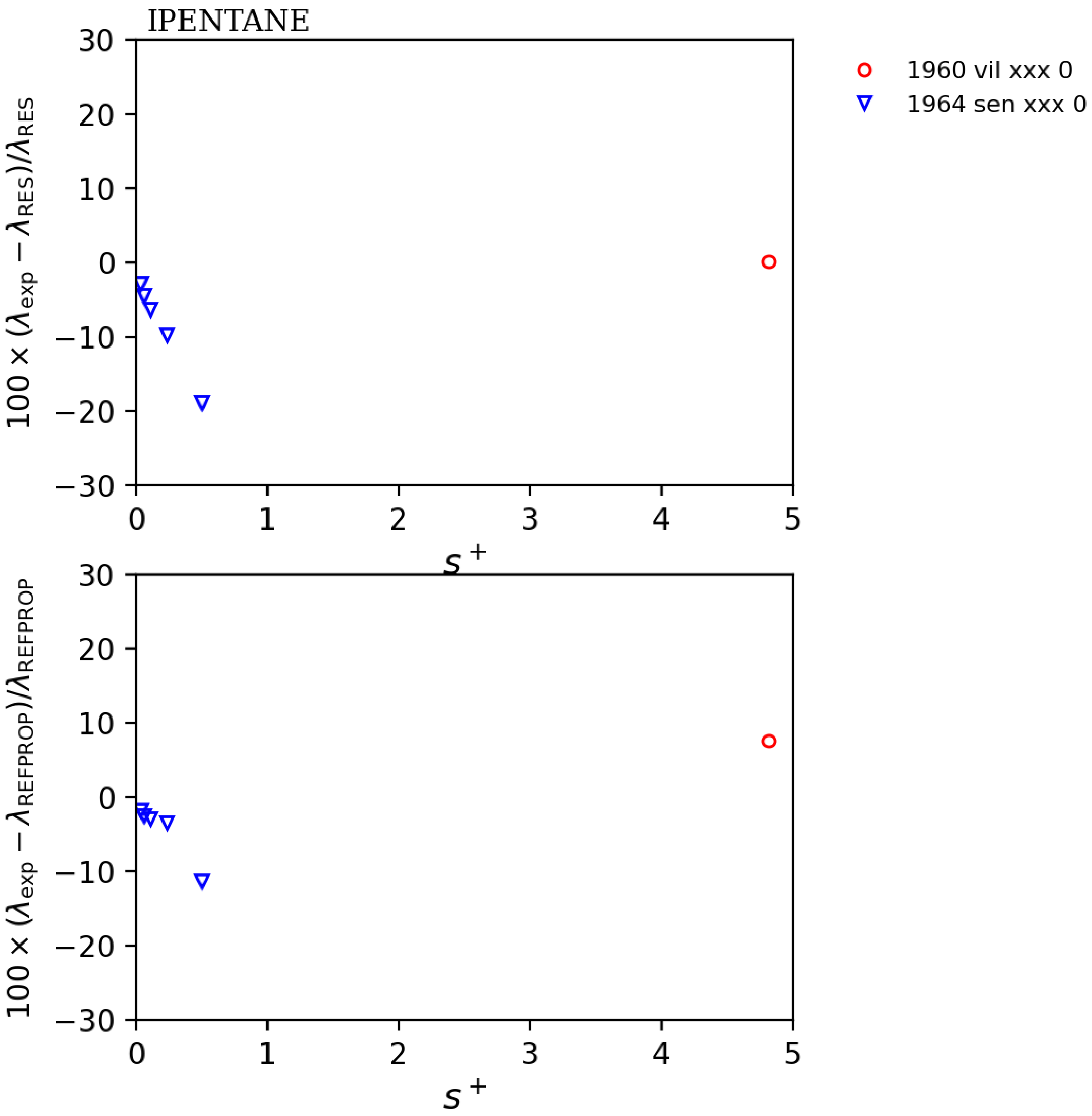

Figure DPR2. IPENTANE

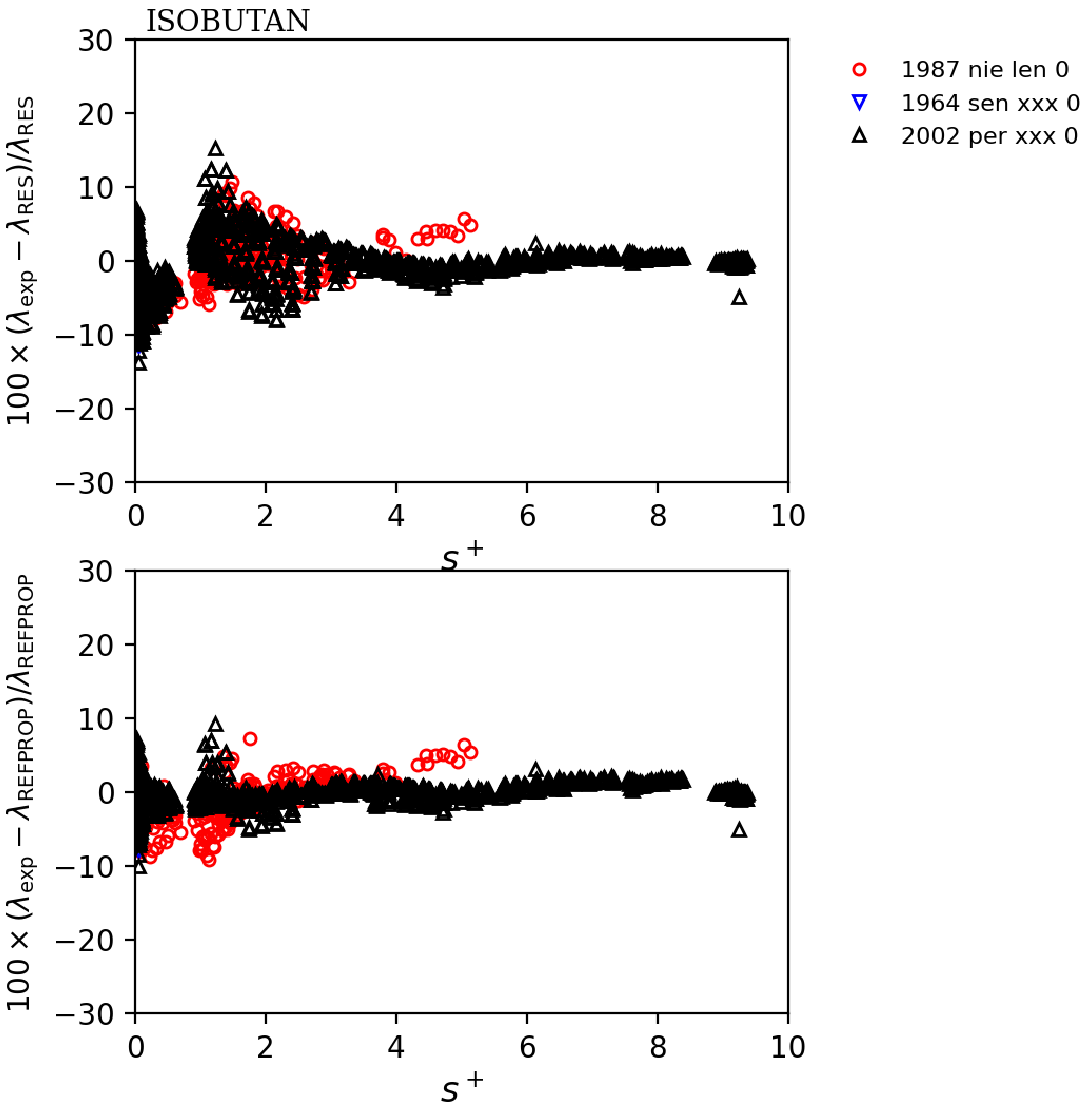

Figure DPR2. ISOBUTAN

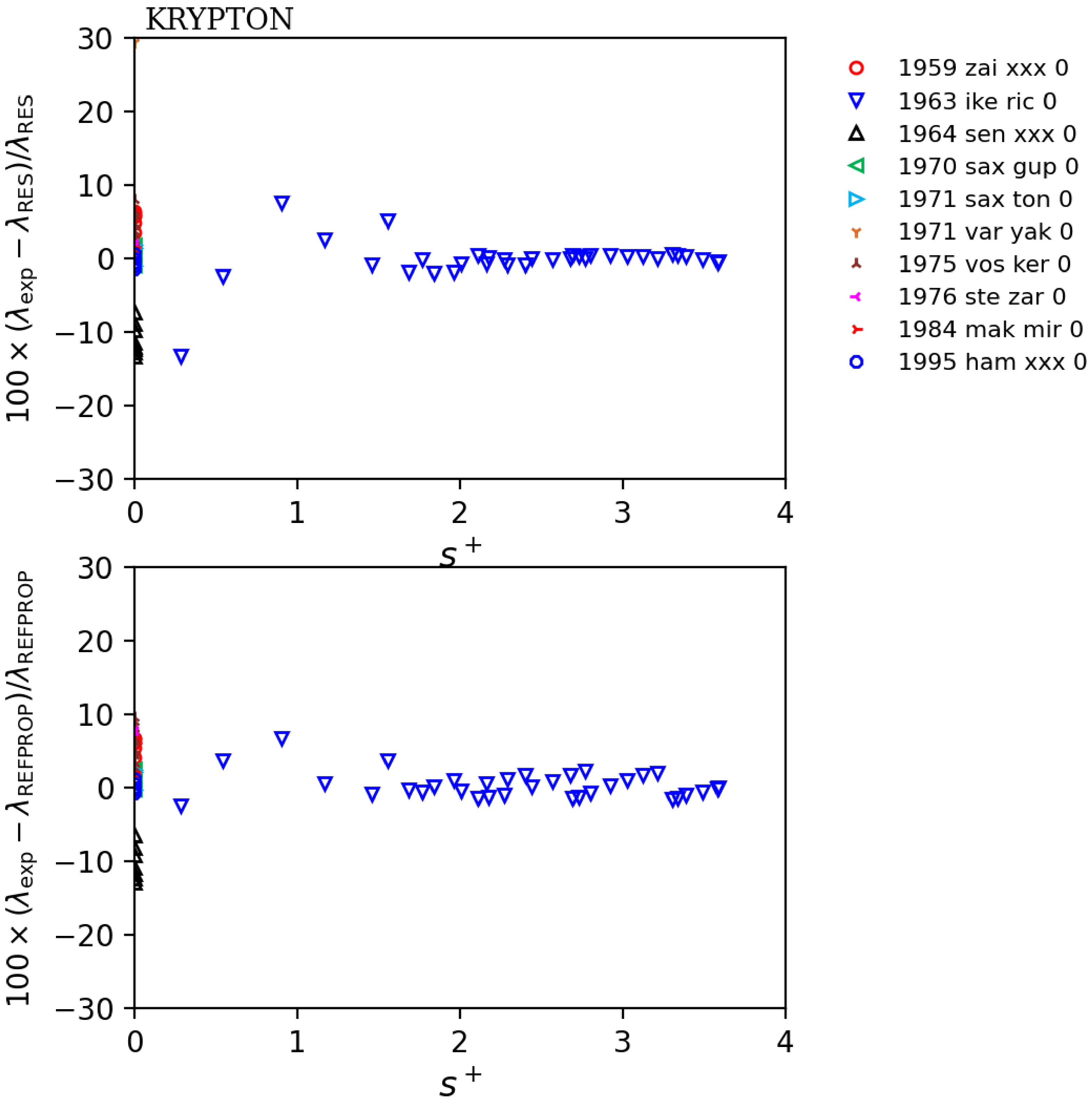

Figure DPR2. KRYPTON

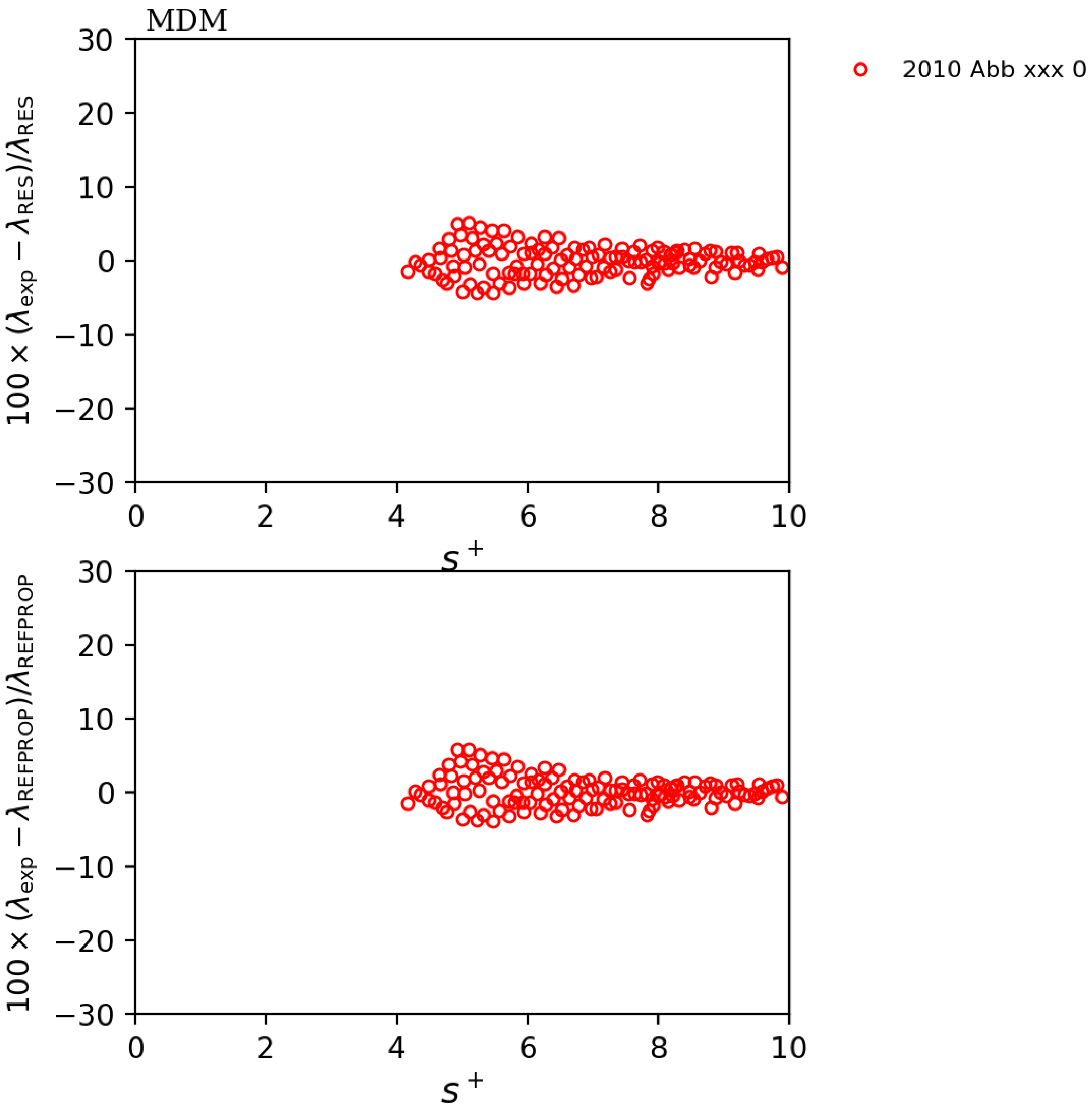

Figure DPR2. MDM

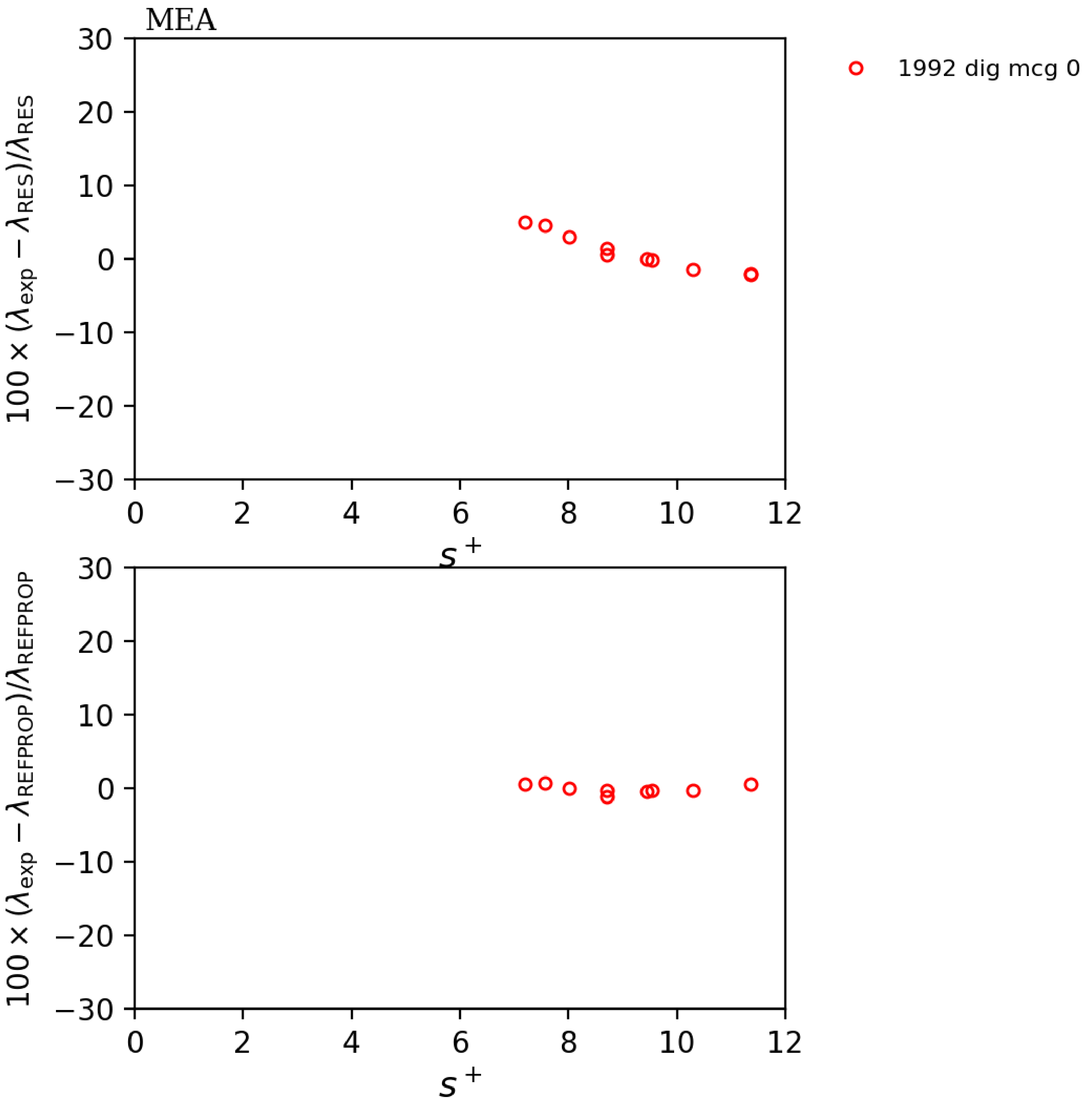

Figure DPR2. MEA

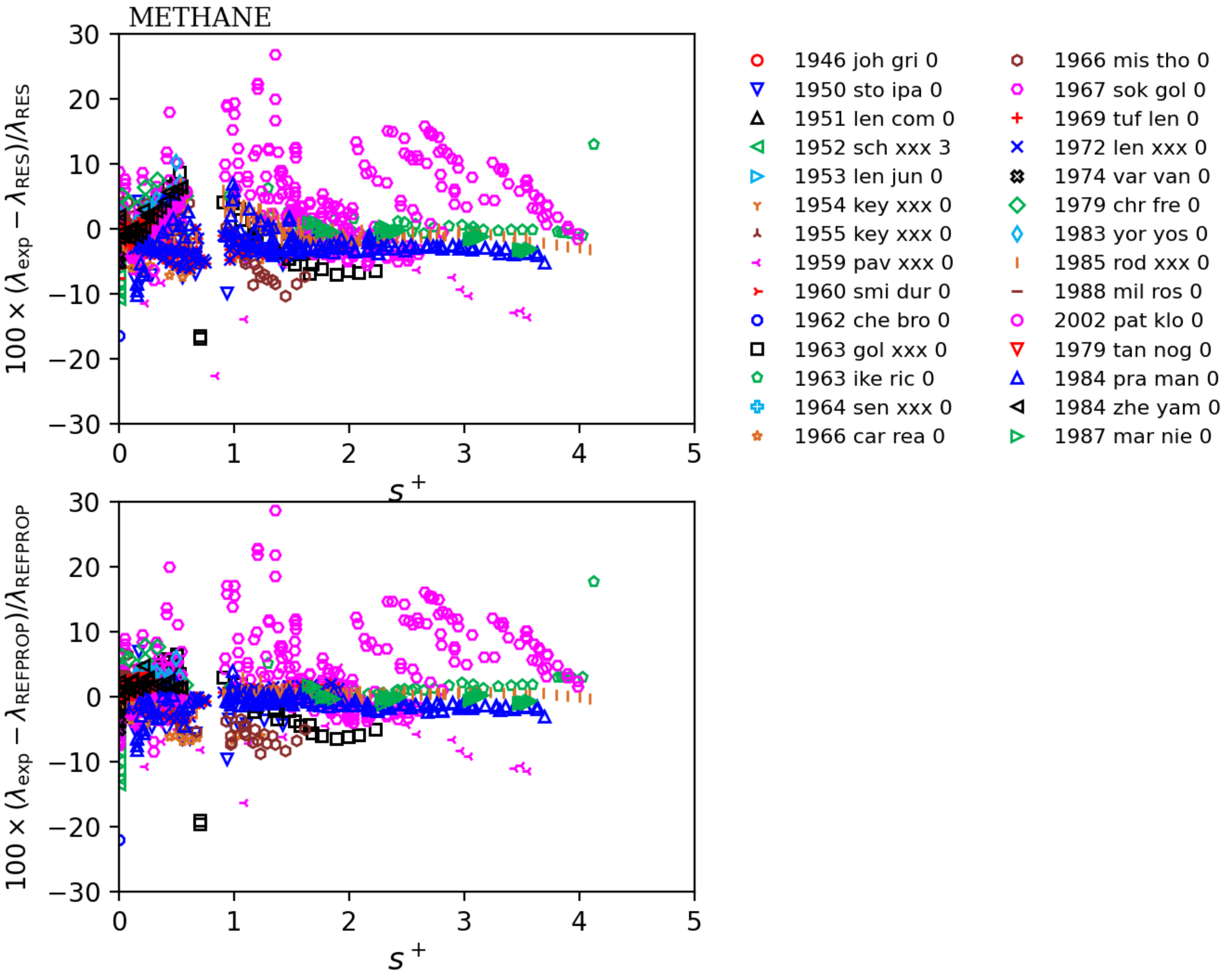

Figure DPR2. METHANE

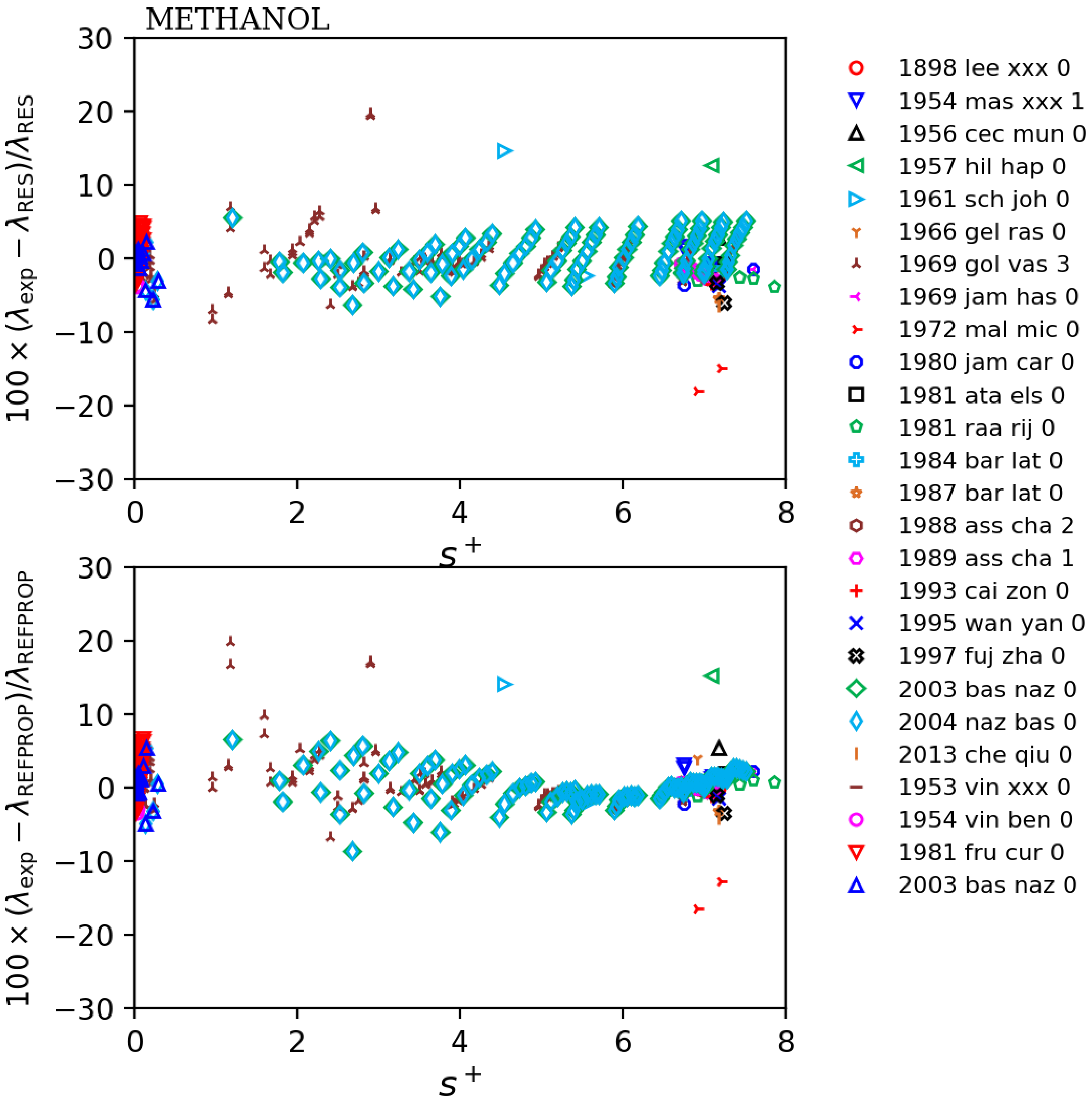

Figure DPR2. METHANOL

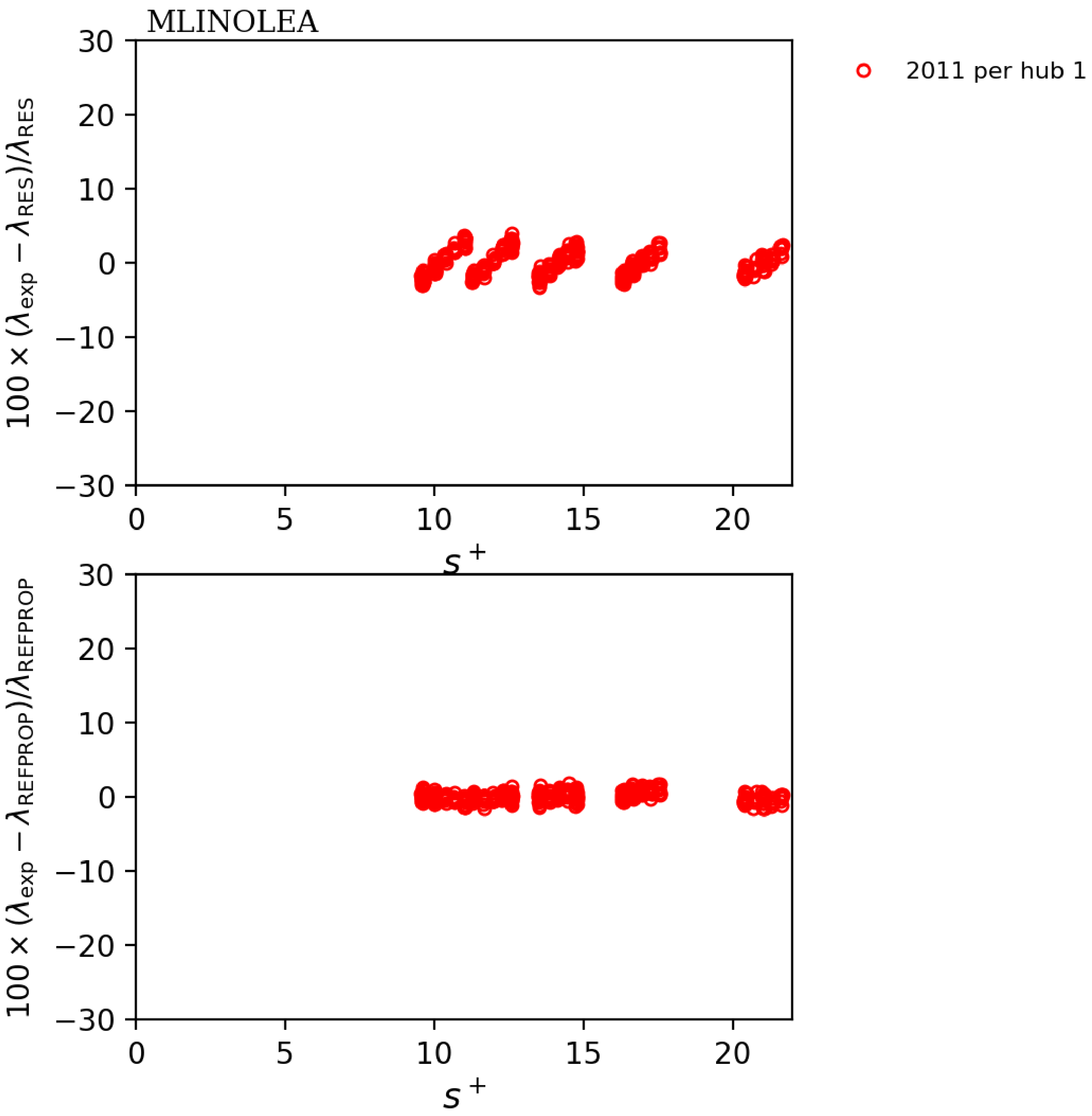

Figure DPR2. MLINOLEA

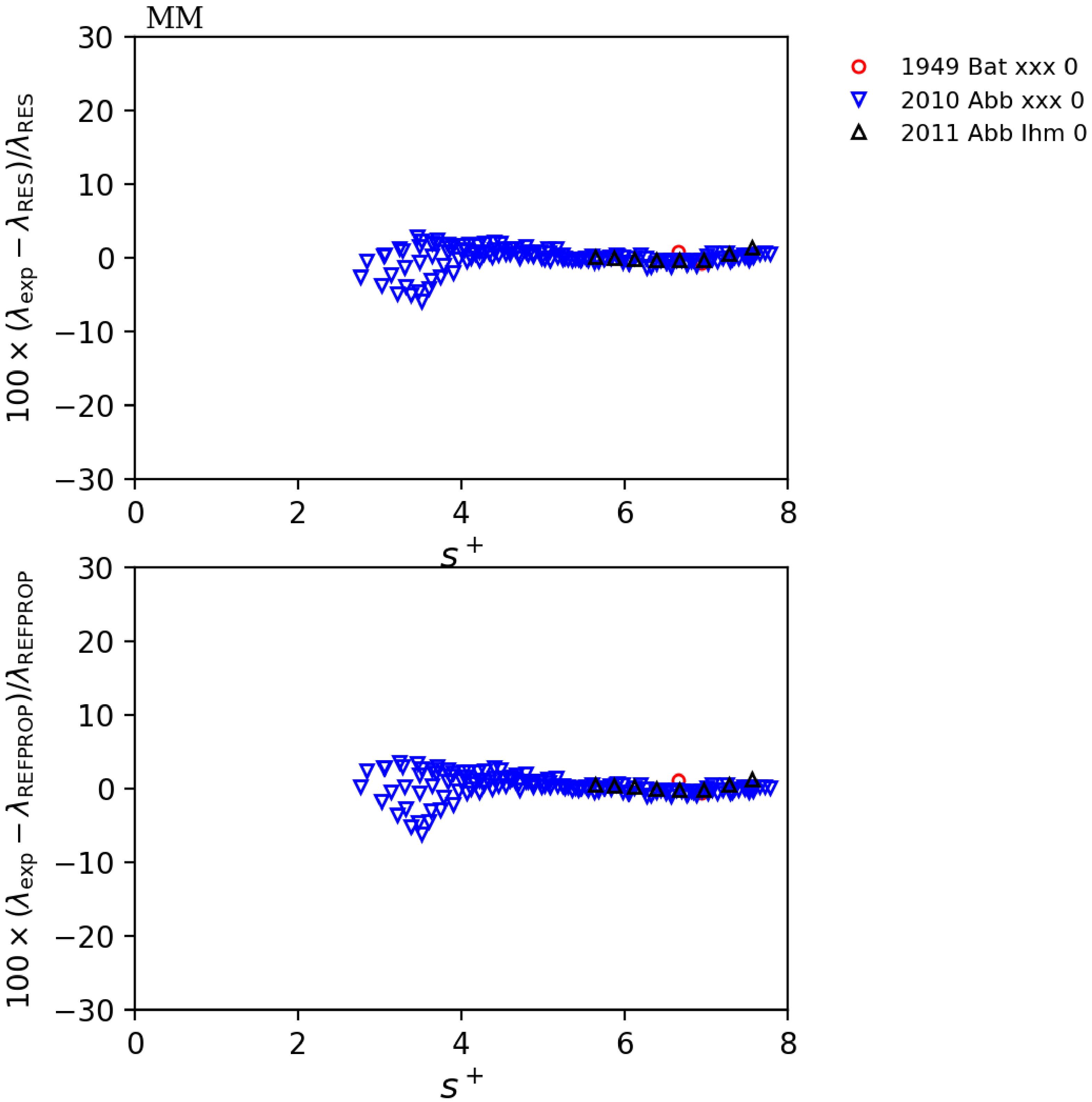

Figure DPR2. MM

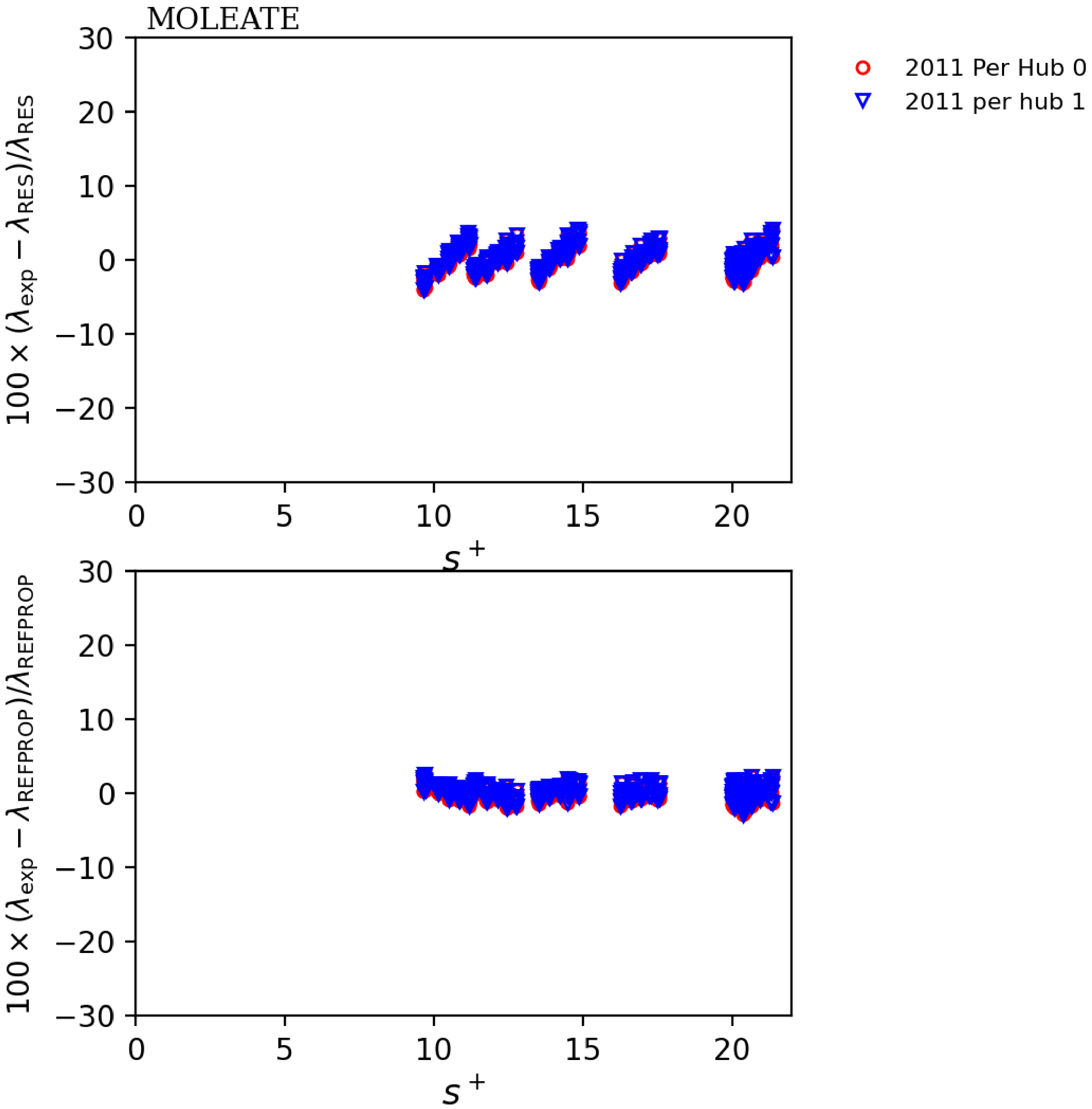

Figure DPR2. MOLEATE

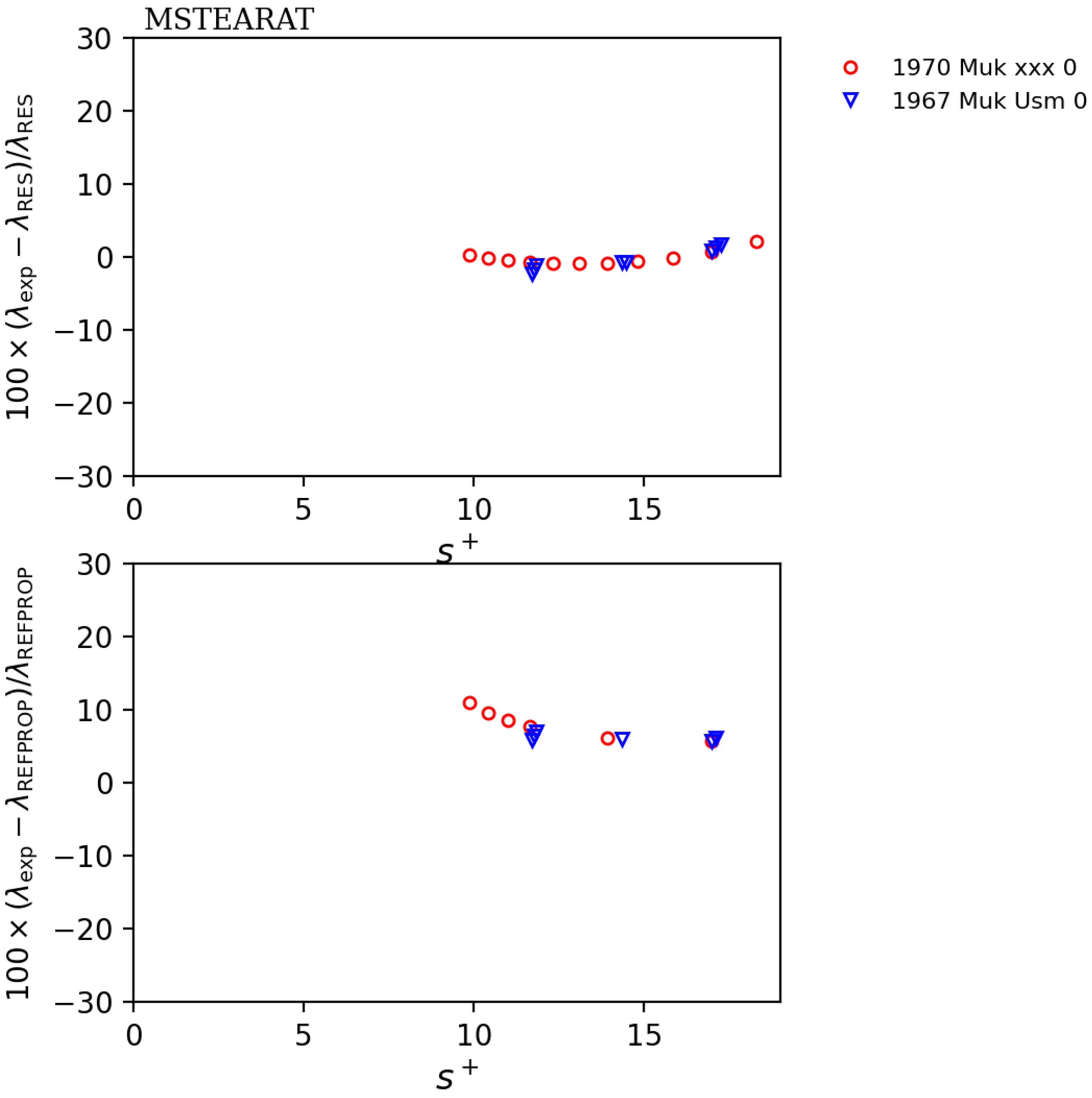

Figure DPR2. MSTEARAT

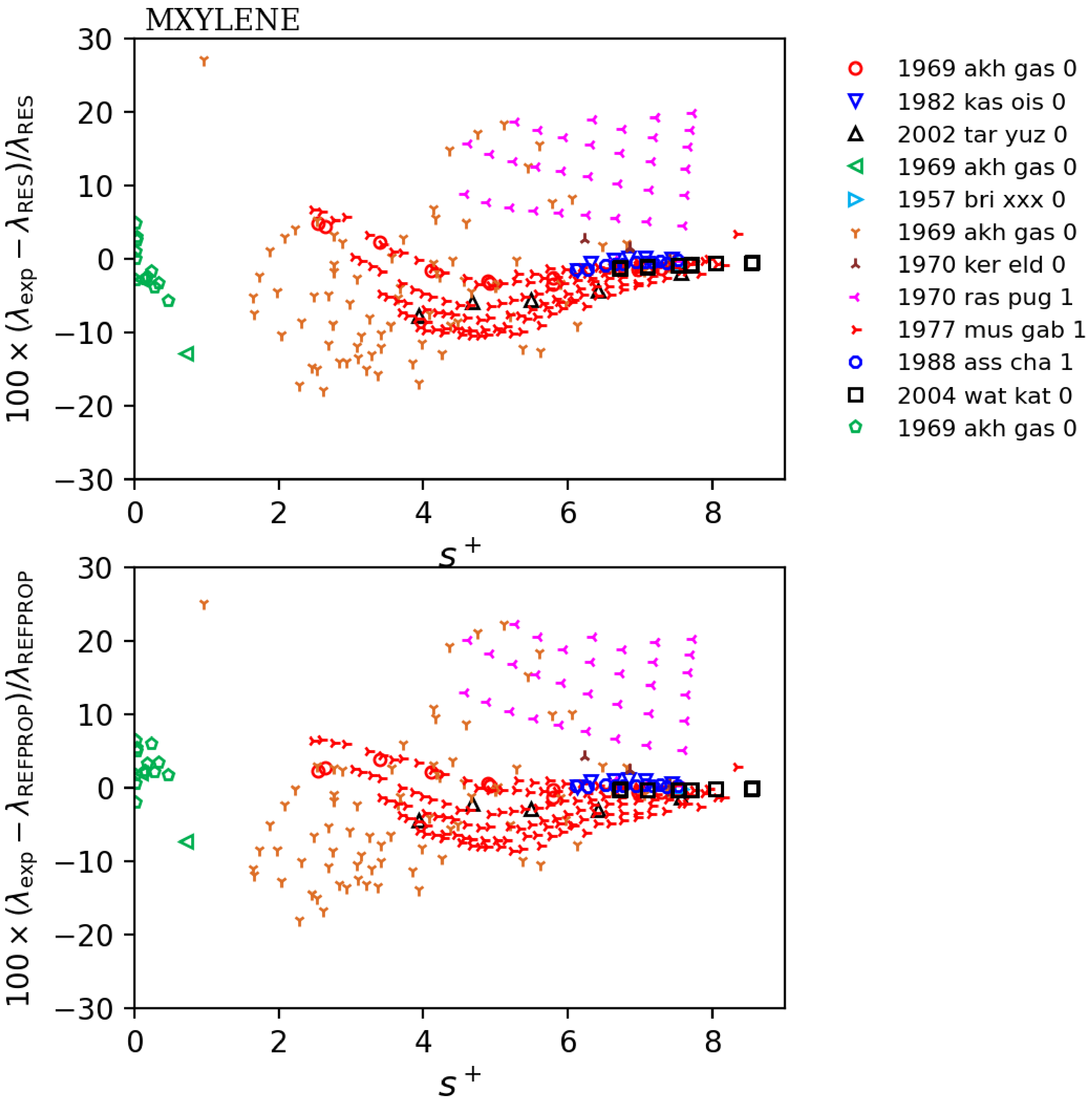

Figure DPR2. MXYLENE

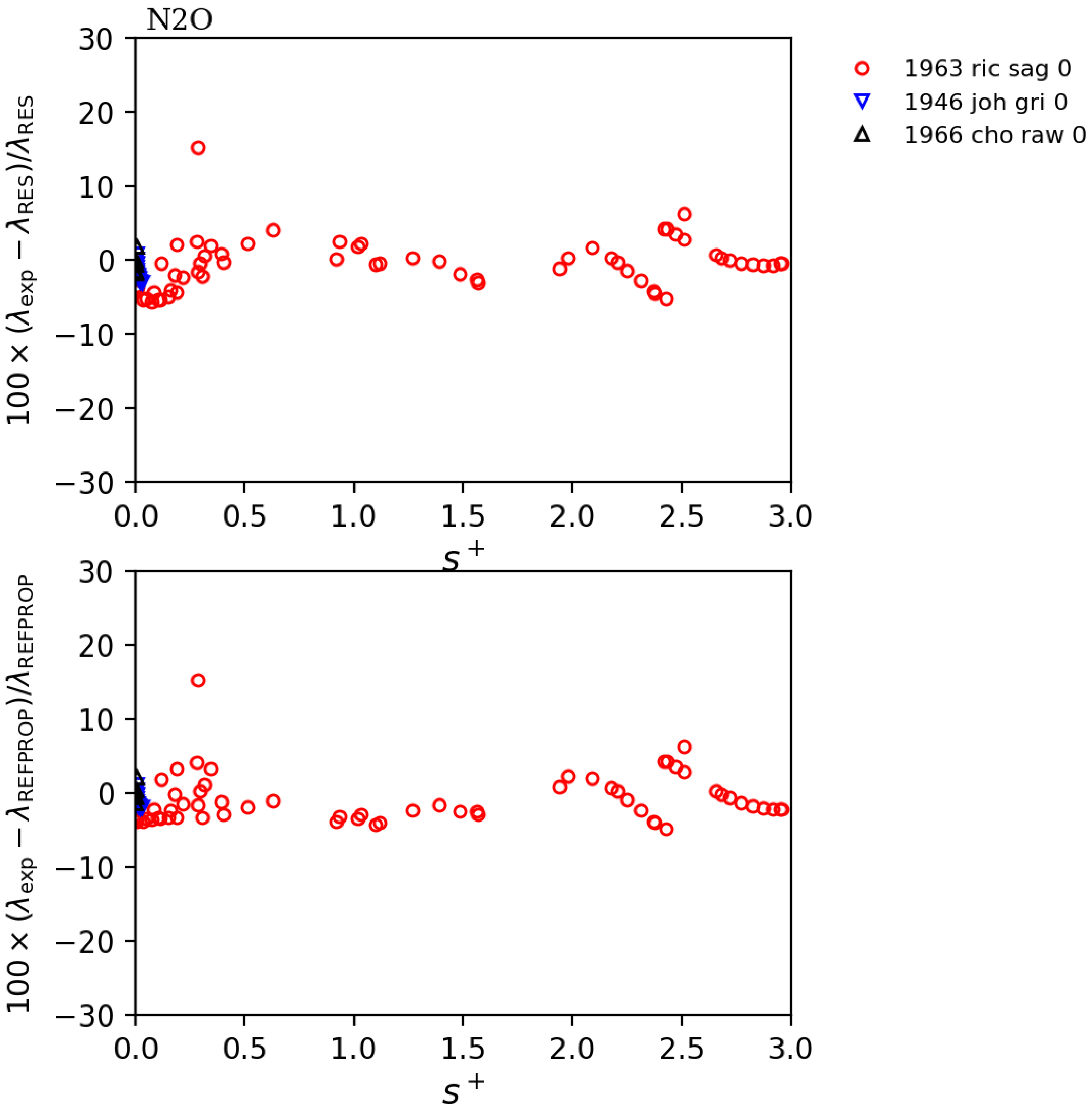

Figure DPR2. N2O

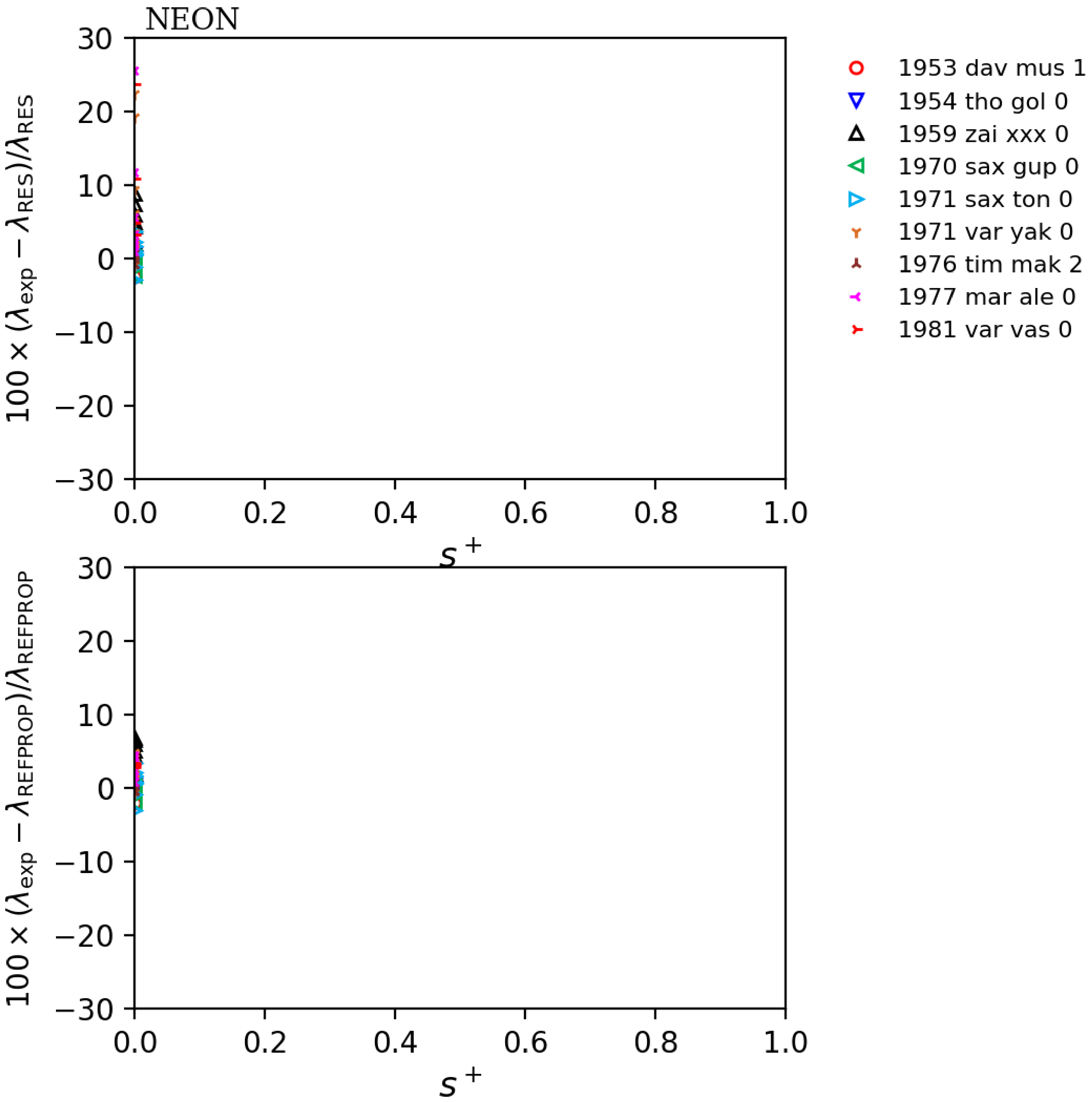

Figure DPR2. NEON

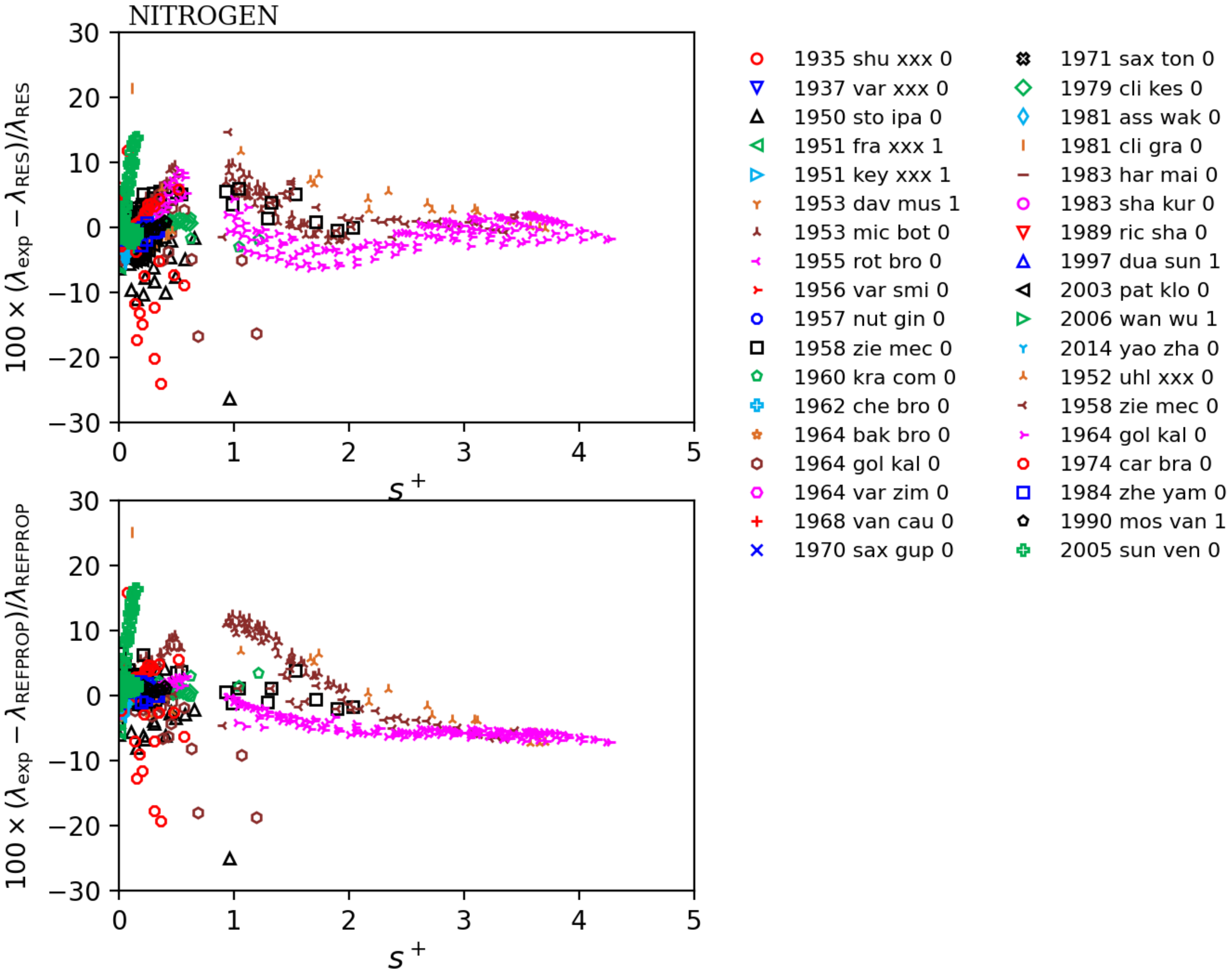

Figure DPR2. NITROGEN

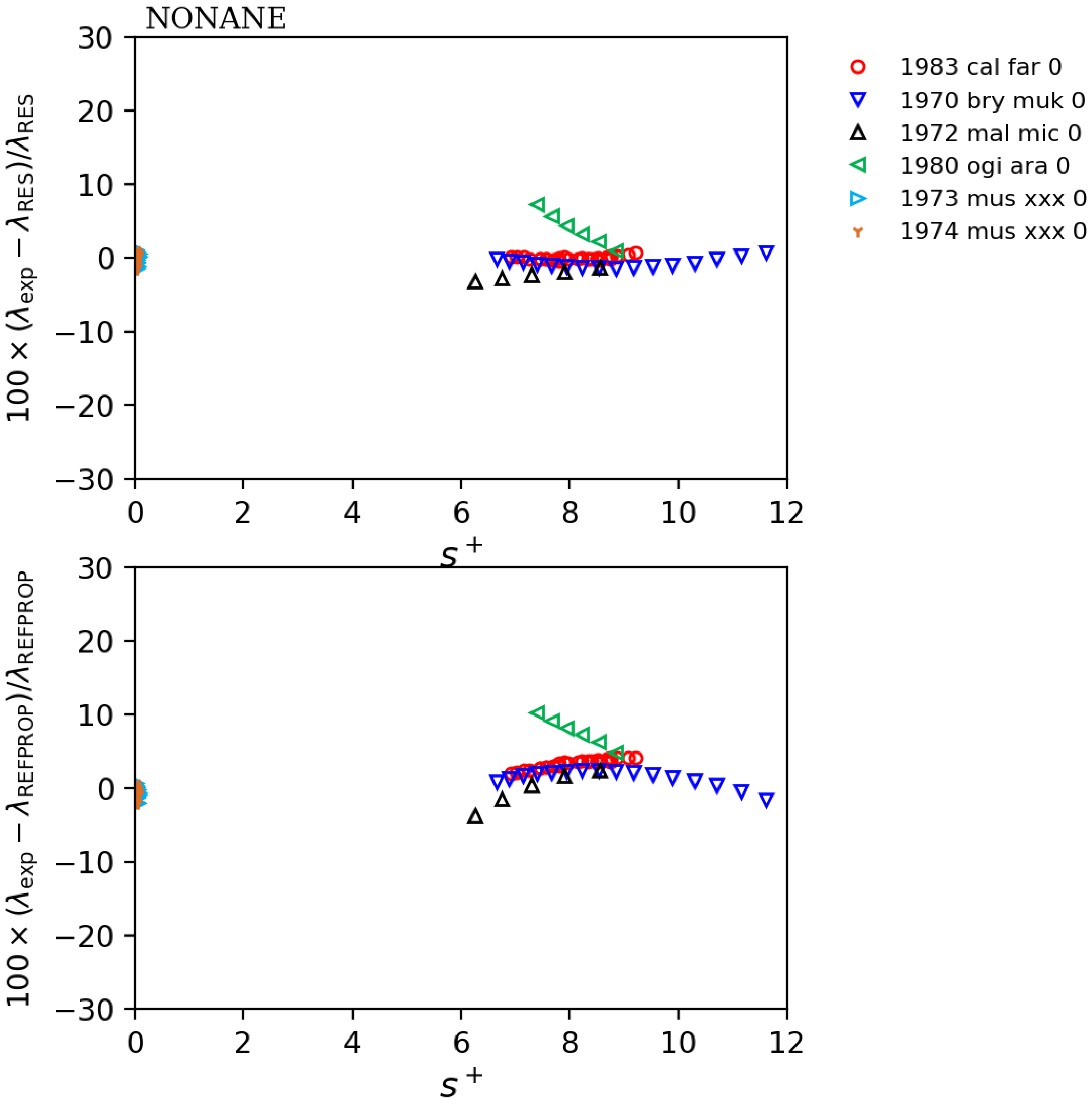

Figure DPR2. NONANE

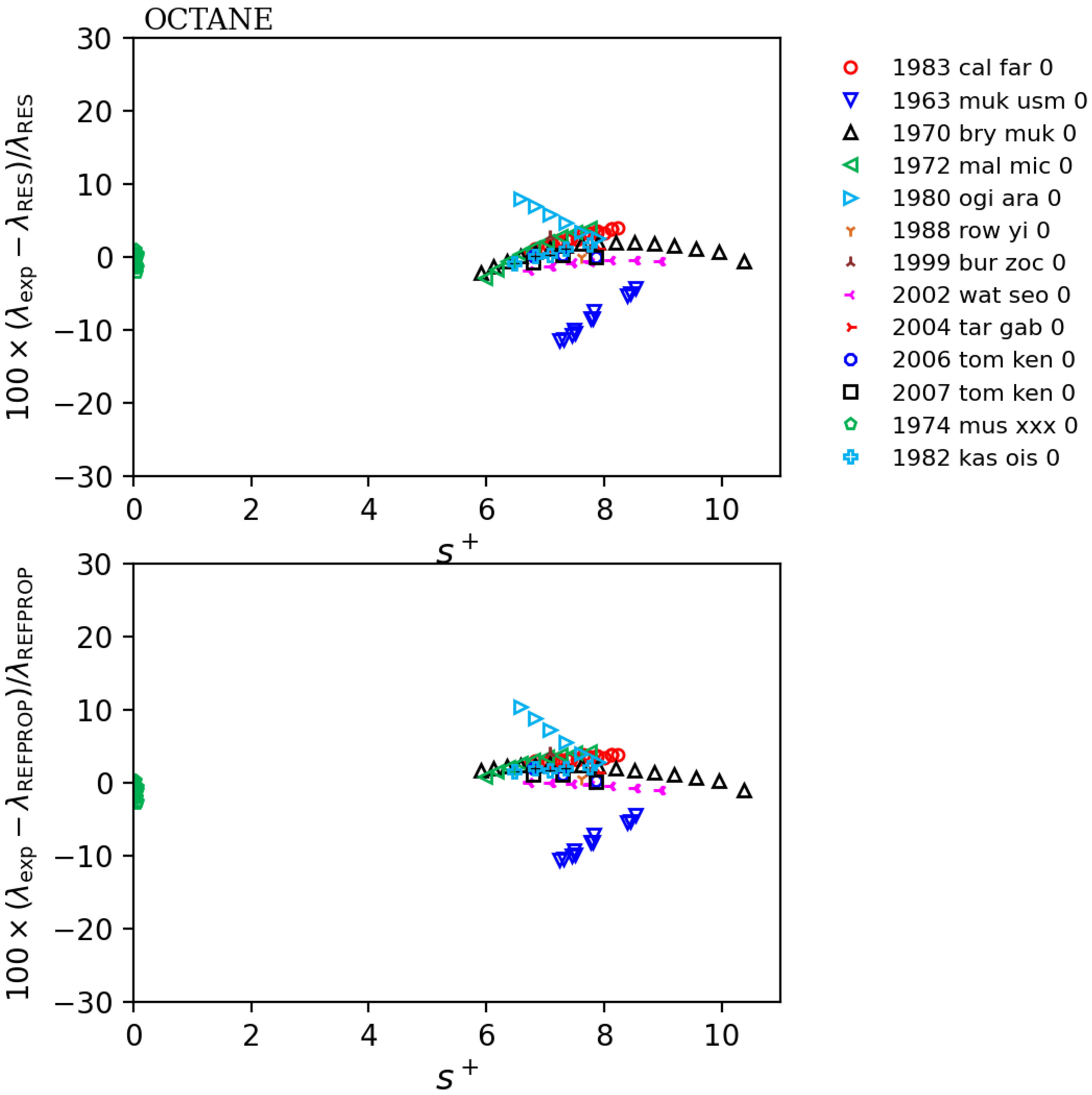

Figure DPR2. OCTANE

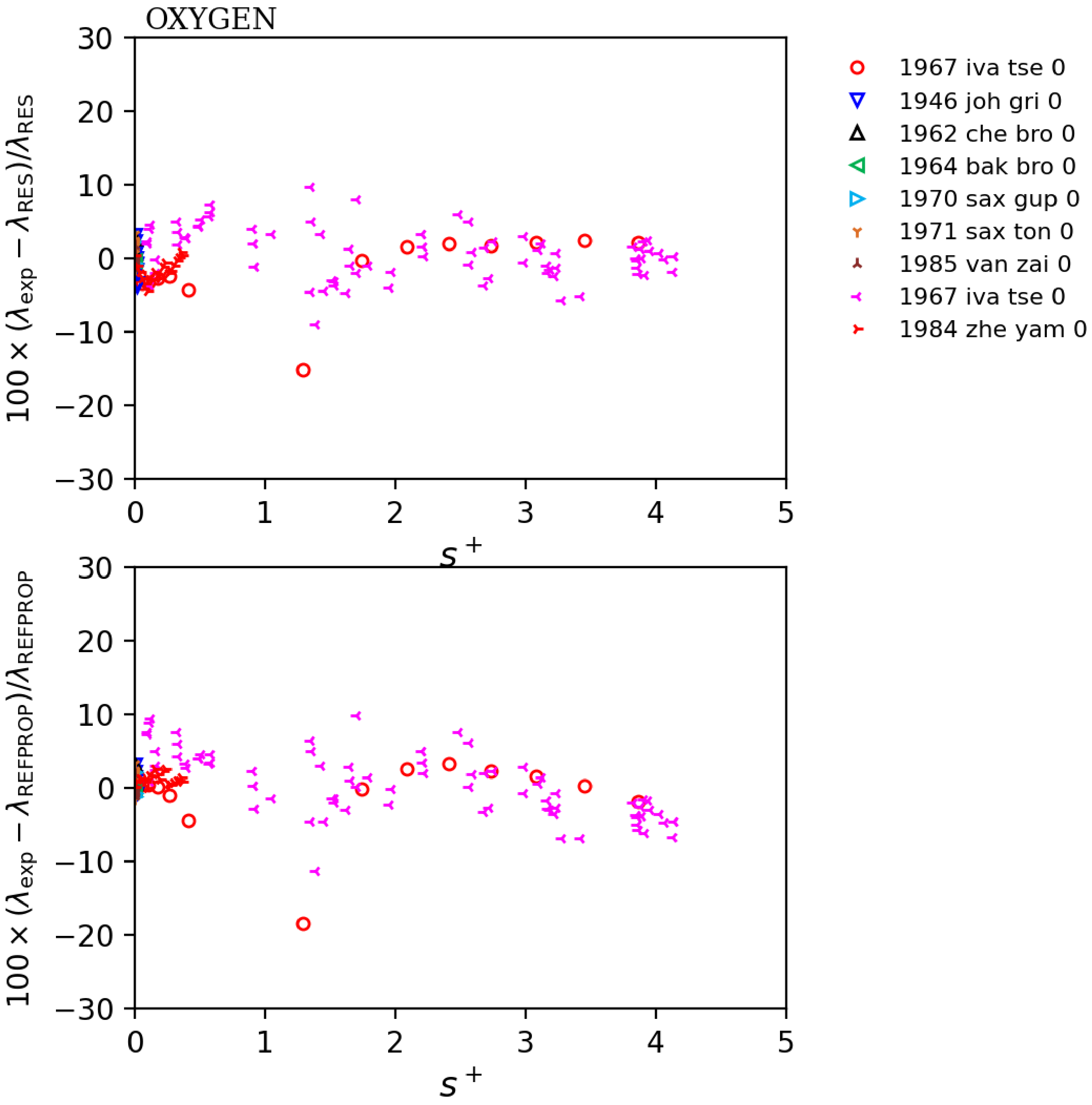

Figure DPR2. OXYGEN

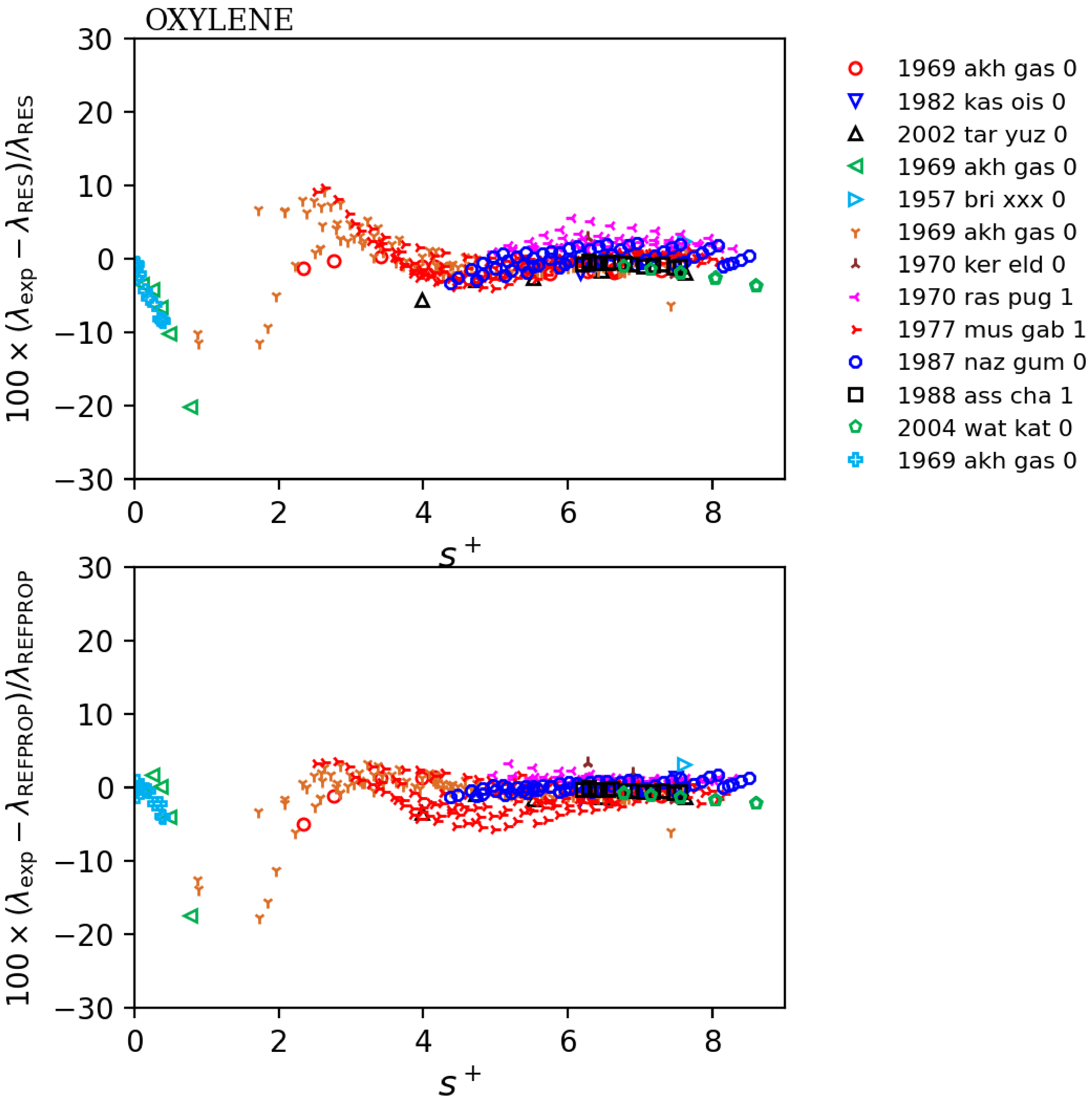

Figure DPR2. OXYLENE

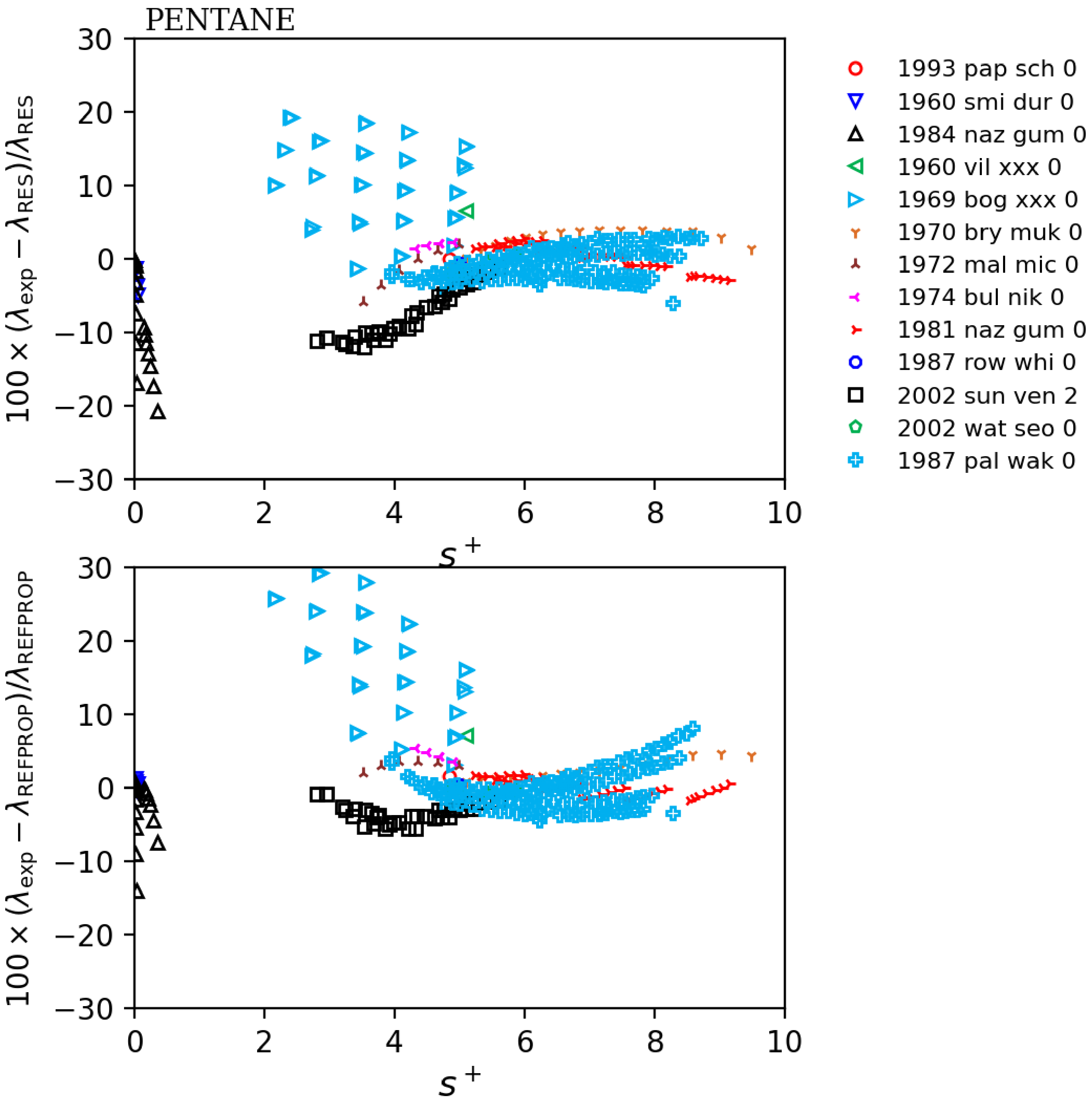

Figure DPR2. PENTANE

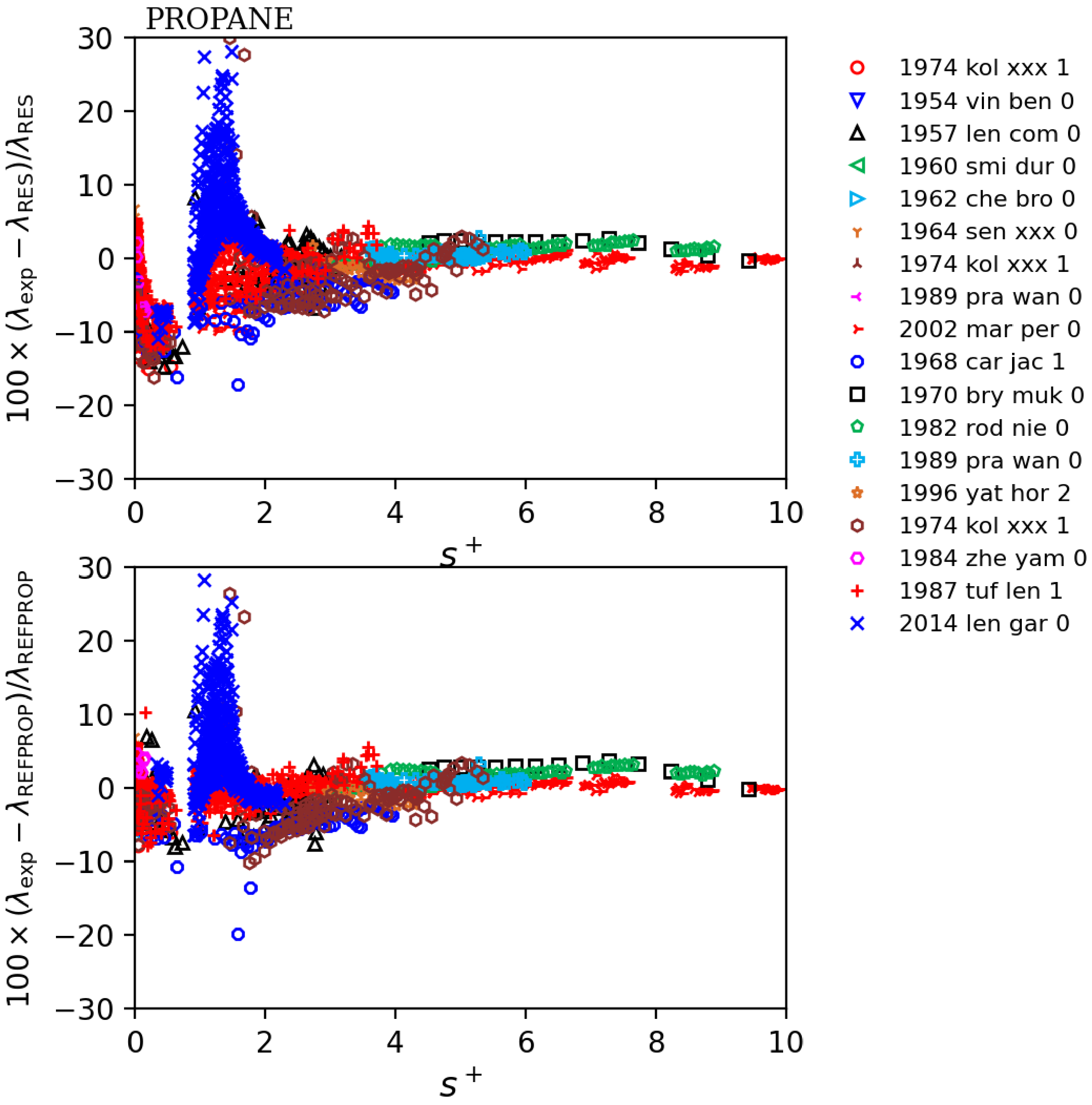

Figure DPR2. PROPANE

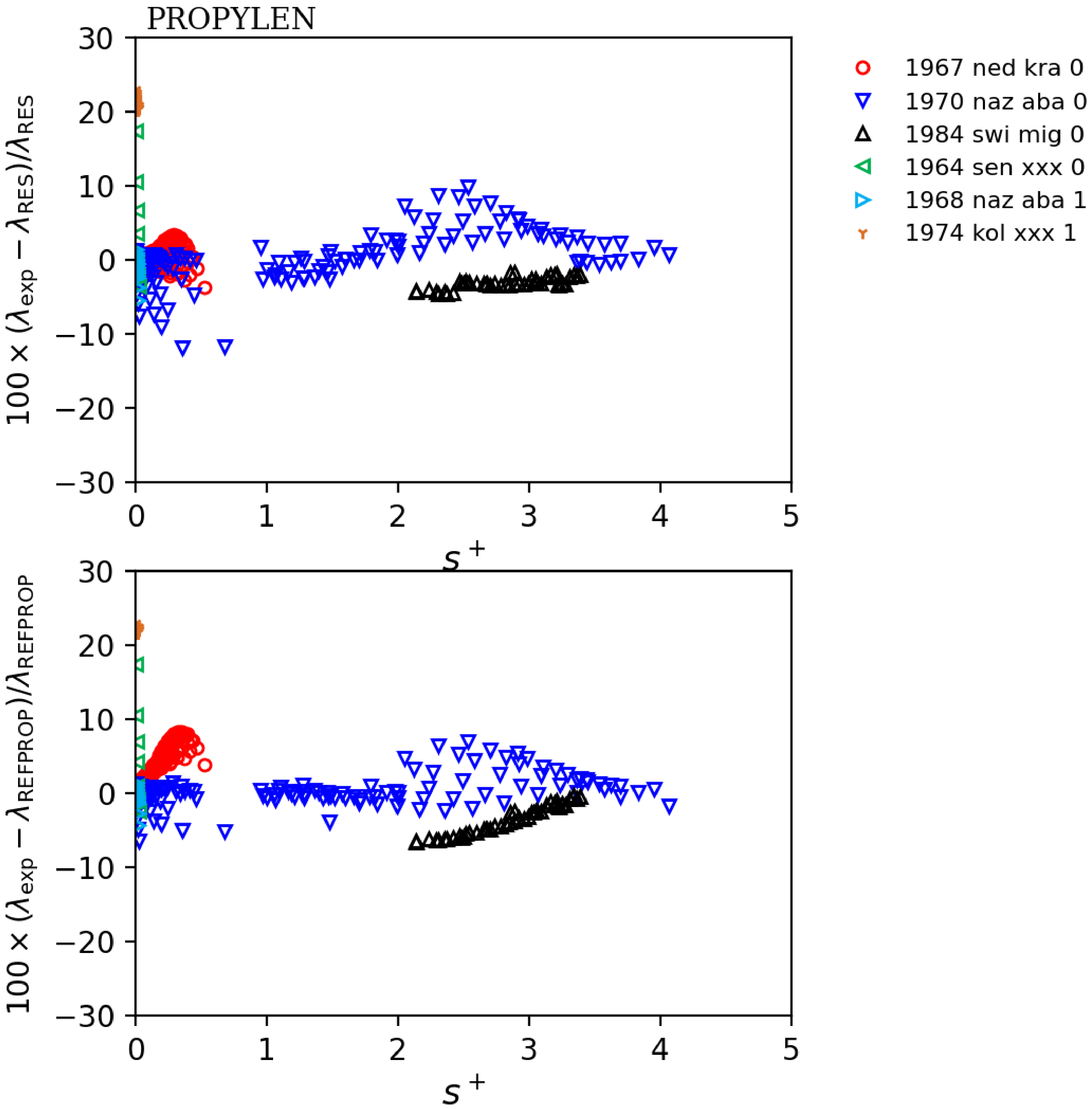

Figure DPR2. PROPYLEN

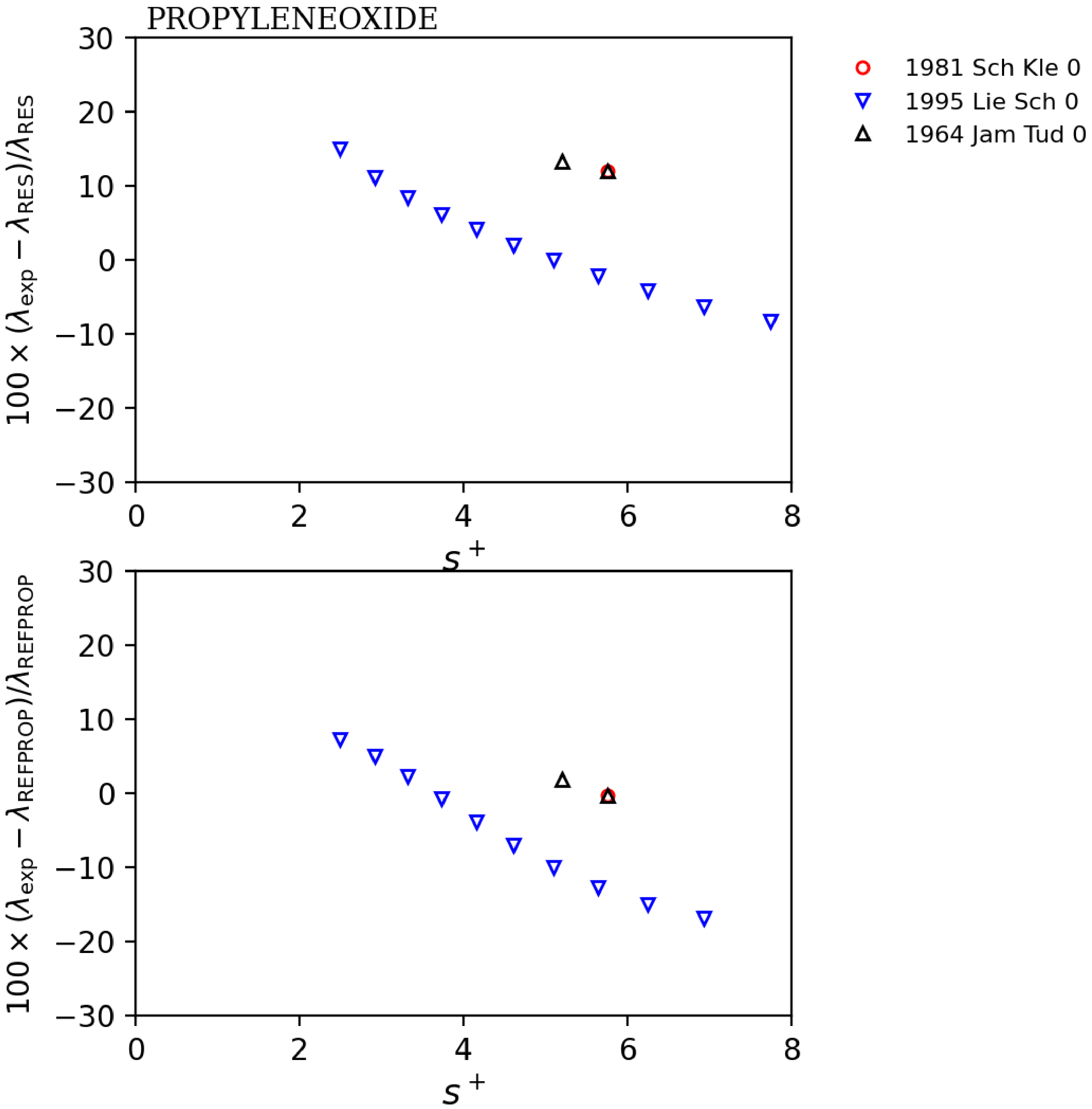

Figure DPR2. PROPYLENEOXIDE

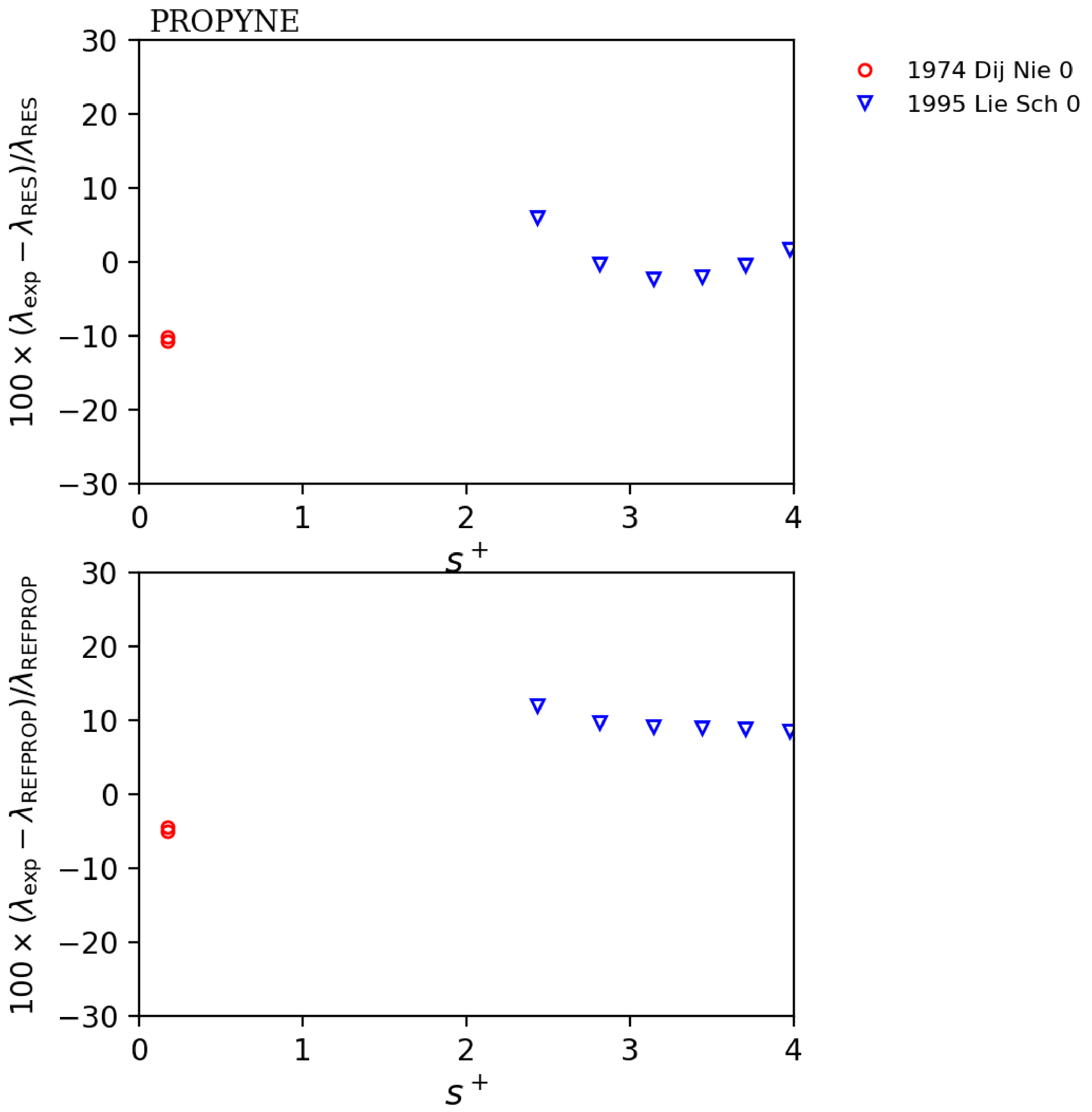

Figure DPR2. PROPYNE

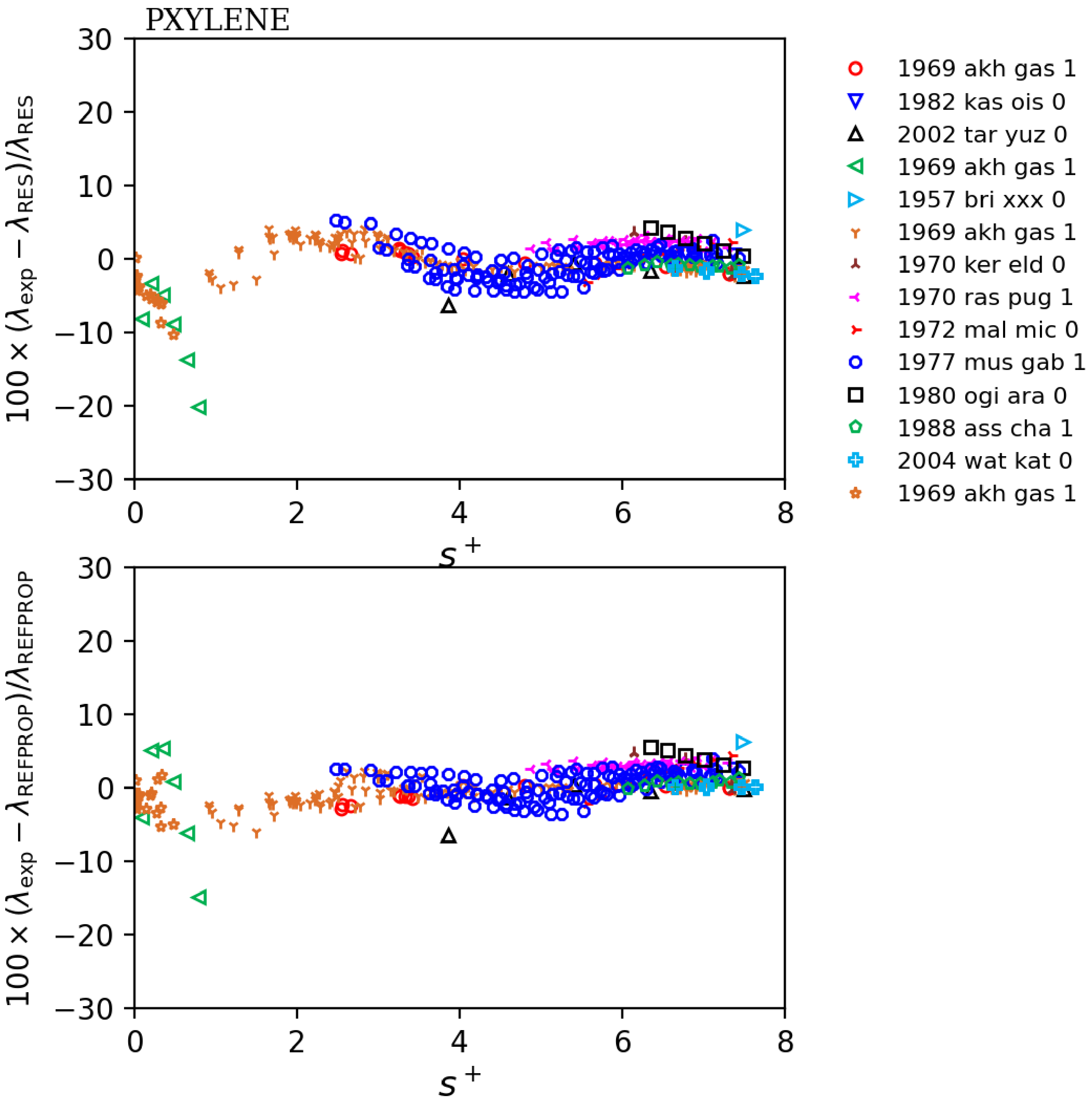

Figure DPR2. PXYLENE

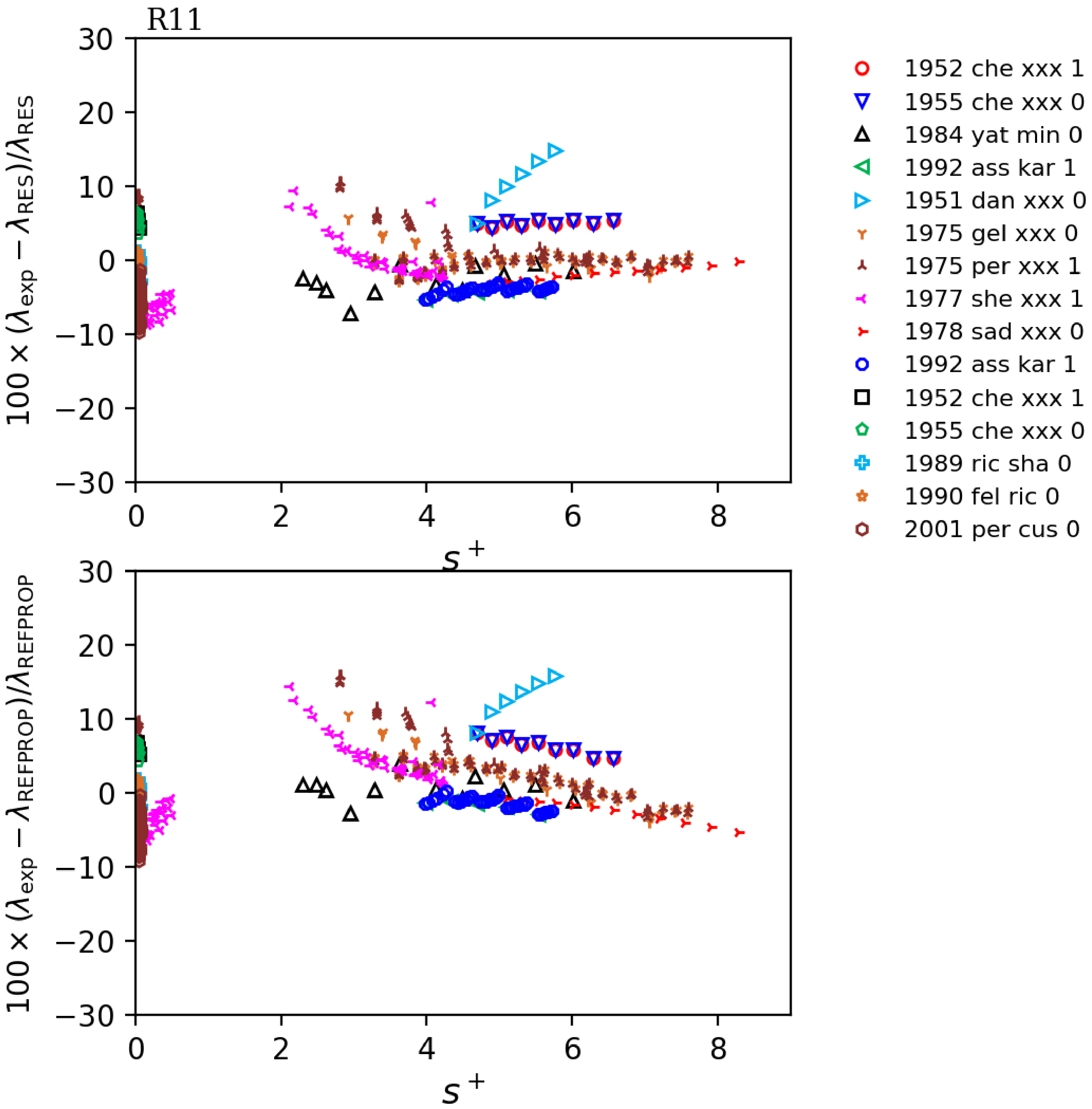

Figure DPR2. R11

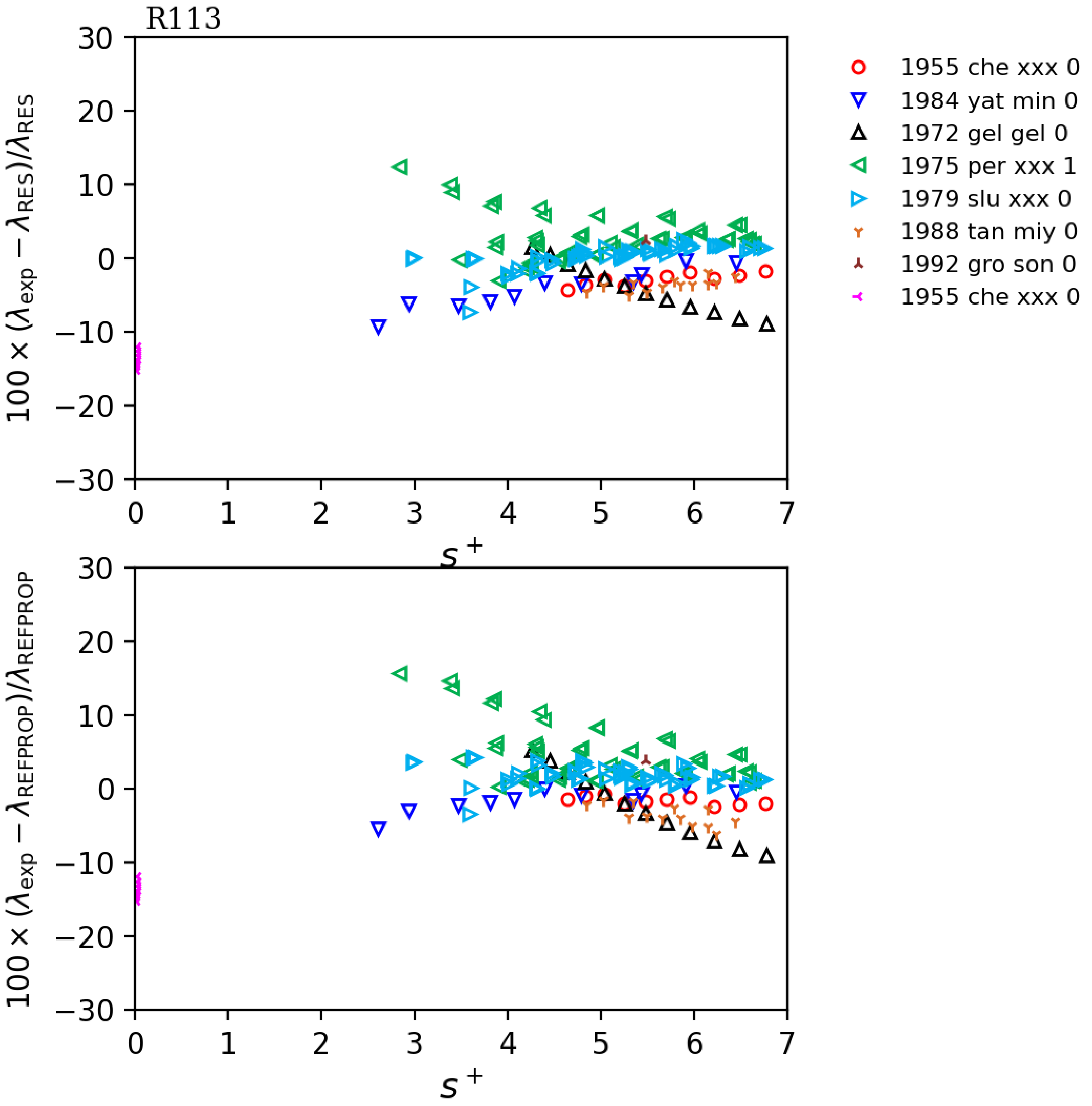

Figure DPR2. R113

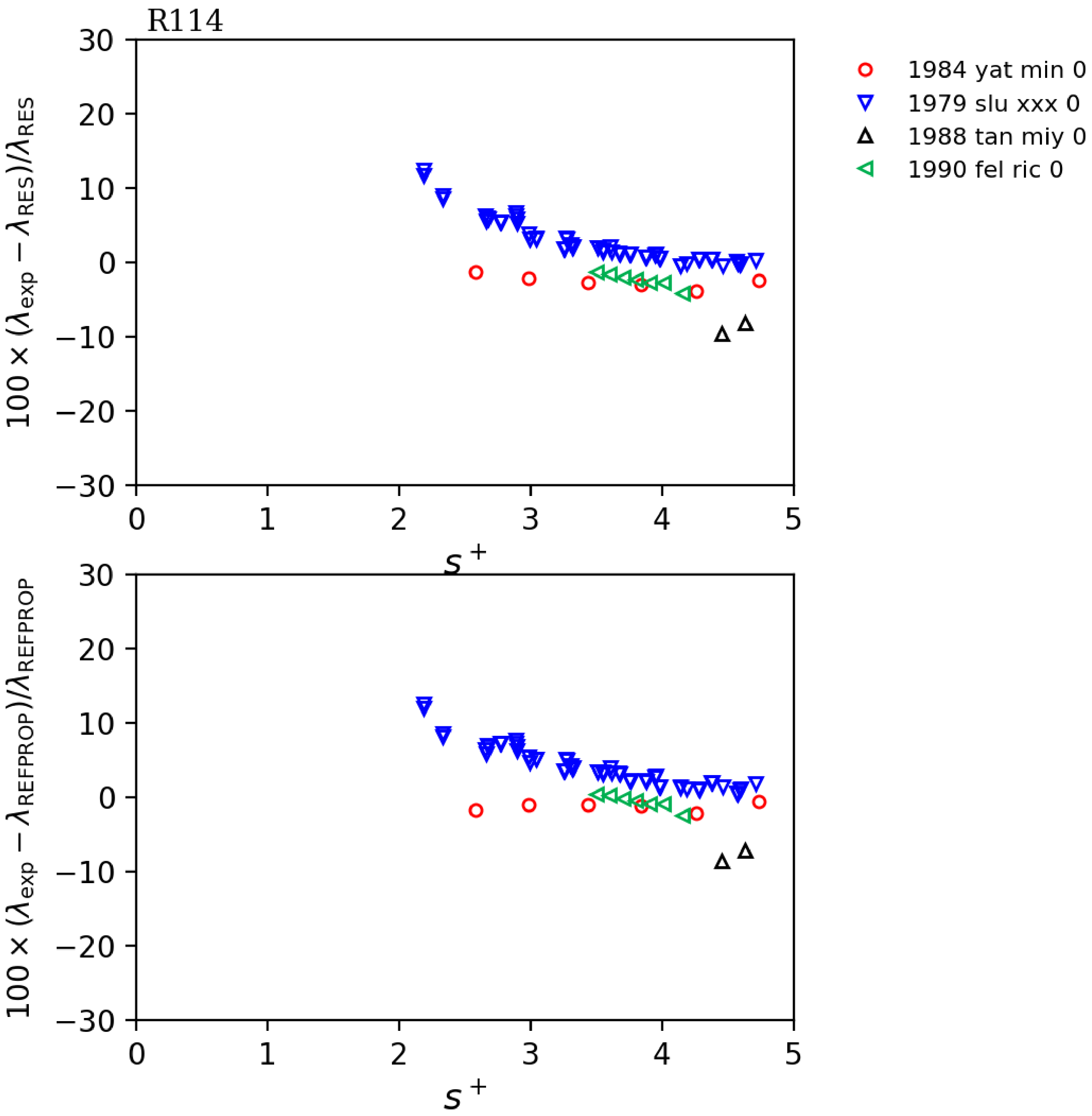

Figure DPR2. R114

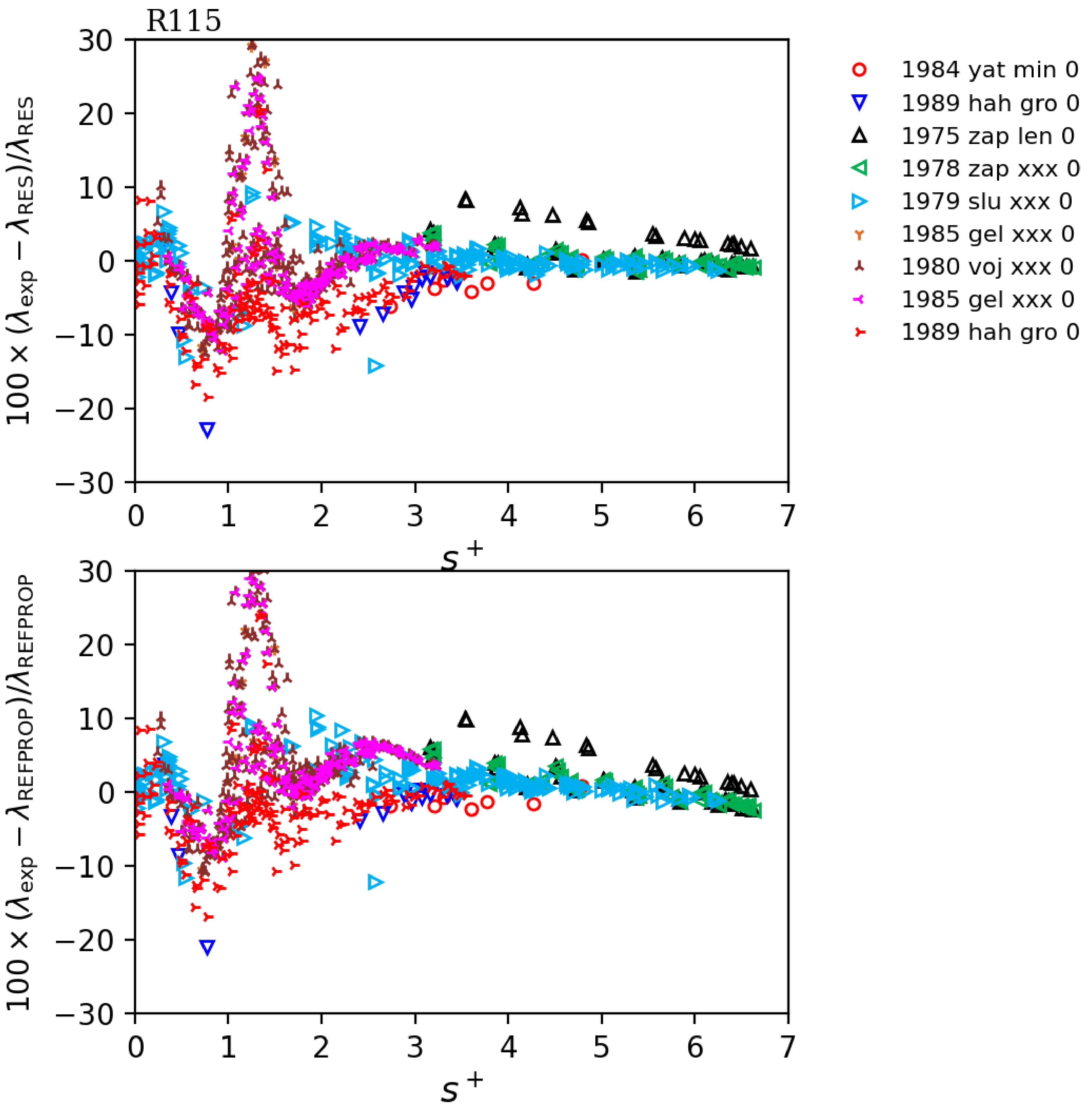

Figure DPR2. R115

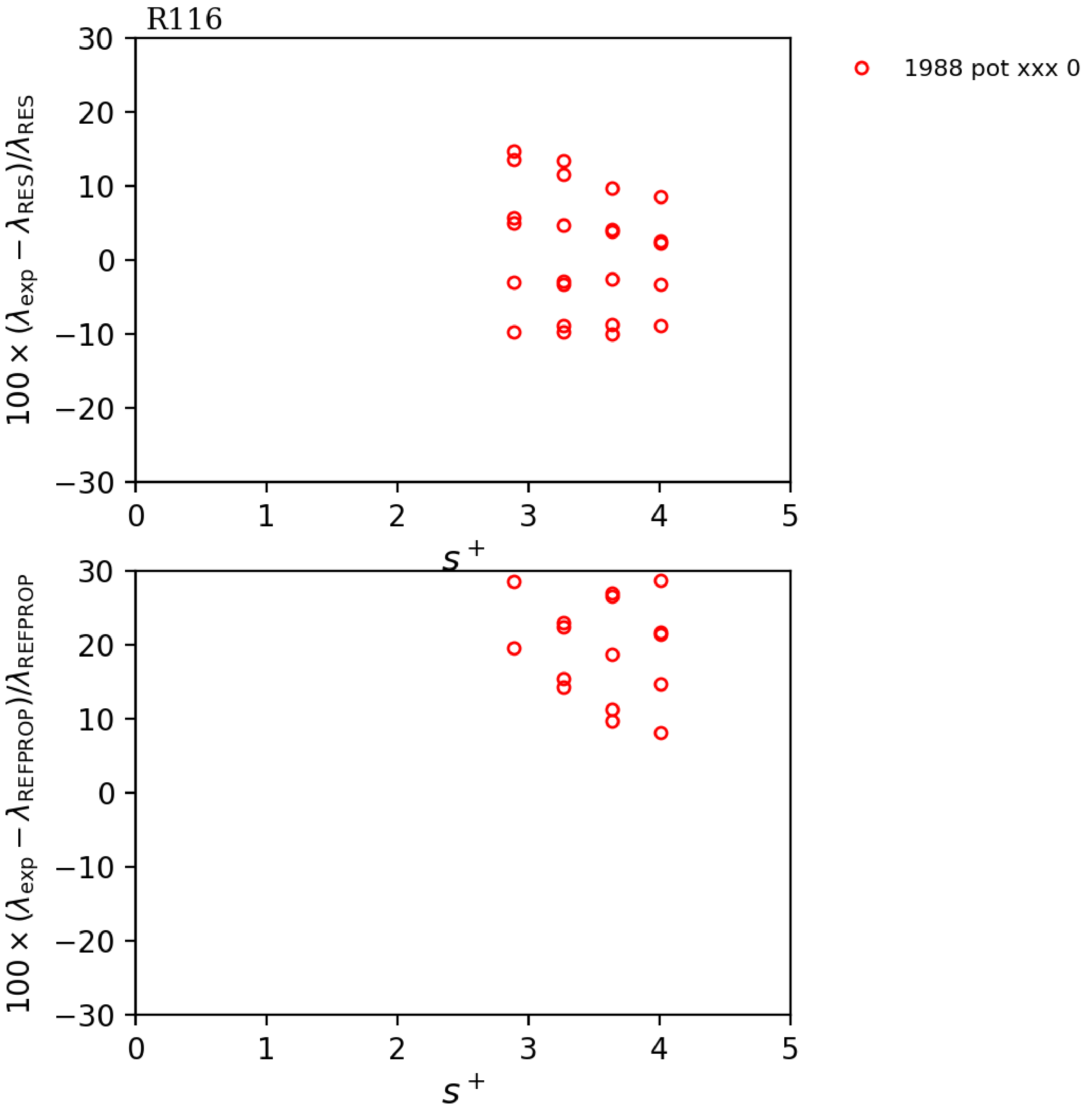

Figure DPR2. R116

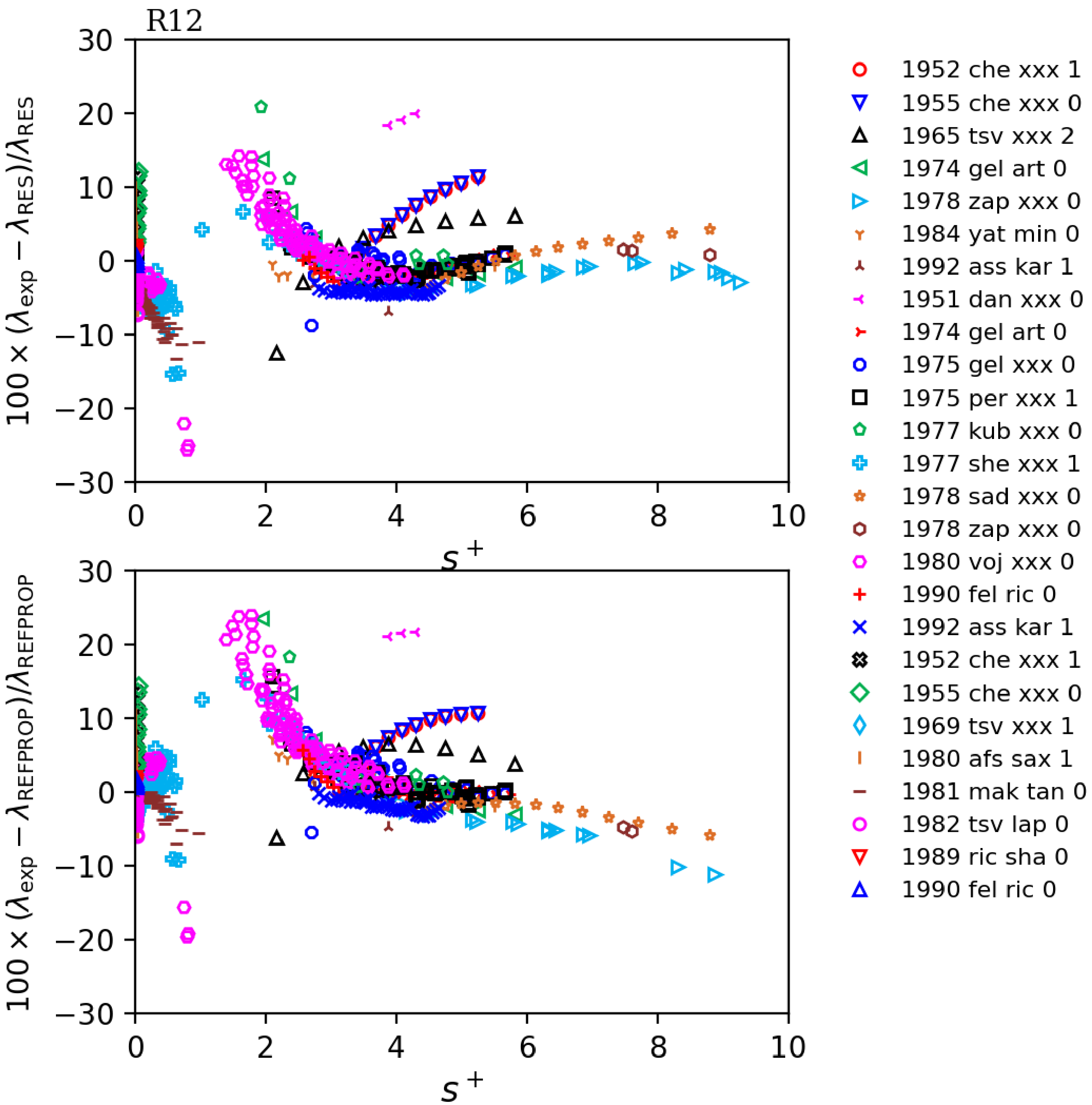

Figure DPR2. R12

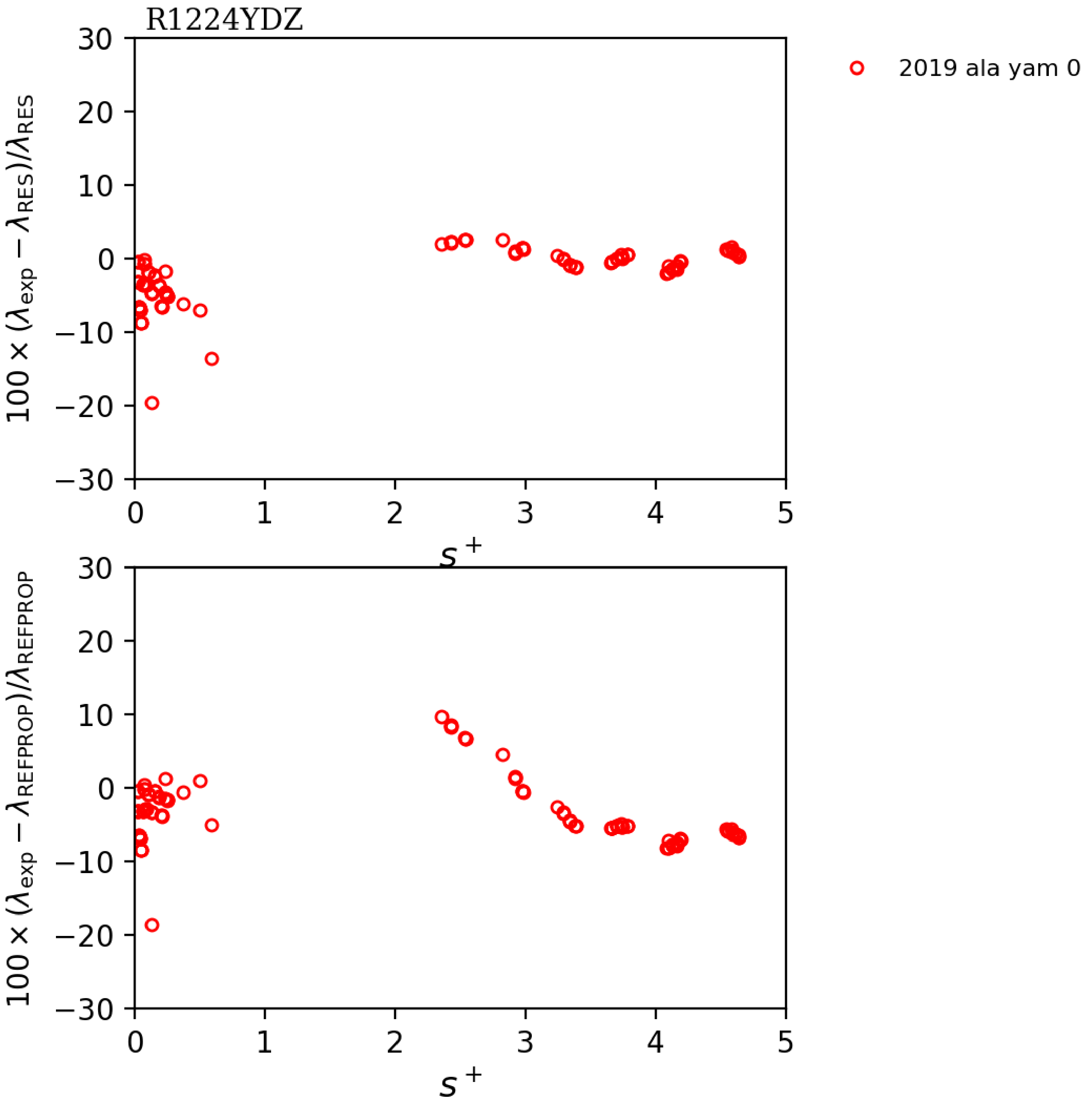

Figure DPR2. R1224YDZ

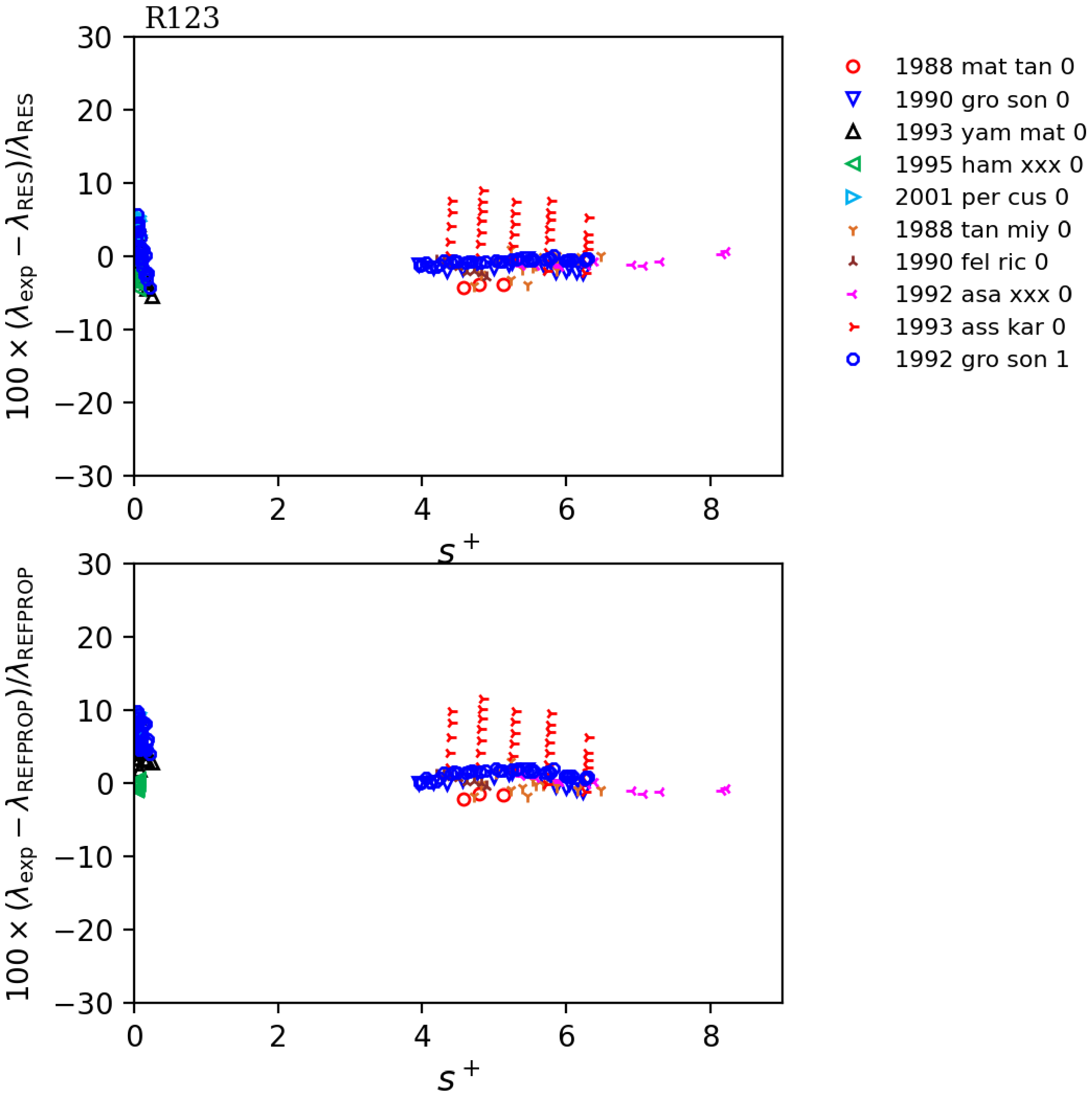

Figure DPR2. R123

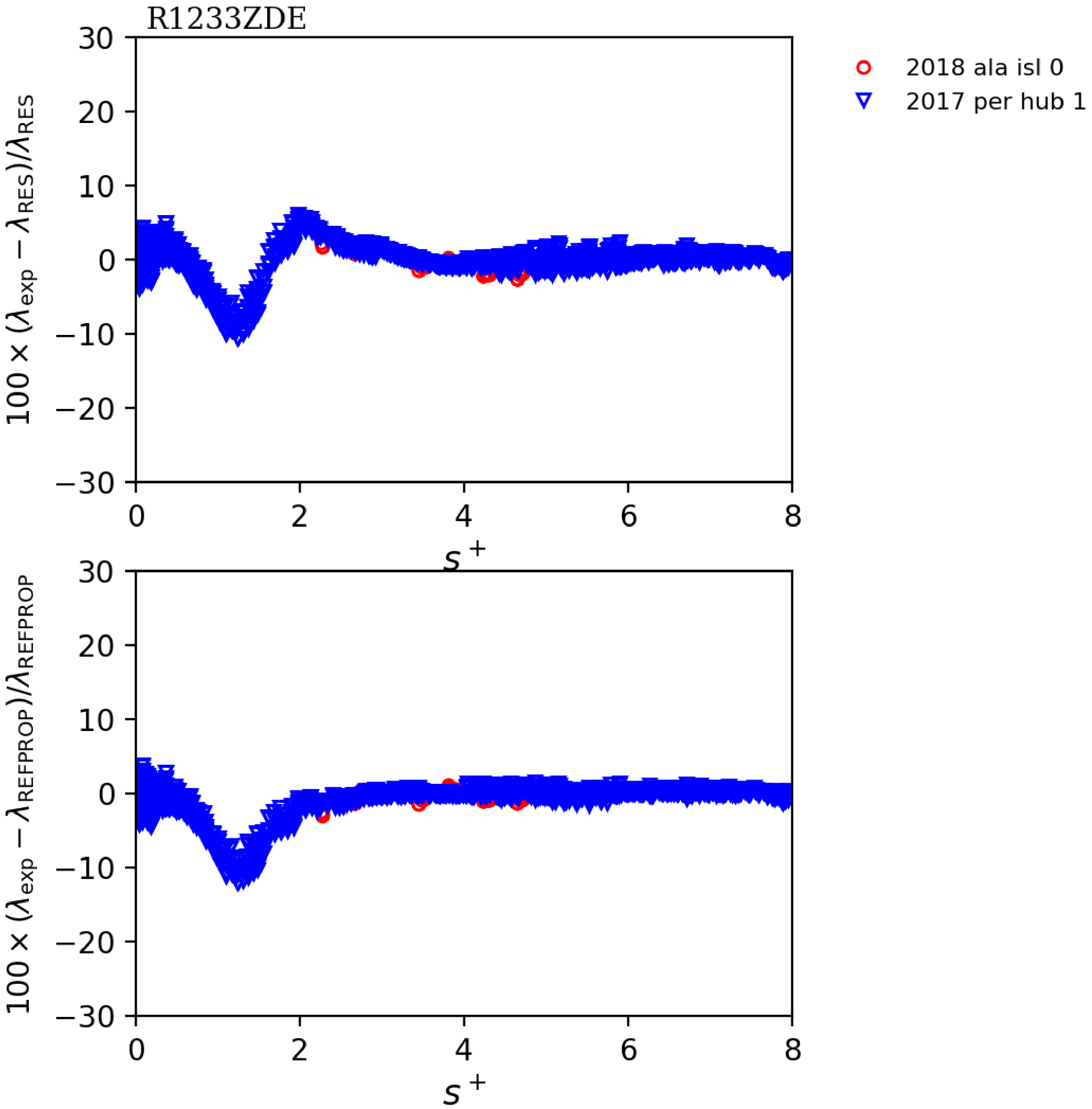

Figure DPR2. R1233ZDE

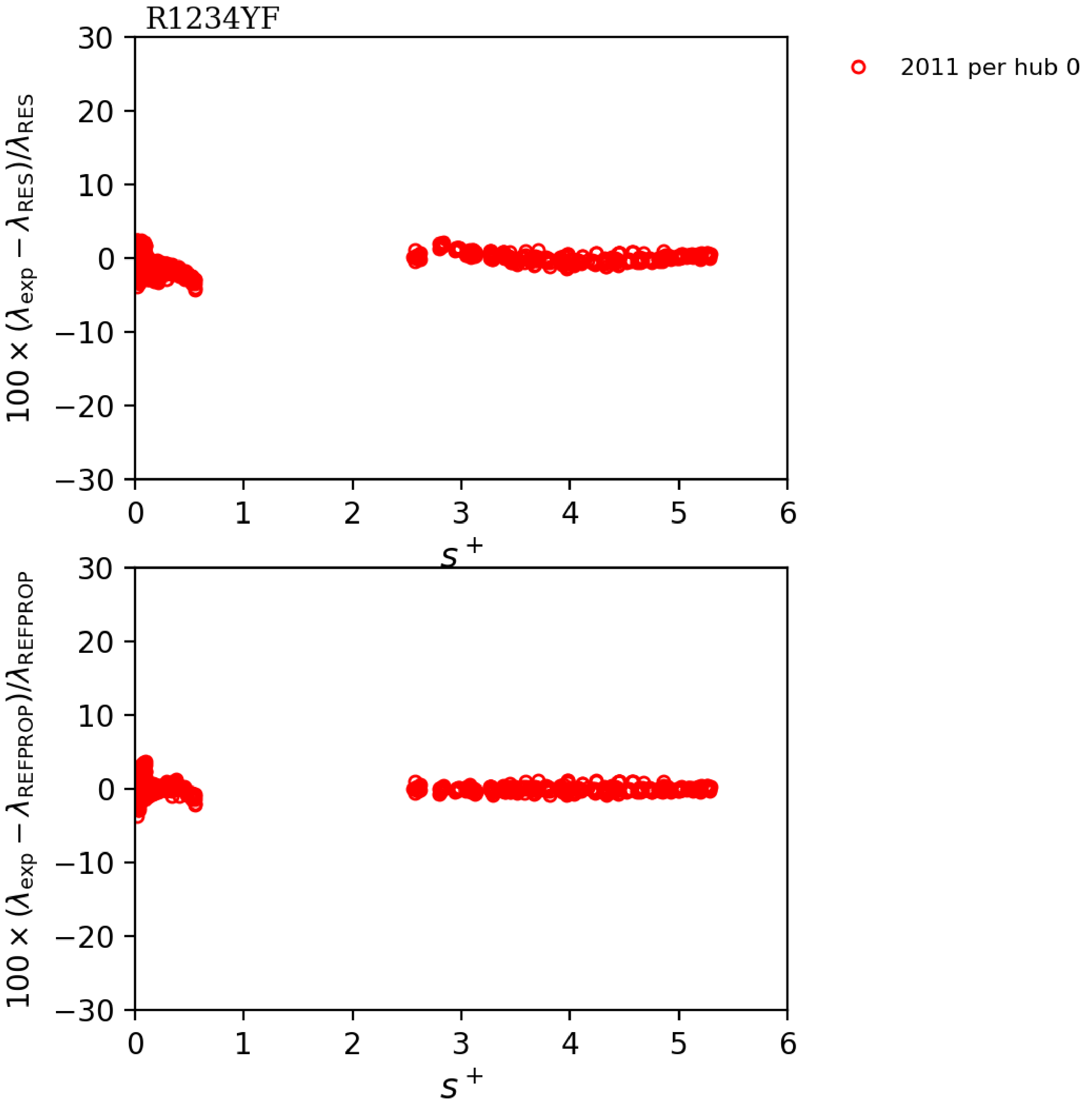

Figure DPR2. R1234YF

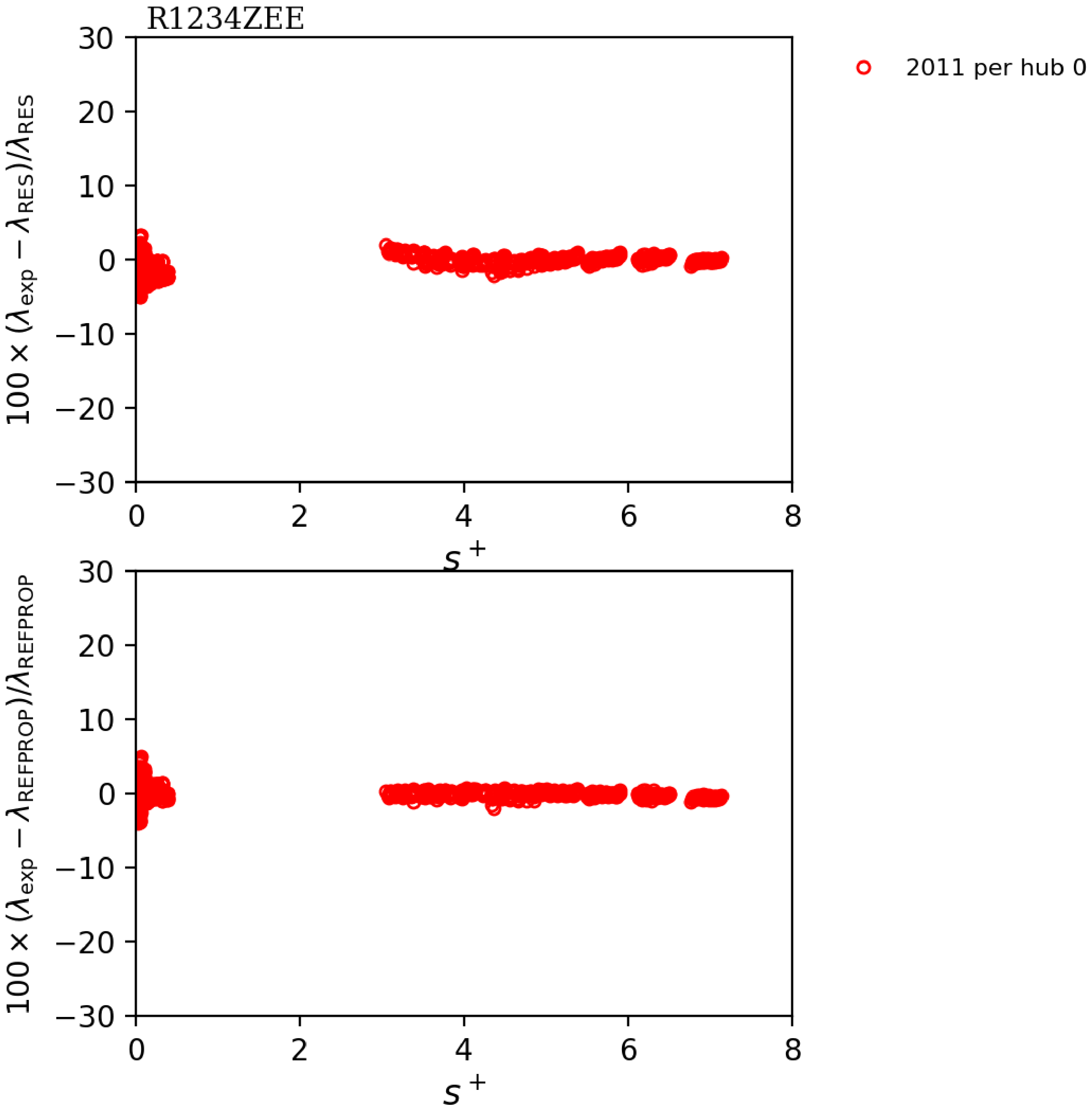

Figure DPR2. R1234ZEE

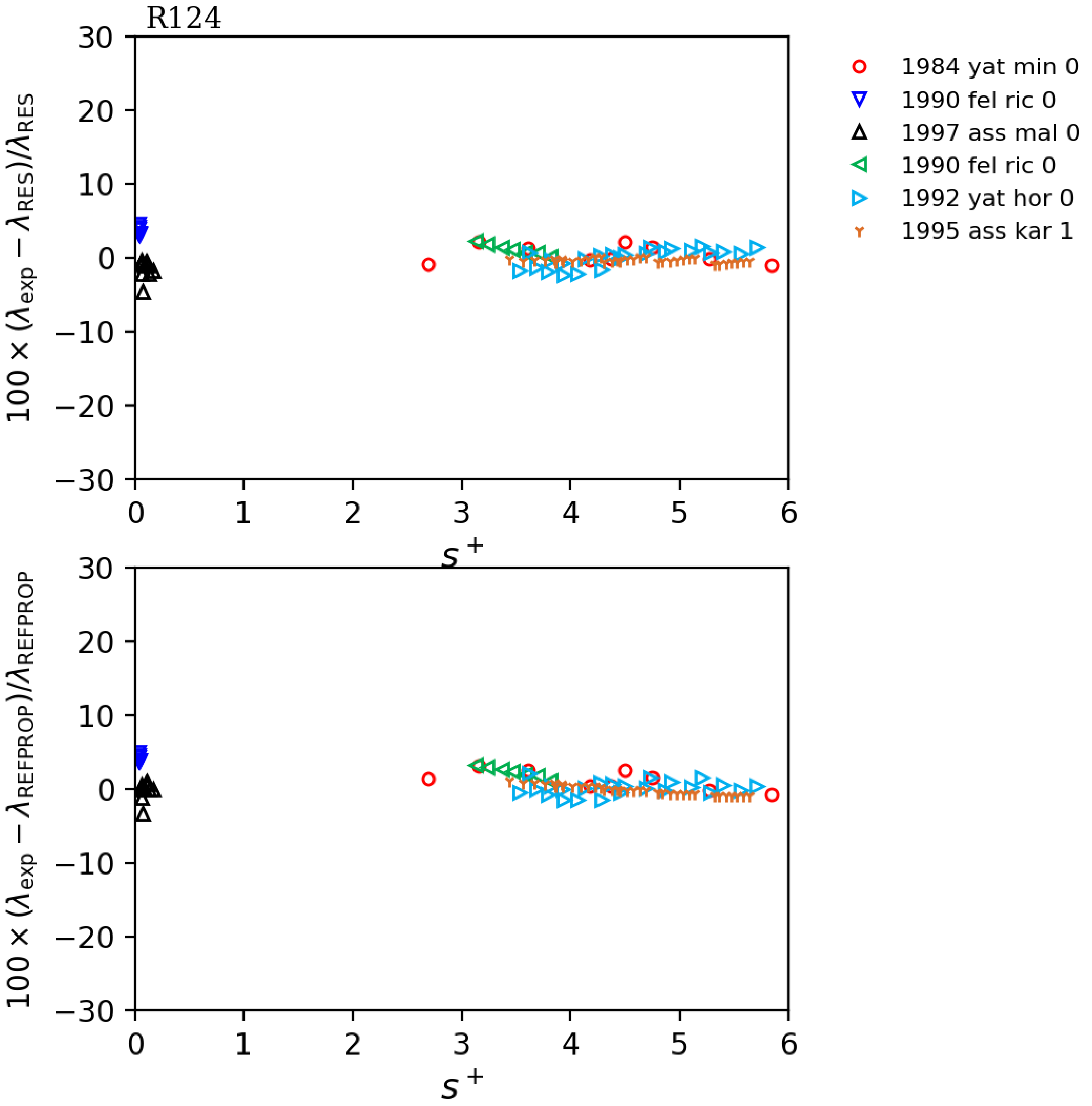

Figure DPR2. R124

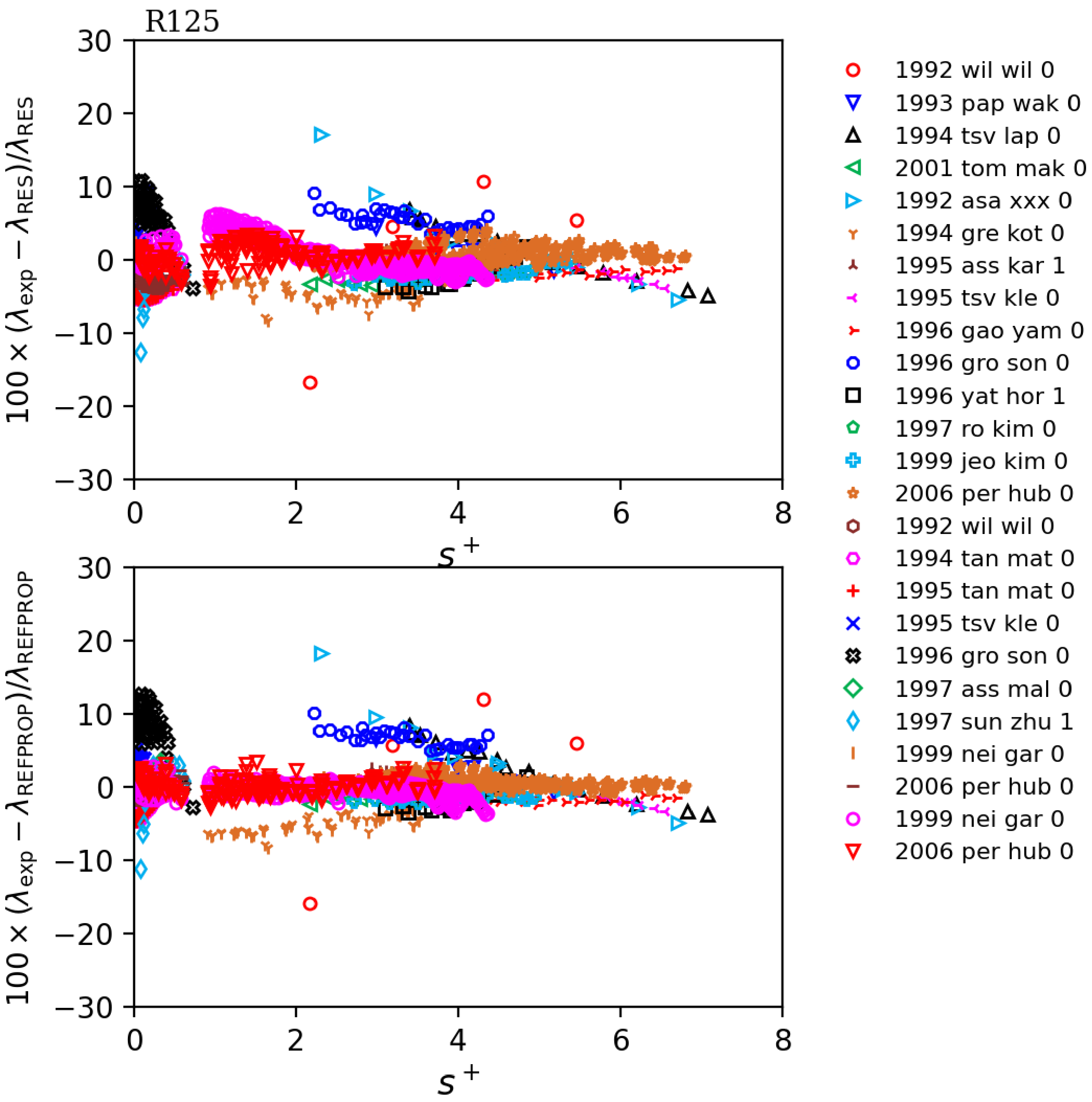

Figure DPR2. R125

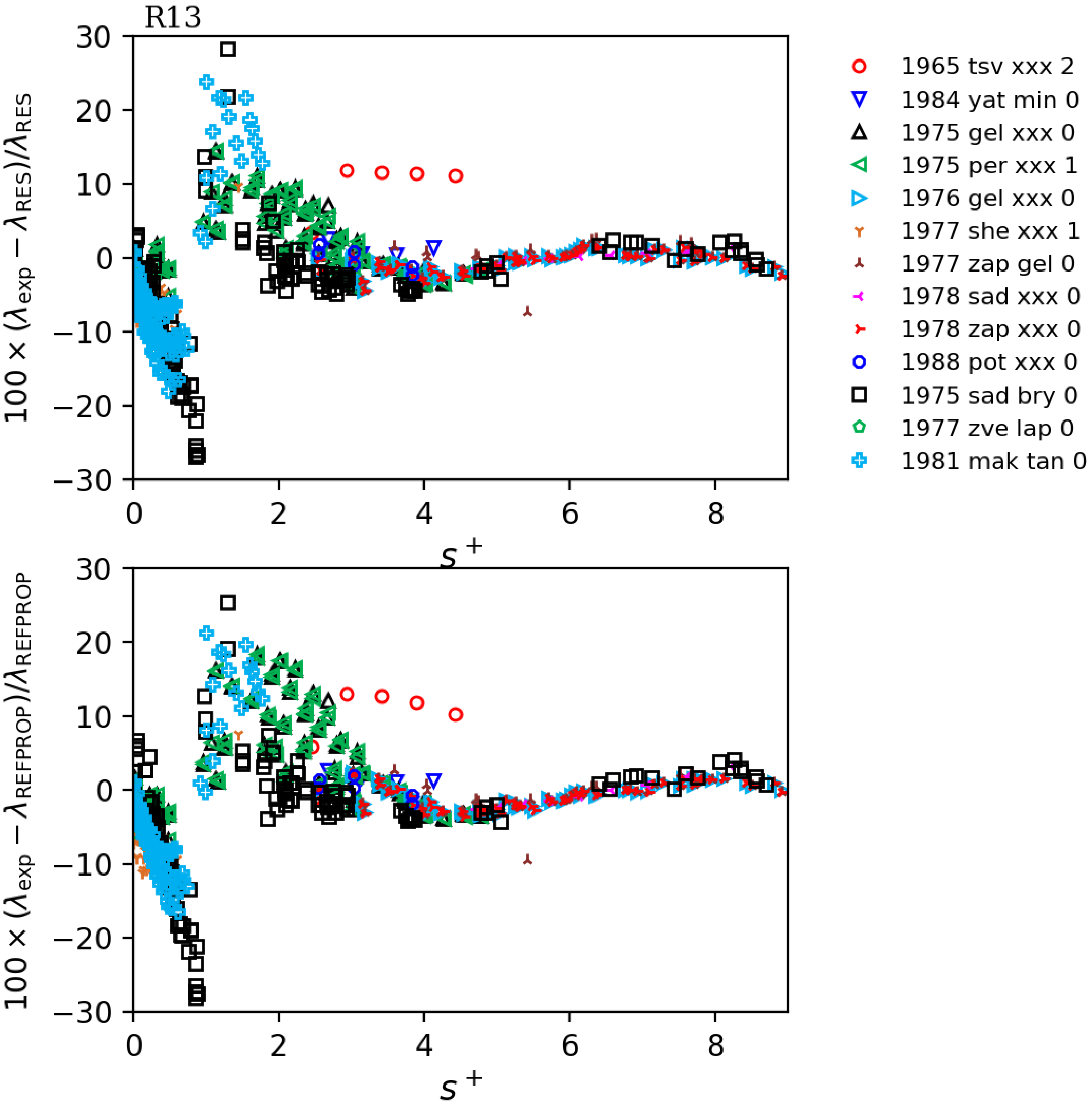

Figure DPR2. R13

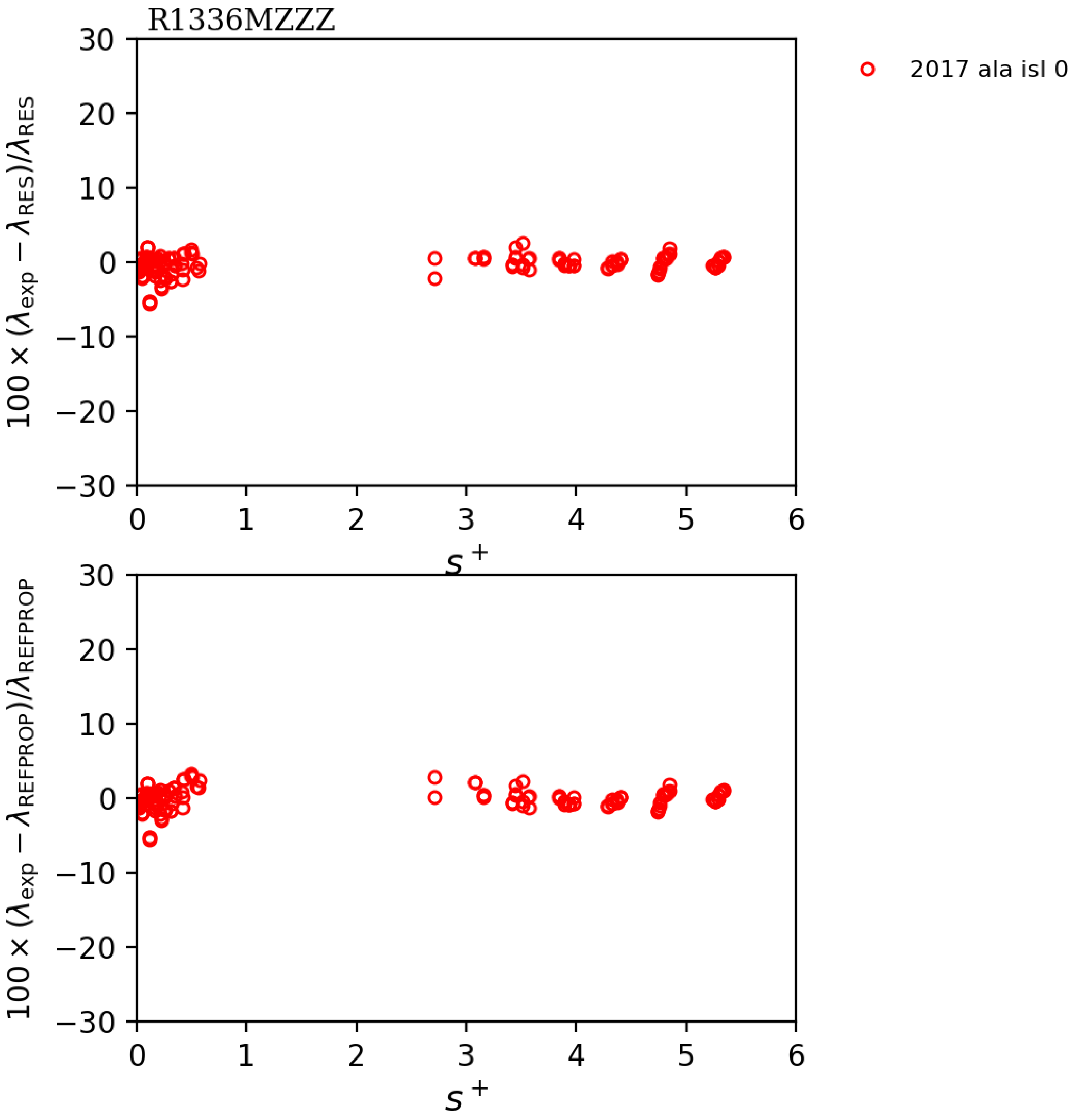

Figure DPR2. R1336MZZZ

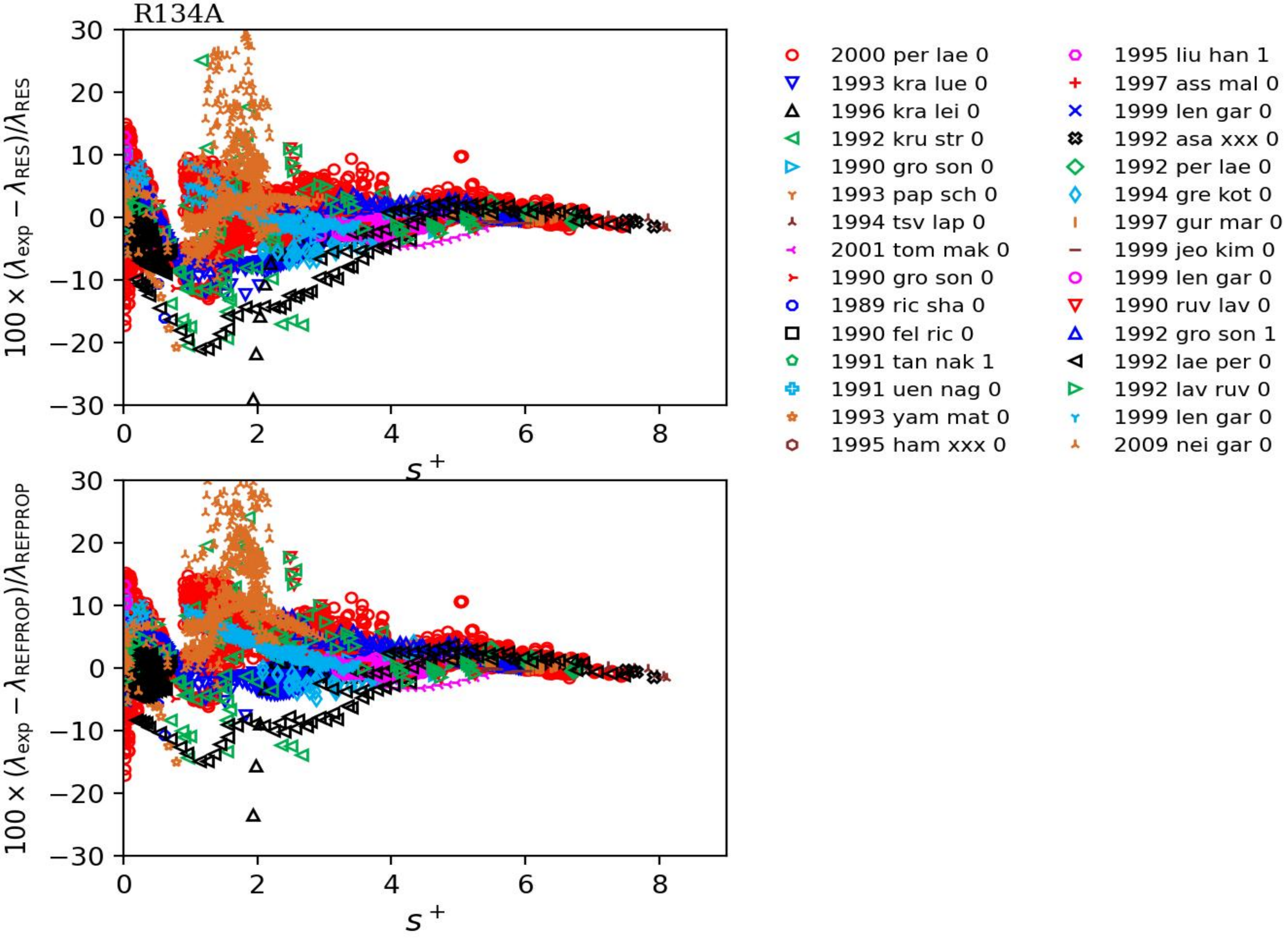

Figure DPR2. R134A

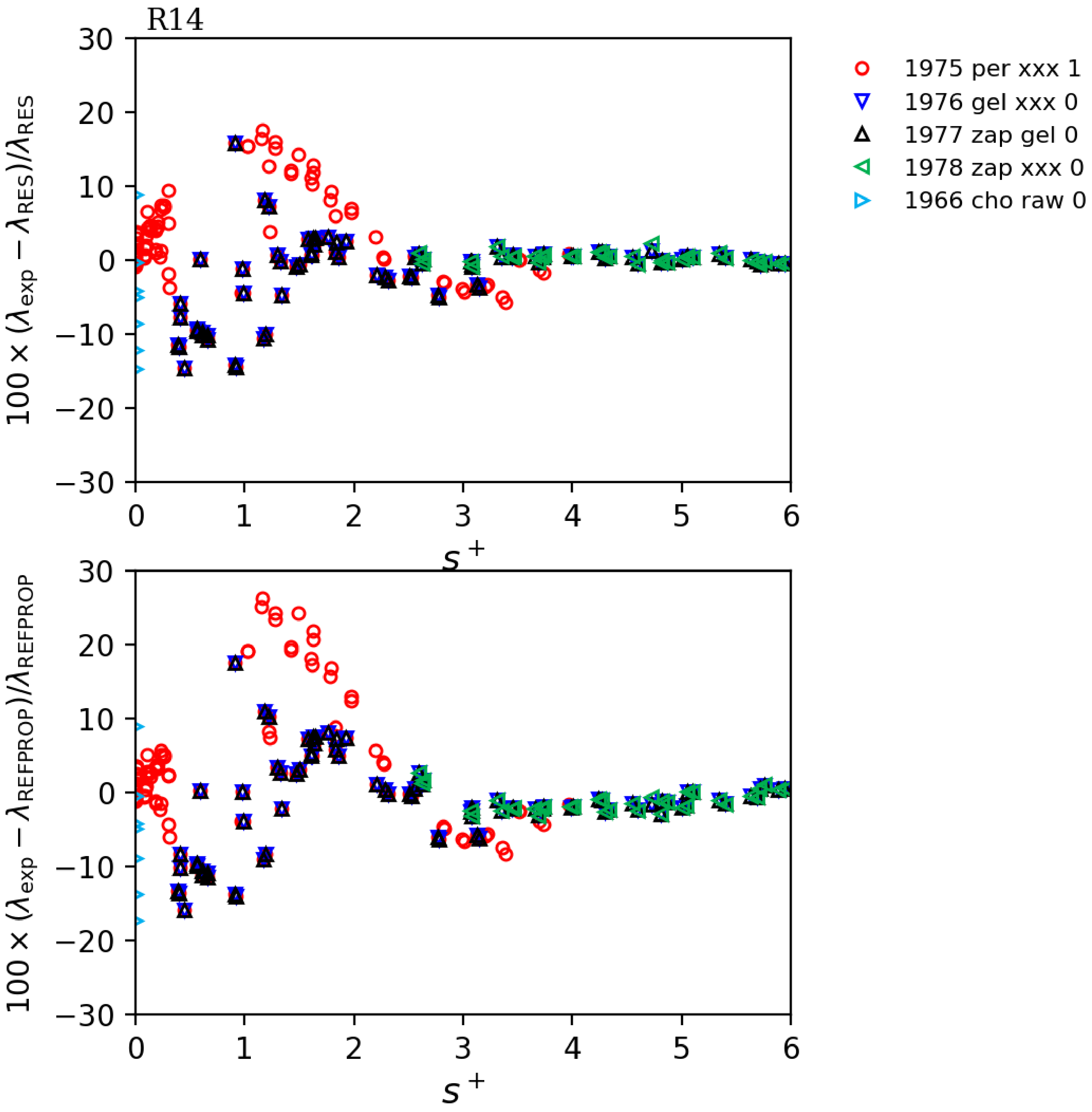

Figure DPR2. R14

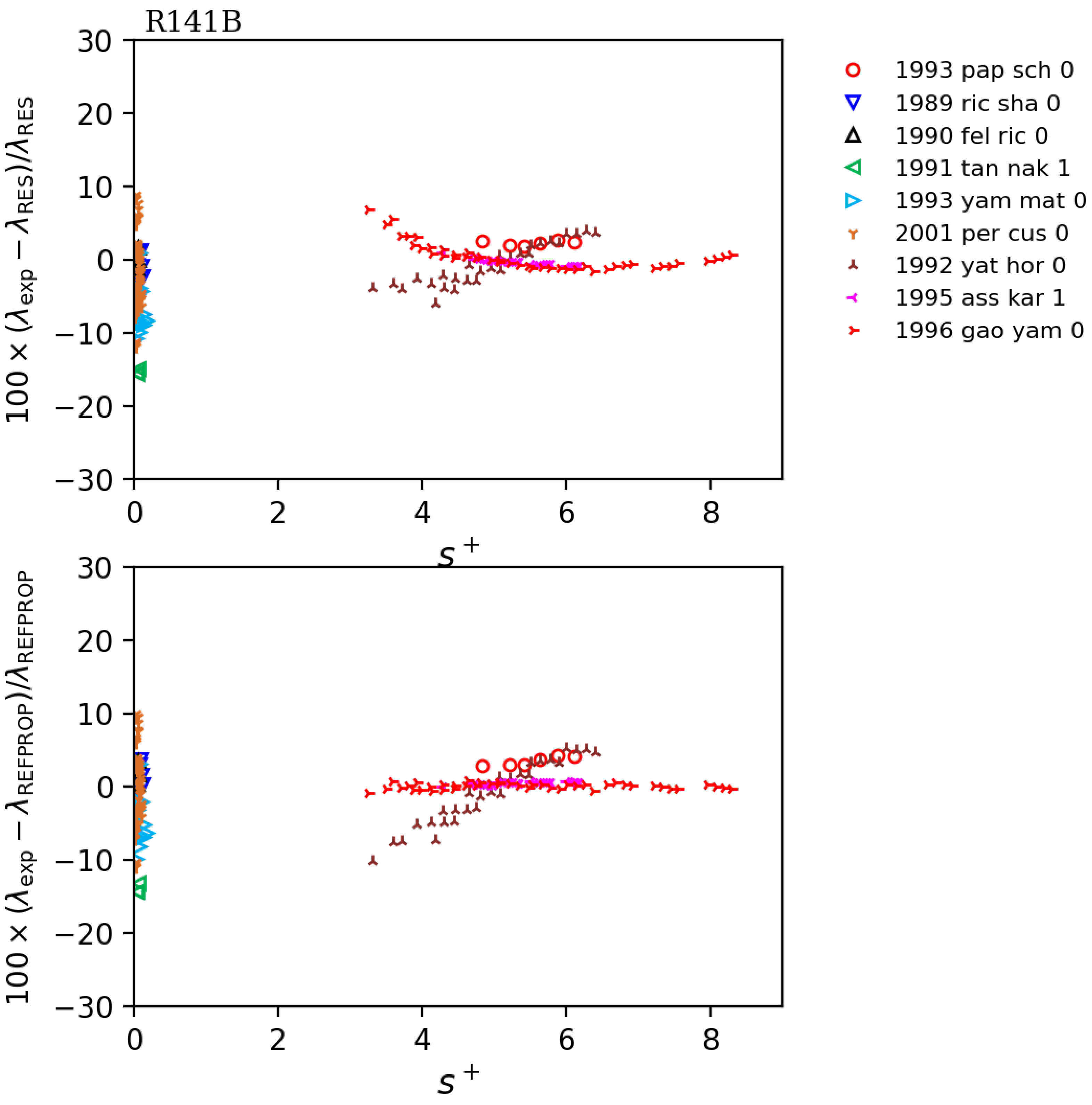

Figure DPR2. R141B

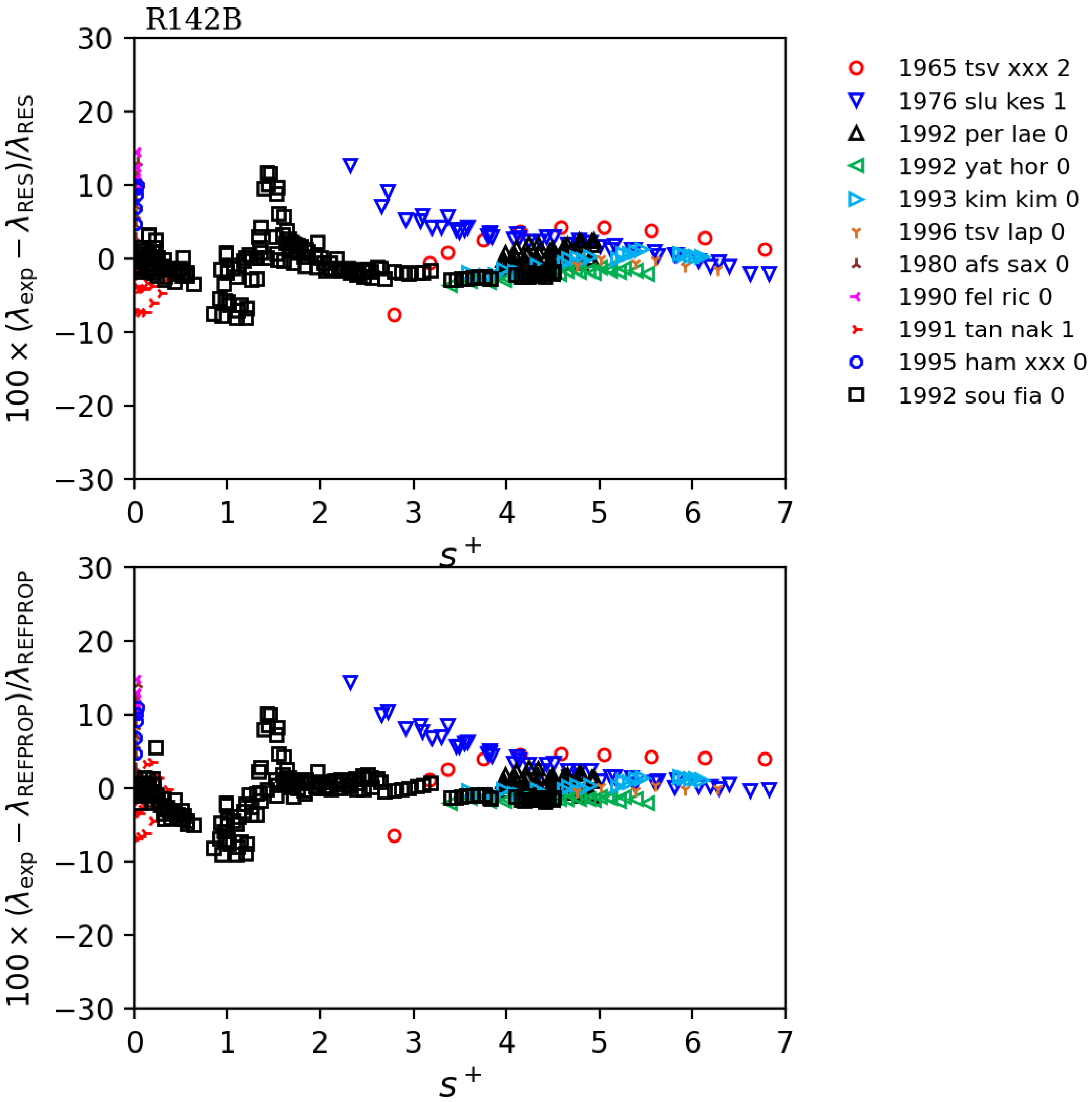

Figure DPR2. R142B

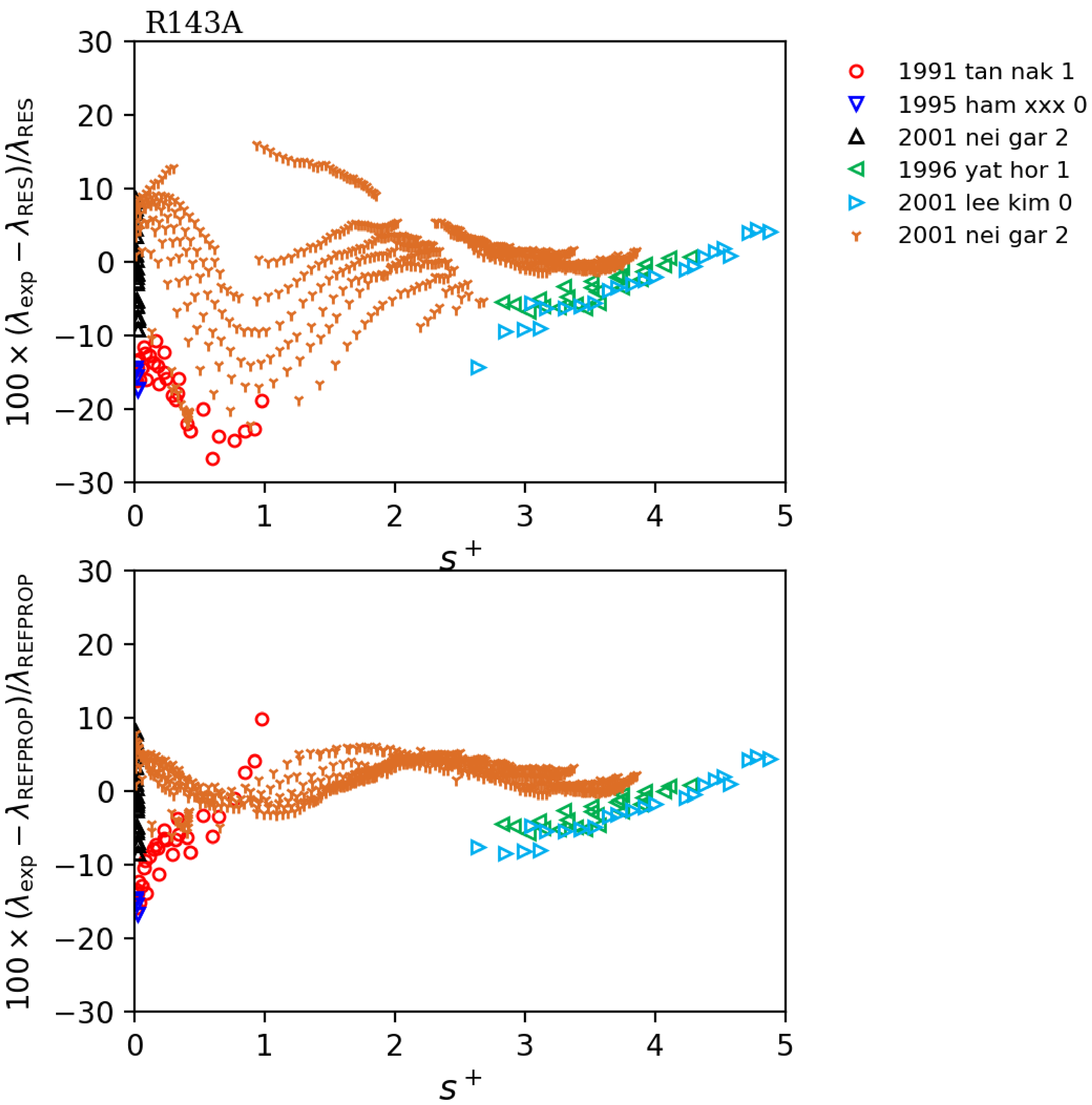

Figure DPR2. R143A

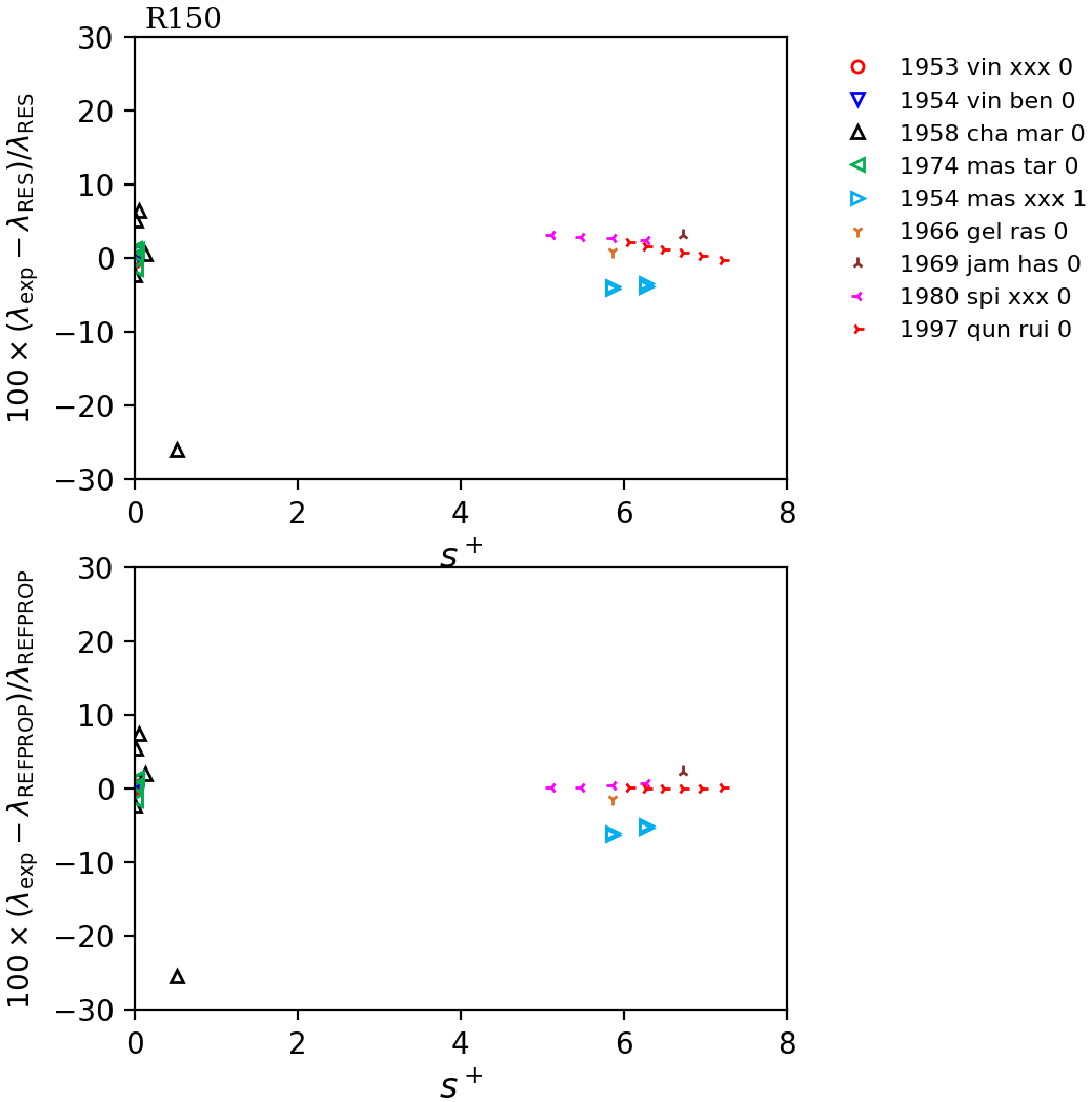

Figure DPR2. R150

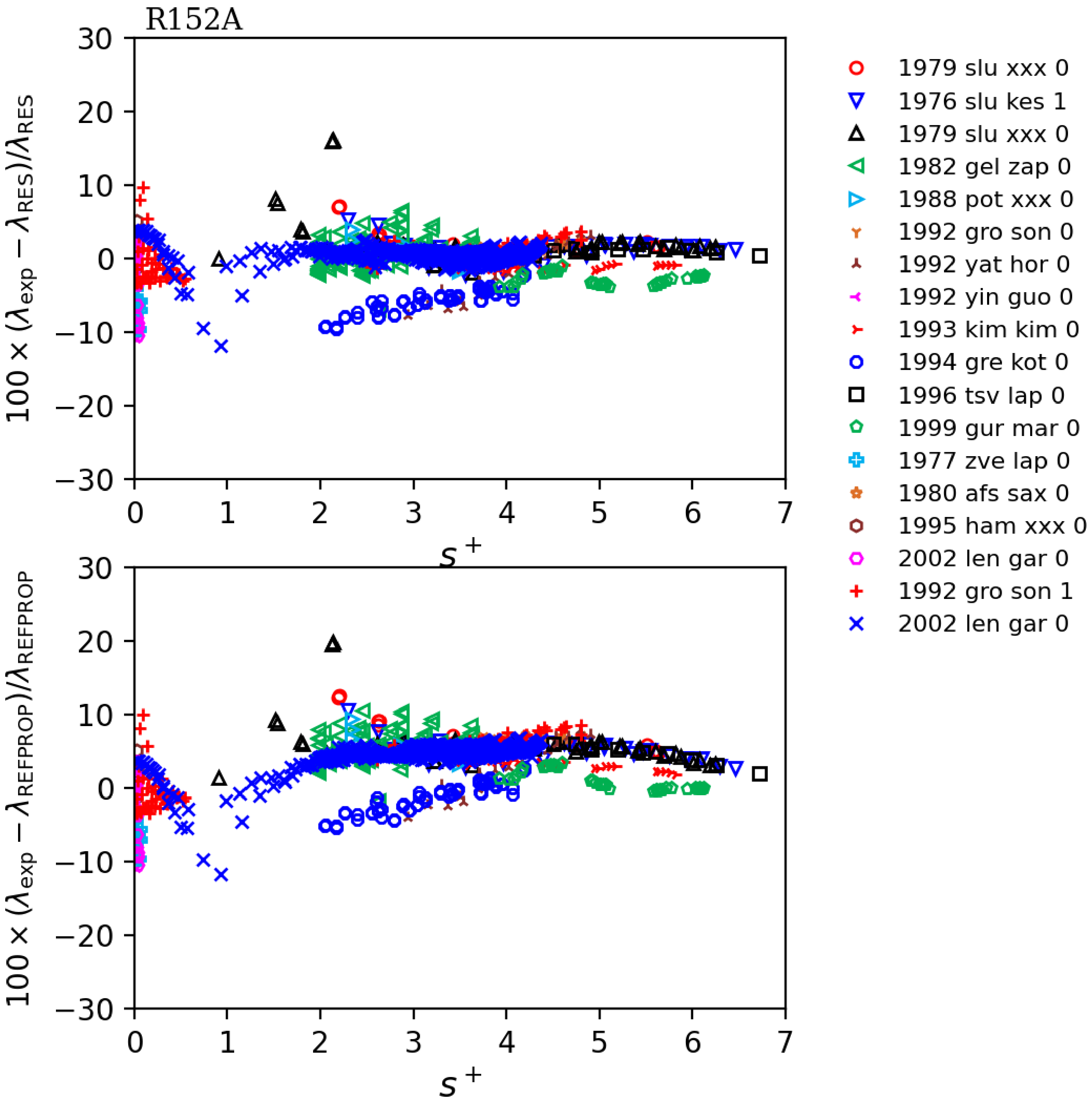

Figure DPR2. R152A

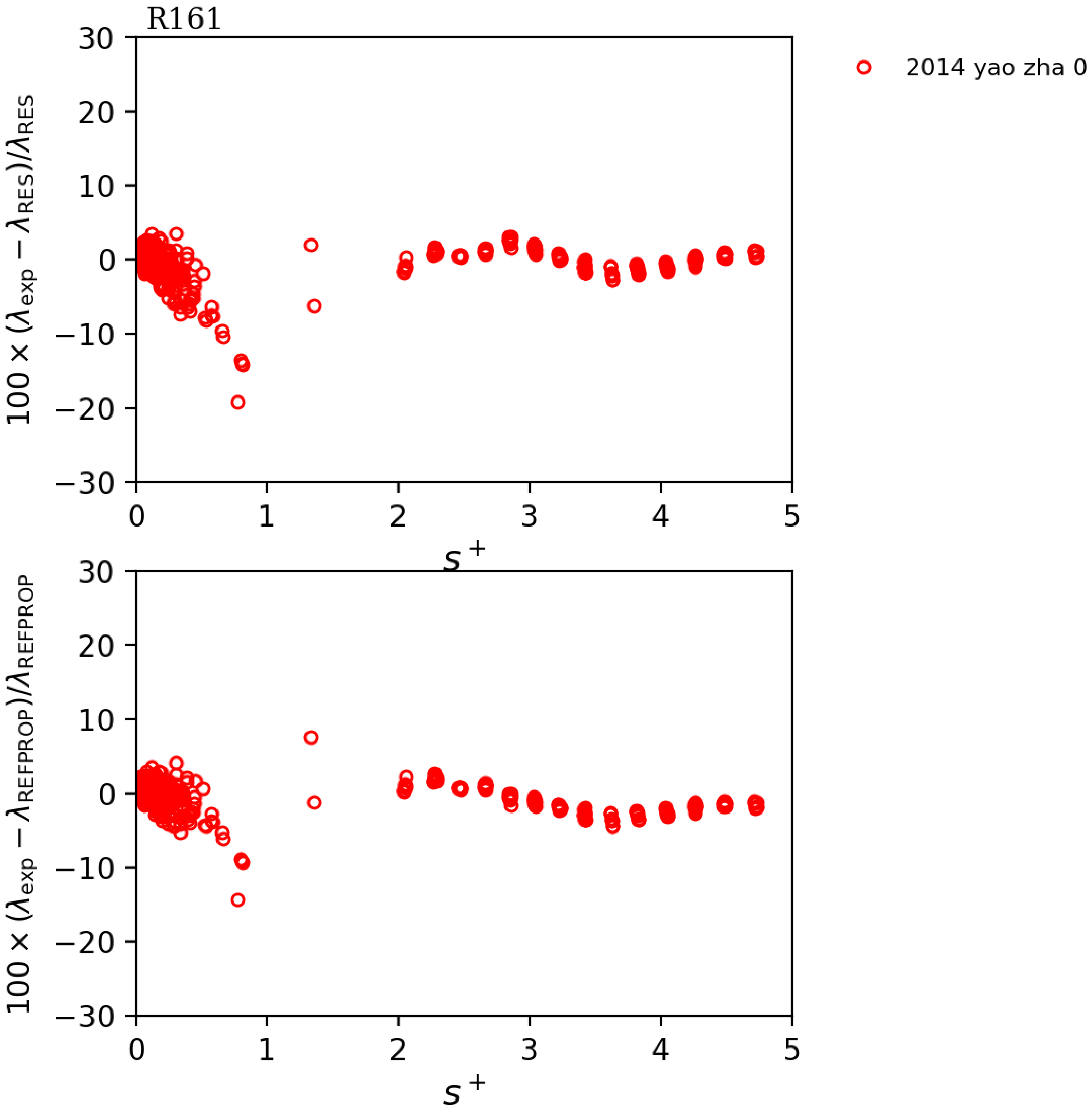

Figure DPR2. R161

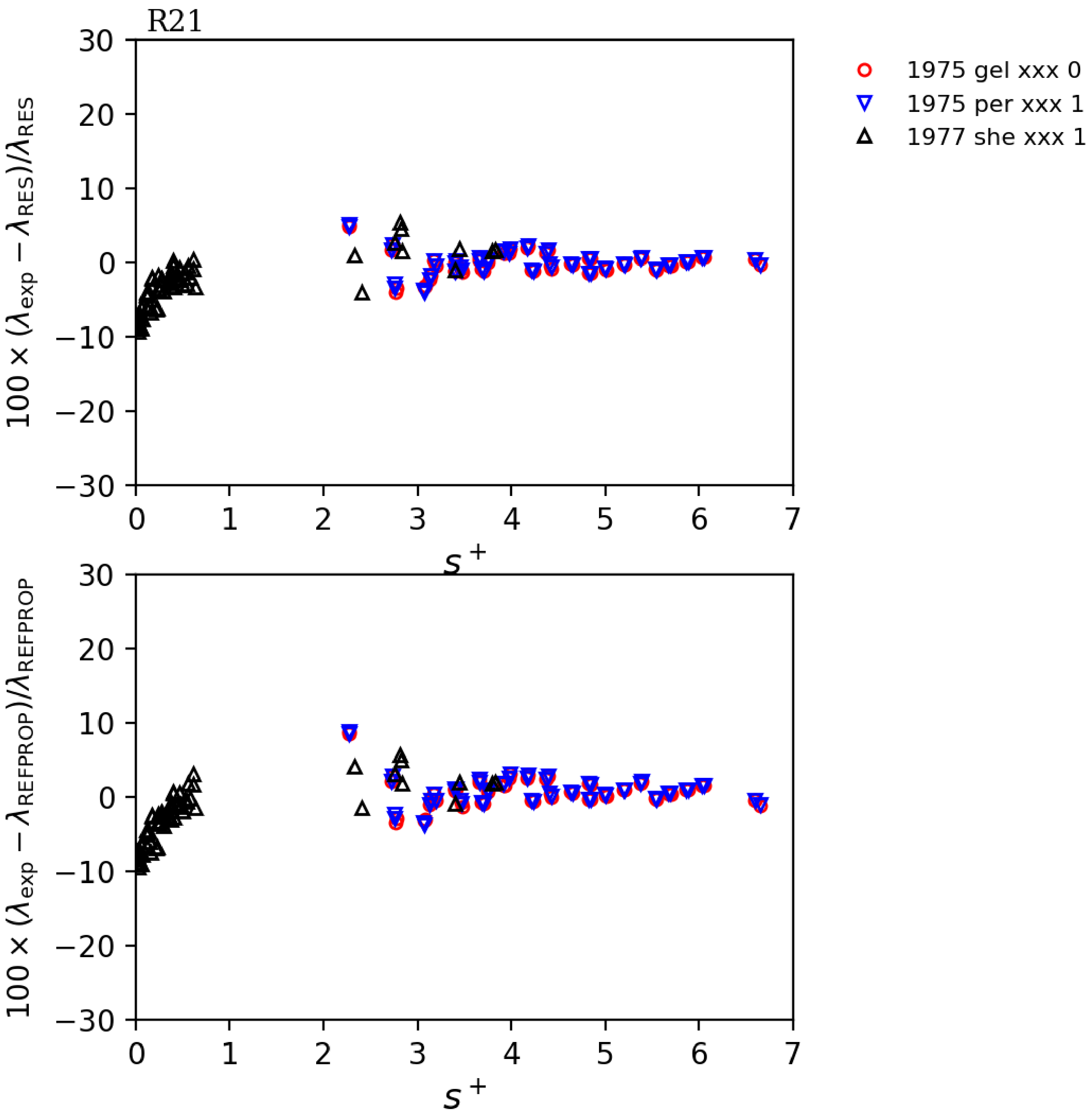

Figure DPR2. R21

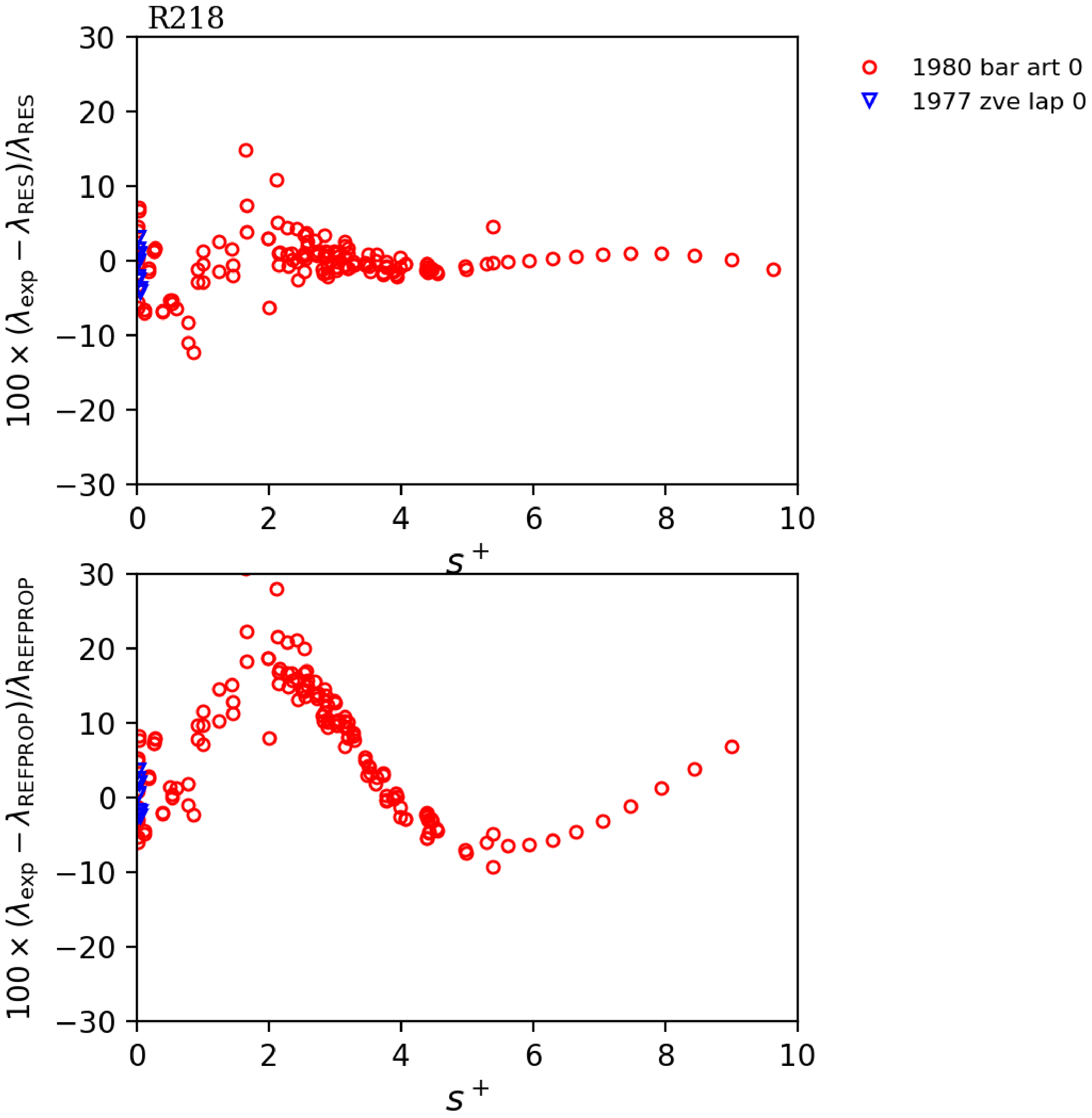

Figure DPR2. R218

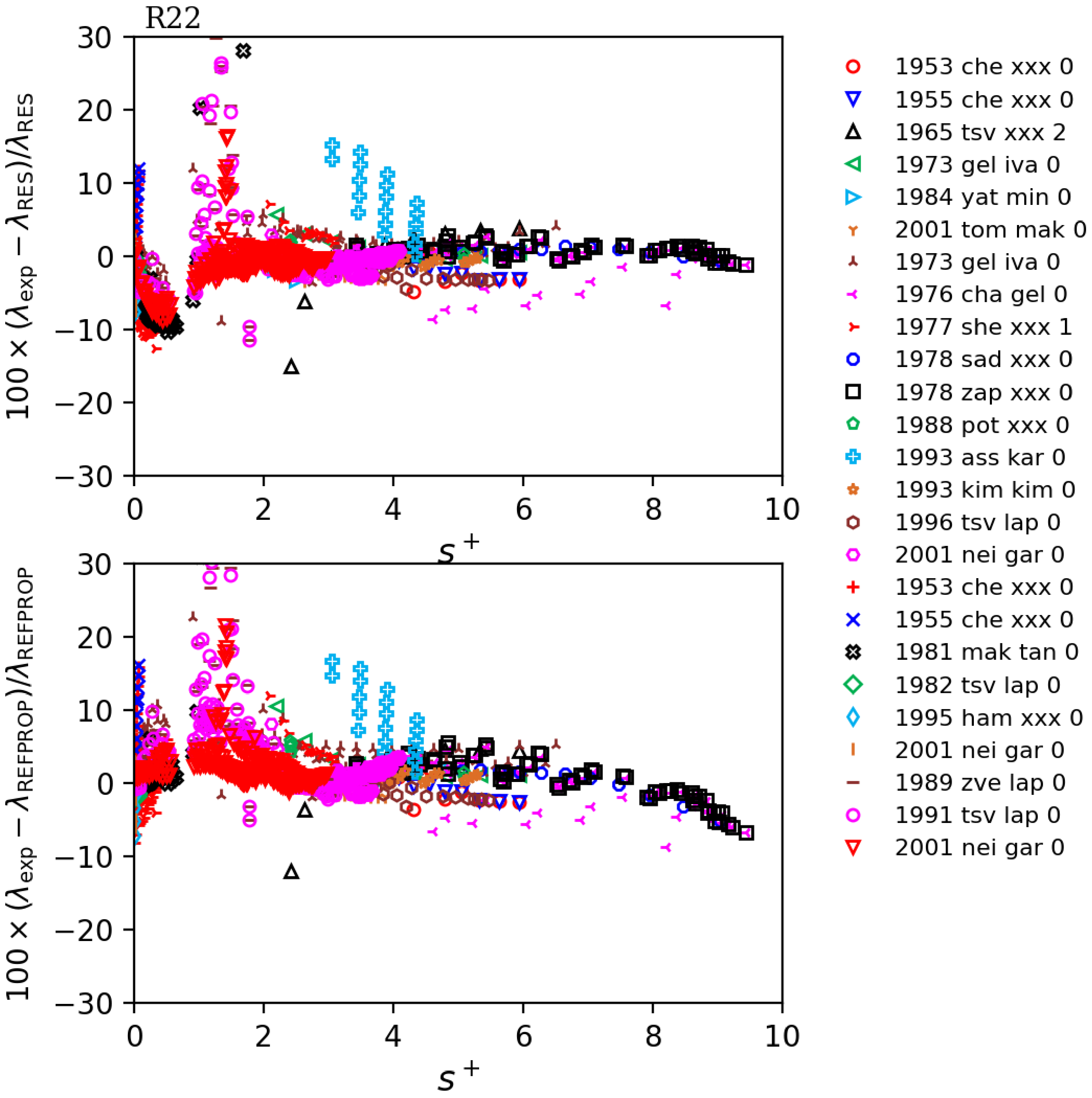

Figure DPR2. R22

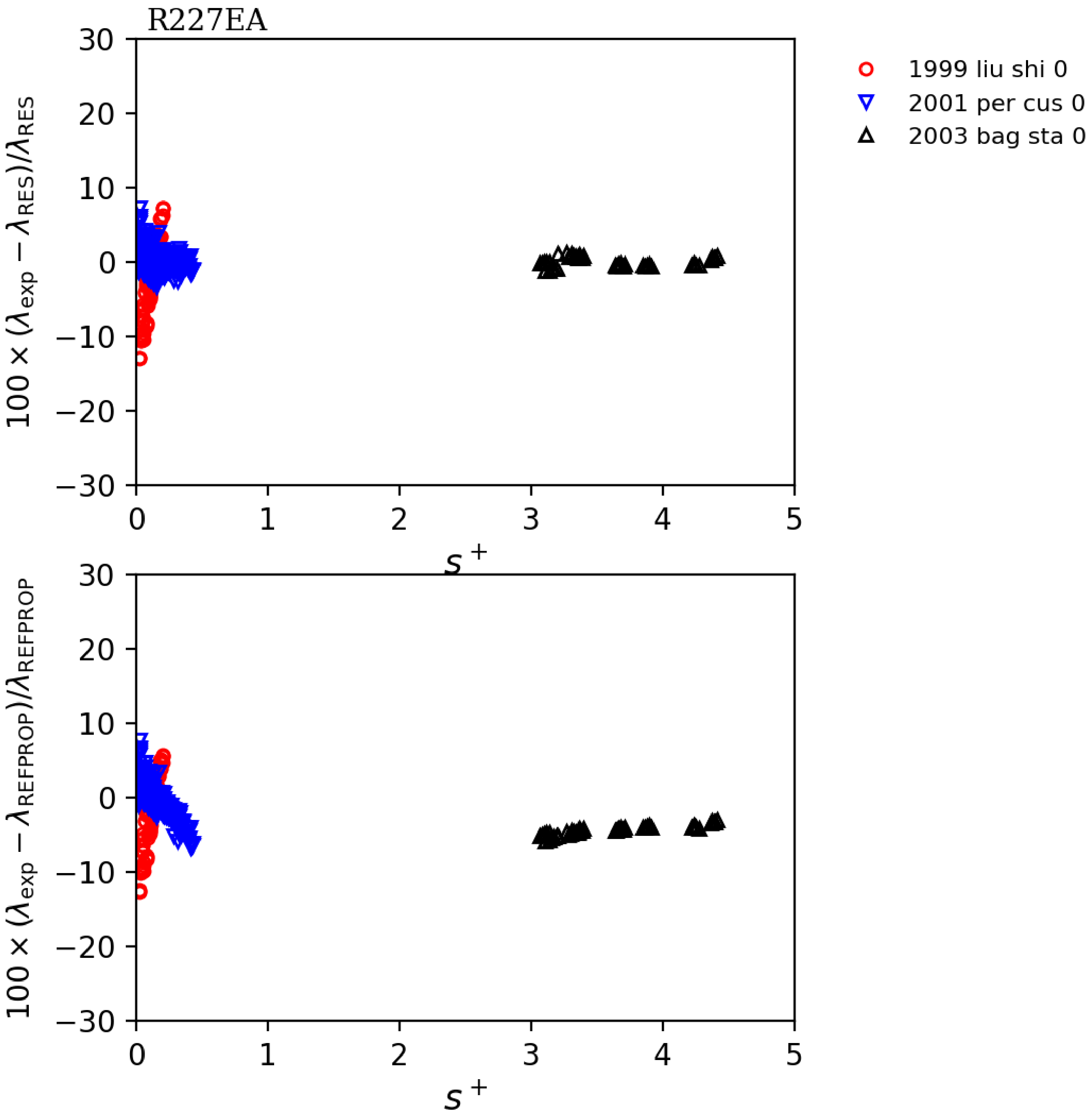

Figure DPR2. R227EA

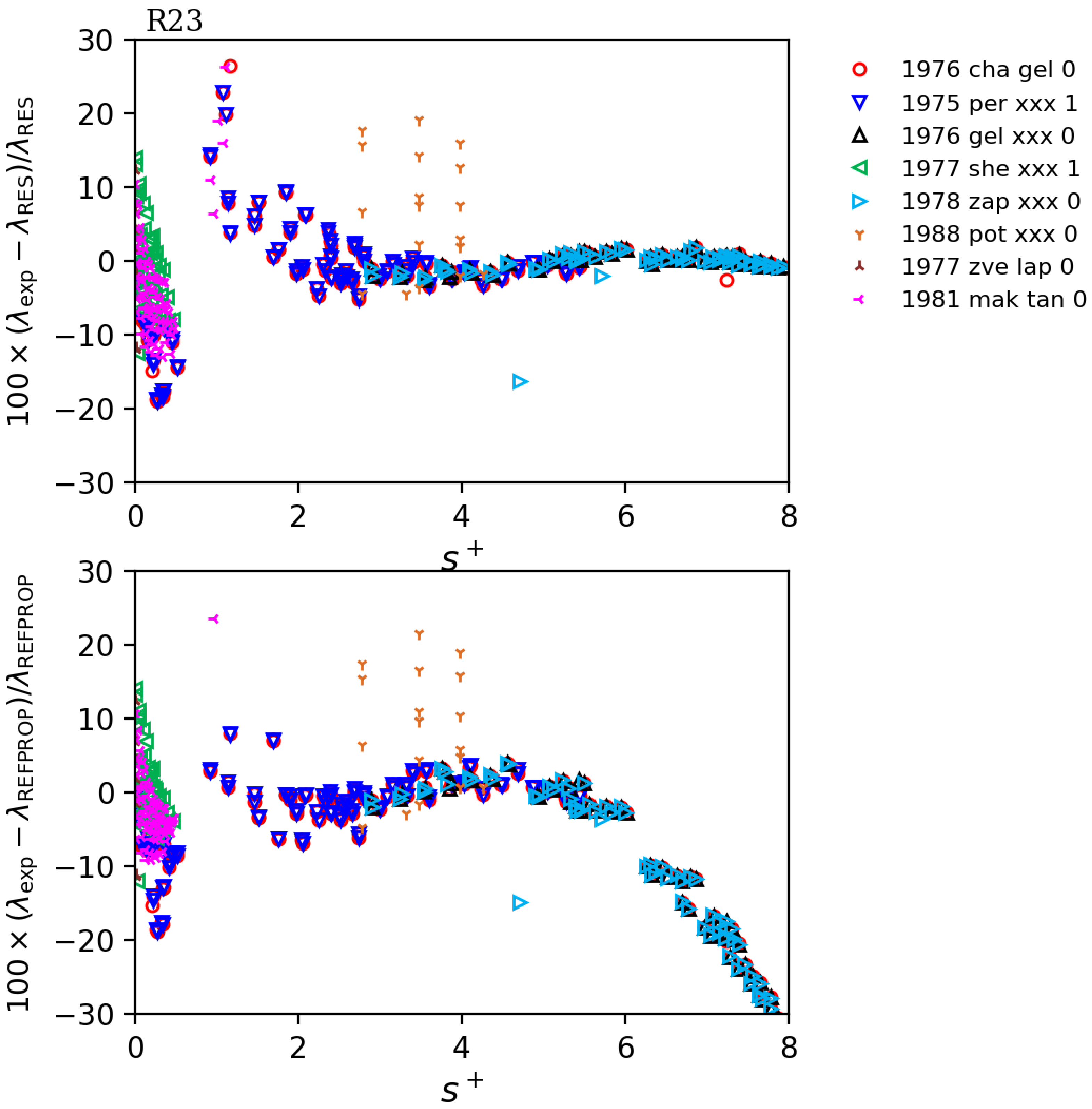

Figure DPR2. R23

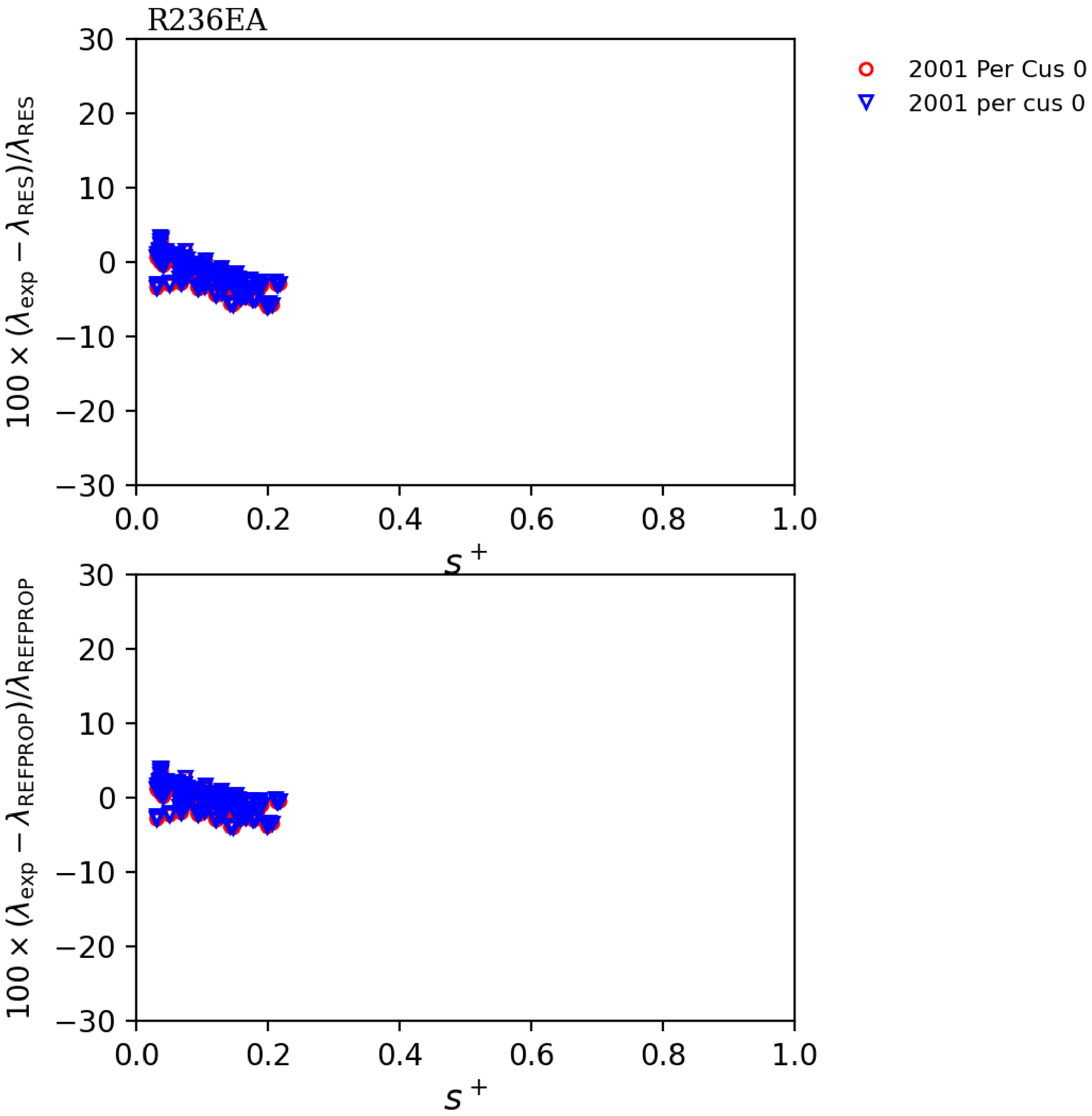

Figure DPR2. R236EA

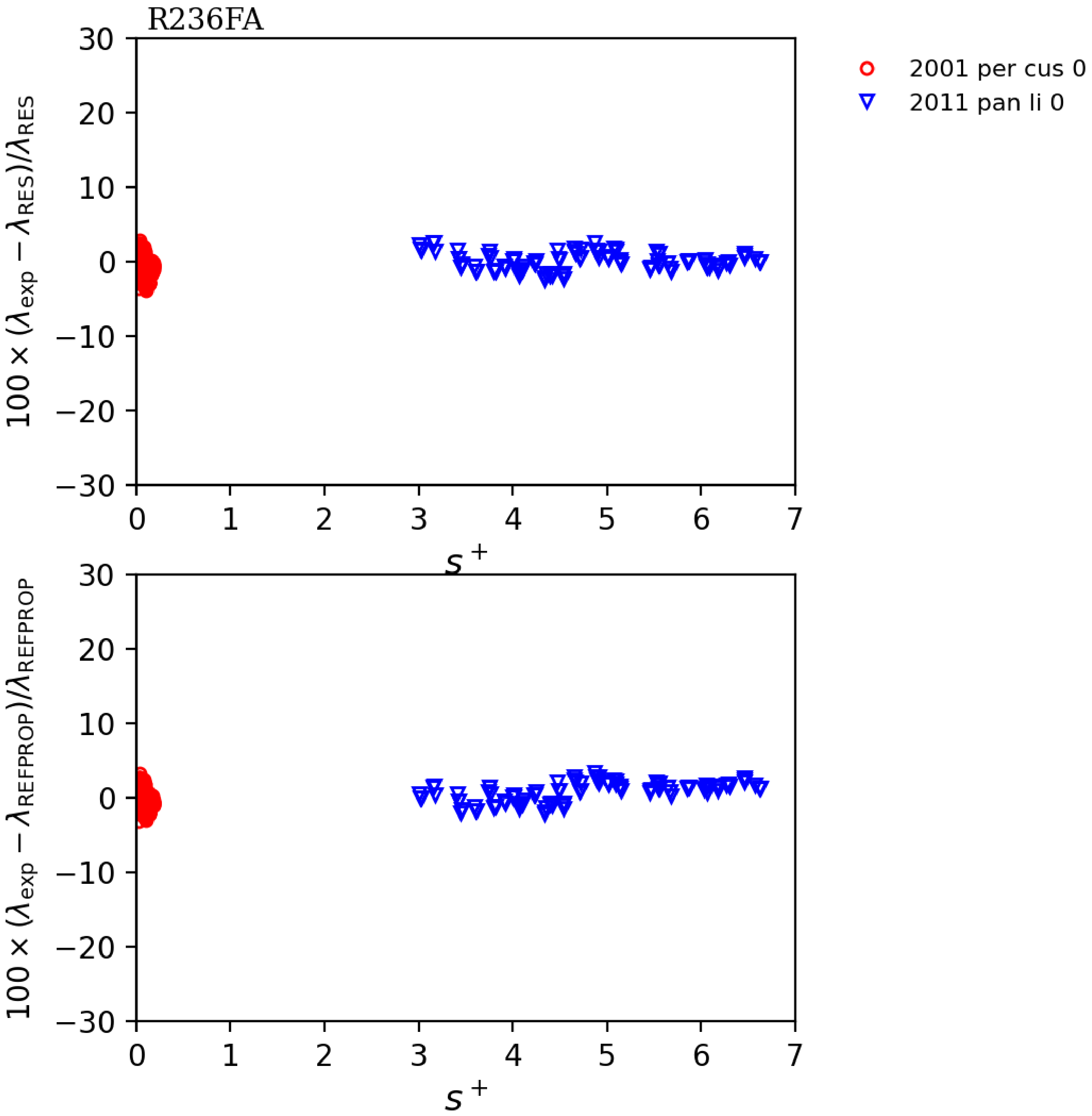

Figure DPR2. R236FA

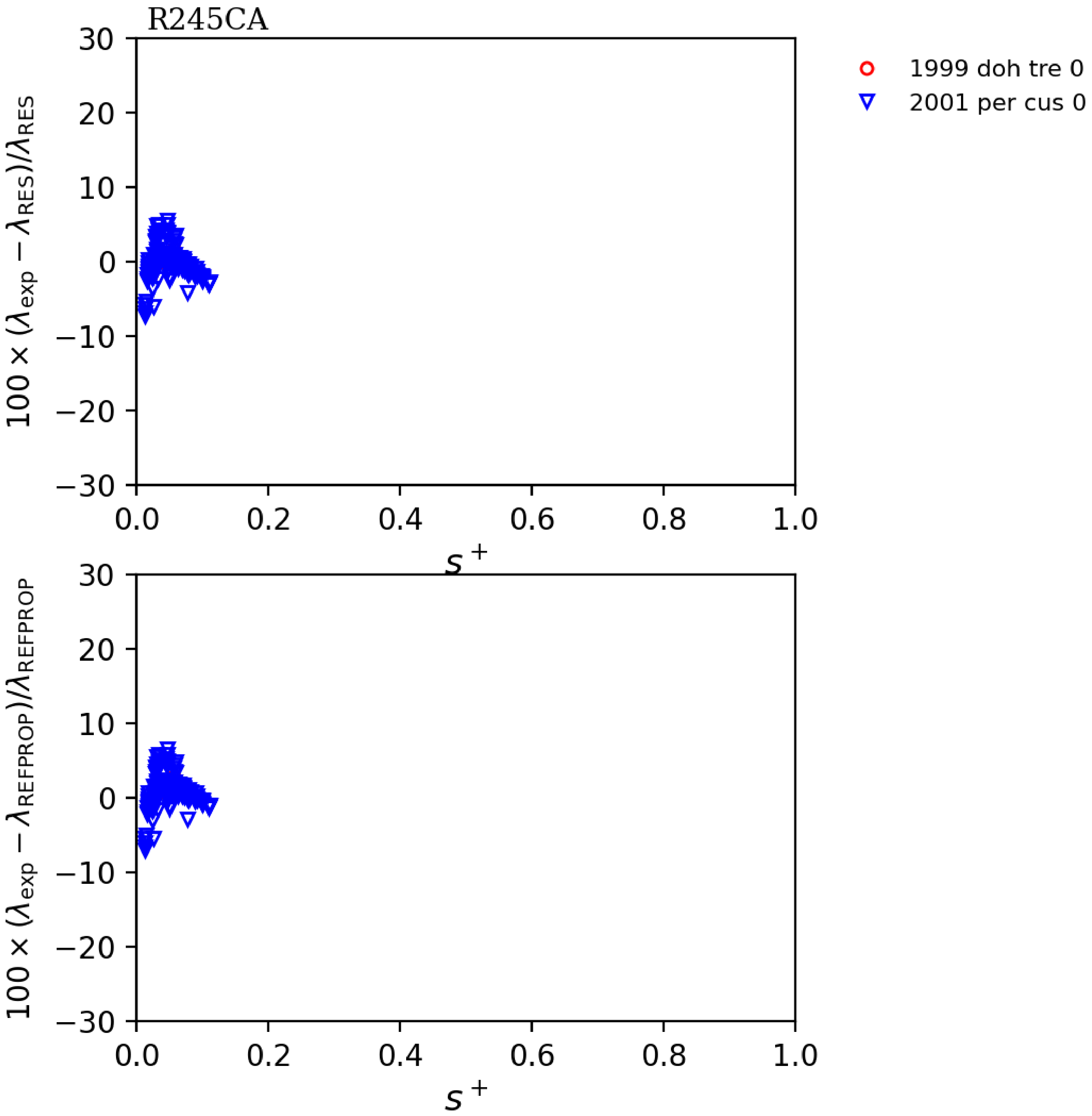

Figure DPR2. R245CA

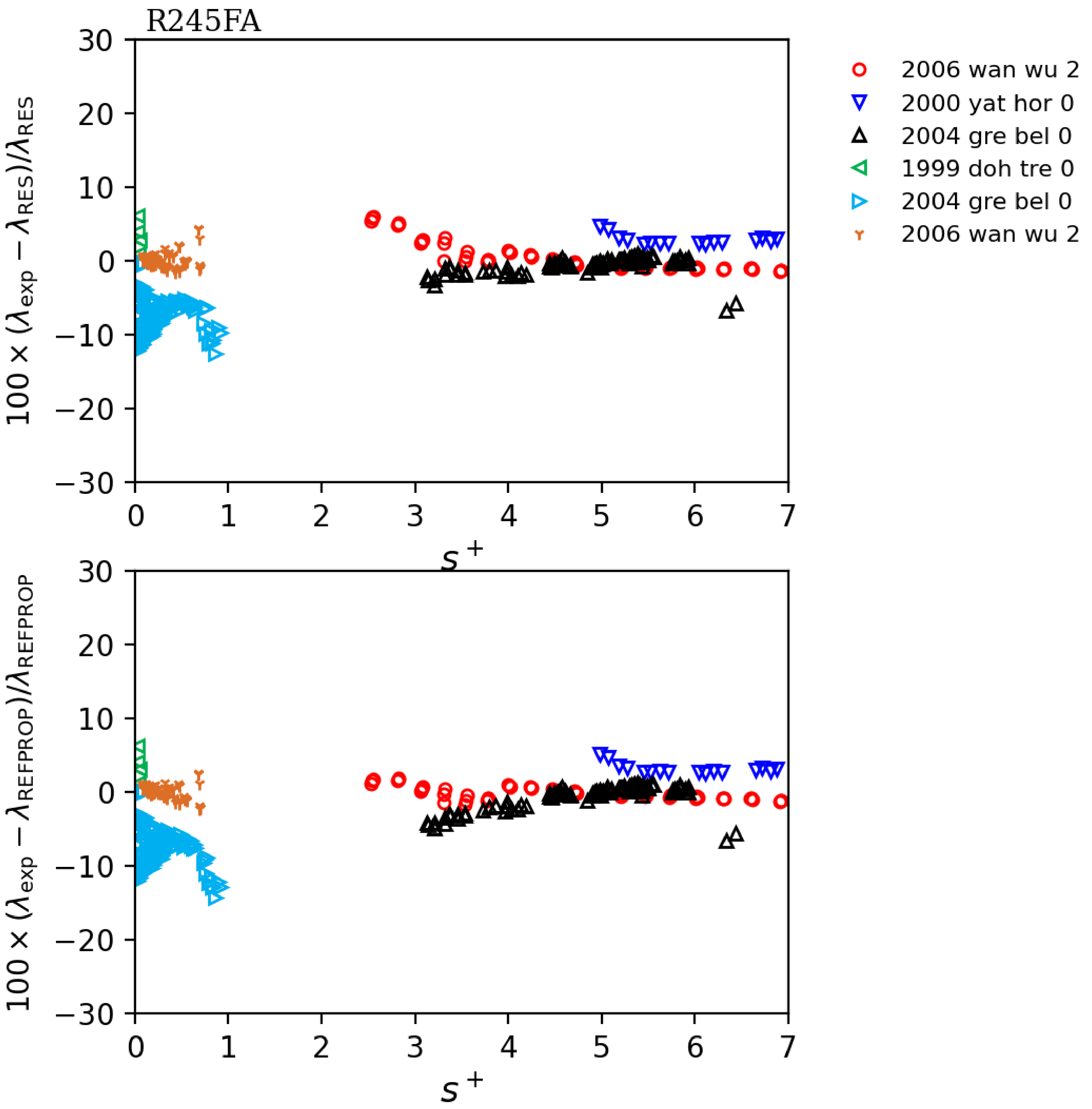

Figure DPR2. R245FA

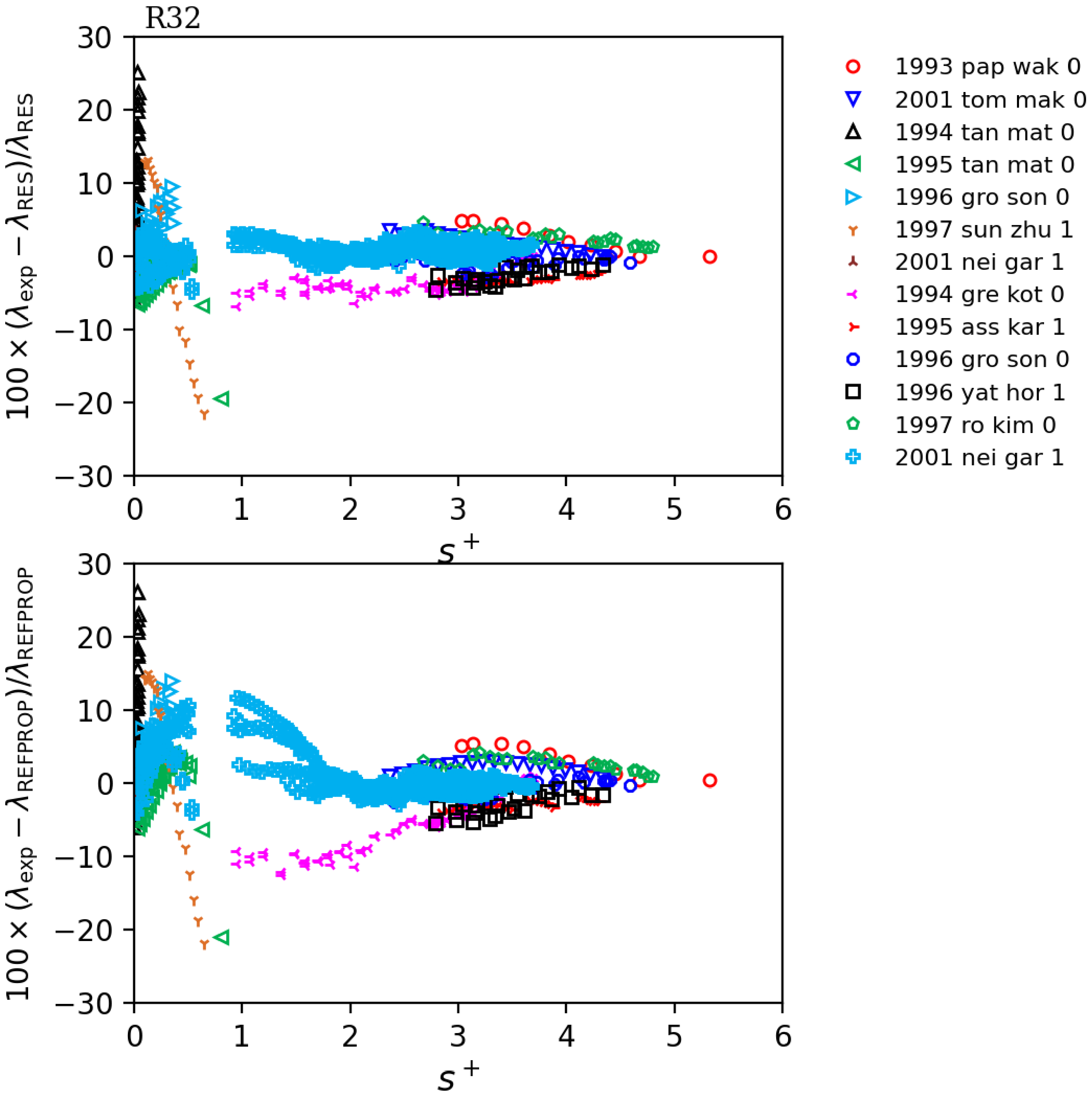

Figure DPR2. R32

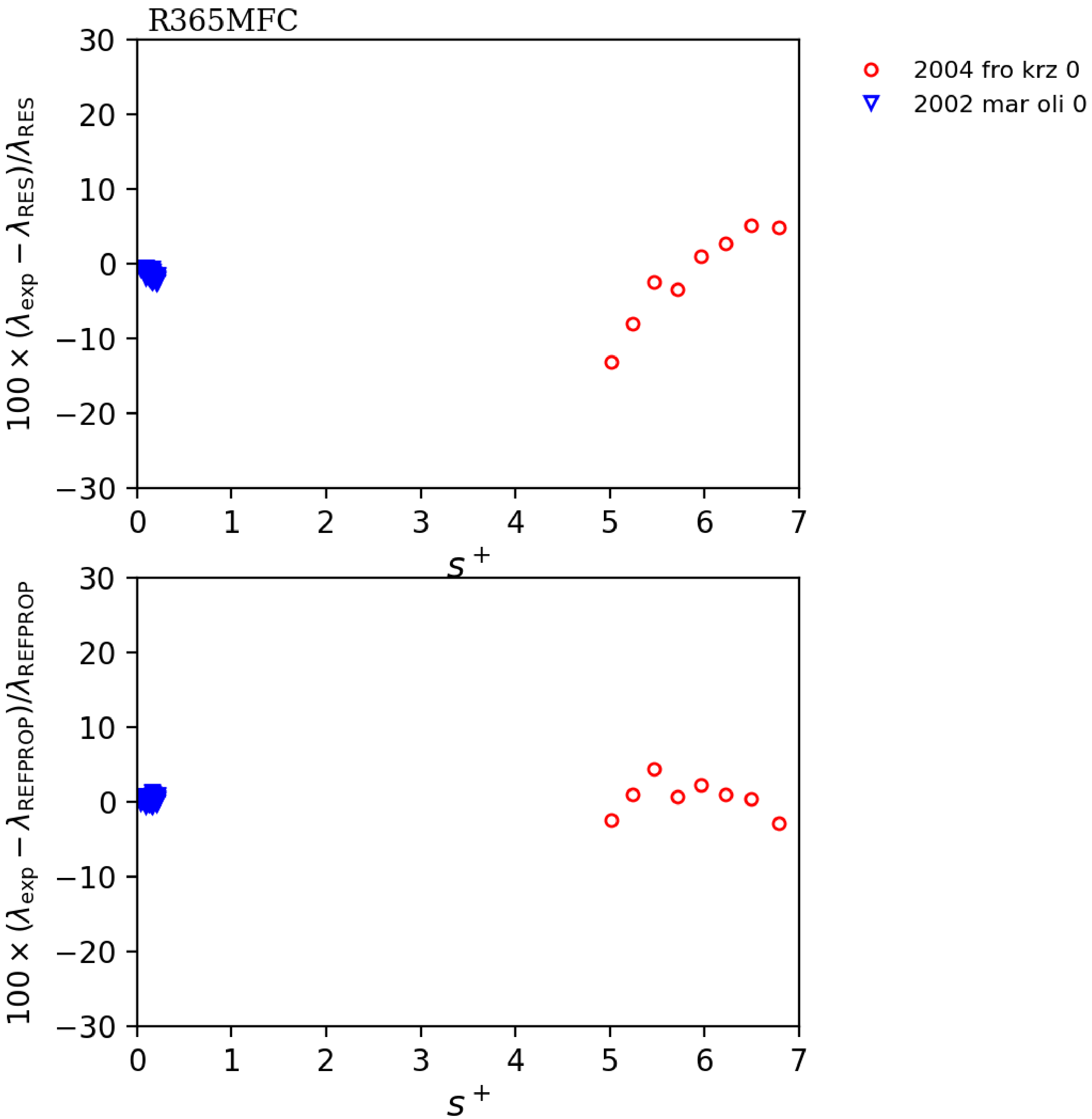

Figure DPR2. R365MFC

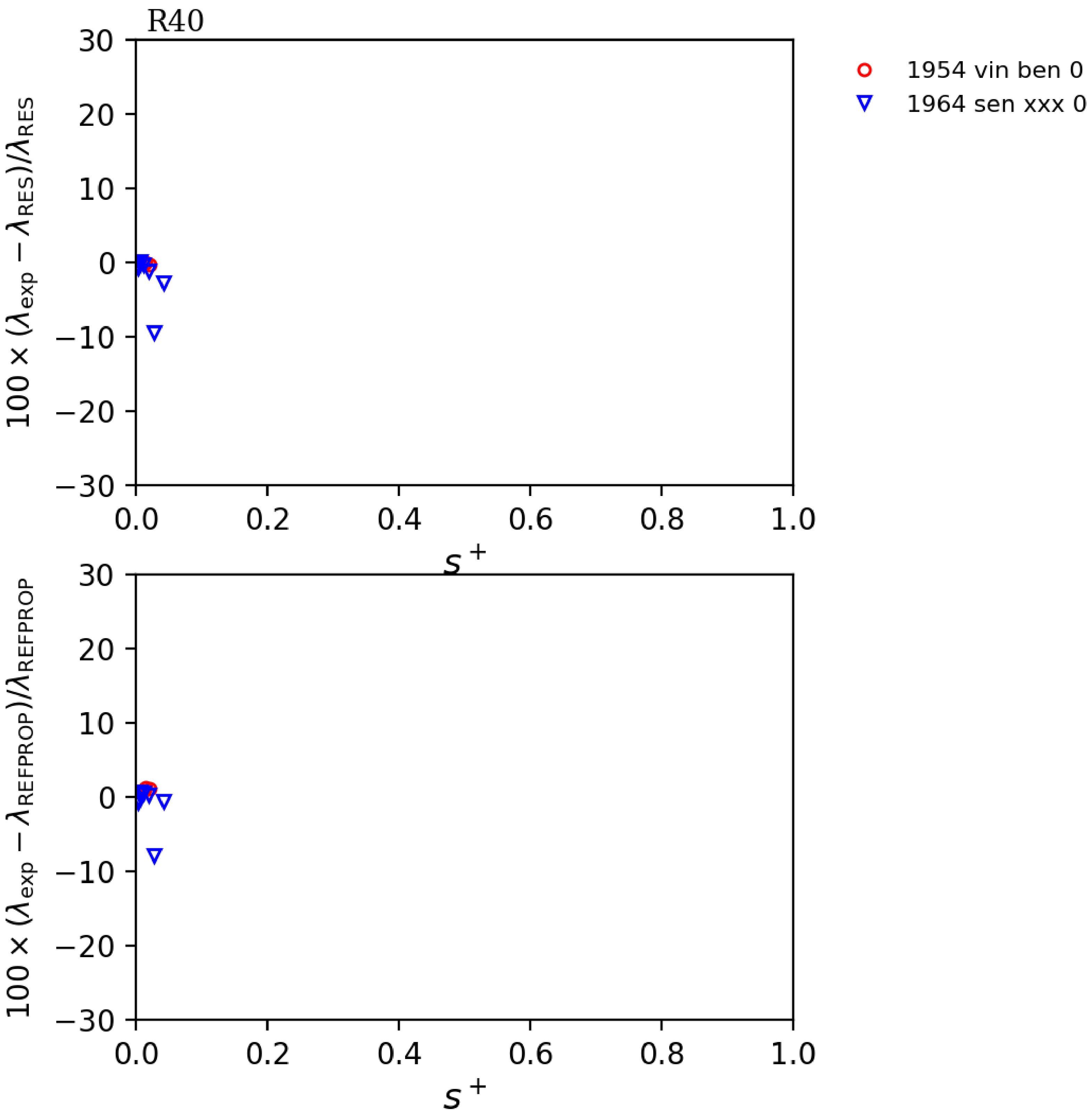

Figure DPR2. R40

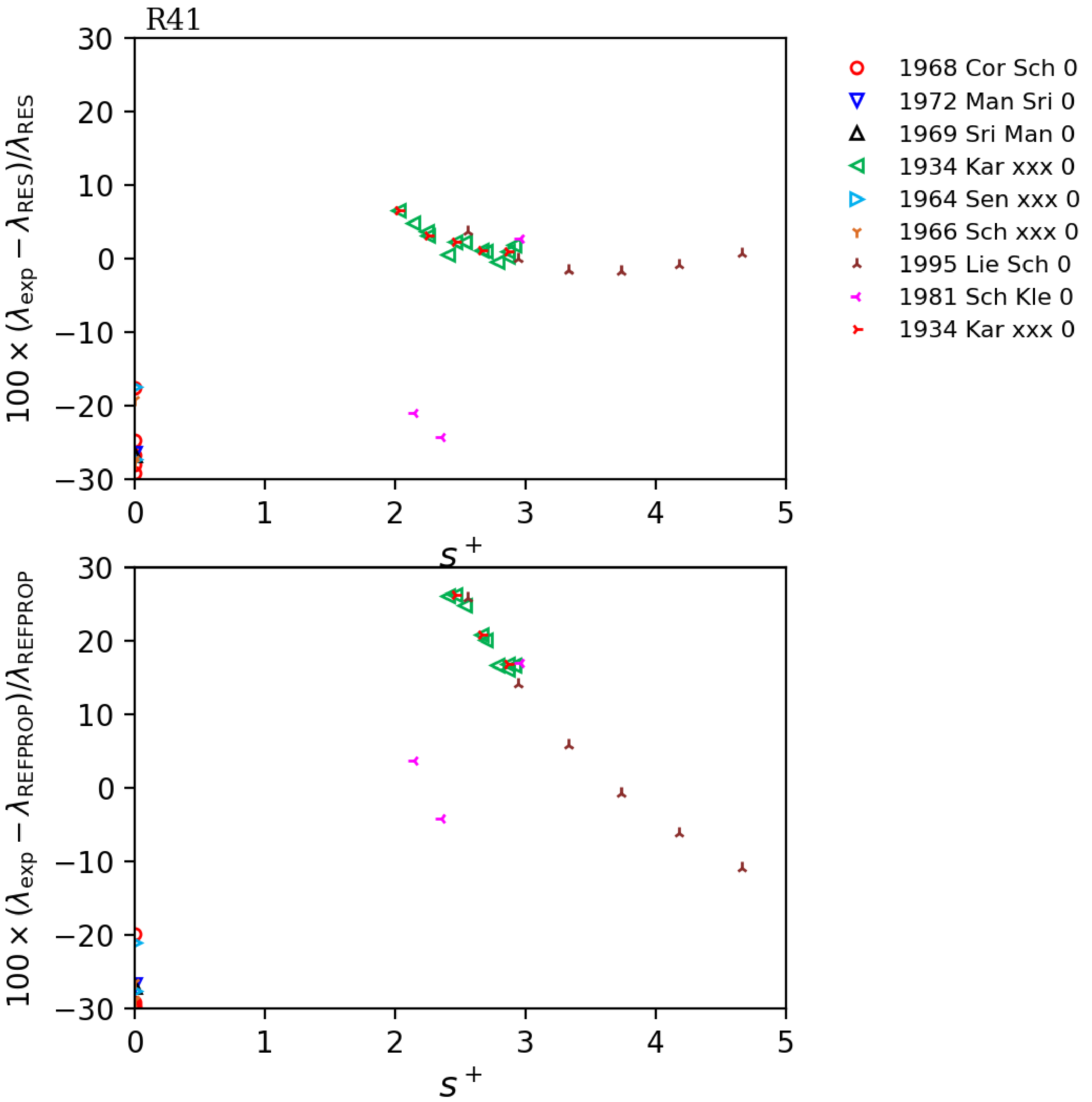

Figure DPR2. R41

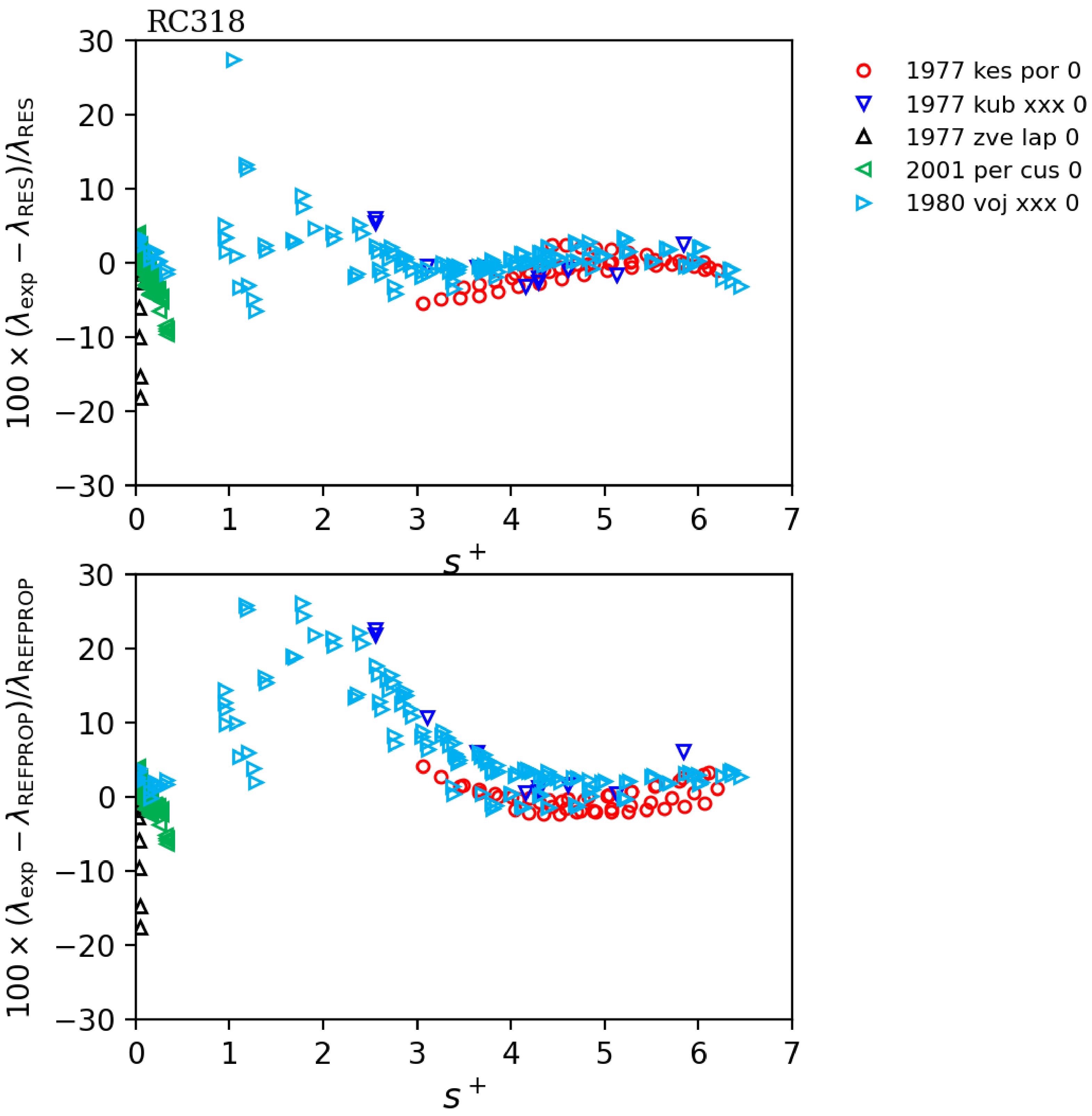

Figure DPR2. RC318

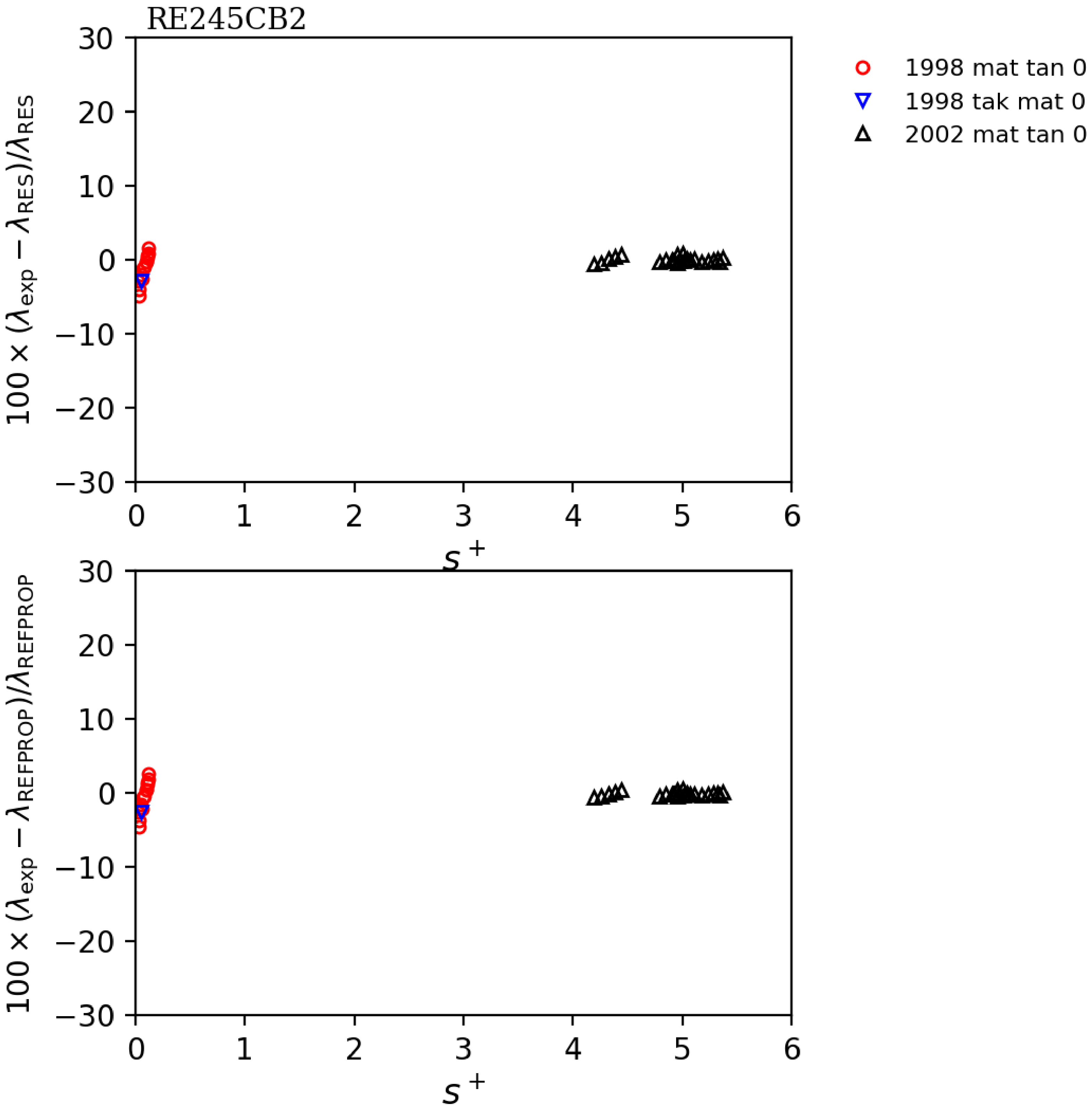

Figure DPR2. RE245CB2

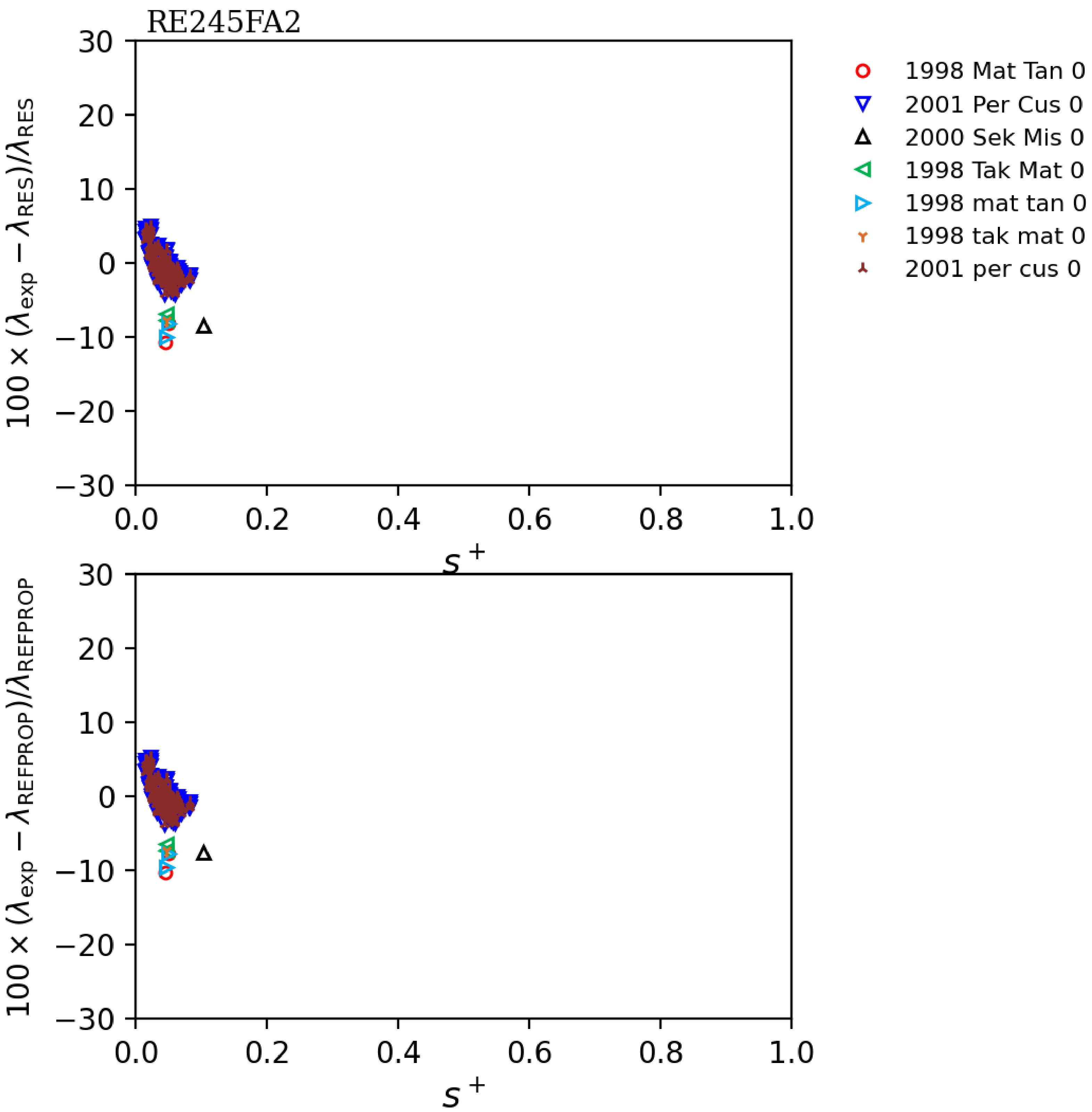

Figure DPR2. RE245FA2

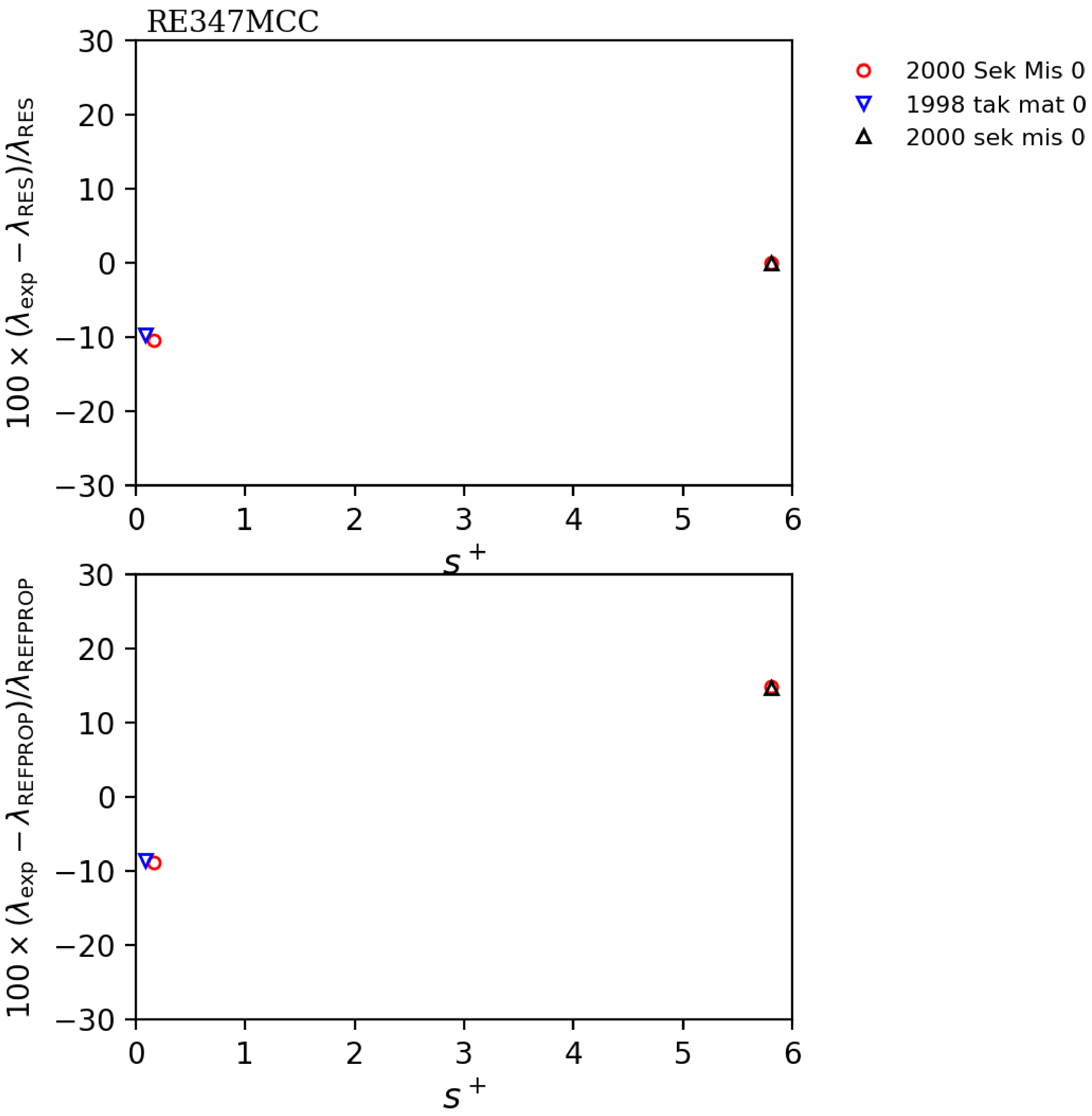

Figure DPR2. RE347MCC

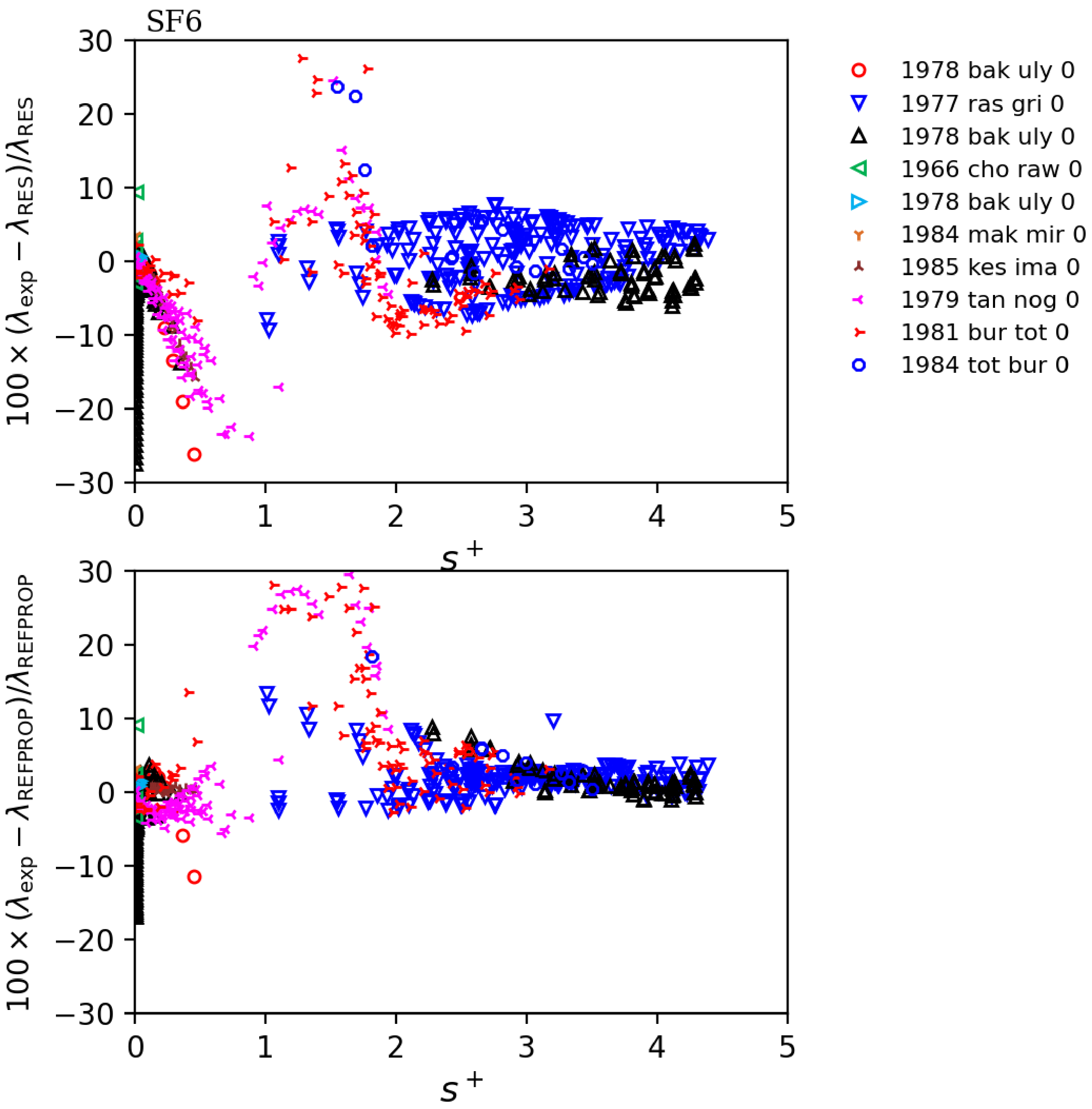

Figure DPR2. SF6

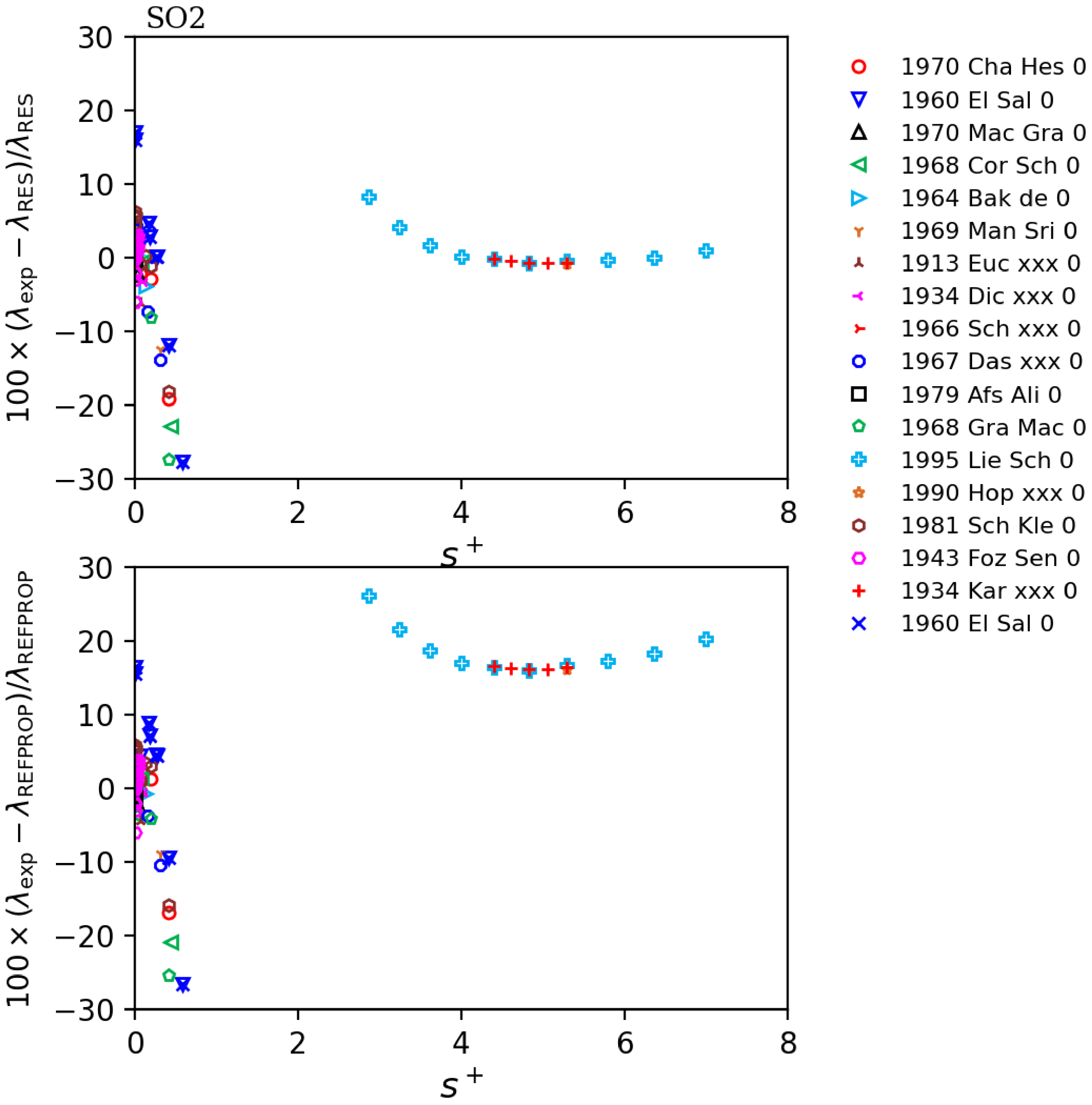

Figure DPR2. SO2

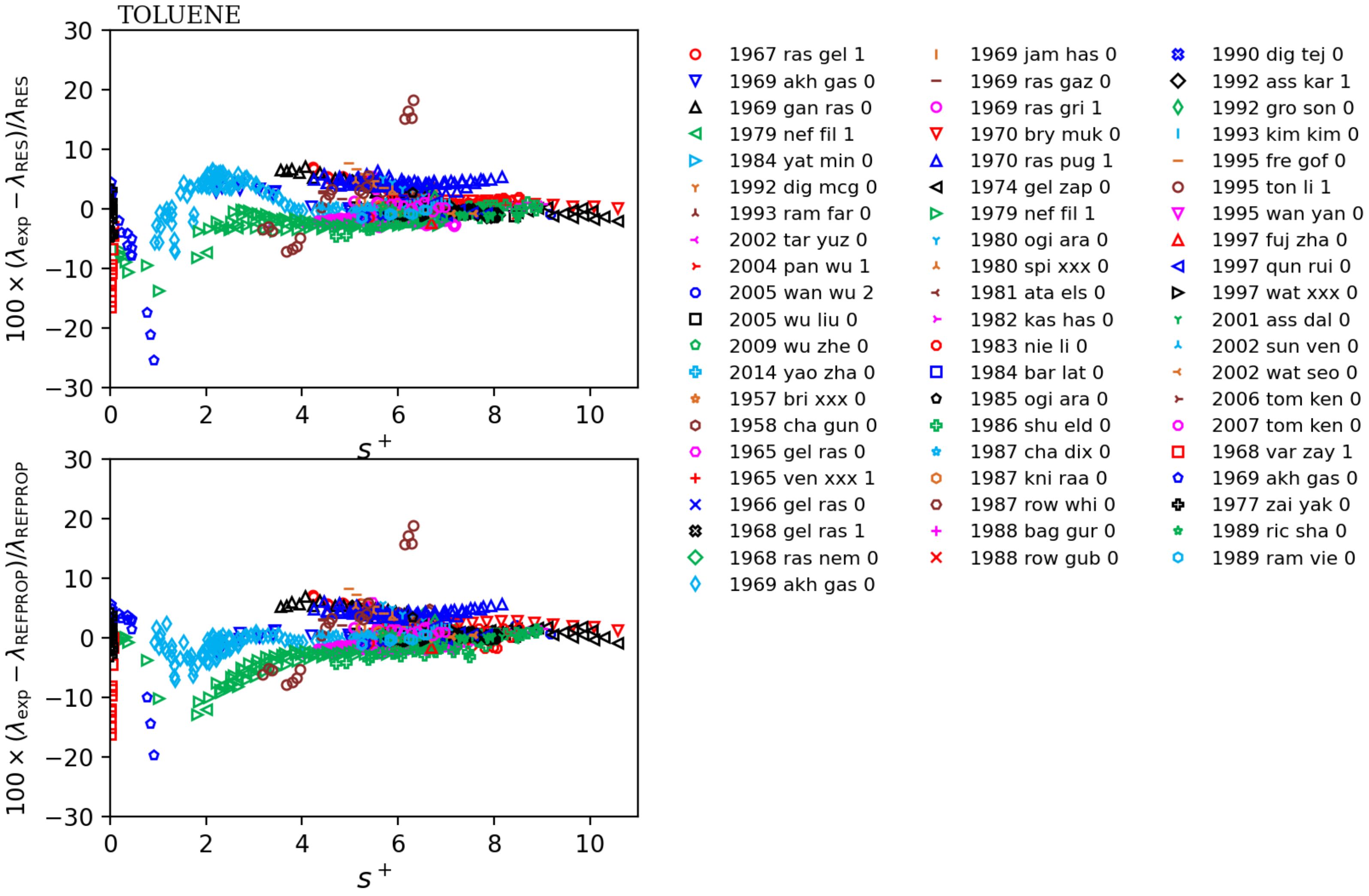

Figure DPR2. TOLUENE

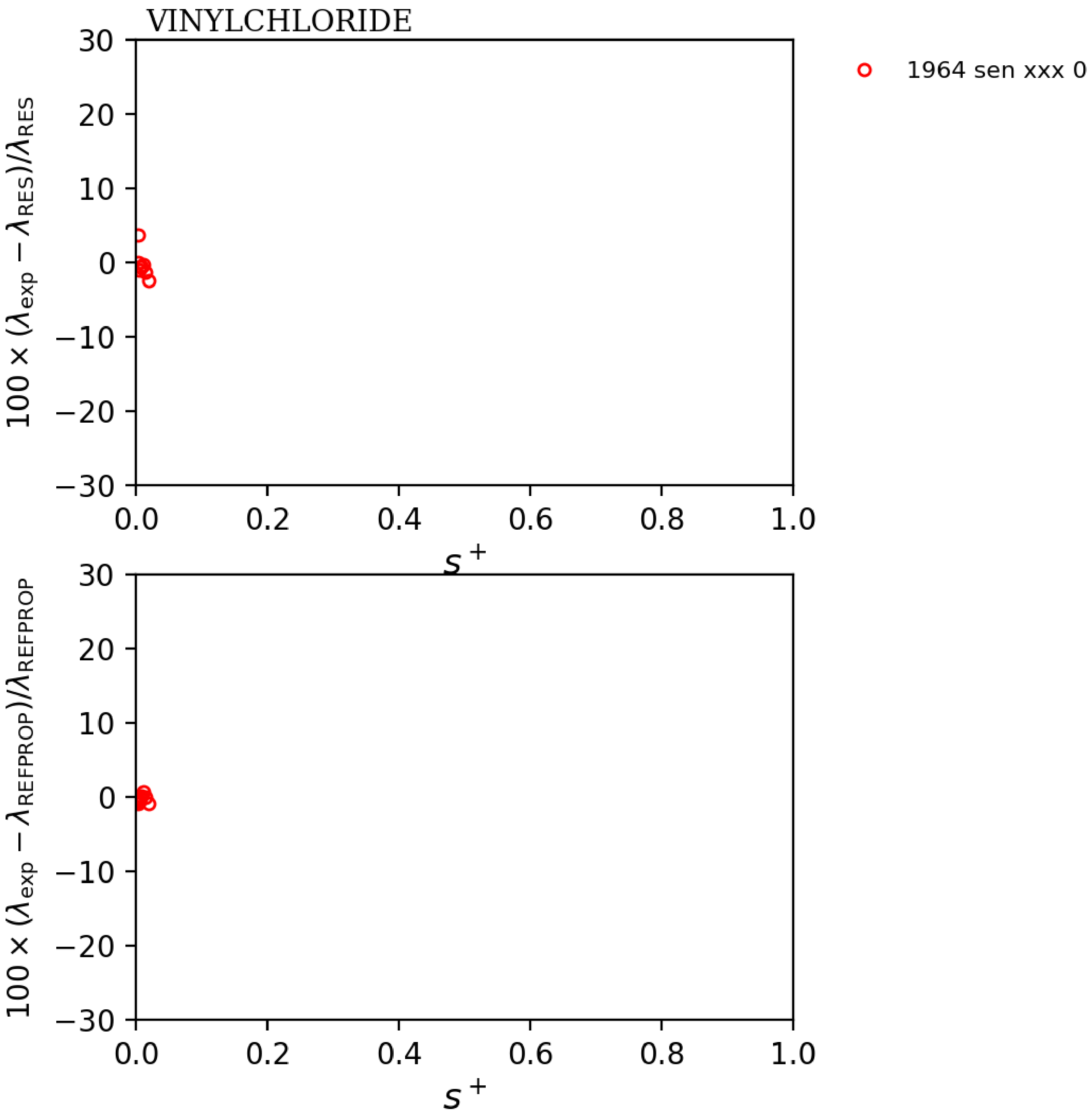

Figure DPR2. VINYLCHLORIDE

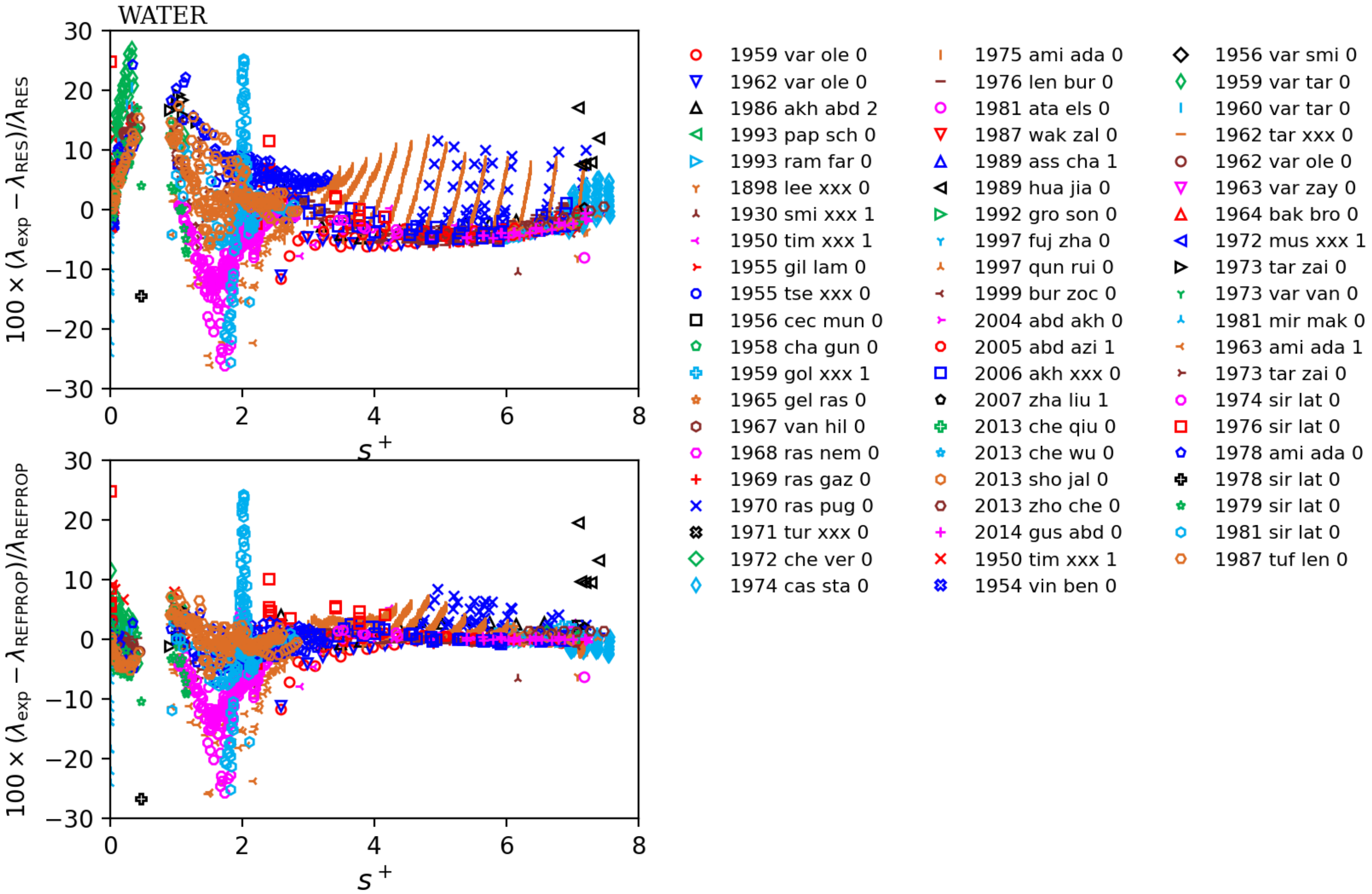

Figure DPR2. WATER

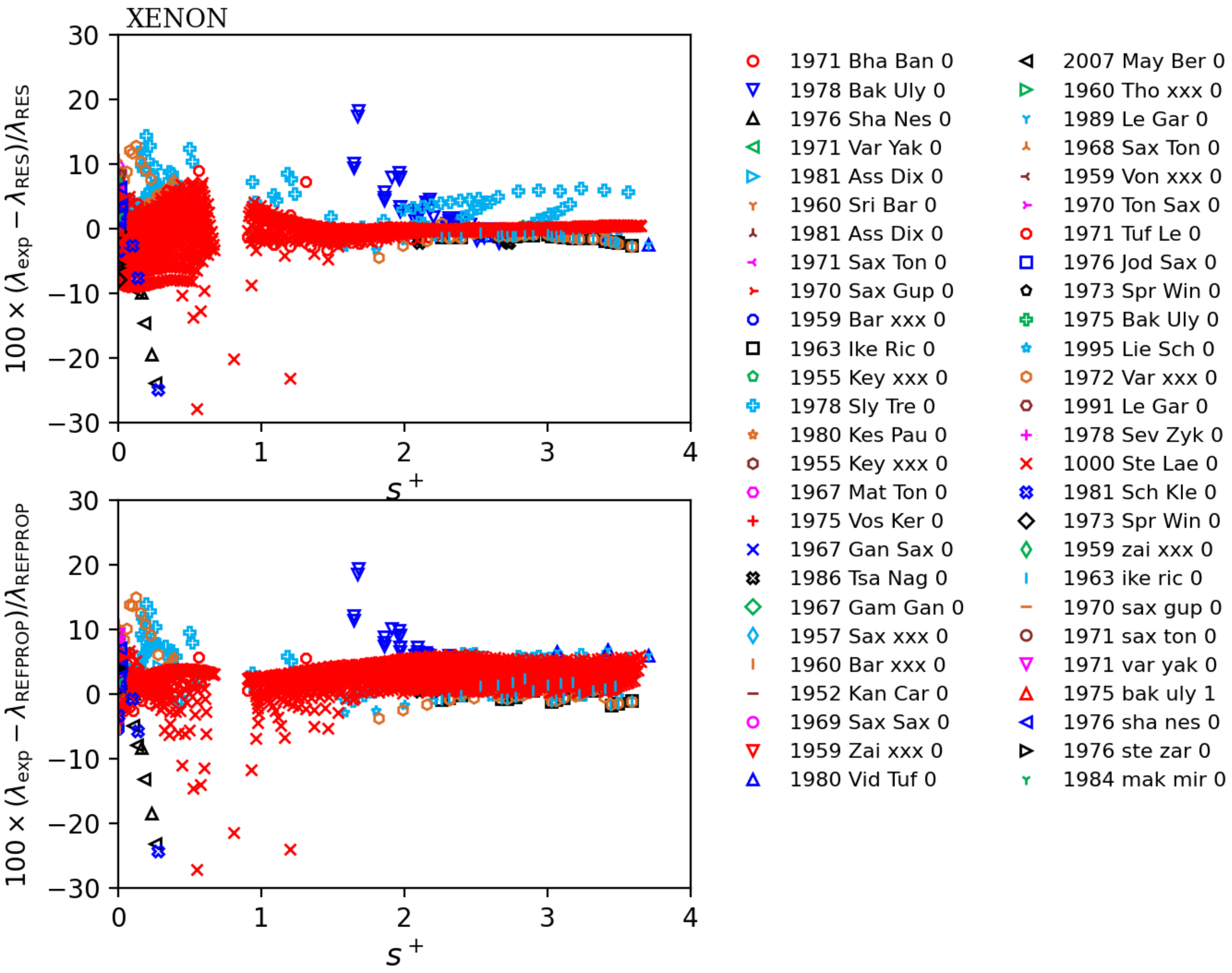

Figure DPR2. XENON

3. Relative deviation from experimental values of each mixture to models

In this section, the dimensionless residual thermal conductivity λ^{+}_{res} as a function of dimensionless residual entropy s^{+} for experimental data of each mixture are plotted. The legends denote the sources of the data, showing the published year,the first three letters of the first author's family name ,and the firstthree letters of the second author's family name ('xxx' if there is no second author). The full citations are given at the Reference section.

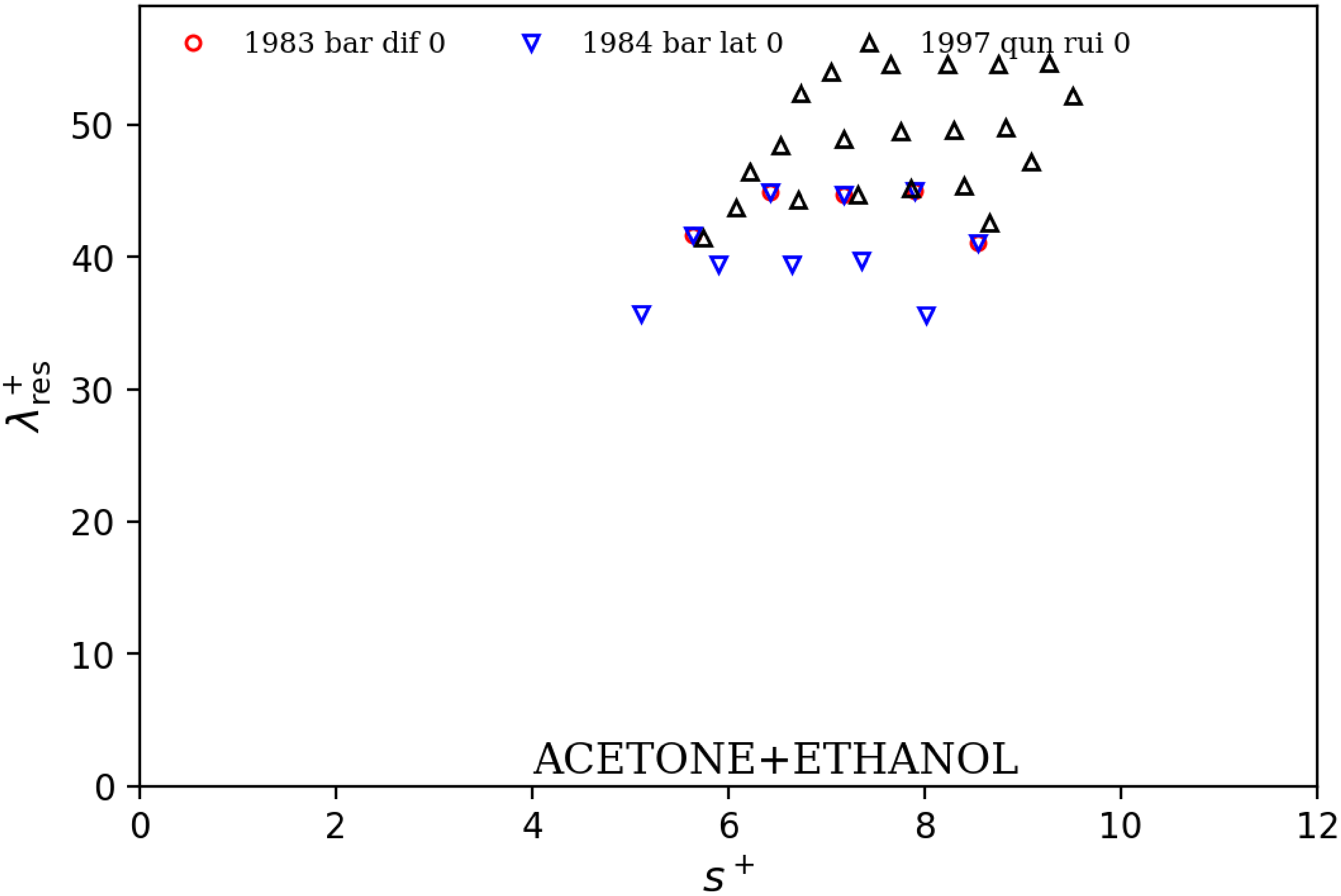

Figure DPR3. ACETONE+ETHANOL

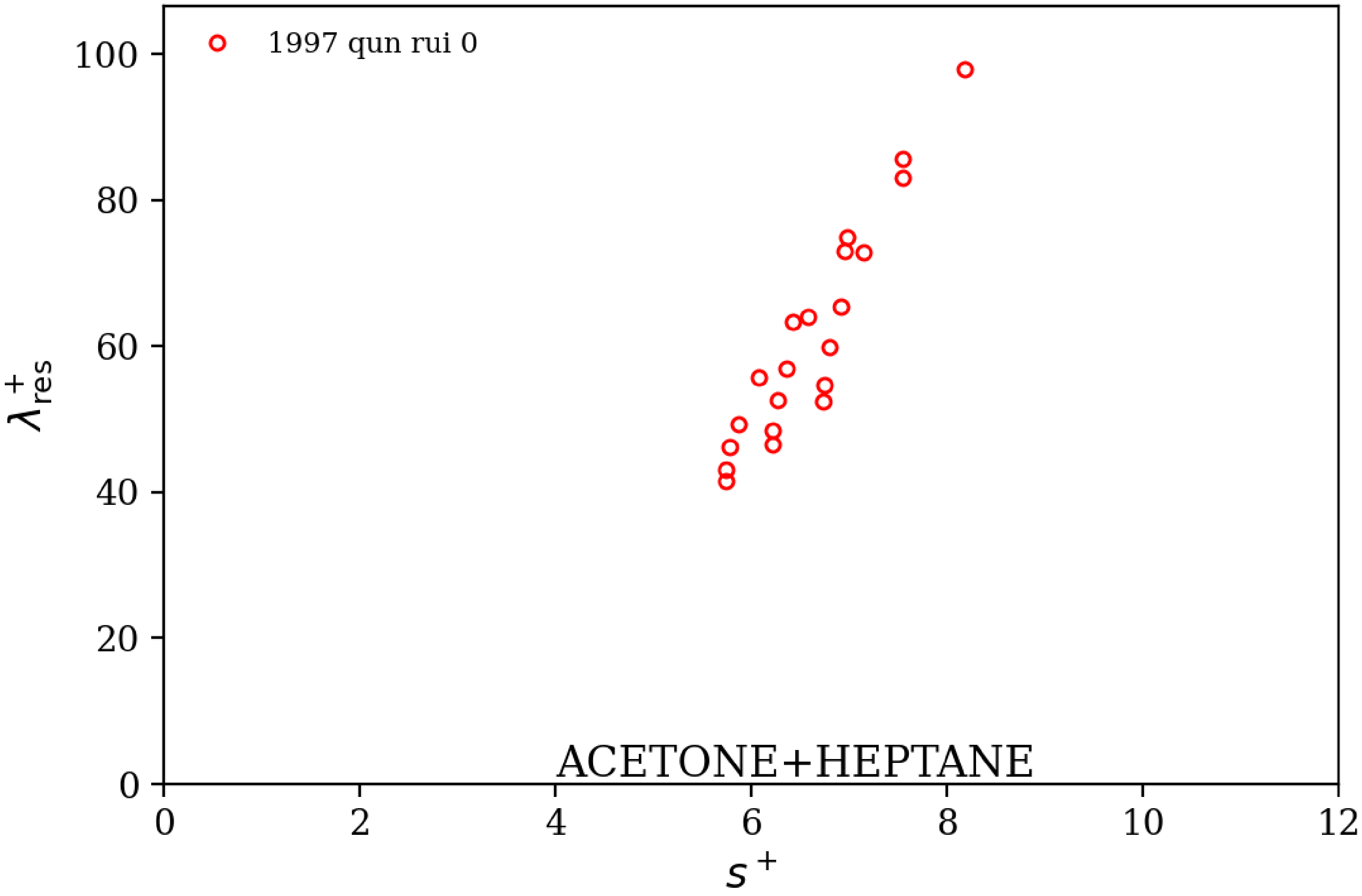

Figure DPR3. ACETONE+HEPTANE

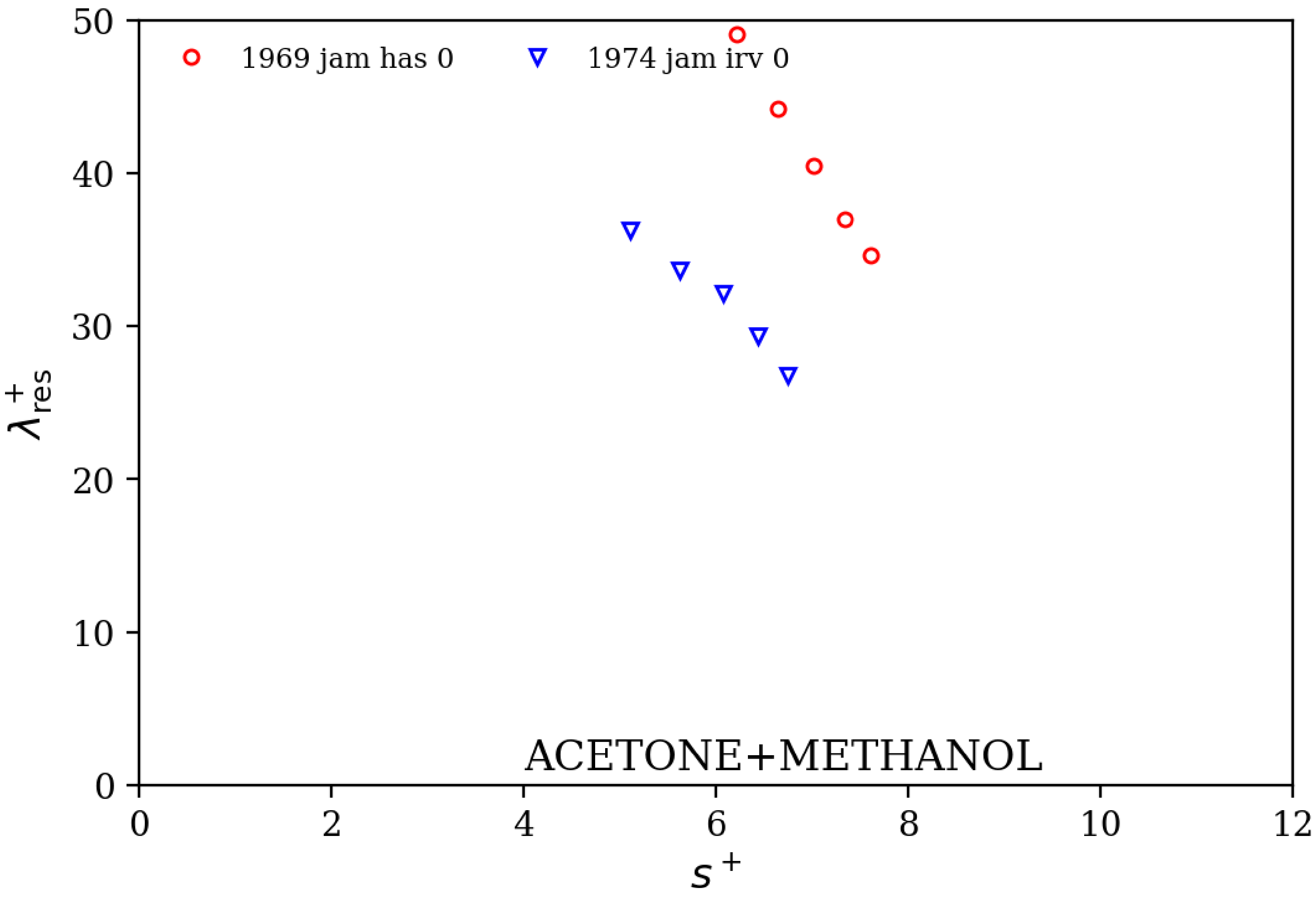

Figure DPR3. ACETONE+METHANOL

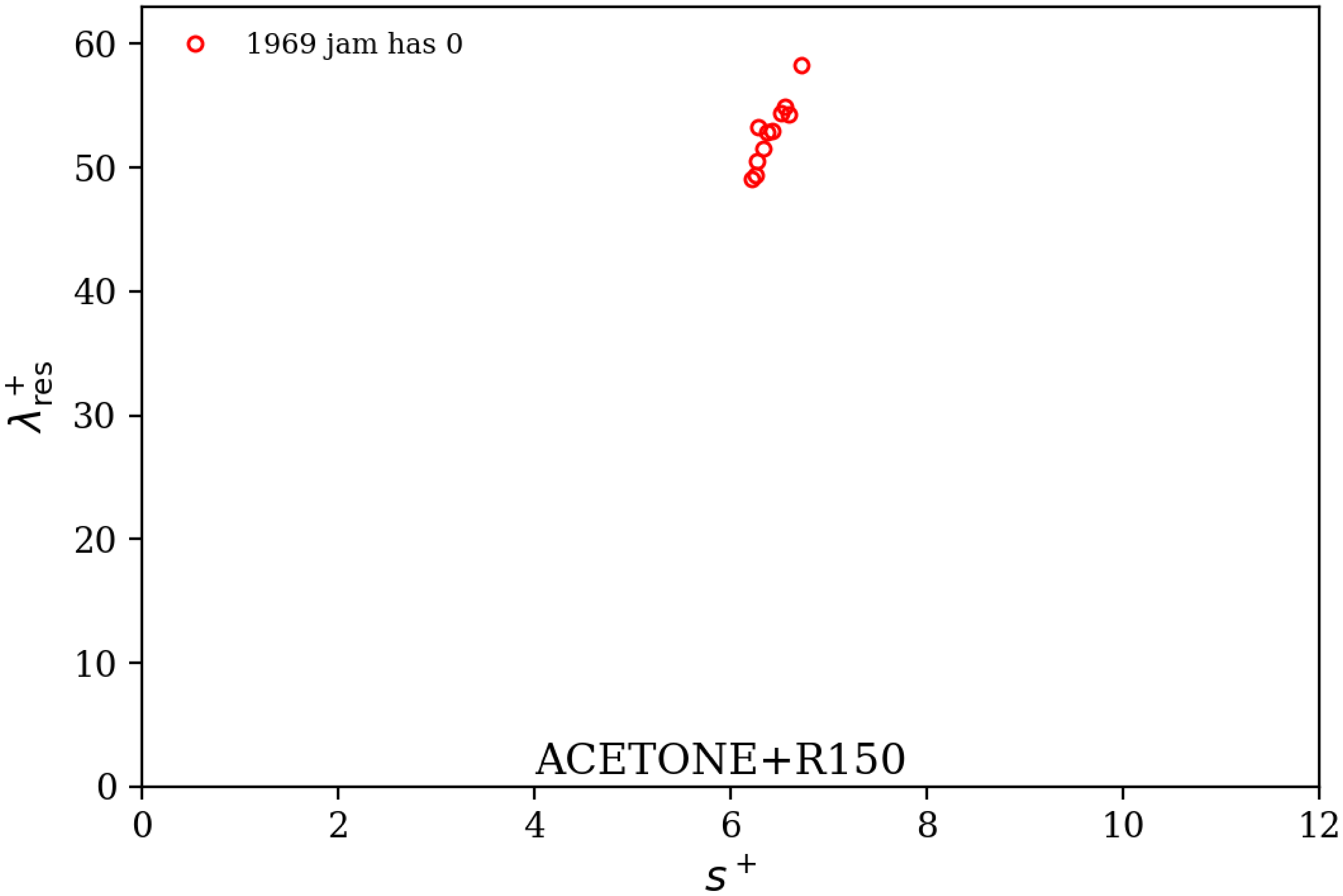

Figure DPR3. ACETONE+R150

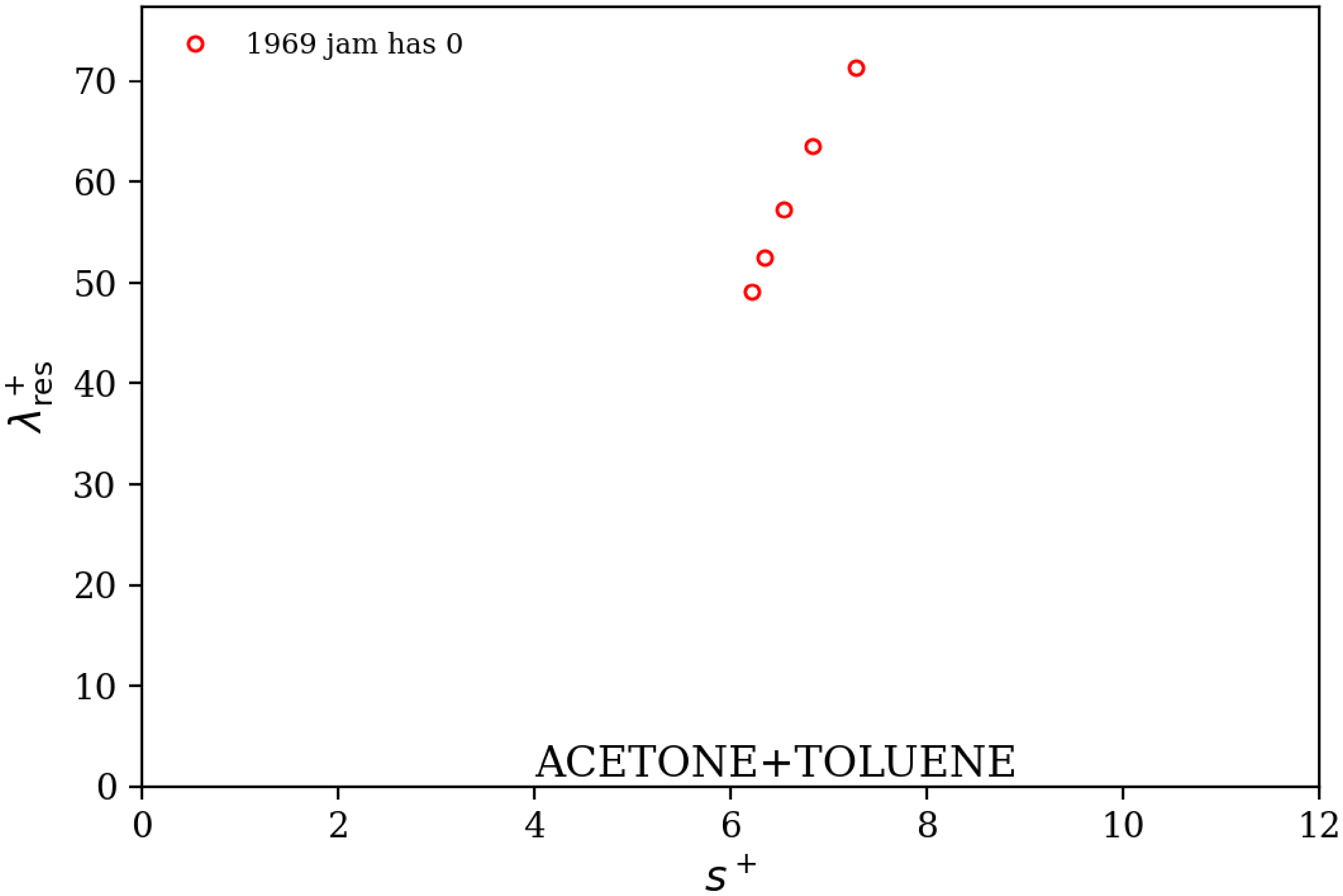

Figure DPR3. ACETONE+TOLUENE

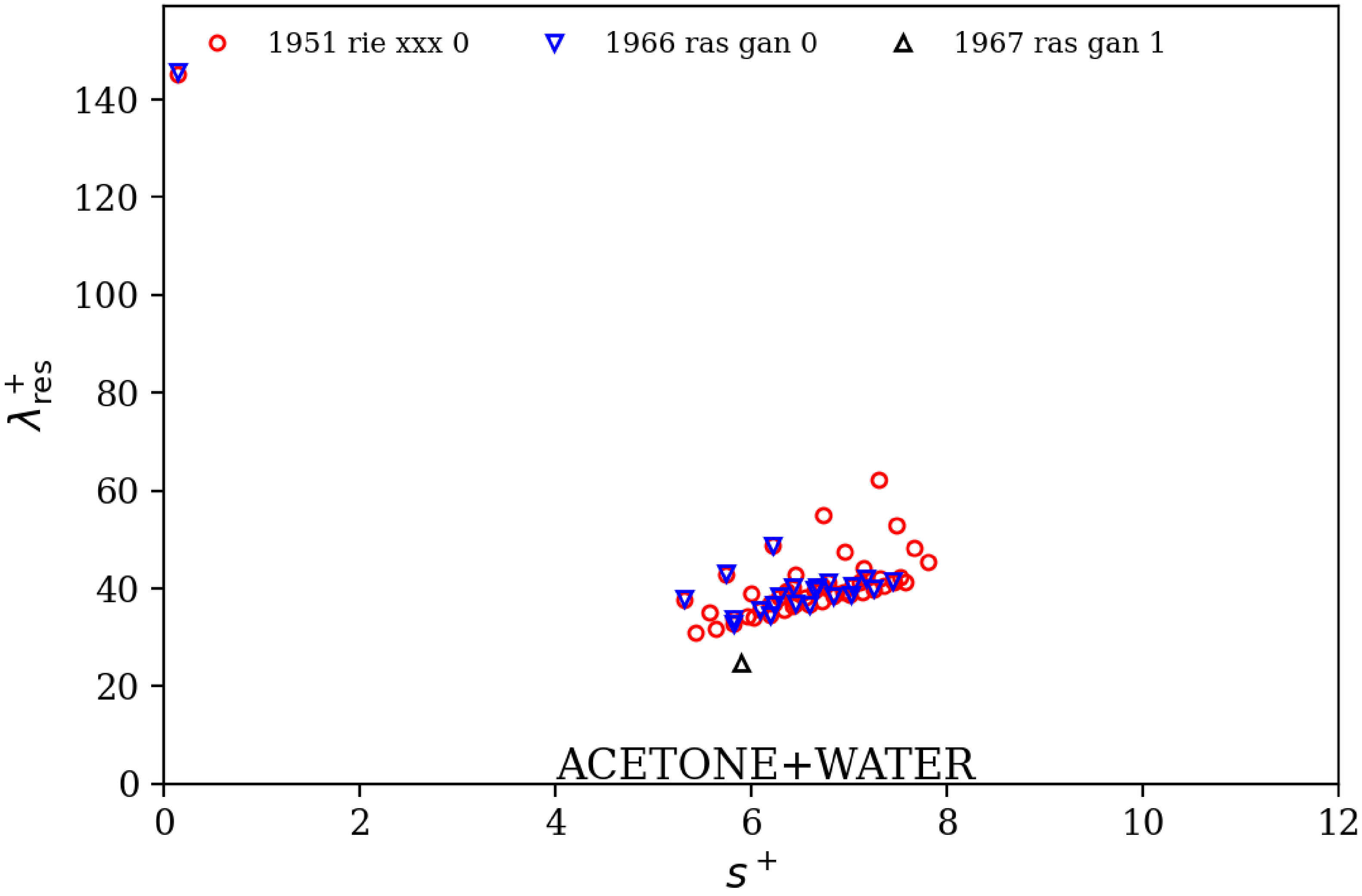

Figure DPR3. ACETONE+WATER

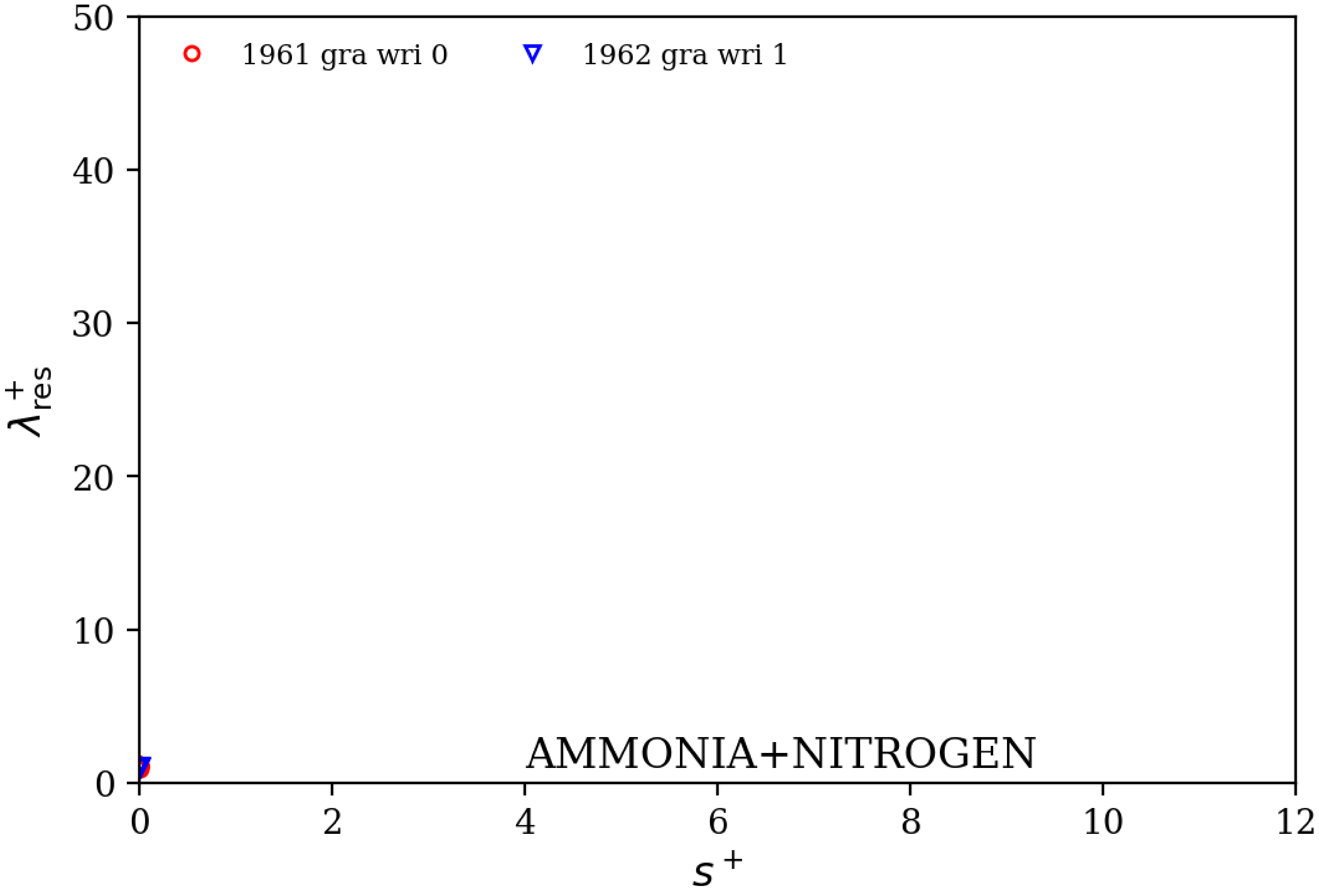

Figure DPR3. AMMONIA+NITROGEN

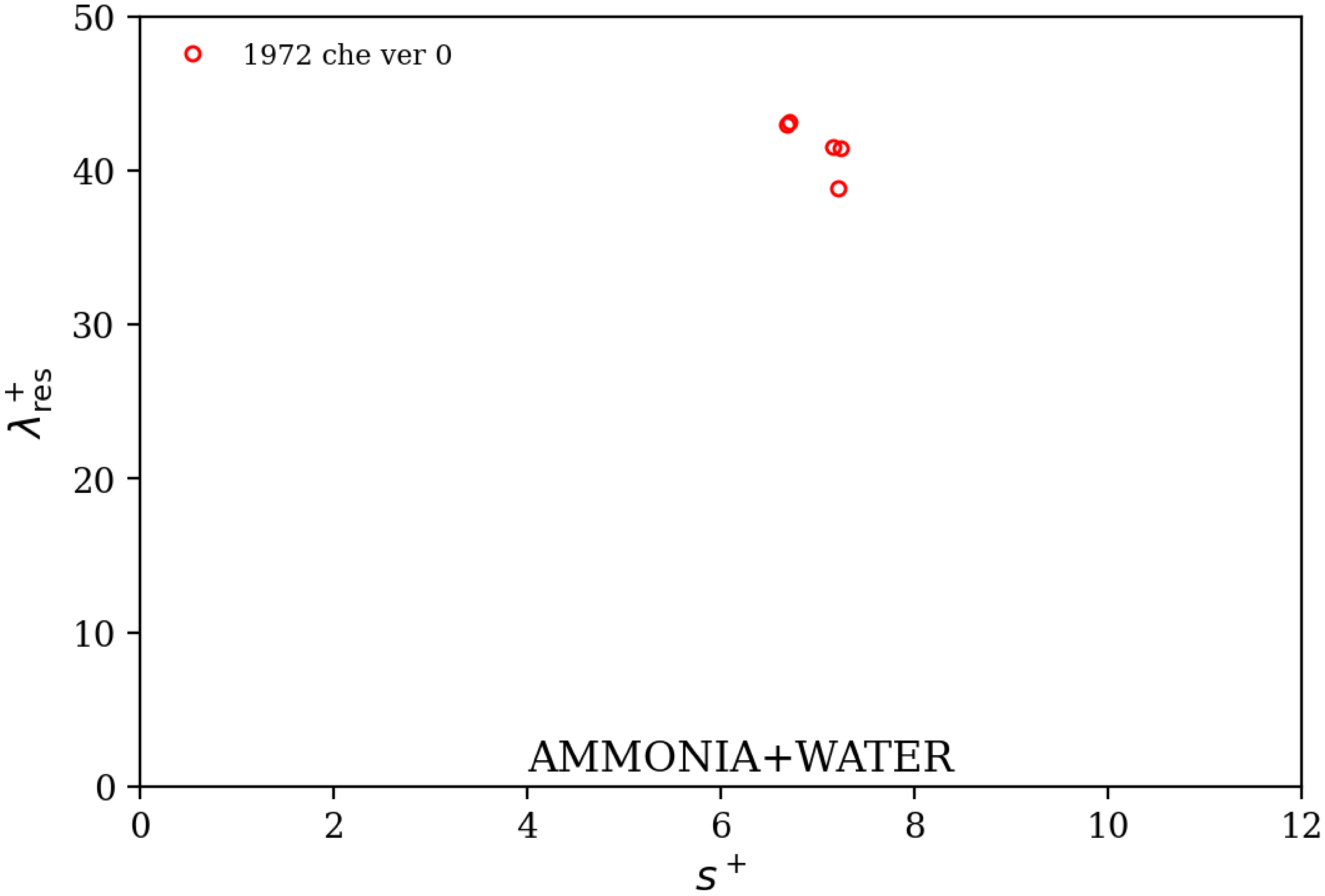

Figure DPR3. AMMONIA+WATER

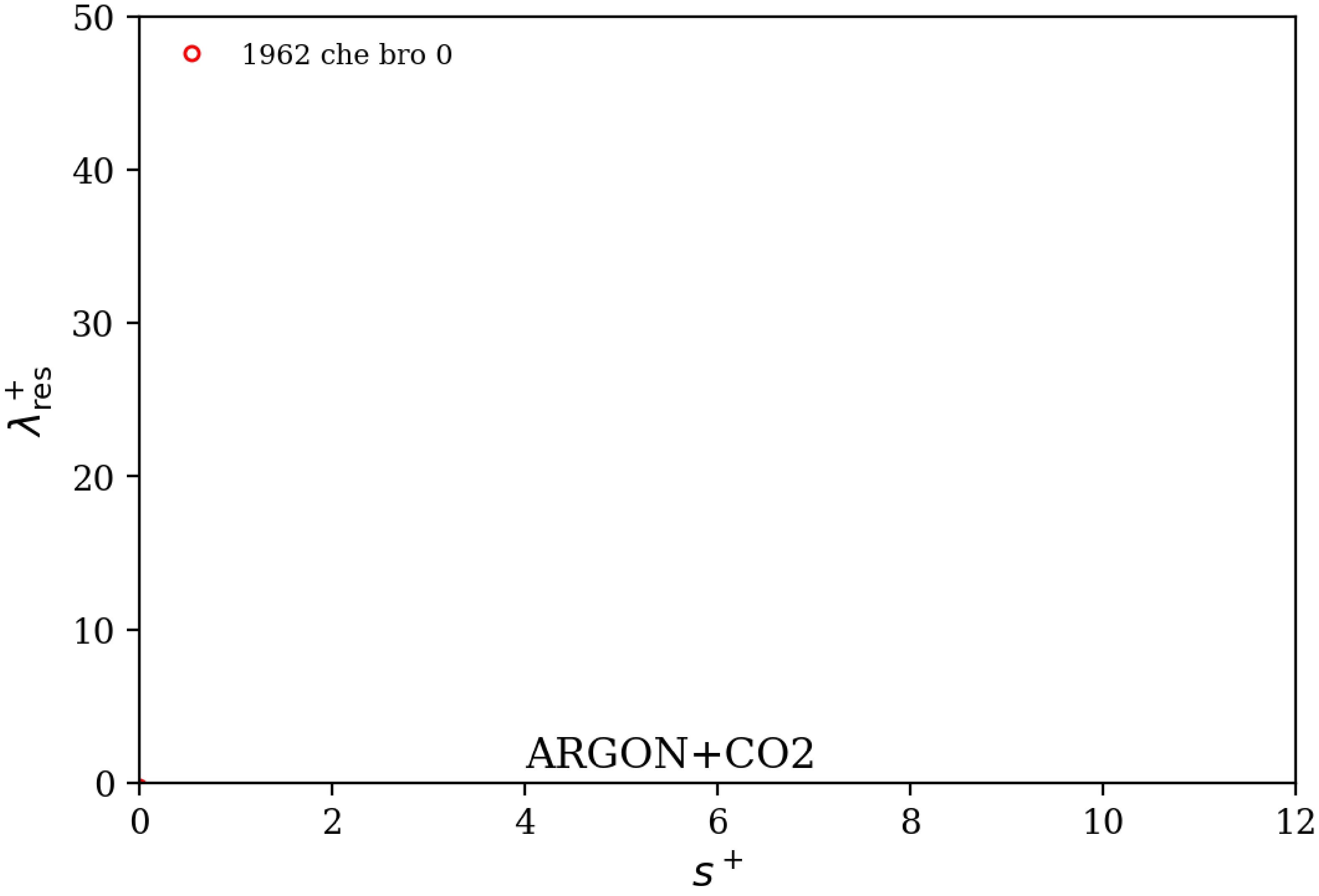

Figure DPR3. ARGON+CO2

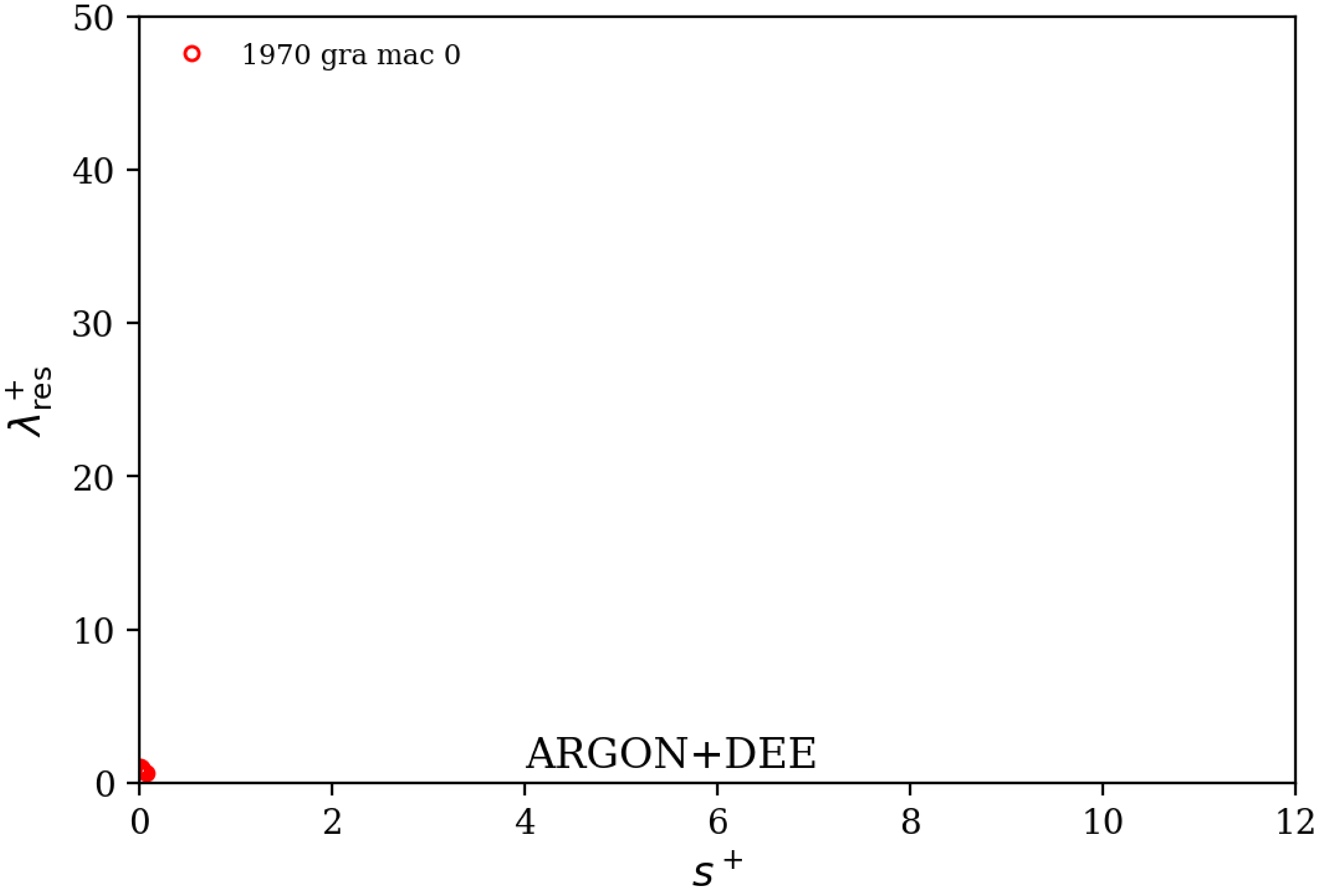

Figure DPR3. ARGON+DEE

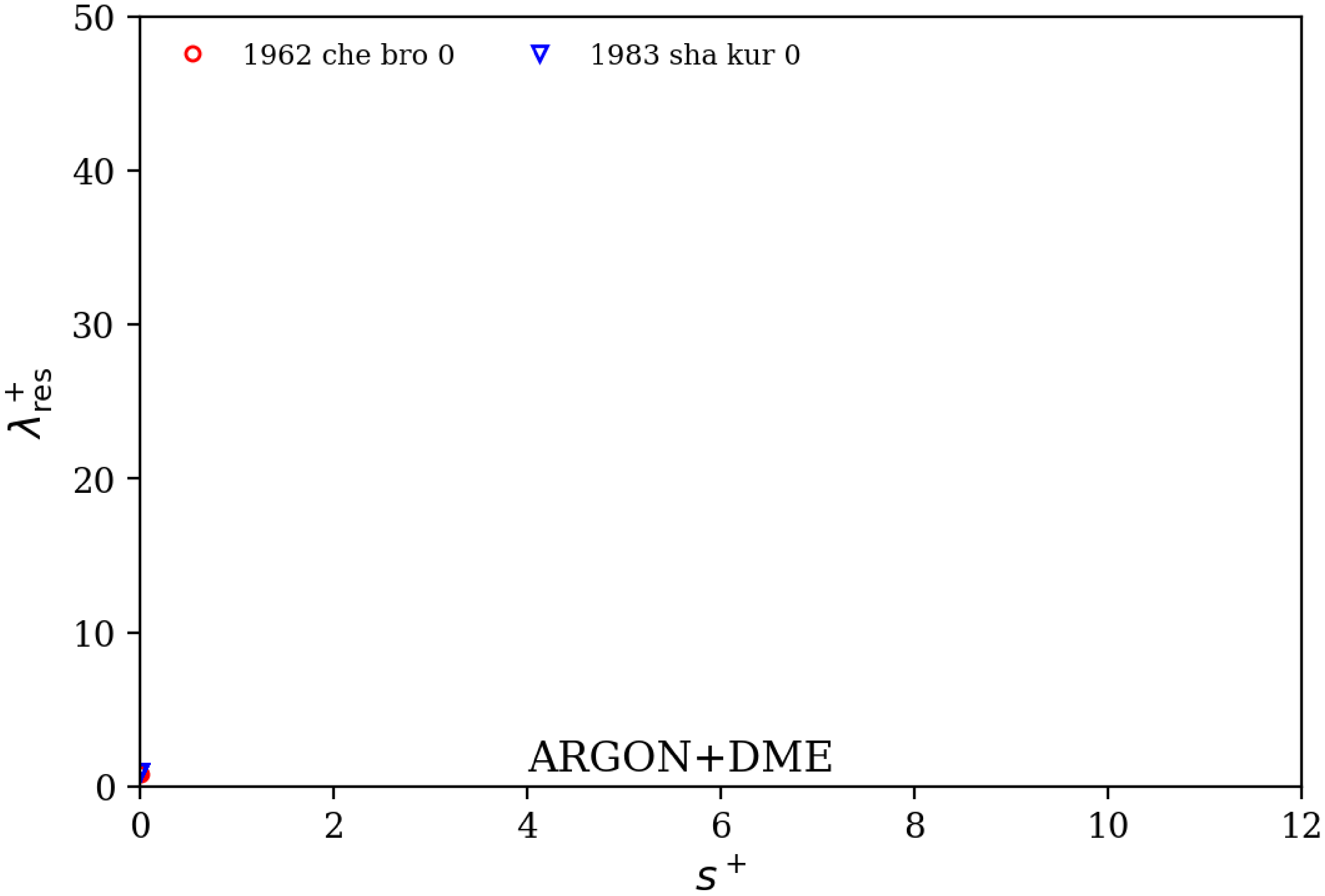

Figure DPR3. ARGON+DME

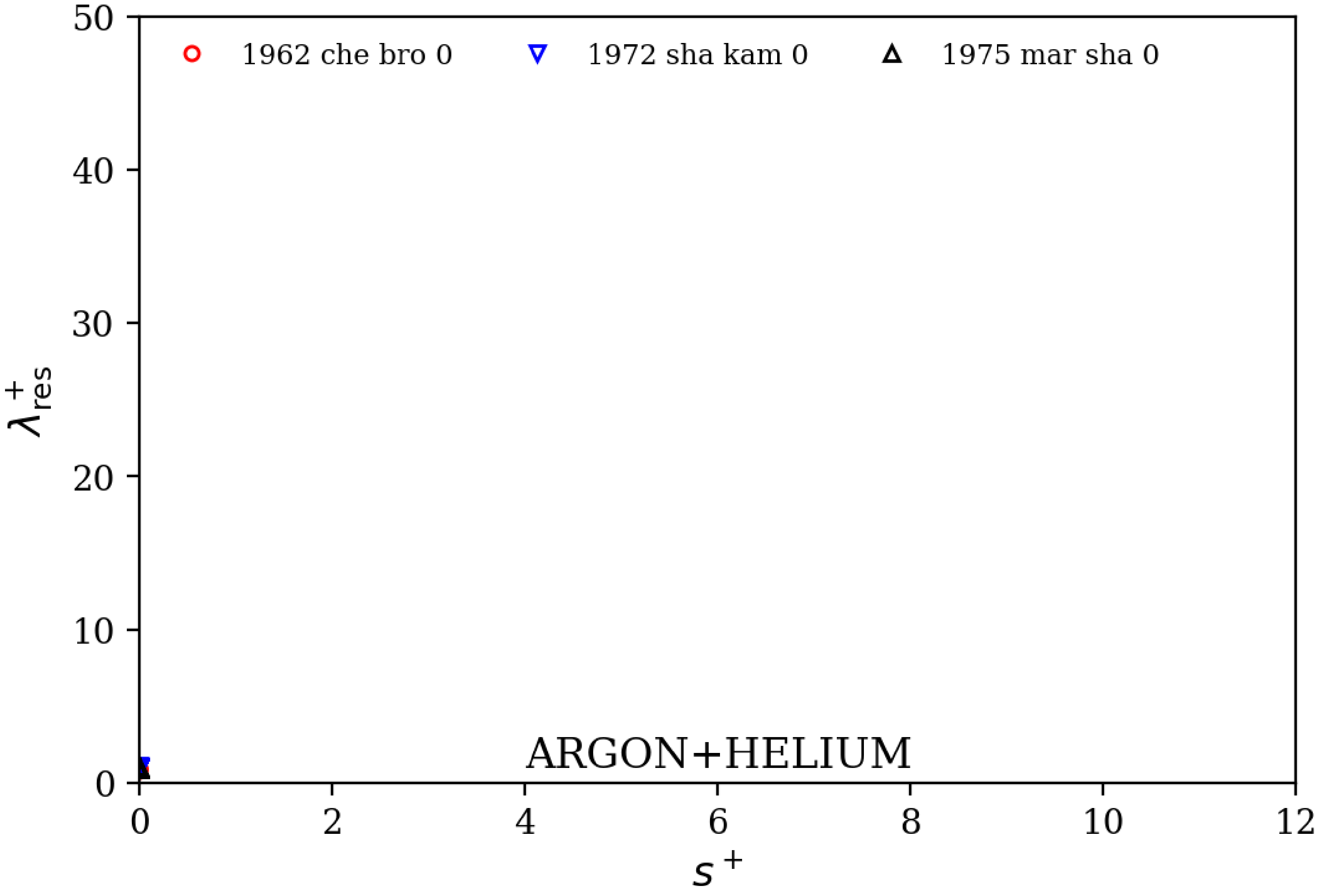

Figure DPR3. ARGON+HELIUM

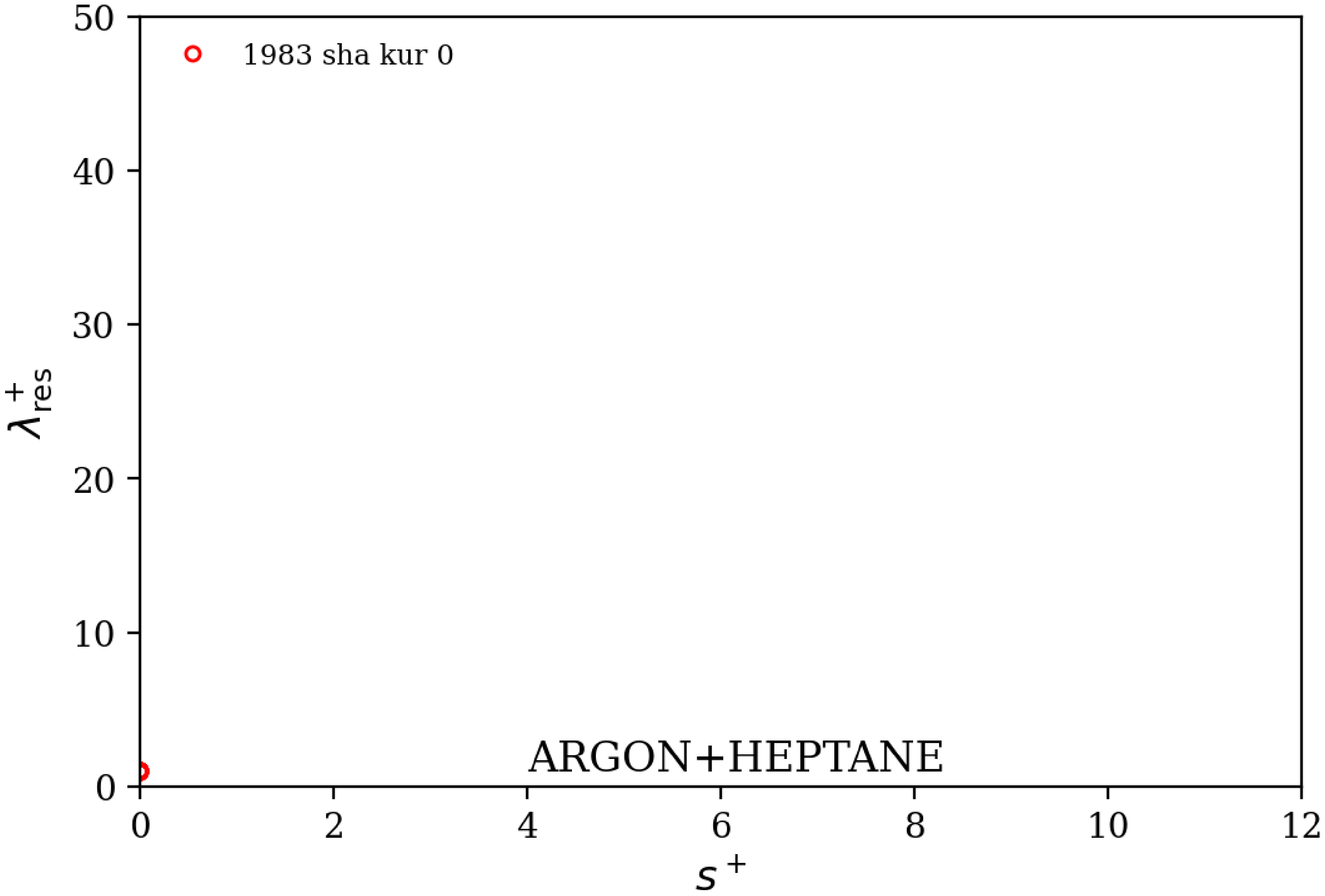

Figure DPR3. ARGON+HEPTANE

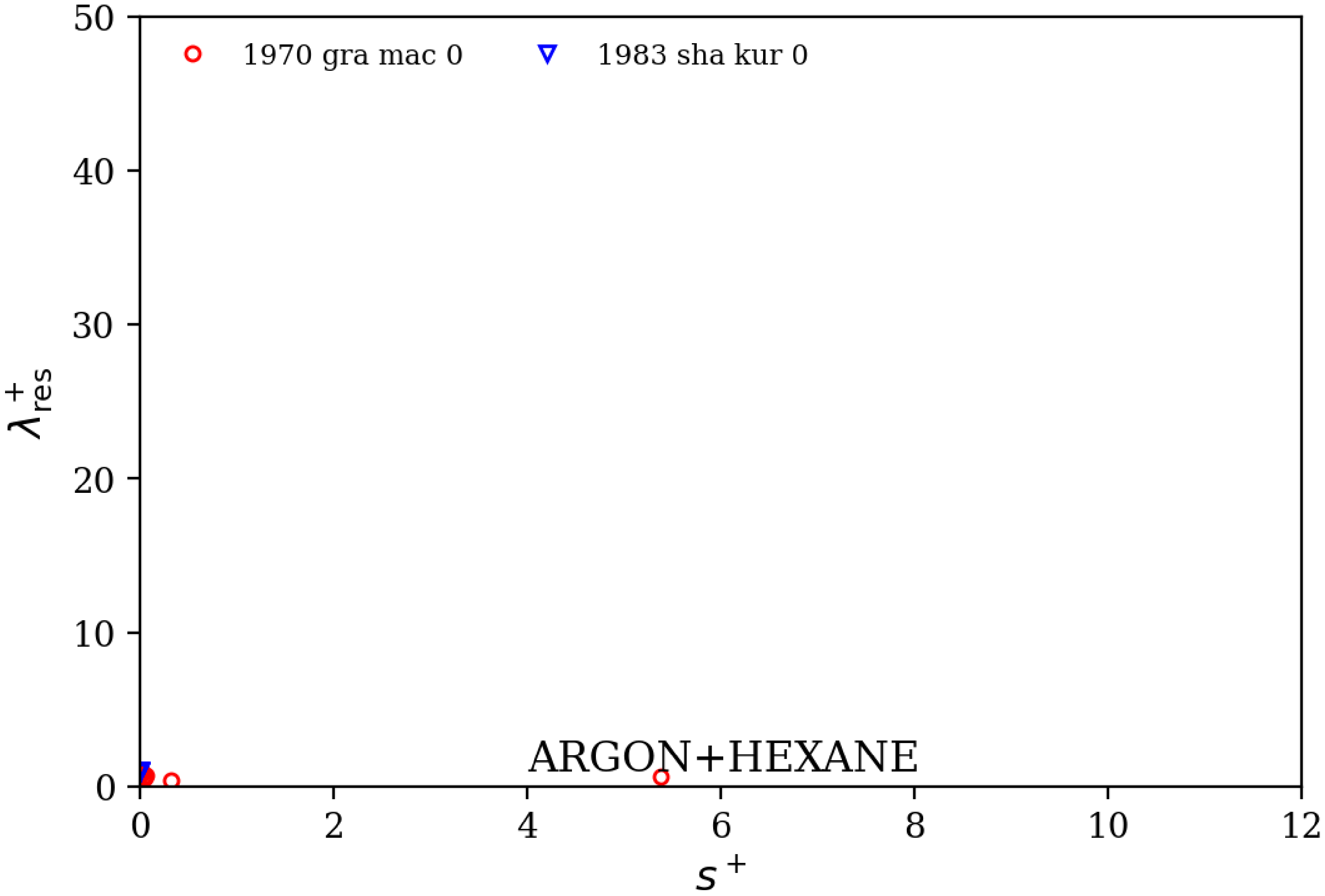

Figure DPR3. ARGON+HEXANE

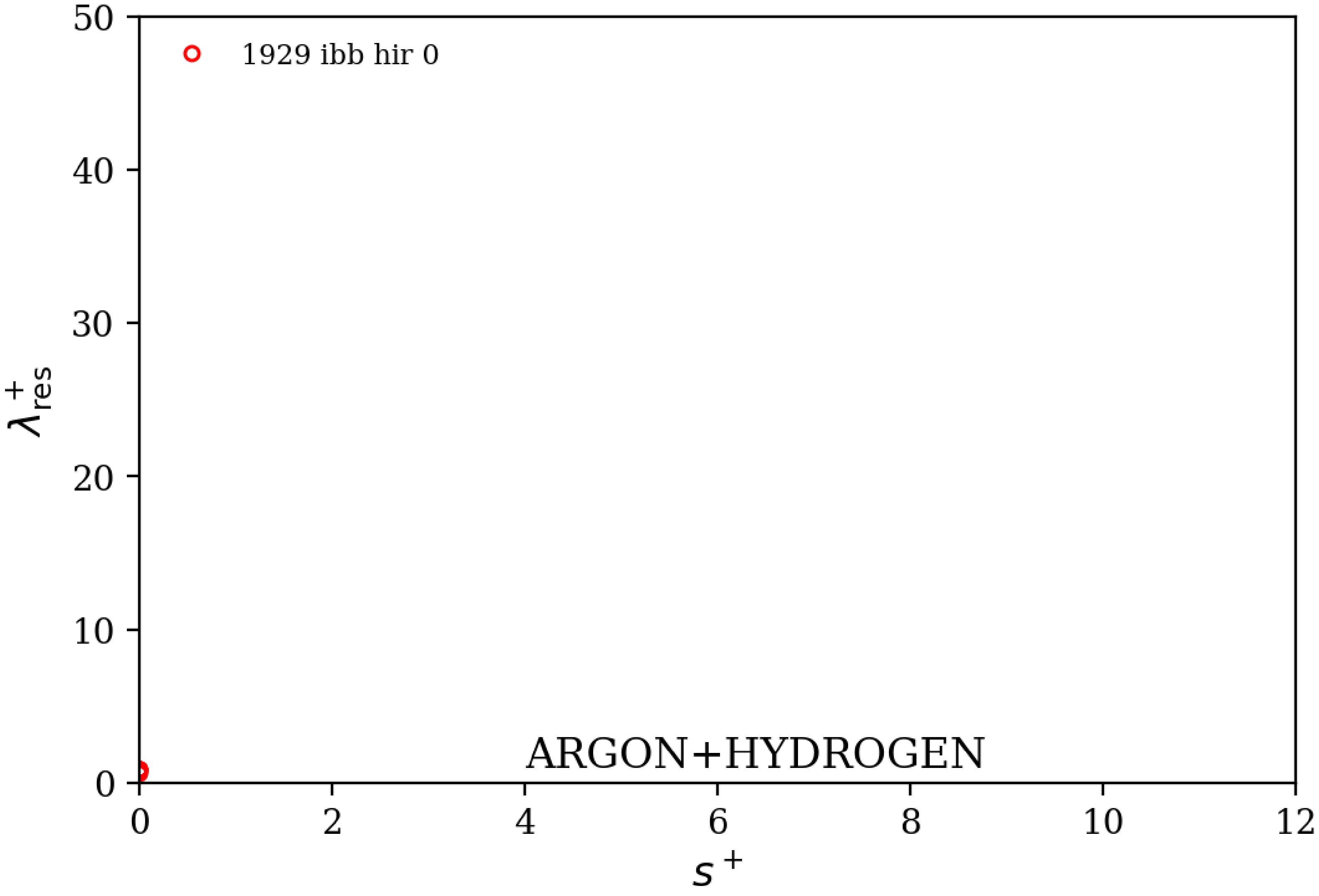

Figure DPR3. ARGON+HYDROGEN

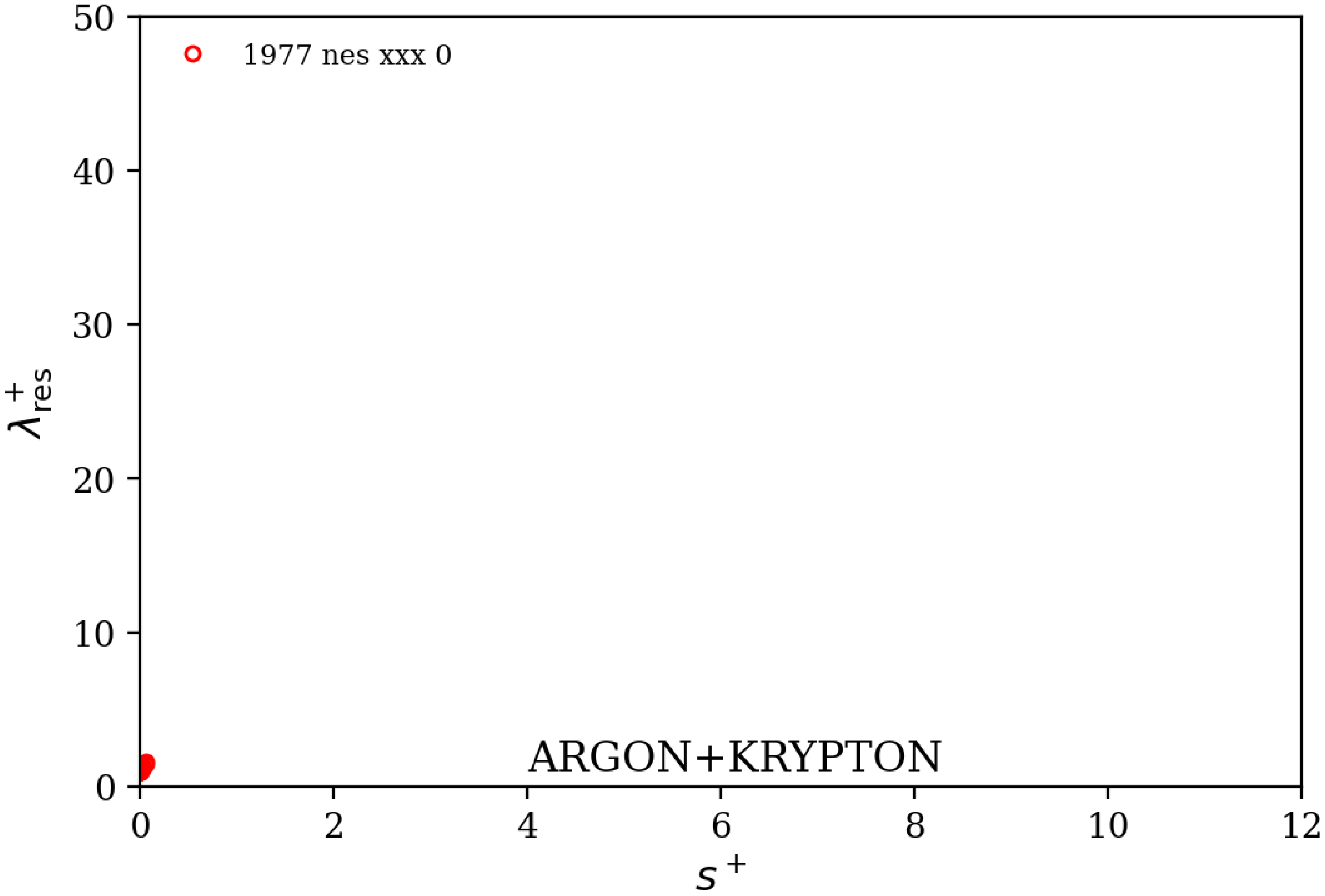

Figure DPR3. ARGON+KRYPTON

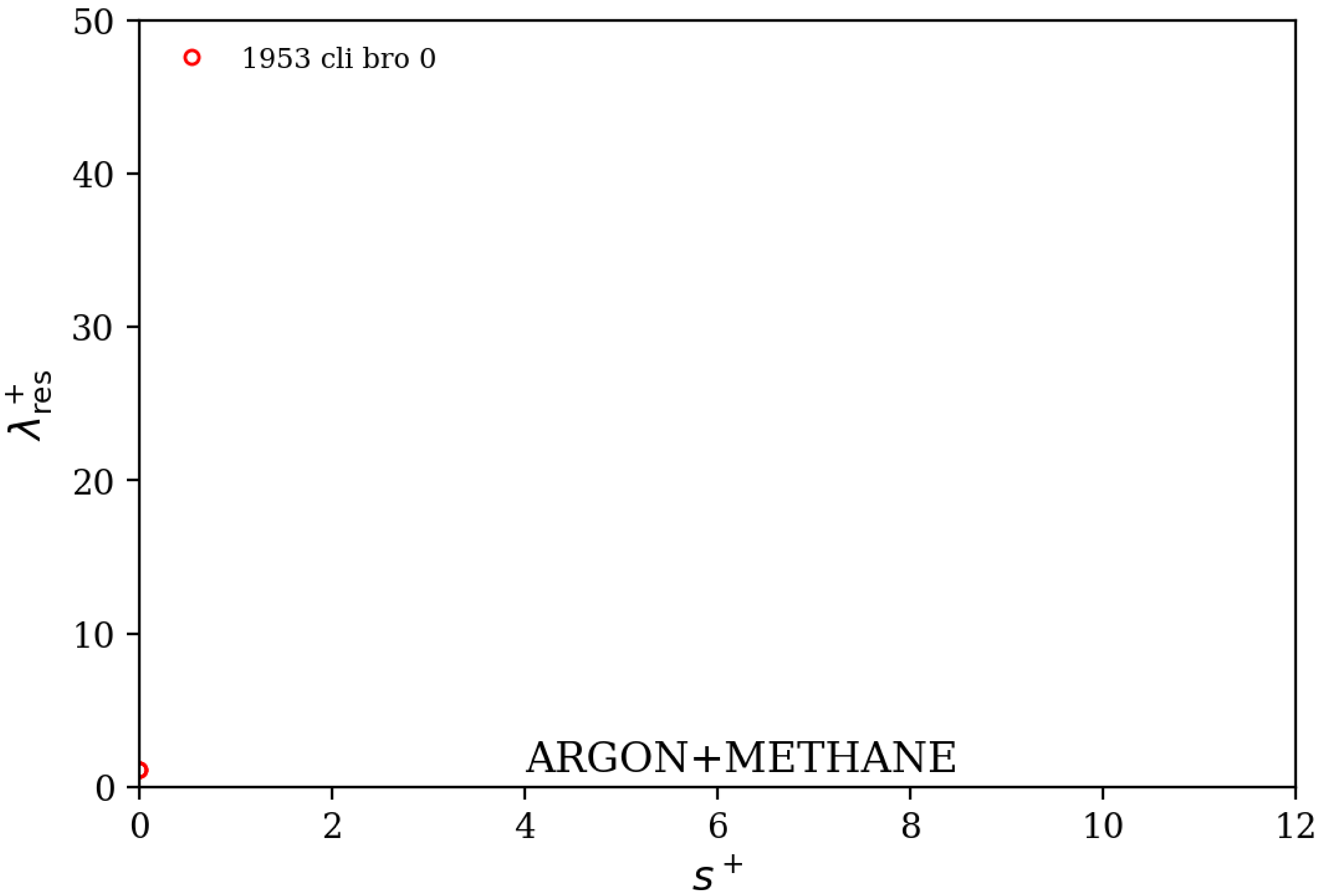

Figure DPR3. ARGON+METHANE

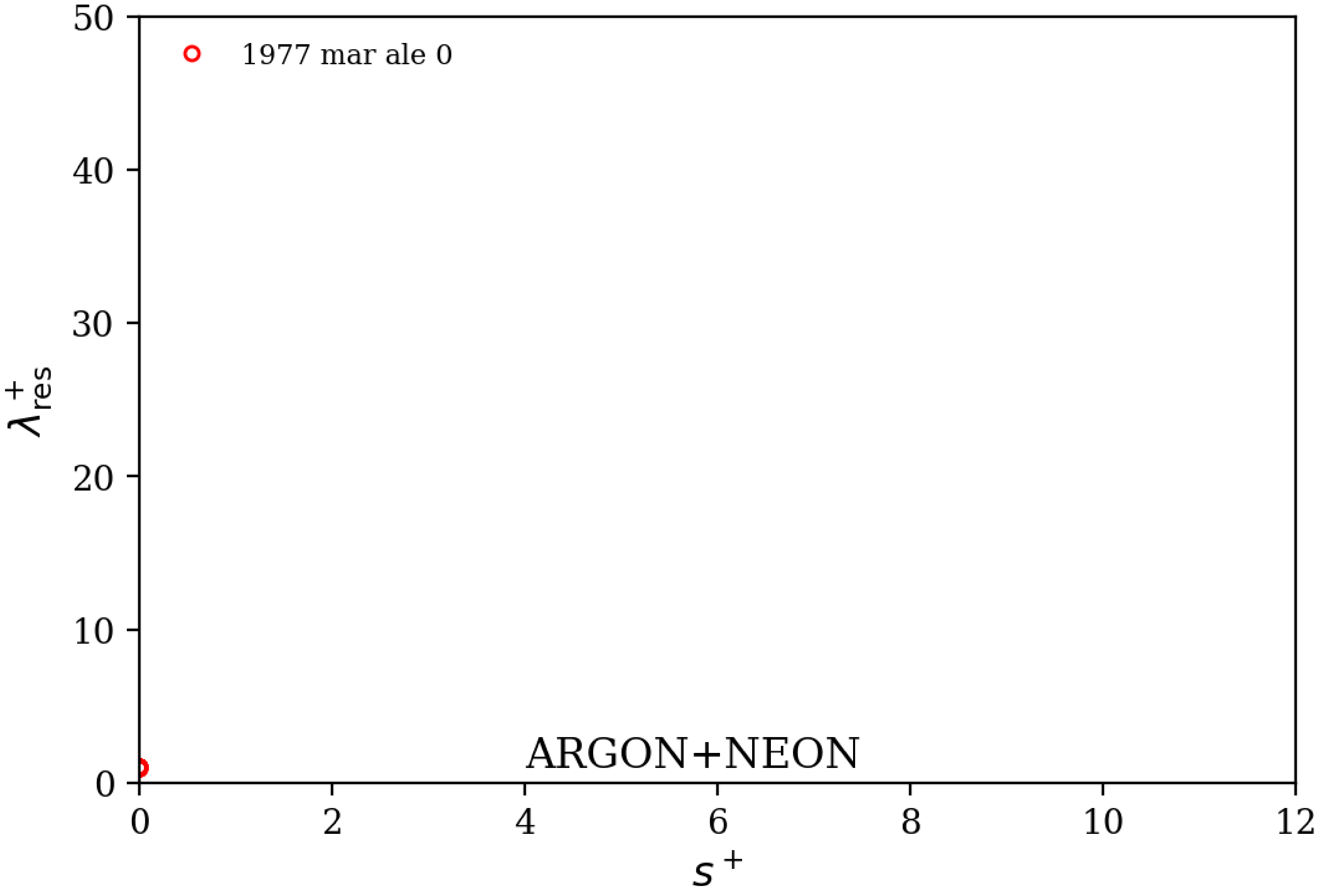

Figure DPR3. ARGON+NEON

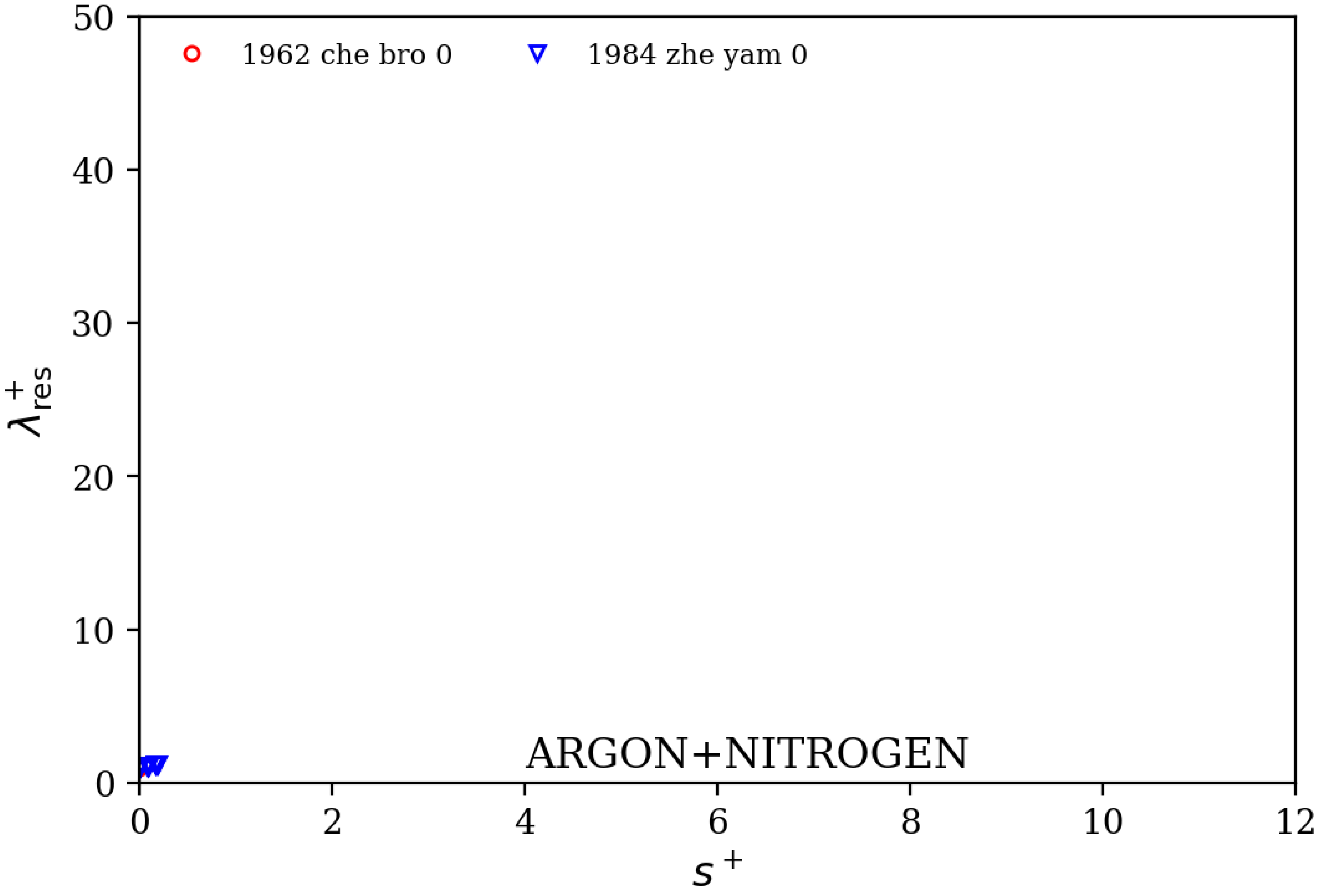

Figure DPR3. ARGON+NITROGEN

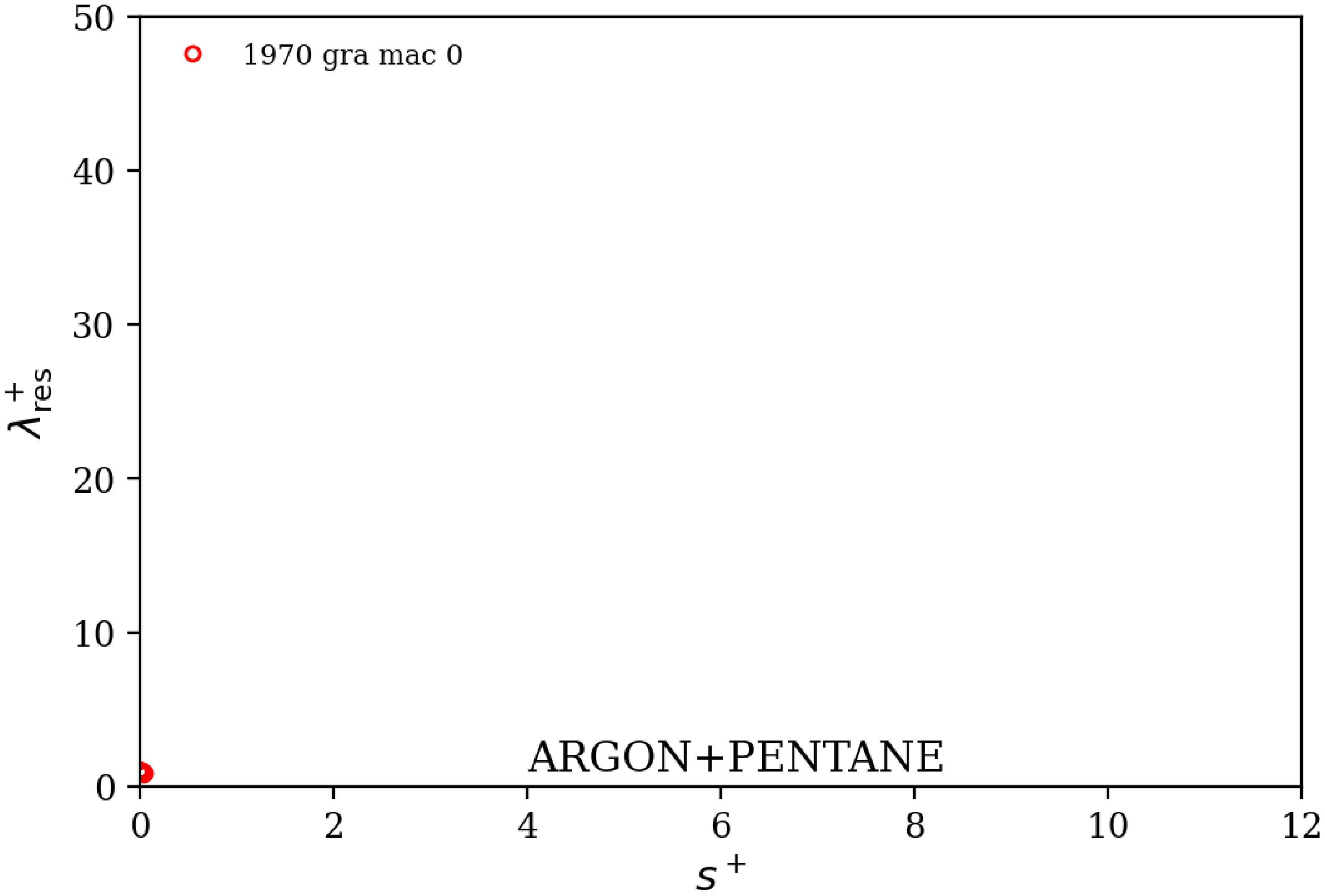

Figure DPR3. ARGON+PENTANE

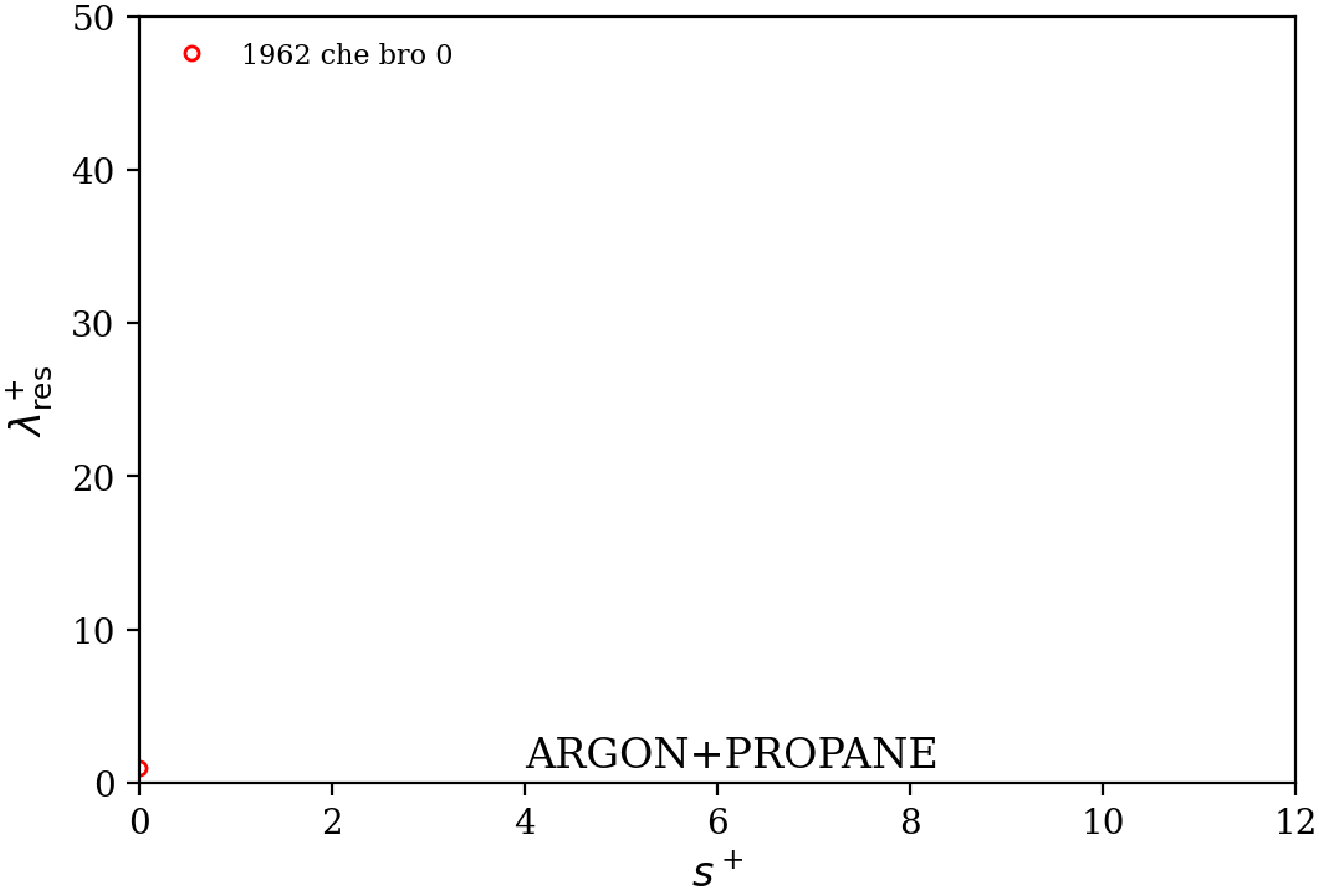

Figure DPR3. ARGON+PROPANE

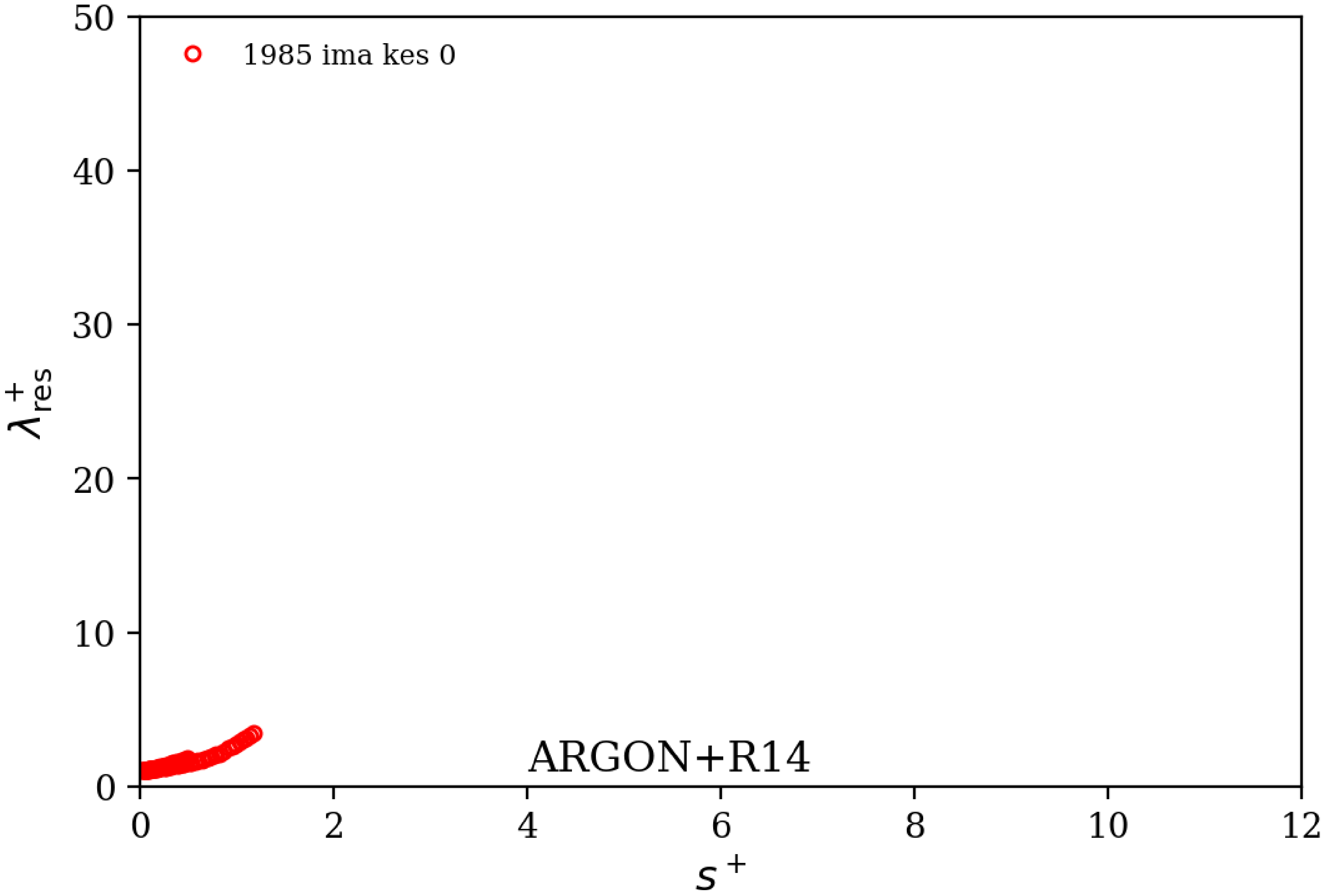

Figure DPR3. ARGON+R14

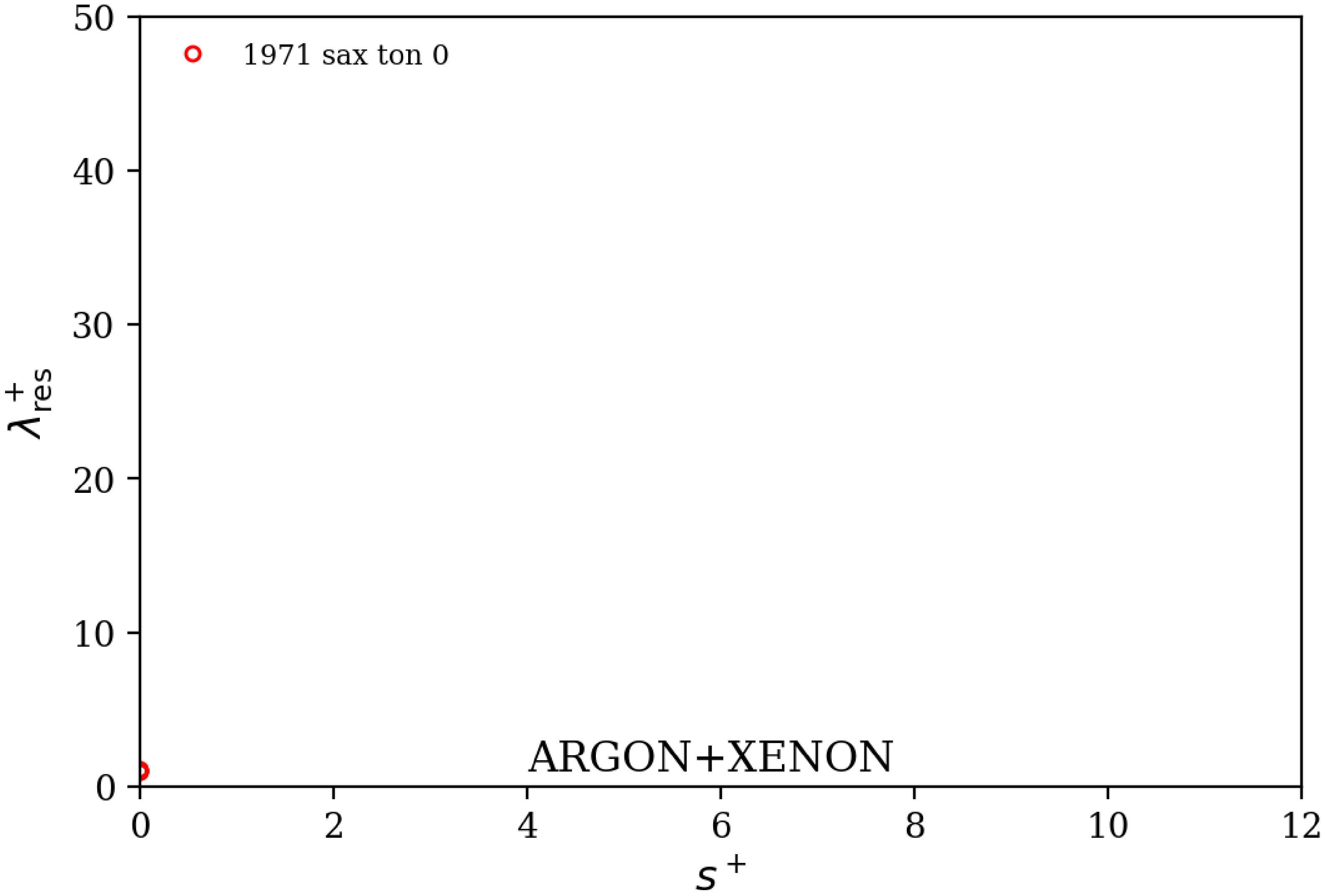

Figure DPR3. ARGON+XENON

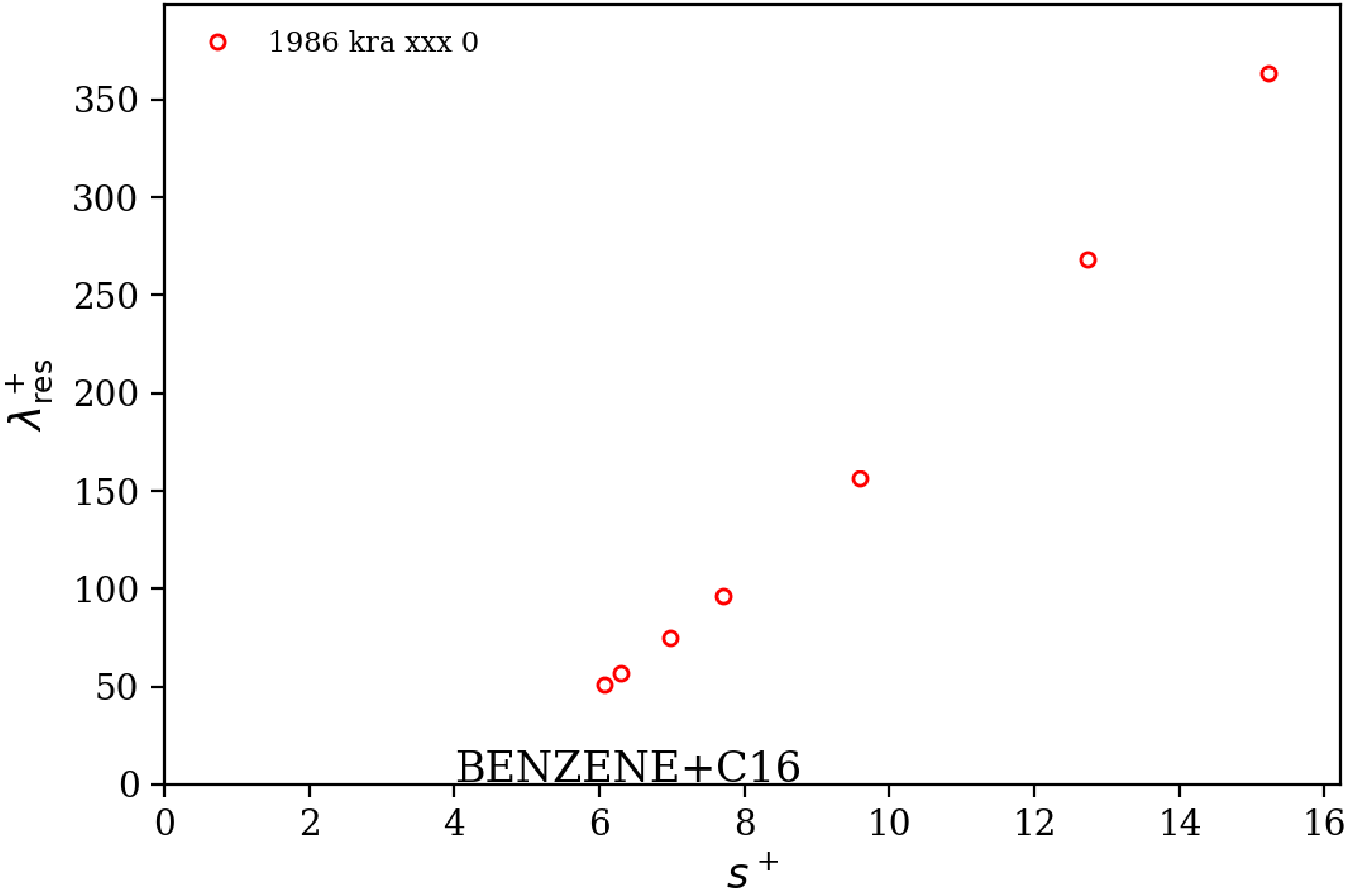

Figure DPR3. BENZENE+C16

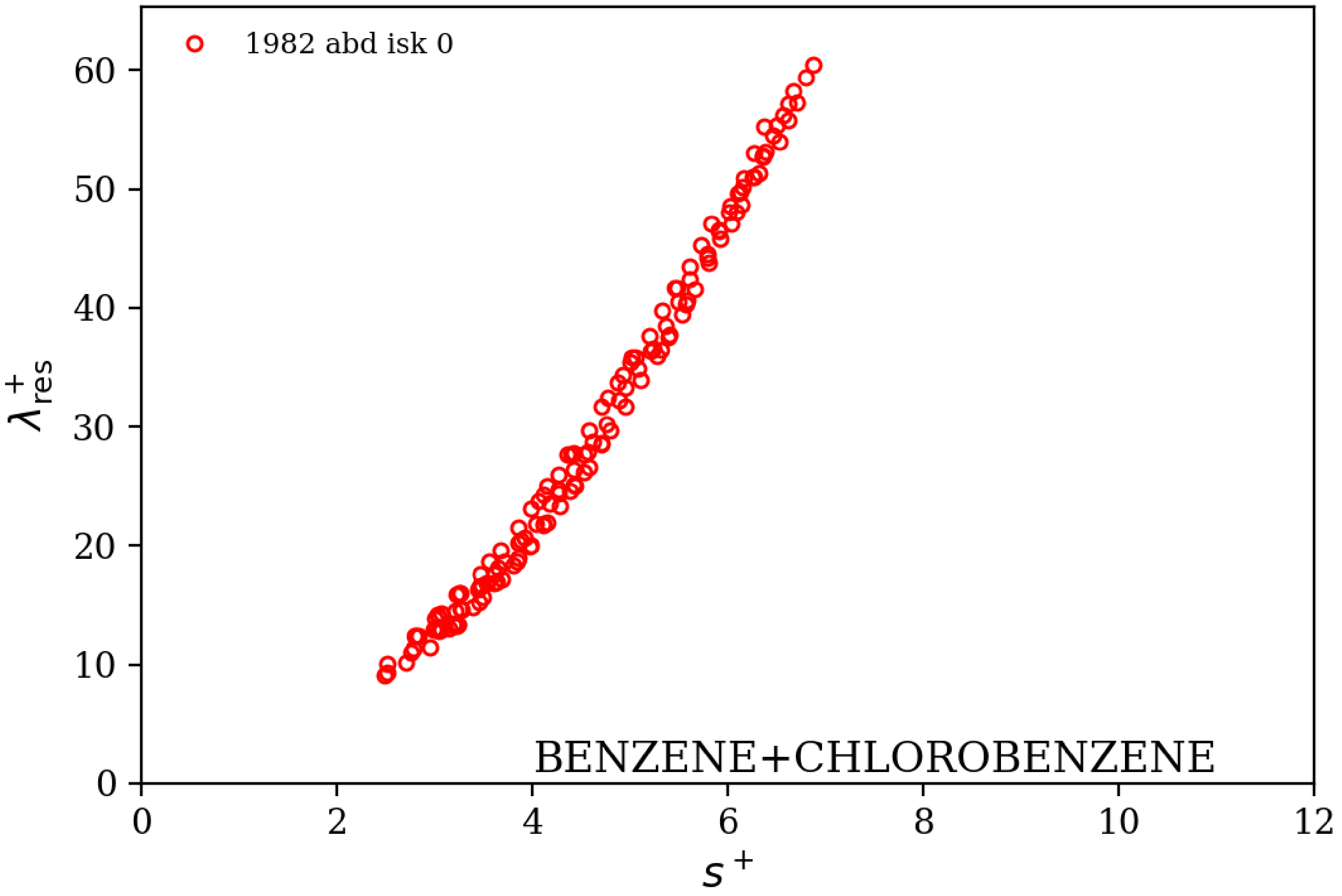

Figure DPR3. BENZENE+CHLOROBENZENE

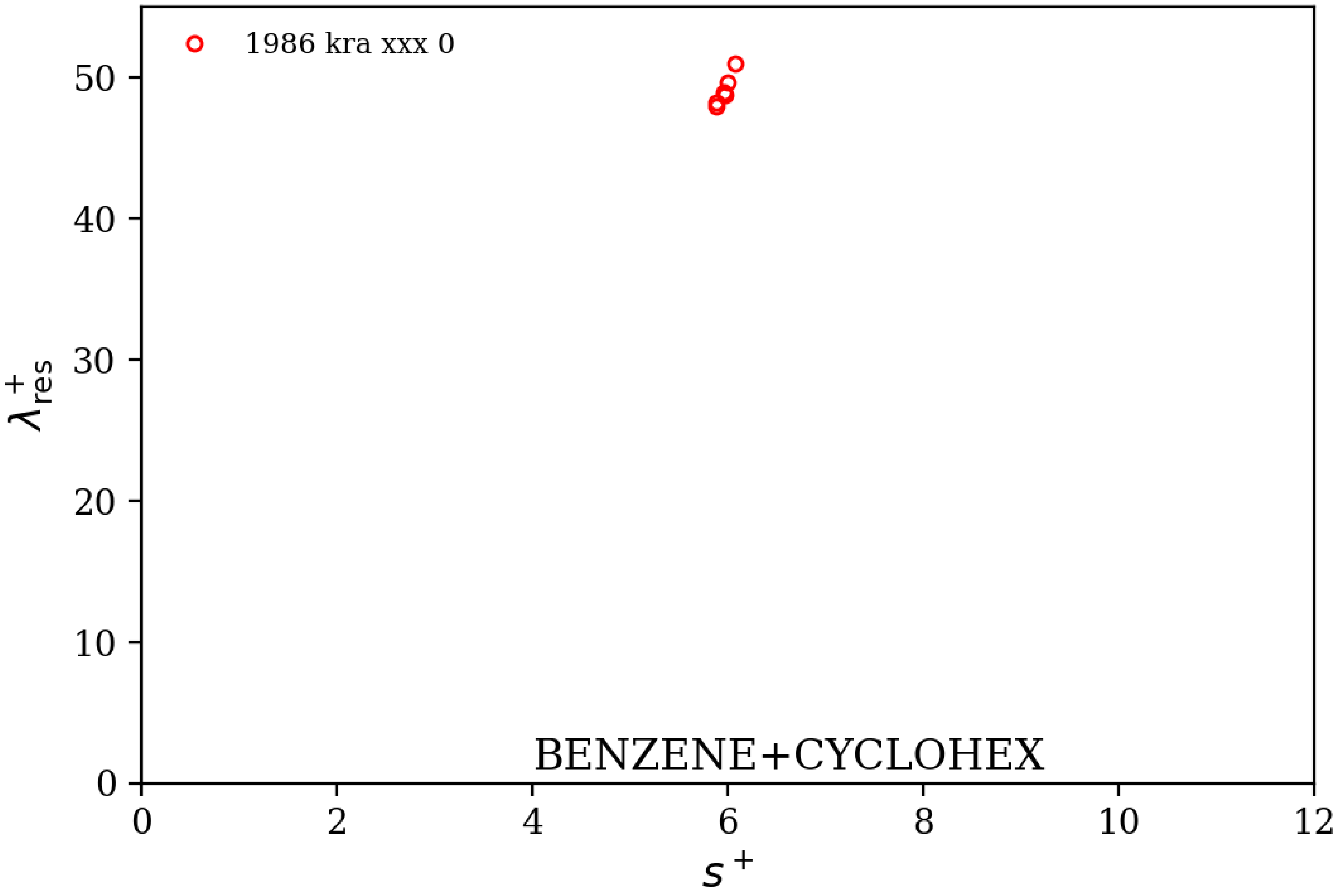

Figure DPR3. BENZENE+CYCLOHEX

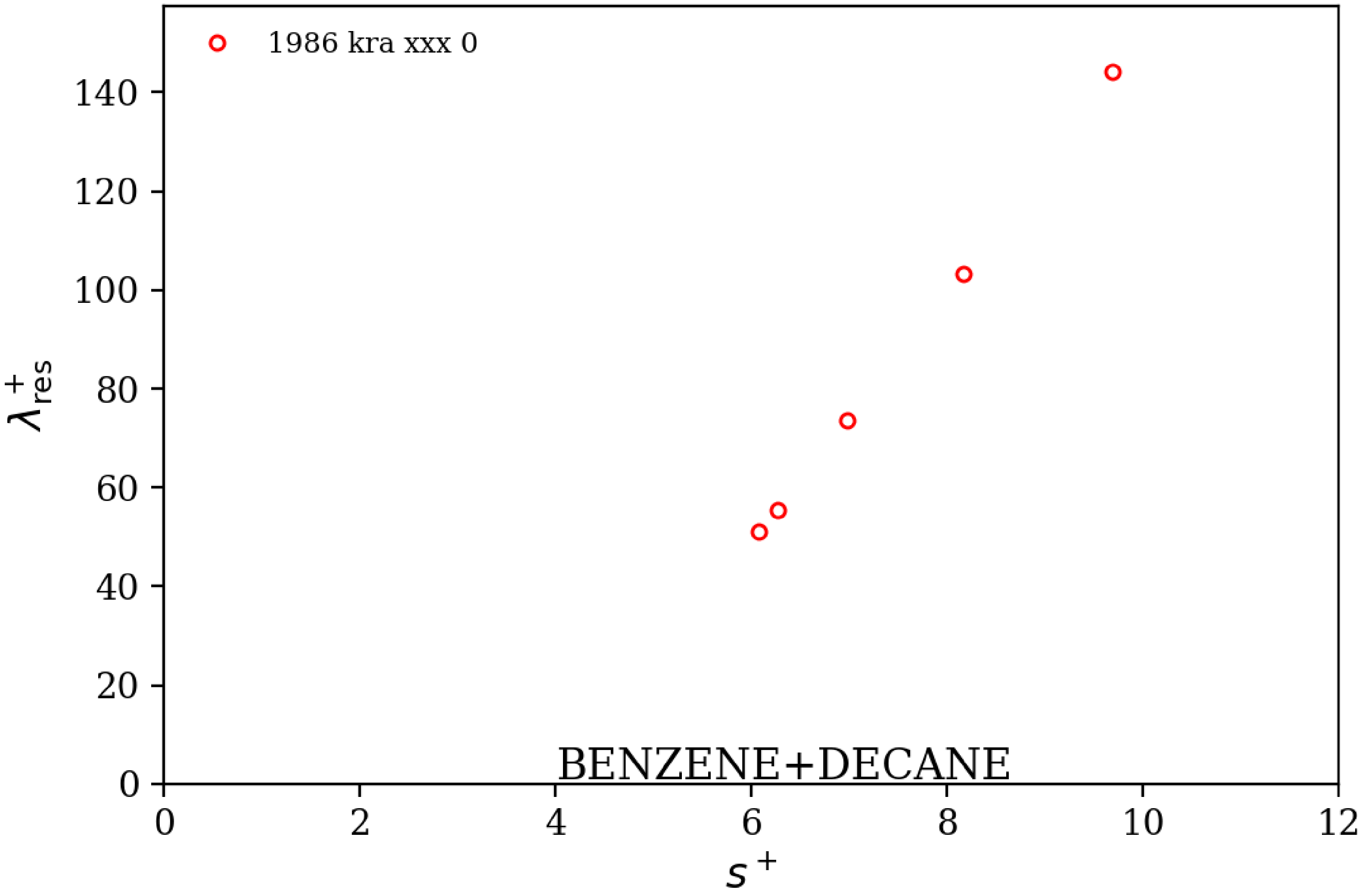

Figure DPR3. BENZENE+DECANE

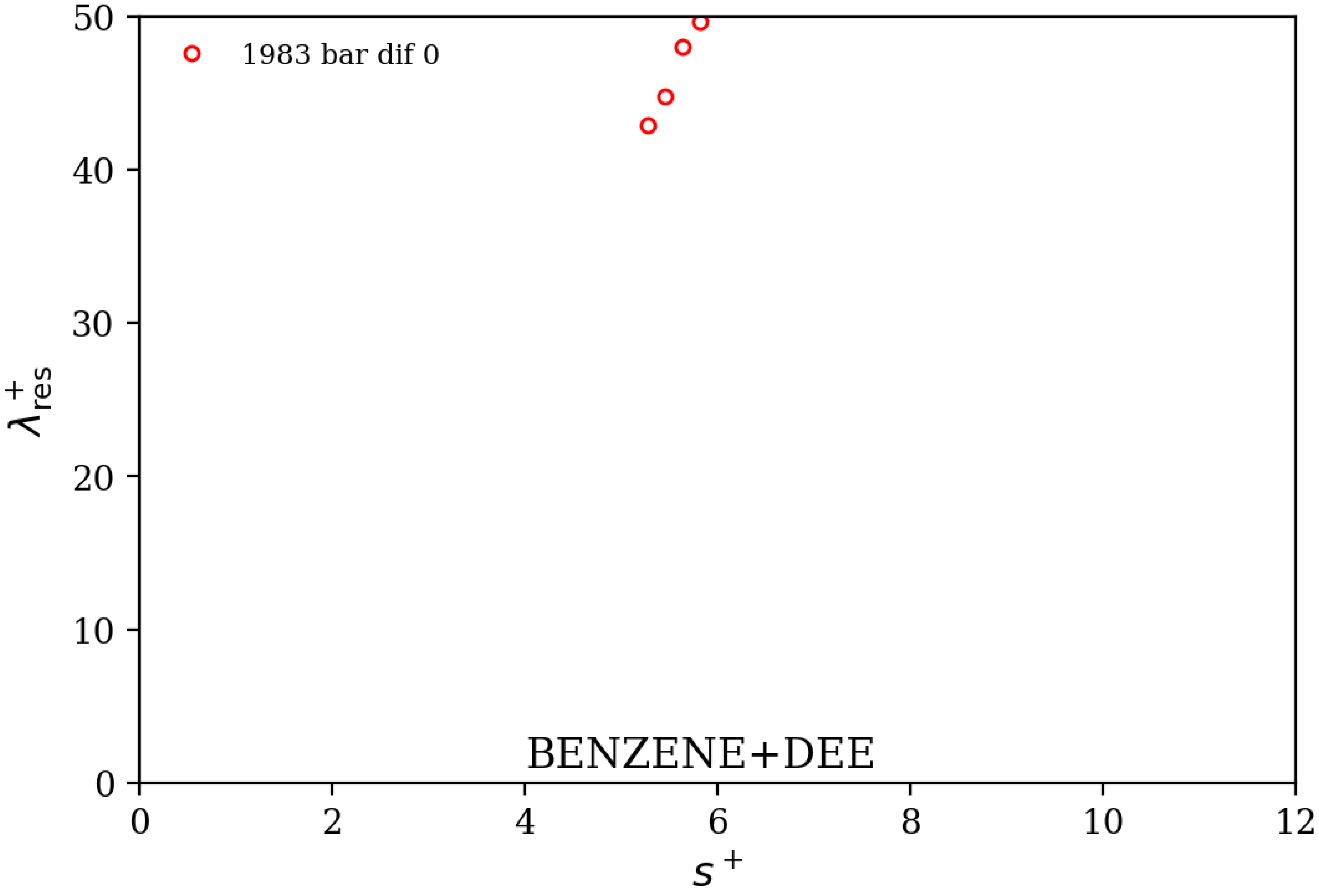

Figure DPR3. BENZENE+DEE

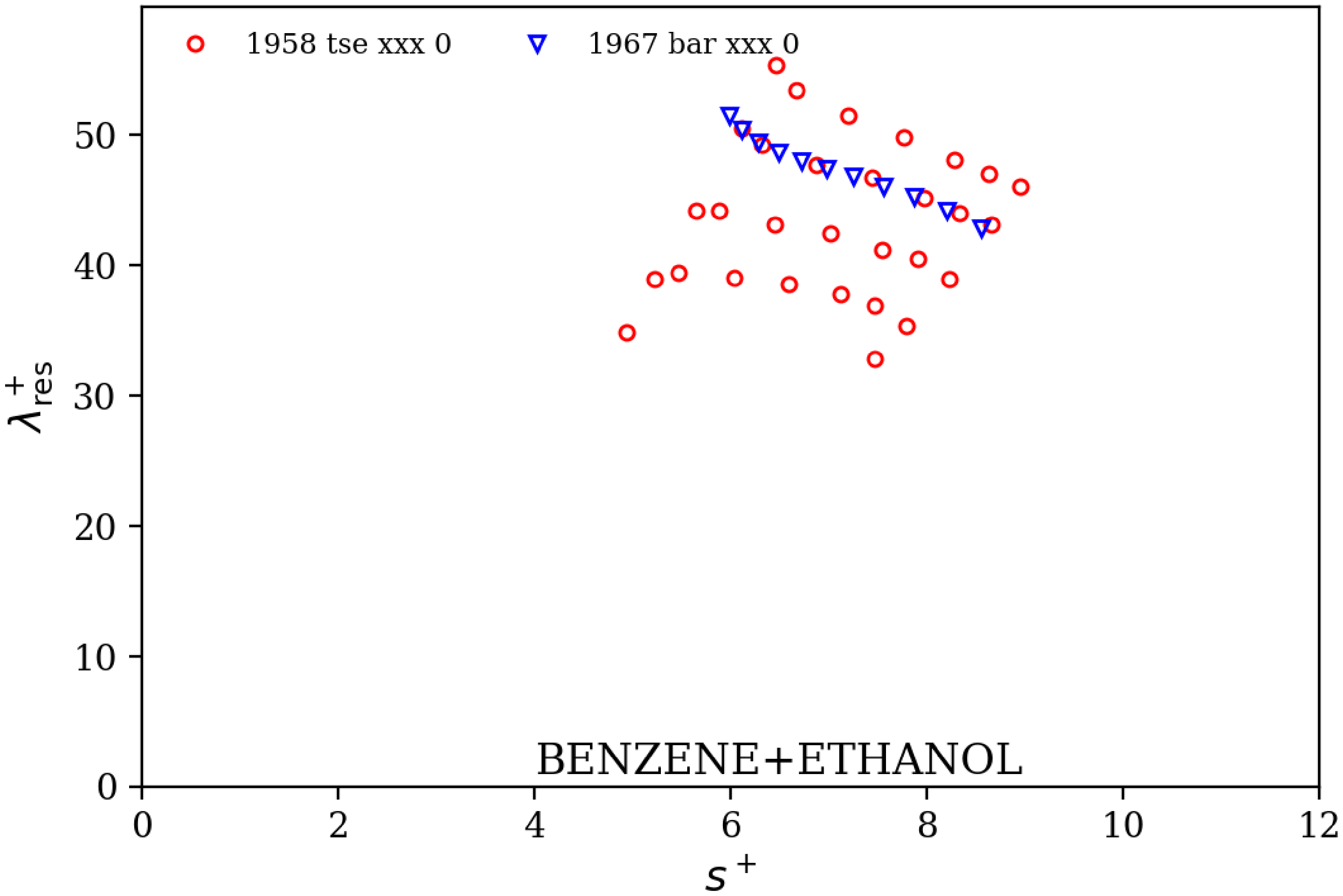

Figure DPR3. BENZENE+ETHANOL

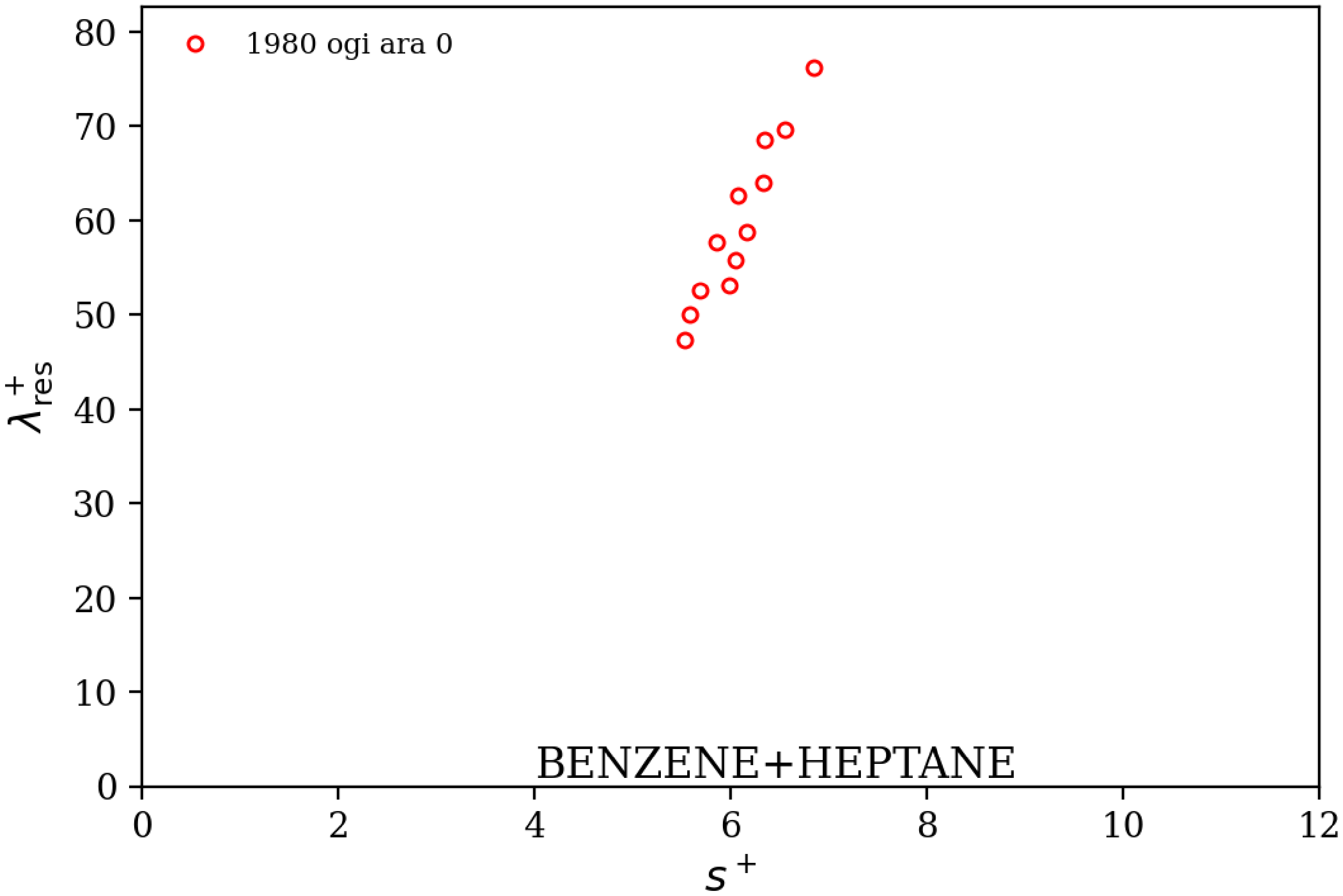

Figure DPR3. BENZENE+HEPTANE

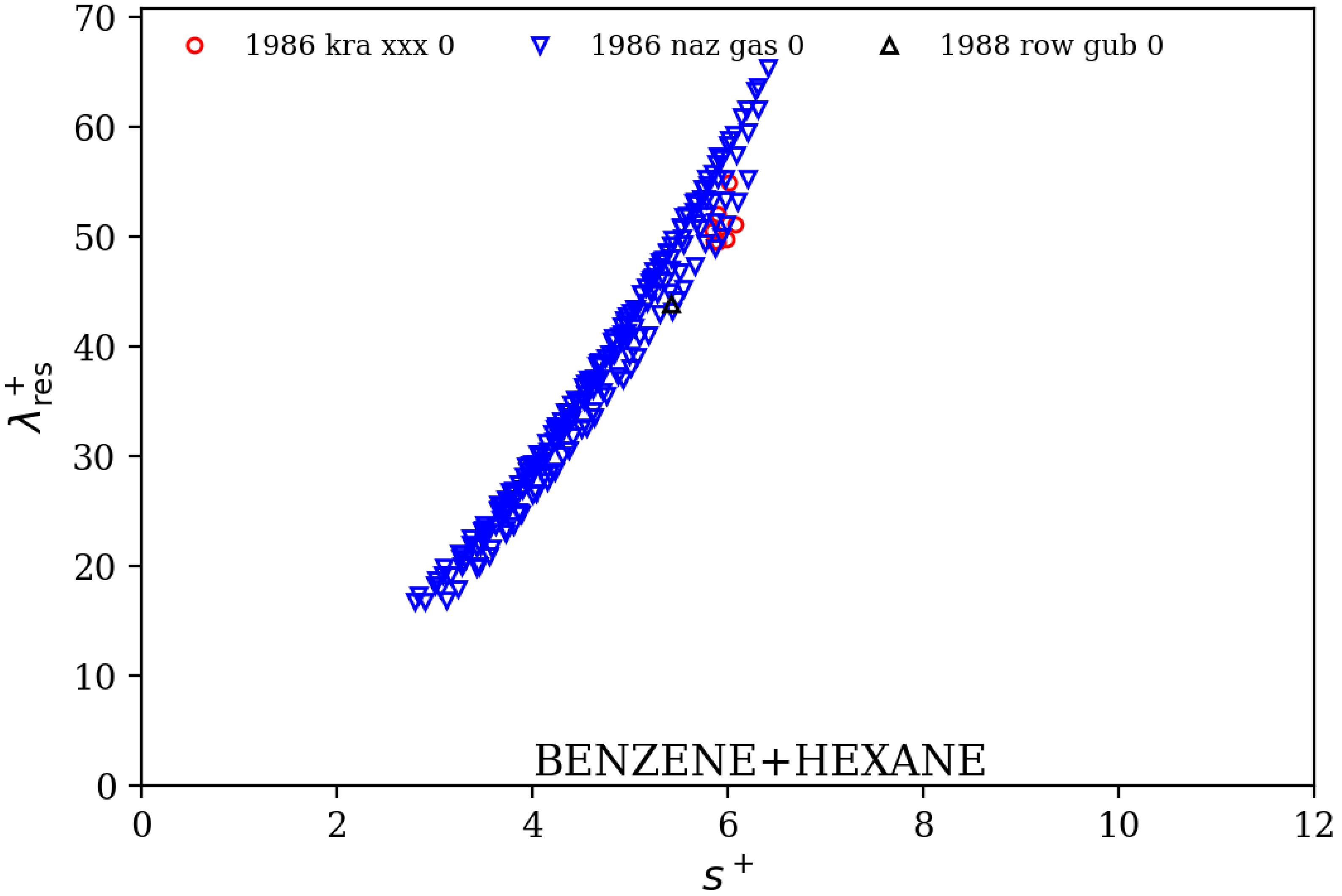

Figure DPR3. BENZENE+HEXANE

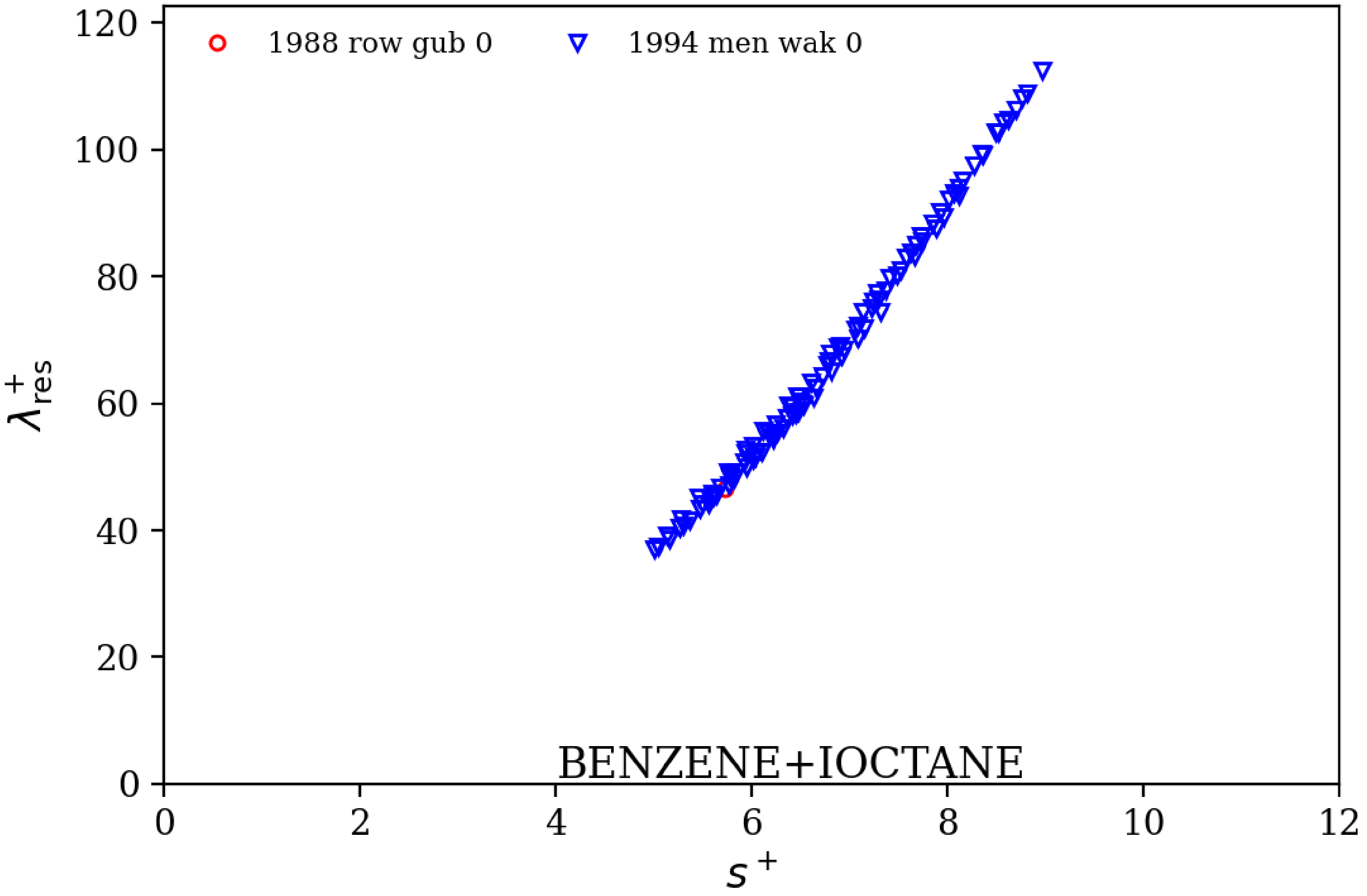

Figure DPR3. BENZENE+IOCTANE

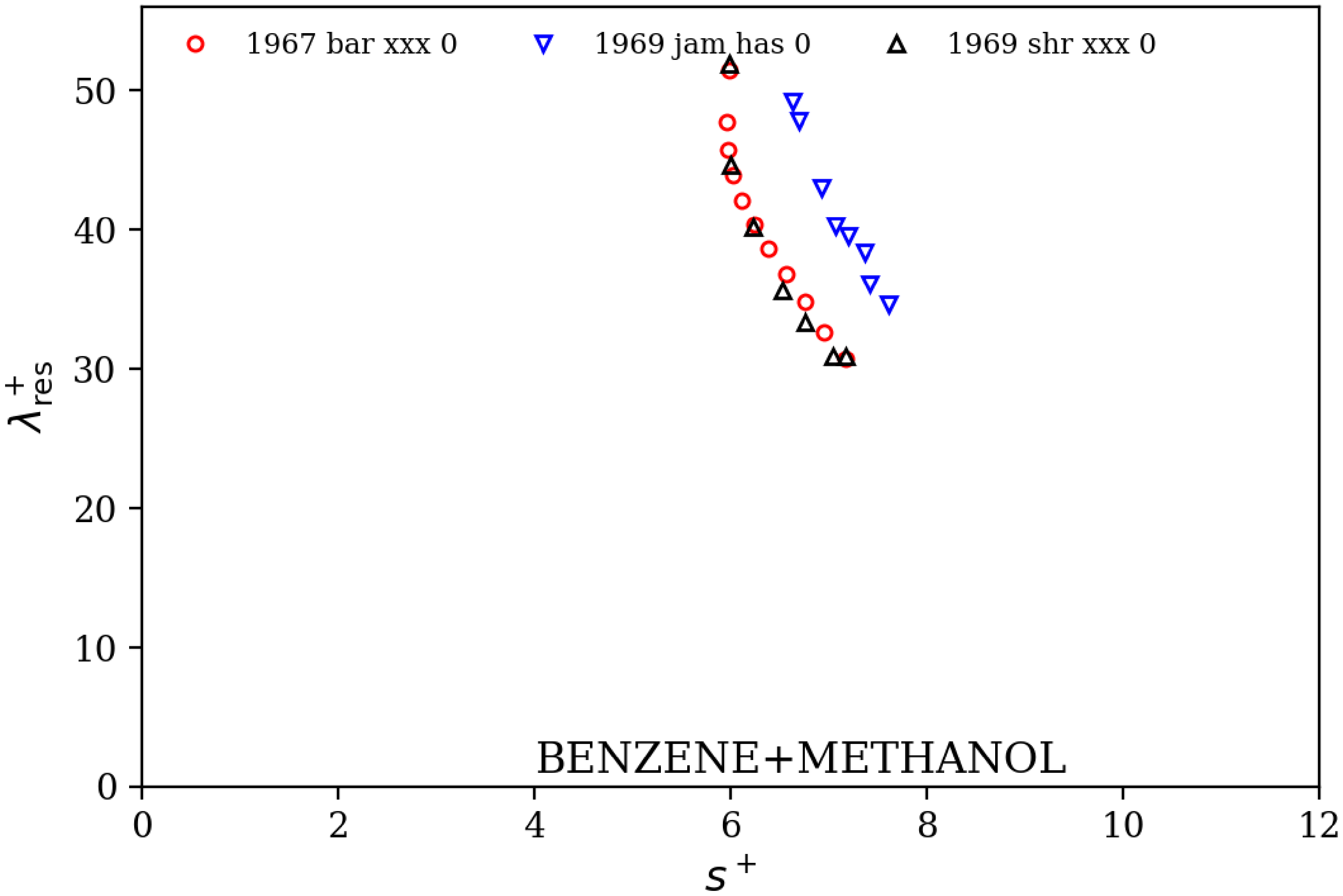

Figure DPR3. BENZENE+METHANOL

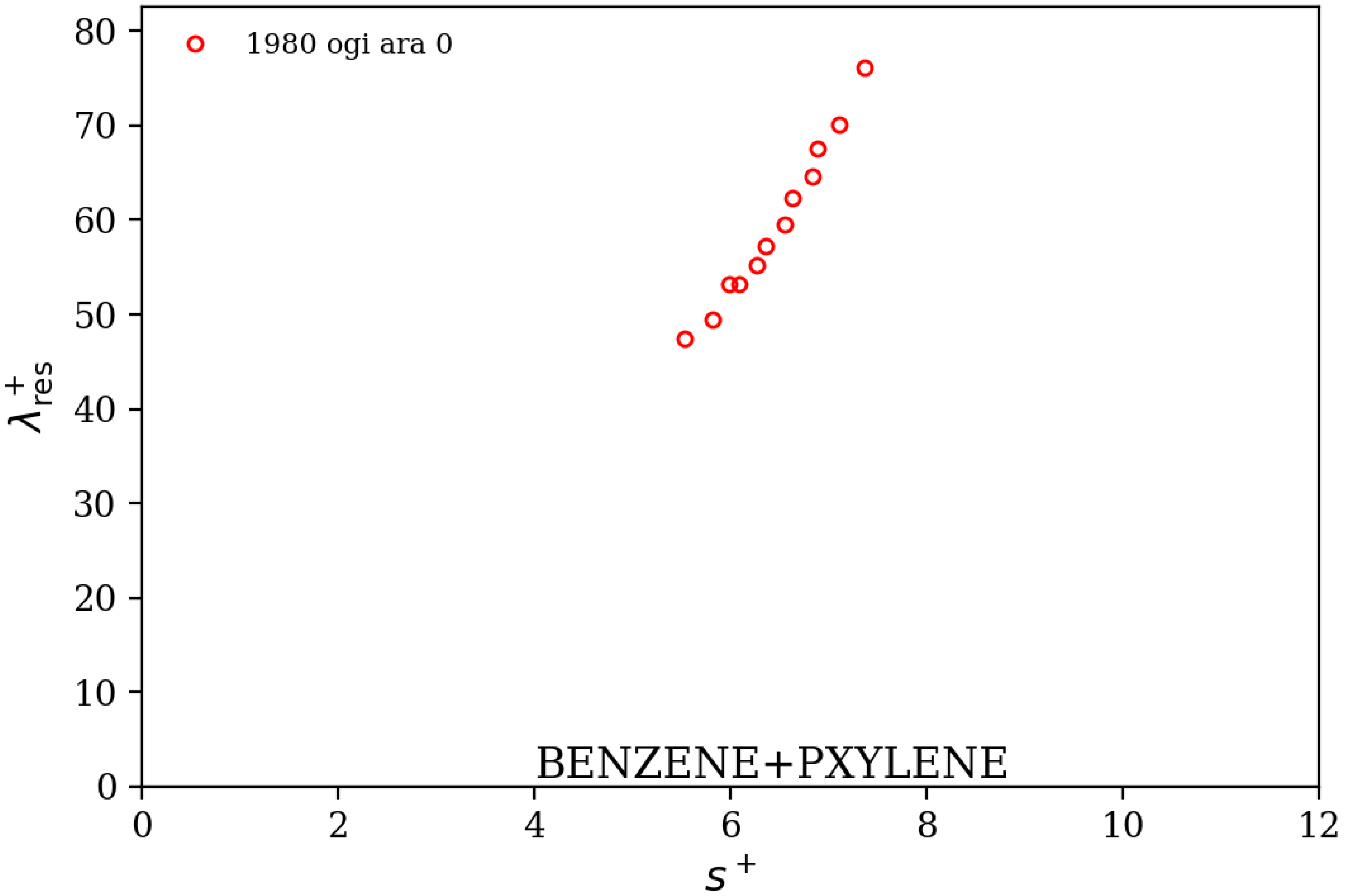

Figure DPR3. BENZENE+PXYLENE

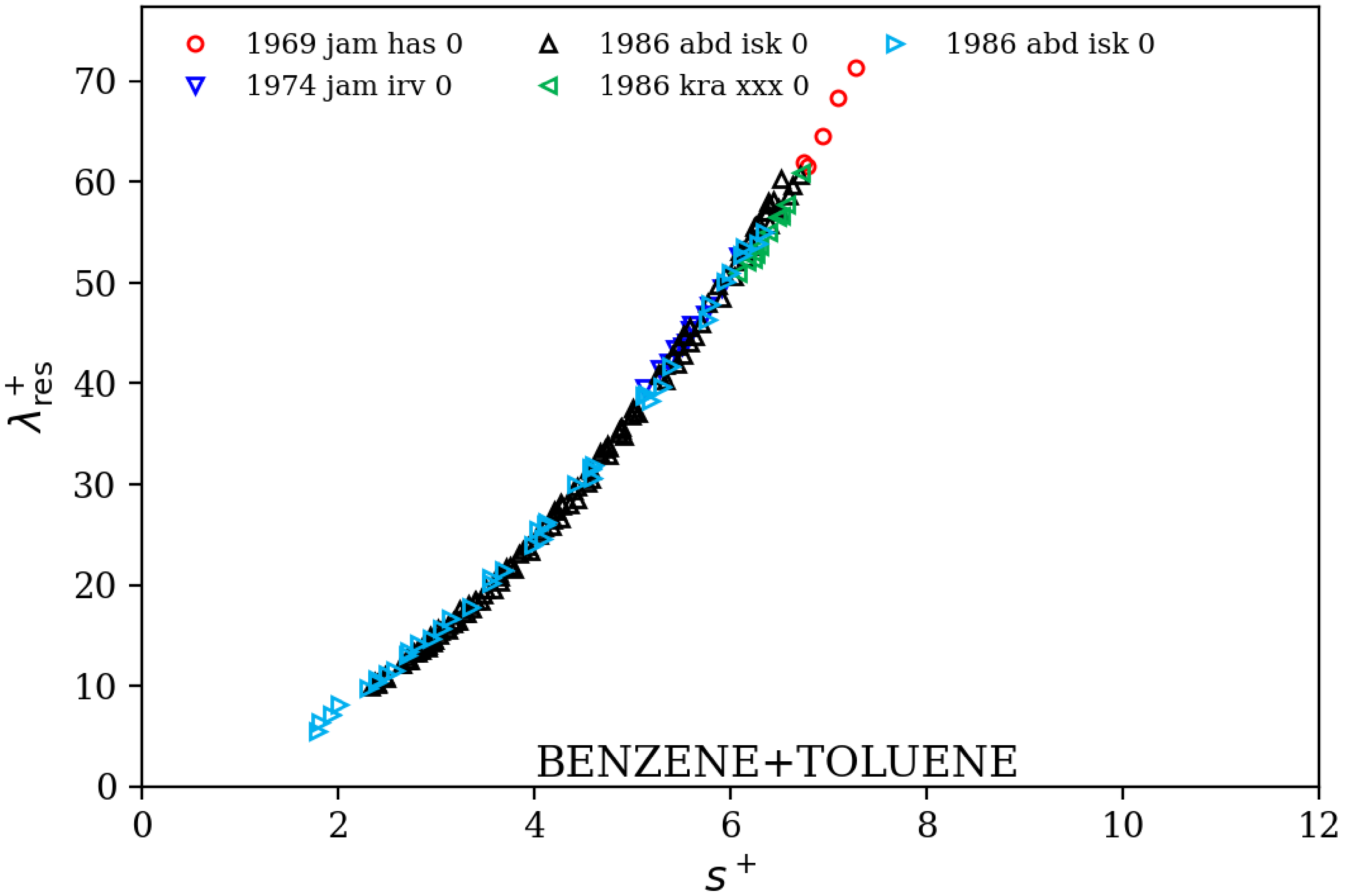

Figure DPR3. BENZENE+TOLUENE

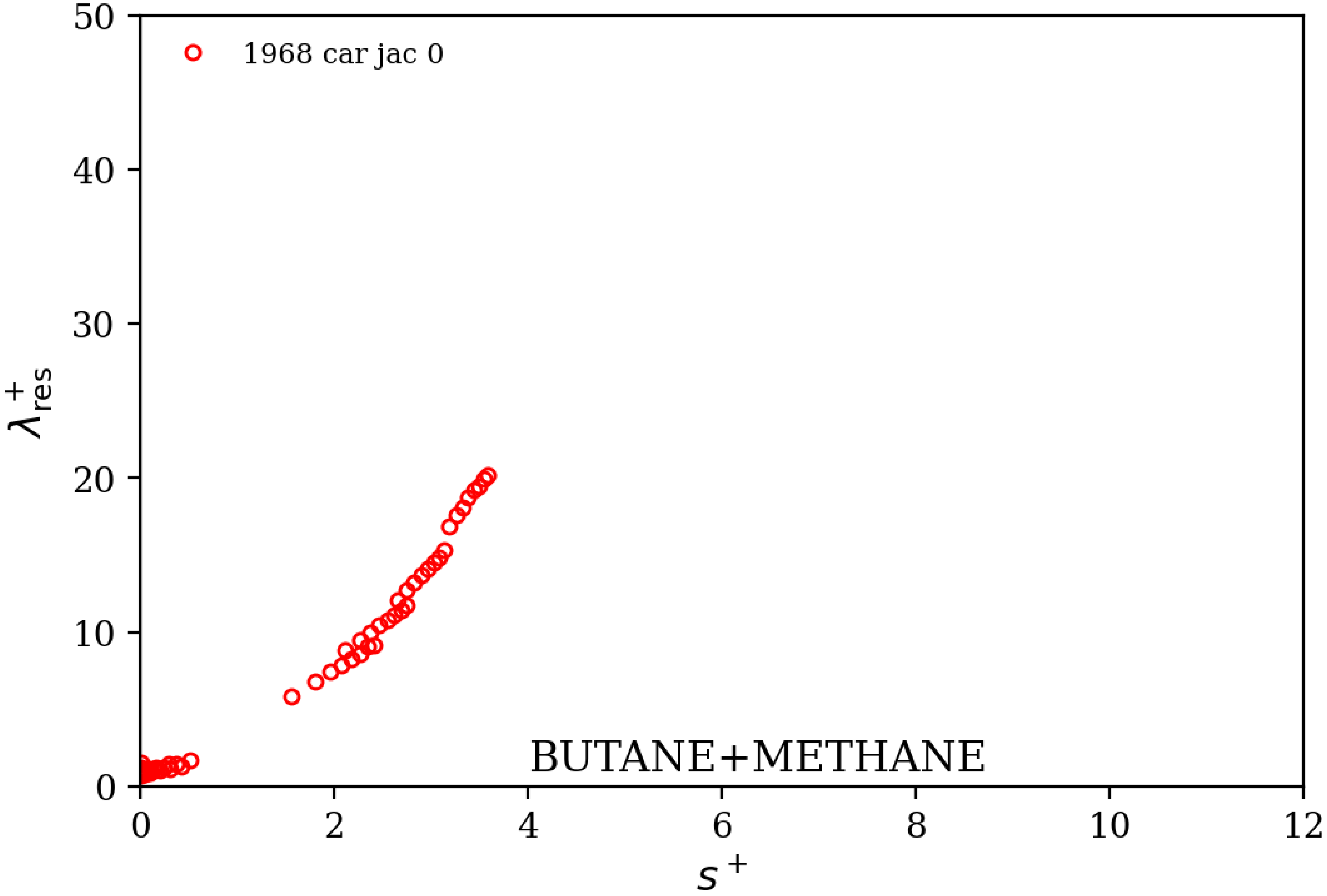

Figure DPR3. BUTANE+METHANE

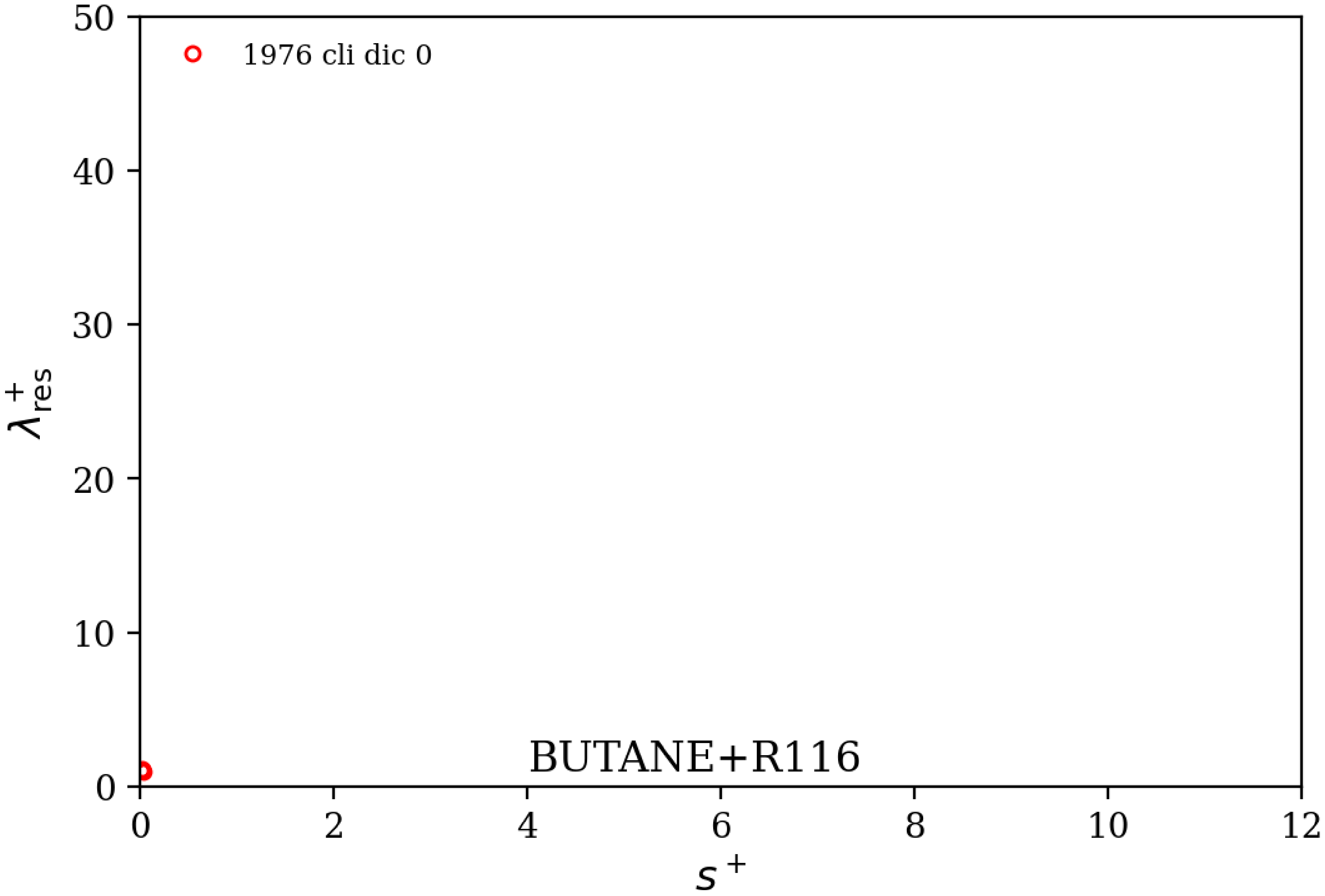

Figure DPR3. BUTANE+R116

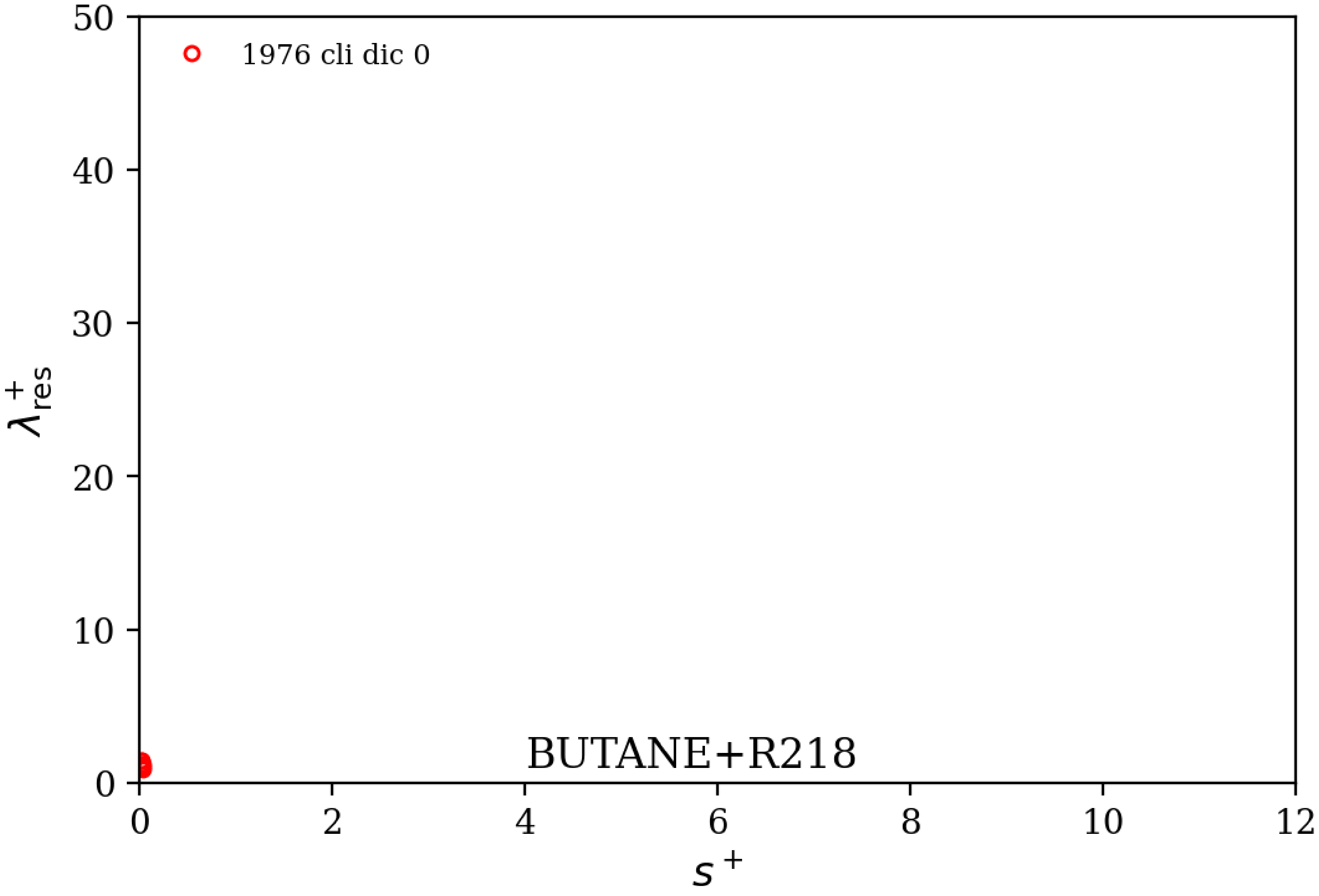

Figure DPR3. BUTANE+R218

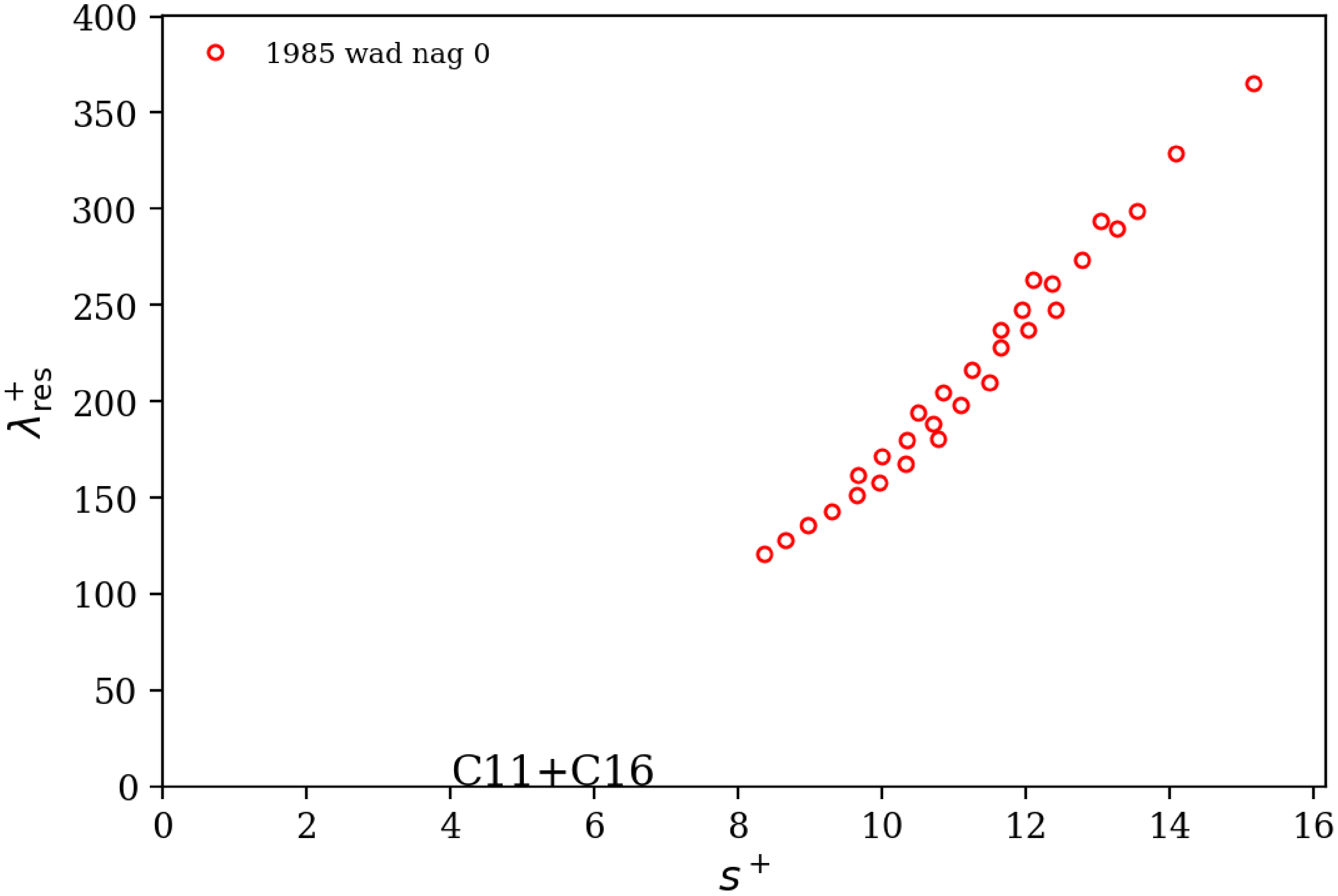

Figure DPR3. C11+C16

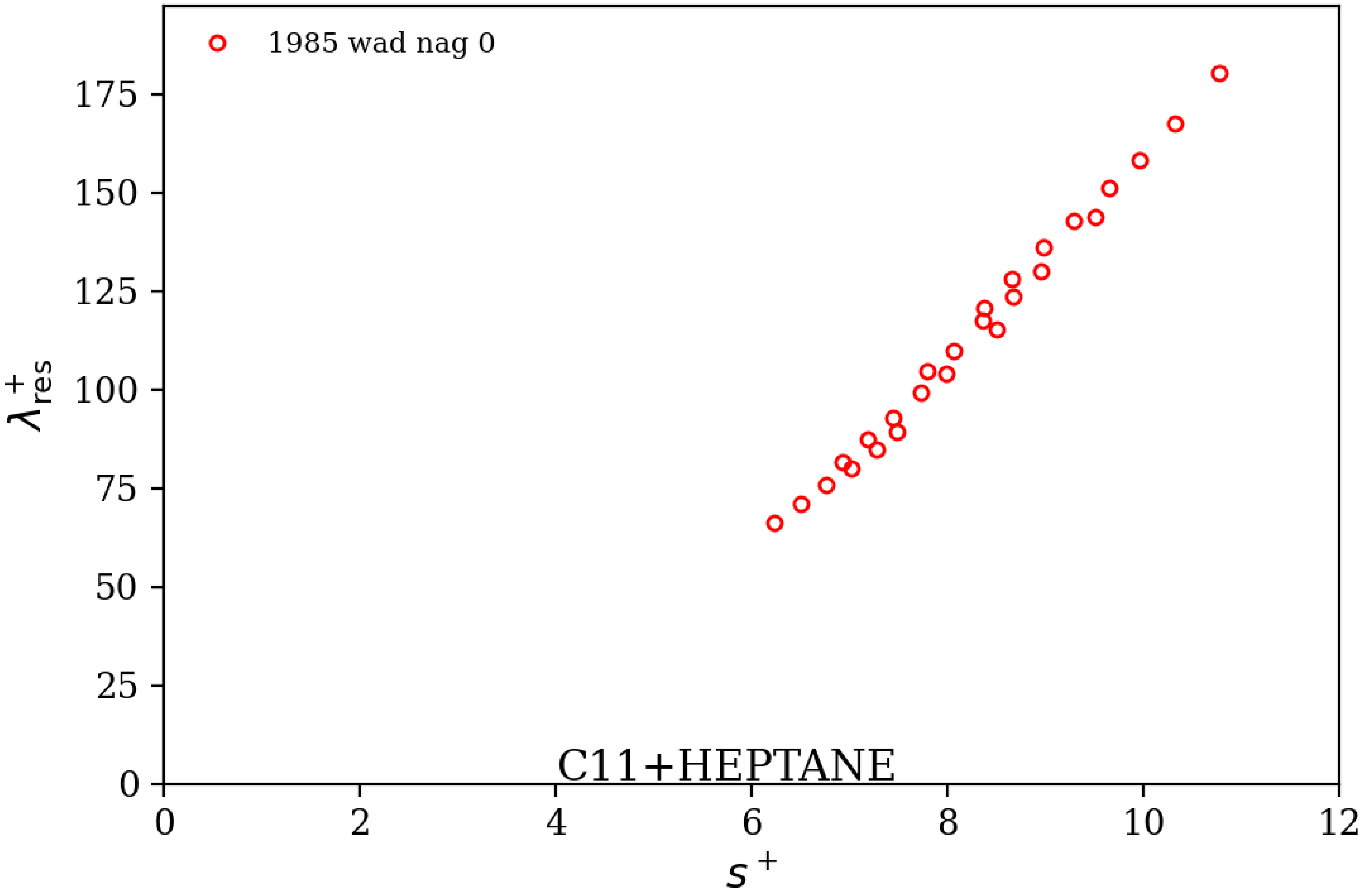

Figure DPR3. C11+HEPTANE

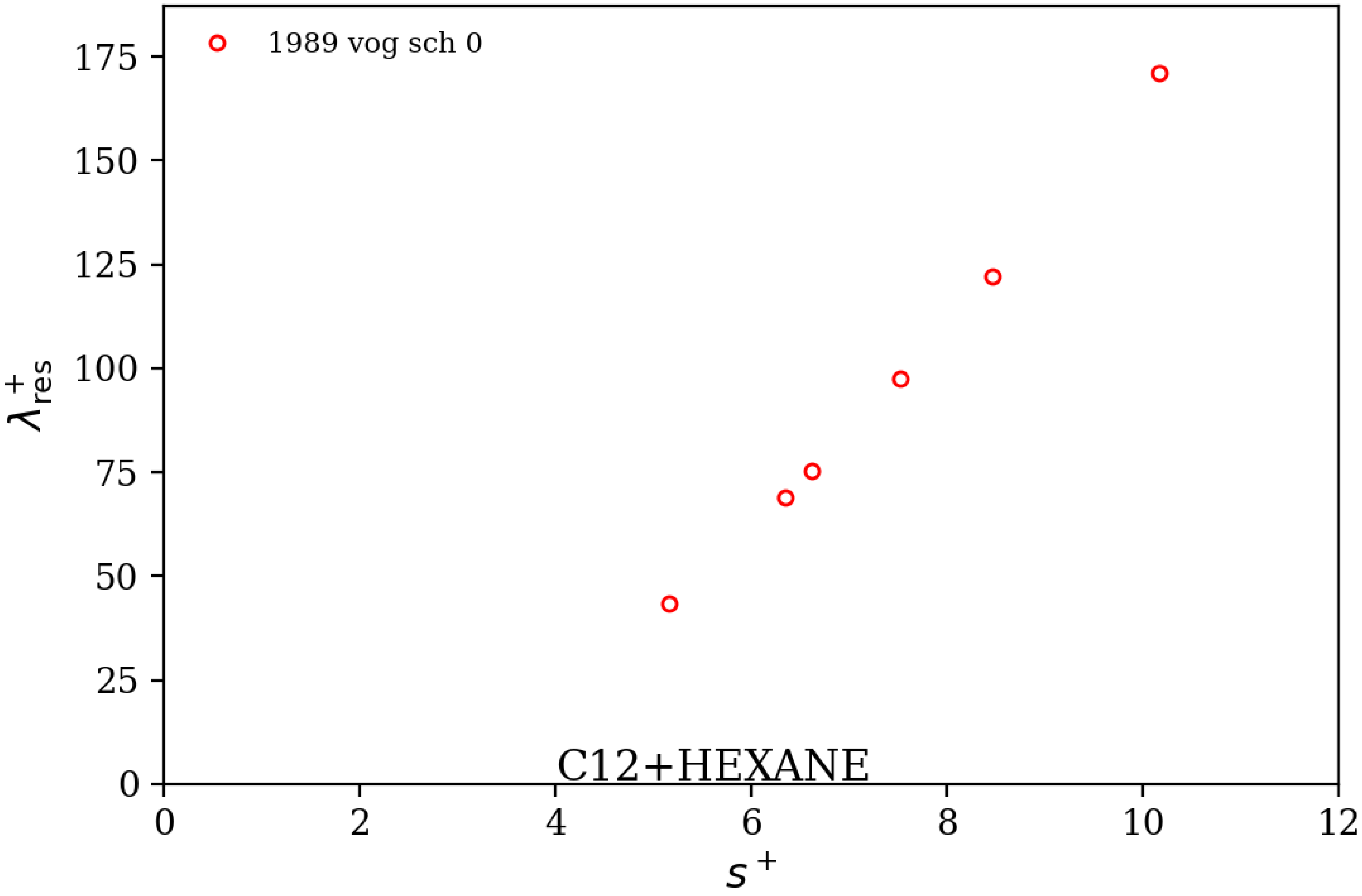

Figure DPR3. C12+HEXANE

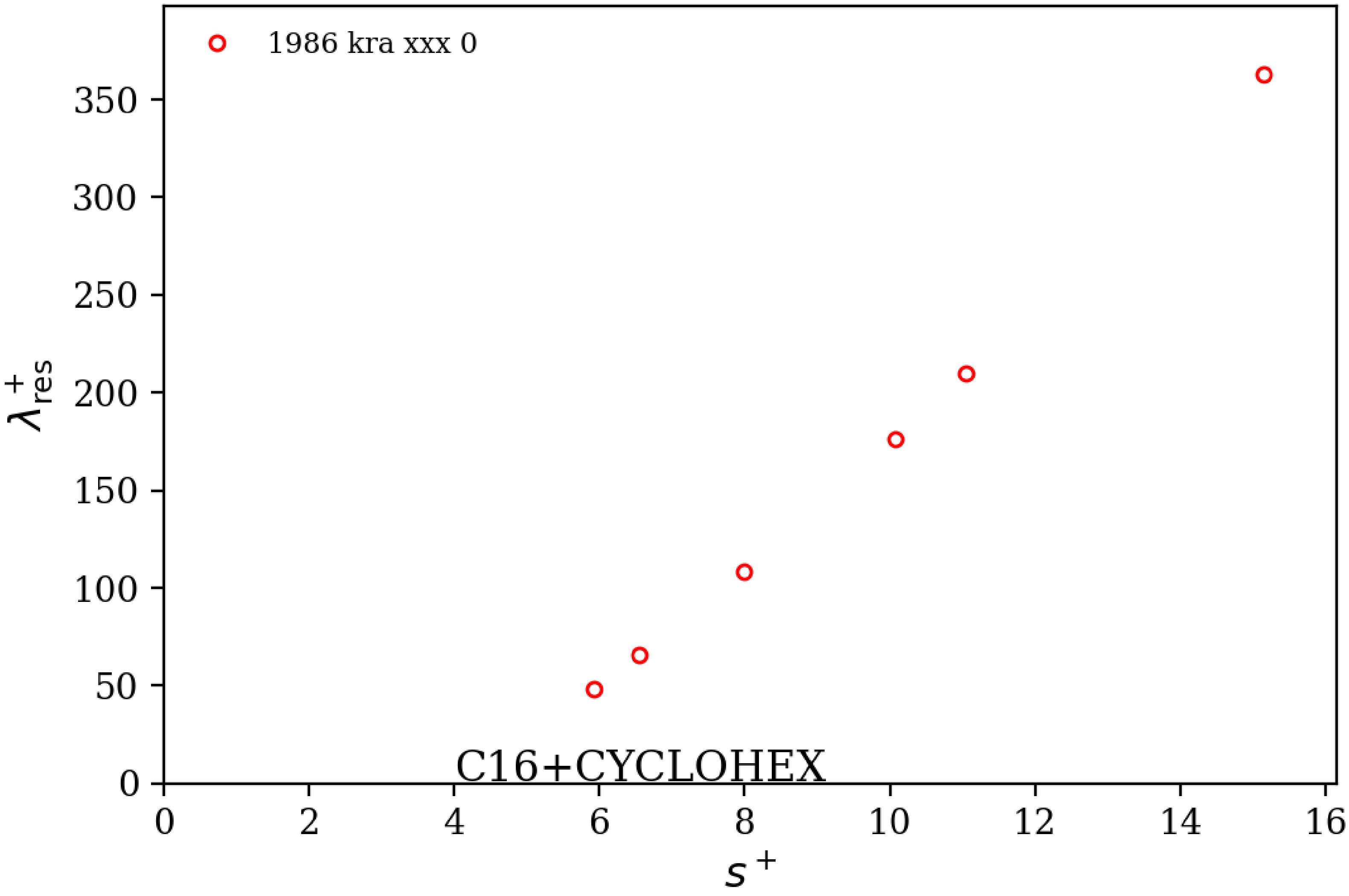

Figure DPR3. C16+CYCLOHEX

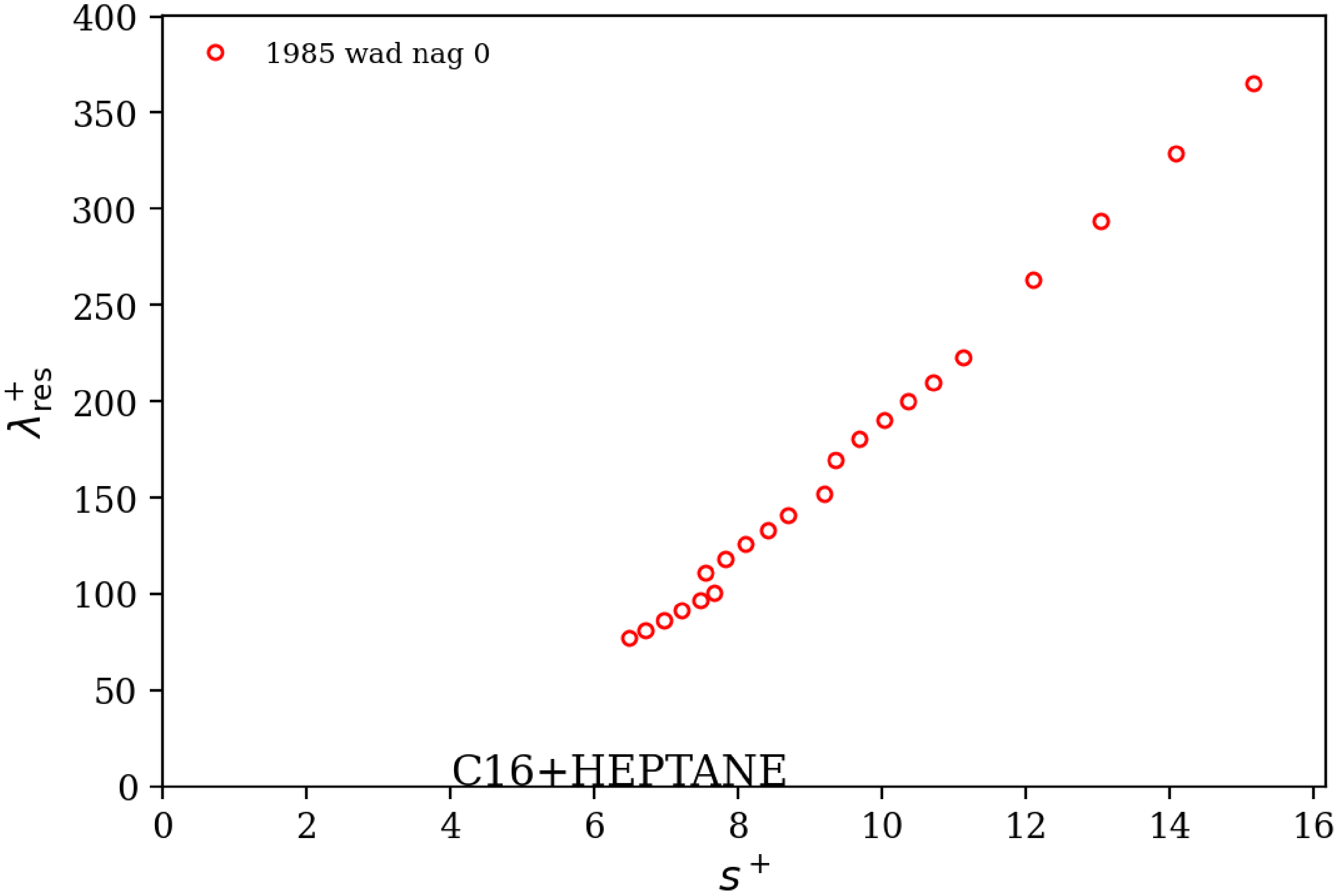

Figure DPR3. C16+HEPTANE

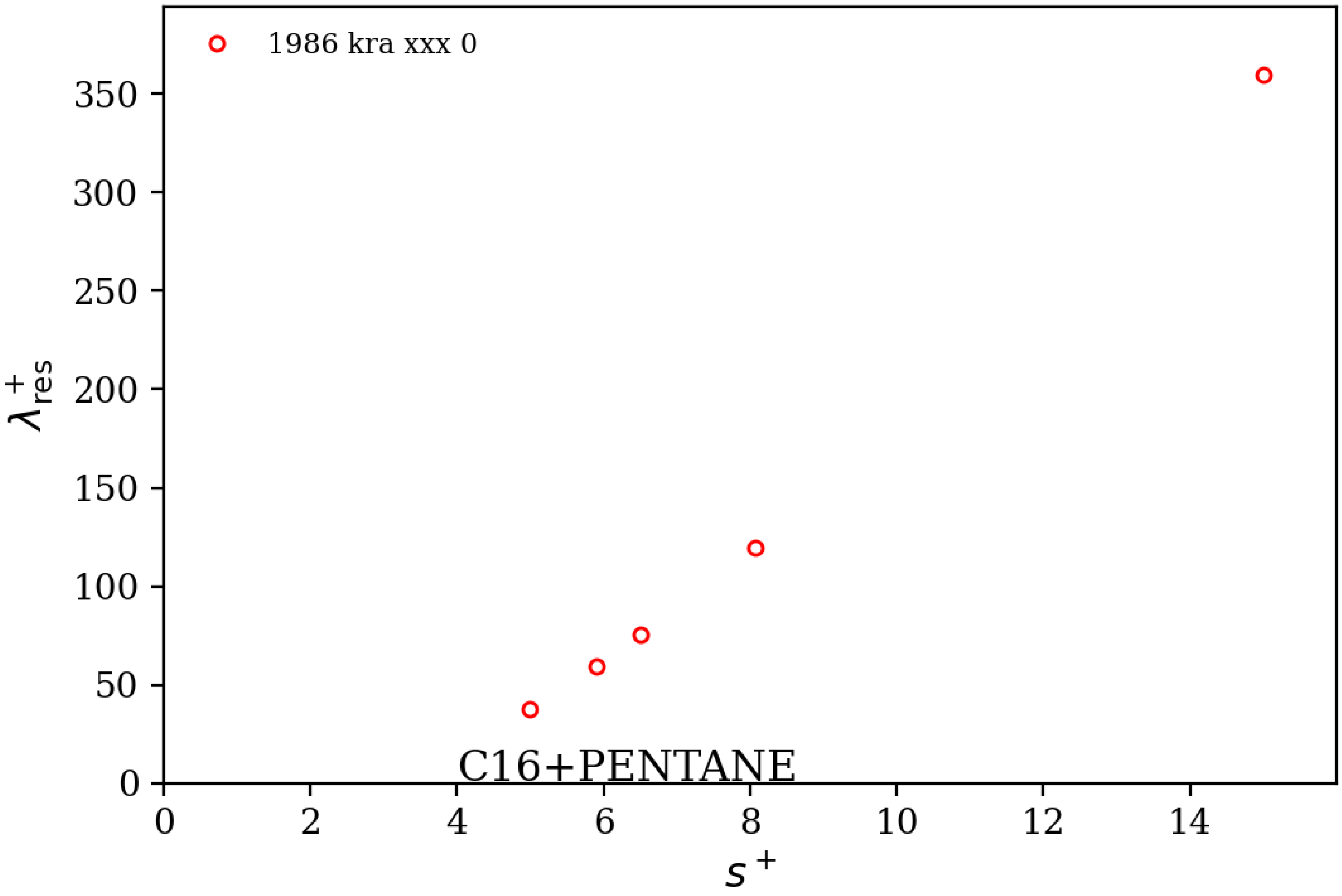

Figure DPR3. C16+PENTANE

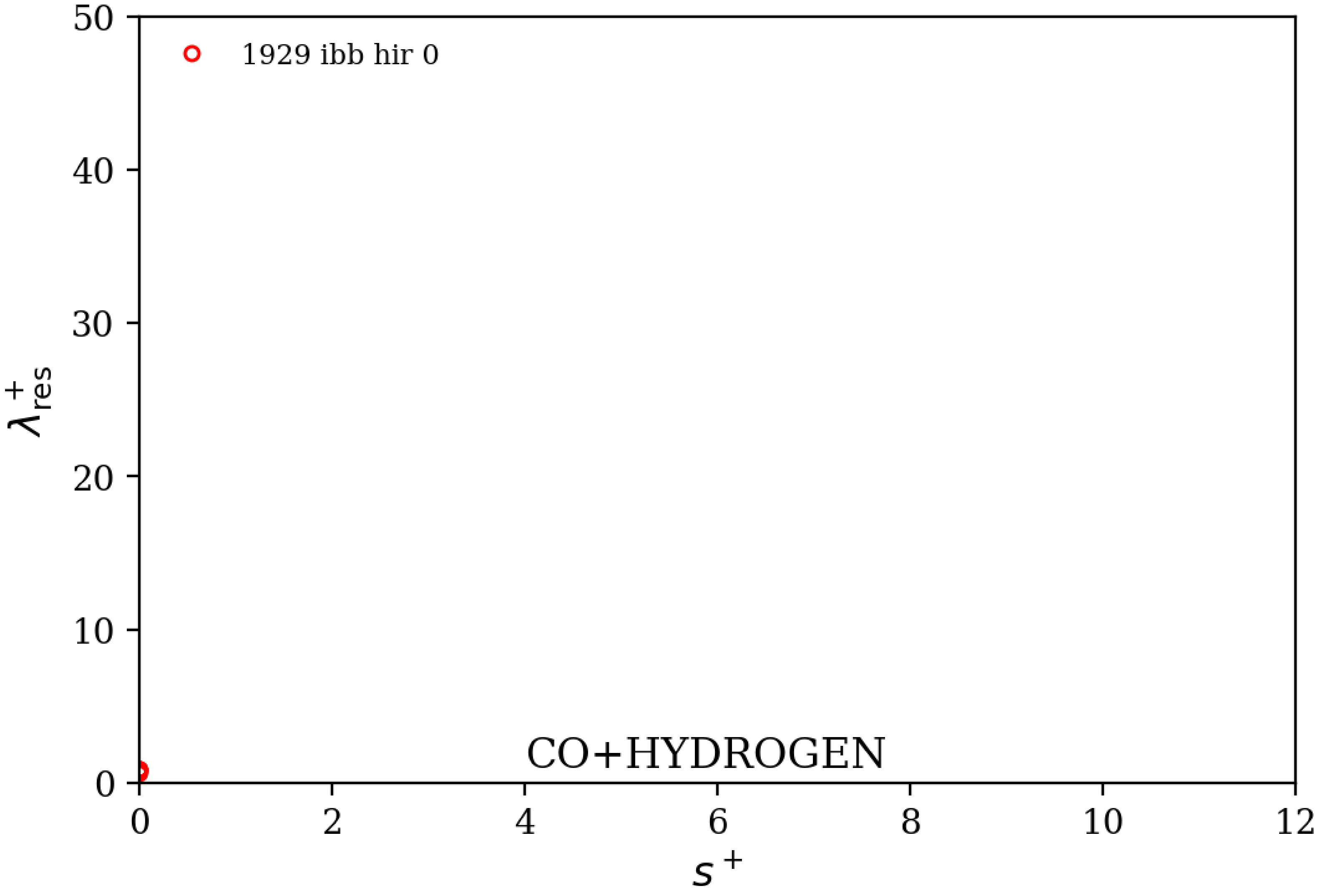

Figure DPR3. CO+HYDROGEN

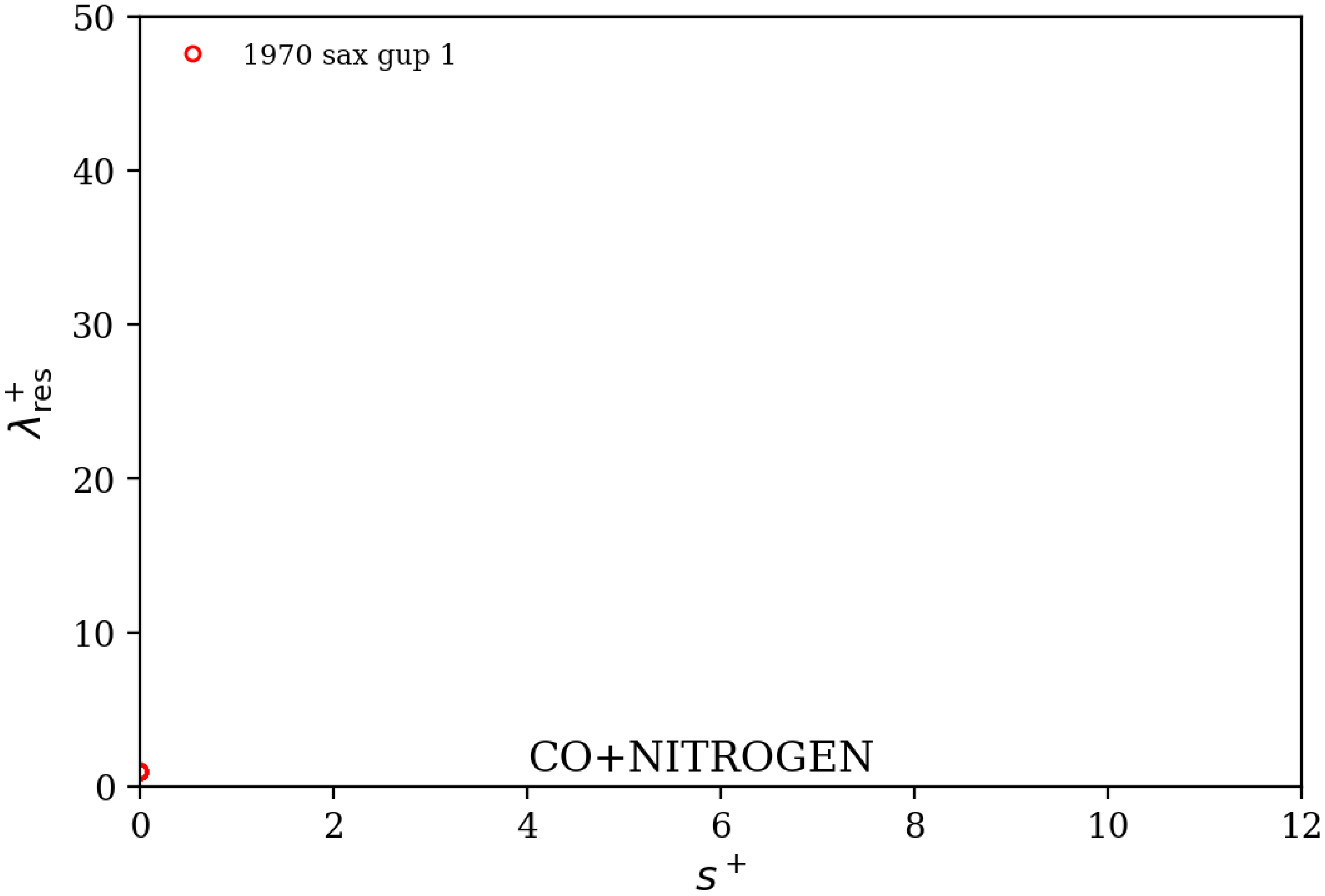

Figure DPR3. CO+NITROGEN

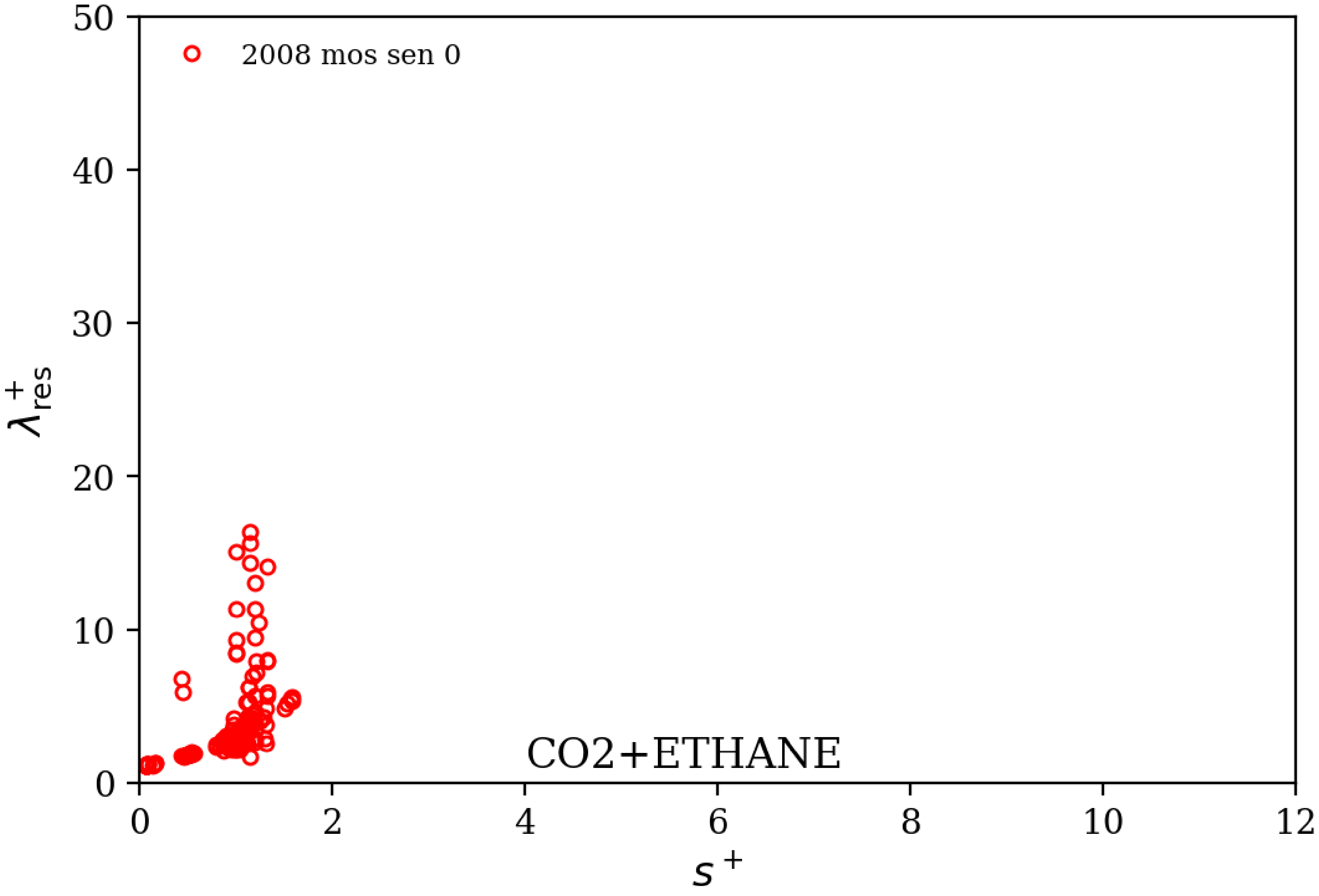

Figure DPR3. CO2+ETHANE

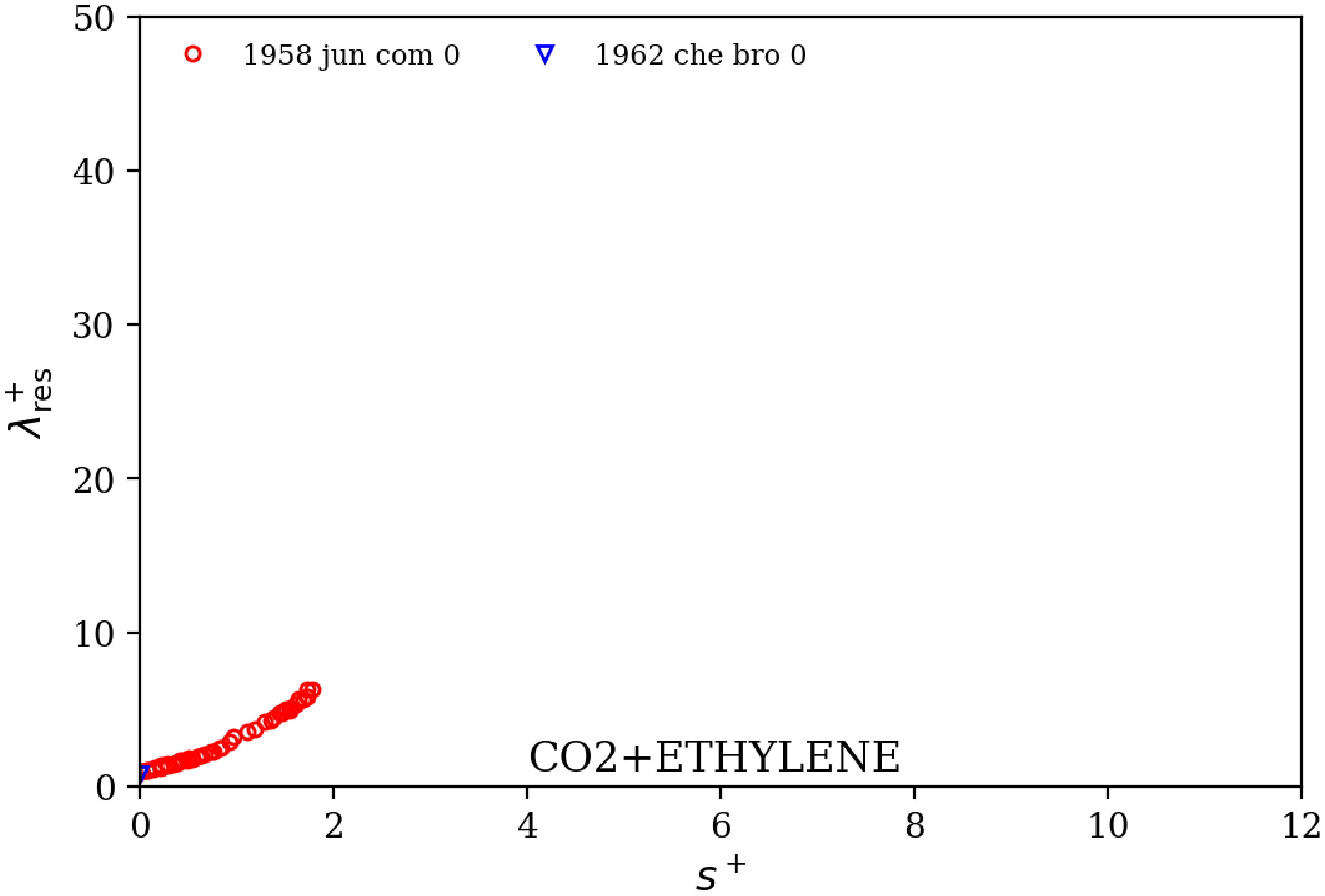

Figure DPR3. CO2+ETHYLENE

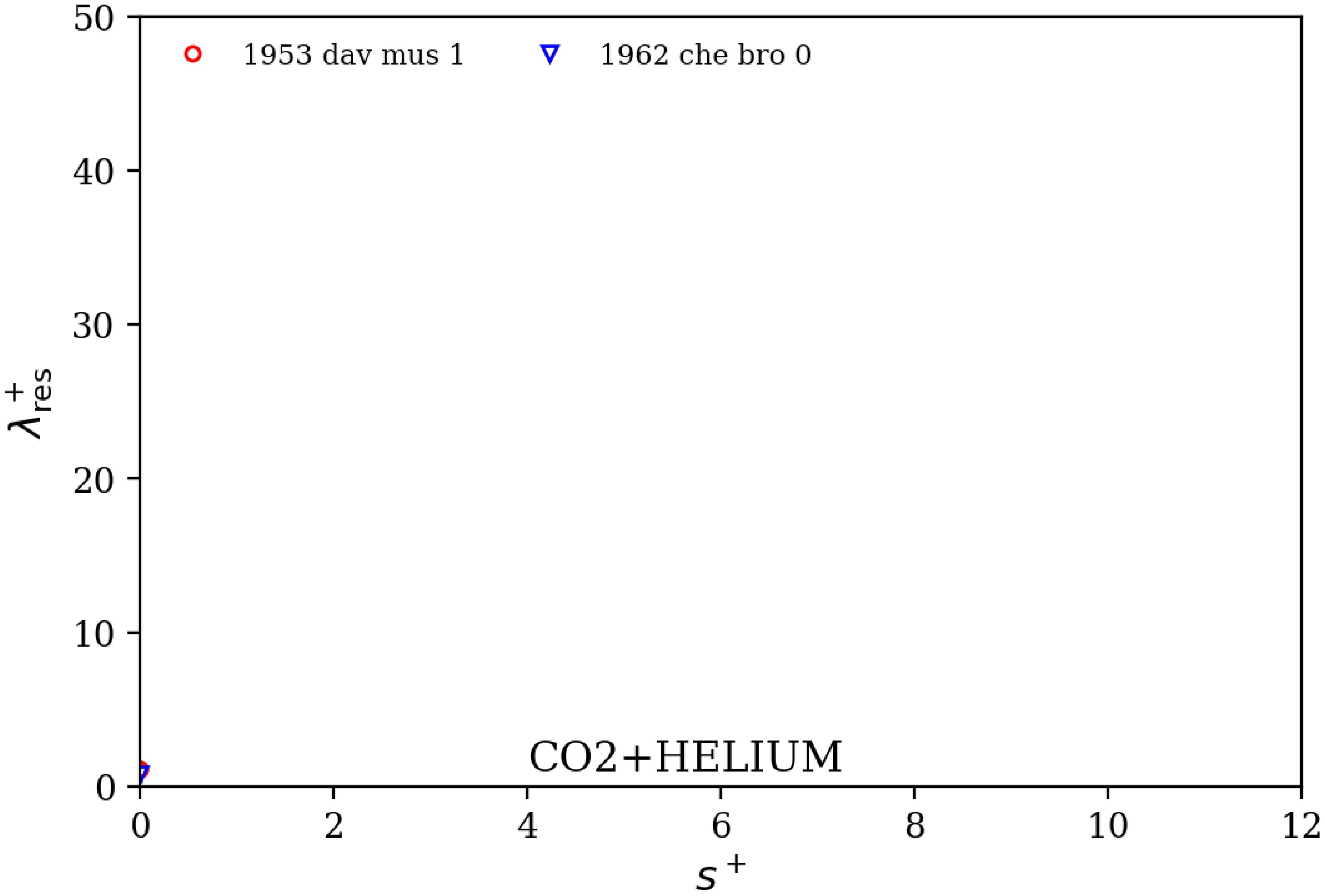

Figure DPR3. CO2+HELIUM

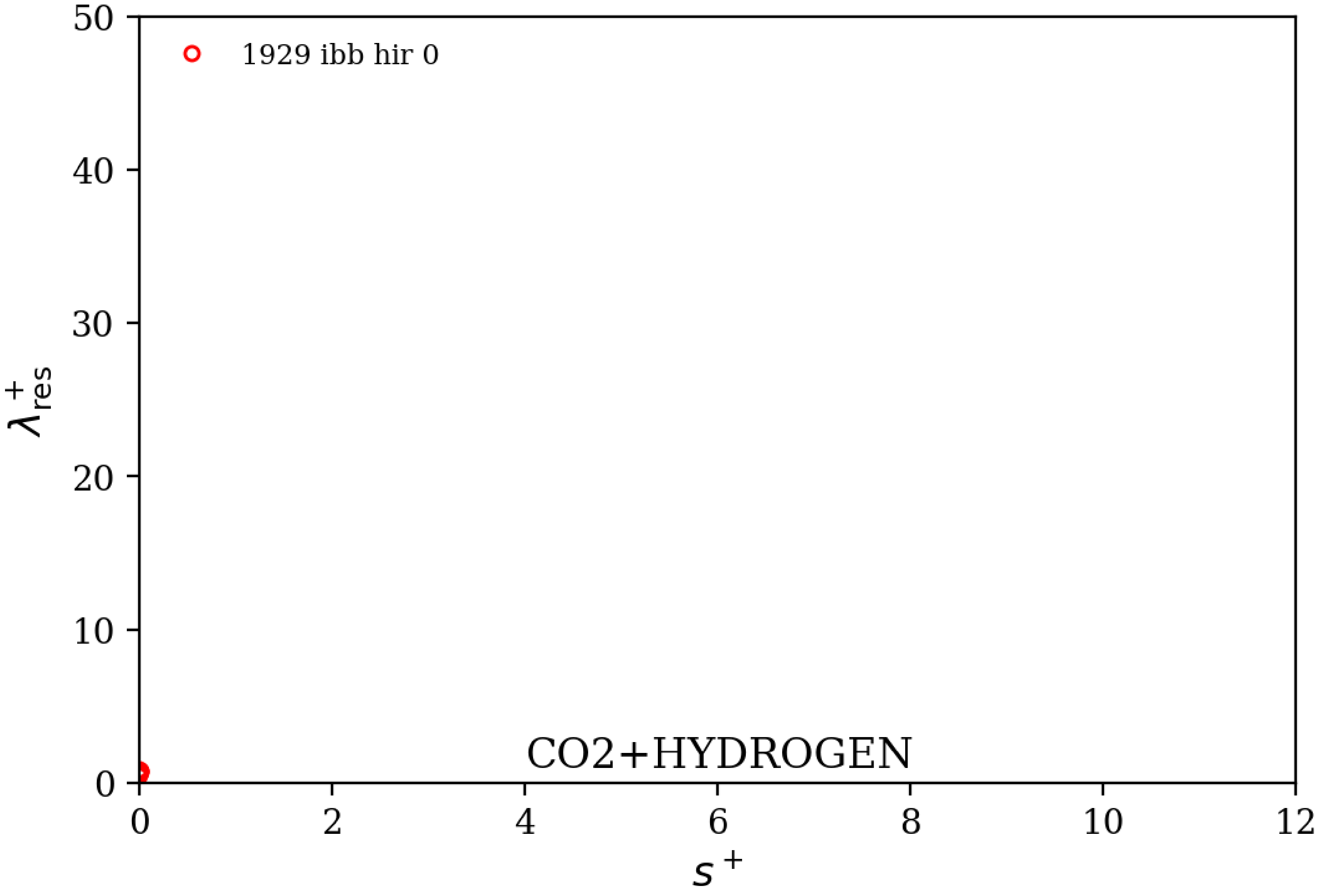

Figure DPR3. CO2+HYDROGEN

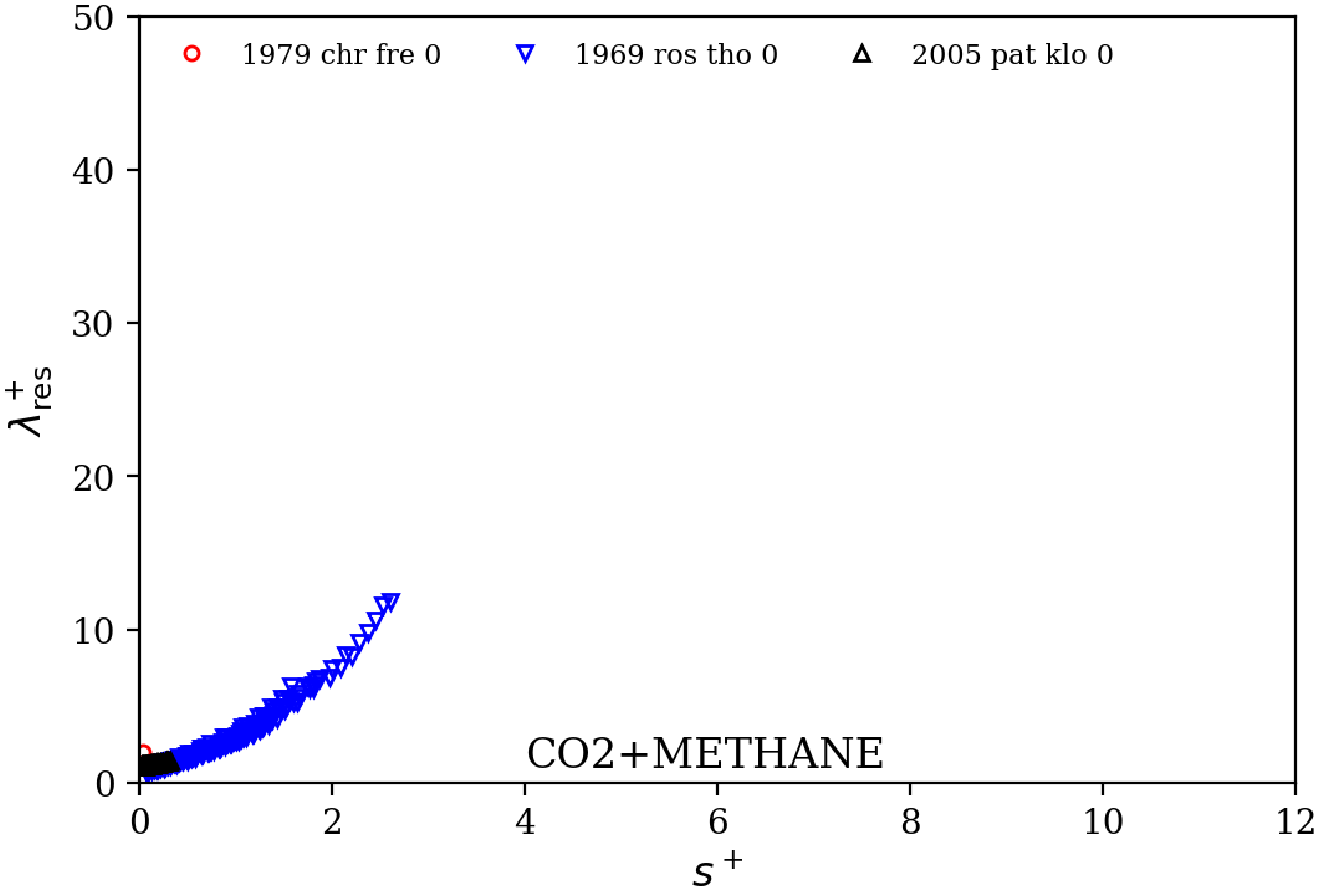

Figure DPR3. CO2+METHANE

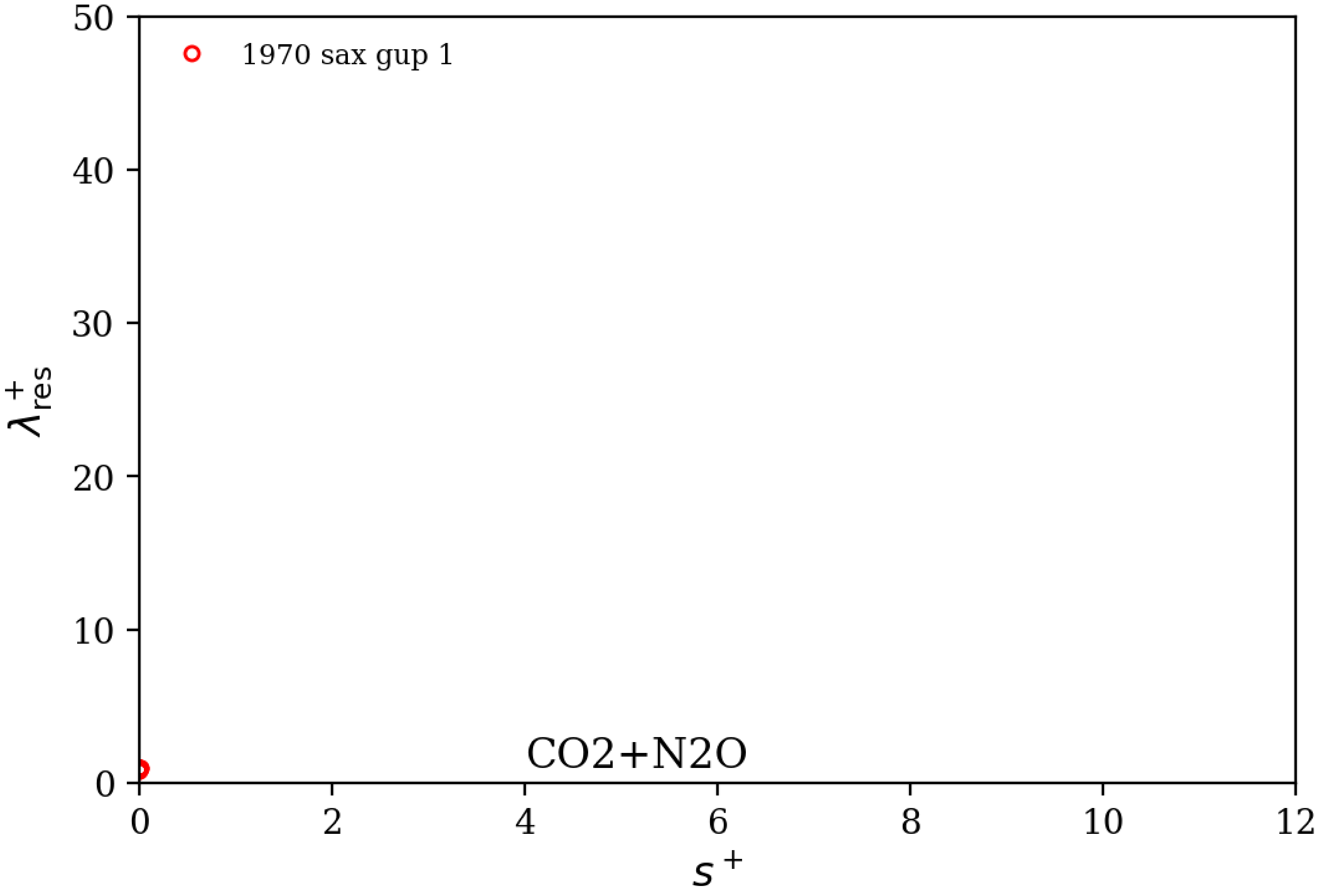

Figure DPR3. CO2+N2O

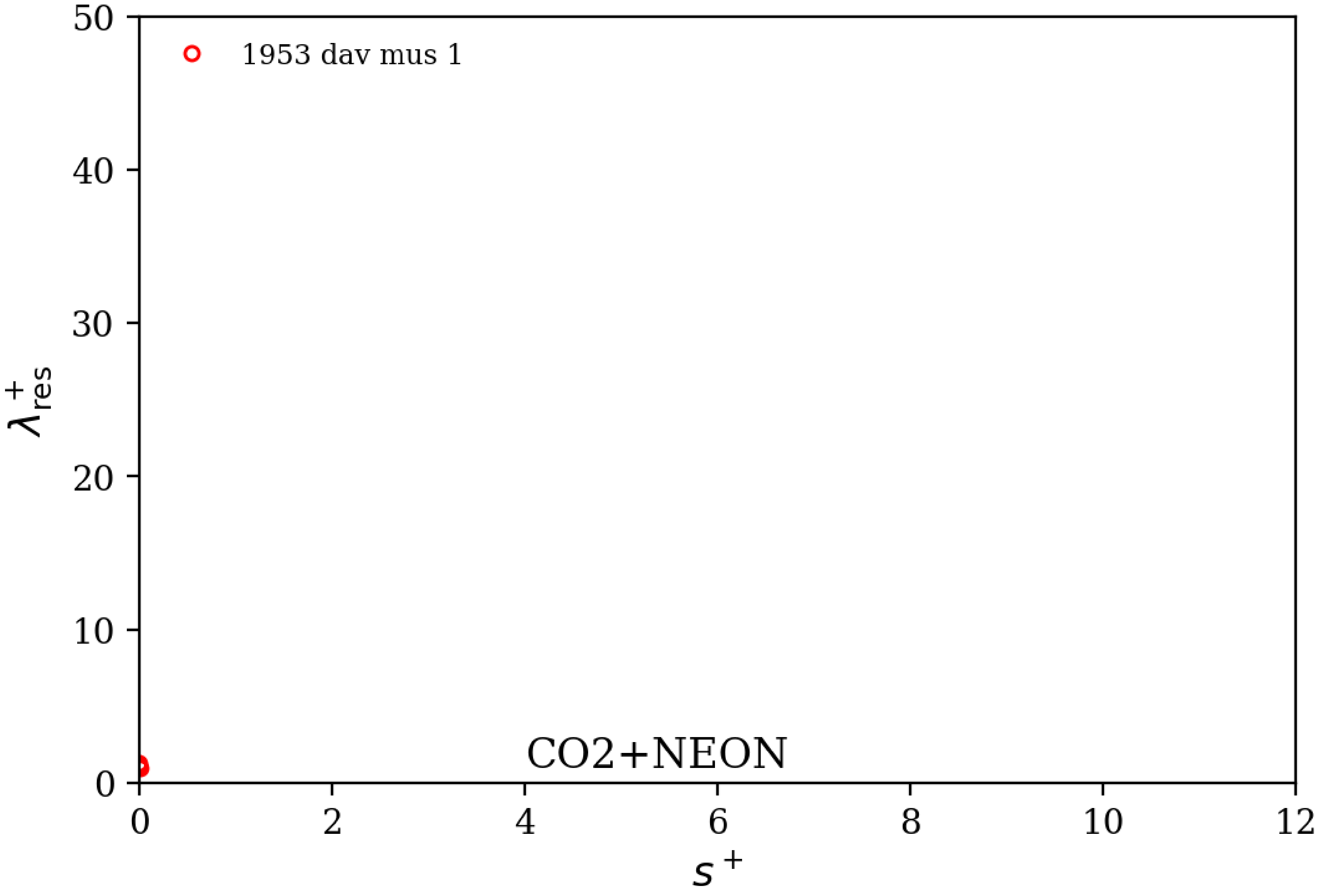

Figure DPR3. CO2+NEON

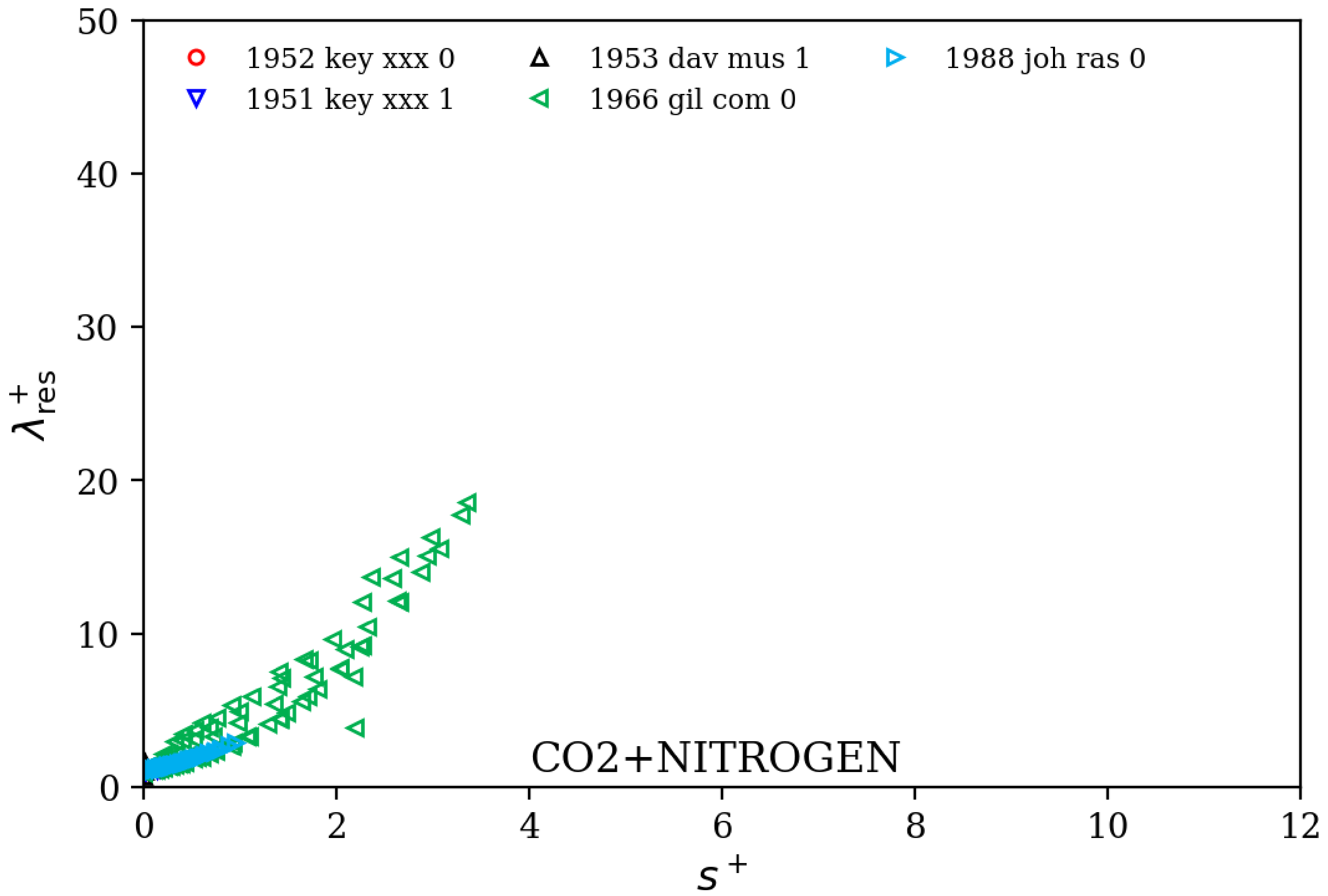

Figure DPR3. CO2+NITROGEN

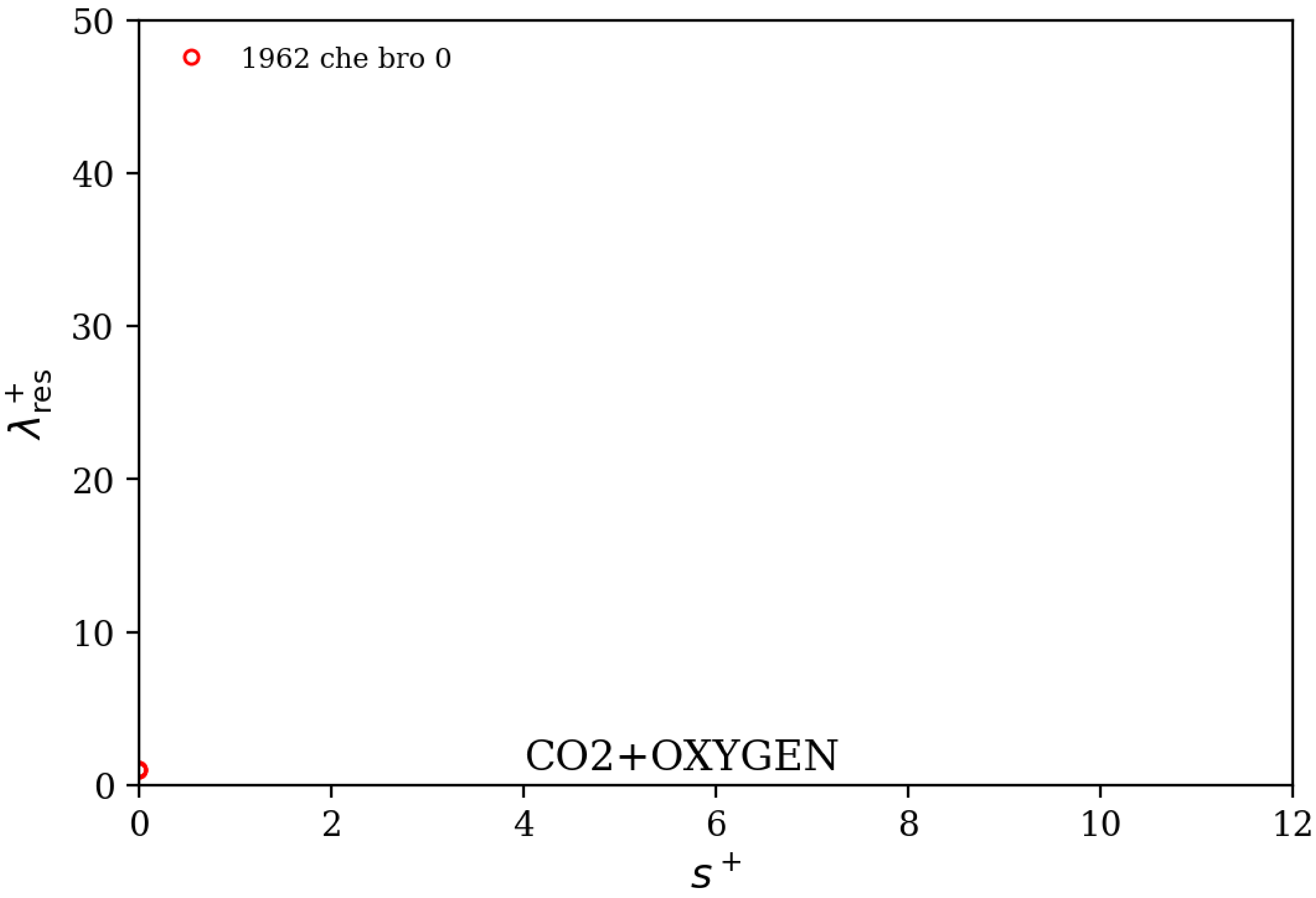

Figure DPR3. CO2+OXYGEN

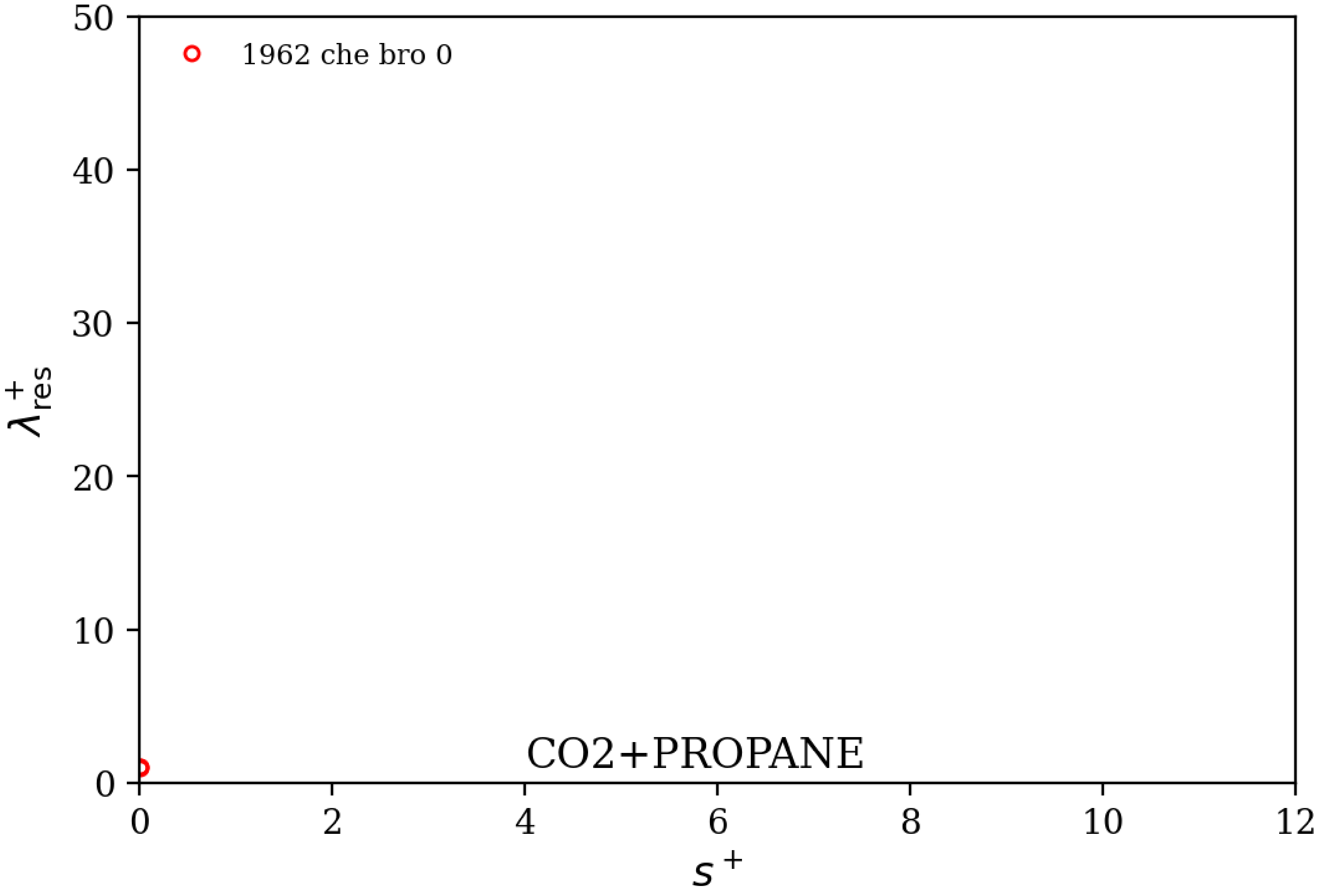

Figure DPR3. CO2+PROPANE

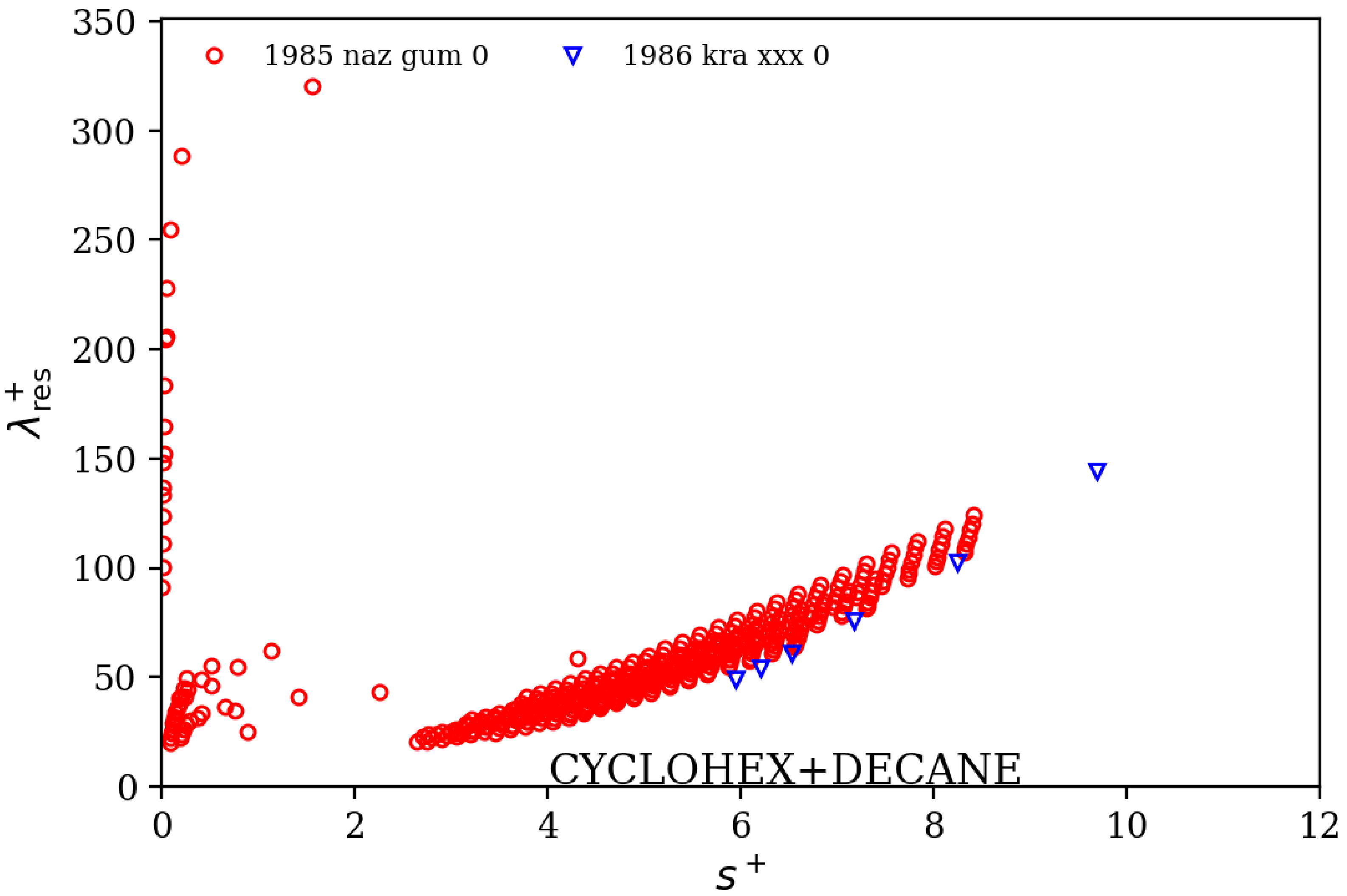

Figure DPR3. CYCLOHEX+DECANE

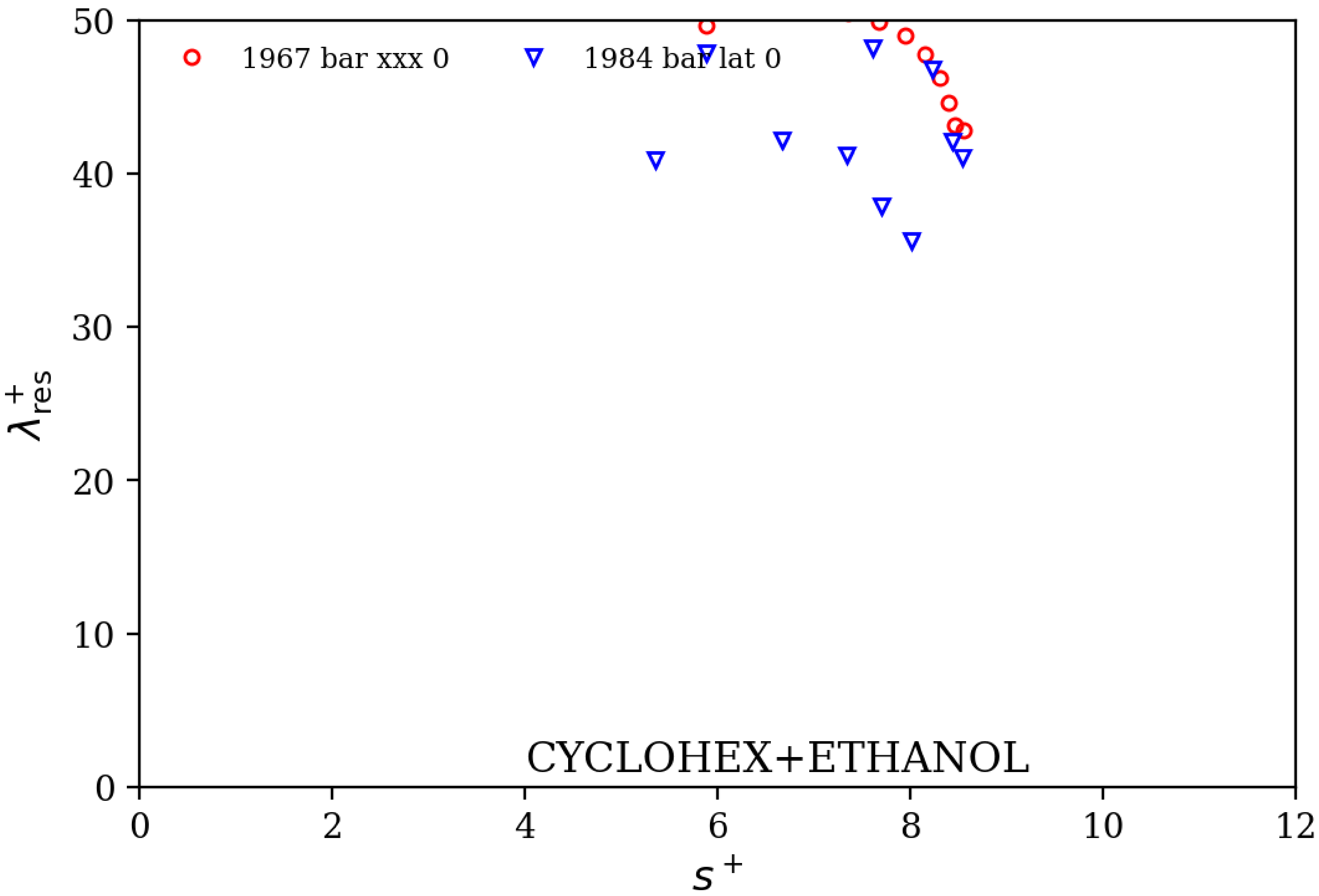

Figure DPR3. CYCLOHEX+ETHANOL

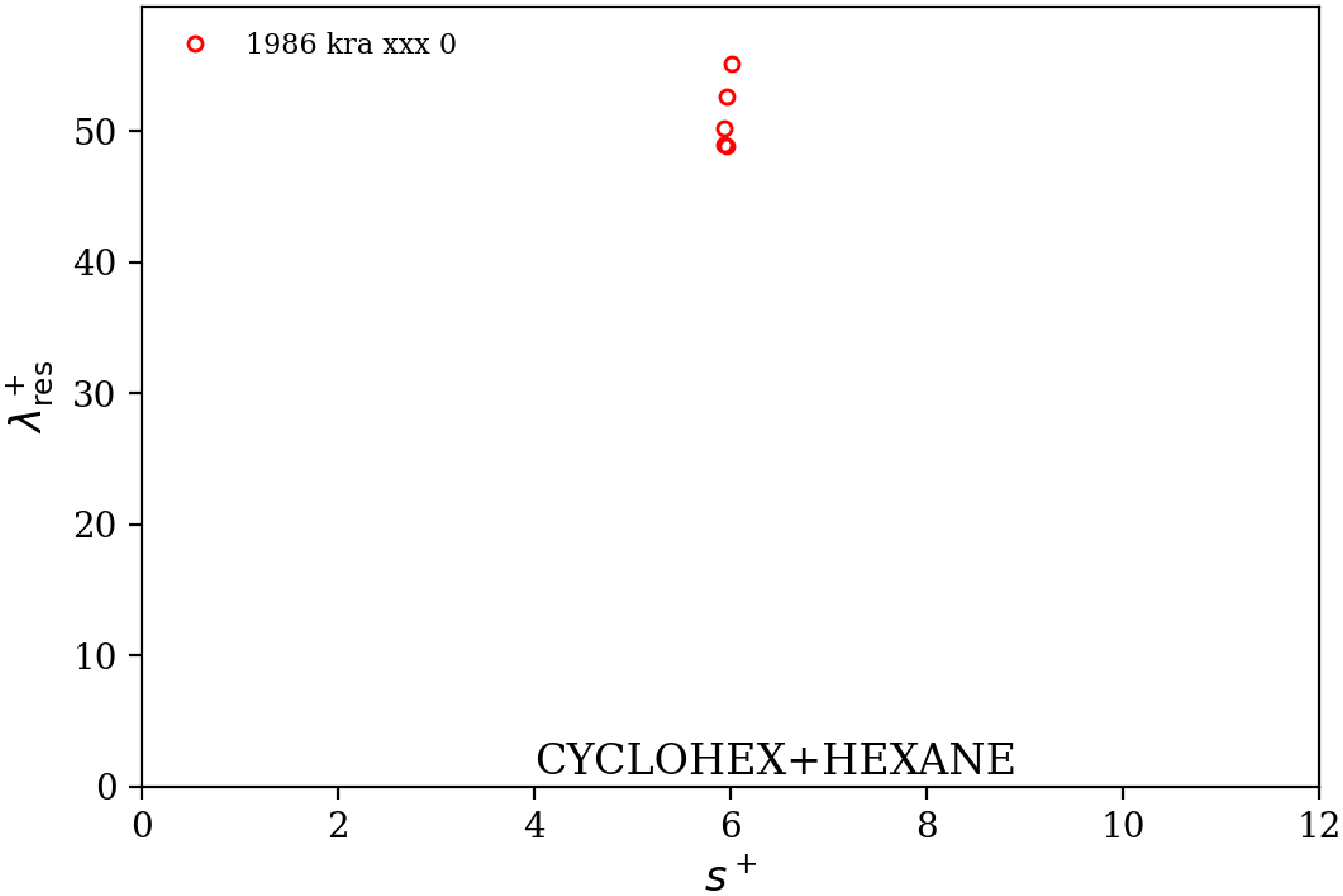

Figure DPR3. CYCLOHEX+HEXANE

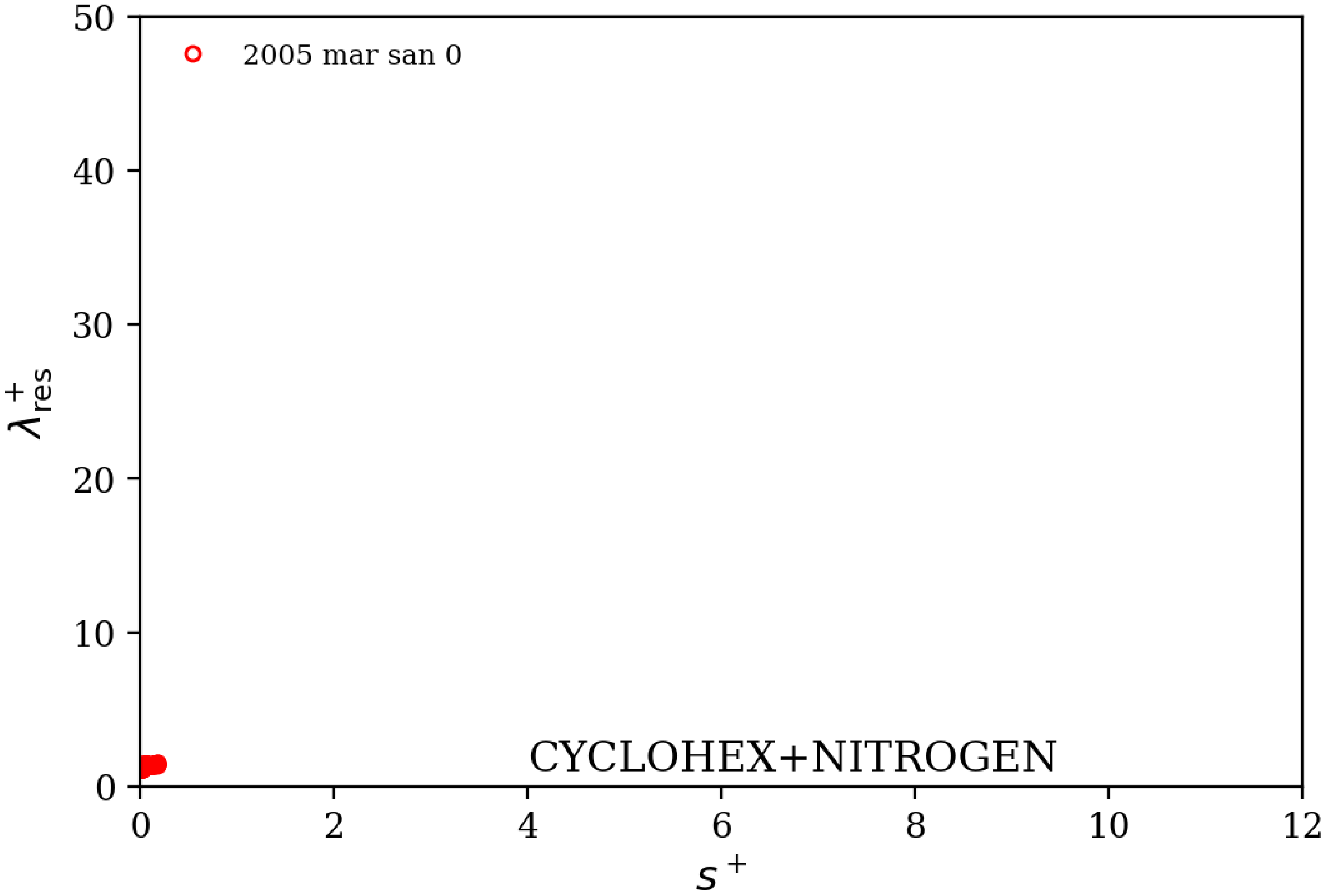

Figure DPR3. CYCLOHEX+NITROGEN

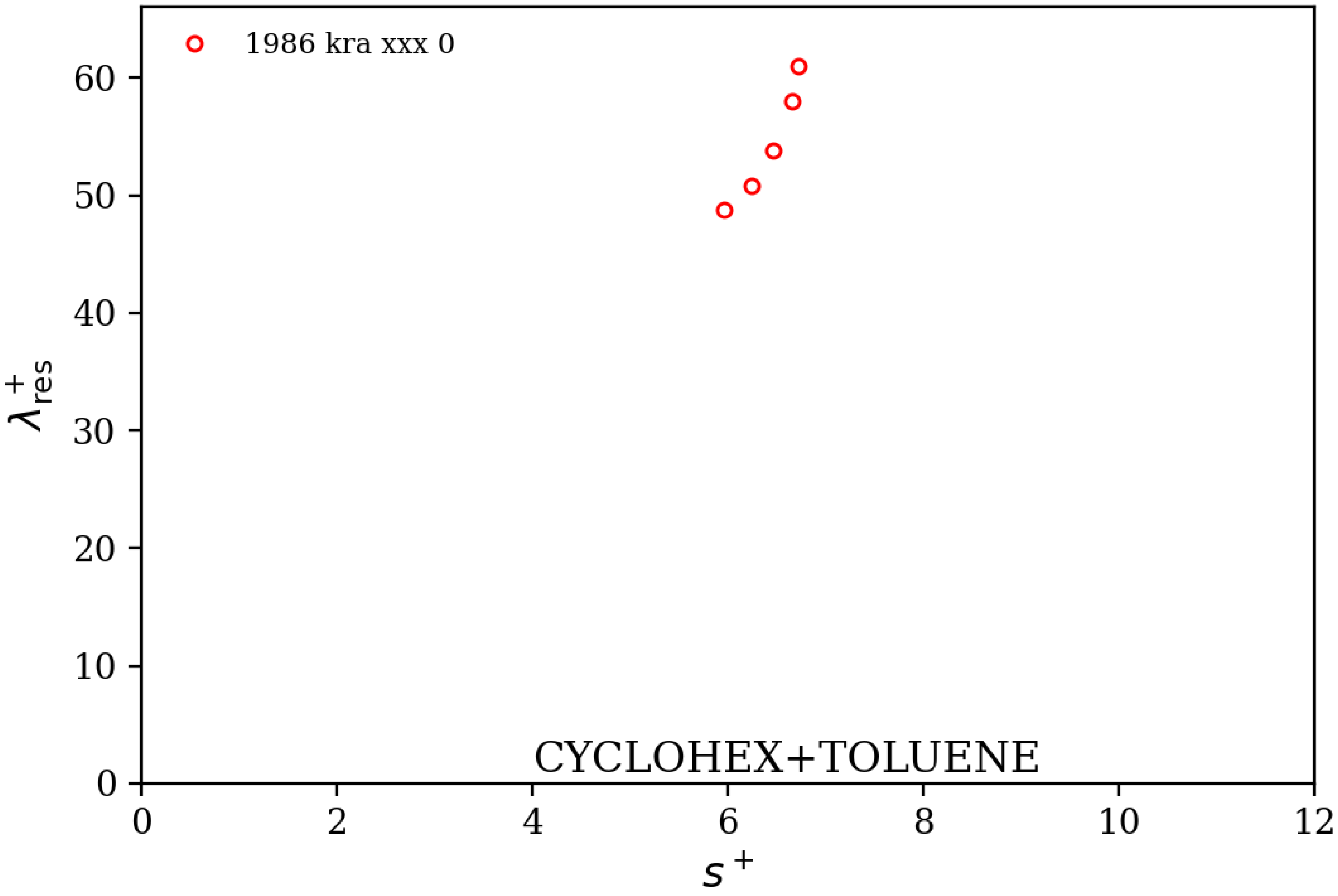

Figure DPR3. CYCLOHEX+TOLUENE

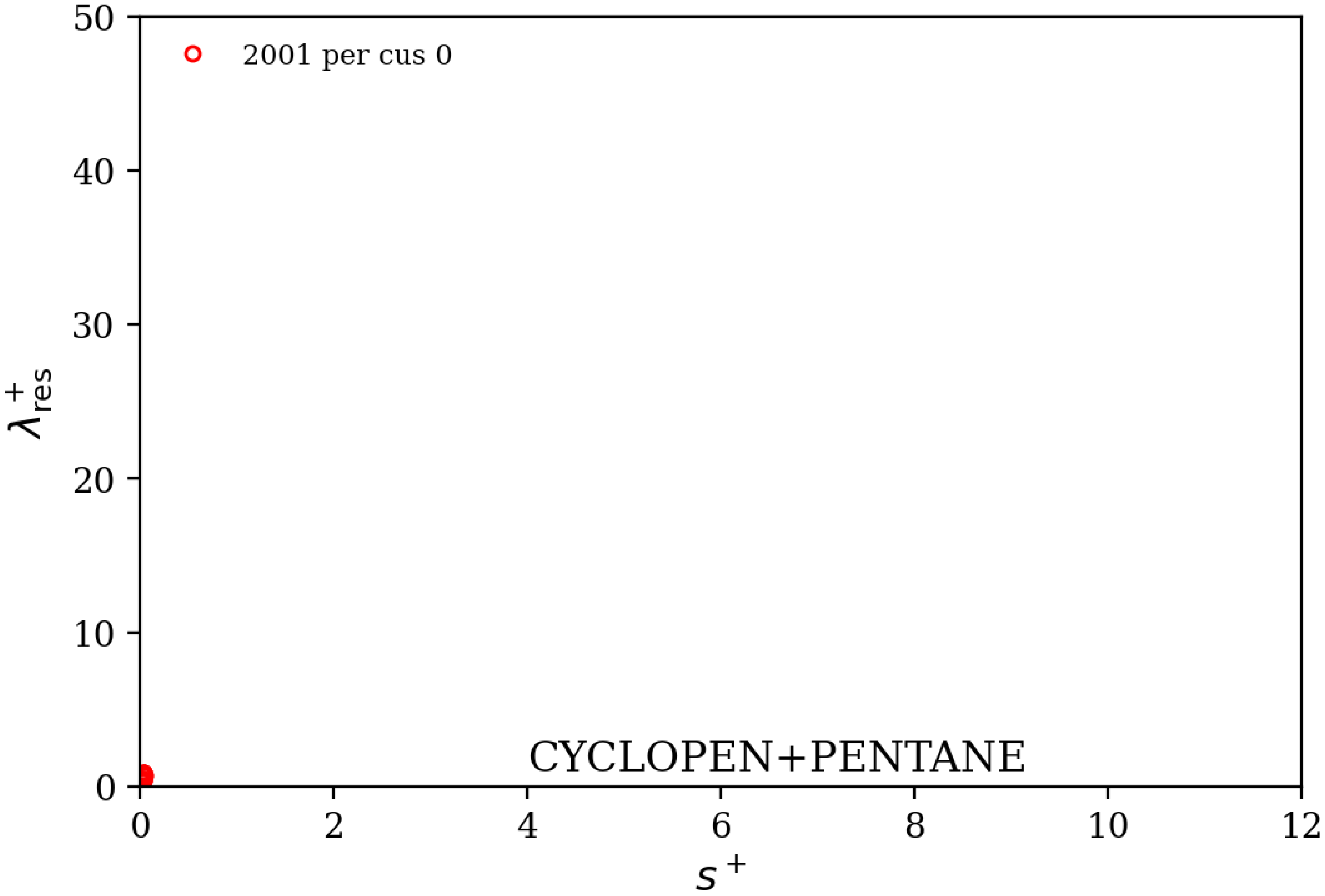

Figure DPR3. CYCLOPEN+PENTANE

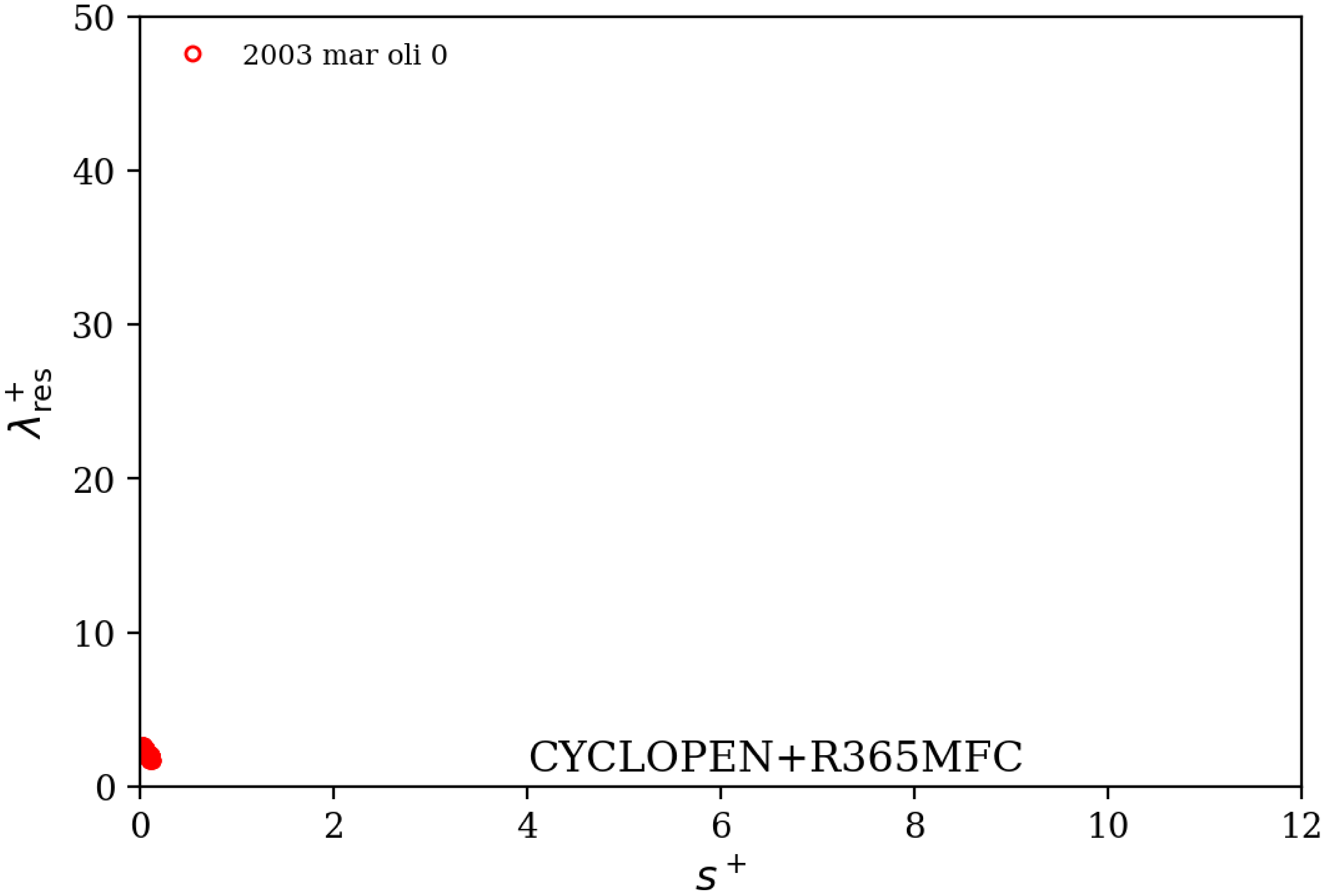

Figure DPR3. CYCLOPEN+R365MFC

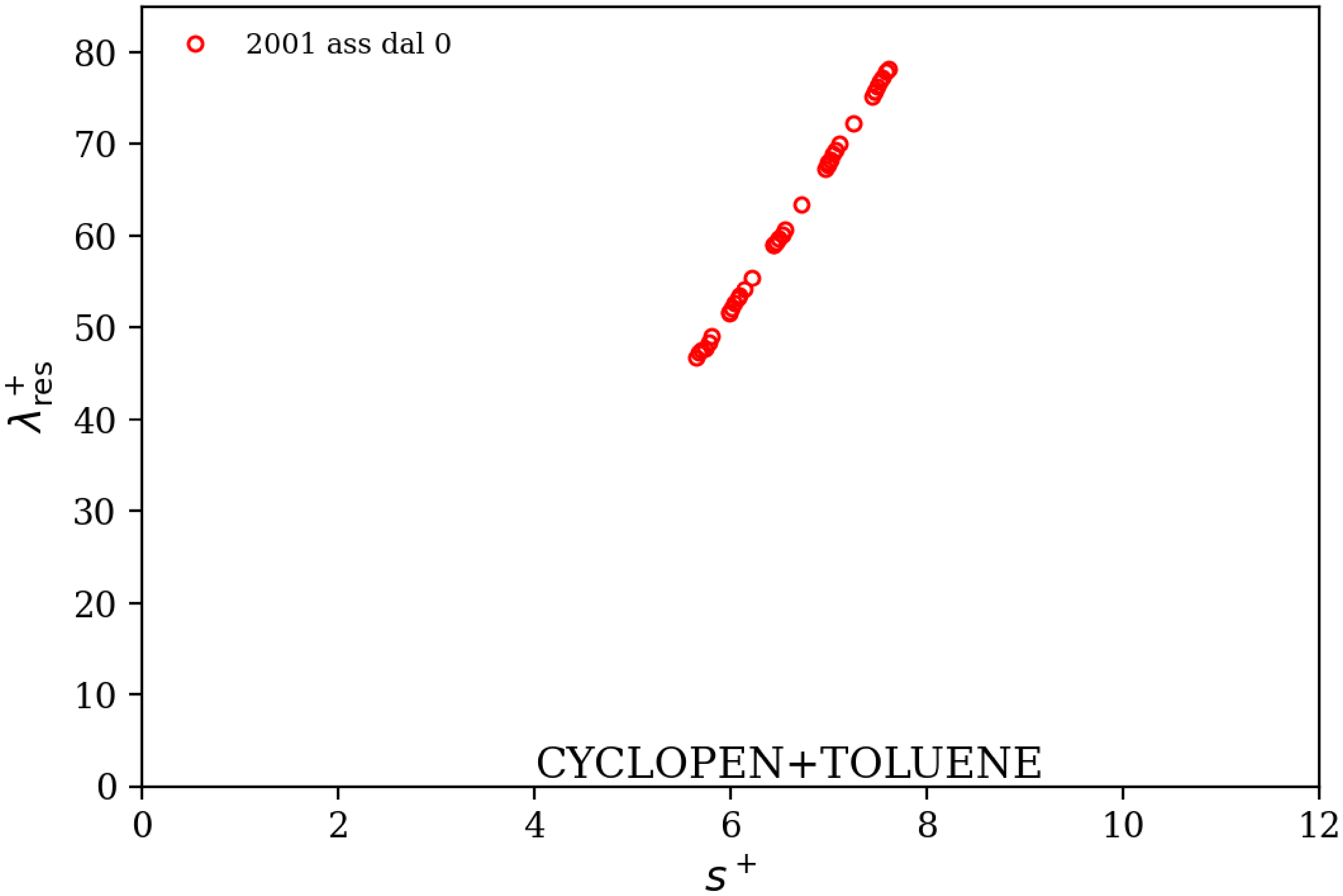

Figure DPR3. CYCLOPEN+TOLUENE

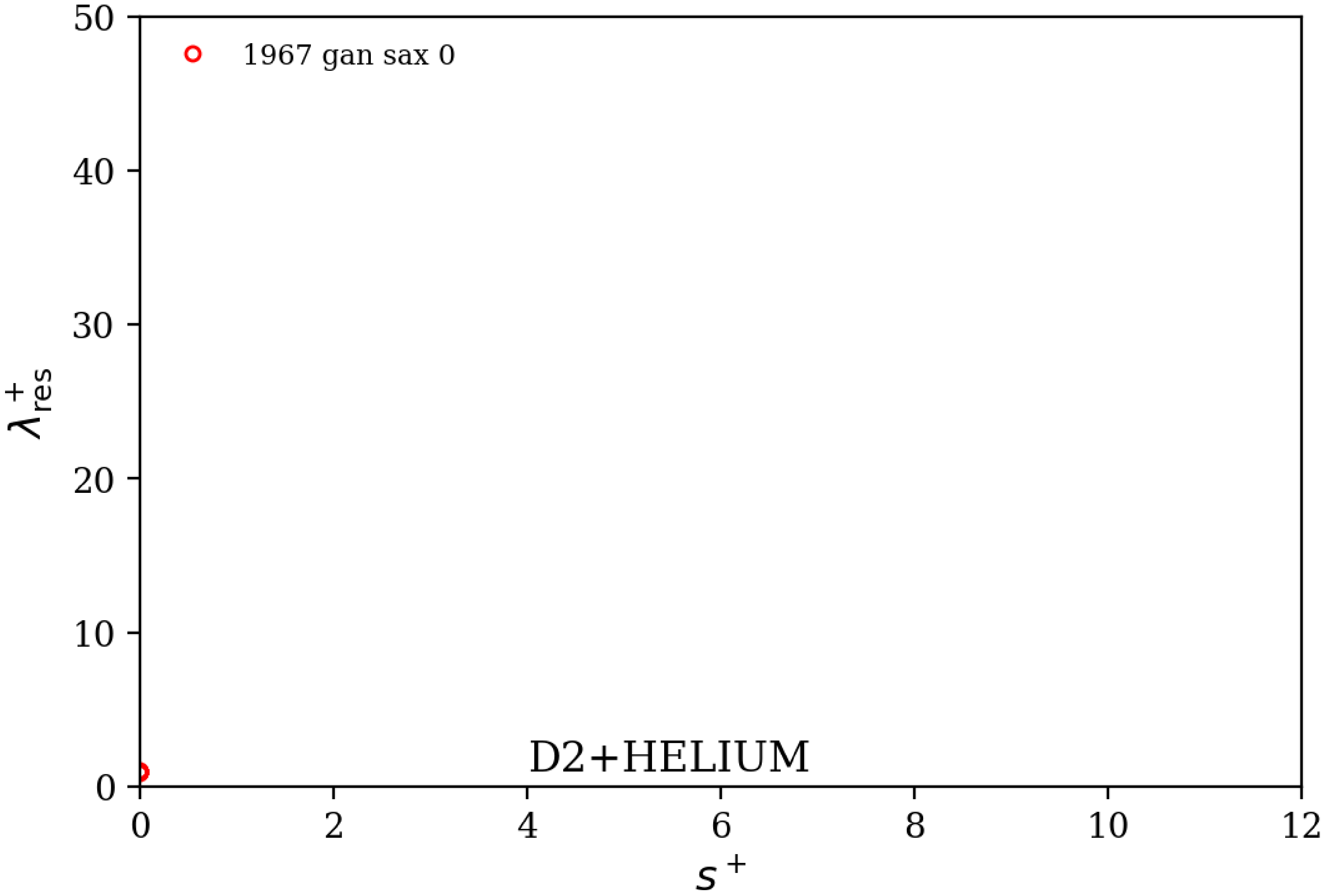

Figure DPR3. D2+HELIUM

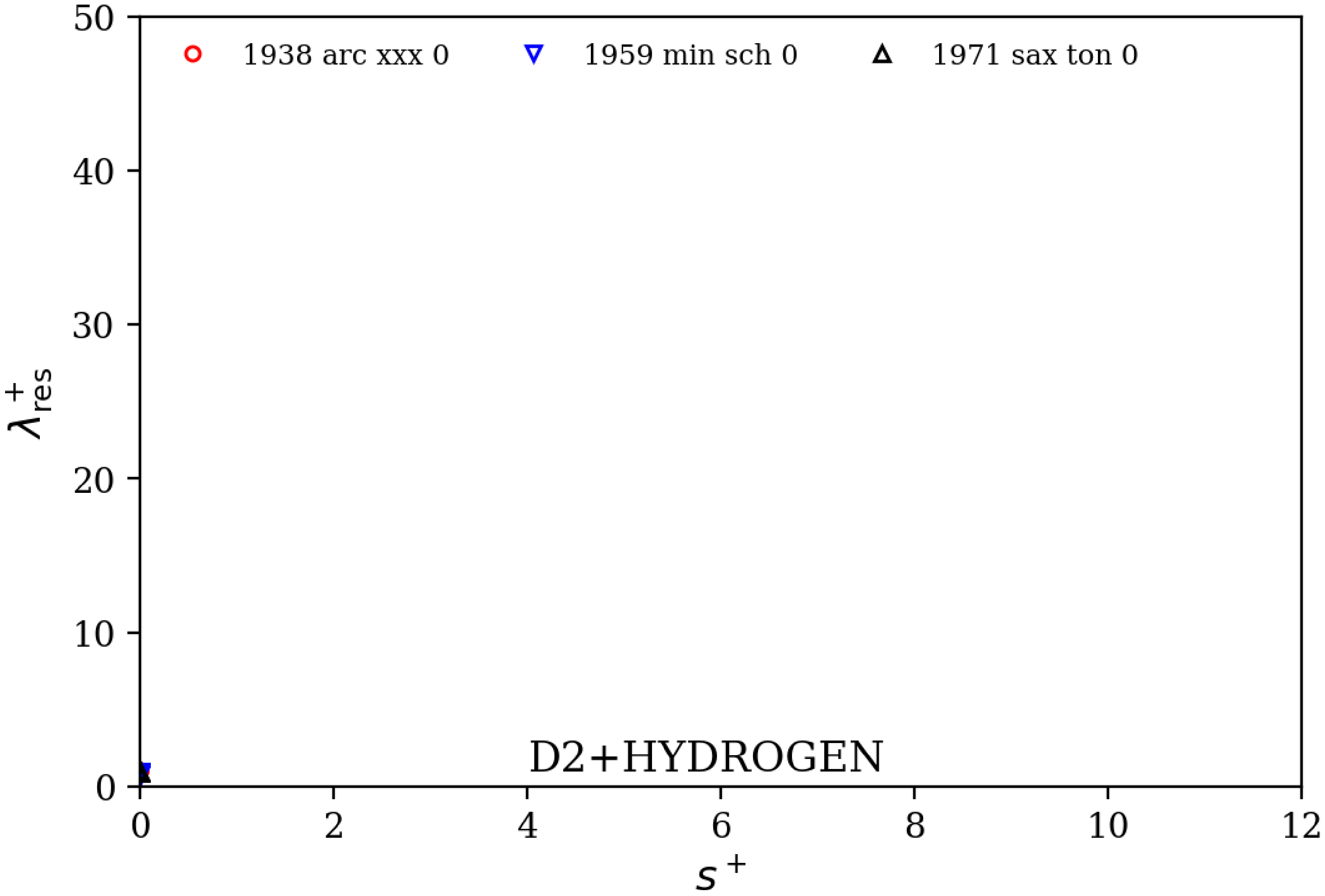

Figure DPR3. D2+HYDROGEN

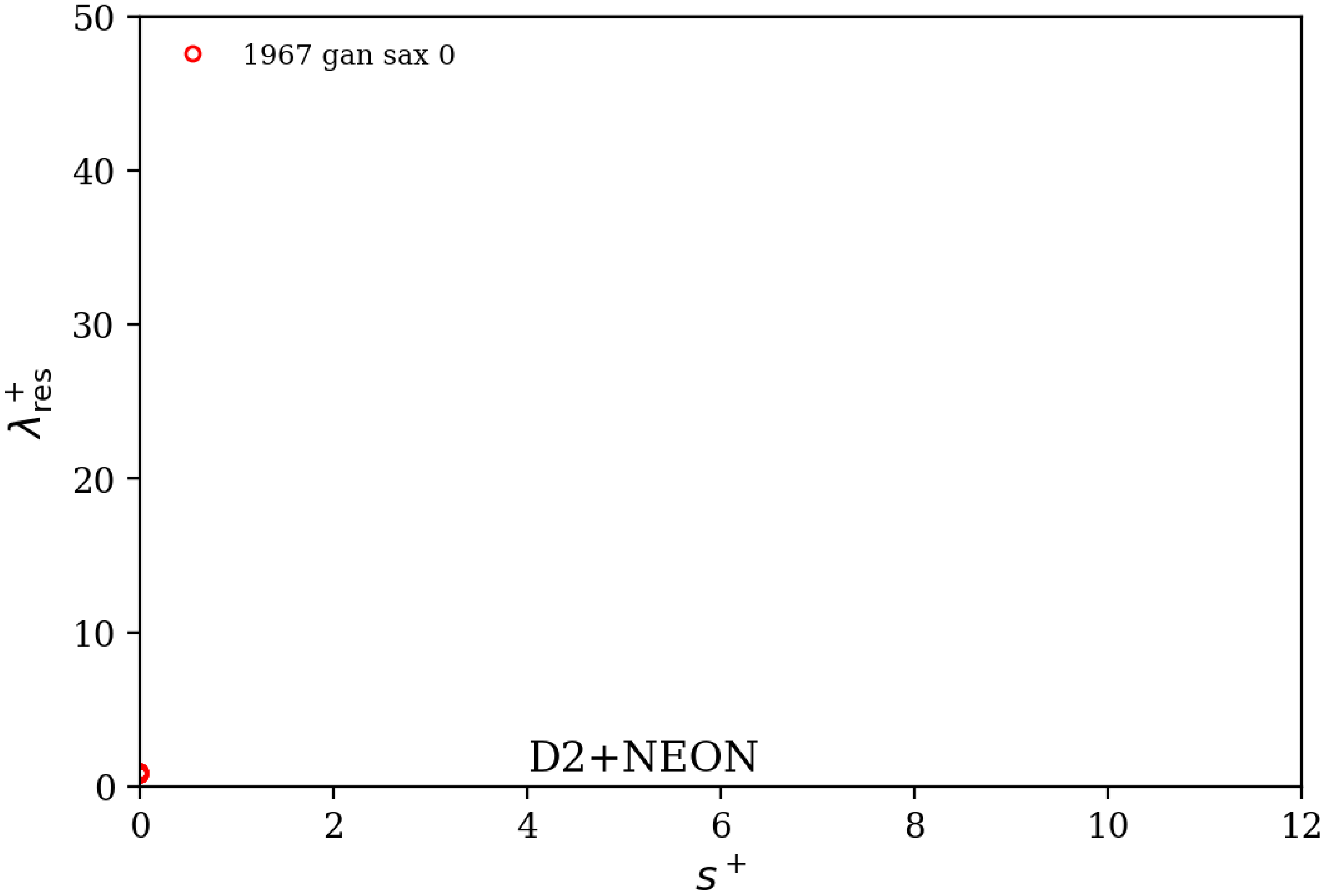

Figure DPR3. D2+NEON

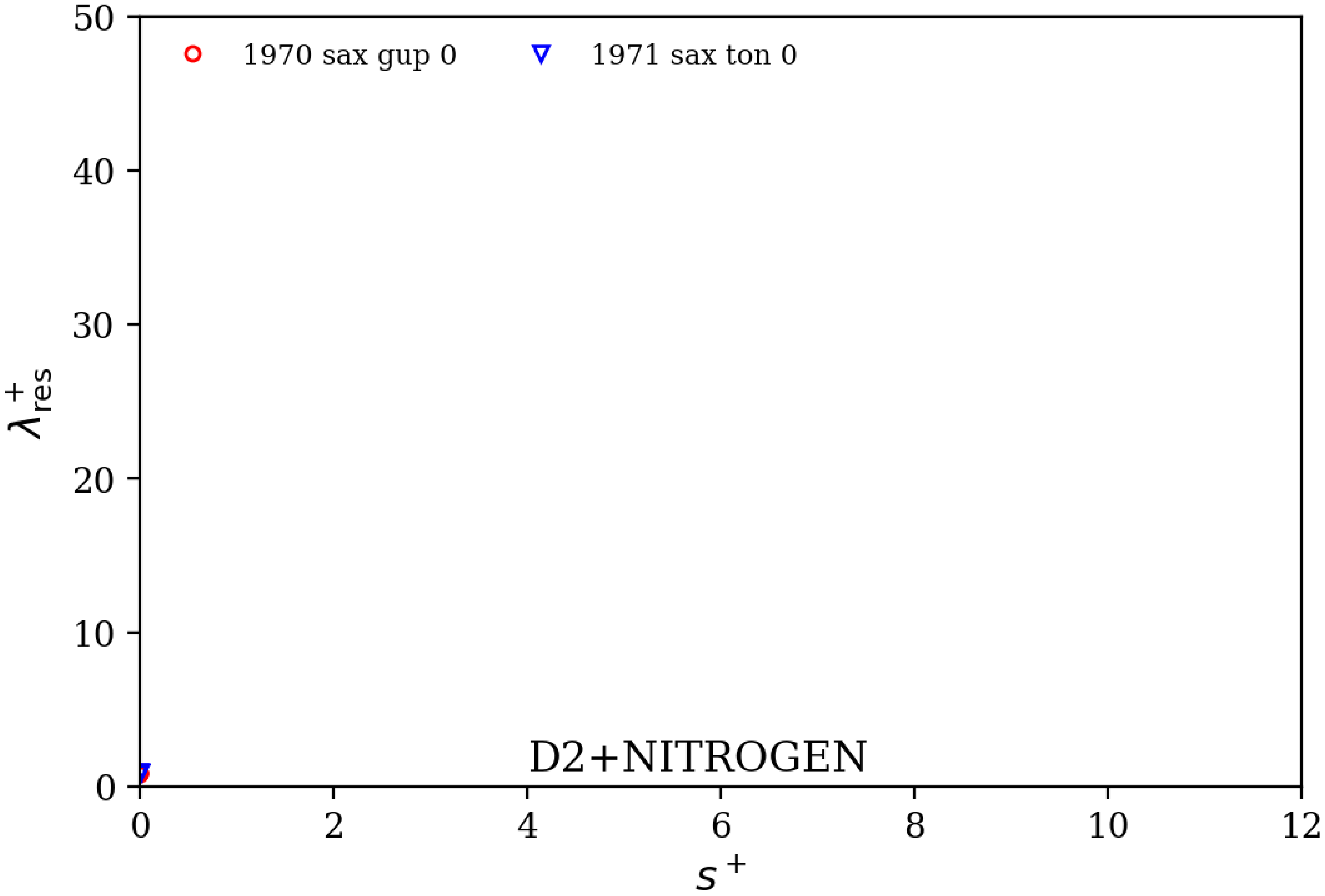

Figure DPR3. D2+NITROGEN

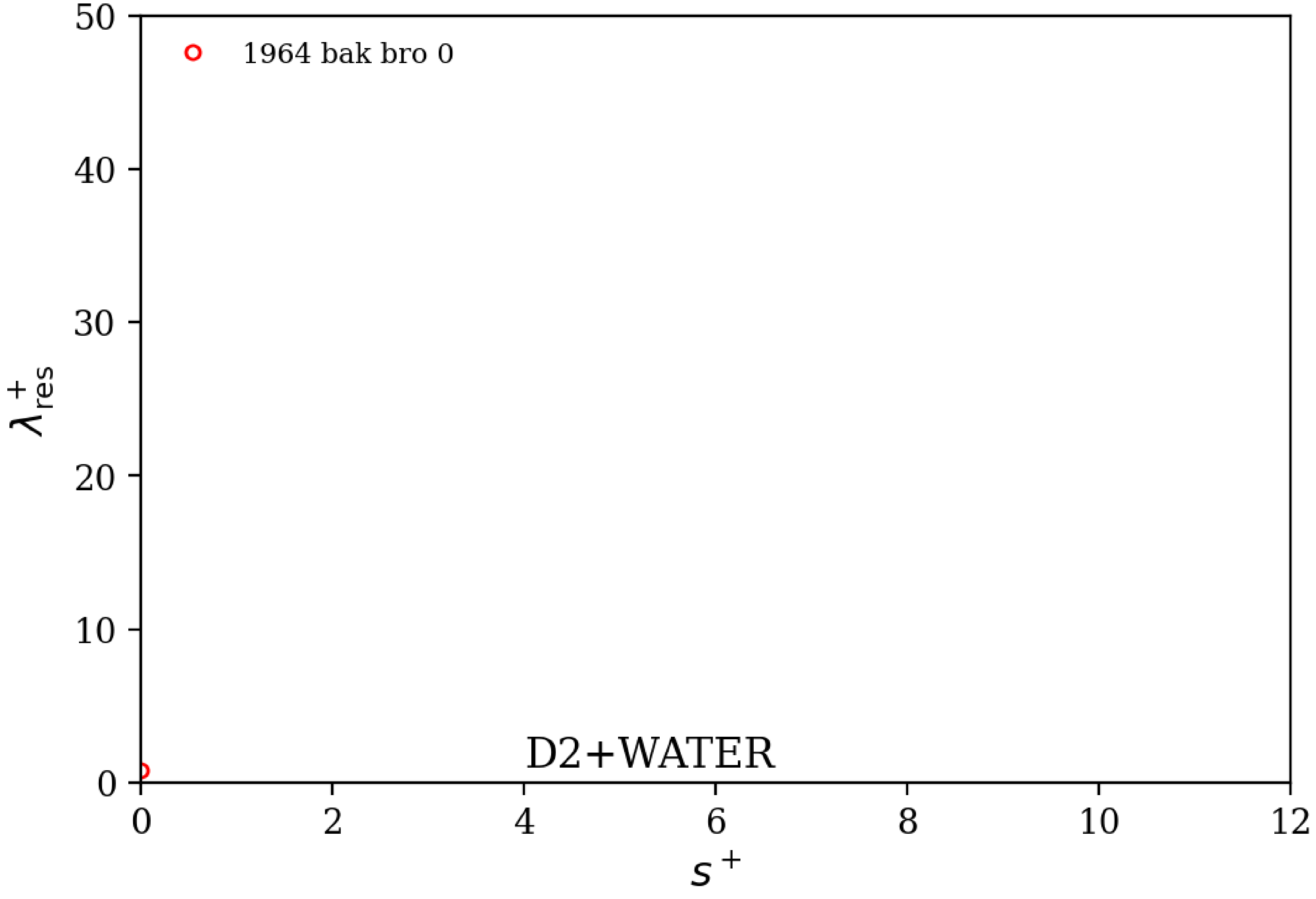

Figure DPR3. D2+WATER

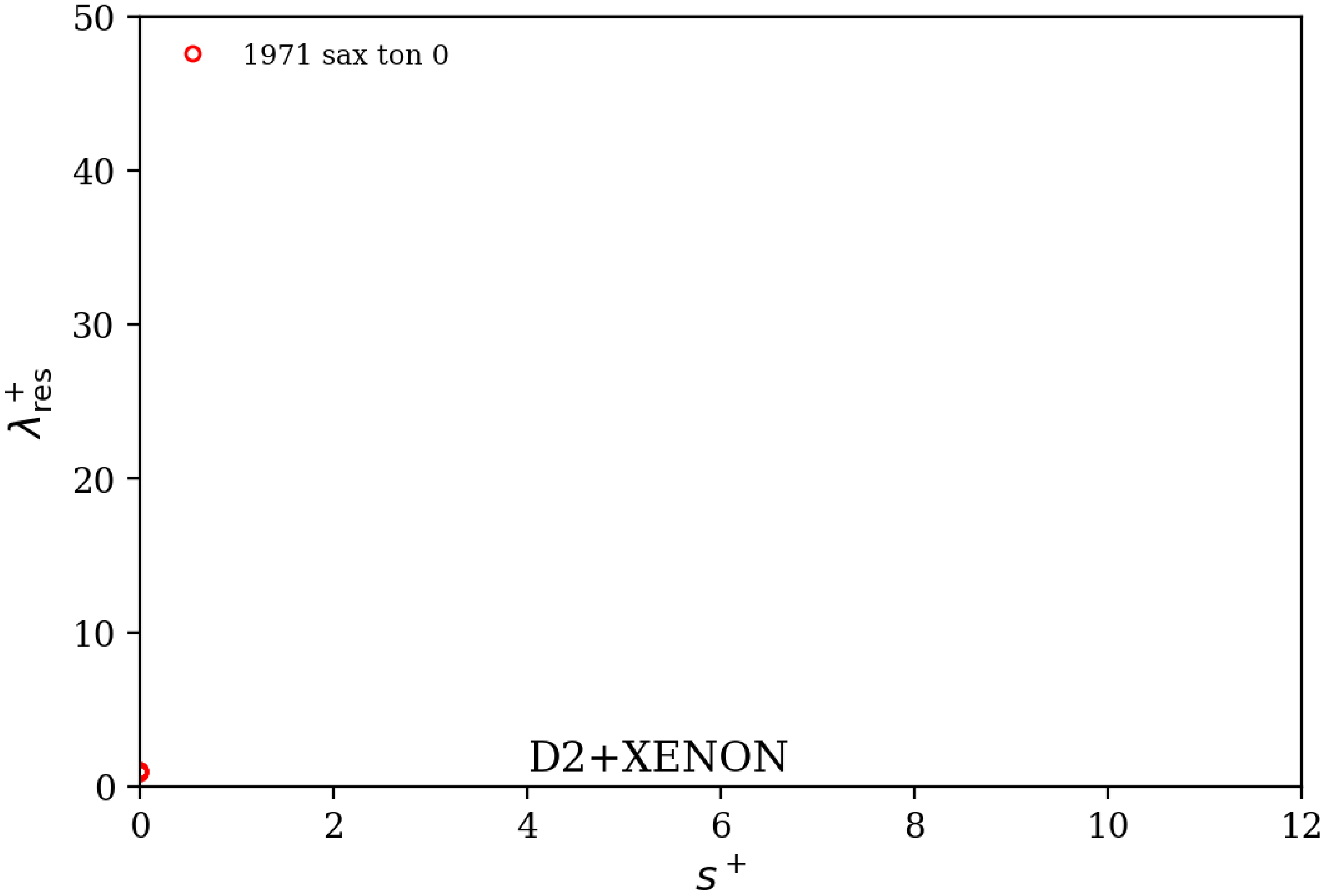

Figure DPR3. D2+XENON

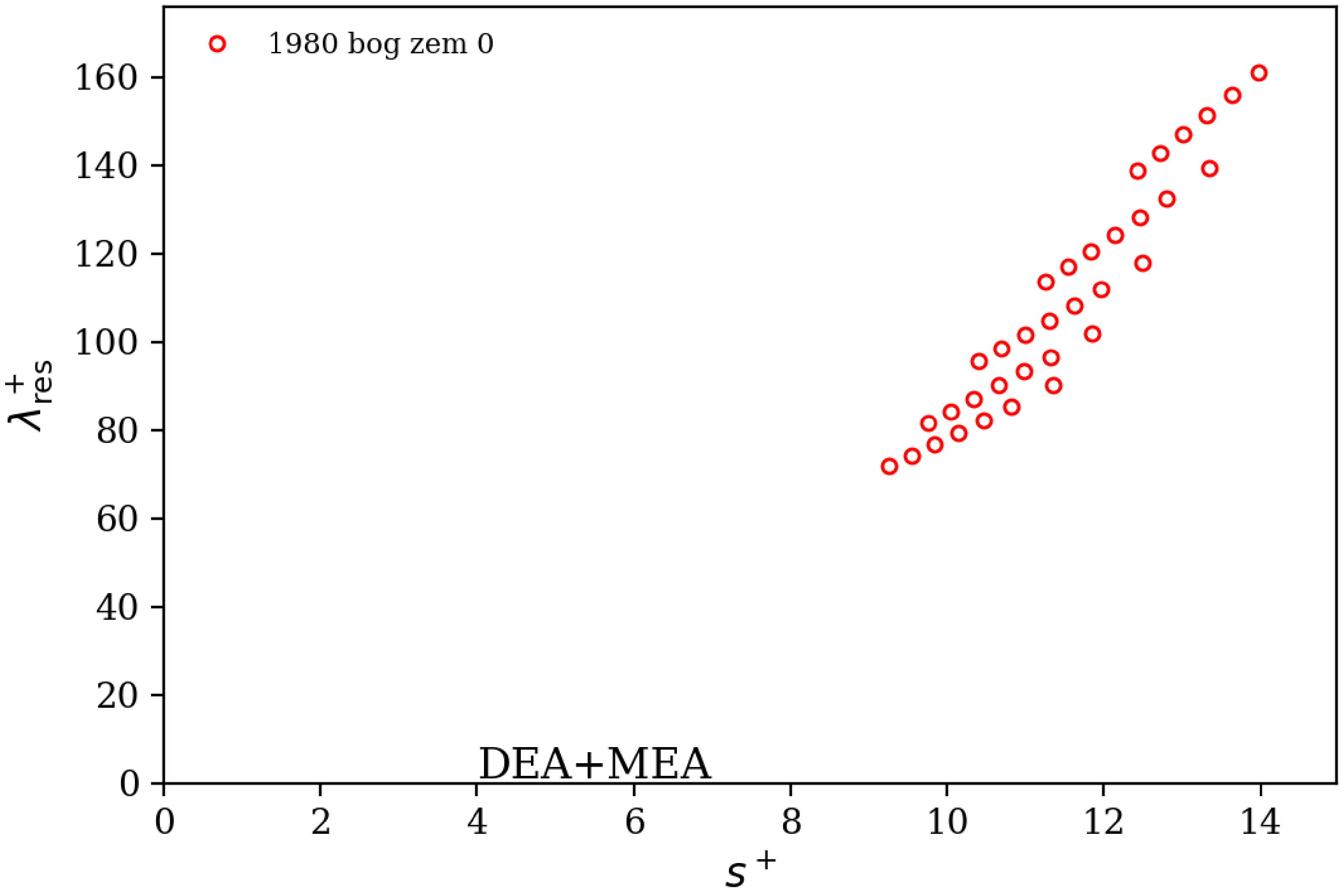

Figure DPR3. DEA+MEA

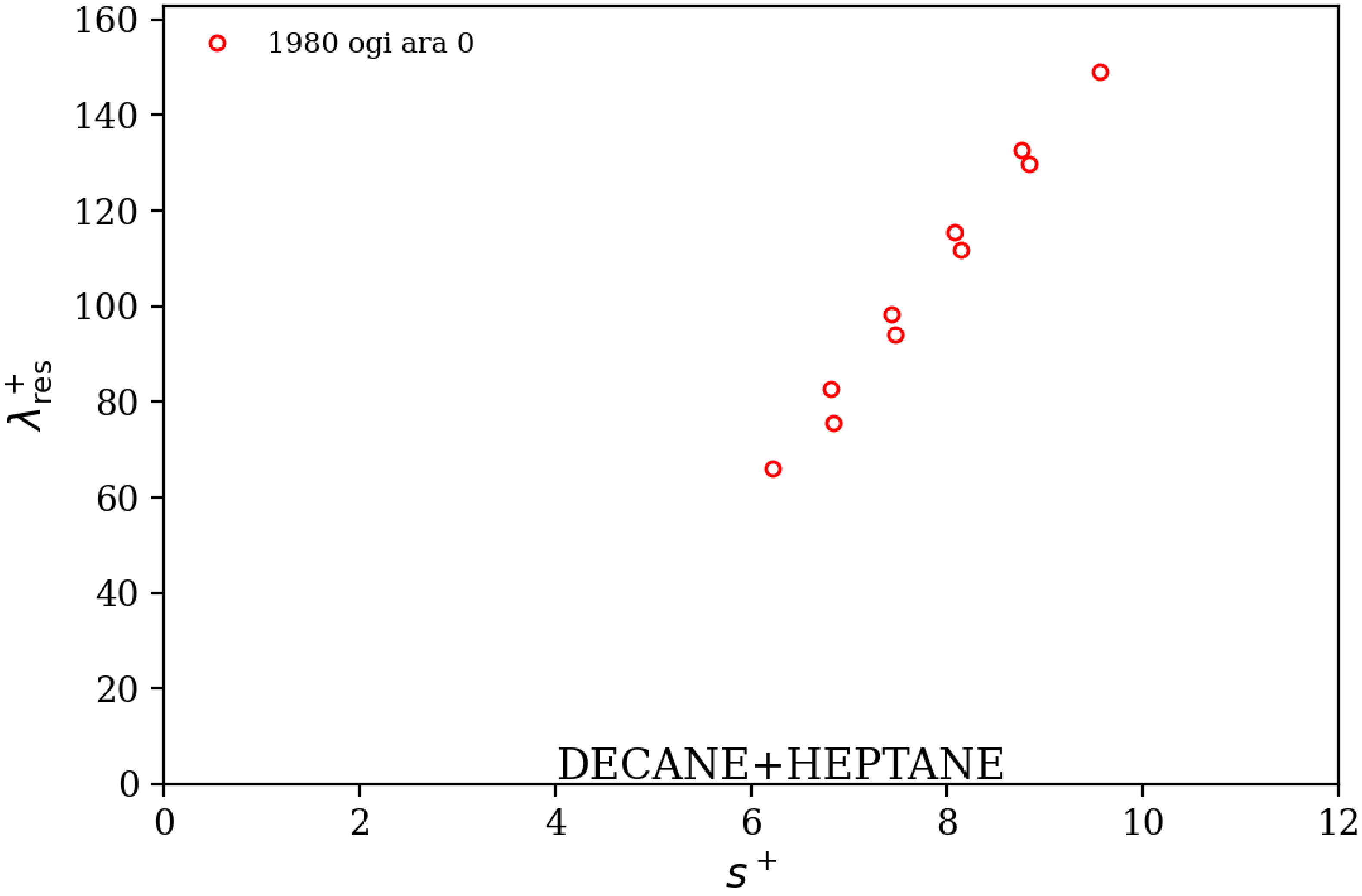

Figure DPR3. DECANE+HEPTANE

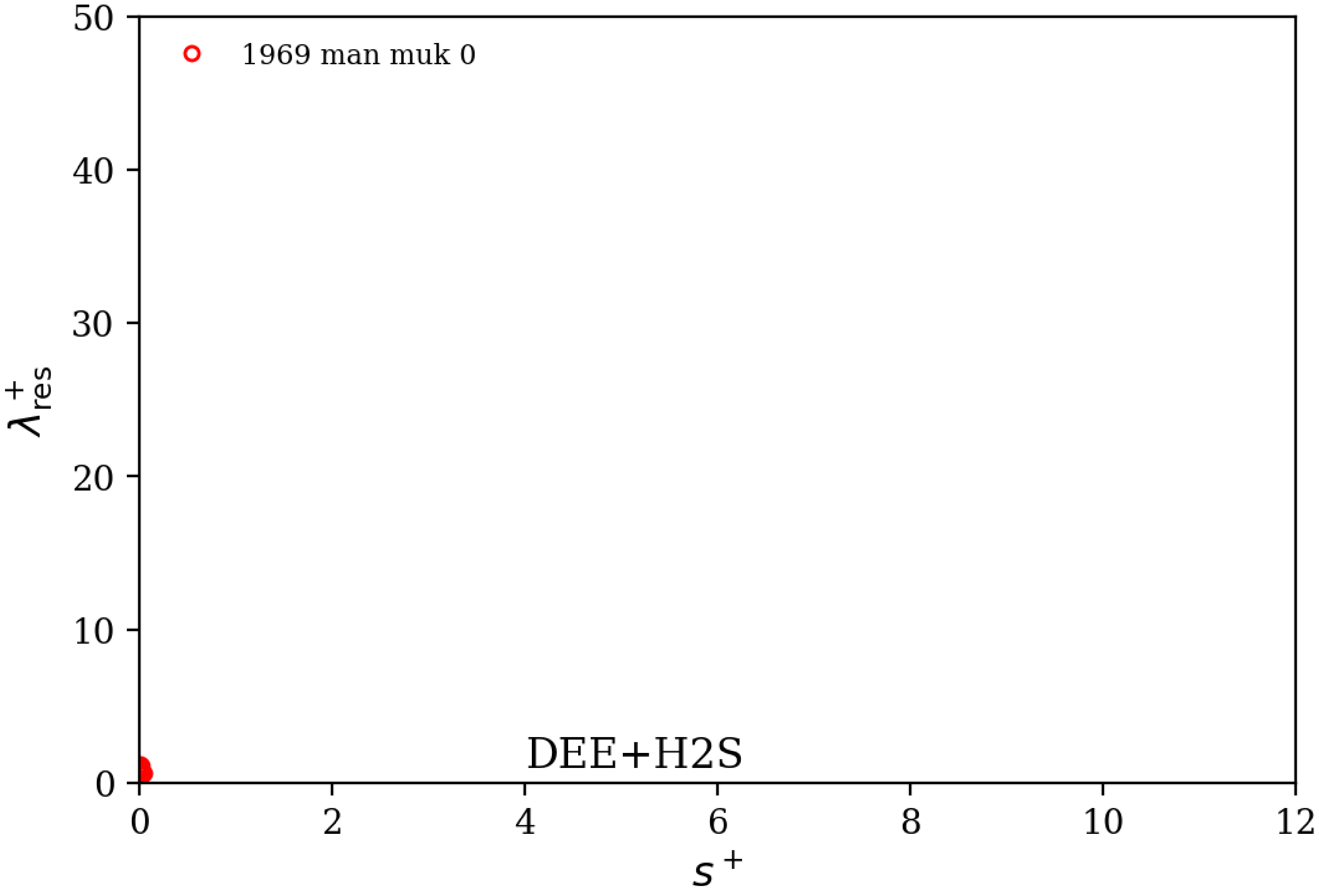

Figure DPR3. DEE+H2S

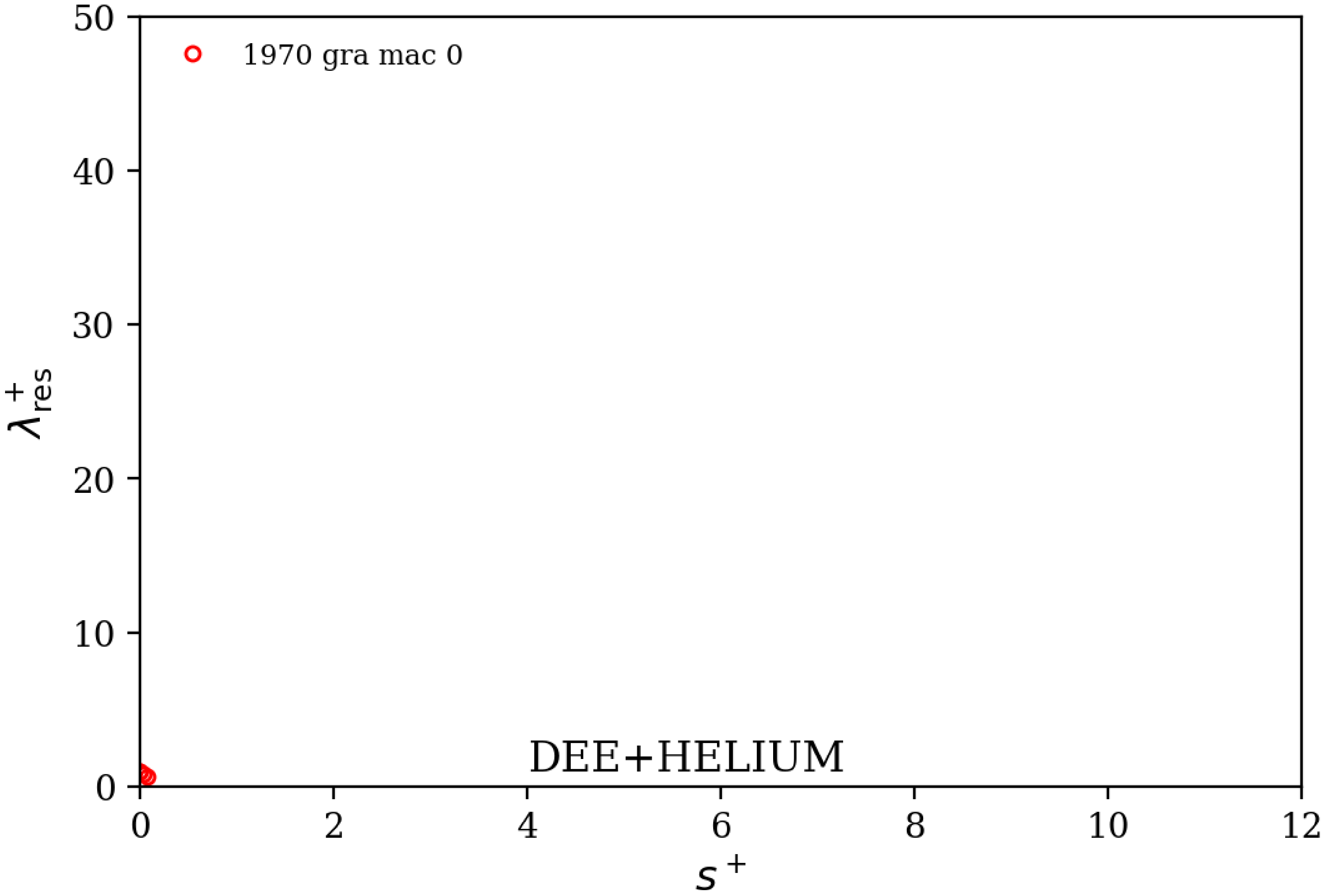

Figure DPR3. DEE+HELIUM

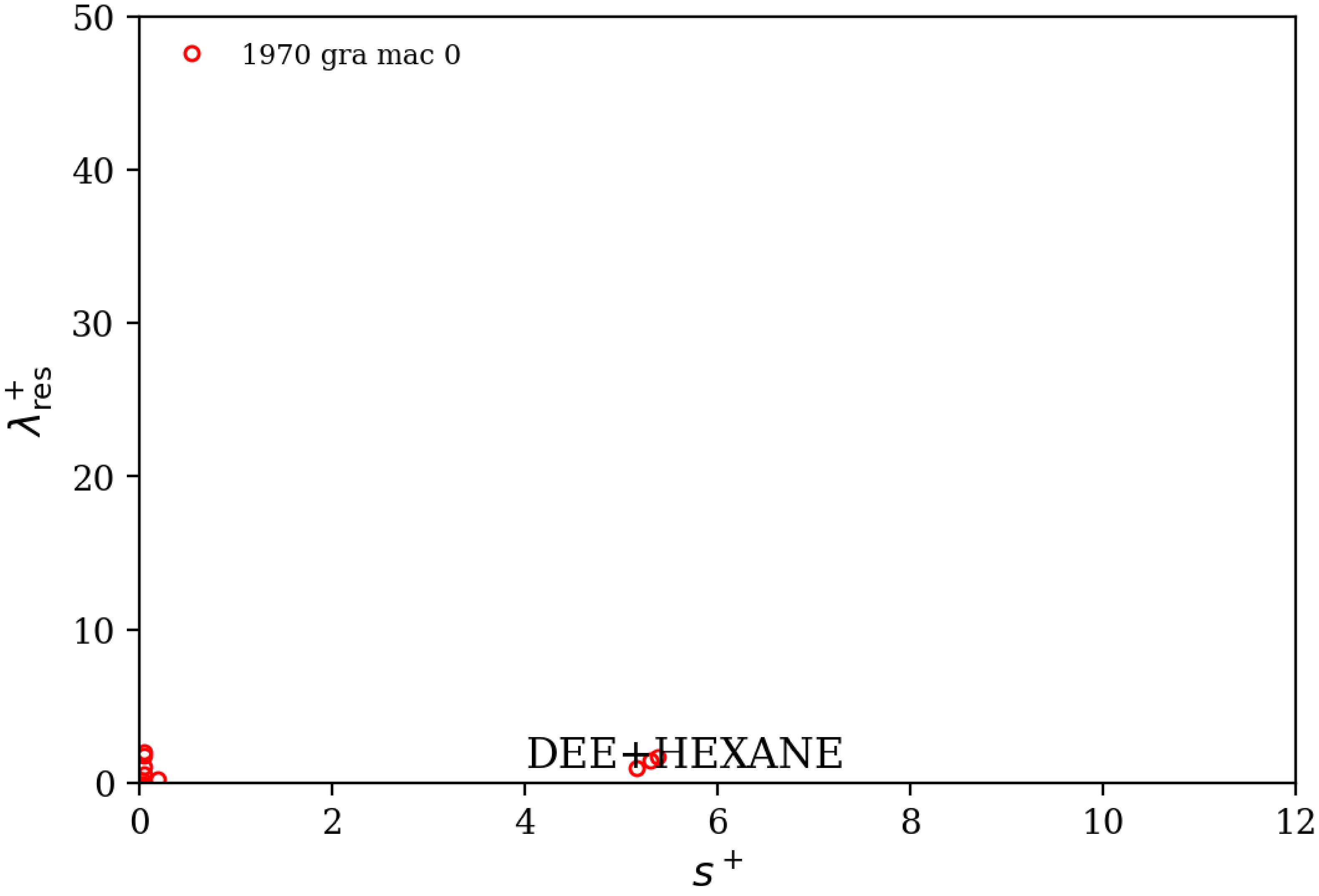

Figure DPR3. DEE+HEXANE

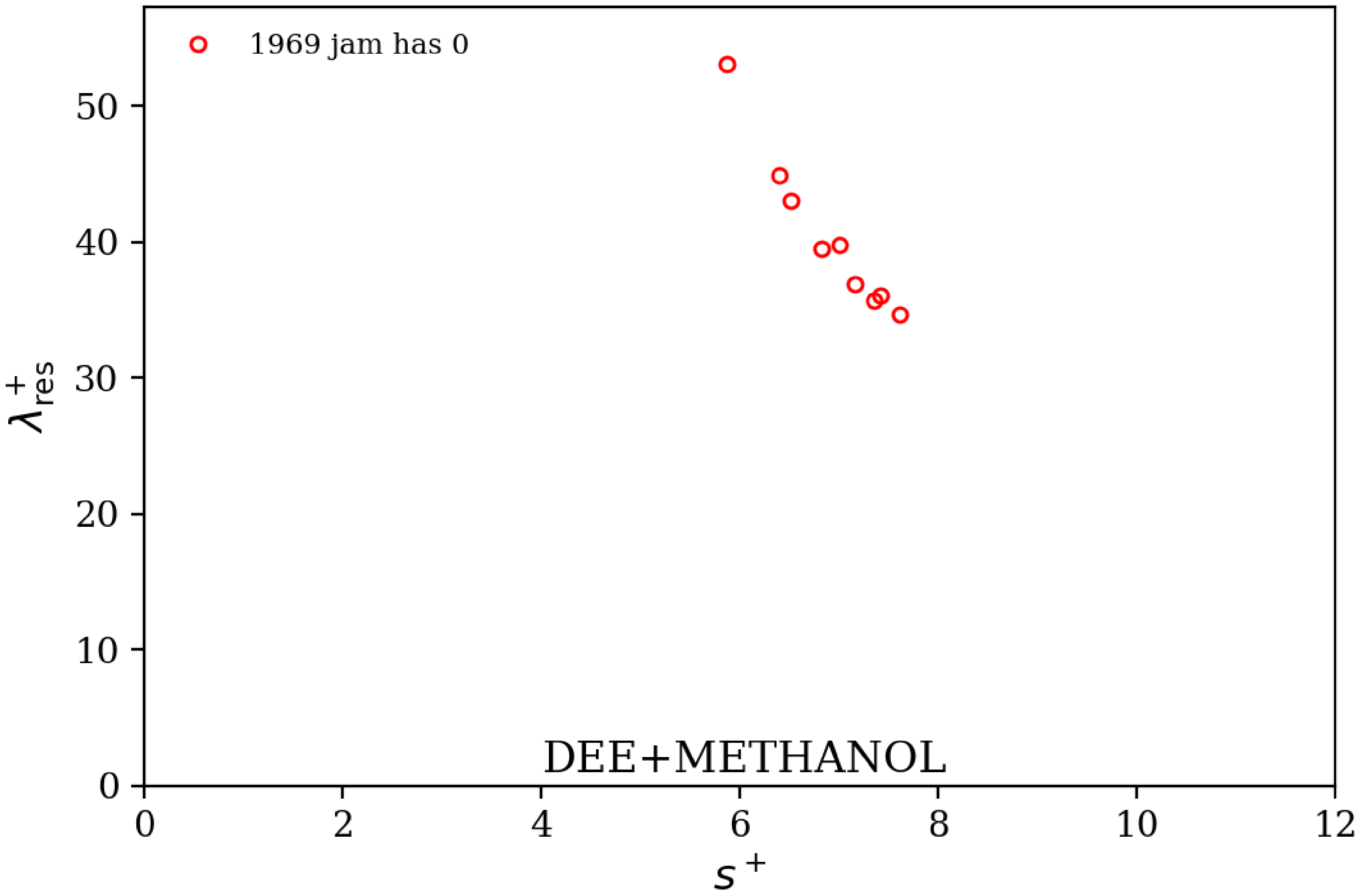

Figure DPR3. DEE+METHANOL

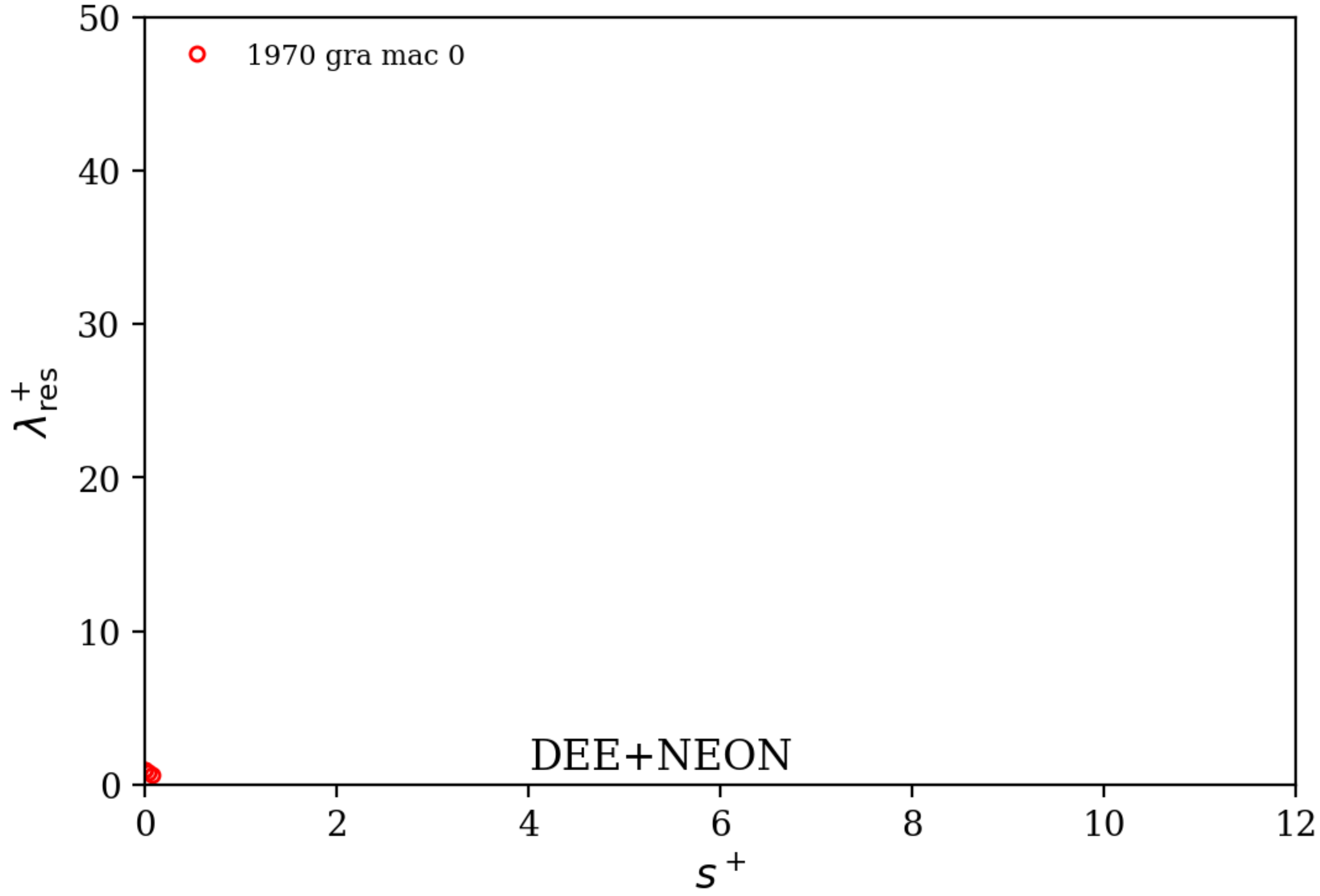

Figure DPR3. DEE+NEON

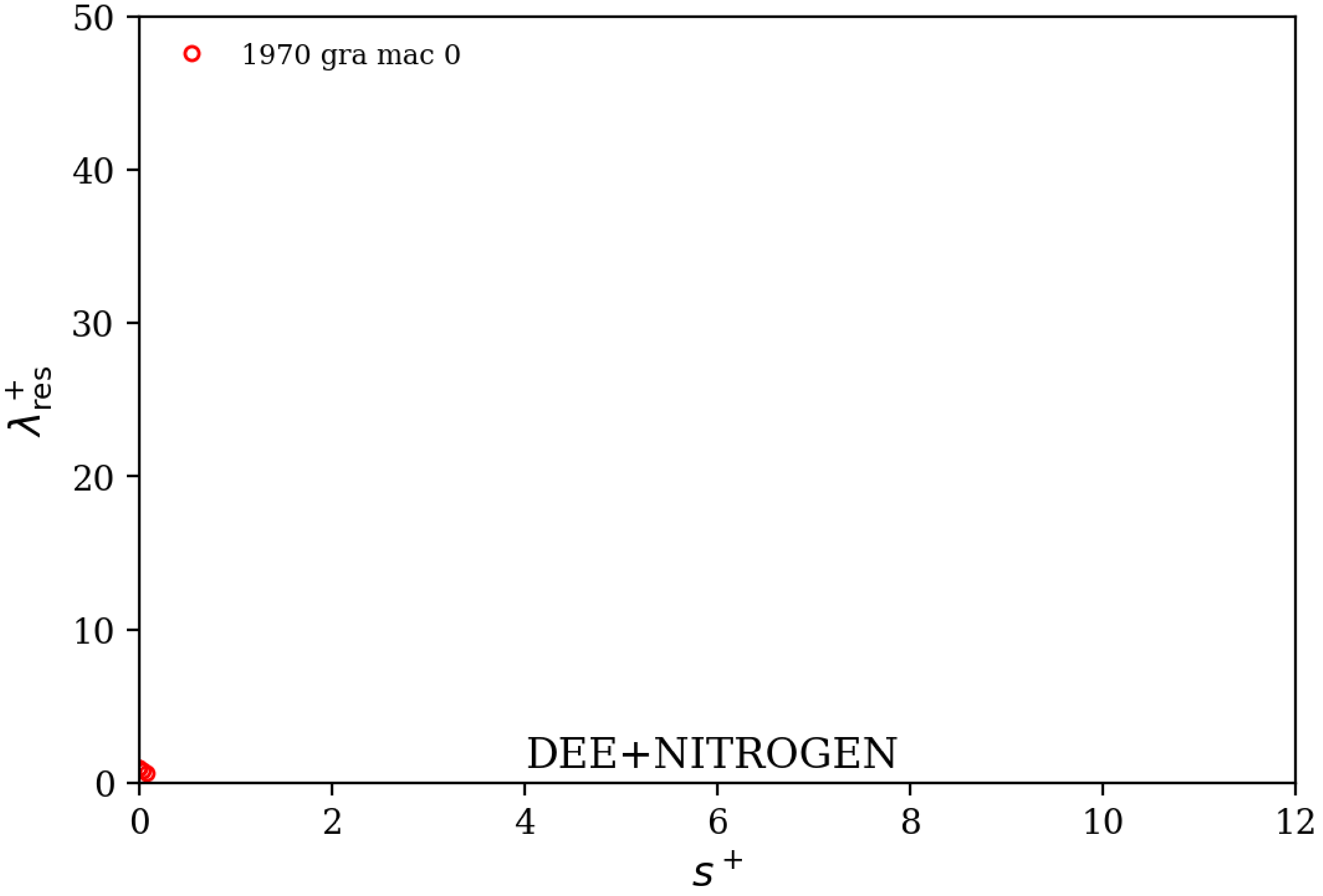

Figure DPR3. DEE+NITROGEN

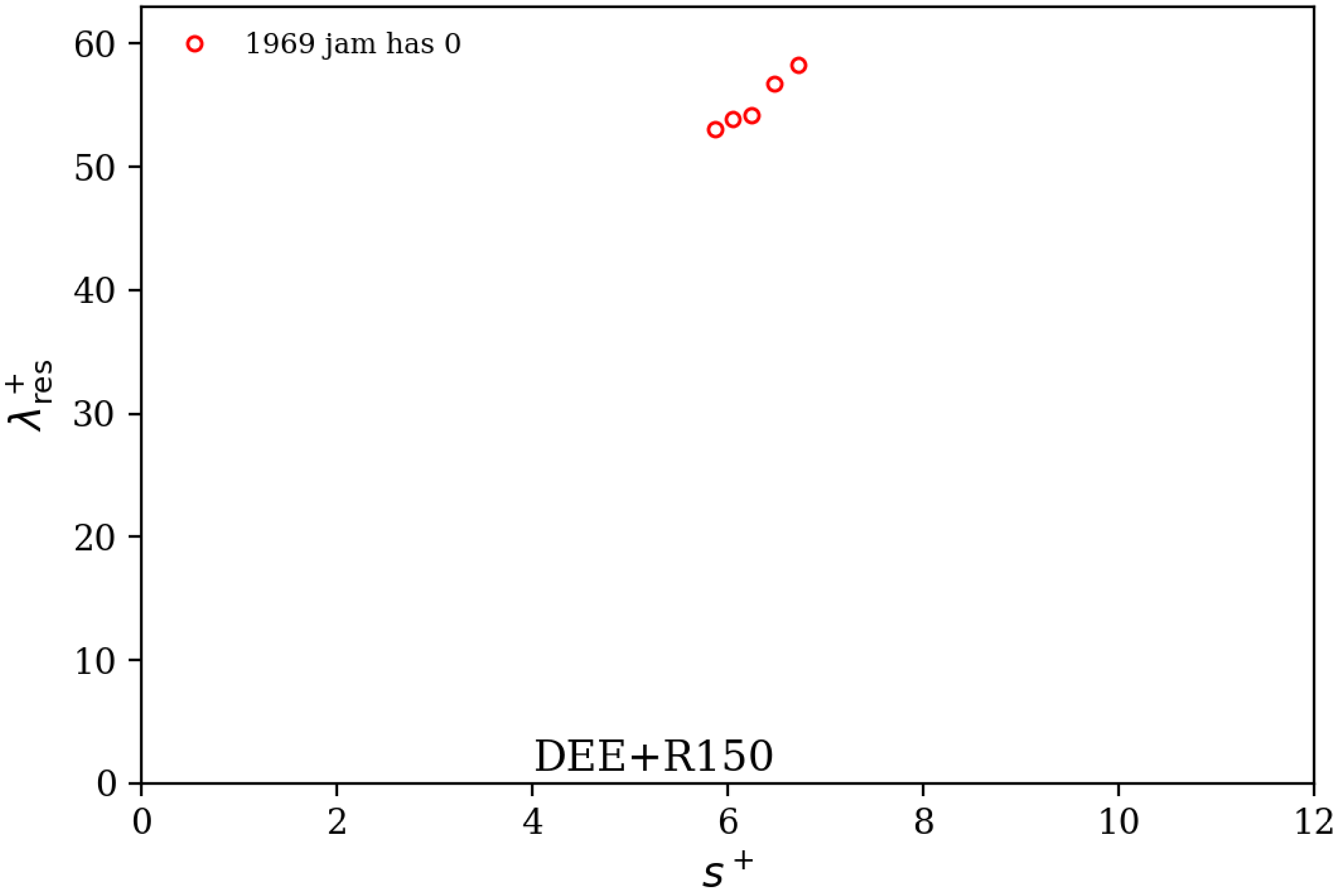

Figure DPR3. DEE+R150

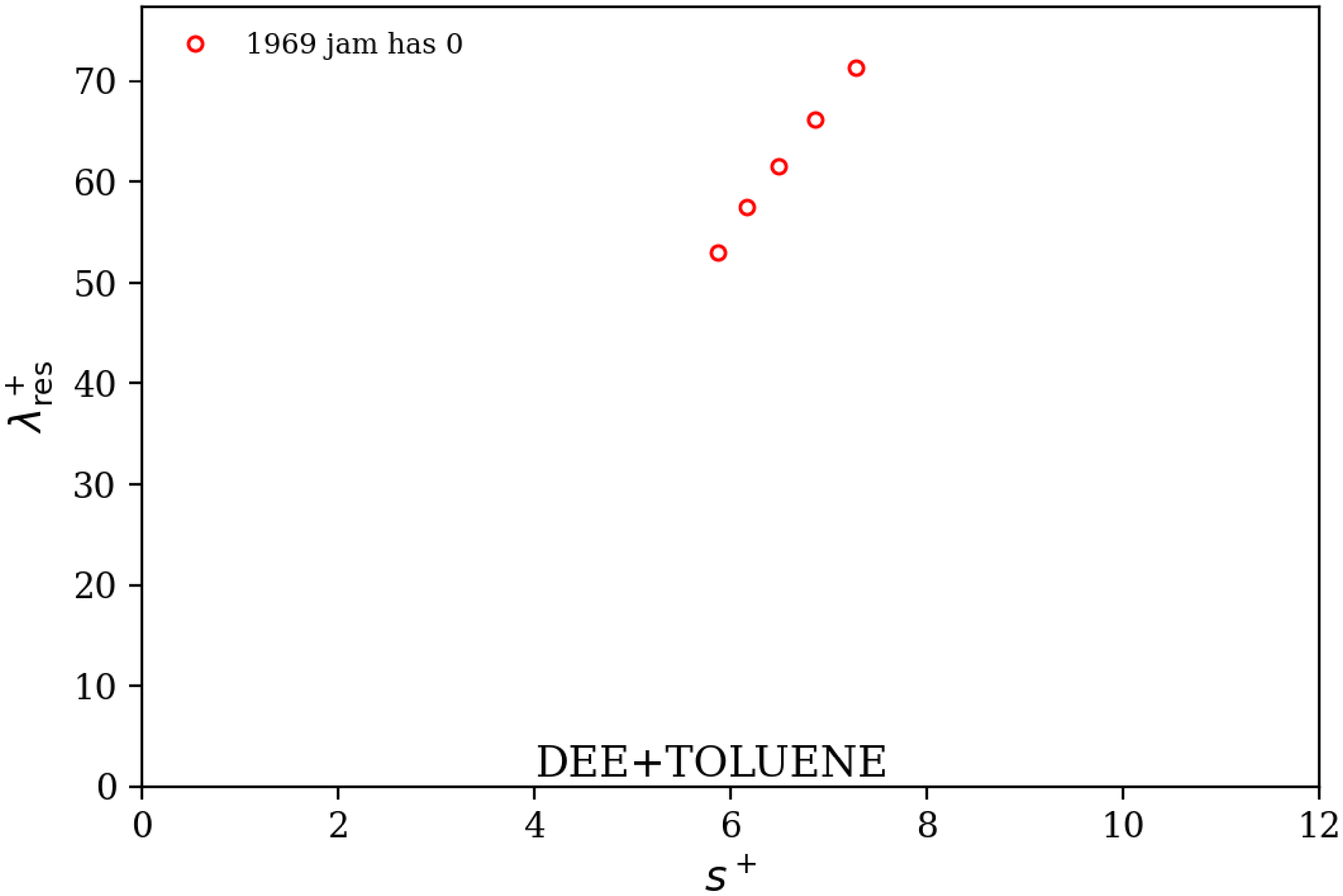

Figure DPR3. DEE+TOLUENE

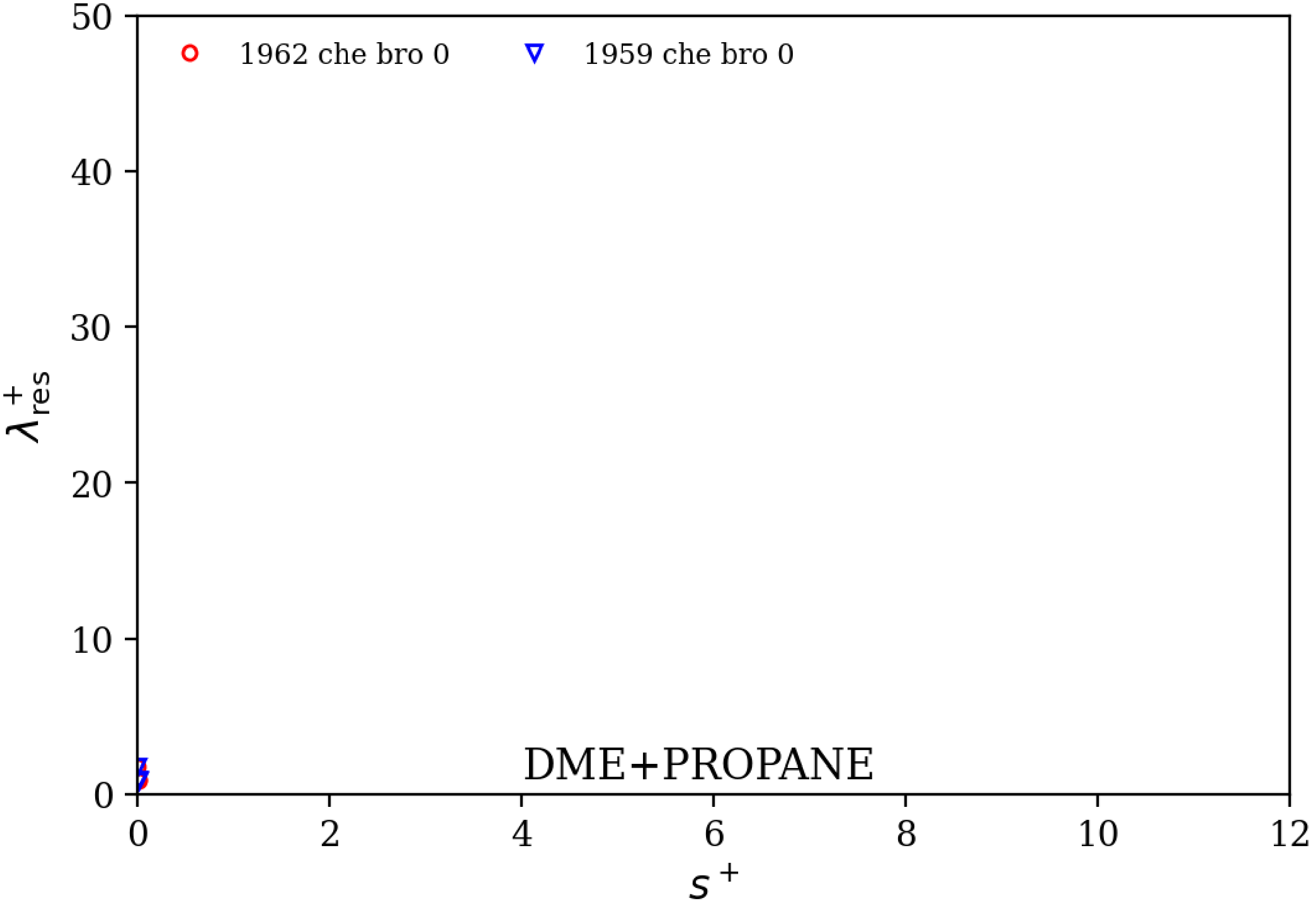

Figure DPR3. DME+PROPANE

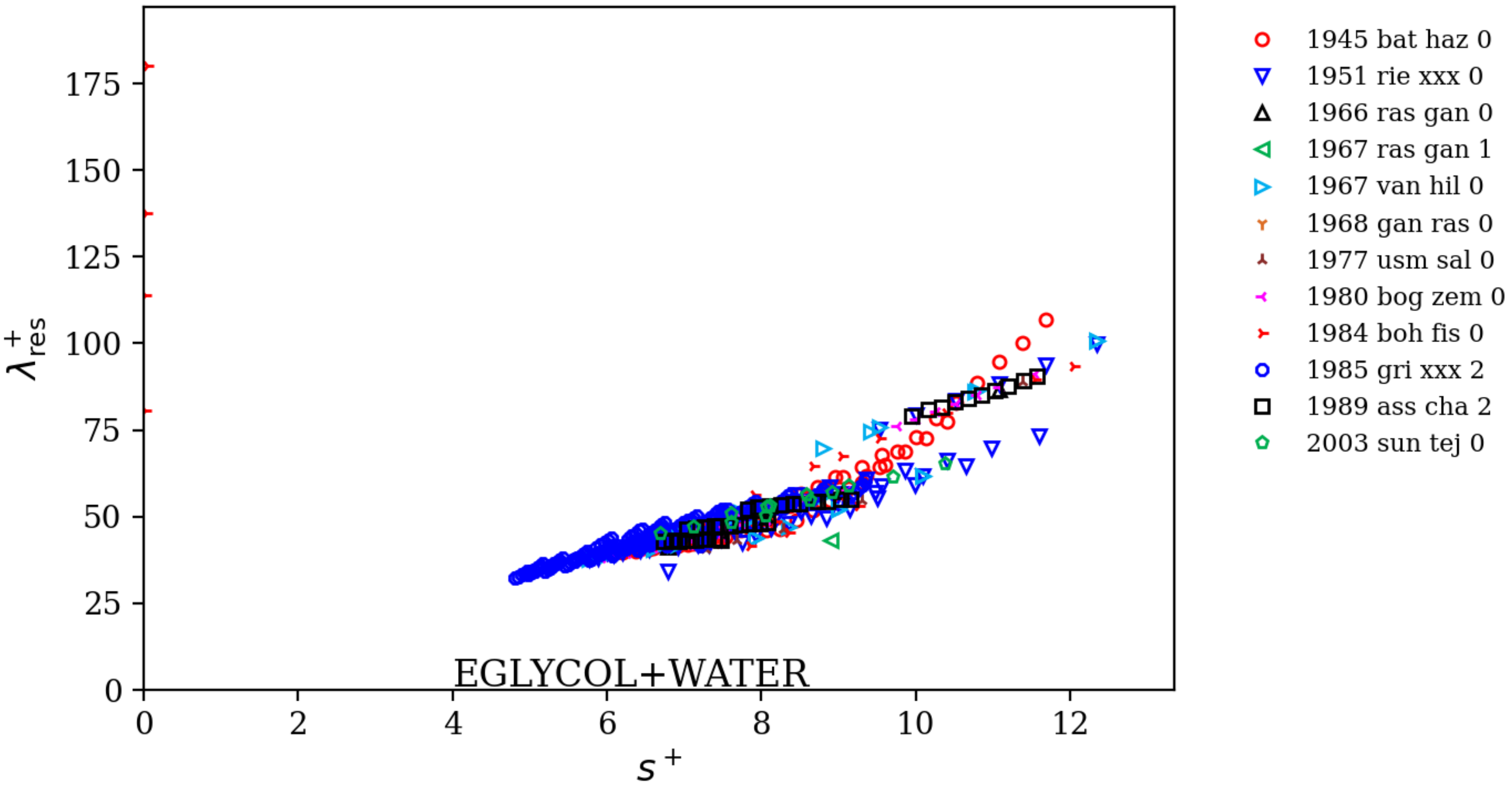

Figure DPR3. EGLYCOL+WATER

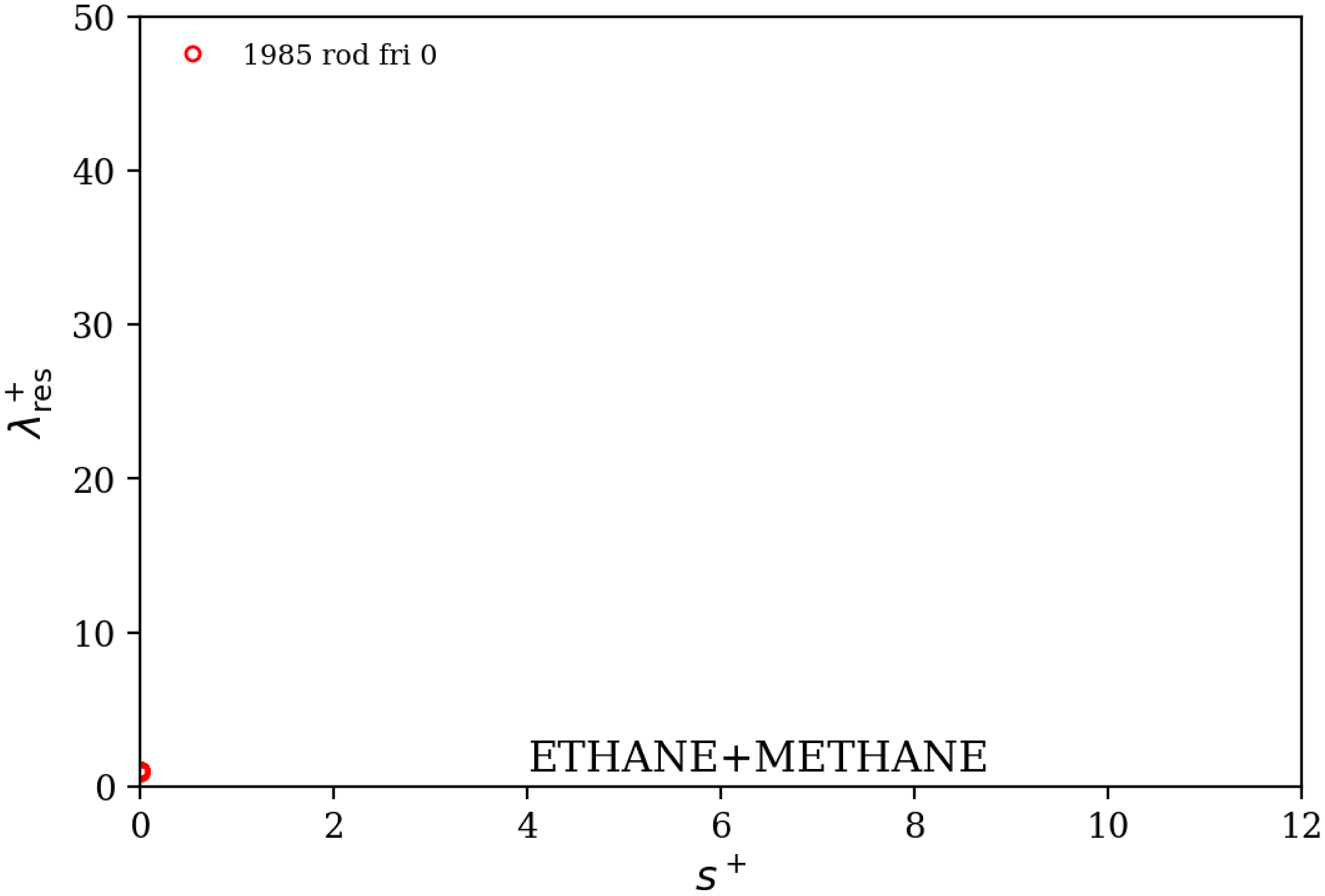

Figure DPR3. ETHANE+METHANE

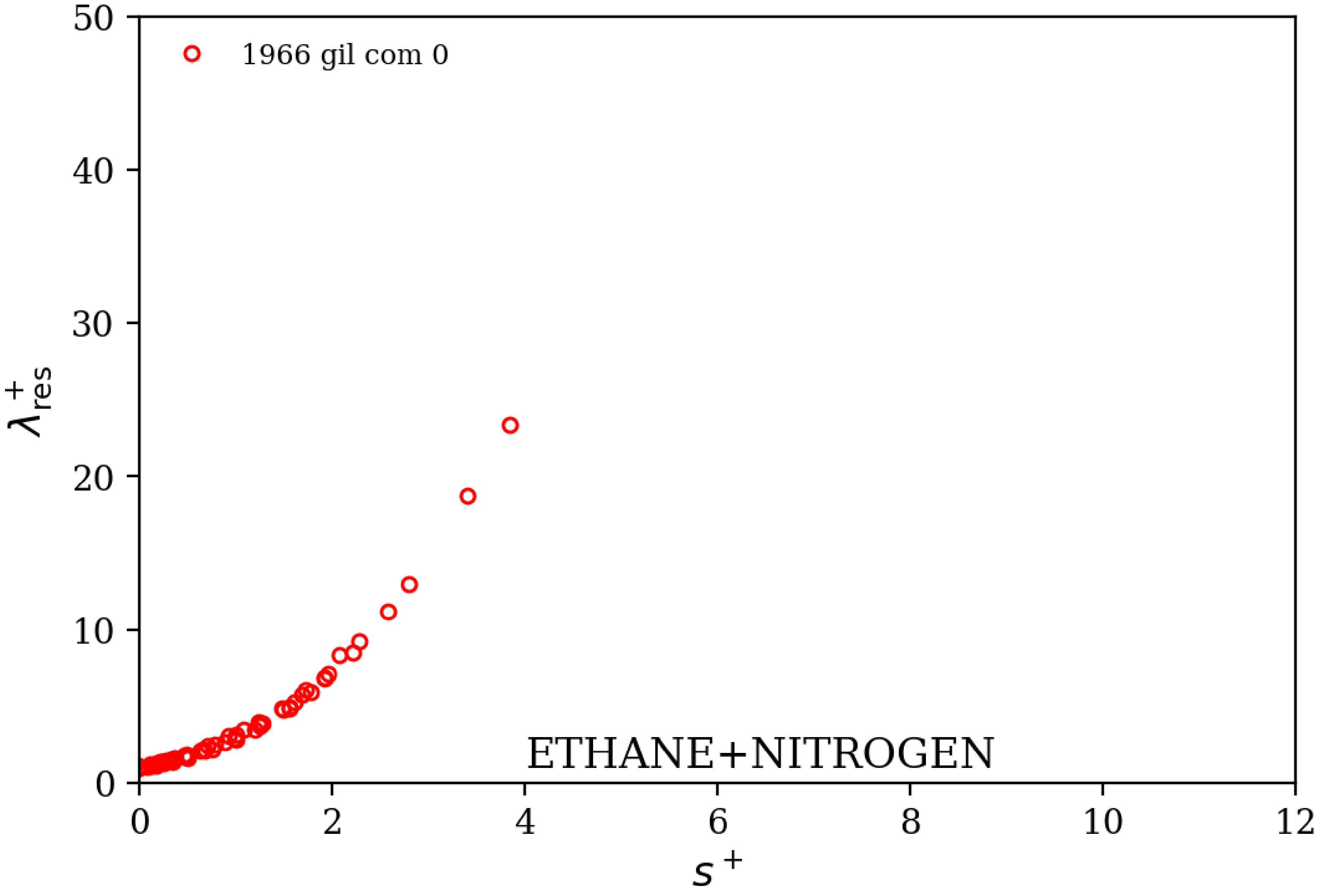

Figure DPR3. ETHANE+NITROGEN

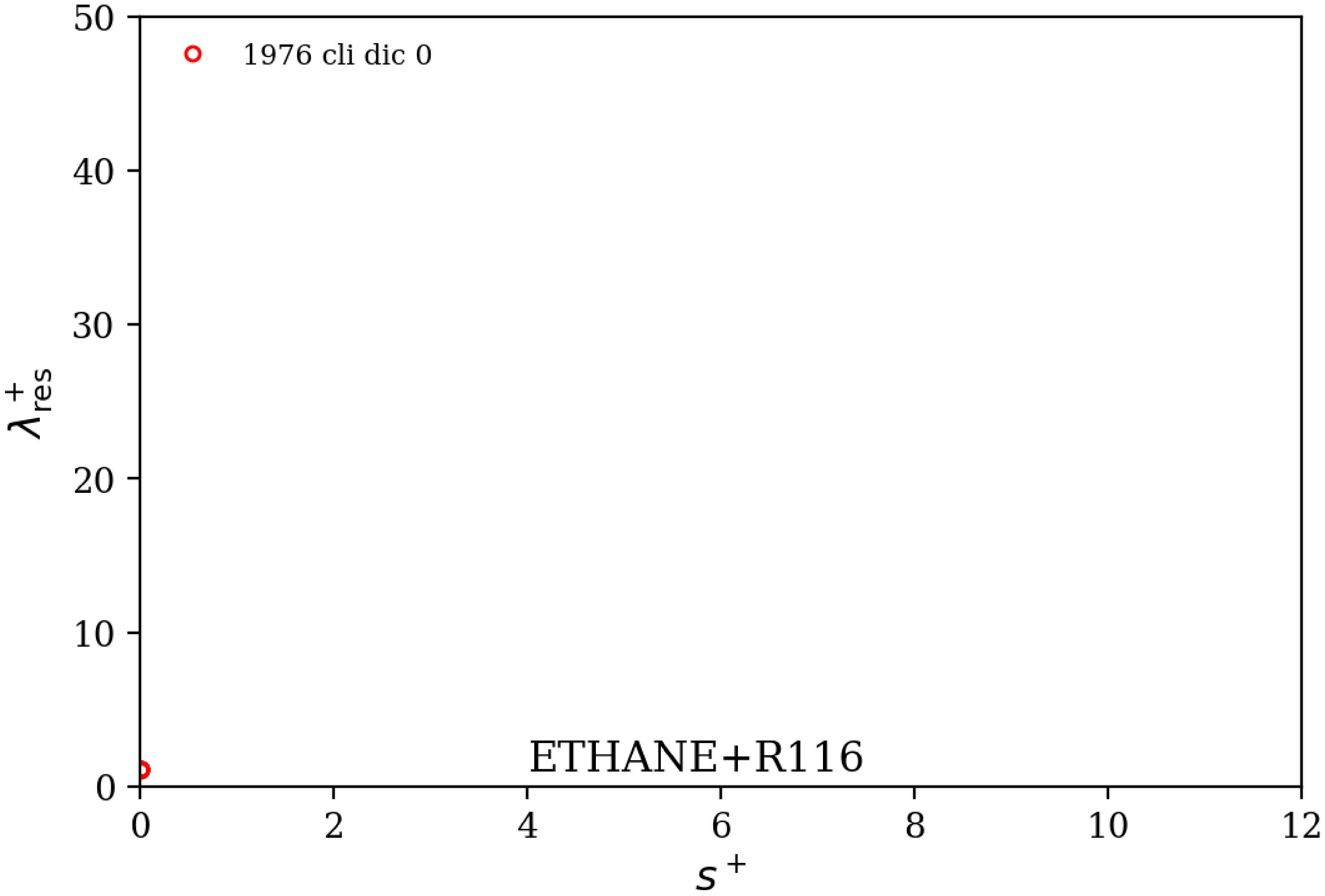

Figure DPR3. ETHANE+R116

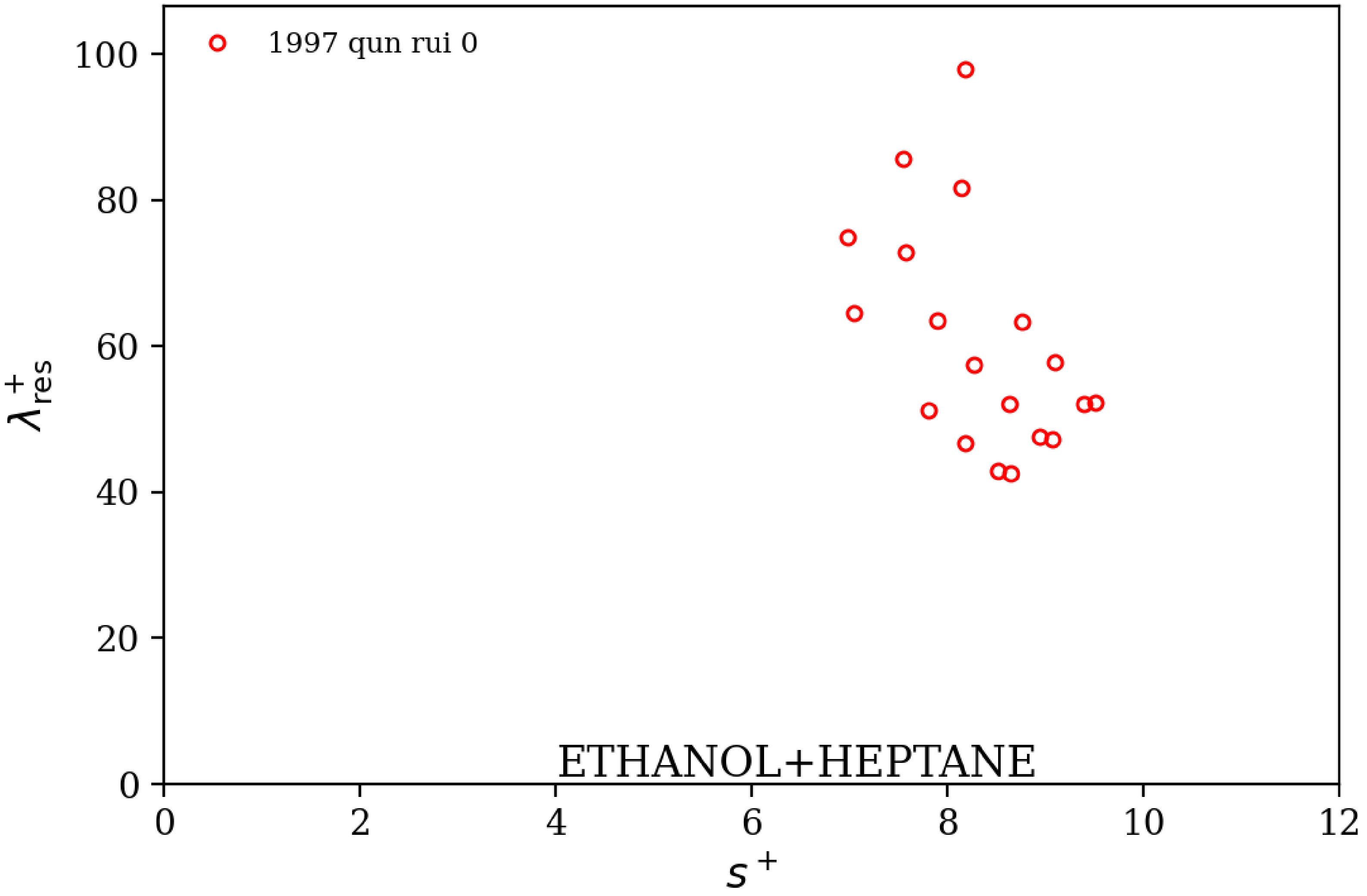

Figure DPR3. ETHANOL+HEPTANE

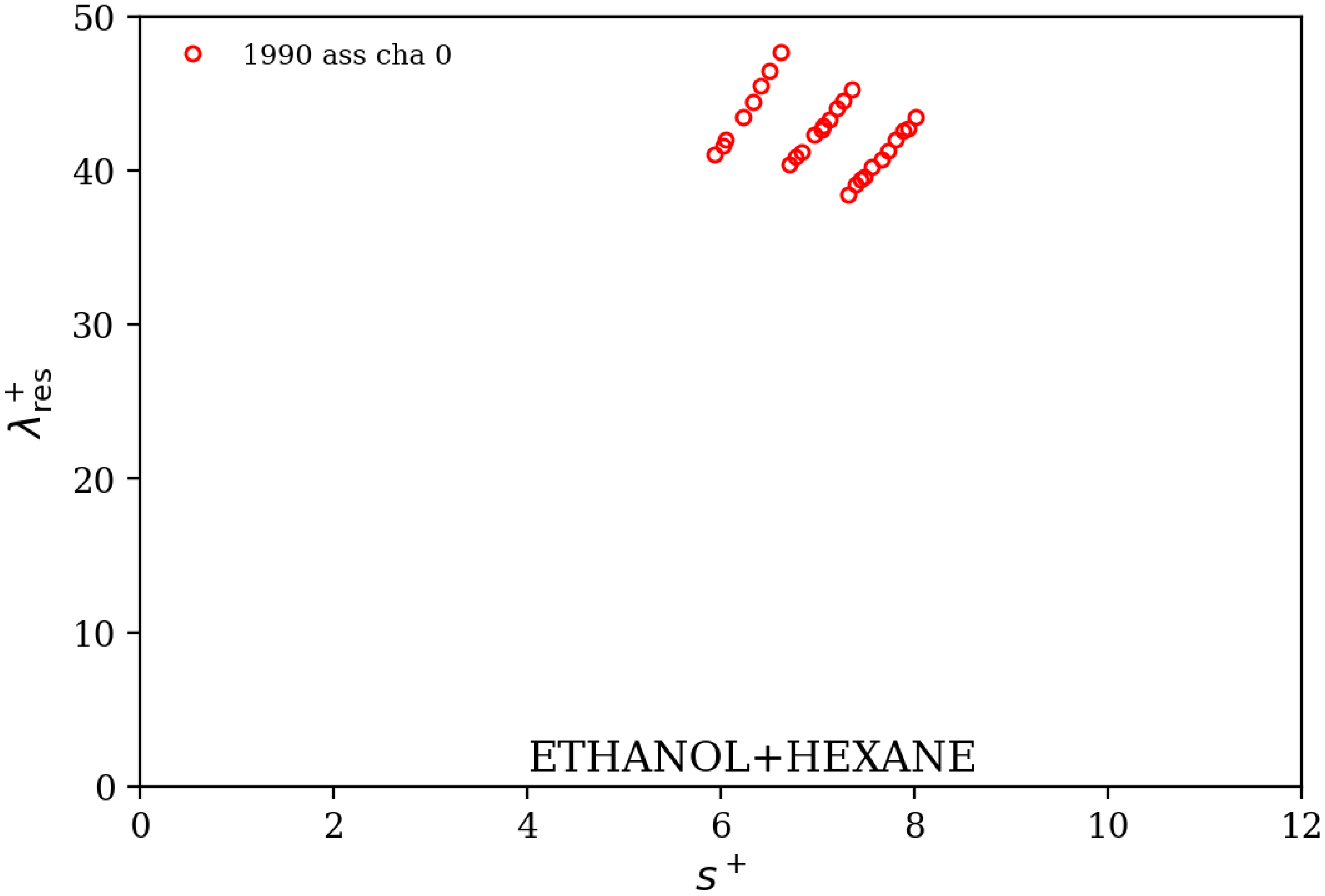

Figure DPR3. ETHANOL+HEXANE

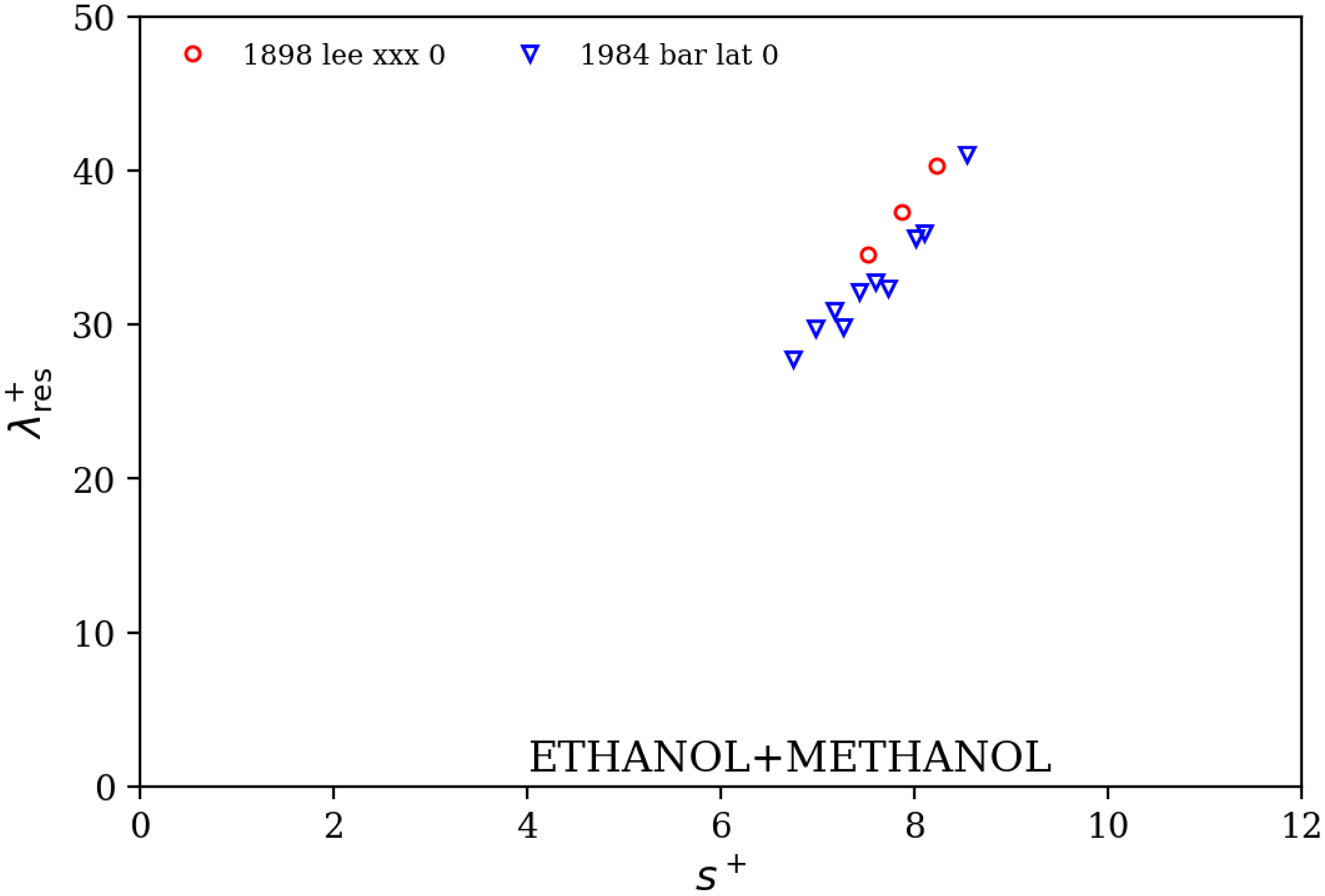

Figure DPR3. ETHANOL+METHANOL

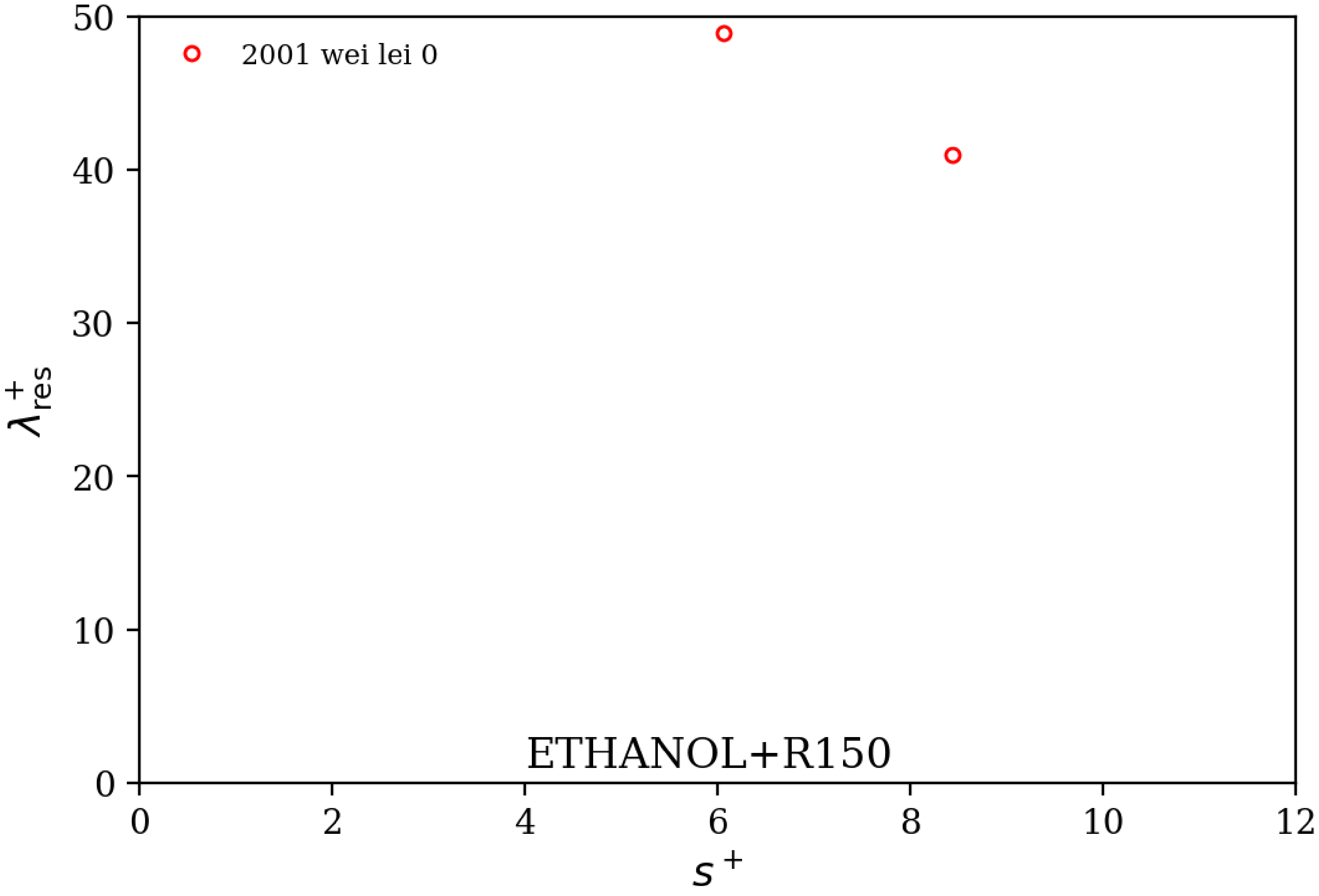

Figure DPR3. ETHANOL+R150

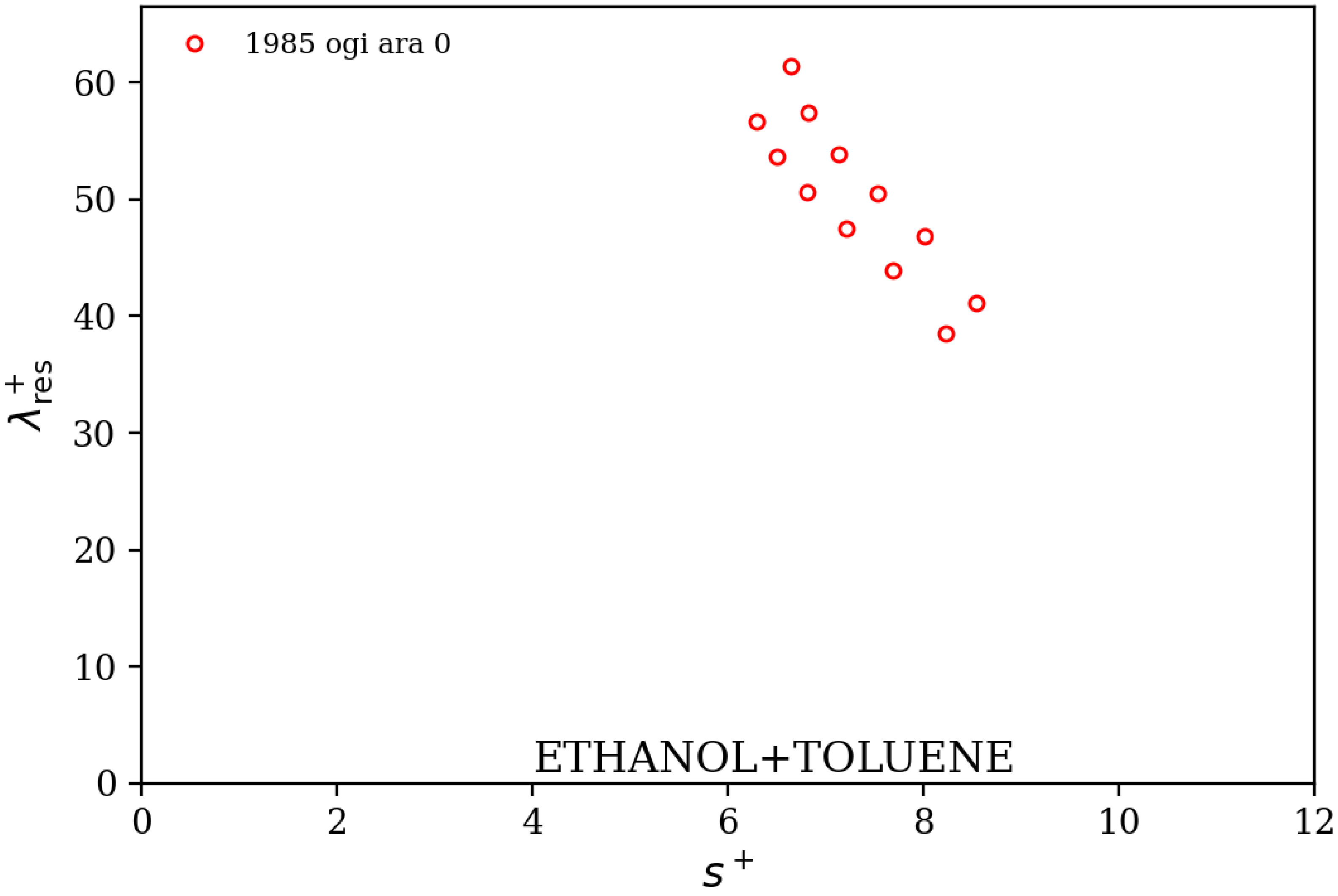

Figure DPR3. ETHANOL+TOLUENE

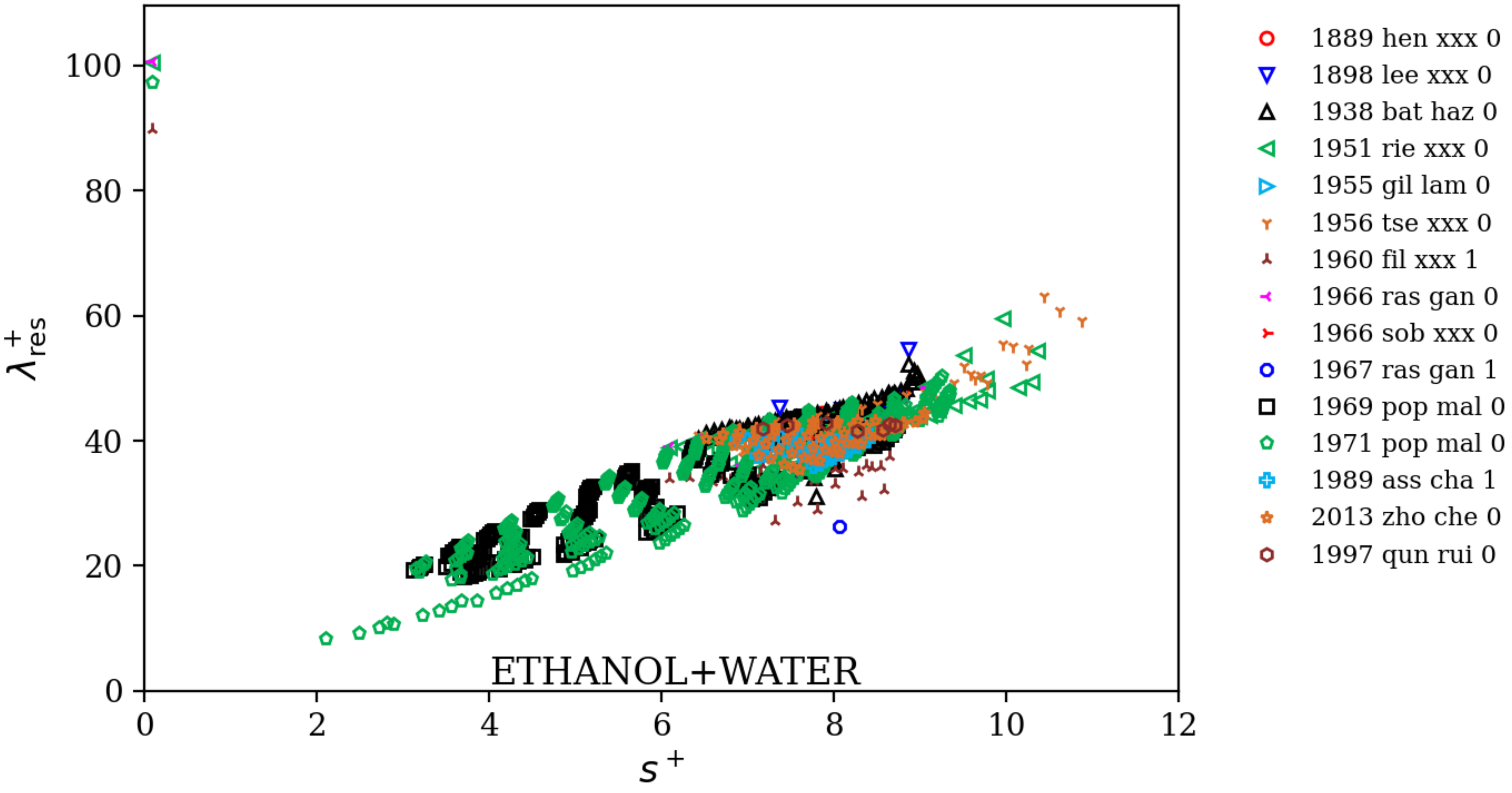

Figure DPR3. ETHANOL+WATER

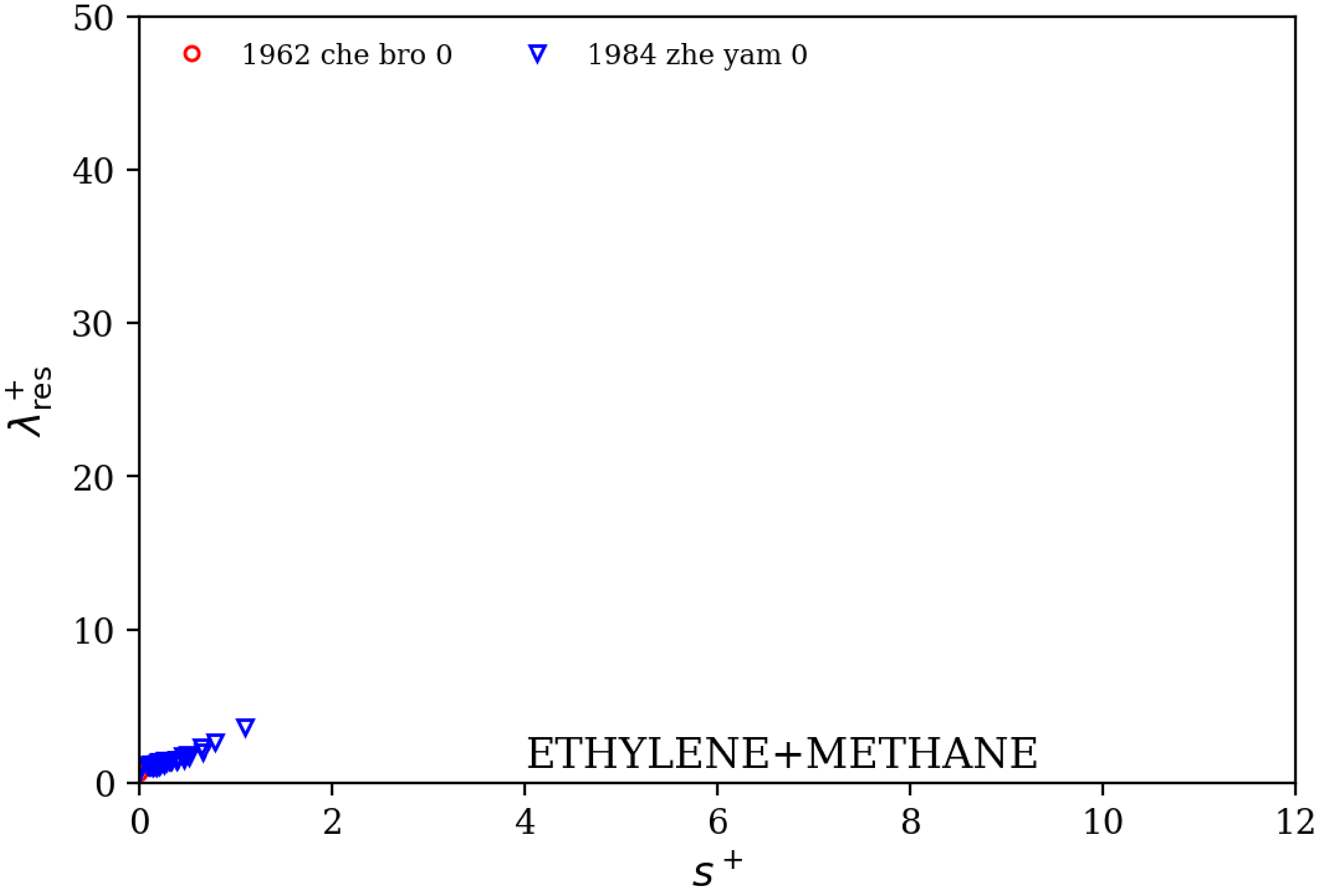

Figure DPR3. ETHYLENE+METHANE

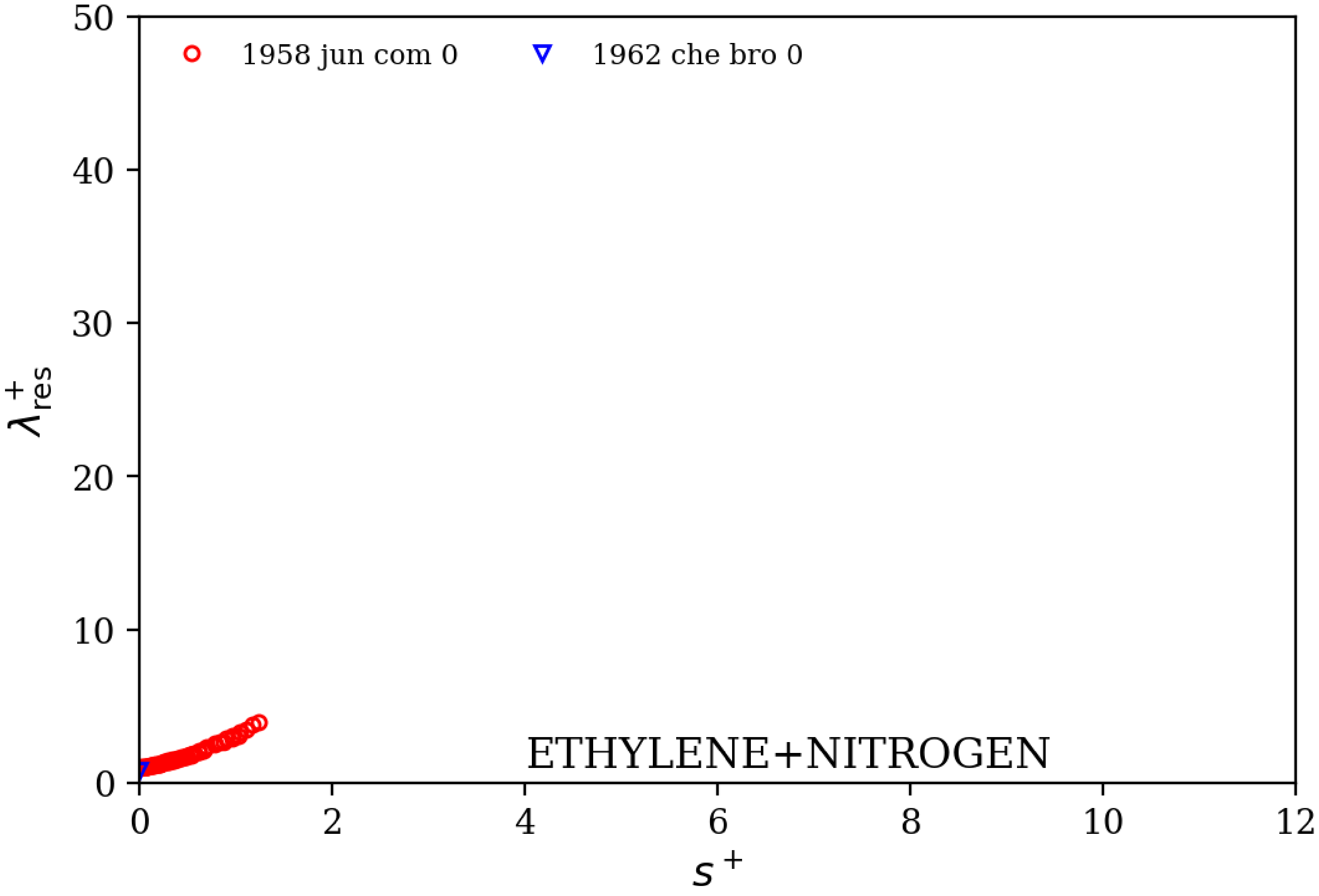

Figure DPR3. ETHYLENE+NITROGEN

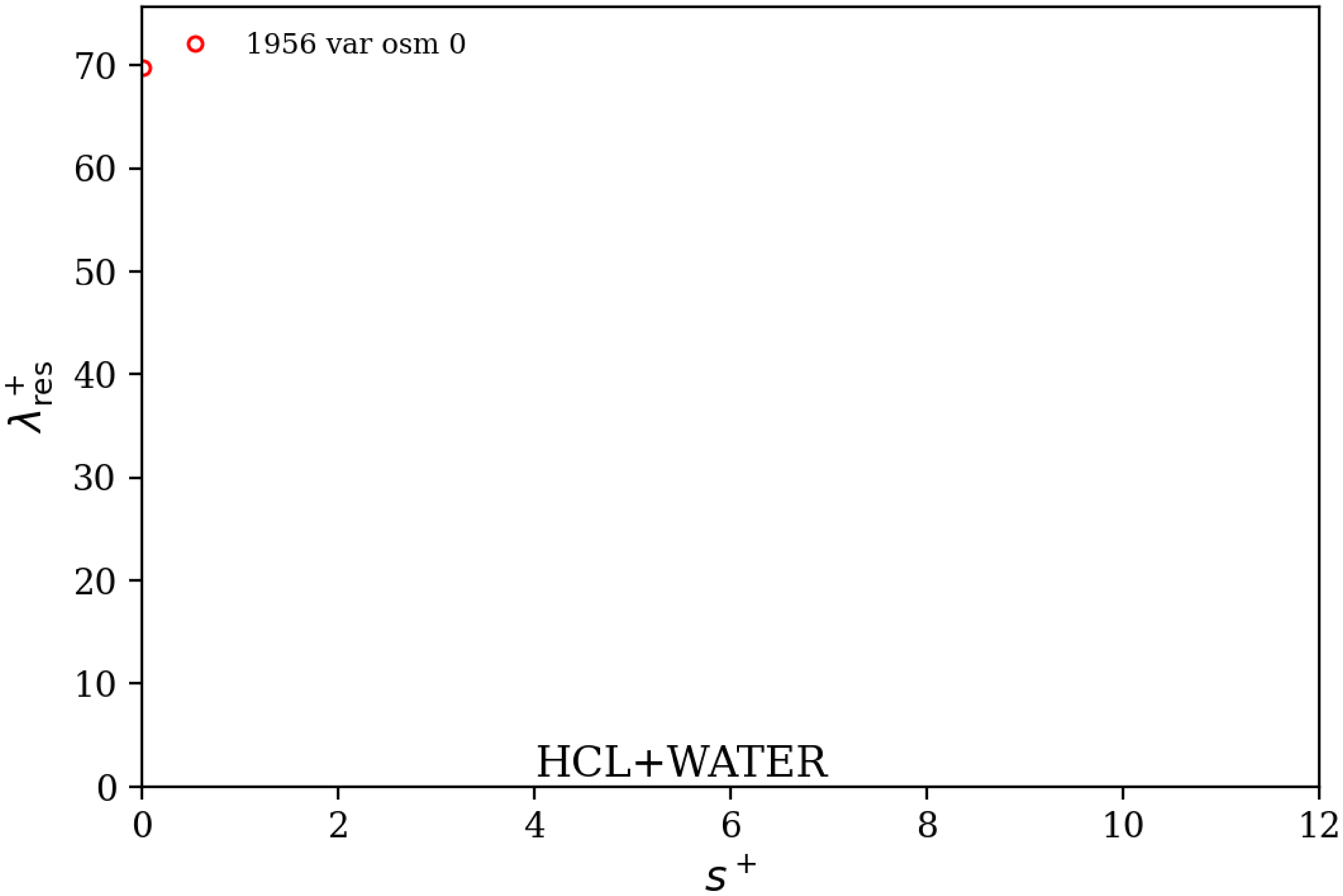

Figure DPR3. HCL+WATER

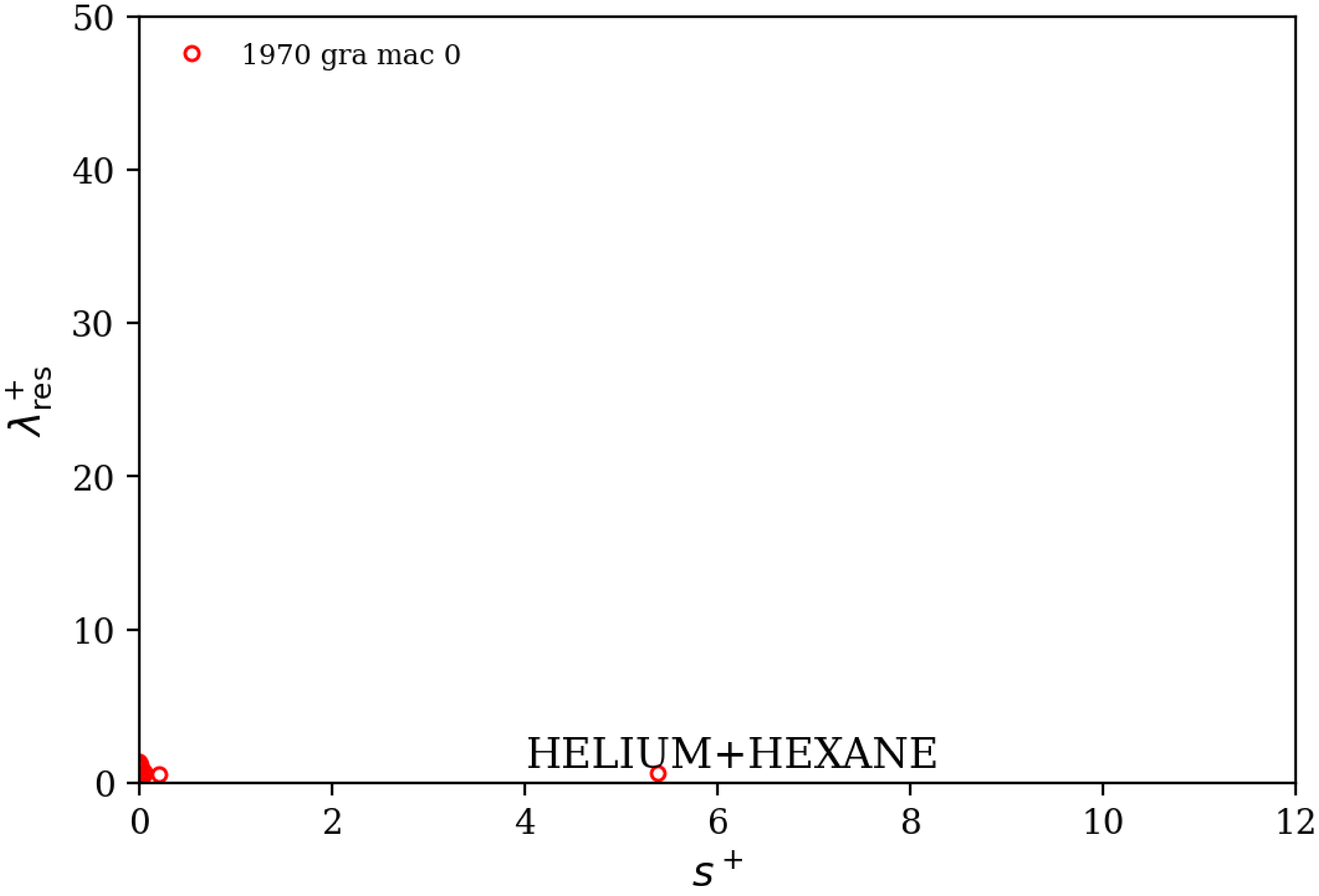

Figure DPR3. HELIUM+HEXANE

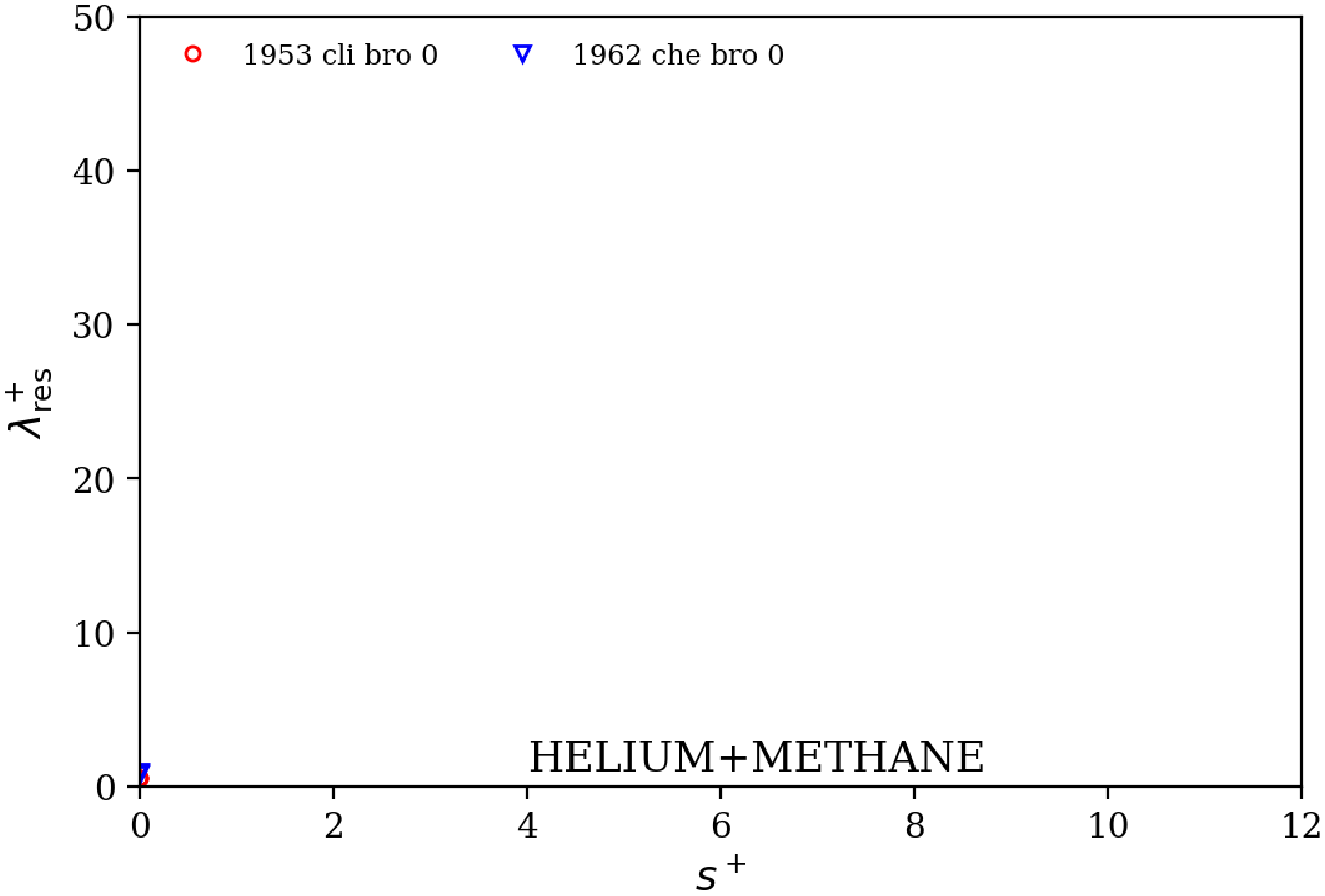

Figure DPR3. HELIUM+METHANE

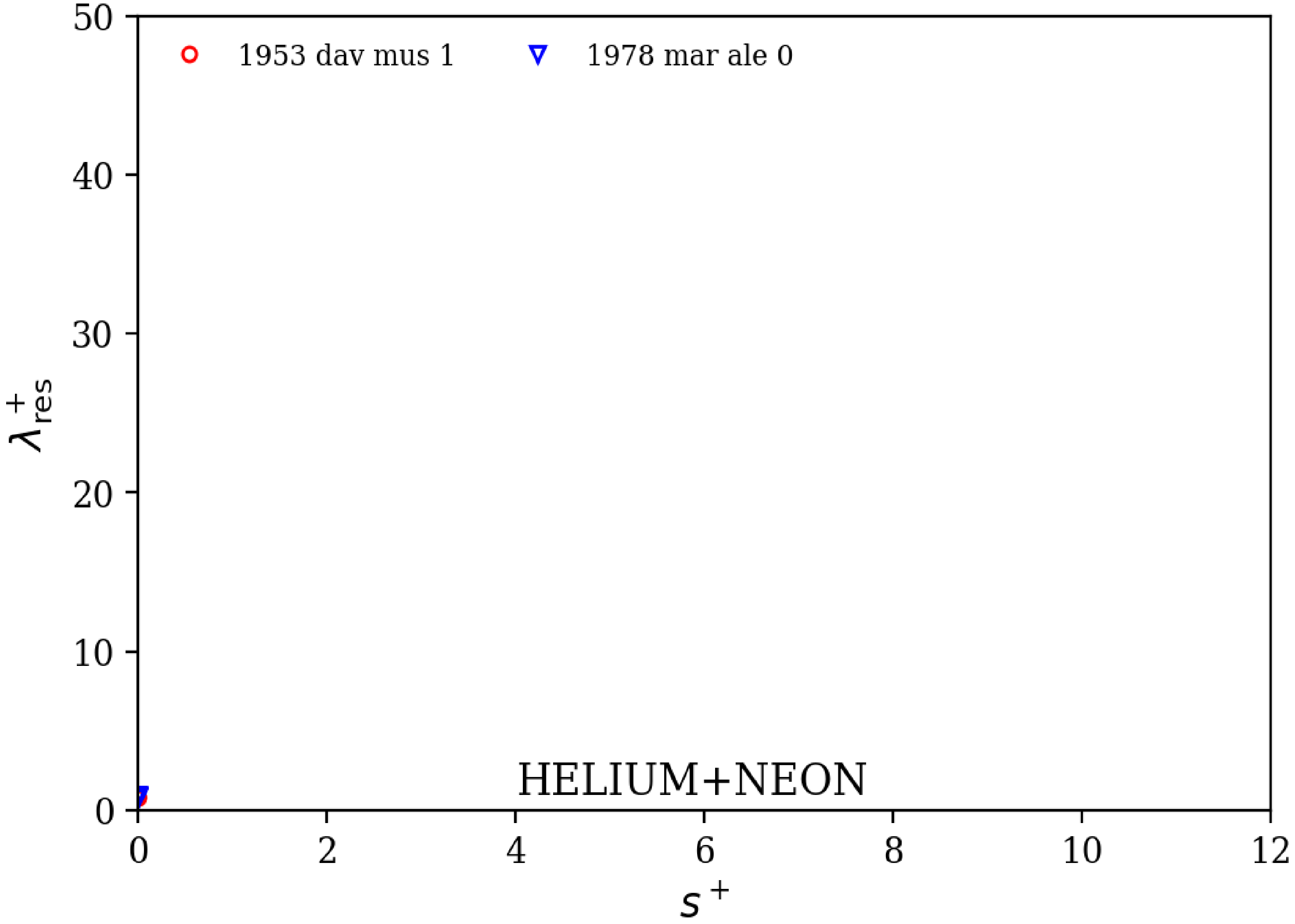

Figure DPR3. HELIUM+NEON

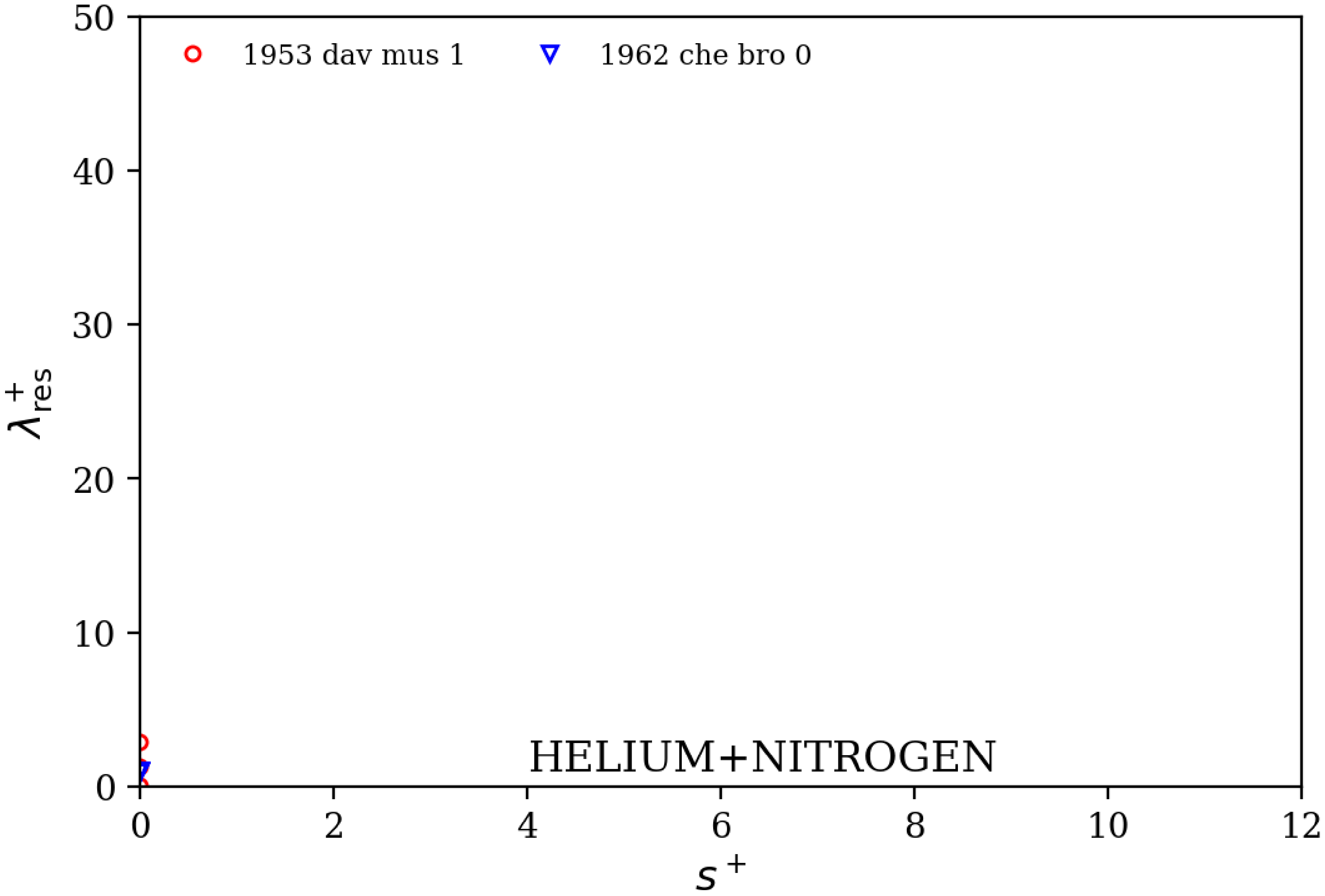

Figure DPR3. HELIUM+NITROGEN

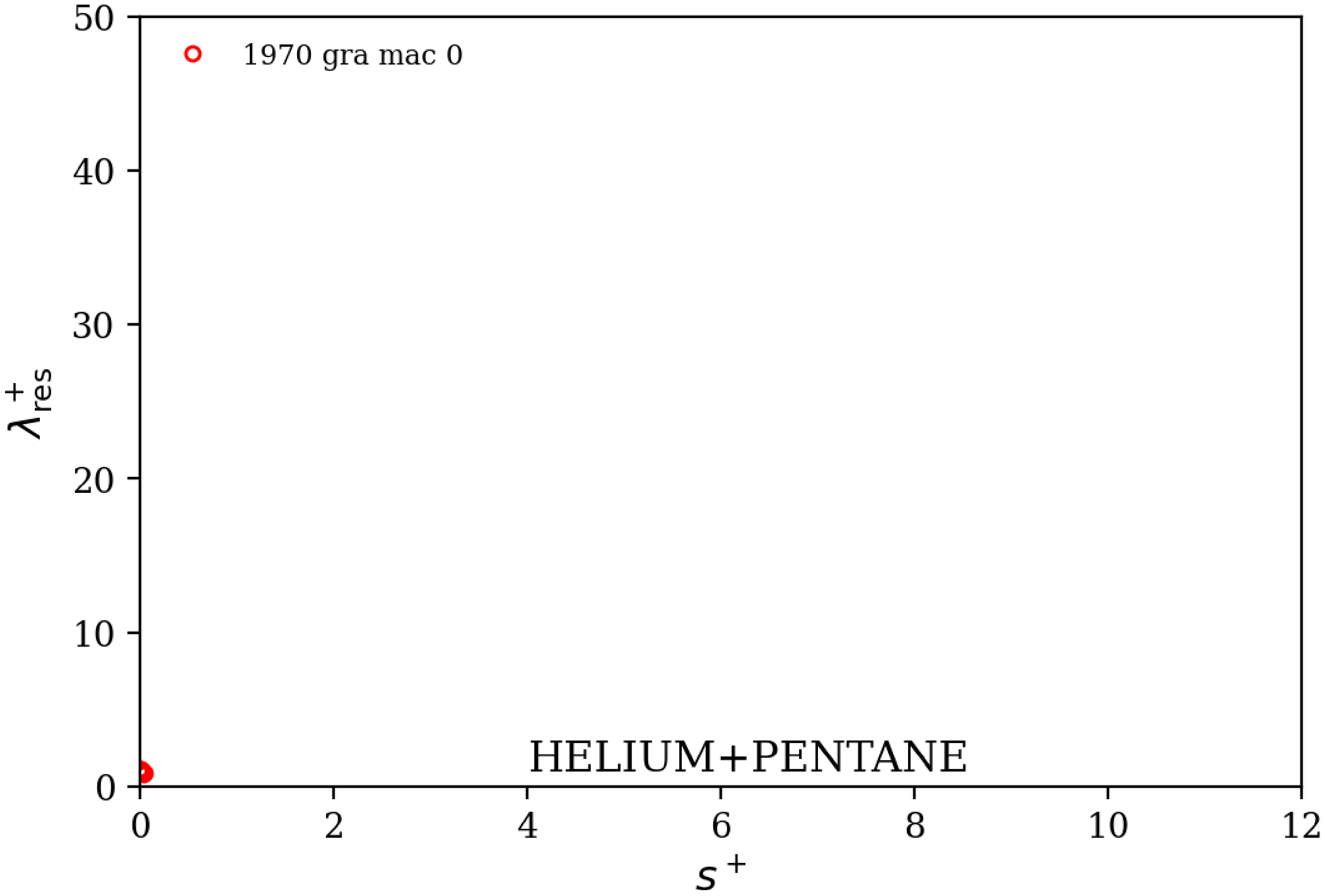

Figure DPR3. HELIUM+PENTANE

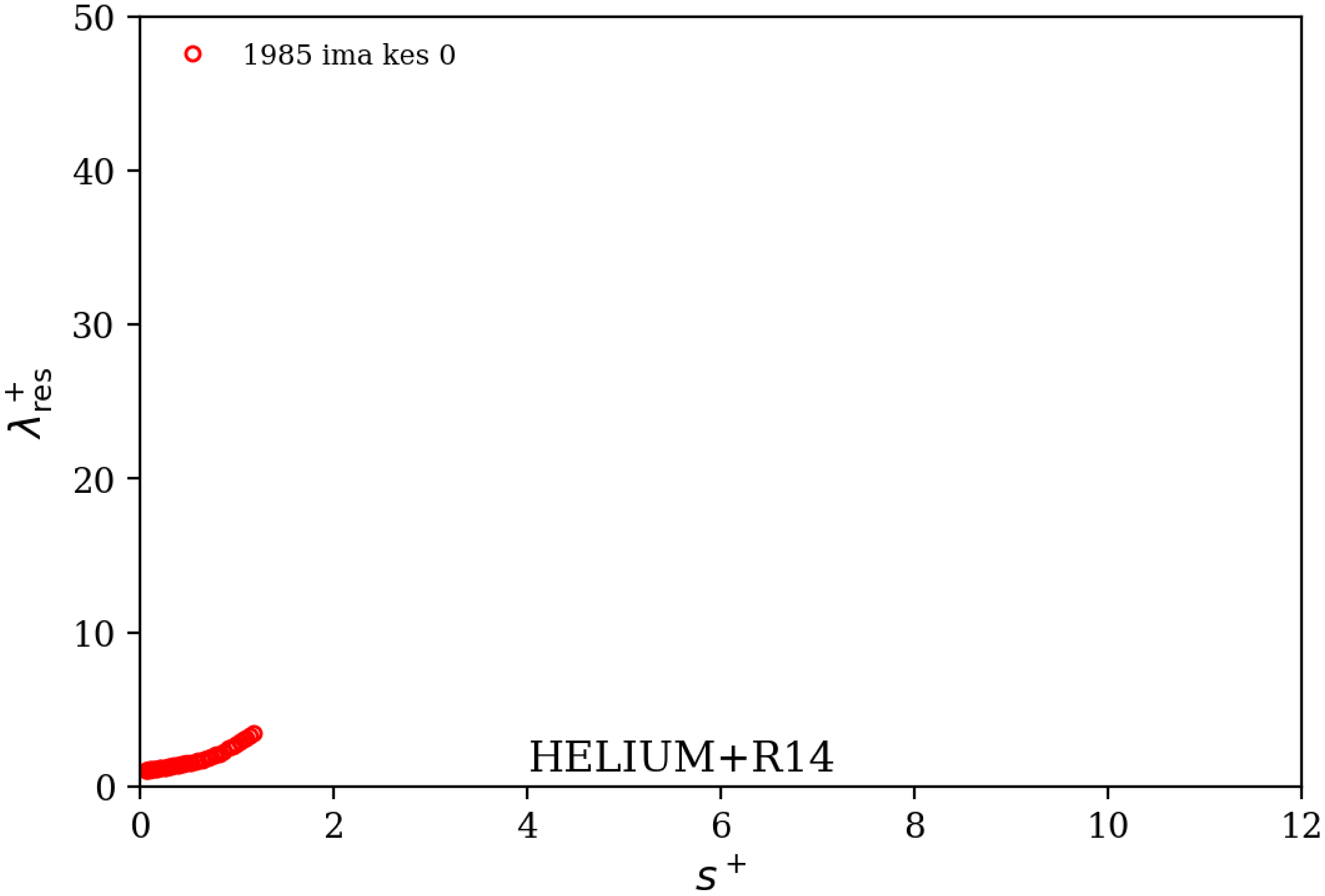

Figure DPR3. HELIUM+R14

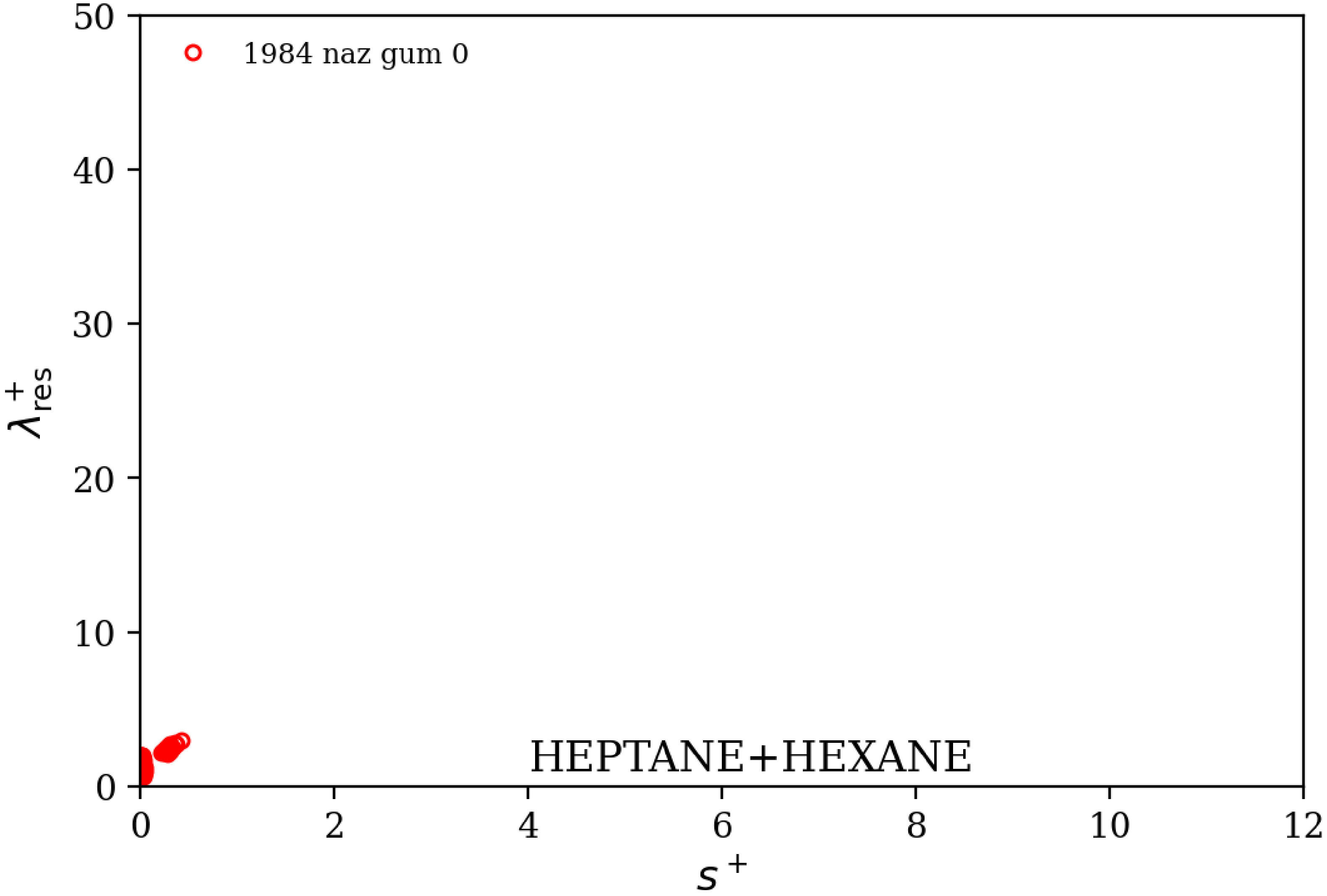

Figure DPR3. HEPTANE+HEXANE

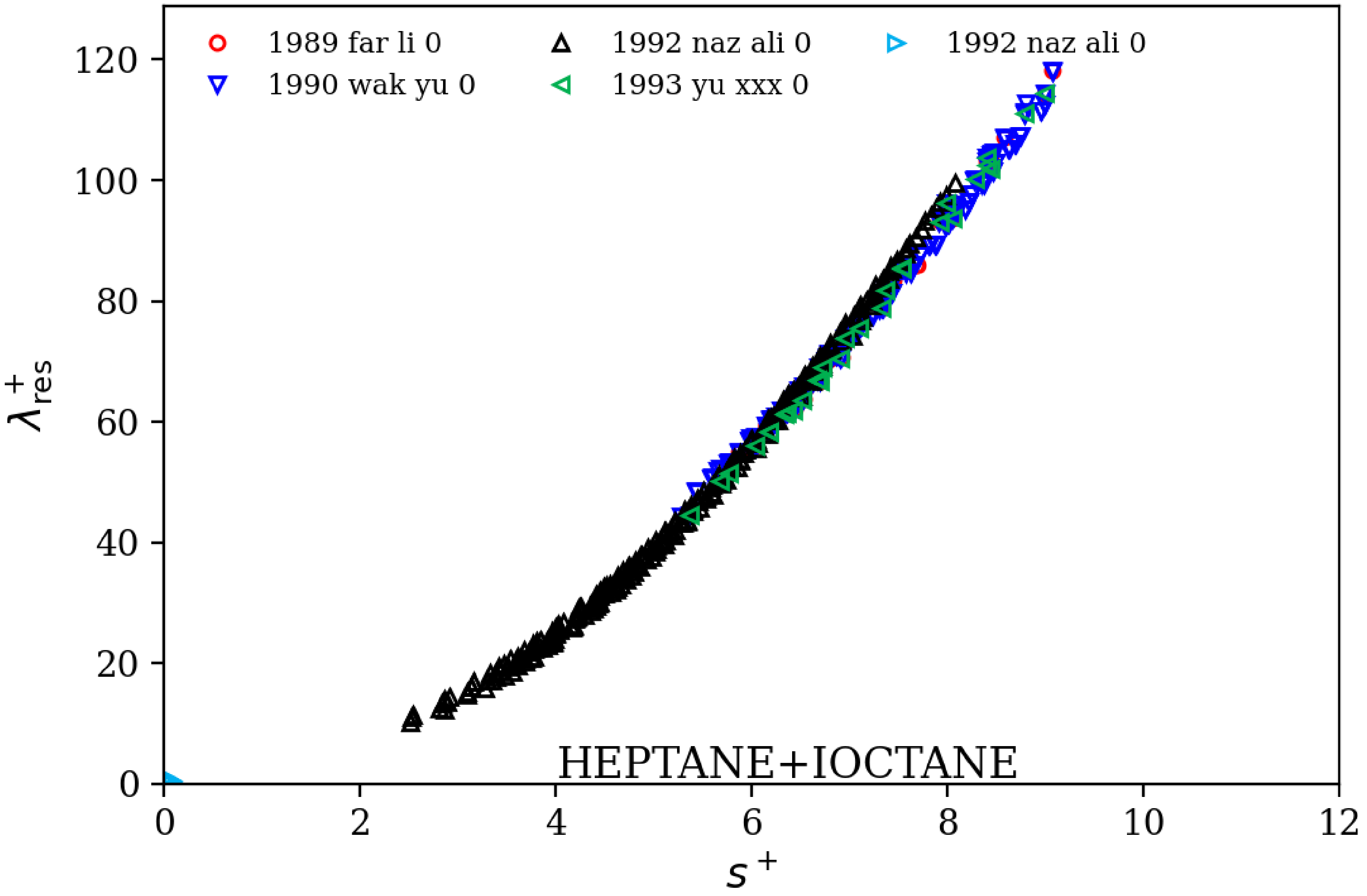

Figure DPR3. HEPTANE+IOCTANE

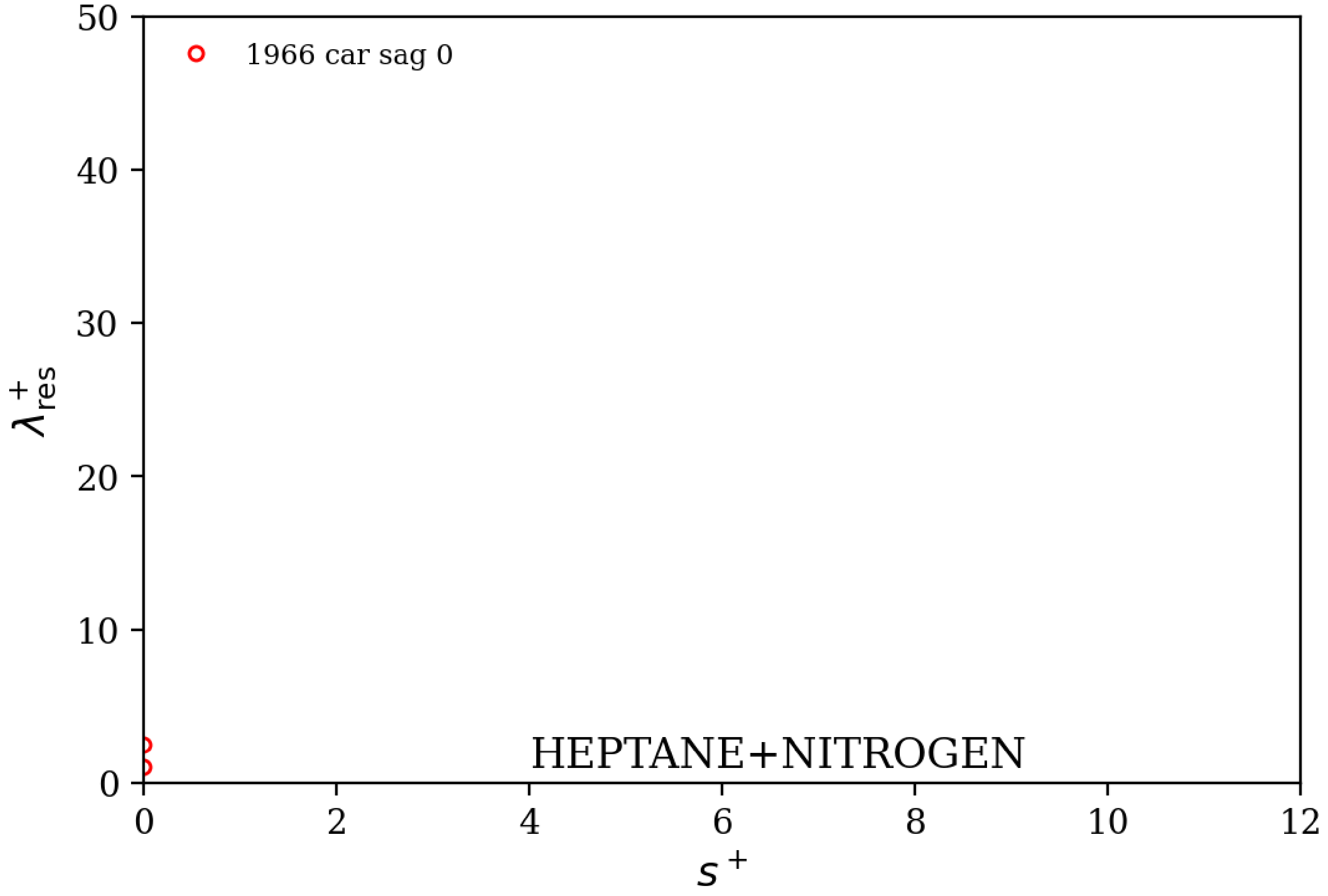

Figure DPR3. HEPTANE+NITROGEN

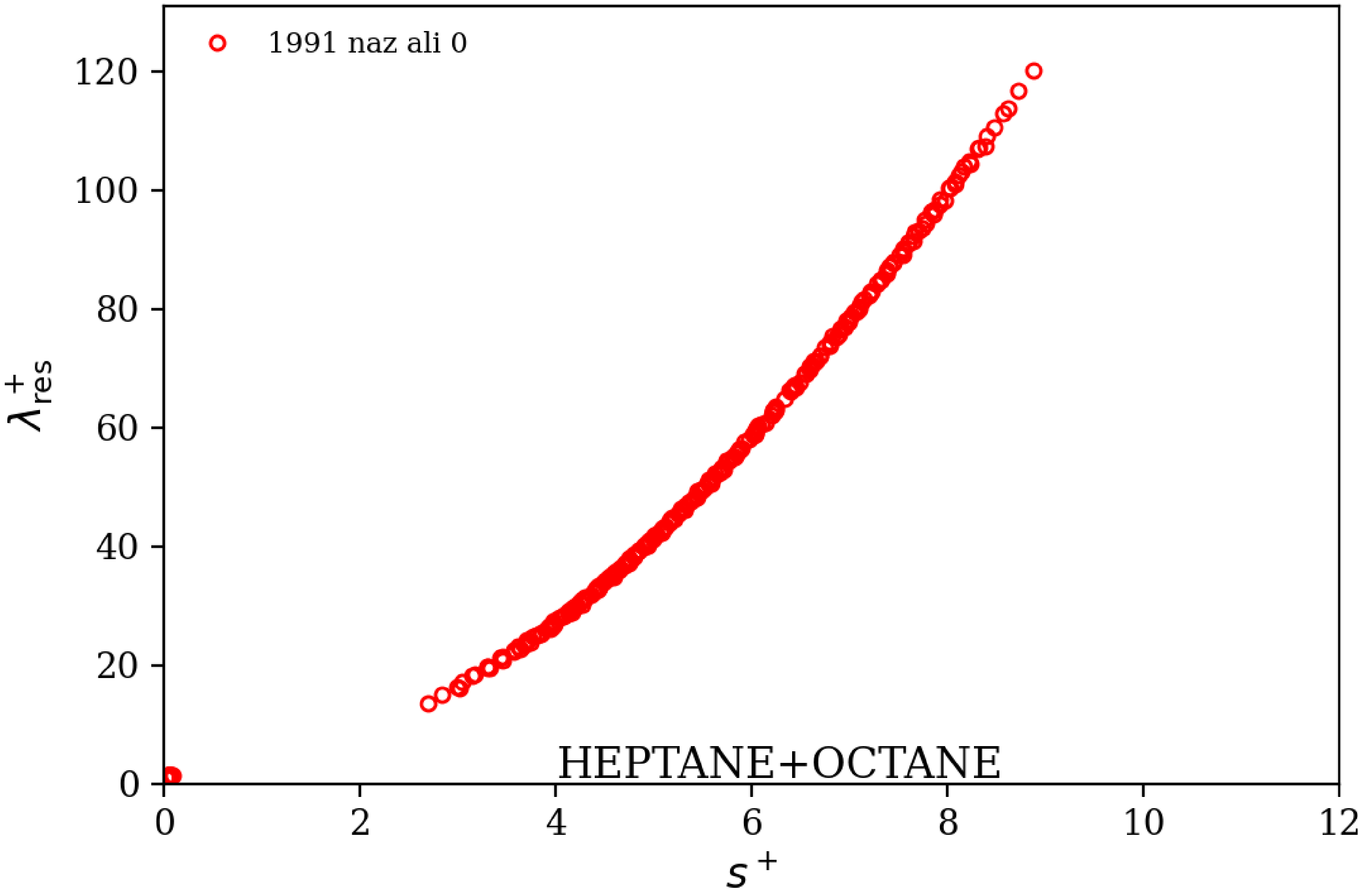

Figure DPR3. HEPTANE+OCTANE

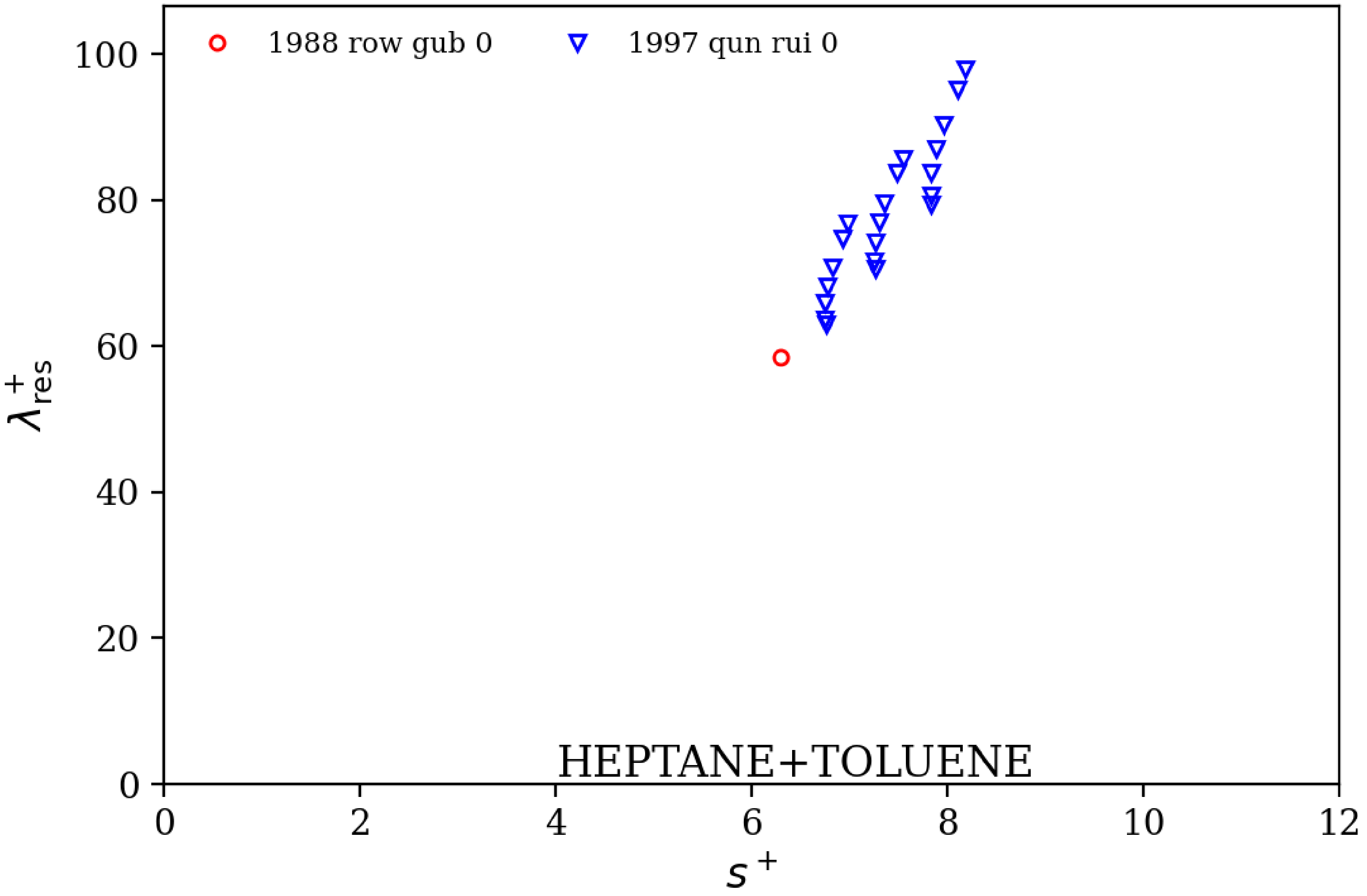

Figure DPR3. HEPTANE+TOLUENE

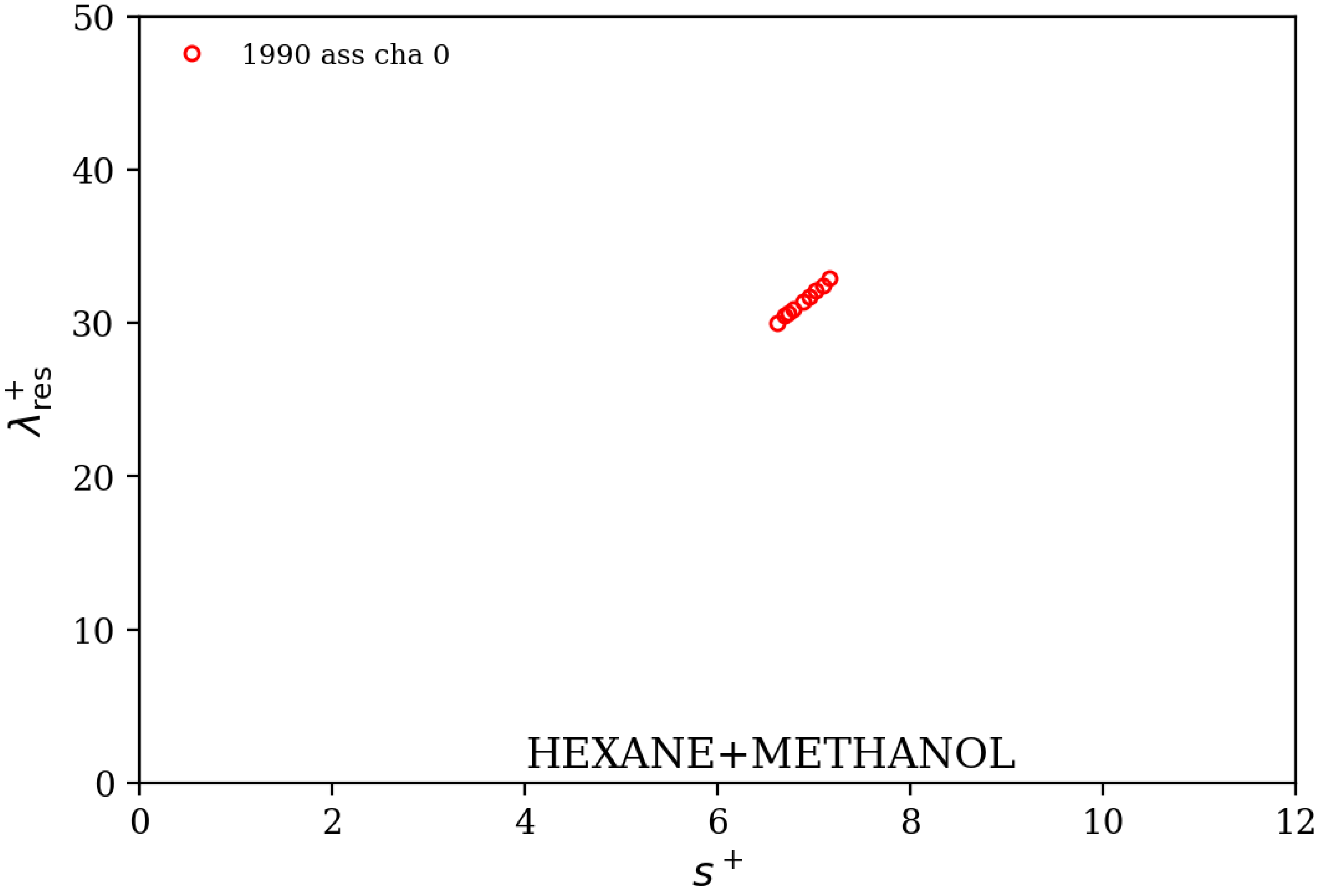

Figure DPR3. HEXANE+METHANOL

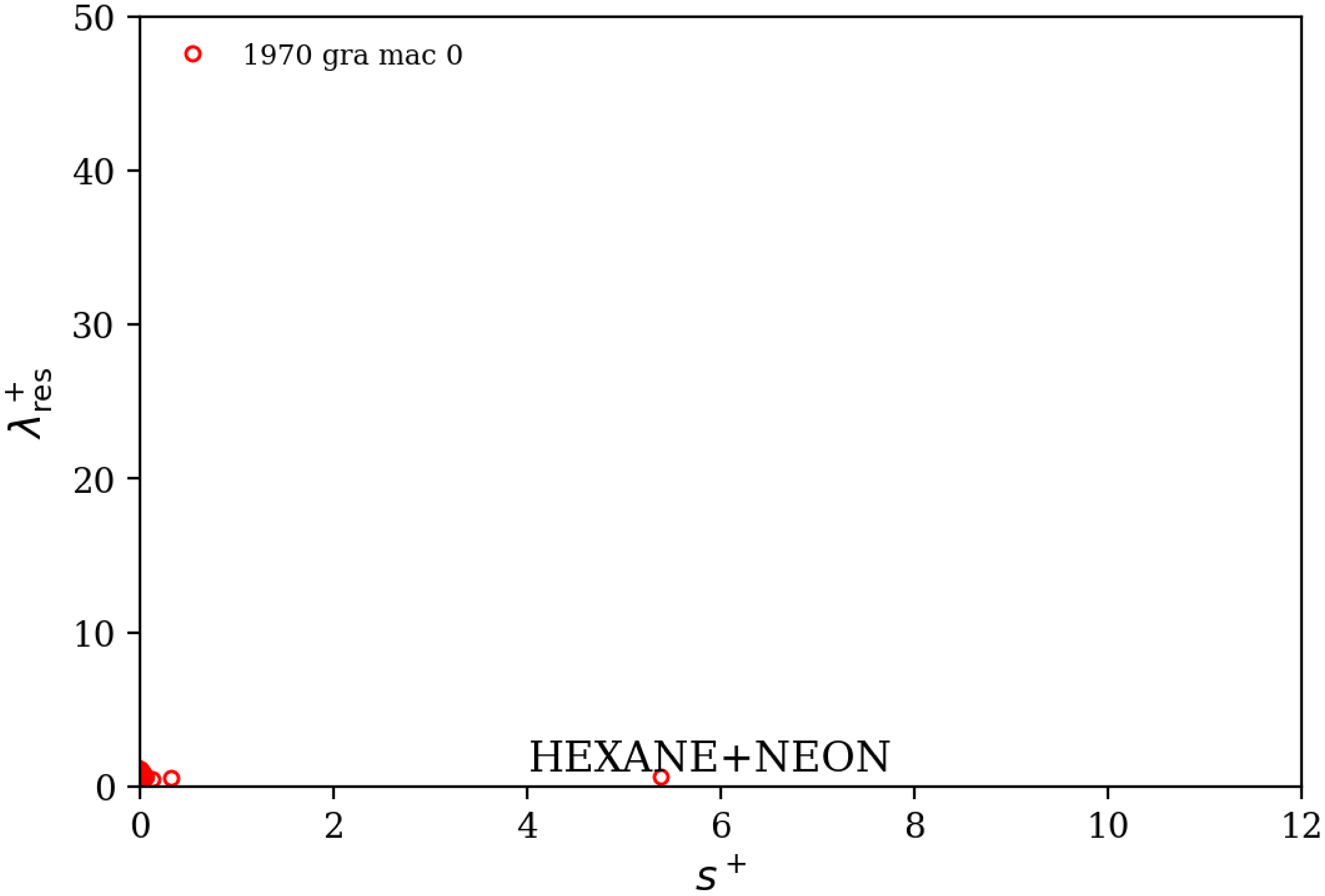

Figure DPR3. HEXANE+NEON

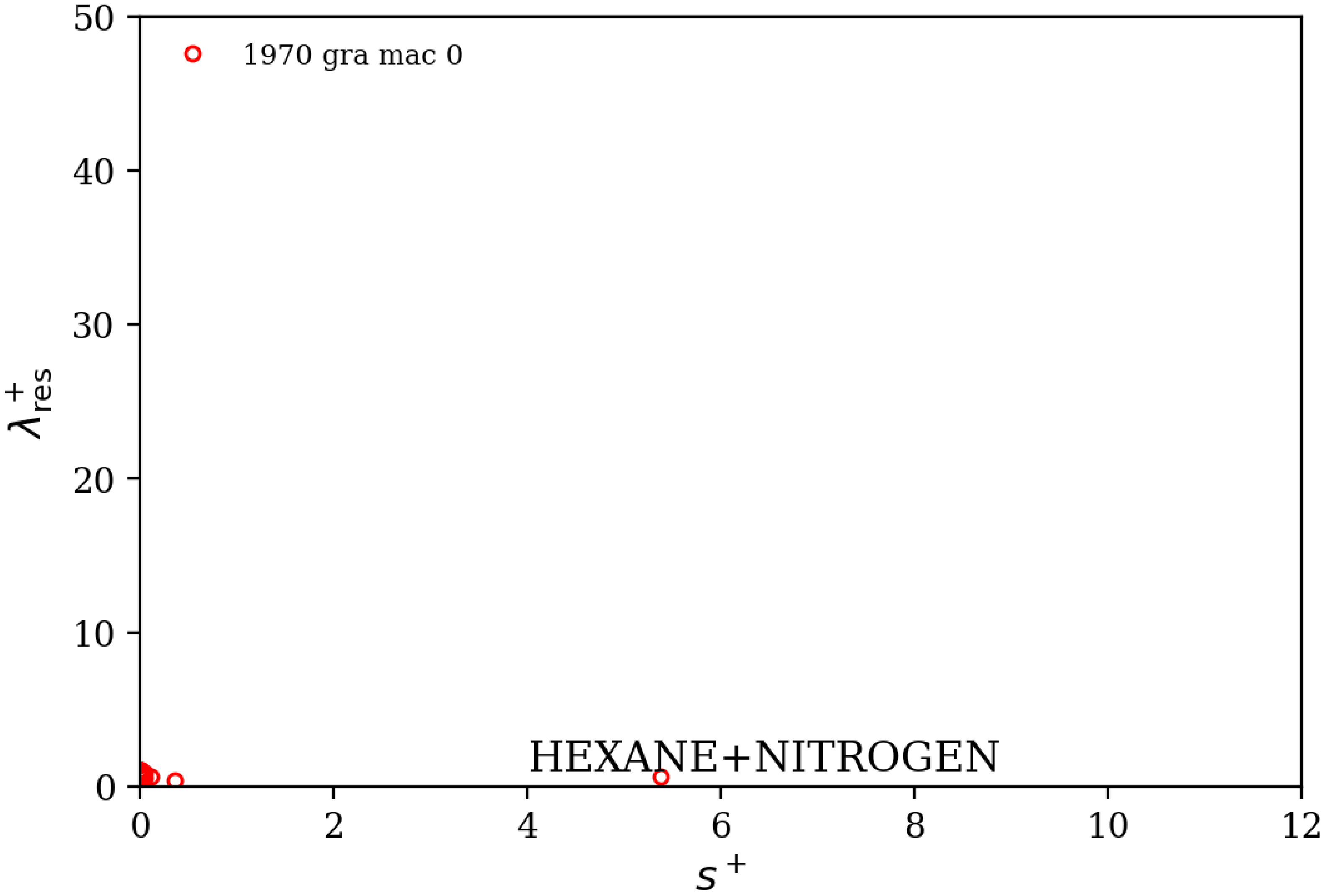

Figure DPR3. HEXANE+NITROGEN

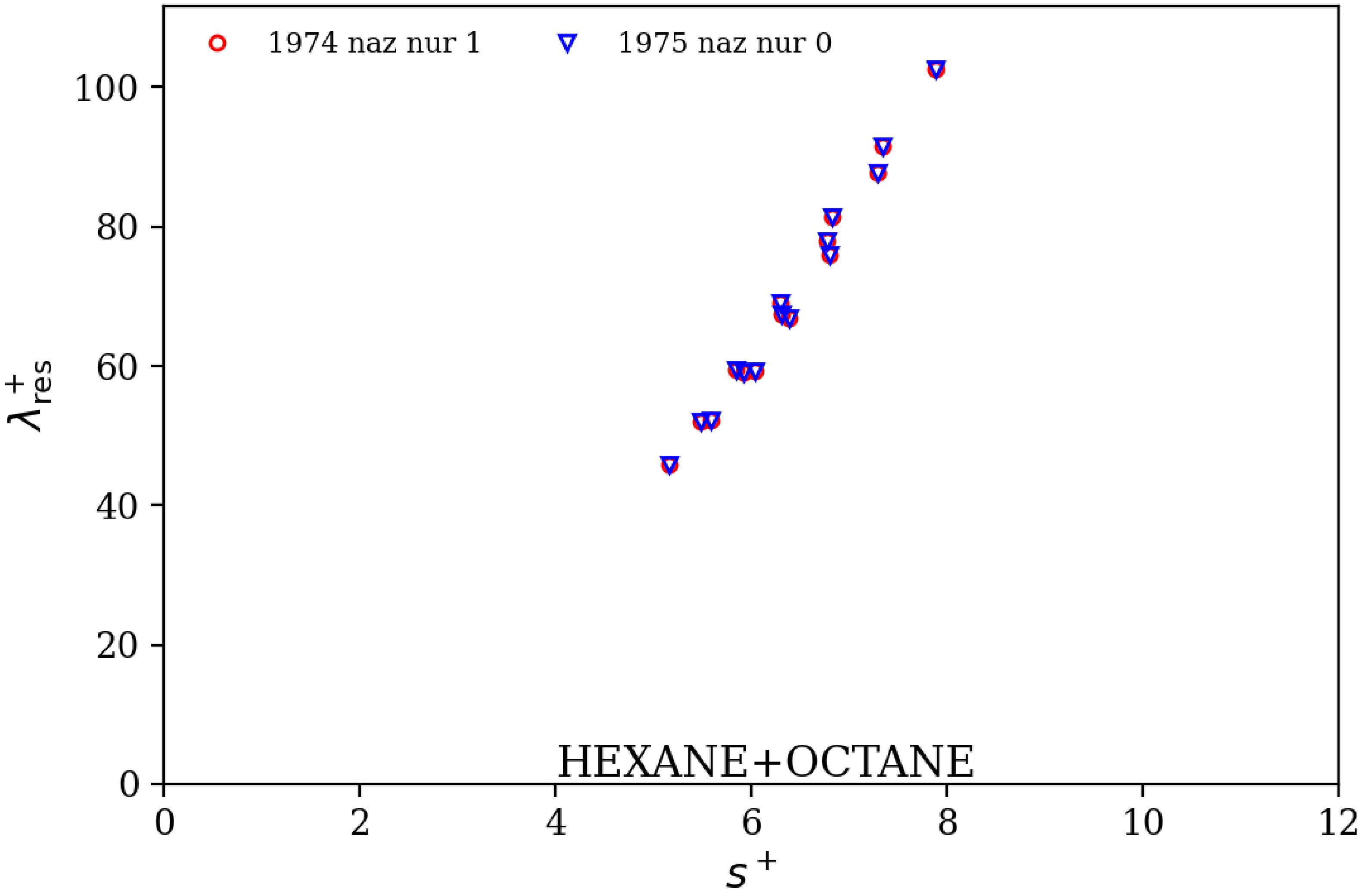

Figure DPR3. HEXANE+OCTANE

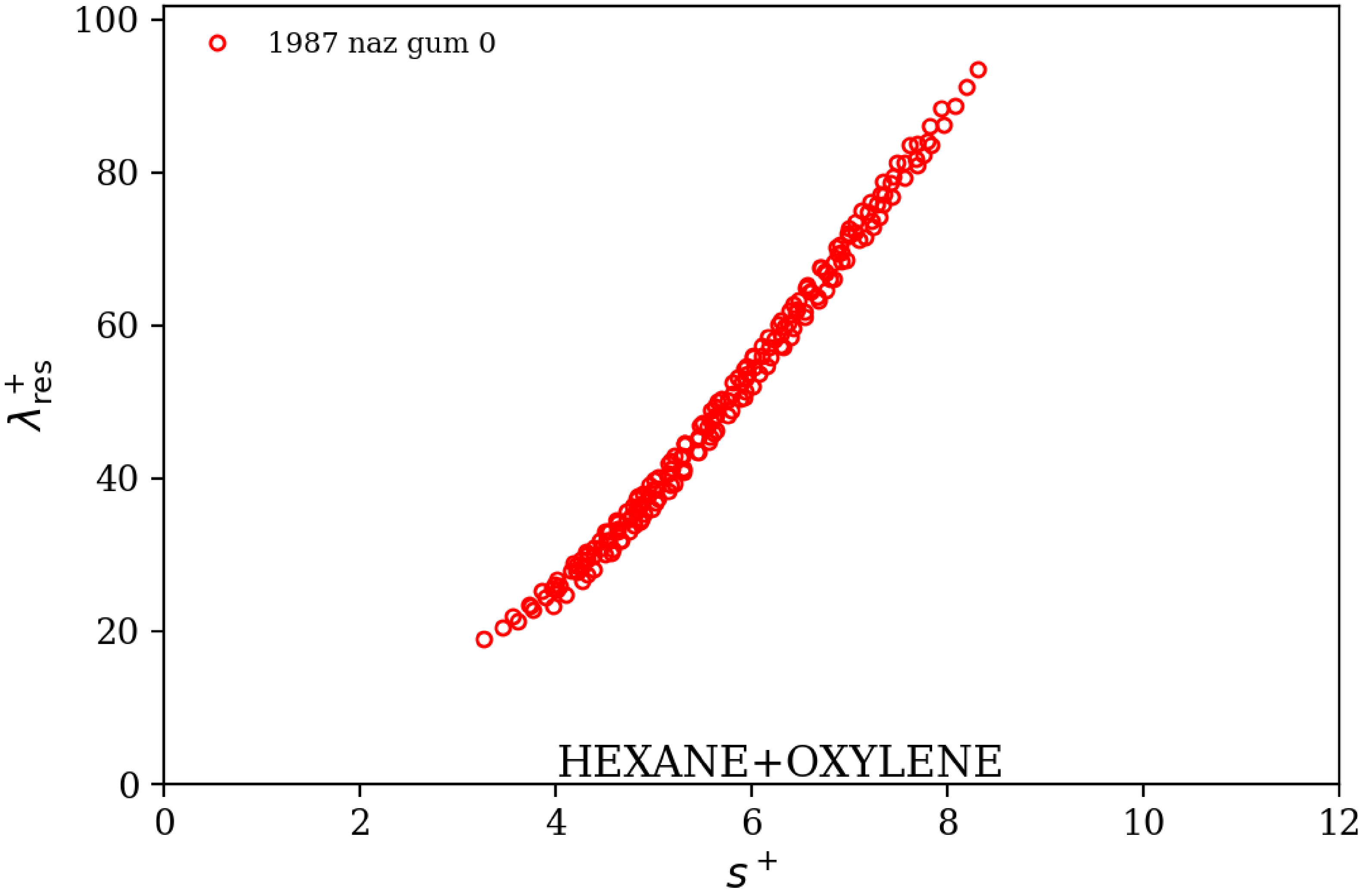

Figure DPR3. HEXANE+OXYLENE

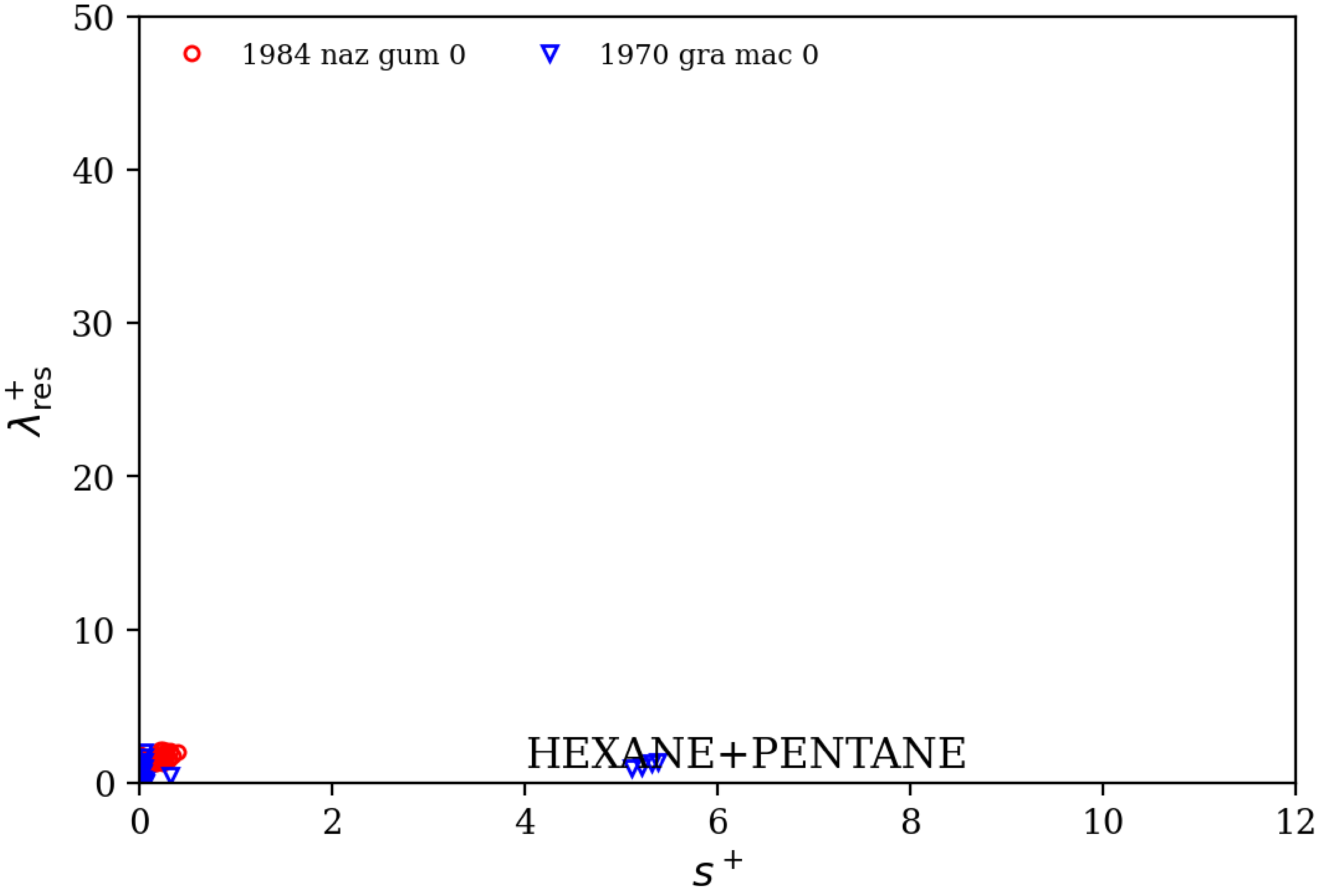

Figure DPR3. HEXANE+PENTANE

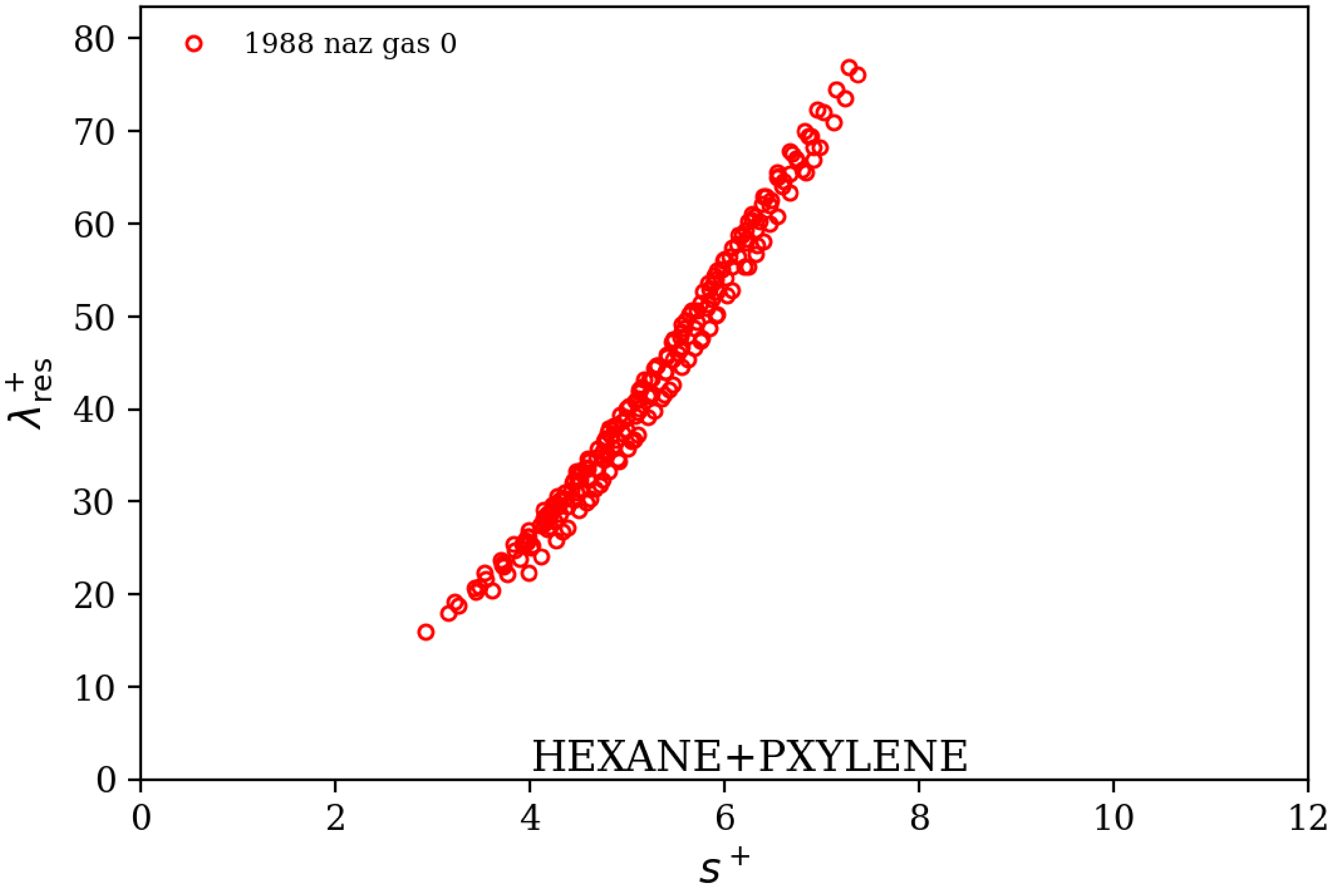

Figure DPR3. HEXANE+PXYLENE

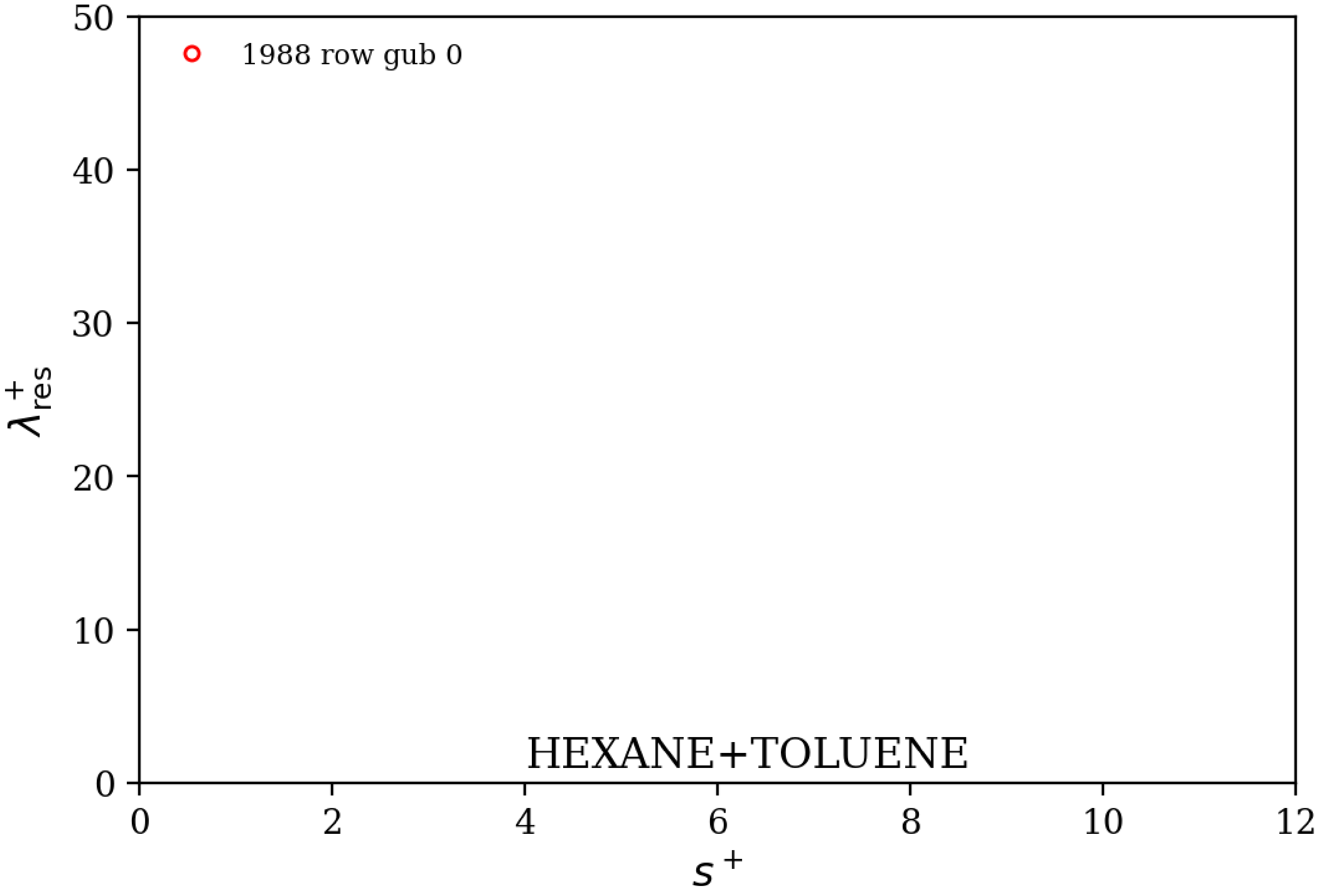

Figure DPR3. HEXANE+TOLUENE

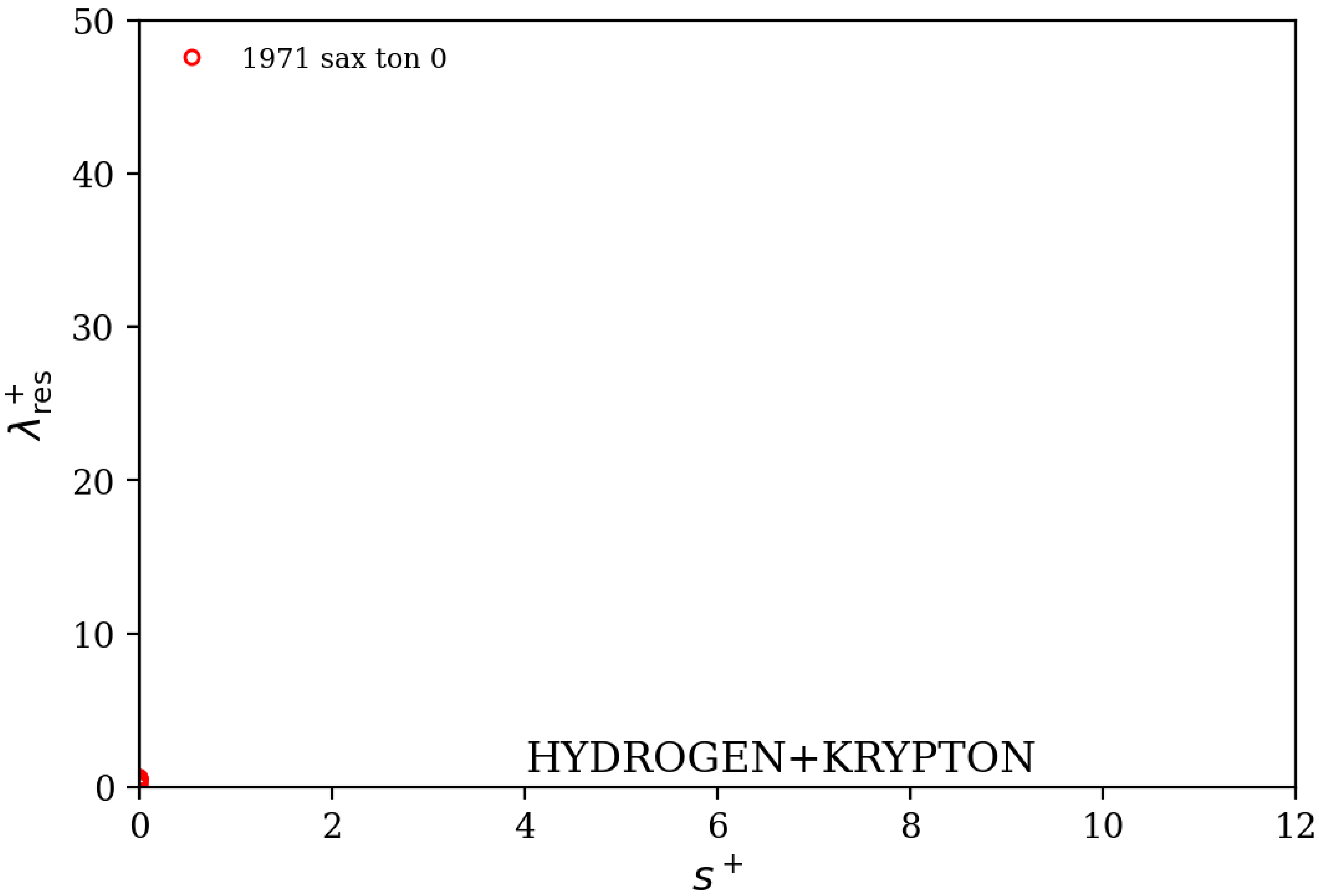

Figure DPR3. HYDROGEN+KRYPTON

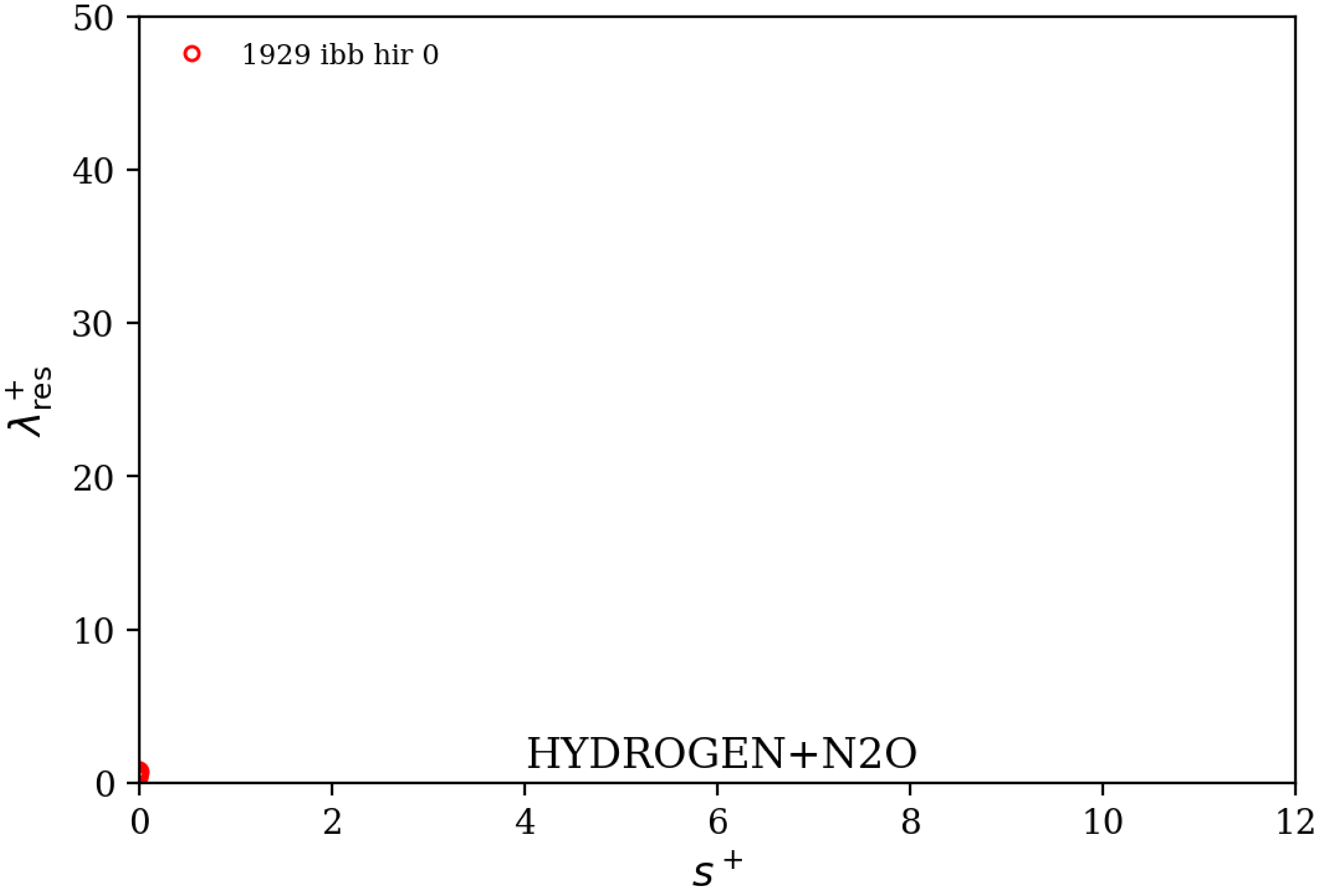

Figure DPR3. HYDROGEN+N2O

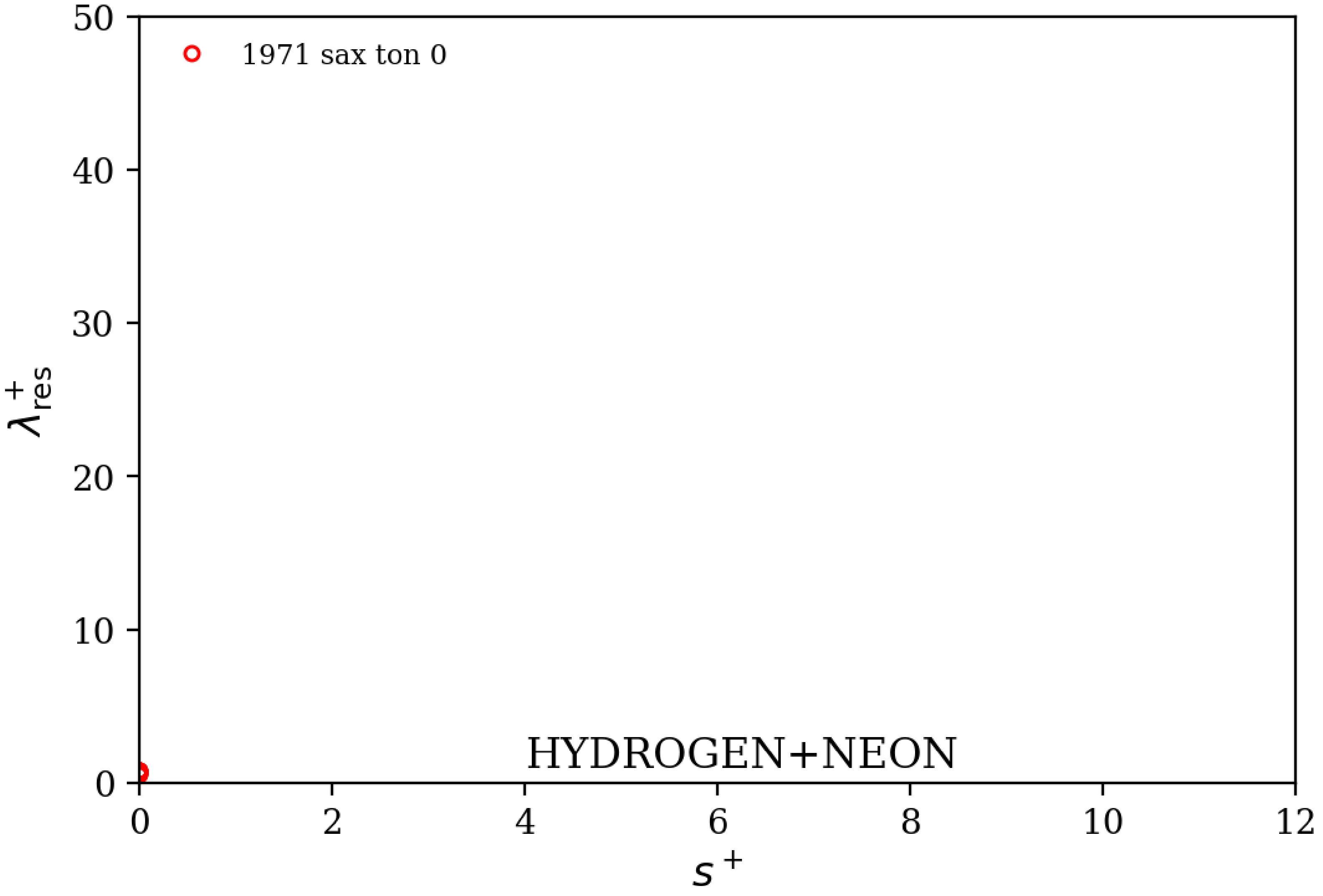

Figure DPR3. HYDROGEN+NEON

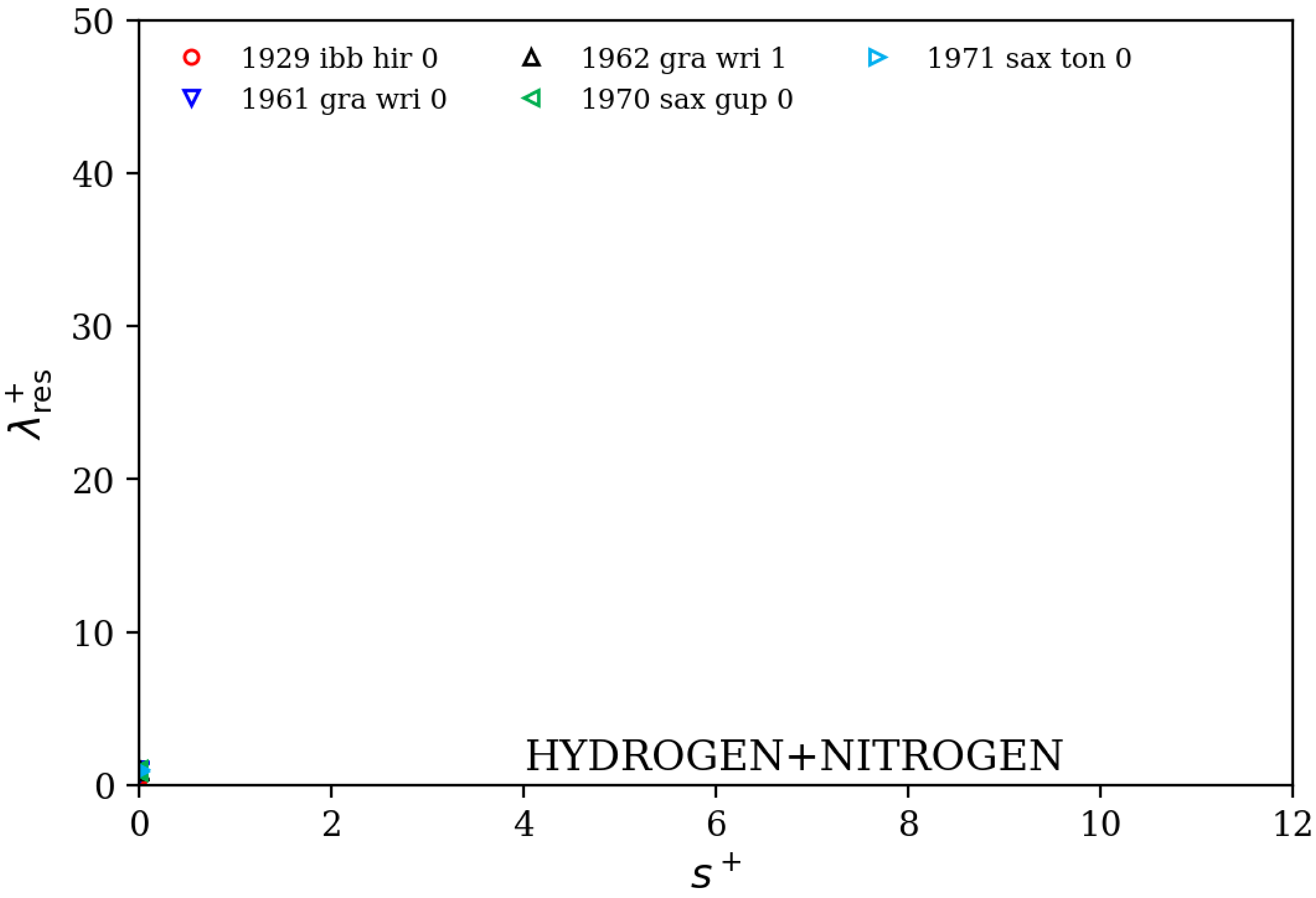

Figure DPR3. HYDROGEN+NITROGEN

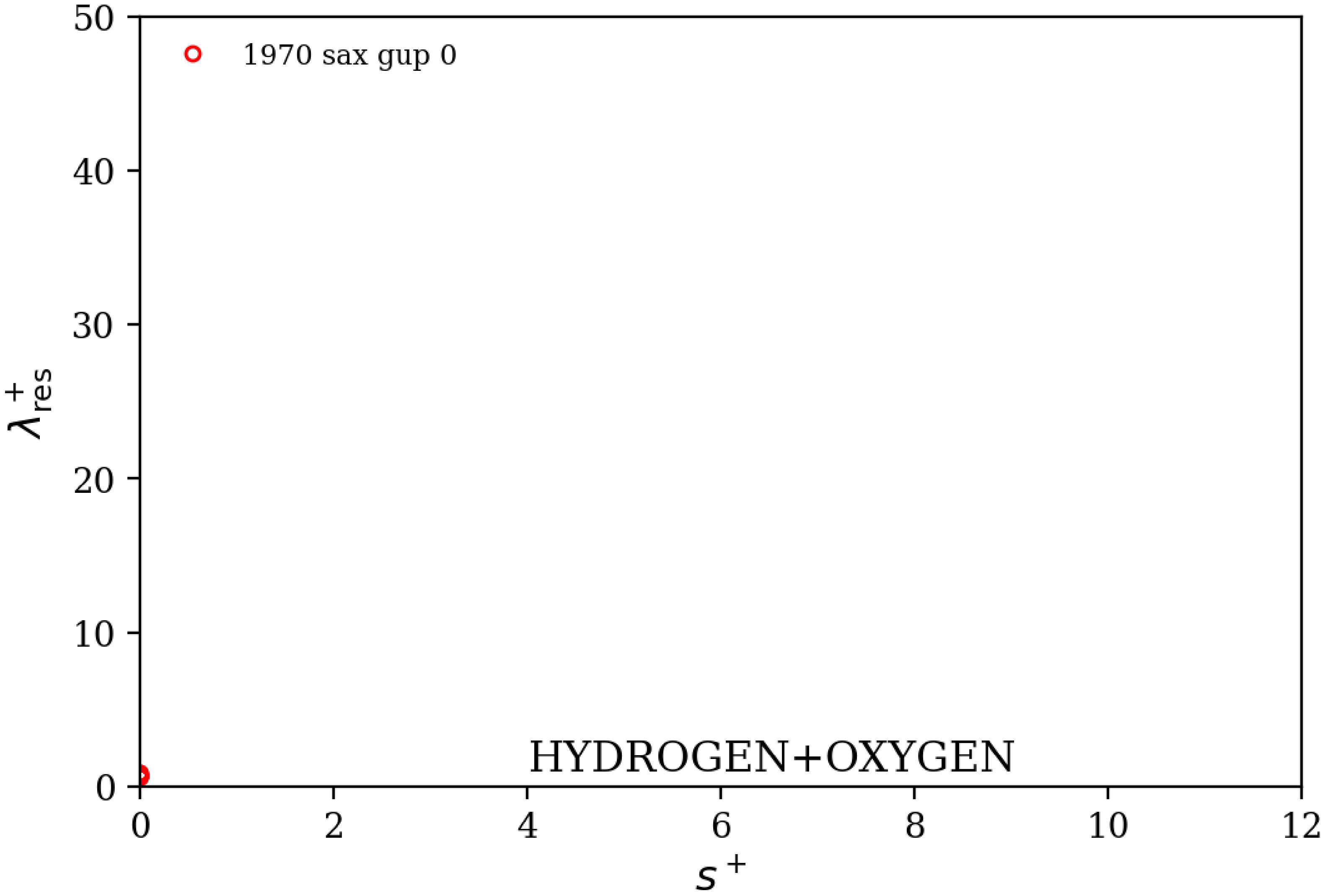

Figure DPR3. HYDROGEN+OXYGEN

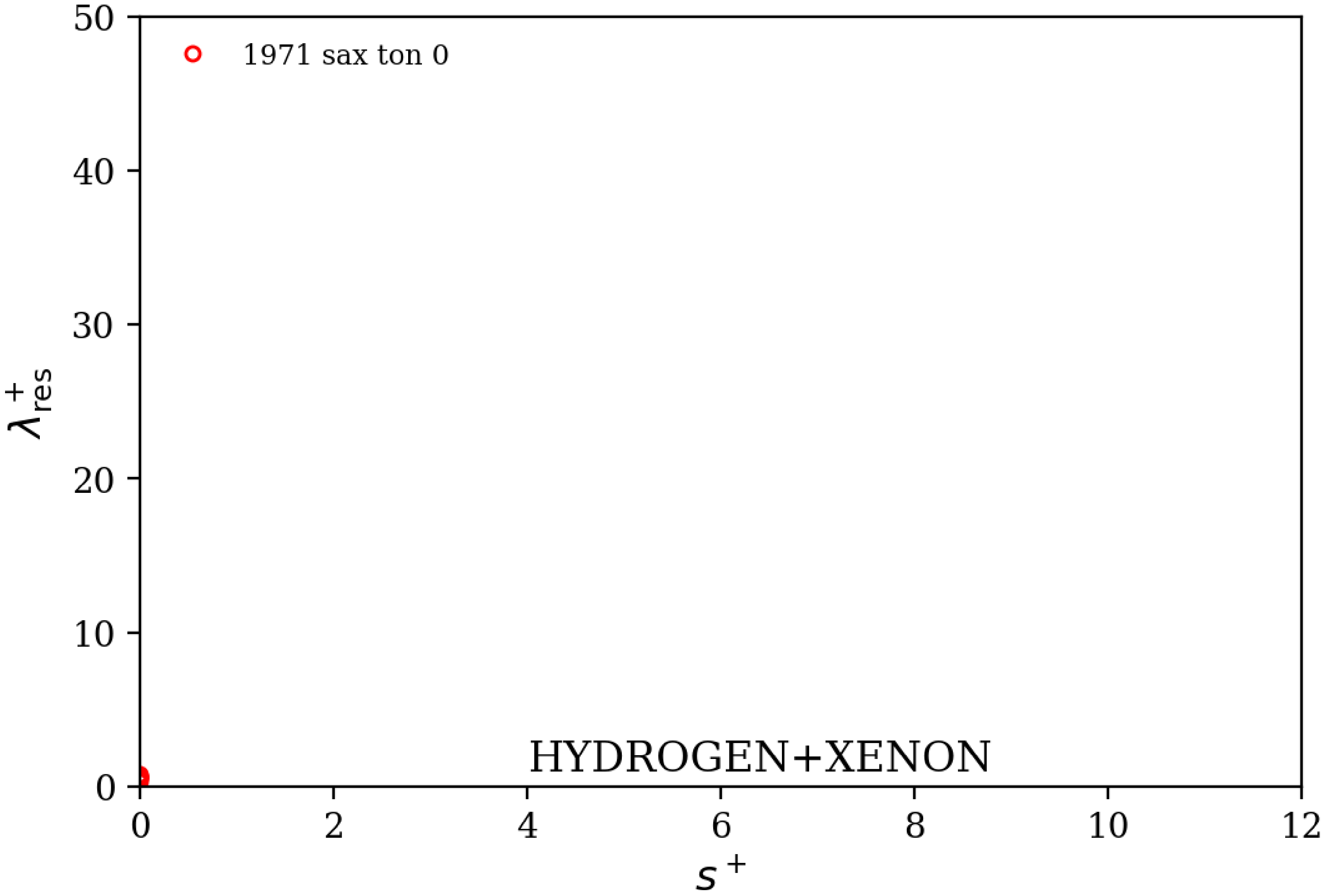

Figure DPR3. HYDROGEN+XENON

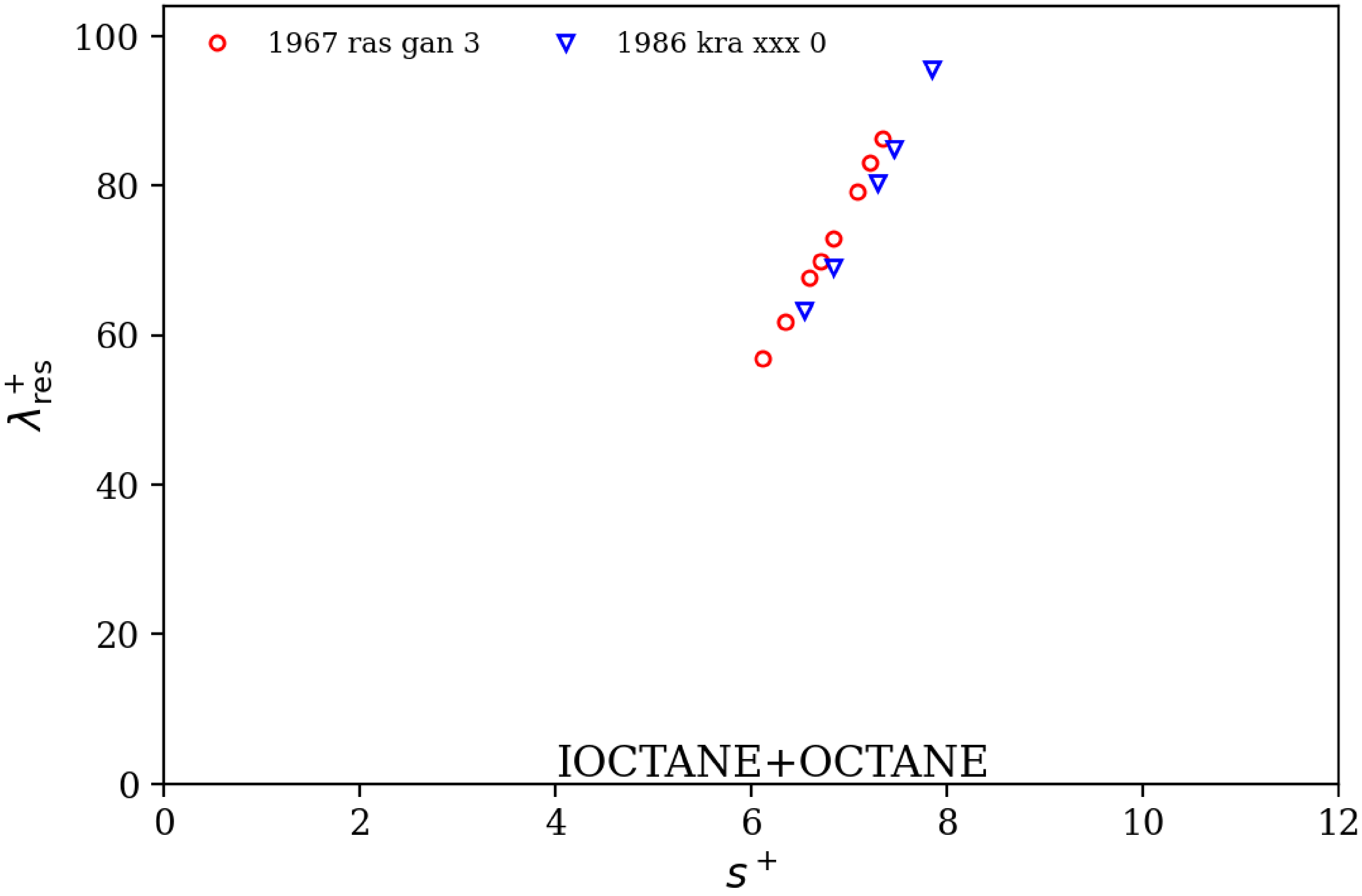

Figure DPR3. IOCTANE+OCTANE

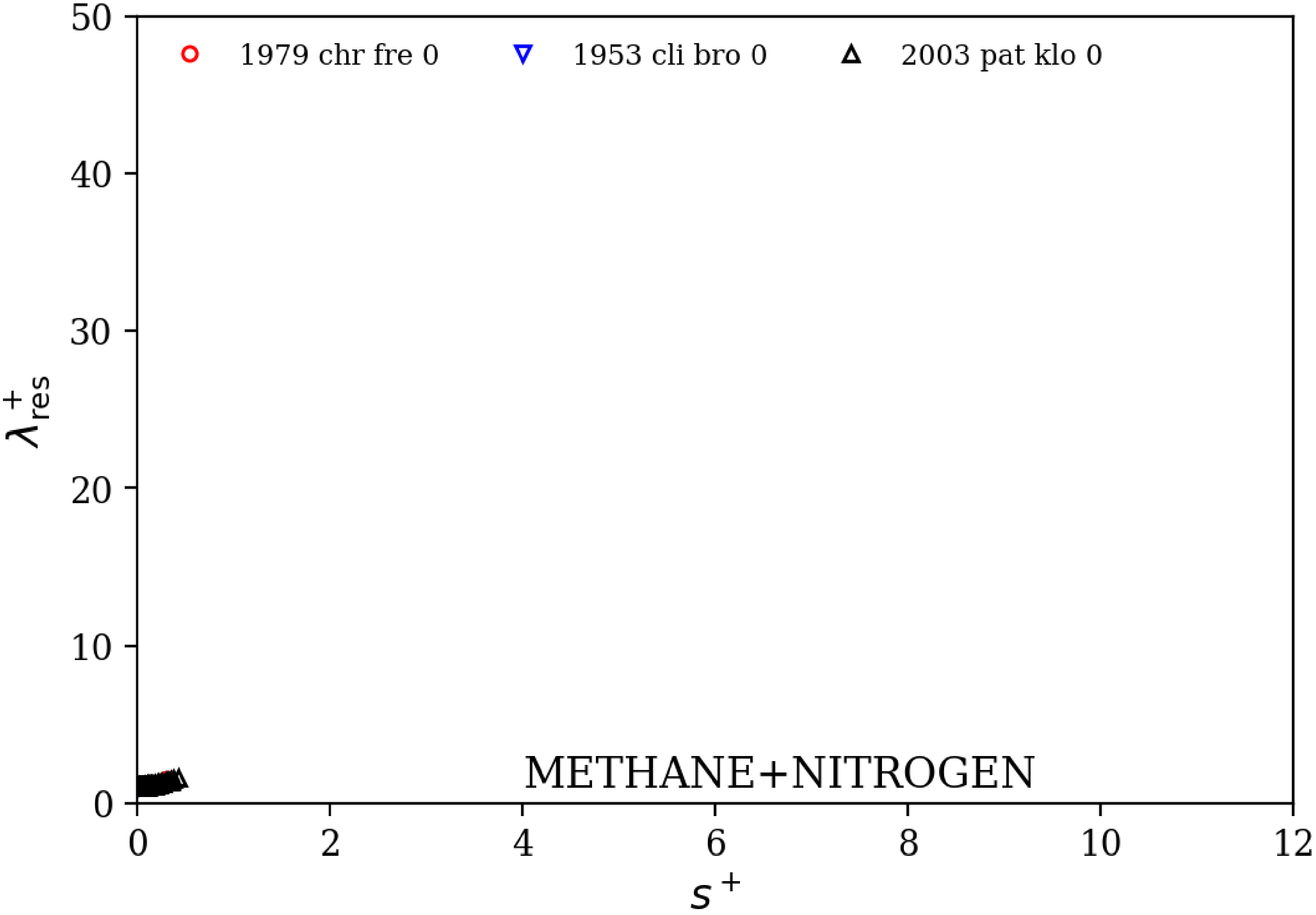

Figure DPR3. METHANE+NITROGEN

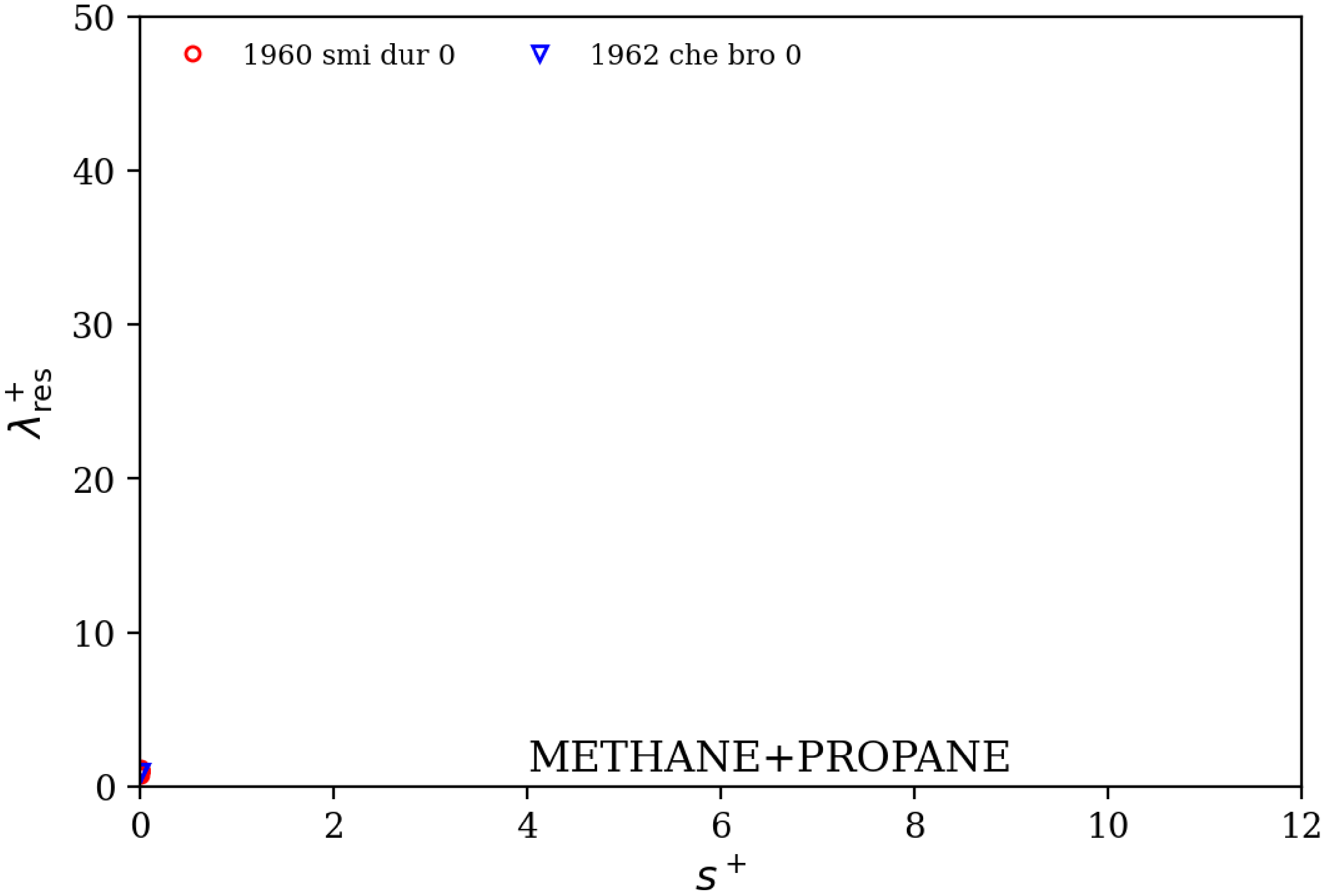

Figure DPR3. METHANE+PROPANE

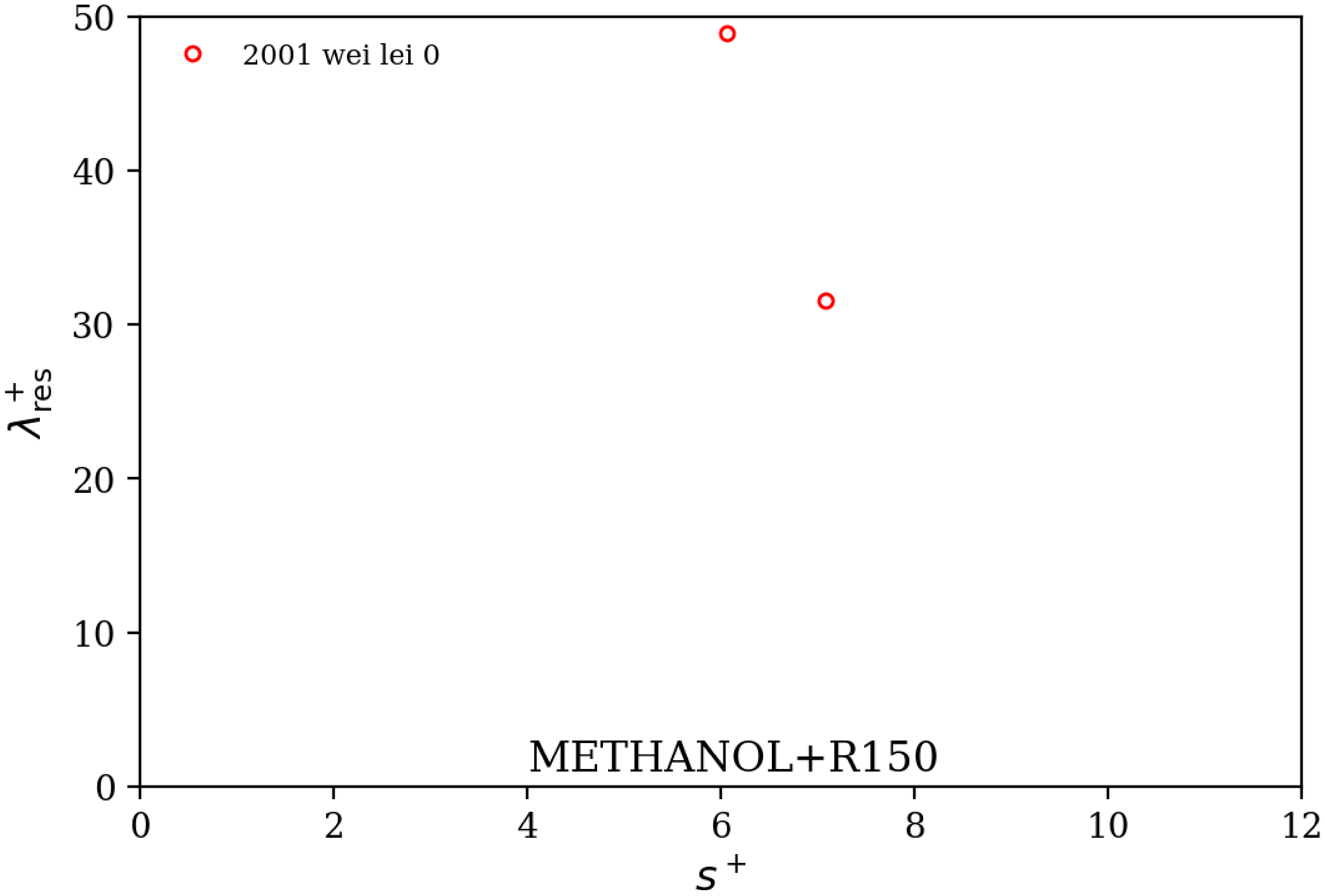

Figure DPR3. METHANOL+R150

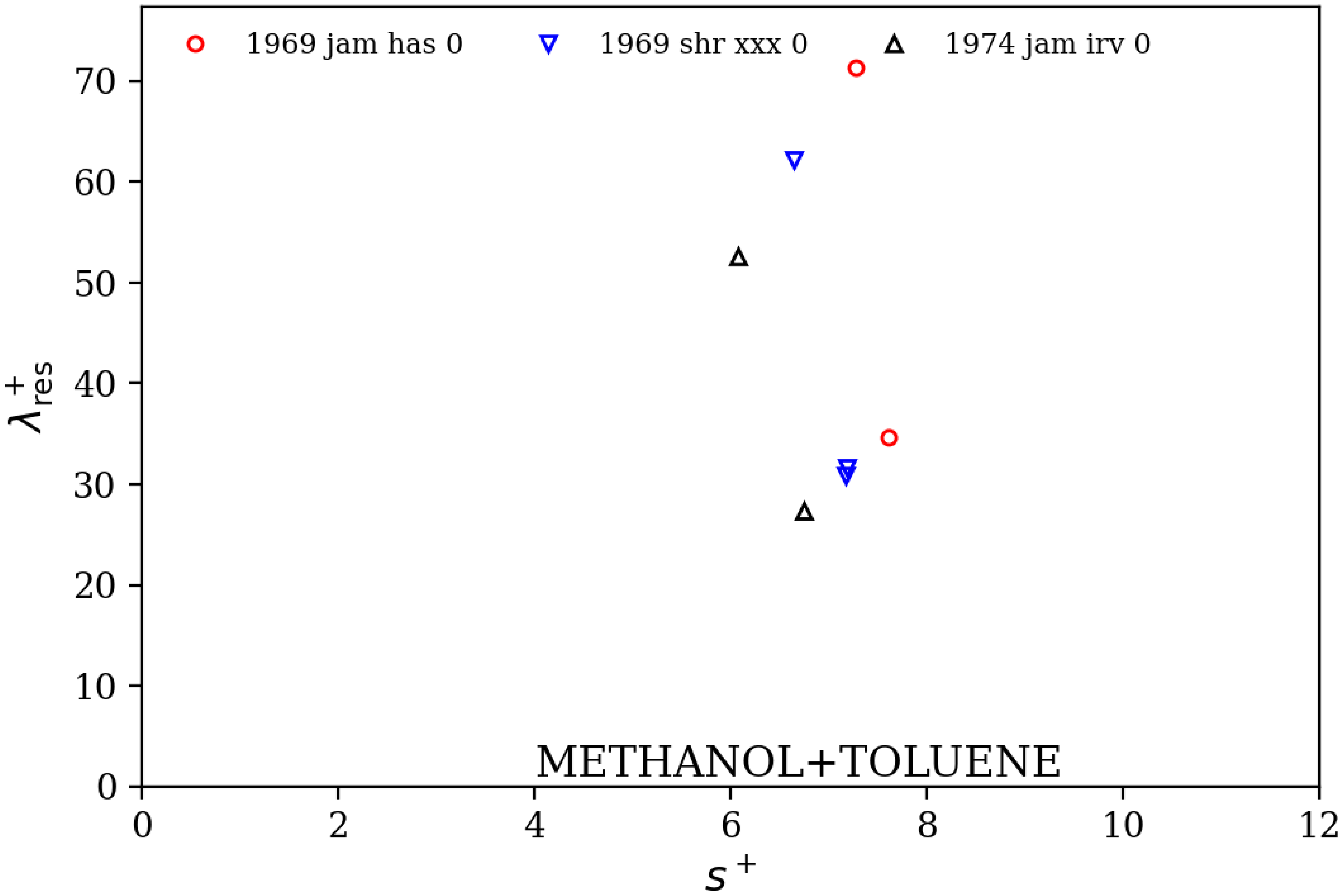

Figure DPR3. METHANOL+TOLUENE

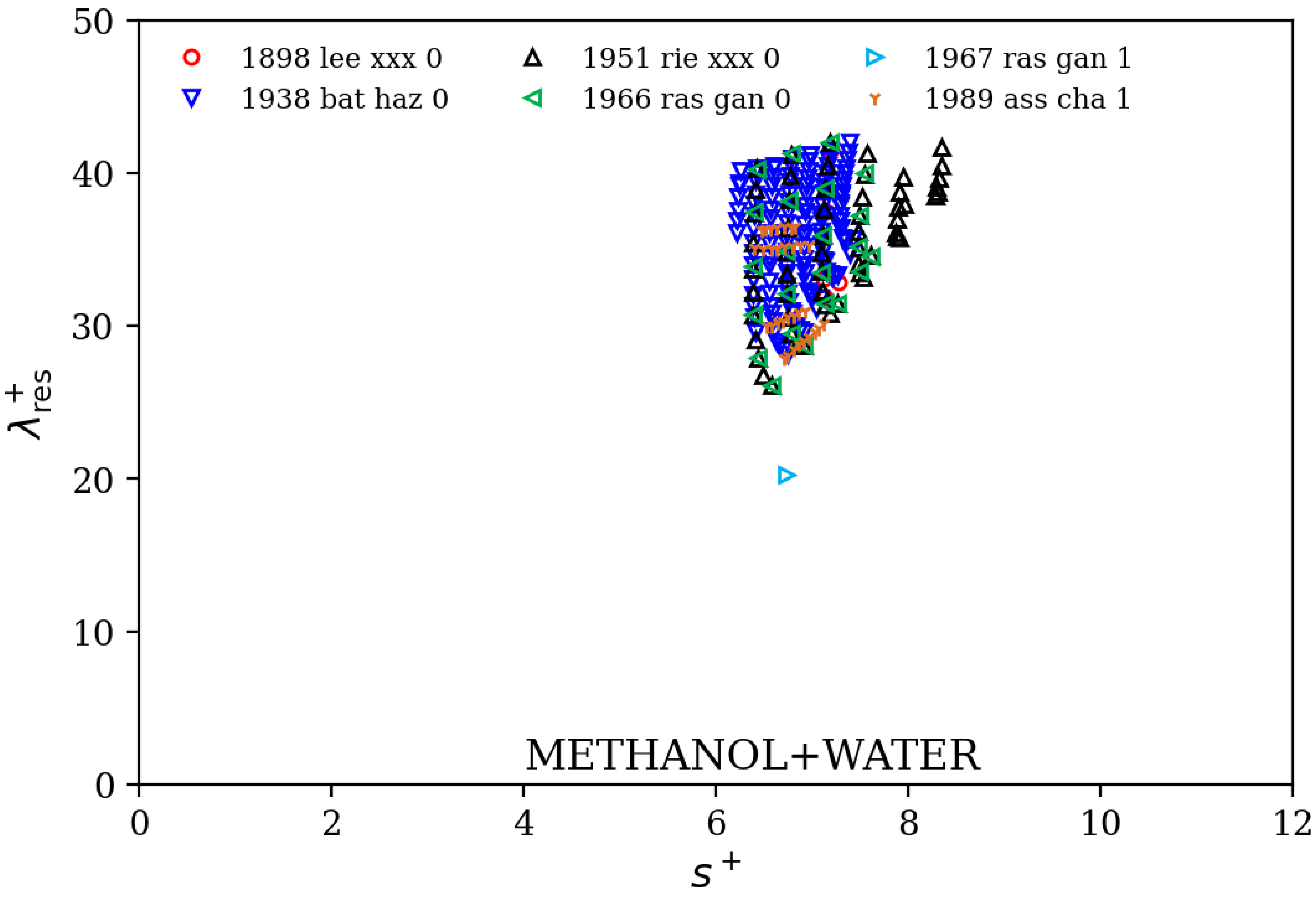

Figure DPR3. METHANOL+WATER

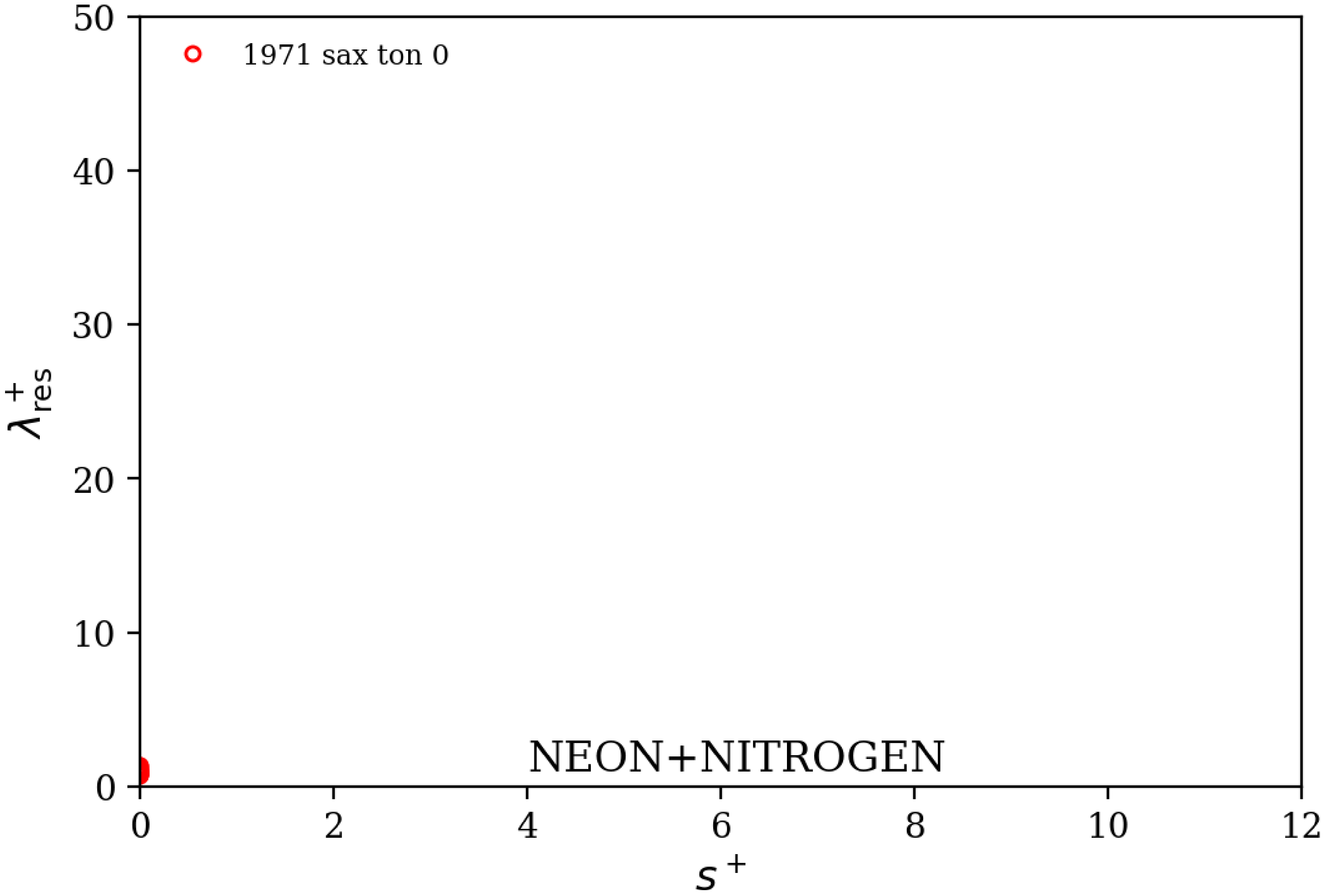

Figure DPR3. NEON+NITROGEN

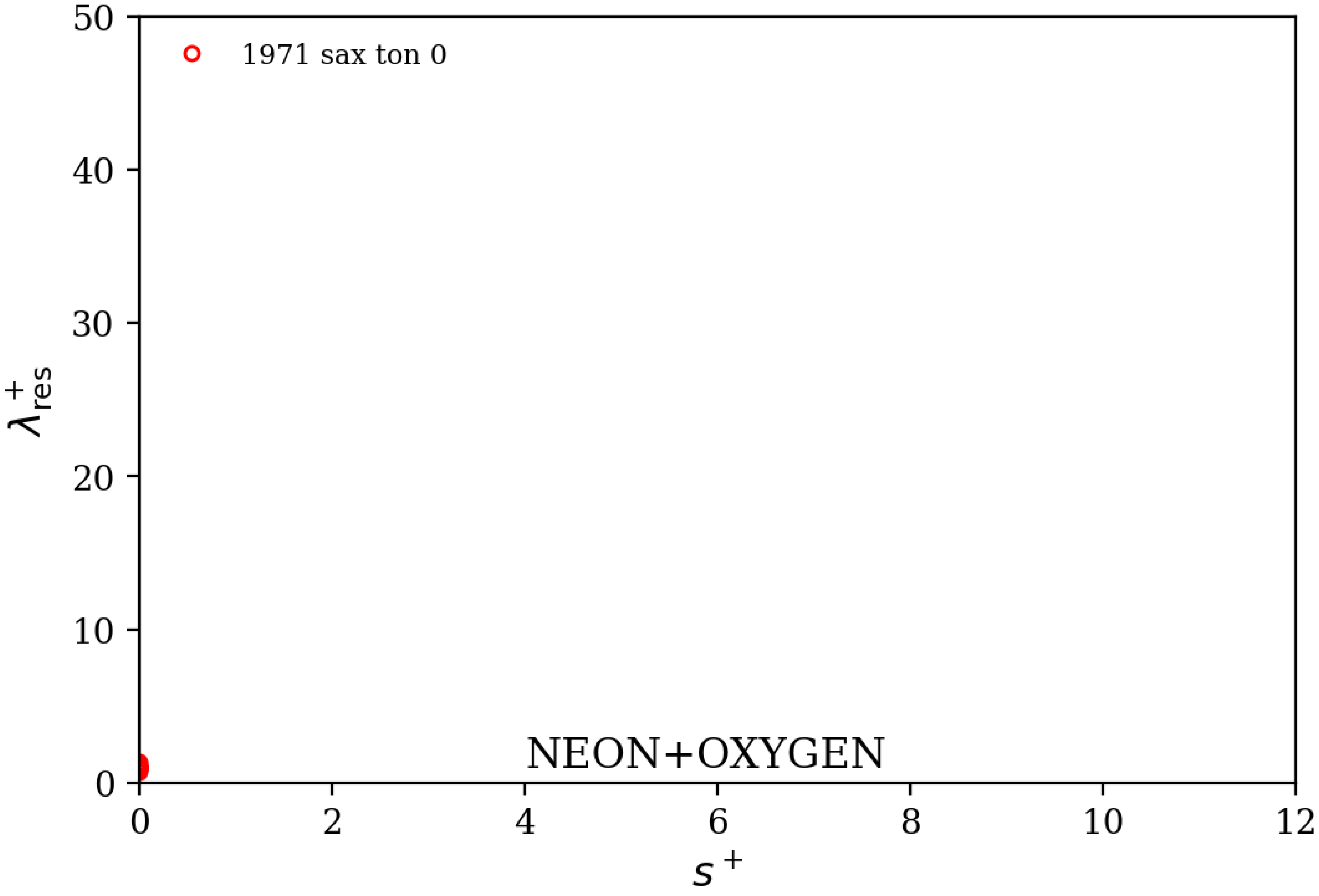

Figure DPR3. NEON+OXYGEN

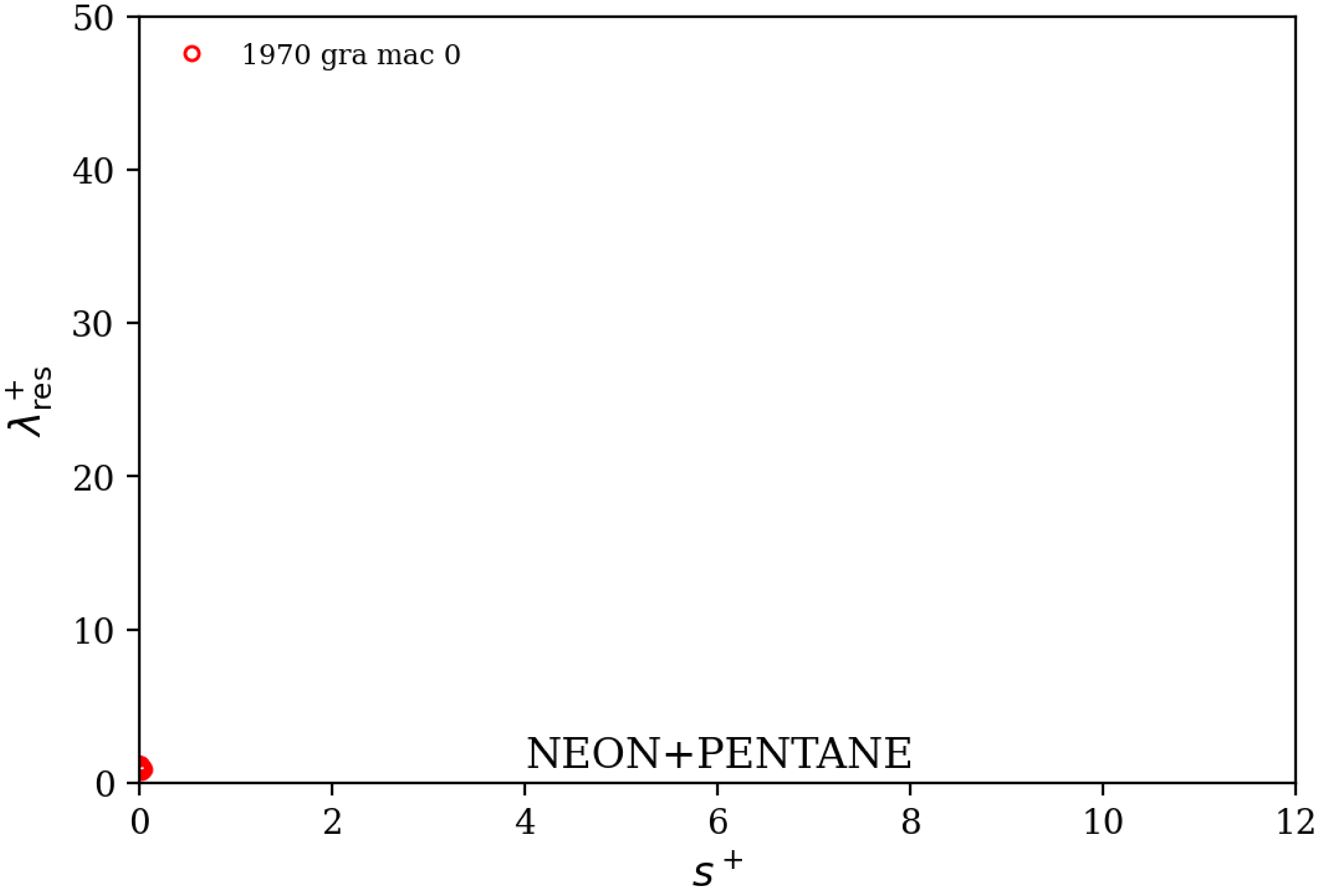

Figure DPR3. NEON+PENTANE

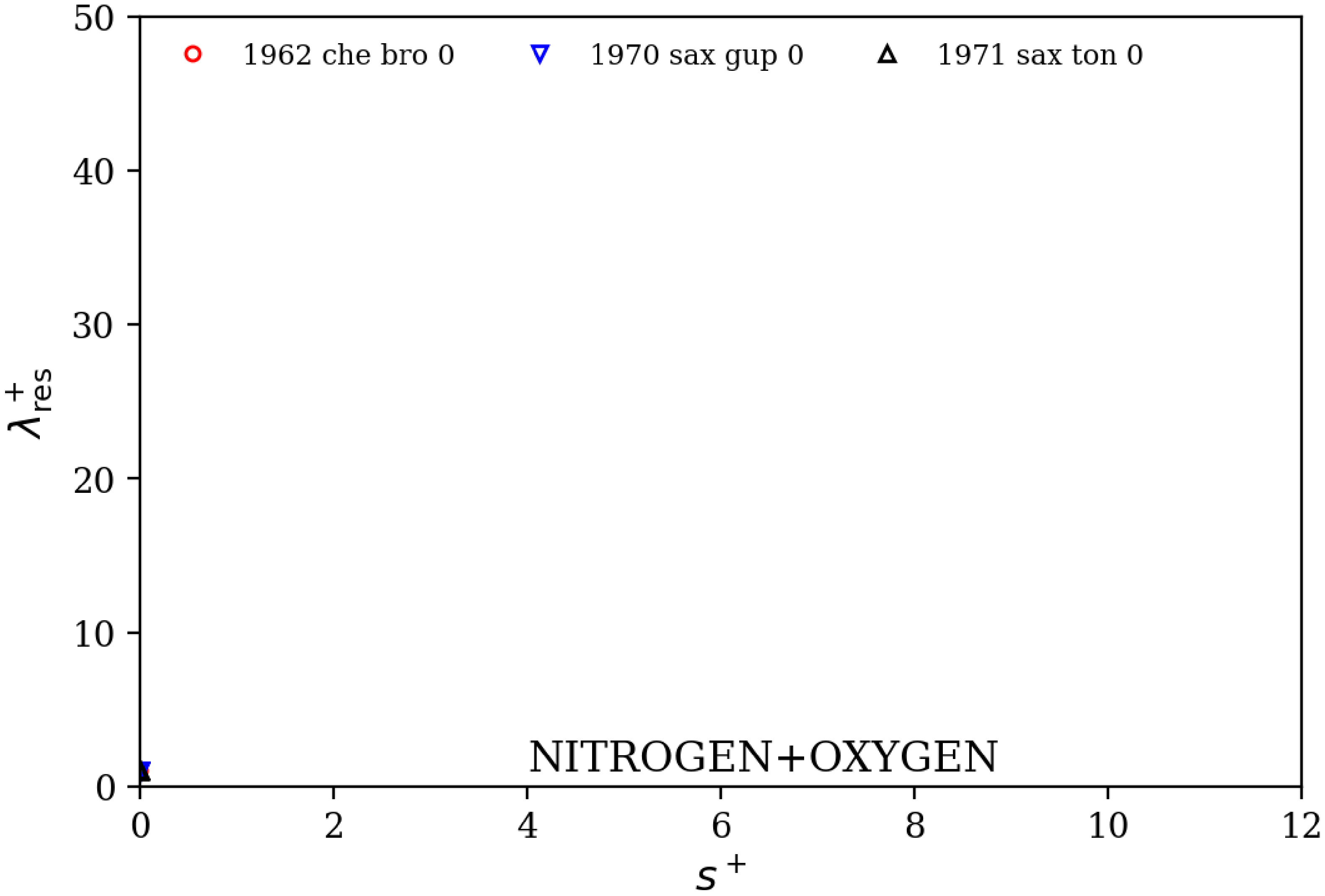

Figure DPR3. NITROGEN+OXYGEN

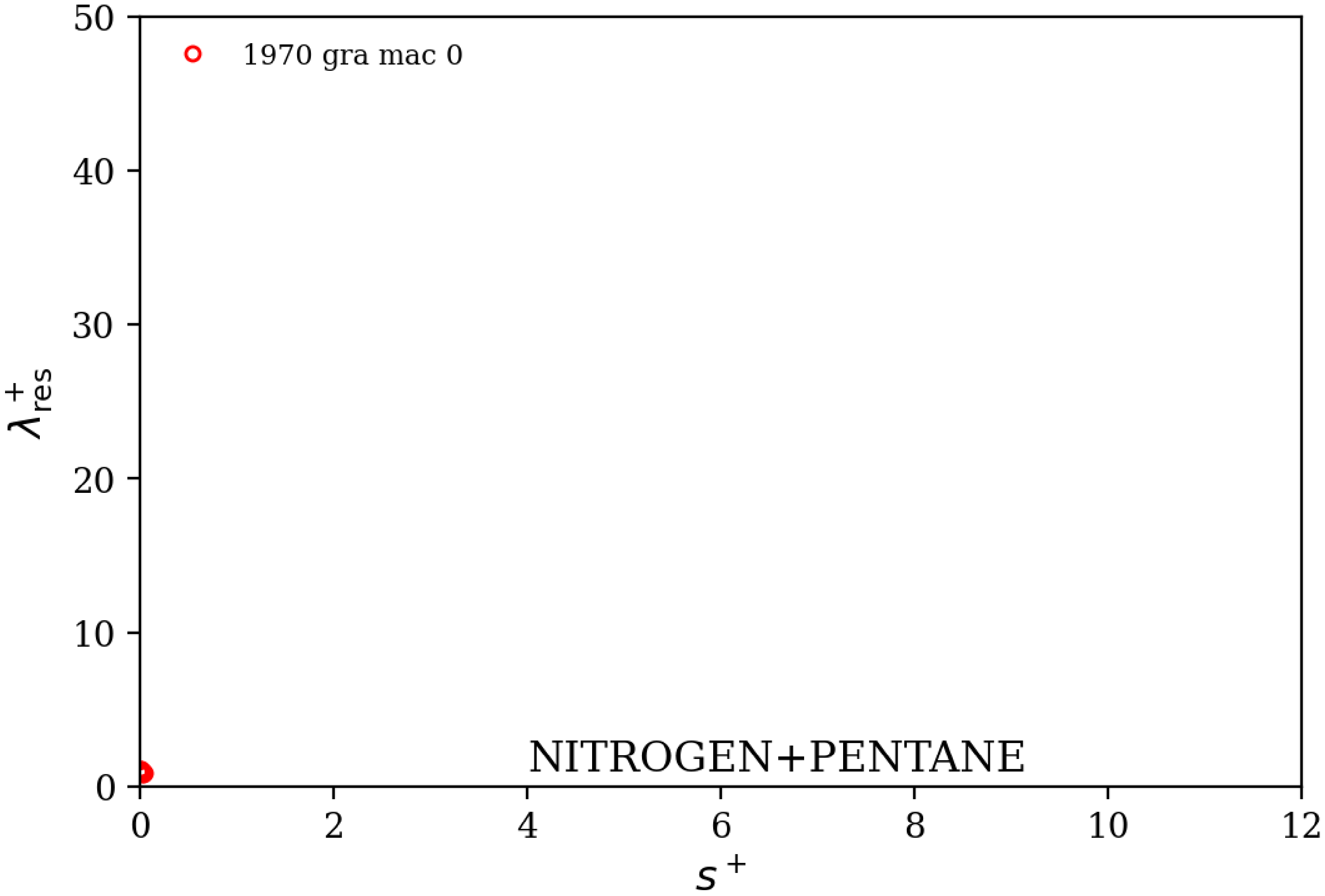

Figure DPR3. NITROGEN+PENTANE

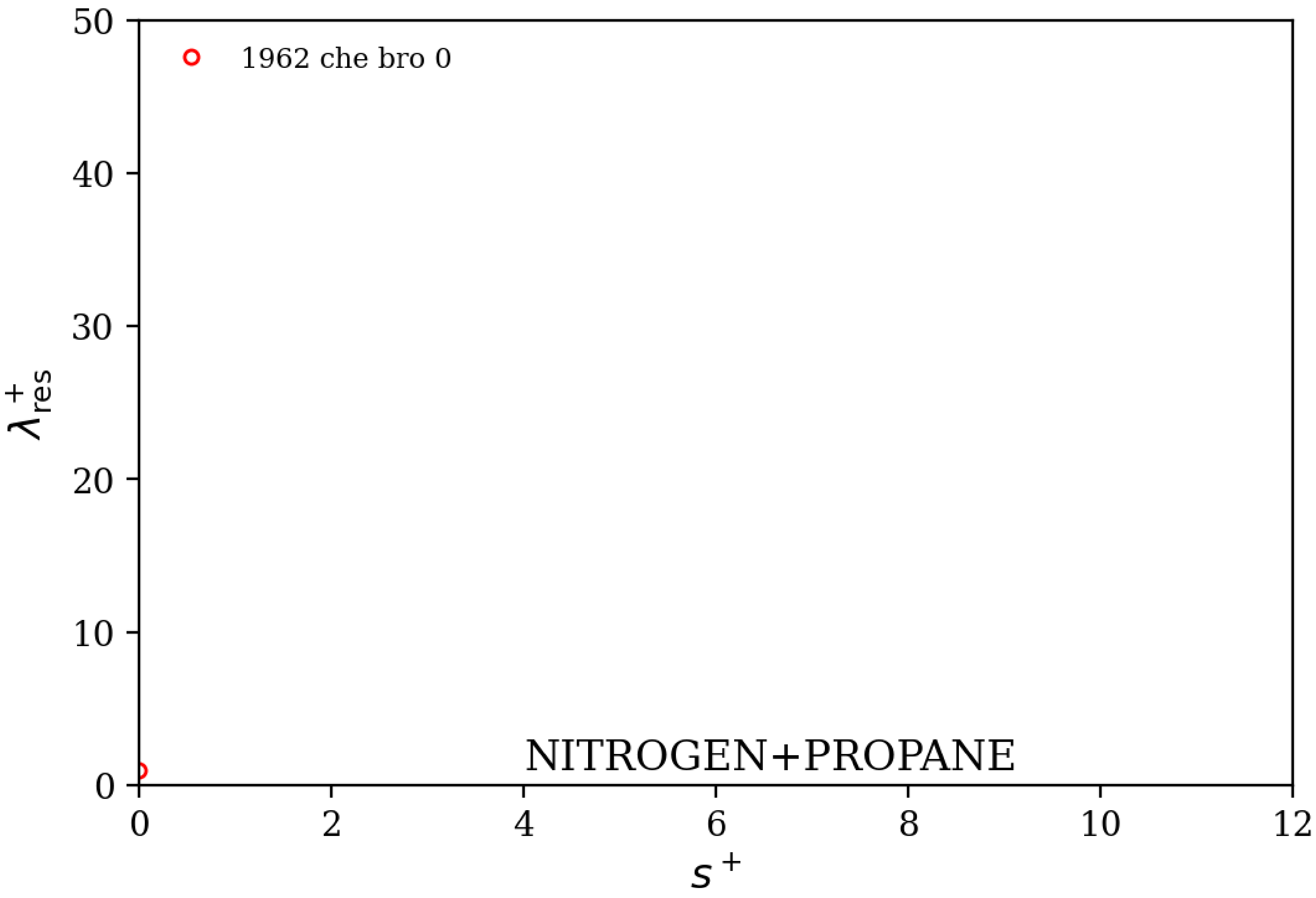

Figure DPR3. NITROGEN+PROPANE

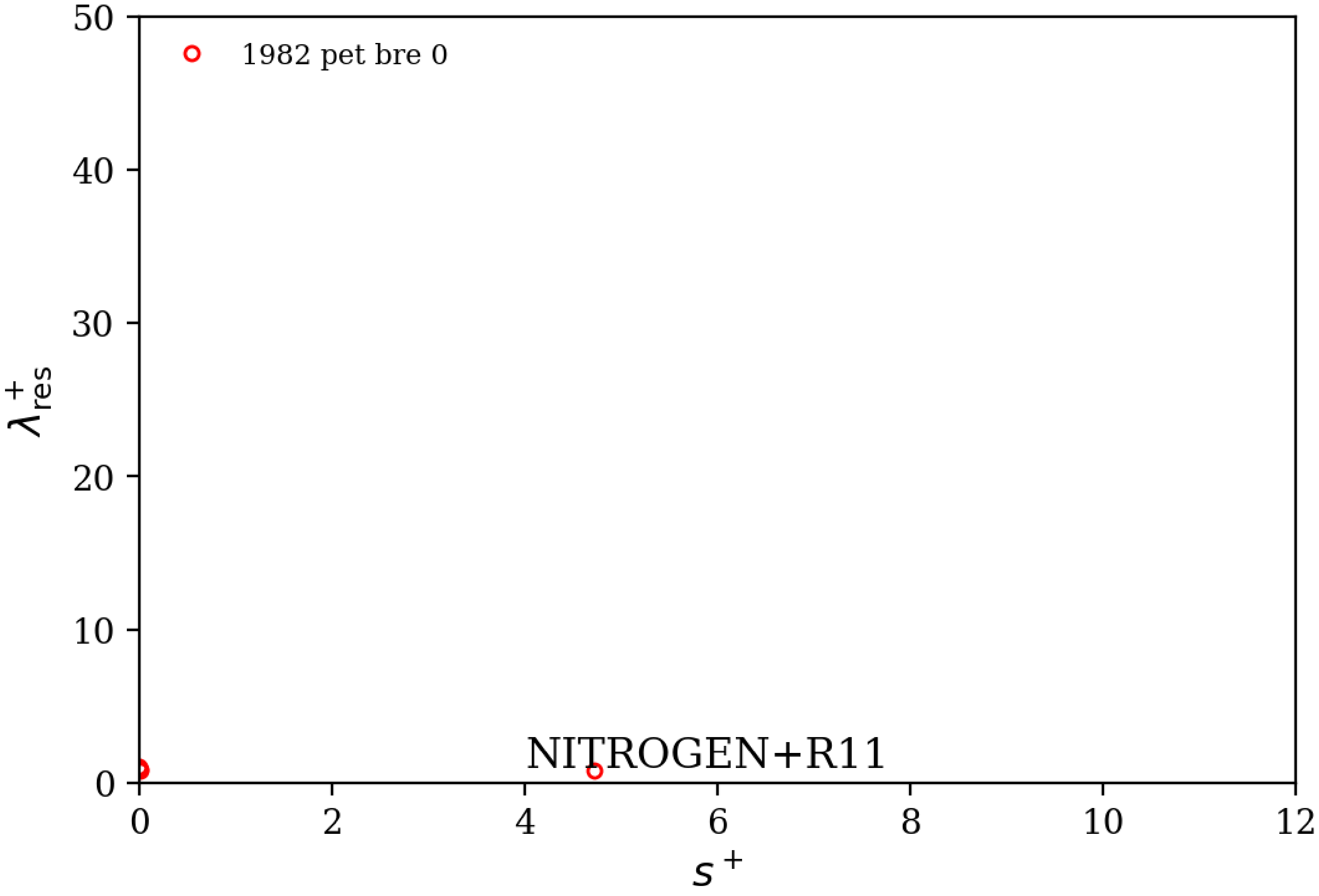

Figure DPR3. NITROGEN+R11

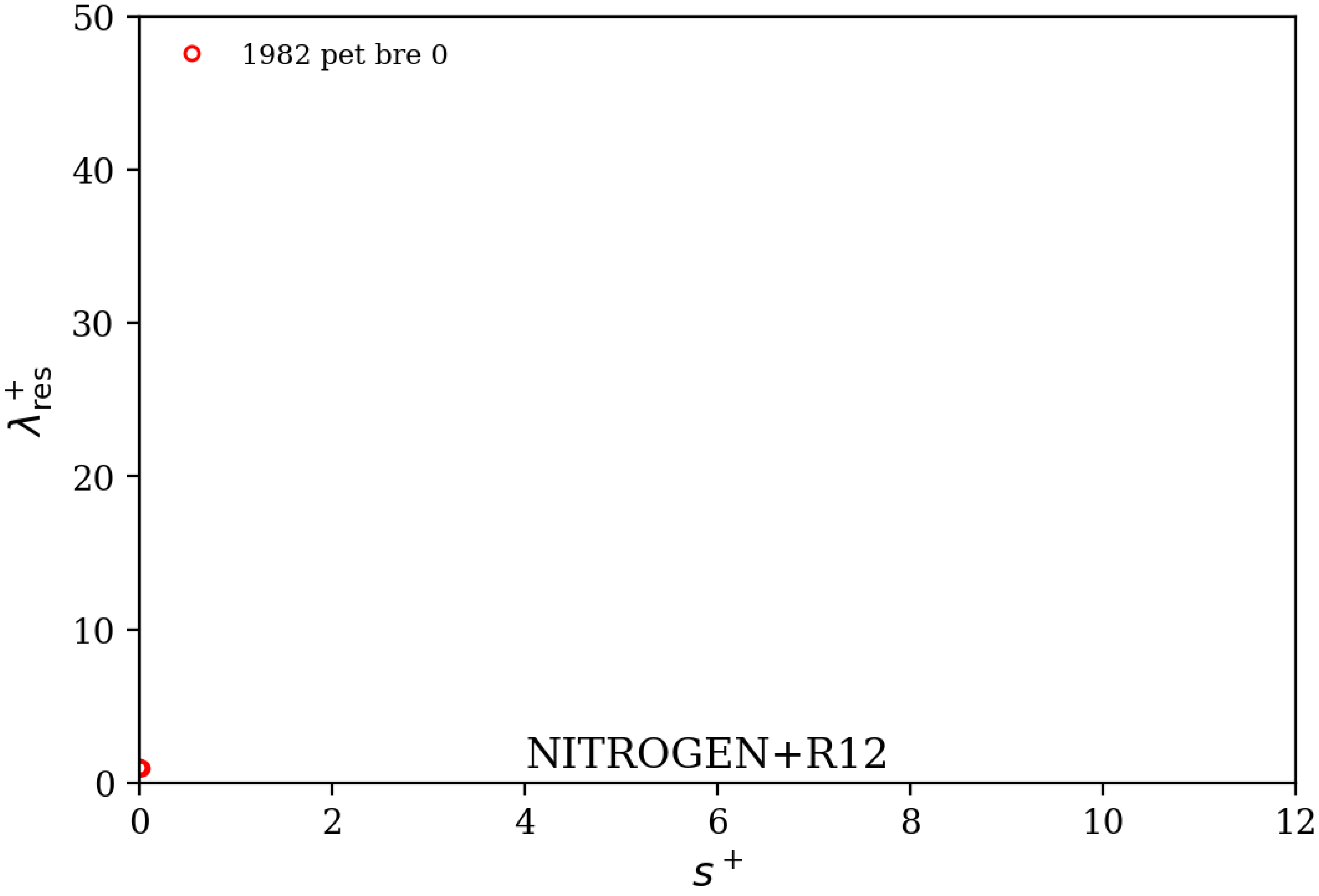

Figure DPR3. NITROGEN+R12

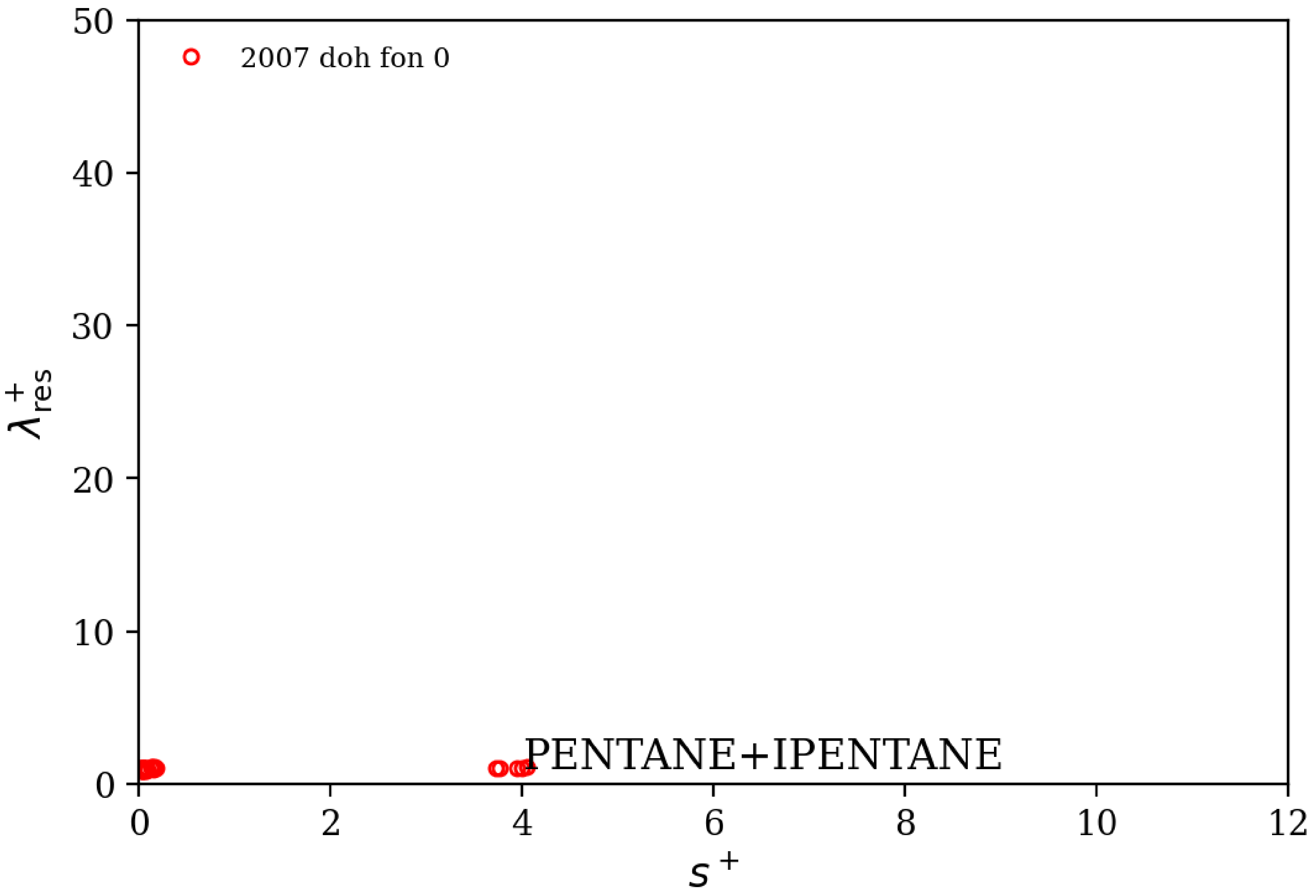

Figure DPR3.PENTANE+IPENTANE

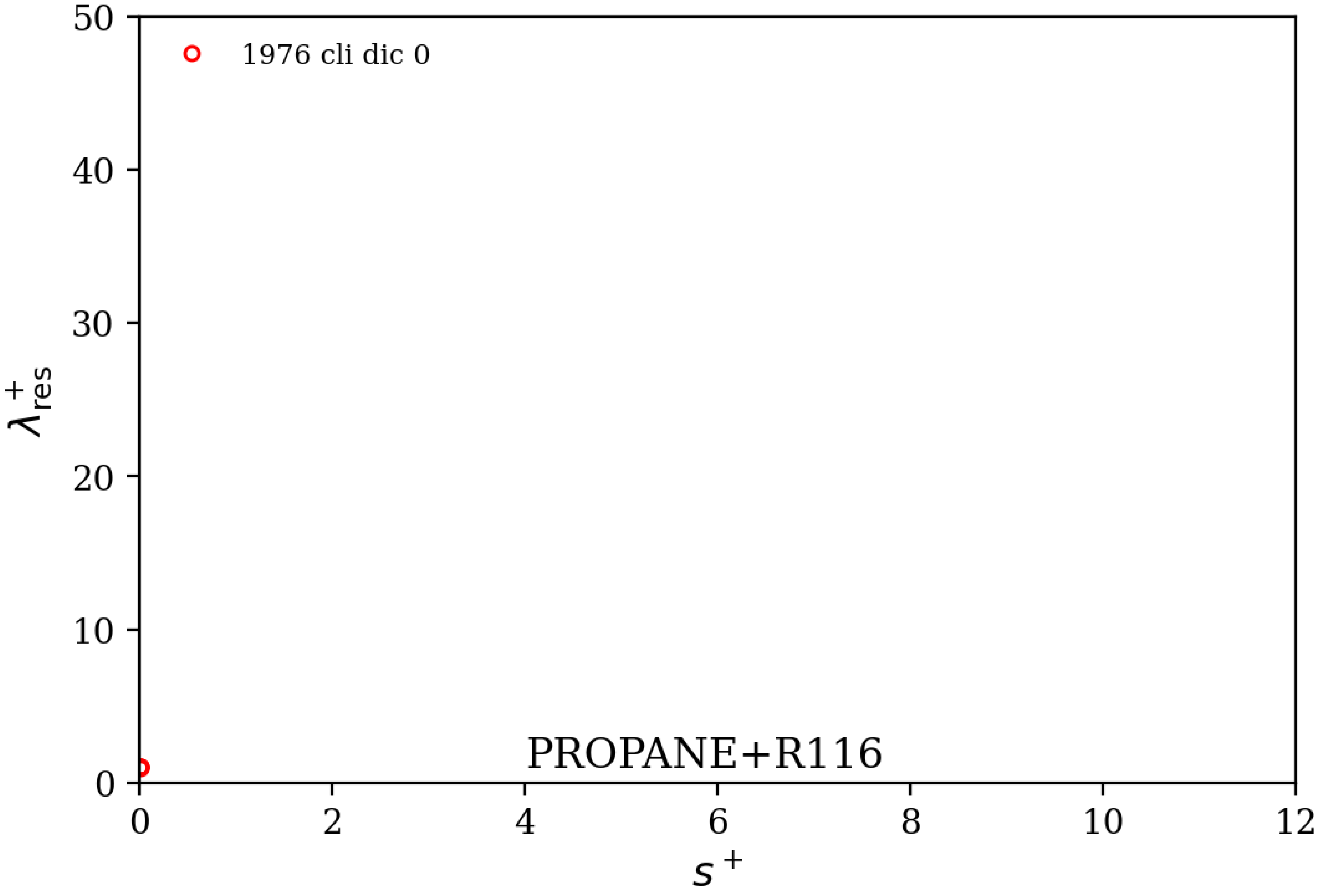

Figure DPR3. PROPANE+R116

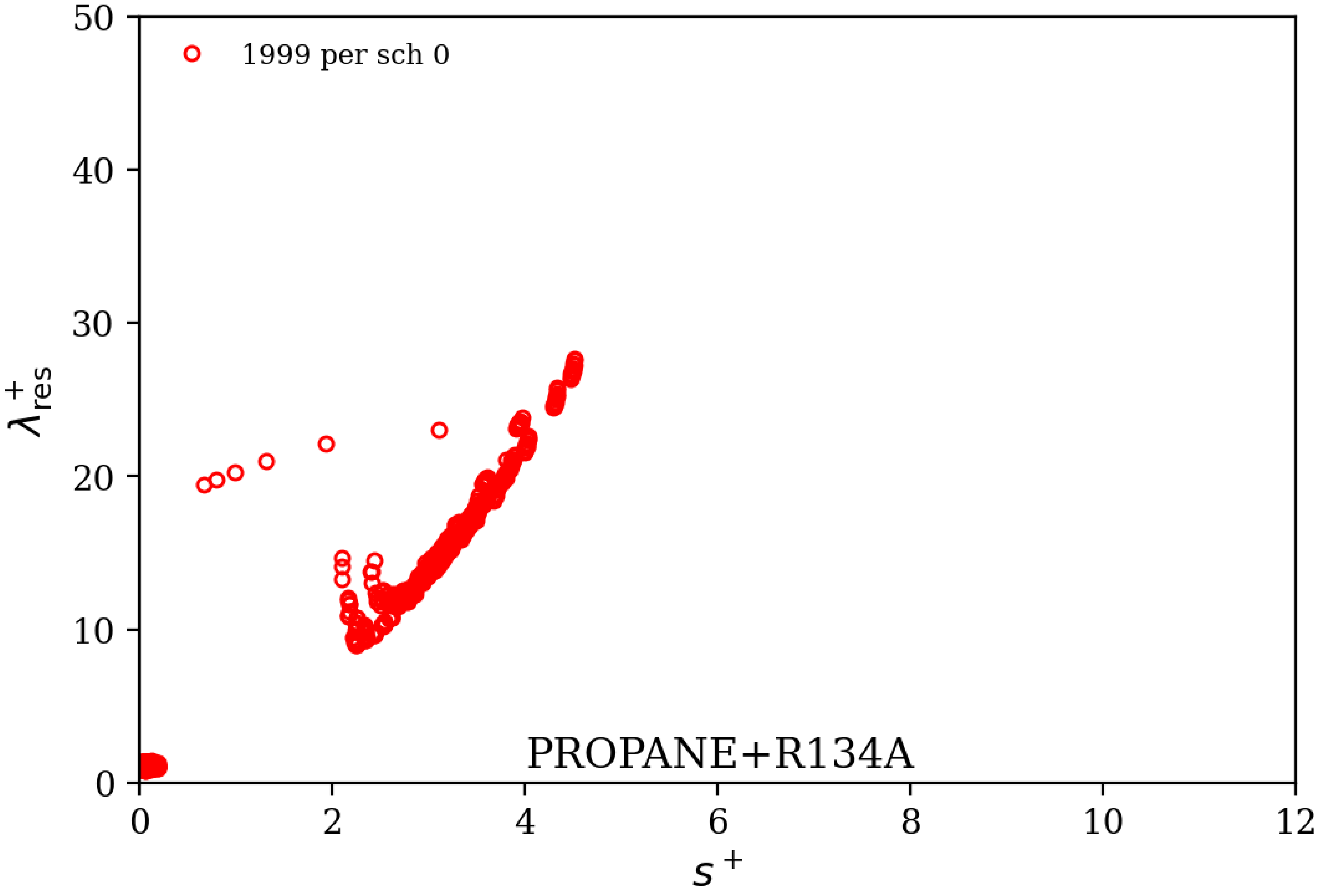

Figure DPR3. PROPANE+R134A

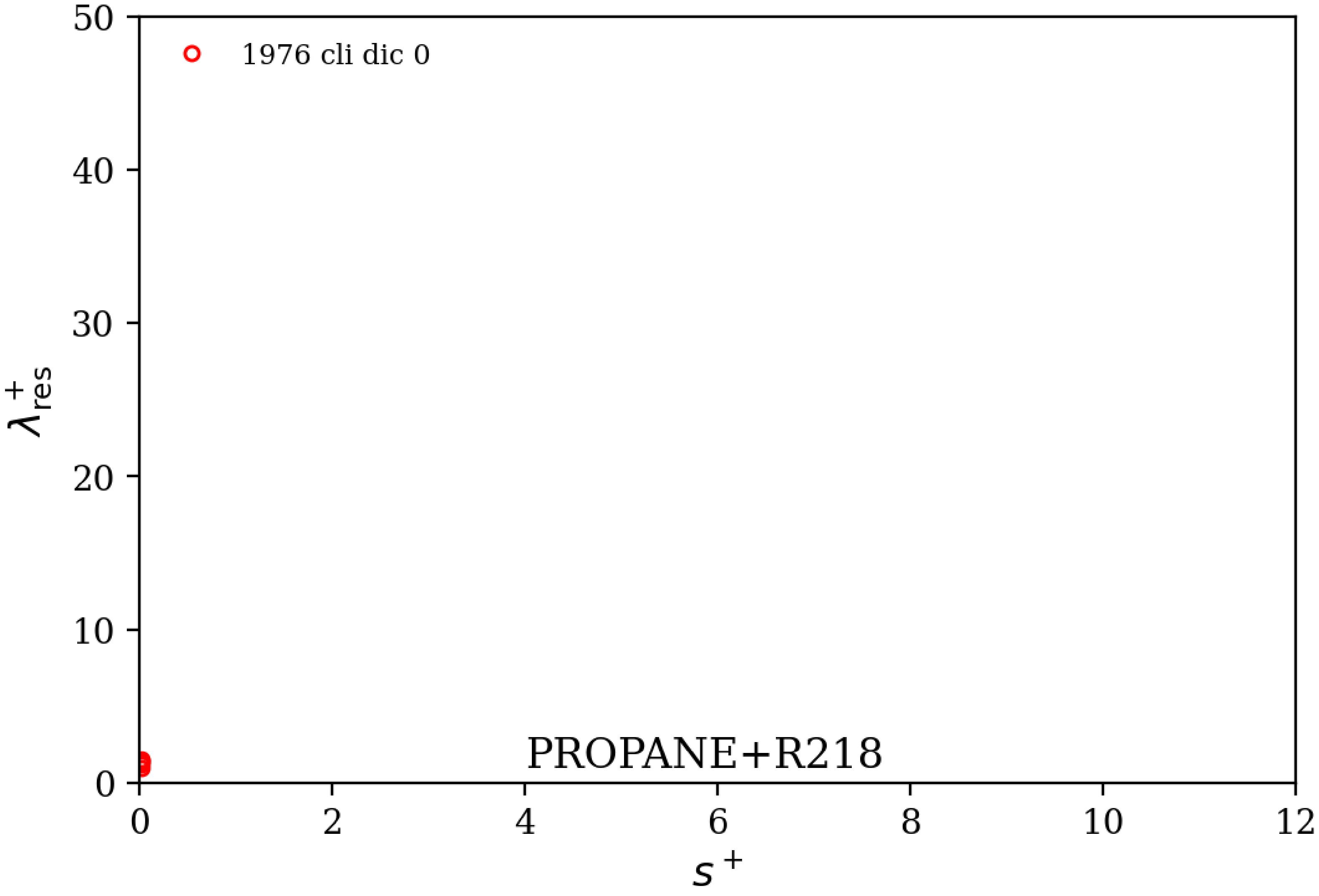

Figure DPR3. PROPANE+R218

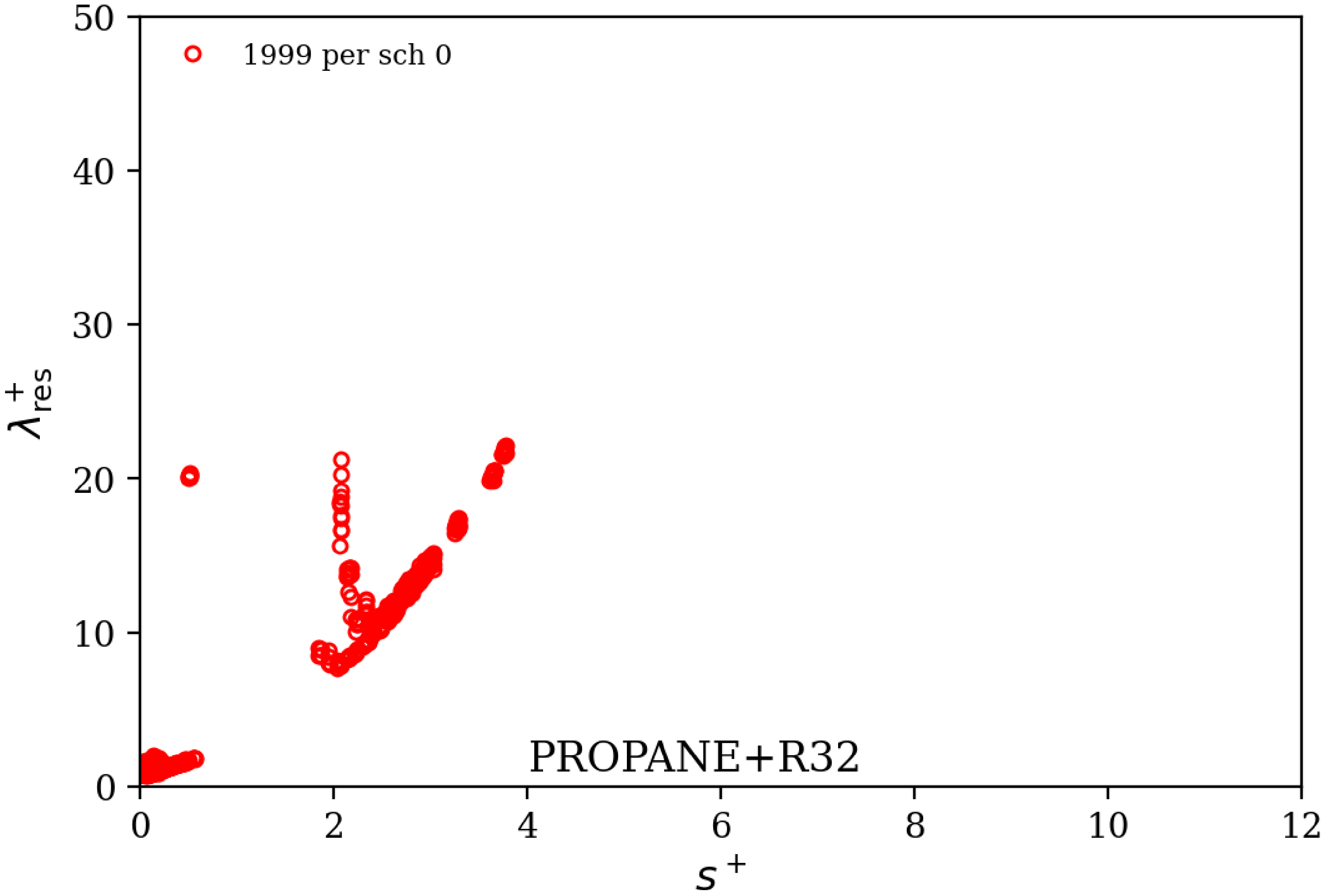

Figure DPR3. PROPANE+R32

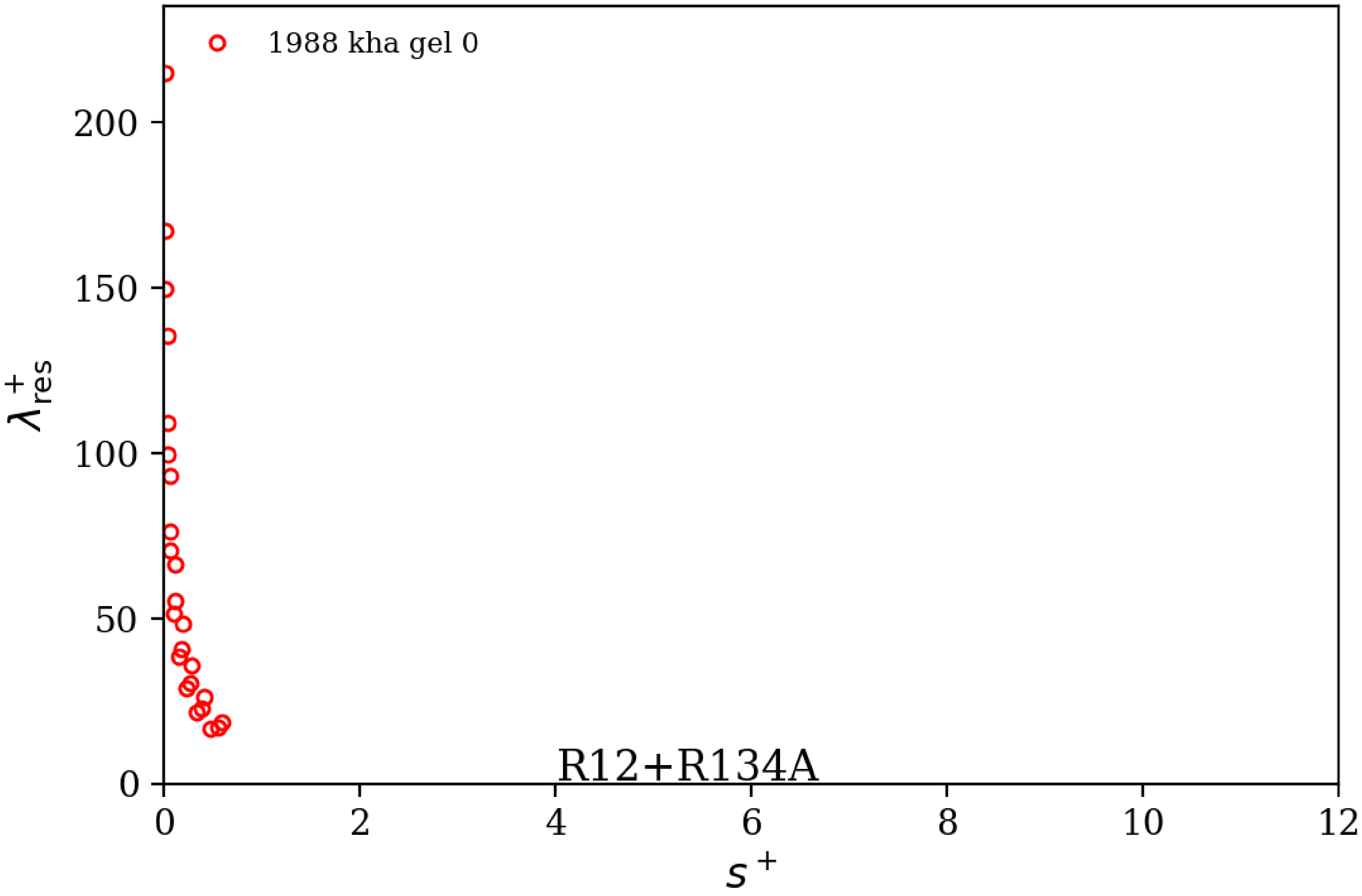

Figure DPR3. R12+R134A

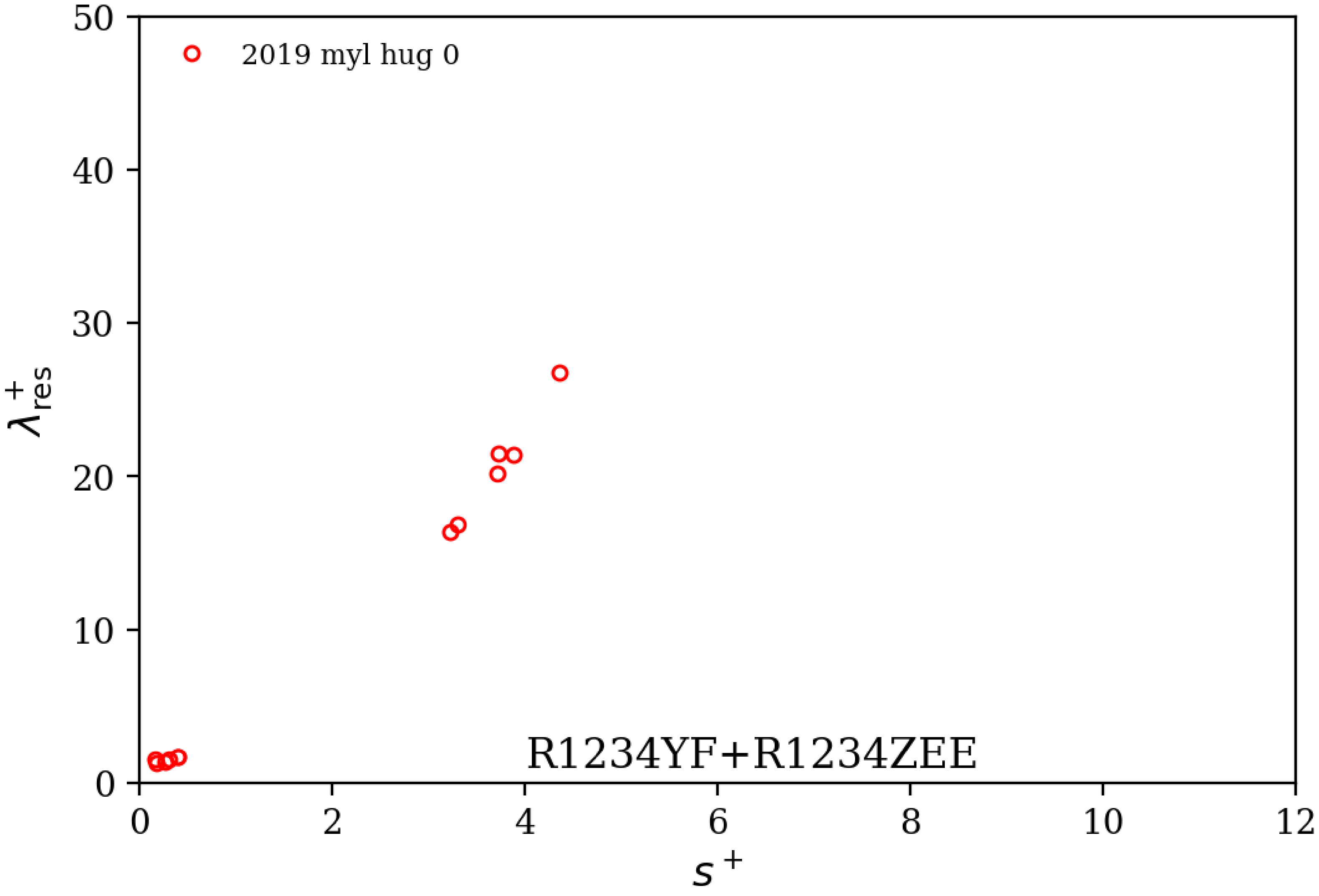

Figure DPR3. R1234YF+R1234ZEE

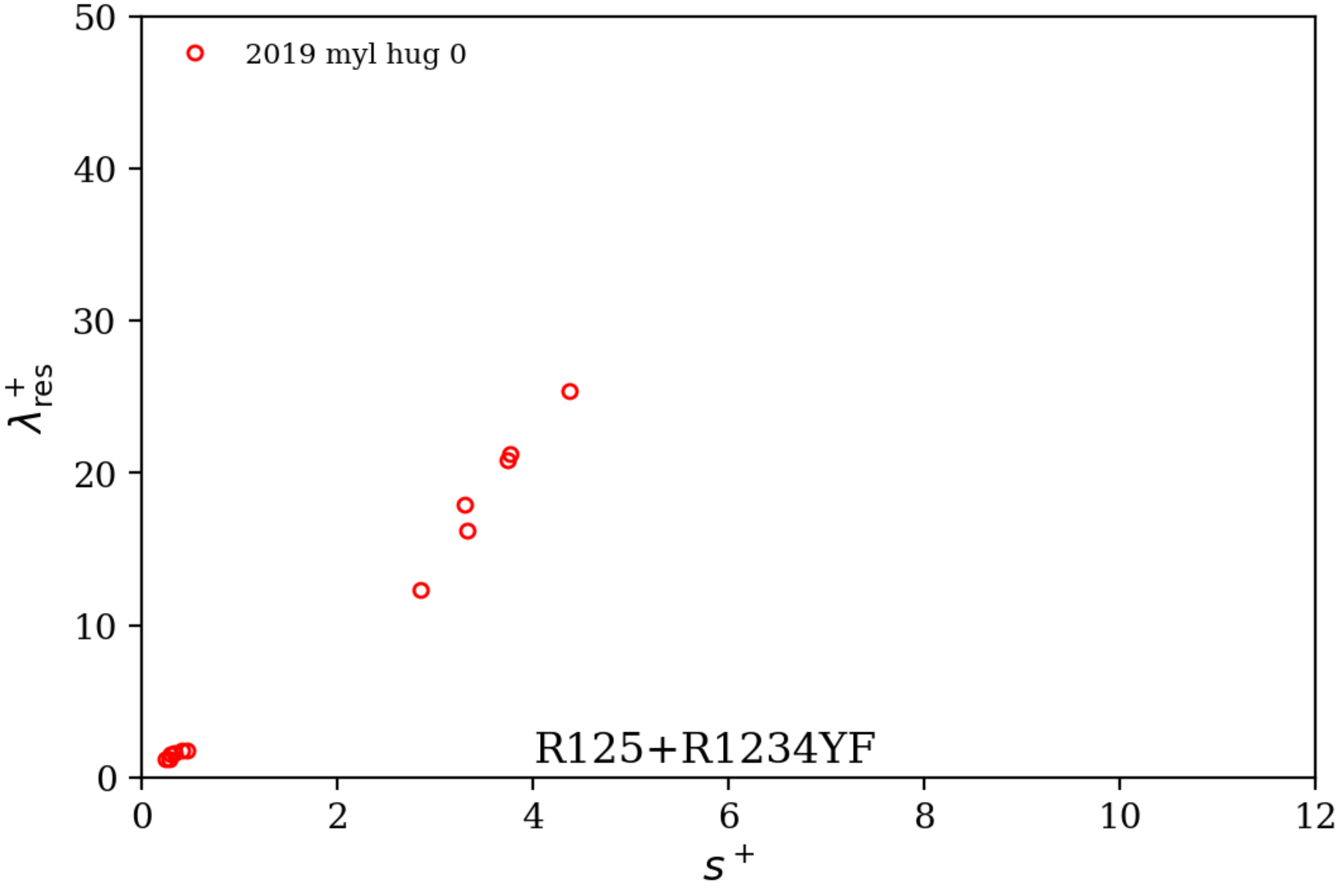

Figure DPR3. R125+R1234YF

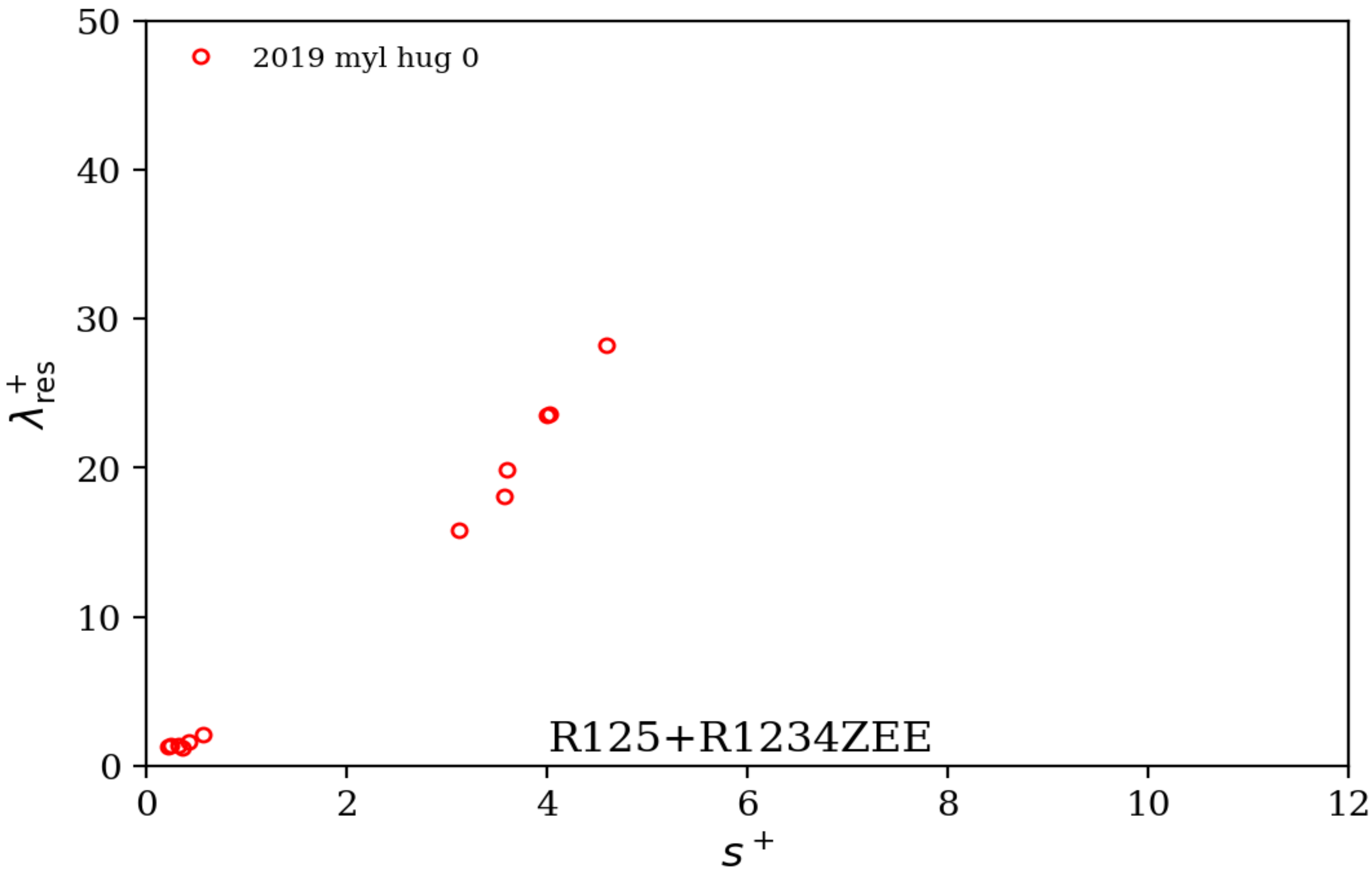

Figure DPR3. R125+R1234ZEE

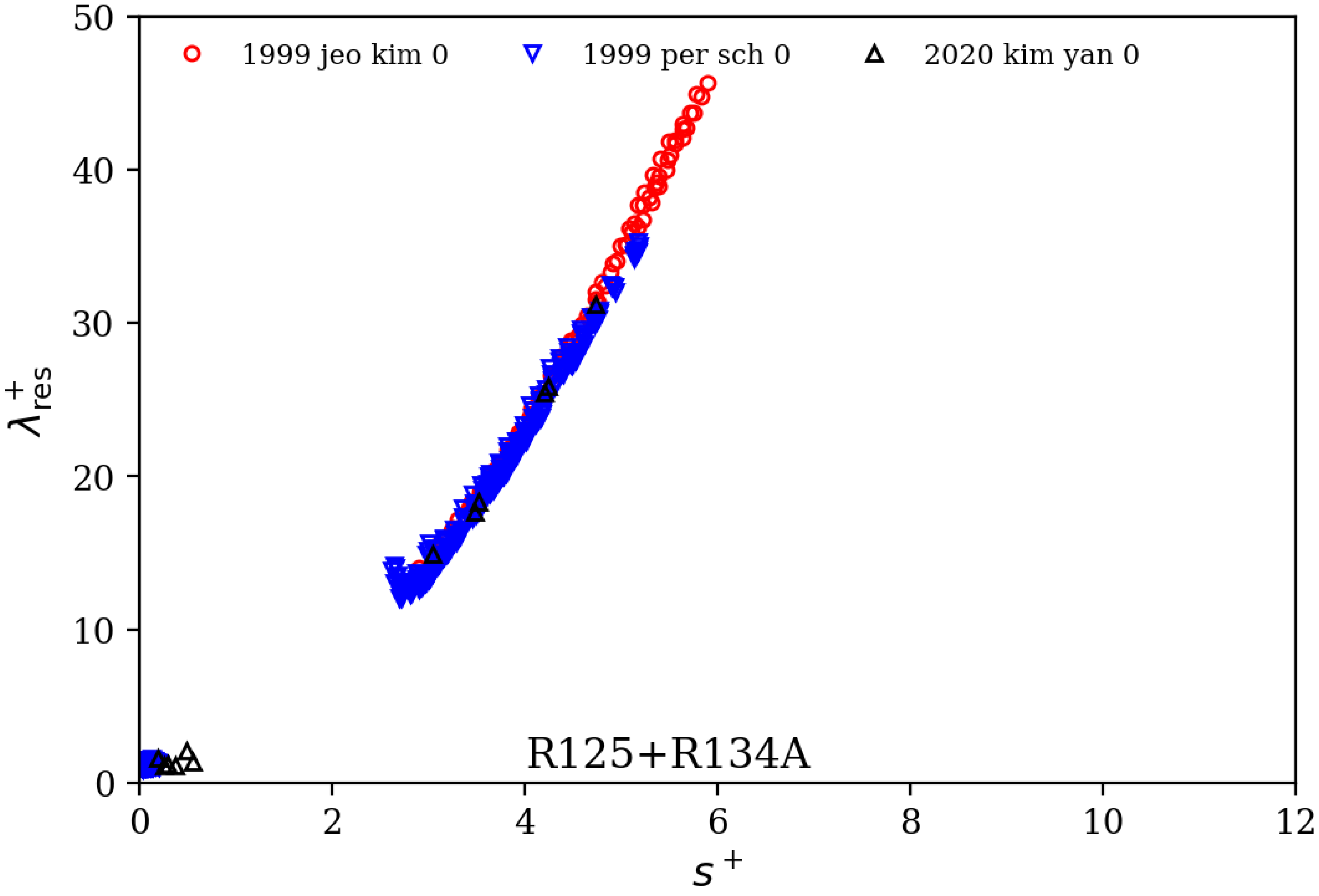

Figure DPR3. R125+R134A

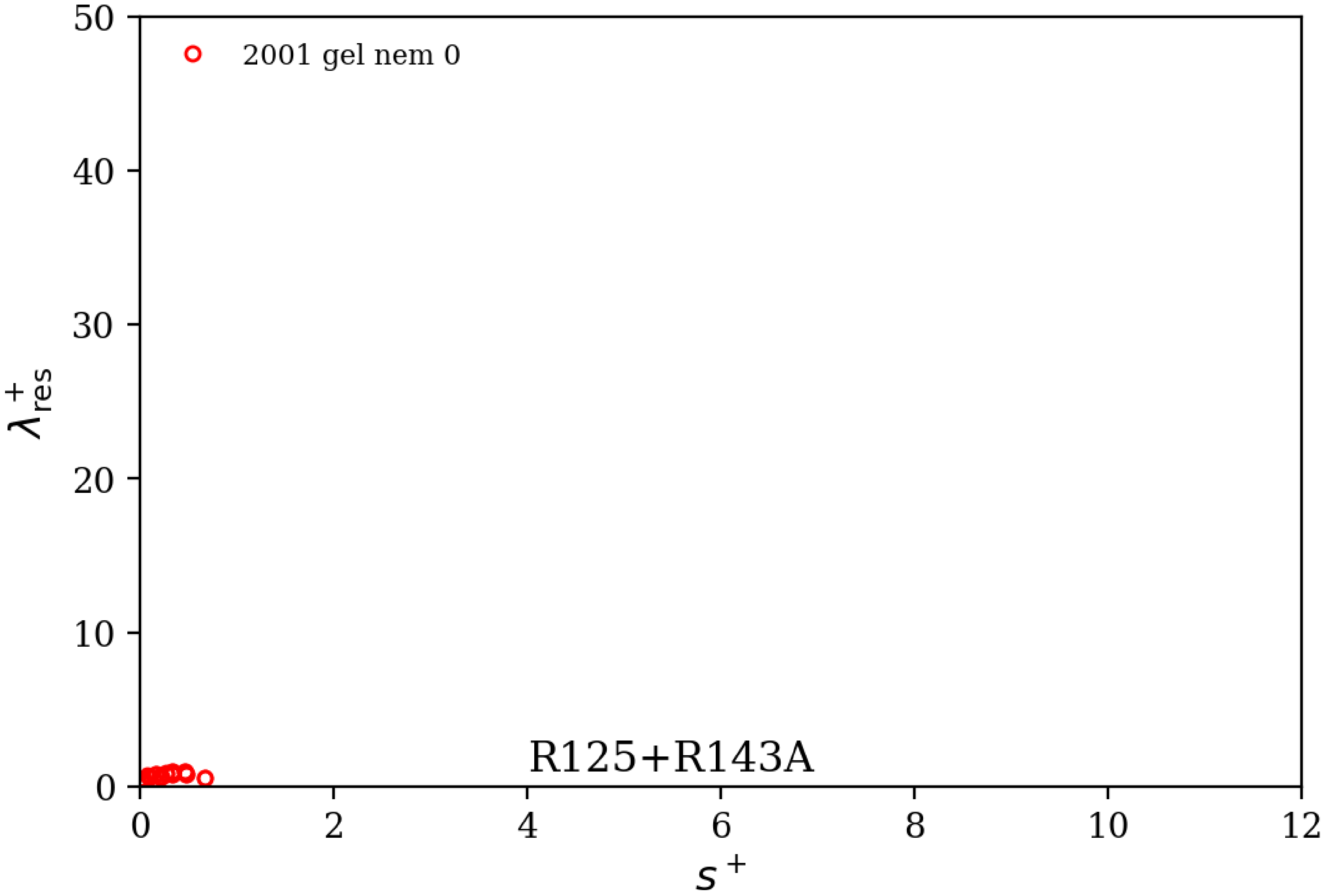

Figure DPR3. R125+R143A

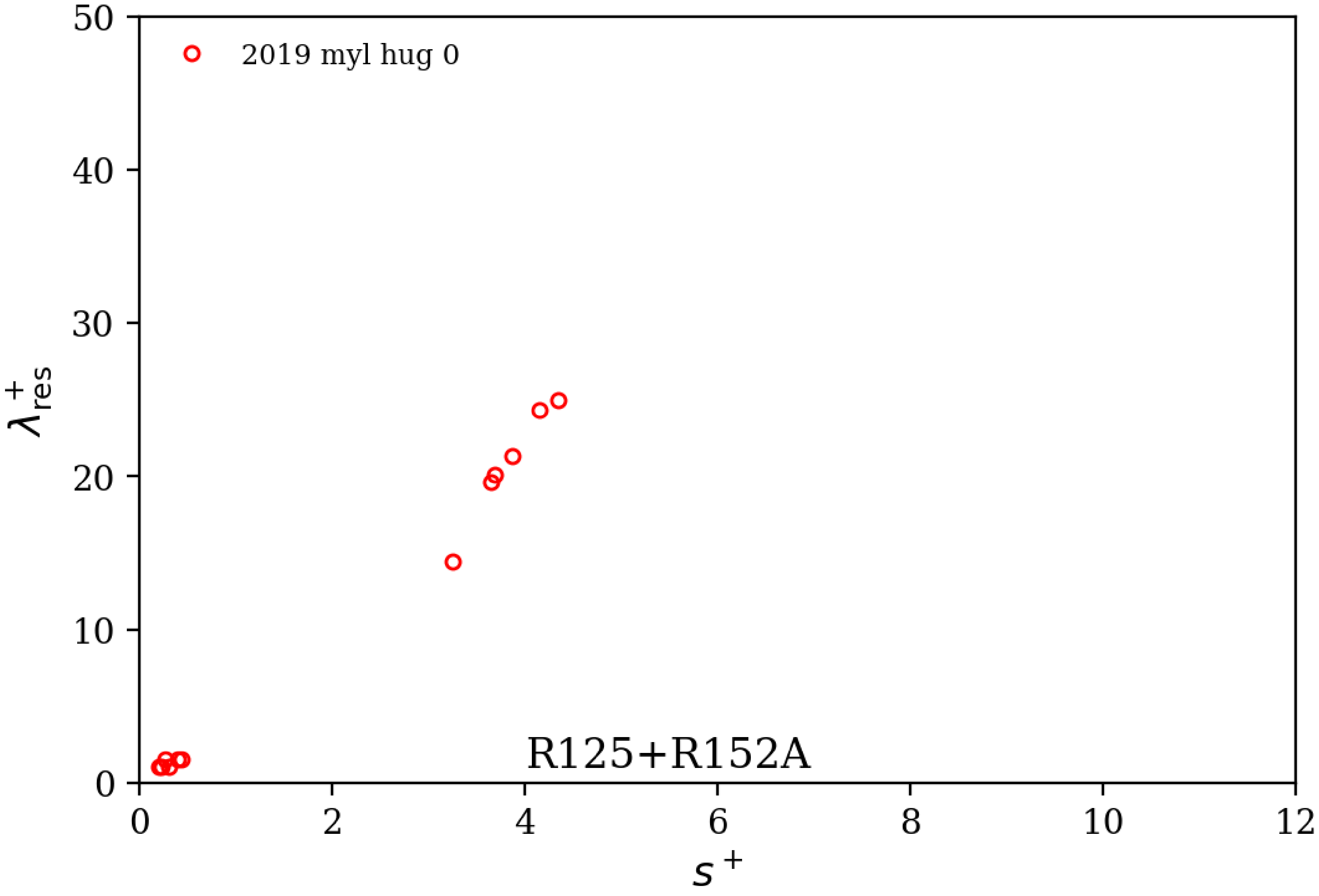

Figure DPR3. R125+R152A

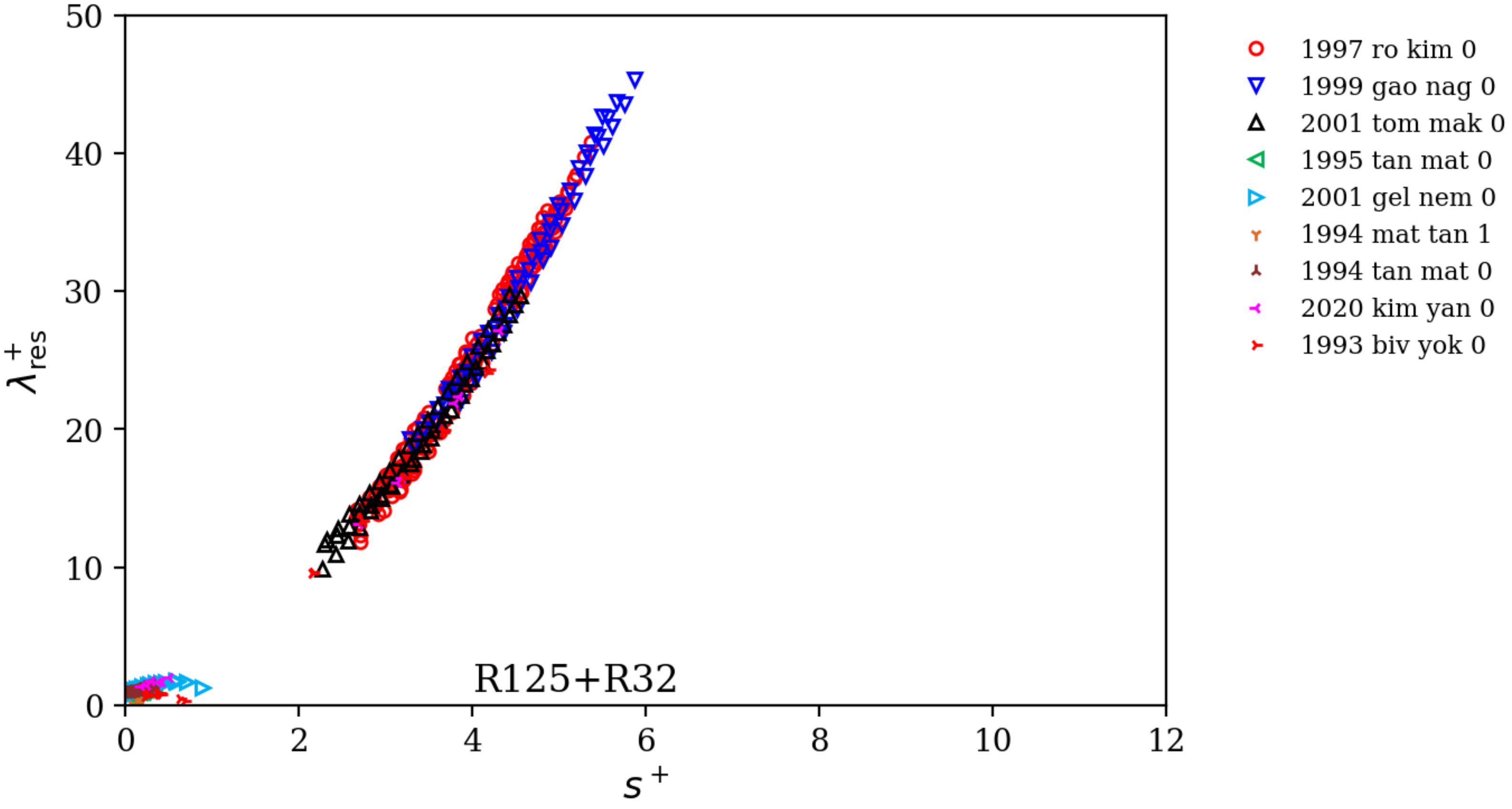

Figure DPR3. R125+R32

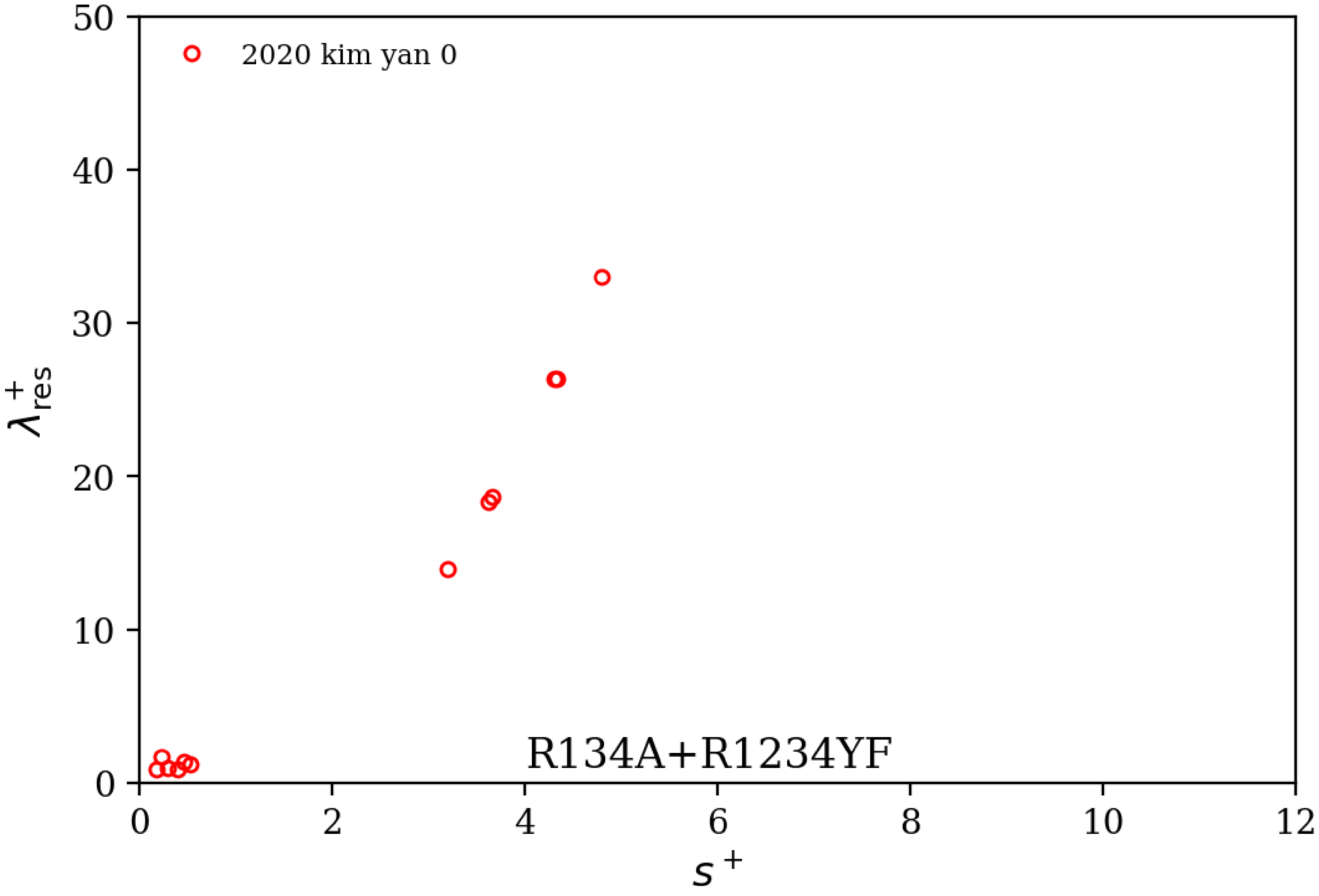

Figure DPR3. R134A+R1234YF

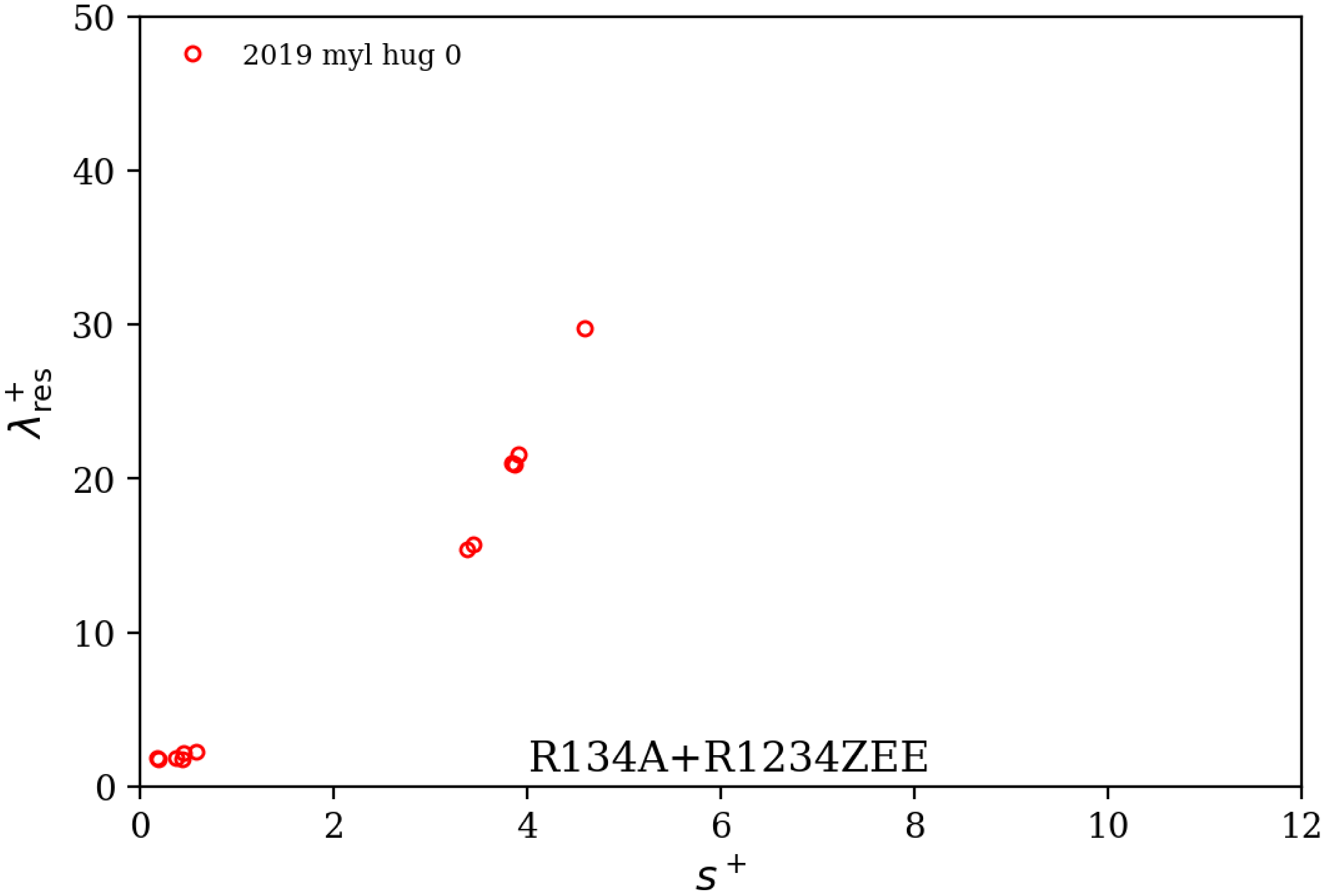

Figure DPR3. R134A+R1234ZEE

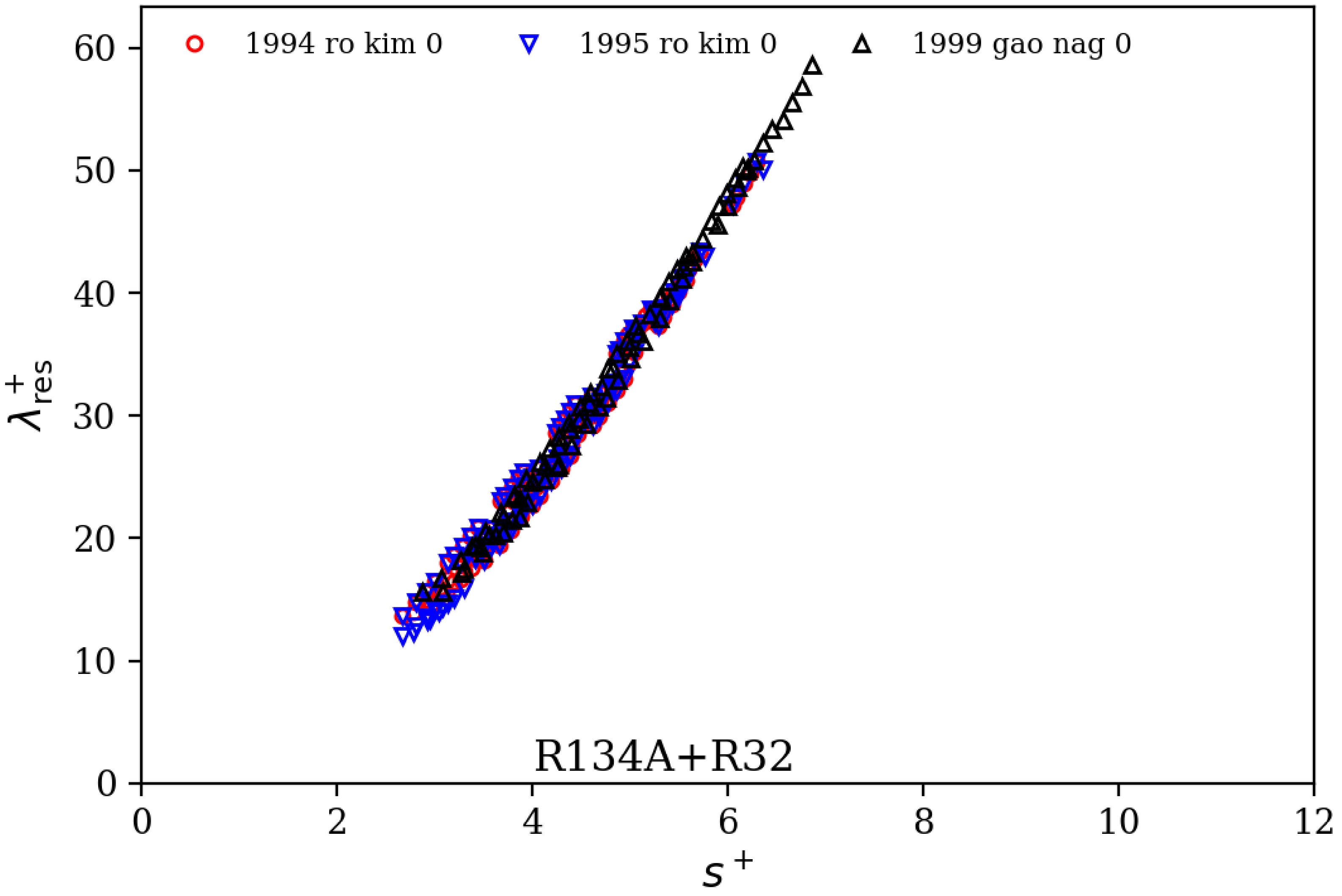

Figure DPR3. R134A+R32

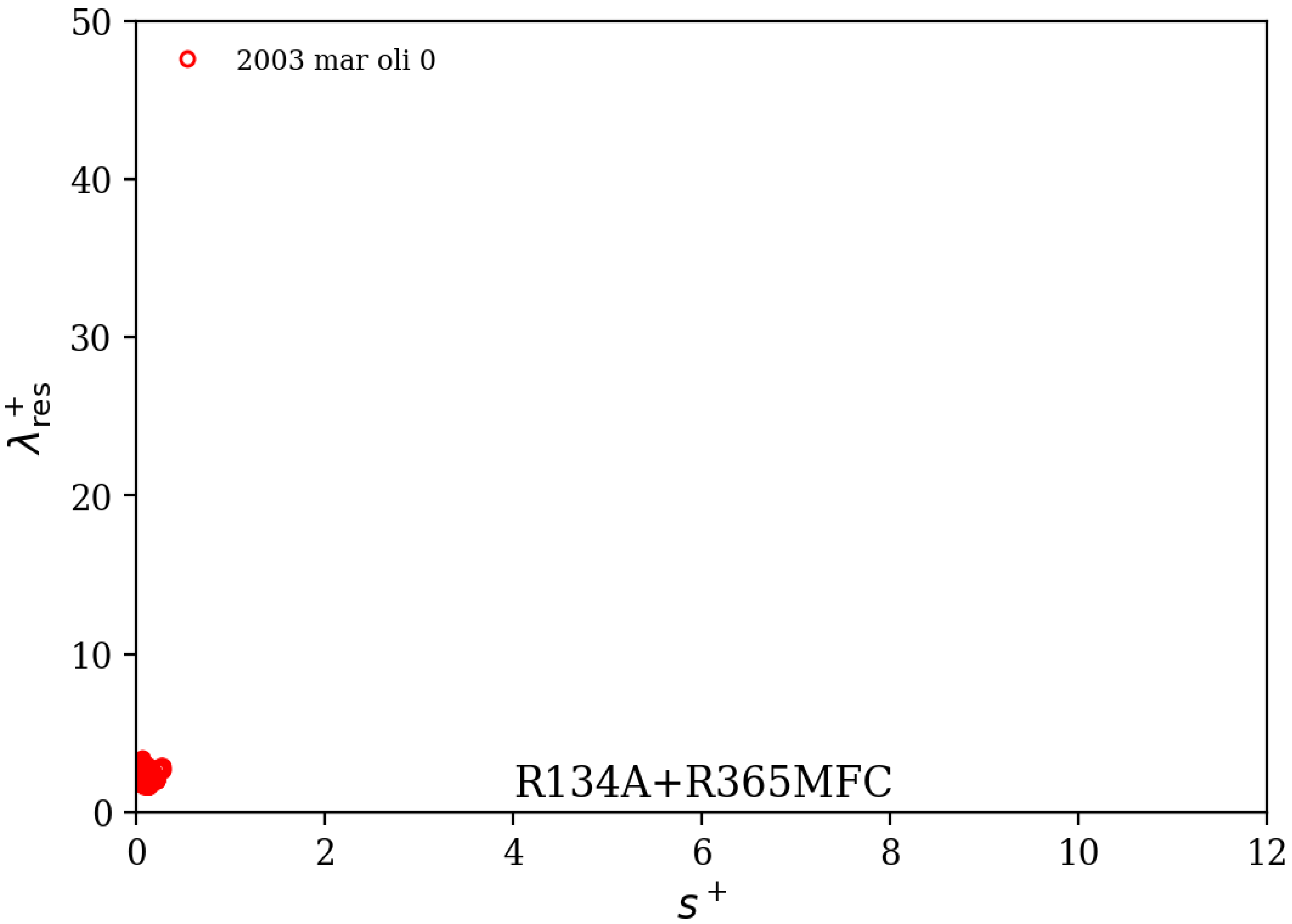

Figure DPR3. R134A+R365MFC

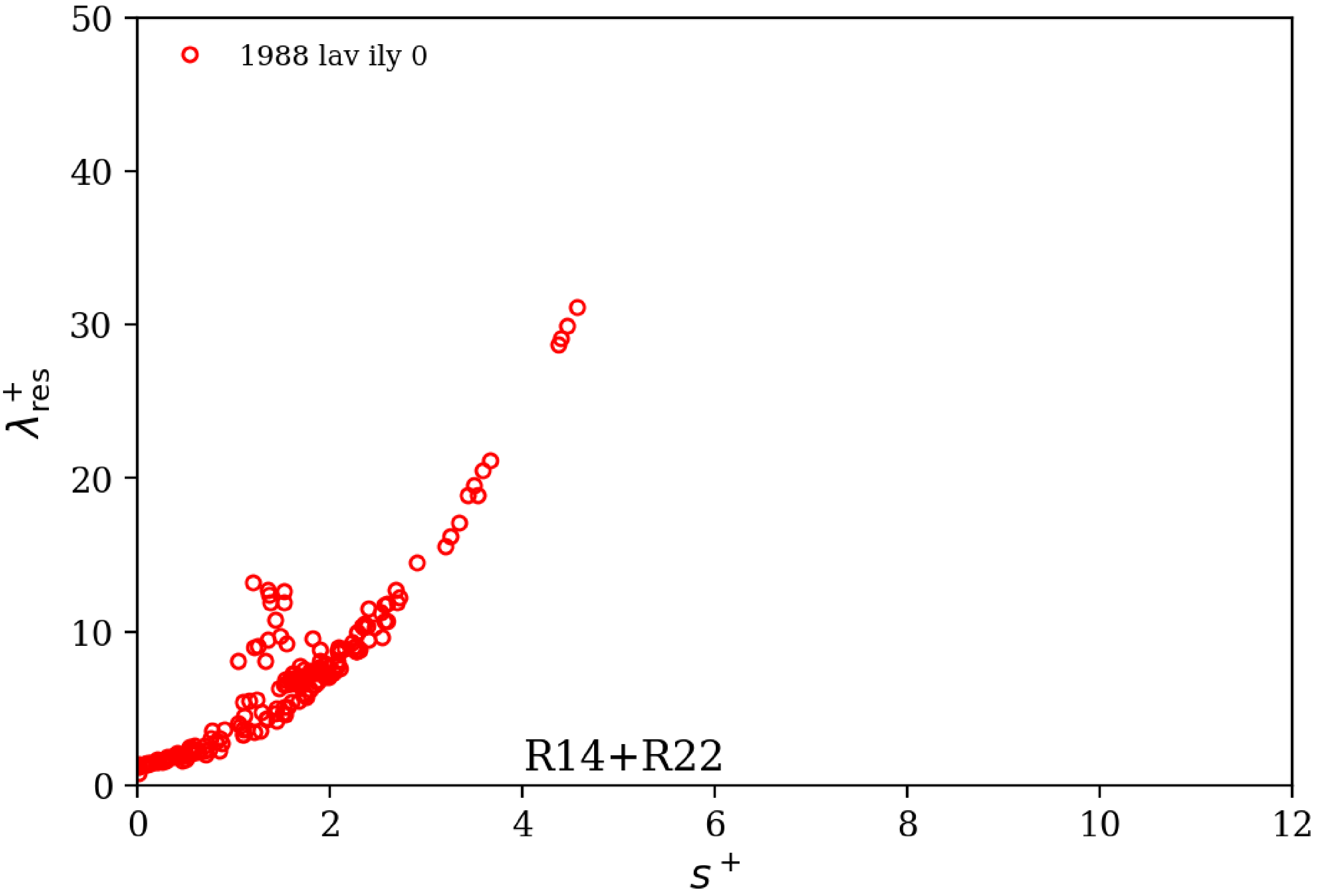

Figure DPR3. R14+R22

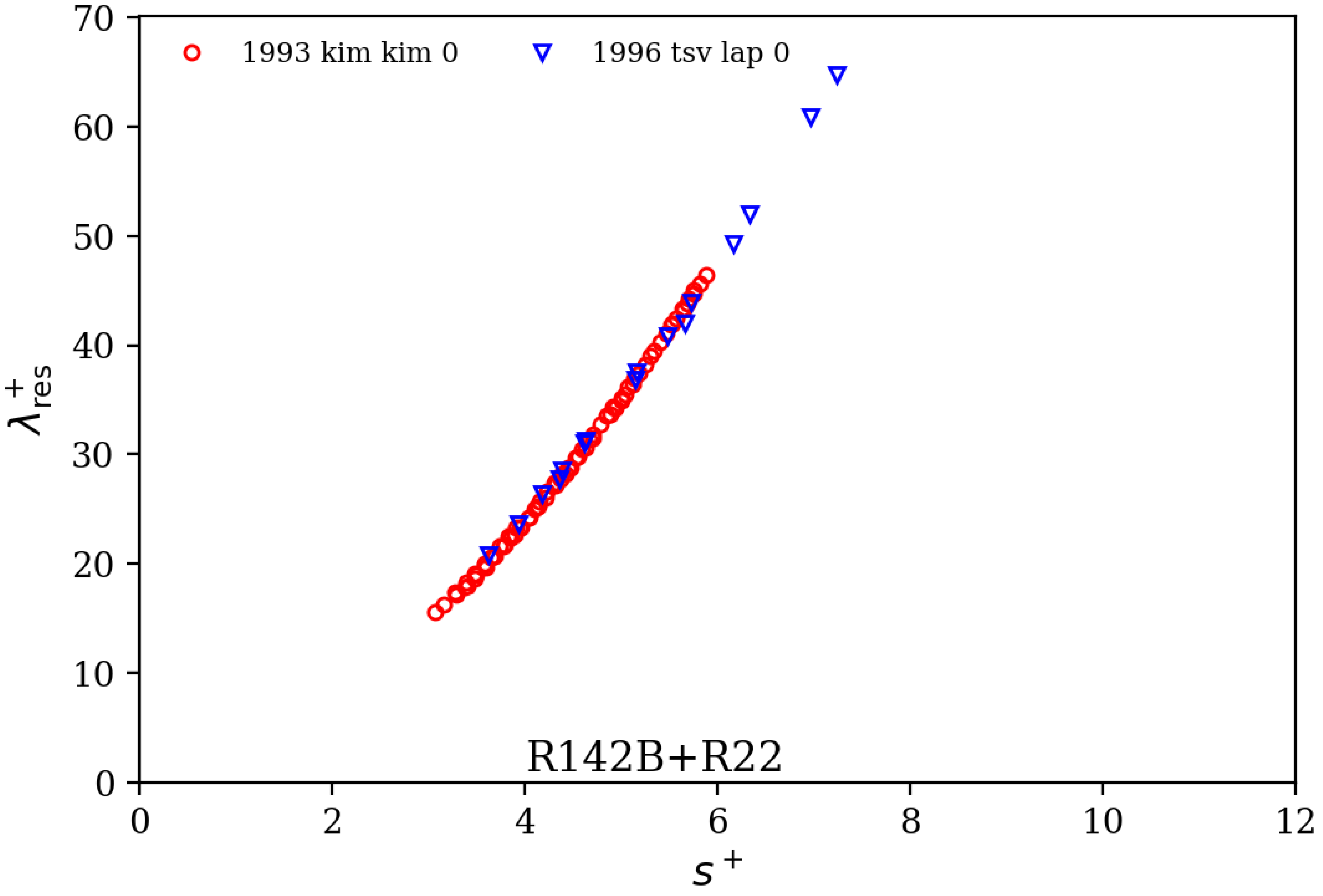

Figure DPR3. R142B+R22

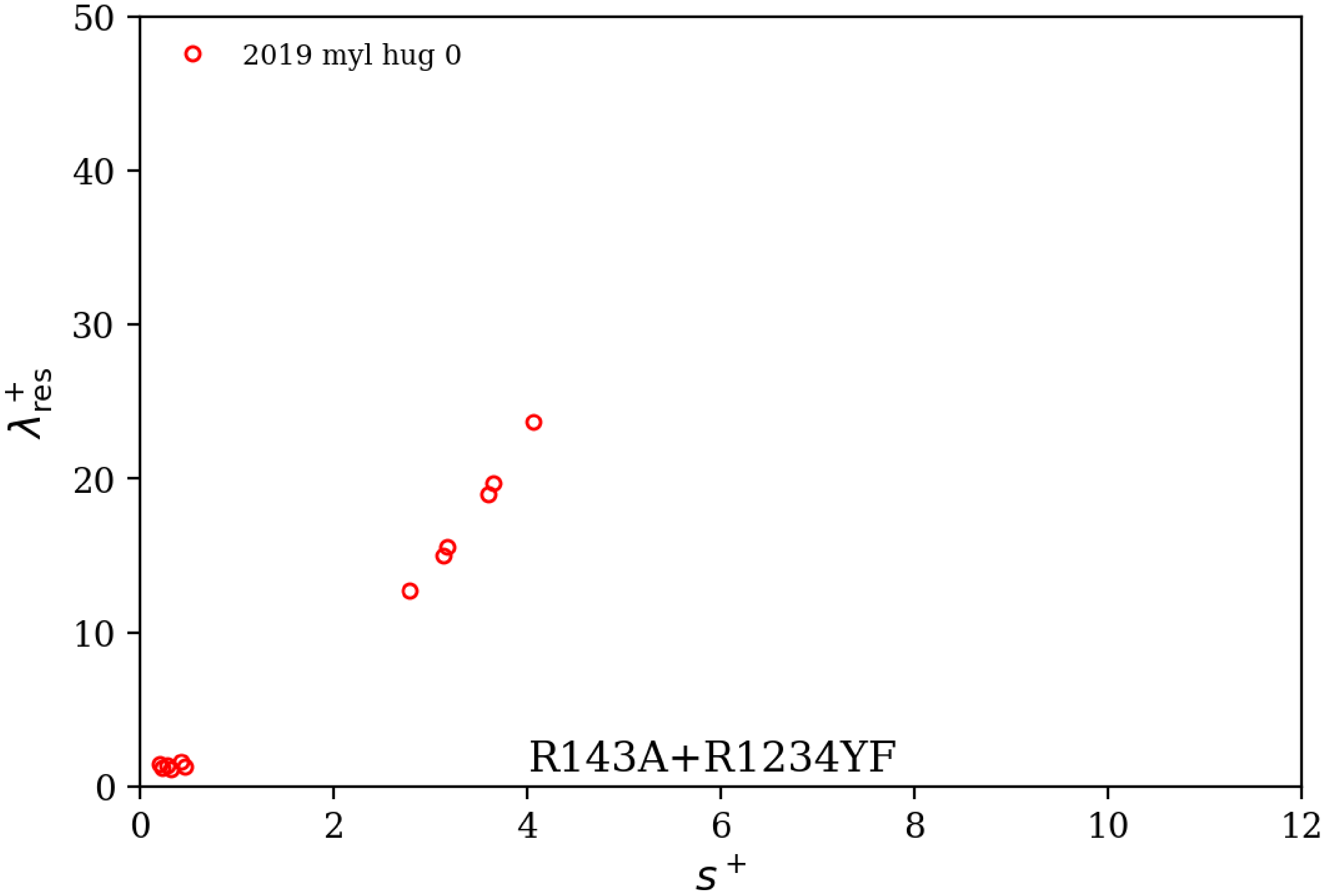

Figure DPR3. R143A+R1234YF

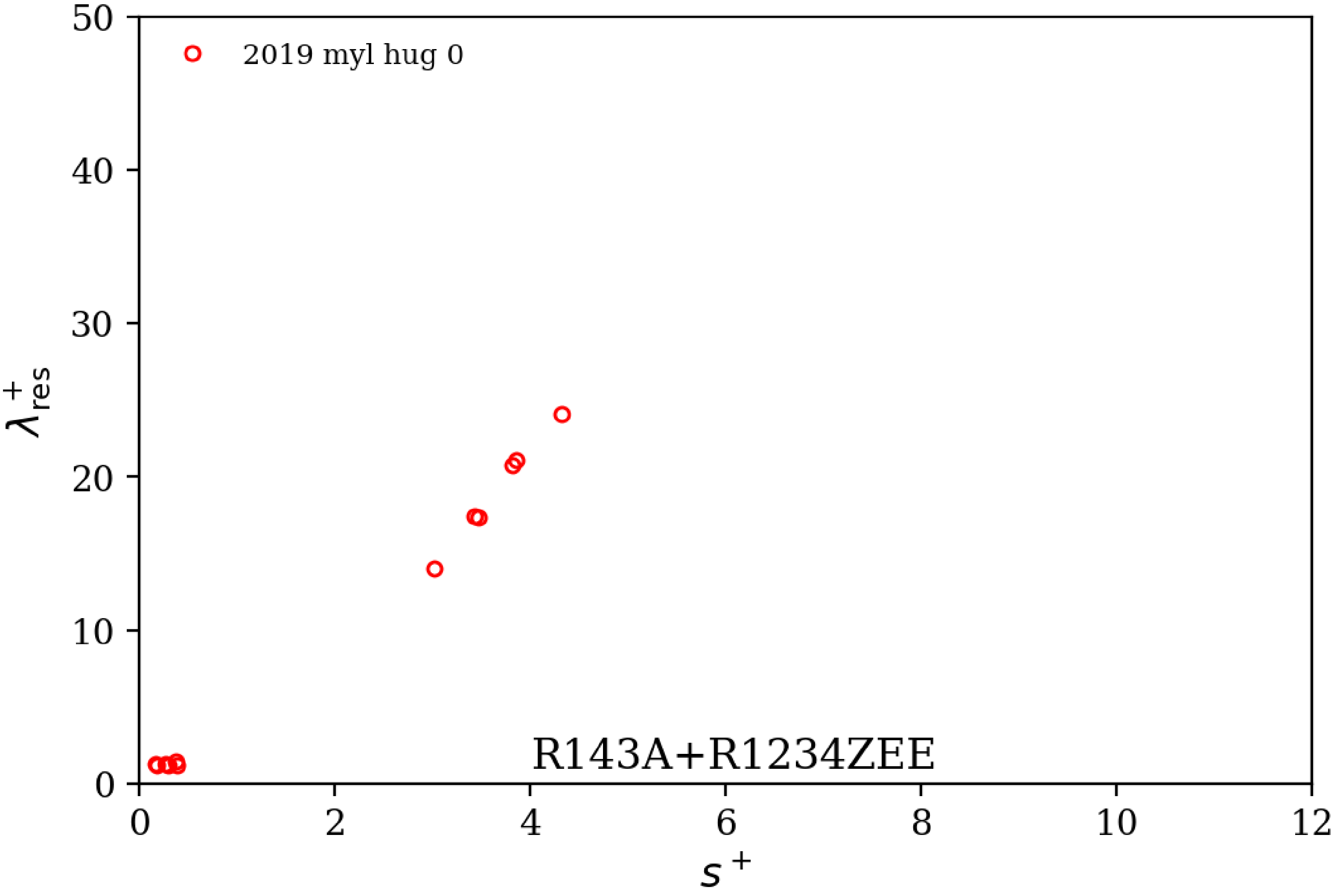

Figure DPR3.R143A+R1234ZEE

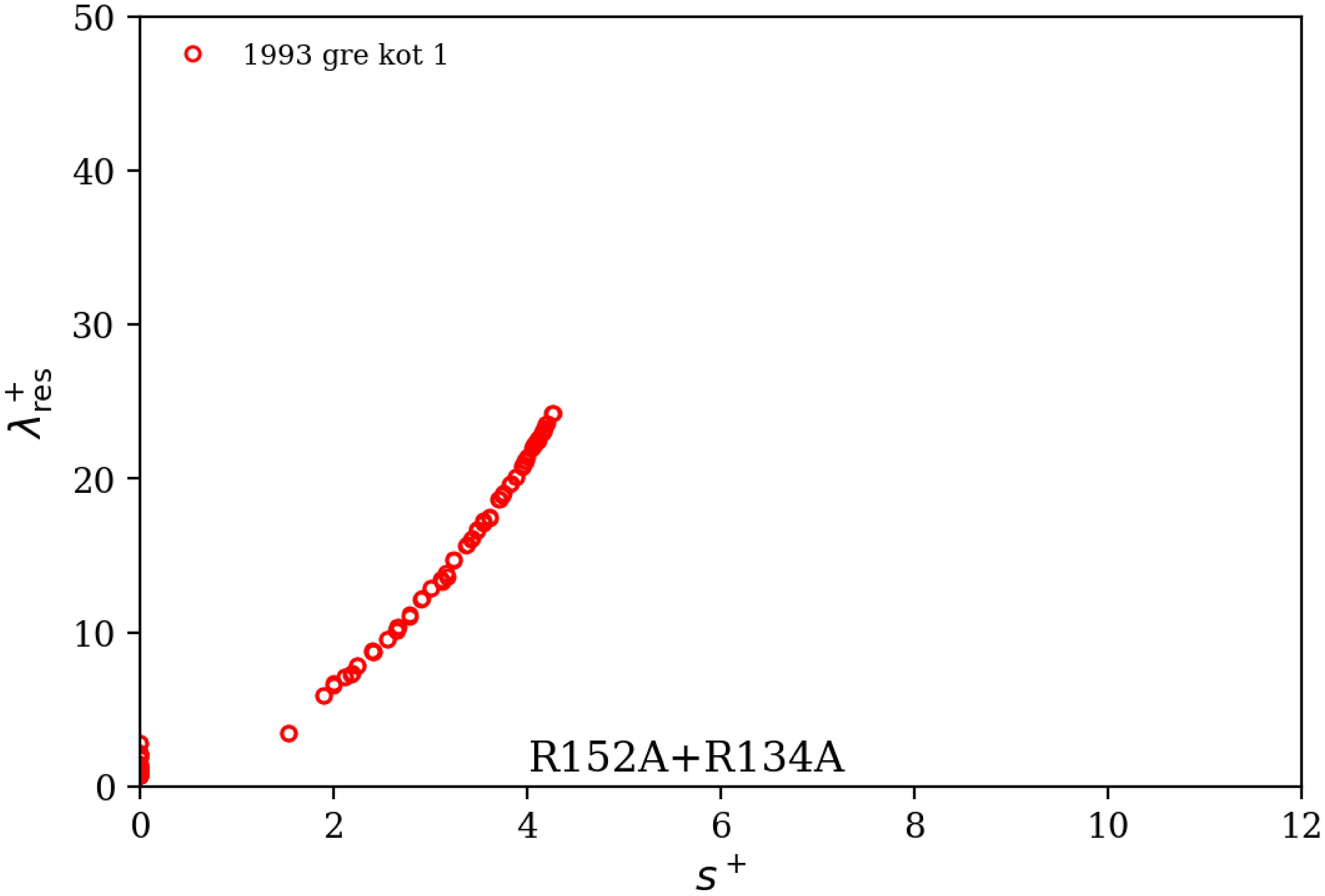

Figure DPR3. R152A+R134A

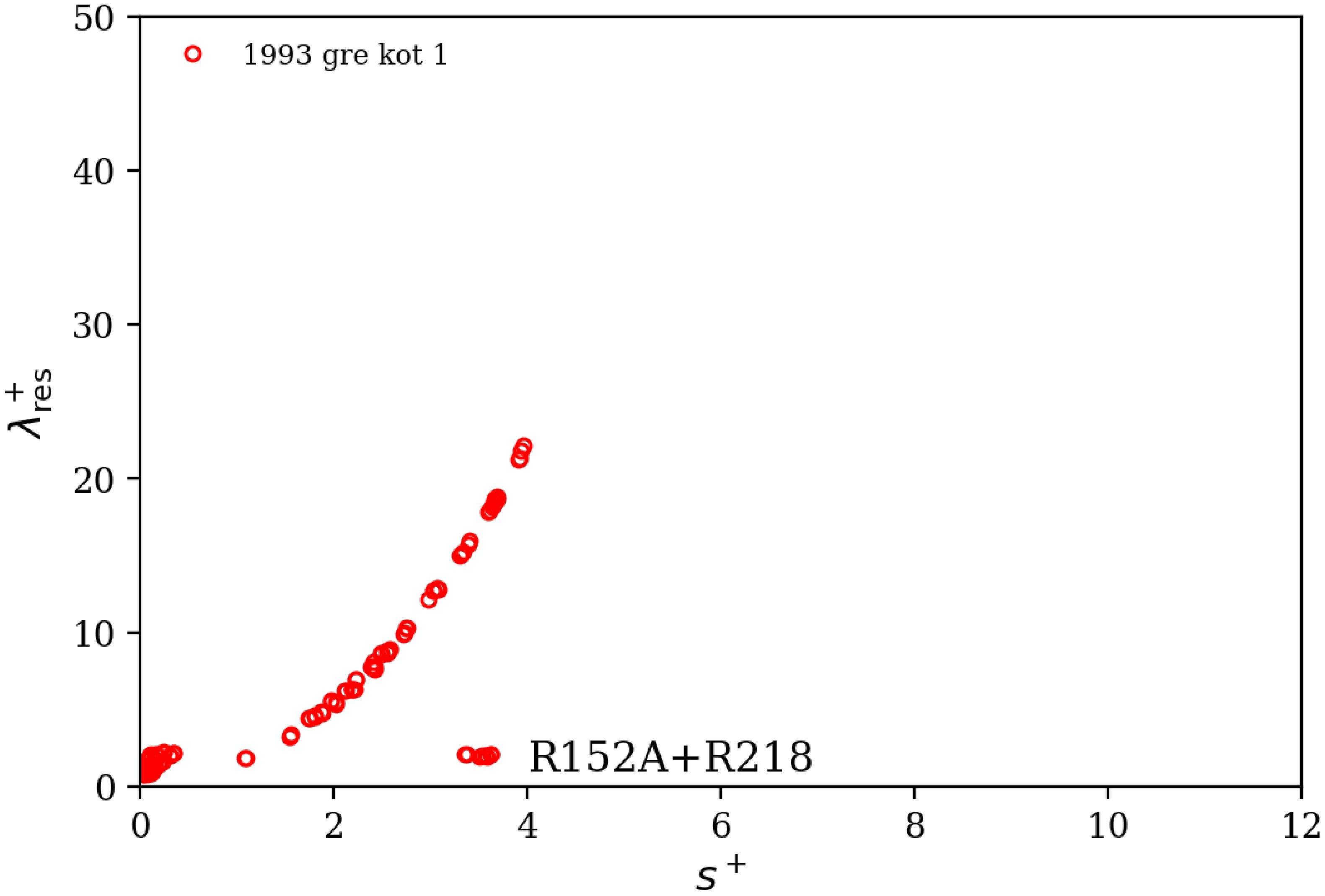

Figure DPR3. R152A+R218

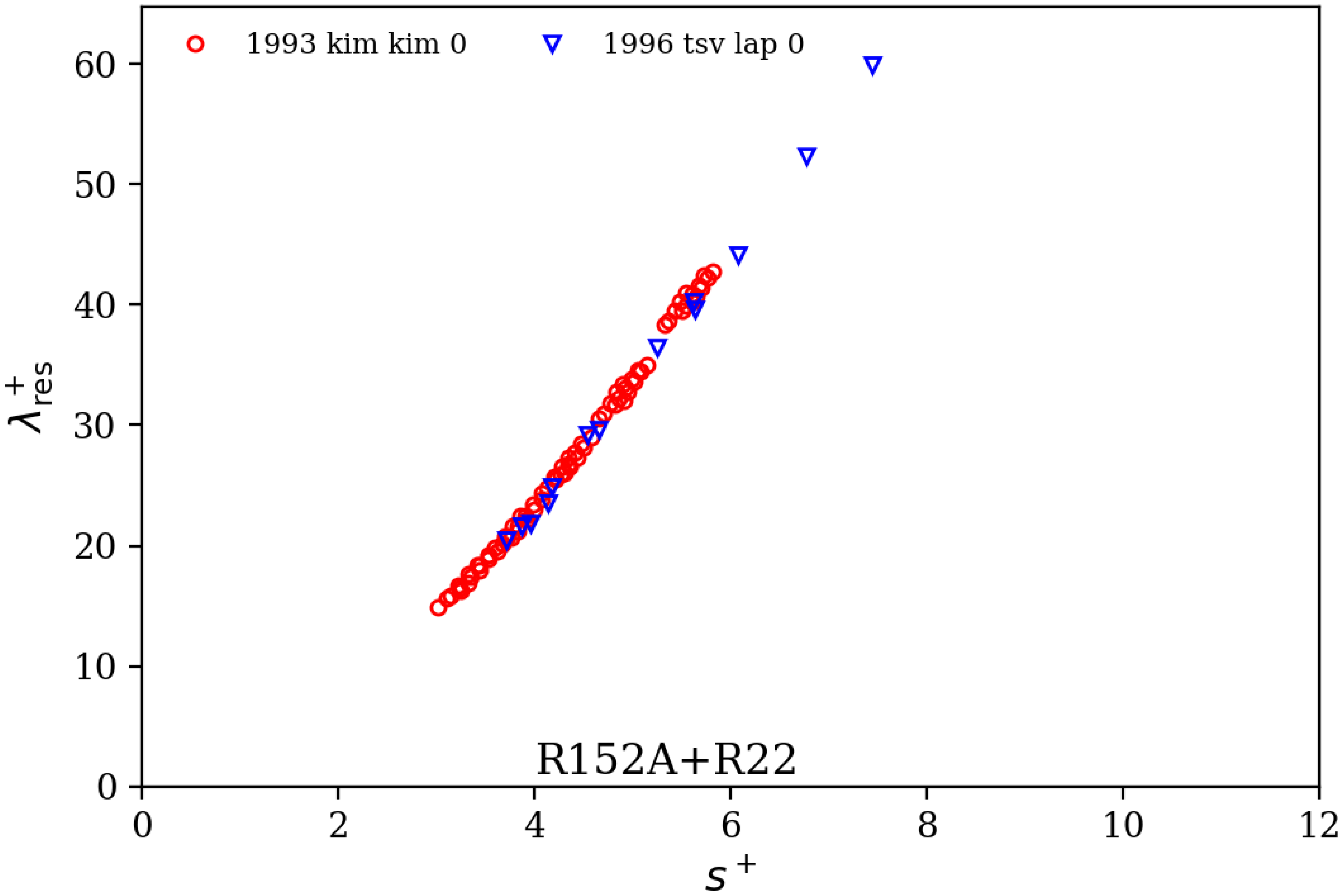

Figure DPR3. R152A+R22

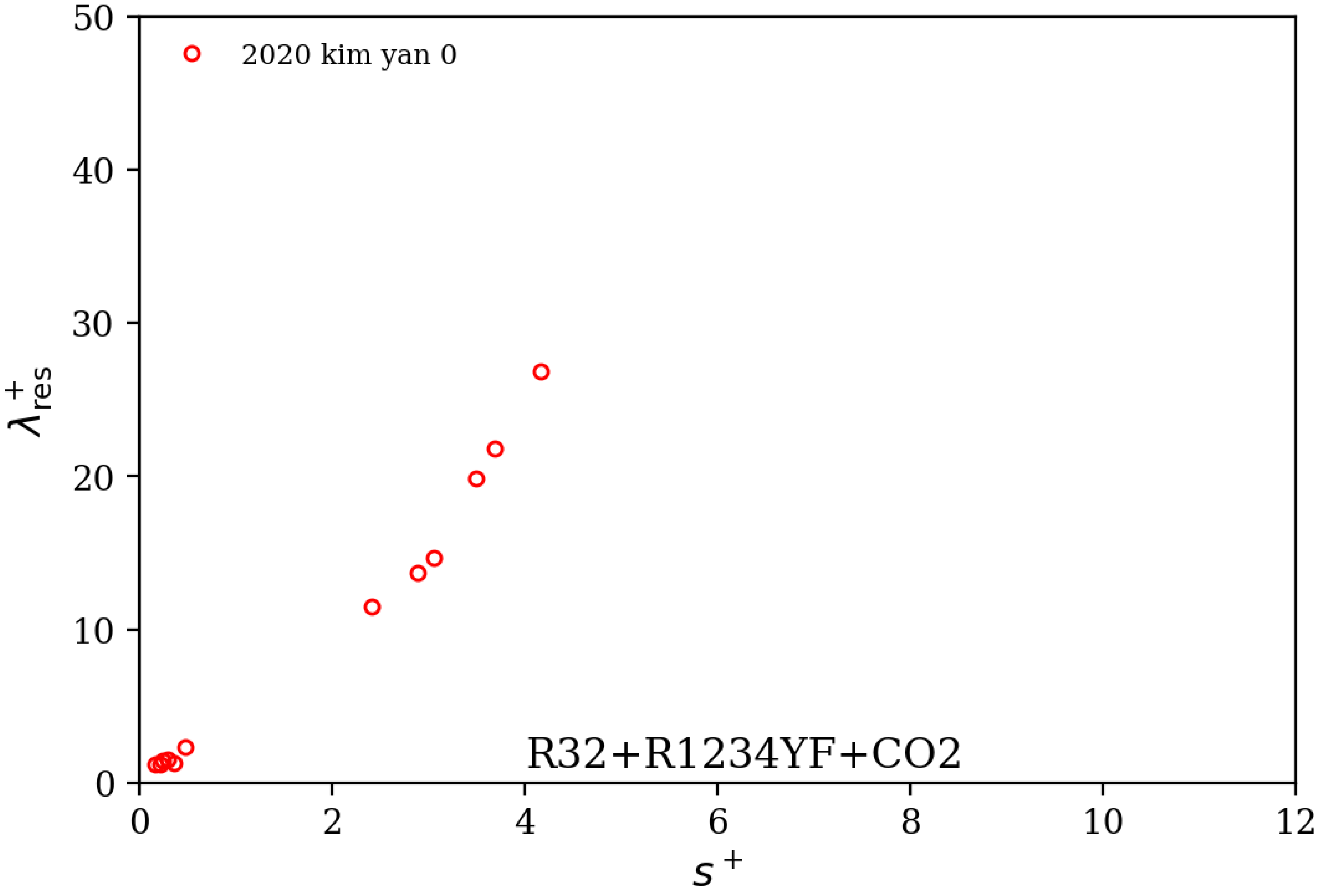

Figure DPR3. R32+R1234YF+CO2

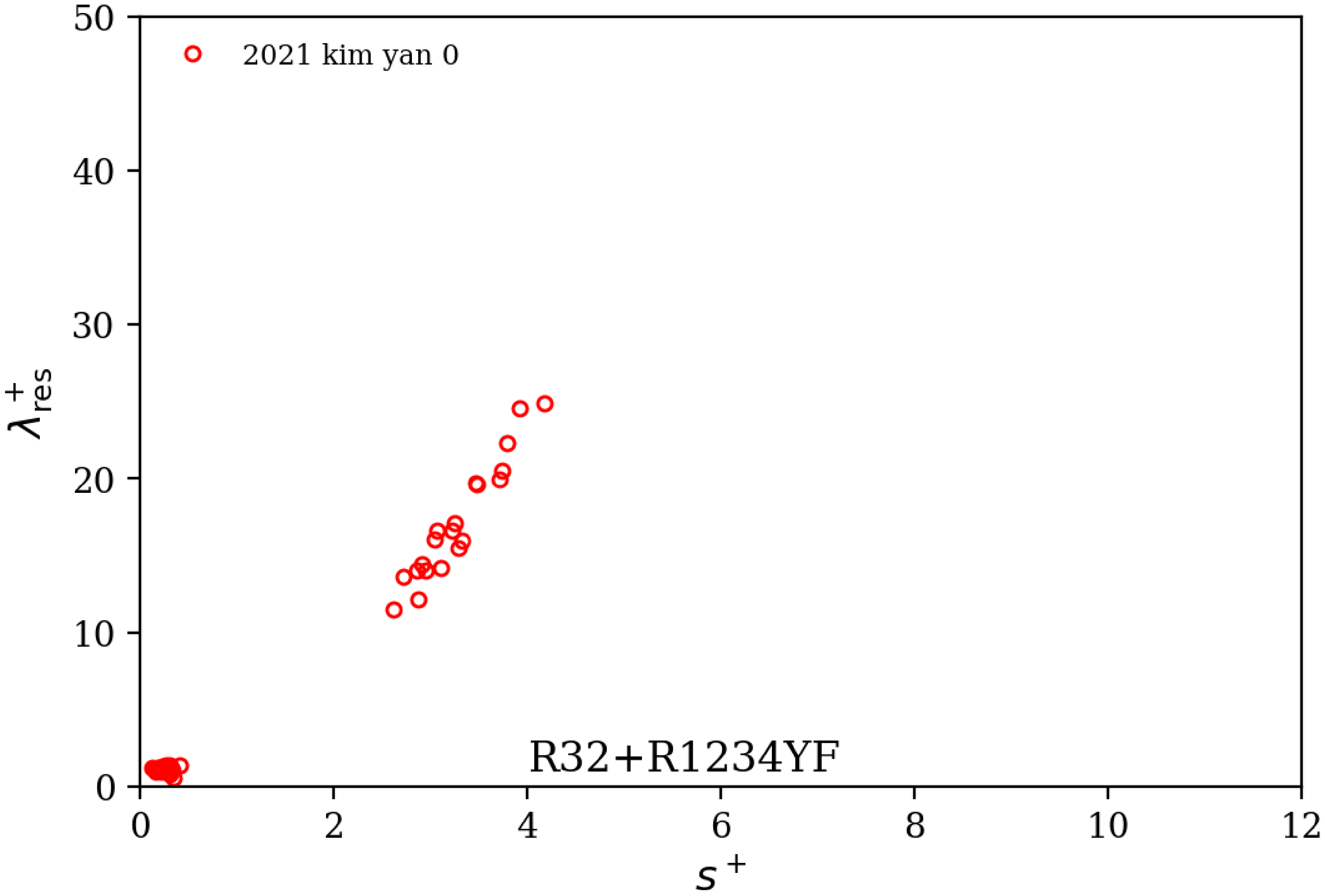

Figure DPR3. R32+R1234YF

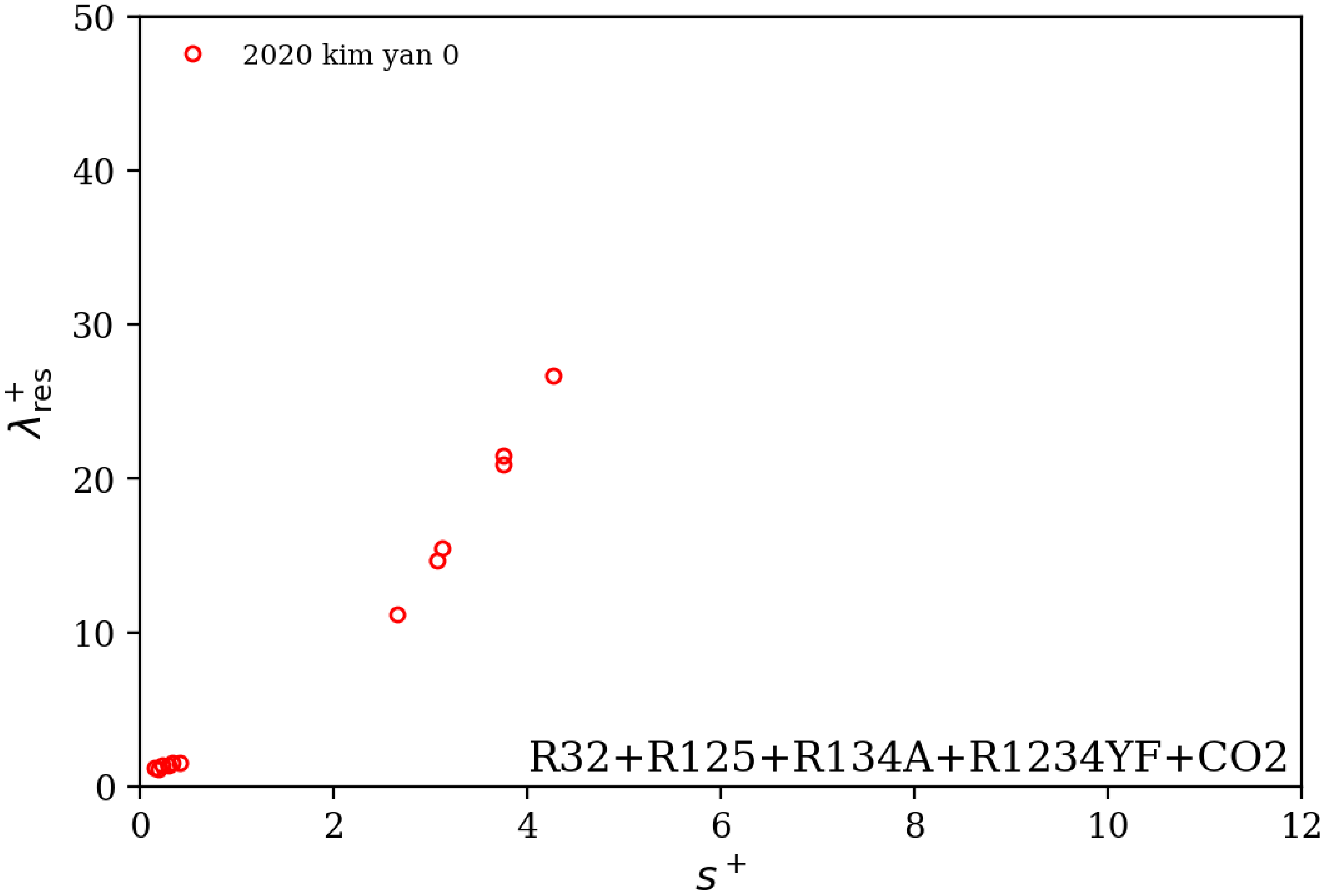

Figure DPR3. R32+R125+R134A+R1234YF+CO2

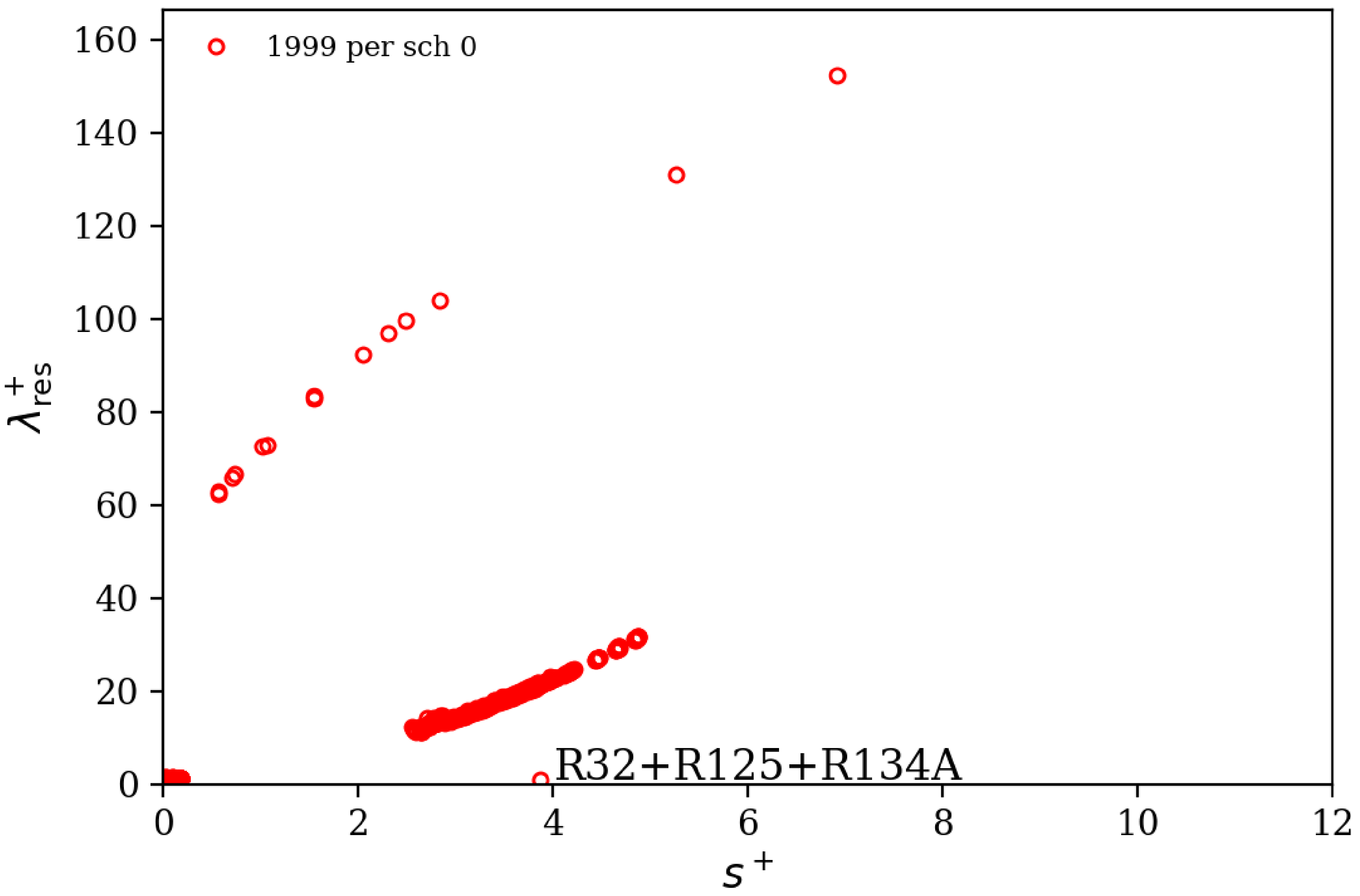

Figure DPR3. R32+R125+R134A

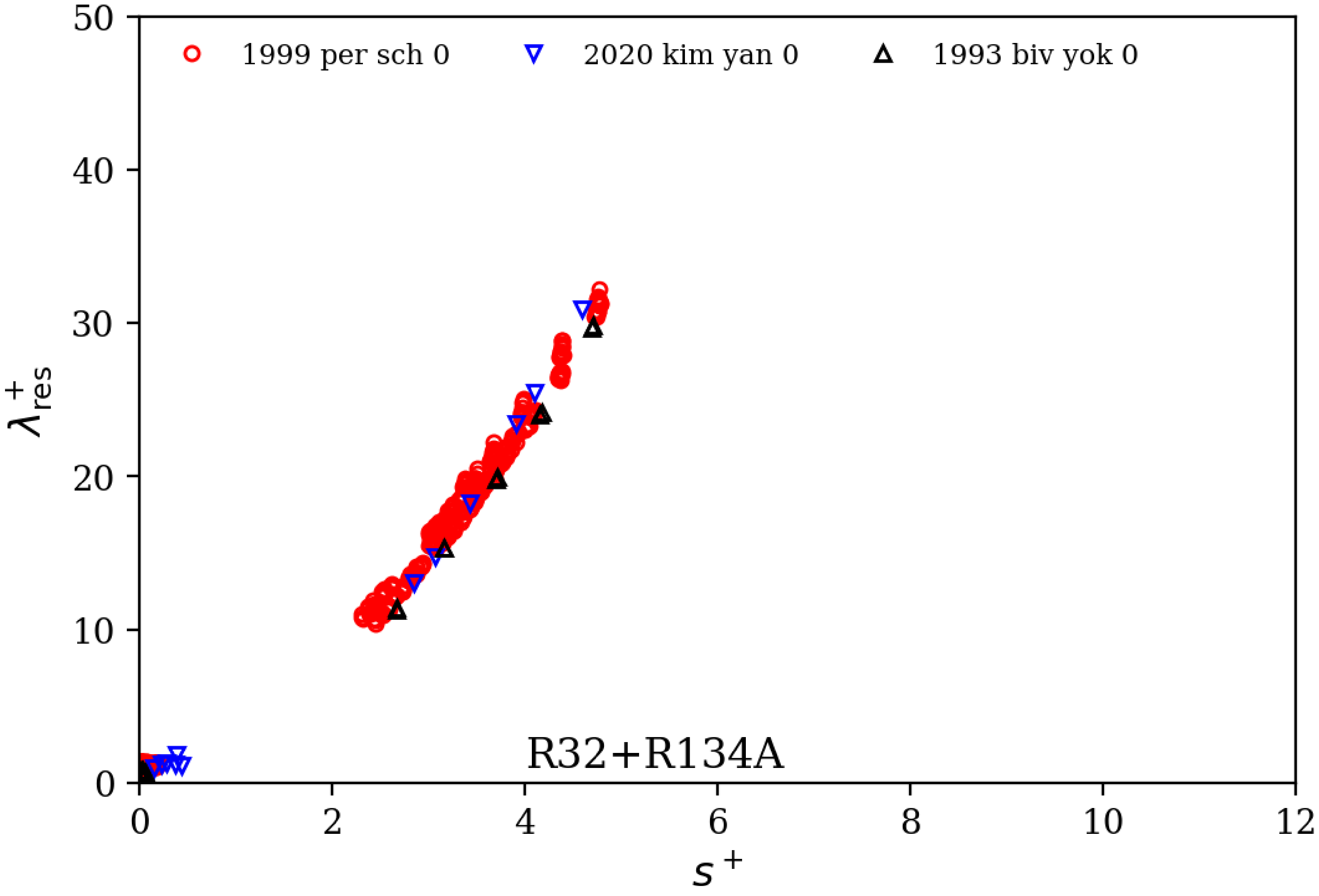

Figure DPR3. R32+R134A

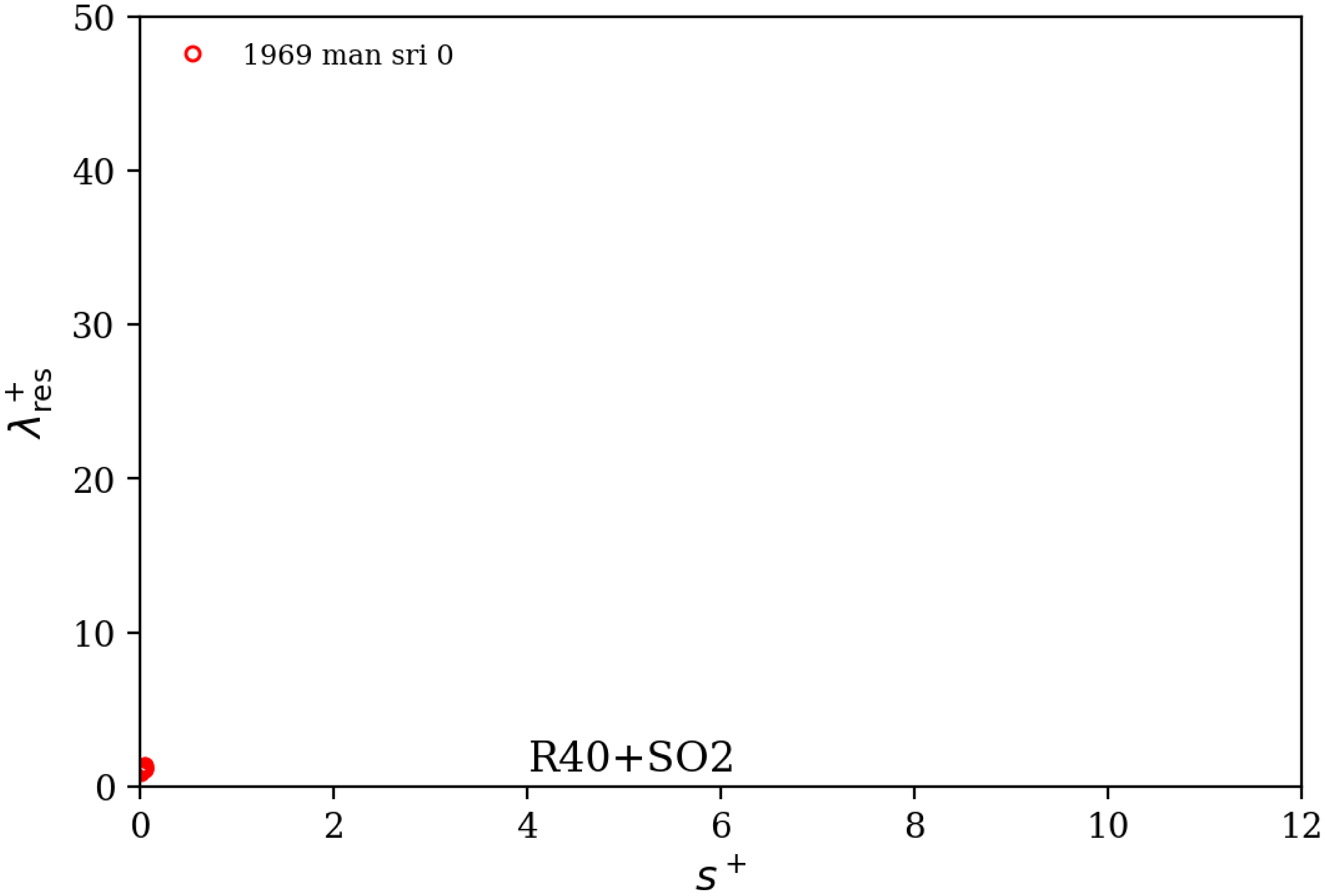

Figure DPR3. R40+SO2

4. Relative deviation from experimental values of each mixture to models

In this section, relative deviation of the experimental thermal conductivity λ_{exp} from values λ_{RES} calculated with the RES model and the best-selected models in REFPORP 10.0are presented for each mixture. The legends denote the sources of the data, showing the published year, the first three letters of the first author's family name, and the first three letters of the second author's family name ('xxx' if there is no second author). The full citations are given at the Reference section.

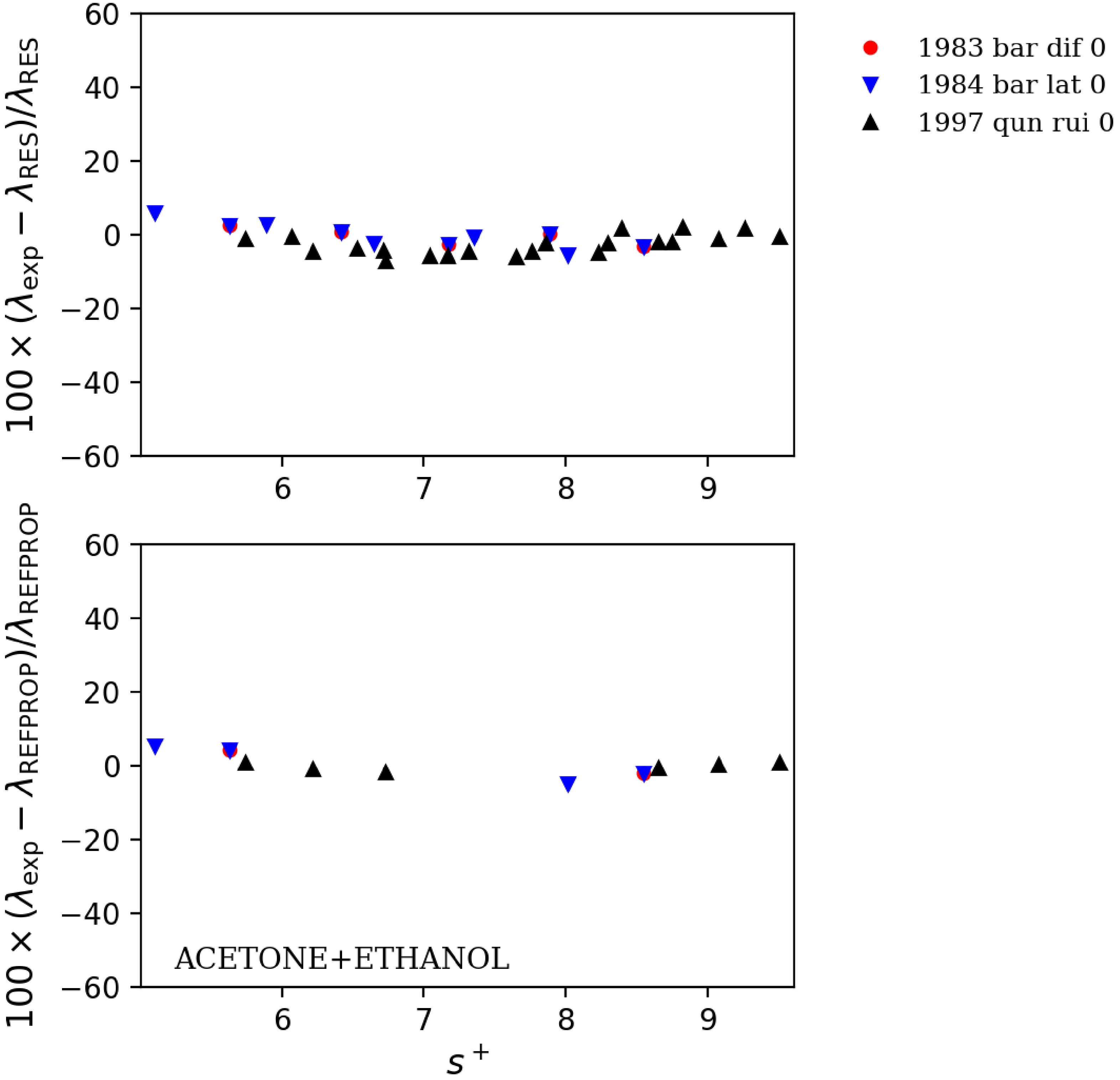

Figure DPR4. ACETONE+ETHANOL

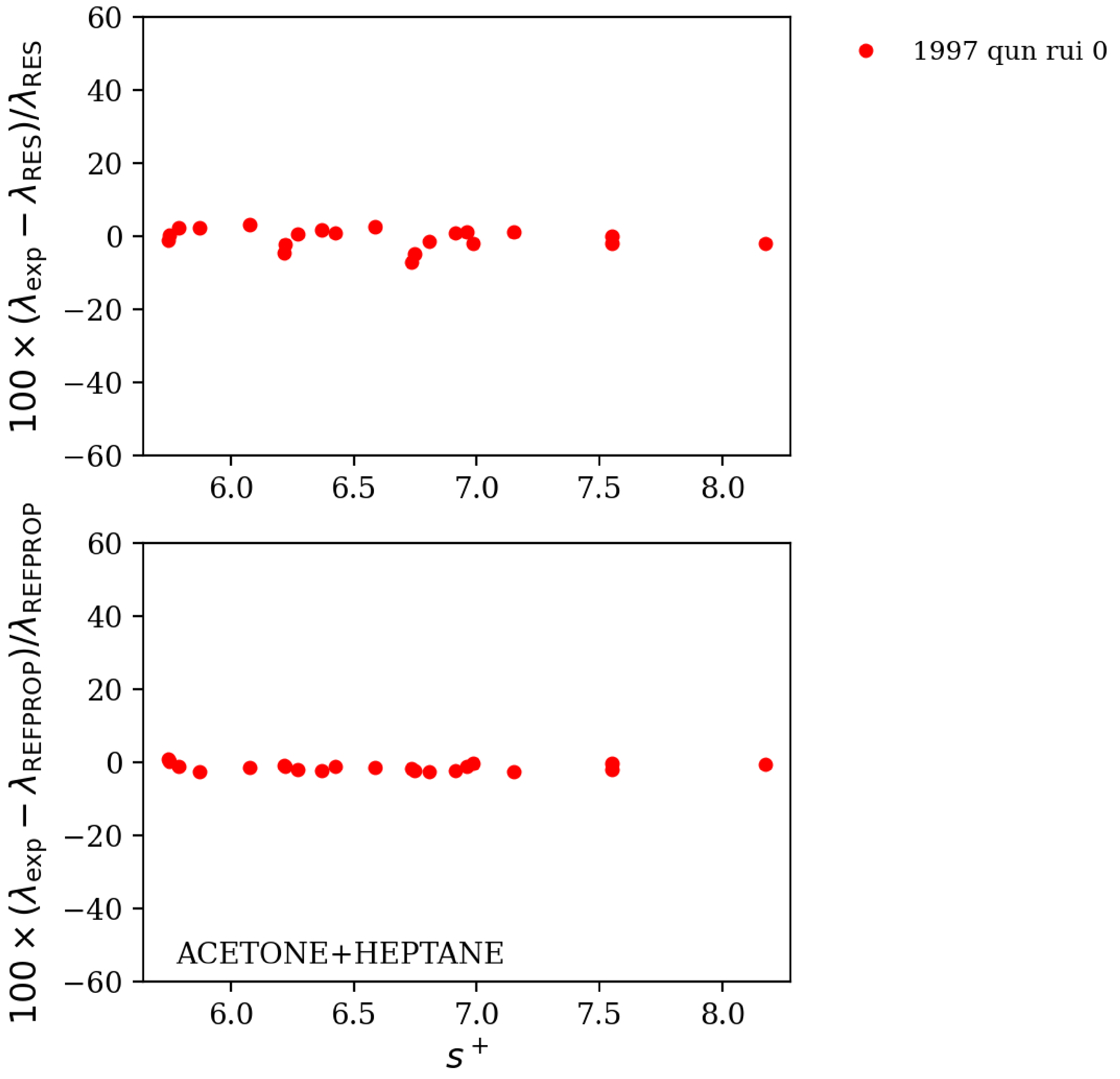

Figure DPR4. ACETONE+HEPTANE

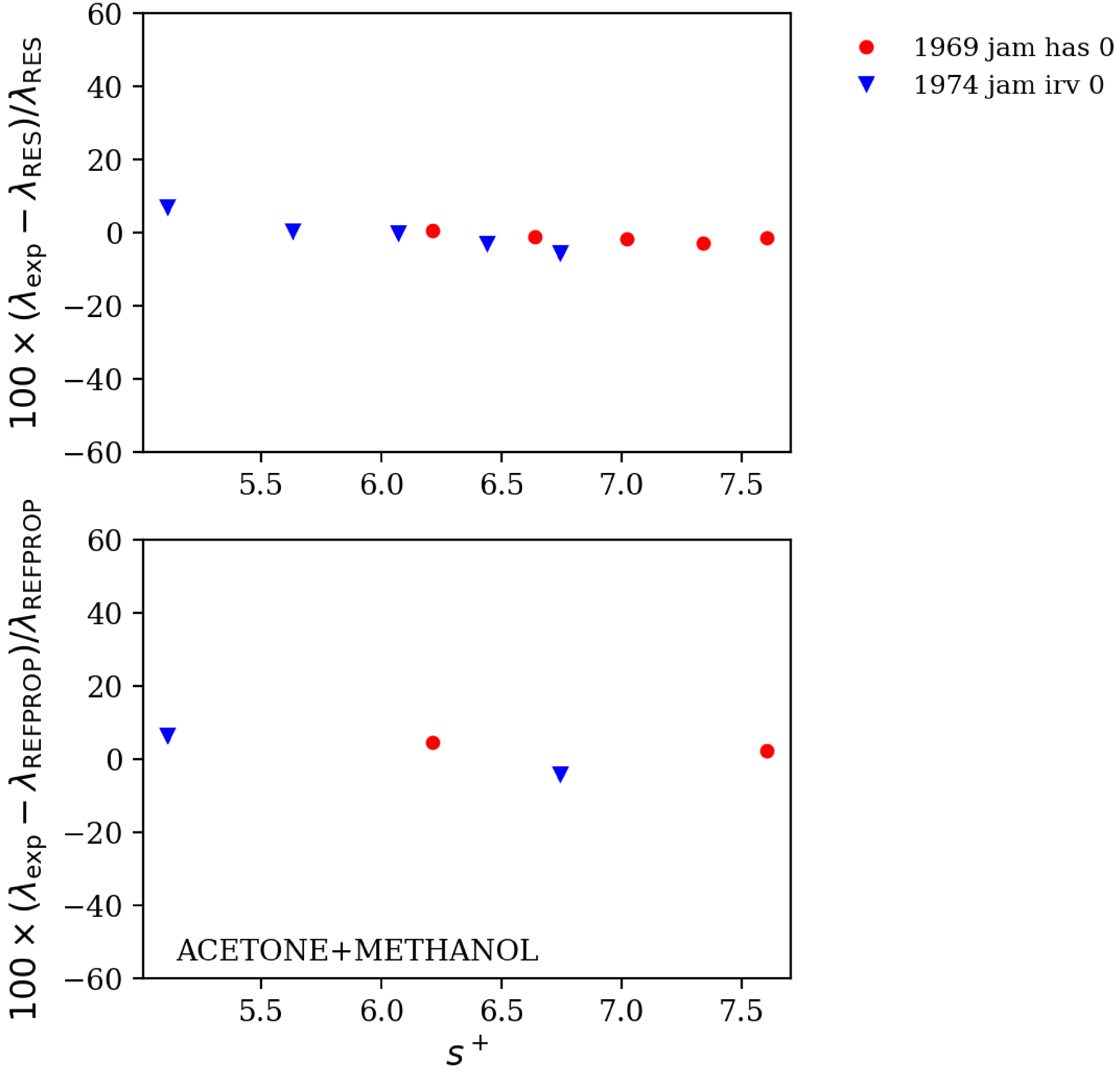

Figure DPR4. ACETONE+METHANOL

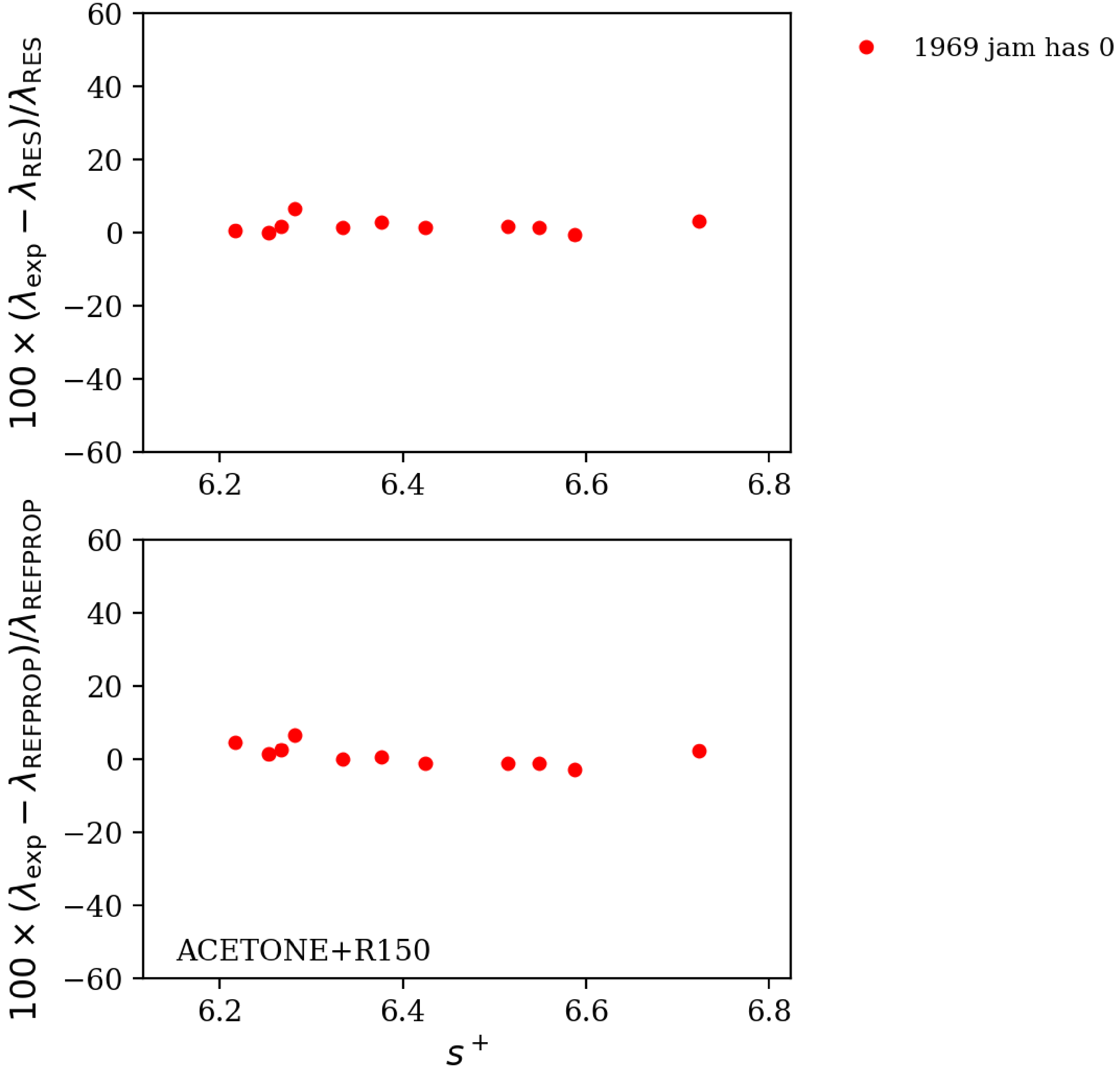

Figure DPR4. ACETONE+R150

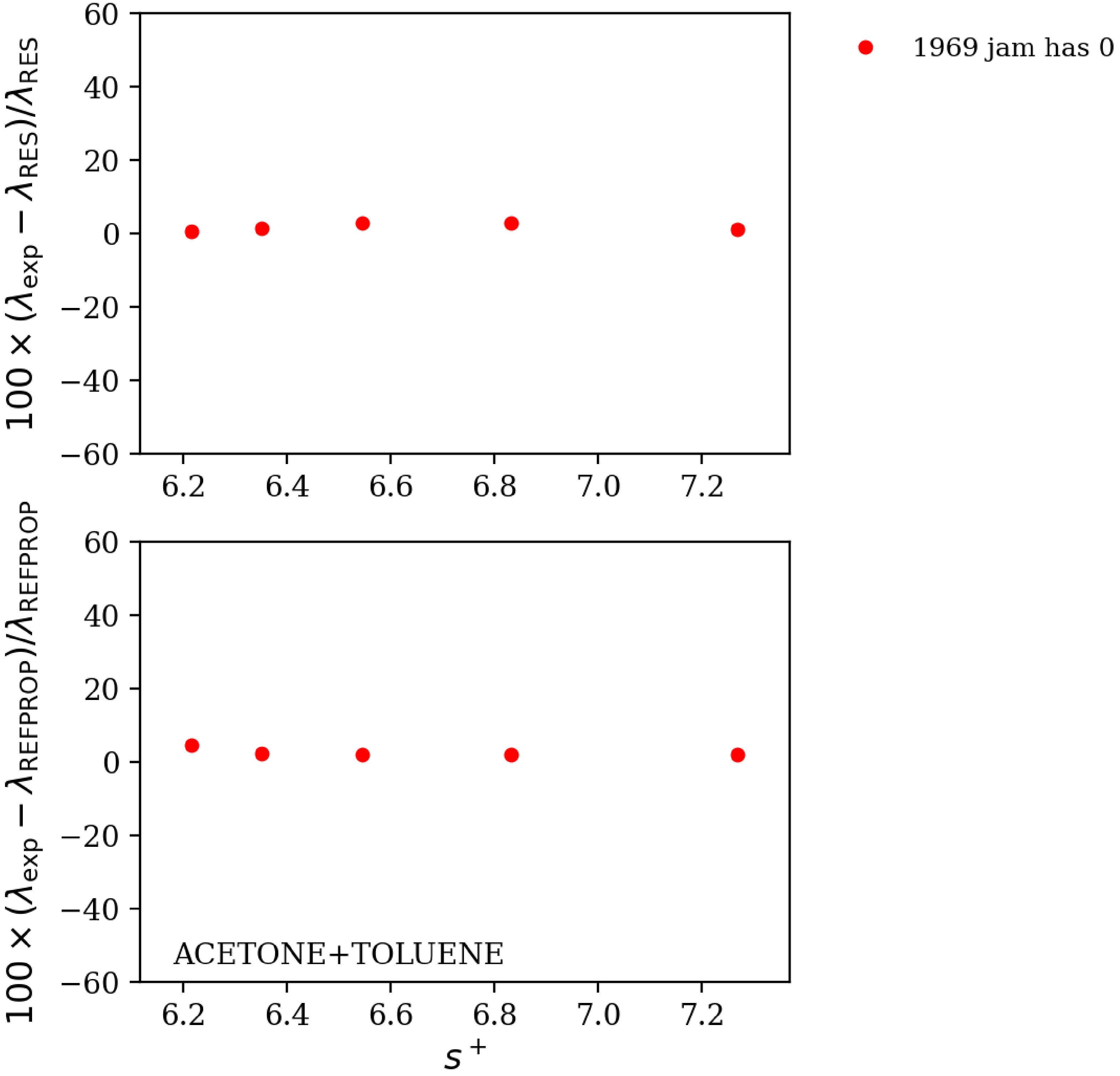

Figure DPR4. ACETONE+TOLUENE

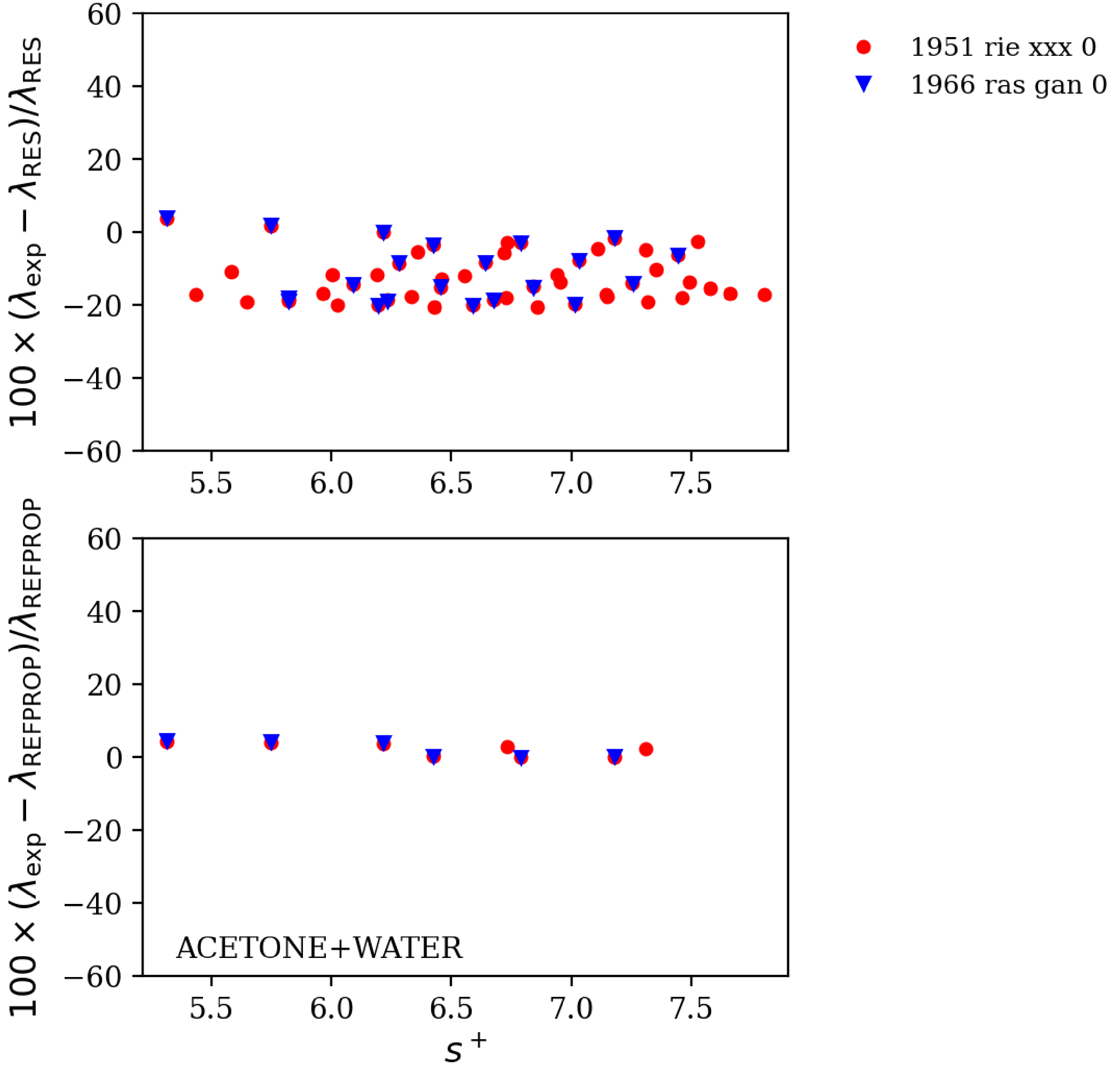

Figure DPR4. ACETONE+WATER

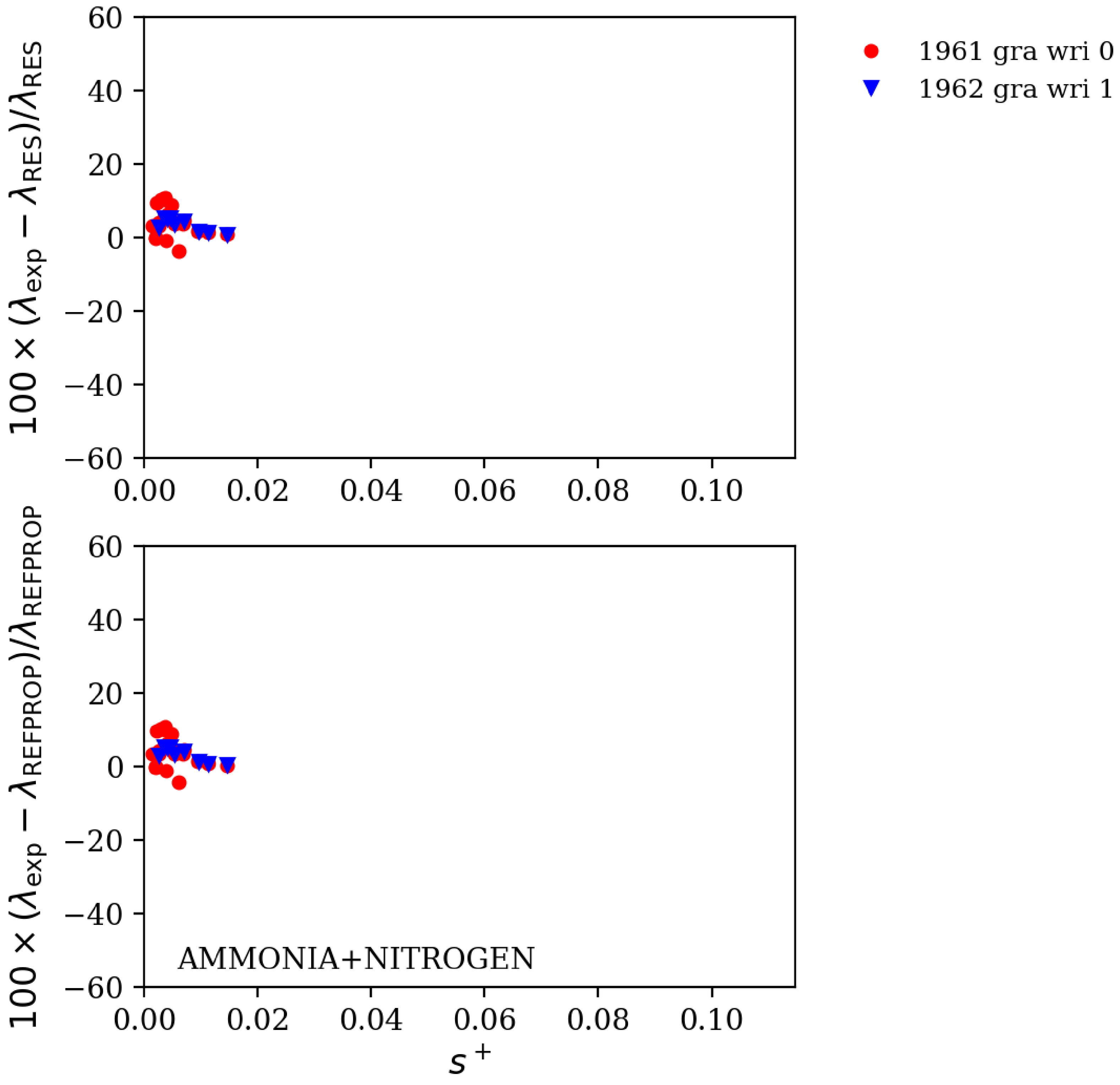

Figure DPR4. AMMONIA+NITROGEN

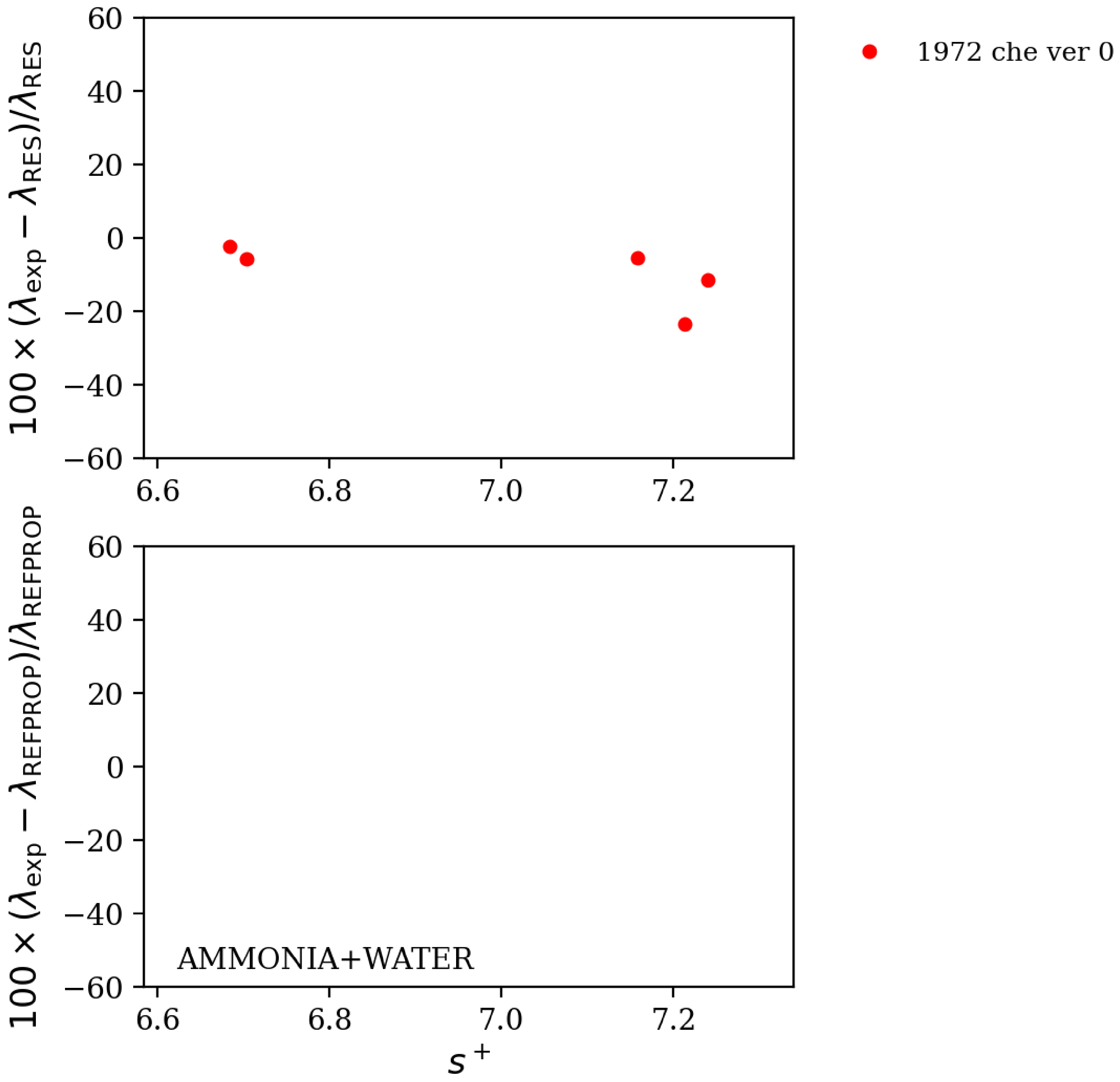

Figure DPR4. AMMONIA+WATER

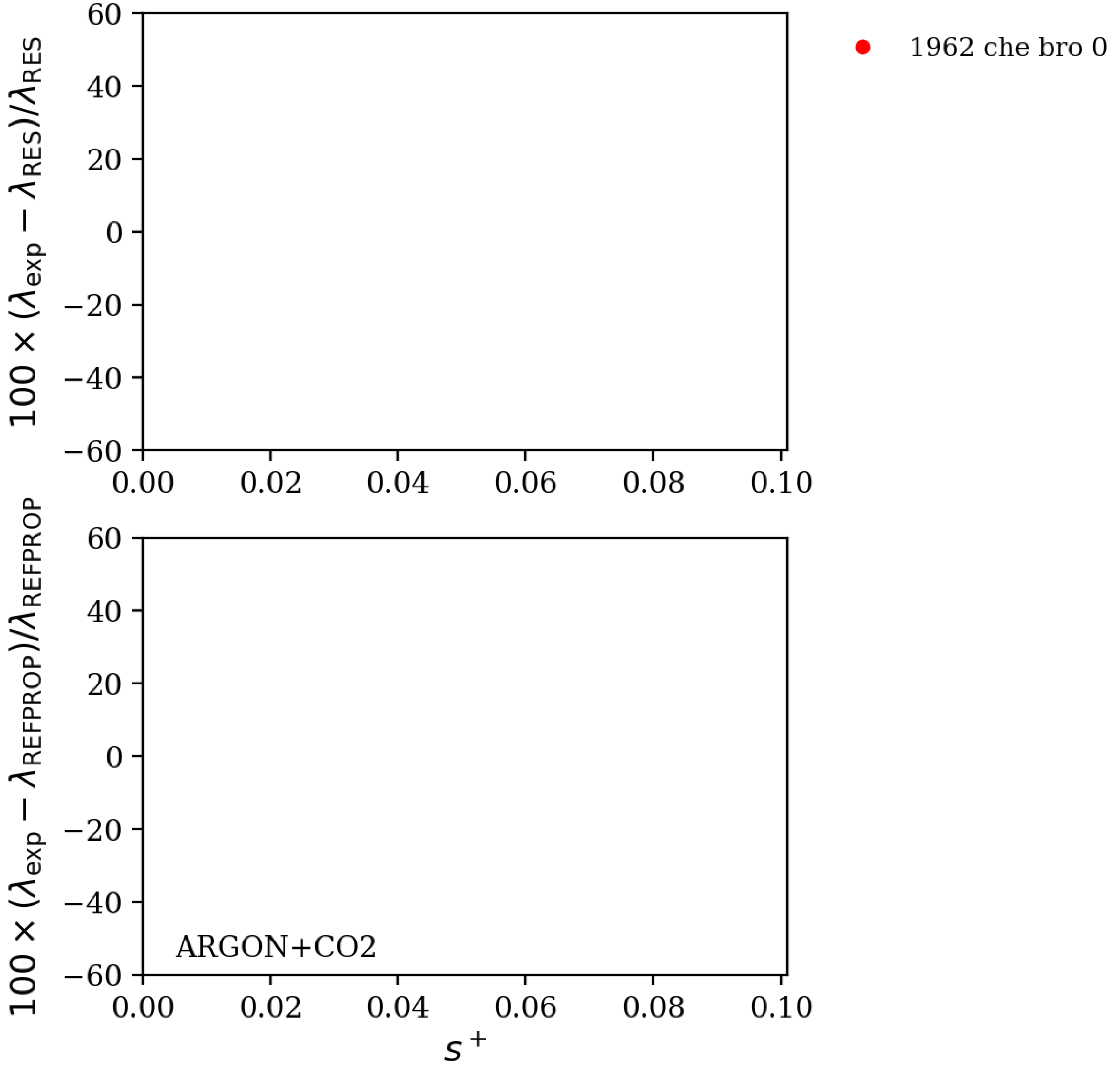

Figure DPR4. ARGON+CO2

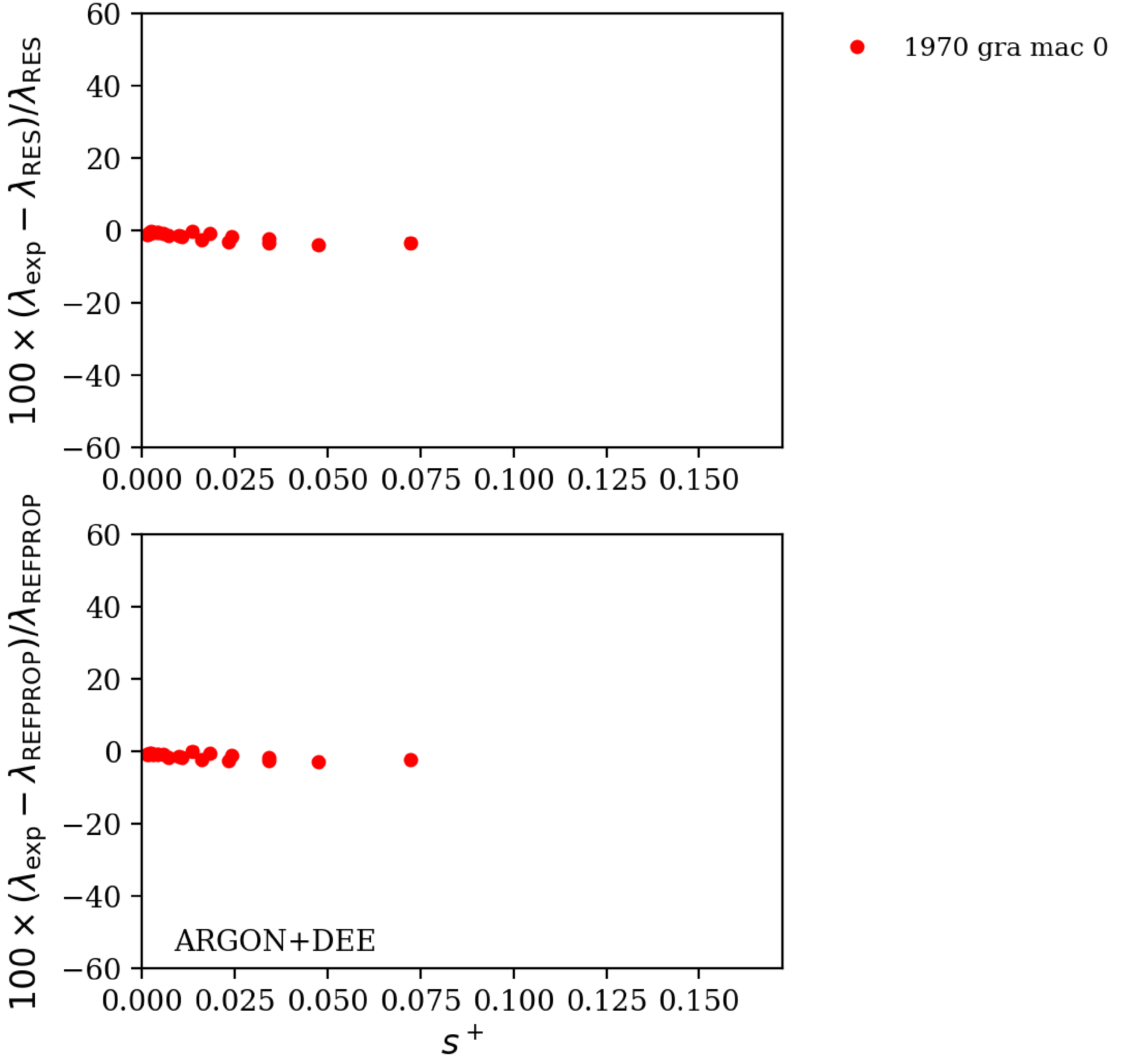

Figure DPR4. ARGON+DEE

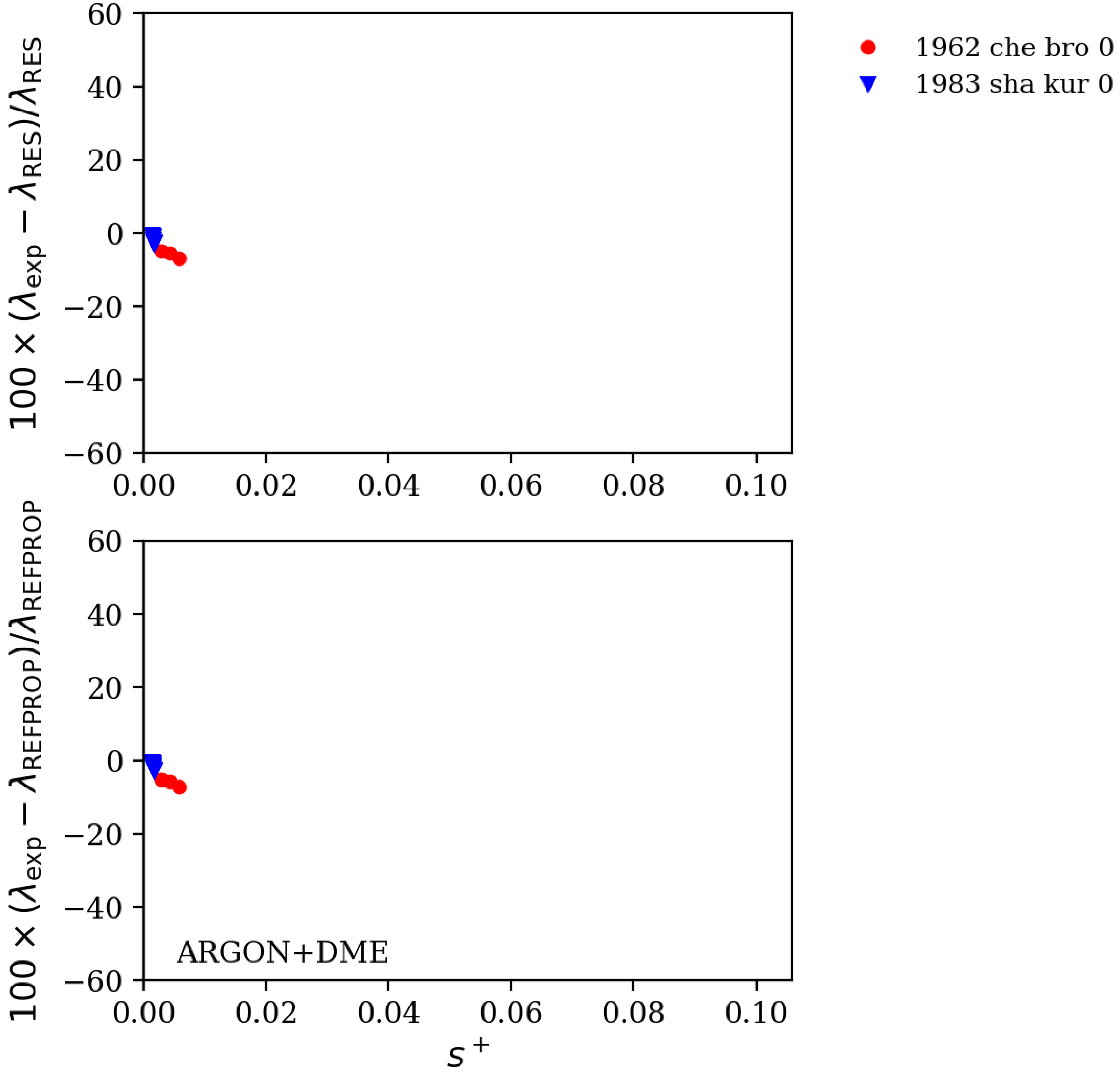

Figure DPR4. ARGON+DME

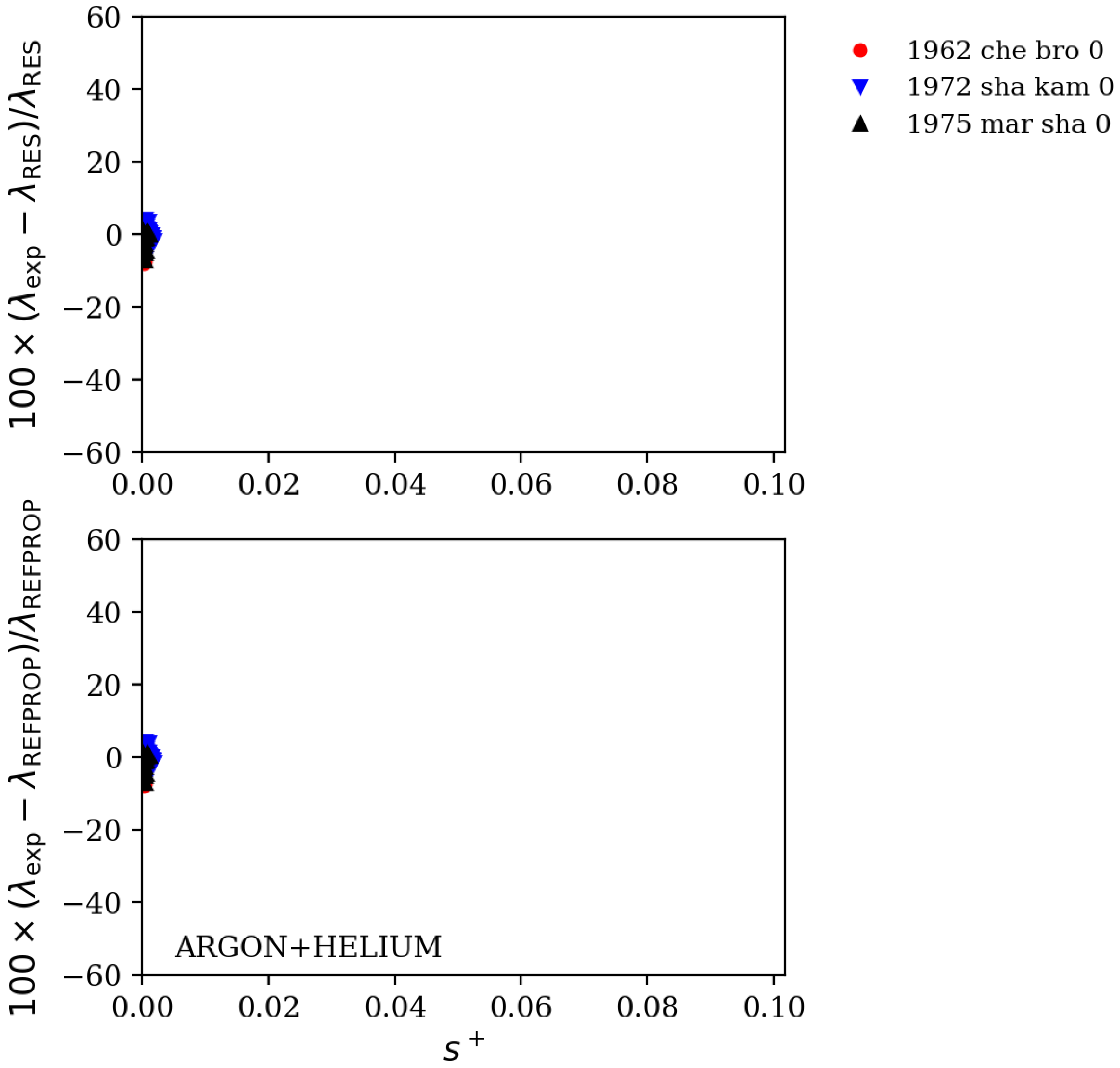

Figure DPR4. ARGON+HELIUM

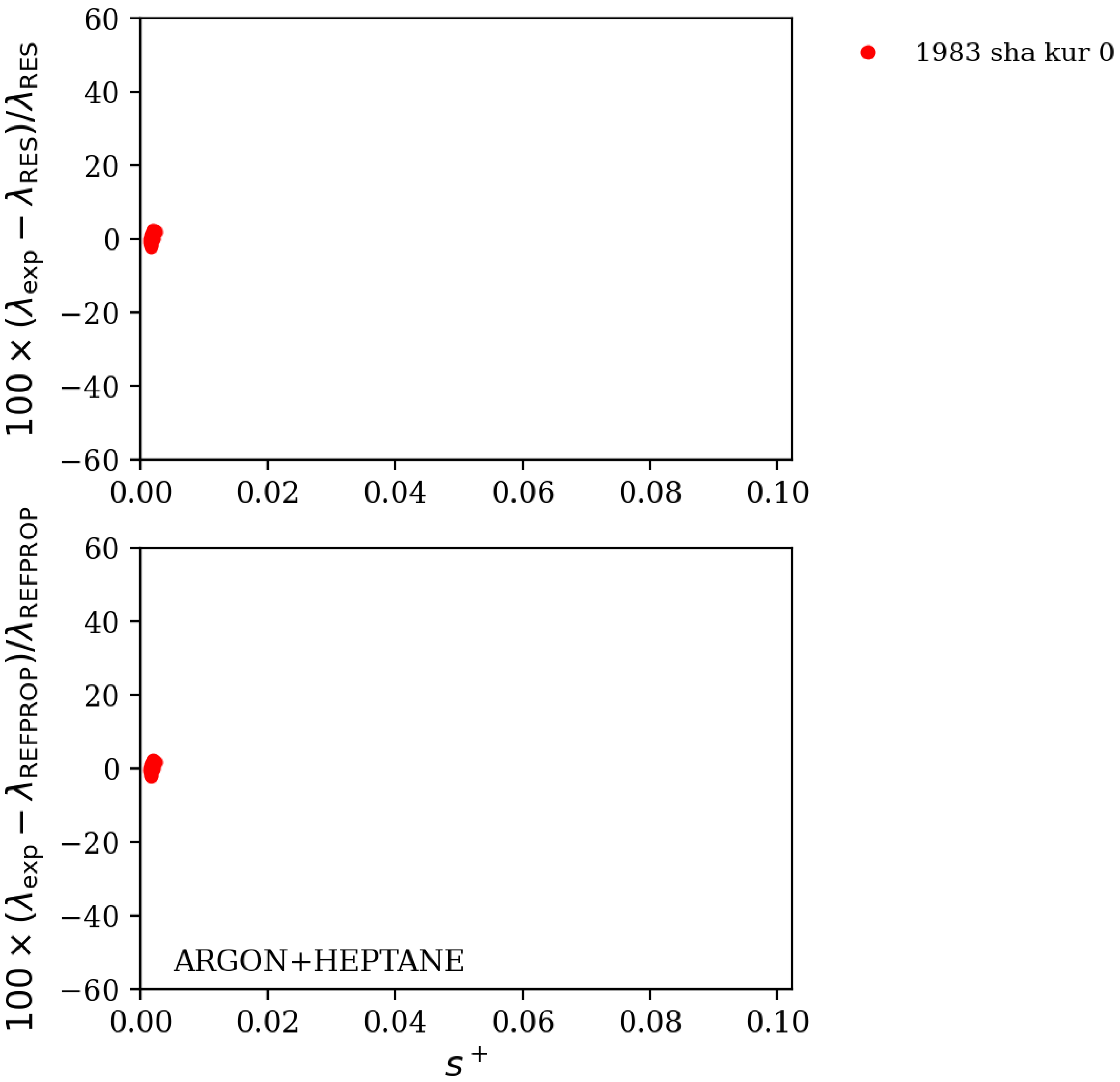

Figure DPR4. ARGON+HEPTANE

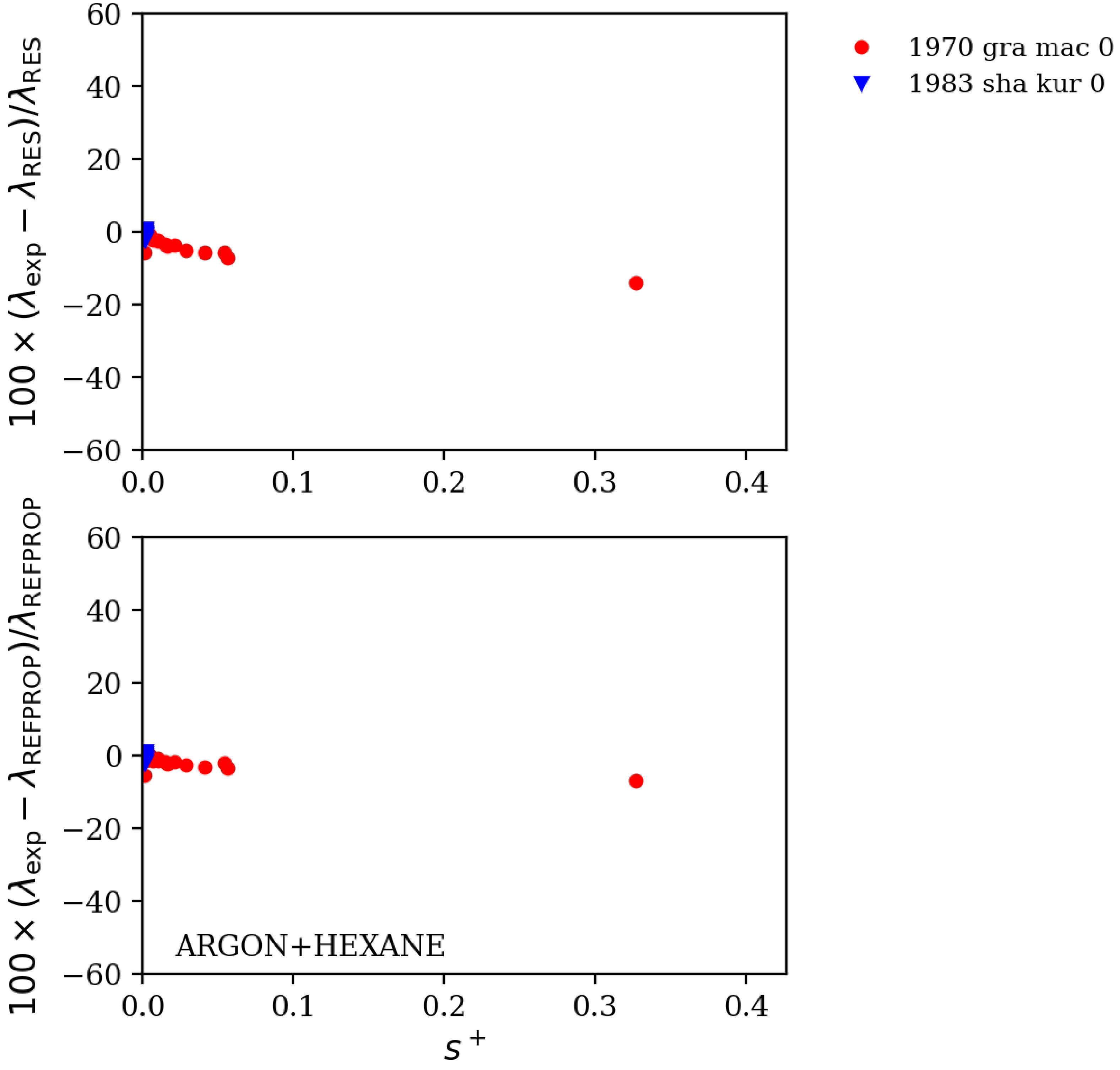

Figure DPR4. ARGON+HEXANE

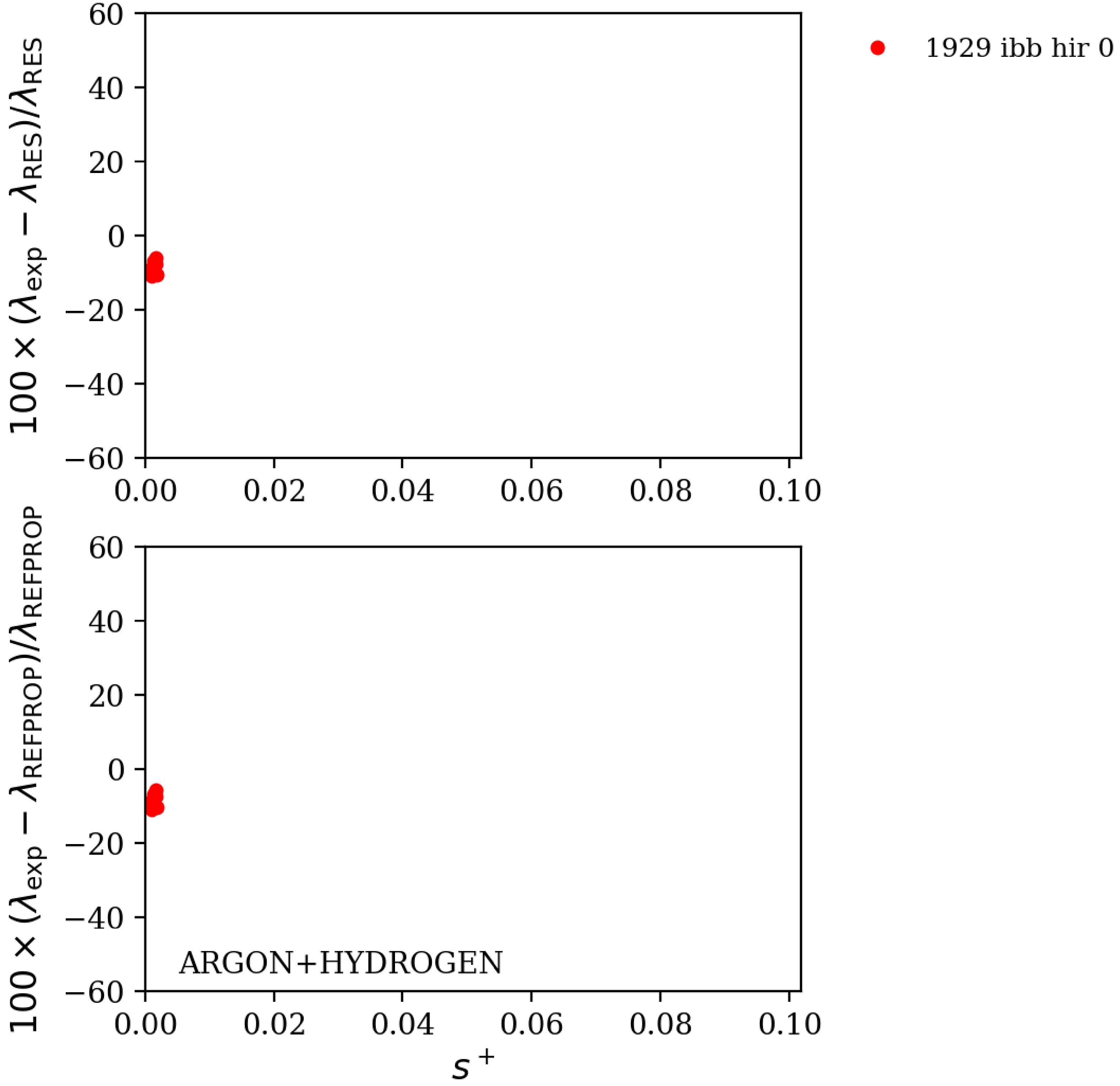

Figure DPR4. ARGON+HYDROGEN

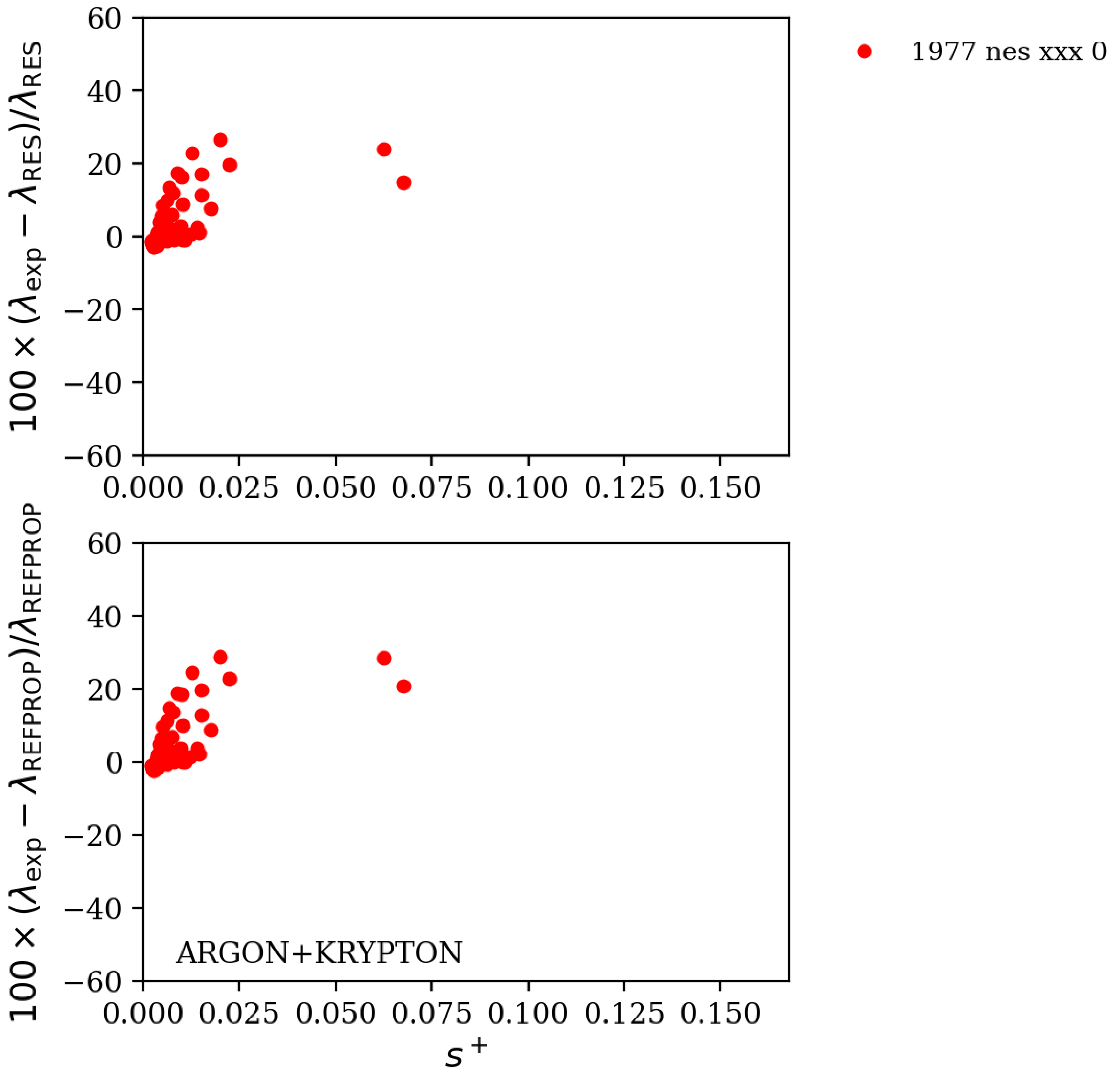

Figure DPR4. ARGON+KRYPTON

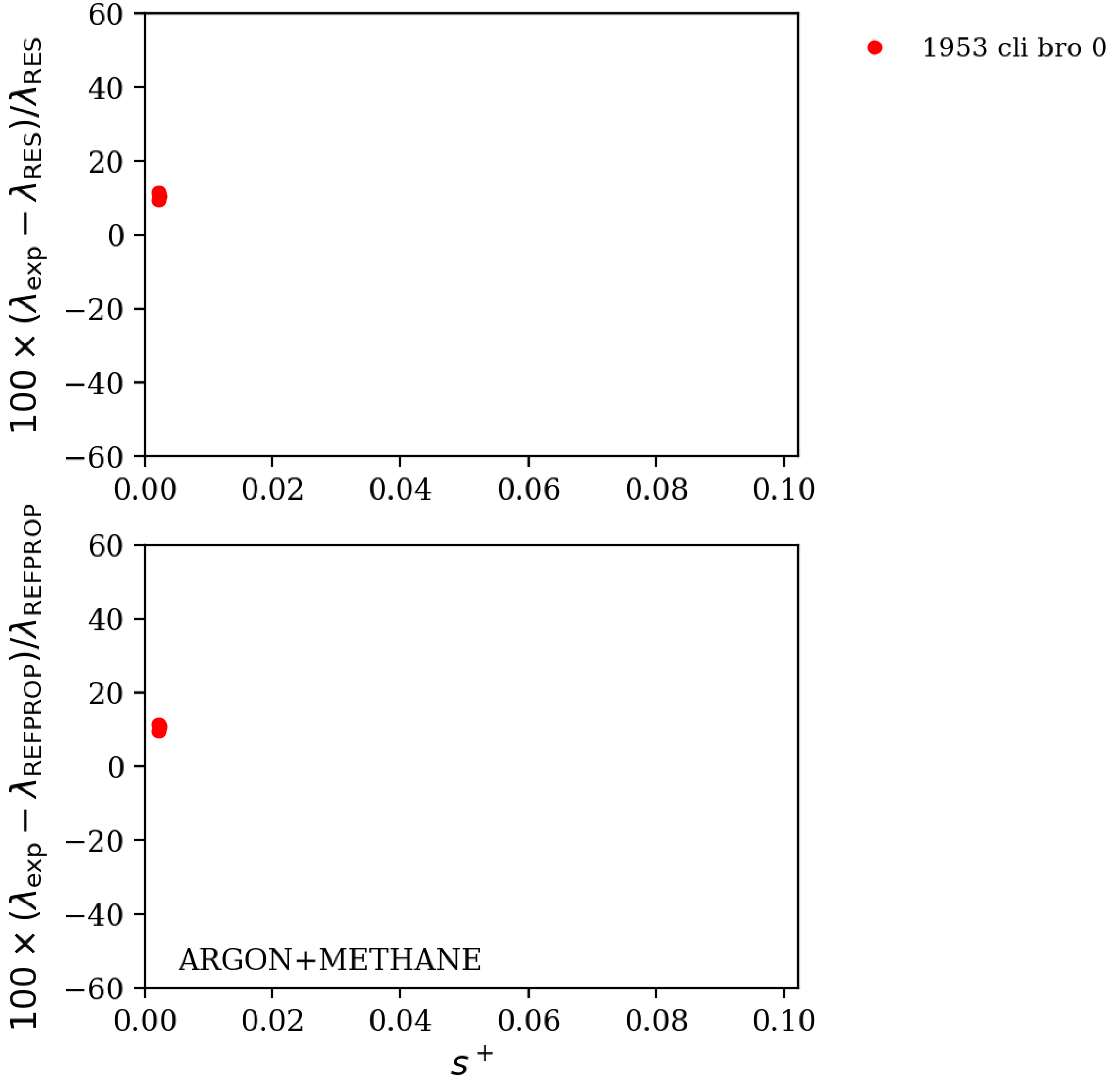

Figure DPR4. ARGON+METHANE

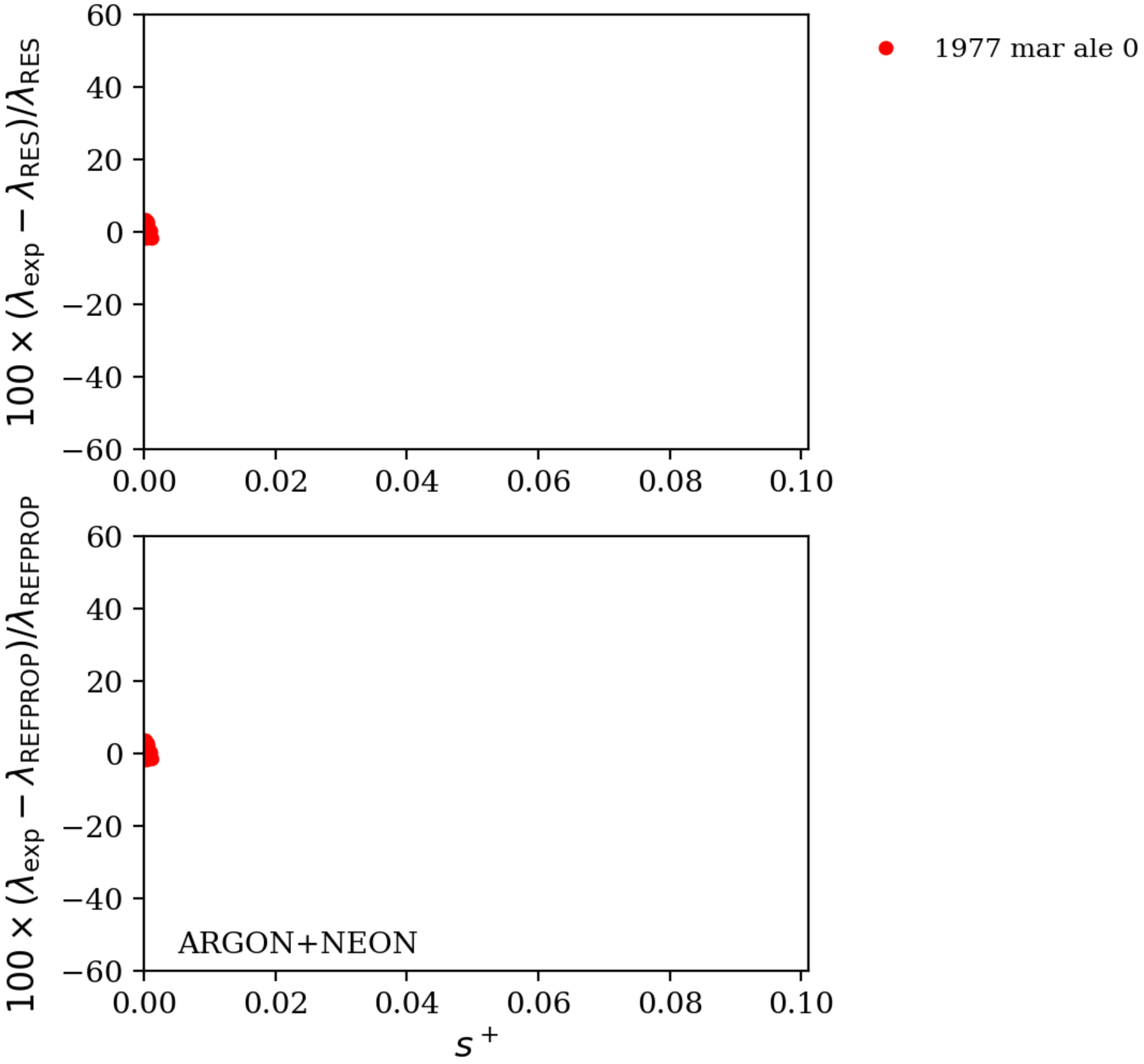

Figure DPR4. ARGON+NEON

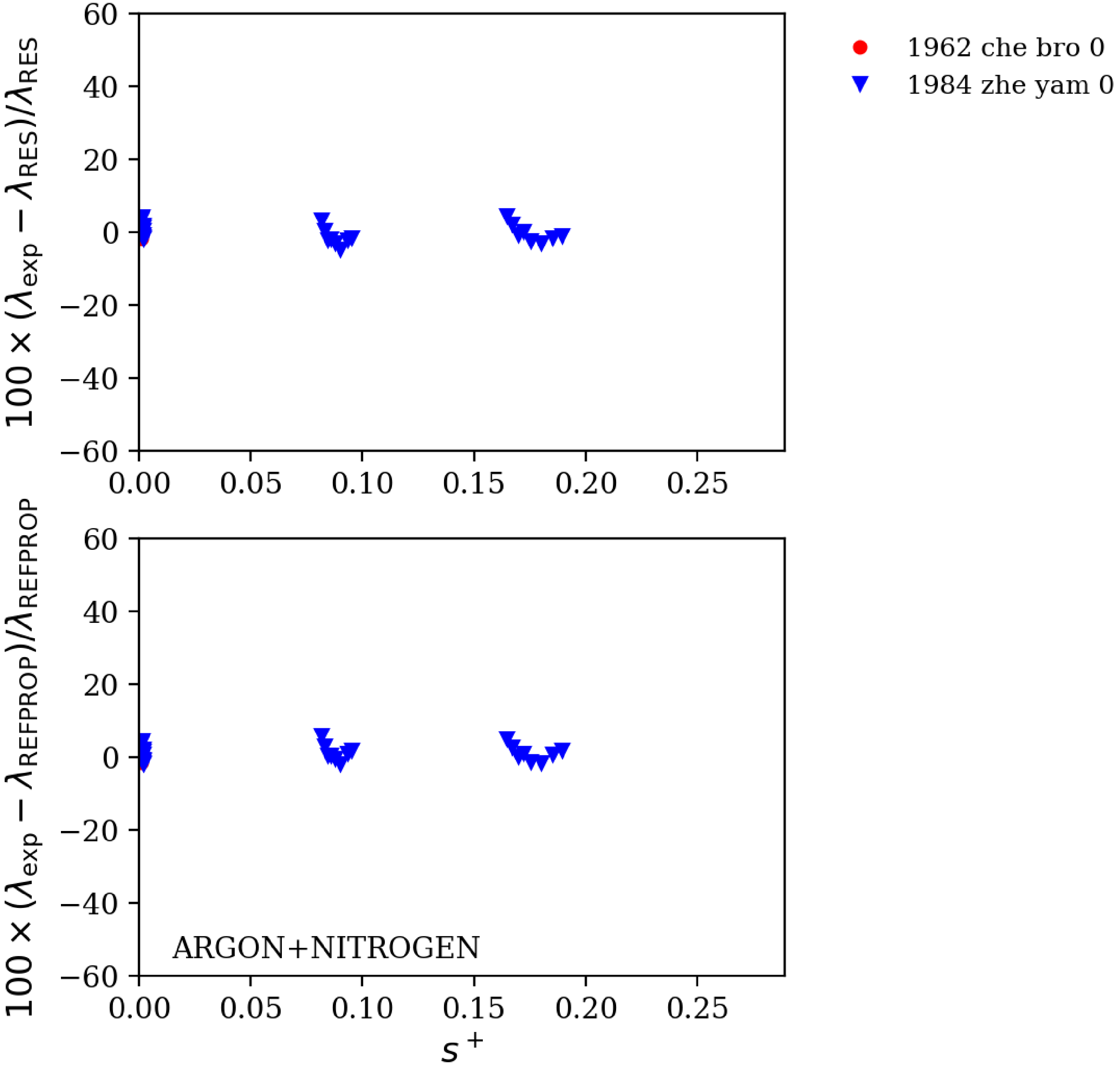

Figure DPR4. ARGON+NITROGEN

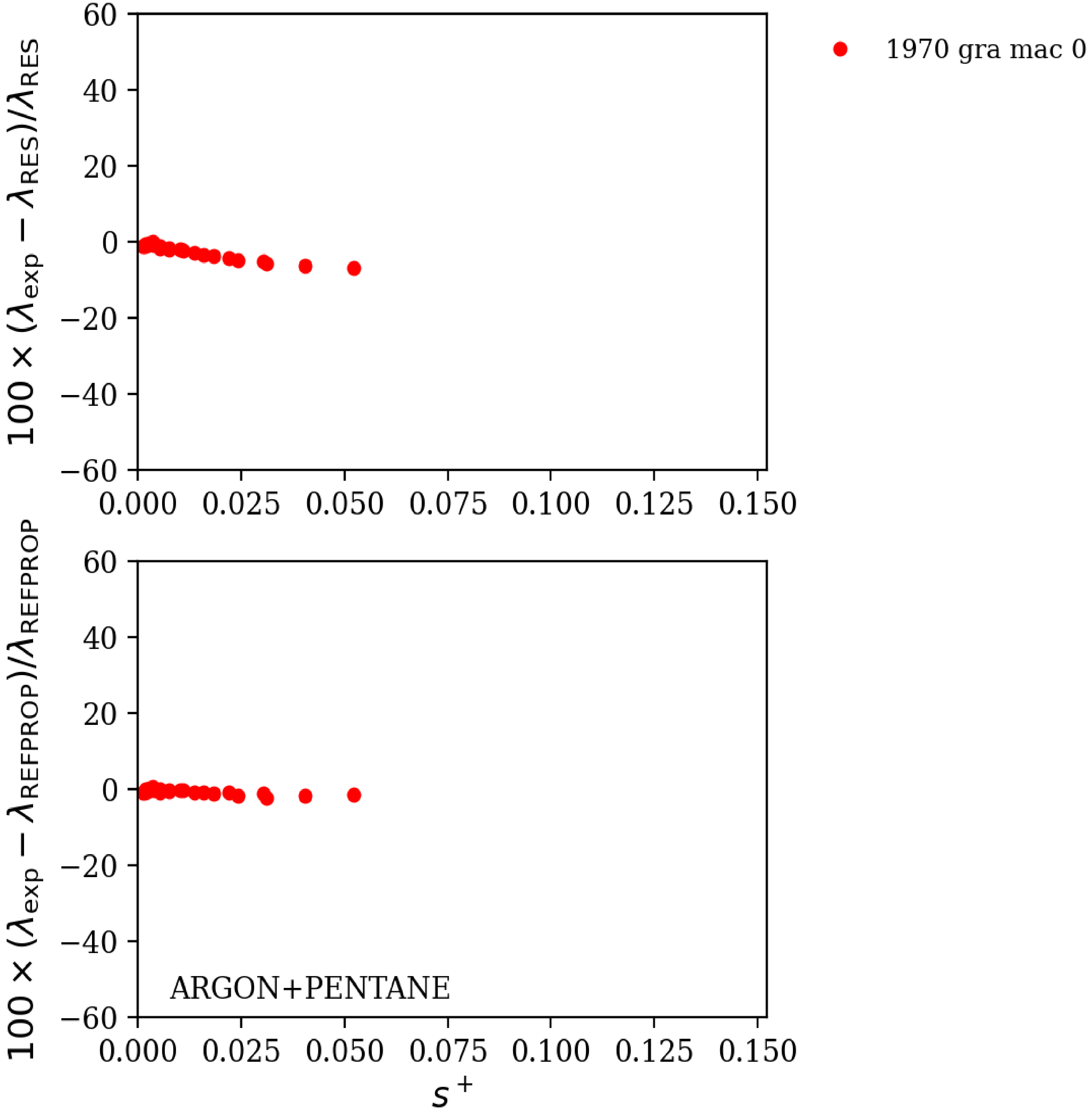

Figure DPR4. ARGON+PENTANE

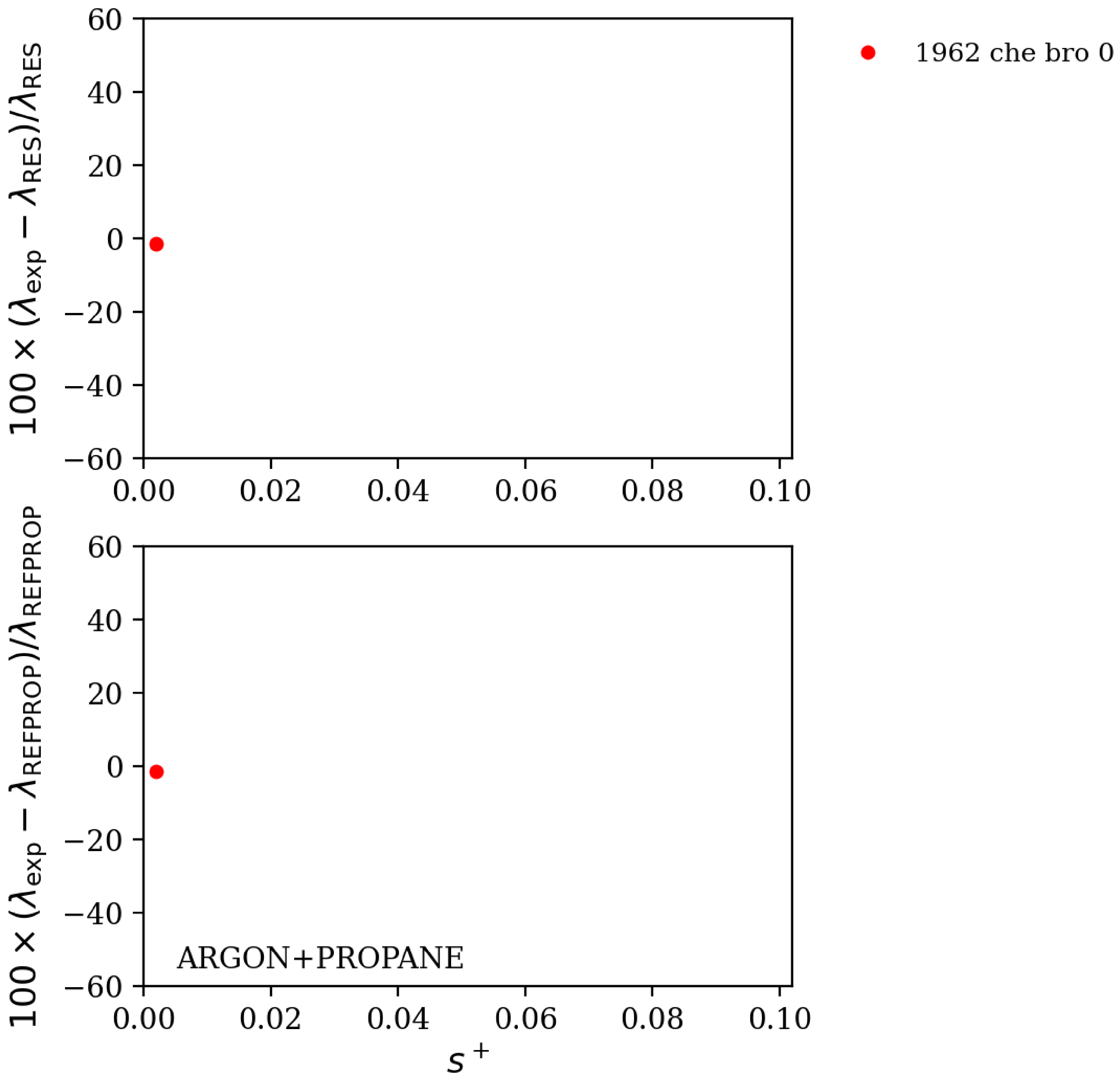

Figure DPR4. ARGON+PROPANE

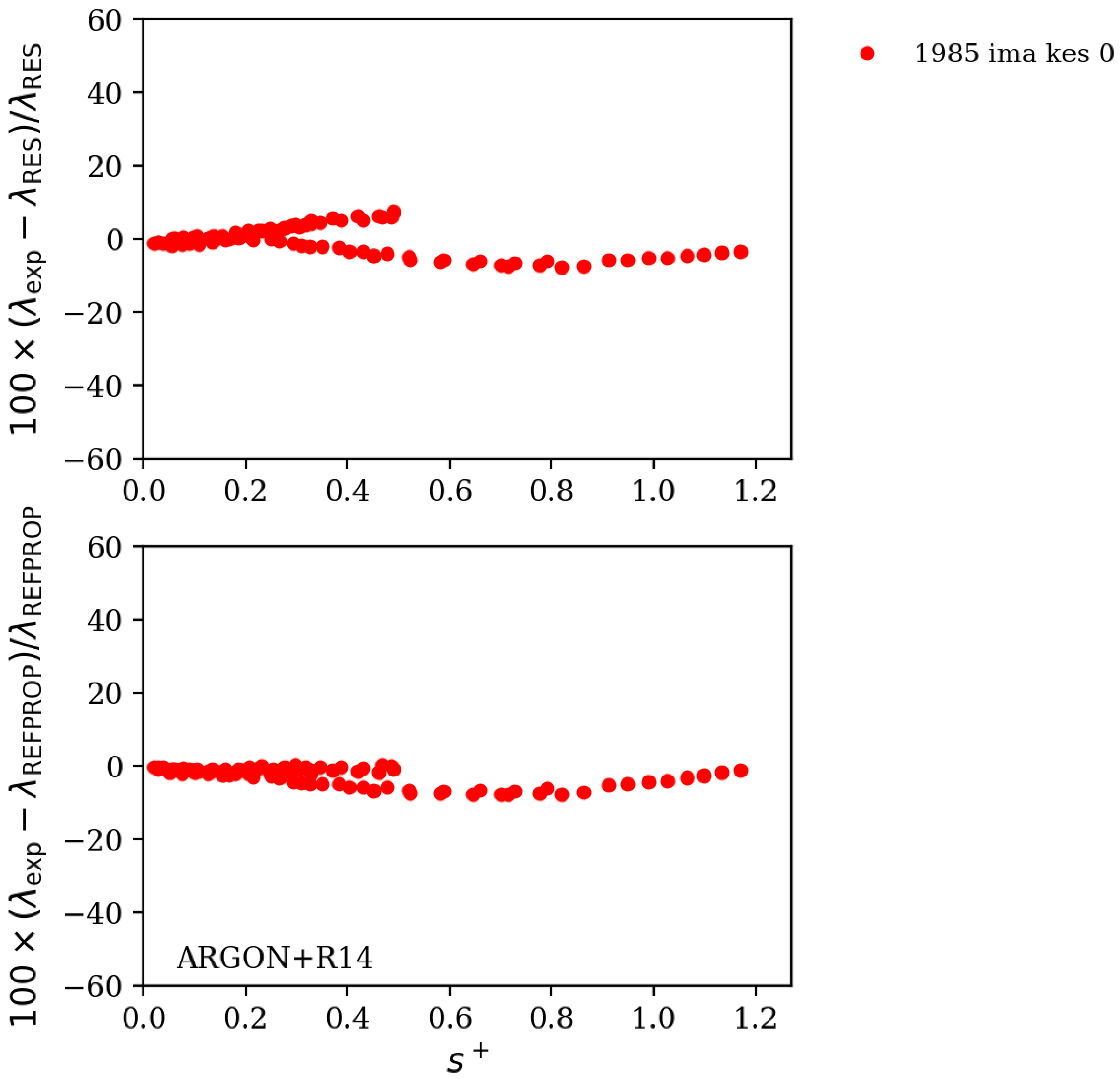

Figure DPR4. ARGON+R14

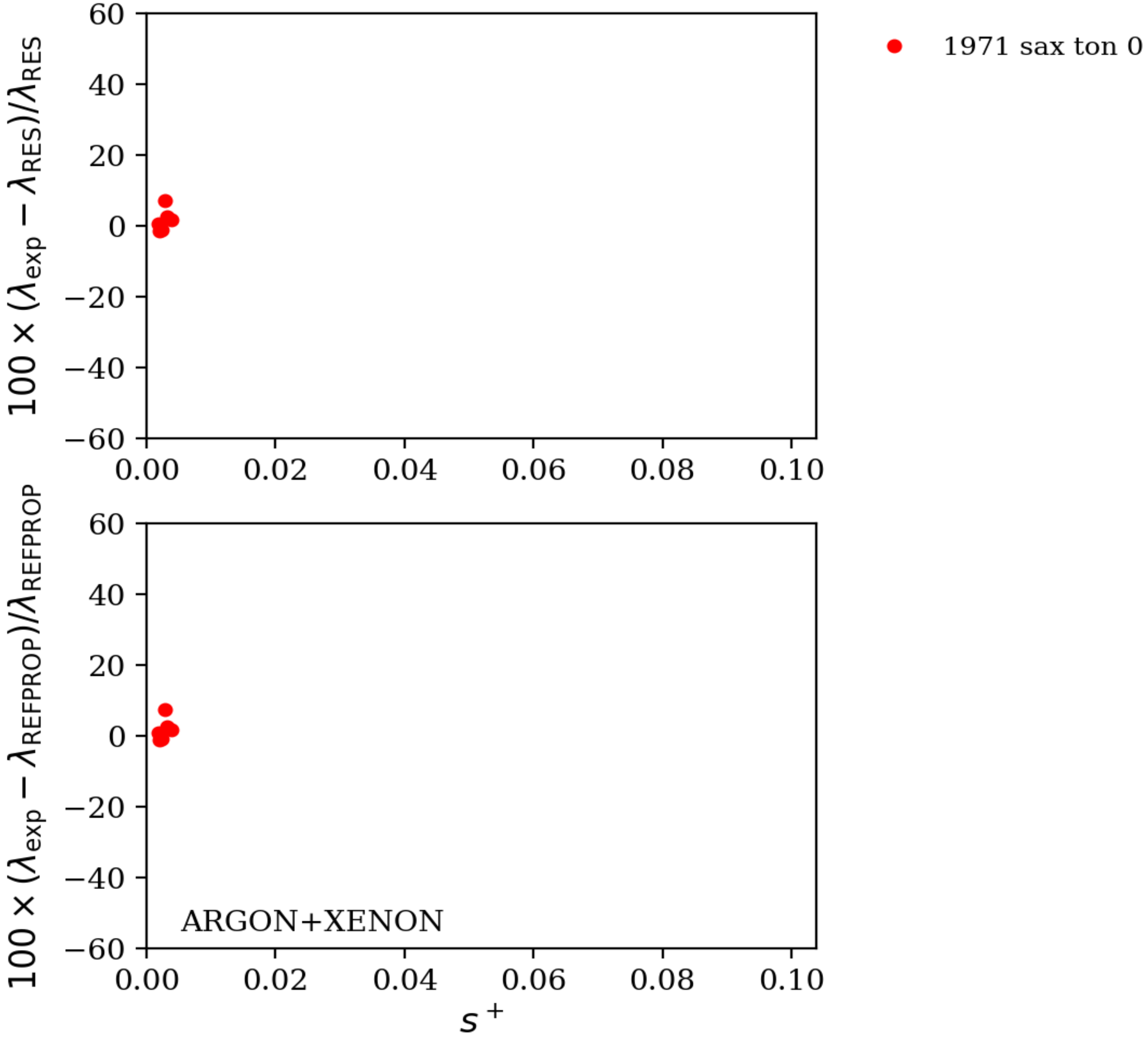

Figure DPR4. ARGON+XENON

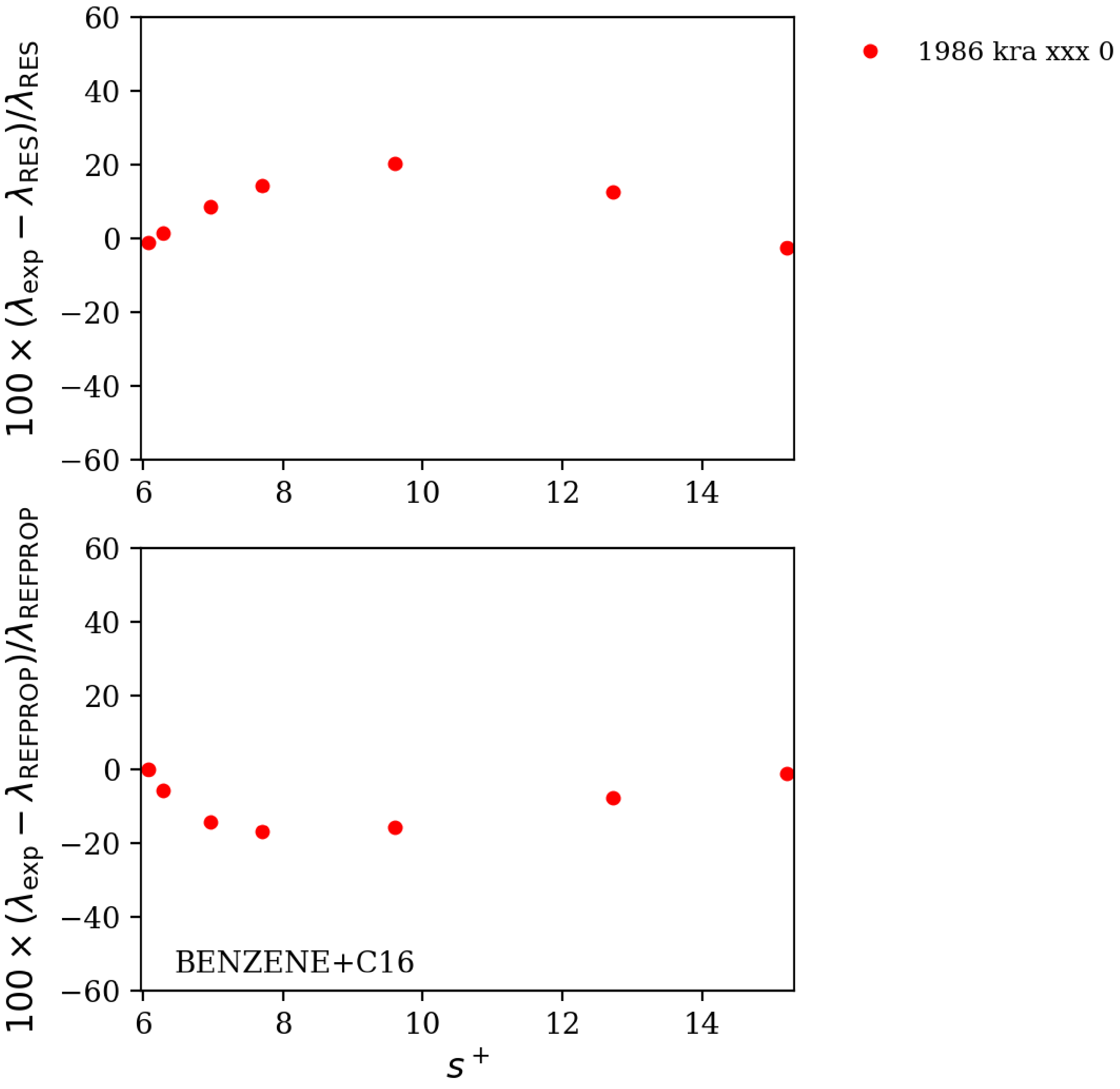

Figure DPR4. BENZENE+C16

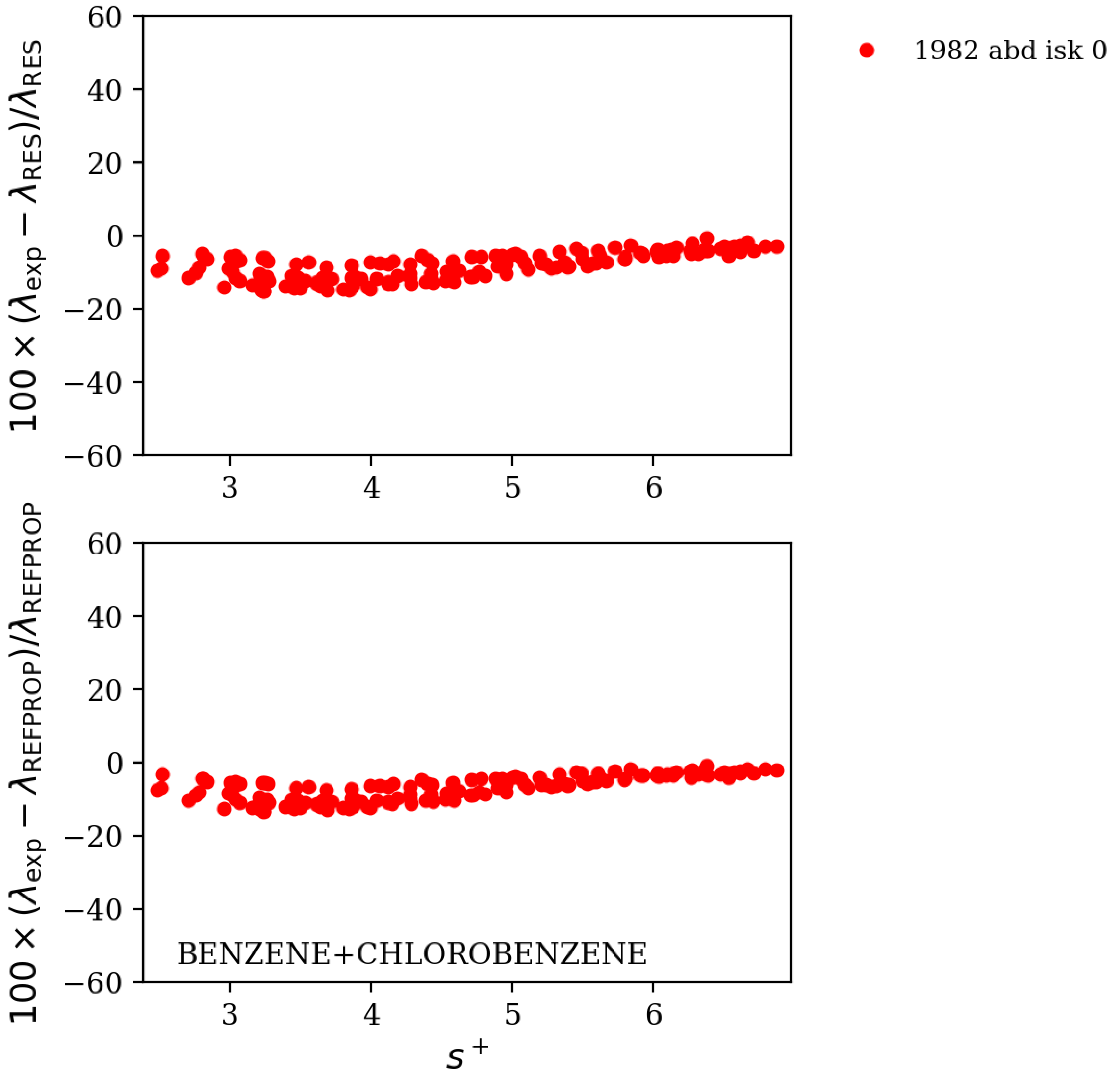

Figure DPR4. BENZENE+CHLOROBENZENE

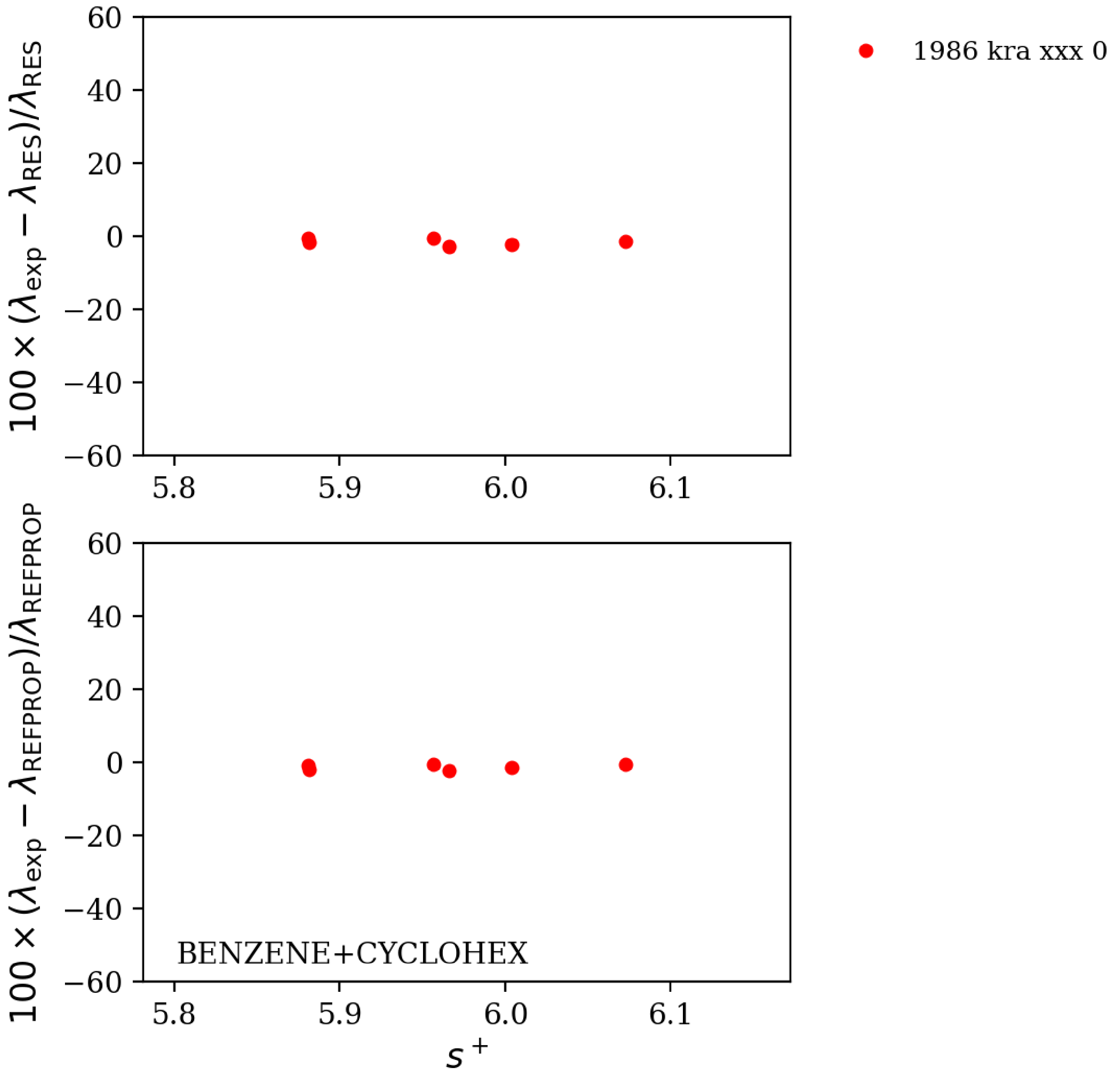

Figure DPR4. BENZENE+CYCLOHEX

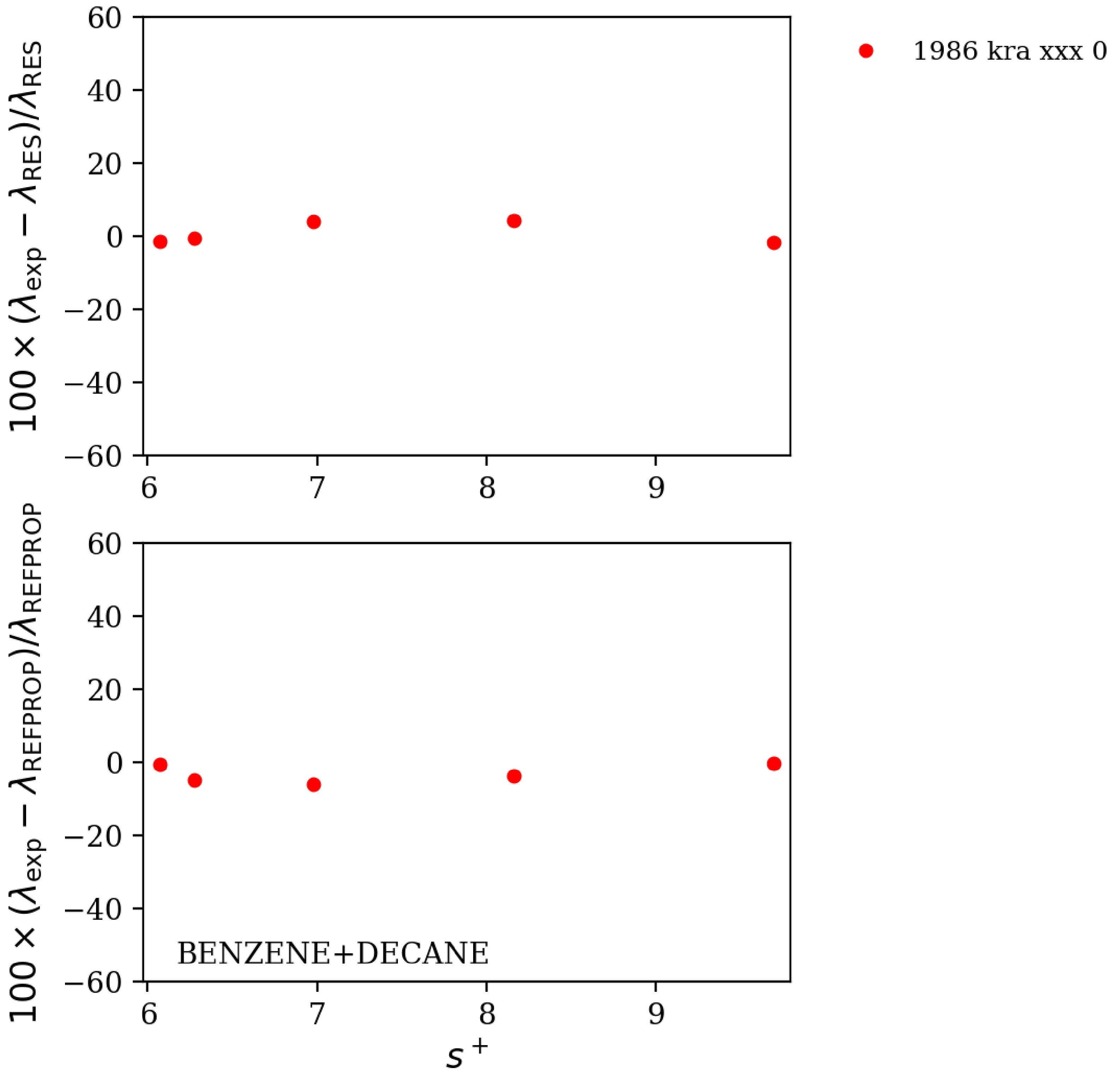

Figure DPR4. BENZENE+DECANE

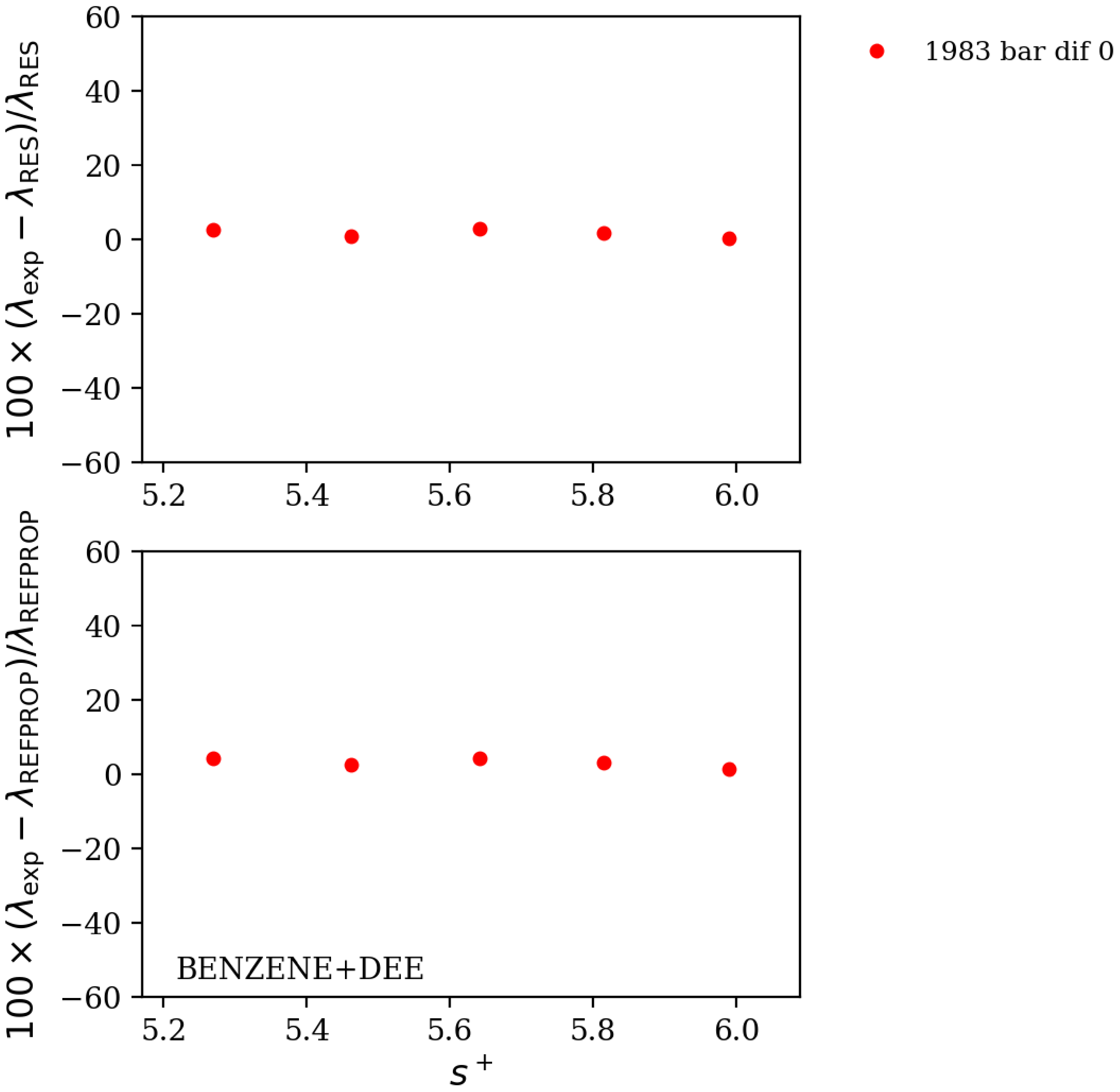

Figure DPR4. BENZENE+DEE

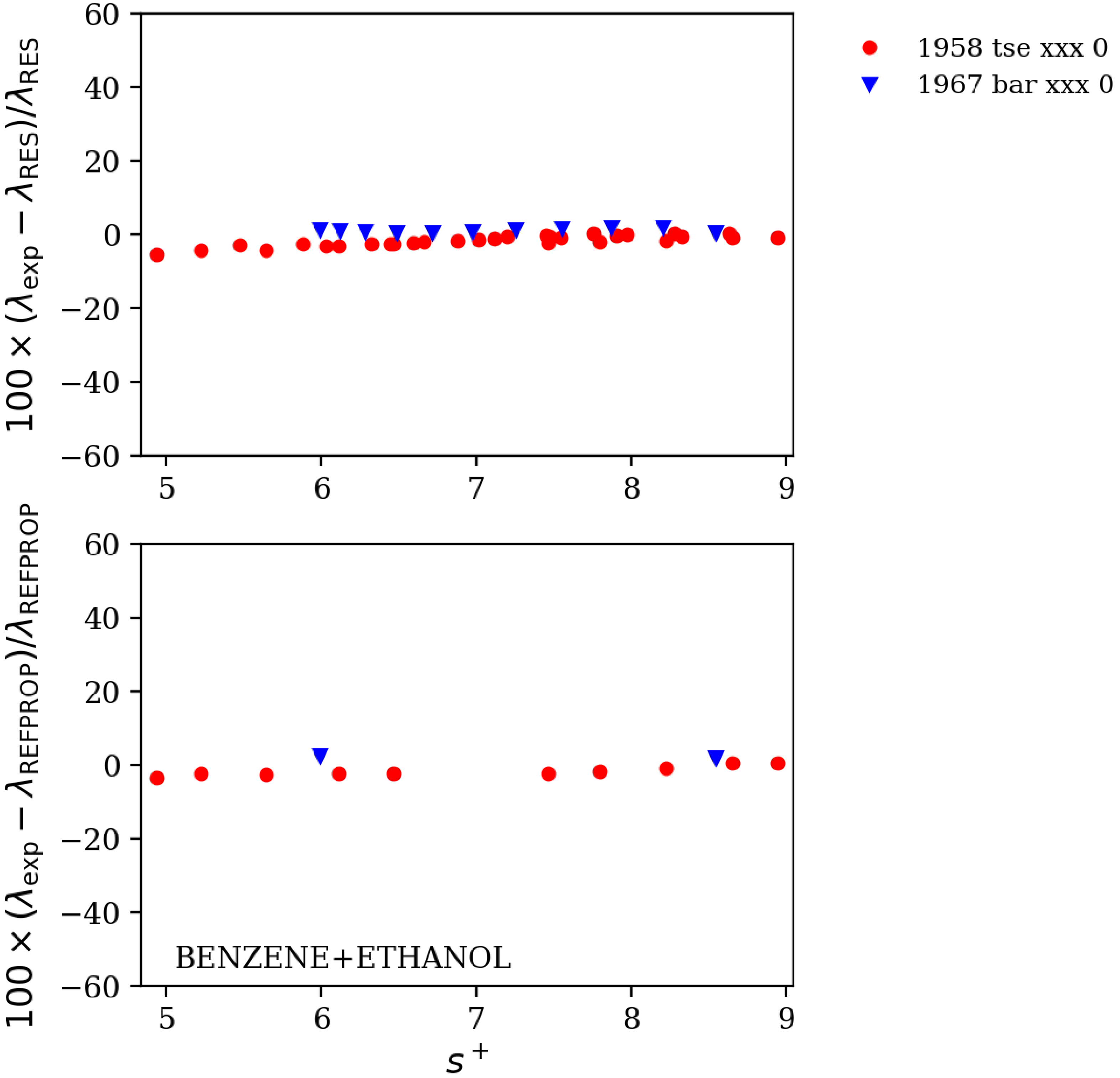

Figure DPR4. BENZENE+ETHANOL

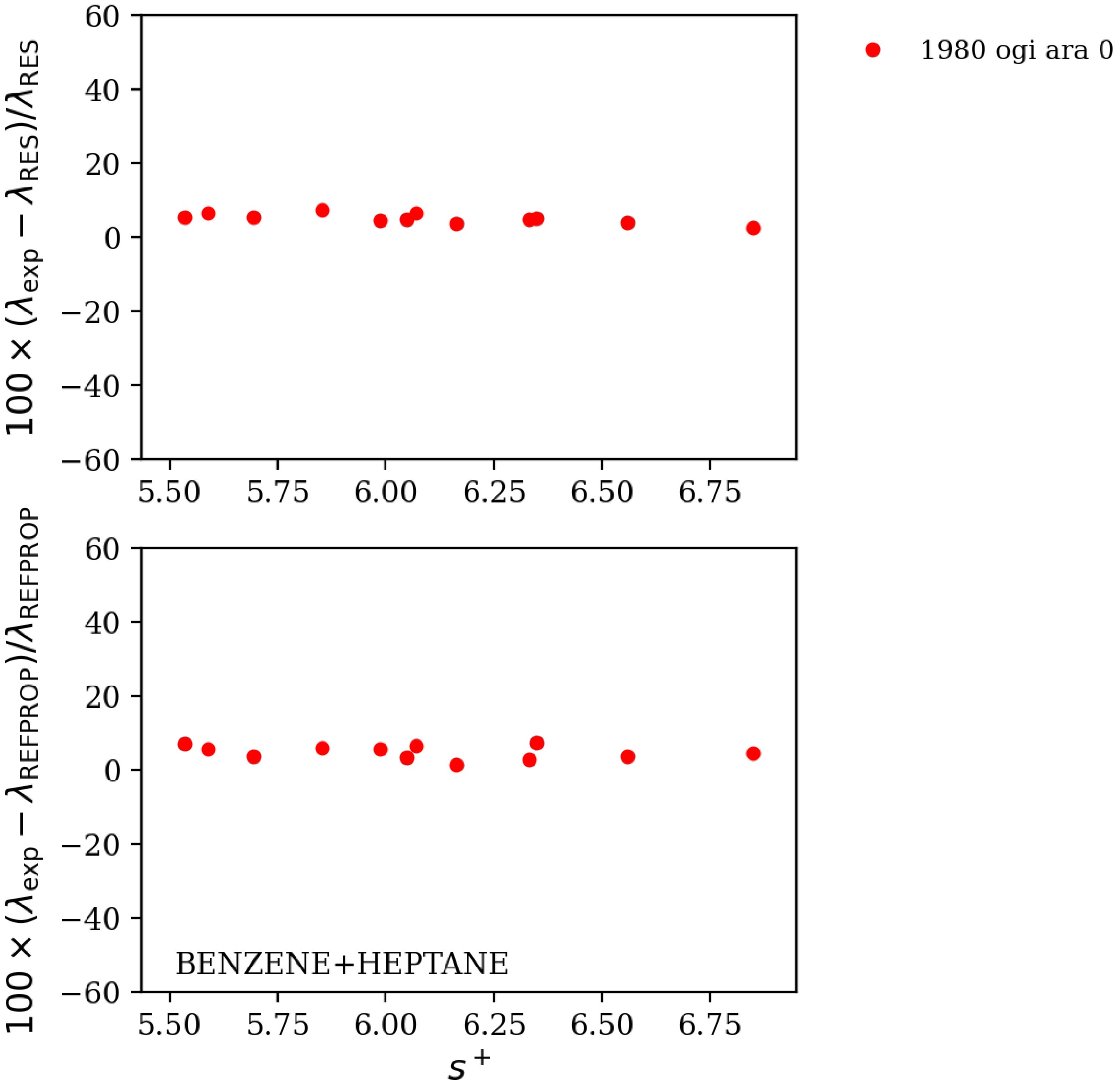

Figure DPR4. BENZENE+HEPTANE

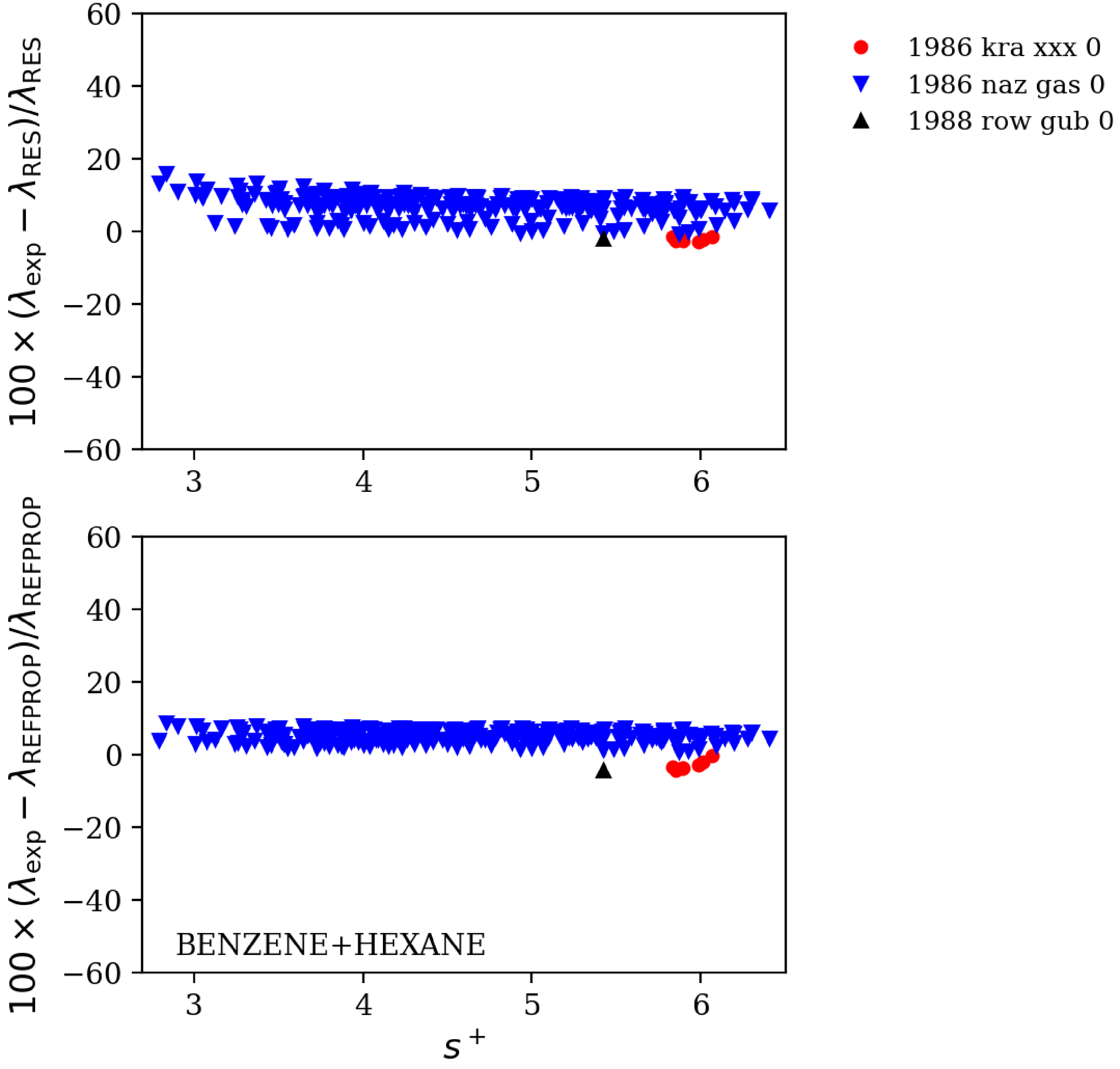

Figure DPR4. BENZENE+HEXANE

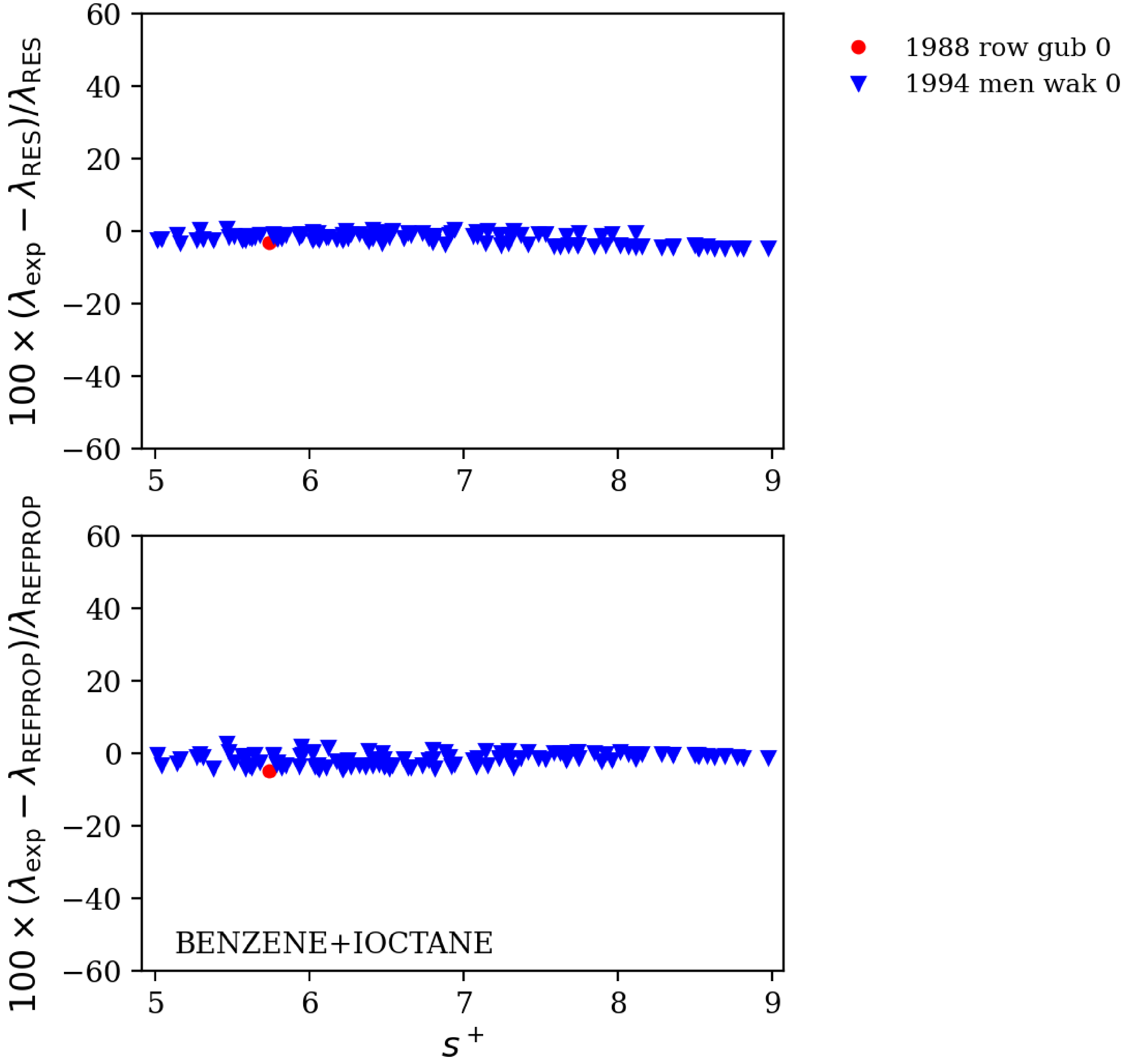

Figure DPR4. BENZENE+IOCTANE

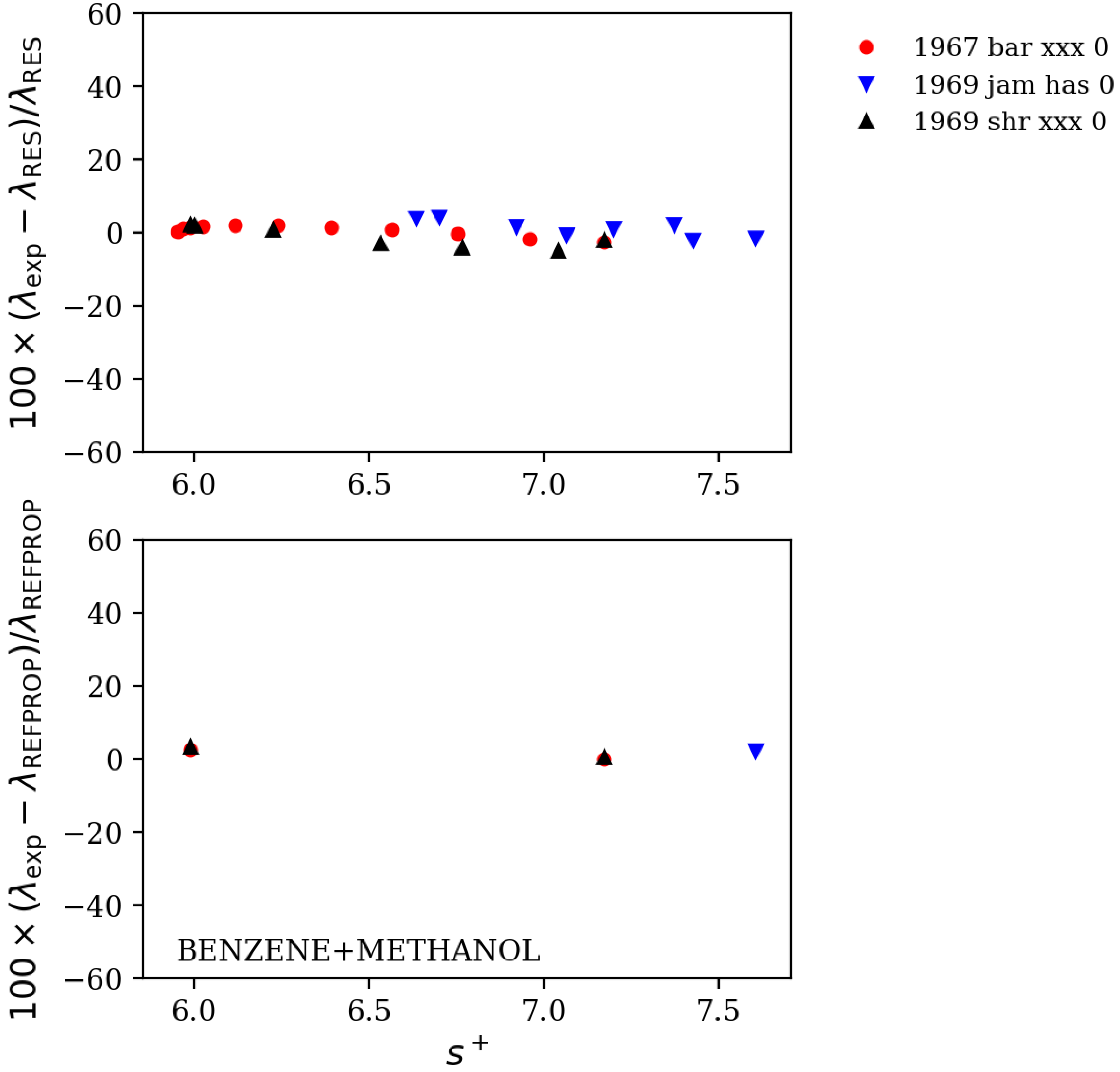

Figure DPR4. BENZENE+METHANOL

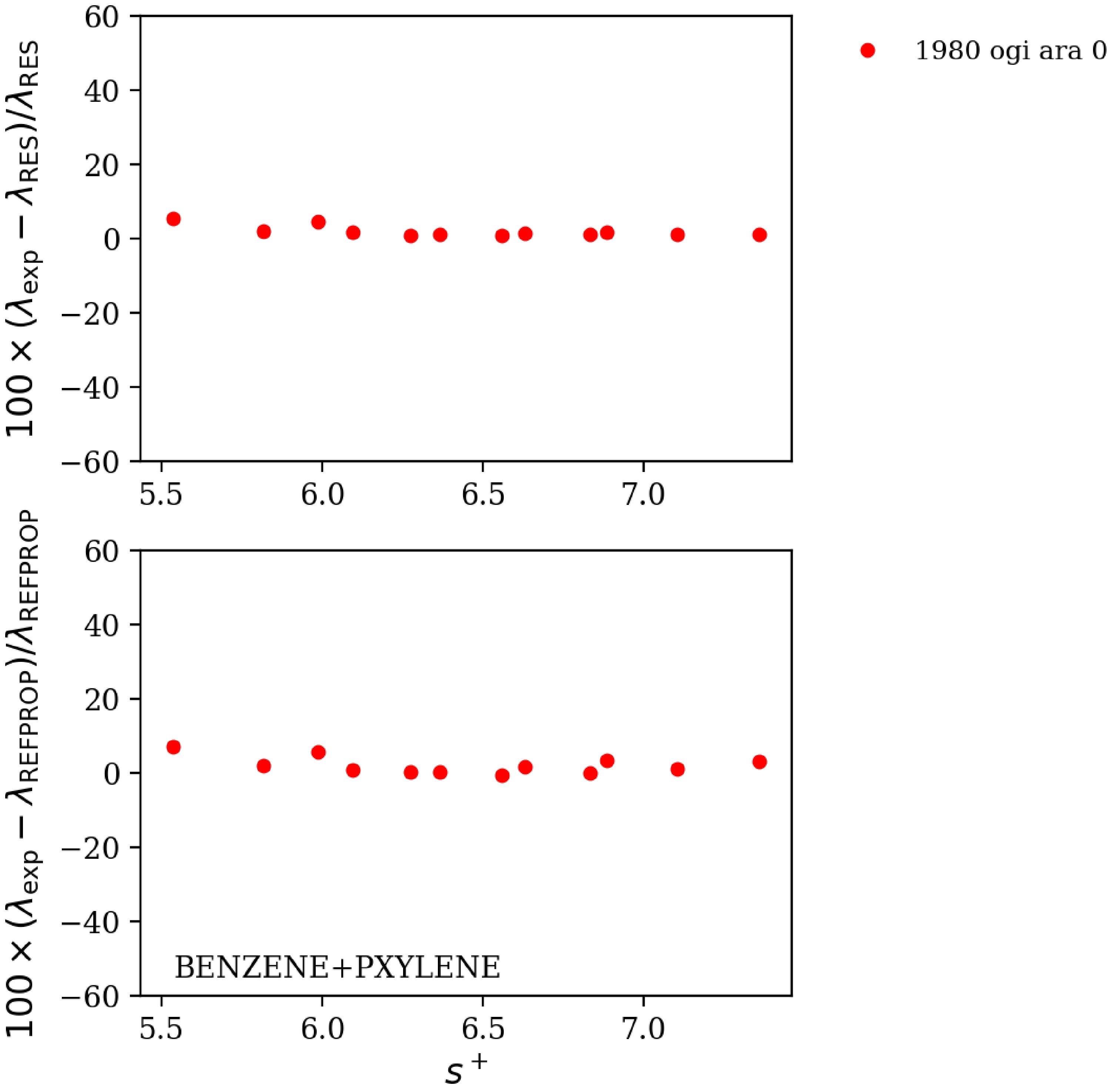

Figure DPR4. BENZENE+PXYLENE

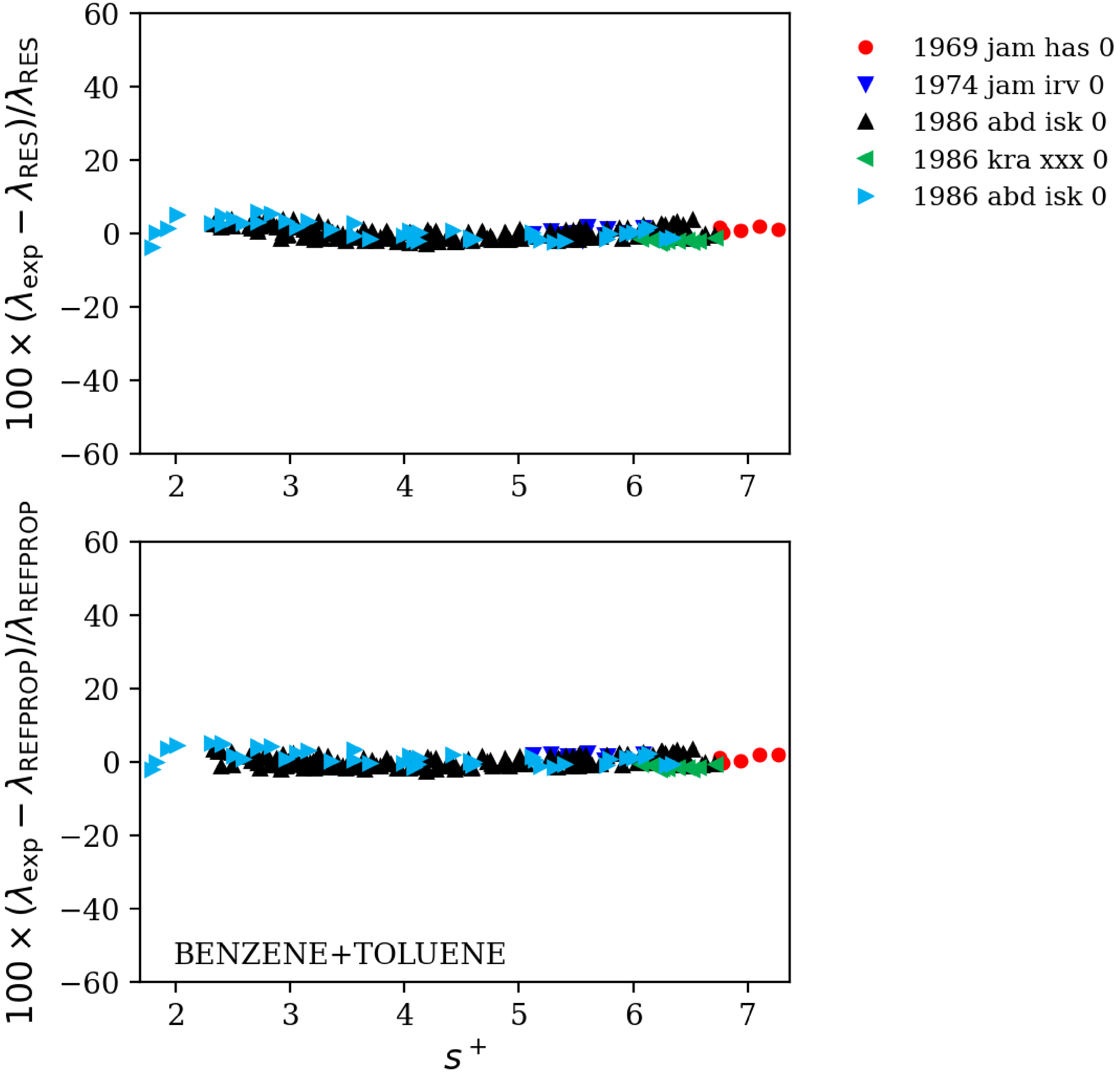

Figure DPR4. BENZENE+TOLUENE

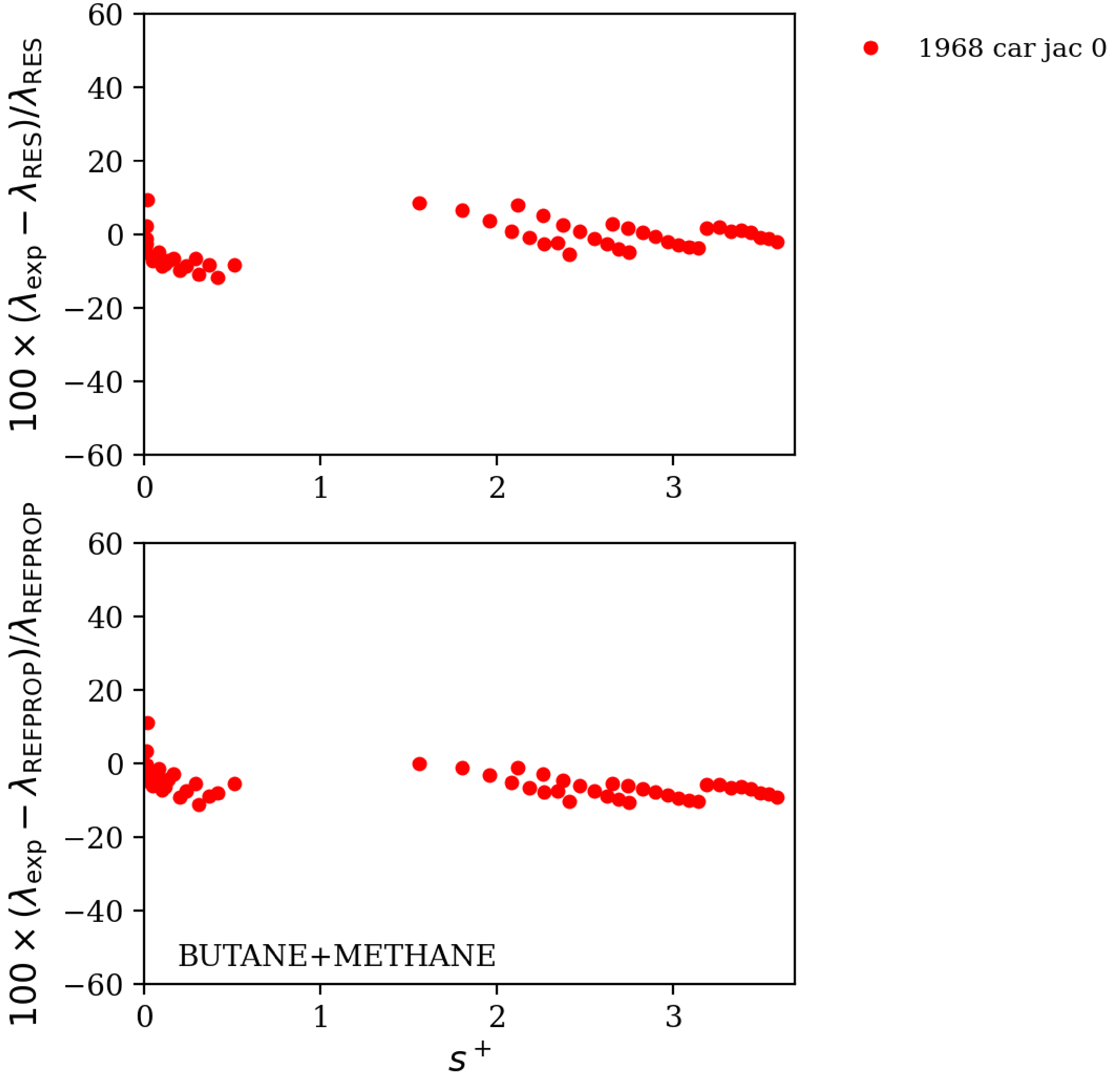

Figure DPR4. BUTANE+METHANE

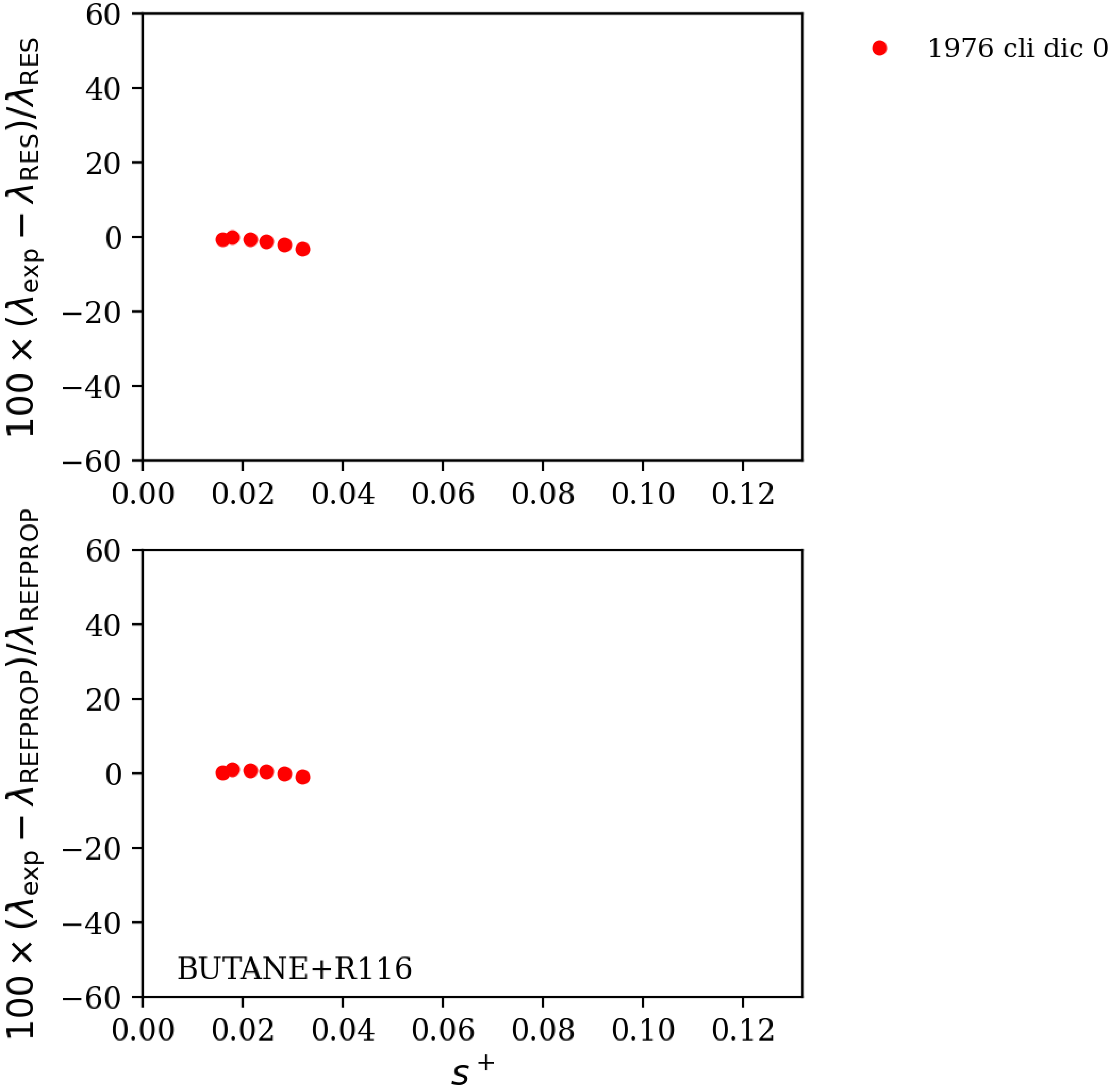

Figure DPR4. BUTANE+R116

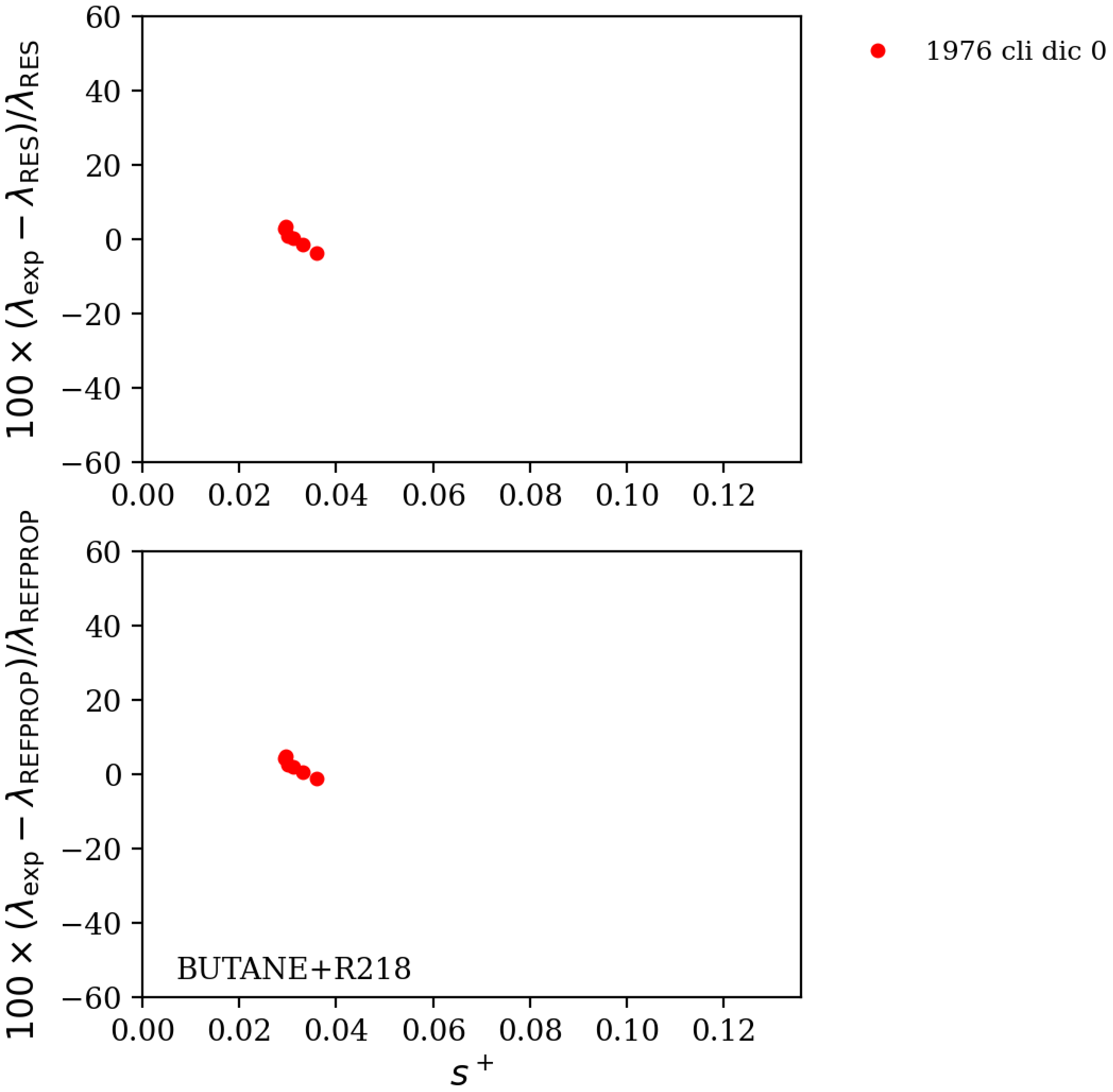

Figure DPR4. BUTANE+R218

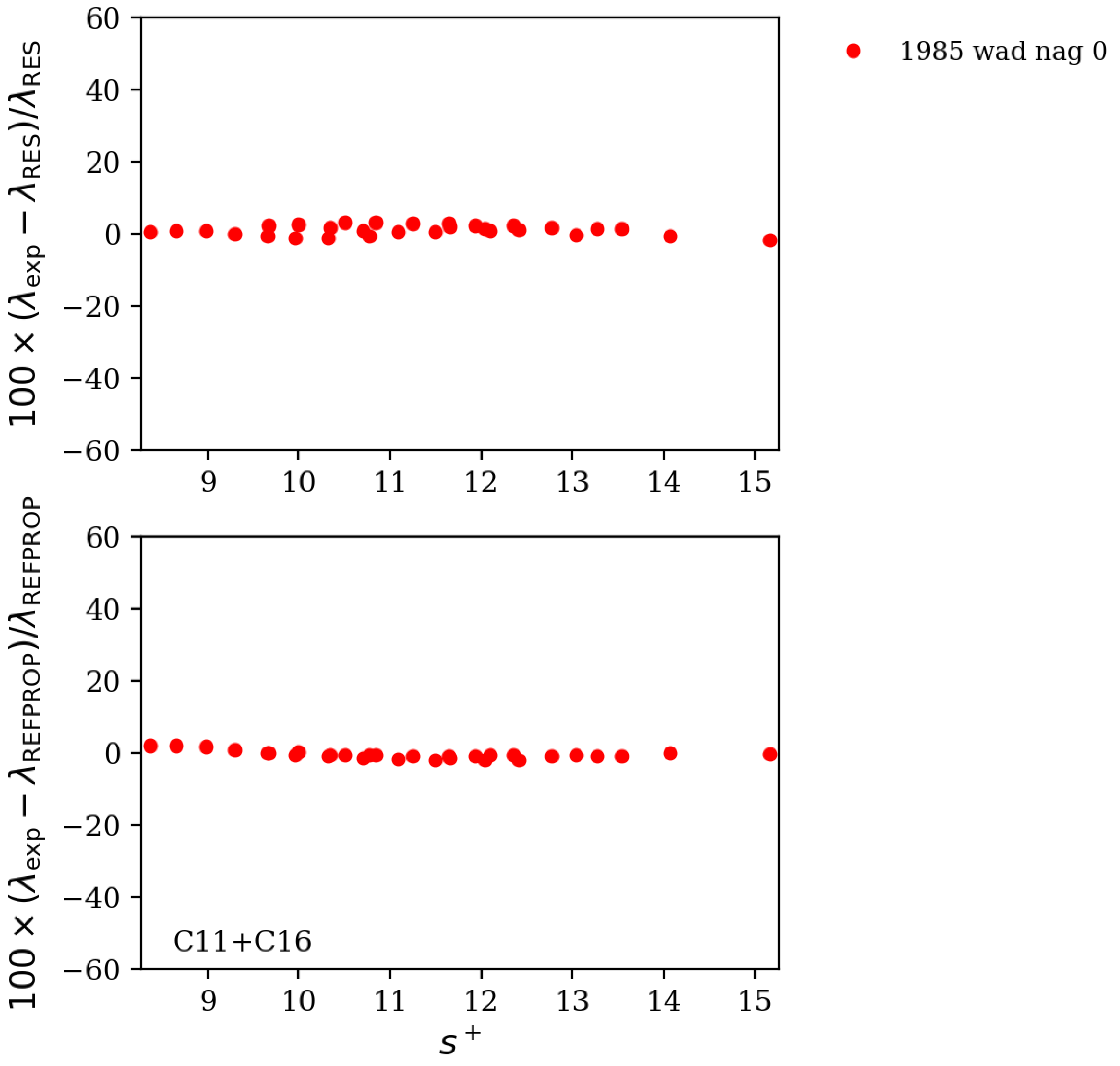

Figure DPR4. C11+C16

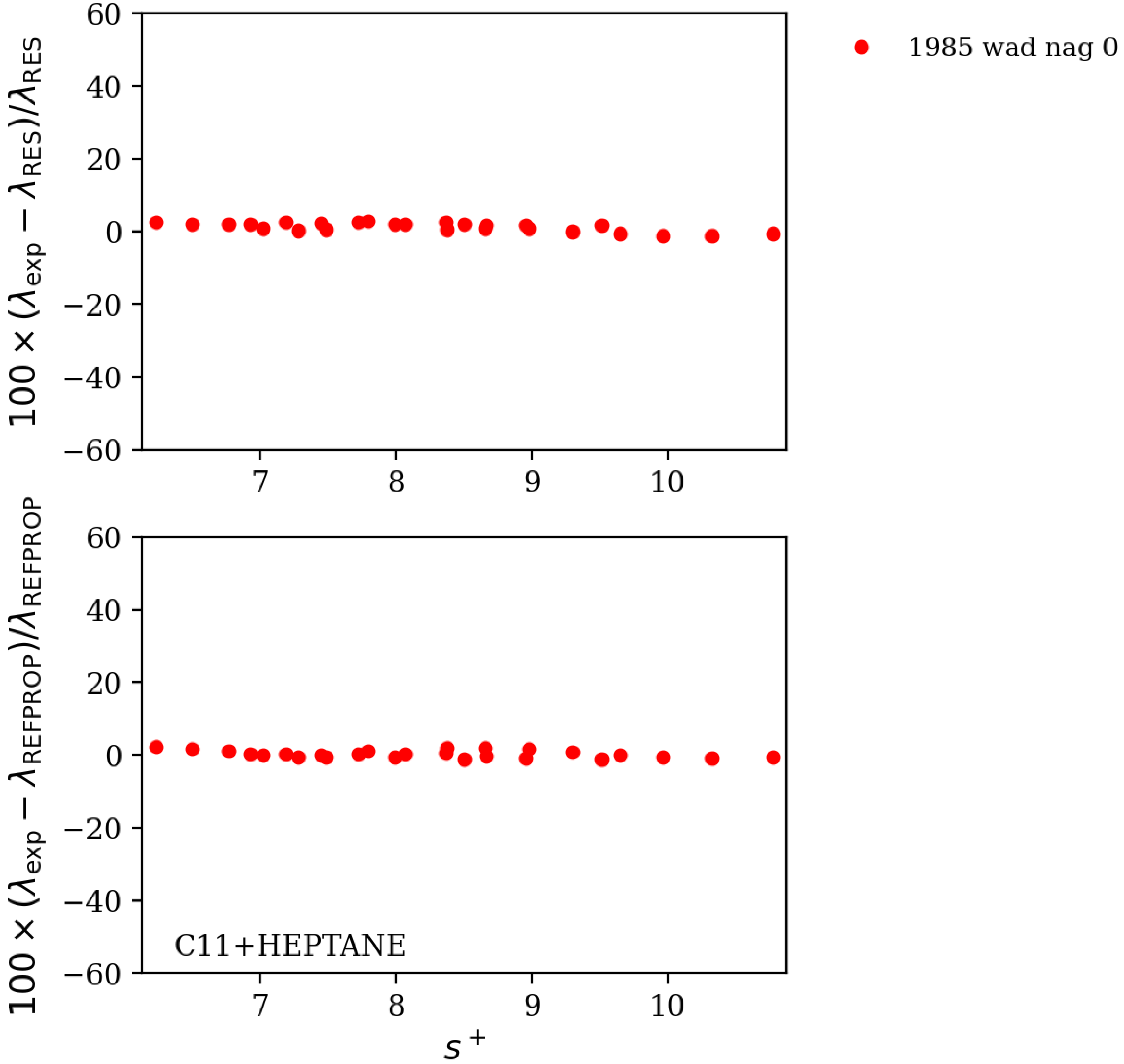

Figure DPR4. C11+HEPTANE

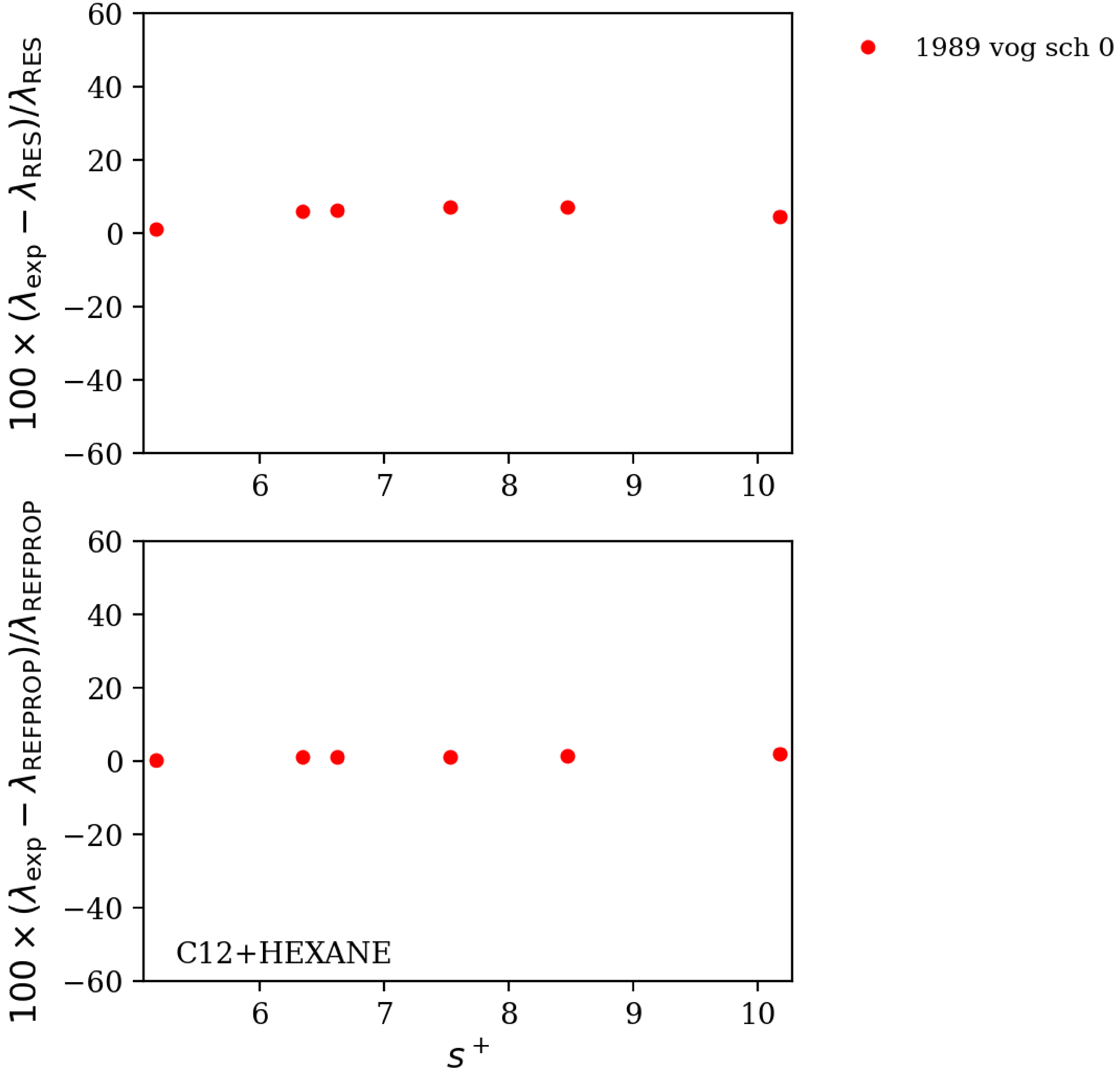

Figure DPR4. C12+HEXANE

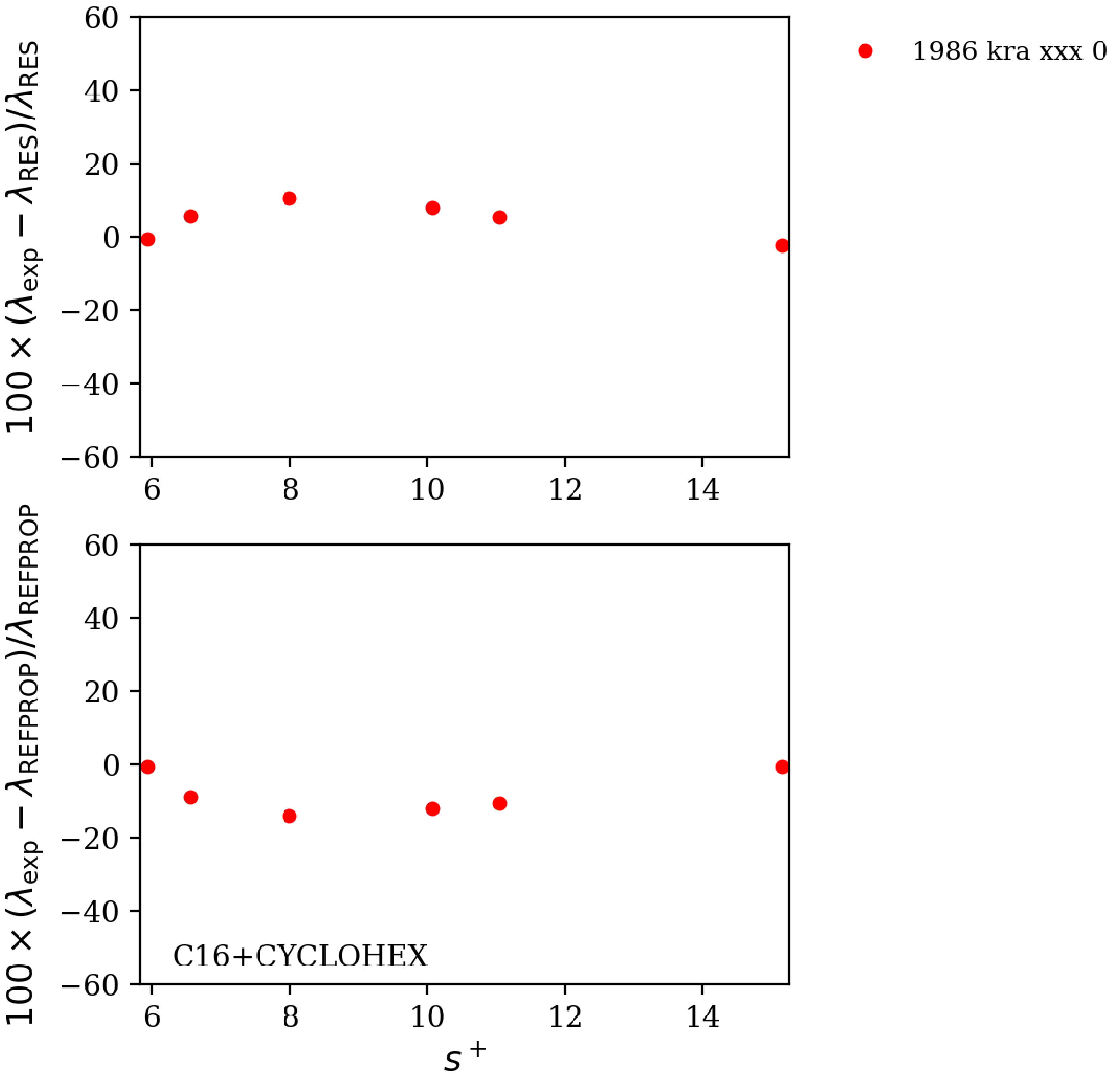

Figure DPR4. C16+CYCLOHEX

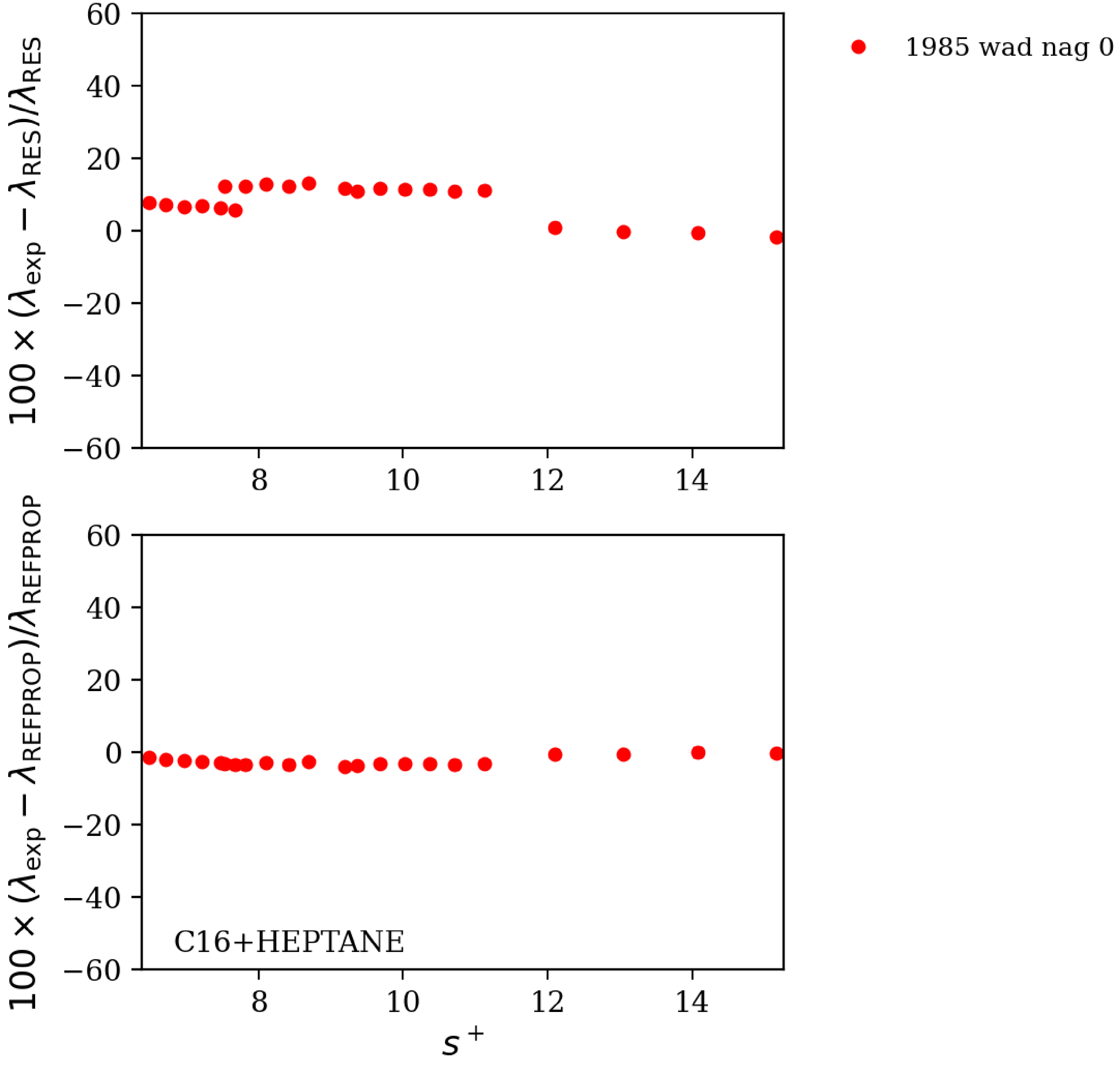

Figure DPR4. C16+HEPTANE

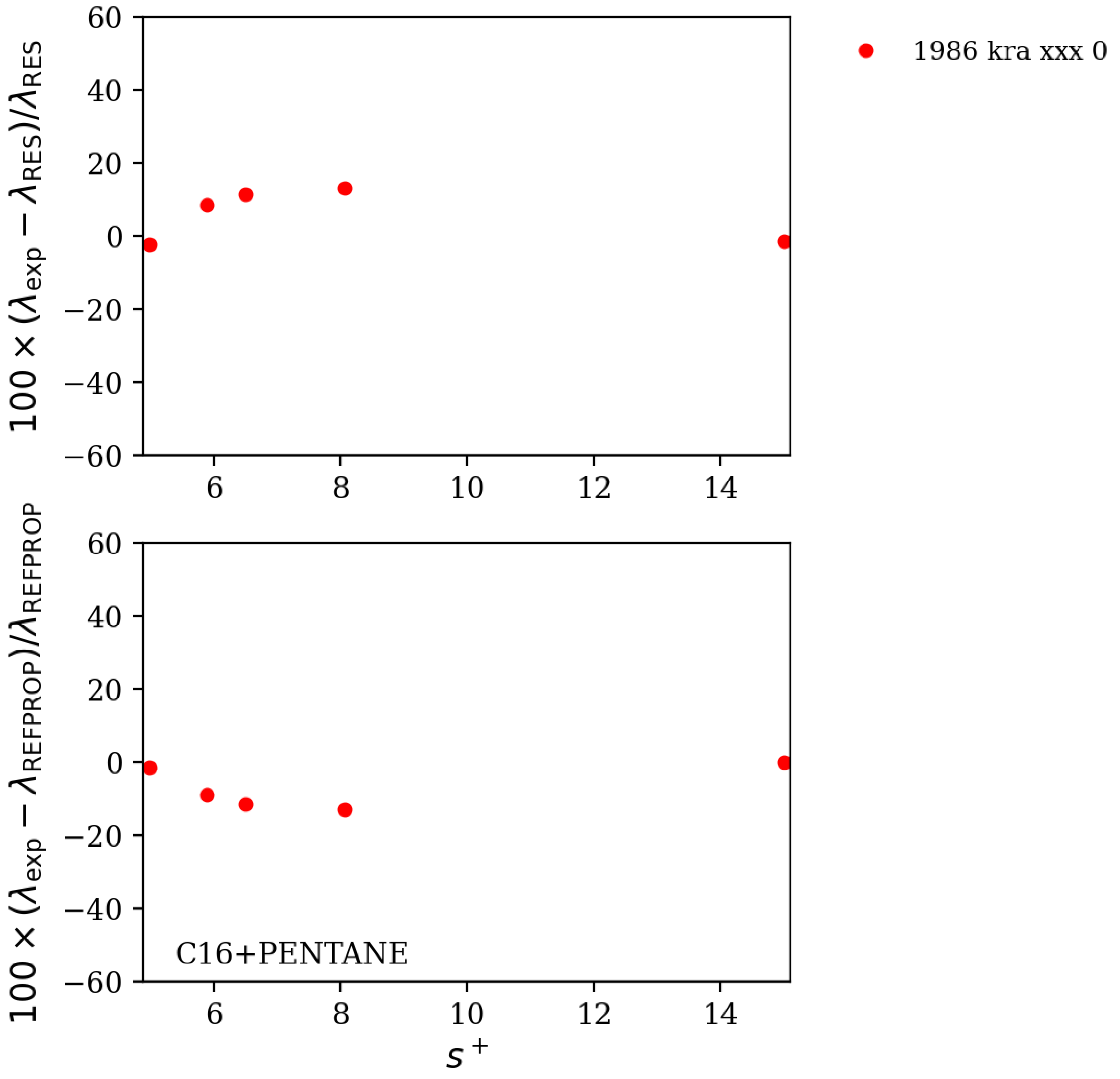

Figure DPR4. C16+PENTANE

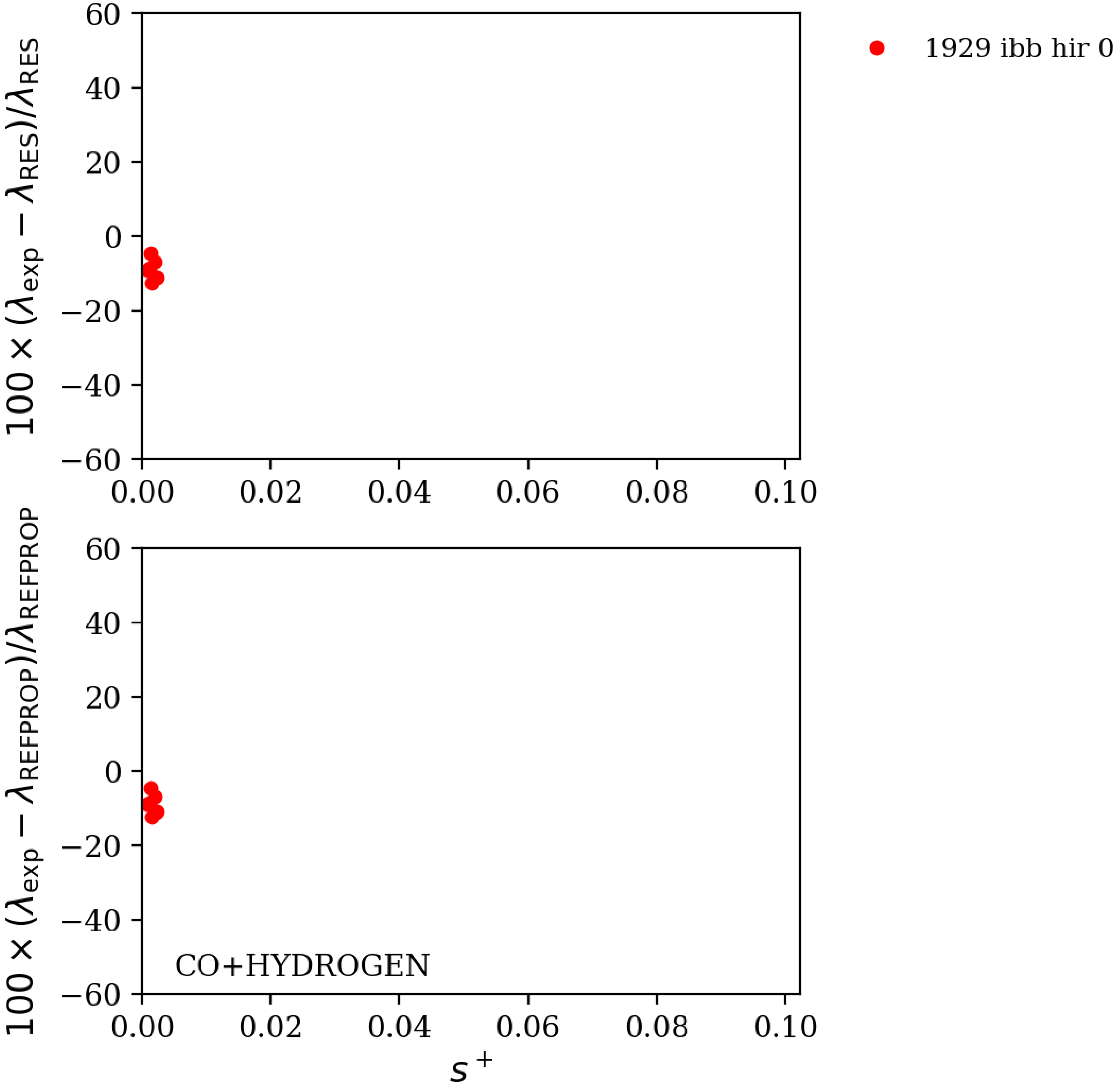

Figure DPR4. CO+HYDROGEN

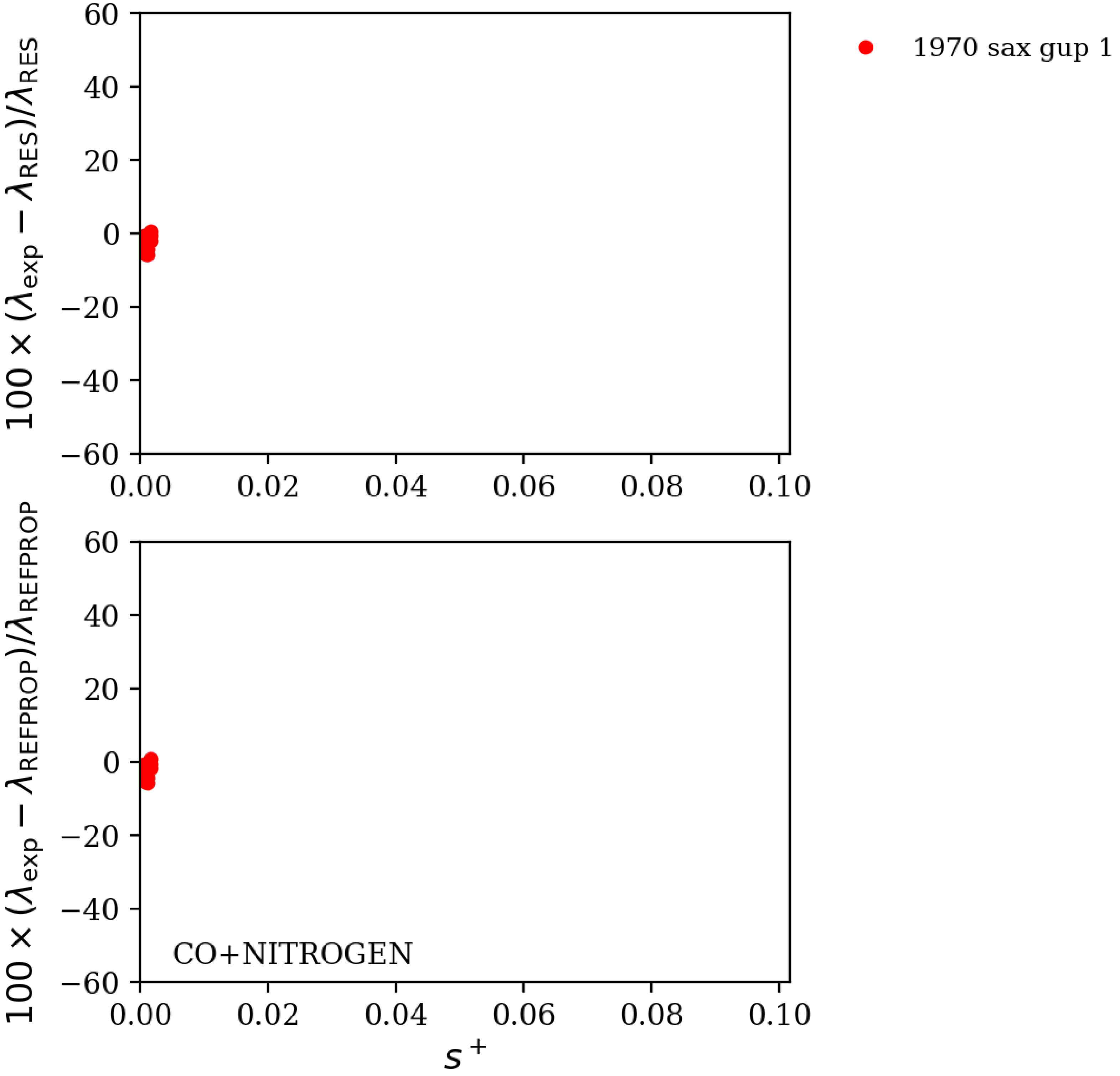

Figure DPR4. CO+NITROGEN

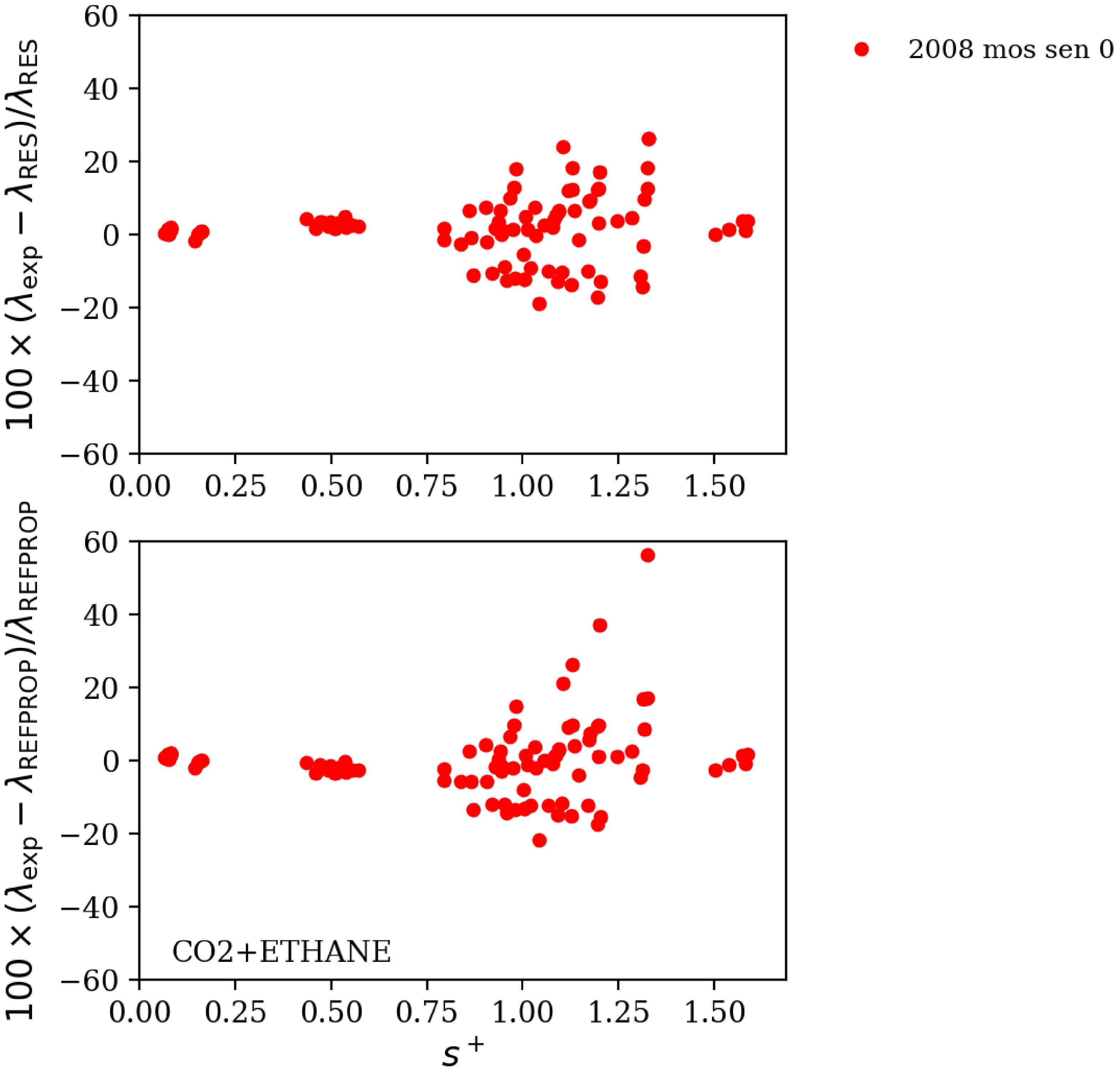

Figure DPR4. CO2+ETHANE

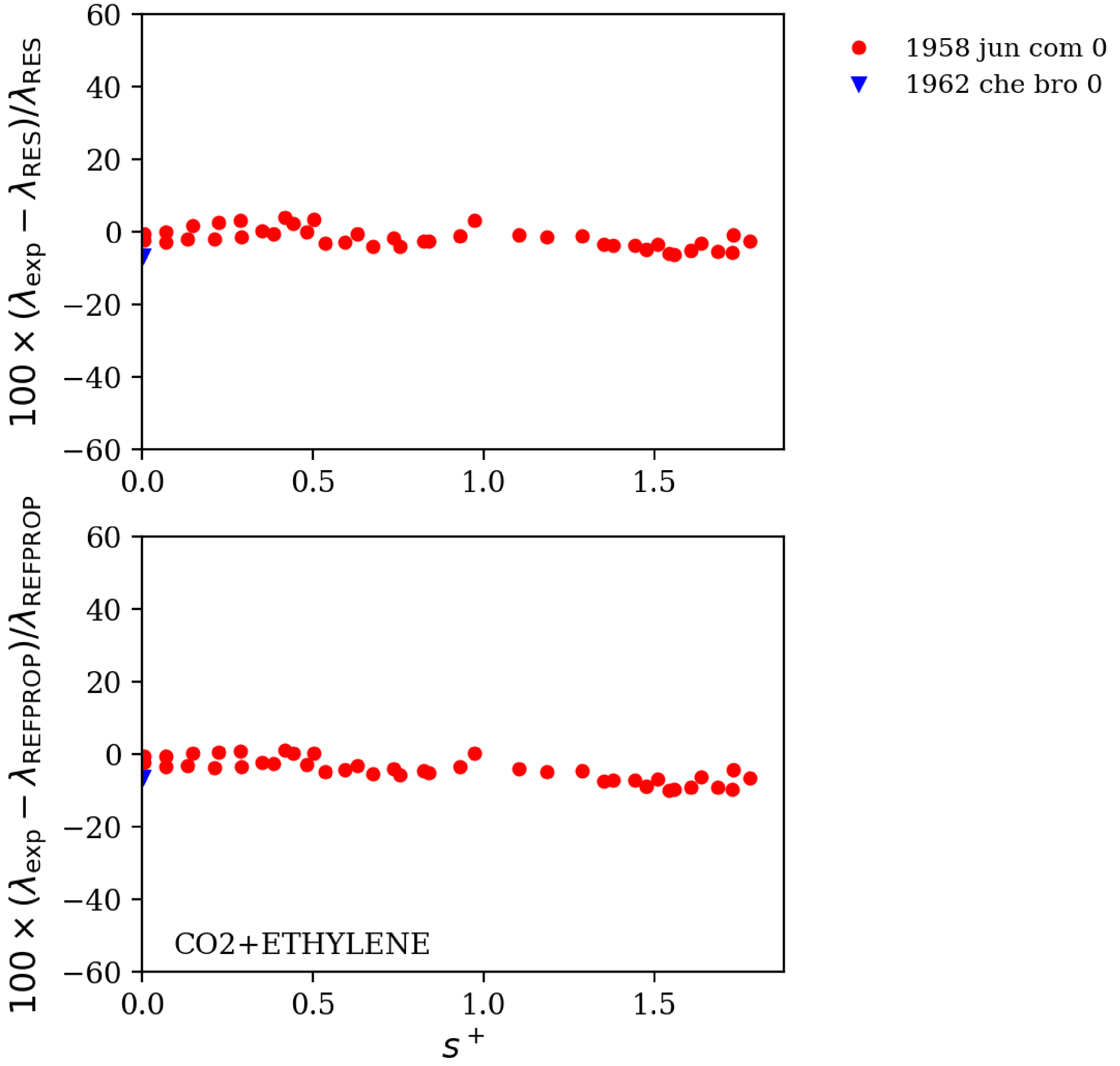

Figure DPR4. CO2+ETHYLENE

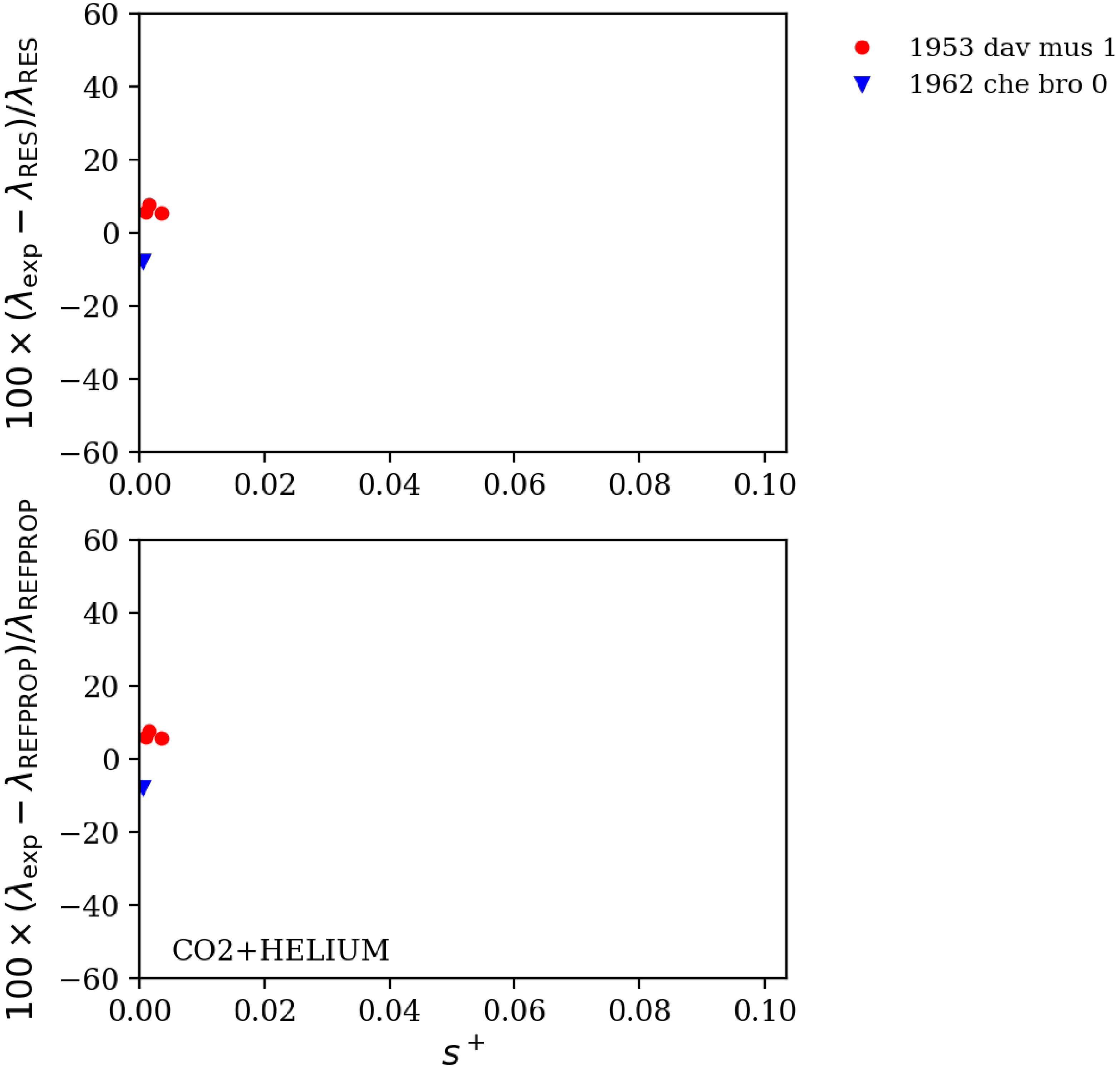

Figure DPR4. CO2+HELIUM

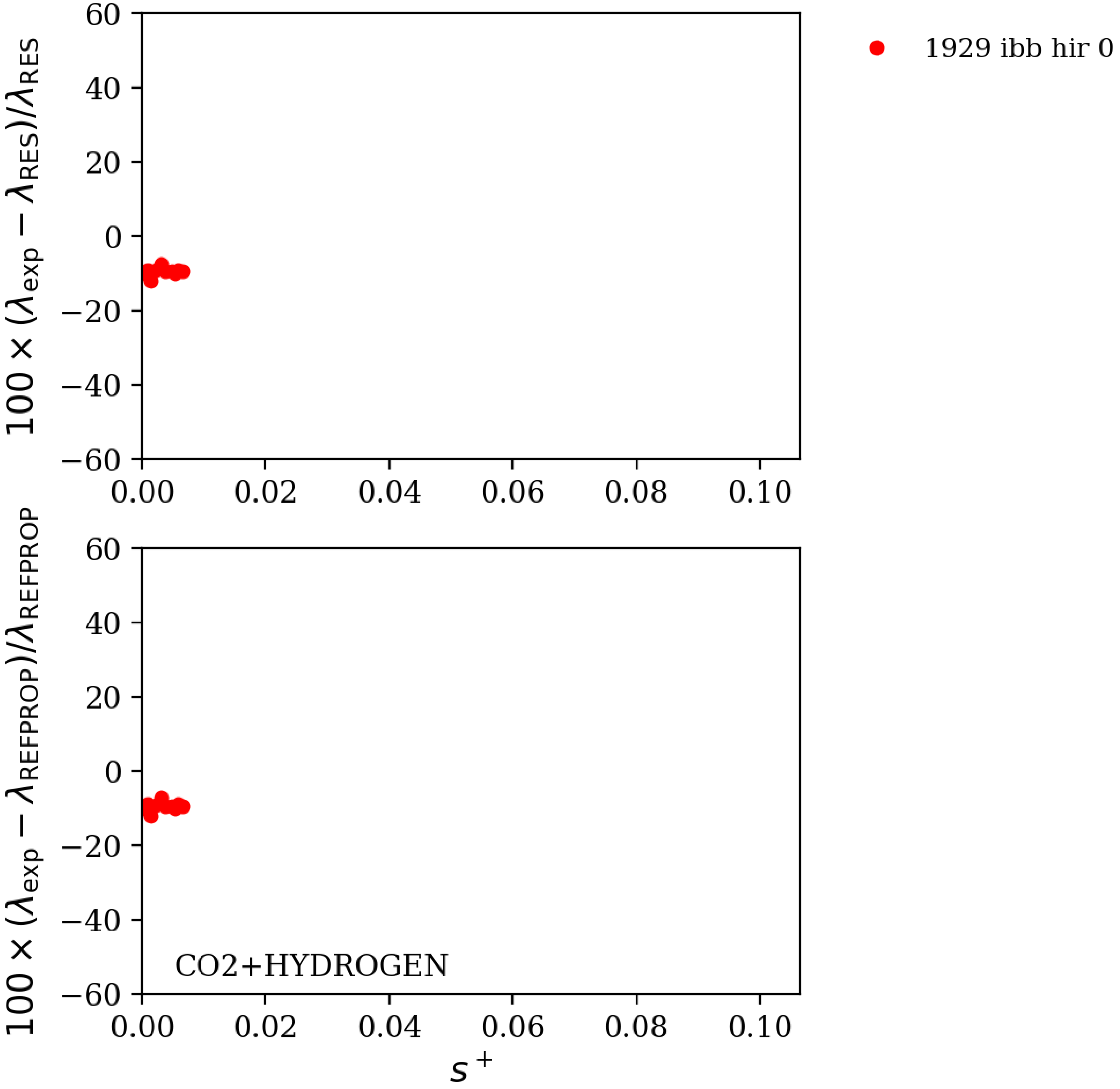

Figure DPR4. CO2+HYDROGEN

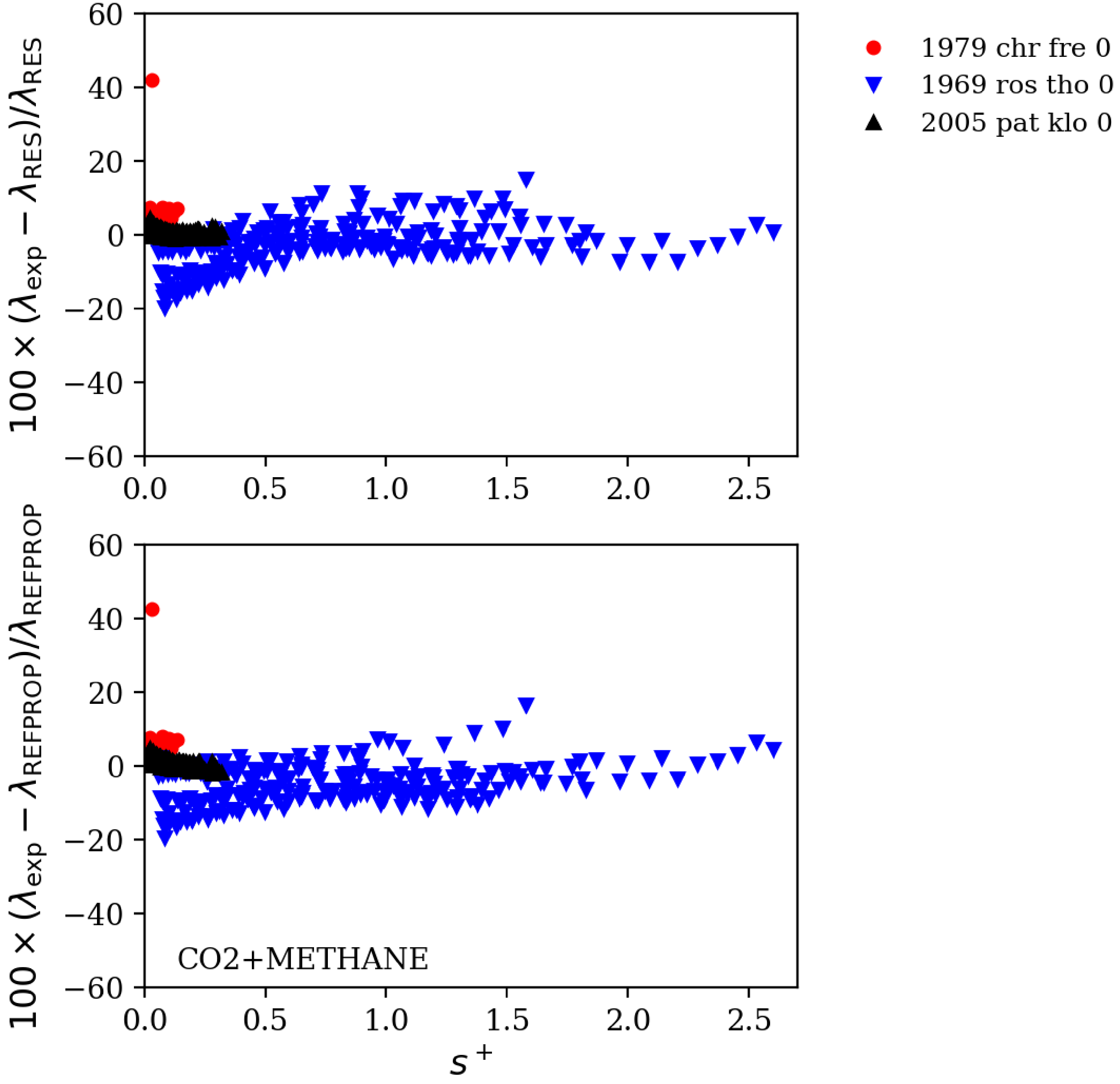

Figure DPR4. CO2+METHANE

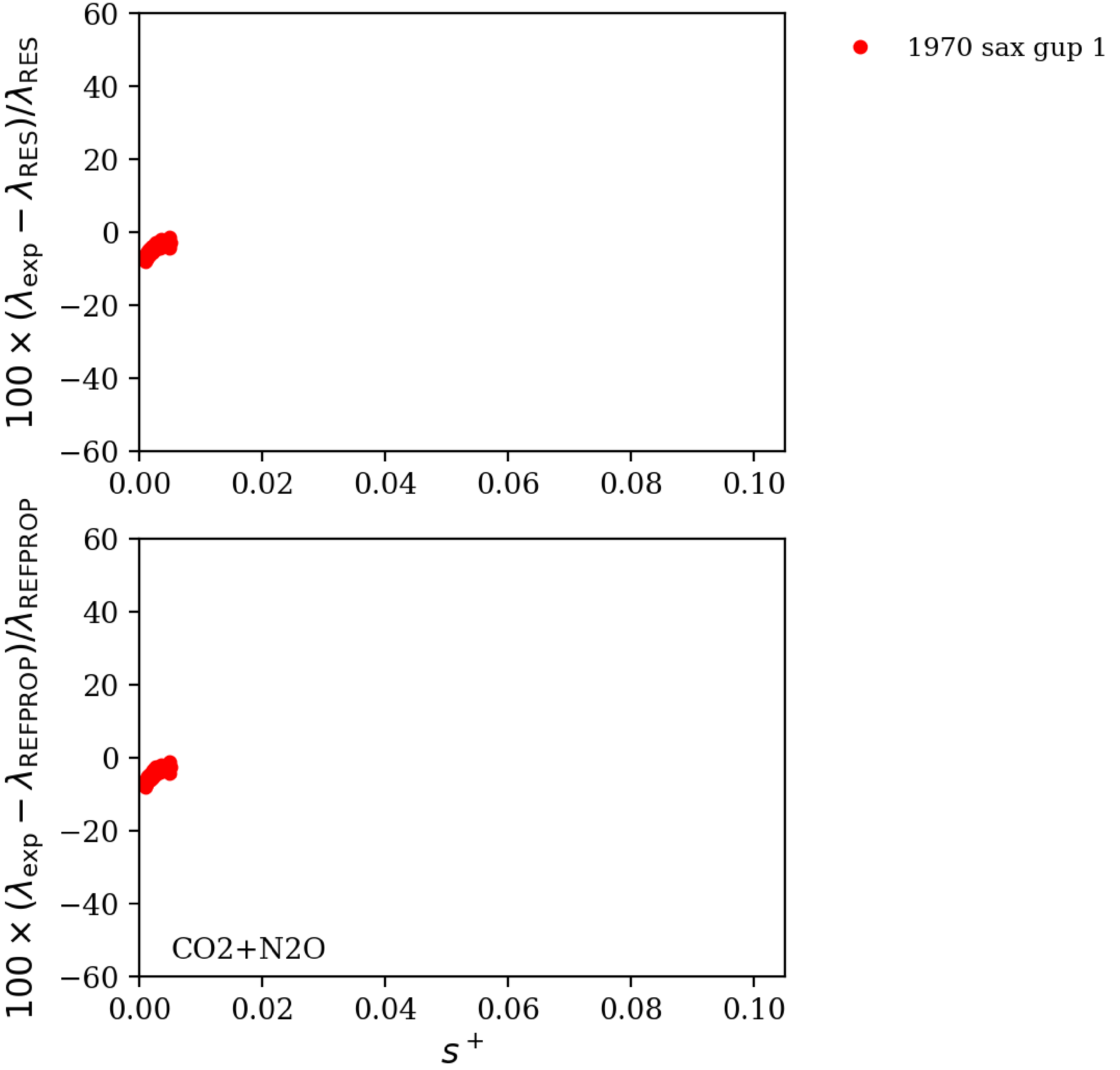

Figure DPR4. CO2+N2O

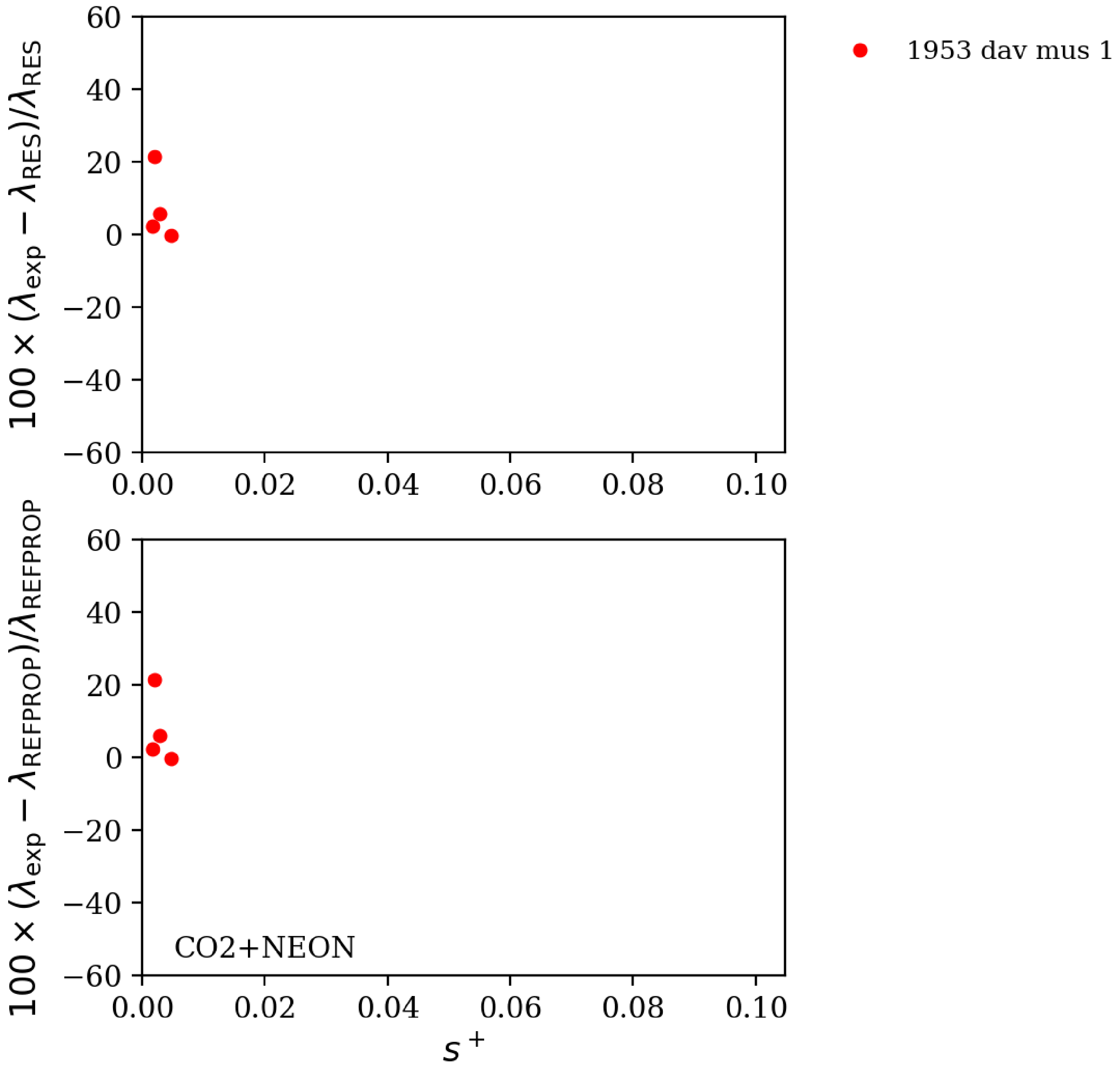

Figure DPR4. CO2+NEON

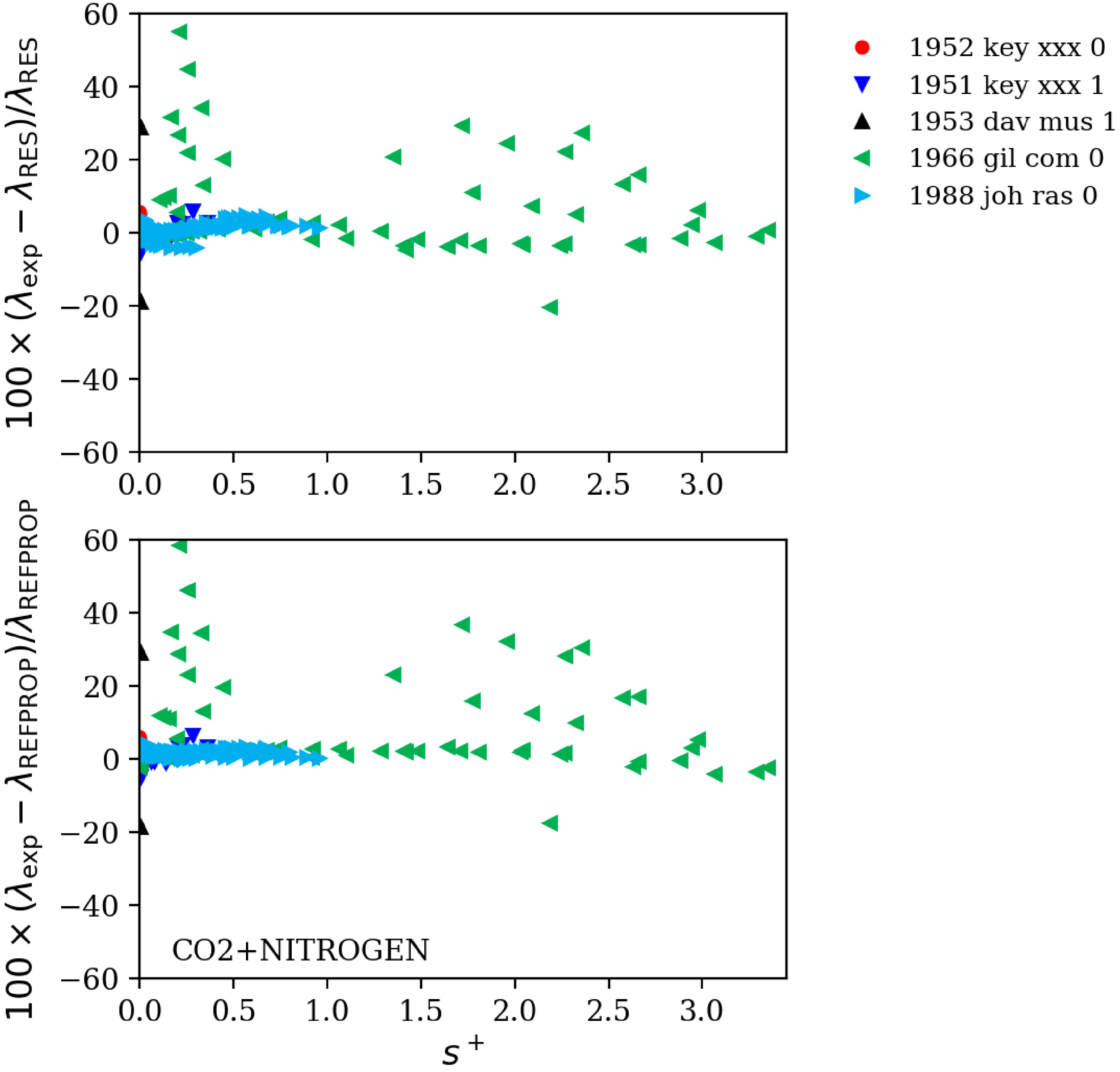

Figure DPR4. CO2+NITROGEN

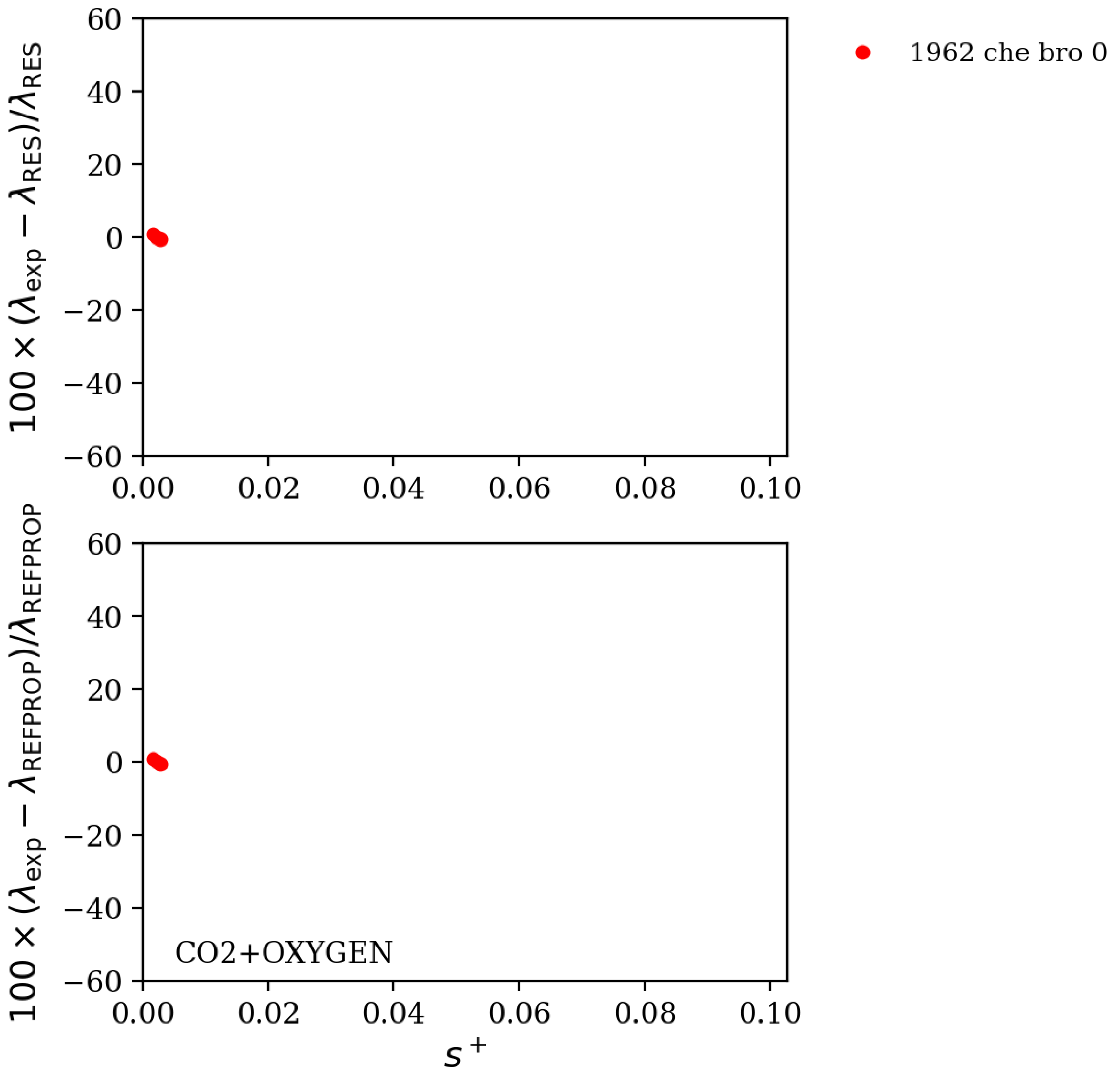

Figure DPR4. CO2+OXYGEN

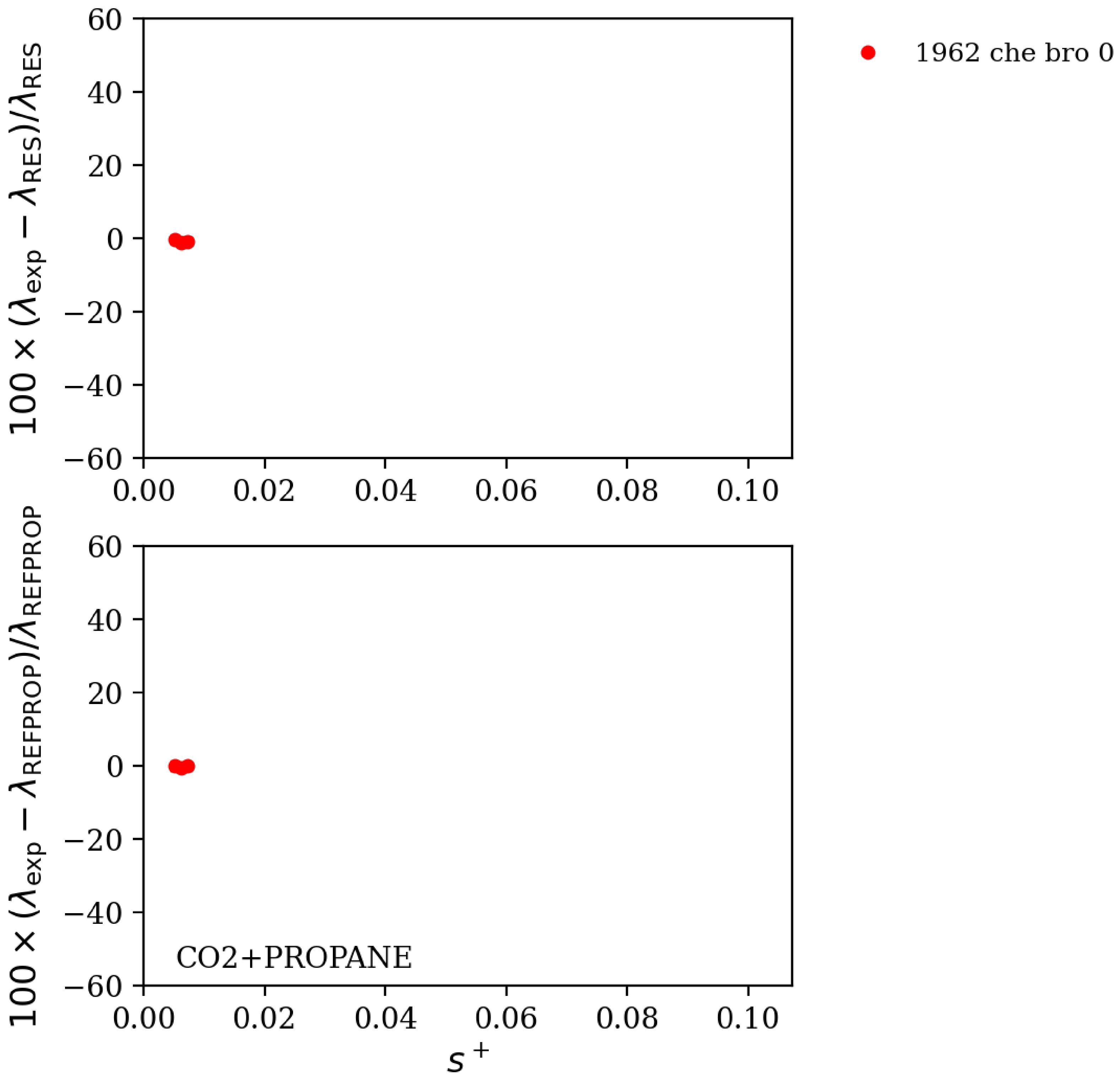

Figure DPR4. CO2+PROPANE

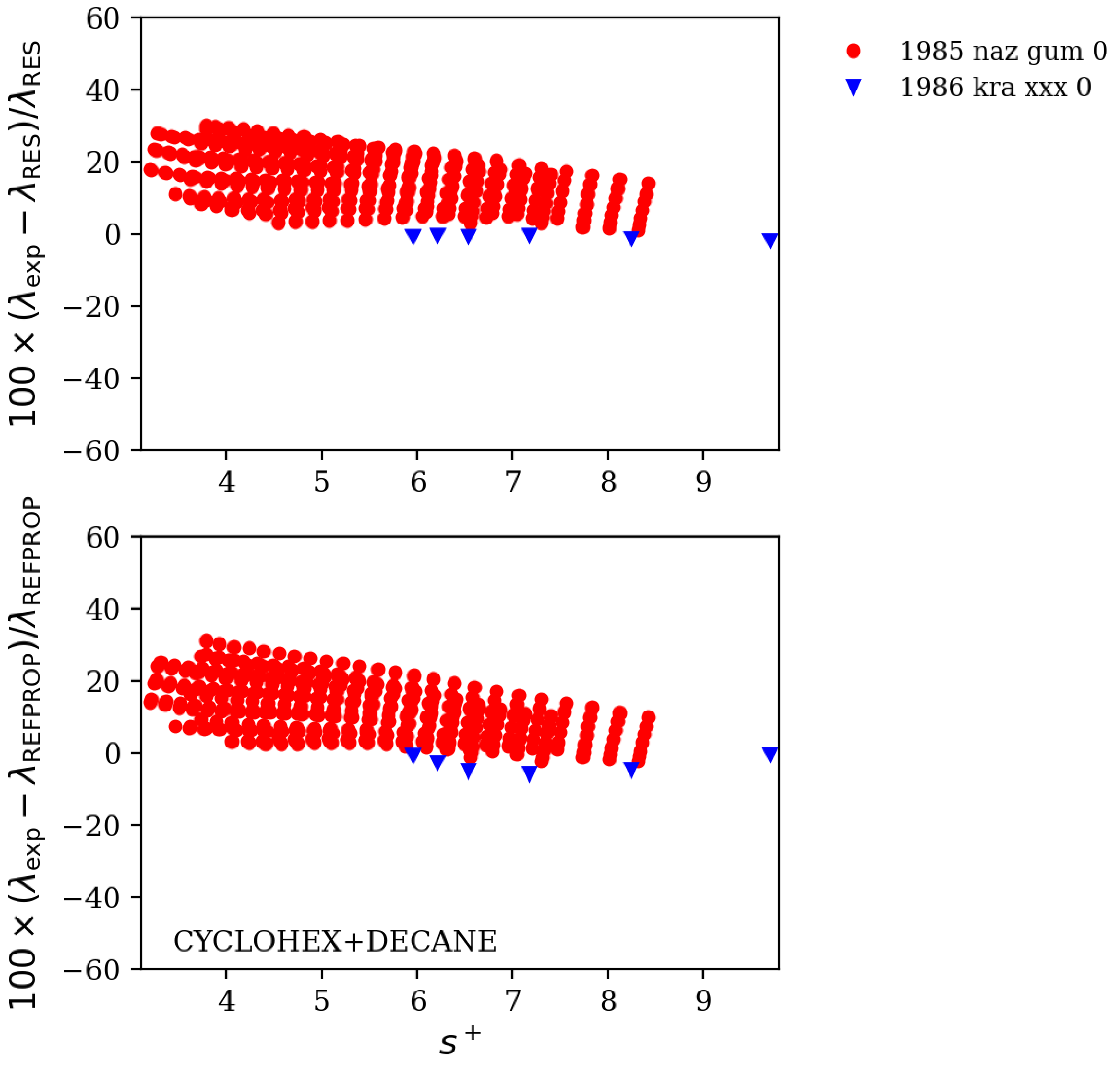

Figure DPR4. CYCLOHEX+DECANE

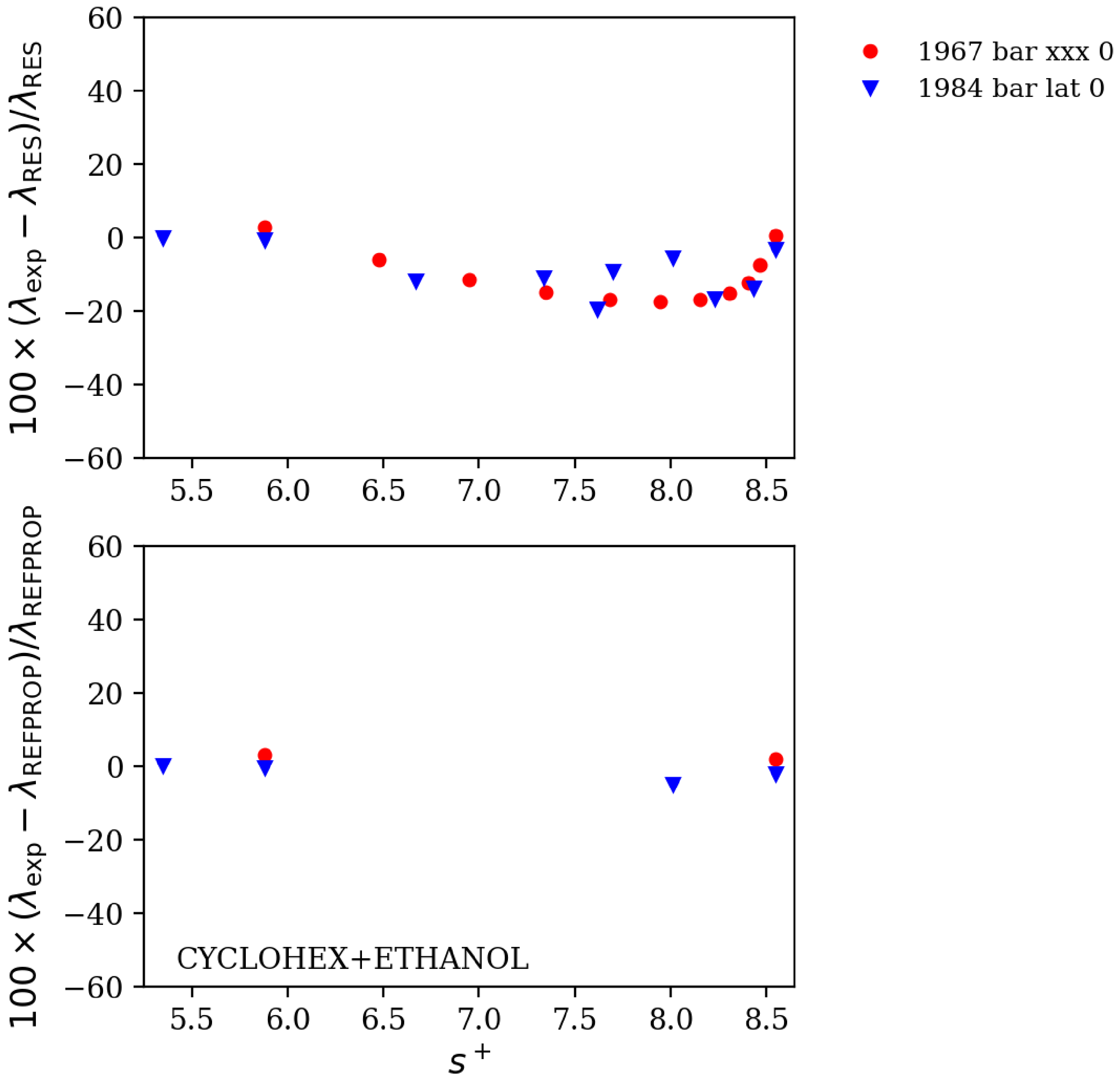

Figure DPR4. CYCLOHEX+ETHANOL

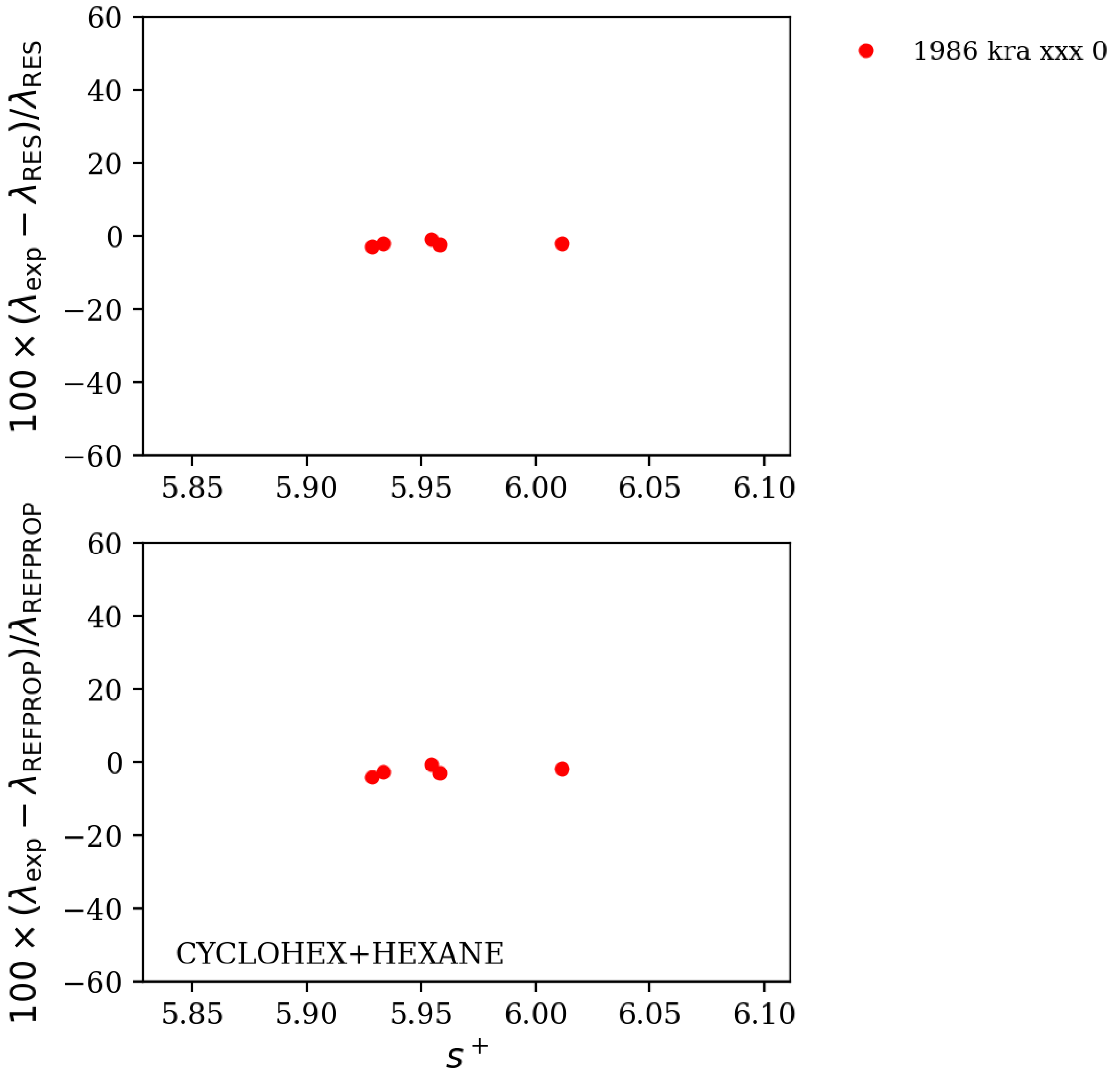

Figure DPR4. CYCLOHEX+HEXANE

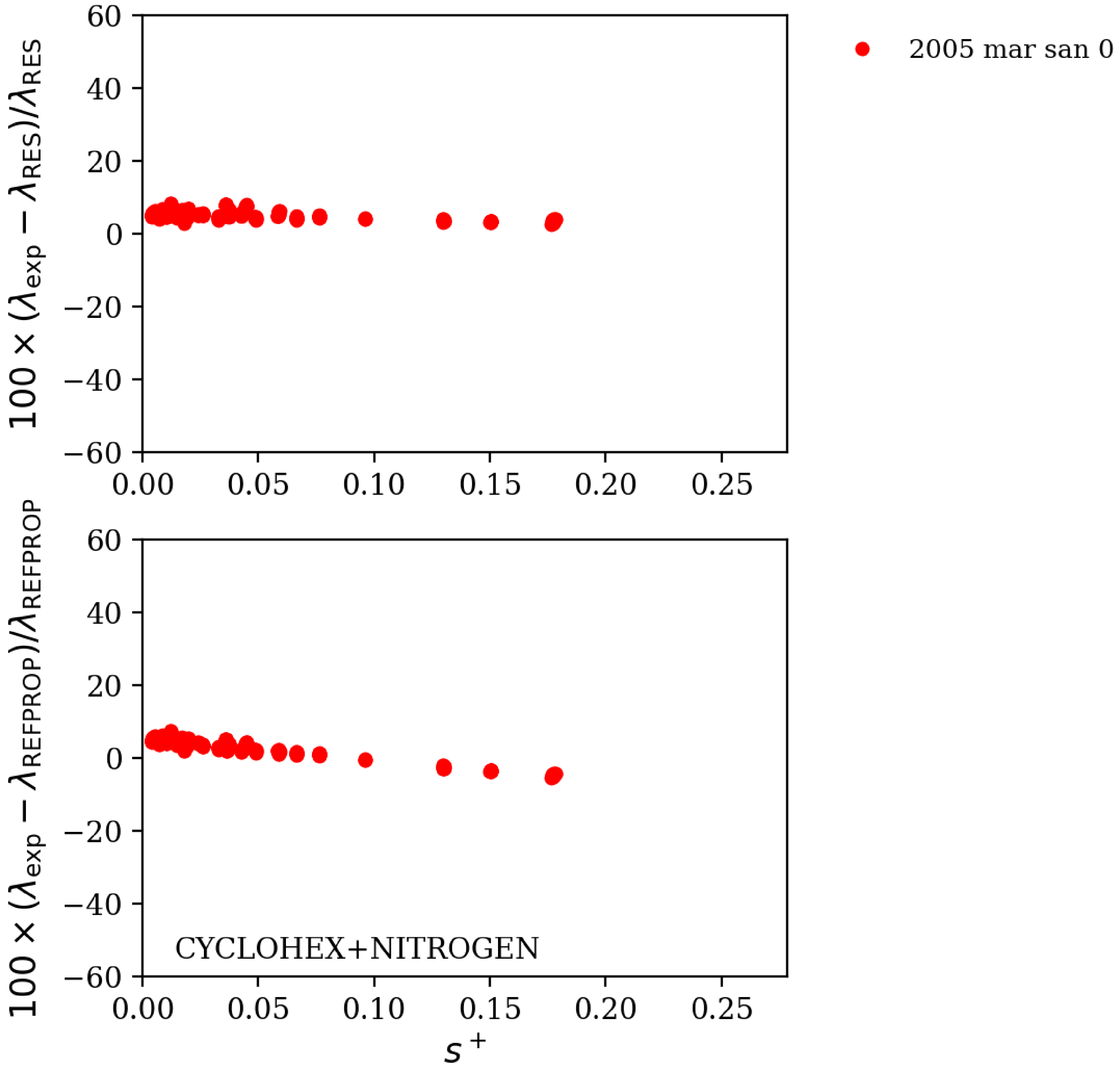

Figure DPR4. CYCLOHEX+NITROGEN

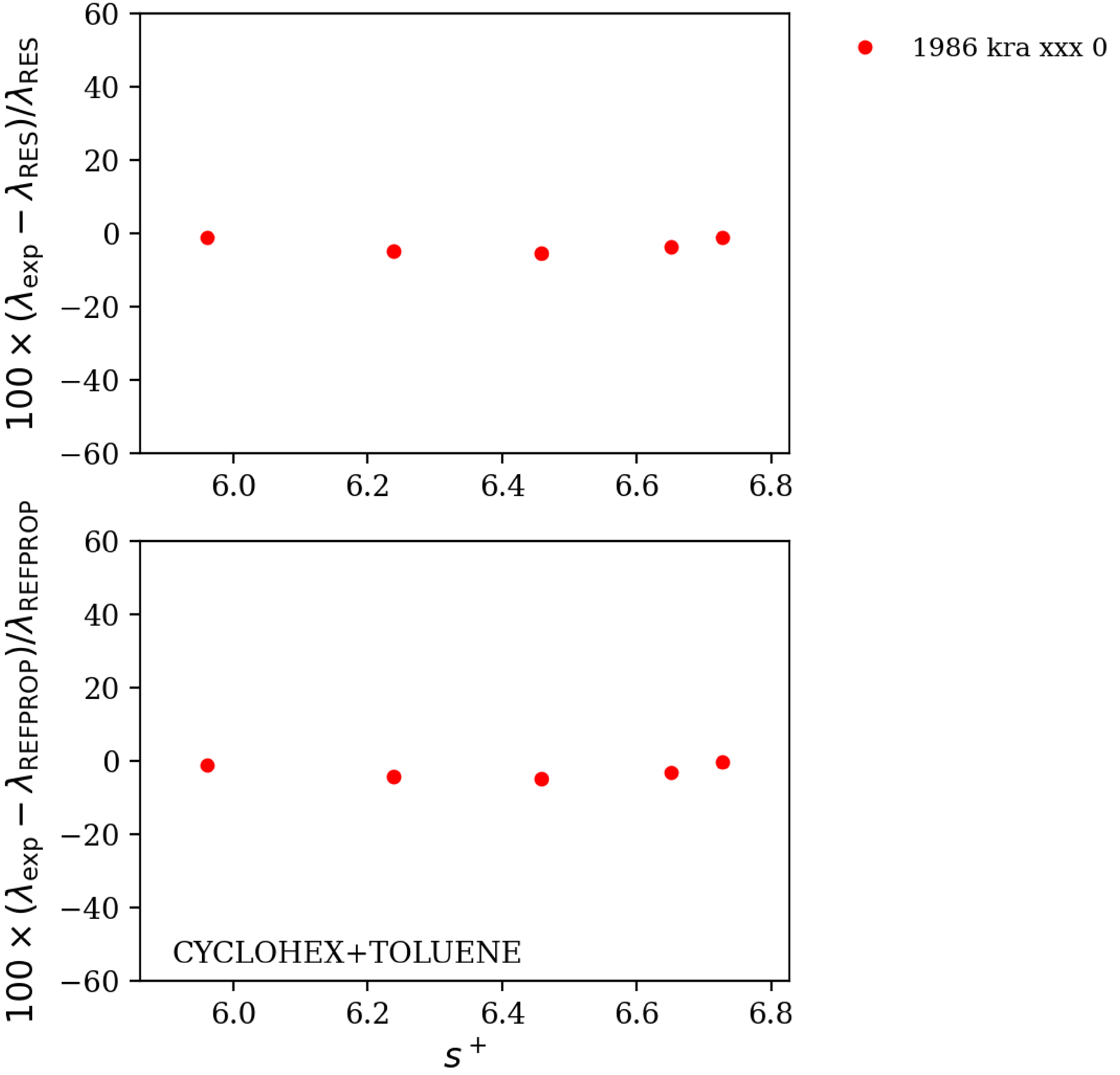

Figure DPR4. CYCLOHEX+TOLUENE

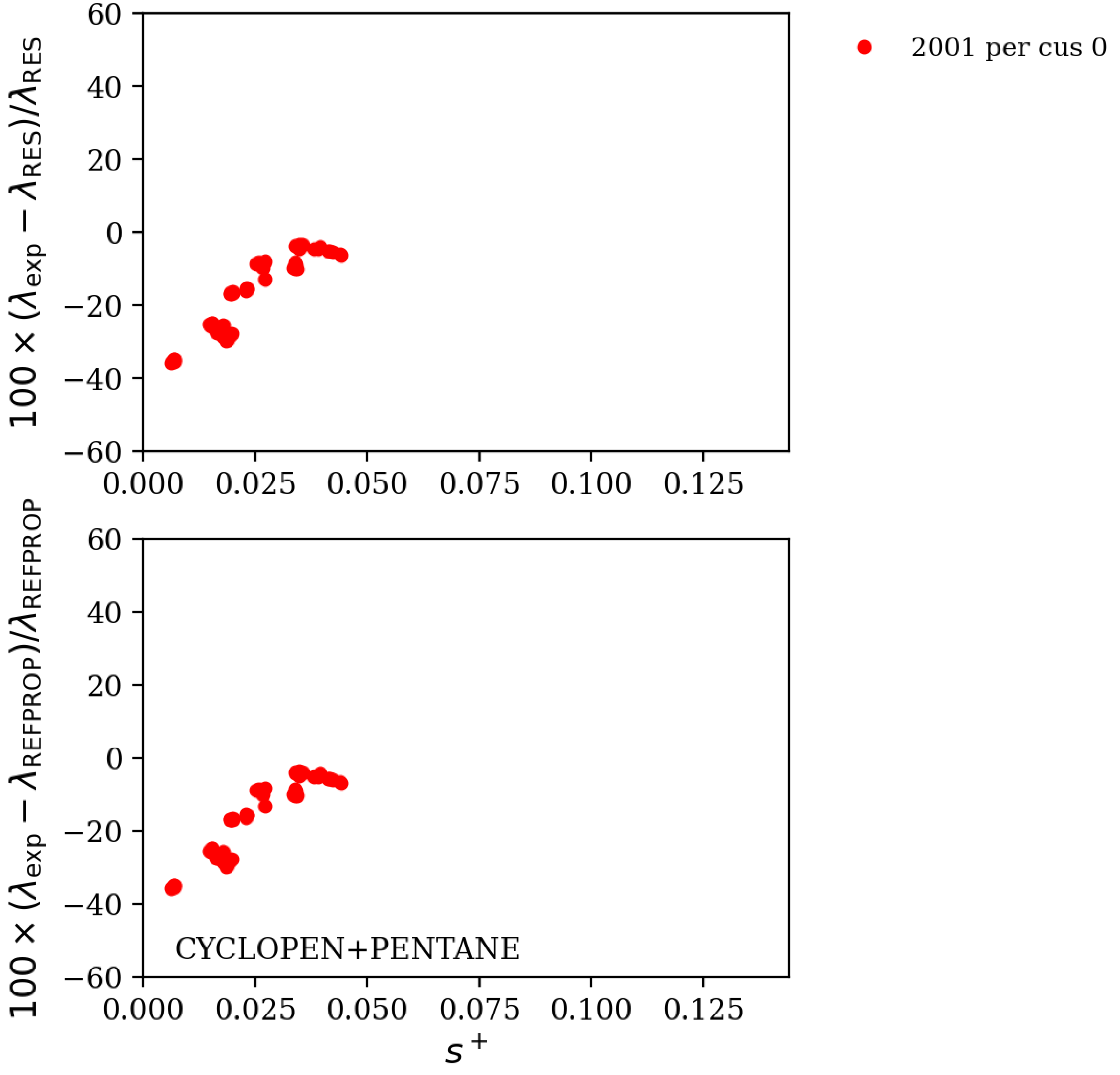

Figure DPR4. CYCLOPEN+PENTANE

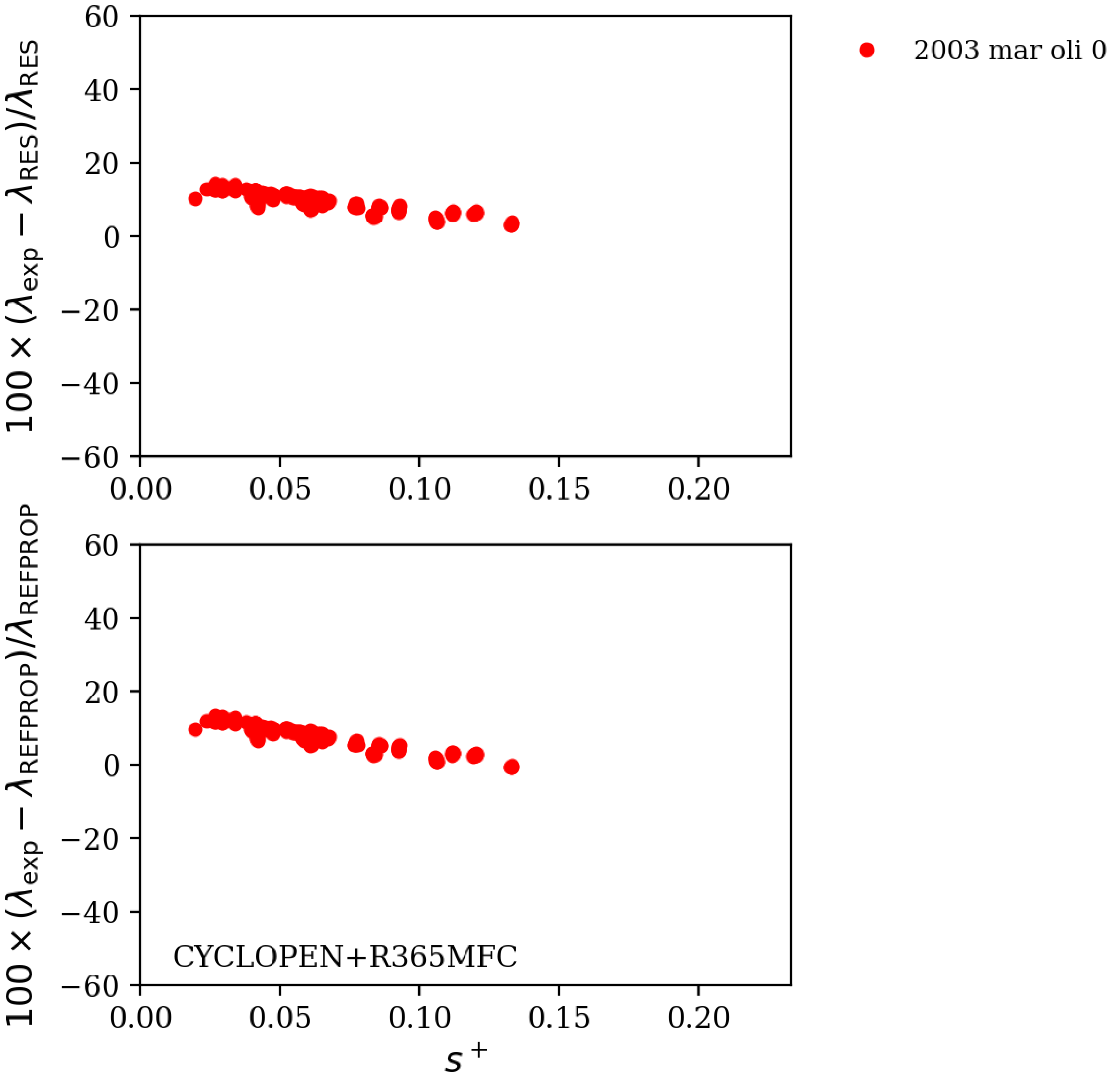

Figure DPR4. CYCLOPEN+R365MFC

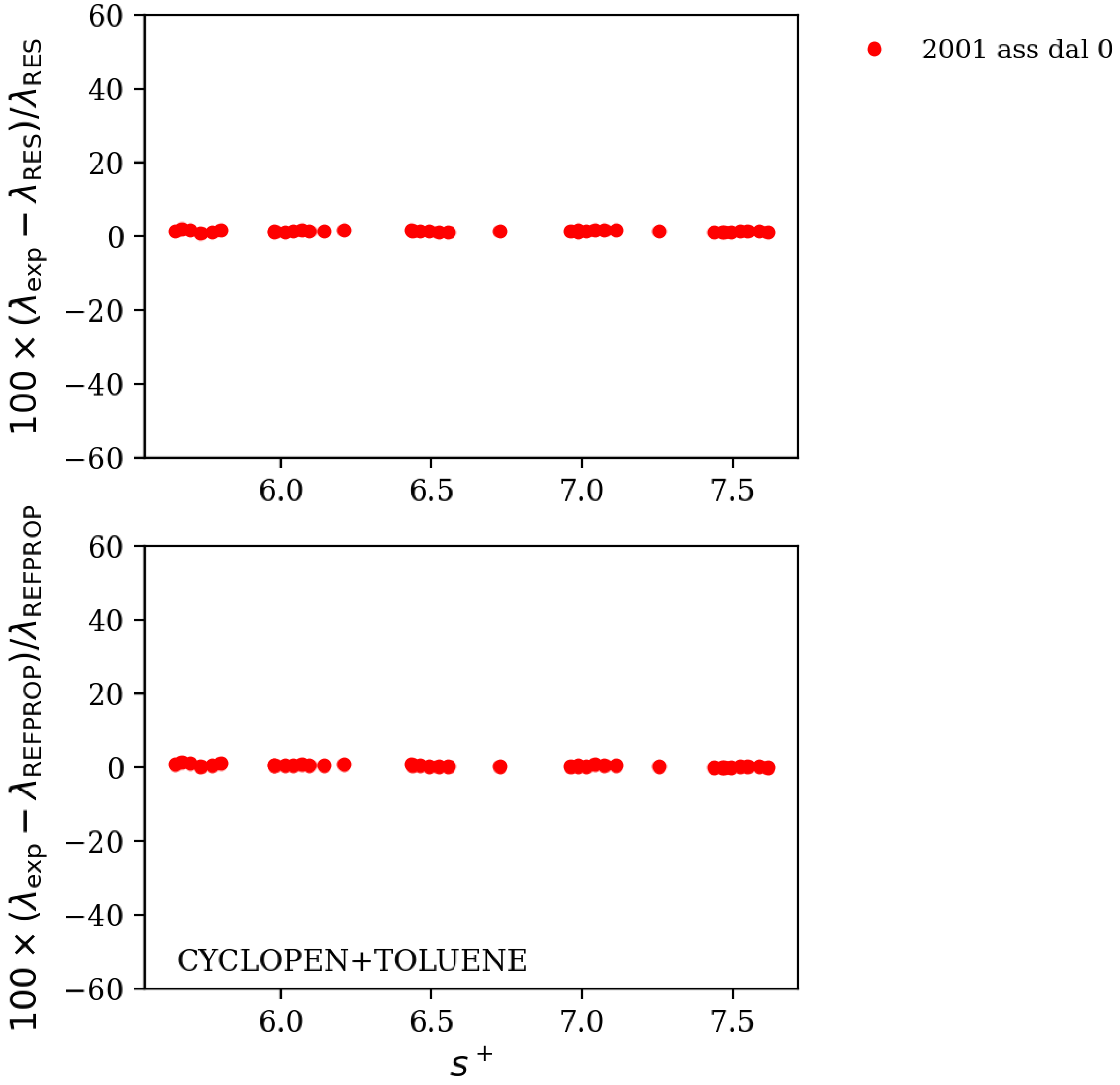

Figure DPR4. CYCLOPEN+TOLUENE

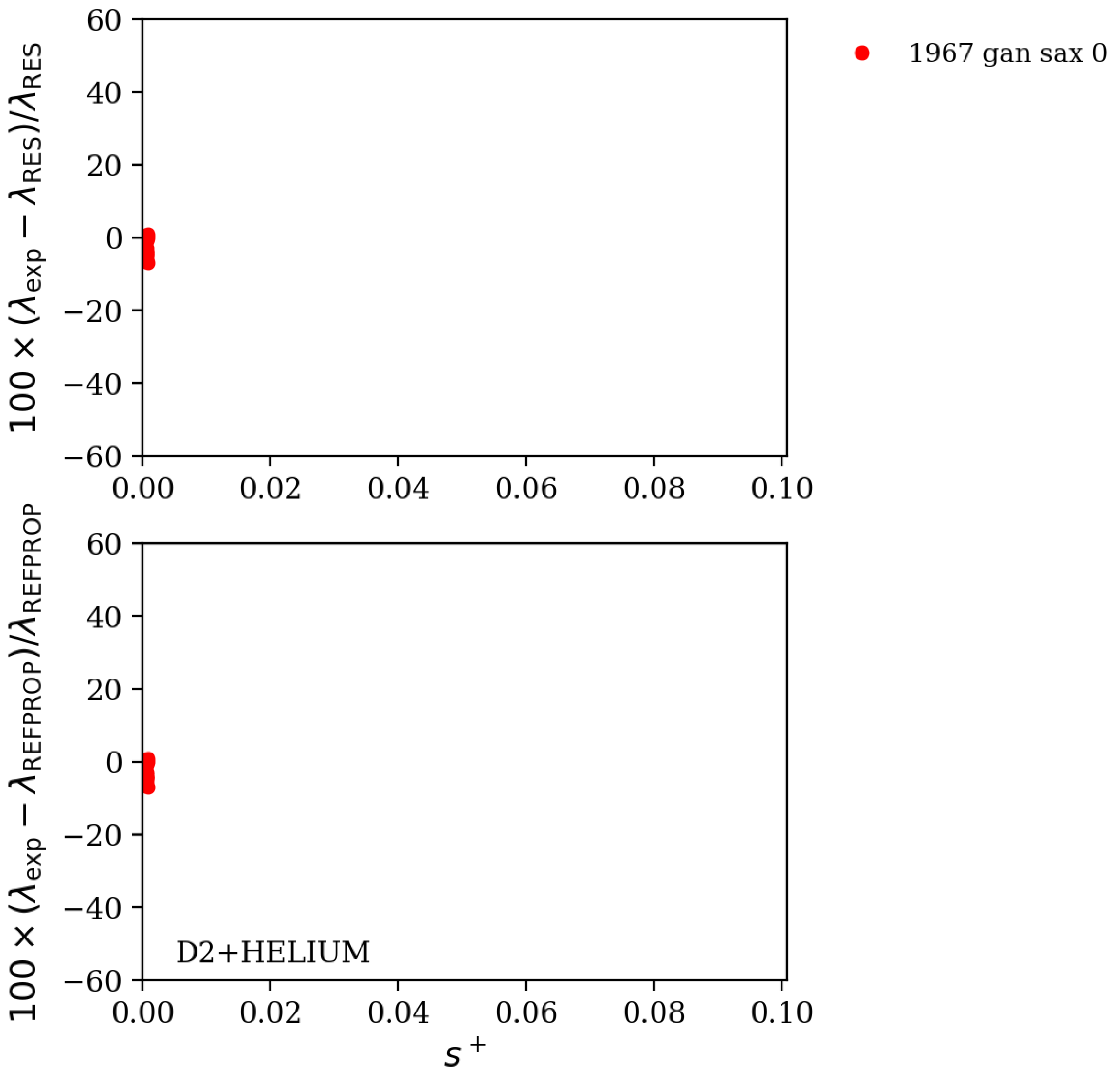

Figure DPR4. D2+HELIUM

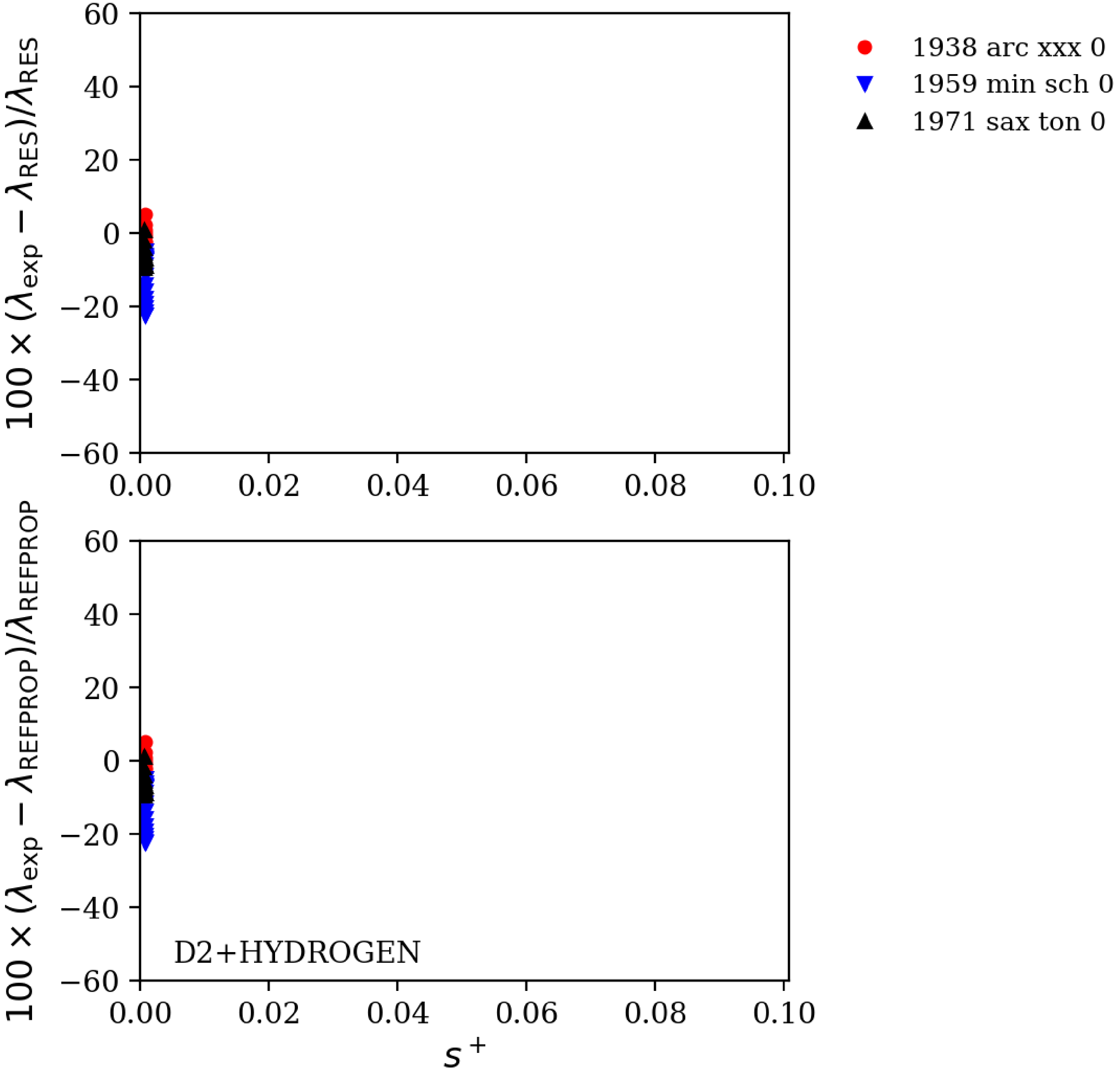

Figure DPR4. D2+HYDROGEN

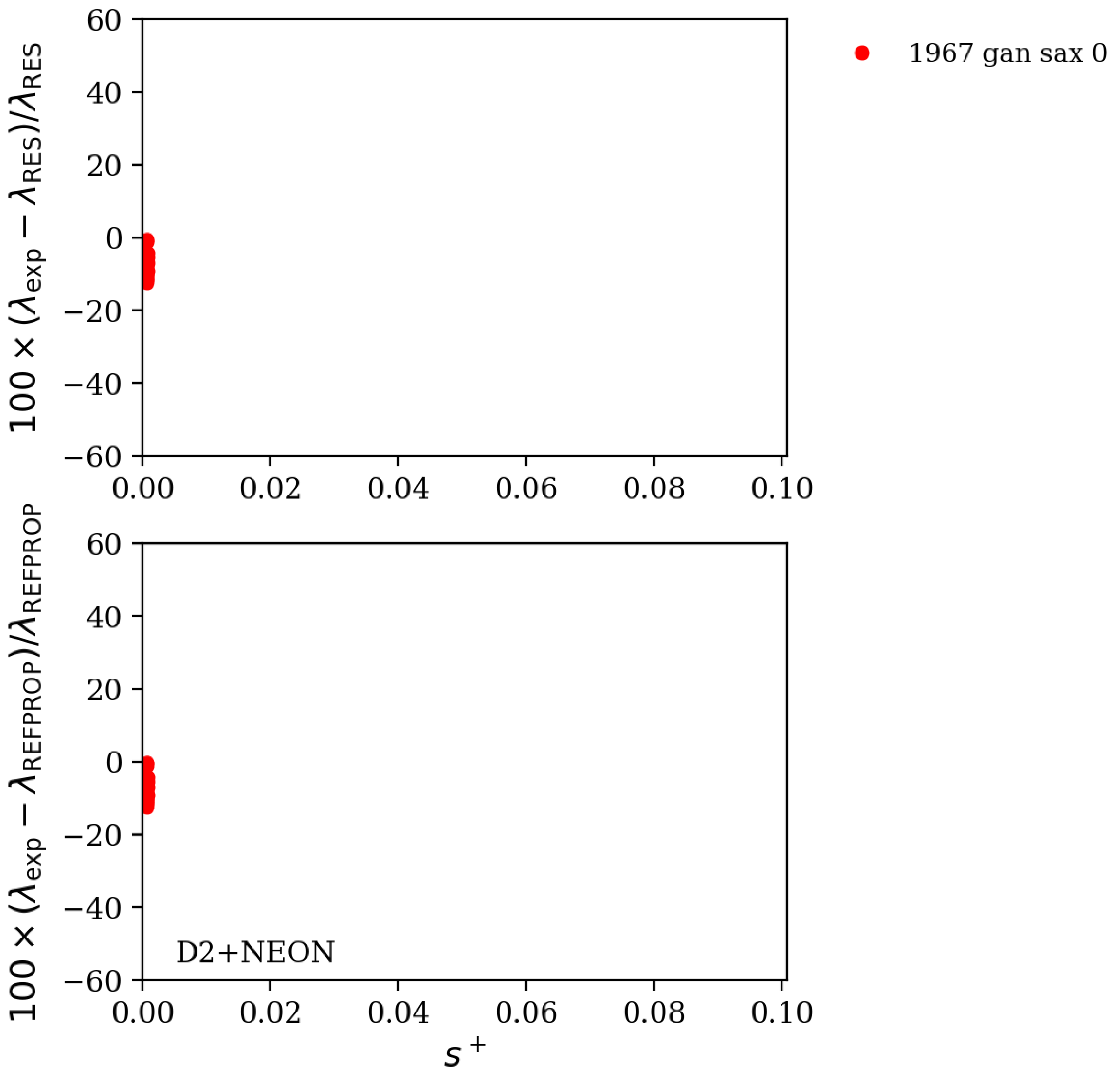

Figure DPR4. D2+NEON

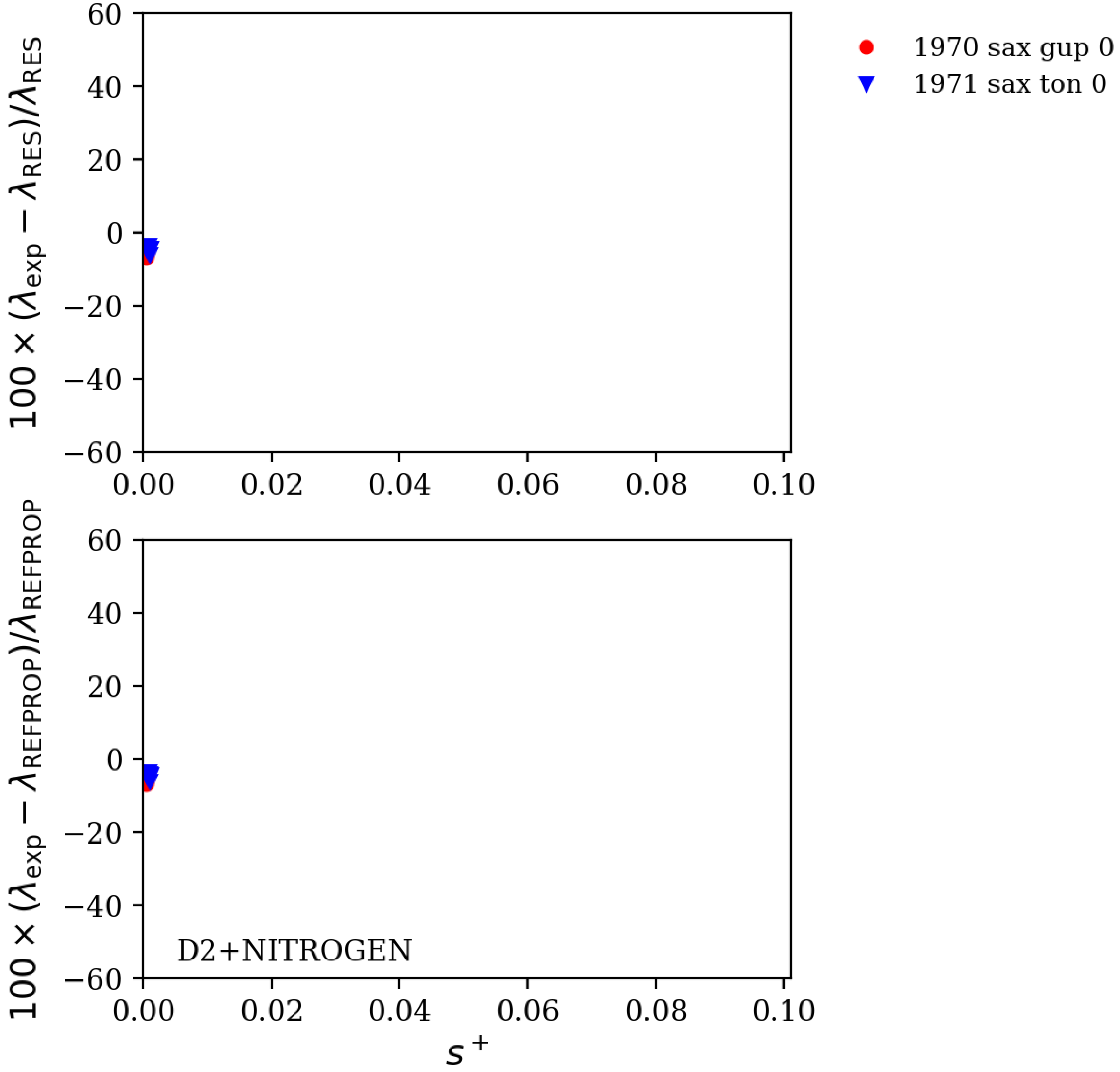

Figure DPR4. D2+NITROGEN

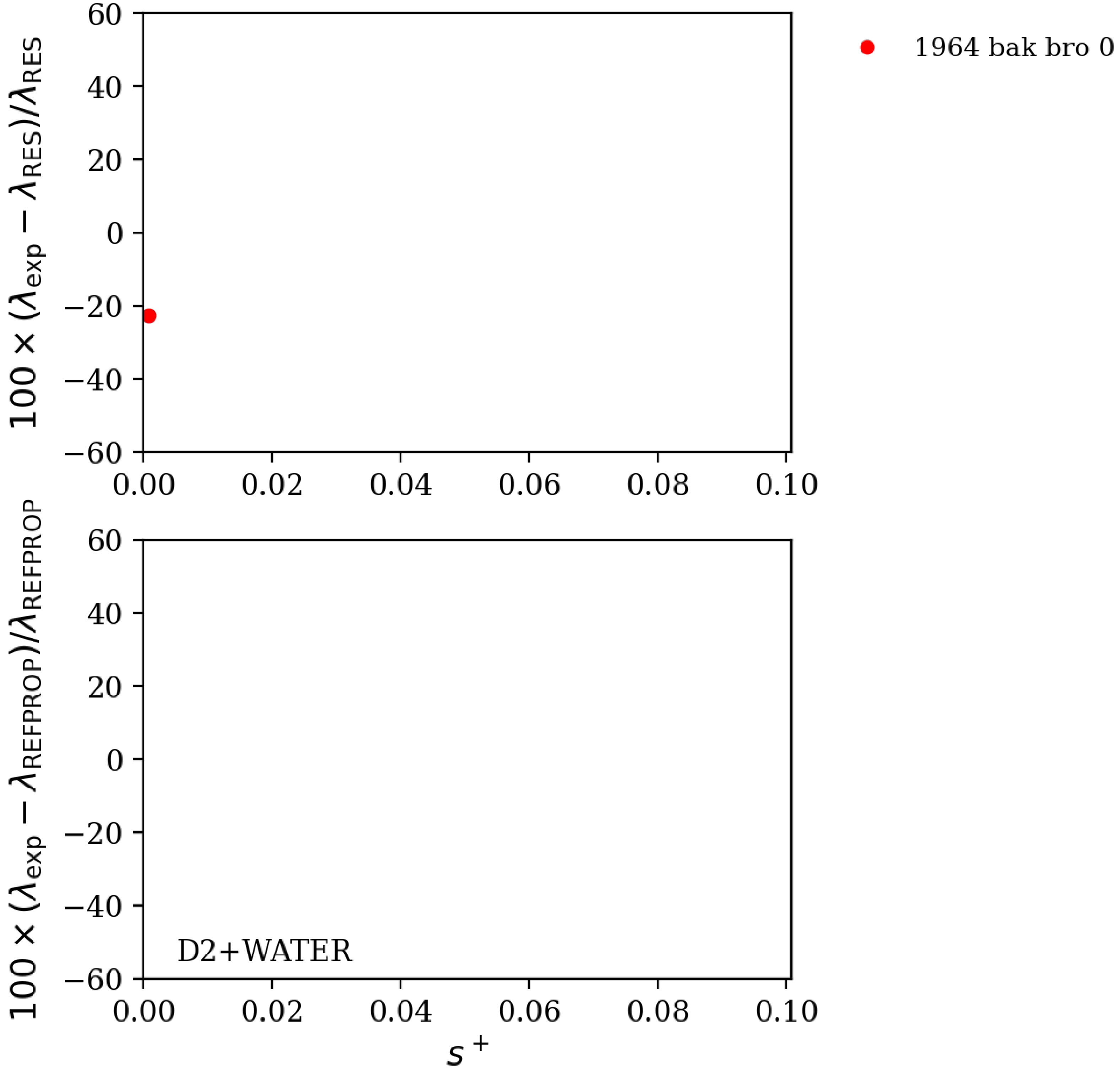

Figure DPR4. D2+WATER

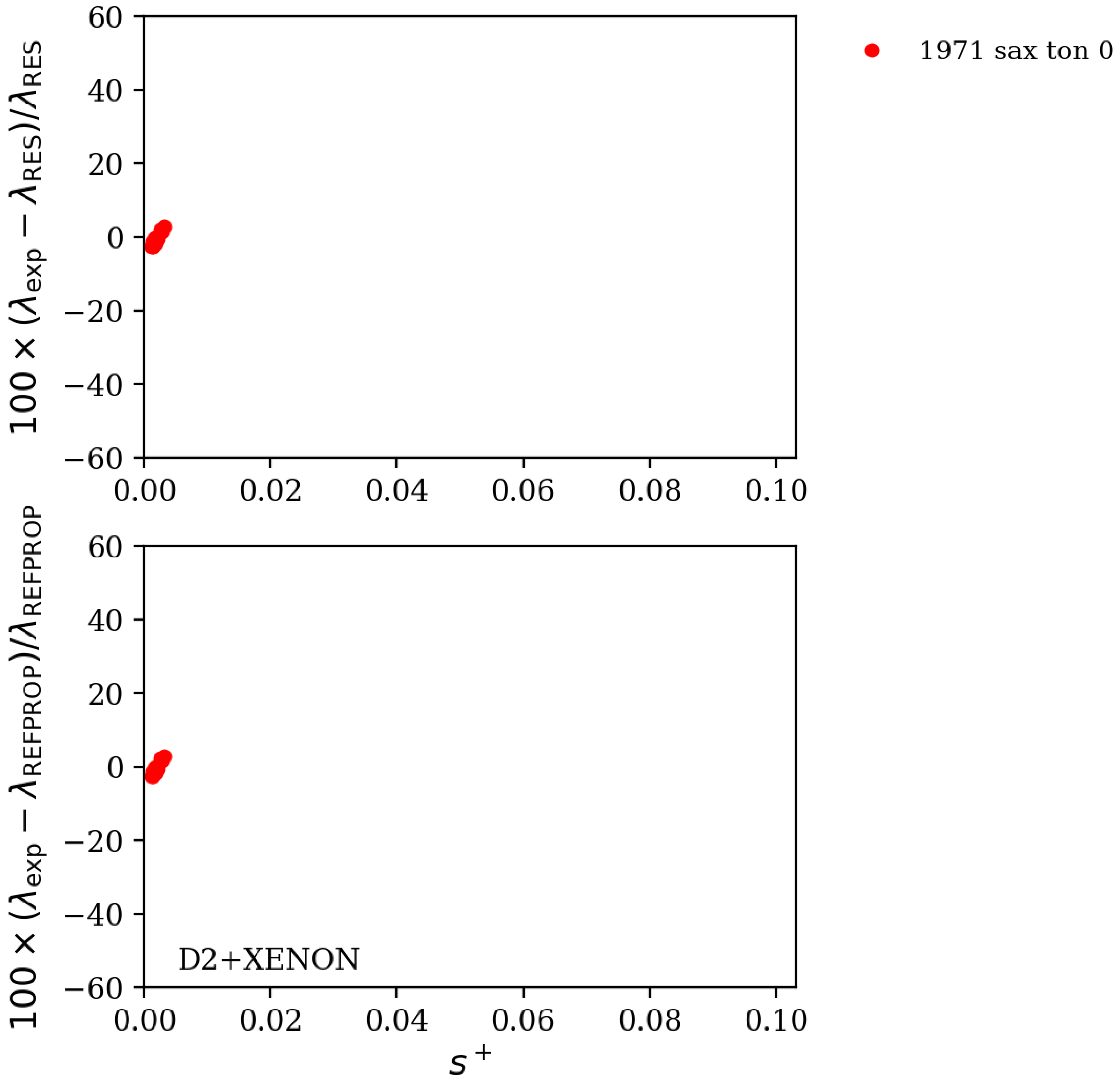

Figure DPR4. D2+XENON

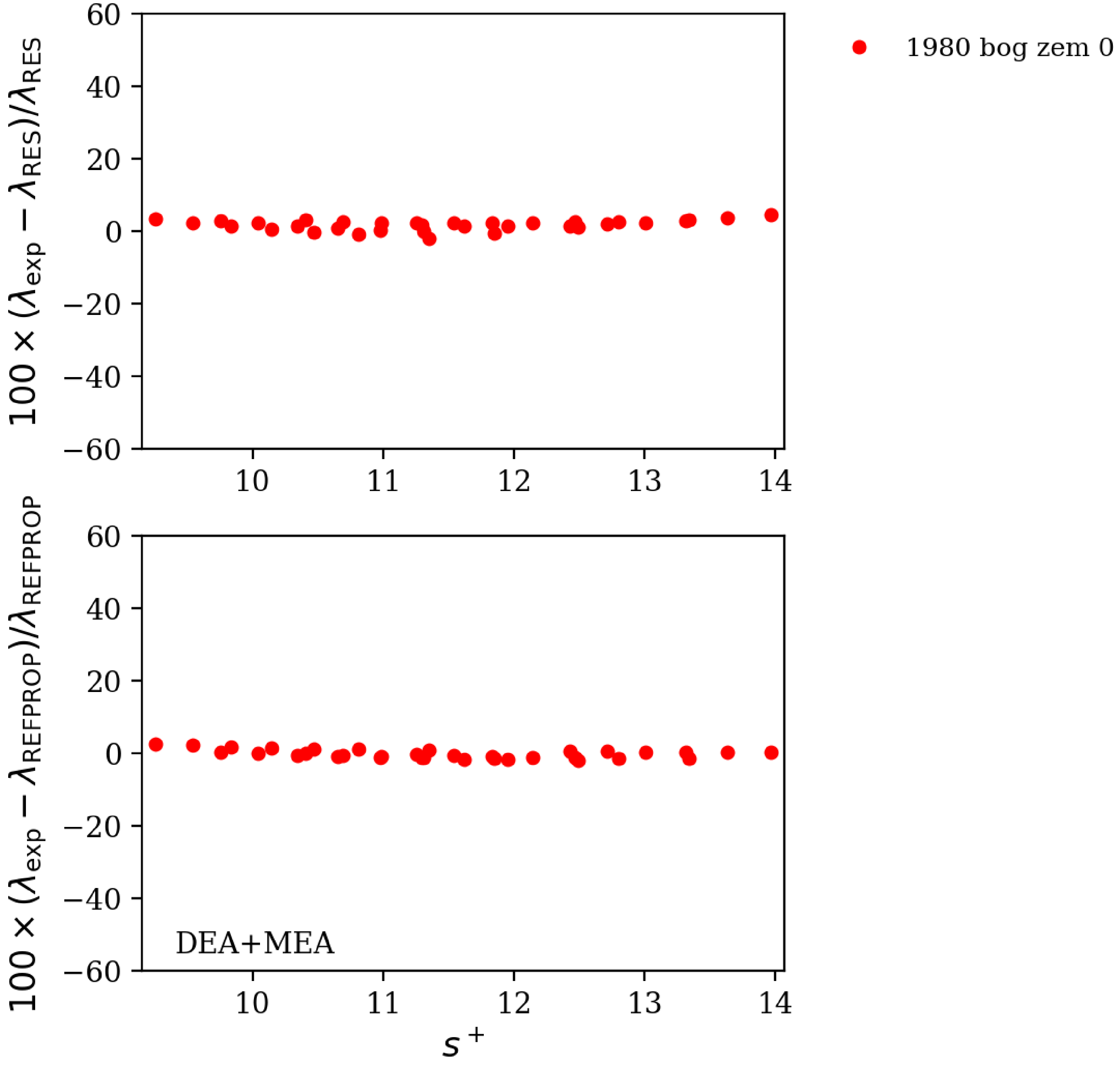

Figure DPR4. DEA+MEA

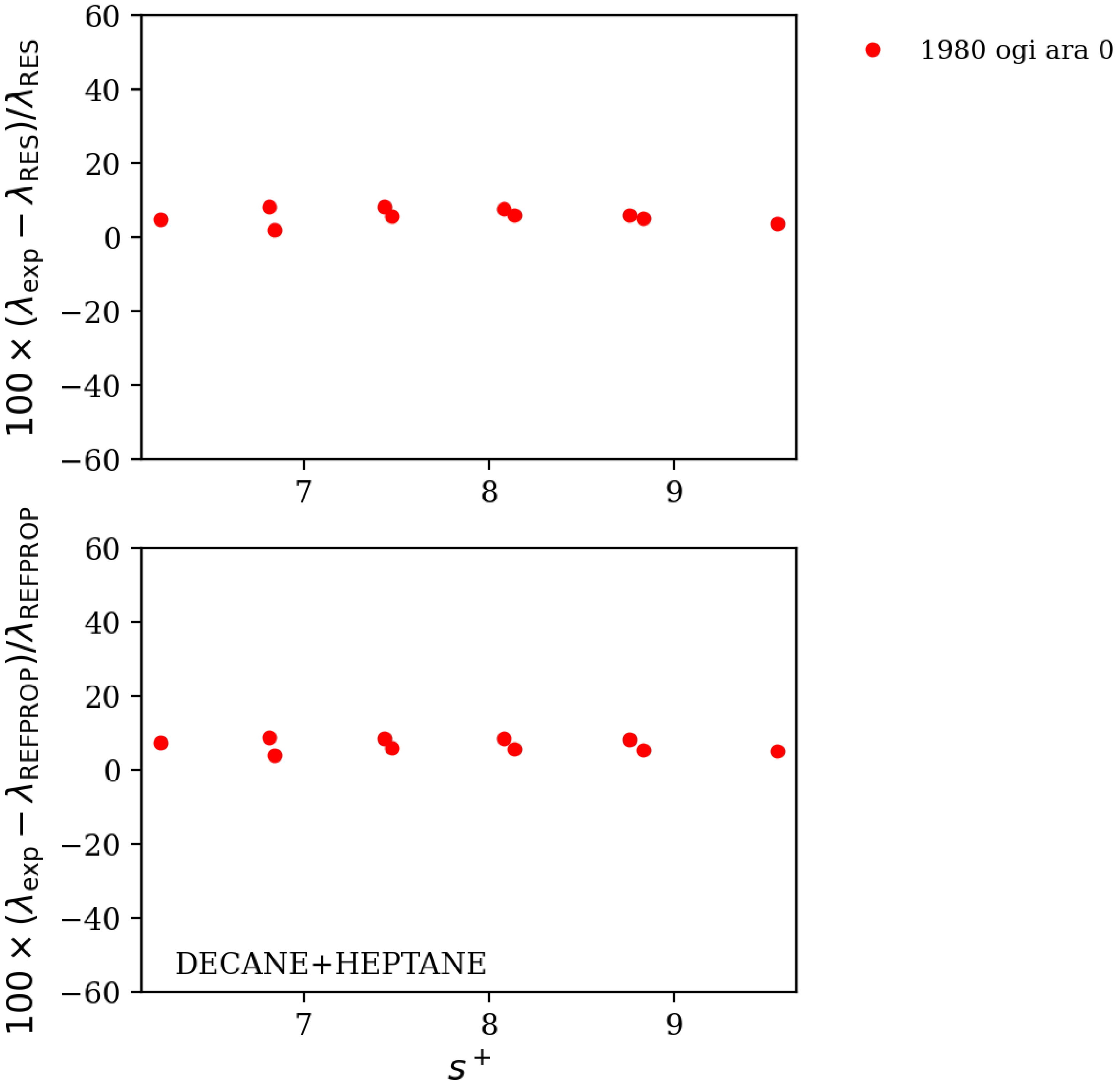

Figure DPR4. DECANE+HEPTANE

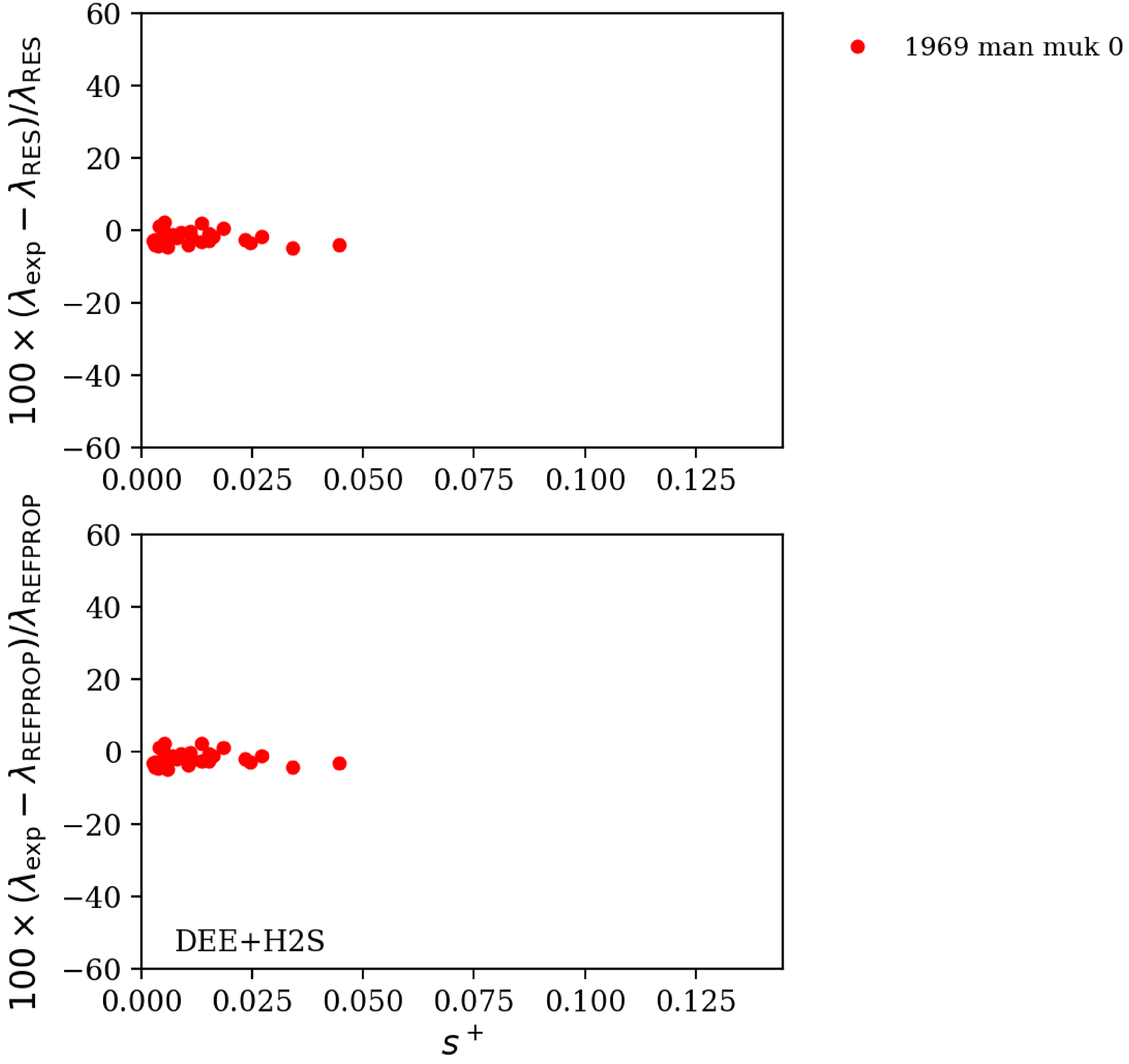

Figure DPR4. DEE+H2S

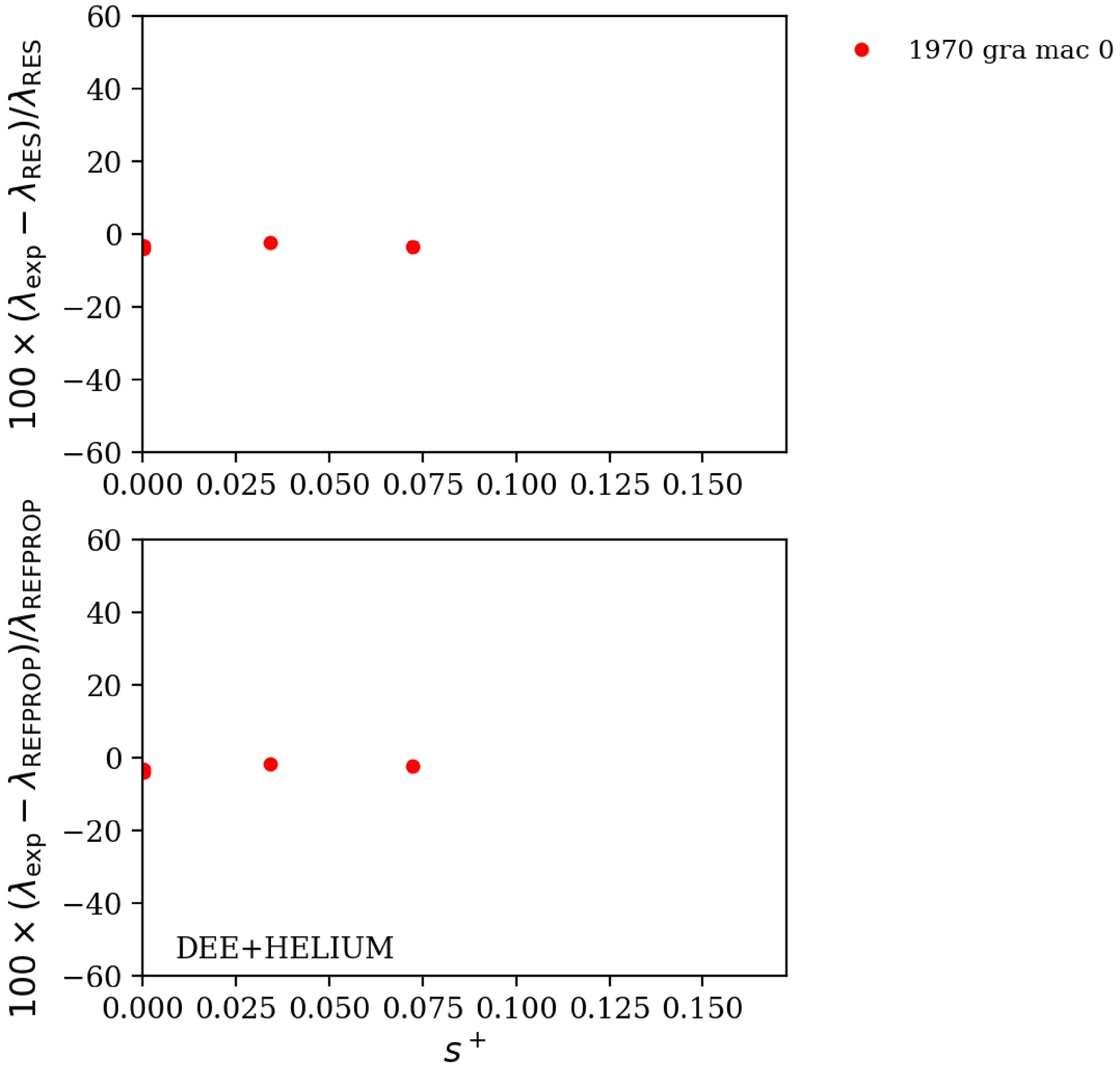

Figure DPR4. DEE+HELIUM

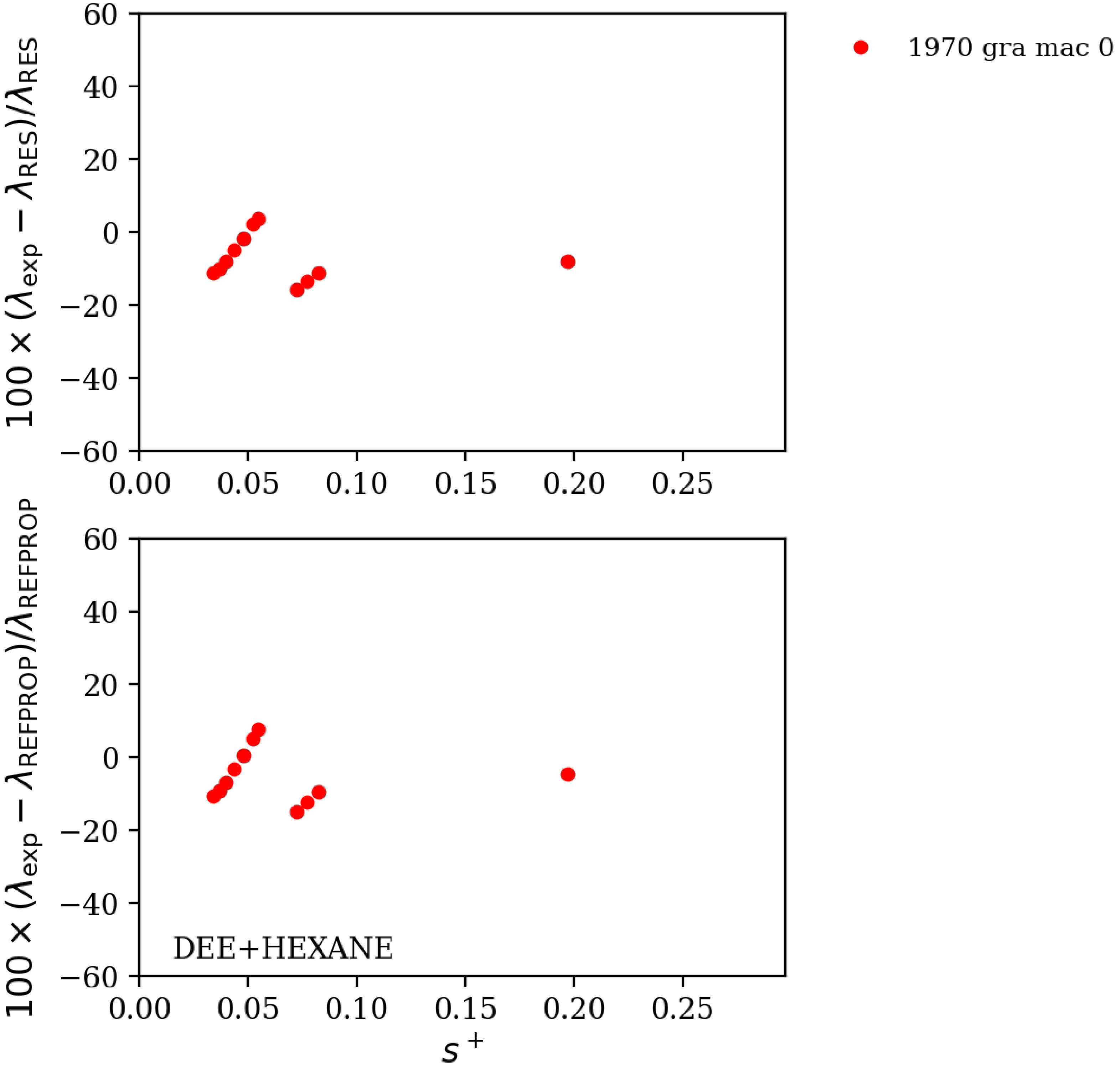

Figure DPR4. DEE+HEXANE

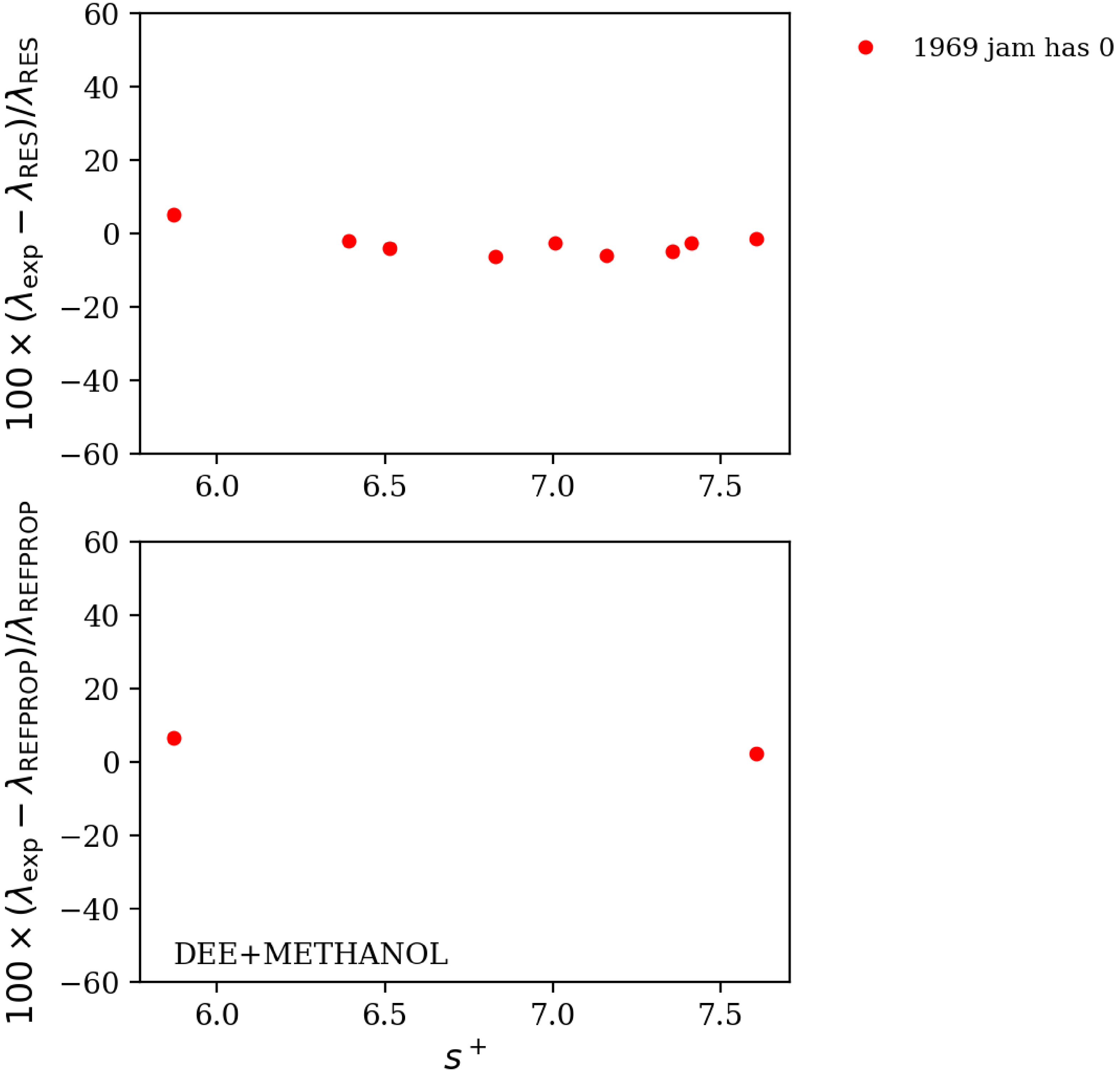

Figure DPR4. DEE+METHANOL

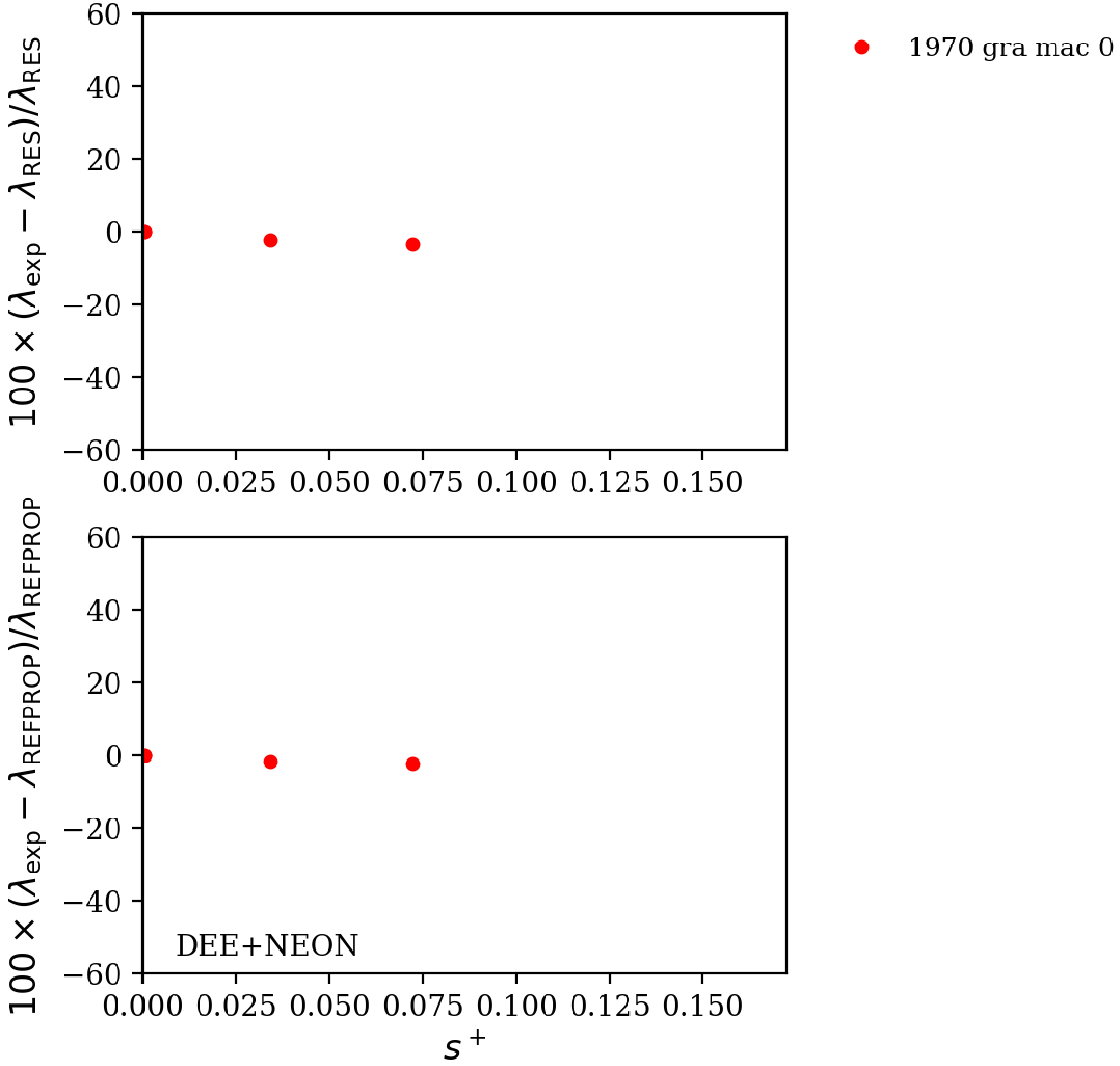

Figure DPR4. DEE+NEON

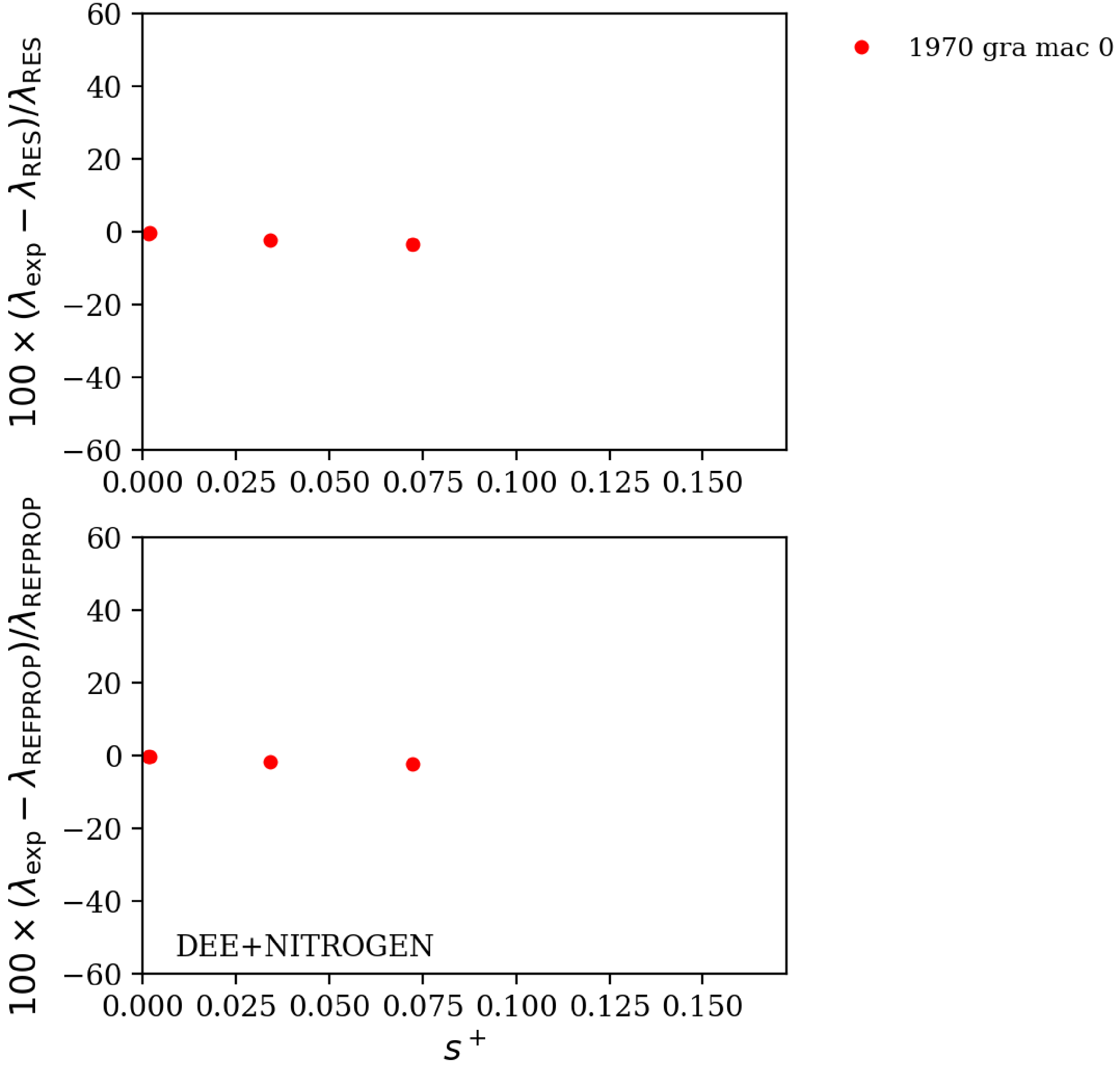

Figure DPR4. DEE+NITROGEN

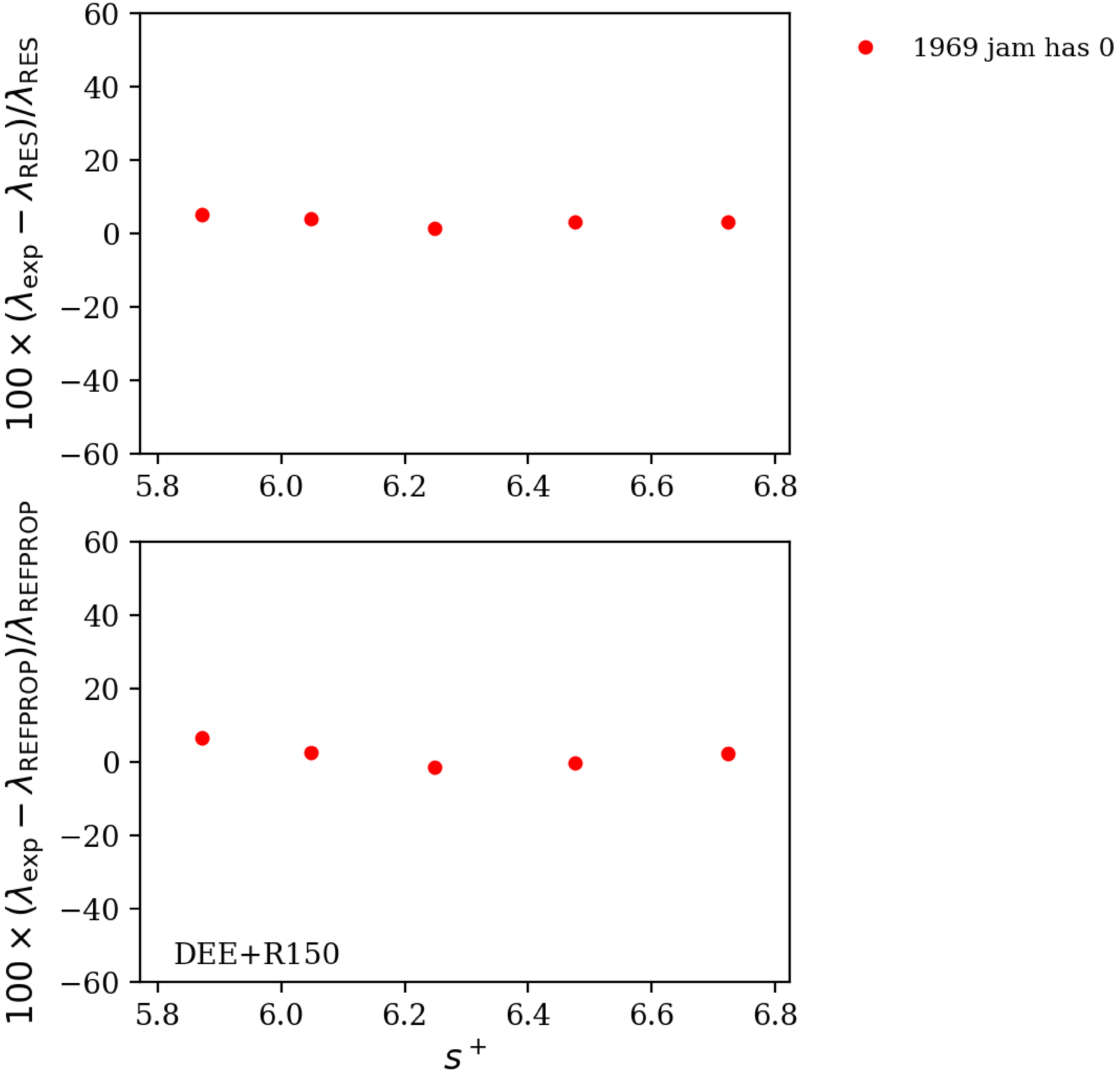

Figure DPR4. DEE+R150

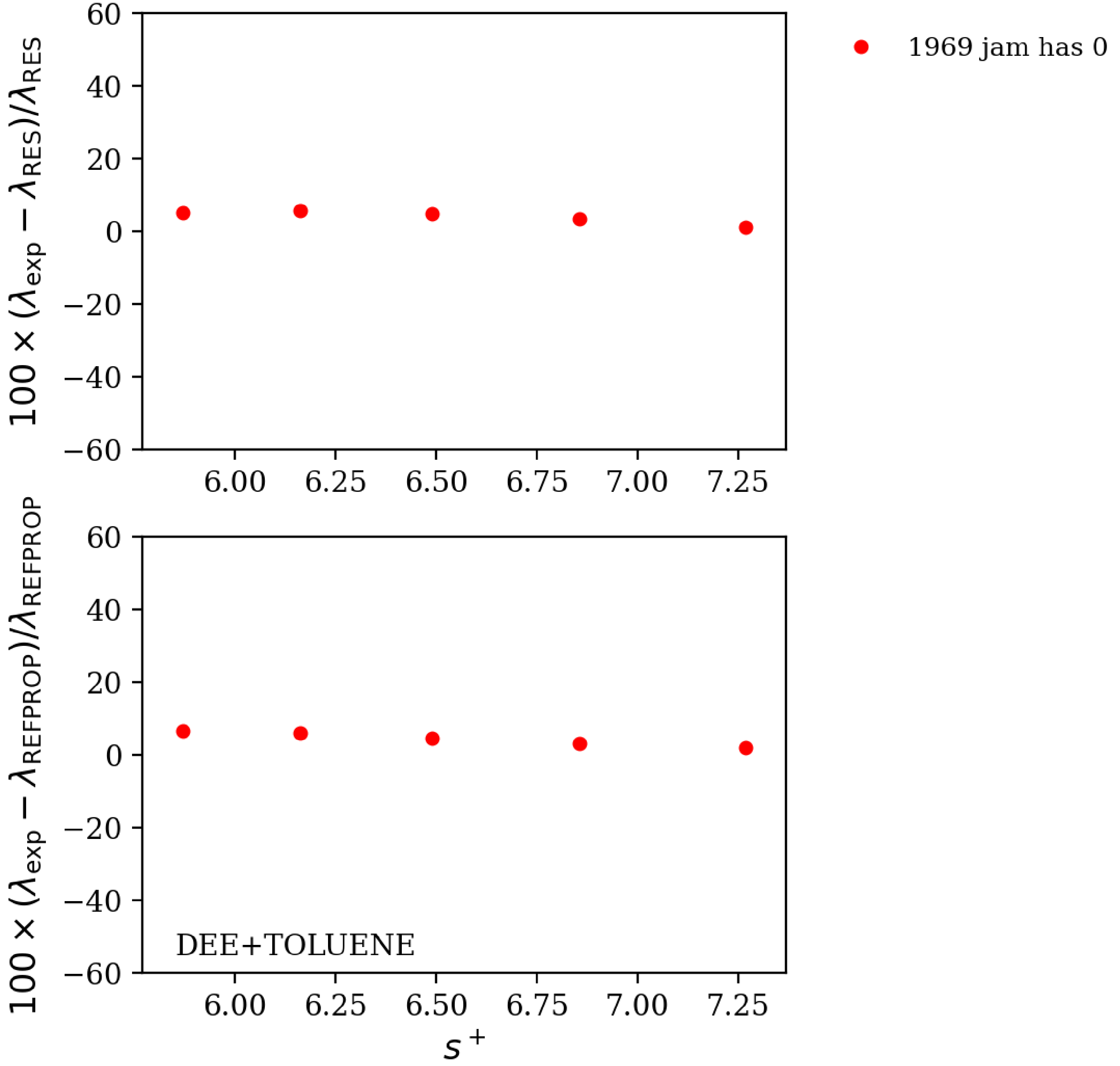

Figure DPR4. DEE+TOLUENE

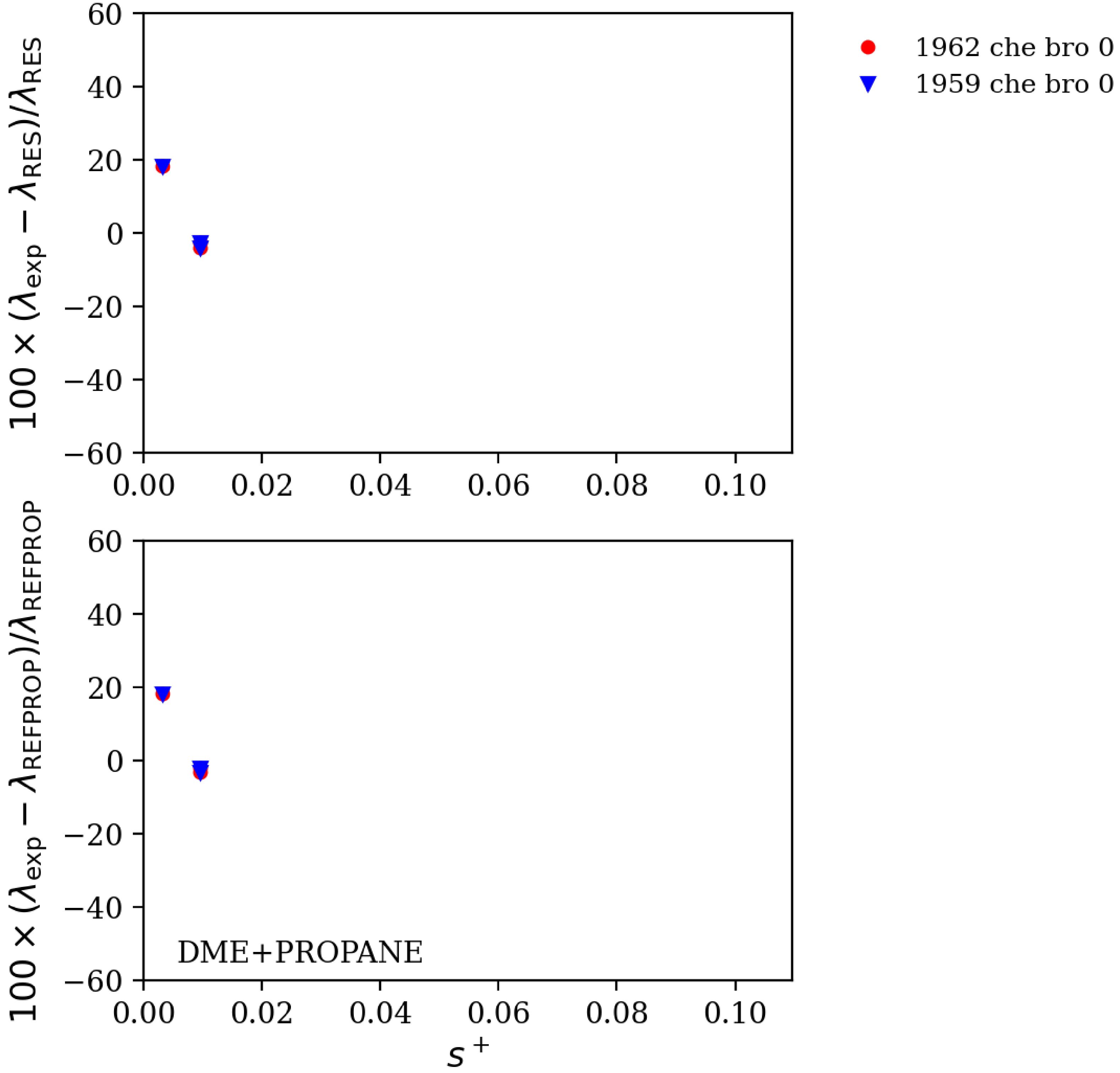

Figure DPR4. DME+PROPANE

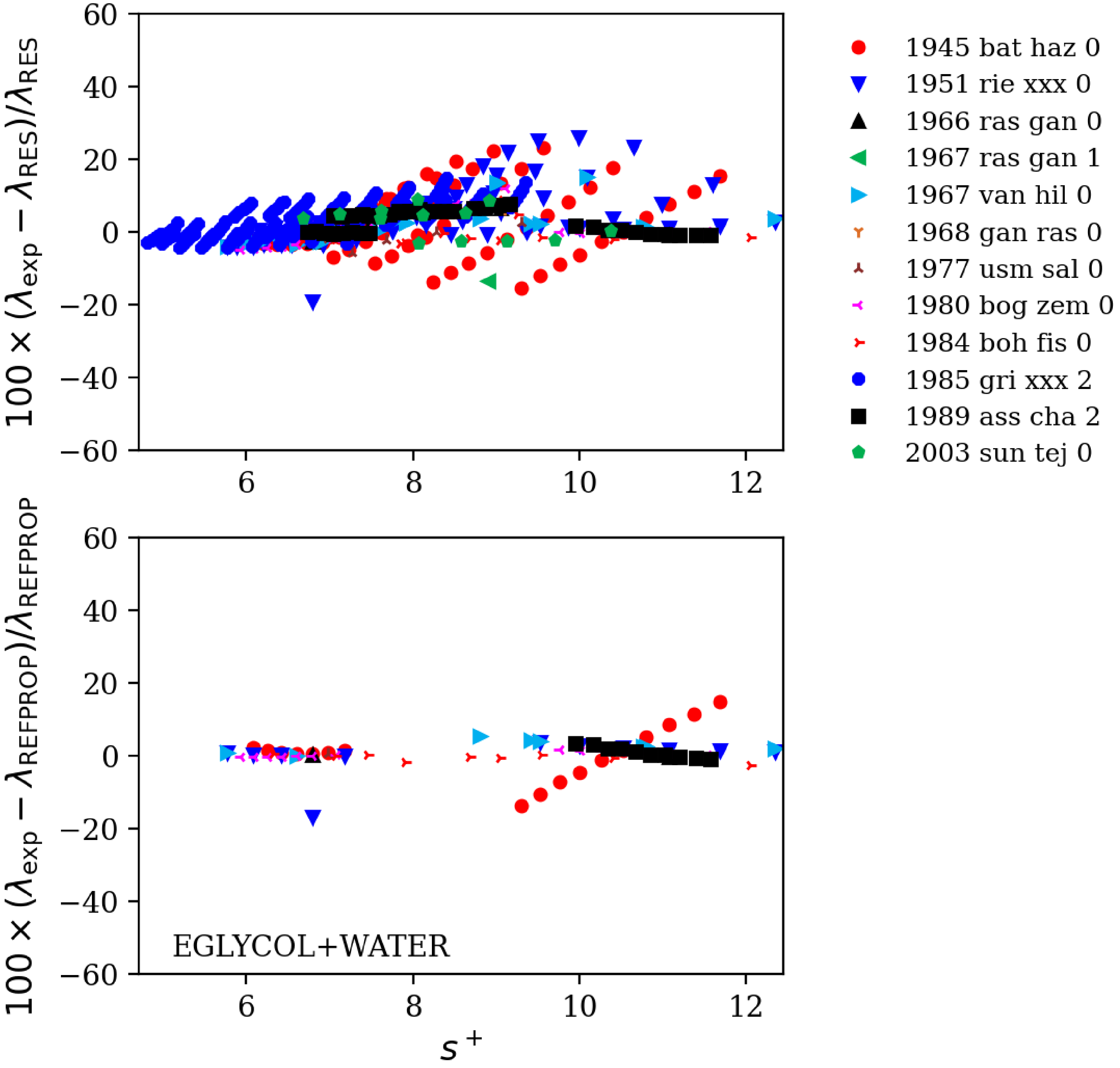

Figure DPR4. EGLYCOL+WATER

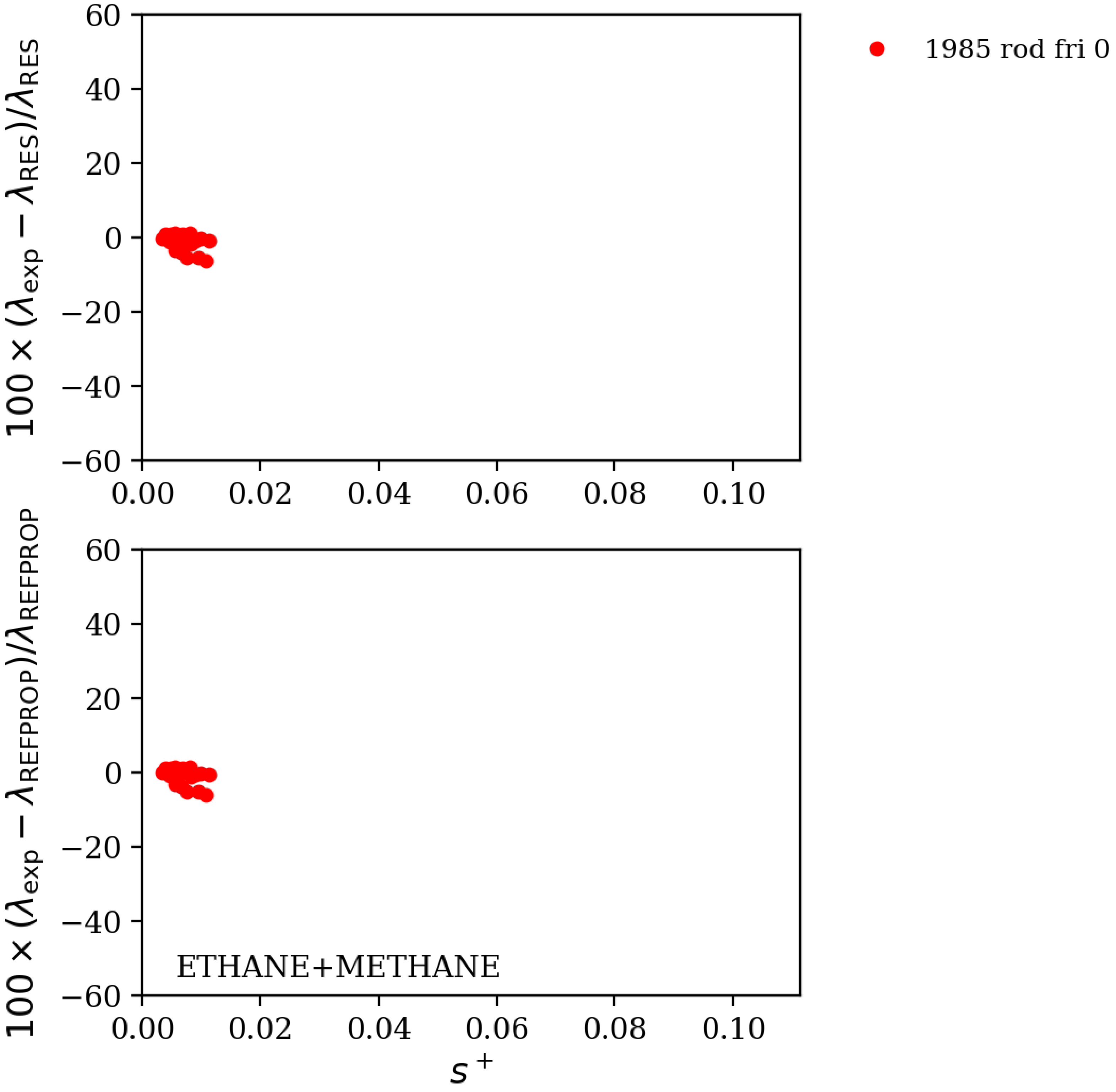

Figure DPR4. ETHANE+METHANE

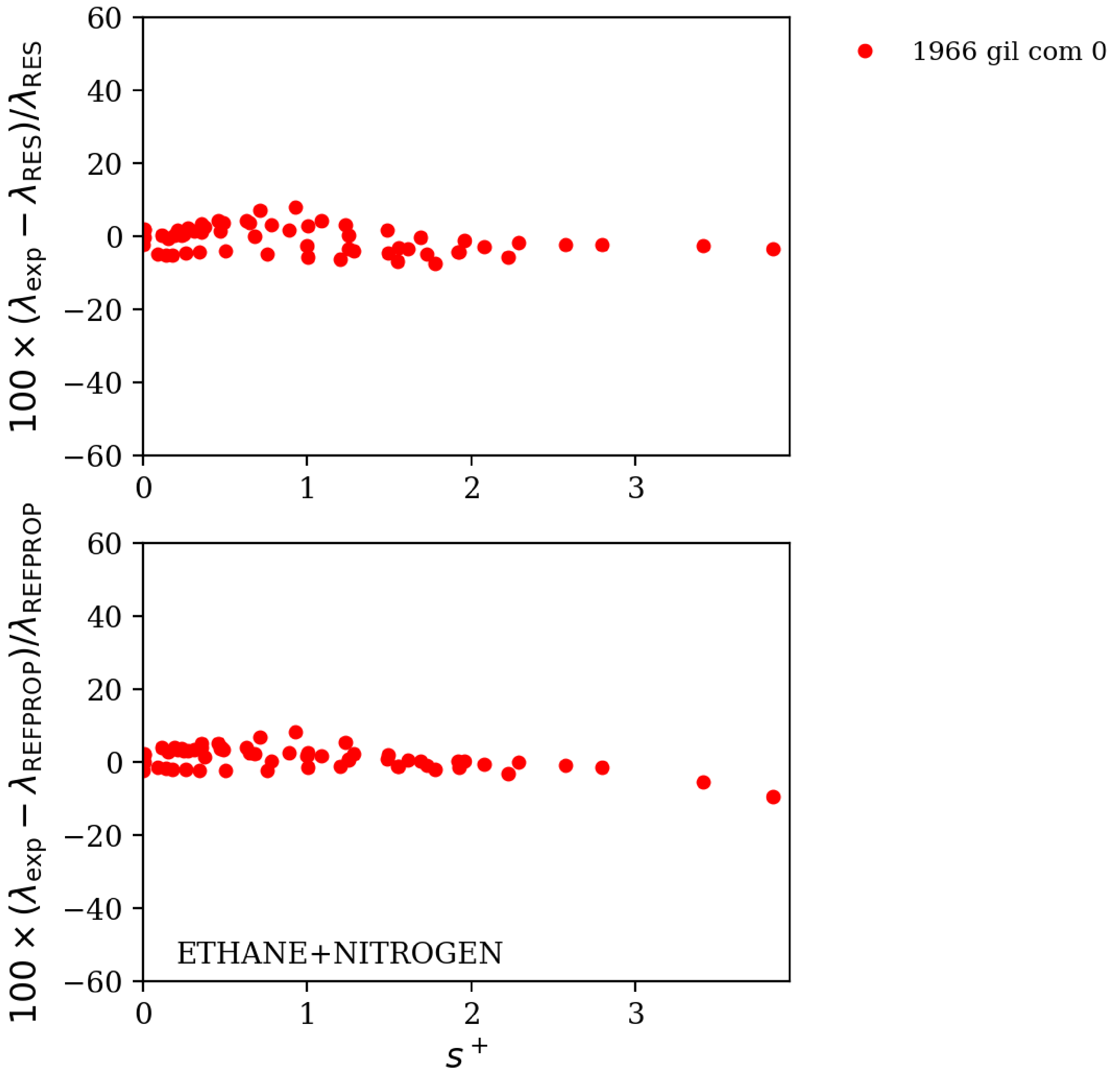

Figure DPR4. ETHANE+NITROGEN

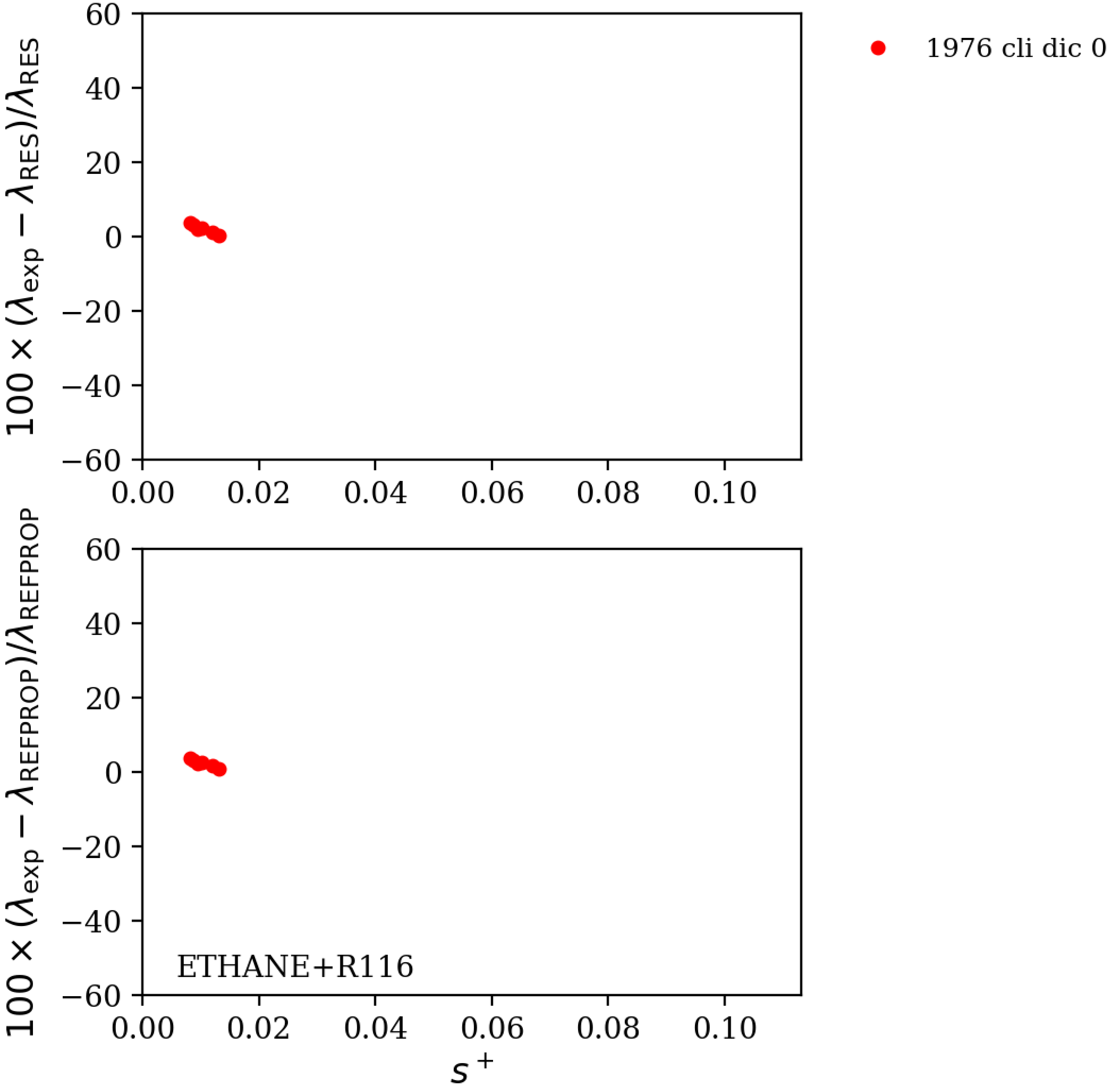

Figure DPR4. ETHANE+R116

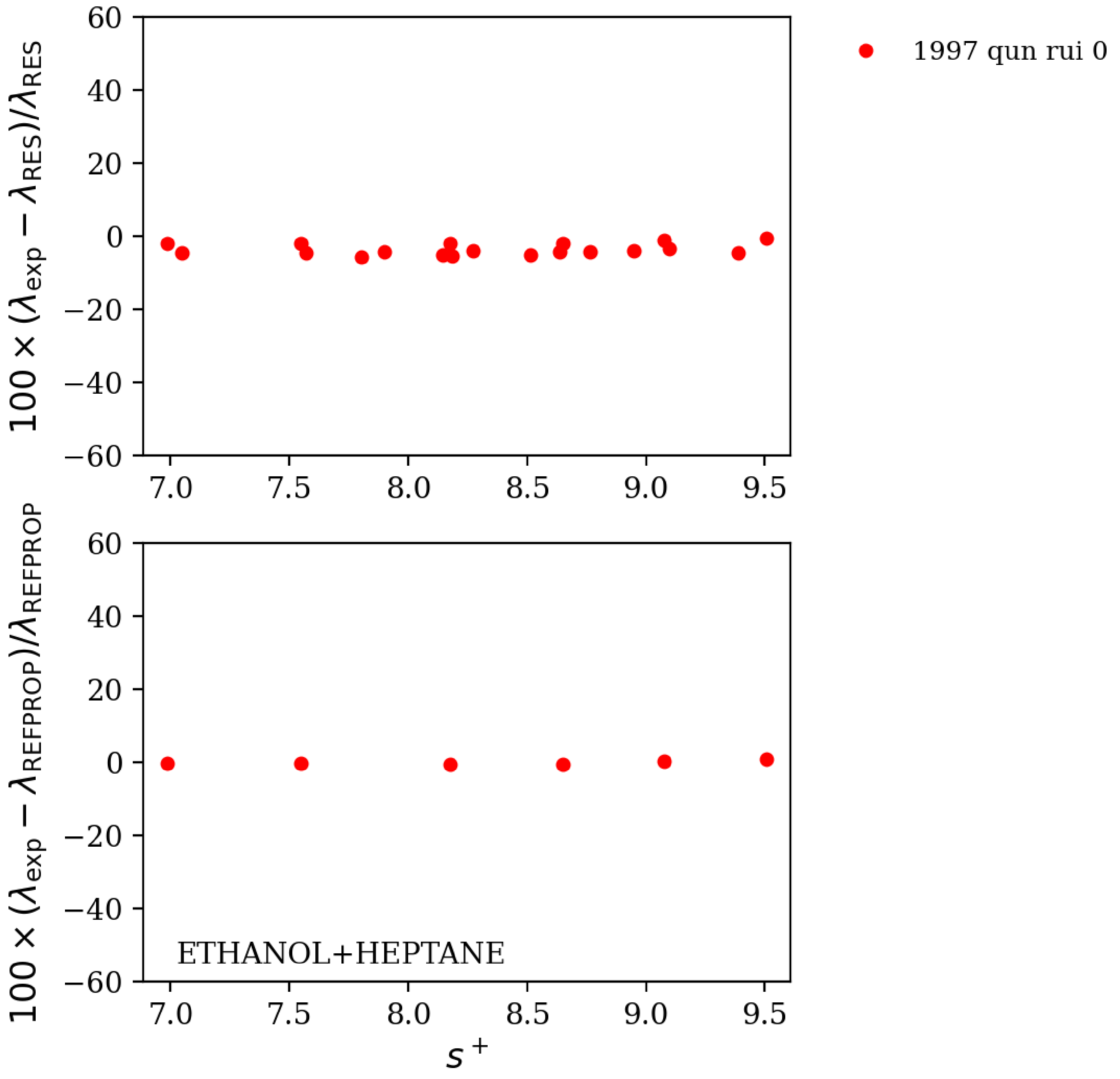

Figure DPR4. ETHANOL+HEPTANE

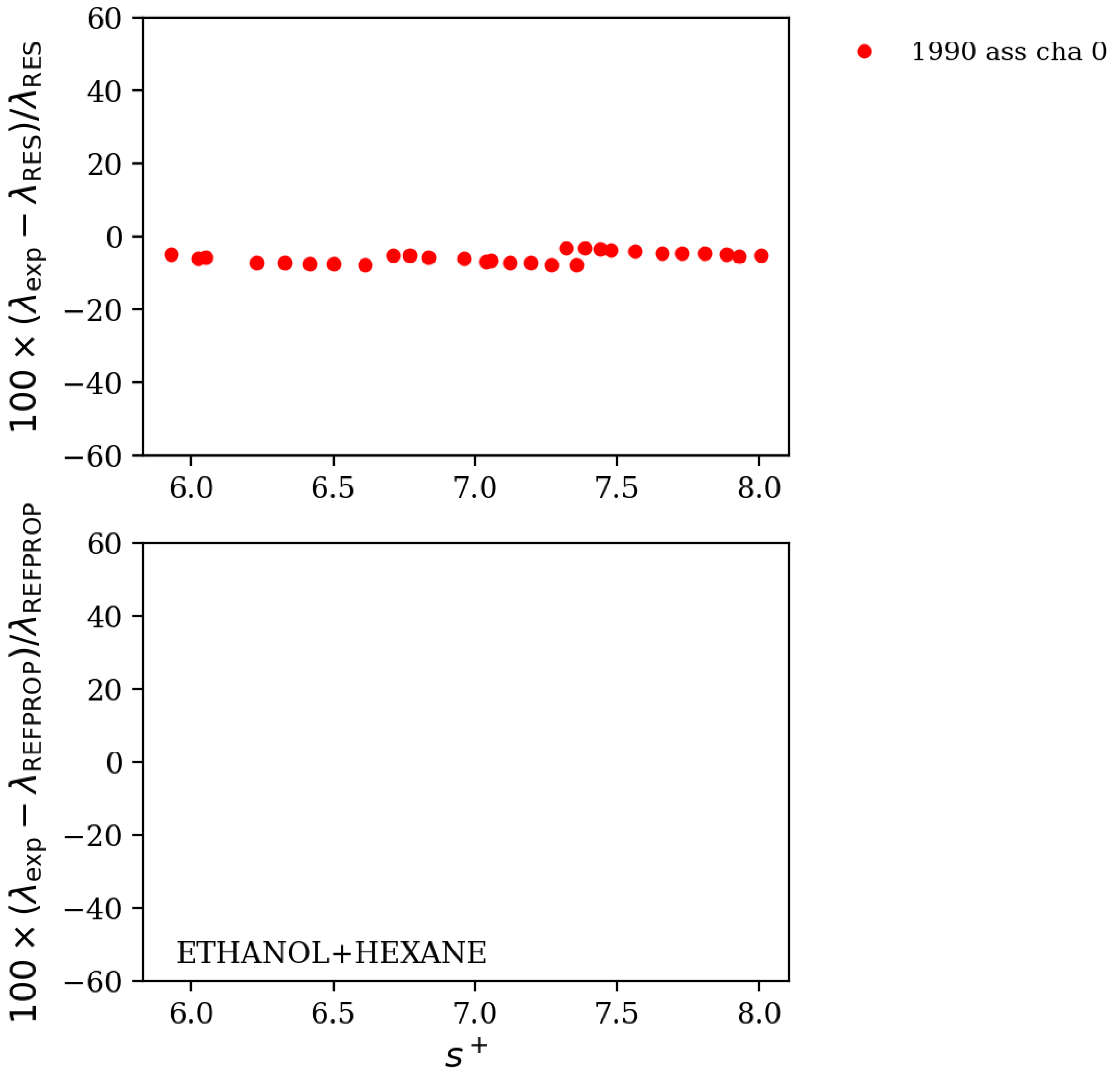

Figure DPR4. ETHANOL+HEXANE

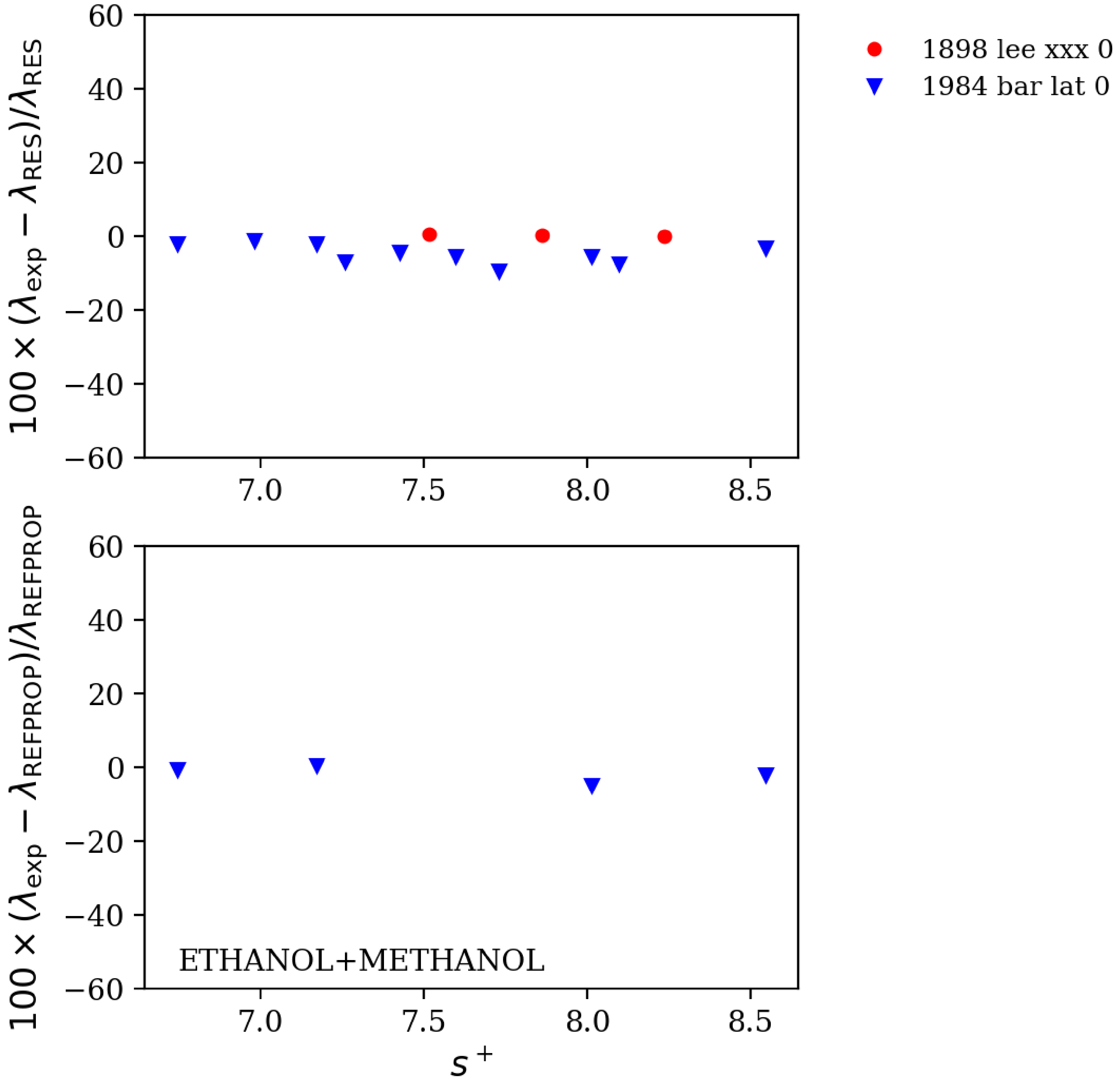

Figure DPR4. ETHANOL+METHANOL

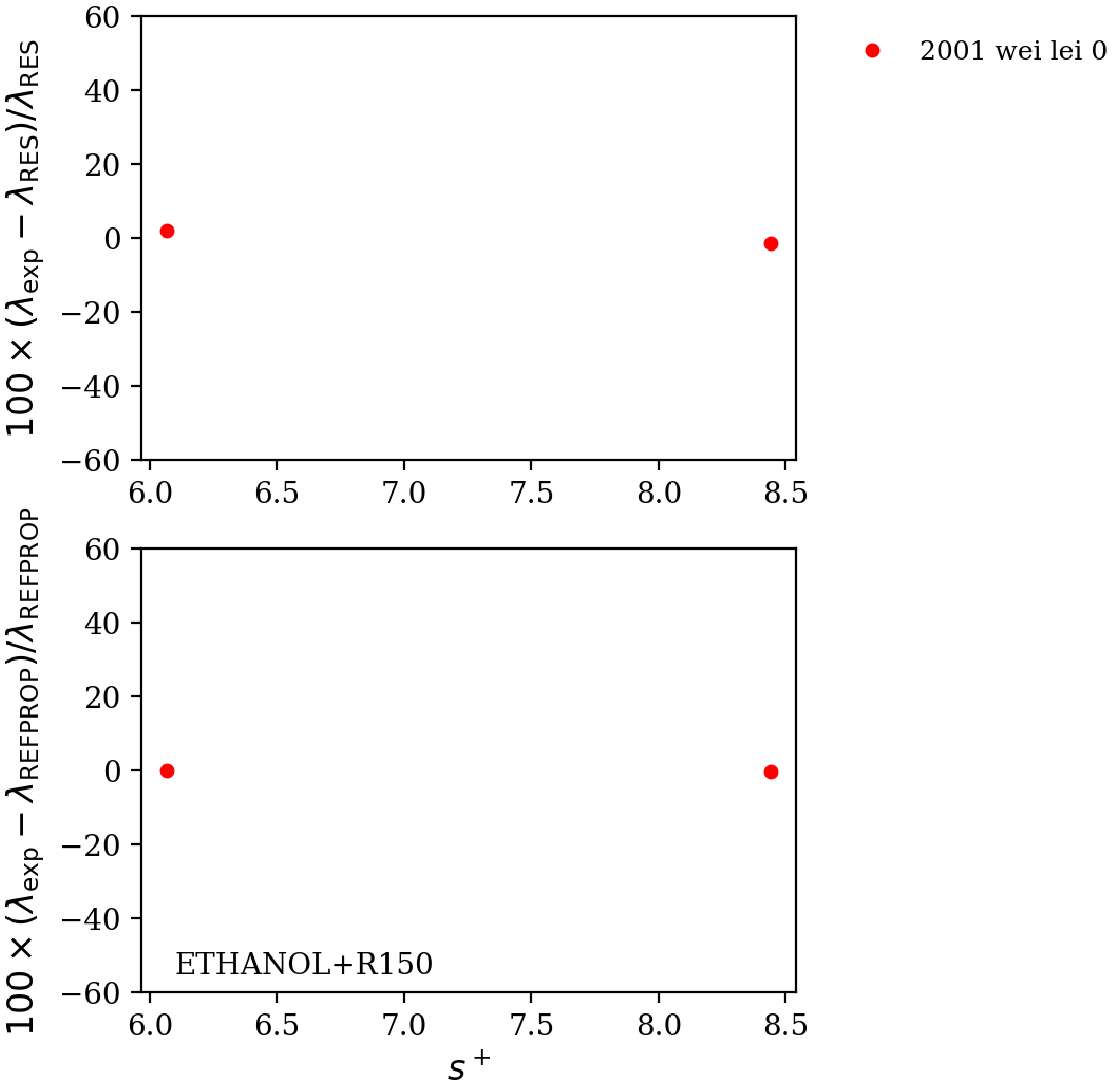

Figure DPR4. ETHANOL+R150

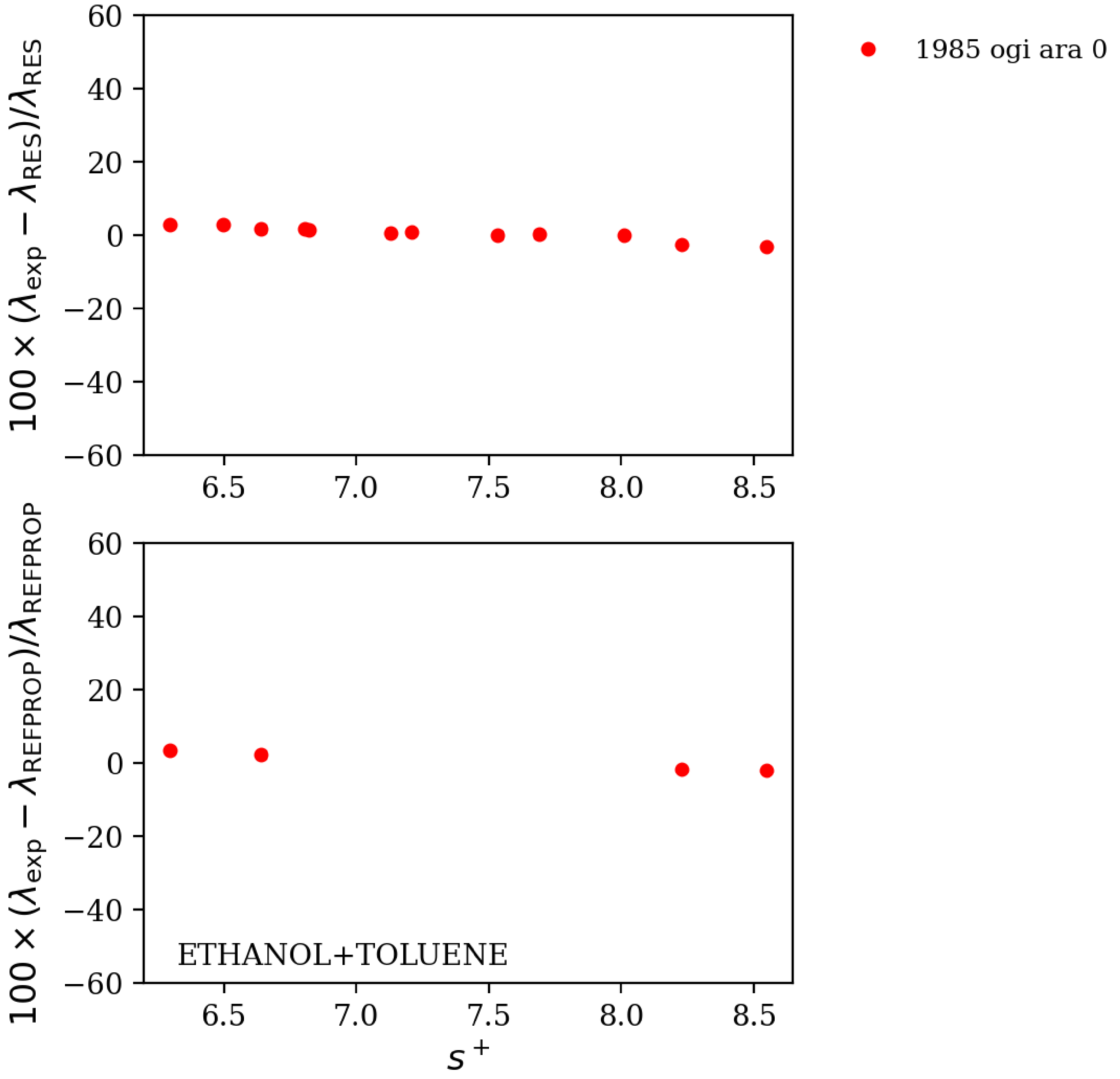

Figure DPR4. ETHANOL+TOLUENE

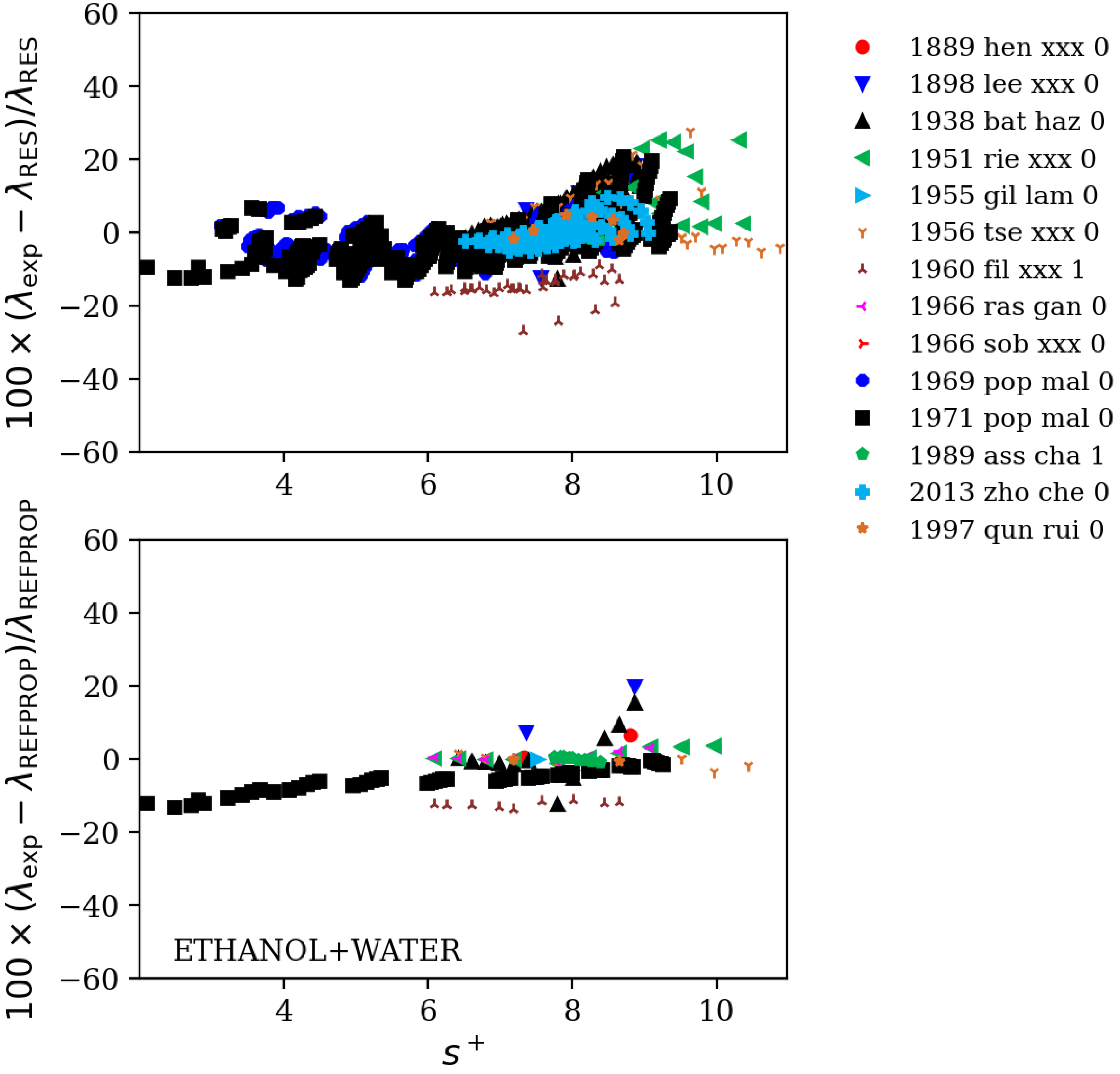

Figure DPR4. ETHANOL+WATER

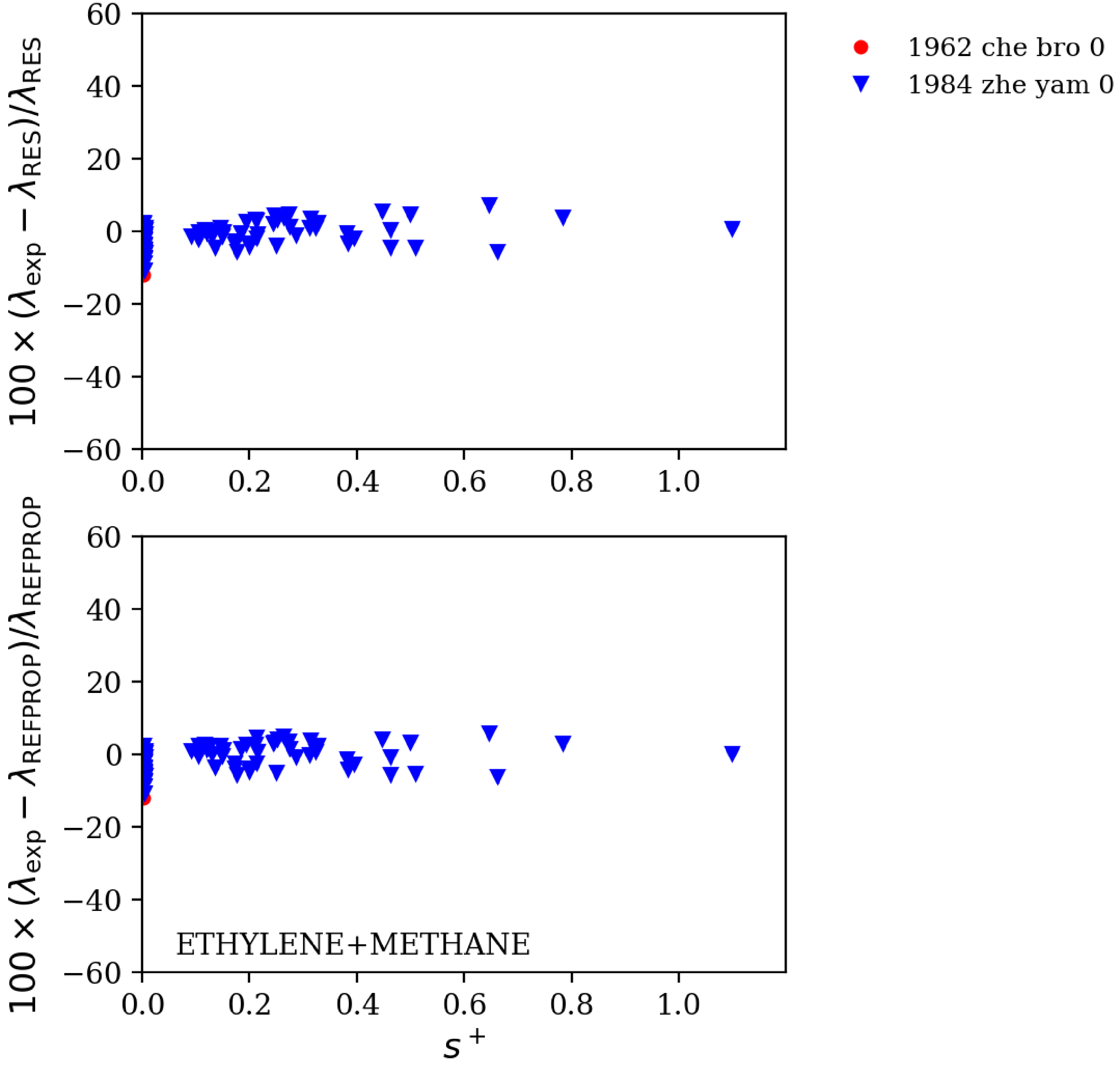

Figure DPR4. ETHYLENE+METHANE

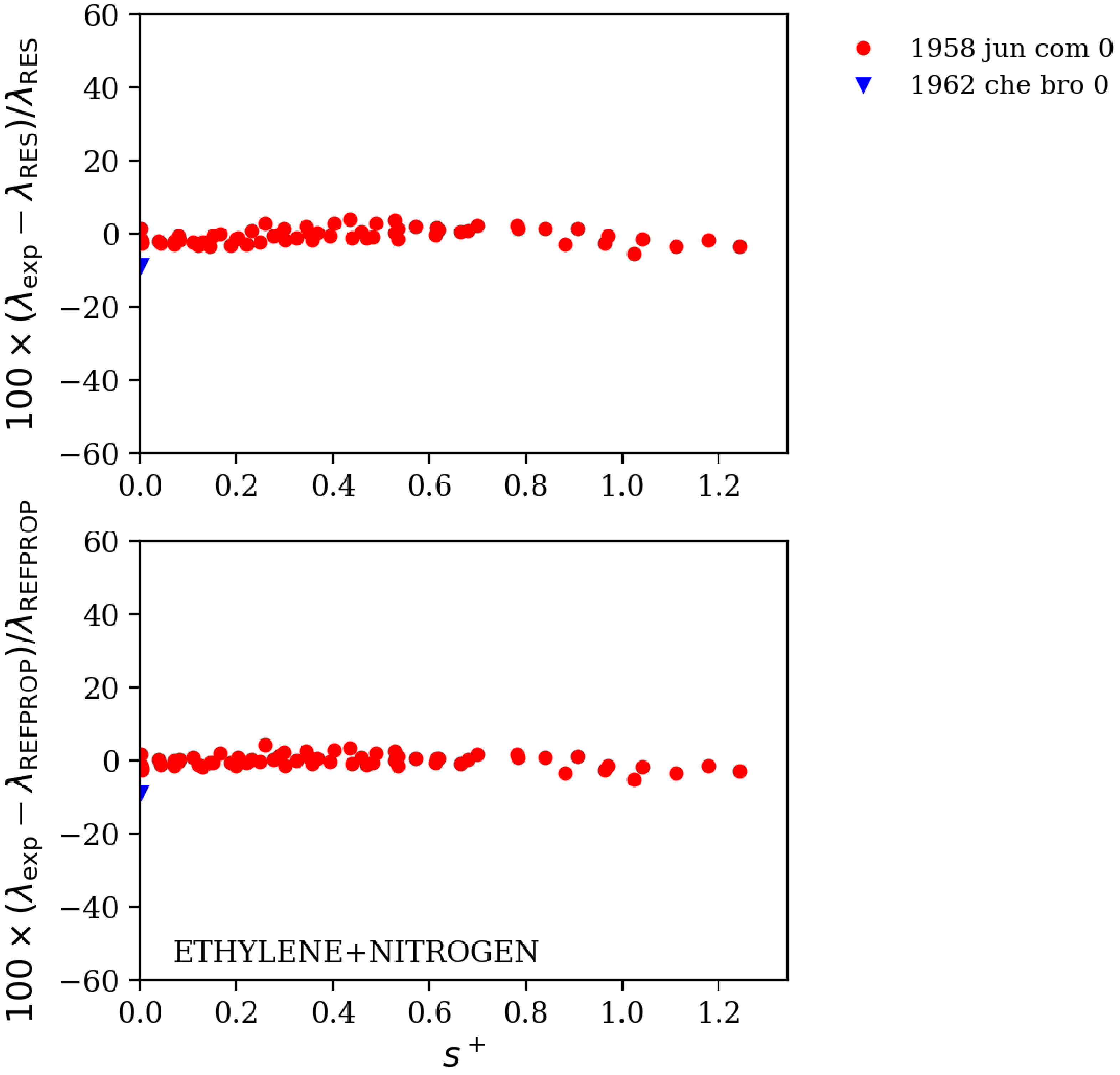

Figure DPR4. ETHYLENE+NITROGEN

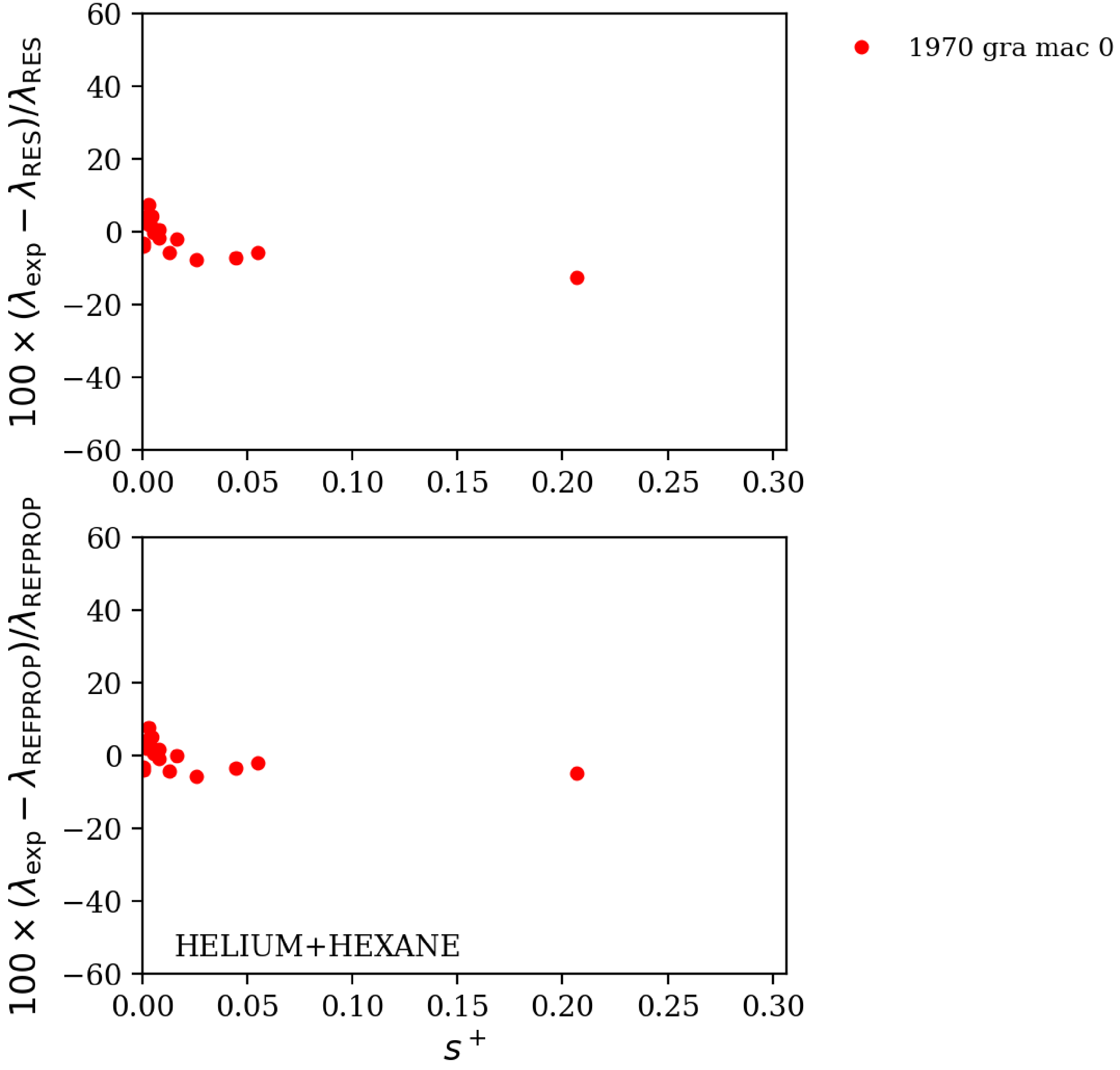

Figure DPR4. HELIUM+HEXANE

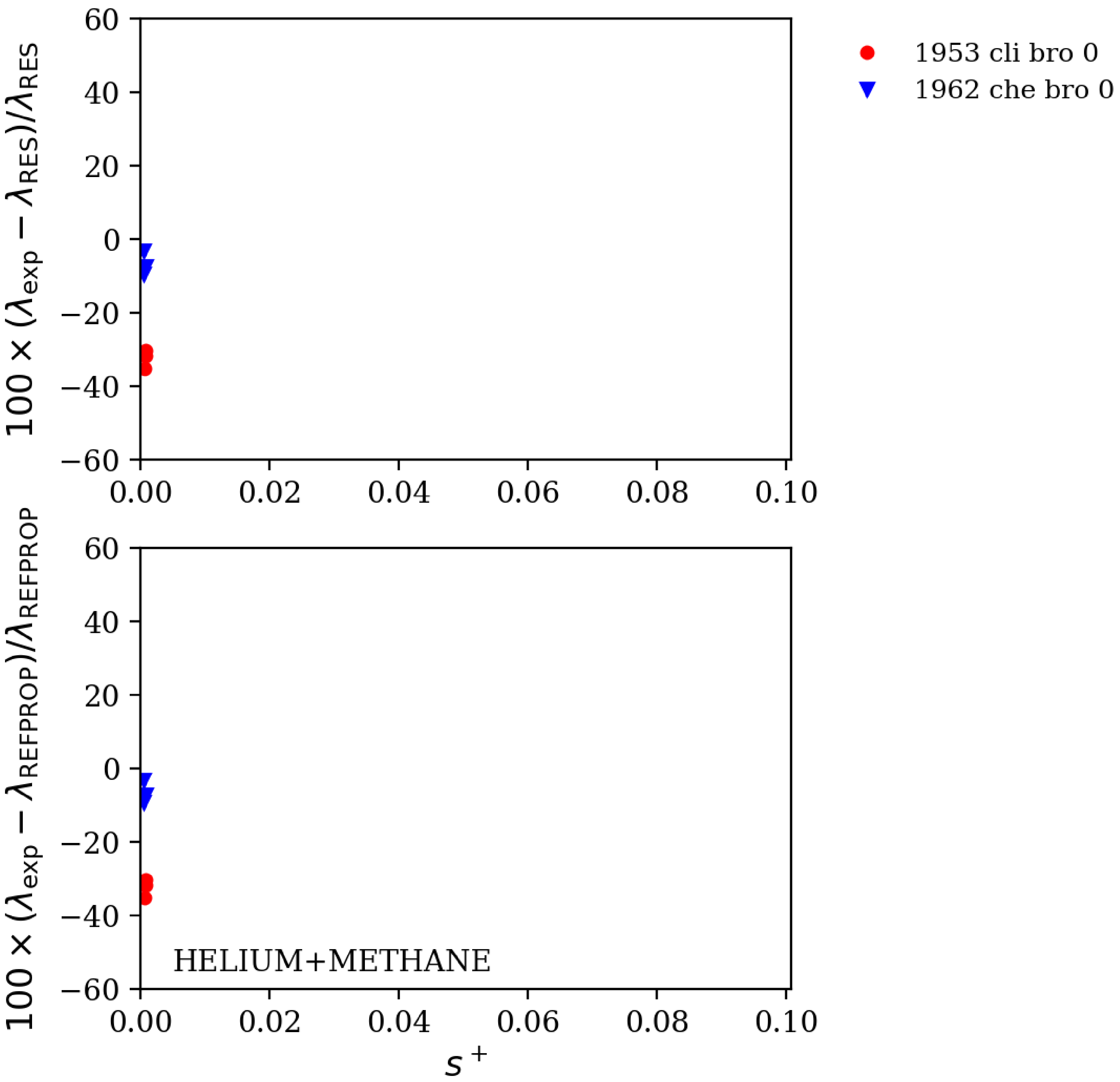

Figure DPR4. HELIUM+METHANE

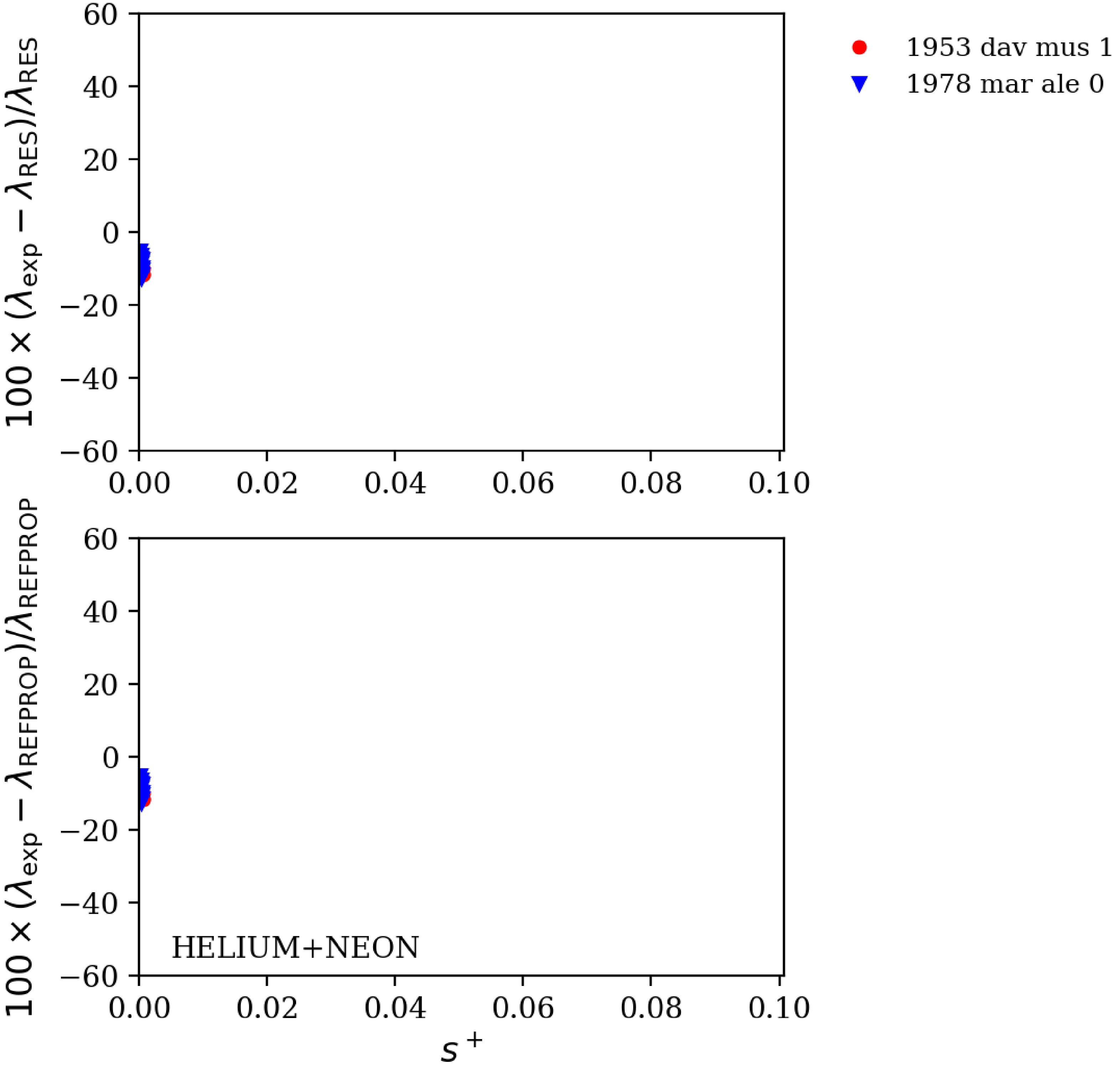

Figure DPR4. HELIUM+NEON

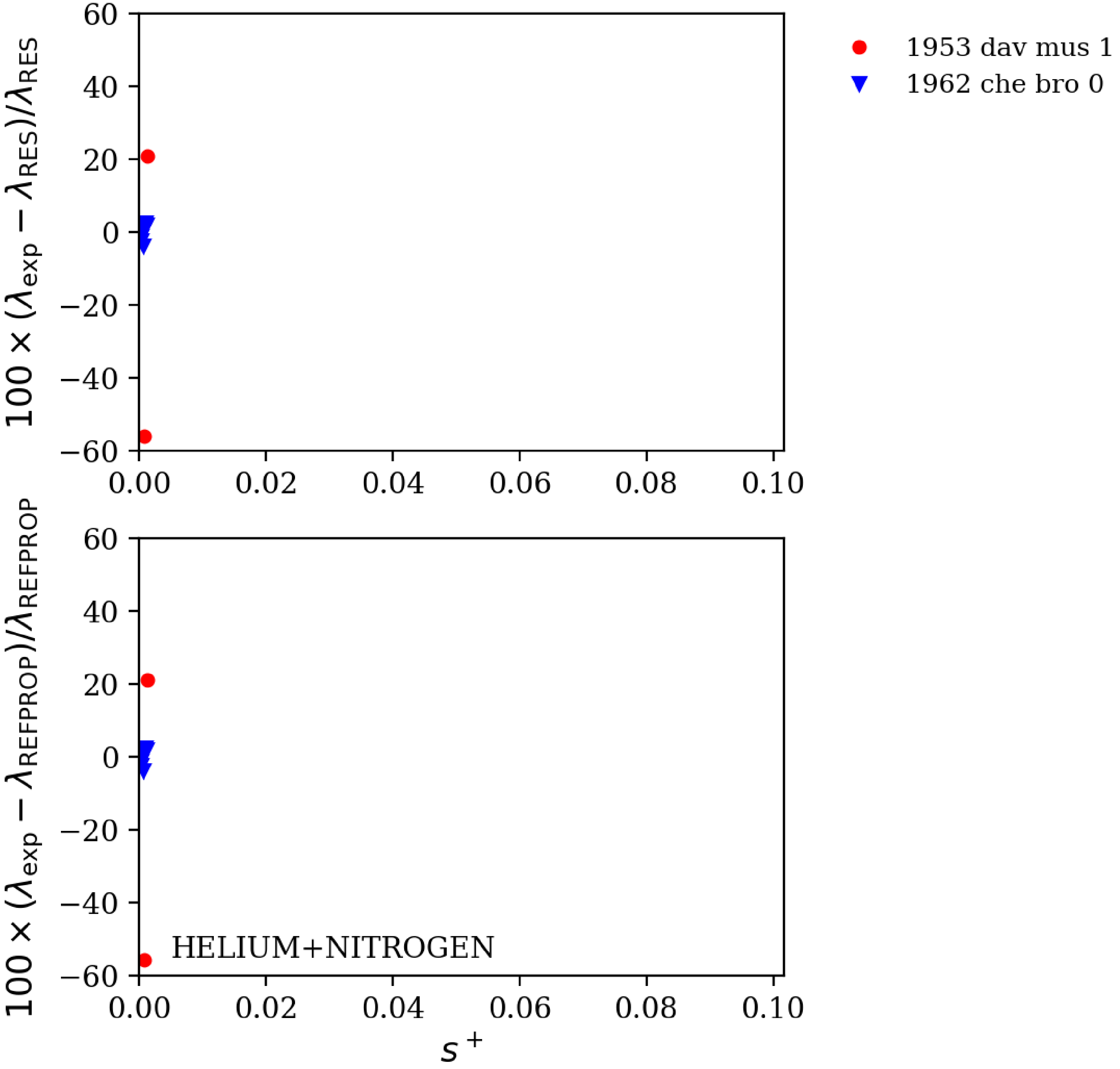

Figure DPR4. HELIUM+NITROGEN

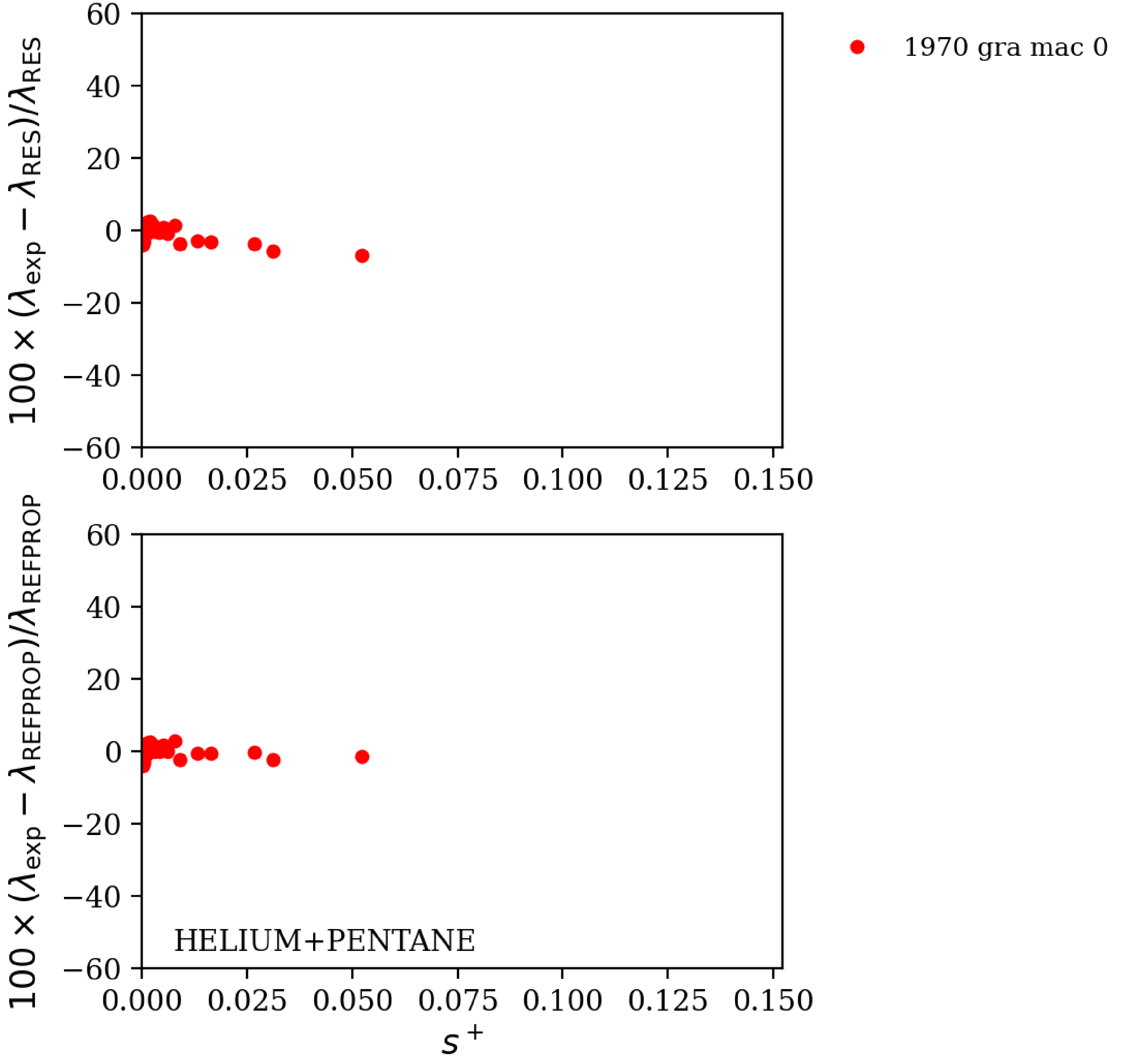

Figure DPR4. HELIUM+PENTANE

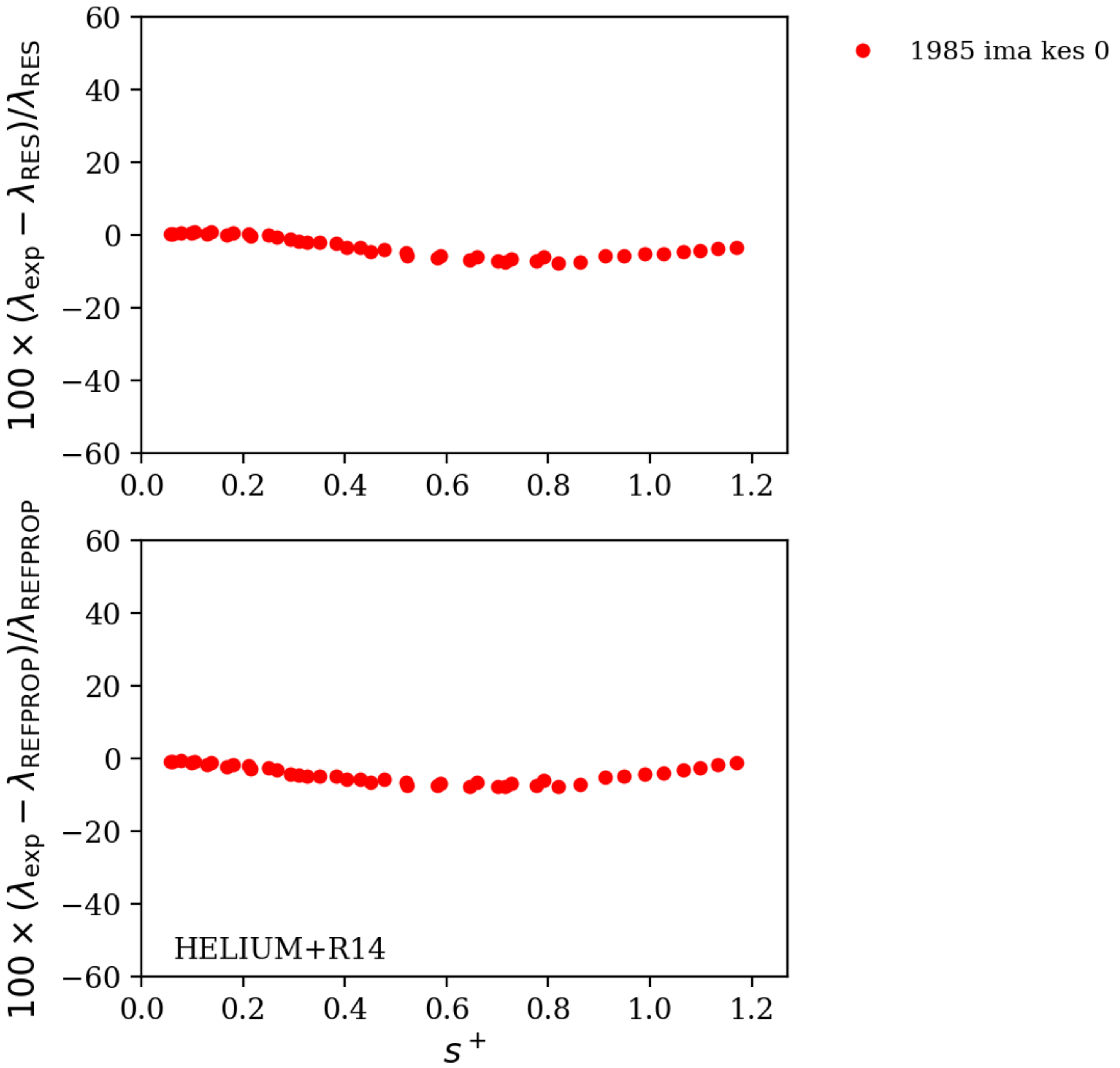

Figure DPR4. HELIUM+R14

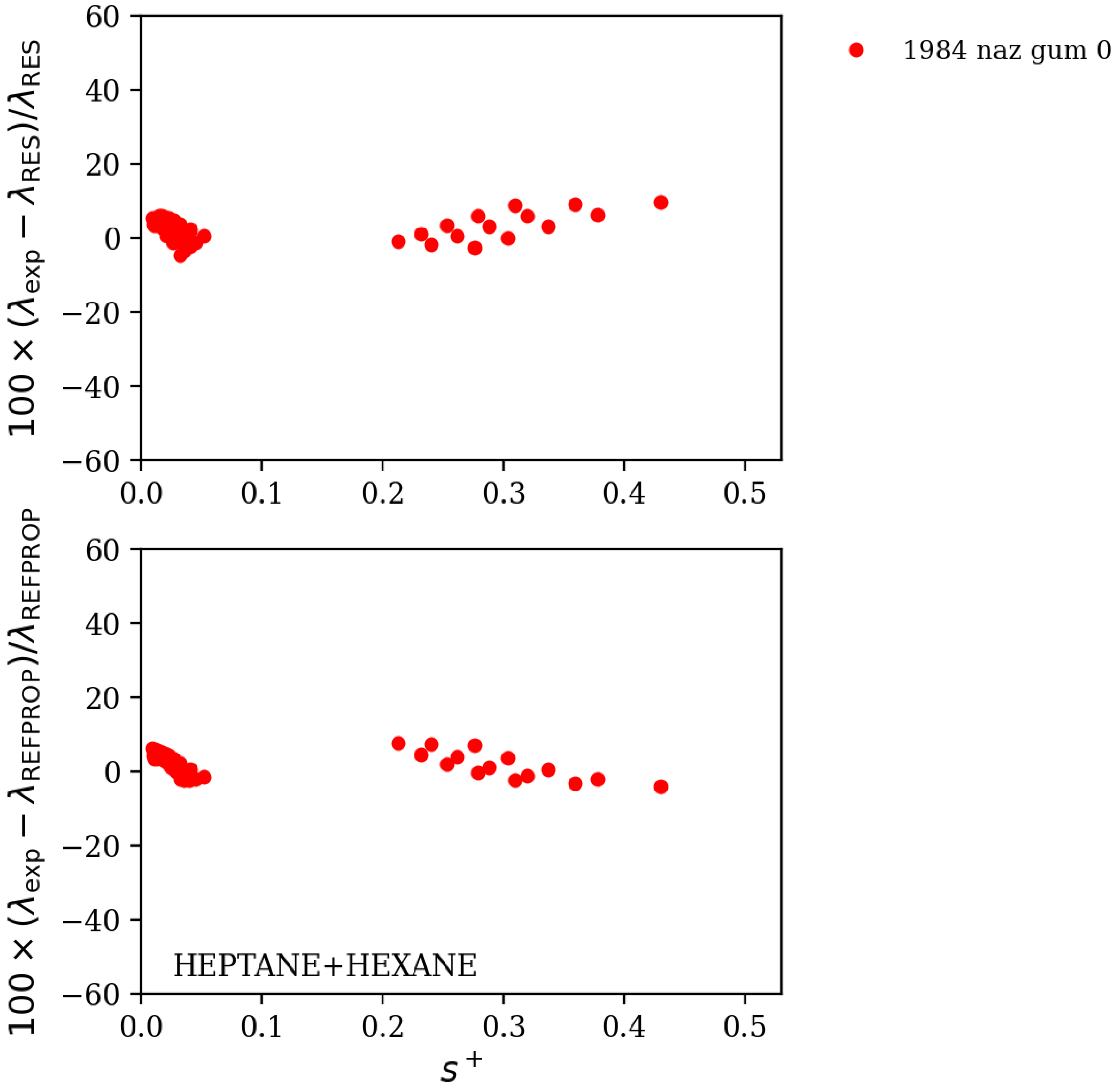

Figure DPR4. HEPTANE+HEXANE

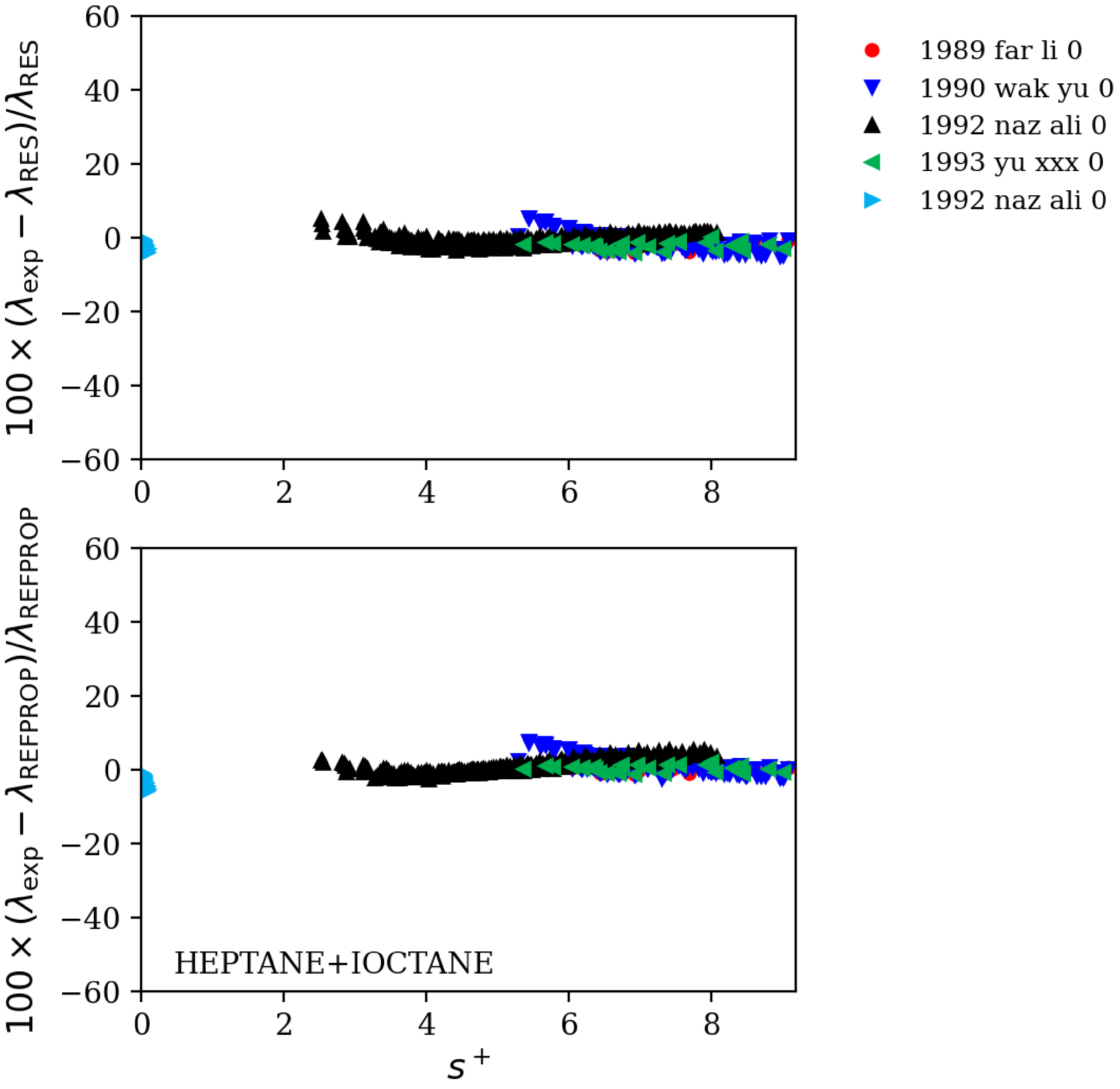

Figure DPR4. HEPTANE+IOCTANE

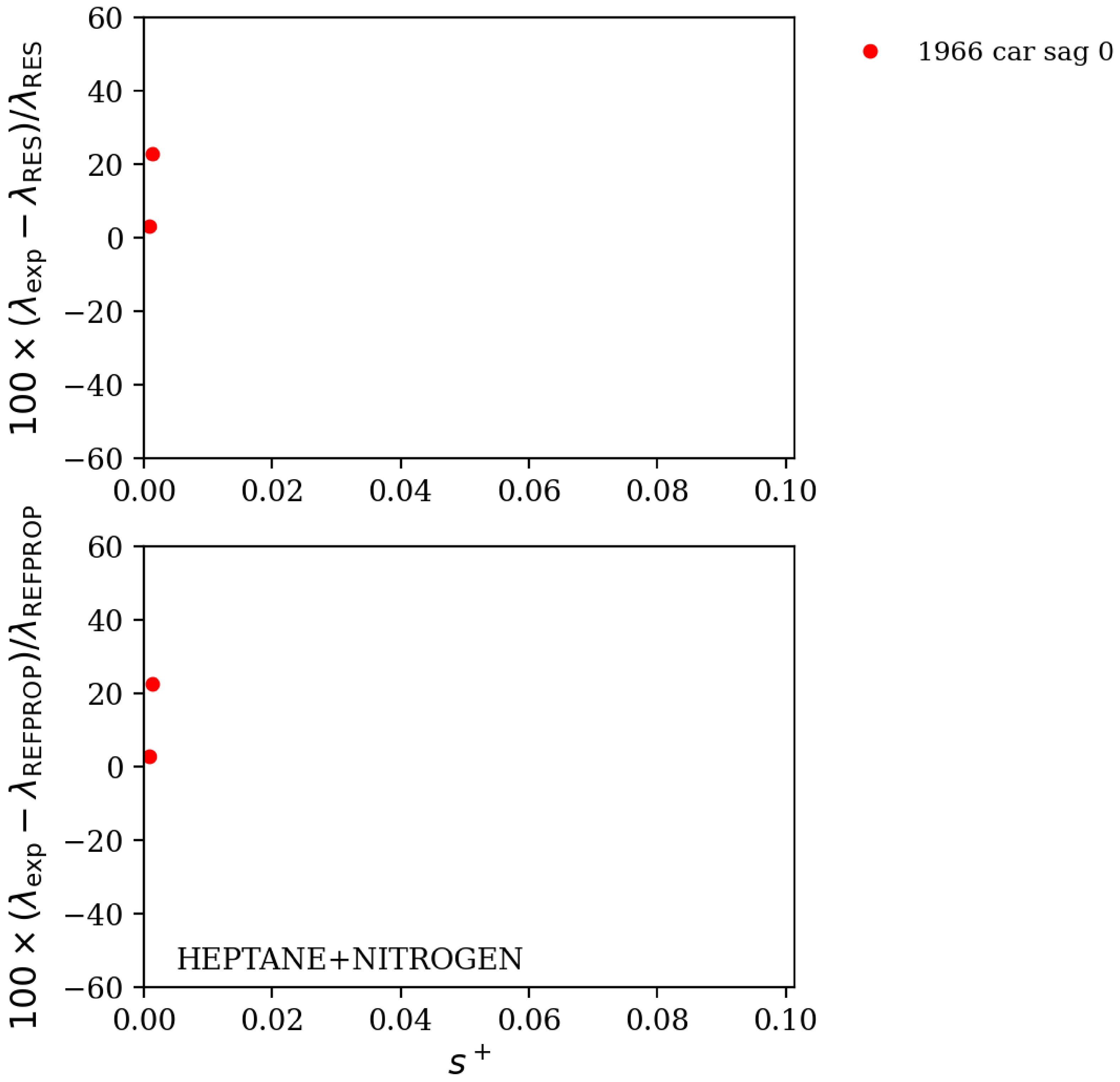

Figure DPR4. HEPTANE+NITROGEN

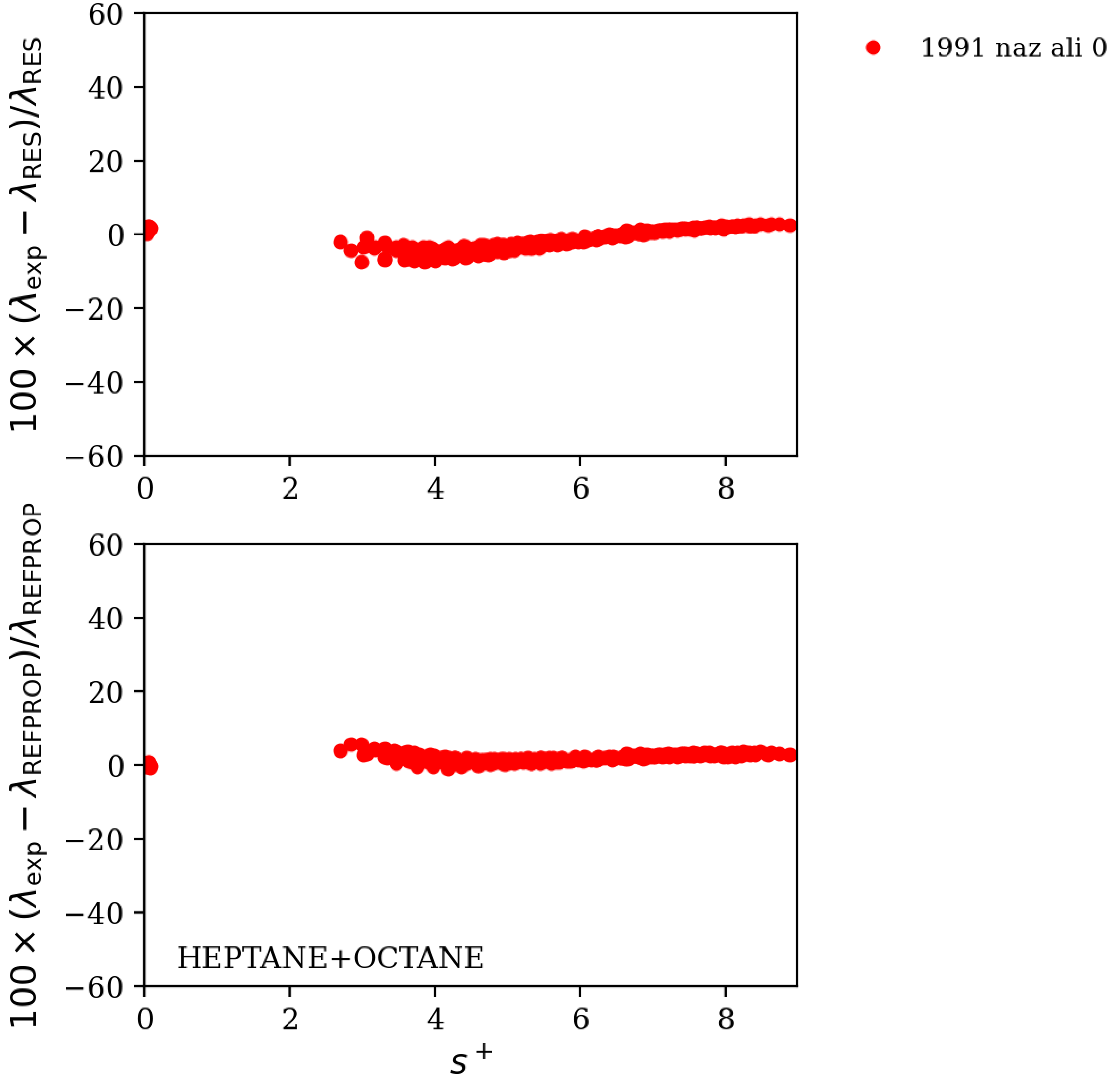

Figure DPR4. HEPTANE+OCTANE

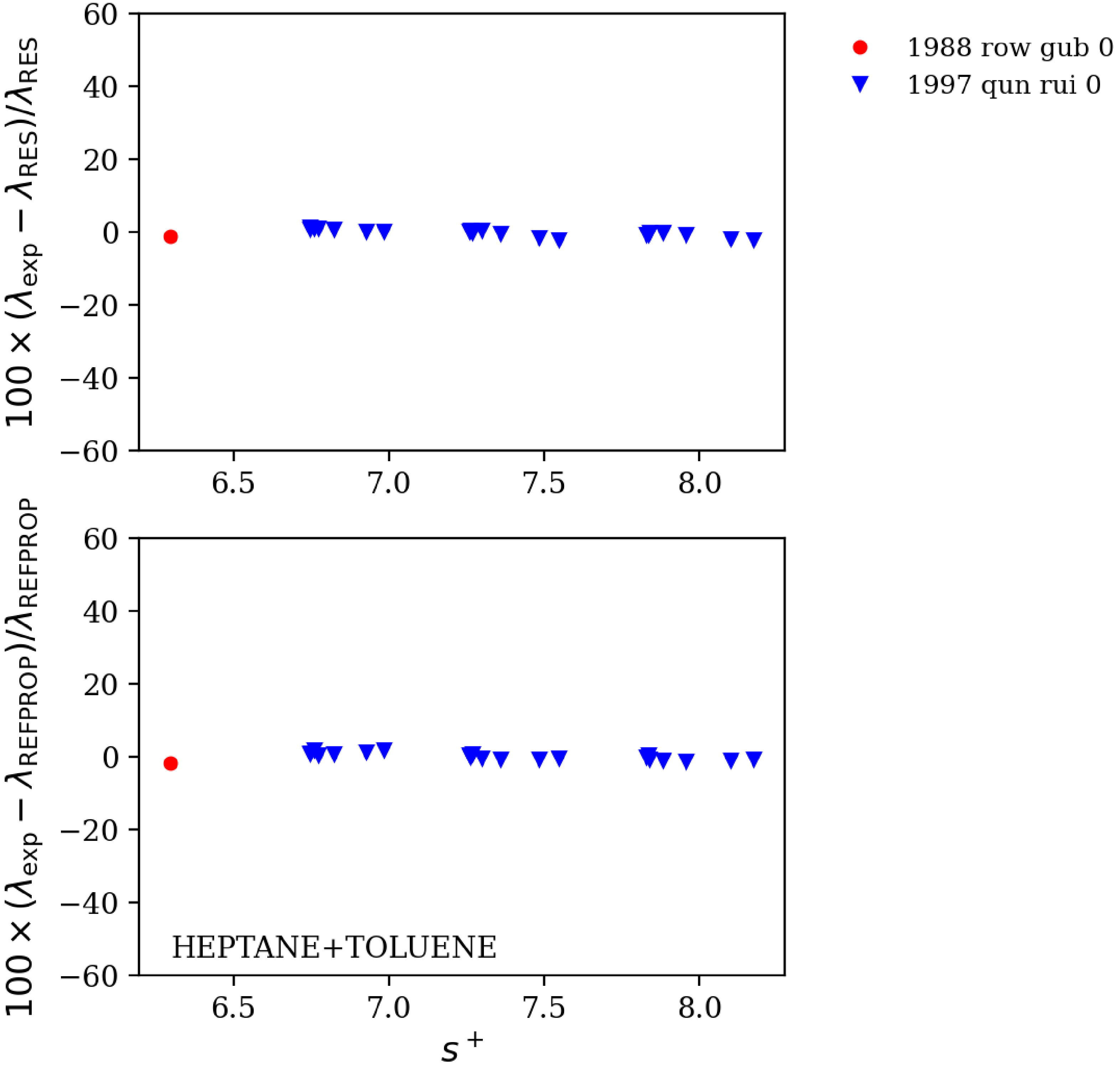

Figure DPR4. HEPTANE+TOLUENE

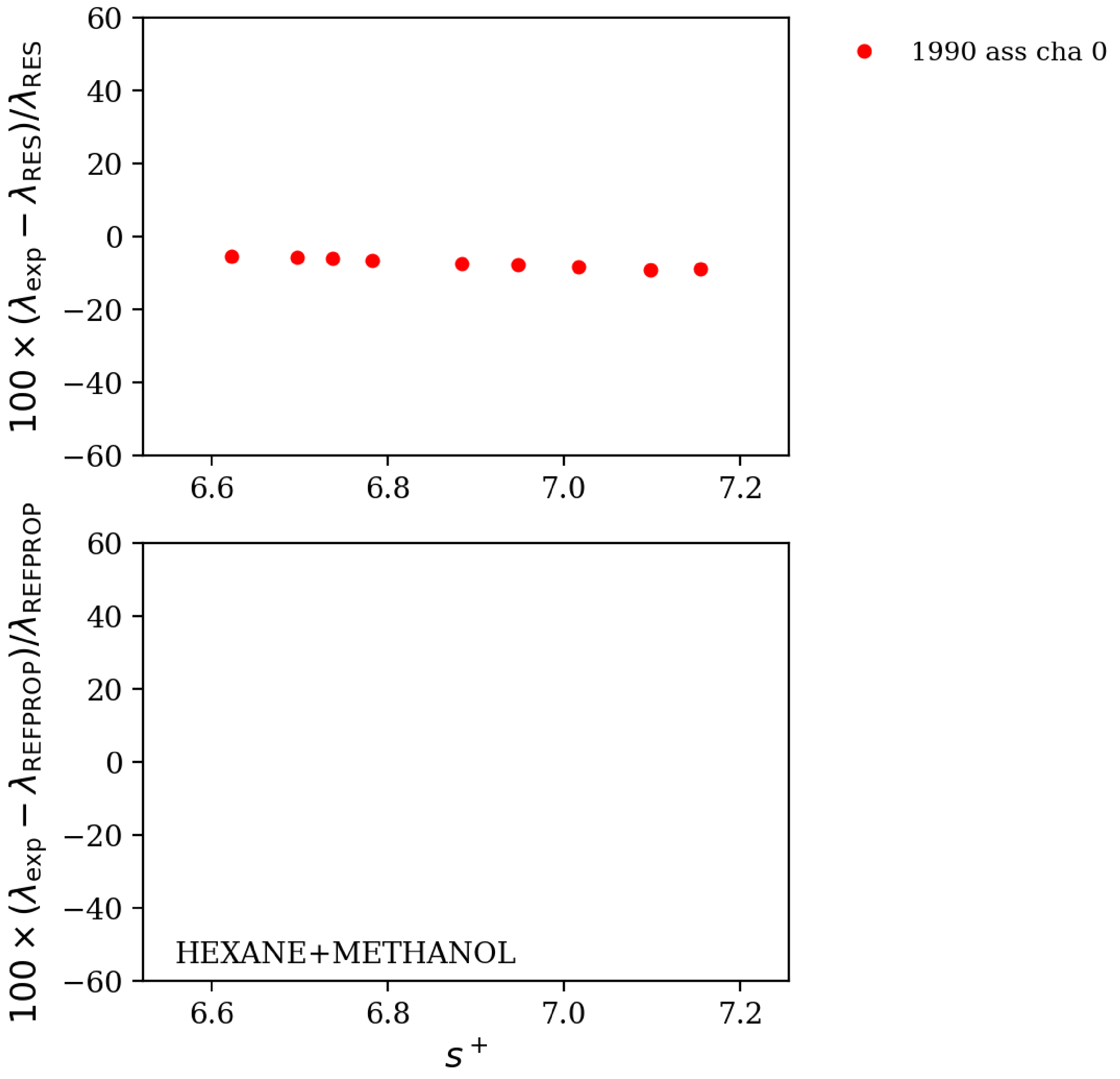

Figure DPR4. HEXANE+METHANOL

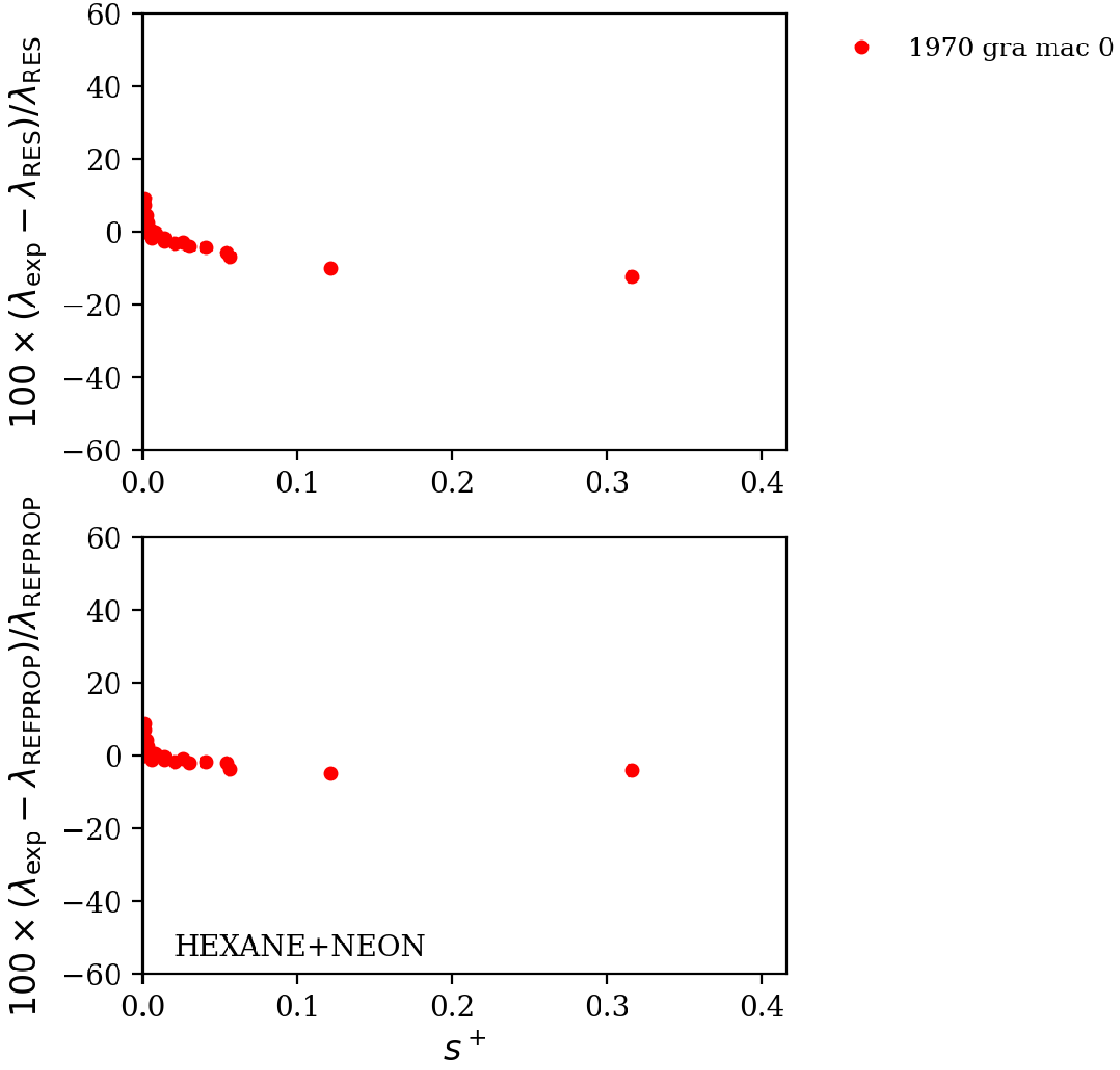

Figure DPR4. HEXANE+NEON

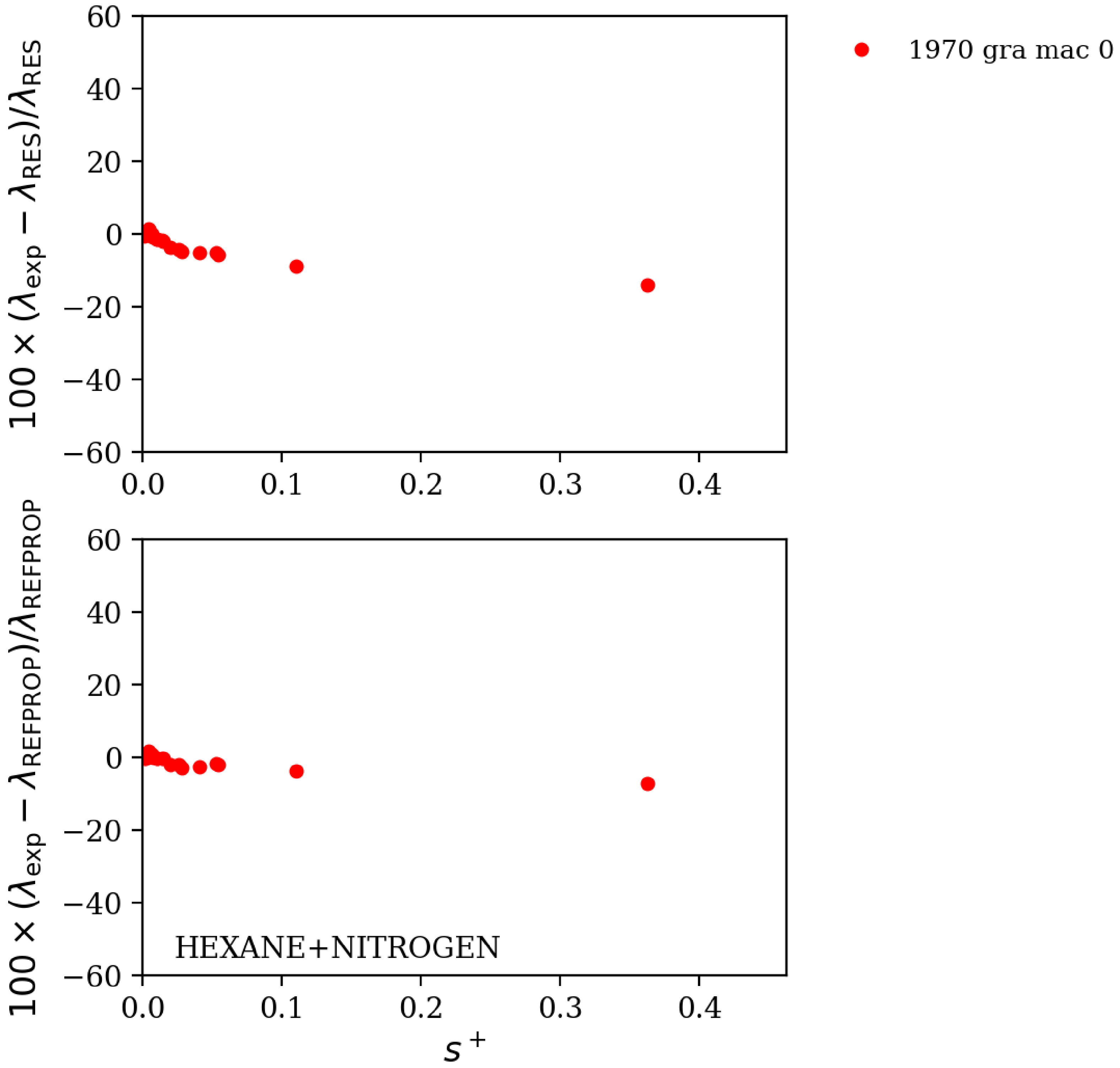

Figure DPR4. HEXANE+NITROGEN

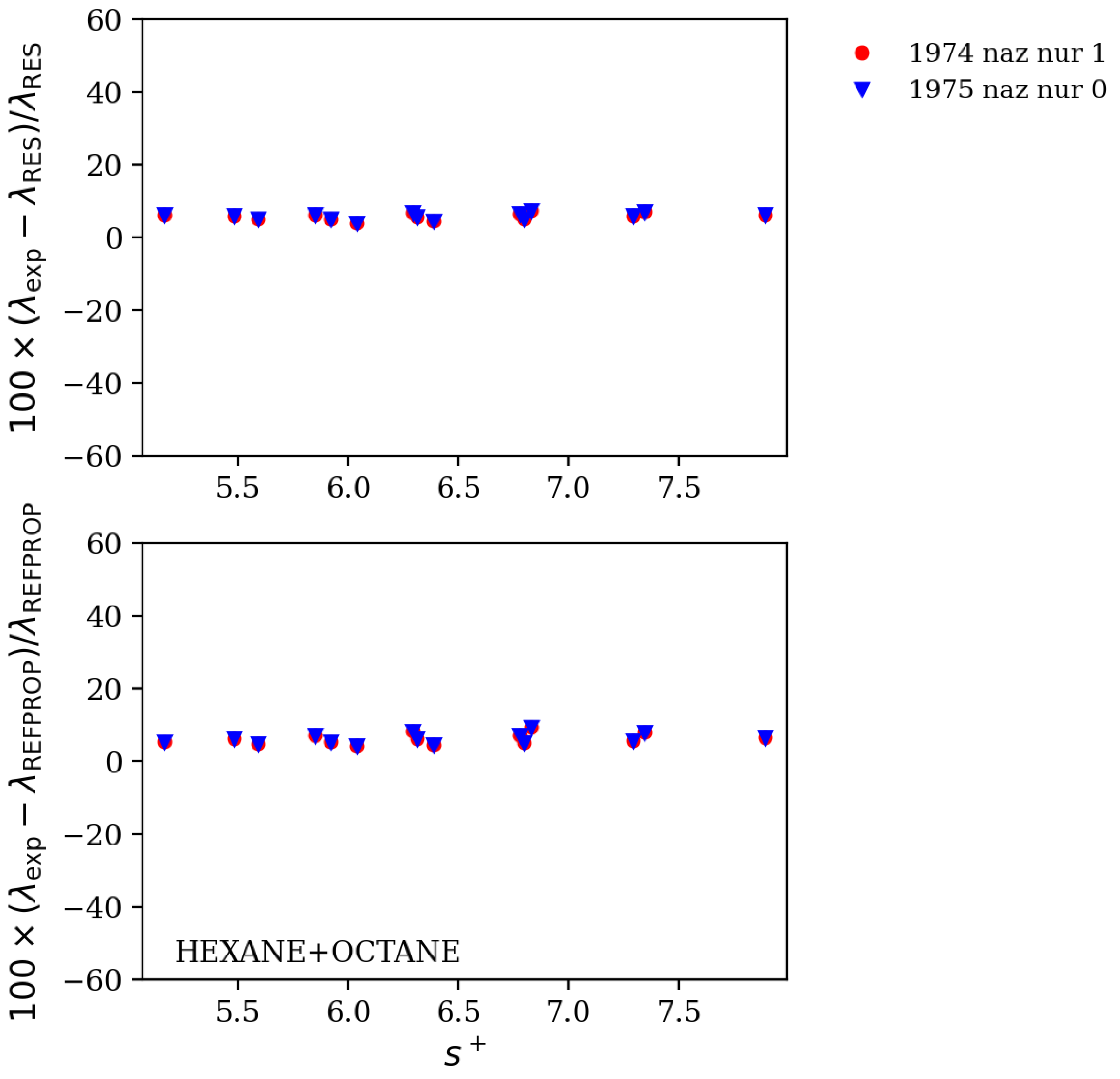

Figure DPR4. HEXANE+OCTANE

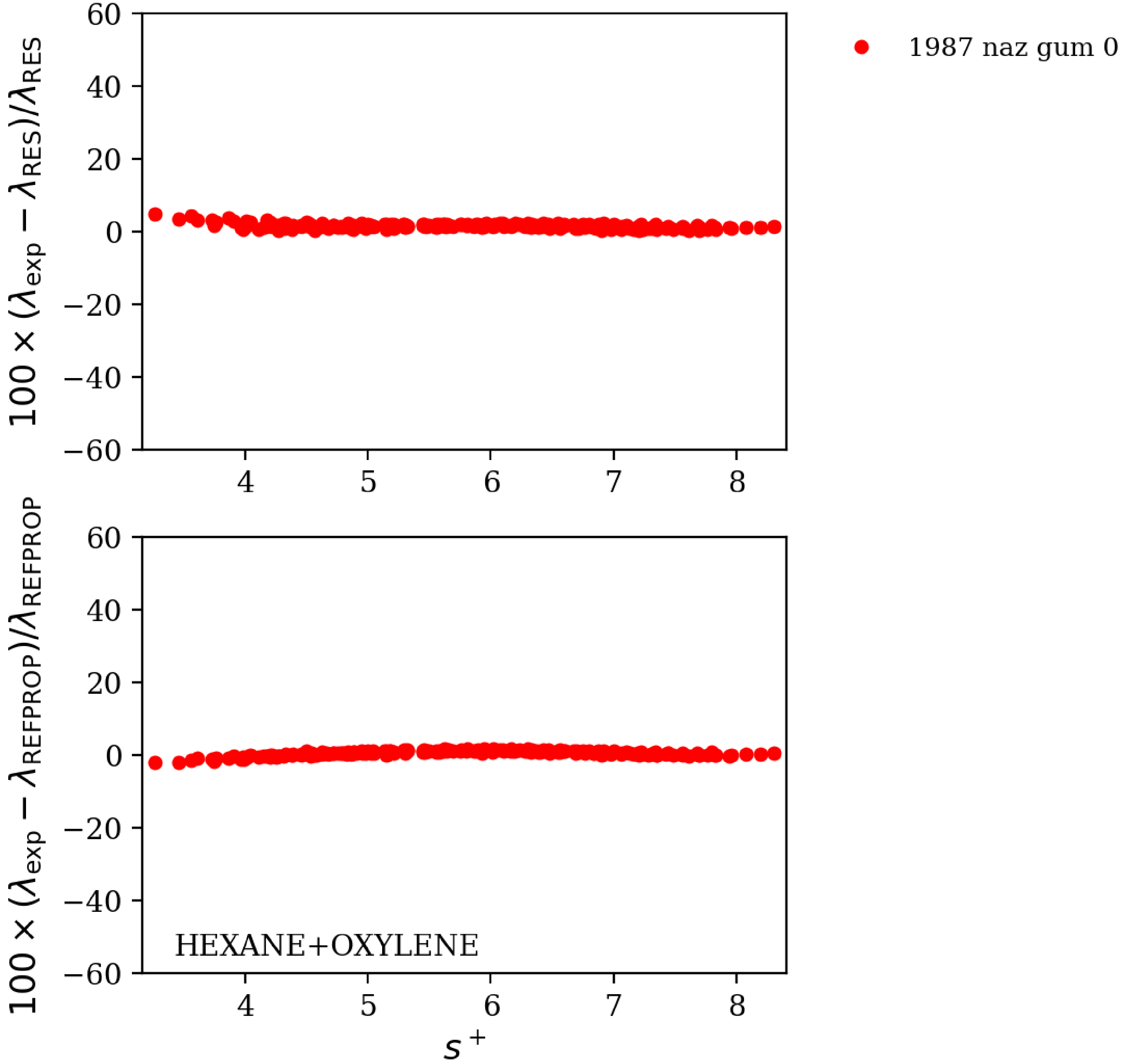

Figure DPR4. HEXANE+OXYLENE

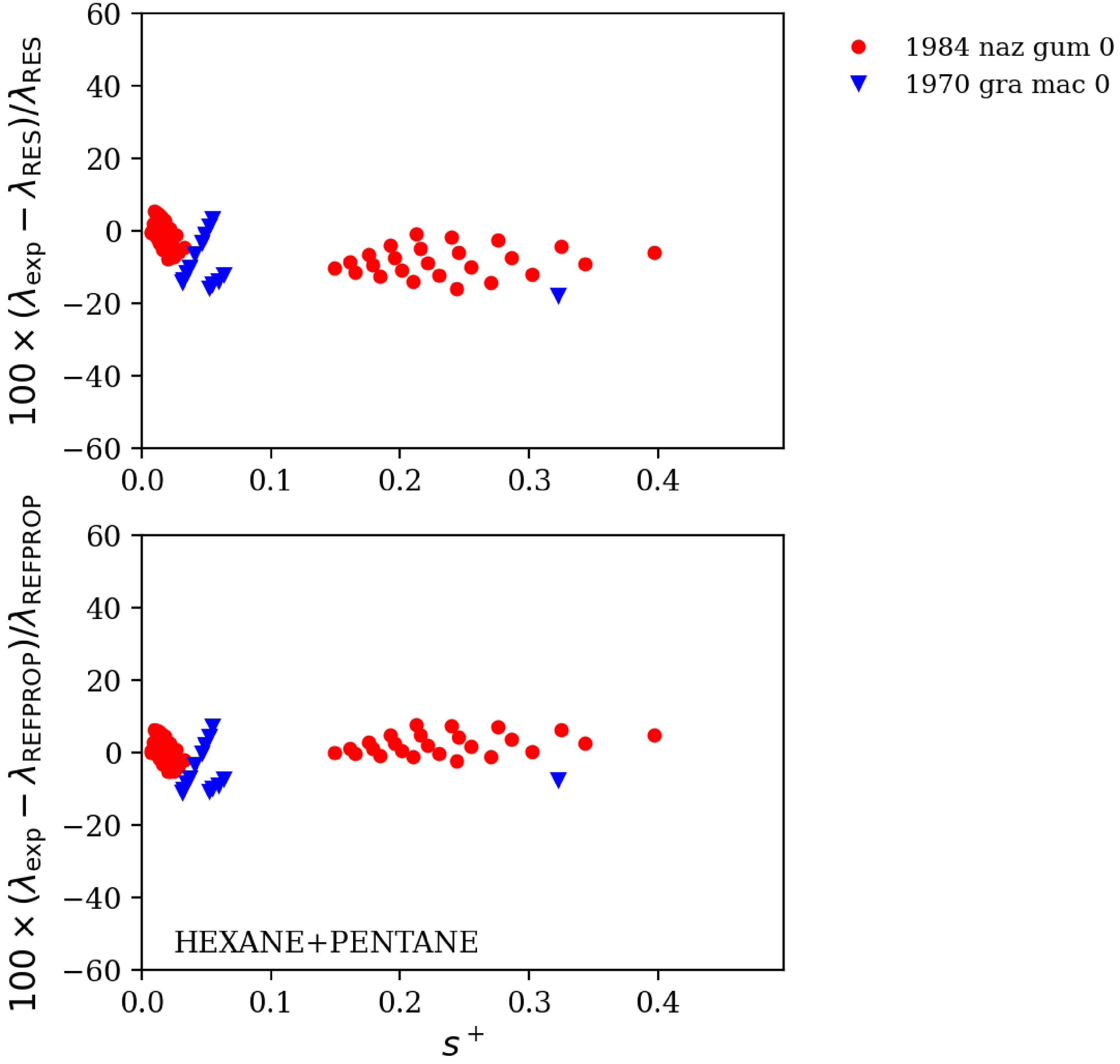

Figure DPR4. HEXANE+PENTANE

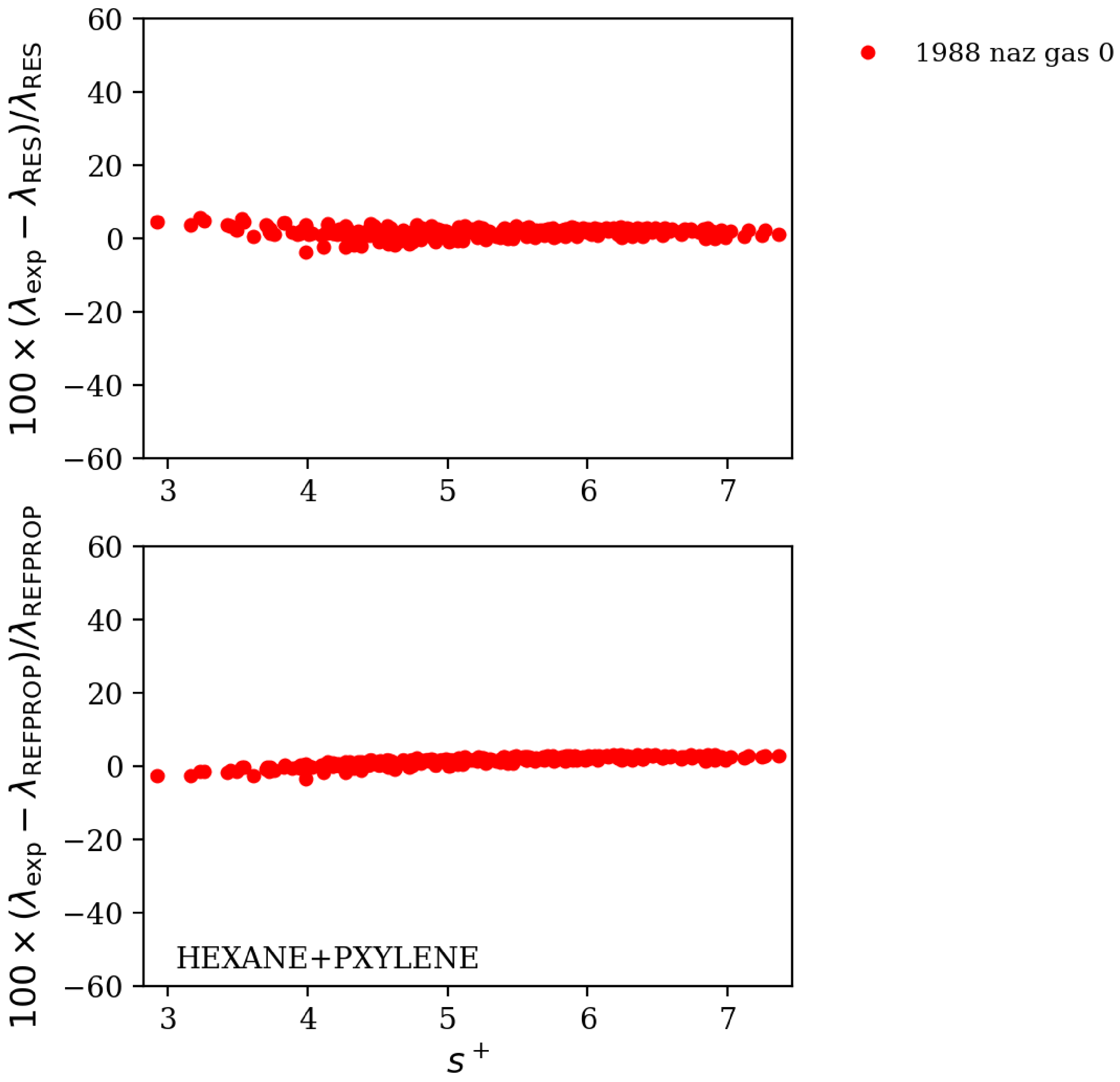

Figure DPR4. HEXANE+PXYLENE

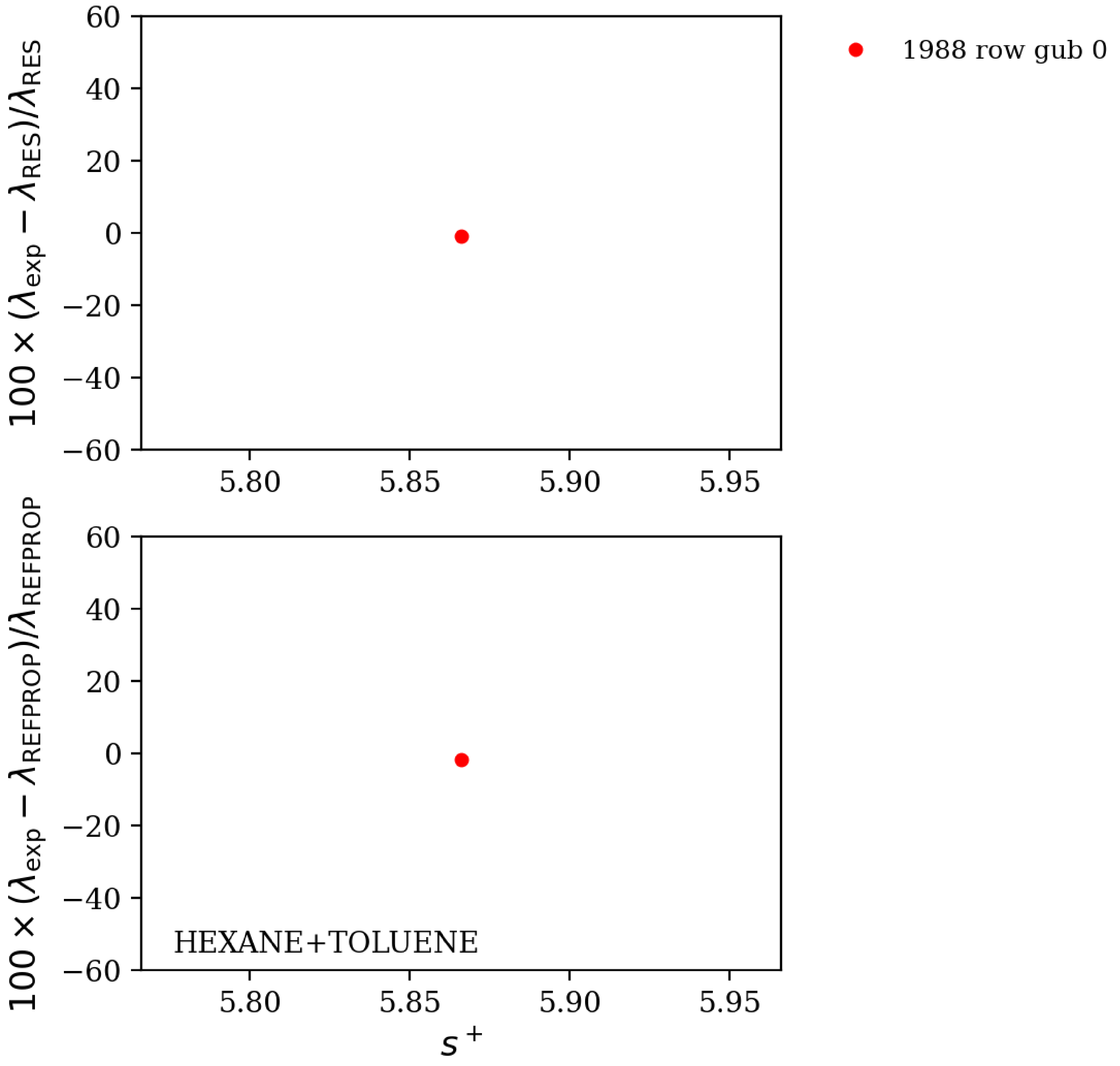

Figure DPR4. HEXANE+TOLUENE

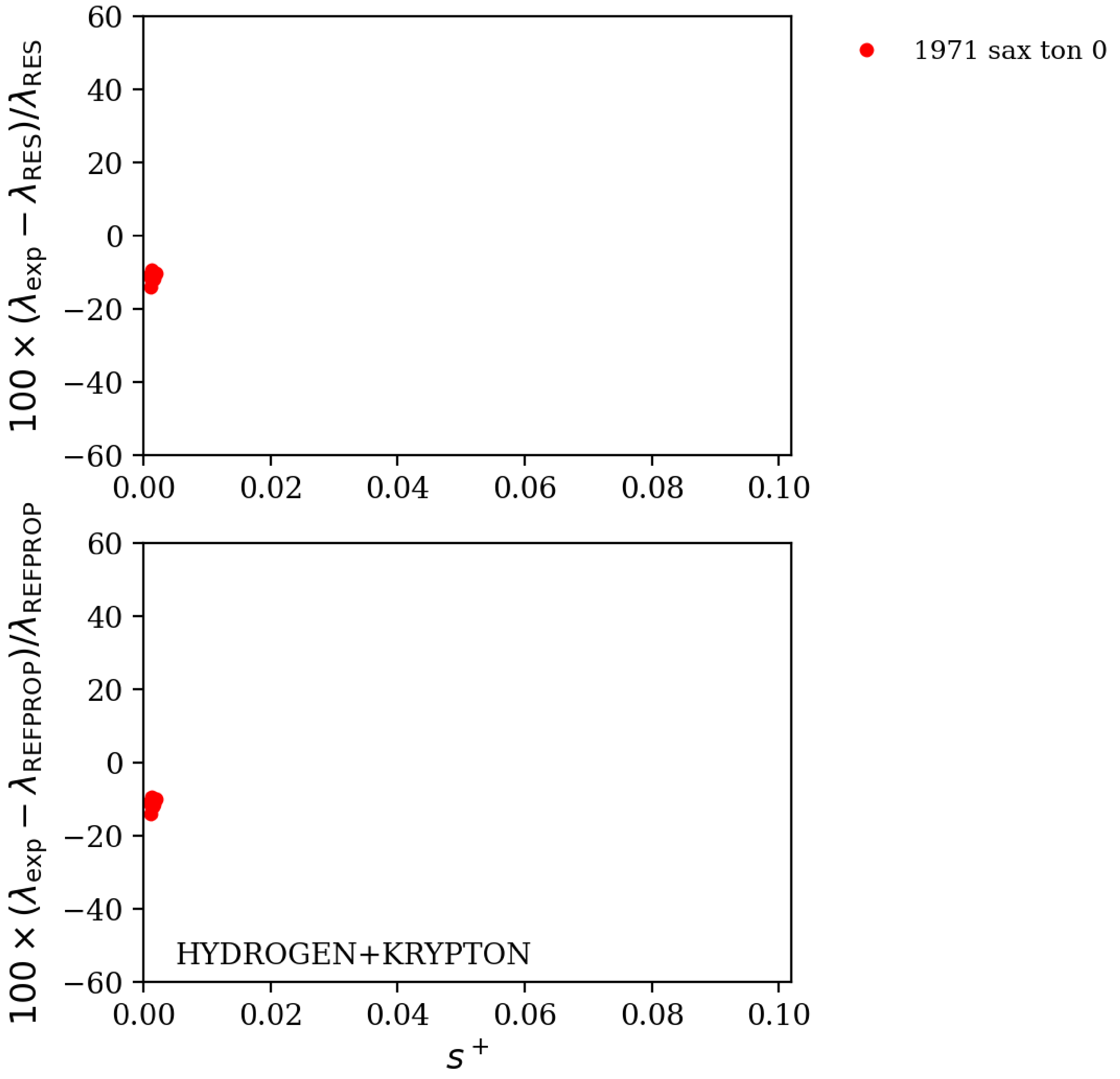

Figure DPR4. HYDROGEN+KRYPTON

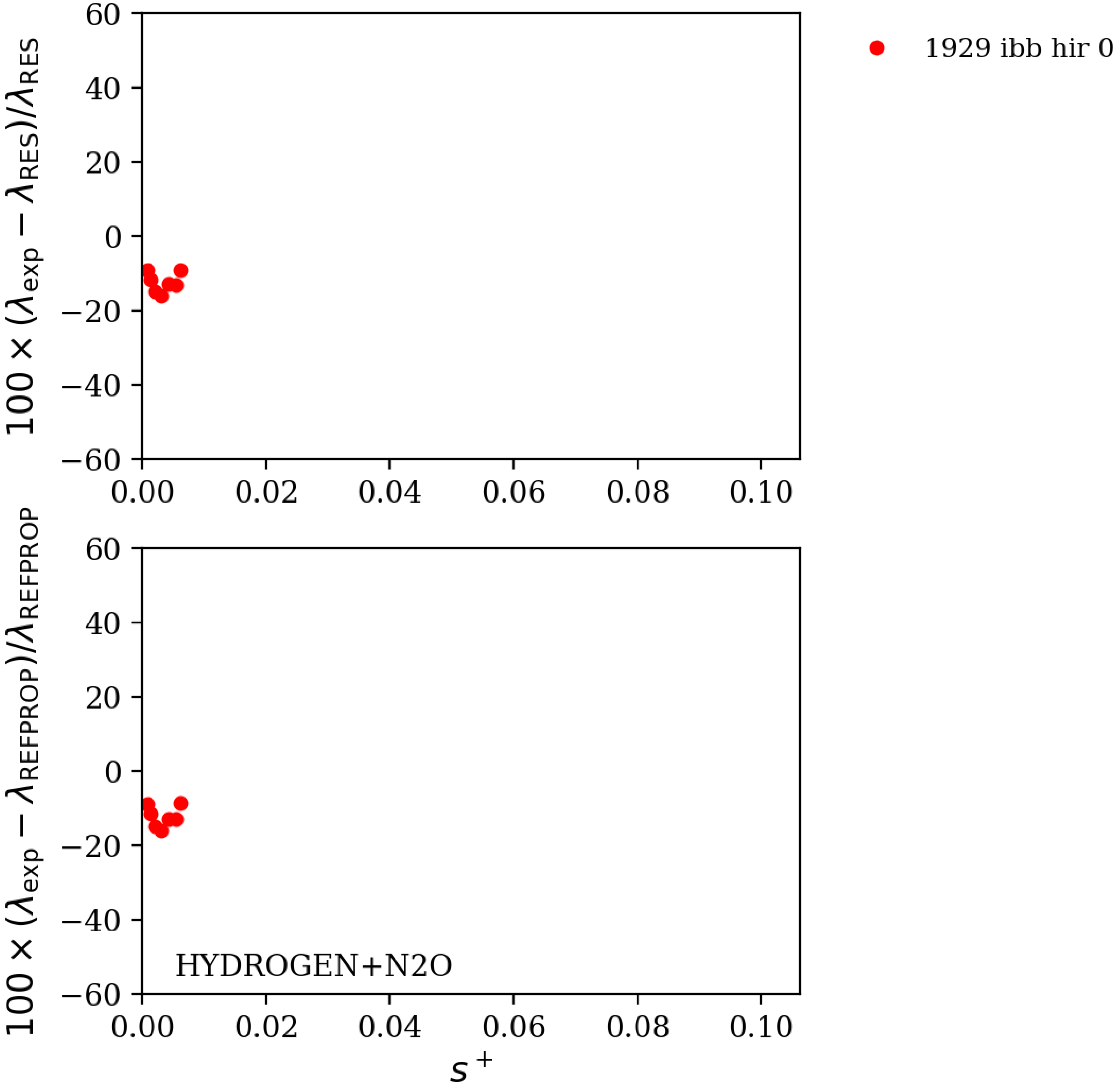

Figure DPR4. HYDROGEN+N2O

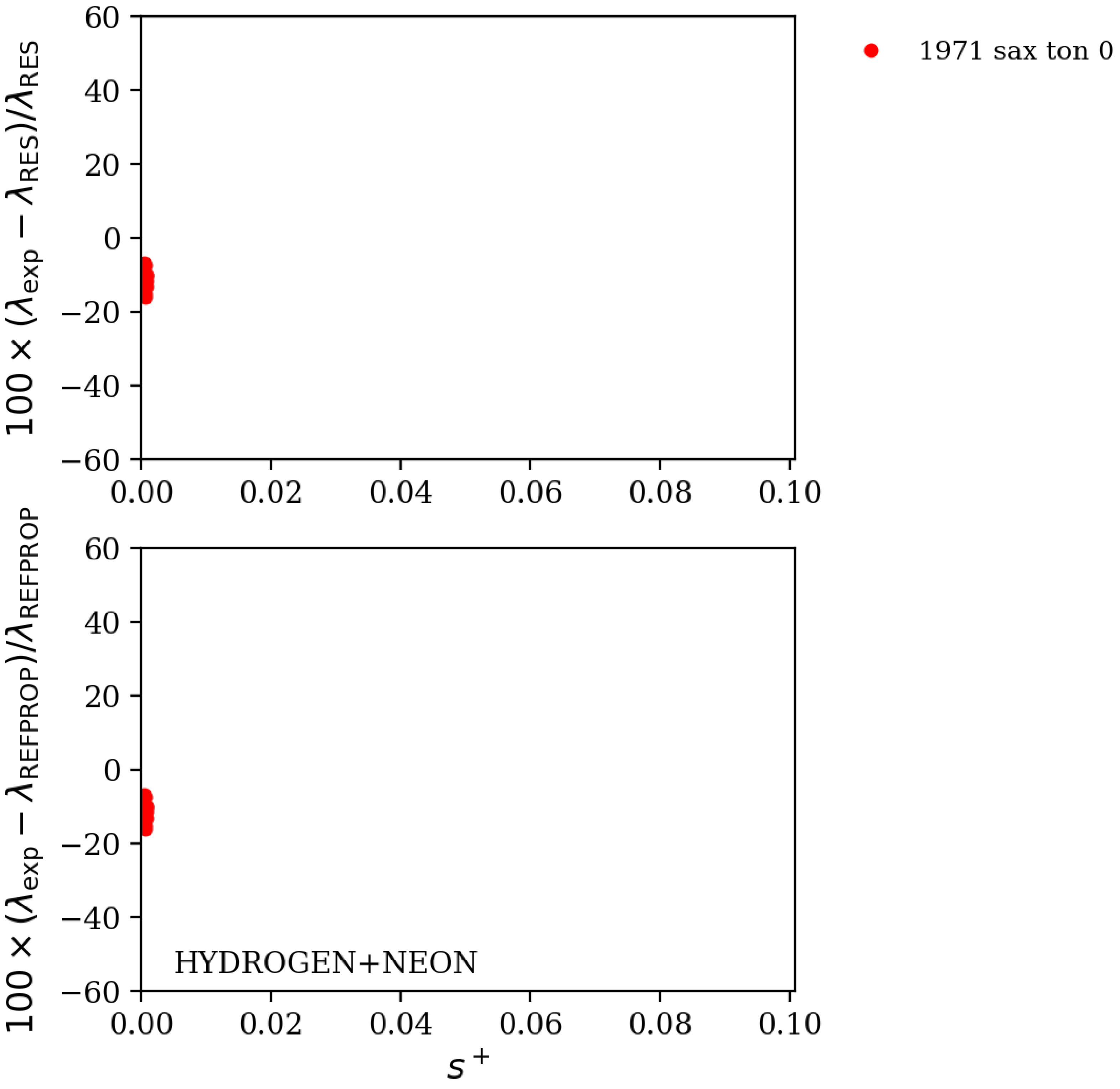

Figure DPR4. HYDROGEN+NEON

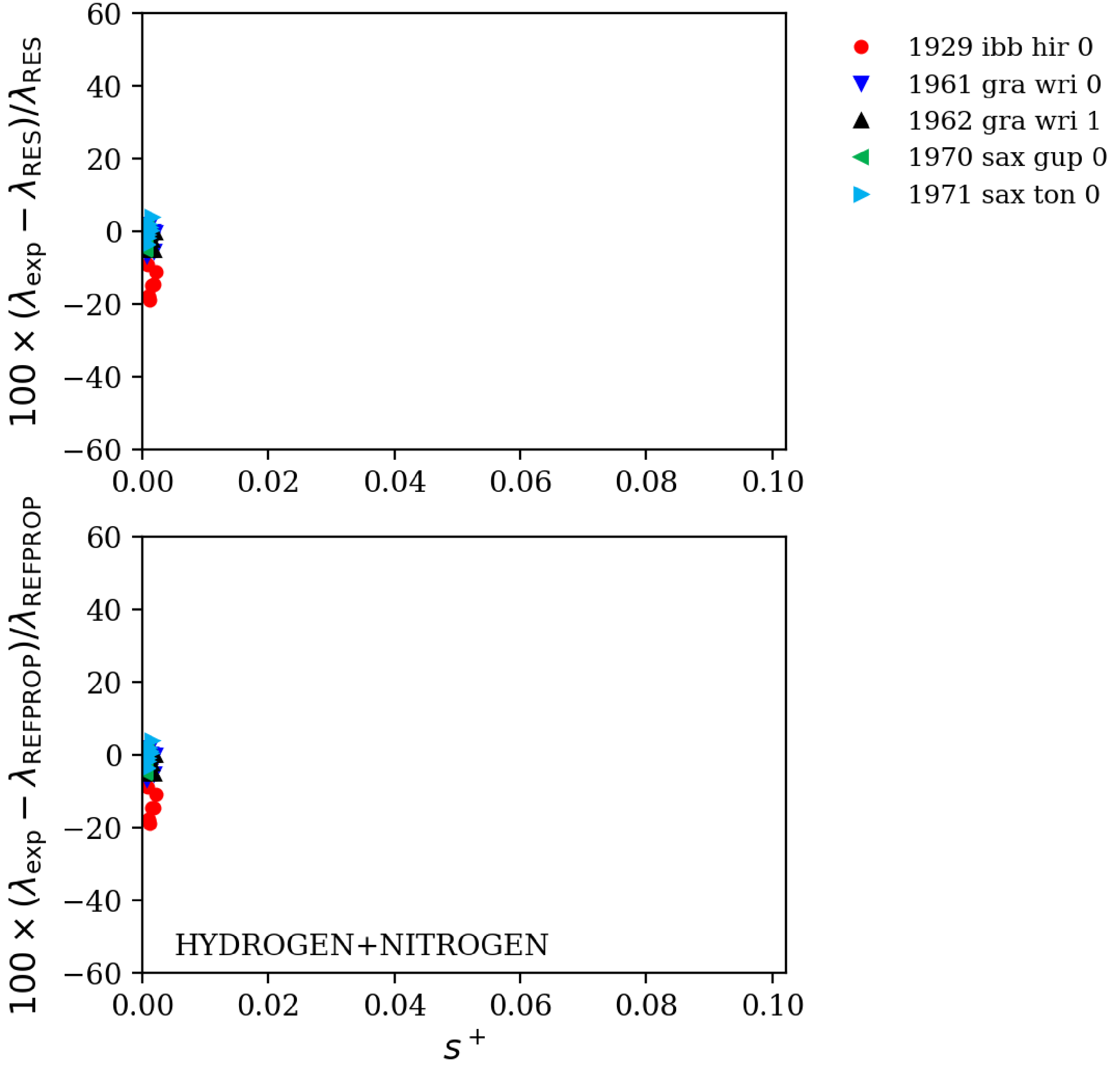

Figure DPR4. HYDROGEN+NITROGEN

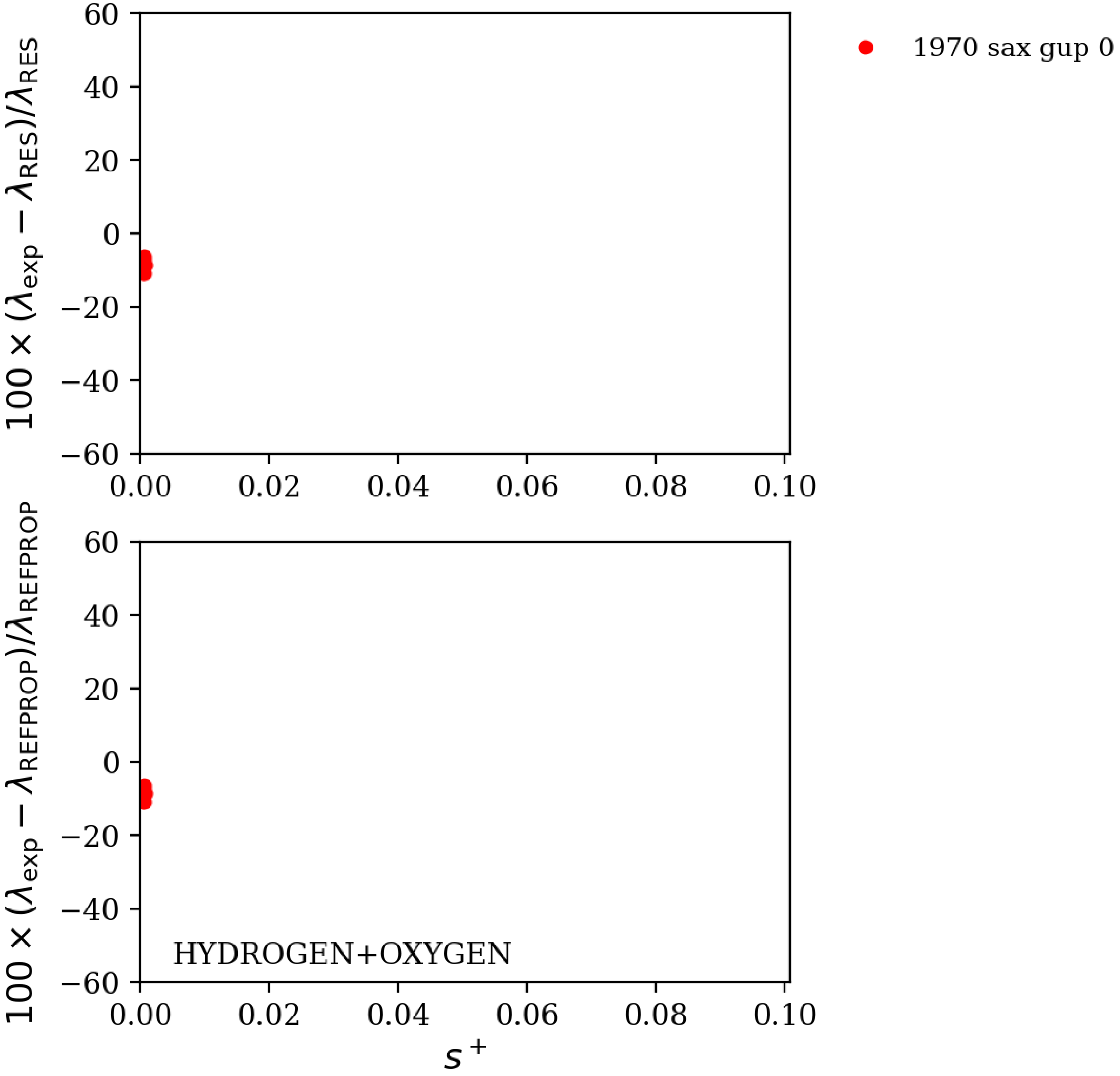

Figure DPR4. HYDROGEN+OXYGEN

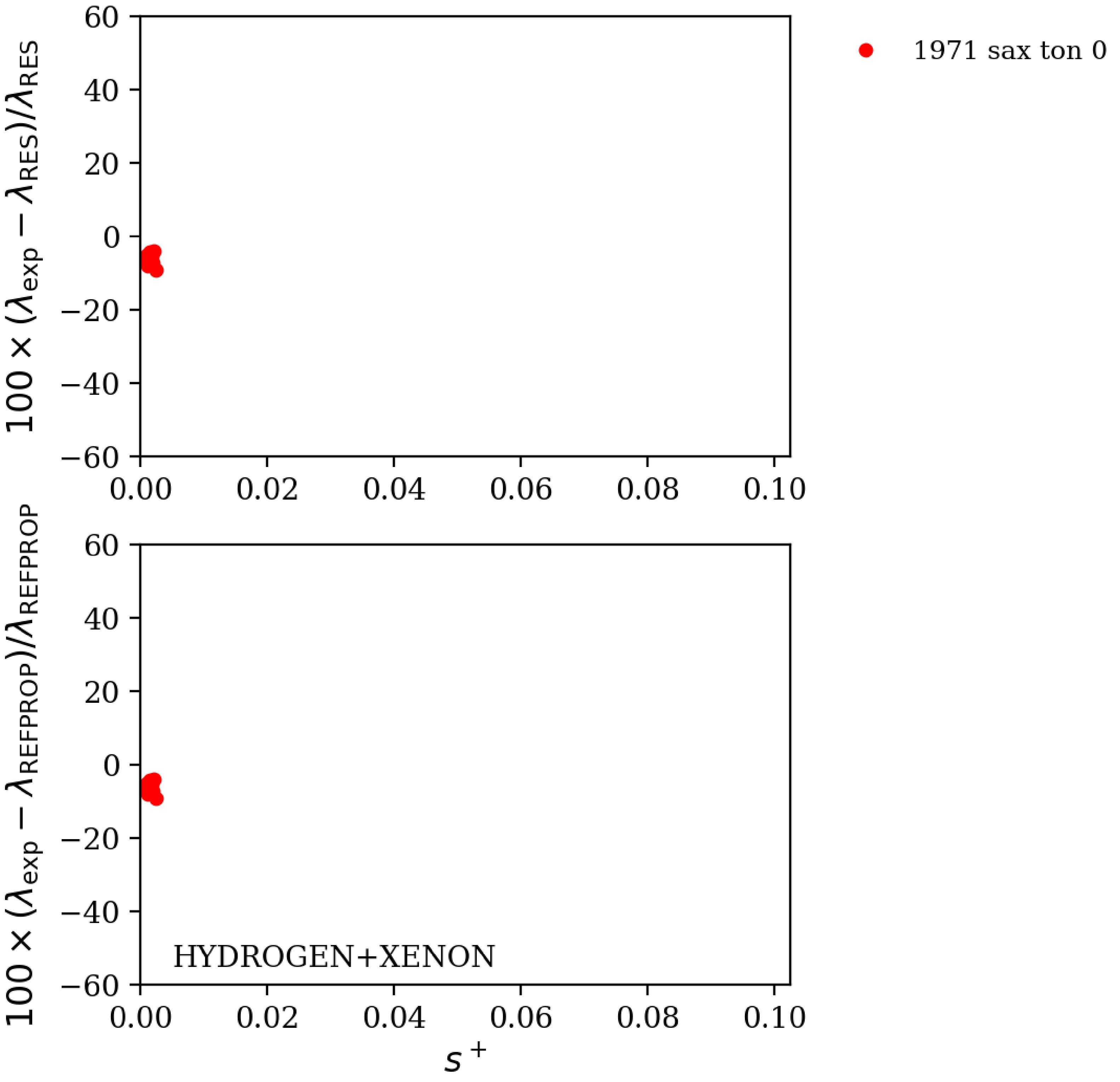

Figure DPR4. HYDROGEN+XENON

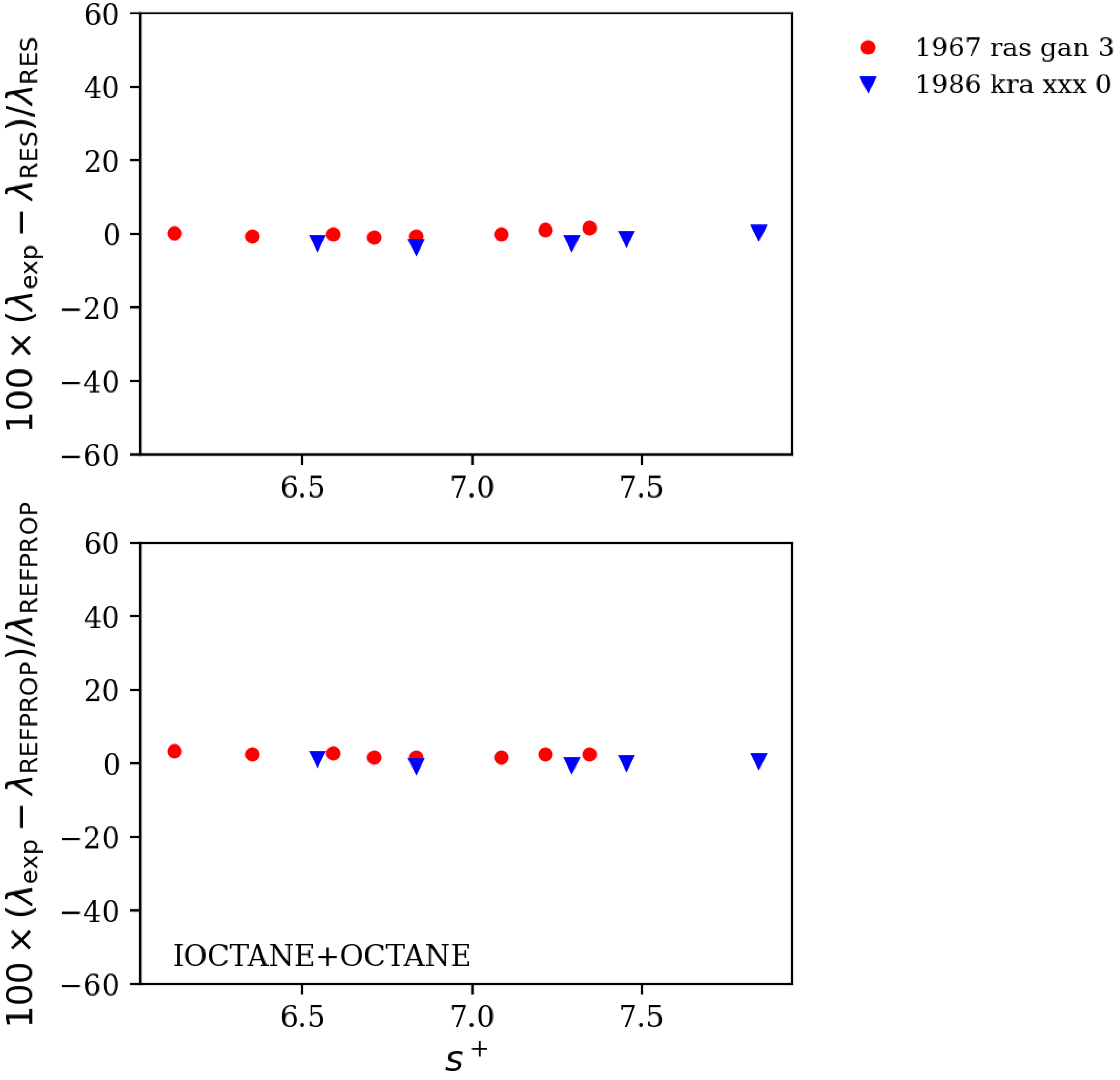

Figure DPR4. IOCTANE+OCTANE

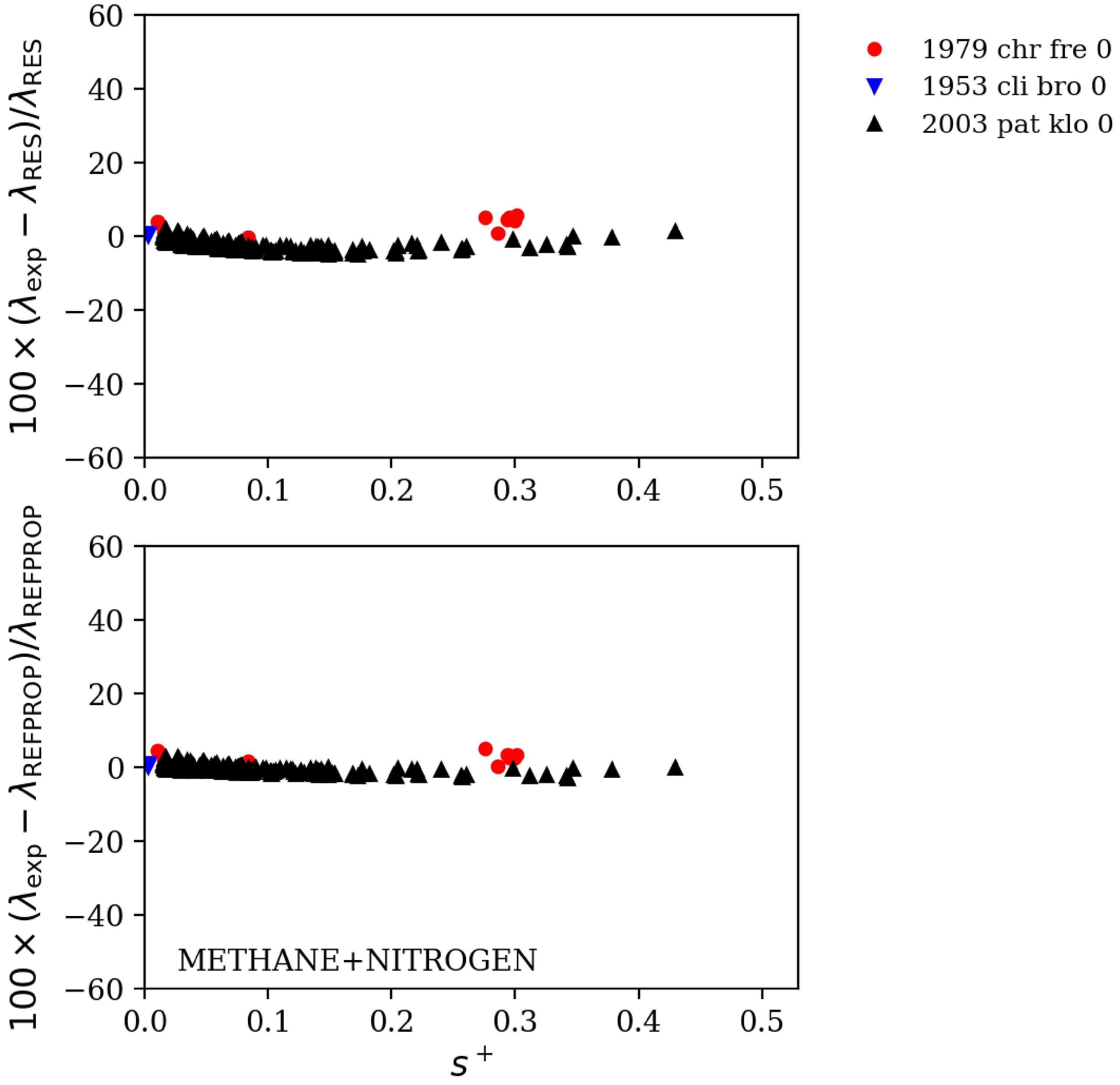

Figure DPR4. METHANE+NITROGEN

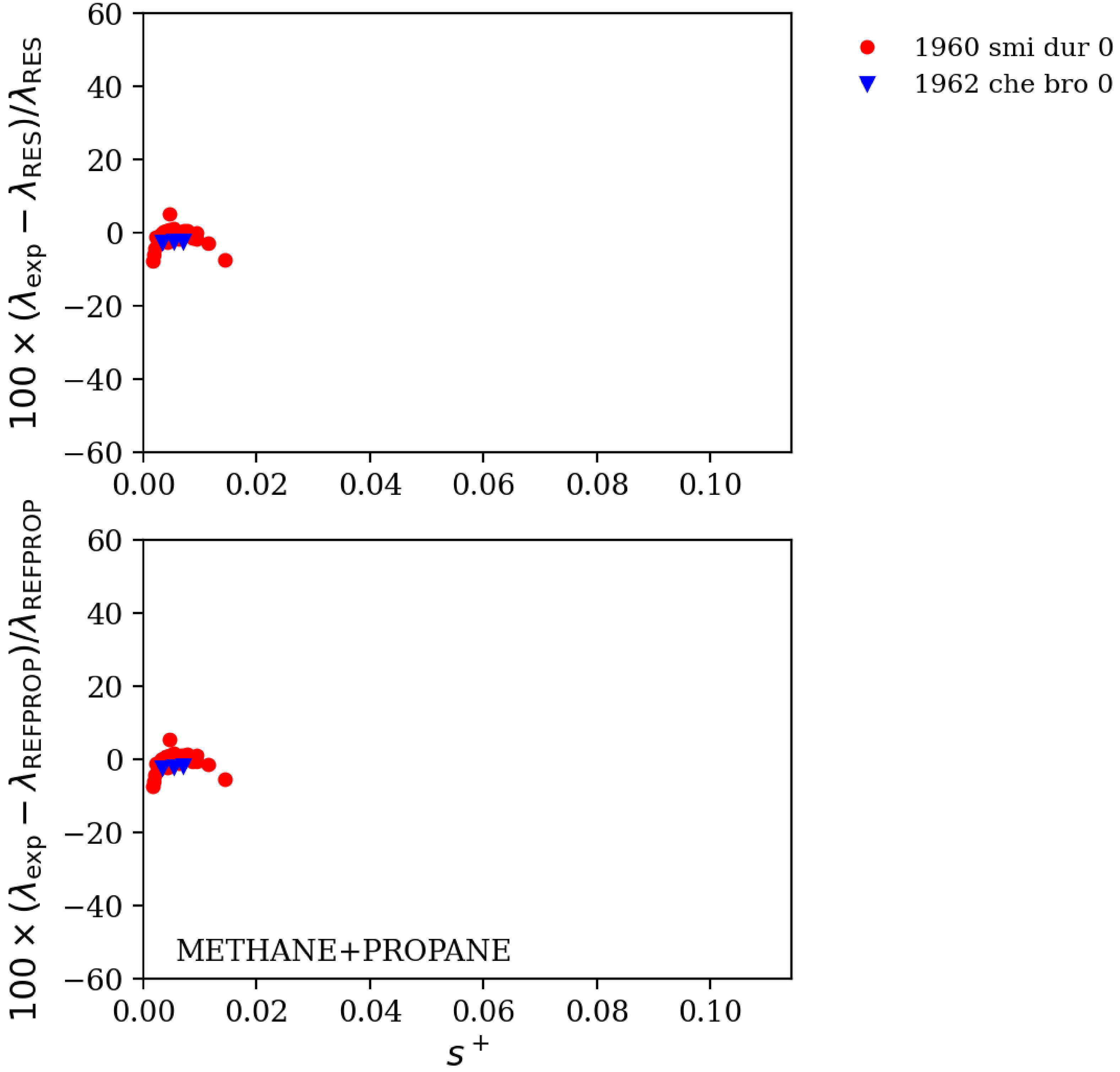

Figure DPR4. METHANE+PROPANE

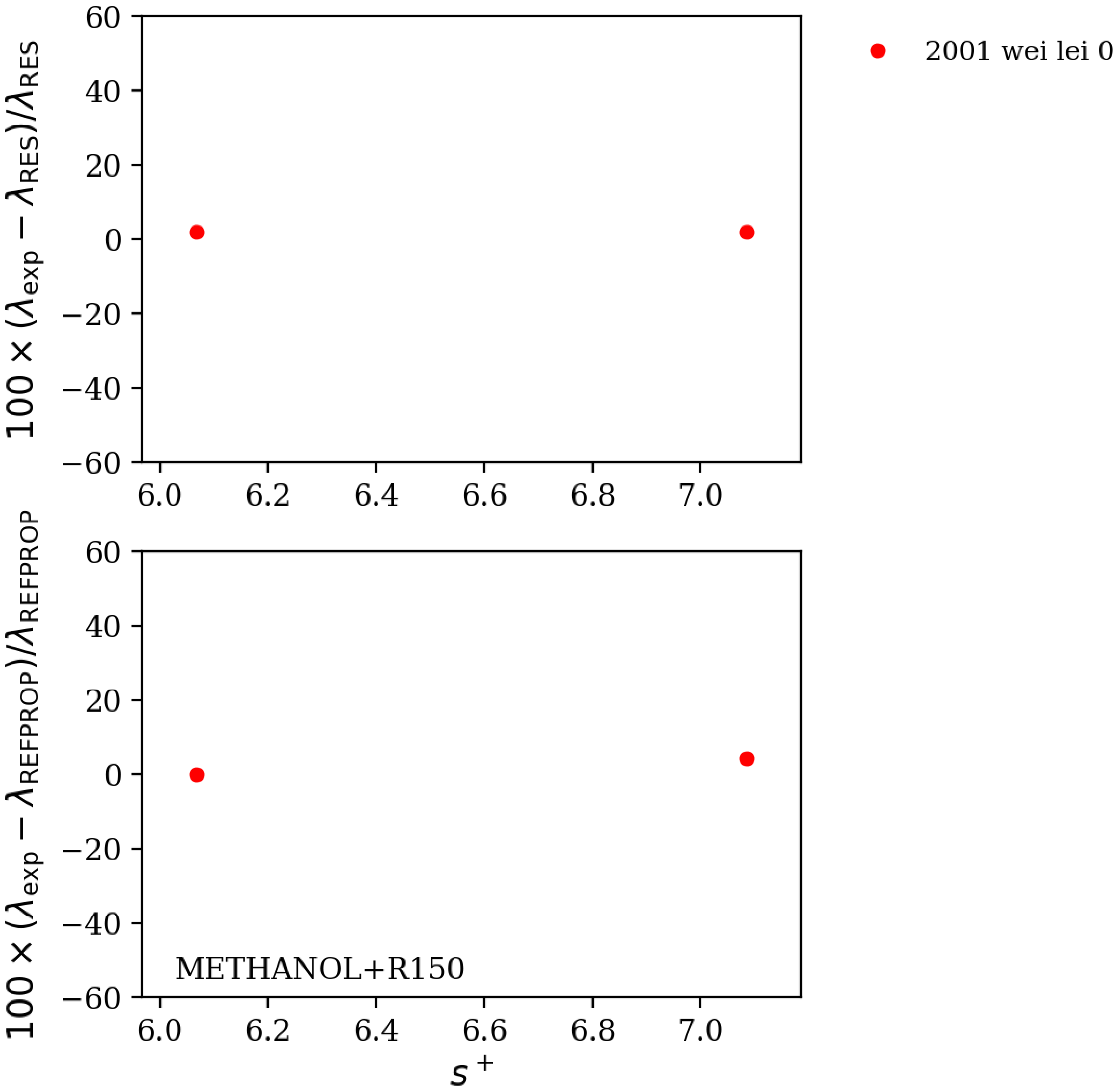

Figure DPR4. METHANOL+R150

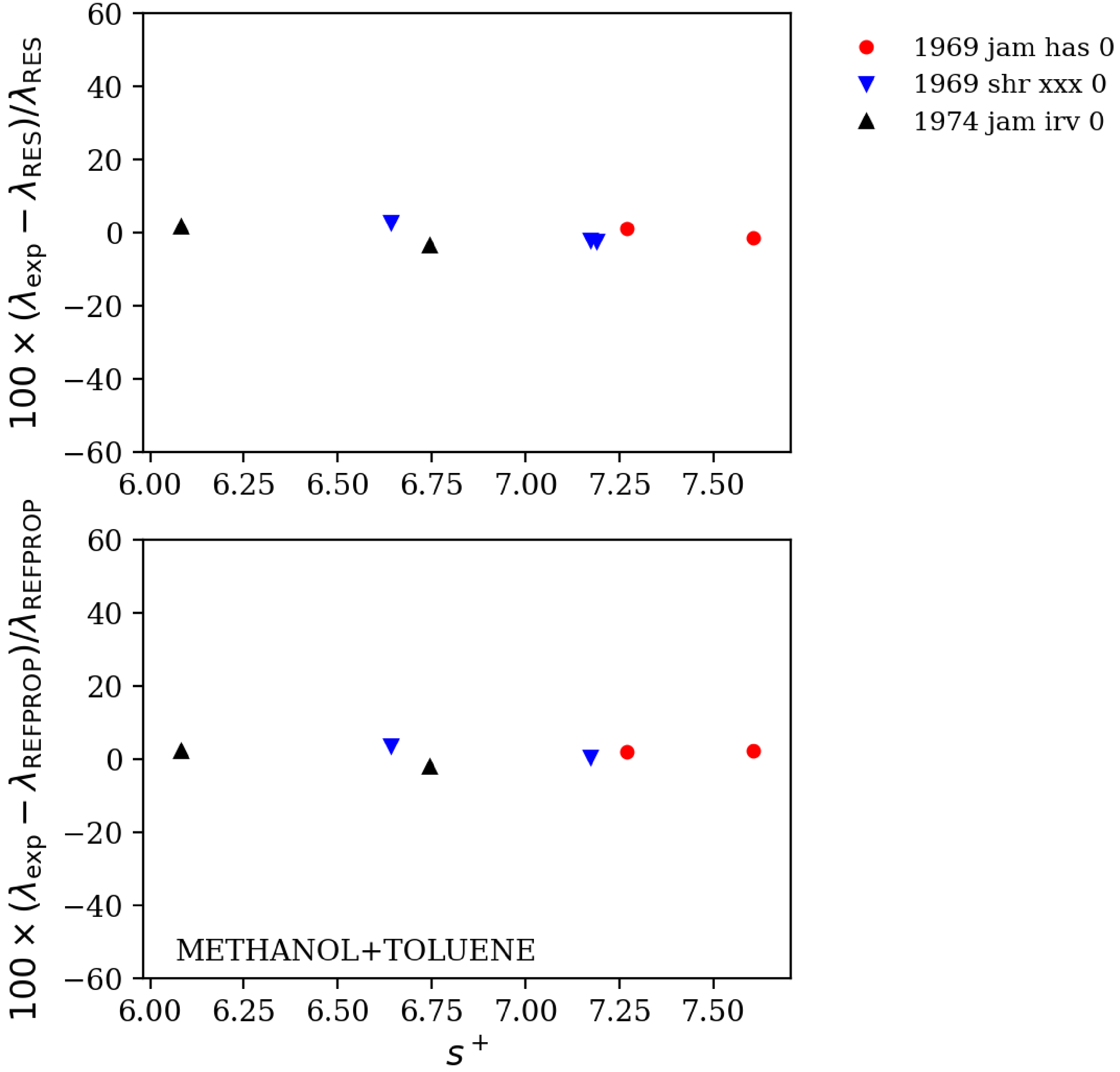

Figure DPR4. METHANOL+TOLUENE

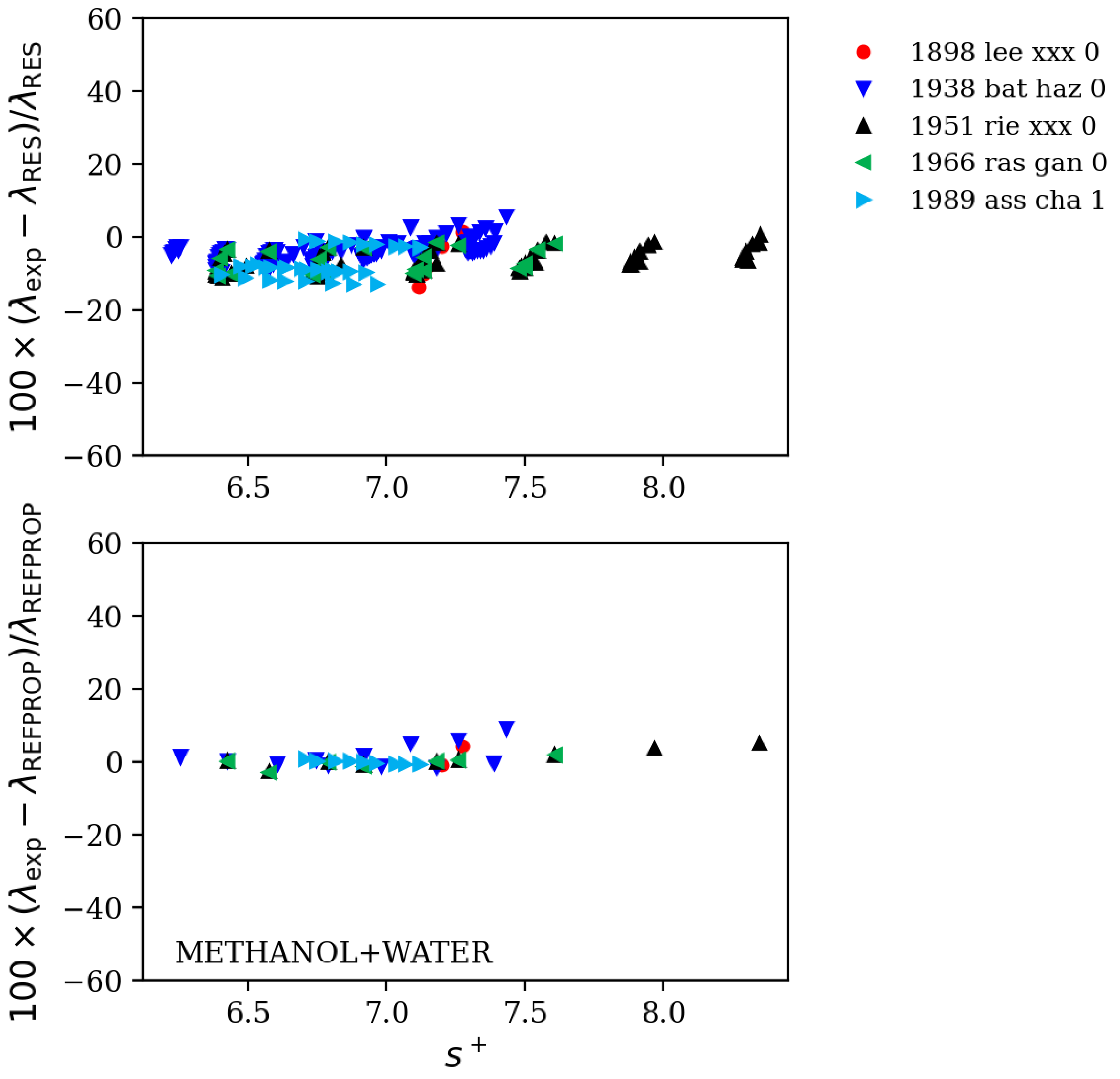

Figure DPR4. METHANOL+WATER

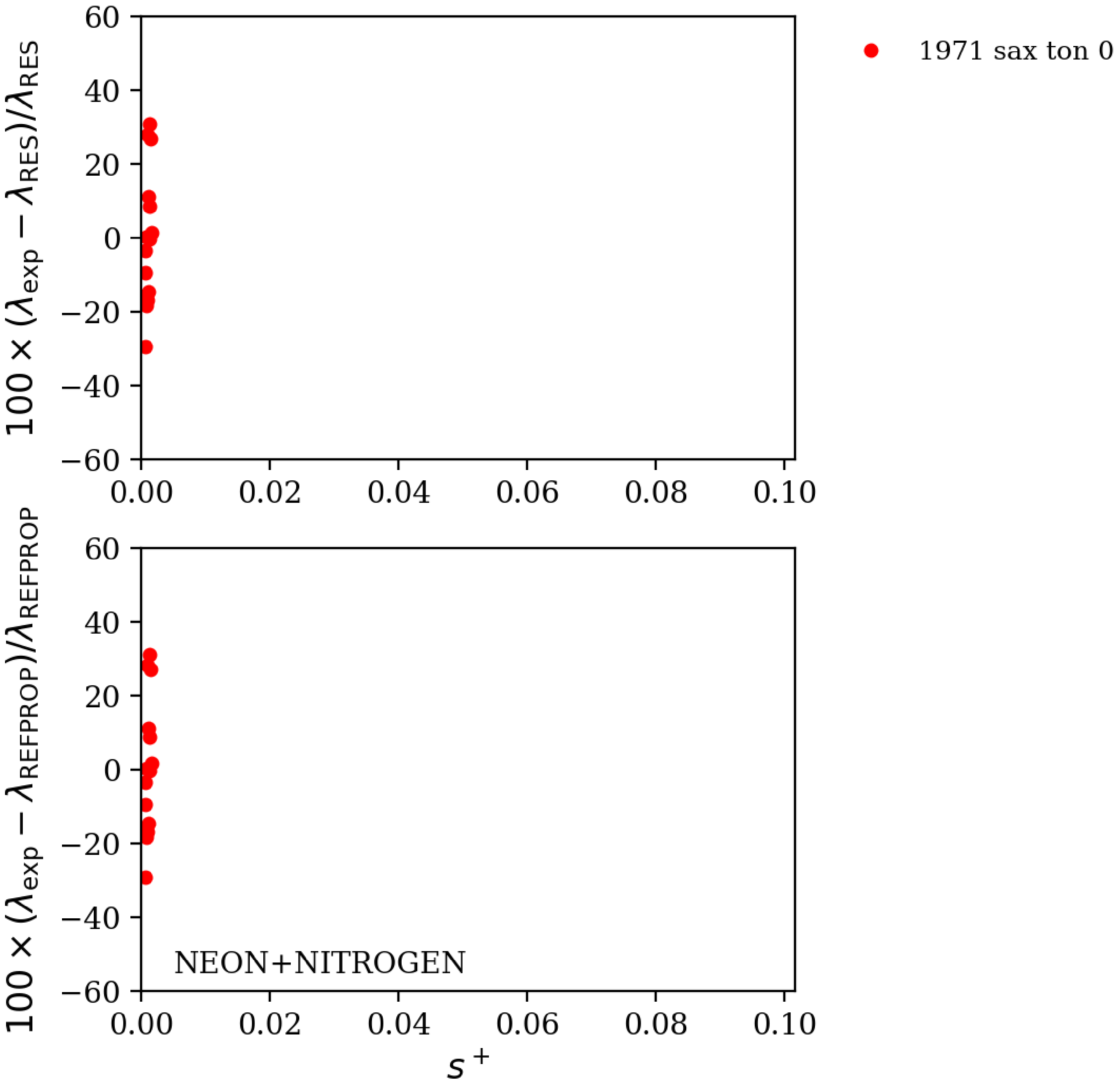

Figure DPR4. NEON+NITROGEN

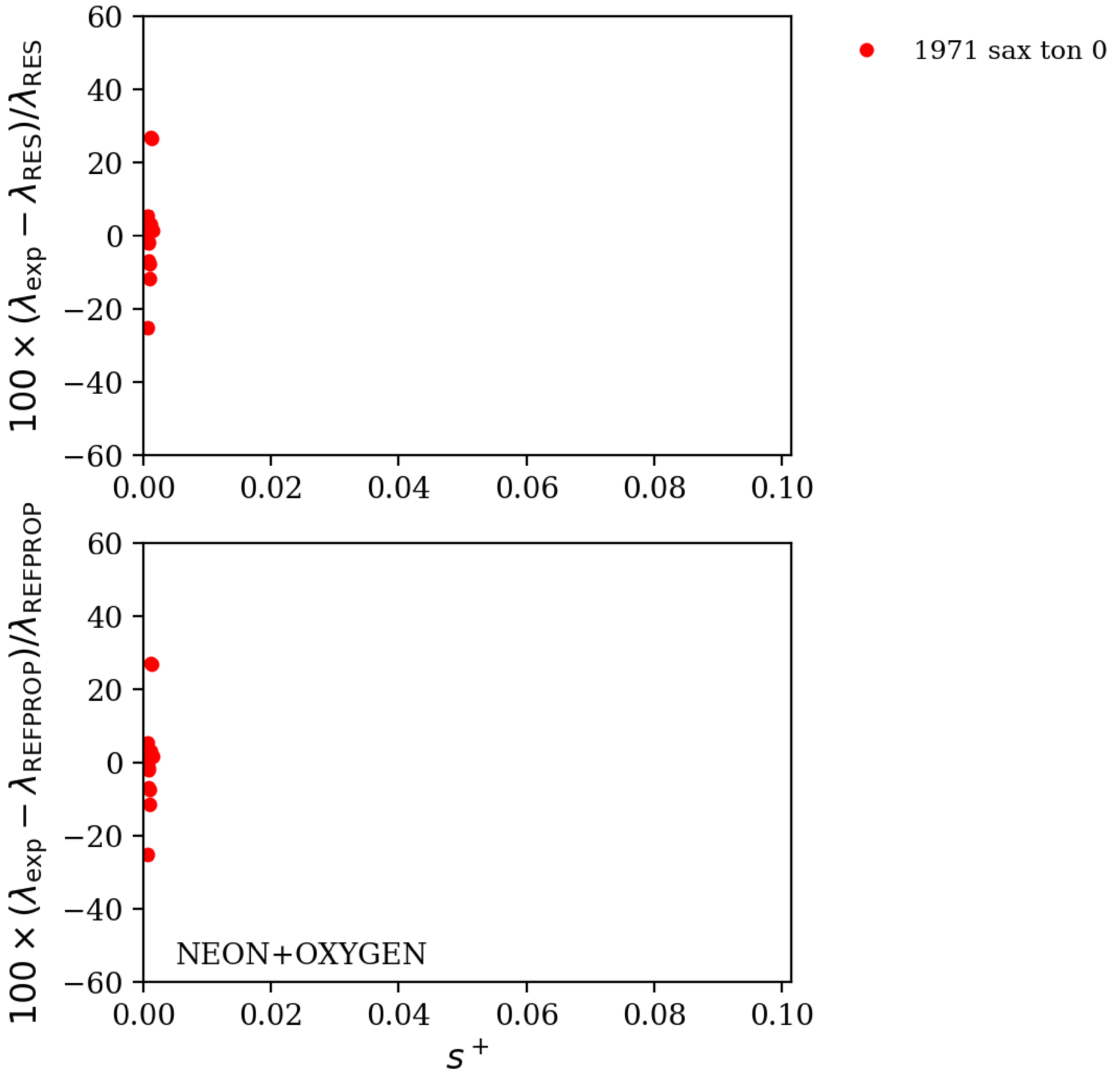

Figure DPR4. NEON+OXYGEN

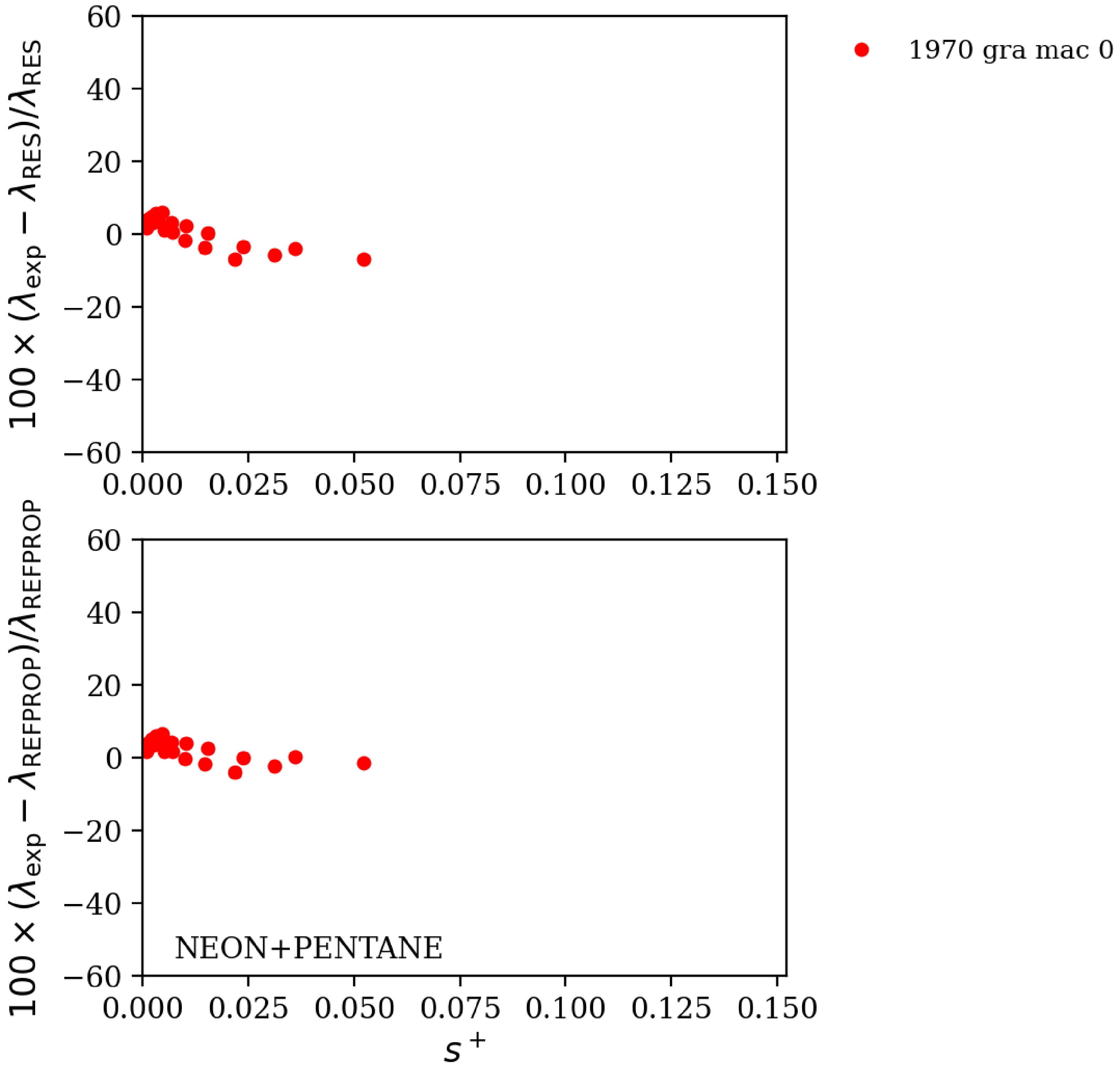

Figure DPR4. NEON+PENTANE

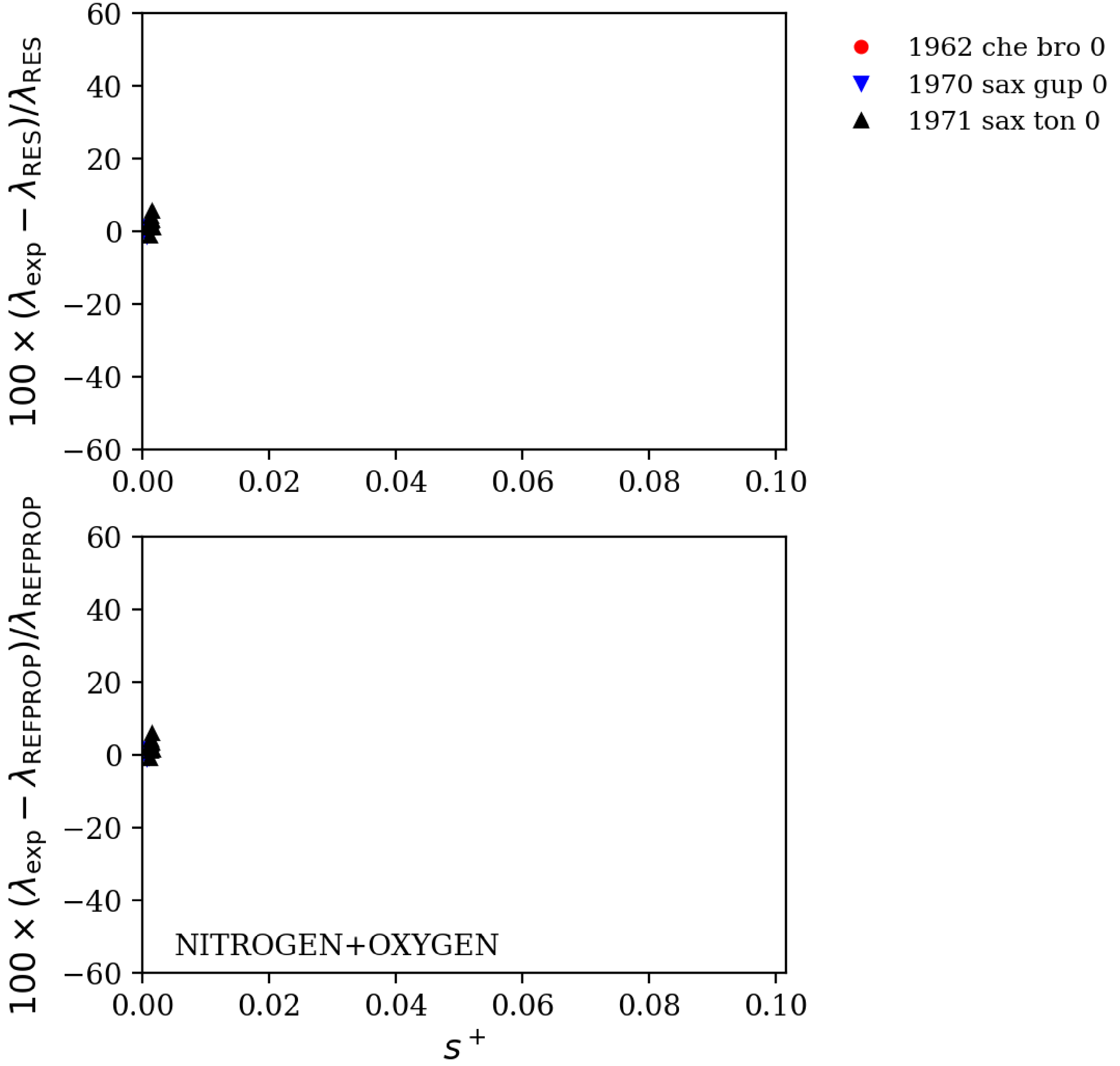

Figure DPR4. NITROGEN+OXYGEN

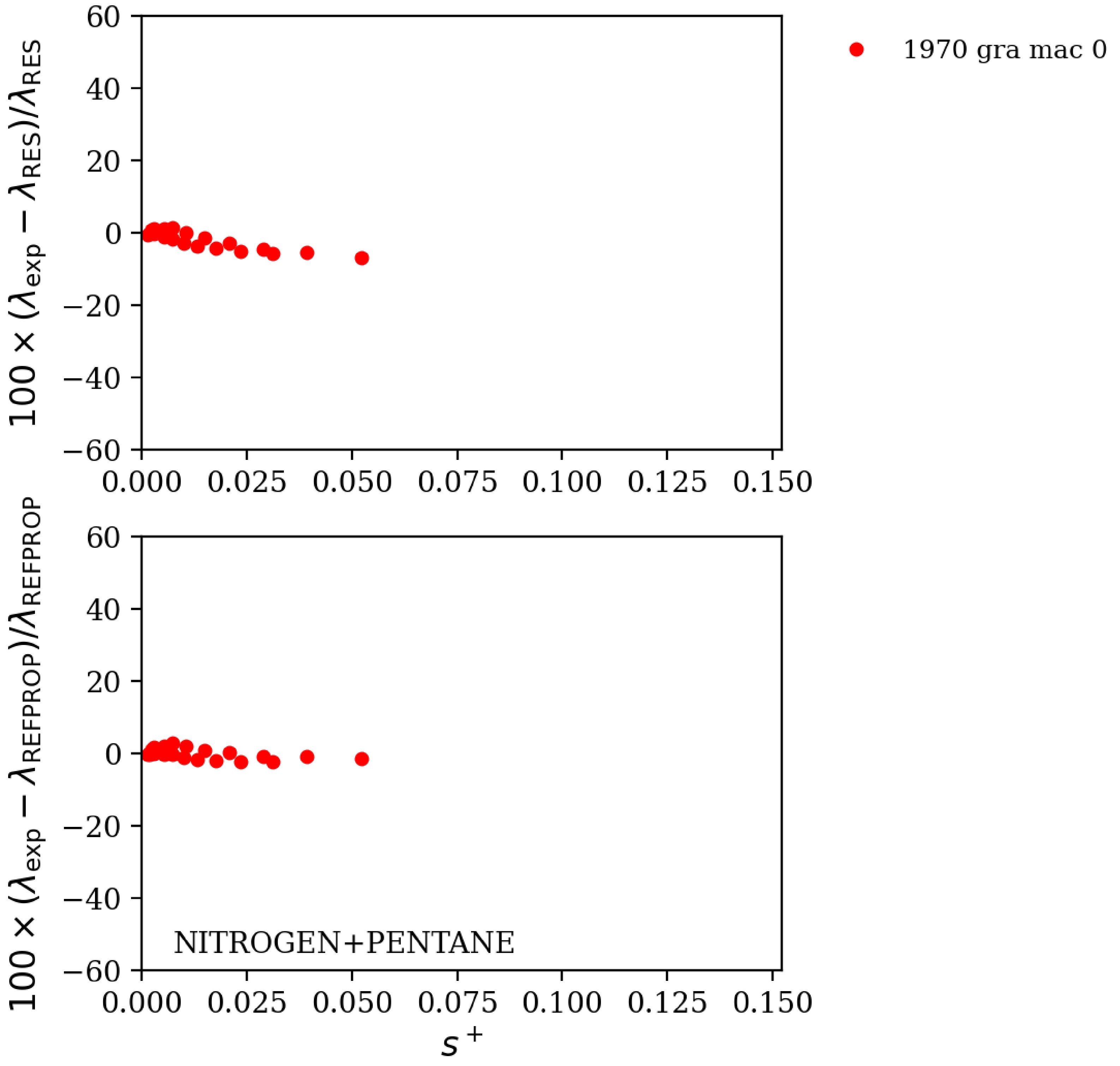

Figure DPR4. NITROGEN+PENTANE

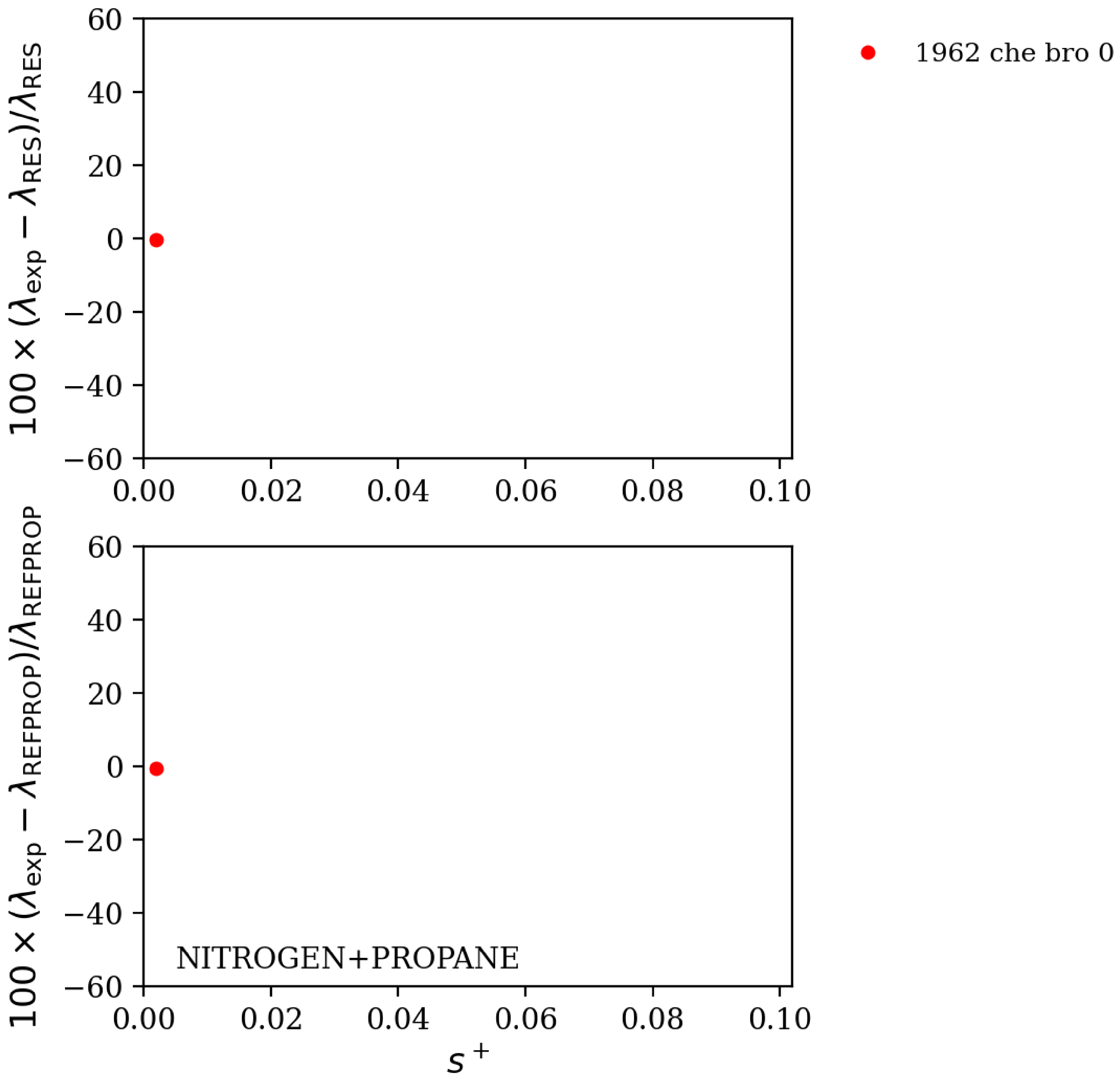

Figure DPR4. NITROGEN+PROPANE

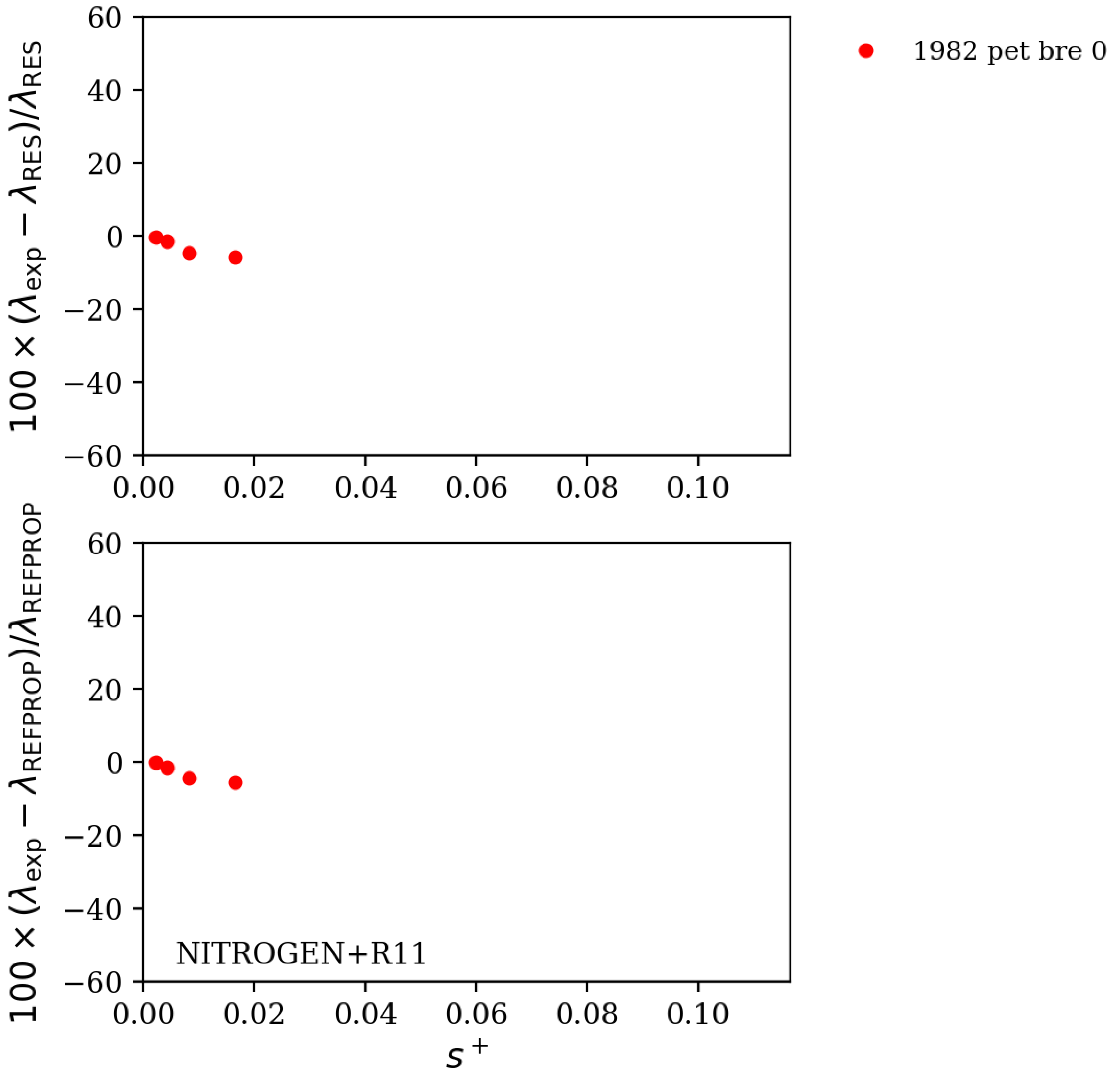

Figure DPR4. NITROGEN+R11

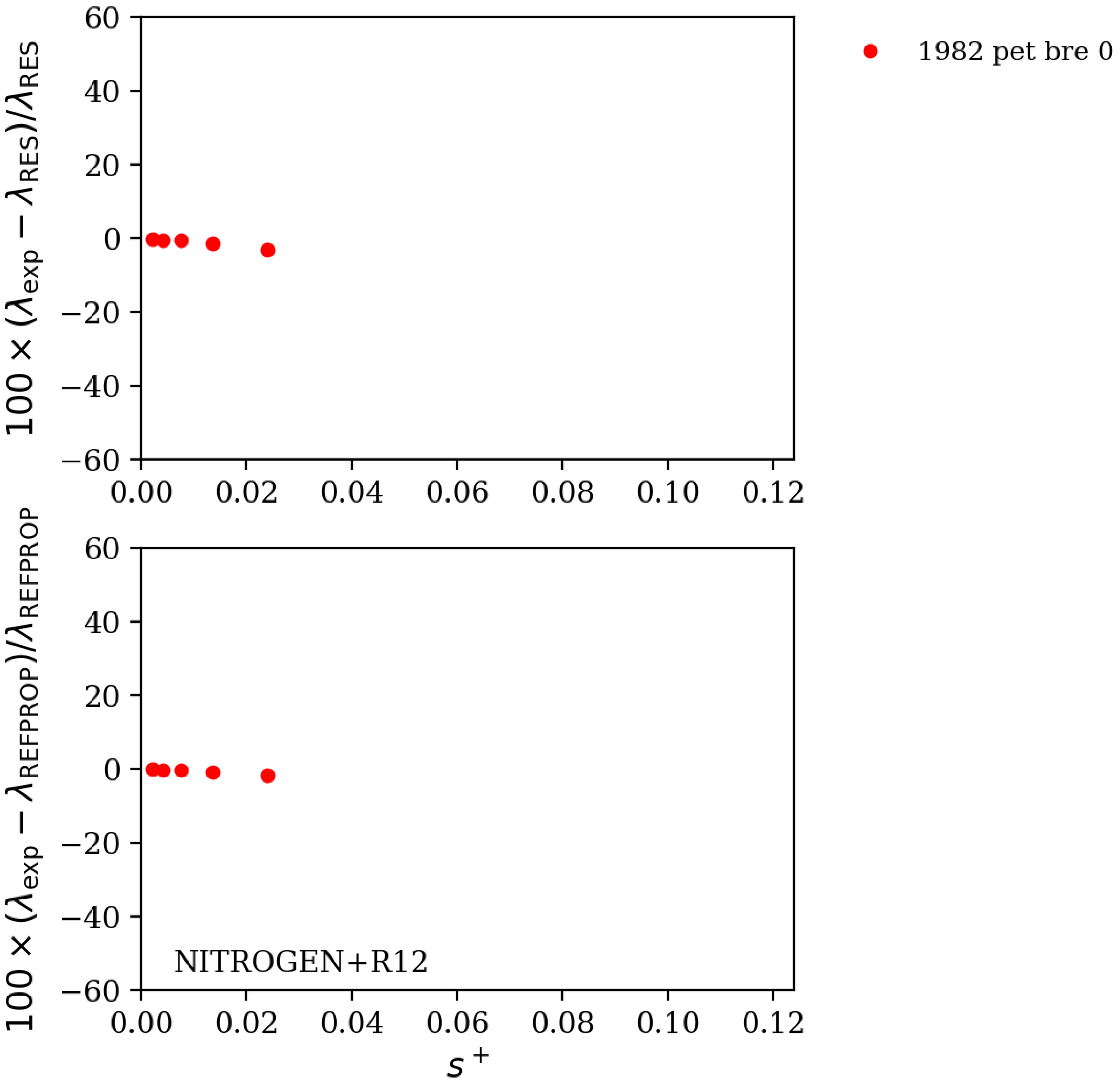

Figure DPR4. NITROGEN+R12

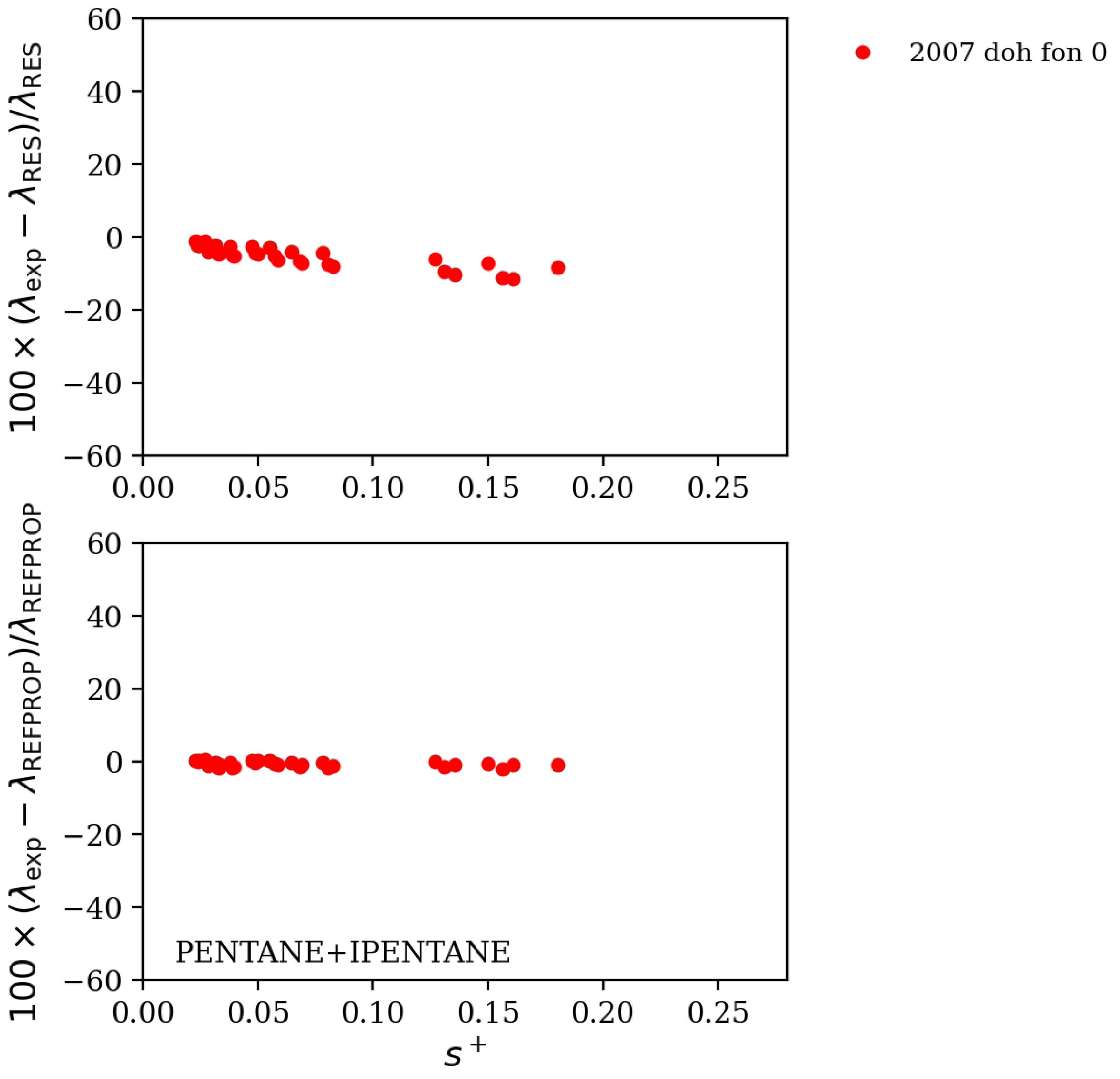

Figure DPR4. PENTANE+IPENTANE

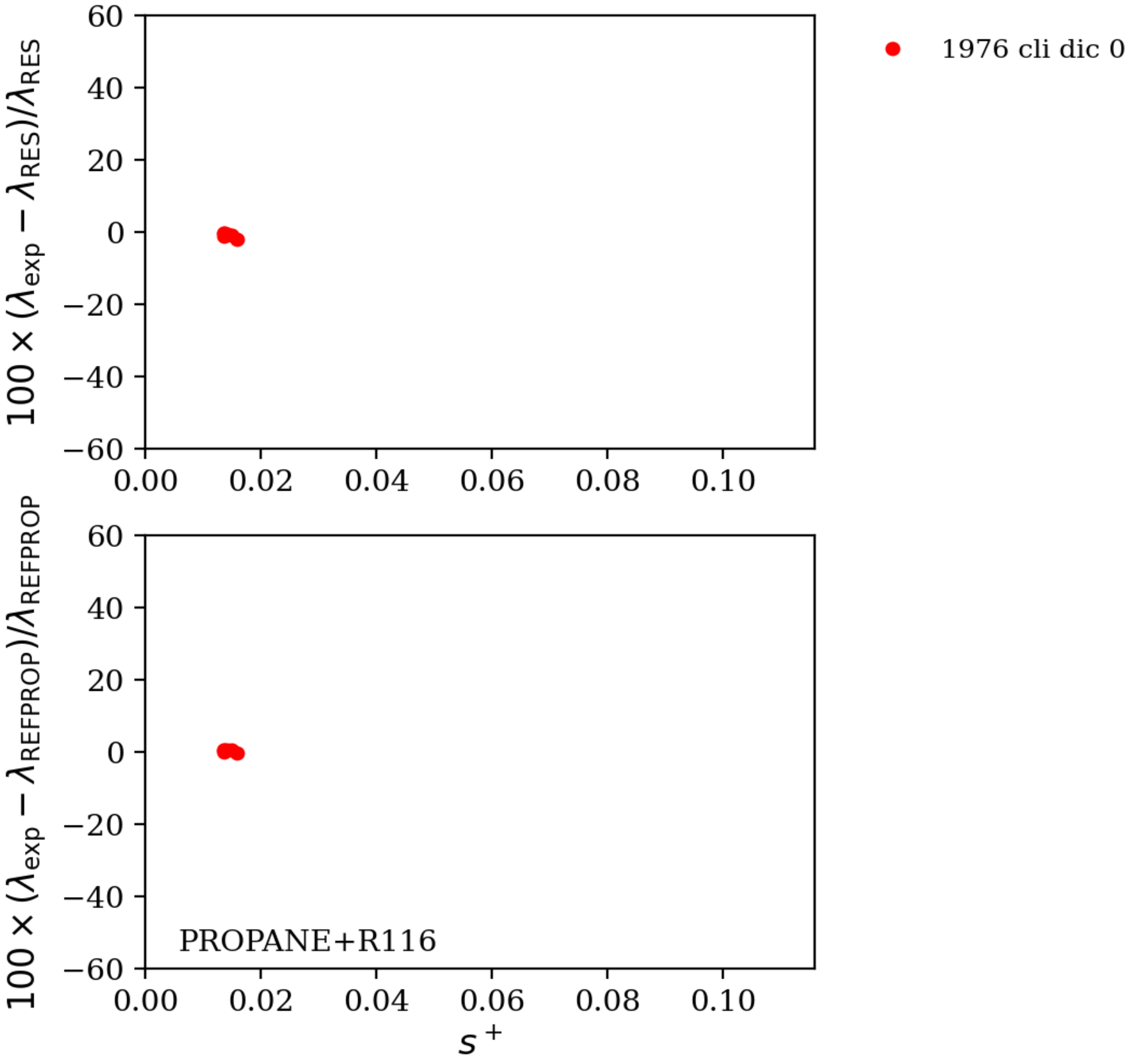

Figure DPR4.PROPANE+R116

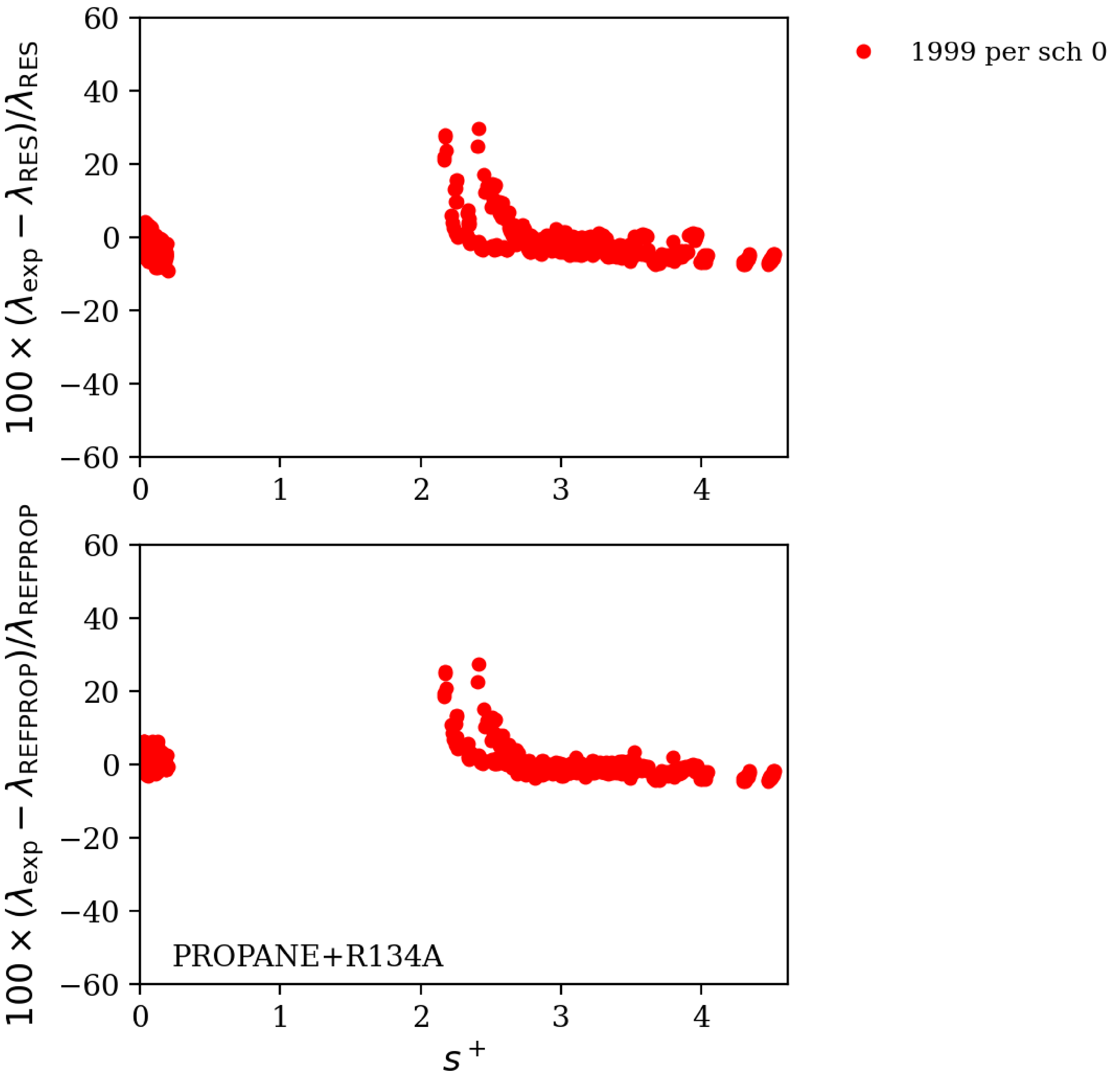

Figure DPR4. PROPANE+R134A

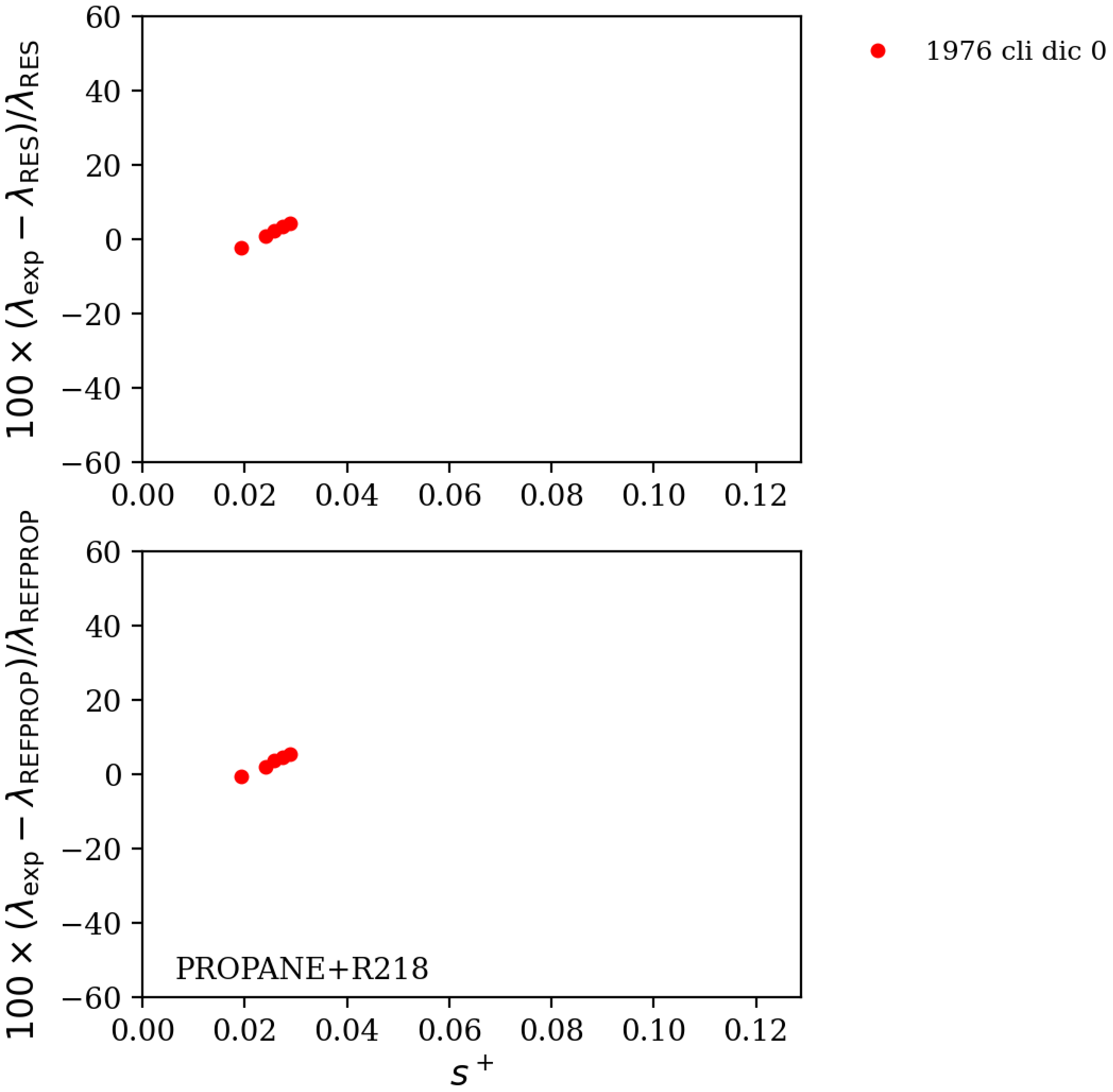

Figure DPR4. PROPANE+R218

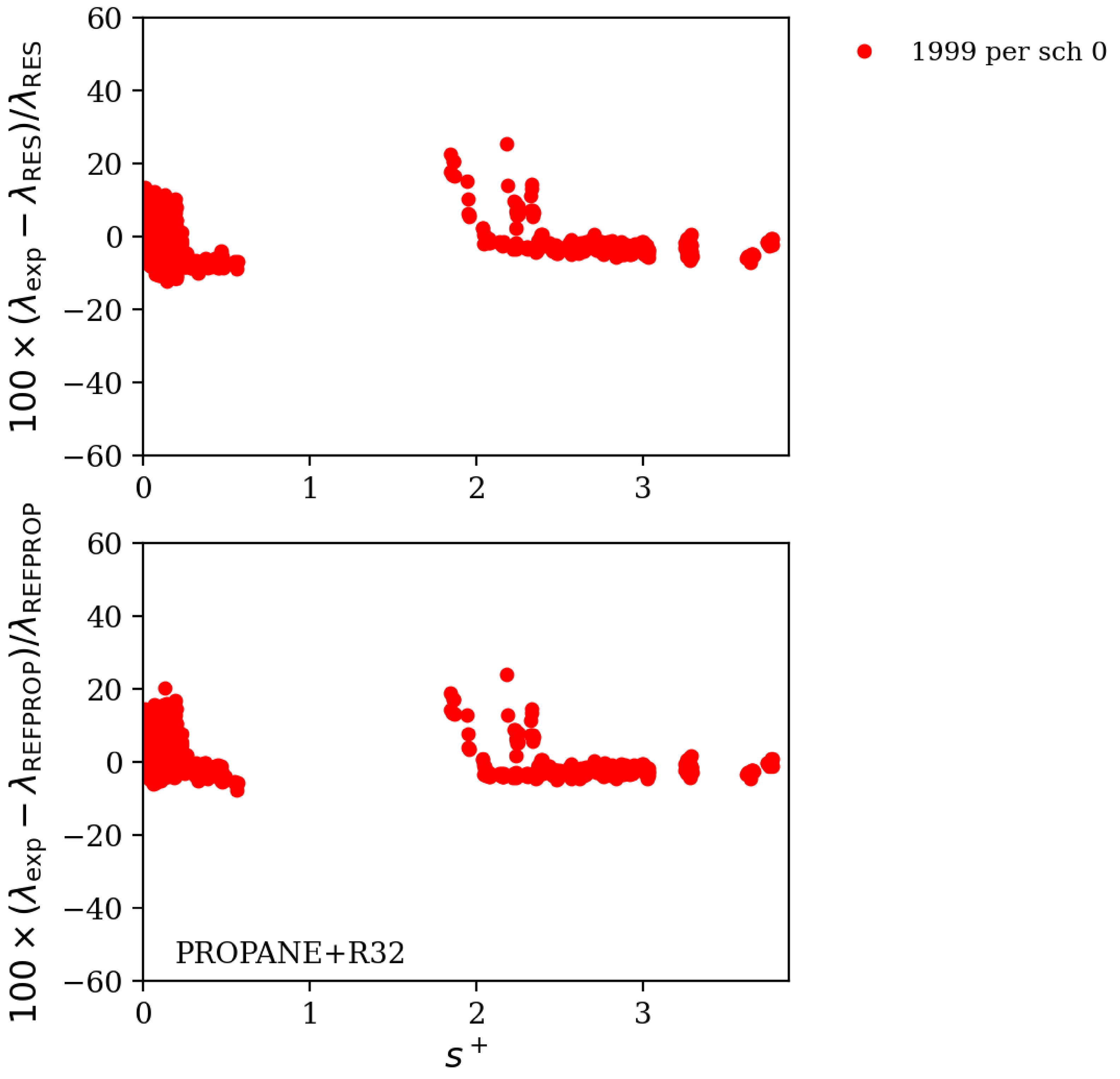

Figure DPR4. PROPANE+R32

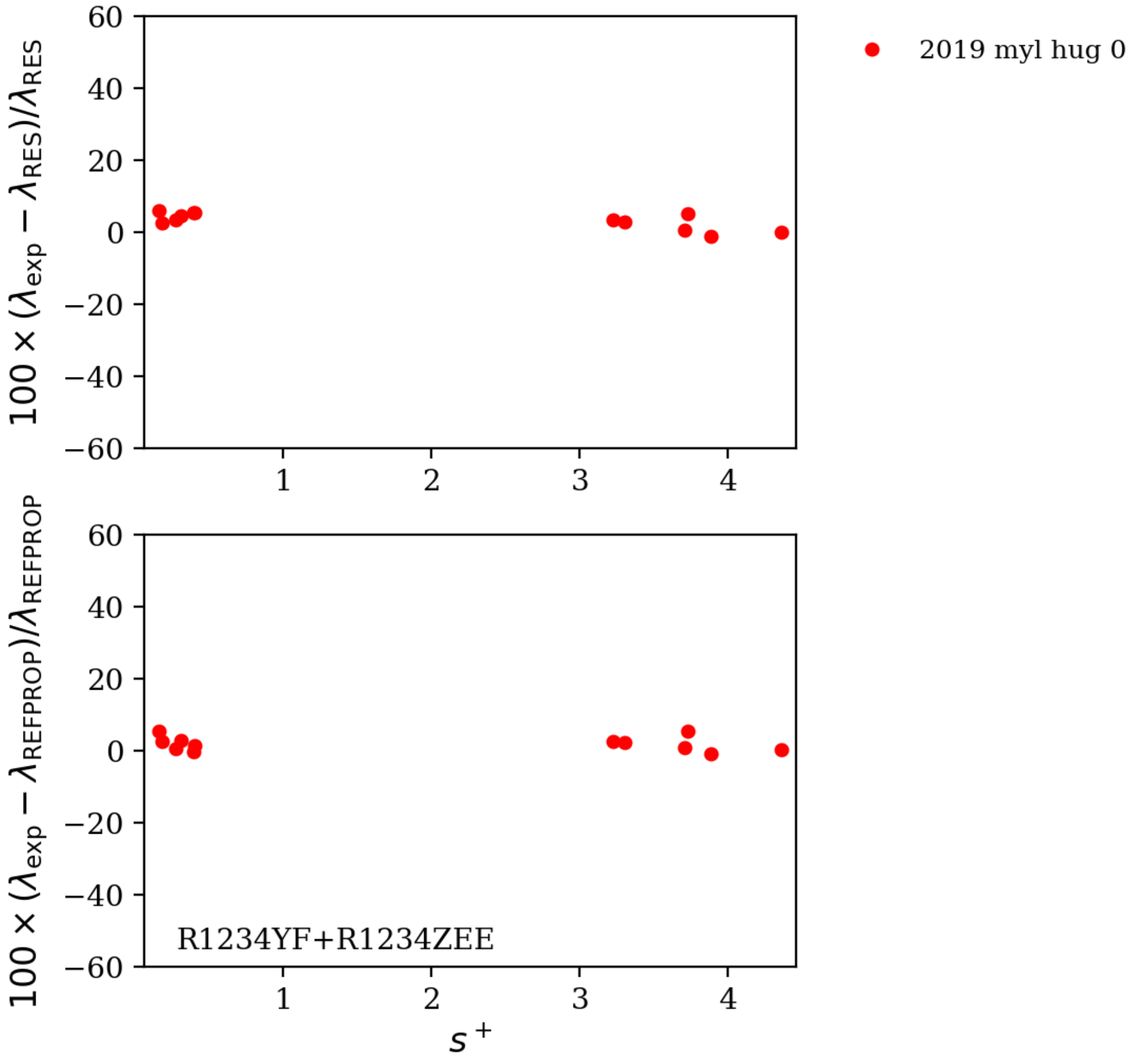

Figure DPR4. R1234YF+R1234ZEE

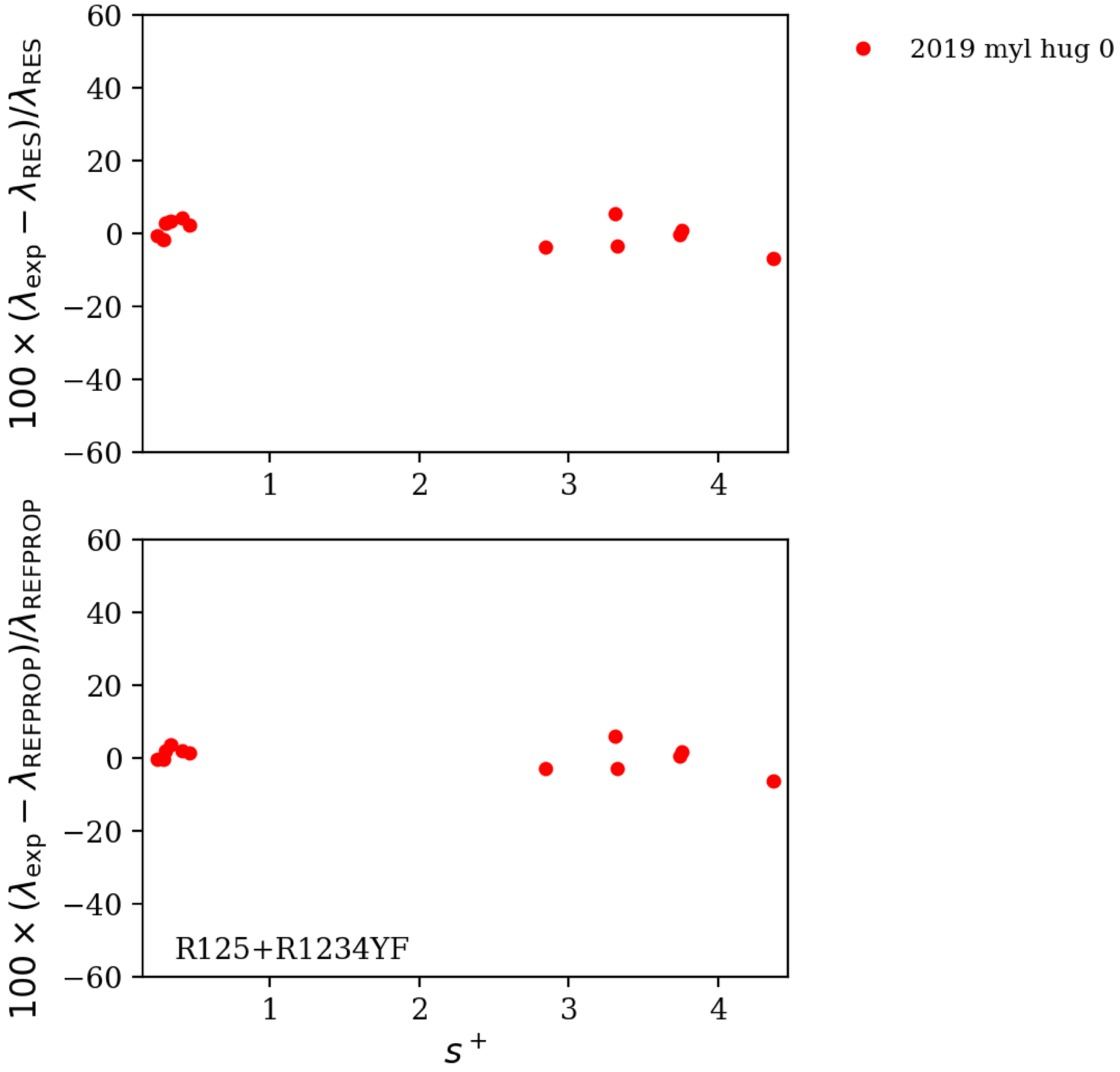

Figure DPR4. R125+R1234YF

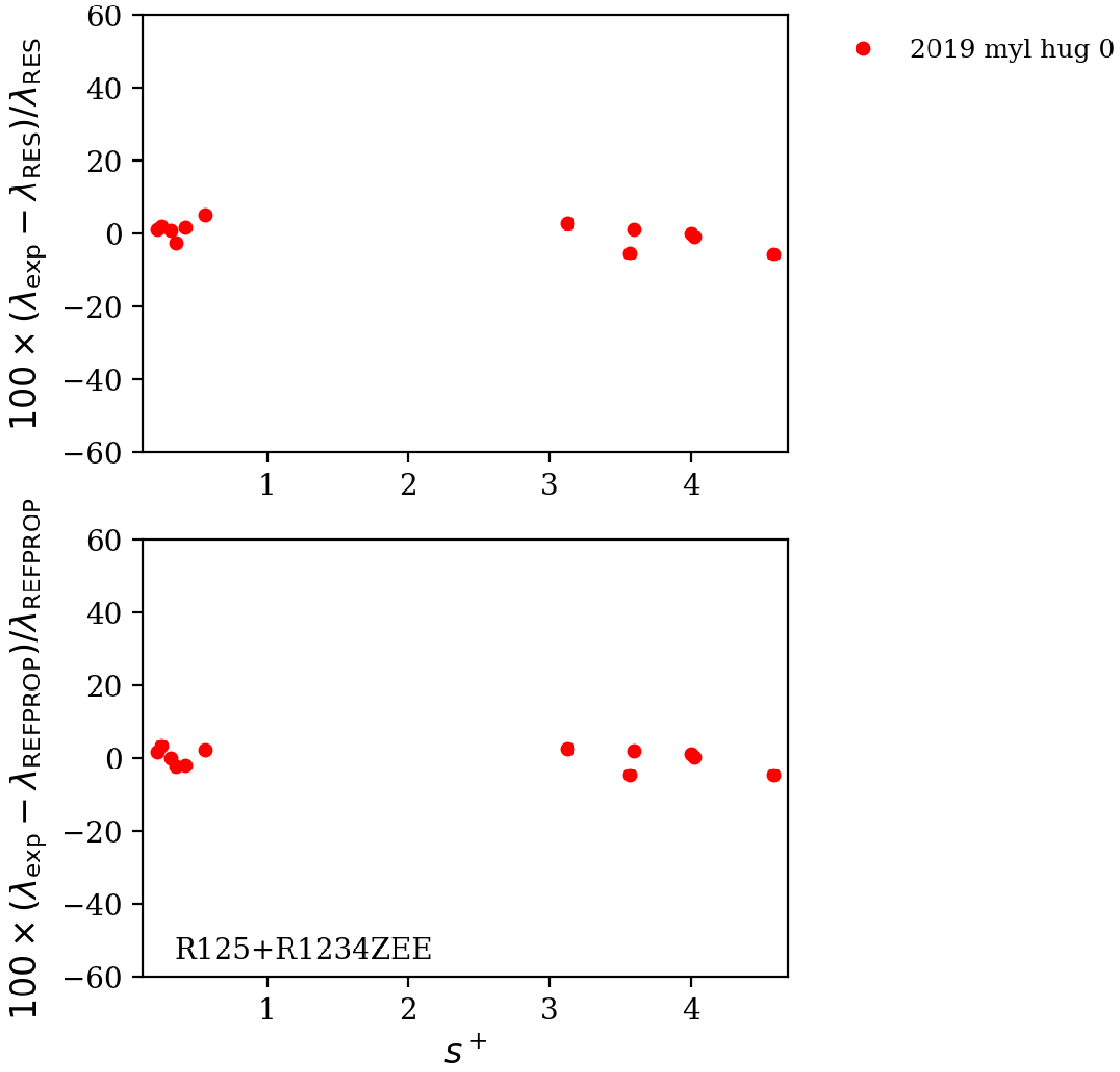

Figure DPR4. R125+R1234ZEE

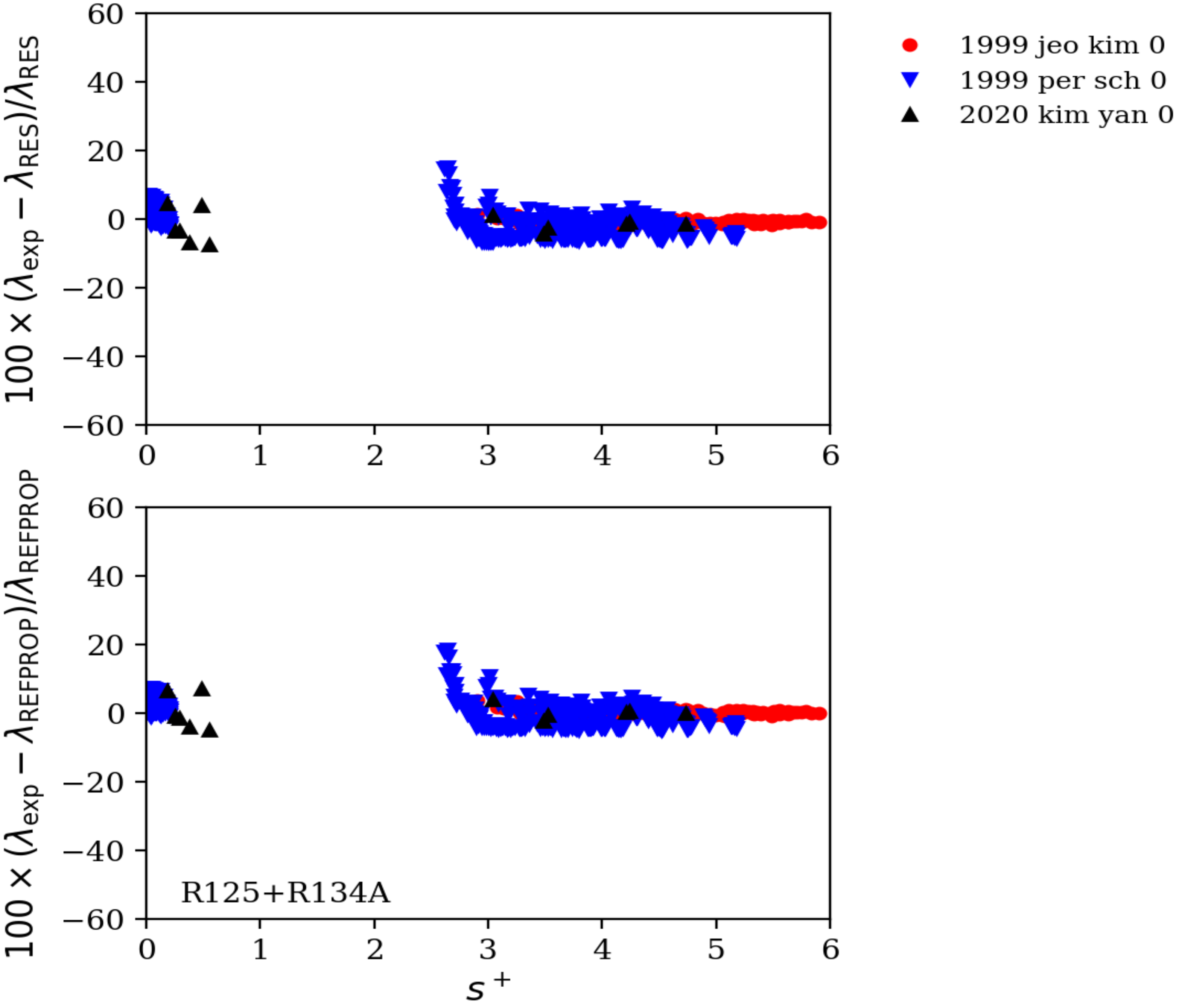

Figure DPR4. R125+R134A

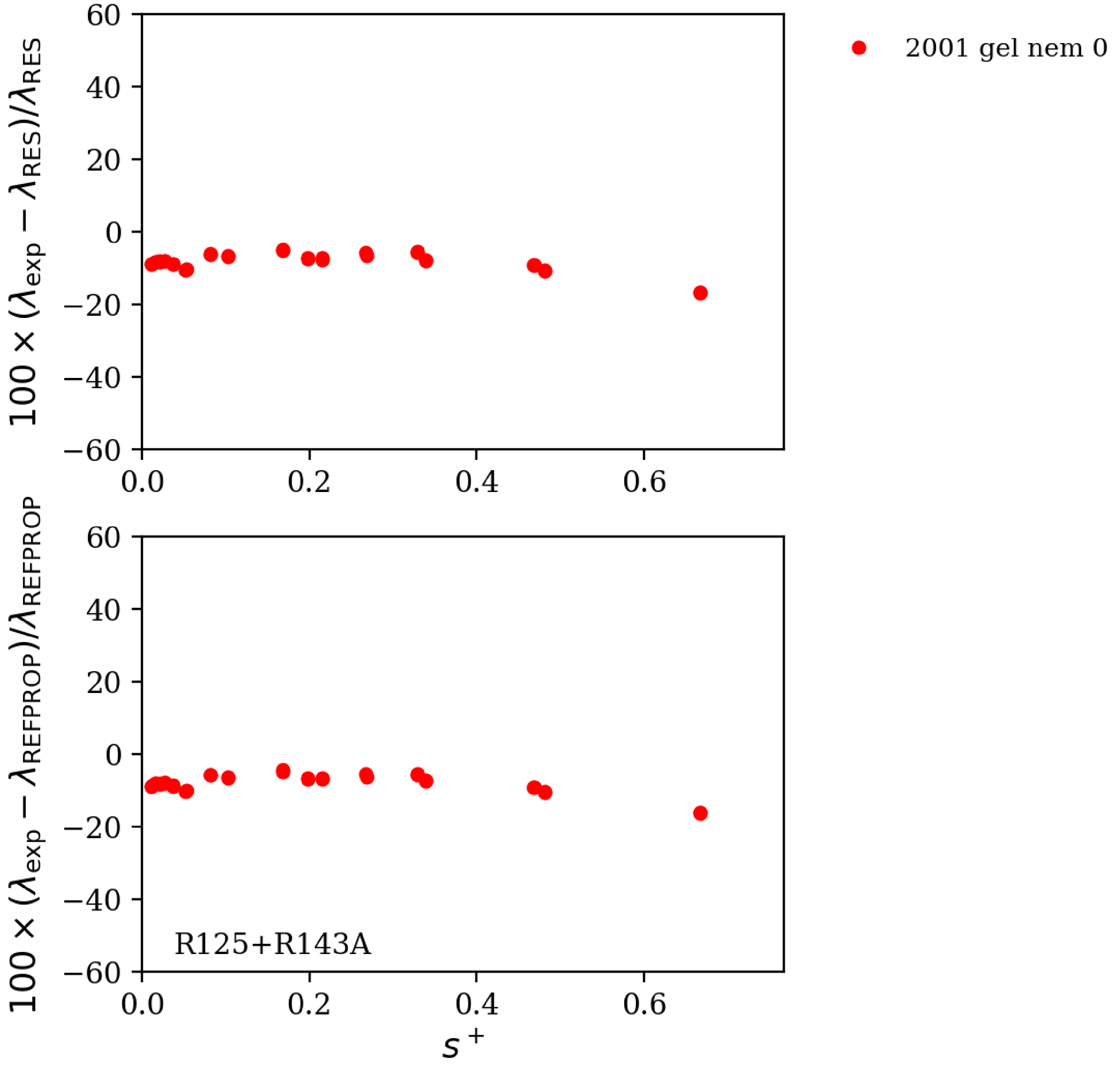

Figure DPR4. R125+R143A

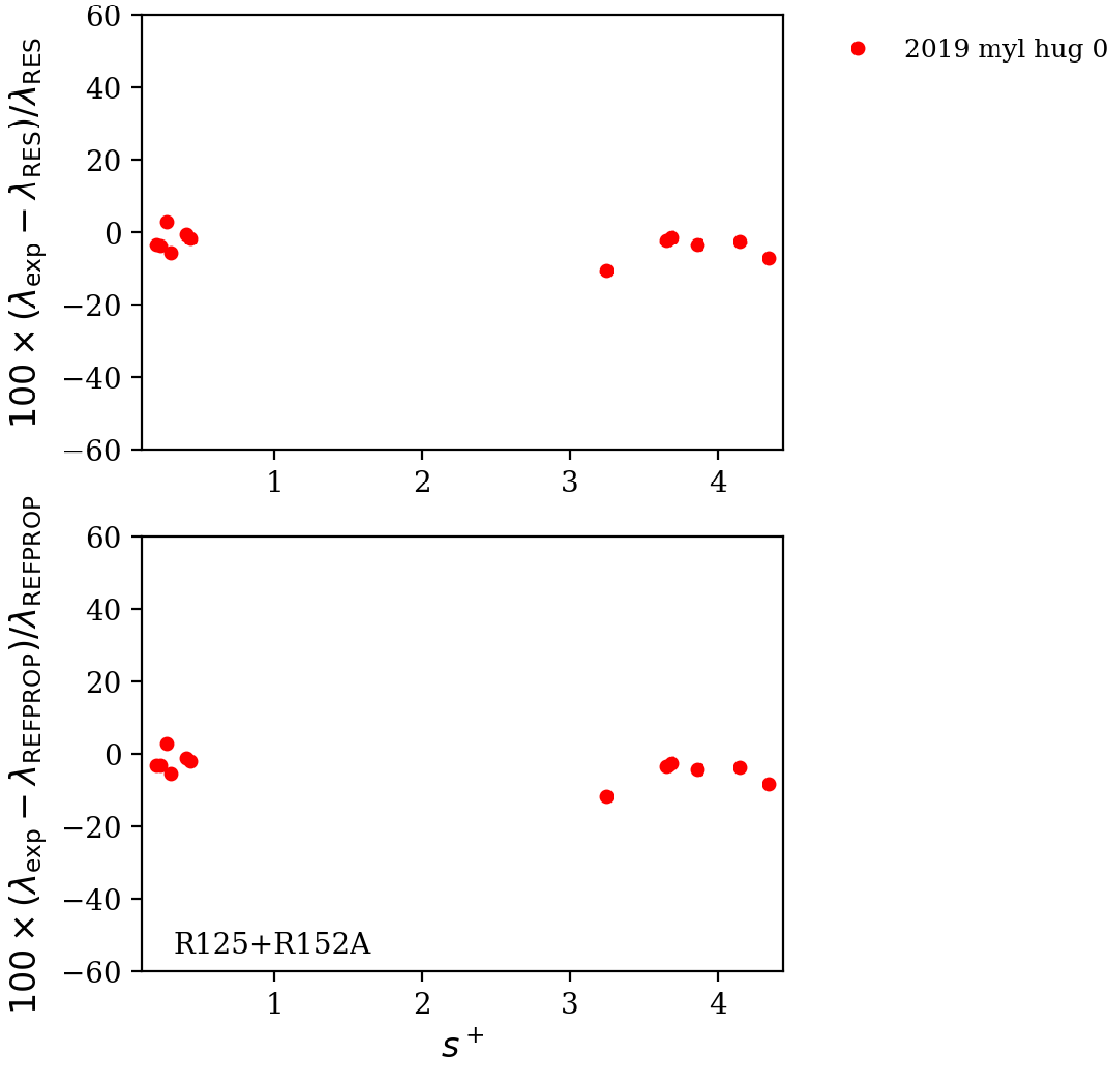

Figure DPR4. R125+R152A

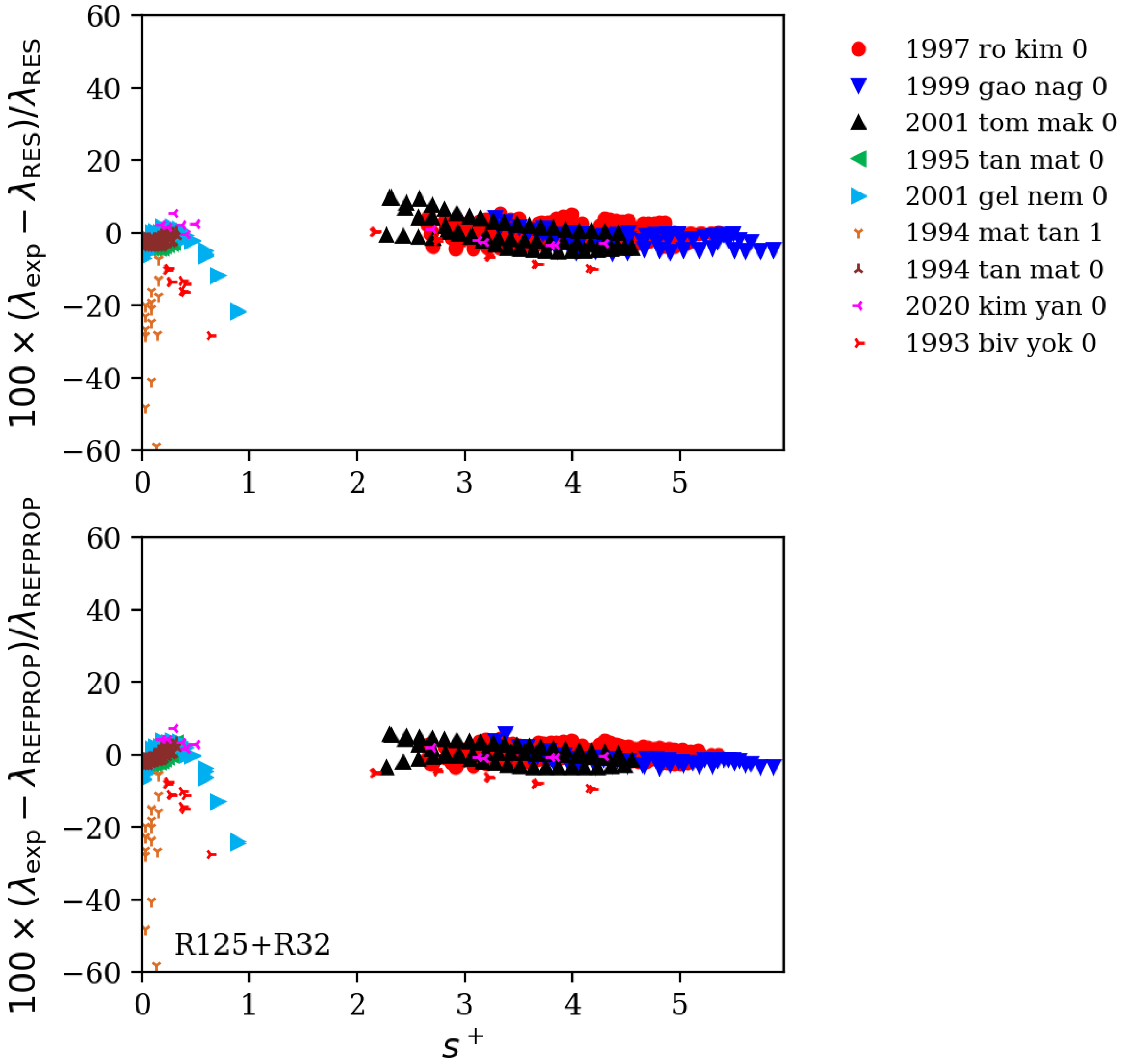

Figure DPR4. R125+R32

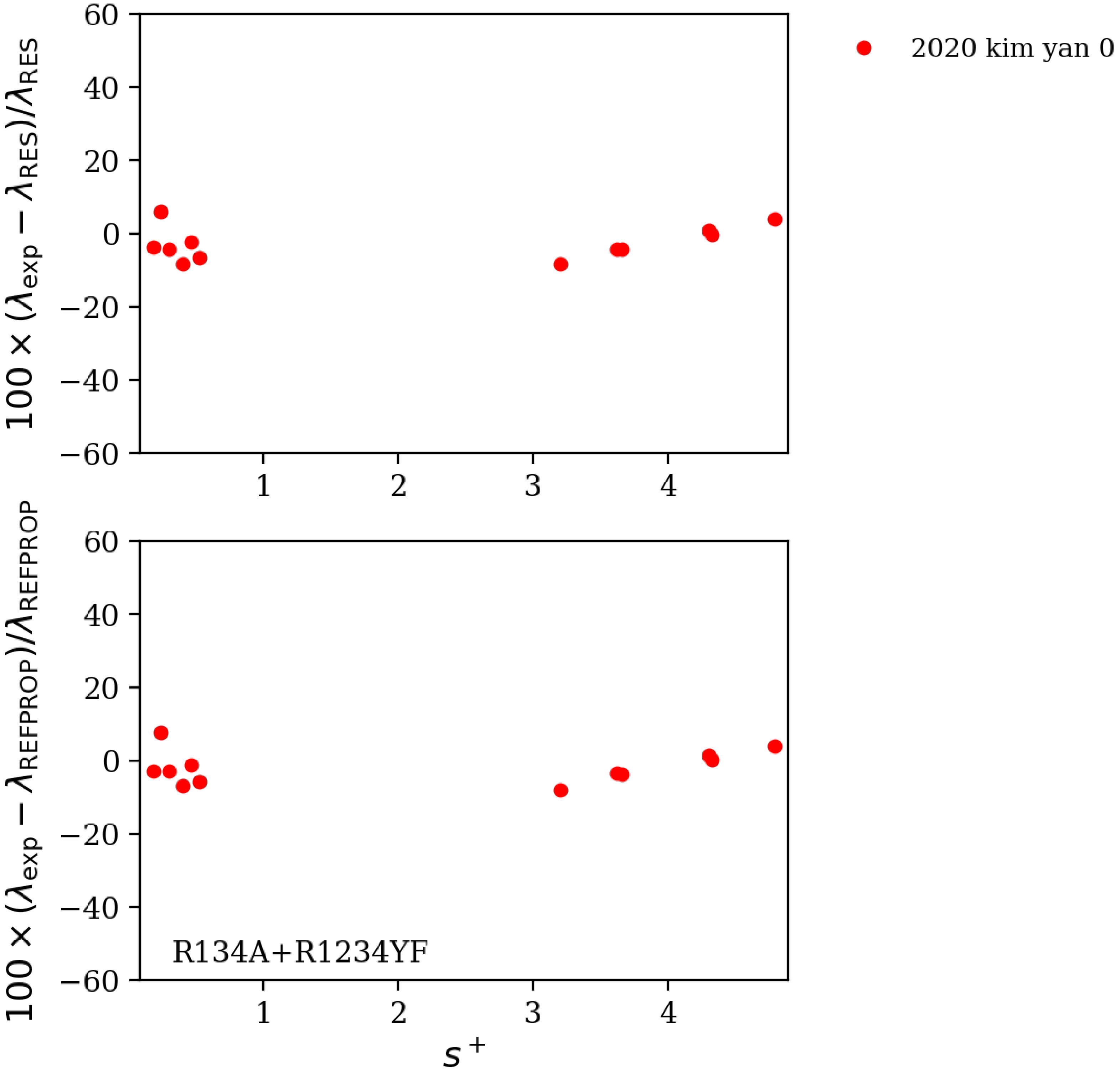

Figure DPR4. R134A+R1234YF

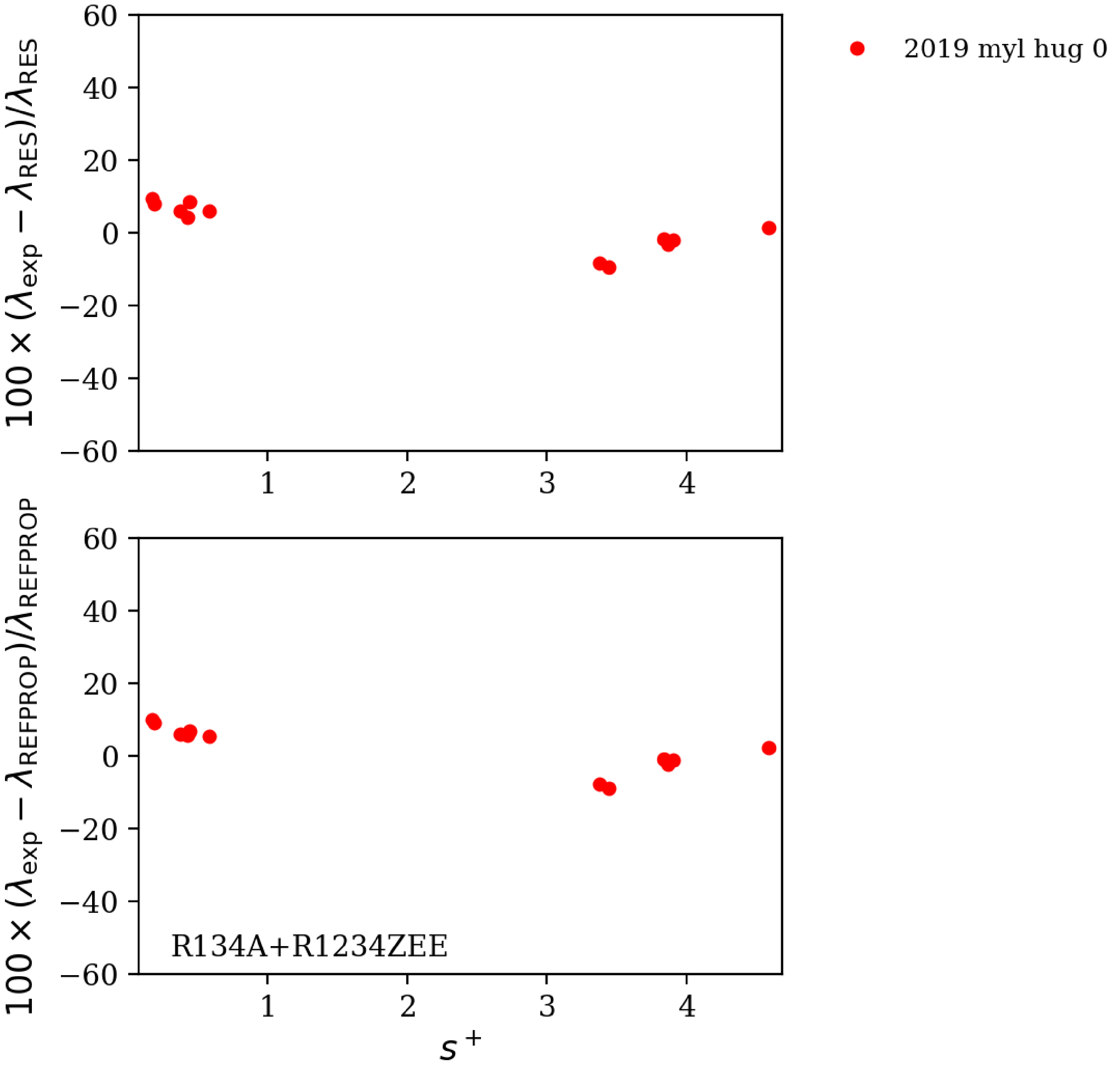

Figure DPR4. R134A+R1234ZEE

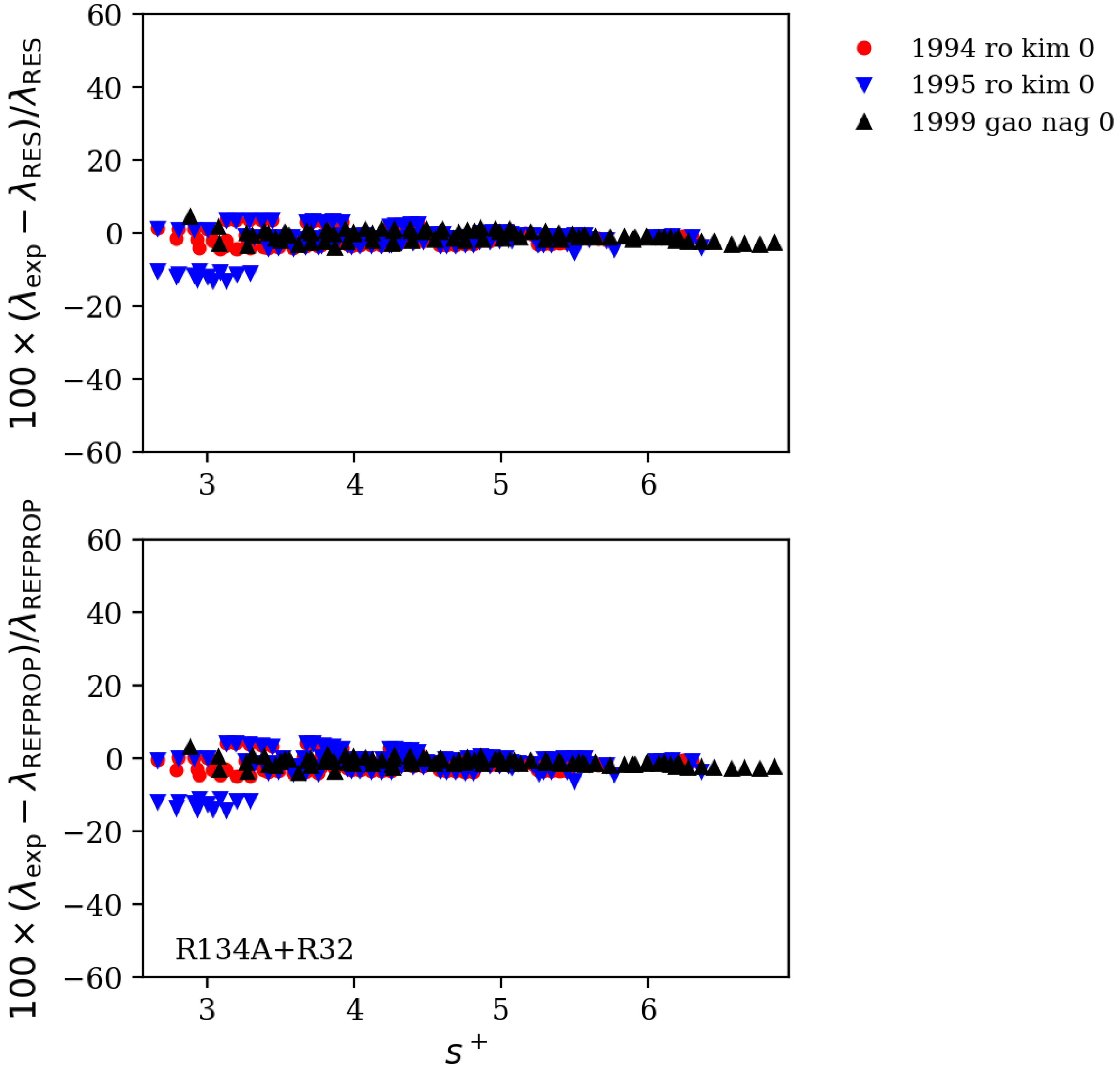

Figure DPR4. R134A+R32

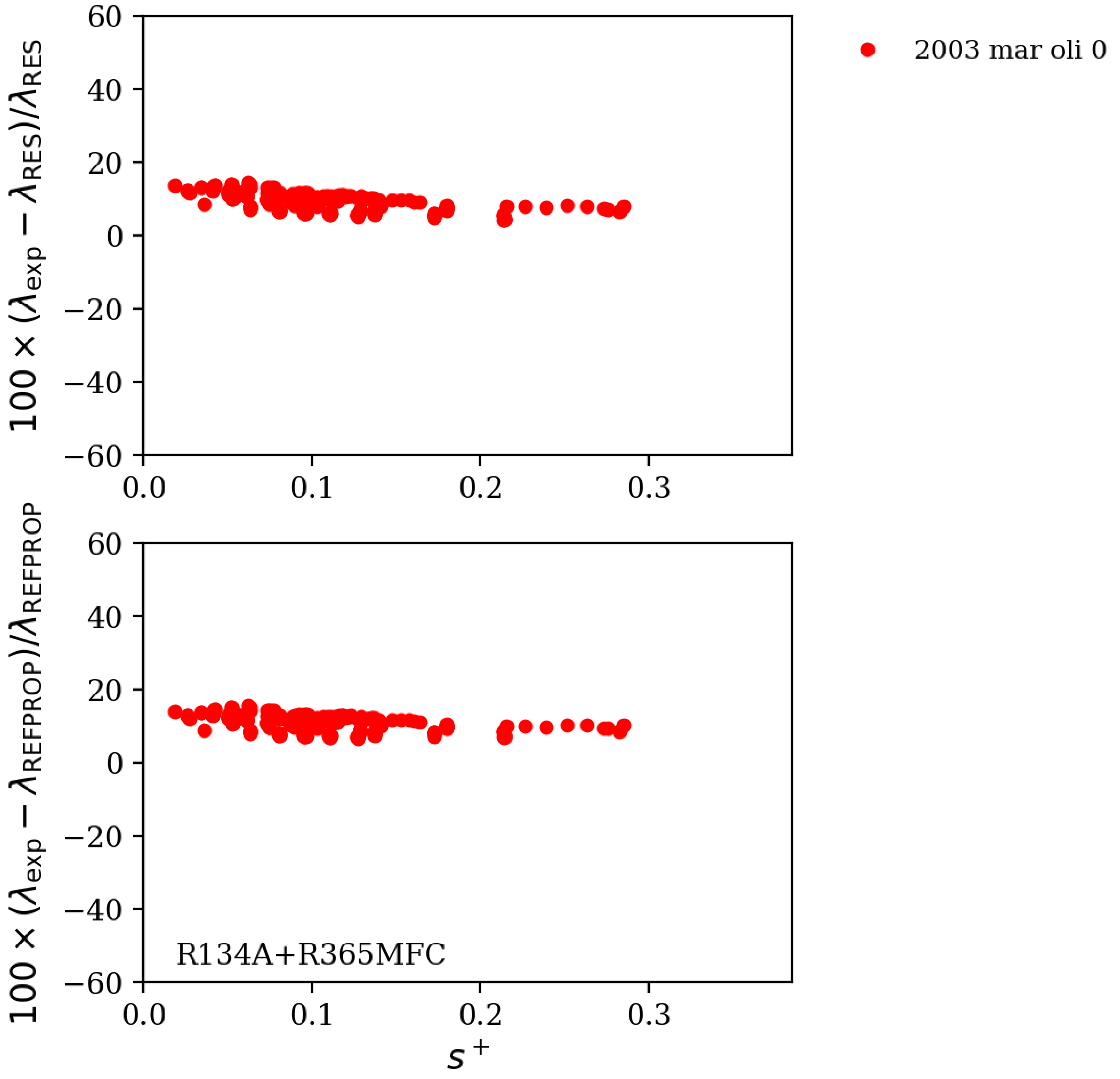

Figure DPR4. R134A+R365MFC

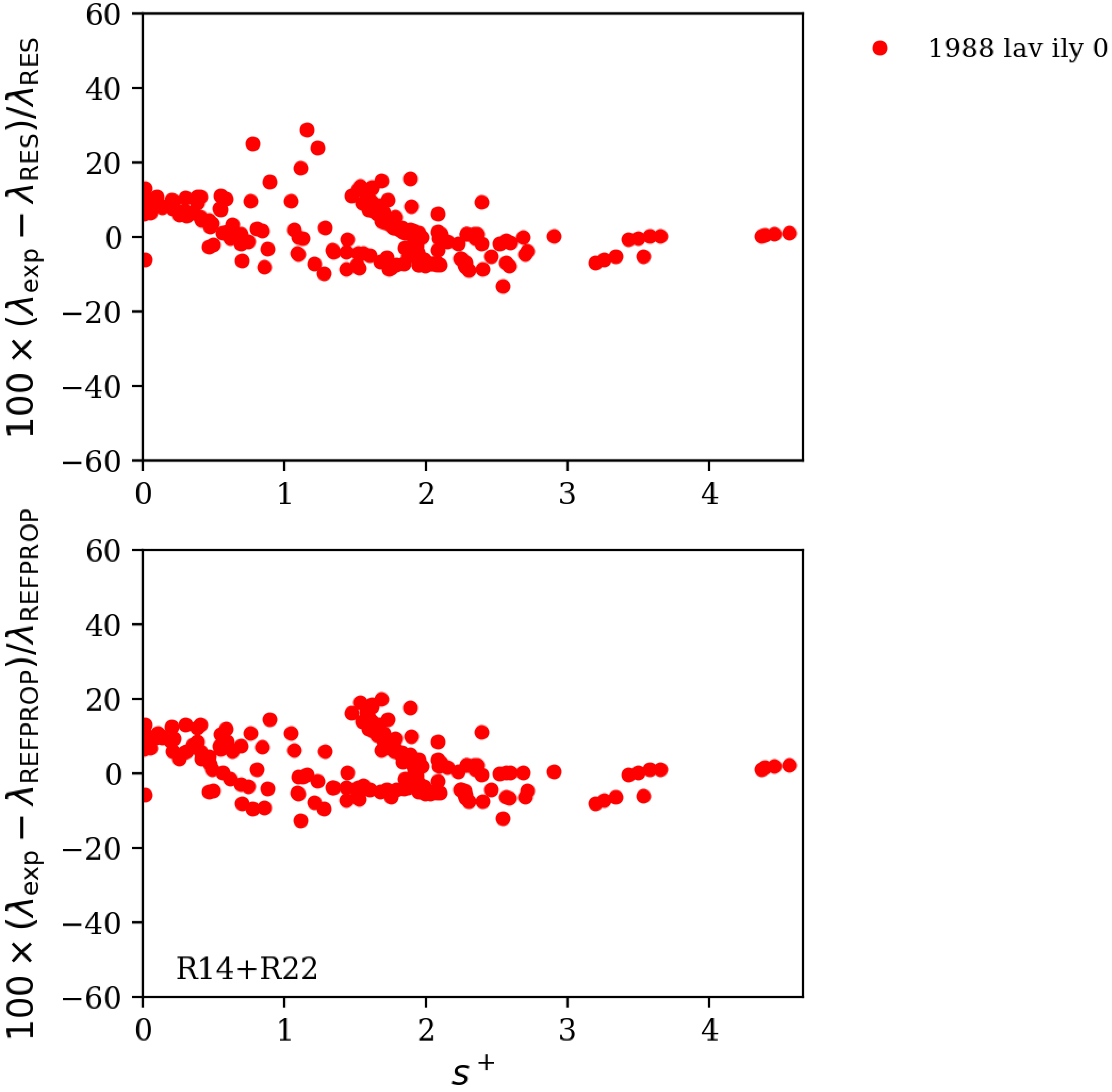

Figure DPR4. R14+R22

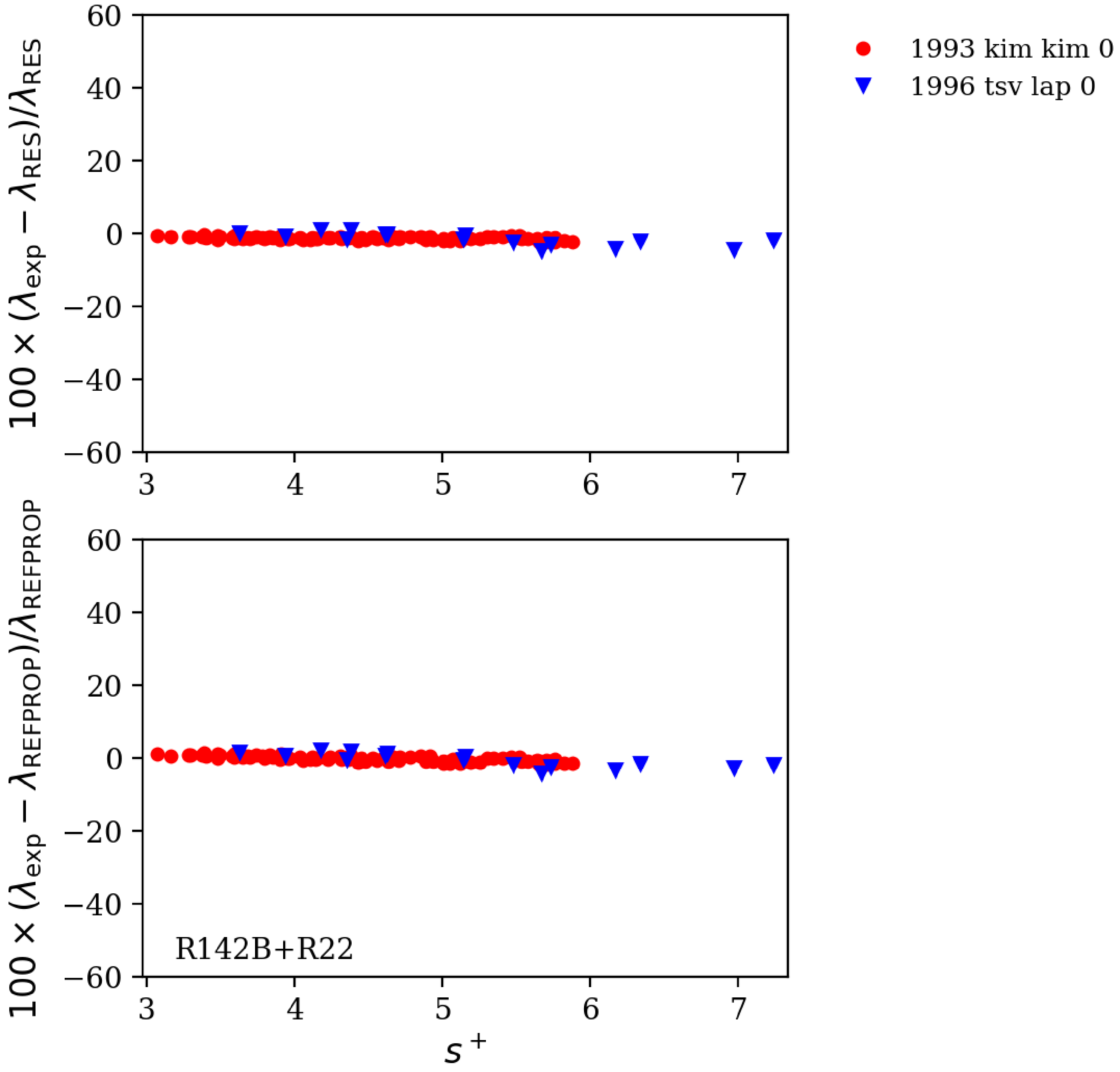

Figure DPR4. R142B+R22

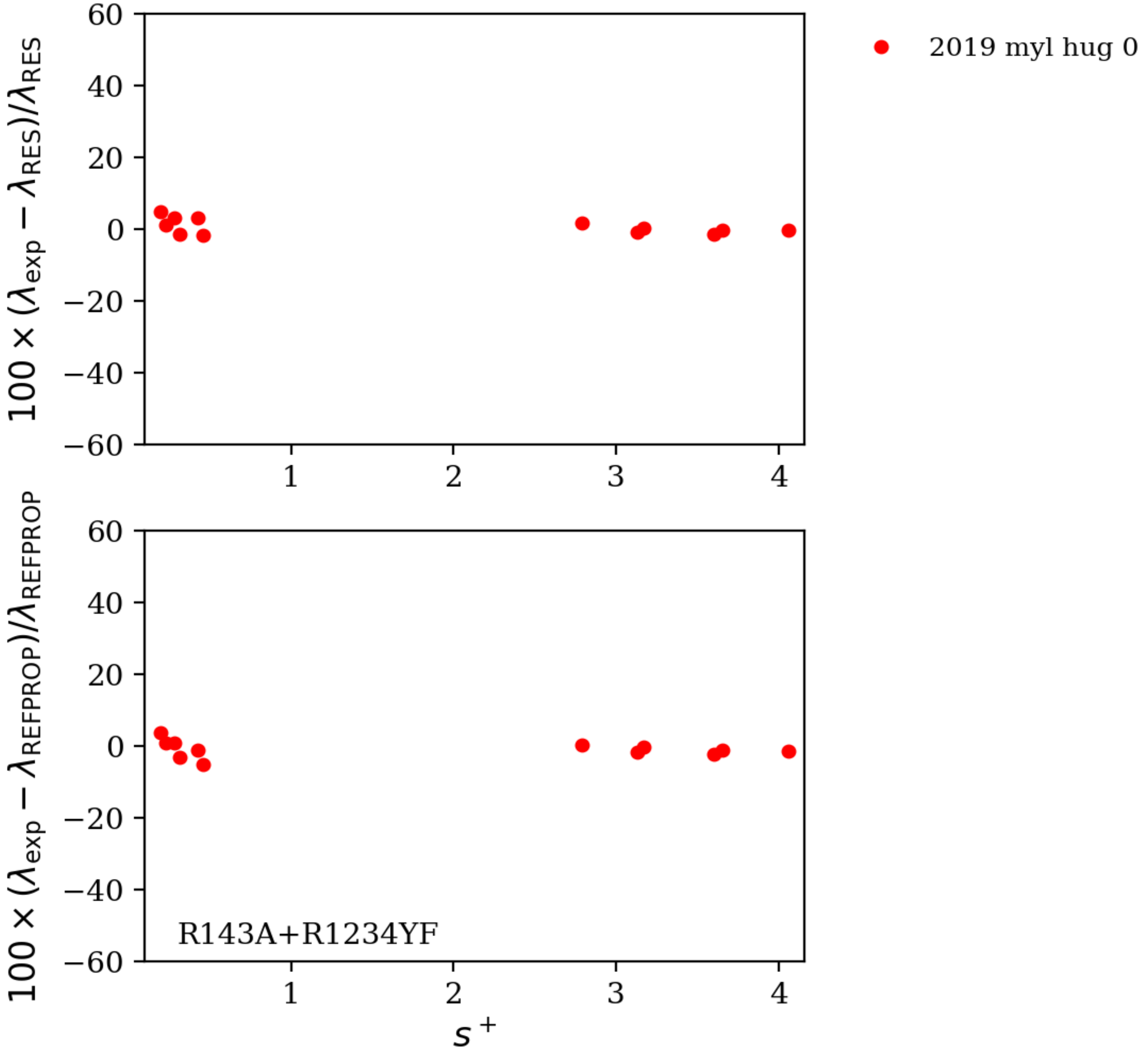

Figure DPR4. R143A+R1234YF

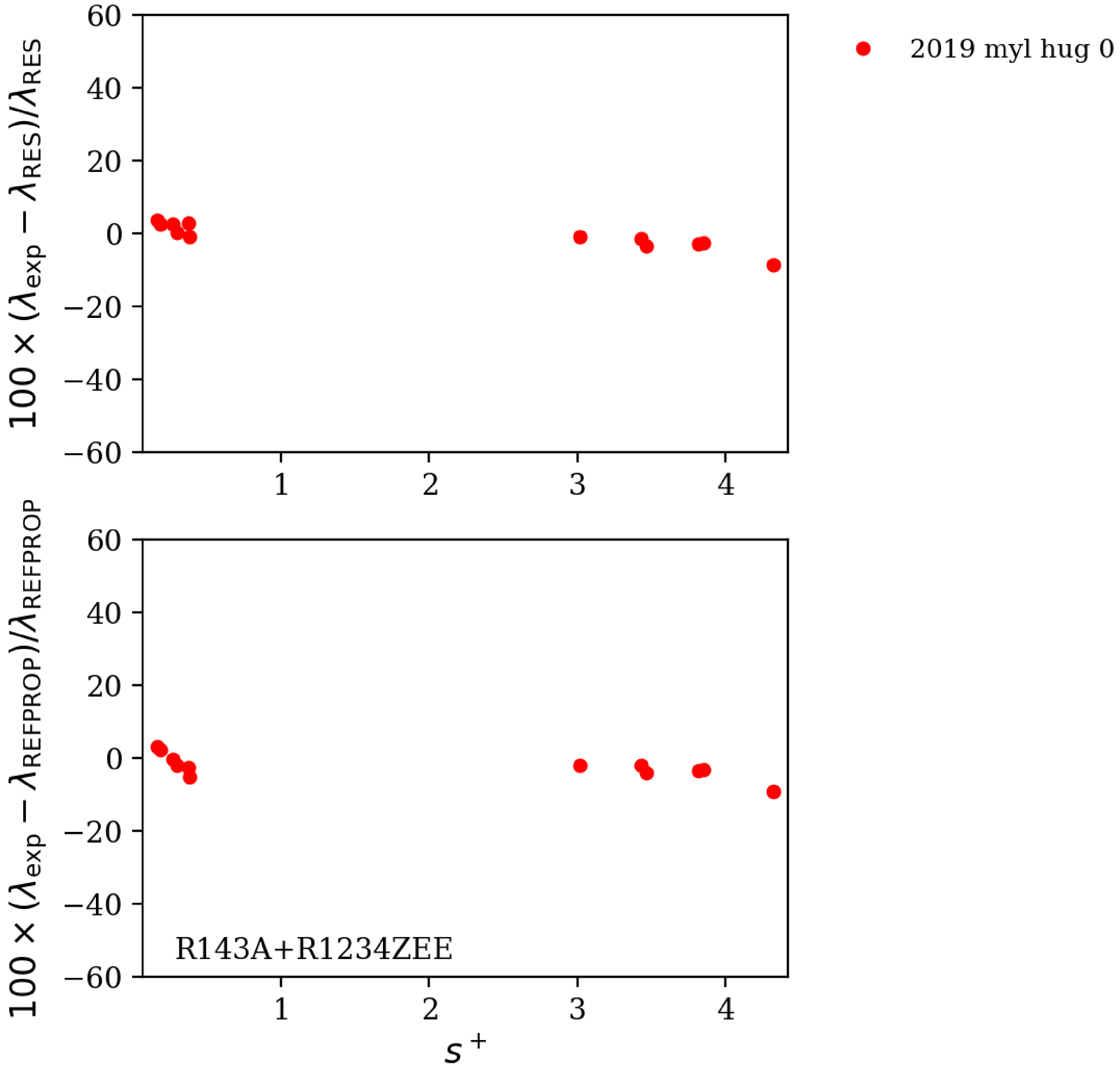

Figure DPR4. R143A+R1234ZEE

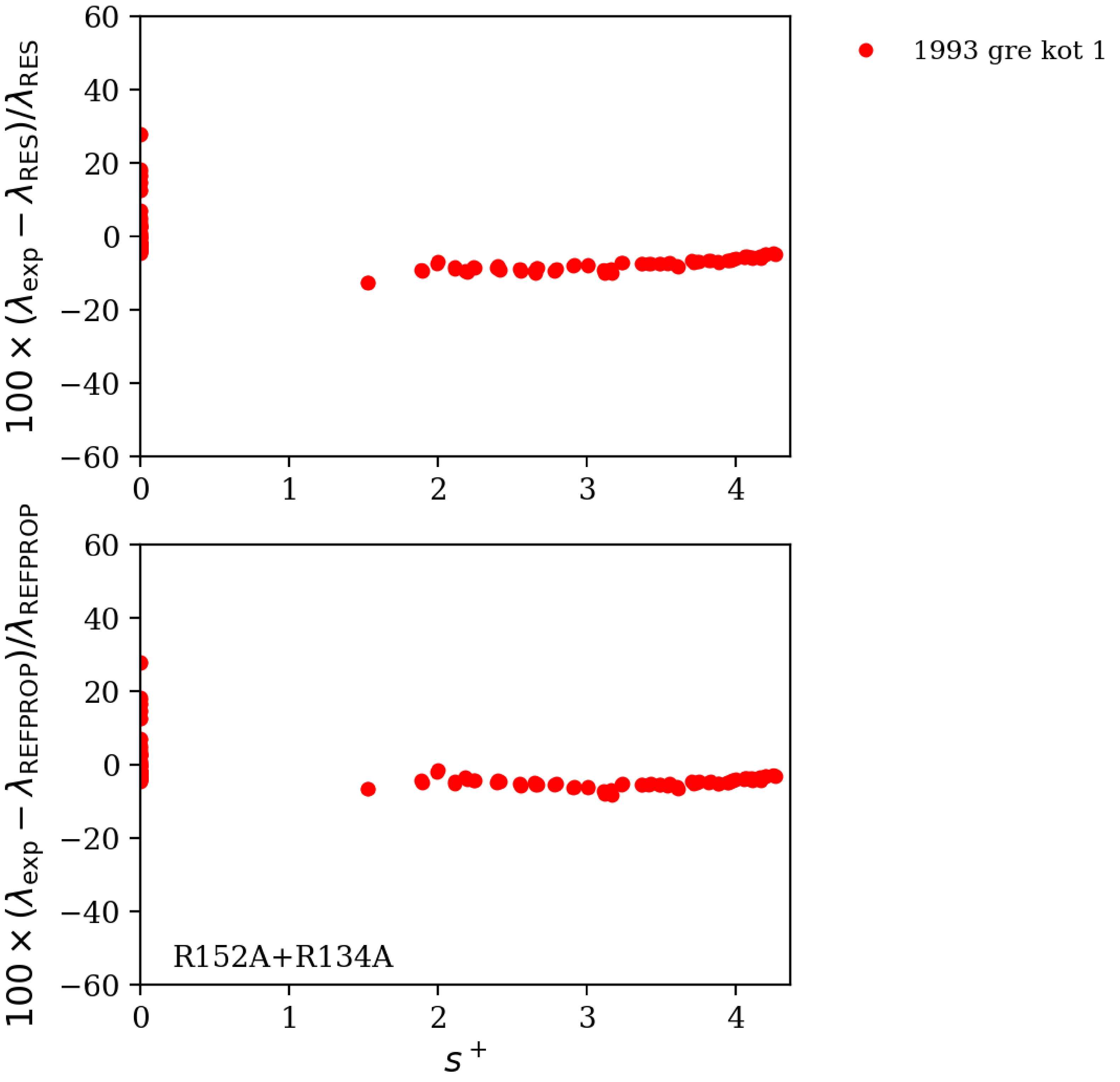

Figure DPR4. R152A+R134A

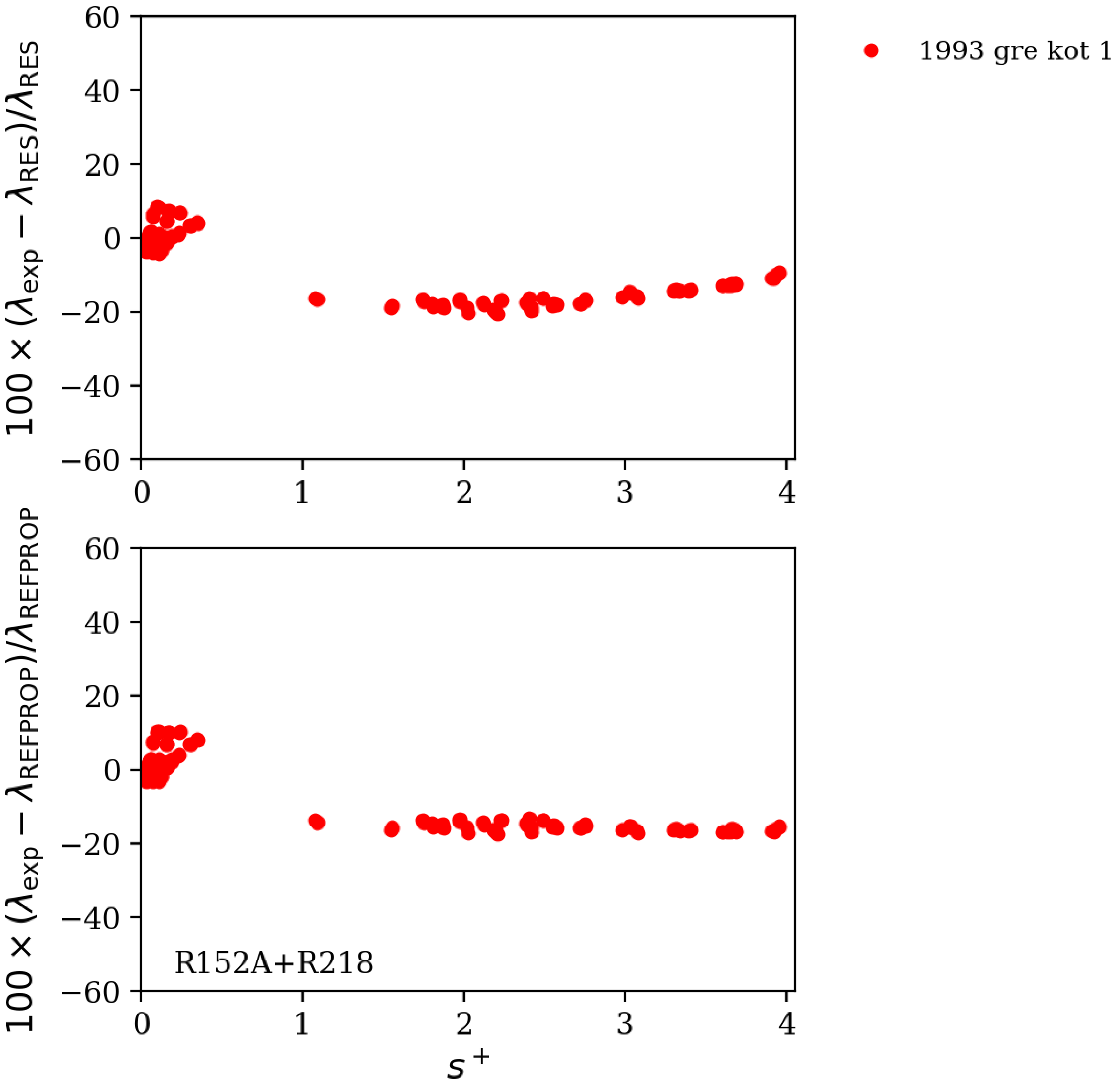

Figure DPR4. R152A+R218

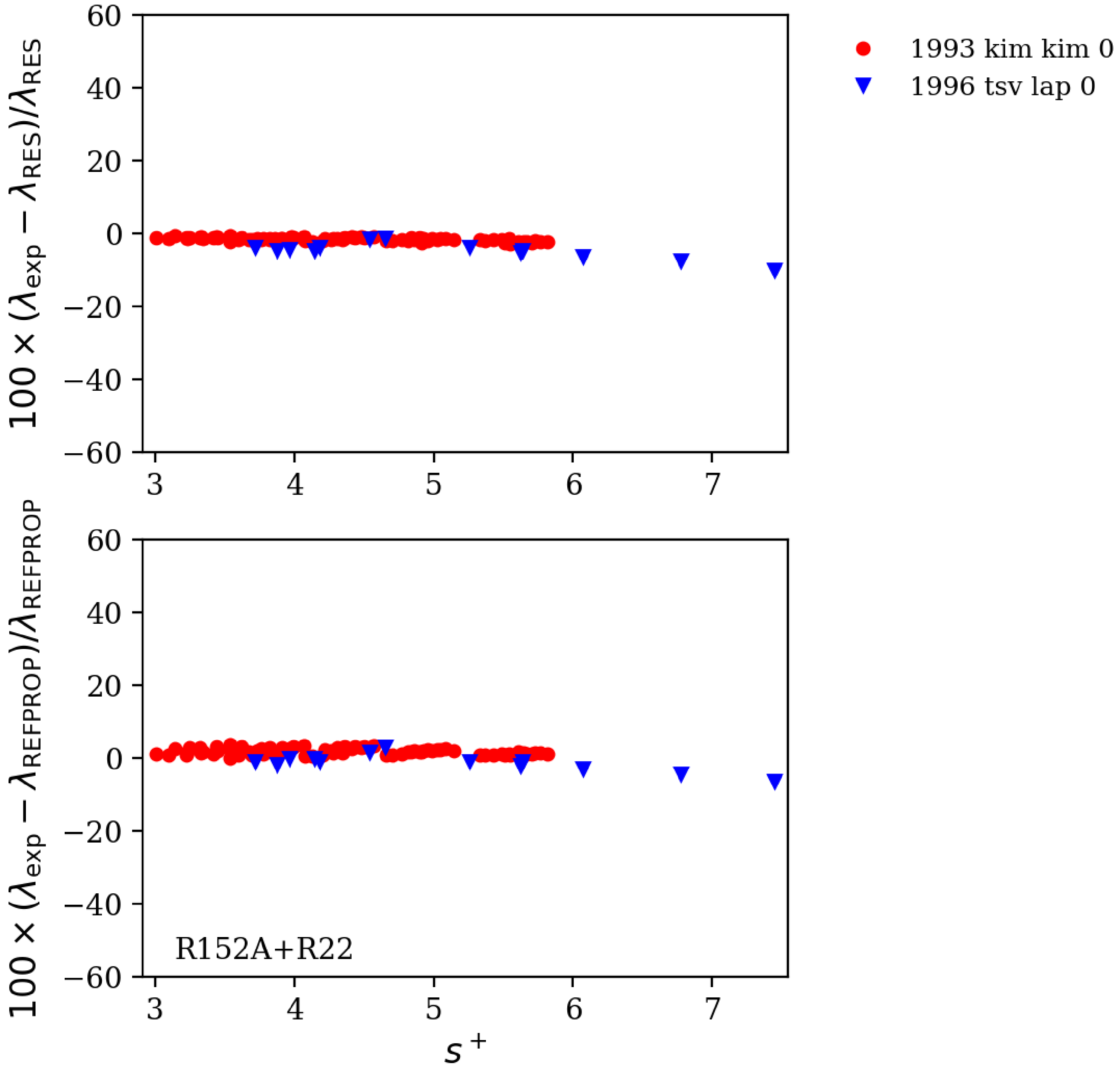

Figure DPR4. R152A+R22

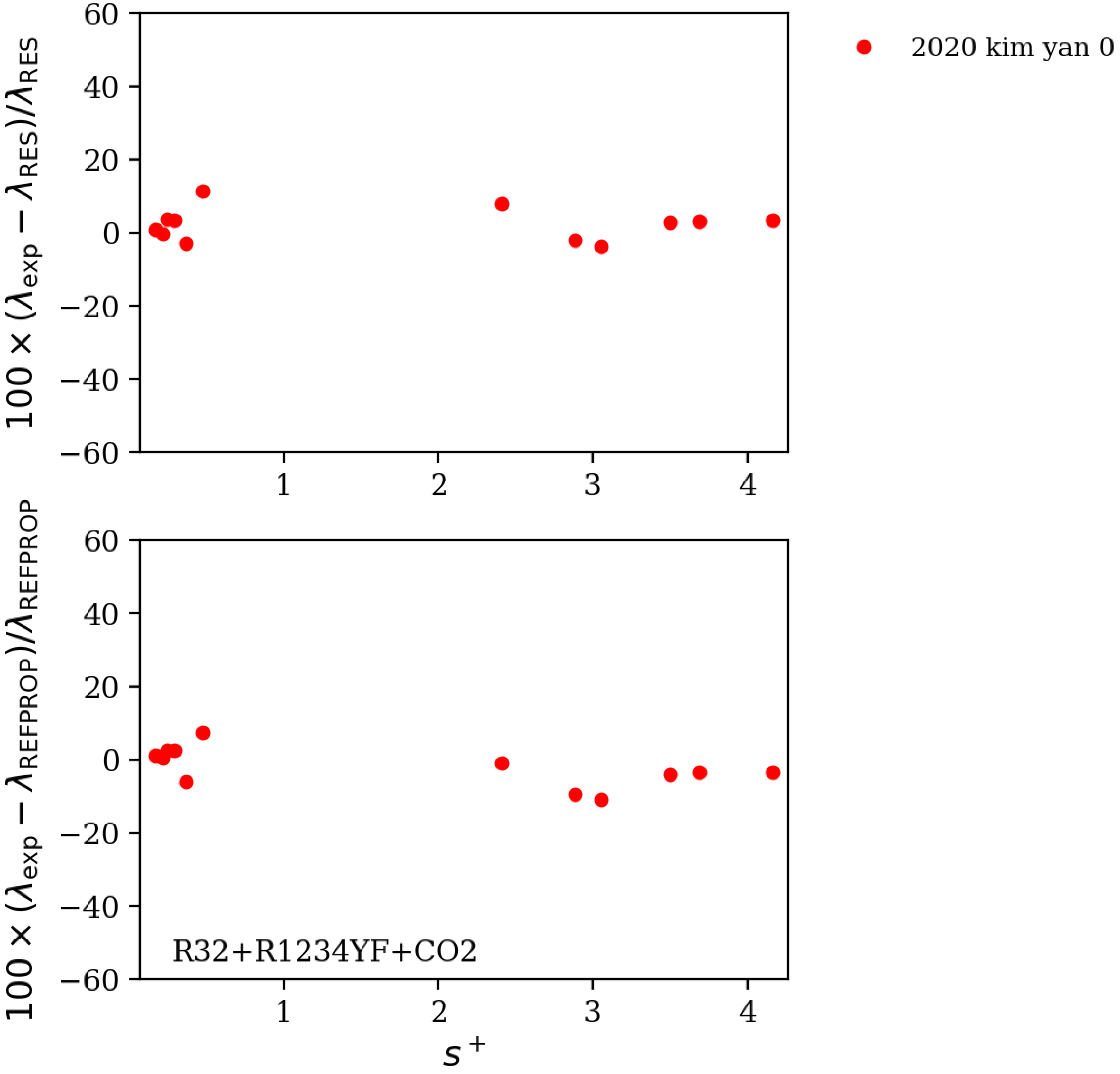

Figure DPR4. R32+R1234YF+CO2

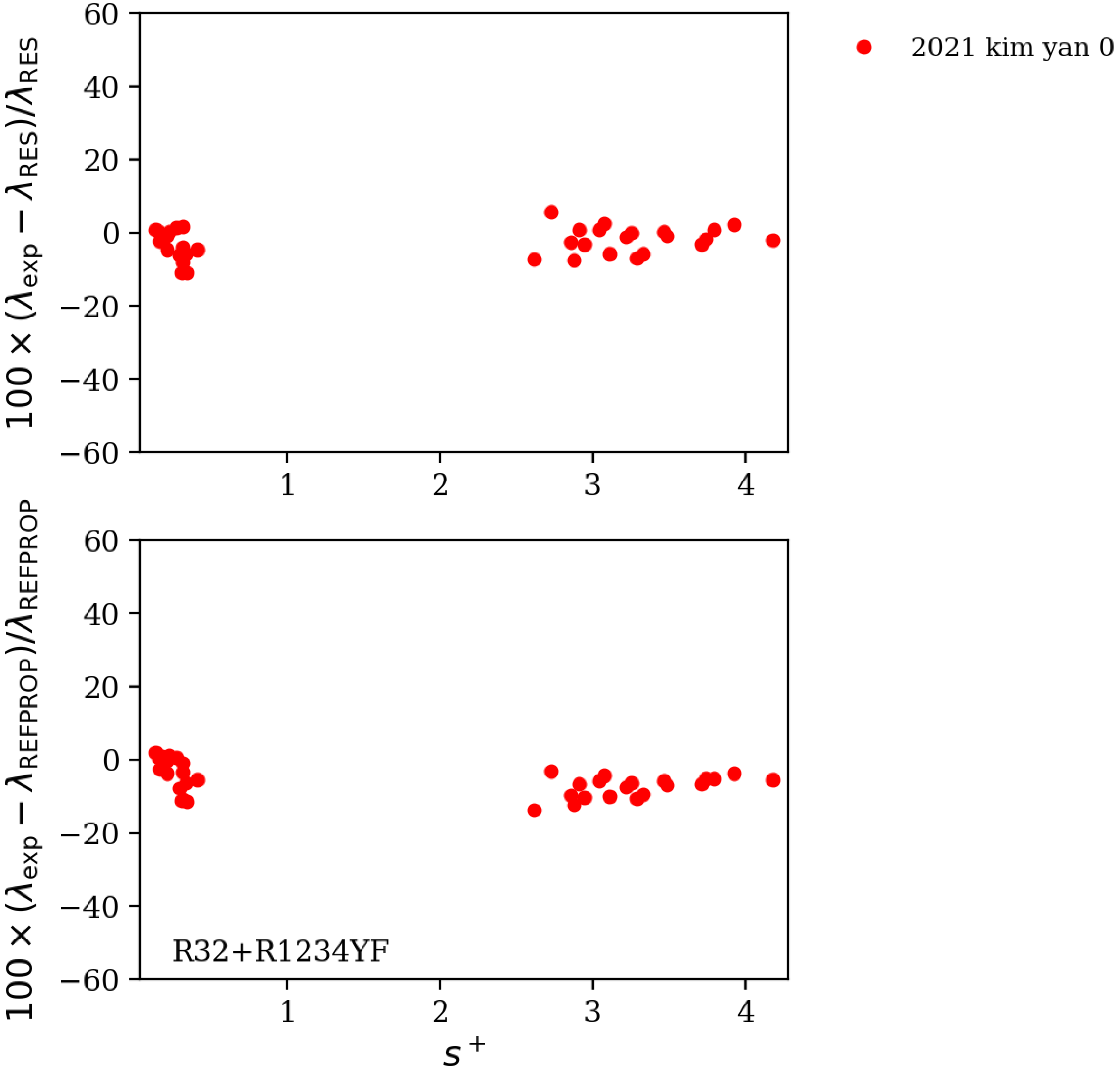

Figure DPR4. R32+R1234YF

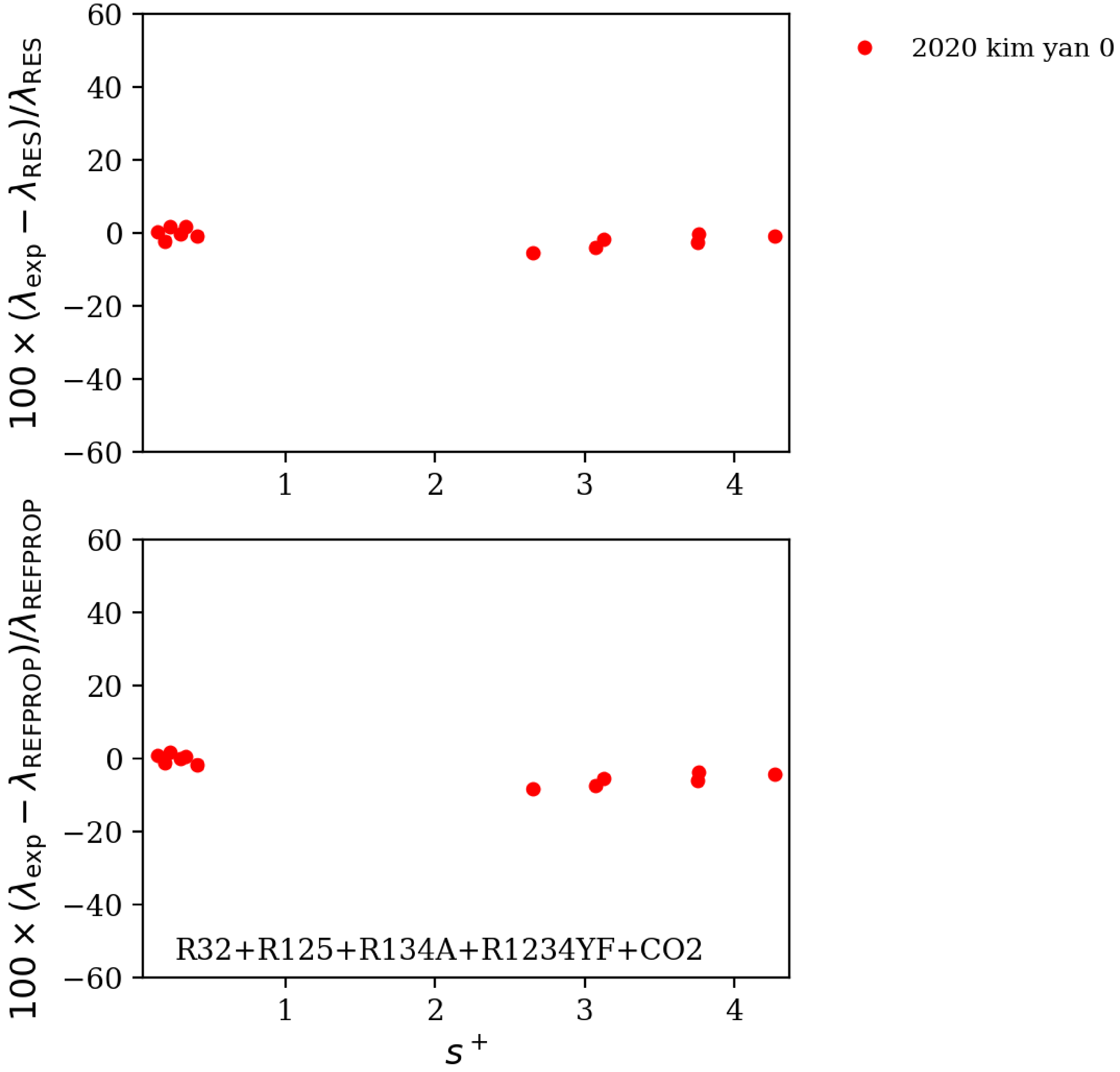

Figure DPR4. R32+R125+R134A+R1234YF+CO2

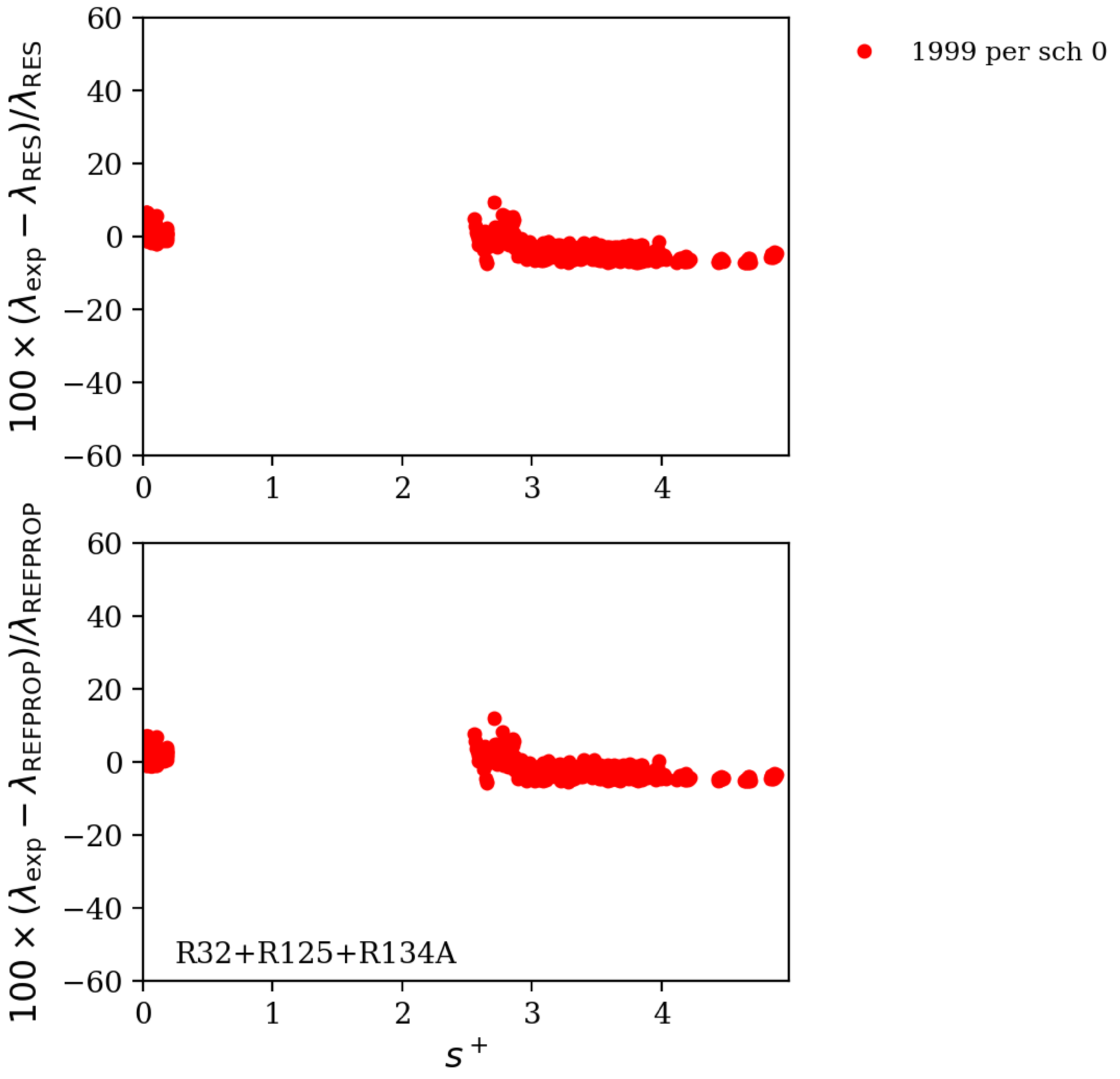

Figure DPR4. R32+R125+R134A

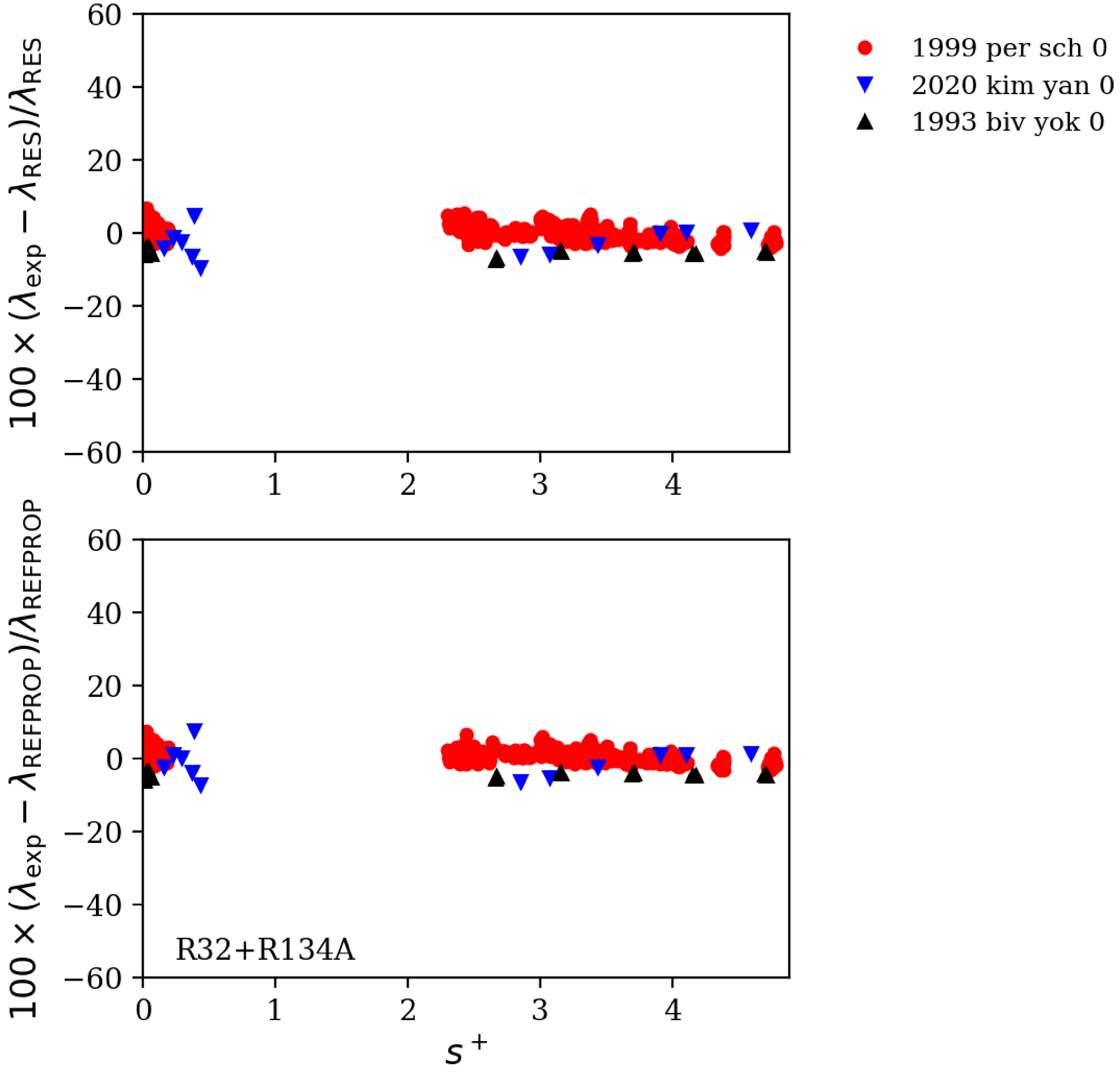

Figure DPR4. R32+R134A

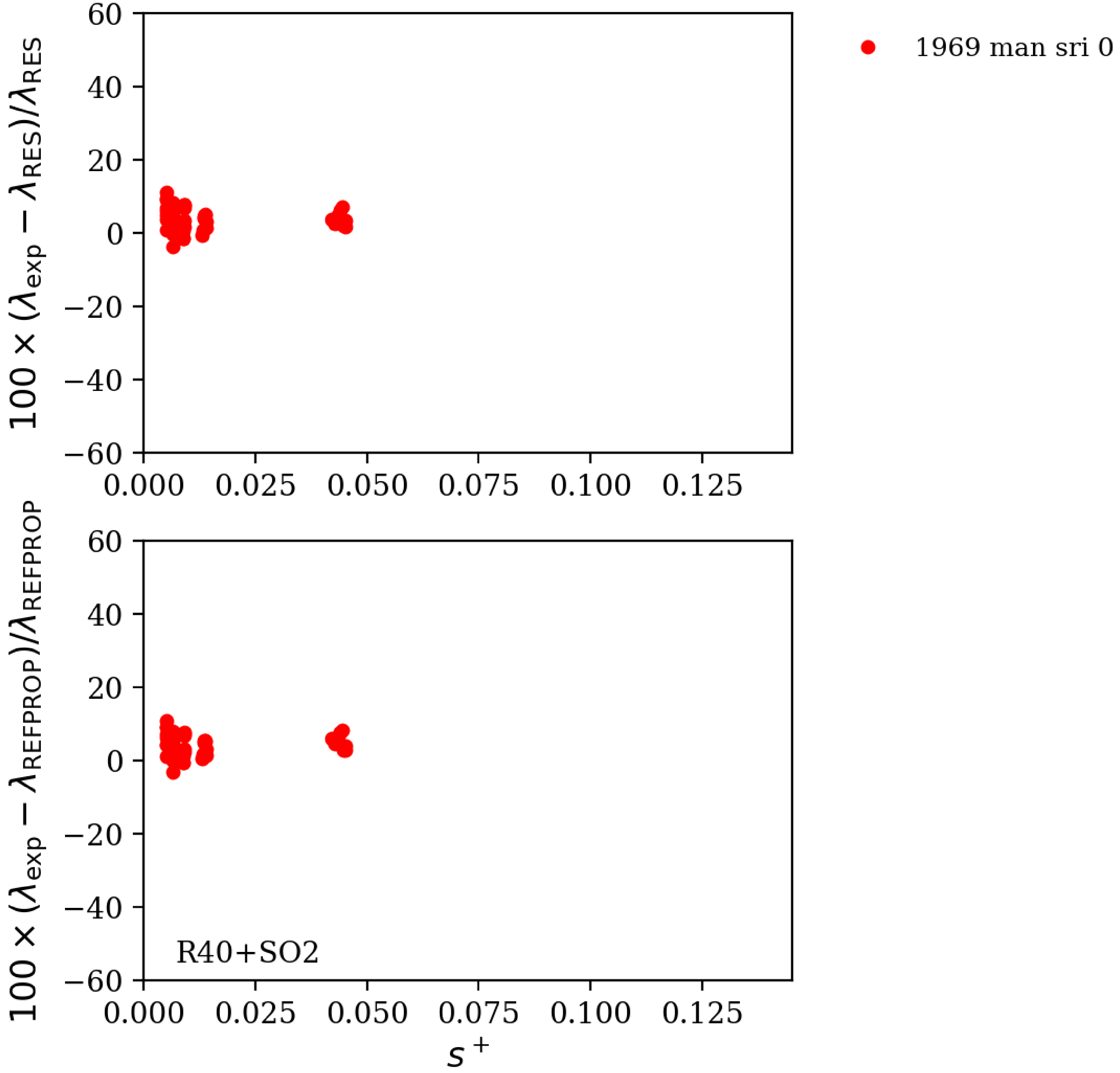

Figure DPR4. R40+SO2

REFREFCE

Note:It is hard to find out all source literature of the experimental data, therefore, some are not list here.

(1) Wilson, L. C.; Wilding, W. V.; Wilson, G. M.; Rowley, R. L.; Felix, V. M.; Chisolm-Carter, T. Thermophysical Properties of HFC-125. *Fluid Phase Equilib.* **1992**, *80*, 167–177. https://doi.org/10.1016/0378-3812(92)87065-u.

(2) Watanabe, H. Accurate Measurement of the Thermal Conductivity and Thermal Diffusivity of Toluene and N-Heptane. *Int. J. Thermophys.* **1997**, *18*, 313–325. https://doi.org/10.1007/bf02575163.

(3) Vanicheva, N. A.; Zaitseva, L. S.; Yakush, L. V. Experimental Determination of the Thermal Conductivity of Oxygen in the Gas Phase (300-1000 K). *Inzh.-Fiz. Zh.* **1985**, *49*, 94.

(4) Thomas, L. B.; Golike, R. C. A Comparative Study of Accommodation Coefficients by the Temperature Jump and Low-Pressure Methods and Thermal Conductivities of He, Ne, and CO2. *J. Chem. Phys.* **1954**, *22*, 300.

(5) Tarzimanov, A. A.; Zainullin, M. M. Experimental Study of the Thermal Conductivity of Heavy Water Vapor at Temperatures 230-550 C and at Pressures up to 800 Bar. *Teploenergetika* **1974**, 61–66.

(6) Tarzimanov, A. A.; Gabitov, F. R. Study of the Thermophysical Properties of Liquids in Impuls Heating Flux Method. *Teplofiz. Vys. Temp.* **2004**, *42*, 236–242.

(7) Takada, N.; Matsuo, S.; Tanaka, Y.; Sekiya, A. Gaseous Thermal Conductivities of New Hydrofluoroethers (HFEs). *J. Fluorine Chem.* **1998**, *91*, 81–85. https://doi.org/10.1016/s0022-1139(98)00202-4.

(8) Stoliarov, E. A.; Ipat'ev, V. V.; Theodorovich, V. P. Transport Fenomena in Compressed Gases. I. Determination of Heat Conductivity Coefficients of Compressed Gases (H2, N2, Air, CH4 and CO2). *Zh. Fiz. Khim.* **1950**, *24*, 166–176.

(9) Spirin, G. G. Study of the Molecular Thermal Conductivity of Organic Liquids. *Inzh.-Fiz. Zh.* **1980**, *38*, 656–661.

(10) Slusarev, V. V.; Kessel'man, P. M. Study of the Thermal Conductivity of Toluene and Some Diflouroethanes at High Pressures. *Izv. Vyssh. Uchebn. Zaved., Neft Gaz* **1976**, *19*, 109.

(11) Senftleben, H.; Gladisch. A Method of Determining the Specific Heats, the Viscosities and the Thermal Conductivities of Gases (in German). *Z. Phys.* **1949**, *125*, 653.

(12) Sekiya, A.; Misaki, S. The Potential of Hydrofluoroethers to Replace CFCs, HCFCs, and PFCs. *J. Fluorine Chem.* **2000**, *101*, 215–221.

(13) Sakiadis, B. C. C.; Coates, J. Studies of Thermal Conductivty of Liquids, Pt. I. *AIChE J.* **1955**, *1*, 275–288.

(14) Rothman, A. J.; Bromley, L. A. High Temperature Thermal Conductivity of Gases. Measurements on Nitrogen, Carbon Dioxide, Argon, and Nitrogen-Carbon Dioxide Mixtures at Temperatures up to 775 Degrees C. *Ind. Eng. Chem.* **1955**, *47*, 899.

(15) Mukhamedzyanov, G. K.; Usmanov, A. G. Thermal Conductivity of Higher Saturated Hydrocarbons. *Izv. Vyssh. Uchebn. Zaved., Neft Gaz* **1967**, *10*, 76–80.

(16) Marsh, K. N.; Perkins, R.; Ramires, M. L. V. Measurement and Correlation of the Thermal Conductivity of Propane from 86 K to 600 K at Pressures to 70 MPa. *J. Chem. Eng. Data* **2002**, *47*, 932–940. https://doi.org/10.1021/je010001m.

(17) LeNeindre, B.; Garrabos, Y. Measurements of the Thermal Conductivity of HFC-134a in the Temperature Range from 300 to 530 K and at Pressures up to 50 MPa. *Int. J. Thermophys.* **1999**, *20*, 1379–1401. https://doi.org/10.1023

/a:1021480803361.
(18) Jamieson, D. L.; Cartwright, G. Thermal Conductivity of Associated Fluids. *J. Chem. Eng. Data* **1980**, *25*, 199–201. https://doi.org/10.1021/je60086a026.
(19) Guseinov, K. D.; Mirzoev, B. M. Experimental Study of the Thermal Conductivity of Gaseous Benzene Homologous. *Izv. Vyssh. Uchebn. Zaved., Neft Gaz* **1975**, *18*, 28.
(20) Golubev, I. F. Thermal Conductivity of .Epsylon.-Caprolactam and Its Aqueous Solutions. *Trudy GIAP* **1959**, 30–37.
(21) Gambhir, R. S.; Gandhi, J. M.; Saxena, S. C. Thermal Conductivity of Rare Gases, Deuterium and Air. *Indian J. Pure Appl. Phys.* **1967**, *5*, 457.
(22) El-Sharkawy, A. A.; Kenawy, M. A.; Dakroury, A. Z. Thermal Properties of Some Aliphatic and Aromatic Compounds in the Temperature Range of 20-100 Deg. C. *High Temp. - High Pressures* **1983**, *15*, 391.
(23) DiGuilio, R.; Teja, A. S. Thermal Conductivity of Poly(Ethylene Glycoles) and Their Binary Mixtures. *J. Chem. Eng. Data* **1990**, *35*, 117–121. https://doi.org/10.1021/je00060a005.
(24) Chen, W.; Qiu, L.; Liang, S.; Zheng, X.; Tang, D. Measurement of Thermal Conductivities of [Mmim]DMP/CH3OH and [Mmim]DMP/H2O by Freestanding Sensor-Based 3w Technique. *Thermochim. Acta* **2013**, *560*, 1–6. https://doi.org/10.1016/j.tca.2013.02.020.
(25) Carmichael, L. T.; Berry, V. M.; Sage, B. H. Thermal Conductivity of Fluids. Ethane. *J. Chem. Eng. Data* **1963**, *8*, 281–285. https://doi.org/10.1021/je60018a001.
(26) Assael, M. J.; Karagiannidis, L. Measurements of the Thermal Conductivity of Liquid R32, R124, R125, and R14 1b. *Int. J. Thermophys.* **1995**, *16*, 851–865. https://doi.org/10.1007/bf02093468.
(27) Akhundov, T. S.; Gasanova, N. E. Experimental Study of Thermal Conductivity for Toluene. *Izv. Vyssh. Uchebn. Zaved., Neft Gaz* **1969**, *12*, 59–63.
(28) Zederberg, v. n.; Popov, V. N. Experimental Invesnigation of He Thermal Conductivity. *Teploenergetika* **1958**, 61–65.
(29) Yata, J.; Hori, M.; Hagiwara, T.; Minamiyama, T. Thermal Conductivity of Propane and Butane in the Liquid Phase. *Fluid Phase Equilib.* **1996**, *125*, 267–274. https://doi.org/10.1016/s0378-3812(96)03091-9.
(30) Vidal, D.; Tufeu, R.; Garrabos, Y.; Le Neindre, B. Thermophysical Properties of Noble Gases at Room Temperature up to 1 GPa. *High-Pressure Sci. Technol.* **1980**, *2*, 692.
(31) Vargaftik, N. B.; Vanicheva, N. A.; Yakush, L. V. Thermal Conductivity of Heavy Water in the Gas Phase. *Inzh.-Fiz. Zh.* **1973**, *25*, 336–340.
(32) Vargaftik, N. B.; Vanicheva, N. A. Experimental Study of the Thermal Conductivity of C6D6, C6H6,C6H12, C6D12, in the Gaseous Phase. *Inzh.-Fiz. Zh.* **1977**, *32*, 406–409.
(33) Vargaftik, N. B.; Tarzimanov, A. A. The Experimental Investigation the Thermal Conductivity of the Water Steam under High Parameters of State. *Teploenergetika* **1960**, 12–16.
(34) Vargaftik, N. B.; Tarzimanov, A. A. Experimental Study of the Thermal Conductivity of High-Pressure Water Steam. *Teploenergetika* **1959**, 15–20.
(35) Van Dael, W.; Cauvenbergh, H. Measurements of the Thermal Conductivity of Gases. I. Experimental Method. Data for Pure Gases. *Physica (Amsterdam)* **1968**,

40, 165–172. https://doi.org/10.1016/0031-8914(68)90014-1.
(36) Tufeu, R.; LeNeindre, B.; Bury, P. Determination Du Coefficient de Conductibilite ThermiQuI Du Methane de 25A 450 C et Jusaui' a 1000 Bars. *Physica (Amsterdam)* **1969**, *44*, 81.
(37) Tsvetkov, O. B.; Laptev, Yu. A. *Proc. Symp. Thermophys. Prop., 8th*; 1982; Vol. Vol. II.
(38) Timrot, D. L. *Par Vysokih Parametrov v Energetike (Steam of High Parameters in Energetics)*; 1950.
(39) Shul' ga, V. M.; El' Darov, F. G.; Atanov, Yu. A.; Kuyumchev, A. A. Thermal Conductivity and Heat Capacity of Liquid Toluene at Temperatures between 255 and 400 K and at Pressures up to 1000 MPa. *Int. J. Thermophys.* **1986**, *7*, 1147. https://doi.org/10.1007/bf00503972.
(40) Shashkov, A. G.; Nesterov, N. A.; Sudnik, V. M.; Aleinikova, V. I. Experimental Study of the Thermal Conductivity of Rare Gas at Low Temperatures. *Inzh.-Fiz. Zh.* **1976**, *30*, 671–679.
(41) Sakiadis, B. C. C.; Coates, J. Thermal Condictivity of Liquids III. *AIChE J.* **1957**, *3*, 121.
(42) Roder, H. M.; Nieto de Castro, C. A. Thermal Conductivity of Liquid Propane. *J. Chem. Eng. Data* **1982**, *27*, 12–15. https://doi.org/10.1021/je00027a002.
(43) Ro, S. T.; Kim, M. S.; Jeong, S. U. Liquid Thermal Conductivity of Binary Mixtures of Difluoromethane (R32) and Pentafluoroethane (R125). *Int. J. Thermophys.* **1997**, *18*, 991–999. https://doi.org/10.1007/bf02575243.
(44) Prasad, R. C.; Wang, G.; Venart, J. E. S. The Thermal Conductivity of Propane. *Int. J. Thermophys.* **1989**, *10*, 1013. https://doi.org/10.1007/bf00503170.
(45) Perkins, R. A.; Huber, M. L. Measurement and Correlation of the Thermal Conductivities of Biodiesel Constituent Fluids: Methyl Oleate and Methyl Linoleate. *Energy Fuels* **2011**, *25*, 2383. https://doi.org/10.1021/ef200417x.
(46) Patek, J.; Klomfar, J.; Capla, L.; Buryan, P. Thermal Conductivity of Carbon Dioxide Methane Mixtures at Temperatures Between 300 and 425K and at Pressures up to 12 MPa. *Int. J. Thermophys.* **2005**, *26*, 577–592. https://doi.org/10.1007/s10765-005-5566-6.
(47) Pan, J.; Wu, J. T.; Liu, Z. G.; Jin, X. G. Measurement of the Thermal Conductivity of Liquid Dimethoxymethane from 240 to 362K. *Int. J. Thermophys.* **2004**, *25*, 701–708. https://doi.org/10.1023/b:ijot.0000034233.54418.d7.
(48) Palczewska-Tulinska, M.; Oracz, P. Selected Physicochemical Properties of Hexamethylcyclotrisiloxane, Octamethylcyclotetrasiloxane, and Decamethylcyclopentasiloxane. *J. Chem. Eng. Data* **2005**, *50*, 1711–1719. https://doi.org/10.1021/je050173+.
(49) Ogiwara, K.; Arai, Y. Measurement and Prediction of Thermal Conductivity of Liquid Organic Compounds. *Netsu Bussei* **1987**, *1*, 52.
(50) Mustafaev, R. A.; Gabulov, D. M. Experimental Study of the Thermal Conductivity of Ethylbenzene at High Temperatures and Pressures. *Teplofiz. Vys. Temp.* **1977**, *15*, 209–210.
(51) Marrucho, I. M.; Oliveira, N. S.; Dohrn, R. Vapor-Phase Thermal Conductivity, Vapor Pressure, and Liquid Density of R365mfc. *J. Chem. Eng. Data* **2002**, *47*, 554–558. https://doi.org/10.1021/je015534+.
(52) Li, X.; Wu, J.; Dang, Q. Thermal Conductivity of Liquid Diethyl Ether, Diisopropyl

Ether, and Di-n-Butyl Ether from (233 to 373) K at Pressures up to 30 MPa. *J. Chem. Eng. Data* **2010**, *55*, 1241–1246. https://doi.org/10.1021/je900615b.
(53) Hammerschmidt, U. Thermal Conductivity of a Wide Range of Alternative Refrigerants Measured with an Improved Guarded Hot-Plate Apparatus. *Int. J. Thermophys.* **1995**, *16*, 1203–1211. https://doi.org/10.1007/bf02081288.
(54) Golubev, I. F.; Sokolova, V. P. Thermal Conductivity of Ammonia at Various Temperatures and Pressures. *Teploenergetika* **1964**, 64–67.
(55) Gollis, M. H.; Belenyessy, L. I.; Gudzinowicz, B. J.; Koch, S. D.; Smith, J. O.; Wineman, R. J. Evaluation of Pure Hydrocarbons as Jet Fuels. *J. Chem. Eng. Data* **1962**, *7*, 311–316. https://doi.org/10.1021/je60013a044.
(56) Geller, Z. I.; Rastorguev, Yu. L.; Ganiev, Yu. A. Thermal Conductivity of Selected Solvents. *Izv. Vyssh. Uchebn. Zaved., Neft Gaz* **1965**, *8*, 79–83.
(57) Geller, V.; Zaporozhan, G. V. Study Thermal Conductivity of Toluene at Low Temperatures. *Izv. Vyssh. Uchebn. Zaved., Neft Gaz* **1974**, *17*, 69–71.
(58) Dohrn, R.; Treckmann, R.; Heinemann, T. Vapor-Phase Thermal Conductivity of 1,1,1,2,2-Pentafluoropropane, 1,1,1,3,3-Pentafluoropropane, 1,1,2,2,3-Pentafluoropropane and Carbon Dioxide. *Fluid Phase Equilib.* **1999**, *158–160*, 1021–1028. https://doi.org/10.1016/s0378-3812(99)00126-0.
(59) Dijkema, K. M.; Niessen, J. G.; Copray, C. A. Determination of Thermal Conductivity, Accomodation Coefficient and Free Convection of 40 Gases with a Thermistor Bridge and with the Thin Wire Capillary Method. In *Pro. XIIIth Thermal Conductivity Conference, Univ. of Missouri*; 1974; pp 152–165.
(60) Correia, P.; Schramm, B.; Schaefer, K. Thermal Conductivity of Gases Over a Wide Temperature Range. *Ber. Bunsen-Ges. Phys. Chem.* **1968**, *72*, 393–399.
(61) Charitidou, E.; Molidou, C.; Assael, M. J. The Thermal Conductivity and Viscosity of Benzene. *Int. J. Thermophys.* **1988**, *9*, 37. https://doi.org/10.1007/bf00503998.
(62) Chaikin, A. M.; Markevich, A. M. ? *Zh. Fiz. Khim.* **1958**, *32*, 116–120.
(63) Cecil, O. B.; Munch, R. H. Thermal Conductivity of Some Organic Liquids. *Ind. Eng. Chem.* **1956**, *48*, 437–440.
(64) Amirkhanov, K. I.; Adamov, A. P.; Magomedov, U. B. Experimental Study of the Thermal Conductivity of Water at Temperatures from 25 to 350 C and at Pressures from 0.1 to 245.3 MPa. *Teplofiz. Vys. Temp.* **1975**, *13*, 89–93.
(65) Zaitseva, L. S. Experimental Study of the Thermal Conductivity of Monoatomic Gases in a Wide Temperature Range. *Zh. Tekh. Fiz.* **1959**, *29*, 497–505.
(66) Yata, J.; Hori, M.; Kobayashi, K.; Minamiyama, T. Thermal Conductivity of Alternative Refrigerants in the Liquid Phase. *Int. J. Thermophys.* **1996**, *17*, 561–571. https://doi.org/10.1007/bf01441503.
(67) Watanabe, H.; Seong, D. J. The Thermal Conductivity and Thermal Diffusivity of Liquid N-Alkanes: CnH2n+2 (N=5 to 10) and Toluene. *Int. J. Thermophys.* **2002**, *23*, 337–356. https://doi.org/10.1023/a:1015158401299.
(68) Wang, Y.; Wu, J.; Xue, Z.; Liu, Z. Thermal Conductivity of HFC-245fa from (243 to 413) K. *J. Chem. Eng. Data* **2006**, *51*, 1424–1428. https://doi.org/10.1021/je060116w.
(69) Wang, C. Y.; Yang, M.-L. A New Calorimeter for Measuring Rapidly the Thermal Conductivity of Liquids. *Thermochim. Acta* **1995**, *255*, 365–370. https://doi.org/10.1016/0040-6031(94)02013-e.
(70) Voss, S. F.; Sloan, E. D. Thermal Conductivity and Heat Capacity of Synthetic Fuel

Components. *Int. J. Thermophys.* **1989**, *10*, 1029–1040. https://doi.org/10.1007/bf00503171.
(71) Usmanov, I. U.; Salikhov, A. S. Thermal Conductivity of Three-Component Solutions of Liquids. *Izv. Vyssh. Uchebn. Zaved., Neft Gaz* **1975**, *18*, 61–64.
(72) Tufeu, R.; Bury, P.; Neindre, B. L. Thermal Conductivity Measurement of Heavy Water over a Wide Range of Temperature and Pressure. *J. Chem. Eng. Data* **1986**, *31*, 246–249. https://doi.org/10.1021/je00044a032.
(73) Tsvetkov, O. B.; Laptev, Yu. A.; Asambaev, A. G. Thermal Conductivity of Refrigerants R123, R134a, and R125 at Low Temperatures. *Int. J. Thermophys.* **1994**, *15*, 203–214. https://doi.org/10.1007/bf01441582.
(74) Tarzimanov, A. A.; Yuzmukhametov, F. D.; Gabitov, F. R.; Sharafutdinov, R. A.; Shakirov, N. Z. Thermal Conductivity and Thermodiffusivity of Liquid Aromatic Hydrocarbons, Not Corrected with Radiation Heat Transfer. *Teplofiz. Vys. Temp.* **2002**, *40*, 568–574.
(75) Sun, L.; Venart, J. E. S.; Prasad, R. C. The Thermal Conductivity, Thermal Diffusivity, and Specific Heat of Liquid n-Pentane1. *Int. J. Thermophys.* **2002**, *23*, 391–420. https://doi.org/10.1023/a:1015105402207.
(76) Sun, L.-Q.; Zhu, M.-S.; Han, L.-Z.; Lin, Z.-Z. Thermal Conductivity of Gaseous Difluoromethane and Pentafluoroethane near the Saturation Line. *J. Chem. Eng. Data* **1997**, *42*, 179–182. https://doi.org/10.1021/je960245k.
(77) Smith, J. F. D. Thermal Conductivity of Liquids. *Ind. Eng. Chem.* **1930**, *22*, 1246–1251.
(78) Slusarev, V. V. The Investigation of Thermal Conductivity of Freons of the Ethane Type of Fluorocarbons. PhD Thesis, Odessa Technological Institute of Food Industry, 1979.
(79) Shakhverdiev, A. N.; Naziev, D. Y.; Aliev, A. M.; Aliev, N. S. Molecular Thermal Conductivity of N-Heptane at High Pressures. *Izv. Vyssh. Uchebn. Zaved., Neft Gaz* **1991**, 61–64.
(80) Saxena, S. C.; Tondon, P. K. Experimental Data and Procedures for Predicting Thermal Conductivity of Binary Mixtures of Nonpolar Gases. *J. Chem. Eng. Data* **1971**, *16*, 212–220. https://doi.org/10.1021/je60049a032.
(81) Richter, G. N.; Sage, B. H. Thermal Conductivity of Fluids. Ammonia. *J. Chem. Eng. Data* **1964**, *9*, 75–78. https://doi.org/10.1021/je60020a022.
(82) Raal, J. D.; Rijsdijk, R. L. Measurement of Alcohol Thermal Conductivities Using a Relative Strain- Compensated Hot-Wire Method. *J. Chem. Eng. Data* **1981**, *26*, 351–359. https://doi.org/10.1021/je00026a001.
(83) Papadaki, M.; Wakeham, W. A. Thermal Conductivity of R32 and R125 in the Liquid Phase at the Saturation Vapor Pressure. *Int. J. Thermophys.* **1993**, *14*, 1215–1220. https://doi.org/10.1007/bf02431285.
(84) Ogiwara, K.; Arai, Y.; Saito, S. Thermal Conductivity of Liquids and Their Mixtures for Hydrocarbons and Alcohols. *J. Chem. Eng. Jpn.* **1985**, *18*, 273.
(85) Nagasaka, Y.; Nagashima, A. Precise Measurements of the Thermal Conductivity of Toluene and N-Heptane by the Absolute Transient Hot-Wire Method. *Ind. Eng. Chem. Fundam.* **1981**, *20*, 216.
(86) Mallan, G. M.; Michaelian, M. S.; Lockhart, F. J. Liquid Thermal Conductivities of Organic Compounds and Petroleum Fractions. *J. Chem. Eng. Data* **1972**, *17*, 412–415. https://doi.org/10.1021/je60055a028.

(87) Leng, D. E.; Commings, E. W. Thermal Condictivity of Propane. *Ind. Eng. Chem.* **1957**, *49*, 2042.
(88) LeNeindre, B. Contribution a L' etude Esperimentale de La Conductivite Thermique de Quelqves Fluides a Haute Temperature et a Haute Pression. *Int. J. Heat Mass Transfer* **1972**, *15*, 1. https://doi.org/10.1016/0017-9310(72)90162-7.
(89) Laesecke, A.; Perking, R. A.; Nieto de Castro, C. A. Thermal Conductivity of R134a. *Fluid Phase Equilib.* **1992**, *80*, 263–274. https://doi.org/10.1016/0378-3812(92)87073-v.
(90) Grigor' ev, B. A.; Ishkhanov, A. M. Study of the Thermal Conductivity Oil Hydrocarbons at High Pressures. *Inzh.-Fiz. Zh.* **1981**, *41*, 491–499.
(91) Geller, Z. I.; Geller, V. Z.; Eizenveis, V. P. Experimental Study to Determine the Thermal Conductivity of Bromided Freons F-113 V2 and F-114 V2. *Inzh.-Fiz. Zh.* **1972**, *22*, 209.
(92) Geller, V. Z. *Teplofiz. Svoistva Veshch. Mater.*; 1985.
(93) Frezzotti, D.; Goffredi, G.; Bencini, E. Thermal Conductivity Measurements of Cis- and Trans- Decahydronaphthalene Isomers Using a Steady-State Coaxial Cylinders Method. *Thermochim. Acta* **1995**, *265*, 119–128. https://doi.org/10.1016/0040-6031(95)02416-y.
(94) Filipov, L. P.; Laushkina, L. A. Study of Thernmal Conductance and Heat Capacity of Liquids I. Ismers of Alkanes. *Zh. Fiz. Khim.* **1984**, *58*, 1068–1071.
(95) Desmarest, P.; Tufeu, R. Thermal Conductivity of Ethane in the Critical Region. *Int. J. Thermophys.* **1987**, *8*, 293. https://doi.org/10.1007/bf00503943.
(96) Clerc, H.; Tufeu, R.; Neindre, B. L. Experimental Determination of the Thermal Conductivity of Hydrogen Isotopes (Hydrogen and Deuterium) and Helium Isotopes (Helium-3 and Helium-4) from 25deg C to 100deg C and up to 60 MPa. In *Symposium on Thermophysical Properties*; 1977.
(97) Challoner, A. R.; Gundry, H. A.; Powell, R. W. A Radial Heat Flow Apparatus for Liquid Thermal Conductivity Determinations. *Proc. R. Soc. London, Ser. A* **1958**, *245*, 259.
(98) Castelli, V. J.; Stanley, E. M. Thermal Conductivity of Distilled Water as Function of Pressure and Temperature. *J. Chem. Eng. Data* **1974**, *19*, 8–11. https://doi.org/10.1021/je60060a021.
(99) Carmichael, L. T.; Sage, B. H. Thermal Conductivity of Fluids. n-Butane. *J. Chem. Eng. Data* **1964**, *9*, 511–515. https://doi.org/10.1021/je60023a012.
(100) Bogatov, G. F. Thermal Conductivity of Liquid N-Pentane. *Izv. Vyssh. Uchebn. Zaved., Neft Gaz* **1969**, *12*, 60–68.
(101) Bakulin, S. S.; Ulybin, S. A.; Zherdev, E. N. Experimental Study of the Thermal Conductivity of Xenon at Temperatures from 400 to 1400 K. *Teplofiz. Vys. Temp.* **1975**, *13*, 760–763.
(102) Atalla, S. R.; El-Sharkawy, A. A.; Gasser, F. A. Measurement of Thermal Properties of Liquids with an a.c. Heated-Wire Technique. *Int. J. Thermophys.* **1981**, *2*, 155–162. https://doi.org/10.1007/bf00503938.
(103) Abdulagatov, I. M.; Akhmedova-Azizova, L. A. A.; Azizov, N. D. Thermal Conductivity of Binary Aqueous NaBr and KBr and Ternary H2O + NaBr + KBr Solutions at Temperatures from (294 to 577) K and Pressures up to 40 MPa. *J. Chem. Eng. Data* **2004**, *49*, 1727–1737. https://doi.org/10.1021/je049814b.
(104) Zaporozhan, G. V.; Lenskiy, L. R.; Baryshev, V. P.; Geller, V. Z. Investigation of the

Thermal Conductivity of Freons 218 and 115. *Izv. Vyssh. Uchebn. Zaved., Energ.* **1975**, 146–160.
(105) Yamamoto, R.; Matsuo, S.; Tanaka, Y. Thermal Conductivity of Halogenated Ethanes, HFC-134a, HCFC-123, and HCFC-1 41b. *Int. J. Thermophys.* **1993**, *14*, 79–90. https://doi.org/10.1007/bf00522663.
(106) Wang, Y.; Wu, J.; Liu, Z. Thermal Conductivity of Dimethyl Ether in Saturated Liquid Phase. *Xi' an Jiaotong Daxue Xuebao* **2005**, *39*, 1026–1034.
(107) Vargaftik, N. B.; Zimina, N. K. Thermal Conductivity of Argon at High Temperatures. *Teplofiz. Vys. Temp.* **1964**, *2*, 716.
(108) Vargaftik, N. B.; Oleshuk, O. N. Thermal Conductivity of Heavy Water. *Teploenergetika* **1962**, 64–66.
(109) Tomida, D.; Kenmochi, S.; Tsukada, T.; Yokoyama, C. Measurements of Thermal Conductivity of 1-Butyl-3-Methylimidazolium Tetrafluoroborate at High Pressure. *Netsu Bussei* **2006**, *20*, 173–178. https://doi.org/10.2963/jjtp.20.173.
(110) Timrot, D. L.; Makhrov, V. V. Thermoelectrical Method Determination of the Thermal Conductivity of Gases and Liquids. Study of the Thermal Conductivity of Acetic Acide Vapors. *Inzh.-Fiz. Zh.* **1976**, *31*, 965–972.
(111) Tanaka, Y.; Miyake, A.; Kashiwagi, H.; Makita, T. Thermal Conductivity of Liquid Halogenated Ethanes under High Pressures. *Int. J. Thermophys.* **1988**, *9*, 465. https://doi.org/10.1007/bf00503147.
(112) Sirota, A. M.; Latunin, V. I.; Belyaeva, G. M. Experimental Study of the Thermal Conductivity Maximums of Water in the Crtical Region. *Teploenergetika* **1974**, 52–58.
(113) Schuler; Michel. *Z. Naturforsch., A: Astrophys. Phys. Phys. Chem.* **1956**, *11*, 403.
(114) Saxena, S. C.; Gupta, G. P. Experimental Data and Procedures for Predicting Thermal Conductivity of Multicomponent Mixtures of Nonpolar Gases. *J. Chem. Eng. Data* **1970**, *15*, 98–107. https://doi.org/10.1021/je60044a042.
(115) Rastorguev, Yu. L.; Nemzer, V. G. Apparatus for the Study of Thermal Conductivity of High Temperature Working Fluids in the Liquid Phase. *Teploenergetika* **1968**, 78–81.
(116) Rastorguev, Yu. L.; Grigor' ev, B. A.; Ishkhanov, A. M. Experimental Study of the Thermal Conductivity of Heavy Water at High Pressures. *Teploenergetika* **1975**, 81–83.
(117) Ramires, M. L. V.; Vieira dos Santos, F. J.; Mardolcar, U. V.; Nieto de Castro, C. A. The Thermal Conductivity of Benzene and Toluene. *Int. J. Thermophys.* **1989**, *10*, 1005. https://doi.org/10.1007/bf00503169.
(118) Patek, J.; Klomfar, J.; Capla, L.; Buryan, P. Thermal Conductivity of Nitrogen-Methane Mixtures at Temperatures Between 300 and 425 K and at Pressures up to 16 MPa. *Int. J. Thermophys.* **2003**, *24*, 923–935. https://doi.org/10.1023/a:1025024127880.
(119) Neindre, B. L.; Garrabos, Y.; Gumerov, F.; Sabirzianov, A. Measurements of the Thermal Conductivity of HFC-134a in the Supercritical Region. *J. Chem. Eng. Data* **2009**, *54*, 2678–2688. https://doi.org/10.1021/je900210h.
(120) Mustafaev, R. A.; Ragimov, R. S.; Ganiev, D. K.; Garadzhaev, B. G. Thermal Conductivity of Complex Ether of Silicyl Acid in the Wide Temperature Range. *Izv. Vyssh. Uchebn. Zaved., Neft Gaz* **1994**, 58–60.
(121) Mustafaev, R. A. Apparatus for the Theremophysical Study of Liquids at High

Parameters of State at the Continuously Heating Regime. *Inzh.-Fiz. Zh.* **1977**, *32*, 826–833.
(122) Matsuo, S.; Tanaka, Y.; Tanada, N.; Yamamoto, H.; Sekiya, A. Gaseous Thermal Conductivities of Fluorinated Methyl Ethyl Ethers. *J. Chem. Eng. Data* **1998**, *43*, 473–476. https://doi.org/10.1021/je9702854.
(123) Manna, A.; Srivastava, B. N. Thermal Conductivity of Methyl Chloride - Sulfur Dioxide Mixtures. *J. Chem. Phys.* **1969**, *51*, 2137–2140.
(124) Madzhidov, K.; Khalilov, M. Study of the Thermal Conductivity of Dimethyl Ketone at Various Temperatures and Pressures. *Zh. Fiz. Khim.* **1978**, *52*, 542–545.
(125) Lenoir, J. M.; Junk, W. A.; Commings, E. W. Measurement and Correlation of Thermal Condictivities of Gases at High Pressure. *Chem. Eng. Prog.* **1953**, *49*, 539.
(126) Le Neindre, B.; Garrabos, Y. Thermal Conductivity of HFC-152a in the Temperature Range 300 to 455 K at Pressures up to 55 MPa. *High Temp. - High Pressures* **2002**, *34*, 307–314.
(127) Kestin, J.; Paul, R.; Clifford, A. A.; Wakeham, W. A. Absolute Determination of the Thermal Conductivity of the Noble Gases at Room Termperature up to 35 MPa. *Phys. A (Amsterdam, Neth.)* **1980**, *100*, 349–369. https://doi.org/10.1016/0378-4371(80)90125-9.
(128) Kestin, J.; Imaishi, N. Thermal Conductivity of Sulfur Hexafluoride. *Int. J. Thermophys.* **1985**, *6*, 107–118. https://doi.org/10.1007/bf00500026.
(129) Gurova, A. N.; Mardolcar, U. V.; Nieto de Castro, C. A. The Thermal Conductivity of Liquid 1,1,1,2-Tetrafluoroethane (HFC 1 34a). *Int. J. Thermophys.* **1997**, *18*, 1077–1087. https://doi.org/10.1007/bf02575250.
(130) Golubev, I. F.; Kal’ sina, M. V. Thermal Conductivity of Nitrogen and Hydrogen at Temperatures from 20 to -195 C and Pressures from 1 to 500 Bar. *Gaz. Prom.* **1964**, *9*, 41–43.
(131) Dickins, B. G. The Effect of Accommodation on Heat Conduction through Gases. *Proc. R. Soc. London, Ser. A* **1934**, *143*, 517.
(132) Chen, Q.-L.; Wu, K.-J.; He, C.-H. Thermal Conductivities of [EMIM][EtSO4], [EMIM][EtSO4] + C2H5OH, [EMIM][EtSO4] + H2O, and [EMIM][EtSO4] + C2H5OH + H2O at T = (283.15 to 343.15) K. *J. Chem. Eng. Data* **2013**, *58*, 2058–2064. https://doi.org/10.1021/je400268t.
(133) Carey, C.; Bradshaw, J.; Lin, E.; Carnevale, E. H. *Experimental Determination of Gas Properties at High Temperatures and/or Pressures*; AEDC-TR-74-33; Panametrics Inc. Experimetnal Determination of Gas Properties at High Temperatures and/or Pressures: Waltham, MA, 1974.
(134) Beirao, S. G. S.; Ramires, M. L. V.; Dix, M.; Nieto de Castro, C. A. A New Instrument for the Measurement of the Thermal Conductivity of Fluids. *Int. J. Thermophys.* **2006**, *27*, 1018–1041. https://doi.org/10.1007/s10765-006-0093-7.
(135) Assael, M. J.; Karagiannidis, E. Measurements of the Thermal Conductivity of R22, R123, and R134a in the Temperature Range 250-340 K at Pressures up to 30 MPa. *Int. J. Thermophys.* **1993**, *14*, 183–197. https://doi.org/10.1007/bf00507807.
(136) Assael, M. J.; Charitidou, E.; Avgoustiniatos, S. The Thermal Conductivity of Xylene Isomers in the Temperature Range 290-360 K. *Int. J. Thermophys.* **1988**, *9*, 501. https://doi.org/10.1007/bf00503149.

(137) Afshar, R.; Saxena, S. C. Transport Properties of Freon-152a and Freon-142b in the Temperature Range of 280 510 K. *Int. J. Thermophys.* **1980**, *1*, 51–59. https://doi.org/10.1007/bf00506271.
(138) Zhou, J.-C.; Che, Y.-Y.; Wu, K.-J.; Shen, J.; He, C.-H. Thermal Conductivity of DMSO + C2H5OH, DMSO + H2O, and DMSO + C2H5OH + H2O Mixtures at T = (278.15 to 338.15) K. *J. Chem. Eng. Data* **2013**, *58*, 663–670. https://doi.org/10.1021/je301171y.
(139) Zaitseva, L. S. Experimentalnoe Issledovanie Teploprovodnosti Parov Benzina B-70, Kerosina T-1, Topliva T-5, Geptana. *Tr. Mosk. Aviats. Inst.* **1961**, 79–93.
(140) Wu, J.; Li, X.; Zheng, H.; Assael, M. J. Thermal Conductivity of Liquid Dimethyl Ether from (233 to 373) K at Pressures up to 30 MPa. *J. Chem. Eng. Data* **2009**, *54*, 1720–1723. https://doi.org/10.1021/je800964k.
(141) Vojtenko, A. K. Investigation of the Thermal Conductivity of Ome Freons in the Wide Range of Parameters Including the Critical Region. PhD Thesis, Grozny Petroleum Institute, 1980.
(142) Varlashkin, P. G.; Thompson, J. C. Thermal Conductivity of Liquid Ammonia. *J. Chem. Eng. Data* **1963**, *8*, 526–526. https://doi.org/10.1021/je60019a014.
(143) Vanderkooi, W. N.; Hildenbrand, D. L.; Stull, D. R. Liquid Thermal Conductivities: The Apparatus, Values for Several Glycols and Their Aqueous Solutions, and Five High Molecular Weight Hydrocarbons. *J. Chem. Eng. Data* **1967**, *12*, 377–379. https://doi.org/10.1021/je60034a023.
(144) Tufeu, R.; LeNeindre, B. Thermal Conductivity of Propane in the Temperature Range 25-305.Degree.C and Pressure Range 1-70 MPa. *Int. J. Thermophys.* **1987**, *8*, 27. https://doi.org/10.1007/bf00503222.
(145) Tsvetkov, O. B.; Laptev, Yu. A. Thermal Conductivity of Difluoromonochloromethane in the Critical Region. *Int. J. Thermophys.* **1991**, *12*, 53–65. https://doi.org/10.1007/bf00506122.
(146) Tsvetkov, O. B.; Kletskii, A. V.; Laptev, Yu. A.; Asambaev, A. J.; Zausaev, I. A. Thermal Conductivity and PVT Measurements of Pentafluoroethane (Refrigerant HFC-125). *Int. J. Thermophys.* **1995**, *16*, 1185–1192. https://doi.org/10.1007/bf02081286.
(147) Tarzimanov, A. A.; Zainullin, M. M. The Results of the Measurements of the Thermal Conductivity of Water Vapor at Pressures up to 1000 Bar. *Teploenergetika* **1973**, 2–10.
(148) Swift, G. W.; Migllori, A. Measurement of the Thermal Conductivity and Viscosity of Liquid Propylene. *J. Chem. Eng. Data* **1984**, *29*, 56–59. https://doi.org/10.1021/je00035a019.
(149) Slusar', V. P.; Tret'yakov, V. M.; Rudenko, N. S. Thermal Conductivity of Argon at Constant Density and Pressures up to 2600 Atm. *Ukr. Fiz. Zh.* **1977**, *22*, 1070–1074.
(150) Senftleben, H. *Z. Angew. Phys.* **1953**, *5*, 33.
(151) Prasad, R. C.; Mani, N.; Venart, J. E. S. Thermal Conductivity of Methane. *Int. J. Thermophys.* **1984**, *5*, 265. https://doi.org/10.1007/bf00507836.
(152) Perkins, R. Measurement and Correlation of the Thermal Conductivity of Isobutane from 114 K to 600 K at Pressures to 70 MPa. *J. Chem. Eng. Data* **2002**, *47*, 1272–1279. https://doi.org/10.1021/je010121u.
(153) Papadaki, M.; Schmitt, M.; Seitz, A.; Stephan, K.; Taxis, B.; Wakeham, W. A.

Thermal Conductivity of R134a and R141b within the Temperature Range 240-30 7 K at the Saturation Vapor Pressuree. *Int. J. Thermophys.* **1993**, *14*, 173–181. https://doi.org/10.1007/bf00507806.
(154) Nefedov, S. N.; Filippov, L. P. Experimental Study of the Thermophysical Properties of Toluene. Thermal Conductivity. *Izv. Vyssh. Uchebn. Zaved., Neft Gaz* **1979**, *22*, 47–51.
(155) Naziev, Y. M.; Nurbediev, A. A.; Abasov, A. A. Thermal Conductivity Pf N-Heptane Ant High Pressure. *Chem. Tech.* **1971**, *23*, 738.
(156) Naziev, Y. M.; Bashirov, M. M.; Abdulagatov, I. M. High-Temperature and High-Pressure Experimental Thermal Conductivity for the Pure Methanol and Binary Systems Methanol + n-Propanol, Methanol + n-Octanol, and Methanol + n-Undecanol. *Fluid Phase Equilib.* **2004**, *226*, 221–235. https://doi.org/10.1016/j.fluid.2004.08.033.
(157) Minter, C. C.; Schuldiner, S. Thermal Conductivity of Equilibrated Mixtures of H2, D2, and HD. *J. Chem. Eng. Data* **1959**, *4*, 223–226. https://doi.org/10.1021/je60003a010.
(158) Mardolcar, U. V.; Nieto de Castro, C. A. The Thermal Conductivity of Liquid Methane. *Ber. Bunsen-Ges. Phys. Chem.* **1987**, *91*, 152.
(159) Mallan, G. M. Thermal Conductivity of Liquids. PhD Thesis, University of Southern California, 1968.
(160) Lee, S. H.; Kim, M. S.; Ro, S. T. Themal Conductivity of 1,1,1-Trifluoroethane (R143a) and R404A in the Liquid Phase. *J. Chem. Eng. Data* **2001**, *46*, 1013–1015. https://doi.org/10.1021/je000230t.
(161) Konstantinov, V. A.; Manzhelii, V. G.; Smirnov, S. A.; Tolkachev, A. M. Heat Transfer in Solid Carbon Dioxide and Nitrogen Oxide (N2O) : Dependence on Temperature and Volume. *Fiz. Nizk. Temp. (Kiev)* **1988**, *14*, 189–195.
(162) Kolomiets, A. Y. Experimental Investigation of the Thermal Conductivity of Liqht Hydrocarbons and Their Mixtures. PhD Thesis, Moscow Energy Institute, 1974.
(163) Kim, S. H.; Kim, D. S.; Kim, M. S.; Ro, S. T. The Thermal Conductivity of R22, R142b, R152a, and Their Mixtures in the Liquid State. *Int. J. Thermophys.* **1993**, *14*, 937–950. https://doi.org/10.1007/bf00502116.
(164) Keyes, F. G. Thermal Conductivity of Gases. *Trans. Am. Soc. Mech. Eng.* **1954**, *76*, 809–818.
(165) Hahne, E.; Gross, U.; Song, Y. W. The Thermal Conductivity of R115 in the Critical Region. *Int. J. Thermophys.* **1989**, *10*, 687–700. https://doi.org/10.1007/bf00507989.
(166) Guseinov, G. G.; Abdulagatov, I. M. Thermal-Conductivity Measurements of Aqueous Orthophosphoric Acid Solutions in the Temperature Range from (293 to 400) K and at Pressures up to 15 MPa. *Int. J. Thermophys.* **2014**, *35*, 218–245. https://doi.org/10.1007/s10765-014-1598-0.
(167) Gross, U.; Song, Y. W.; Hahne, E. Thermal Conductivity of the New Refrigerants R134a, R152a, and R123 Measured by the Transient Hot-Wire Method. *Int. J. Thermophys.* **1992**, *13*, 957–983. https://doi.org/10.1007/bf01141209.
(168) Gross, U.; Song, Y. W.; Hahne, E. Measurements of Liquid Thermal Conductivity and Diffusivity by the Transient Hot-Strip Method. *Fluid Phase Equilib.* **1992**, *76*, 273–282. https://doi.org/10.1016/0378-3812(92)85094-o.
(169) Geller, V. Z.; Ivanchenko, S. I.; Peredriy, V. G. Experimental Investigation of

Dynamic Viscosity and Thermal Conductivity Coeficients of Difluorudichloromethane. *Izv. Vyssh. Uchebn. Zaved., Neft Gaz* **1973**, 61–65.
(170) Franck, H. G. *Angew. Chem.* **1951**, *63*, 260.
(171) Franck, E. U. Temperature Dependence of the Thermal Conductivity of Several Gases. *Z. Elektrochem.* **1951**, *55*, 636.
(172) DiGuilio, R. M.; McGregor, W. L.; Teja, A. S. Thermal Conductivities of the Ethanolamines. *J. Chem. Eng. Data* **1992**, *37*, 242–245. https://doi.org/10.1021/je00006a029.
(173) Cheung, H.; Bromley, L. A.; Wilke, C. R. Thermal Conductivity of Gas Mixtures. *AIChE J.* **1962**, *8*, 221–228.
(174) Cherneeva, L. I. Investigation of the Thermal Conductivity of Freons. *Kholod. Tekhn.* **1955**, *32*, 23–24.
(175) Carmichael, L. T.; Reamer, H. H.; Sage, B. H. Thermal Conductivity of Fluids. Methane. *J. Chem. Eng. Data* **1966**, *11*, 52–57. https://doi.org/10.1021/je60028a014.
(176) Cai, G.; Zong, H.; Yu, Q.; Lin, R. Thermal Conductivity of Alcohols with Acetonitrile and N,N- Dimethylformamide. *J. Chem. Eng. Data* **1993**, *38*, 332–335. https://doi.org/10.1021/je00010a038.
(177) Akhundov, T. S. Experimental Study of the Thermal Conductivity of Ethylbenzene. *Izv. Vyssh. Uchebn. Zaved., Neft Gaz* **1974**, *17*, 24–62.
(178) Afshar, R.; Saxena, S. C. Transport Properties of Dichlorodifluoromethane in the Temperature Range 28 0-510K. *Chem. Eng. Commun.* **1980**, *4*, 539–545.
(179) Abbas, R.; Ihmels, E. C.; Enders, S.; Gmehling, J. Measurement of Transport Properties for Selected Siloxanes and Their Mixtures Used as Working Fluids for Organic Rankine Cycles. *Ind. Eng. Chem. Res.* **2011**, *50*, 8756–8763. https://doi.org/10.1021/ie2002632.
(180) Zvetkob, O. B.; Laptev, Yu. A.; Vasilkov, A. I. *The Results of Measurements of Thermal Conductivity of Gaseous Frons with the Heating Wire Method*; 2; Mashinyi i Apparatyi Cholodilnoj, Kriogennoh Techniki i Kondizionirovaniyi Vosducha: 54-6, Leningradskij Tech. Inst., 1977.
(181) Yao, C.; Zhao, X.; Lv, S.; Guo, Z. Thermal Conductivity of Ethyl Fluoride (HFC161). *Fluid Phase Equilib.* **2014**, *375*, 228–235. https://doi.org/10.1016/j.fluid.2014.04.038.
(182) Uhlir, A. Thermal Conductivity of Fluid Argon and Nitrogen. *J. Chem. Phys.* **1952**, *20*, 463.
(183) Tufeu, R.; LeNeindre, B. Thermal Conductivity of Steam from 250 to 510.Degree.C at Pressures up to 95 MPa Including the Critical Region. *Int. J. Thermophys.* **1987**, *8*, 283. https://doi.org/10.1007/bf00503942.
(184) Totsky, E. E.; Burinsky, V. V.; Nikodimov, S. P. The Thermal Conductivity of Sulfur Hexafluoride. *Teplofiz. Vys. Temp.* **1984**, *22*, 48.
(185) Tarzimanov, A. A. Thermal Conductivity of Water near the Saturation Curve. *Teploenergetika* **1962**, 73–77.
(186) Tanaka, Y.; Noguchi, M.; Kubota, H.; Makita, T. Thermal Conductivity of Gaseous Methane and Sulfur Hexabluoride Under Pressure. *J. Chem. Eng. Jpn.* **1979**, *12*, 171.
(187) Tanaka, Y.; Matsuo, S.; Taya, S. Gaseous THermoal COndictivity of Difljoromethane (HFC-32), Pentafluoroethane (HFC), and Their Mixtures. In

Sympositum on Thermophysical Properties, 12-th; Boulder CO, 1994.
(188) Stefanov, B.; Zarkova, L.; Oliver, D. A. Measurements of the Thermal Conductivity of Gases and Vapor up to 2500 K. *Teplofiz. Vys. Temp.* **1976**, *14*, 56–66.
(189) Schottky, W. E. Zur Messung Der Warmeleitfahigkeit von Gasen Bei Hoheren Temperaturen. *Z. Elektrochem.* **1952**, *56*, 889.
(190) Rowley, R. L.; Yi, S.-C.; Gubler, V.; Stoker, J. M. Mutual Diffusivity, Thermal COnductivity, and Heat of Transport in Binary Liquid Mixtures of Alkanes in Carbon Tetrachloride. *Fluid Phase Equilib.* **1987**, *36*, 219–233. https://doi.org/10.1016/0378-3812(87)85025-2.
(191) Rastorguev, Yu. L.; Grigor' ev, B. A.; Ishkhanov, A. M. Experimental Study of the Thermal Conductivity of Sulfur Hexafluoride at High Pressures. *Teploenergetika* **1977**, 78–81.
(192) Qun-Fang, L.; Ruisen, L.; Dan-Yan, N.; Yu-Chun, H. Thermal Conductivities of Some Organic Solvents and Their Binary Mixtures. *J. Chem. Eng. Data* **1997**, *42*, 971–974. https://doi.org/10.1021/je960351m.
(193) Perkins, R.; Ramires, M. L. V.; Nieto de Castro, C. A.; Cusco, L. Measurement and Correlation of the Thermal Conductivity of Butane from 135 K to 600 K at Pressures to 70 MPa. *J. Chem. Eng. Data* **2002**, *47*, 1263–1271. https://doi.org/10.1021/je0101202.
(194) Perkins, R. A.; Hammerschmidt, U.; Huber, M. L. Measurement and Correlation of the Thermal Conductivity of Methylcyclohexane and Propylcyclohexane from 300 K to 600 K at Pressures to 60 MPa. *J. Chem. Eng. Data* **2008**, *53*, 2120–2127. https://doi.org/10.1021/je800255r.
(195) Parkinson, W. J. Thermal Conductivity of Binary Liquid Mixtures. PhD Thesis, University of Southern California, 1974.
(196) Palavra, A. M. F.; Wakeham, W. A.; Zalaf, M. Thermal Conductivity of Normal Pentane in the Temperature Range 306-360 K at Pressures up to 0.5 GPa. *Int. J. Thermophys.* **1987**, *8*, 305. https://doi.org/10.1007/bf00503944.
(197) Nuttall, R. L.; Ginnings, D. C. Thermal Conductivity of Nitrogen from 50 to 500 C and 1 to 100 Atm. *J. Res. Natl. Bur. Stand. (U. S.)* **1957**, *58*, 271.
(198) Nieto de Castro, C. A.; Tufeu, R.; LeNeindre, B. Thermal Conductivity Meassurement of N-Butane Over Wide Temperature and Pressure Ranges. *Int. J. Thermophys.* **1983**, *4*, 11. https://doi.org/10.1007/bf00504479.
(199) Naziev, Y. M.; Gumbatov, A. M.; Akhmedov, A. K. Study of the Thermal Conductivity of N-Pentane, n-Heptane and Their Mixtures with n-Hexane in the Gaseous State at High Temperatures. *Izv. Vyssh. Uchebn. Zaved., Neft Gaz* **1984**, *27*, 50–53.
(200) Naziev, Y. M.; Gumbatov, A. M.; Akhmedov, A. K. Study of the Thermal Conductivity of Some Liquid Hydrocarbons at Various Temperaturs and Pressures. *Izv. Vyssh. Uchebn. Zaved., Neft Gaz* **1981**, 43–47.
(201) Missenard, F. A. Thermal Conductivities of Pure Gases at Different Temperatures and Pressures. *Revue Generale de Thermique* **1966**, *5*, 125–137.
(202) Miroshnichenko, V. I.; Makhrov, V. V. Experimental Study of the Thermal Conductivity of Water at Temperatures up to 100 C. *Teploenergetika* **1981**, 64–65.
(203) Li, S. F. Y.; Maitland, G. C.; Wakeham, W. A. Thermal Conductivity of Benzene and Cyclohexane in the Temperature Range 36 -90 C at Pressures up to 0.33 GPa. *Int.*

J. Thermophys. **1984**, *5*, 351–365. https://doi.org/10.1007/bf00500866.
(204) LeNeindre, B.; Bury, P.; Tufeu, R.; Vodar, B. Thermal Conductivity Coefficients of Watrer and Heavy Water in the Liquid State up to 370 Deg C. *J. Chem. Eng. Data* **1976**, *21*, 265–274. https://doi.org/10.1021/je60070a018.
(205) Kardos, A. The Heat Conductivities of Various Liquids. *Z. Gesamte Kalte-Ind.* **1934**, *41*, 29–35.
(206) Kannuluik, W. G.; Carman, E. H. The Thermal Conductivity of Rare Gases. *Proc. Phys. Soc., Sect. B* **1952**, *65*, 701.
(207) Guseinov, K. D.; Klimova, T. F. Study of the Thermal Conductivity of Amyl- and Isoamyl Propionate. *Inzh.-Fiz. Zh.* **1980**, *39*, 658–663.
(208) Grebenkov, A. J.; Beliayeva, O. V.; Klepatski, P. M.; Saplitsa, V. V.; Timofeyev, B. D.; Tsurbelev, V. P.; Zayats, T. A. *Thermophysical Properties of R245fa*; 1256-RP; Joint Institute for Power and Nuclear Research: Sosny (Belarus), 2004.
(209) Das Gupta, A. THERMAL CONDUCTIVITY OF BINARY MIXTURES OF SULPHUR DIOXIDE AND INERT GASES. *Int. J. Heat Mass Transfer* **1967**, *10*, 921–929.
(210) Clifford, A. A.; Kestin, J.; Wakeham, W. A. The Thermal Conductivity of Hydrogen, Deuterium and Their Mixtures Near Room Temperature within the Pressure Range 2-36 MPa. *Ber. Bunsen-Ges. Phys. Chem.* **1980**, *84*, 9–18.
(211) Burgdorf, R.; Zocholl, A.; Arlt, W.; Knapp, H. Thermophysical Properties of Binary Liquid Mixtures of Polyether and N-Alkane at 298.15 and 323.15 K: Heat of Mixing, Heat Capacity, Viscosity, Density and Thermal Conductivity. *Fluid Phase Equilib.* **1999**, *164*, 225–255. https://doi.org/10.1016/s0378-3812(99)00234-4.
(212) Bakulin, S. S.; Ulybin, S. A. Thermal Conductivity of Sulfur Hexafluoride at Temperatures from 230 to 350 K and at Pressures up to 50 MPa. *Teplofiz. Vys. Temp.* **1978**, *16*, 59–66.
(213) Baker, C. E.; Brokaw, R. S. Thermal Conductivities of Gaseous H2O2, D2O, and the Equimolar H2O-D2O Mixture. *J. Chem. Phys.* **1964**, *40*, 1523–1528. https://doi.org/10.1063/1.1725357.
(214) Assael, M. J.; Wakeham, W. A. Thermal Conductivity of Four Polyatomic Gases. *J. Chem. Soc., Faraday Trans. 1* **1981**, *77*, 697–707.
(215) Yata, J.; Minamiyama, T.; Tanaka, S. Measurement of Thermal Conductivity of Liquid Fluorocarbons. *Int. J. Thermophys.* **1984**, *5*, 209. https://doi.org/10.1007/bf00505501.
(216) Watanabe, H. Thermal Conductivity and Thermal Diffusivity of Sixteen Isomers of Alkanes: CnH2n+2 (n = 6 to 8). *J. Chem. Eng. Data* **2003**, *48*, 124–136. https://doi.org/10.1021/je020125e.
(217) Vilim, O. Thermal Conductivity of Hydrocarbons. *Collect. Czech. Chem. Commun.* **1960**, *25*, 993.
(218) Vargaftik, N. B.; Vanicheva, N. A. Experimental Study of the Thermal Conductivity of Some Deterium Containing Compounds in the Gas Phase. *Inzh.-Fiz. Zh.* **1974**, *26*, 282–286.
(219) Turnbull, A. G. Thermal Conductivity of Phosphoric Acid-Water Mixtures at 25 C. *J. Chem. Eng. Data* **1971**, *16*, 79–83. https://doi.org/10.1021/je60048a028.
(220) Tsvetkov, O. B. Thermal Conductivity of Gaseous Difluorodichloromethane. *Inzh.-Fiz. Zh.* **1969**, *16*, 510–515.
(221) Tsederberg, N. V. Thermal Conductivity of a Binary Solution of Benzene in Ethyl Alcohol. *Nauchn. Dokl. Vyssh. Shk., Energ.* **1958**, 189–192.

(222) Tarzimanov, A. A.; Sharafutdinov, R. A.; Gabitov, F. R. Thermal Conductivity of Liquid N-Alkanes and 1-Alkenes Not Distorted by Radiative Energy Transfer. II. Correlation of Experimental Data in a Wide Range of Parameters of State. *Inzh.-Fiz. Zh.* **1990**, *59*, 827–831.
(223) Tarzimanov, A. A.; Mashirov, V. E. Experimental Investigation of the Thermal Conductivity of Vapours of Normal Saturated Hydrocarbons at Temperatures up to 450 Deg C. *Teploenergetika* **1967**, *14*, 67.
(224) Sun, L.; Venart, J. E. S.; Prasad, R. C. The Thermal Conductivity, Thermal Diffusivity and Isobaric Heat Capacity of Toluene and Argon. *Int. J. Thermophys.* **2002**, *23*, 1487–1535. https://doi.org/10.1023/a:1020733832332.
(225) Slyusar, V. P.; Tret'yakov, V. M.; Rudenko, N. S. Thermal Conductivity of Krypton and Xenon at Constant Density and Pressure up to 2700 Atm. Law of Corresponding States. *Fiz. Nizk. Temp. (Kiev)* **1978**, *4*, 764–774.
(226) Schuster, F. *Z. Elektrochem.* **1926**, *32*, 550.
(227) Saxena, S. C.; Saxena, V. K. Thermal Conductivity Data for Hydrogen and Deuterium in the Range 100-1100 Degrees C. *J. Phys. A: Gen. Phys.* **1970**, *3*, 309–320.
(228) Rowley, R. L.; Gubler, V. Thermal Conductivities in Seven Ternary Liquid Mixtures at 40 Deg C and 1 Atm. *J. Chem. Eng. Data* **1988**, *33*, 5–8. https://doi.org/10.1021/je00051a002.
(229) Rastorguev, Yu. L.; Pugach, V. V. Experimental Study of the Thermal Conductivity of Water at High Pressures. *Teploenergetika* **1970**, 77–79.
(230) Rastorguev, Yu. L.; Grigor'ev, B. A.; Bogatov, G. F. Experimental Study of the Thermal Conductivity of Toluene at High Pressures. *Inzh.-Fiz. Zh.* **1969**, *17*, 847–855.
(231) Rastorguev, Yu. L.; Bogatov, G. F.; Grigor'ev, B. A. Thermal Conductivity of Liquids. n-Decane. *Izv. Vyssh. Uchebn. Zaved., Neft Gaz* **1969**, *12*, 69–73.
(232) Rastorguev, Yu. L.; Bogatov, G. F.; Grigor'ev, B. A. Thermal Conductivity of Liquids. Heptane. *Izv. Vyssh. Uchebn. Zaved., Neft Gaz* **1968**, 59.
(233) Prasad, R. C.; Venart, J. E. S. Thermal Conductivity of Ethane from 290 to 600K at Pressures up to 700 Bar, Including the Critical Region. *Int. J. Thermophys.* **1984**, *5*, 367–385. https://doi.org/10.1007/bf00500867.
(234) Perkins, R. A.; Huber, M. L. Measurement and Correlation of the Thermal Conductivity of Pentafluoroethane (R125) from 190 K to 512 K at Pressures to 70 MPa. *J. Chem. Eng. Data* **2006**, *51*, 898–904. https://doi.org/10.1021/je050372t.
(235) Pastoriza-Gallego, M. J.; Lugo, L.; Cabaleiro, D.; Legido, J. L.; Pineiro, M. M. Thermophysical Profile of Ethylene Glycol-Based ZnO Nanofluids. *J. Chem. Thermodyn.* **2014**, *73*, 23–30. https://doi.org/10.1016/j.jct.2013.07.002.
(236) Ogiwara, K.; Arai, Y.; Saito, S. Thermal Conductivities of Liquid Alcohols and Their Binary Mixtures. *J. Chem. Eng. Jpn.* **1982**, *15*, 335–342.
(237) Naziev, Y. M.; Shakhverdiev, A. N.; Abasov, A. A. Study of the Thermal Conductivity of Gaseous and Liquid Methyl- and Ethylcyclohexane at High Pressures and Temperatures. *Izv. Vyssh. Uchebn. Zaved., Neft Gaz* **1979**, *22*, 54–57.
(238) Naziev, Y. M.; Gumbatov, A. M.; Gasanov, A. S.; Abasov, A. A. Experimental Study of the Thermal Conductivity of Liquid Binary Mixtures of Hexane with Ortho-Xylene. *Zh. Fiz. Khim.* **1987**, *61*, 36.

(239) Naziev, A. M.; Aliev, M. A. Teploprovodnost' i Teploemkost' n-Decane Pri Razlichnyh Temperaturah i Davleniah. *Inzh.-Fiz. Zh.* **1973**, *24*, 1033–1038.
(240) Mustafaev, R. A. Experimental Investigation of the Thermal COnductivity of Hydrocarbons in Liquid and Vapor Phase. *Teplofiz. Vys. Temp.* **1974**, *12*, 883.
(241) Mustafaev, R. A. Thermal Conductivity of High Saturated Hydrocarbons in the Wide Temperature and Pressure Ranges. *Inzh.-Fiz. Zh.* **1973**, *24*, 663–668.
(242) Marchenkov, E. I.; Aleinikova, V. I. Study of the Thermal Conductivity of Neon and Argon-Neon Mixture at High Temperatures. *Inzh.-Fiz. Zh.* **1977**, *33*, 848–856.
(243) Madzhidov, K. Experimental Study of the Thermal Conductivity Coefficient of Diethyl Ether at Various Temperatures and Pressures. *Izv. Vyssh. Uchebn. Zaved., Neft Gaz* **1983**, 56–60.
(244) Lenoir, J. M.; Commings, E. W. Thermal Conductivity of Gases. Measurement at High Pressure. *Chem. Eng. Prog.* **1951**, *47*, 223.
(245) Kruppa, B.; Straub, J. Measurement of Thermal Diffusivity of the Refrigerants R22 and R134a by Means of Dynamic Light Scattering. *Fluid Phase Equilib.* **1992**, *80*, 305–321. https://doi.org/10.1016/0378-3812(92)87077-z.
(246) Hemminger, W. The Thermal Conductivity of Gases: Incorrect Results Due to Desorbed Air. *Int. J. Thermophys.* **1987**, *8*, 317–333. https://doi.org/10.1007/bf00503945.
(247) Gao, X.; Yamada, T.; Nagasaka, Y.; Nagashima, A. The Thermal Conductivity of CFC Alternatives HFC-125 and HCFC-141b in the Liquid Phase. *Int. J. Thermophys.* **1996**, *17*, 279–292. https://doi.org/10.1007/bf01443393.
(248) Fujii, M.; Zhang, X.; Imaishi, N.; Fujiwara, S.; Sakamoto, T. Simultaneous Measurements of Thermal Conductivity and Thermal Diffusivity of Liquids Under Microgravity Conditions. *Int. J. Thermophys.* **1997**, *18*, 327–339. https://doi.org/10.1007/bf02575164.
(249) Frurip, D. J.; Curtiss, L. A.; Blander, M. Thermal Conductivity Measurements and Molecular Association in a Series of Alcohol Vapors: Methanol, Ethanol, Isopropanol, and t-Butanol. *Int. J. Thermophys.* **1981**, *2*, 115–132. https://doi.org/10.1007/bf00503936.
(250) Christensen, P. L.; Fredenslund, A. Thermal Conductivity of Gaseous Mixtures of Methane with Nitrogen and Carbon Dioxide. *J. Chem. Eng. Data* **1979**, *24*, 281–283. https://doi.org/10.1021/je60083a034.
(251) Cherneeva, L. I. Investigation of the Thermal Conductivity of Freon-22. *Kholod. Tekhn.* **1953**, *30*, 60–63.
(252) Charitidou, E.; Dix, M.; Assael, M. J.; Nieto de Castro, C. A.; Wakeham, W. A. A Computer-Controlled Instrument for the Measurement of the Thermal Conductivity of Liquids. *Int. J. Thermophys.* **1987**, *8*, 511. https://doi.org/10.1007/bf00503639.
(253) Chaikovskiy, V. F.; Geller, V. Z.; Gorykin, S. F.; Artamonov, S. D.; Bondar', G. E.; Ivanchenko, S. I.; Lenskiy, L. R.; Peredriy, V. G. Comprehensive Investigation of the Thermophysical Properties of Most Important and Promising Regrigerants in Liquid and Gaseous Phase. In *Teplofiz. Svoistva Zhidkostey, Collect. Vol.*; Nauka: Moscow, 1976; pp 108–117.
(254) Bashirov, M. M.; Naziev, Y. M. Study of the Thermal Conductivity of Methanol+n-Hexanol Solutions at High Parameters of State. *Teplofiz. Vys. Temp.* **2003**, *41*,

527–533.
(255) Baroncini, C.; Latini, G.; Pierpaoli, P. Thermal Conductivity of Organic Liquid Binary Mixtures: Measurements and Prediction Methods. *Int. J. Thermophys.* **1984**, *5*, 387–401. https://doi.org/10.1007/bf00500868.
(256) Amirkhanov, Kh. I.; Adamov, A. P.; Magomedov, U. B. Experimental Study of the Thermal Conductivity of Water Vapors at Temperatures from 375 to 600 C and at Prerssures up to 250 MPa. *Inzh.-Fiz. Zh.* **1978**, *34*, 217–220.
(257) Amirkhanov, Kh. I.; Adamov, A. P.; Magomedov, U. B. Experimental Study of the Thermal Conductivity of Heavy Water Vapors at Temperatures from 375 to 600 C and at Pressures up to 250 MPa. *Inzh.-Fiz. Zh.* **1978**, *34*, 114–117.
(258) Akhmedova-Azizova, L. A. A. Thermal Conductivity of Aqueous Mg(NO3)2, Ca(NO3)2, and Ba(NO3)2 Solutions at High Temperatures and High Pressures. *J. Chem. Eng. Data* **2006**, *51*, 510–517. https://doi.org/10.1021/je050393n.
(259) Abdulagatov, I. M.; Azizov, N. D. Thermal Conductivity and Viscosity of Aqueous K2SO4 Solutions at Temperatures from 298 to 575K and at Pressures up to 30MPa. *Int. J. Thermophys.* **2005**, *26*, 593–635. https://doi.org/10.1007/s10765-005-5567-5.
(260) Zaporozhan, G. V. Investigation of the Thermal Conductivity of Some Freons at Low Temperatures. PhD Thesis, Grozny Petroleum Institute, 1978.
(261) Yorizane, M.; Yoshimura, S.; Masuoka, H.; Yoshida, H. Thermal Condictivity of Pure Gases at High Pressure by Use of Coaxial Cylindrical Cell. *Ind. Eng. Chem. Fundam.* **1983**, *22*, 454.
(262) Wang, Y.; Wu, J.; Liu, Z. Thermal Conductivity of Gaseous Dimethyl Ether from (263 to 383) K. *J. Chem. Eng. Data* **2006**, *51*, 164–168. https://doi.org/10.1021/je050305z.
(263) Voshinin, a. a.; Kerzhentsev, V. V.; Studnikov, E. L.; Yakush, L. V. Experimental Study of the Thermal Conductivity of Krypton at High Temperatures. *Inzh.-Fiz. Zh.* **1975**, *28*, 821–825.
(264) Smith, W. J. S.; Durbin, L. D.; Kobayashi, R. Thermal Conductivity of Light Hydrocarbons and Methane-Propane Mixtures at Low Pressures. *J. Chem. Eng. Data* **1960**, *5*, 316–321. https://doi.org/10.1021/je60007a023.
(265) Salavera, D.; Patil, K. R.; Coronas, A. Thermal Conductivity Measurement of Polyglycol Alkyl Ethers at Temperatures from (303.15 to 393.15) K. *J. Chem. Eng. Data* **2010**, *55*, 1449–1452. https://doi.org/10.1021/je900641w.
(266) Ryabtsev, N. I.; Kazaryan, V. A. The Measurement of Thermal Conductivity of I-Butylene. *Gazov. Delo* **1970**, 36–38.
(267) Roder, H. M. *Experimental Thermal Conductivity Values for Hydrogen, Methane, Ethane and Propane*; 84–3006; Natl. Bur. Stand., 1984.
(268) Rastorguev, Yu. L.; Pugach, V. V. Study of the Thermal Conductivity of Aromatic Hydrocarbons at High Pressures. *Izv. Vyssh. Uchebn. Zaved., Neft Gaz* **1970**, 69–72.
(269) Popov, V. N.; Malov, B. A. Thermal Conductivity of the Ethanol-Water System in the Temperature Range 2 0-300 C and at Pressures up to 30 MNm-2. *Teploenergetika* **1971**, *18*, 88–90.
(270) Perkins, R. A.; Huber, M. L. Measurement and Correlation of the Thermal Conductivity of 2,3,3,3-Tetrafluoroprop-1-Ene (R1234yf) and Trans-1,3,3,3-Tetrafluoropropene (R1234ze(E)). *J. Chem. Eng. Data* **2011**, *56*, 4868–4874.

https://doi.org/10.1021/je200811n.
(271) Ogiwara, K.; Arai, Y.; Saito, S. Thermal Conductivities of Liquid Hydrocarbons and Their Binary Mixtures. *Ind. Eng. Chem. Fundam.* **1980**, *19*, 295–300.
(272) Nieto de Castro, C. A.; Dix, M.; Fareleira, J. M. N. A.; Li, S. F. Y.; Wakeham, W. A. Thermal Conductivity of Chlorobenzene at Pressures up to 430 MPa. *Physica A (Amsterdam)* **1989**, *156*, 534.
(273) Neindre, B. L.; Garrabos, Y.; Kim, M. S. Measurements of the Thermal Conductivity of HFC-143a in the Temperature Range from 300 to 500 K at Pressures up to 50 MPa. *Int. J. Thermophys.* **2001**, *22*, 723–748. https://doi.org/10.1023/a:1010718814377.
(274) Neindre, B. L.; Garrabos, Y. Measurements of the Thermal Conductivity of HFC-32 (Difluoromethane) in the Temperature Range from 300 to 465 K at Pressures up to 50 MPa. *Int. J. Thermophys.* **2001**, *22*, 701–722. https://doi.org/10.1023/a:1010766730306.
(275) Mostert, R.; vandenBerg, H. R.; vanderGulik, P. S. Measurements of the Thermal Conductivity of Nitrogen with a Parallel-Plate Instrument. *Int. J. Thermophys.* **1990**, *11*, 597. https://doi.org/10.1007/bf00500849.
(276) Millat, J.; Ross, M.; Wakeham, W. A.; Zalaf, M. The Thermal Conductivity of Ethylene and Ethane. *Int. J. Thermophys.* **1988**, *9*, 481. https://doi.org/10.1007/bf00503148.
(277) Menashe, J.; Wakeham, W. A. Absolute Measurements of the Thermal Conducitvity of Liquids at Pressures up to 500 MPa. *Ber. Bunsen-Ges. Phys. Chem.* **1981**, *85*, 340–347.
(278) Mason, H. L. Thermal Conductivity of Some Industrial Liquids from 0 to 100C. *Trans. Am. Soc. Mech. Eng.* **1954**, *76*, 817–821.
(279) Makita, T.; Tanaka, Y.; Morimoto, Y.; Noguchi, M.; Kubota, H. Thermal Conductivity of Gaseous Fluorocarbon Refrigerants R 12, R13, and R 23, under Pressure. *Int. J. Thermophys.* **1981**, *2*, 249–268. https://doi.org/10.1007/bf00504188.
(280) Liu, M.; Han, L.; Zhang, Y.; Zhu, M. Experimental Study of the Thermal Conductivity of Saturated HFC-134a Vapor. *J. Eng. Thermophys.* **1995**, *16*, 273–275.
(281) Jeong, S. U.; Kim, M. S.; Ro, S. T. Liquid Thermal Conductivity of Binary Mixtures of Pentafluoroethane(R125) and 1,1,1,2-Tetrafluoroethane(R134a). *Int. J. Thermophys.* **1999**, *20*, 55–62. https://doi.org/10.1023/a:1021469928377.
(282) Hansen, K. C.; Tsao, L.-H.; Aminabhavi, T. M.; Yaws, C. L. Gaseous Thermal Conductivity of Hydrogen Chloride, Hydrogen Bromide, Boron Trichloride, and Boron Trifluoride in the Temperature Range from 55 to 380 .Degree.C. *J. Chem. Eng. Data* **1995**, *40*, 18–20. https://doi.org/10.1021/je00017a004.
(283) Gutweiler, J.; Raw, C. J. G. Transport Properties of Polar Gas Mixtures II. Heat Conductivites of Ammonia-Methylamine Mixtures. *J. Chem. Phys.* **1968**, *48*, 2413–2415.
(284) Guseinov, K. D.; Gylmanov, A. A.; Mirzoev, B. M. Study of the Thermal Conductivity of Aromatic Hydrocarbons. *Zh. Fiz. Khim.* **1976**, *50*, 212–214.
(285) Gross, U.; Song, Y. W. Thermal Conductivities of New Refrigerants R125 and R32 Measured by the Transient Hot-Wire Method. *Int. J. Thermophys.* **1996**, *17*, 607–619. https://doi.org/10.1007/bf01441507.

(286) Grigor’ev, B. A.; Ishkhanov, A. M.; Rastorguev, Yu. L.; Pugach, V. V. Thermal Conductivity of Cyclohexane and Cyclohexene at High Pressures. *Izv. Vyssh. Uchebn. Zaved., Neft Gaz* **1980**, *23*, 48–51.
(287) Gray, P.; Maczek, A. O. S. Thermal Conductivites, Viscosities, and Diffusion Coefficients of Mixtures Containing Two Polar Gases. In *Proc. Symp. Thermophys. Prop.*; 1968; pp 380–391.
(288) Golubev, I. F.; Vasil’kovskaya, T. N. Thermal Conductivity of Methyl and Ethyl Alcohols at Differnt Temperatures and Pressures. *Teploenergetika* **1969**, 77–82.
(289) Gillam, D. G.; Lamm, O. Precision Measurements of the Thermal Conductivities of Certain Liquids the Hot Wire Method. *Acta Chem. Scand.* **1955**, *9*, 657.
(290) Geller, V. Z.; Zaporozhan, G. V.; Ilushenko, S. V. Investigation of the Thermal Conducctivity of Freon 152A About the Saturated Line. *Promyshlennaya Teplotekhnika* **1982**, *4*, 77–80.
(291) Fleming, F. P.; Silva, L. de A.; Lima, G. dos S. V.; Herzog, I.; Orlande, H. R. B.; Daridon, J.-L.; Pauly, J.; Azevedo, L. F. A. Thermal Conductivity of Heavy, Even-Carbon Number n-Alkanes (C22 to C32). *Fluid Phase Equilib.* **2018**, *477*, 78–86. https://doi.org/10.1016/j.fluid.2018.08.016.
(292) Fellows, B. R.; Richard, R. G.; Shankland, I. R. Thermal Conductivity Data for Some Environmentally Acceptable Fluorocarbons. *Therm. Conduct.* **1990**, *21*, 311–325.
(293) Eucken, A. On the Temperature Dependence of the Thermal Conductivity of Some Gases. *Phys. Z.* **1911**, *12*, 1101.
(294) Davidson, J. M.; Music, J. F. *Rep.*; HW-29021; U. S. A. E. C., 1953.
(295) Danilova, G. A. Thermal Conductivity of Liquid Freons. *Kholod. Tekhn.* **1951**, *28*, 22–28.
(296) Cherneeva, L. Investigation of Thermal Conductivity of Freons. *Kholod. Tekhn.* **1952**, 55–58.
(297) Akhundov, T. S.; Gasanova, N. E. *Izv. Vyssh. Uchebn. Zaved., Neft Gaz* **1969**, *12*, 73.
(298) Zheng, X. Y.; Yamamoto, S.; Yoshida, H.; Masuoka, H.; Yorizane, M. Measurement and Correlation of the Thermal Conductivities for Several Dense Fluids and Mixtures. *J. Chem. Eng. Jpn.* **1984**, *17*, 237.
(299) Vargaftik, N. B.; Smirnova, E. N. About Temperature Dependence of the Thermal Conductivity of Water Steam. *Zh. Tekh. Fiz.* **1956**, *26*, 1251–1261.
(300) Thomas, L. H. Viscosity and Molecular Association. III. Association of Phenols and Amides. *J. Chem. Soc.* **1960**, 4906–4914. https://doi.org/10.1039/jr9600004906.
(301) Tarzimanov, A. A.; Gabitov, F. R. Thermal Conductivity of Water Vapor at Pressures up to 30 MPa and Temperatures to 700.Degree.C. *Teploenergetika* **1989**, 5.
(302) Srivastava, B. N.; Manna, A. Thermal Conductivity of Methyl Chloride-Hydrogen Sulphide Gas Mixtures. *J. Phys. A: Gen. Phys.* **1969**, *2*, 697–701.
(303) Spirin, G. G. Measurements of the Thermal Conductivity of Overheated Liquids. *Inzh.-Fiz. Zh.* **1978**, *35*, 445–449.
(304) Shestova, A. I. The Investigation of Thermal Conductivity of Freons of the Methane Type. PhD Thesis, Institute of Theoretical Physics, 1977.
(305) Rowley, R. L.; White, G. L. Thermal Conductivities of Ternary Liquid Mixtures. *J. Chem. Eng. Data* **1987**, *32*, 63–69. https://doi.org/10.1021/je00047a019.
(306) Roder, H. M.; Nieto de Castro, C. A.; Mardolcar, U. V. The Thermal Conductivity of

Liquid Argon for Temperatures between 11 and 140 K with Pressures to 70 MPa. *Int. J. Thermophys.* **1987**, *8*, 521. https://doi.org/10.1007/bf00503640.
(307) Richard, R. G.; Shankland, I. R. A Transient Hot-Wire Method for Measuring the Thermal Conductivity of Gases and Liquids. *Int. J. Thermophys.* **1989**, *10*, 673–686. https://doi.org/10.1007/bf00507988.
(308) Rastorguev, Y. L.; Bogatav, G. F.; Grigor' ev, B. A. A Study of the Thermal Conductivity of Higher N-Alkanes. *Khim. Tekhnol. Topliv Masel* **1974**, 54–58.
(309) Powers, R. W.; Mattox, R. W.; Johnston, H. L. Thermal Conductivity of Condensed Gases. II. The Thermal Conductivities of Liquid Normal and of Liquid Parahydrogen from 15 to 27 Degrees K. *J. Am. Chem. Soc.* **1954**, *76*, 5972–5973. https://doi.org/10.1021/ja01652a021.
(310) Perking, R. A.; Laesecke, A.; Nieto de Castro, C. A. Polarized Transient Hot Wire Thermal Conductivity Measurements. *Fluid Phase Equilib.* **1992**, *80*, 275–286. https://doi.org/10.1016/0378-3812(92)87074-w.
(311) Pavlovich, N. V. Study of the Thermal Conductivity of Natural Gases and Methane. *Gaz. Prom.* **1959**, 45–49.
(312) Nieto de Castro, C. A.; Li, S. F. Y.; Maitland, G. C.; Wakeham, W. A. Thermal Conductivity of Toluene in the Temperature Range 35-90 Deg C at Pressures up to 600 MPa. *Int. J. Thermophys.* **1983**, *4*, 311–327. https://doi.org/10.1007/bf01178782.
(313) Neindre, B. L.; Garrabos, Y.; Sabirzianov, A.; Goumerov, F. Measurements of the Thermal Conductivity of Chlorodifluoromethane in the Temperature Range of 300K to 515 K and at a Pressures up to 55 Mpa. *J. Chem. Eng. Data* **2001**, *46*, 193–201. https://doi.org/10.1021/je0002078.
(314) Neindre, B. L.; Garrabos, Y. Measurements of the Thermal Conductivity of HFC-125 in the Temperature Range from 300 to 515 K at Pressures up to 53 MPa. *Int. J. Thermophys.* **1999**, *20*, 375–399. https://doi.org/10.1023/a:1022692601764.
(315) Naziev, Y. M.; Abasov, A. A. Investigation of the Thermal Conductivity of Propylene. *Khim. Tekhnol. Topliv Masel* **1970**, 22–26.
(316) Mustafaev, R. A.; Gabulov, D. M. Experimental Study of the Thermal Conductivity of Aromatic Hydrocarbons at High Temperatures and Pressures. *Inzh.-Fiz. Zh.* **1977**, *33*, 857–863.
(317) Mustafaev, R. A. Thermal Conductivity of the Vapors of Normal Saturated Hydrocarbons at High Temperatures. *Izv. Vyssh. Uchebn. Zaved., Neft Gaz* **1973**, *16*, 71–74.
(318) Mostert, R.; vandenBerg, H. R.; vanderGulik, P. S. The Thermal Conductivity of Ethane in the Critical Region. *Int. J. Thermophys.* **1989**, *10*, 409. https://doi.org/10.1007/bf01133537.
(319) May, E. F.; Berg, R. F.; Moldover, M. R. Reference Viscosities of H2, CH4, Ar, and Xe at Low Densities. *Int. J. Thermophys.* **2007**, *28*, 1085–1110. https://doi.org/10.1007/s10765-007-0198-7.
(320) Mardolcar, U. V.; Nieto de Castro, C. A.; Wakeham, W. A. Thermal Conductivity of Argon in the Teperature Range 107 to 423 K. *Int. J. Thermophys.* **1986**, *7*, 259–272. https://doi.org/10.1007/bf00500153.
(321) Mamedov, R. T.; Guseinov, S. O. Thermal Conductivity of Some Paraffin Hydrocarbons and Nitriles at Low Temperatures. *Izv. Vyssh. Uchebn. Zaved., Neft*

Gaz **1988**, *31*, 59.
(322) Lavrenchenko, G. K.; Ruvinskii, G. Y.; Iljushenko, S. V.; Kanaev, V. V. Thermophysical Properties of Refrigerant R134a. *Int. J. Refrig.* **1992**, *15*, 386–392.
(323) Lambert, J. D.; Cotton, K. J.; Pailthorpe, M. W.; Robinson, A. M.; Scrivins, J.; Vale, W. R. F.; Young, R. M. Transport Properties of Gaseous Hydrocarbons. *Proc. R. Soc. London, Ser. A* **1955**, *231*, 280–290. https://doi.org/10.1098/rspa.1955.0173.
(324) Knibbe, P. G.; Raal, J. D. Simultaneous Measurement of the Thermal Conductivity and Thermal Diffusivity of Liquids. *Int. J. Thermophys.* **1987**, *8*, 181. https://doi.org/10.1007/bf00515201.
(325) Gregory, H.; Archer, C. T. Experimental Determination of the Thermal Conductivities of Gases. *Proc. R. Soc. London, Ser. A* **1926**, *110*, 91–122.
(326) Geller, V. Z.; Artamonov, C. D.; Zaporozhan, G. V.; Peredriy, V. G. Experimental Investigation of the Thermal Conductivity of Freon-12. *Inzh.-Fiz. Zh.* **1974**, *27*, 72–77.
(327) Geier, H.; Schaefer, K. Thermal Conductivity of Pure Gases and Gas Mixtures between 0 Degrees and 1200 Degrees C. *Allg. Waermetech.* **1961**, *10*, 70.
(328) Eucken, A. Ubr Das Warmeleitvermogen, Die Soezifische Warme Und Die Innere Reibung Der Gase. *Phys. Z.* **1913**, *14*, 324.
(329) Carmichael, L. T.; Sage, B. H. Thermal Conductivity of Fluids. n-Decane. *J. Chem. Eng. Data* **1967**, *12*, 210–213. https://doi.org/10.1021/je60033a015.
(330) Carmichael, L. T.; Jacobs, J.; Sage, B. H. Thermal Conductivity of Fluids. Propane. *J. Chem. Eng. Data* **1968**, *13*, 40–46. https://doi.org/10.1021/je60036a014.
(331) Baroncini, C.; Latini, G.; Piazza, F. Hot-Wire Transient Method for Measuring the Thermal Conductivity of Liquids. Predictions for Families of Alcohols and Aldehydes. *High Temp. - High Pressures* **1987**, *19*, 51.
(332) Baginskii, A. V.; Stankus, S. V. Thermodynamic and Transport Properties of Liquid HFC-227ea. *Int. J. Thermophys.* **2003**, *24*, 953–961. https://doi.org/10.1023/a:1025080212858.
(333) Assael, M. J.; Malamataris, N.; Karagiannidis, L. Measurements of the Thermal Conductivity of Refrigerants in the Vapor Phase. *Int. J. Thermophys.* **1997**, *18*, 341–352. https://doi.org/10.1007/bf02575165.
(334) Zhao, H.; Liu, Z.; Zhang, M.; Ma, X.; Zhang, J. The Measurement of Thermal Conductivity of Compound Adsorbent Used by Thermal Probe. *Shanghai Jiaotong Daxue Xuebao* **2007**, *41*, 5;437.
(335) Yata, J.; Hori, M.; Kurahashi, T.; Minamiyama, T. Thermal Conductivity of Alternative Fluorocarbons in Liquid Phase. *Fluid Phase Equilib.* **1992**, *80*, 287–296. https://doi.org/10.1016/0378-3812(92)87075-x.
(336) Wu, J.; Zheng, H.; Qian, X.; Li, X.; Assael, M. J. Thermal Conductivity of Liquid Dimethoxyethane from 243 to 353K at Pressures up to 30 MPa. *Int. J. Thermophys.* **2009**, *30*, 385–396. https://doi.org/10.1007/s10765-008-0549-z.
(337) Wu, J.; Liu, Z.; Jin, X.; Pan, J. Thermal Conductivity of Some Oxygenated Fuels and Additives in the Saturated Liquid Phase. *J. Chem. Eng. Data* **2005**, *50*, 102–104. https://doi.org/10.1021/je049818g.
(338) Weber, L. A. Thermal Conductivity of Oxygen in the Critical Region. *Int. J. Thermophys.* **1982**, *3*, 117. https://doi.org/10.1007/bf00503635.
(339) Vargaftik, N. B.; Vaselevskaya, Y. D. Thermal Conductivity of Helium at Temperatures from 300 to 6000 K. *Inzh.-Fiz. Zh.* **1982**, *42*, 412–416.

(340) Ueno, Y.; Nagasaka, Y.; Nagashima, A. The Thermal Conductivity of HCFC-123 and HFC-134a in the Gaseous Phase. In *Proc. Symp. Thermophys. Prop., 12th*; Tokyo, Japan, 1991; pp 225–228.
(341) Ubbink, J. B. Thermal Conductivity of Gaseous Hydrogen and of Gaseous Deuterium. *Comm. Phys. Lab. Univ. Leiden* **1948**, 1–10.
(342) Tomimura, T.; Maki, S.; Zhang, X.; Fujii, M. Measurements of Thermal Conductivity and Thermal Diffusivity of Alternative Refrigerants in Liquid Phase with a Transient Short-Hot-Wire Method. *Netsu Bussei* **2001**, *15*, 9–14. https://doi.org/10.2963/jjtp.15.9.
(343) Tomida, D.; Kenmochi, S.; Tsukada, T.; Qiao, K.; Yokoyama, C. Thermal Conductivities of [Bmim][PF6], [Hmim][PF6], and [Omim][PF6] from 294 to 335 K at Pressures up to 20 MPa. *Int. J. Thermophys.* **2007**, *28*, 1147–1160. https://doi.org/10.1007/s10765-007-0241-8.
(344) Sirota, A. M.; Latunin, V. I.; Belyaeva, G. M.; Gol' dshtein, I. i. Experimental Study of the Thermal Conductivity Maximums of Water in the Critical Region. *Teploenergetika* **1978**, 21–26.
(345) Sirota, A. M.; Latunin, V. I.; Belyaeva, G. M. Experimental Study of the Thermal Conductivity Maximum of Water near the Critical Point. *Teploenergetika* **1976**, 61–67.
(346) Senftleben, H. New Values of Thermal Conductivity and Specific Heat at Different Temperatures for a Series of Gases. *Z. Angew. Phys.* **1964**, *17*, 86–87.
(347) Scheffy, W. J.; Johnson, E. F. Thermal Conductivities of Liquids at High Temperatures. *J. Chem. Eng. Data* **1961**, *6*, 245–249. https://doi.org/10.1021/je60010a019.
(348) Sadykov, A. K.; Brykov, V. P.; Mukhamedzyanov, G. K. Thermal Conductivity of Low-Temperature Freons. *Teplo- Massoobmen Khim. Tekhnol.* **1975**, 31–35.
(349) Ruvinskii, G. Y.; Lavrenchenko, G. K.; Il' yushenko, S. V. Thermophysical Properties of R-134a. *Kholod. Tekhn.* **1990**, *67*, 20–26.
(350) Ramires, M. L. V.; Fareleira, J. M. N. A.; Nieto de Castro, C. A.; Dix, M.; Wakeham, W. A. The Thermal Conductivity of Toluene and Water. *Int. J. Thermophys.* **1993**, *14*, 1119–1130. https://doi.org/10.1007/bf00503676.
(351) Naziev, Y. M.; Abasov, A. A.; Nurberdiev, A. A.; Shakhverdiev, A. N. Study of the Thermal Conductivity of Cyclohexane at High Pressures. *Zh. Fiz. Khim.* **1974**, *48*, 434–436.
(352) Naziev, D. Y.; Aliev, A. M. Research of Thermal Conductivity of Binary Mixtures of N-Heptane-Isooctane at High Parameters of State. *Teplofiz. Vys. Temp.* **1992**, *30*, 294(-298).
(353) Mukhamedzyanov, G. K.; Usmanov, A. G.; Tarzimanov, A. A. Experimental Determination of the Thermal Conductivity of Saturated Hydrocarbons. *Izv. Vyssh. Uchebn. Zaved., Neft Gaz* **1963**, *6*, 75–79.
(354) Magomedov, U. B. Thermal Conductivity of Heavy Water at High Pressures and Its Density Dependence. *Inzh.-Fiz. Zh.* **1989**, *56*, 587.
(355) Krauss, R.; Luettmer-Strathmann, J.; Sengers, J. V.; Stephan, K. Transport Properties of 1,1,1,2-Tetrafluoroethane (R 134a). *Int. J. Thermophys.* **1993**, *14*, 951–988. https://doi.org/10.1007/bf00502117.
(356) Keyes, F. G. Thermal Conductivity of Gases. *Trans. Am. Soc. Mech. Eng.* **1955**, *77*, 1395.

(357) Keyes, F. G. Measurements of the Heat Conductivity of Nitrogen-Carbon Dioxide Mixtures. *Trans. Am. Soc. Mech. Eng.* **1951**, *73*, 597–603.
(358) Kashiwagi, H.; Oishi, M.; Tanaka, Y.; Kabota, H.; Makita, T. Thermal Condictivity of Fourteen Liquids in the Temperature Range 298-373 K. *Int. J. Thermophys.* **1982**, *3*, 101. https://doi.org/10.1007/bf00503634.
(359) Jamieson, D. T.; Hastings, E. H. *Thermal Conductivity, 8th Conference Proceeding, Ho, C. Y., Tayler, R. E., Eds.*; 1969.
(360) Huang, S.; Jiang, Z.; Du, J.; Lin, C.; Pan, D. Thermal Conductivity of Liquid Measured by Line Source Probe(LSP). *Huaxue Gongcheng* **1989**, *17*, 62–67.
(361) Hamrin, Jr., C. E.; Thodos, G. The Thermal Conductivity of Hydrogen for Pressures up to 660 Atm and Temperatures between 1.6 and 74.6 Degrees C. *Phys. Norv.* **1966**, *32(5)*, 918–932.
(362) Gregory, H. S. The Effect of Temperature on the Thermal Conductivity and the Accommodation Coefficient of Hydrogen. *Proc. R. Soc. London, Ser. A* **1935**, *149*, 35–56.
(363) Craven, P. M.; Lambert, J. D. The Viscosities of Organic Vapors. *Proc. R. Soc. (London)* **1951**, *205*, 439–449.
(364) Clifford, A. A.; Gray, P.; Johns, A. I.; Scott, A. C.; Watson, J. T. R. Thermal Conductivity of Argon, Nitrogen, and Hydrogen between 300 and 400 K and up to 25 MPa. *J. Chem. Soc., Faraday Trans. 1* **1981**, *77*, 2679–2691. https://doi.org/10.1039/f19817702679.
(365) Chernen’kaya, E. I.; Vernigora, G. A. Experimental Determination of the Thermal Conductivity of Aqueous Salt and Ammonia Solutions at 25 and 50 C. *Zh. Prikl. Khim.* **1972**, *45*, 1704–1707.
(366) Brykov, V. P.; Mukhamedzyanov, G. K.; Usmanov, A. G. Experimental Investigation of the Thermal Conductivity of Organic Fluids at Low Temperatures. *Inzh.-Fiz. Zh.* **1970**, *18*, 82.
(367) Barnard, J. A. *Trans. Faraday Soc.* **1959**, *55*, 947.
(368) Baglai, A. K.; Gurarii, L. L.; Kuleshov, G. G. Physical Properties of Compounds Used in Vitamin Synthesis. *J. Chem. Eng. Data* **1988**, *33*, 512–518. https://doi.org/10.1021/je00054a035.
(369) Assael, M. J.; Charitidou, E.; Nieto de Castro, C. A. Absolute Measurements of the Thermal Conductivity of Alcohols by the Transient Hot-Wire Technique. *Int. J. Thermophys.* **1988**, *9*, 813. https://doi.org/10.1007/bf00503247.
(370) Asambaev, A. Z. Thermal Conductivity of Liquid and Gaseous Freons and Their Mixtures. PhD Thesis, Institute of Technology for Refrigiration Industry, 1992.
(371) Akhundov, T. S.; Abdullaev, F. G.; Azizbekova, Z. A.; Dzhamalov, I. M.; Iskenderov, A. I.; Agaeva, D. A. Effect of Thermal Conductivity of Liquid on the Efficienicy of the Mixing Process of Heat Edging with Hot Water. *Izv. Vyssh. Uchebn. Zaved., Neft Gaz* **1986**, *29*, 56–59.
(372) Adamov, A. P.; Gasanov, G. D. Experimental Study of the Thermal Conductivity of Helium. *Teplofiz. Vys. Temp.* **1971**, *9*, 52–58.
(373) Zederberg, N. V.; Popov, V. N. The Experimental Investigation of the Thermal Conductivity of the Helium. *Teploenergetika* **1960**, 82–87.
(374) Zaporozhan, G. V.; Geller, V. Z. Experimental Investigation of the Thermal Conductivity Coefficient of Freons R-13 and R-14 at Low Temperature. *Zh. Fiz. Khim.* **1977**, *51*, 1056–1059.

(375) Yin, J. M.; Guo, J. X.; Zhao, Z. Y.; Tan, L. C.; Zhao, M. Thermal Conductivity of HFC152a. *Fluid Phase Equilib.* **1992**, *80*, 297–303. https://doi.org/10.1016/0378-3812(92)87076-y.
(376) Yata, J.; Hori, M.; Niki, M.; Isono, Y.; Yanagitani, Y. Coexistence Curve of HFC-134a and Thermal Conductivity of HFC-245fa. *Fluid Phase Equilib.* **2000**, *174*, 221–229. https://doi.org/10.1016/s0378-3812(00)00429-5.
(377) Yakush, L. V.; Vanicheva, N. A.; Zaitseva, L. S. Thermal Conductivity of Methanol-D3, Methanol, Ethane- D6, and Ethane in the Gas Phase. *Inzh.-Fiz. Zh.* **1979**, *37*, 472.
(378) Vargaftik, N. B.; Zayzeva, L. S. Thermal Conductivity of Heavy Water in the Gas Phase. *Inzh.-Fiz. Zh.* **1963**, *6*, 3–6.
(379) Tsvetkov, O. B. Study of the Thermal Conductivity of Liquid Freons. *Inzh.-Fiz. Zh.* **1965**, *9*, 810–815.
(380) Tsederberg, N. V. Study of Thermal Conductivity of Binary Solutions (Solutions of Ethyl Alcohol in Water). *Tr. Mosk. Energ. Inst.* **1955**, 13–27.
(381) Tarzimanov, A. A.; Arslanov, V. A. Thermal Conductivity of Carbon Dioxide at High Pressures. *Teplo- Massoobmen Khim. Tekhnol.* **1978**, 13.
(382) Tanaka, Y.; Matsuo, S.; Taya, S. Gaseous Thermal Conductivity of Difluoromethane (HFC-32), Pentafluoroethane (HFC-125), and Their Mixtures. *Int. J. Thermophys.* **1995**, *16*, 121–131. https://doi.org/10.1007/bf01438963.
(383) Srivastava, B. N.; Barua, A. K. Thennal Conductivity of Binary Mixtures of Diatomic and Monatomic Gases. *J. Chem. Phys.* **1960**, *32*, 427–435.
(384) Spencer-Gregory, H.; Dock, E. H. The Effect of Temperature on the Thermal Conductivity and the Accommodation Coefficient of Hydrogen below 0 Degrees C. *Philos. Mag.* **1938**, *25(166)*, 129–147.
(385) Sousa, A. T.; Fialho, P. S.; Nieto de Castro, C. A.; Tufeu, R.; LeNeindre, B. The Thermal Conductivity of 1-Chloro-1,1-Difluoroethane (HCFC-142b). *Int. J. Thermophys.* **1992**, *13*, 383–399. https://doi.org/10.1007/bf00503878.
(386) Shokouhi, M.; Jalili, A. H.; Mohammadian, A. H.; Hosseini-Jenab, M.; Nouri, S. S. Heat Capacity, Thermal Conductivity and Thermal Diffusivity of Aqueous Sulfolane Solutions. *Thermochim. Acta* **2013**, *560*, 63–70. https://doi.org/10.1016/j.tca.2013.03.017.
(387) Shashkov, A. G.; Kurbatov, V. A.; Zolotukhina, A. F.; Abramenko, T. N. Experimental Investigation of the Thermal Conductivity of Vapor-Gas Mixtures with Low Vapor Concentration. *Inzh.-Fiz. Zh.* **1983**, *45*, 275–282.
(388) Salceanu, C.; Bojin, S. On the Thermal Conductivity of Gases and Vapors. *C. R. Hebd. Seances Acad. Sci.* **1956**, *243*, 237.
(389) Rowley, R. L.; Yi, S.-C.; Gubler, D. V.; Stoker, J. M. Mutual Diffusivity, Thermal Conductivity, and Heat of Transport in Binary Liquid Mixtures of Alkanes in Chloroform. *J. Chem. Eng. Data* **1988**, *33*, 362–366. https://doi.org/10.1021/je00053a038.
(390) Rastorguev, Yu. L.; Gazdiev, M. A. Study of the Thermal Conductivity of Manyatomic Alcohols. *Inzh.-Fiz. Zh.* **1969**, *17*, 72–79.
(391) Potapov, M. D. The Thermal Conductivity of Liquid Binary Mixturesof Halogenated Hydrocarbons. PhD Thesis, Odessa Technological Institute of Food Industry, 1988.
(392) Peredriy, V. G. Investigation of the Thermal Conductivity of Freons of Methane

and Ethane Rows. PhD Thesis, Odessa Technological Institute of Food Industry, 1975.
(393) Michels, A.; Botzen, A. The Thermal Conductivity of Nitrogen at Pressures up to 2500 Atm. *Physica (Amsterdam)* **1953**, *19*, 585.
(394) Magerramov, S. G. Determination of the Thermal Conductivity of Liquids and Gases at High Parameters of State. *Izv. Vyssh. Uchebn. Zaved., Neft Gaz* **1982**, 64–66.
(395) LeNeindre, B.; Garrabos, Y.; Nikravech, M. Measurements of the Thermal Conductivity of Propane in the Supercritical Region. *J. Chem. Eng. Data* **2014**, *59*, 3422–3433. https://doi.org/10.1021/je500395f.
(396) Lees, C. H. X. On the Thermal Conductivities of Single and Mixed Solids and Liquids and Their Variation with Temperature. *Philos. Trans. R. Soc. London, Ser. A* **1898**, *191*, 399–440.
(397) Kramer, F. R.; Comings, E. W. Thermal Conductivity of Butane at High Pressure: Correlation with Other Gases. *J. Chem. Eng. Data* **1960**, *5*, 462–467. https://doi.org/10.1021/je60008a014.
(398) Kraft, K.; Leipertz, A. Thermal Diffusivity and Sound Velocity of Round-Robin R134a. *Fluid Phase Equilib.* **1996**, *125*, 245–255. https://doi.org/10.1016/s0378-3812(96)03079-8.
(399) Kannuluik, W. G.; Martin, L. H. The Thermal Conductivity of Some Gases at 0 Degrees C. *Proc. R. Soc. London, Ser. A* **1934**, *144*, 496.
(400) Jamieson, D. T.; Tudhope, J. S. A SIMPLE DEVICE FOR MEASURING THE THERMAL CONDUCTIVITY OF LIQUIDS WITH MODERATE ACCURACY. *J. Inst. Pet.* **1964**, *50*, 150–153.
(401) Ishkhanov, A. M.; Grigor' ev, B. A.; Pugach, V. V. Study of the Thermal Conductivity of Cyclopentane at High Pressures. *Izv. Vyssh. Uchebn. Zaved., Neft Gaz* **1981**, *36*, 50.
(402) Geller, Z. I.; Rastorguev, Yu. L.; Ganiev, Yu. A. Thermal Conductivity of Binary System of Selective Solvents. *Izv. Vyssh. Uchebn. Zaved., Neft Gaz* **1966**, *9*, 88–90.
(403) Geller, V. Z. Thermal Conductivity of Some Liquid Refrigerants at Low Temperature. In *Teplofiz. Svoistva Veshchestv Mater., Collect. Vol.*; No. 9, Rabinovich, V. A., Ed., Standards Publ.: Moscow, 1976; pp 147–161.
(404) Fareleira, J. M. N. A.; Li, S. F. Y.; Maitland, G. C.; Wakeham, W. A. Thermal Conductivity of Two Branched Alkanes in the Temperature Rang 36-88.Degree.C at Pressures up to 0.6 GPa. *High Temp. - High Pressures* **1984**, *16*, 427.
(405) Duan, Y.-Y.; Sun, L.-Q.; Shi, L.; Zhu, M.-S.; Han, L.-Z. Thermal Conductivity of Gaseous Trifluoroiodomethane (CF3I). *J. Chem. Eng. Data* **1997**, *42*, 890–893. https://doi.org/10.1021/je9700378.
(406) Choy, P.; Raw, C. J. G. Thermal Conductivity of Some Polyatomic Gases at Moderately High Temperatures. *J. Chem. Phys.* **1966**, *45*, 1413–1417.
(407) Carmichael, L. T.; Sage, B. H. Viscosity and Thermalconductivity of Nitrogen-n-Heptane and Nitrogen- n-Octane Mixtures Octane Mixtures. *AIChE J.* **1966**, *12*, 559–562.
(408) Cabaleiro, D.; Nimo, J.; Pastoriza-Gallego, M. J.; Pineiro, M. M.; Legido, J. L.; Lugo, L. Thermal Conductivity of Dry Anatase and Rutile Nano-Powders and Ethylene and Propylene Glycol-Based TiO2 Nanofluids. *J. Chem. Thermodyn.* **2015**, *83*,

67–76. https://doi.org/10.1016/j.jct.2014.12.001.
(409) Bulanov, N. V.; Nikitin, E. D.; Skripov, V. P. Thermal Conductivbity of Liquids in the Metastable State. *Inzh.-Fiz. Zh.* **1974**, *26*, 204–207.
(410) Baker, C. E.; Brokaw, R. S. Thermal Conductivities of Ordinary and Isotopically Substituted Polar Gases and Their Equimolar Mixtures. *J. Chem. Phys.* **1965**, *43*, 3519–3528. https://doi.org/10.1063/1.1696510.
(411) Assael, M. J.; Dix, M.; Lucas, A.; Wakeham, W. A. Absolute Determination of the Thermal Conductivity of the Noble Gases and Two of Their Binary Mixtures as a Function of Density. *J. Chem. Soc., Faraday Trans. 1* **1981**, *77*, 439–464. https://doi.org/10.1039/f19817700439.
(412) Assael, M. J.; Dalaouti, N. K. Thermal Conductivity of Toluene+Cyclopentane Mixtures: Measurements and Prediction. *Int. J. Thermophys.* **2001**, *22*, 659–678. https://doi.org/10.1023/a:1010759629398.
(413) Assael, M. J.; Charitidou, E.; Nieto de Castro, C. A.; Wakeham, W. A. The Thermal Conductivity of N-Hexane, n-Heptane and n-Decane by the Transient Hot-Wire Method. *Int. J. Thermophys.* **1987**, *8*, 663. https://doi.org/10.1007/bf00500786.
(414) Amirkhanov, Kh. I.; Adamov, A. P.; Gasanov, G. D. Experimental Investigation of the Thermal Conductivity of Argon at Low Temperatures (in Russian). *Inzh.-Fiz. Zh.* **1972**, *22*, 835.
(415) Adamov, A. P.; Magomedov, U. B. Experimental Study of the Thermal Conductivity of Heavy Water at Temperatures of 20-200 C and Pressures 0.1-400 MPa. *Teploenergetika* **1987**, *25*, 1237–1239.
(416) Zvetkob, O. B.; Laptev, Yu. A. *Teplofiz. Svoistva Veshch. Mater.*; 1989.
(417) Vines, R. G.; Bennett, L. A. The Thermal Conductivity of Organic Vapors. The Relation between Thermal Conductivity and Viscosity and the Significance of the Eucken Factor. *J. Chem. Phys.* **1954**, *22*, 360–366.
(418) Vines, R. G. The Thermal Conductivity of Organic Vapors: The Influence of Molecular Interaction. *Aust. J. Chem.* **1953**, *6*, 1–26.
(419) Venart, J. E. S. Liquid Thermal Conductivity Measurements. *J. Chem. Eng. Data* **1965**, *10*, 239–241. https://doi.org/10.1021/je60026a009.
(420) Vargaftik, N. B.; Yakush, L. V. Experimental Study of the Thermal Conductivity of Neon, Krypton, Xenon in the Wide Temperature Range. *Inzh.-Fiz. Zh.* **1971**, *21*, 491–499.
(421) Vargaftik, N. B. Pressure Dependence of the Coefficients of Thermal Conductivity of Gases and Vapor. *Zh. Tekh. Fiz.* **1937**, *7*, 1199.
(422) Tufeu, R.; Garrabos, Y.; LeNeindre, B. Thermal Conductivity and Thermal Diffusivity of Ethane Along The Critical Isochore. In *Proc. 16th Int. Conf. Therm. Conduct.*; Chicago, 1979; p 605.
(423) Tsvetkov, O. B.; Laptev, Yu. A.; Asambaev, A. G. The Thermal Conductivity of Binary Mixtures of Liquid R22 and R142b and R15 2a at Low Temperatures. *Int. J. Thermophys.* **1996**, *17*, 597–606. https://doi.org/10.1007/bf01441506.
(424) Tong, J.; Li, T. Experimental Determinanationa of the Thermal Conductivities of Several Organic Compounds and Correlation of Thermal Conductivity Data. *J. Eng. Thermophys.* **1995**, *16*, 133–136.
(425) Timrot, D. L.; Umanskii, A. S.; Koroleva, V. V. Thermal Conductivity of Helium, Hydrogen and Argon at High Temperatures (in Russian). *Teplofiz. Svoistva Zhidk. Gazov. Vys. Temp. Plazmy, Tr. Vses. Conf.* **1969**, *1966*, 207–216.

(426) Tanaka, Y.; Nakata, M.; Makita, T. Thermal Conductivity of Gaseous HFC-134a, HFC-143a, HCFC-141b, and HCFC-142 b. *Int. J. Thermophys.* **1991**, *12*, 949–963. https://doi.org/10.1007/bf00503512.
(427) Sun, L.; Venart, J. E. S. Thermal Conductivity, Thermal Diffusivity, and Heat Capacity of Gaseous Argon and Nitrogen. *Int. J. Thermophys.* **2005**, *26*, 325–372. https://doi.org/10.1007/s10765-005-4502-0.
(428) Sokolova, V. P.; Golubev, I. F. Teploprovodnost' Methane Pri Razlichnyh Temperaturah i Davleniah. *Teploenergetika* **1967**, *14*, 90.
(429) Sirota, A. M.; Latunin, V. I.; Nikolaeva, N. E. Experimental Study of the Thermal Conductivity Maximums of Water in the Critical Region. *Teploenergetika* **1981**, 72–73.
(430) Sirota, A. M.; Latunin, V. I.; Gol' dshtein, I. i.; Nikolaeva, N. E. Experimental Study of the Thermal Conductivity Maximums of Water in the Critical Region. *Teploenergetika* **1979**, 67–69.
(431) Schindler, D. L. The Heterogeneous Phase Behavior of the Helium-Propane, Nitrogen-Propane, and Helium-Nitrogen-Propane Systems. PhD Thesis, University of Kansas, 1966.
(432) Sadyikov, A. Kh. Experimental Investigation of Some Thermophysical Properties of Polyoxy Compounds. PhD Thesis, Kazan Technical Institute for Regrigeration, 1978.
(433) Roder, H. M.; Diller, D. E. Thermal Conductivity of Gaseous and Liquid Hydrogen. *J. Chem. Phys.* **1970**, *52*, 5928–5948.
(434) Roder, H. M. Thermal Conductivity of Methane for Temperatures between 110 and 310K with Pressures to 70 Mpa. *Int. J. Thermophys.* **1985**, *6*, 119–142. https://doi.org/10.1007/bf00500027.
(435) Patek, J.; Klomfar, J. Measurement of the Thermal Conductivity of Argon and Methane: A Test of a Transient Hot-Wire Apparatus. *Fluid Phase Equilib.* **2002**, *198*, 147–163. https://doi.org/10.1016/s0378-3812(01)00763-4.
(436) Naziev, Y. M.; Aliev, M. A. Study of the Thermophysical Properties of N-Undecane; n-Heptadecane and n-Octadecane at Various Temperatures and Pressures. *Izv. Vyssh. Uchebn. Zaved., Neft Gaz* **1973**, *16*, 73–76.
(437) Mustafaev, R. A. Dynamic Method of Thermal Conductivity Measurements at High Temperatures. *Inzh.-Fiz. Zh.* **1972**, *22*, 850–858.
(438) Misic, D.; Thodos, G. Thermal Condictivity Measurements of Methane in the Dense Gaseous State. *Physica (Amsterdam)* **1966**, *32*, 885.
(439) Marchenkov, E. I.; Shashkov, A. G. Study of the Thermal Conductivity of Helium in the Temperature Range from 400 to 1500 K from the Apparatus with Molibdanium Measuring Cell. *Inzh.-Fiz. Zh.* **1974**, *26*, 1089–1097.
(440) Makhrov, V. V.; Miroshnichenko, V. I. Experimental Study of the Thermal Conductivity and Temperature Jump in Non-Reacting Gases at Low Pressures. *Inzh.-Fiz. Zh.* **1984**, *47*, 376–383.
(441) Kubkov, A. T. Experimental Investigation of the Thermal Conductivity of Mixture R-12 + R- C318. In *Teplofiz. Svoistva Veshchestv Mater.*; Collect. Vol. No. 11, Rabinovich, V. A., Ed., Standards Publ.: Moscow, 1977; pp 47–54.
(442) Kerimov, A. M.; El' Darov, F. G.; El' Darov, G. S. A Relative Method of Determining the Thermal Conductivity of Hydrocarbons. *Izv. Vyssh. Uchebn. Zaved., Neft Gaz* **1970**, *13*, 77–80.

(443) Kashiwagi, H.; Hashimoto, T.; Tanaka, Y.; Kubota, H.; Makita, T. Thermal Conductivity and Density of Toluene in the Temperature Range 273-373 K at Pressures up to 250 MPa. *Int. J. Thermophys.* **1982**, *3*, 201–215. https://doi.org/10.1007/bf00503316.
(444) Johnston, H. L.; Grilly, E. R. The Thermal Conductivites of Eight Common Gases between 80 and 380 K. *J. Chem. Phys.* **1946**, *14*, 233–238. https://doi.org/10.1063/1.1724125.
(445) Ivanova, Z. A.; Tsederberg, N. V.; Popov, V. N. Experimental Study of Thermal Conductivity of Oxygen. *Teploenergetika* **1967**, 74–77.
(446) Hildenbrand, D. L.; Happe, J. A. Thermal Conductivities of Some Liquid Nitrate Esters. *J. Phys. Chem.* **1957**, *61*, 1415–1417.
(447) Haran, E. N.; Maitland, G. C.; Mustafa, M.; Wakeham, W. A. The Thermal Conductivity of Argon, Nitrogen and Carbon Monoxide in the Temperature Range 300-430 k at Pressures up to MPa. *Ber. Bunsen-Ges. Phys. Chem.* **1983**, *87*, 657–663.
(448) Guseinov, K. D.; Mirzoev, B. M. Experimental Determination of the Thermal Conductivity and P-v-t Relation of Liquid Chloro- and Isopropylbenzene. *Izv. Vyssh. Uchebn. Zaved., Neft Gaz* **1975**, *18*, 57–60.
(449) Gurova, A. N.; Mardolcar, U. V.; Nieto de Castro, C. A. Thermal Conductivity of 1,1-Difluoroethane(HFC-152a). *Int. J. Thermophys.* **1999**, *20*, 63–72. https://doi.org/10.1023/a:1021422012447.
(450) Grebenkov, A. J.; Kotelevsky, Y. G.; Saplitza, V. V.; Beljaeva, O. V.; Zajatz, T. A.; Timofeev, B. D. Experimental Study of Thermal Conductivity of Some Ozone Safe Refrigerants and Speed of Sound in Their Liquid Phase. In *Joint Meeting of IIR Commissions B1, B2, E1, and E2*; Padova, Italy, 1994; pp 419–429.
(451) Grebenkov, A. J.; Hulse, R.; Pham, H.; Singh, R. Physical Properties and Equation of State for Trans-1,3,3,3-Tetrafluoropropene. In *3rd Int. Inst. Refrigerantion Conference on Thermophysical Properties and Transfer Processes of Refrigerants*; Boulder, CO, pp Paper No. 191 Pages 1-19, 2009.
(452) Geller, V.; Rastorguev, Yu. L. Thermal Conductivity of Toluene. *Teploenergetika* **1968**, 80–81.
(453) Briggs, D. K. H. Thermal Conductivity of Liquids. *Ind. Eng. Chem.* **1957**, *49*, 418–421.
(454) Assael, M. J.; Charitidou, E.; Wakeham, W. A. Absolute Measurements of the Thermal Conductivity of Mixtures of Alcohols with Water. *Int. J. Thermophys.* **1989**, *10*, 793. https://doi.org/10.1007/bf00514476.
(455) Amirkhanov, Kh. I.; Adamov, A. P.; Magomedov, U. B. Experimental Study of the Thermal Conductivity of Ordinary and Heavy Water at Temperatures 25-350 C and Pressures from 0.1 to 245.3 mH/M*82. *Inzh.-Fiz. Zh.* **1975**, *29*, 825–830.
(456) Amirkhanov, Kh. I.; Adamov, A. P.; Magomedov, U. B. Experimental Study of the Thermal Conductivity of Heavy Water at Temperatures from 25 to 250 C and Pressures up to 245.3 MPa. *Teplofiz. Vys. Temp.* **1974**, *12*, 1128–1131.
(457) Amirkhanov, Kh. I.; Adamov, A. P. Experimental Study of the Thermal Conductivity of Argon. *Teploenergetika* **1970**, 61–65.
(458) Amirkhanov, Kh. I.; Adamov, A. P. Thermal Conductivity of Water in the Nearcritical Ans Supercritical Conditions. *Teploenergetika* **1963**, 69–72.
(459) Akhundov, T. S. Thermal Conductivity of Benzene. *Izv. Vyssh. Uchebn. Zaved.,*

Neft Gaz **1974**, *17*, 78.
(460) Watanabe, H.; Kato, H. Thermal Conductivity and Thermal Diffusivity of Twenty-Nine Liquids: Alkenes, Cyclic (Alkanes, Alkenes, Alkadienes, Aromatics), and Deuterated Hydrocarbons. *J. Chem. Eng. Data* **2004**, *49*, 809–825. https://doi.org/10.1021/je034162x.
(461) Wakeham, W. A.; Zalaf, M. The Thermal Conductivity of Some Electrically Conducting Liquids. *Fluid Phase Equilib.* **1987**, *36*, 183–194. https://doi.org/10.1016/0378-3812(87)85022-7.
(462) Vargaftik, N. B.; vasilevskaya, Y. D. Thermal Conductivity of Neon and Argon at High Temperatures. *Inzh.-Fiz. Zh.* **1981**, *40*, 473–481.
(463) Smith, J. F. D. The Thermal Conductivity of Liquids. *Trans. Am. Soc. Mech. Eng.* **1936**, *58*, 719–725.
(464) Rastorguev, Yu. L.; Geller, V. Z. New Version of the Measuring Cell for the Study of Thermal Conductivity of Liquids and Gases by the Hot Wire Technique. *Inzh.-Fiz. Zh.* **1967**, *13*, 16–23.
(465) Perkins, R.; Cusco, L.; Howley, J.; Laesecke, A.; Matthes, S.; Ramires, M. V. L. Thermal Conductivities of Alternatives to CFC-11 for Foam Insulation. *J. Chem. Eng. Data* **2001**, *46*, 428–432. https://doi.org/10.1021/je990337k.
(466) Pan, J.; Li, X.; Wu, J. Thermal Conductivity of Liquid 1,1,1,3,3,3-Hexafluoropropane (HFC-236fa) from 253 K to 373 K and Pressure up to 30 MPa. *Fluid Phase Equilib.* **2011**, *304*, 64–67. https://doi.org/10.1016/j.fluid.2011.02.015.
(467) Naziev, Y. M.; Abasov, A. A. Study of the Thermal Conductivity of Gaseous Unsaturated Hydrocarbons at Atmospheric Pressure and Various Temperatures. *Izv. Vyssh. Uchebn. Zaved., Neft Gaz* **1968**, *11*, 63–66.
(468) Millat, J.; Ross, M.; Wakeham, W. A.; Zalaf, M. The Thermal Conductivity of Neon, Methane and Tetrafluoromethane. *Physica A (Amsterdam)* **1988**, *148*, 124–152.
(469) Maczek, A. O. S.; Gray, P. Thermal Conductivities of Gaseous Mixtures Containing Polar Gases. *Trans. Faraday Soc.* **1970**, *65*, 1473–1489.
(470) Kuyumchev, A. A.; Shul' ga, V. M.; Atanov, Yu. A. Thermal Physical Properties of N-Nonane and n-Decane at Pressures up to 1-GPa. *Teplofiz. Vys. Temp.* **1988**, *26*, 727.
(471) Ikenberry, L. D.; Rice, S. A. On the Kinetic Theory of Dense Fluids. XIV Experimental and Theoretical Studies of Thermal Conductivity in Liquid Ar, Kr, Xe, and CH4. *J. Chem. Phys.* **1963**, *39*, 1561–1571. https://doi.org/10.1063/1.1734480.
(472) Gross, U.; Song, Y. W.; Kallweit, J.; Hahne, E. Thermal Conductivity of Saturated R123 and R134a - Transient Hot Wire Measurements. *Sci. Tech. Froid* **1990**, 103–108.
(473) Geller, V. Z. Investigation of the Thermal Conductivity of Methane Row Freons. In *Teplofiz. Svoistva Veshchestv Mater.*; No. 8, Rabinovich, V. A., Ed., Standards Publ.: Moscow, 1975; pp 162–176.
(474) Ganiev, Yu. A. Thermal Conductivity of Glycols. *Zh. Fiz. Khim.* **1969**, *43*, 239–240.
(475) Clifford, A. A.; Kestin, J.; Wakeham, W. A. 1979. *Physica A (Amsterdam)* **1979**, *97*, 729.
(476) Burinsky, V. V.; Totstkii, E. E.; Nikodimov, S. P. The Results of the Experimental Study of the Thermal Conductivity of SF6 in the Critical Region. *Teplofiz. Vys.*

Temp. **1981**, *19*, 514–518.
(477) Assael, M. J.; Karagiannidis, E.; Wakeham, W. A. Measurements of the Thermal Conductivity of R11 and R12 in the Temperature Range 250-340 K at Pressures up to 30 MPa. *Int. J. Thermophys.* **1992**, *13*, 735–751. https://doi.org/10.1007/bf00503903.
